# Correlation between Organelle Genetic Variation and RNA Editing in Dinoflagellates Associated with the Coral *Acropora digitifera*

**DOI:** 10.1093/gbe/evaa042

**Published:** 2020-02-27

**Authors:** Eiichi Shoguchi, Yuki Yoshioka, Chuya Shinzato, Asuka Arimoto, Debashish Bhattacharya, Noriyuki Satoh

**Affiliations:** e1 Marine Genomics Unit, Okinawa Institute of Science and Technology Graduate University, Onna, Okinawa, Japan; e2 Atmosphere and Ocean Research Institute, The University of Tokyo, Kashiwanoha, Kashiwa, Japan; e4 Department of Biochemistry and Microbiology, Rutgers University, New Brunswick, New Jersey

**Keywords:** Symbiodiniaceae, mitochondrial and plastid genomes, RNA editing, genetic variation, dinoflagellates, host coral populations

## Abstract

In order to develop successful strategies for coral reef preservation, it is critical that the biology of both host corals and symbiotic algae are investigated. In the Ryukyu Archipelago, which encompasses many islands spread over ∼500 km of the Pacific Ocean, four major populations of the coral *Acropora digitifera* have been studied using whole-genome shotgun (WGS) sequence analysis (Shinzato C, Mungpakdee S, Arakaki N, Satoh N. 2015. Genome-wide single-nucleotide polymorphism (SNP) analysis explains coral diversity and recovery in the Ryukyu Archipelago. Sci Rep. 5:18211.). In contrast, the diversity of the symbiotic dinoflagellates associated with these *A. digitifera* populations is unknown. It is therefore unclear if these two core components of the coral holobiont share a common evolutionary history. This issue can be addressed for the symbiotic algal populations by studying the organelle genomes of their mitochondria and plastids. Here, we analyzed WGS data from ∼150 adult *A. digitifera*, and by mapping reads to the available reference genome sequences, we extracted 2,250 sequences representing 15 organelle genes of Symbiodiniaceae. Molecular phylogenetic analyses of these mitochondrial and plastid gene sets revealed that *A. digitifera* from the southern Yaeyama islands harbor a different Symbiodiniaceae population than the islands of Okinawa and Kerama in the north, indicating that the distribution of symbiont populations partially matches that of the four host populations. Interestingly, we found that numerous SNPs correspond to known RNA-edited sites in 14 of the Symbiodiniaceae organelle genes, with mitochondrial genes showing a stronger correspondence than plastid genes. These results suggest a possible correlation between RNA editing and SNPs in the two organelle genomes of symbiotic dinoflagellates.

## Introduction

Symbiotic dinoflagellates in the family Symbiodiniaceae (previously known as the genus *Symbiodinium*) live together with many host organisms in coral reefs, including corals, sea anemones, bivalves, sponges, acoels, and forminiferans, in addition to existing as free-living cells (Hirose et al. 2008; Yamashita and Koike 2013; Pochon et al. 2014; Lajeunesse et al. 2018; González-Pech et al. 2019). Over the last 20 years, molecular phylogenetic analyses of the nuclear ribosomal DNA (rDNA) have revealed the high genetic diversity of Symbiodiniaceae (Rowan and Powers 1992; Coffroth and Santos 2005; Pochon et al. 2014). Population genetic analyses of Symbiodiniaceae have relied on comparisons of the internal transcribed spacer regions (ITS1 and ITS2) of nuclear rDNA, noncoding regions associated with the plastid *psbA* gene, and microsatellites (Thornhill et al. 2014). The existence of dozens of Symbiodiniaceae species has been suggested by phylogenetic analysis of these noncoding sequence data. However, in spite of the existence of draft genomes from Symbiodiniaceae (Shoguchi et al. 2013; Lin et al. 2015; Aranda et al. 2016; Liu et al. 2018; Shoguchi et al. 2018), whole-genome shotgun (WGS) sequence data have not yet been used for population genomic analysis of these algae.

**Fig. 1.**
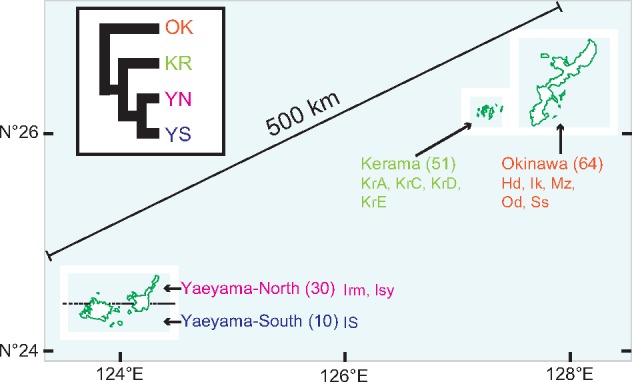
—Schematic diagram showing the sampling sites used for the population analysis of the coral *Acropora digitifera* in the Ryukyu Archipelago, Japan. Using whole-genome SNP analysis of *A. digitifera*, four clusters comprising Okinawa (OK), Kerama (KR), Yaeyama-North (YN), and Yaeyama-South (YS) were identified by Shinzato et al. (2015). The inset (top left) indicates phylogenetic relationships among the clusters based on an inferred tree of *A. digitifera* populations. The numbers in the parentheses indicate the coral sample numbers at each location. The information for Okinawa prefecture in white boxes was obtained from the National Land Numerical Information System (http://nlftp.mlit.go.jp/ksj/gmlold/index.html; last accessed January 17, 2019). The following islands, excluding the sampling locations, are omitted. Hd, Hedo; Ik, Ikei; Irm, Uehara; IS, Oohama; Isy, Kabira; KrA, Geruma; KrC, Yakabi; KrD, Aka; KrE, Zamami; Mz, Manza; Od, Ohdo; Ss, Sesoko.

Genome sequence data from the coral *Acropora* *digitifera* (Shinzato et al. 2011, 2015) have been used as a reference to study single-nucleotide polymorphisms (SNPs) in WGS reads from 155 coral individuals. These data revealed the population structure of *A. digitifera* in the Ryukyu Archipelago, Japan (Shinzato et al. 2015). Four major clusters or populations were found in this study: Okinawa (OK), Kerama (KR), Yaeyama-North (YN), and Yaeyama-South (YS) (fig. 1). There is approximately a distance of 500 km between the northern (OK and KR) and southern islands (Yaeyama) that were sampled (fig. 1). In addition, the previous genome-wide population genetic analysis of *A. digitifera* showed that these four populations had limited connectivity, particularly between OK and KR (Shinzato et al. 2015). Because KR is often considered a source for OK population recovery, this result provides an important cautionary note with regard to local conservation efforts. Namely, the transplantation of KR corals to OK coasts may not always be appropriate to facilitate the recovery of OK wild corals. The fertilized eggs of the coral *A. digitifera* do not have the symbiotic dinoflagellates. *Acropora* *digitifera* acquires symbiotic algae horizontally (acquired from the seawater environment) when they are in the planula larval stage (Harii et al. 2009). In the population genetic study of Shinzato et al. (2015), coral branches, including symbiotic dinoflagellates, were sampled for WGS analysis. It is therefore likely that Symbiodiniaceae genomes remain in these samples, which can also be analyzed to gain a perspective on symbiont distribution within the host populations. In particular, high copy number organelle genomes provide an ideal target for such an approach.

Among Symbiodiniaceae, mitochondrial (mt) and plastid (pt) genomes from *Breviolum minutum* (previously known as *Symbiodinium minutum*) are available as reference sequences (Mungpakdee et al. 2014; Shoguchi et al. 2015). Transcriptome analyses showed that all organelle protein-coding genes undergo RNA editing. This process is a posttranscriptional modification that is mediated by specific enzymes (Takenaka et al. 2013). It has been reported that pt RNA editing in land plants exhibits site-specific sensitivity for temperature and is inhibited by high temperature (Karcher and Bock 2002). The temperature sensitivity of RNA editing may also be related to the diversity of the organelle response to a changing environment in the Symbiodiniaceae, but this issue is poorly understood.

In this study, we analyzed Symbiodiniaceae organelle genome data from ∼150 individuals of *A. digitifera*. Our study posed two major questions: 1) do the phylogenies of organelle genes in the Symbiodiniaceae recapitulate host relationships that show the presence of local populations, and 2) do SNPs among the Symbiodiniaceae organelles have a potential relationship with RNA-editing events.

## Results and Discussion

### Diversity of Symbiodiniaceae Organelle Sequences in *A. digitifera*

We used *B. minutum* as the reference to extract Symbiodiniaceae organelle sequences from the Illumina database (DRA003938) derived from 155 *A. digitifera* holobionts (fig. 1). Five samples with low coverage of protein-coding sequences were removed for each organelle genome analysis, leaving 150 samples for downstream analysis (see Materials and Methods). As a result, we recovered 450 representative sequences from 3 genes in the mt genome and 1,800 representative sequences from 12 genes in the pt genome (supplementary data sets S1 and S2, Supplementary Material online).

**Fig. 2.**
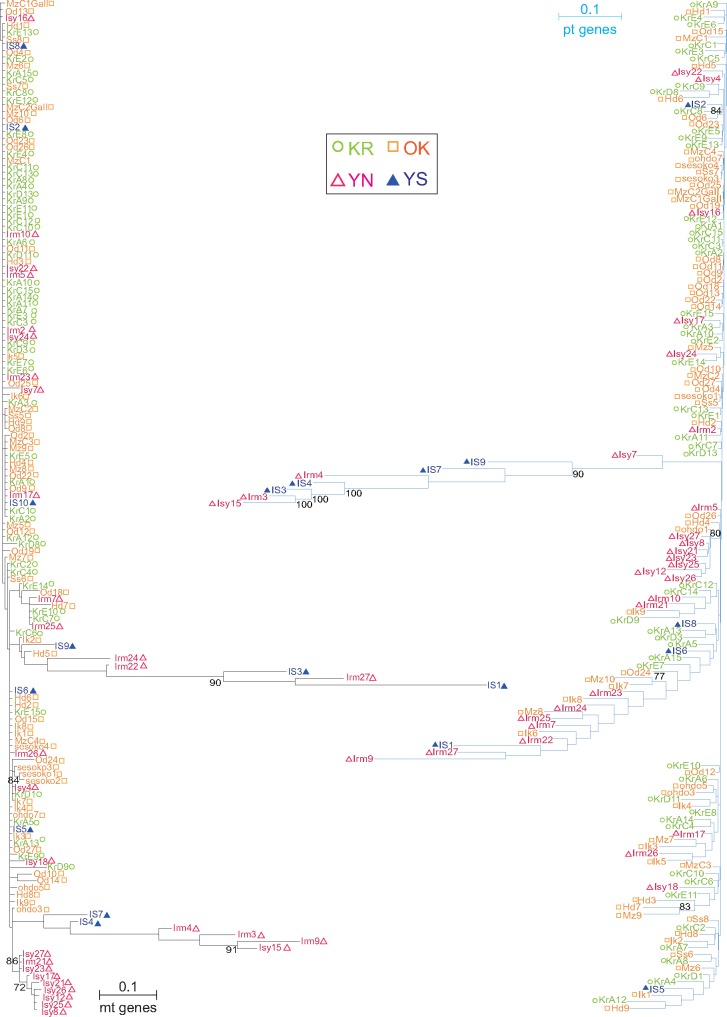
—Maximum likelihood trees inferred from organelle genes of Symbiodiniaceae populations. Only nodes with ≥70% bootstrap support are indicated in the tree. Three concatenated mt genes were used to reconstruct the tree on the left side of the figure. Six YN samples are clustered with moderate high bootstrap support (72%). Some Symbiodiniaceae sequences from the Yaeyama area have long branches. A total of 12 concatenated pt genes were used to reconstruct the tree on the right side of the figure. Yaeyama Symbiodiniaceae sequences with long branches also correspond to the samples with long branches in the mt gene tree (Irm3, Irm4, Irm9, Irm27, Isy15, IS1, IS3, and IS7).

To determine whether the evolutionary histories of organelle genes in the Symbiodiniaceae populations recapitulate host relationships, representative sequences from each compartment were used to build phylogenies. We hypothesized that the four clusters found in the population analysis of the host *A. digitifera* were also present in the associated Symbiodiniaceae populations (fig. 1). The phylogenetic tree inferred from the three mt genes showed the clustering of the specimens from YN with high bootstrap support (fig. 2, bottom left). However, we failed to detect the four clusters identified in the analysis of the host coral. Thus, the mitochondrial sequence data supported only the presence of the YN group in the associated Symbiodiniaceae populations. In addition, sequences from seven YN individuals and three YS individuals exhibited a long branch (fig. 2). This suggests that *A. digitifera* from the Yaeyama islands (YN and YS) harbor a different Symbiodiniaceae population from those of Okinawa Island (OK) and the Kerama islands (KR), in addition to a common population in the Ryukyu Archipelago.

Twelve protein-coding genes (*psbA*, *psbB*, *psbC*, *psbD*, *psbE*, *psbI*, *petB*, *petD*, *psaA*, *psaB*, *atpA*, and *atpB*) are encoded in Symbiodiniaceae pt DNA. These have plasmid-like structures referred to as minicircles (Zhang et al. 1999) that each encodes a single-gene (1.8–3.3 kb) in *B. minutum* (Mungpakdee et al. 2014). A ML tree of plastid genes showed that some of the sequences from YN and YS individuals cluster with high bootstrap support (fig. 2). The four host clusters, KR, OK, YN, and YS (inset of fig. 1), were also absent from the pt data, although seven populations from YN are clustered with an OK population. Many of the Yaeyama samples (YN and YS) that had a long branch in mt gene trees also had a long branch in pt gene trees (fig. 2, middle right). The populations of YN and YS were clustered with 90% bootstrap support. These results suggest that some of the southern *A. digitifera* individuals maintain different Symbiodiniaceae populations from the remaining corals. The holobionts from the southern islands may be more diversified than those of the northern islands in the Ryukyu Archipelago.

Our analysis of Symbiodiniaceae populations using organelle genomes suggests that the *A. digitifera* clusters in the southern region may contain a different, locally adapted population of symbiotic algae. To validate the presence of a different Symbiodiniaceae, we studied ITS2 sequences in the WGS data (supplementary table 1, Supplementary Material online). The detected ITS types supporting the majority belong to the genus *Cladocopium* (clade C type in previous classification). Interestingly, the *Durusdinium* (clade D in previous classification) were found only in the WGS data of the Yaeyama samples, supporting the presence of different populations in the southern region of the Ryukyu Archipelago. Therefore, future studies should focus on both coral and Symbiodiniaceae populations to understand the establishment of coral reefs in different areas.

### Diversity of Organelle Genes and Possible RNA Editing Sites

RNA editing has been analyzed in detail for transcripts from dinoflagellate mt and pt genes (Lin et al. 2002; Zauner et al. 2004; Zhang et al. 2008; Klinger et al. 2018). The conservation patterns of edited sites from mt mRNAs have been studied among core dinoflagellates, including the basally diverging *Amphidinium* and the Symbiodiniaceae (Zhang et al. 2008). A recent report has discussed the dynamics and evolution of RNA editing in dinoflagellate plastid genomes using a large data set of dinoflagellates (Klinger et al. 2018).

**Fig. 3.**
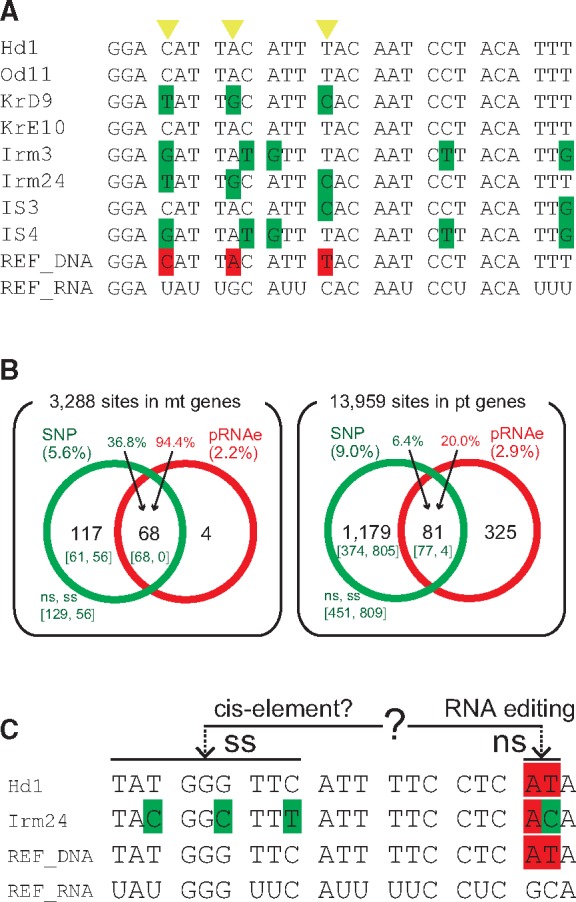
—The relationship between SNPs and possible RNA editing (pRNAe) sites. (*A*) Alignment of a region of the *cob* sequence in mt DNA is shown with the RNA editing sites (highlighted in red) of the reference sequences. The correspondence between SNP and pRNAe is marked with yellow arrowheads. (*B*) SNPs from 3,288 sites in mt genes (left) and from 13,959 sites in pt genes (right) were identified by comparing 150 coral holobiont samples. The numbers in parentheses show the percentage of SNPs and pRNAe. The SNP percentage in pt genes was higher than in mt genes. Comparison with pRNAe shows that many pRNAe sites in mt genes correspond to SNP sites. The numbers in square brackets indicate nonsynonymous (ns) and synonymous substitution (ss) SNPs, respectively. The numbers on each gene are shown in supplementary figure S1, Supplementary Material online. (*C*) Hypothesis for the relationship between nonsynonymous and synonymous SNPs in organelle genomes of the Symbidiniaceae. As an example, a region of the *psaA* alignment is shown and indicates that stretches of ∼20 nucleotides in sites upstream of edited sites may provide a specific sequence context recognized by editing activity (Takenaka et al. 2013).

To examine the relationship between SNPs and RNA editing sites, we used 2,250 sequences (supplementary data sets S1 and S2, Supplementary Material online) from each of the three mt genes and 12 pt genes from 150 samples recovered from the Symbiodiniaceae organelle genomes. By aligning sequences from each of the three mt genes and 12 pt genes from the 150 samples, we determined the percentages of SNPs in the genes (fig. 3*A*;table 1; supplementary fig. S1, Supplementary Material online). Even though the SNP percentages in *petB* (6.4%) (42/657) and *petD* (5.2%) (25/477) were slightly lower than those in the other pt genes (table 1; supplementary fig. S1, Supplementary Material online), the total SNP percentage of pt genes (9.0%; 1,260/13,959) was higher than that in mt genes (5.6%; 185/3,288) (fig. 3*B*).

**Table 1:** Correspondence between Detected SNP Sites and Potential RNA Editing Sites

Gene	Analyzed Site	No. of SNPs (%)	No. of pRNAe^a^	No. of Correspondences
Mitochondria				
*cox1*	1,455	57 (3.9)	29	26
*cox3*	771	56 (7.3)	24	24
*cob*	1,062	72 (1.8)	19	18
Plastid				
*psbA*	1,029	103 (10.0)	3	2
*psbB*	1,500	137 (9.1)	30	6
*psbC*	1,359	143 (10.5)	26	7
*psbD*	1,074	90 (8.4)	8	4
*psbE*	234	17 (7.3)	9	2
*psbI*	108	16 (14.8)	3	0
*petB*	657	42 (6.4)	23	3
*petD*	477	25 (5.2)	33	7
*psaA*	2,022	153 (7.6)	100	17
*psaB*	2,094	194 (9.3)	79	12
*atpA*	1,434	143 (10.0)	43	9
*atpB*	1,971	197 (10.0)	49	12

a Mungpakdee et al. (2014).

We did not have transcriptome data from the Symbiodiniaceae populations, therefore, we studied the data from *B. mimutum* (Mungpakdee et al. 2014; Shoguchi et al. 2015) and defined the known edited sites as possible RNA editing sites (pRNAe) in our data. By comparing SNPs and pRNAe in the three mt genes, we found that 68 sites were shared between these two data sets (fig. 3*B*). These account for 36.8% (68/185) of the SNP sites and 94.4% (68/72) of the pRNAe sites (fig. 3*B*;supplementary fig. S1, Supplementary Material online). The SNPs of the shared sites potentially contain the nucleotides prior to and after RNA editing (fig. 3*A*), suggesting that gain (or loss) of RNA editing sites may cause the polymorphism.

Within the pt data, the percentages of SNP sites and pRNAe sites were 9.0% (1260/13,959) and 2.9% (406/13,959), respectively, with the shared sites totaling 81 (fig. 3*B*). The reason for the lower percentage of shared sites in pt mRNA than those in mt mRNA may be explained by a recent report that suggested individual RNA editing sites in dinoflagellate plastids are species-specific and not highly conserved (Klinger et al. 2018). Alternatively, SNPs among RNA editing sites may be low in the pt genome of the Symbiodiniaceae. Finally, to characterize the high SNP percentage of pt genes, we classified the sites into SNPs with nonsynonymous or synonymous substitutions (ns or ss) (fig. 3*B* and supplementary fig. S1, Supplementary Material online). The sites of synonymous SNPs (809) exceeded those of nonsynonymous SNPs (451) in pt genes, although synonymous sites were less than nonsynonymous sites in mt genes (fig. 3*B*;supplementary fig. S1, Supplementary Material online). In land plant organelles, mRNA editing relies on cis-binding sites for trans-acting editing-site-specific proteins encoded in the nucleus (Lynch et al. 2006). We hypothesize that some of the nonsynonymous SNPs correspond to RNA editing sites, and that the potential cis-binding sites may relate to the presence of many synonymous SNPs in pt genes (fig. 3*C*). The simultaneous sequencing of genomes and transcriptomes from single-Symbiodiniaceae cells is needed to better understand pt SNP and the pRNAe data.

In summary, we analyzed the genetic diversity of two organelle genomes from Symbiodiniaceae hosted by four *A. digitifera* populations. Our results show that corals in the southern sites (YN and YS) contain a different Symbiodiniaceae population from those in the north (OK and KR). Some of the same algal symbionts are, however, shared by these areas (fig. 2). This suggests the presence of complex relationships among the southern holobiont populations. Many of the SNP sites in the mt DNA from the symbiotic dinoflagellates correspond to known RNA editing sites (fig. 3). The sharing of these sites is apparently at a lower percentage in pt genes (6.4%) than in mt genes (36.8%) when using hypothetical RNA editing data. Future studies of the relationship between local climate change and the diversity of organelle genome sequences (including RNA editing) may provide critical insights into environmental adaptability among Symbiodiniaceae populations (Baker 2003; Hidaka 2016).

## Materials and Methods

### WGS Data and Read-Mapping of Symbiodiniaceae Sequences

We assumed that the WGS sequences [accession no. DRA003938] in the population genomic analysis of *A. digitifera* (Shinzato et al. 2015) include dinoflagellate genome data, in particular of high copy number organelle genomes. The sequencing coverage of each WGS data set from 155 coral individuals was ∼7× on average for ∼447 Mb of the reference genome (version 1.1) of *A. digitifera* (Shinzato et al. 2015). The deposited WGS reads without further processing (DRA003938) were used for mapping. The reference sequences (∼2–291 kb) for protein-coding genes from the organelle genomes of *B. minutum* are publicly available [accession nos. LC002801–LC002802; JX094304 and JX094335]. Using Bowtie v0.7.12 with default parameters (Li and Durbin 2009), the read data from 155 individuals of *A. digitifera* were mapped separately to the reference organelle data. A majority of the mapped sites were gene-encoding regions. The average mapped read coverage to the organelle genes were ∼35× for mitochondrial genes and ∼9× for plastid genes. The dominant nucleotides from the mapped reads were selected at each site, and the representative gene sequences determined in each sample. Our method does not rule out the possibility that representative sequences may include hybrid data because short-read data (75–100 bp paired-end reads) were used from the coral holobionts (DRA003938). Five samples with low coverage of protein-coding sequences were removed for each organelle genome analysis (one mt genome sample from KR and four from OK; four pt genome samples from OK and one from YS), and 150 samples were used. These representative sequences are available within the online supplementary materials (supplementary data sets S1 and S2, Supplementary Material online).

The ITS2 sequences from the Symbiodiniaceae nuclear genome were initially studied using a BlastN (<1e–20) search as queries of the ITS2 databases (Franklin et al. 2012). If a read hits multiple ITS2 sequences, the ITS2 type with the highest BLAST score was assigned to it. A total of 51 of the 151 samples had more than one read with an assigned ITS2 type (supplementary table 1, Supplementary Material online).

### Molecular Phylogenetic Analyses

The mt genome from *B. minutum* encodes three protein-coding genes: *cox1*, *cox3*, and *cob*. The nucleotide sequences of these three mt genes were concatenated. A total of 150 representative sequences from the Symbiodiniaceae populations associated with the host coral were aligned for each of the three genes using MAFFT (Katoh and Standley 2013). Phylogenetic model selection for the aligned and concatenated organeller genes was performed using ModelTest-NG version 0.1.5 (Darriba et al. 2019). We performed molecular phylogenetic analyses of the aligned sequences using the GTR+I+Gamma model suggested by the evolutionary model selection. Maximum likelihood (ML) analysis was performed using RAxML version 8.2.10 (Stamatakis 2014) with 100 bootstrap replicates. Similarly, a molecular phylogenetic tree of pt genes was constructed from twelve protein-coding genes. Trees were edited using iTOL 5.3 (Letunic and Bork 2019).

### Genetic Variation and pRNAe

The nucleotide sites lacking 100% conservation among the 150 populations (supplementary data sets S1 and S2, Supplementary Material online) comprised potential SNPs. We manually confirmed the alignments with MView (Chojnacki et al. 2017) and Hyphy in MEGA7 (Kumar et al. 2016). The RNA-edited sites from the reported *B. minutum* organelle data (Mungpakdee et al. 2014; Shoguchi et al. 2015) were compared with the SNP data. The numbers of ns and ss from the 150 aligned sequences were counted using Hyphy. If both ns and ss were located on a particular codon SNP site, it was counted as an ns.

## Supplementary Material

evaa042_Supplementary_Data

## Notes

**Data deposition:** Raw sequence data have been deposited in DDBJ under accession DRA003938.
