# Supplementary material for: Correlation between Organelle Genetic Variation and RNA Editing in Dinoflagellates Associated with the Coral Acropora digitifera
Source: Genome Biol Evol. 2020 Feb 27;12(3):203–9. doi: 10.1093/gbe/evaa042 (PMC7144361; doi:10.1093/gbe/evaa042)
Supplement: evaa042_Supplementary_Data [file evaa042_supplementary_data.zip › Supplemental_data_Shoguchi_et_al_20190912-2.pdf]

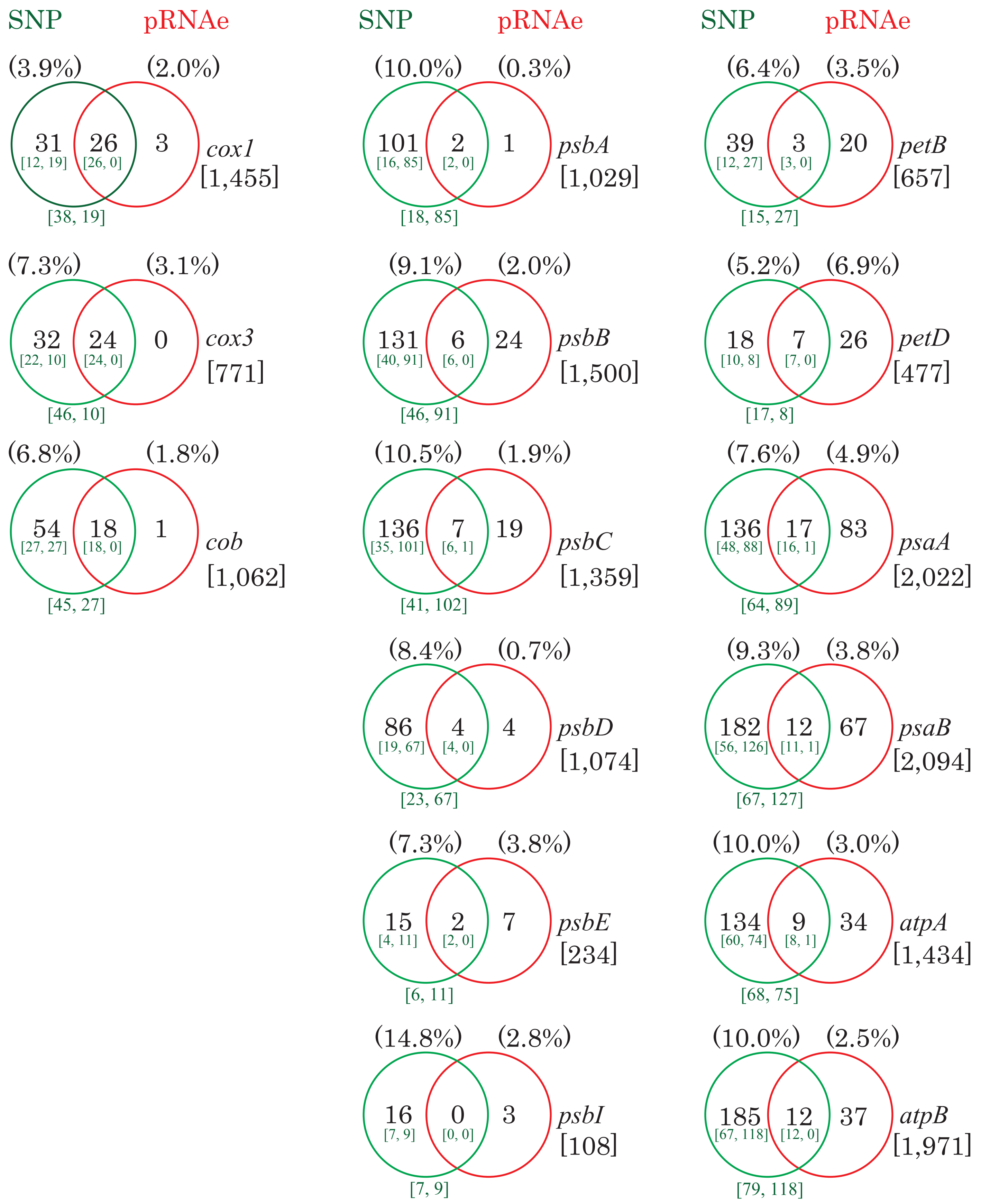

Supplementary Fig. S1. The relationship between SNPs and possible RNA editing (pRNAe) sites. SNP and pRNAe percentages for each gene are shown in parentheses. The numbers in square brackets show the aligned sequence length for each gene. Numbers in the green square brackets indicate non-synonymous (ns) and synonymous substitution (ss) SNPs, respectively.

Dataset S1. The nucleotide sequences of Symbiodiniciae mitochondrial (mt) genomes from 150 individuals of *Acropora digitifera* and the reference sequences from *Breviolum minutum*

```
>Hd1_cox1
ataaatctcttaaccattaatttcctatttcattcactagttaaaaattgtaatcataaa
ggcttaggaatctattatttattatctggattcatctttggaatctccggtacattaata
tcagtccttataagaatagaattatattcttcaggaaataggattatatctccagaaaac
cagaacttctataatataagcattacattgcatggctttcttatgattttctttttagta
atgcctggcttgtttggaggatttggaaattattttgtacctatctttcaagggtctcca
gaagtggtatatcctagagtcaataatttttctatcttaattcttttgctttcatatctt
ttcctaatcctttctttaatctcagaatttggaggtggtacagggtggacgctctaccca
ccattatccacttcttttatgactttatcaccttcaagtacaggaaatcttatatttgga
ttaataatctctggtatatcttcatgtcttacatctcttaacttttggacaacaattcat
tttctgagatcttattatctgatattatcttctatcccattatttccttgggctttcttg
attacagctttcatgcttttattaacattaccaatcttatctggtacacttcttttaata
ttgggtgatcttcattctaatacacttttctttgatccaatatttggaggagatcctata
ttctatcaacatttattttggtttttggacatccagaagtttacatattaataattcct
gcatttgggatcatttccataataatttctggtattttacagttaataatctttgctaac
caatcaatgatctttgccatgtcatctatttctcttcttggaggtcttgtttggggacat
catatgtatactgtaggtttagaaagtgatacaagagcttattttacaggagttacaatc
ttaatatccttaccaactggtacaaaaatctttaattggctttttacatatctctcaaat
ccaccattattacaccttagaattacttctgtcttcctctcacatctctttttattaatg
tttacgataggtgggtcaacaggaataattcttggaaatggtgcagtggatctaggatta
catgatacatattatgttgtagcacattttcattttgttctttctttaggagctataatt
gctatcttctctggaataatcttgaatggagaaaagattgttgctactaagaatttatta
ctttcatcctcatgtacactctctctttatcatttacatttaatatttattggtattctt
cttaccttttccccaatgcatttcttaggatttaatgttatgccaagaagaatcccatcc
tttccagattcttttcattcctggaattccctgtcatctattggatcaggaataactttc
ctatctttttctatg
>Hd2_cox1
ataaatctcttaaccattaatttcctatttcattcactagttaaaaattgtaatcataaa
ggcttaggaatctattatttattatctggattcatctttggaatctccggtacattaata
tcagtccttataagaatagaattatattcttcaggaaataggattatatctccagaaaac
cagaacttctataatataagcattacattgcatggctttcttatgattttctttttagta
atgcctggcttgtttggaggatttggaaattattttgtacctatctttcaagggtctcca
gaagtggtatatcctagagtcaataatttttctatcttaattcttttgctttcatatctt
ttcctaatcctttctttaatctcagaatttggaggtggtacagggtggacgctctaccca
ccattatccacttcttttatgactttatcaccttcaagtacaggaaatcttatatttgga
ttaataatctctggtatatcttcatgtcttacatctcttaacttttggacaacaattcat
tttctgagatcttattatctgatattatcttctatcccattatttccttgggctttcttg
attacagctttcatgcttttattaacattaccaatcttatctggtacacttcttttaata
ttgggtgatcttcattctaatacacttttctttgatccaatatttggaggagatcctata
ttctatcaacatttattttggtttttggacatccagaagtttacatattaataattcct
gcatttgggatcatttccataataatttctggtattttacagttaataatctttgctaac
caatcaatgatctttgccatgtcatctatttctcttcttggaggtcttgtttggggacat
catatgtatactgtaggtttagaaagtgatacaagagcttattttacaggagttacaatc
ttaatatccttaccaactggtacaaaaatctttaattggctttttacatatctctcaaat
ccaccattattacaccttagaattacttctgtcttcctctcacatctctttttattaatg
tttacgataggtgggtcaacaggaataattcttggaaatggtgcagtggatctaggatta
catgatacatattatgttgtagcacattttcattttgttctttctttaggagctataatt
gctatcttctctggaataatcttgaatggagaaaagattgttgctactaagaatttatta
ctttcatcctcatgtacactctctctttatcatttacatttaatatttattggtattctt
cttaccttttccccaatgcatttcttaggatttaatgttatgccaagaagaatcccatcc
tttccagattcttttcattcctggaattccctgtcatctattggatcaggaataactttc
ctatctttttctatg
>Hd3_cox1
ataaatctcttaaccattaatttcctatttcattcactagttaaaaattgtaatcataaa
ggcttaggaatctattatttattatctggattcatctttggaatctccggtacattaata
tcagtccttataagaatagaattatattcttcaggaaataggattatatctccagaaaac
cagaacttctataatataagcattacattgcatggctttcttatgattttctttttagta
```

atgcctggcttgtttggaggatttggaaattattttgtacctatctttcaagggtctcca
gaagtggtatatcctagagtcaataatttttctatcttaattcttttgctttcatatctt
ttcctaatcctttctttaatctcagaatttggaggtggtacagggtggacgctctaccca
ccattatccacttcttttatgactttatcaccttcaagtacaggaaatcttatatttgga
ttaataatctctggtatatcttcatgtcttacatctcttaacttttggacaacaattcat
tttctgagatcttattatctgatattatcttctatcccattatttccttgggctttcttg
attacagctttcatgcttttattaacattaccaatcttatctggtacacttcttttaata
ttgggtgatcttcattctaatacacttttctttgatccaatatttggaggagatcctata
ttctatcaacatttattttggtttttggacatccagaagtttacatattaataattcct
gcatttgggatcatttccataataatttctggtattttacagttaataatctttgctaac
caatcaatgatctttgccatgtcatctatttctcttcttggaggtcttgtttggggacat
catatgtatactgtaggtttagaaagtgatacaagagcttattttacaggagttacaatc
ttaatatccttaccaactggtacaaaaatctttaattggctttttacatatctctcaaat
ccaccattattacaccttagaattacttctgtcttcctctcacatctcttttattaatg
tttacgataggtgggtcaacaggaataattcttggaaatggtgcagtggatctaggatta
catgatacatattatgttgtagcacattttcattttgttctttctttaggagctataatt
gctatcttctctggaataatcttgaatggagaaaagattgttgctactaagaatttatta
ctttcatcctcatgtacactctctctttatcatttacatttaatatttattggtattctt
cttaccttttccccaatgcatttcttaggatttaatgttatgccaagaagaatcccatcc
tttccagattcttttcattcctggaattccctgtcatctattggatcaggaataactttc
ctatctttttctatg
>Hd4_cox1
ttaaatctcttaaccattaatttcctatttcattcactagttaaaaattgtaatcataaa
ggcttaggaatctattatttattatctggattcatctttggaatctccggtacattaata
tcagtccttataagaatagaattatattcttcaggaaataggattatatctccagaaaac
cagaacttctataatataagcattacattgcatggctttcttatgattttctttttagta
atgcctggcttgtttggaggatttggaaattattttgtacctatctttcaagggtctcca
gaagtggtatatcctagagtcaataatttttctatcttaattcttttgctttcatatctt
ttcctaatcctttctttaatctcagaatttggaggtggtacagggtggacgctctaccca
ccattatccacttcttttatgactttatcaccttcaagtacaggaaatcttatatttgga
ttaataatctctggtatatcttcatgtcttacatctcttaacttttggacaacaattcat
tttctgagatcttattatctgatattatcttctatcccattatttccttgggctttcttg
attacagctttcatgcttttattaacattaccaatcttatctggtacacttcttttaata
ttgggtgatcttcattctaatacacttttctttgatccaatatttggaggagatcctata
ttctatcaacatttattttggtttttggacatccagaagtttacatattaataattcct
gcatttgggatcatttccataataatttctggtattttacagttaataatctttgctaac
caatcaatgatctttgccatgtcatctatttctcttcttggaggtcttgtttggggacat
catatgtatactgtaggtttagaaagtgatacaagagcttattttacaggagttacaatc
ttaatatccttaccaactggtacaaaaatctttaattggctttttacatatctctcaaat
ccaccattattacaccttagaattacttctgtcttcctctcacatctcttttattaatg
tttacgataggtgggtcaacaggaataattcttggaaatggtgcagtggatctaggatta
catgatacatattatgttgtagcacattttcattttgttctttctttaggagctataatt
gctatcttctctggaataatcttgaatggagaaaagattgttgctactaagaatttatta
ctttcatcctcatgtacactctctctttatcatttacatttaatatttattggtattctt
cttaccttttccccaatgcatttcttaggatttaatgttatgccaagaagaatcccatcc
tttccagattcttttcattcctggaattccctgtcatctattggatcaggaataactttc
ctatctttttctatg
>Hd5_cox1
ataaatctcttaaccattaatttcctatttcattcactagttaaaaattgtaatcataaa
ggcttaggaatctattatttattatctggattcatctttggaatctccggtacattaata
tcagtccttataagaatagaattatattcttcaggaaataggattatatctccagaaaac
cagaacttctataatataagcattacattgcatggctttcttatgattttctttttagta
atgcctggcttgtttggaggatttggaaattattttgtacctatctttcaagggtctcca
gaagtggtatatcctagagtcaataatttttctatcttaattcttttgctttcatatctt
ttcctaatcctttctttaatctcagaatttggaggtggtacagggtggacgctctaccca
ccattatccacttcttttatgactttatcaccttcaagtacaggaaatcttatatttgga
ttaataatctctggtatatcttcatgtcttacatctcttaacttttggacaacaattcat

tttctgagatcttattatctgatattatcttctatcccattatttccttgggctttcttg
attacagctttcatgcttttattaacattaccaatcttatctggtacacttcttttaata
ttgggtgatcttcattctaatacacttttctttgatccaatatttggaggagatcctata
ttctatcaacatttattttggtttttggacatccagaagtttacatattaataattcct
gcatttgggatcatttccataataatttctggtattttacagttaataatctttgctaac
caatcaatgatctttgccatgtcatctatttctcttcttggaggtcttgtttggggacat
catatgtatactgtaggtttagaaagtgatacaagagcttattttacaggagttacaatc
ttaatatccttaccaactggtacaaaaatctttaattggctttttacatatctctcaaat
ccaccattattacaccttagaattacttctgtcttcctctcacatctctttttattaatg
tttacgataggtgggtcaacaggaataattcttggaaatggtgcagtggatctaggatta
catgatacatattatgttgtagcacattttcattttgttctttctttaggagctataatt
gctatcttctctggaataatcttgaatggagaaaagattgttgctactaagaatttatta
ctttcatcctcatgtacactctctctttatcatttacatttaatatttattggtattctt
cttaccttttccccaatgcatttcttaggatttaatgttatgccaagaagaatcccatcc
tttccagattcttttcattcctggaattccctgtcatctattggatcaggaataactttc
ctatctttttctatg
>Hd6_cox1
ataaatctcttaaccattaatttcctatttcattcactagttaaaaattgtaatcataaa
ggcttaggaatctattatttattatctggattcatctttggaatctccggtacattaata
tcagtccttataagaatagaattatattcttcaggaaataggattatatctccagaaaac
cagaacttctataatataagcattacattgcatggctttcttatgattttctttttagta
atgcctggcttgtttggaggatttggaaattattttgtacctatctttcaagggtctcca
gaagtggtatatcctagagtcaataatttttctatcttaattcttttgctttcatatctt
ttcctaatcctttctttaatctcagaatttggaggtggtacagggtggacgctctaccca
ccattatccacttcttttatgactttatcaccttcaagtacaggaaatcttatatttgga
ttaataatctctggtatatcttcatgtcttacatctcttaacttttggacaacaattcat
tttctgagatcttattatctgatattatcttctatcccattatttccttgggctttcttg
attacagctttcatgcttttattaacattaccaatcttatctggtacacttcttttaata
ttgggtgatcttcattctaatacacttttctttgatccaatatttggaggagatcctata
ttctatcaacatttattttggtttttggacatccagaagtttacatattaataattcct
gcatttgggatcatttccataataatttctggtattttacagttaataatctttgctaac
caatcaatgatctttgccatgtcatctatttctcttcttggaggtcttgtttggggacat
catatgtatactgtaggtttagaaagtgatacaagagcttattttacaggagttacaatc
ttaatatccttaccaactggtacaaaaatctttaattggctttttacatatctctcaaat
ccaccattattacaccttagaattacttctgtcttcctctcacatctctttttattaatg
tttacgataggtgggtcaacaggaataattcttggaaatggtgcagtggatctaggatta
catgatacatattatgttgtagcacattttcattttgttctttctttaggagctataatt
gctatcttctctggaataatcttgaatggagaaaagattgttgctactaagaatttatta
ctttcatcctcatgtacactctctctttatcatttacatttaatatttattggtattctt
cttaccttttccccaatgcatttcttaggatttaatgttatgccaagaagaatcccatcc
tttccagattcttttcattcctggaattccctgtcatctattggatcaggaataactttc
ctatctttttctatg
>Hd7_cox1
ataaatctcttaaccattaatttcctatttcattcactagttaaaaattgtaatcataaa
ggcttaggaatctattatttattatctggattcatctttggaatctccggtacattaata
tcagtccttataagaatagaattatattcttcaggaaataggattatatctccagaaaac
cagaacttctataatataagcattacattgcatggctttcttatgattttctttttagta
atgcctggcttgtttggaggatttggaaattattttgtacctatctttcaagggtctcca
gaagtggtatatcctagagtcaataatttttctatcttaattcttttgctttcatatctt
ttcctaatcctttctttaatctcagaatttggaggtggtacagggtggacgctctaccca
ccattatccacttcttttatgactttatcaccttcaagtacaggaaatcttatatttgga
ttaataatctctggtatatcttcatgtcttacatctcttaacttttggacaacaattcat
tttctgagatcttattatctgatattatcttctatcccattatttccttgggctttcttg
attacagctttcatgcttttattaacattaccaatcttatctggtacacttcttttaata
ttgggtgatcttcattctaatacacttttctttgatccaatatttggaggagatcctata
ttctatcaacatttattttggtttttggacatccagaagtttacatattaataattcct
gcatttgggatcatttccataataatttctggtattttacagttaataatctttgctaac

caatcaatgatctttgccatgtcatctatttctcttcttggaggtcttgtttggggacat
catatgtatactgtaggtttagaaagtgatacaagagcttattttacaggagttacaatc
ttaatatccttaccaactggtacaaaaatctttaattggctttttacatatctctcaaat
ccaccattattacaccttagaattacttctgtcttcctctcacatctctttttattaatg
tttacgataggtgggtcaacaggaataattcttggaaatggtgcagtggatctaggatta
catgatacatattatgttgtagcacattttcattttgttctttctttaggagctataatt
gctatcttctctggaataatcttgaatggagaaaagattgttgctactaagaatttatta
ctttcatcctcatgtacactctctctttatcatttacatttaatatttattggtattctt
cttaccttttccccaatgcatttcttaggatttaatgttatgccaagaagaatcccatcc
tttccagattcttttcattcctggaattccctgtcatctattggatcaggaataactttc
ctatctttttctatg
>Hd8_cox1
ataaatctcttaaccattaatttcctatttcattcactagttaaaaattgtaatcataaa
ggcttaggaatctattatttattatctggattcatctttggaatctccggtacattaata
tcagtccttataagaatagaattatattcttcaggaaataggattatatctccagaaaac
cagaacttctataatataagcattacattgcatggctttcttatgattttctttttagta
atgcctggcttgtttggaggatttggaaattattttgtacctatctttcaagggtctcca
gaagtggtatatcctagagtcaataatttttctatcttaattcttttgctttcatatctt
ttcctaatcctttctttaatctcagaatttggaggtggtacagggtggacgctctaccca
ccattatccacttcttttatgactttatcaccttcaagtacaggaaatcttatatttgga
ttaataatctctggtatatcttcatgtcttacatctcttaacttttggacaacaattcat
tttctgagatcttattatctgatattatcttctatcccattatttccttgggctttcttg
attacagctttcatgcttttattaacattaccaatcttatctggtacacttcttttaata
ttgggtgatcttcattctaatacacttttctttgatccaatatttggaggagatcctata
ttctatcaacatttattttggttttttggacatccagaagtttacatattaataattcct
gcatttgggatcatttccataataatttctggtattttacagttaataatctttgctaac
caatcaatgatctttgccatgtcatctatttctcttcttggaggtcttgtttggggacat
catatgtatactgtaggtttagaaagtgatacaagagcttattttacaggagttacaatc
ttaatatccttaccaactggtacaaaaatctttaattggctttttacatatctctcaaat
ccaccattattacaccttagaattacttctgtcttcctctcacatctctttttattaatg
tttacgataggtgggtcaacaggaataattcttggaaatggtgcagtggatctaggatta
catgatacatattatgttgtagcacattttcattttgttctttctttaggagctataatt
gctatcttctctggaataatcttgaatggagaaaagattgttgctactaagaatttatta
ctttcatcctcatgtacactctctctttatcatttacatttaatatttattggtattctt
cttaccttttccccaatgcatttcttaggatttaatgttatgccaagaagaatcccatcc
tttccagattcttttcattcctggaattccctgtcatctattggatcaggaataactctc
ctatctttttctatg
>Hd9_cox1
ataaatctcttaaccattaatttcctatttcattcactagttaaaaattgtaatcataaa
ggcttaggaatctattatttattatctggattcatctttggaatctccggtacattaata
tcagtccttataagaatagaattatattcttcaggaaataggattatatctccagaaaac
cagaacttctataatataagcattacattgcatggctttcttatgattttctttttagta
atgcctggcttgtttggaggatttggaaattattttgtacctatctttcaagggtctcca
gaagtggtatatcctagagtcaataatttttctatcttaattcttttgctttcatatctt
ttcctaatcctttctttaatctcagaatttggaggtggtacagggtggacgctctaccca
ccattatccacttcttttatgactttatcaccttcaagtacaggaaatcttatatttgga
ttaataatctctggtatatcttcatgtcttacatctcttaacttttggacaacaattcat
tttctgagatcttattatctgatattatcttctatcccattatttccttgggctttcttg
attacagctttcatgcttttattaacattaccaatcttatctggtacacttcttttaata
ttgggtgatcttcattctaatacacttttctttgatccaatatttggaggagatcctata
ttctatcaacatttattttggttttttggacatccagaagtttacatattaataattcct
gcatttgggatcatttccataataatttctggtattttacagttaataatctttgctaac
caatcaatgatctttgccatgtcatctatttctcttcttggaggtcttgtttggggacat
catatgtatactgtaggtttagaaagtgatacaagagcttattttacaggagttacaatc
ttaatatccttaccaactggtacaaaaatctttaattggctttttacatatctctcaaat
ccaccattattacaccttagaattacttctgtcttcctctcacatctctttttattaatg
tttacgataggtgggtcaacaggaataattcttggaaatggtgcagtggatctaggatta

catgatacatattatgttgtagcacattttcattttgttctttctttaggagctataatt
gctatcttctctggaataatcttgaatggagaaaagattgttgctactaagaatttatta
ctttcatcctcatgtacactctctctttatcatttacatttaatatttattggtattctt
cttaccttttccccaatgcatttcttaggatttaatgttatgccaagaagaatcccatcc
tttccagattcttttcattcctggaattccctgtcatctattggatcaggaataactttc
ctatctttttctatg
>IS1_cox1
ataaatctcttaaccattaatttcctatttcattcattagttaaaaattgtaatcataaa
ggcttaggaatctattatttattatctggattcatctttggaatctccggtacattaata
tcagtccttatgagaatagaattatattcttcaggaaataggattatatctccagaaaac
cagaacttctataatataagcattacattgcatggccttcttatgattttctttttagta
atgcctggcttgtttggaggatttggaaattattttgtacctatctttcaagggtctcca
gaagtggtatatcctagagtcaataatttttctatcttaattattttgctttcatatctt
ttcctaatcctttctttaatctcagaatttggaggtggtacaggatggacgctctaccca
ccattatccacttcttttatgactttatcaccttcaagtacaggaaatcttatatttgga
ttaataatctctggtatatcttcatgtcttacatctcttaacttttggacaactattcat
ttattgagatcttattctctgatattatcttctgtcccattatttccttgggctttcttg
attacagctttcatgcttttattaacattaccagtcttatctggtacacttcttttagta
ttgggtgatcttcattcaaatacacttttctttgatccagtatttggaggagatcctgta
ctctatcaacatttattttggtttttggacatccagaagtttacatattaataattcct
gcatttgggatcatttccataataatttctggtgttttacaattaattatttttgctaac
caatcaatgatctttgccatgtcatctatttctcttcttggaggtcttgtttggggacat
catatgtatactgtaggtttagaaagtgatacaagagcttattttacaggagttacaatc
ttaatatccttaccaactggtacaaaaatatttaattggctttttacatatctctccaat
ccaccattattacaccttaggattacttctgtcttcctctcacatctatttttattaatg
tttacggtaggtgggtcaacaggagtaattcttggaaatggtgcagtggatctaggatta
catgatacatattatgttgtagcacattttcattttgttctttctttaggagctataatt
gctatattctctggaatagtcttgaatggagaaaagattgttgctactaagagtttatta
ctttcatcctcatgtacactctctctttatcatttacatttaatatttattggtattctt
cttaccttttcaccaatgcatttcttaggatttaatgtaatgccaagaagaatcccatcc
tttccagattcttttcattcctggaattccctgtcatctattggatcaggaataactttc
ctatctttttctatg
>IS10_cox1
ttaaatctcttaaccattaatttcctatttcattcactagttaaaaattgtaatcataaa
ggcttaggaatctattatttattatctggattcatctttggaatctccggtacattaata
tcagtccttataagaatagaattatattcttcaggaaataggattatatctccagaaaac
cagaacttctataatataagcattacattgcatggctttcttatgattttctttttagta
atgcctggcttgtttggaggatttggaaattattttgtacctatctttcaagggtctcca
gaagtggtatatcctagagtcaataatttttctatcttaattcttttgctttcatatctt
ttcctaatcctttctttaatctcagaatttggaggtggtacagggtggacgctctaccca
ccattatccacttcttttatgactttatcaccttcaagtacaggaaatcttatatttgga
ttaataatctctggtatatcttcatgtcttacatctcttaacttttggacaacaattcat
tttctgagatcttattatctgatattatcttctatcccattatttccttgggctttcttg
attacagctttcatgcttttattaacattaccaatcttatctggtacacttcttttaata
ttgggtgatcttcattctaatacacttttctttgatccaatatttggaggagatcctata
ttctatcaacatttattttggtttttggacatccagaagtttacatattaataattcct
gcatttgggatcatttccataataatttctggtattttacagttaataatctttgctaac
caatcaatgatctttgccatgtcatctatttctcttcttggaggtcttgtttggggacat
catatgtatactgtaggtttagaaagtgatacaagagcttattttacaggagttacaatc
ttaatatccttaccaactggtacaaaaatctttaattggctttttacatatctctcaaat
ccaccattattacaccttagaattacttctgtcttcctctcacatctctttttattaatg
tttacgataggtgggtcaacaggaataattcttggaaatggtgcagtggatctaggatta
catgatacatattatgttgtagcacattttcattttgttctttctttaggagctataatt
gctatcttctctggaataatcttgaatggagaaaagattgttgctactaagaatttatta
ctttcatcctcatgtacactctctctttatcatttacatttaatatttattggtattctt
cttaccttttccccaatgcatttcttaggatttaatgttatgccaagaagaatcccatcc
tttccagattcttttcattcctggaattccctgtcatctattggatcaggaataactttc

ctatctttttctatg
>IS2_cox1
ataaatctcttaaccattaatttcctatttcattcactagttaaaaattgtaatcataaa
ggcttaggaatctattatttattatctggattcatctttggaatctccggtacattaata
tcagtccttataagaatagaattatattcttcaggaaataggattatatctccagaaaac
cagaacttctataatataagcattacattgcatggctttcttatgattttctttttagta
atgcctggcttgtttggaggatttggaaattattttgtacctatctttcaagggtctcca
gaagtggtatatcctagagtcaataatttttctatcttaattcttttgctttcatatctt
ttcctaatcctttctttaatctcagaatttggaggtggtacagggtggacgctctaccca
ccattatccacttcttttatgactttatcaccttcaagtacaggaaatcttatatttgga
ttaataatctctggtatatcttcatgtcttacatctcttaacttttggacaacaattcat
tttctgagatcttattatctgatattatcttctatcccattatttccttgggctttcttg
attacagctttcatgcttttattaacattaccaatcttatctggtacacttcttttaata
ttgggtgatcttcattctaatacacttttctttgatccaatatttggaggagatcctata
ttctatcaacatttattttggttttttggacatccagaagtttacatattaataattcct
gcatttgggatcatttccataataatttctggtattttacagttaataatctttgctaac
caatcaatgatctttgccatgtcatctatttctcttcttggaggtcttgtttggggacat
catatgtatactgtaggtttagaaagtgatacaagagcttattttacaggagttacaatc
ttaatatccttaccaactggtacaaaaatctttaattggctttttacatatctctcaaat
ccaccattattacaccttagaattacttctgtcttcctctcacatctctttttattaatg
tttacgataggtgggtcaacaggaataattcttggaaatggtgcagtggatctaggatta
catgatacatattatgttgtagcacattttcattttgttctttctttaggagctataatt
gctatcttctctggaataatcttgaatggagaaaagattgttgctactaagaatttatta
ctttcatcctcatgtacactctctctttatcatttacatttaatatttattggtattctt
cttaccttttccccaatgcatttcttaggatttaatgttatgccaagaagaatcccatcc
tttccagattcttttcattcctggaattccctgtcatctattggatcaggaataactttc
ctatctttttctatg
>IS3_cox1
ataaatctcttaaccattaatttcctatttcattcactagttaaaaattgtaatcataaa
ggcttaggaatctattatttattatctggattcatctttggaatctccggtacattaata
tcagtccttatgagaatagaattatattcttcaggaaataggattatatctccagaaaac
cagaacttctataatgtaagcattacattgcatggccttcttatgattttctttttagta
atgcctggcttgtttggaggatttggaaattattttgtacctatctttcaagggtctcca
gaagtggtatatcctagagtcaataatttttctatcttaattcttttcctttcatatctt
ttcctaatcctttctttaatctcagaatttggaggtggtacaggatggacgctctaccca
ccattatccacttcttttatgactttatcaccttcaagtgtaggaaatcttatatttgga
ttaataatctctggtatatcttcatctcttacatctcttaacttttggacaactattcat
ttattgagatcttattatctgatattatcttctattccattatttccttgggctttcttg
attacagctttcatgcttttattaacattaccaatcttatctggaacacttcttttaata
ttgggtgatcttcattcaaatacacttttctttgatccagtatttggaggagatcctata
ttctatcaacatttattttggttttttggacatccagaagtttacatattaataattcct
gcatttggggtcatttccatagtaatttctggggtttcacaattaattatttttgctaac
caatcaatgatctttgccatgtcatctatttctcttcttggaggtcttgtttggggacat
catatgtatactgtaggtttagaaagtgatacaagagcttattttacaggagttacaata
ttaatatccttaccaactggtacaaaaatatttaattggctttttacatatctctccaat
ccaccattattacaccttagaattacttctgtcttcctctcacatctctttttattaatg
tttacgataggtgggtcaacaggaataattcttggaaatggtgcagtggatctaggatta
catgatacatattatgttgtagcacattttcattttgttctttctttaggagctataatt
gctatattctctggaataatcttgaatggagaaaagattgttgctaataagaatttatta
ctttcatcctcatgtacactctctctttatcatttacatttagtatttgttggtattctt
cttaccttttccccaatgcatttcttaggatttaatcttatgccaagaagaatcccatcc
tttccagattcttttcattcctggaattccctgtcatctattggatcaggaataactctc
ctatctttttctatg
>IS4_cox1
ataaatctcttaaccattaatttcctatttcattcactagttaaaaattgtaatcataaa
ggcttaggaatctattatttattatctggattcatctttggaatctccggtacattaata
tcagtccttataagaatagaattatattcttcaggaaataggattatatctccagaaaac

cagaacttctataatataagcattacattgcatggctttcttatgattttctttttagta
atgcctggcttgtttggaggatttggaaattattttgtacctatctttcaagggtctcca
gaagtggtatatcctagagtcaataatttttctatcttaattattttgctttcatatctt
ttcctaatcctttctttaatctcagaatttggaggtggtacaggatggacgctctaccca
ccattatccacttcttttatgactttatcaccttcaagtacaggaaatcttatatttgga
ttaataatctctggtatatcttcatgtcttacatctcttaacttttggacaactattcat
ttattgagatcttattatctgatattatcttctattccattatttccttgggctttcttg
attacagctttcatgcttttattaacattaccaatcttatctggtacacttcttttaata
ttgggtgatcttcattctaatacacttttctttgatccagtatttggaggagatcctata
ttctatcaacatttattttggtttttggacatccagaagtttacatattaataattcct
gcatttgggatcatttccataataatttctggtattttacaattaattatttttgctaac
caatcaatgatctttgccatgtcatctatttctcttcttggaggtcttgtttggggacat
catatgtatactgtaggtttagaaagtgatacaagagcttattttacaggagttacaatc
ttaatatccttaccaactggtacaaaaaatctttaattggctttttacatatctctcaaat
ccaccattattacaccttaggattacttctgtcttcctctcacatctatttttattaatg
tttacgataggtgggtcaacaggaataattcttggaaatggtgcagtggatctaggatta
catgatacatattatgttgtagcacattttcattttgttctttctttaggagctataatt
gctatcttctctggaataatcttgaatggagaaaagattgttgctactaagaatttatta
ctttcatcctcatgtacactctctctttatcatttacatttaatatttattggtattctt
cttacctttccccaatgcatttcttaggatttaatgttatgccaagaagaatcccatcc
tttccagattcttttcattcctggaattccctgtcatctattggatcaggaataactttc
ctatctttttctatg
>IS5_cox1
ataaatctcttaaccattaatttcctatttcattcactagttaaaaattgtaatcataaa
ggcttaggaatctattatttattatctggattcatctttggaatctccggtacattaata
tcagtccttataagaatagaattatattcttcaggaaataggattatatctccagaaaac
cagaacttctataatataagcattacattgcatggctttcttatgattttctttttagta
atgcctggcttgtttggaggatttggaaattattttgtacctatctttcaagggtctcca
gaagtggtatatcctagagtcaataatttttctatcttaattcttttgctttcatatctt
ttcctaatcctttctttaatctcagaatttggaggtggtacagggtggacgctctaccca
ccattatccacttcttttatgactttatcaccttcaagtacaggaaatcttatatttgga
ttaataatctctggtatatcttcatgtcttacatctcttaacttttggacaacaattcat
tttctgagatcttattatctgatattatcttctatcccattatttccttgggctttcttg
attacagctttcatgcttttattaacattaccaatcttatctggtacacttcttttaata
ttgggtgatcttcattctaatacacttttctttgatccaatatttggaggagatcctata
ttctatcaacatttattttggtttttggacatccagaagtttacatattaataattcct
gcatttgggatcatttccataataatttctggtattttacagttaataatctttgctaac
caatcaatgatctttgccatgtcatctatttctcttcttggaggtcttgtttggggacat
catatgtatactgtaggtttagaaagtgatacaagagcttattttacaggagttacaatc
ttaatatccttaccaactggtacaaaaaatctttaattggctttttacatatctctcaaat
ccaccattattacaccttagaattacttctgtcttcctctcacatctctttttattaatg
tttacgataggtgggtcaacaggaataattcttggaaatggtgcagtggatctaggatta
catgatacatattatgttgtagcacattttcattttgttctttctttaggagctataatt
gctatcttctctggaataatcttgaatggagaaaagattgttgctactaagaatttatta
ctttcatcctcatgtacactctctctttatcatttacatttaatatttattggtattctt
cttacctttccccaatgcatttcttaggatttaatgttatgccaagaagaatcccatcc
tttccagattcttttcattcctggaattccctgtcatctattggatcaggaataactttc
ctatctttttctatg
>IS6_cox1
ataaatctcttaaccattaatttcctatttcattcactagttaaaaattgtaatcataaa
ggcttaggaatctattatttattatctggattcatctttggaatctccggtacattaata
tcagtccttataagaatagaattatattcttcaggaaataggattatatctccagaaaac
cagaacttctataatataagcattacattgcatggctttcttatgattttctttttagta
atgcctggcttgtttggaggatttggaaattattttgtacctatctttcaagggtctcca
gaagtggtatatcctagagtcaataatttttctatcttaattcttttgctttcatatctt
ttcctaatcctttctttaatctcagaatttggaggtggtacagggtggacgctctaccca
ccattatccacttcttttatgactttatcaccttcaagtacaggaaatcttatatttgga

ttaataatctctggtatatcttcatgtcttacatctcttaacttttggacaacaattcat
tttctgagatcttattatctgatattatcttctatcccattatttccttgggctttcttg
attacagctttcatgcttttattaacattaccaatcttatctggtacacttcttttaata
ttgggtgatcttcattctaatacacttttctttgatccaatatttggaggagatcctata
ttctatcaacatttattttggttttttggacatccagaagtttacatattaataattcct
gcatttgggatcatttccataataatttctggtattttacagttaataatctttgctaac
caatcaatgatctttgccatgtcatctatttctcttcttggaggtcttgtttggggacat
catatgtatactgtaggtttagaaagtgatacaagagcttattttacaggagttacaatc
ttaatatccttaccaactggtacaaaaatctttaattggctttttacatatctctcaaat
ccaccattattacaccttagaattacttctgtcttcctctcacatctcttttttattaatg
tttacgataggtgggtcaacaggaataattcttggaaatggtgcagtggatctaggatta
catgatacatattatgttgtagcacattttcattttgttctttctttaggagctataatt
gctatcttctctggaataatcttgaatggagaaaagattgttgctactaagaatttatta
ctttcatcctcatgtacactctctctttatcatttacatttaatatttattggtattctt
cttacctttttccccaatgcatttcttaggatttaatgttatgccaagaagaatcccatcc
tttccagattcttttcattcctggaattccctgtcatctattggatcaggaataactttc
ctatctttttctatg
>IS7_cox1
ataaatctcttaaccattaatttcctatttcattcactagttaaaaattgtaatcataaa
ggcttaggaatctattatttattatctggattcatctttggaatctccggtacattaata
tcagtccttataagaatagaattatattcttcaggaaataggattatatctccagaaaac
cagaacttctataatataagcattacattgcatggctttcttatgattttctttttagta
atgcctggcttgtttggaggatttggaaattattttgtacctatctttcaagggtctcca
gaagtggtatatcctagagtcaataattttttctatcttaattattttgctttcatatctt
ttcctaatcctttctttaatctcagaatttggaggtggtacaggatggacgctctaccca
ccattatccacttcttttatgactttatcaccttcaagtacaggaaatcttatatttgga
ttaataatctctggtatatcttcatgtcttacatctcttaacttttggacaactattcat
ttattgagatcttattatctgatattatcttctattccattatttccttggtctttcttg
attacagctttcatgcttttattaacattaccaatcttatctggaacacttcttttaata
ttgggtgatcttcattctaatacacttttctttgatccaatatttggaggagatcctata
ttctatcaacatttattttggttttttggacatccagaagtttacatattaataattcct
gcatttgggatcatttccataataatttctggtattttacaattaattatttttgctaac
caatcaatgatctttgccatgtcatctatttctcttcttggaggtcttgtttggggacat
catatgtatactgtaggtttagaaagtgatacaagagcttattttacaggagttacaatt
ttaatatccttaccaactggtacaaaaatatttaattggctttttacatatctctcaaat
ccaccattattacaccttagaattacttctgtcttcctctcacatctatttttattaatg
tttacgataggtgggtcaacaggaataattcttggaaatggtgcagtggatctaggatta
catgatacatattatgttgtagcacattttcattttgttctttctttaggagctataatt
gctatattctctggaataatcttgaatggagaaaagattgttgctactaagaatttatta
ctttcatcctcatgtacactctctctttatcatttacatttaatatttattggtattctt
cttacctttttccccaatgcatttcttaggatttaatgttatgccaagaagaatcccatcc
tttccagattcttttcattcctggaattccctgtcatctattggatcaggaataactttc
ctatctttttctatg
>IS8_cox1
ataaatctcttaaccattaatttcctatttcattcactagttaaaaattgtaatcataaa
ggcttaggaatctattatttattatctggattcatctttggaatctccggtacattaata
tcagtccttataagaatagaattatattcttcaggaaataggattatatctccagaaaac
cagaacttctataatataagcattacattgcatggctttcttatgattttctttttagta
atgcctggcttgtttggaggatttggaaattattttgtacctatctttcaagggtctcca
gaagtggtatatcctagagtcaataattttttctatcttaattcttttgctttcatatctt
ttcctaatcctttctttaatctcagaatttggaggtggtacagggtggacgctctaccca
ccattatccacttcttttatgactttatcaccttcaagtacaggaaatcttatatttgga
ttaataatctctggtatatcttcatgtcttacatctcttaacttttggacaacaattcat
tttctgagatcttattatctgatattatcttctatcccattatttccttgggctttcttg
attacagctttcatgcttttattaacattaccaatcttatctggtacacttcttttaata
ttgggtgatcttcattctaatacacttttctttgatccaatatttggaggagatcctata
ttctatcaacatttattttggttttttggacatccagaagtttacatattaataattcct

gcatttgggatcatttccataataatttctggtattttacagttaataatctttgctaac
caatcaatgatctttgccatgtcatctatttctcttcttggaggtcttgtttggggacat
catatgtatactgtaggtttagaaagtgatacaagagcttattttacaggagttacaatc
ttaatatccttaccaactggtacaaaaatctttaattggctttttacatatctctcaaat
ccaccattattacaccttagaattacttctgtcttcctctcacatctcttttattaatg
tttacgataggtgggtcaacaggaataattcttggaaatggtgcagtggatctaggatta
catgatacatattatgttgtagcacattttcattttgttctttctttaggagctataatt
gctatcttctctggaataatcttgaatggagaaaagattgttgctactaagaatttatta
ctttcatcctcatgtacactctctctttatcatttacatttaatatttattggtattctt
cttaccttttccccaatgcatttcttaggatttaatgttatgccaagaagaatcccatcc
tttccagattcttttcattcctggaattccctgtcatctattggatcaggaataactttc
ctatctttttctatg
>IS9_cox1
ataaatctcttaaccattaatttcctatttcattcactagttaaaaattgtaatcataaa
ggcttaggaatctattatttattatctggattcatctttggaatctccggtacattaata
tcagtccttataagaatagaattatattcttcaggaaataggattatatctccagaaaac
cagaacttctataatataagcattacattgcatggctttcttatgattttctttttagta
atgcctggcttgtttggaggatttggaaattattttgtacctatctttcaagggtctcca
gaagtggtatatcctagagtcaataatttttctatcttaattcttttcctttcatatctt
ttcctaatcctttctttaatctcagaatttggaggtggtacagggtggacgctctaccca
ccattatccacttcttttatgactttatcaccttcaagtacaggaaatcttatatttgga
ttaataatctctggtatatcttcatgtcttacatctcttaacttttggacaacaattcat
tttctgagatcttattatctgatattatcttctatcccattatttccttgggctttcttg
attacagctttcatgcttttattaacattaccaatcttatctggaacacttcttttaata
ttgggtgatcttcattctaatacacttttctttgatccaatatttggaggagatcctata
ttctatcaacatttattttggtttttggacatccagaagtttacatattaataattcct
gcatttgggatcatttccataataatttctggtattttacagttaataatctttgctaac
caatcaatgatctttgccatgtcatctatttctcttcttggaggtcttgtttggggacat
catatgtatactgtaggtttagaaagtgatacaagagcttattttacaggagttacaatc
ttaatatccttaccaactggtacaaaaatatttaattggctttttacatatctctcaaat
ccaccattattacaccttagaattacttctgtcttcctctcacatctattttttattaatg
tttacgataggtgggtcaacaggaataattcttggaaatggtgcagtggatctaggatta
catgatacatattatgttgtagcacattttcattttgttctttctttaggagctataatt
gctatcttctctggaataatcttgaatggagaaaagattgttgctaataagaatttatta
ttttcatcctcatgtacactctctctttatcatttacatttaatatttattggtattctt
cttaccttttccccaatgcatttcttaggatttaatgttatgccaagaagaatcccatcc
tttccagattcttttcattcctggaattccctgtcatctattggatcaggaataactttc
ctatctttttctatg
>Ik1_cox1
ataaatctcttaaccattaatttcctatttcattcactagttaaaaattgtaatcataaa
ggcttaggaatctattatttattatctggattcatctttggaatctccggtacattaata
tcagtccttataagaatagaattatattcttcaggaaataggattatatctccagaaaac
cagaacttctataatataagcattacattgcatggctttcttatgattttctttttagta
atgcctggcttgtttggaggatttggaaattattttgtacctatctttcaagggtctcca
gaagtggtatatcctagagtcaataatttttctatcttaattcttttgctttcatatctt
ttcctaatcctttctttaatctcagaatttggaggtggtacagggtggacgctctaccca
ccattatccacttcttttatgactttatcaccttcaagtacaggaaatcttatatttgga
ttaataatctctggtatatcttcatgtcttacatctcttaacttttggacaacaattcat
tttctgagatcttattatctgatattatcttctatcccattatttccttgggctttcttg
attacagctttcatgcttttattaacattaccaatcttatctggtacacttcttttaata
ttgggtgatcttcattctaatacacttttctttgatccaatatttggaggagatcctata
ttctatcaacatttattttggtttttggacatccagaagtttacatattaataattcct
gcatttgggatcatttccataataatttctggtattttacagttaataatctttgctaac
caatcaatgatctttgccatgtcatctatttctcttcttggaggtcttgtttggggacat
catatgtatactgtaggtttagaaagtgatacaagagcttattttacaggagttacaatc
ttaatatccttaccaactggtacaaaaatctttaattggctttttacatatctctcaaat
ccaccattattacaccttagaattacttctgtcttcctctcacatctcttttattaatg

tttacgataggtgggtcaacaggaataattcttggaaatggtgcagtggatctaggatta
catgatacatattatgttgtagcacattttcattttgttctttctttaggagctataatt
gctatcttctctggaataatcttgaatggagaaaagattgttgctactaagaatttatta
ctttcatcctcatgtacactctctctttatcatttacatttaatatttattggtattctt
cttaccttttccccaatgcatttcttaggatttaatgttatgccaagaagaatcccatcc
tttccagattcttttcattcctggaattccctgtcatctattggatcaggaataactttc
ctatctttttctatg
>Ik2_cox1
ataaatctcttaaccattaatttcctatttcattcactagttaaaaattgtaatcataaa
ggcttaggaatctattatttattatctggattcatctttggaatctccggtacattaata
tcagtccttataagaatagaattatattcttcaggaaataggattatatctccagaaaac
cagaacttctataatataagcattacattgcatggctttcttatgattttctttttagta
atgcctggcttgtttggaggatttggaaattattttgtacctatctttcaagggtctcca
gaagtggtatatcctagagtcaataatttttctatcttaattcttttgctttcatatctt
ttcctaatcctttctttaatctcagaatttggaggtggtacagggtggacgctctaccca
ccattatccacttcttttatgactttatcaccttcaagtacaggaaatcttatatttgga
ttaataatctctggtatatcttcatgtcttacatctcttaacttttggacaacaattcat
tttctgagatcttattatctgatattatcttctatcccattatttccttgggctttcttg
attacagctttcatgcttttattaacattaccaatcttatctggtacacttcttttaata
ttgggtgatcttcattctaatacacttttctttgatccaatatttggaggagatcctata
ttctatcaacatttattttggttttttggacatccagaagtttacatattaataattcct
gcatttgggatcatttccataataatttctggtattttacagttaataatctttgctaac
caatcaatgatctttgccatgtcatctatttctcttcttggaggtcttgtttggggacat
catatgtatactgtaggtttagaaagtgatacaagagcttattttacaggagttacaatc
ttaatatccttaccaactggtacaaaaatctttaattggctttttacatatctctcaaat
ccaccattattacaccttagaattacttctgtcttcctctcacatctctttttattaatg
tttacgataggtgggtcaacaggaataattcttggaaatggtgcagtggatctaggatta
catgatacatattatgttgtagcacattttcattttgttctttctttaggagctataatt
gctatcttctctggaataatcttgaatggagaaaagattgttgctactaagaatttatta
ctttcatcctcatgtacactctctctttatcatttacatttaatatttattggtattctt
cttaccttttccccaatgcatttcttaggatttaatgttatgccaagaagaatcccatcc
tttccagattcttttcattcctggaattccctgtcatctattggatcaggaataactttc
ctatctttttctatg
>Ik3_cox1
ataaatctcttaaccattaatttcctatttcattcactagttaaaaattgtaatcataaa
ggcttaggaatctattatttattatctggattcatctttggaatctccggtacattaata
tcagtccttataagaatagaattatattcttcaggaaataggattatatctccagaaaac
cagaacttctataatataagcattacattgcatggctttcttatgattttctttttagta
atgcctggcttgtttggaggatttggaaattattttgtacctatctttcaagggtctcca
gaagtggtatatcctagagtcaataatttttctatcttaattcttttgctttcatatctt
ttcctaatcctttctttaatctcagaatttggaggtggtacagggtggacgctctaccca
ccattatccacttcttttatgactttatcaccttcaagtacaggaaatcttatatttgga
ttaataatctctggtatatcttcatgtcttacatctcttaacttttggacaacaattcat
tttctgagatcttattatctgatattatcttctatcccattatttccttgggctttcttg
attacagctttcatgcttttattaacattaccaatcttatctggtacacttcttttaata
ttgggtgatcttcattctaatacacttttctttgatccaatatttggaggagatcctata
ttctatcaacatttattttggttttttggacatccagaagtttacatattaataattcct
gcatttgggatcatttccataataatttctggtattttacagttaataatctttgctaac
caatcaatgatctttgccatgtcatctatttctcttcttggaggtcttgtttggggacat
catatgtatactgtaggtttagaaagtgatacaagagcttattttacaggagttacaatc
ttaatatccttaccaactggtacaaaaatctttaattggctttttacatatctctcaaat
ccaccattattacaccttagaattacttctgtcttcctctcacatctctttttattaatg
tttacgataggtgggtcaacaggaataattcttggaaatggtgcagtggatctaggatta
catgatacatattatgttgtagcacattttcattttgttctttctttaggagctataatt
gctatcttctctggaataatcttgaatggagaaaagattgttgctactaagaatttatta
ctttcatcctcatgtacactctctctttatcatttacatttaatatttattggtattctt
cttaccttttccccaatgcatttcttaggatttaatgttatgccaagaagaatcccatcc

tttccagattcttttcattcctggaattccctgtcatctattggatcaggaataactttc
ctatctttttctatg
>Ik4_cox1
ataaatctcttaaccattaatttcctatttcattcactagttaaaaattgtaatcataaa
ggcttaggaatctattatttattatctggattcatctttggaatctccggtacattaata
tcagtccttataagaatagaattatattcttcaggaaataggattatatctccagaaaac
cagaacttctataatataagcattacattgcatggctttcttatgattttctttttagta
atgcctggcttgtttggaggatttggaaattattttgtacctatctttcaagggtctcca
gaagtggtatatcctagagtcaataatttttctatcttaattcttttgctttcatatctt
ttcctaatcctttctttaatctcagaatttggaggtggtacagggtggacgctctaccca
ccattatccacttcttttatgactttatcaccttcaagtacaggaaatcttatatttgga
ttaataatctctggtatatcttcatgtcttacatctcttaacttttggacaacaattcat
tttctgagatcttattatctgatattatcttctatcccattatttccttgggctttcttg
attacagctttcatgcttttattaacattaccaatcttatctggtacacttcttttaata
ttgggtgatcttcattctaatacacttttctttgatccaatatttggaggagatcctata
ttctatcaacatttattttggtttttggacatccagaagtttacatattaataattcct
gcatttgggatcatttccataataatttctggtattttacagttaataatctttgctaac
caatcaatgatctttgccatgtcatctatttctcttcttggaggtcttgtttggggacat
catatgtatactgtaggtttagaaagtgatacaagagcttattttacaggagttacaatc
ttaatatccttaccaactggtacaaaaatctttaattggctttttacatatctctcaaat
ccaccattattacaccttagaattacttctgtcttcctctcacatctcttttattaatg
tttacgataggtgggtcaacaggaataattcttggaaatggtgcagtggatctaggatta
catgatacatattatgttgtagcacattttcattttgttctttctttaggagctataatt
gctatcttctctggaataatcttgaatggagaaaagattgttgctactaagaatttatta
ctttcatcctcatgtacactctctctttatcatttacatttaatatttattggtattctt
cttaccttttccccaatgcatttcttaggatttaatgttatgccaagaagaatcccatcc
tttccagattcttttcattcctggaattccctgtcatctattggatcaggaataactttc
ctatctttttctatg
>Ik5_cox1
ataaatctcttaaccattaatttcctatttcattcactagttaaaaattgtaatcataaa
ggcttaggaatctattatttattatctggattcatctttggaatctccggtacattaata
tcagtccttataagaatagaattatattcttcaggaaataggattatatctccagaaaac
cagaacttctataatataagcattacattgcatggctttcttatgattttctttttagta
atgcctggcttgtttggaggatttggaaattattttgtacctatctttcaagggtctcca
gaagtggtatatcctagagtcaataatttttctatcttaattcttttgctttcatatctt
ttcctaatcctttctttaatctcagaatttggaggtggtacagggtggacgctctaccca
ccattatccacttcttttatgactttatcaccttcaagtacaggaaatcttatatttgga
ttaataatctctggtatatcttcatgtcttacatctcttaacttttggacaacaattcat
tttctgagatcttattatctgatattatcttctatcccattatttccttgggctttcttg
attacagctttcatgcttttattaacattaccaatcttatctggtacacttcttttaata
ttgggtgatcttcattctaatacacttttctttgatccaatatttggaggagatcctata
ttctatcaacatttattttggtttttggacatccagaagtttacatattaataattcct
gcatttgggatcatttccataataatttctggtattttacagttaataatctttgctaac
caatcaatgatctttgccatgtcatctatttctcttcttggaggtcttgtttggggacat
catatgtatactgtaggtttagaaagtgatacaagagcttattttacaggagttacaatc
ttaatatccttaccaactggtacaaaaatctttaattggctttttacatatctctcaaat
ccaccattattacaccttagaattacttctgtcttcctctcacatctcttttattaatg
tttacgataggtgggtcaacaggaataattcttggaaatggtgcagtggatctaggatta
catgatacatattatgttgtagcacattttcattttgttctttctttaggagctataatt
gctatcttctctggaataatcttgaatggagaaaagattgttgctactaagaatttatta
ctttcatcctcatgtacactctctctttatcatttacatttaatatttattggtattctt
cttaccttttccccaatgcatttcttaggatttaatgttatgccaagaagaatcccatcc
tttccagattcttttcattcctggaattccctgtcatctattggatcaggaataactttc
ctatctttttctatg
>Ik6_cox1
ataaatctcttaaccattaatttcctatttcattcactagttaaaaattgtaatcataaa
ggcttaggaatctattatttattatctggattcatctttggaatctccggtacattaata

tcagtccttataagaatagaattatattcttcaggaaataggattatatctccagaaaac
cagaacttctataatataagcattacattgcatggctttcttatgattttctttttagta
atgcctggcttgtttggaggatttggaaattattttgtacctatctttcaagggtctcca
gaagtggtatatcctagagtcaataatttttctatcttaattcttttgctttcatatctt
ttcctaatcctttctttaatctcagaatttggaggtggtacagggtggacgctctaccca
ccattatccacttcttttatgactttatcaccttcaagtacaggaaatcttatatttgga
ttaataatctctggtatatcttcatgtcttacatctcttaacttttggacaacaattcat
tttctgagatcttattatctgatattatcttctatcccattatttccttgggctttcttg
attacagctttcatgcttttattaacattaccaatcttatctggtacacttcttttaata
ttgggtgatcttcattctaatacacttttctttgatccaatatttggaggagatcctata
ttctatcaacatttattttggttttttggacatccagaagtttacatattaataattcct
gcatttgggatcatttccataataatttctggtattttacagttaataatctttgctaac
caatcaatgatctttgccatgtcatctatttctcttcttggaggtcttgtttggggacat
catatgtatactgtaggtttagaaagtgatacaagagcttattttacaggagttacaatc
ttaatatccttaccaactggtacaaaaatctttaattggctttttacatatctctcaaat
ccaccattattacaccttagaattacttctgtcttcctctcacatctctttttattaatg
tttacgataggtgggtcaacaggaataattcttggaaatggtgcagtggatctaggatta
catgatacatattatgttgtagcacattttcattttgttctttctttaggagctataatt
gctatcttctctggaataatcttgaatggagaaaagattgttgctactaagaatttatta
ctttcatcctcatgtacactctctctttatcatttacatttaatatttattggtattctt
cttaccttttccccaatgcatttcttaggatttaatgttatgccaagaagaatcccatcc
tttccagattcttttcattcctggaattccctgtcatctattggatcaggaataactttc
ctatctttttctatg
>Ik7_cox1
ttaaatctcttaaccattaatttcctatttcattcactagttaaaaattgtaatcataaa
ggcttaggaatctattatttattatctggattcatctttggaatctccggtacattaata
tcagtccttataagaatagaattatattcttcaggaaataggattatatctccagaaaac
cagaacttctataatataagcattacattgcatggctttcttatgattttctttttagta
atgcctggcttgtttggaggatttggaaattattttgtacctatctttcaagggtctcca
gaagtggtatatcctagagtcaataatttttctatcttaattcttttgctttcatatctt
ttcctaatcctttctttaatctcagaatttggaggtggtacagggtggacgctctaccca
ccattatccacttcttttatgactttatcaccttcaagtacaggaaatcttatatttgga
ttaataatctctggtatatcttcatgtcttacatctcttaacttttggacaacaattcat
tttctgagatcttattatctgatattatcttctatcccattatttccttgggctttcttg
attacagctttcatgcttttattaacattaccaatcttatctggtacacttcttttaata
ttgggtgatcttcattctaatacacttttctttgatccaatatttggaggagatcctata
ttctatcaacatttattttggttttttggacatccagaagtttacatattaataattcct
gcatttgggatcatttccataataatttctggtattttacagttaataatctttgctaac
caatcaatgatctttgccatgtcatctatttctcttcttggaggtcttgtttggggacat
catatgtatactgtaggtttagaaagtgatacaagagcttattttacaggagttacaatc
ttaatatccttaccaactggtacaaaaatctttaattggctttttacatatctctcaaat
ccaccattattacaccttagaattacttctgtcttcctctcacatctctttttattaatg
tttacgataggtgggtcaacaggaataattcttggaaatggtgcagtggatctaggatta
catgatacatattatgttgtagcacattttcattttgttctttctttaggagctataatt
gctatcttctctggaataatcttgaatggagaaaagattgttgctactaagaatttatta
ctttcatcctcatgtacactctctctttatcatttacatttaatatttattggtattctt
cttaccttttccccaatgcatttcttaggatttaatgttatgccaagaagaatcccatcc
tttccagattcttttcattcctggaattccctgtcatctattggatcaggaataactttc
ctatctttttctatg
>Ik8_cox1
ataaatctcttaaccattaatttcctatttcattcactagttaaaaattgtaatcataaa
ggcttaggaatctattatttattatctggattcatctttggaatctccggtacattaata
tcagtccttataagaatagaattatattcttcaggaaataggattatatctccagaaaac
cagaacttctataatataagcattacattgcatggctttcttatgattttctttttagta
atgcctggcttgtttggaggatttggaaattattttgtacctatctttcaagggtctcca
gaagtggtatatcctagagtcaataatttttctatcttaattcttttgctttcatatctt
ttcctaatcctttctttaatctcagaatttggaggtggtacagggtggacgctctaccca

ccattatccacttcttttatgactttatcaccttcaagtacaggaaatcttatatttgga
ttaataatctctggtatatcttcatgtcttacatctcttaacttttggacaacaattcat
tttctgagatcttattatctgatattatcttctatcccattatttccttgggctttcttg
attacagctttcatgcttttattaacattaccaatcttatctggtacacttcttttaata
ttgggtgatcttcattctaatacacttttctttgatccaatatttggaggagatcctata
ttctatcaacatttattttggtttttggacatccagaagtttacatattaataattcct
gcatttgggatcatttccataataatttctggtattttacagttaataatctttgctaac
caatcaatgatctttgccatgtcatctatttctcttcttggaggtcttgtttggggacat
catatgtatactgtaggtttagaaagtgatacaagagcttattttacaggagttacaatc
ttaatatccttaccaactggtacaaaaatctttaattggctttttacatatctctcaaat
ccaccattattacaccttagaattacttctgtcttcctctcacatctcttttattaatg
tttacgataggtgggtcaacaggaataattcttggaaatggtgcagtggatctaggatta
catgatacatattatgttgtagcacattttcattttgttctttctttaggagctataatt
gctatcttctctggaataatcttgaatggagaaaagattgttgctactaagaatttatta
ctttcatcctcatgtacactctctctttatcatttacatttaatatttattggtattctt
cttaccttttccccaatgcatttcttaggatttaatgttatgccaagaagaatcccatcc
tttccagattcttttcattcctggaattccctgtcatctattggatcaggaataactttc
ctatctttttctatg
>Ik9_cox1
ataaatctcttaaccattaatttcctatttcattcactagttaaaaattgtaatcataaa
ggcttaggaatctattatttattatctggattcatctttggaatctccggtacattaata
tcagtccttataagaatagaattatattcttcaggaaataggattatatctccagaaaac
cagaacttctataatataagcattacattgcatggctttcttatgattttctttttagta
atgcctggcttgtttggaggatttggaaattattttgtacctatctttcaagggtctcca
gaagtggtatatcctagagtcaataatttttctatcttaattcttttgctttcatatctt
ttcctaatcctttctttaatctcagaatttggaggtggtacagggtggacgctctaccca
ccattatccacttcttttatgactttatcaccttcaagtacaggaaatcttatatttgga
ttaataatctctggtatatcttcatgtcttacatctcttaacttttggacaacaattcat
tttctgagatcttattatctgatattatcttctatcccattatttccttgggctttcttg
attacagctttcatgcttttattaacattaccaatcttatctggtacacttcttttaata
ttgggtgatcttcattctaatacacttttctttgatccaatatttggaggagatcctata
ttctatcaacatttattttggtttttggacatccagaagtttacatattaataattcct
gcatttgggatcatttccataataatttctggtattttacagttaataatctttgctaac
caatcaatgatctttgccatgtcatctatttctcttcttggaggtcttgtttggggacat
catatgtatactgtaggtttagaaagtgatacaagagcttattttacaggagttacaatc
ttaatatccttaccaactggtacaaaaatctttaattggctttttacatatctctcaaat
ccaccattattacaccttagaattacttctgtcttcctctcacatctcttttattaatg
tttacgataggtgggtcaacaggaataattcttggaaatggtgcagtggatctaggatta
catgatacatattatgttgtagcacattttcattttgttctttctttaggagctataatt
gctatcttctctggaataatcttgaatggagaaaagattgttgctactaagaatttatta
ctttcatcctcatgtacactctctctttatcatttacatttaatatttattggtattctt
cttaccttttccccaatgcatttcttaggatttaatgttatgccaagaagaatcccatcc
tttccagattcttttcattcctggaattccctgtcatctattggatcaggaataactttc
ctatctttttctatg
>Irm10_cox1
ataaatctcttaaccattaatttcctatttcattcactagttaaaaattgtaatcataaa
ggcttaggaatctattatttattatctggattcatctttggaatctccggtacattaata
tcagtccttataagaatagaattatattcttcaggaaataggattatatctccagaaaac
cagaacttctataatataagcattacattgcatggctttcttatgattttctttttagta
atgcctggcttgtttggaggatttggaaattattttgtacctatctttcaagggtctcca
gaagtggtatatcctagagtcaataatttttctatcttaattcttttgctttcatatctt
ttcctaatcctttctttaatctcagaatttggaggtggtacagggtggacgctctaccca
ccattatccacttcttttatgactttatcaccttcaagtacaggaaatcttatatttgga
ttaataatctctggtatatcttcatgtcttacatctcttaacttttggacaacaattcat
tttctgagatcttattatctgatattatcttctatcccattatttccttgggctttcttg
attacagctttcatgcttttattaacattaccaatcttatctggtacacttcttttaata
ttgggtgatcttcattctaatacacttttctttgatccaatatttggaggagatcctata

ttctatcaacatttattttggtttttggacatccagaagtttacatattaataattcct
gcatttgggatcatttccataataatttctggtattttacagttaataatctttgctaac
caatcaatgatctttgccatgtcatctatttctcttcttggaggtcttgtttggggacat
catatgtatactgtaggtttagaaagtgatacaagagcttattttacaggagttacaatc
ttaatatccttaccaactggtacaaaaatctttaattggctttttacatatctctcaaat
ccaccattattacaccttagaattacttctgtcttcctctcacatctctttttattaatg
tttacgataggtgggtcaacaggaataattcttggaaatggtgcagtggatctaggatta
catgatacatattatgttgtagcacattttcattttgttctttctttaggagctataatt
gctatcttctctggaataatcttgaatggagaaaagattgttgctactaagaatttatta
ctttcatcctcatgtacactctctctttatcatttacatttaatatttattggtattctt
cttaccttttccccaatgcatttcttaggatttaatgttatgccaagaagaatcccatcc
tttccagattcttttcattcctggaattccctgtcatctattggatcaggaataactttc
ctatctttttctatg
>Irm17_cox1
ttaaatctcttaaccattaatttcctatttcattcactagttaaaaattgtaatcataaa
ggcttaggaatctattatttattatctggattcatctttggaatctccggtacattaata
tcagtccttataagaatagaattatattcttcaggaaataggattatatctccagaaaac
cagaacttctataatataagcattacattgcatggctttcttatgattttctttttagta
atgcctggcttgtttggaggatttggaaattattttgtacctatctttcaagggtctcca
gaagtggtatatcctagagtcaataatttttctatcttaattcttttgctttcatatctt
ttcctaatcctttctttaatctcagaatttggaggtggtacagggtggacgctctaccca
ccattatccacttcttttatgactttatcaccttcaagtacaggaaatcttatatttgga
ttaataatctctggtatatcttcatgtcttacatctcttaacttttggacaacaattcat
tttctgagatcttattatctgatattatcttctatcccattatttccttgggctttcttg
attacagctttcatgcttttattaacattaccaatcttatctggtacacttcttttaata
ttgggtgatcttcattctaatacacttttctttgatccaatatttggaggagatcctata
ttctatcaacatttattttggtttttggacatccagaagtttacatattaataattcct
gcatttgggatcatttccataataatttctggtattttacagttaataatctttgctaac
caatcaatgatctttgccatgtcatctatttctcttcttggaggtcttgtttggggacat
catatgtatactgtaggtttagaaagtgatacaagagcttattttacaggagttacaatc
ttaatatccttaccaactggtacaaaaatctttaattggctttttacatatctctcaaat
ccaccattattacaccttagaattacttctgtcttcctctcacatctctttttattaatg
tttacgataggtgggtcaacaggaataattcttggaaatggtgcagtggatctaggatta
catgatacatattatgttgtagcacattttcattttgttctttctttaggagctataatt
gctatcttctctggaataatcttgaatggagaaaagattgttgctactaagaatttatta
ctttcatcctcatgtacactctctctttatcatttacatttaatatttattggtattctt
cttaccttttccccaatgcatttcttaggatttaatgttatgccaagaagaatcccatcc
tttccagattcttttcattcctggaattccctgtcatctattggatcaggaataactttc
ctatctttttctatg
>Irm2_cox1
ataaatctcttaaccattaatttcctatttcattcactagttaaaaattgtaatcataaa
ggcttaggaatctattatttattatctggattcatctttggaatctccggtacattaata
tcagtccttataagaatagaattatattcttcaggaaataggattatatctccagaaaac
cagaacttctataatataagcattacattgcatggctttcttatgattttctttttagta
atgcctggcttgtttggaggatttggaaattattttgtacctatctttcaagggtctcca
gaagtggtatatcctagagtcaataatttttctatcttaattcttttgctttcatatctt
ttcctaatcctttctttaatctcagaatttggaggtggtacagggtggacgctctaccca
ccattatccacttcttttatgactttatcaccttcaagtacaggaaatcttatatttgga
ttaataatctctggtatatcttcatgtcttacatctcttaacttttggacaacaattcat
tttctgagatcttattatctgatattatcttctatcccattatttccttgggctttcttg
attacagctttcatgcttttattaacattaccaatcttatctggtacacttcttttaata
ttgggtgatcttcattctaatacacttttctttgatccaatatttggaggagatcctata
ttctatcaacatttattttggtttttggacatccagaagtttacatattaataattcct
gcatttgggatcatttccataataatttctggtattttacagttaataatctttgctaac
caatcaatgatctttgccatgtcatctatttctcttcttggaggtcttgtttggggacat
catatgtatactgtaggtttagaaagtgatacaagagcttattttacaggagttacaatc
ttaatatccttaccaactggtacaaaaatctttaattggctttttacatatctctcaaat

ccaccattattacaccttagaattacttctgtcttcctctcacatctctttttattaatg
tttacgataggtgggtcaacaggaataattcttggaaatggtgcagtggatctaggatta
catgatacatattatgttgtagcacattttcattttgttctttctttaggagctataatt
gctatcttctctggaataatcttgaatggagaaaagattgttgctactaagaatttatta
ctttcatcctcatgtacactctctctttatcatttacatttaatatttattggtattctt
cttacctttttccccaatgcatttcttaggatttaatgttatgccaagaagaatcccatcc
tttccagattcttttcattcctggaattccctgtcatctattggatcaggaataactttc
ctatctttttctatg
>Irm21_cox1
ataaatctcttaaccattaatttcctatttcattcactagttaaaaattgtaatcataaa
ggcttaggaatctattatttattatctggattcatctttggaatctccggtacattaata
tcagtccttataagaatagaattatattcttcaggaaataggattatatctccagaaaac
cagaacttctataatataagcattacattgcatggctttcttatgattttctttttagta
atgcctggcttgtttggaggatttggaaattattttgtacctatctttcaagggtctcca
gaagtggtatatcctagagtcaataatttttctatcttaattcttttgctttcatatctt
ttcctaatcctttctttaatctcagaatttggaggtggtacagggtggacgctctaccca
ccattatccacttcttttatgactttatcaccttcaagtacaggaaatcttatatttgga
ttaataatctctggtatatcttcatgtcttacatctcttaacttttggacaacaattcat
tttctgagatcttattatctgatattatcttctatcccattatttccttgggctttcttg
attacagctttcatgcttttattaacattaccaatcttatctggtacacttcttttaata
ttgggtgatcttcattctaatacacttttctttgatccaatatttggaggagatcctata
ttctatcaacatttattttggtttttggacatccagaagtttacatattaataattcct
gcatttgggatcatttccataataatttctggtattttacagttaataatctttgctaac
caatcaatgatctttgccatgtcatctatttctcttcttggaggtcttgtttggggacat
catatgtatactgtaggtttagaaagtgatacaagagcttattttacaggagttacaatt
ttaatatccttaccaactggtacaaaaatctttaattggctttttacatatctctcaaat
ccaccattattacaccttagaattacttctgtcttcctctcacatctctttttattaatg
tttacgataggtgggtcaacaggaataattcttggaaatggtgcagtggatctaggatta
catgatacatattatgttgtagcacattttcattttgttctttctttaggagctataatt
gctatcttctctggaataatcttgaatggagaaaagattgttgctactaagaatttatta
ctttcatcctcatgtacactctctctttatcatttacatttaatatttattggtattctt
cttacctttttccccaatgcatttcttaggatttaatgttatgccaagaagaatcccatcc
tttccagattcttttcattcctggaattccctgtcatctattggatcaggaataactttc
ctatctttttctatg
>Irm22_cox1
ataaatctcttaaccattaatttcctatttcattcactagttaaaaattgtaatcataaa
ggcttaggaatctattatttattatctggattcatctttggaatctccggtacattaata
tcagtccttataagaatagaattatattcttcaggaaataggattatatctccagaaaac
cagaacttctataatgtaagcattacattgcatggccttcttatgattttctttttagta
atgcctggcttgtttggaggatttggaaattattttgtacctatctttcaagggtctcca
gaagtggtatatcctagagtcaataatttttctatcttaattcttttgctttcatatctt
ttcctaatcctttctttaatctcagaatttggaggtggtacagggtggacgctctaccca
ccattatccacttcttttatgactttatcaccttcaagtacaggaaatcttatatttgga
ttaataatctctggtatatcttcatgtcttacatctcttaacttttggacaacaattcat
tttctgagatcttattatctgatattatcttctgtcccattatttccttgggctttcttg
attacagctttcatgcttttattaacattaccaatcttatctggtacacttcttttaata
ttgggtgatcttcattctaatacacttttctttgatccaatatttggaggagatcctata
ttctatcaacatttattttggtttttggacatccagaagtttacatattaataattcct
gcatttggggtcatttccatagtaatttctggggtttcacagttaataatctttgctaac
caatcaatgatctttgccatgtcatctatttctcttcttggaggtcttgtttggggacat
catatgtatactgtaggtttagaaagtgatacaagagcttattttacaggagttacaatc
ttaatatccttaccaactggtacaaaaatctttaattggctttttacatatctctcaaat
ccaccattattacaccttagaattacttctgtcttcctctcacatctctttttattaatg
tttacgataggtgggtcaacaggaataattcttggaaatggtgcagtggatctaggatta
catgatacatattatgttgtagcacattttcattttgttctttctttaggagctataatt
gctatcttctctggaataatcttgaatggagaaaagattgttgctactaagaatttatta
ctttcatcctcatgtacactctctctttatcatttacatttaatatttattggtattctt

cttaccttttccccaatgcatttcttaggatttaatgttatgccaagaagaatcccatcc
tttccagattcttttcattcctggaattccctgtcatctattggatcaggaataactctc
ctatctttttctatg
>Irm23_cox1
ataaatctcttaaccattaatttcctatttcattcactagttaaaaattgtaatcataaa
ggcttaggaatctattatttattatctggattcatctttggaatctccggtacattaata
tcagtccttataagaatagaattatattcttcaggaaataggattatatctccagaaaac
cagaacttctataatataagcattacattgcatggctttcttatgattttctttttagta
atgcctggcttgtttggaggatttggaaattattttgtacctatctttcaagggtctcca
gaagtggtatatcctagagtcaataatttttctatcttaattcttttgctttcatatctt
ttcctaatcctttctttaatctcagaatttggaggtggtacagggtggacgctctaccca
ccattatccacttcttttatgactttatcaccttcaagtacaggaaatcttatatttgga
ttaataatctctggtatatcttcatgtcttacatctcttaacttttggacaacaattcat
tttctgagatcttattatctgatattatcttctatcccattatttccttgggctttcttg
attacagctttcatgcttttattaacattaccaatcttatctggtacacttcttttaata
ttgggtgatcttcattctaatacacttttctttgatccaatatttggaggagatcctata
ttctatcaacatttattttggtttttggacatccagaagtttacatattaataattcct
gcatttgggatcatttccataataatttctggtattttacagttaataatctttgctaac
caatcaatgatctttgccatgtcatctatttctcttcttggaggtcttgtttggggacat
catatgtatactgtaggtttagaaagtgatacaagagcttattttacaggagttacaatc
ttaatatccttaccaactggtacaaaaatctttaattggctttttacatatctctcaaat
ccaccattattacaccttagaattacttctgtcttcctctcacatctctttttattaatg
tttacgataggtgggtcaacaggaataattcttggaaatggtgcagtggatctaggatta
catgatacatattatgttgtagcacattttcattttgttctttctttaggagctataatt
gctatcttctctggaataatcttgaatggagaaaagattgttgctactaagaatttatta
ctttcatcctcatgtacactctctctttatcatttacatttaatatttattggtattctt
cttaccttttccccaatgcatttcttaggatttaatgttatgccaagaagaatcccatcc
tttccagattcttttcattcctggaattccctgtcatctattggatcaggaataactttc
ctatctttttctatg
>Irm24_cox1
ataaatctcttaaccattaatttcctatttcattcactagttaaaaattgtaatcataaa
ggcttaggaatctattatttattatctggattcatctttggaatctccggtacattaata
tcagtccttatgagaatagaattatattcttcaggaaataggattatatctccagaaaac
cagaacttctataatataagcattacattgcatggctttcttatgattttctttttagta
atgcctggcttgtttggaggatttggaaattattttgtacctatctttcaagggtctcca
gaagtggtatatcctagagtcaataatttttctatcttaattcttttgctttcatatctt
ttcctaatcctttctttaatctcagaatttggaggtggtacagggtggacgctctaccca
ccattatccacttcttttatgactttatcaccttcaagtgtaggaaatcttatatttgga
ttaataatctctggtatatcttcatctcttacatctcttaacttttgggtaacaattcat
tttctgagatcttattatctgatattatcttctgtcccattatttccttgggctttcttg
attacagctttcatgcttttattaacattaccaatcttatctggtacacttcttttaata
ttgggtgatcttcattctaatacacttttctttgatccaatatttggaggagatcctata
ttctatcaacatttattttggtttttggacatccagaagtttacatattaataattcct
gcatttgggatcatttccataataatttctggggtttcacagttaataatctttgctaac
caatcaatgatctttgccatgtcatctatttctcttcttggaggtcttgtttggggacat
catatgtatactgtaggtttagaaagtgatacaagagcttattttacaggagttacaatc
ttaatatccttaccaactggtacaaaaatctttaattggctttttacatatctctcaaat
ccaccattattacaccttagaattacttctgtcttcctctcacatctctttttattaatg
tttacgataggtgggtcaacaggaataattcttggaaatggtgcagtggatctaggatta
catgatacatattatgttgtagcacattttcattttgttctttctttaggagctataatt
gctatcttctctggaataatcttgaatggagaaaagattgttgctactaagaatttatta
ctttcatcctcatgtacactctctctttatcatttacatttaatatttattggtattctt
cttaccttttccccaatgcatttcttaggatttaatgttatgccaagaagaatcccatcc
tttccagattcttttcattcctggaattccctgtcatctattggatcaggaataactttc
ctatctttttctatg
>Irm25_cox1
ataaatctcttaaccattaatttcctatttcattcactagttaaaaattgtaatcataaa

ggcttaggaatctattatttattatctggattcatctttggaatctccggtacattaata
tcagtccttataagaatagaattatattcttcaggaaataggattatatctccagaaaac
cagaacttctataatataagcattacattgcatggctttcttatgattttctttttagta
atgcctggcttgtttggaggatttggaaattattttgtacctatctttcaagggtctcca
gaagtggtatatcctagagtcaataatttttctatcttaattcttttgctttcatatctt
ttcctaatcctttctttaatctcagaatttggaggtggtacagggtggacgctctaccca
ccattatccacttcttttatgactttatcaccttcaagtacaggaaatcttatatttgga
ttaataatctctggtatatcttcatgtcttacatctcttaacttttggacaacaattcat
tttctgagatcttattatctgatattatcttctatcccattatttccttgggctttcttg
attacagctttcatgcttttattaacattaccaatcttatctggtacacttcttttaata
ttgggtgatcttcattctaatacacttttctttgatccaatatttggaggagatcctata
ttctatcaacatttattttggtttttggacatccagaagtttacatattaataattcct
gcatttgggatcatttccataataatttctggtattttacagttaataatctttgctaac
caatcaatgatctttgccatgtcatctatttctcttcttggaggtcttgtttggggacat
catatgtatactgtaggtttagaaagtgatacaagagcttattttacaggagttacaatc
ttaatatccttaccaactggtacaaaaatctttaattggctttttacatatctctcaaat
ccaccattattacaccttagaattacttctgtcttcctctcacatctctttttattaatg
tttacgataggtgggtcaacaggaataattcttggaaatggtgcagtggatctaggatta
catgatacatattatgttgtagcacattttcattttgttctttctttaggagctataatt
gctatcttctctggaataatcttgaatggagaaaagattgttgctactaagaatttatta
ctttcatcctcatgtacactctctctttatcatttacatttaatatttattggtattctt
cttaccttttccccaatgcatttcttaggatttaatgttatgccaagaagaatcccatcc
tttccagattcttttcattcctggaattccctgtcatctattggatcaggaataactttc
ctatctttttctatg
>Irm26_cox1
ataaatctcttaaccattaatttcctatttcattcactagttaaaaattgtaatcataaa
ggcttaggaatctattatttattatctggattcatctttggaatctccggtacattaata
tcagtccttataagaatagaattatattcttcaggaaataggattatatctccagaaaac
cagaacttctataatataagcattacattgcatggctttcttatgattttctttttagta
atgcctggcttgtttggaggatttggaaattattttgtacctatctttcaagggtctcca
gaagtggtatatcctagagtcaataatttttctatcttaattcttttgctttcatatctt
ttcctaatcctttctttaatctcagaatttggaggtggtacagggtggacgctctaccca
ccattatccacttcttttatgactttatcaccttcaagtacaggaaatcttatatttgga
ttaataatctctggtatatcttcatgtcttacatctcttaacttttggacaacaattcat
tttctgagatcttattatctgatattatcttctatcccattatttccttgggctttcttg
attacagctttcatgcttttattaacattaccaatcttatctggtacacttcttttaata
ttgggtgatcttcattctaatacacttttctttgatccaatatttggaggagatcctata
ttctatcaacatttattttggtttttggacatccagaagtttacatattaataattcct
gcatttgggatcatttccataataatttctggtattttacagttaataatctttgctaac
caatcaatgatctttgccatgtcatctatttctcttcttggaggtcttgtttggggacat
catatgtatactgtaggtttagaaagtgatacaagagcttattttacaggagttacaatc
ttaatatccttaccaactggtacaaaaatctttaattggctttttacatatctctcaaat
ccaccattattacaccttagaattacttctgtcttcctctcacatctctttttattaatg
tttacgataggtgggtcaacaggaataattcttggaaatggtgcagtggatctaggatta
catgatacatattatgttgtagcacattttcattttgttctttctttaggagctataatt
gctatcttctctggaataatcttgaatggagaaaagattgttgctactaagaatttatta
ctttcatcctcatgtacactctctctttatcatttacatttaatatttattggtattctt
cttaccttttccccaatgcatttcttaggatttaatgttatgccaagaagaatcccatcc
tttccagattcttttcattcctggaattccctgtcatctattggatcaggaataactttc
ctatctttttctatg
>Irm27_cox1
ataaatctcttaaccattaatttcctatttcattcattagttaaaaattgtaatcataaa
ggcttaggaatctattatttattatctggattcatctttggaatctccggtacattaata
tcagtccttataagaatagaattatattcttcaggaaataggattatatctccagaaaac
cagaacttctataatgtaagcattacattgcatggccttcttatgattttctttttagta
atgcctggcttgtttggaggatttggaaattattttgtacctatctttcaagggtctcca
gaagtggtatatcctagagtcaataatttttctatcttaattcttttcctttcatatctt

ttcctaatcctttctttaatctcagaatttggaggtggtacagggtggacgctctaccca
ccattatccacttcttttatgactttatcaccttcaagtgtaggaaatcttatatttgga
ttaataatctctggtatatcttcatgtcttacatctcttaacttttggacaactattcat
ttattgagatcttattatctgatattatcttctattccattatttccttggtctttcttg
attacagctttcatgcttttattaacattaccaatcttatctggaacacttcttttaata
ttgggtgatcttcattcaaatacacttttctttgatccaatatttggaggagatcctgta
ttctatcaacatttattttggtttttggacatccagaagtttacatattaataattcct
gcatttgggatcatttccataataatttctggtattttacaattaattatttttgctaac
caatcaatgatctttgccatgtcatctatttctcttcttggaggtcttgtttggggacat
catatgtatactgtaggtttagaaagtgatacaagagcttattttacaggagttacaatc
ttaatatccttaccaactggtacaaaaatatttaattggctttttacatatctctccaat
ccaccattattacaccttaggattacttctgtcttcctctcacatctatttttattaatg
tttacggtaggtgggtcaacaggagtaattcttggaaatggtgcagtggatctagcatta
catgatacatattatgttgtagcacattttcattttgttctttctttaggagctgtaatt
gctatcttctctggaatagtcttgaatggagaaaagattgttgctactaagagtttatta
ttttcatcctcatgtacactctctctttatcatttacatttaatatttattggtattctt
cttaccttttcaccaatgcatttcttaggatttaatgttatggcaagaagaatcccatcc
tttccagattcttttcattcctggaattccctgtcatctattggatcaggaataactttc
ctatctttttctatg
>Irm3_cox1
ataaatctcttaaccattaatttcctatttcattcattagttaaaaattgtaatcataaa
ggcttaggaatctattatttattatctggattcatctttggaatctccggtacattaata
tcagtccttataagaatagaattatattcttcaggaaataggattatatctccagaaaac
cagaacttctataatataagcattacattgcatggctttcttatgattttctttttagta
atgcctggcttgtttggaggatttggaaattattttgtacctatctttcaagggtctcca
gaagtggtatatcctagagtcaataatttttctatcttaattcttttgctttcatatctt
ttcctaatcctttctttaatctcagaatttggaggtggtacaggatggacgctctaccca
ccattatccacttcttttatgactttatcaccttcaagtacaggaaatcttatatttgga
ttaataatctctggtatatcttcatgtcttacatctcttaacttttggacaactattcat
ttattgagatcttattatctgatattatcttctattccattatttccttggtctttcttg
attacagctttcatgcttttattaacattaccaatcttatctggaacacttcttttaata
ttgggtgatcttcattctaatacaattttctttgatccaatatttggaggagatcctata
ttctatcaacatttattttggtttttggacatccagaagtttacatattaataattcct
gcatttgggatcatttccataataatttctggtattttacaattaattatttttgctaac
caatcaatgatctttgccatgtcatctatttctcttcttggaggtcttgtttggggacat
catatgtatactgtaggtttagaaagtgatacaagagcttattttacaggagttacaatc
ttaatatccttaccaactggtacaaaaatatttaattggctttttacatatctctccaat
ccaccattattacaccttaggattacttctgtcttcctctcacatctatttttattaatg
tttacgataggtgggtcaacaggaataattcttggaaatggtgcagtggatctaggatta
catgatacatattatgttgtagcacattttcattttgttctttctttaggagctataatt
gctatattctctggaataatcttgaatggagaaaagattgttgctaataagaatttatta
ttttcatcctcatgtacactctctctttatcatttacatttaatatttattggtattctt
cttaccttttcaccaatgcatttcttaggatttaatgtaatggcaagaagaatcccatcc
tttccagattcttttcattcctggaattccctgtcatctattggatcaggaataactttc
ctatctttttctatg
>Irm4_cox1
ataaatctcttaaccattaatttcctatttcattcattagttaaaaattgtaatcataaa
ggcttaggaatctattatttattatctggattcatctttggaatctccggtacattaata
tcagtccttataagaatagaattatattcttcaggaaataggattatatctccagaaaac
cagaacttctataatataagcattacattgcatggctttcttatgattttctttttagta
atgcctggcttgtttggaggatttggaaattattttgtacctatctttcaagggtctcca
gaagtggtatatcctagagtcaataatttttctatcttaattcttttgctttcatatctt
ttcctaatcctttctttaatctcagaatttggaggtggtacaggatggacgctctaccca
ccattatccacttcttttatgactttatcaccttcaagtacaggaaatcttatatttgga
ttaataatctctggtatatcttcatgtcttacatctcttaacttttggacaactattcat
ttattgagatcttattatctgatattatcttctattccattatttccttggtctttcttg
attacagctttcatgcttttattaacattaccaatcttatctggaacacttcttttaata

ttgggtgatcttcattctaatacaattttctttgatccaatatttggaggagatcctata
ttctatcaacatttattttggttttttggacatccagaagtttacatattaataattcct
gcatttgggatcatttccataataatttctggtattttacaattaattattttttgctaac
caatcaatgatctttgccatgtcatctatttctcttcttggaggtcttgtttggggacat
catatgtatactgtaggtttagaaagtgatacaagagcttattttacaggagttacaatc
ttaatatccttaccaactggtacaaaaatatttaattggctttttacatatctctcaaat
ccaccattattacaccttaggattacttctgtcttcctctcacatctatttttattaatg
tttacgataggtgggtcaacaggaataattcttggaaatggtgcagtggatctaggatta
catgatacatattatgttgtagcacattttcattttgttctttctttaggagctataatt
gctatattctctggaataatcttgaatggagaaaagattgttgctaataagaatttatta
ttttcatcctcatgtacactctctctttatcatttacatttaatatttattggtattctt
cttaccttttcaccaatgcatttcttaggatttaatgtaatggcaagaagaatcccatcc
tttccagattcttttcattcctggaattccctgtcatctattggatcaggaataactttc
ctatctttttctatg
>Irm5_cox1
ataaatctcttaaccattaatttcctatttcattcactagttaaaaattgtaatcataaa
ggcttaggaatctattatttattatctggattcatctttggaatctccggtacattaata
tcagtccttataagaatagaattatattcttcaggaaataggattatatctccagaaaac
cagaacttctataatataagcattacattgcatggctttcttatgattttctttttagta
atgcctggcttgtttggaggatttggaaattattttgtacctatctttcaagggtctcca
gaagtggtatatcctagagtcaataatttttctatcttaattcttttgctttcatatctt
ttcctaatcctttctttaatctcagaatttggaggtggtacagggtggacgctctaccca
ccattatccacttcttttatgactttatcaccttcaagtacaggaaatcttatatttgga
ttaataatctctggtatatcttcatgtcttacatctcttaacttttggacaacaattcat
tttctgagatcttattatctgatattatcttctatcccattatttccttgggctttcttg
attacagctttcatgcttttattaacattaccaatcttatctggtacacttcttttaata
ttgggtgatcttcattctaatacacttttctttgatccaatatttggaggagatcctata
ttctatcaacatttattttggttttttggacatccagaagtttacatattaataattcct
gcatttgggatcatttccataataatttctggtattttacagttaataatctttgctaac
caatcaatgatctttgccatgtcatctatttctcttcttggaggtcttgtttggggacat
catatgtatactgtaggtttagaaagtgatacaagagcttattttacaggagttacaatc
ttaatatccttaccaactggtacaaaaatctttaattggctttttacatatctctcaaat
ccaccattattacaccttagaattacttctgtcttcctctcacatctctttttattaatg
tttacgataggtgggtcaacaggaataattcttggaaatggtgcagtggatctaggatta
catgatacatattatgttgtagcacattttcattttgttctttctttaggagctataatt
gctatcttctctggaataatcttgaatggagaaaagattgttgctactaagaatttatta
ctttcatcctcatgtacactctctctttatcatttacatttaatatttattggtattctt
cttaccttttccccaatgcatttcttaggatttaatgttatgccaagaagaatcccatcc
tttccagattcttttcattcctggaattccctgtcatctattggatcaggaataactttc
ctatctttttctatg
>Irm7_cox1
ataaatctcttaaccattaatttcctatttcattcactagttaaaaattgtaatcataaa
ggcttaggaatctattatttattatctggattcatctttggaatctccggtacattaata
tcagtccttataagaatagaattatattcttcaggaaataggattatatctccagaaaac
cagaacttctataatataagcattacattgcatggctttcttatgattttctttttagta
atgcctggcttgtttggaggatttggaaattattttgtacctatctttcaagggtctcca
gaagtggtatatcctagagtcaataatttttctatcttaattcttttgctttcatatctt
ttcctaatcctttctttaatctcagaatttggaggtggtacagggtggacgctctaccca
ccattatccacttcttttatgactttatcaccttcaagtacaggaaatcttatatttgga
ttaataatctctggtatatcttcatgtcttacatctcttaacttttggacaacaattcat
tttctgagatcttattatctgatattatcttctatcccattatttccttgggctttcttg
attacagctttcatgcttttattaacattaccaatcttatctggtacacttcttttaata
ttgggtgatcttcattctaatacacttttctttgatccaatatttggaggagatcctata
ttctatcaacatttattttggttttttggacatccagaagtttacatattaataattcct
gcatttgggatcatttccataataatttctggtattttacagttaataatctttgctaac
caatcaatgatctttgccatgtcatctatttctcttcttggaggtcttgtttggggacat
catatgtatactgtaggtttagaaagtgatacaagagcttattttacaggagttacaatc

ttaatatccttaccaactggtacaaaaatctttaattggctttttacatatctctcaaat
ccaccattattacaccttagaattacttctgtcttcctctcacatctctttttattaatg
tttacgataggtgggtcaacaggaataattcttggaaatggtgcagtggatctaggatta
catgatacatattatgttgtagcacattttcattttgttctttctttaggagctataatt
gctatcttctctggaataatcttgaatggagaaaagattgttgctactaagaatttatta
ctttcatcctcatgtacactctctctttatcatttacatttaatatttattggtattctt
cttaccttttccccaatgcatttcttaggatttaatgttatgccaagaagaatcccatcc
tttccagattcttttcattcctggaattccctgtcatctattggatcaggaataactttc
ctatctttttctatg
>Irm9_cox1
ataaatctcttaaccattaatttcctatttcattcattagttaaaaattgtaatcataaa
ggcttaggaatctattatttattatctggattcatctttggaatctccggtacattaata
tcagtccttataagaatagaattatattcttcaggaaataggattatatctccagaaaac
cagaacttctataatataagcattacattgcatggctttcttatgattttctttttagta
atgcctggcttgtttggaggatttggaaattattttgtacctatctttcaagggtctcca
gaagtggtatatcctagagtcaataatttttctatcttaattattttgctttcatatctt
ttcctaatcctttctttaatctcagaatttggaggtggtacaggatggacgctctaccca
ccattatccacttcttttatgactttatcaccttcaagtacaggaaatcttatatttgga
ttaataatctctggtatatcttcatctcttacatctcttaacttttgggcaactattcat
ttattgagatcttattatctgatattatcttctattccattatttccttggtctttcttg
attacagctttcatgcttttattaacattaccaatcttatctggaacacttcttttaata
ttgggtgatcttcattctaatacaattttctttgatccaatatttggaggagatcctata
ttctatcaacatttattttggtttttggacatccagaagtttacatattaataattcct
gcatttgggatcatttccataataatttctggtattttacaattaattatttttgctaac
caatcaatgatctttgccatgtcatctatttctcttcttggaggtcttgtttggggacat
catatgtatactgtaggtttagaaagtgatacaagagcttattttacaggagttacaatc
ttaatatccttaccaactggtacaaaaatatttaattggctttttacatatctctccaat
ccaccattattacaccttaggattacttctgtcttcctctcacatctatttttattaatg
tttacgataggtgggtcaacaggaataattcttggaaatggtgcagtggatctaggatta
catgatacatattatgttgtagcacattttcattttgttctttctttaggagctataatt
gctatattctctggaataatcttgaatggagaaaagattgttgctaataagaatttatta
ttttcatcctcatgtacactctctctttatcatttacatttaatatttattggtattctt
cttaccttttcaccaatgcatttcttaggatttaatgtaatggcaagaagaatcccatcc
tttccagattcttttcattcctggaattccctgtcatctattggatcaggaataactctc
ctatctttttctatg
>Isy12_cox1
ataaatctcttaaccattaatttcctatttcattcactagttaaaaattgtaatcataaa
ggcttaggaatctattatttattatctggattcatctttggaatctccggtacattaata
tcagtccttataagaatagaattatattcttcaggaaataggattatatctccagaaaac
cagaacttctataatataagcattacattgcatggctttcttatgattttctttttagta
atgcctggcttgtttggaggatttggaaattattttgtacctatctttcaagggtctcca
gaagtggtatatcctagagtcaataatttttctatcttaattcttttgctttcatatctt
ttcctaatcctttctttaatctcagaatttggaggtggtacagggtggacgctctaccca
ccattatccacttcttttatgactttatcaccttcaagtacaggaaatcttatatttgga
ttaataatctctggtatatcttcatgtcttacatctcttaacttttggacaacaattcat
tttctgagatcttattatctgatattatcttctatcccattatttccttgggctttcttg
attacagctttcatgcttttattaacattaccaatcttatctggtacacttcttttaata
ttgggtgatcttcattctaatacacttttctttgatccaatatttggaggagatcctata
ttctatcaacatttattttggtttttggacatccagaagtttacatattaataattcct
gcatttgggatcatttccataataatttctggtattttacagttaataatctttgctaac
caatcaatgatctttgccatgtcatctatttctcttcttggaggtcttgtttggggacat
catatgtatactgtaggtttagaaagtgatacaagagcttattttacaggagttacaatt
ttaatatccttaccaactggtacaaaaatctttaattggctttttacatatctctcaaat
ccaccattattacaccttagaattacttctgtcttcctctcacatctctttttattaatg
tttacgataggtgggtcaacaggaataattcttggaaatggtgcagtggatctaggatta
catgatacatattatgttgtagcacattttcattttgttctttctttaggagctataatt
gctatcttctctggaataatcttgaatggagaaaagattgttgctactaagaatttatta

ctttcatcctcatgtacactctctctttatcatttacatttaatatttattggtattctt
cttaccttttccccaatgcatttcttaggatttaatgttatgccaagaagaatcccatcc
tttccagattcttttcattcctggaattccctgtcatctattggatcaggaataactttc
ctatctttttctatg
>Isy15_cox1
ataaatctcttaaccattaatttcctatttcattcattagttaaaaattgtaatcataaa
ggcttaggaatctattatttattatctggattcatctttggaatctccggtacattaata
tcagtccttataagaatagaattatattcttcaggaaataggattatatctccagaaaac
cagaacttctataatataagcattacattgcatggctttcttatgattttctttttagta
atgcctggcttgtttggaggatttggaaattattttgtacctatctttcaagggtctcca
gaagtggtatatcctagagtcaataatttttctatcttaattattttgctttcatatctt
ttcctaatcctttctttaatctcagaatttggaggtggtacaggatggacgctctaccca
ccattatccacttcttttatgactttatcaccttcaagtacaggaaatcttatatttgga
ttaataatctctggtatatcttcatgtcttacatctcttaacttttggacaactattcat
ttattgagatcttattatctgatattatcttctattccattatttccttggtctttcttg
attacagctttcatgcttttattaacattaccaatcttatctggaacacttcttttaata
ttgggtgatcttcattctaatacaattttctttgatccaatatttggaggagatcctata
ttctatcaacatttattttggtttttggacatccagaagtttacatattaataattcct
gcatttgggatcatttccataataatttctggtattttacaattaattatttttgctaac
caatcaatgatctttgccatgtcatctatttctcttcttggaggtcttgtttggggacat
catatgtatactgtaggtttagaaagtgatacaagagcttattttacaggagttacaata
ttaatatccttaccaactggtacaaaaatatttaattggctttttacatatctctcaaat
ccaccattattacaccttaggattacttctgtcttcctctcacatctatttttattaatg
tttacgataggtgggtcaacaggaataattcttggaaatggtgcagtggatctaggatta
catgatacatattatgttgtagcacattttcattttgttctttctttaggagctataatt
gctatattctctggaataatcttgaatggagaaaagattgttgctaataagaatttatta
ttttcatcctcatgtacactctctctttatcatttacatttaatatttattggtattctt
cttaccttttcaccaatgcatttcttaggatttaatgtaatggcaagaagaatcccatcc
tttccagattcttttcattcctggaattccctgtcatctattggatcaggaataactttc
ctatctttttctatg
>Isy16_cox1
ataaatctcttaaccattaatttcctatttcattcactagttaaaaattgtaatcataaa
ggcttaggaatctattatttattatctggattcatctttggaatctccggtacattaata
tcagtccttataagaatagaattatattcttcaggaaataggattatatctccagaaaac
cagaacttctataatataagcattacattgcatggctttcttatgattttctttttagta
atgcctggcttgtttggaggatttggaaattattttgtacctatctttcaagggtctcca
gaagtggtatatcctagagtcaataatttttctatcttaattcttttgctttcatatctt
ttcctaatcctttctttaatctcagaatttggaggtggtacagggtggacgctctaccca
ccattatccacttcttttatgactttatcaccttcaagtacaggaaatcttatatttgga
ttaataatctctggtatatcttcatgtcttacatctcttaacttttggacaacaattcat
tttctgagatcttattatctgatattatcttctatcccattatttccttgggctttcttg
attacagctttcatgcttttattaacattaccaatcttatctggtacacttcttttaata
ttgggtgatcttcattctaatacacttttctttgatccaatatttggaggagatcctata
ttctatcaacatttattttggtttttggacatccagaagtttacatattaataattcct
gcatttgggatcatttccataataatttctggtattttacagttaataatctttgctaac
caatcaatgatctttgccatgtcatctatttctcttcttggaggtcttgtttggggacat
catatgtatactgtaggtttagaaagtgatacaagagcttattttacaggagttacaatc
ttaatatccttaccaactggtacaaaaatctttaattggctttttacatatctctcaaat
ccaccattattacaccttagaattacttctgtcttcctctcacatctctttttattaatg
tttacgataggtgggtcaacaggaataattcttggaaatggtgcagtggatctaggatta
catgatacatattatgttgtagcacattttcattttgttctttctttaggagctataatt
gctatcttctctggaataatcttgaatggagaaaagattgttgctactaagaatttatta
ctttcatcctcatgtacactctctctttatcatttacatttaatatttattggtattctt
cttaccttttccccaatgcatttcttaggatttaatgttatgccaagaagaatcccatcc
tttccagattcttttcattcctggaattccctgtcatctattggatcaggaataactttc
ctatctttttctatg
>Isy17_cox1

ataaatctcttaaccattaatttcctatttcattcactagttaaaaattgtaatcataaa
ggcttaggaatctattatttattatctggattcatctttggaatctccggtacattaata
tcagtccttataagaatagaattatattcttcaggaaataggattatatctccagaaaac
cagaacttctataatataagcattacattgcatggctttcttatgattttctttttagta
atgcctggcttgtttggaggatttggaaattattttgtacctatctttcaagggtctcca
gaagtggtatatcctagagtcaataatttttctatcttaattcttttgctttcatatctt
ttcctaatcctttctttaatctcagaatttggaggtggtacagggtggacgctctaccca
ccattatccacttcttttatgactttatcaccttcaagtacaggaaatcttatatttgga
ttaataatctctggtatatcttcatgtcttacatctcttaacttttggacaacaattcat
tttctgagatcttattatctgatattatcttctatcccattatttccttgggctttcttg
attacagctttcatgcttttattaacattaccaatcttatctggtacacttcttttaata
ttgggtgatcttcattctaatacacttttctttgatccaatatttggaggagatcctata
ttctatcaacatttattttggtttttggacatccagaagtttacatattaataattcct
gcatttgggatcatttccataataatttctggtattttacagttaataatctttgctaac
caatcaatgatctttgccatgtcatctatttctcttcttggaggtcttgtttggggacat
catatgtatactgtaggtttagaaagtgatacaagagcttattttacaggagttacaatt
ttaatatccttaccaactggtacaaaaatctttaattggctttttacatatctctcaaat
ccaccattattacaccttagaattacttctgtcttcctctcacatctctttttattaatg
tttacgataggtgggtcaacaggaataattcttggaaatggtgcagtggatctaggatta
catgatacatattatgttgtagcacattttcattttgttctttctttaggagctataatt
gctatcttctctggaataatcttgaatggagaaaagattgttgctactaagaatttatta
ctttcatcctcatgtacactctctctttatcatttacatttaatatttattggtattctt
cttaccttttccccaatgcatttcttaggatttaatgttatgccaagaagaatcccatcc
tttccagattcttttcattcctggaattccctgtcatctattggatcaggaataactttc
ctatctttttctatg
>Isy18_cox1
ataaatctcttaaccattaatttcctatttcattcactagttaaaaattgtaatcataaa
ggcttaggaatctattatttattatctggattcatctttggaatctccggtacattaata
tcagtccttataagaatagaattatattcttcaggaaataggattatatctccagaaaac
cagaacttctataatataagcattacattgcatggctttcttatgattttctttttagta
atgcctggcttgtttggaggatttggaaattattttgtacctatctttcaagggtctcca
gaagtggtatatcctagagtcaataatttttctatcttaattcttttgctttcatatctt
ttcctaatcctttctttaatctcagaatttggaggtggtacagggtggacgctctaccca
ccattatccacttcttttatgactttatcaccttcaagtacaggaaatcttatatttgga
ttaataatctctggtatatcttcatgtcttacatctcttaacttttggacaacaattcat
tttctgagatcttattatctgatattatcttctatcccattatttccttgggctttcttg
attacagctttcatgcttttattaacattaccaatcttatctggtacacttcttttaata
ttgggtgatcttcattctaatacacttttctttgatccaatatttggaggagatcctata
ttctatcaacatttattttggtttttggacatccagaagtttacatattaataattcct
gcatttgggatcatttccataataatttctggtattttacagttaataatctttgctaac
caatcaatgatctttgccatgtcatctatttctcttcttggaggtcttgtttggggacat
catatgtatactgtaggtttagaaagtgatacaagagcttattttacaggagttacaatc
ttaatatccttaccaactggtacaaaaatctttaattggctttttacatatctctcaaat
ccaccattattacaccttagaattacttctgtcttcctctcacatctctttttattaatg
tttacgataggtgggtcaacaggaataattcttggaaatggtgcagtggatctaggatta
catgatacatattatgttgtagcacattttcattttgttctttctttaggagctataatt
gctatcttctctggaataatcttgaatggagaaaagattgttgctactaagaatttatta
ctttcatcctcatgtacactctctctttatcatttacatttaatatttattggtattctt
cttaccttttccccaatgcatttcttaggatttaatgttatgccaagaagaatcccatcc
tttccagattcttttcattcctggaattccctgtcatctattggatcaggaataactttc
ctatctttttctatg
>Isy21_cox1
ttaaatctcttaaccattaatttcctatttcattcactagttaaaaattgtaatcataaa
ggcttaggaatctattatttattatctggattcatctttggaatctccggtacattaata
tcagtccttataagaatagaattatattcttcaggaaataggattatatctccagaaaac
cagaacttctataatataagcattacattgcatggctttcttatgattttctttttagta
atgcctggcttgtttggaggatttggaaattattttgtacctatctttcaagggtctcca

gaagtggtatatcctagagtcaataatttttctatcttaattcttttgctttcatatctt
ttcctaatcctttctttaatctcagaatttggaggtggtacagggtggacgctctaccca
ccattatccacttcttttatgactttatcaccttcaagtacaggaaatcttatatttgga
ttaataatctctggtatatcttcatgtcttacatctcttaacttttggacaacaattcat
tttctgagatcttattatctgatattatcttctatcccattatttccttgggctttcttg
attacagctttcatgcttttattaacattaccaatcttatctggtacacttcttttaata
ttgggtgatcttcattctaatacacttttctttgatccaatatttggaggagatcctata
ttctatcaacatttattttggttttttggacatccagaagtttacatattaataattcct
gcatttgggatcatttccataataatttctggtattttacagttaataatctttgctaac
caatcaatgatctttgccatgtcatctatttctcttcttggaggtcttgtttggggacat
catatgtatactgtaggtttagaaagtgatacaagagcttattttacaggagttacaatt
ttaatatccttaccaactggtacaaaaatctttaattggctttttacatatctctcaaat
ccaccattattacaccttagaattacttctgtcttcctctcacatctctttttattaatg
tttacgataggtgggtcaacaggaataattcttggaaatggtgcagtggatctaggatta
catgatacatattatgttgtagcacattttcattttgttctttctttaggagctataatt
gctatcttctctggaataatcttgaatggagaaaagattgttgctactaagaatttatta
ctttcatcctcatgtacactctctctttatcatttacatttaatatttattggtattctt
cttaccttttccccaatgcatttcttaggatttaatgttatgccaagaagaatcccatcc
tttccagattcttttcattcctggaattccctgtcatctattggatcaggaataactttc
ctatctttttctatg
>Isy22_cox1
ataaatctcttaaccattaatttcctatttcattcactagttaaaaattgtaatcataaa
ggcttaggaatctattatttattatctggattcatctttggaatctccggtacattaata
tcagtccttataagaatagaattatattcttcaggaaataggattatatctccagaaaac
cagaacttctataatataagcattacattgcatggctttcttatgattttctttttagta
atgcctggcttgtttggaggatttggaaattattttgtacctatctttcaagggtctcca
gaagtggtatatcctagagtcaataatttttctatcttaattcttttgctttcatatctt
ttcctaatcctttctttaatctcagaatttggaggtggtacagggtggacgctctaccca
ccattatccacttcttttatgactttatcaccttcaagtacaggaaatcttatatttgga
ttaataatctctggtatatcttcatgtcttacatctcttaacttttggacaacaattcat
tttctgagatcttattatctgatattatcttctatcccattatttccttgggctttcttg
attacagctttcatgcttttattaacattaccaatcttatctggtacacttcttttaata
ttgggtgatcttcattctaatacacttttctttgatccaatatttggaggagatcctata
ttctatcaacatttattttggttttttggacatccagaagtttacatattaataattcct
gcatttgggatcatttccataataatttctggtattttacagttaataatctttgctaac
caatcaatgatctttgccatgtcatctatttctcttcttggaggtcttgtttggggacat
catatgtatactgtaggtttagaaagtgatacaagagcttattttacaggagttacaatc
ttaatatccttaccaactggtacaaaaatctttaattggctttttacatatctctcaaat
ccaccattattacaccttagaattacttctgtcttcctctcacatctctttttattaatg
tttacgataggtgggtcaacaggaataattcttggaaatggtgcagtggatctaggatta
catgatacatattatgttgtagcacattttcattttgttctttctttaggagctataatt
gctatcttctctggaataatcttgaatggagaaaagattgttgctactaagaatttatta
ctttcatcctcatgtacactctctctttatcatttacatttaatatttattggtattctt
cttaccttttccccaatgcatttcttaggatttaatgttatgccaagaagaatcccatcc
tttccagattcttttcattcctggaattccctgtcatctattggatcaggaataactttc
ctatctttttctatg
>Isy23_cox1
ataaatctcttaaccattaatttcctatttcattcactagttaaaaattgtaatcataaa
ggcttaggaatctattatttattatctggattcatctttggaatctccggtacattaata
tcagtccttataagaatagaattatattcttcaggaaataggattatatctccagaaaac
cagaacttctataatataagcattacattgcatggctttcttatgattttctttttagta
atgcctggcttgtttggaggatttggaaattattttgtacctatctttcaagggtctcca
gaagtggtatatcctagagtcaataatttttctatcttaattcttttgctttcatatctt
ttcctaatcctttctttaatctcagaatttggaggtggtacagggtggacgctctaccca
ccattatccacttcttttatgactttatcaccttcaagtacaggaaatcttatatttgga
ttaataatctctggtatatcttcatgtcttacatctcttaacttttggacaacaattcat
tttctgagatcttattatctgatattatcttctatcccattatttccttgggctttcttg

attacagctttcatgcttttattaacattaccaatcttatctggtacacttcttttaata
ttgggtgatcttcattctaatacacttttctttgatccaatatttggaggagatcctata
ttctatcaacatttattttggtttttggacatccagaagtttacatattaataattcct
gcatttgggatcatttccataataatttctggtattttacagttaataatctttgctaac
caatcaatgatctttgccatgtcatctatttctcttcttggaggtcttgtttggggacat
catatgtatactgtaggtttagaaagtgatacaagagcttattttacaggagttacaatt
ttaatatccttaccaactggtacaaaaatctttaattggctttttacatatctctcaaat
ccaccattattacaccttagaattacttctgtcttcctctcacatctctttttattaatg
tttacgataggtgggtcaacaggaataattcttggaaatggtgcagtggatctaggatta
catgatacatattatgttgtagcacattttcattttgttctttctttaggagctataatt
gctatcttctctggaataatcttgaatggagaaaagattgttgctactaagaatttatta
ctttcatcctcatgtacactctctctttatcatttacatttaatatttattggtattctt
cttaccttttccccaatgcatttcttaggatttaatgttatgccaagaagaatcccatcc
tttccagattcttttcattcctggaattccctgtcatctattggatcaggaataactttc
ctatctttttctatg
>Isy24_cox1
ataaatctcttaaccattaatttcctatttcattcactagttaaaaattgtaatcataaa
ggcttaggaatctattatttattatctggattcatctttggaatctccggtacattaata
tcagtccttataagaatagaattatattcttcaggaaataggattatatctccagaaaac
cagaacttctataatataagcattacattgcatggctttcttatgattttctttttagta
atgcctggcttgtttggaggatttggaaattattttgtacctatctttcaagggtctcca
gaagtggtatatcctagagtcaataatttttctatcttaattcttttgctttcatatctt
ttcctaatcctttctttaatctcagaatttggaggtggtacagggtggacgctctaccca
ccattatccacttcttttatgactttatcaccttcaagtacaggaaatcttatatttgga
ttaataatctctggtatatcttcatgtcttacatctcttaacttttggacaacaattcat
tttctgagatcttattatctgatattatcttctatcccattatttccttgggctttcttg
attacagctttcatgcttttattaacattaccaatcttatctggtacacttcttttaata
ttgggtgatcttcattctaatacacttttctttgatccaatatttggaggagatcctata
ttctatcaacatttattttggtttttggacatccagaagtttacatattaataattcct
gcatttgggatcatttccataataatttctggtattttacagttaataatctttgctaac
caatcaatgatctttgccatgtcatctatttctcttcttggaggtcttgtttggggacat
catatgtatactgtaggtttagaaagtgatacaagagcttattttacaggagttacaatc
ttaatatccttaccaactggtacaaaaatctttaattggctttttacatatctctcaaat
ccaccattattacaccttagaattacttctgtcttcctctcacatctctttttattaatg
tttacgataggtgggtcaacaggaataattcttggaaatggtgcagtggatctaggatta
catgatacatattatgttgtagcacattttcattttgttctttctttaggagctataatt
gctatcttctctggaataatcttgaatggagaaaagattgttgctactaagaatttatta
ctttcatcctcatgtacactctctctttatcatttacatttaatatttattggtattctt
cttaccttttccccaatgcatttcttaggatttaatgttatgccaagaagaatcccatcc
tttccagattcttttcattcctggaattccctgtcatctattggatcaggaataactttc
ctatctttttctatg
>Isy25_cox1
ataaatctcttaaccattaatttcctatttcattcactagttaaaaattgtaatcataaa
ggcttaggaatctattatttattatctggattcatctttggaatctccggtacattaata
tcagtccttataagaatagaattatattcttcaggaaataggattatatctccagaaaac
cagaacttctataatataagcattacattgcatggctttcttatgattttctttttagta
atgcctggcttgtttggaggatttggaaattattttgtacctatctttcaagggtctcca
gaagtggtatatcctagagtcaataatttttctatcttaattcttttgctttcatatctt
ttcctaatcctttctttaatctcagaatttggaggtggtacagggtggacgctctaccca
ccattatccacttcttttatgactttatcaccttcaagtacaggaaatcttatatttgga
ttaataatctctggtatatcttcatgtcttacatctcttaacttttggacaacaattcat
tttctgagatcttattatctgatattatcttctatcccattatttccttgggctttcttg
attacagctttcatgcttttattaacattaccaatcttatctggtacacttcttttaata
ttgggtgatcttcattctaatacacttttctttgatccaatatttggaggagatcctata
ttctatcaacatttattttggtttttggacatccagaagtttacatattaataattcct
gcatttgggatcatttccataataatttctggtattttacagttaataatctttgctaac
caatcaatgatctttgccatgtcatctatttctcttcttggaggtcttgtttggggacat

catatgtatactgtaggtttagaaagtgatacaagagcttattttacaggagttacaatt
ttaatatccttaccaactggtacaaaaatctttaattggctttttacatatctctcaaat
ccaccattattacaccttagaattacttctgtcttcctctcacatctctttttattaatg
tttacgataggtgggtcaacaggaataattcttggaaatggtgcagtggatctaggatta
catgatacatattatgttgtagcacattttcattttgttctttctttaggagctataatt
gctatcttctctggaataatcttgaatggagaaaagattgttgctactaagaatttatta
ctttcatcctcatgtacactctctctttatcatttacatttaatatttattggtattctt
cttaccttttccccaatgcatttcttaggatttaatgttatgccaagaagaatcccatcc
tttccagattcttttcattcctggaattccctgtcatctattggatcaggaataactttc
ctatctttttctatg
>Isy26_cox1
ataaatctcttaaccattaatttcctatttcattcactagttaaaaattgtaatcataaa
ggcttaggaatctattatttattatctggattcatctttggaatctccggtacattaata
tcagtccttataagaatagaattatattcttcaggaaataggattatatctccagaaaac
cagaacttctataatataagcattacattgcatggctttcttatgattttctttttagta
atgcctggcttgtttggaggatttggaaattattttgtacctatctttcaagggtctcca
gaagtggtatatcctagagtcaataatttttctatcttaattcttttgctttcatatctt
ttcctaatcctttctttaatctcagaatttggaggtggtacagggtggacgctctaccca
ccattatccacttcttttatgactttatcaccttcaagtacaggaaatcttatatttgga
ttaataatctctggtatatcttcatgtcttacatctcttaacttttggacaacaattcat
tttctgagatcttattatctgatattatcttctatcccattatttccttgggctttcttg
attacagctttcatgcttttattaacattaccaatcttatctggtacacttcttttaata
ttgggtgatcttcattctaatacacttttctttgatccaatatttggaggagatcctata
ttctatcaacatttattttggtttttggacatccagaagtttacatattaataattcct
gcatttgggatcatttccataataatttctggtattttacagttaataatctttgctaac
caatcaatgatctttgccatgtcatctatttctcttcttggaggtcttgtttggggacat
catatgtatactgtaggtttagaaagtgatacaagagcttattttacaggagttacaatt
ttaatatccttaccaactggtacaaaaatctttaattggctttttacatatctctcaaat
ccaccattattacaccttagaattacttctgtcttcctctcacatctctttttattaatg
tttacgataggtgggtcaacaggaataattcttggaaatggtgcagtggatctaggatta
catgatacatattatgttgtagcacattttcattttgttctttctttaggagctataatt
gctatcttctctggaataatcttgaatggagaaaagattgttgctactaagaatttatta
ctttcatcctcatgtacactctctctttatcatttacatttaatatttattggtattctt
cttaccttttccccaatgcatttcttaggatttaatgttatgccaagaagaatcccatcc
tttccagattcttttcattcctggaattccctgtcatctattggatcaggaataactttc
ctatctttttctatg
>Isy27_cox1
ataaatctcttaaccattaatttcctatttcattcactagttaaaaattgtaatcataaa
ggcttaggaatctattatttattatctggattcatctttggaatctccggtacattaata
tcagtccttataagaatagaattatattcttcaggaaataggattatatctccagaaaac
cagaacttctataatataagcattacattgcatggctttcttatgattttctttttagta
atgcctggcttgtttggaggatttggaaattattttgtacctatctttcaagggtctcca
gaagtggtatatcctagagtcaataatttttctatcttaattcttttgctttcatatctt
ttcctaatcctttctttaatctcagaatttggaggtggtacagggtggacgctctaccca
ccattatccacttcttttatgactttatcaccttcaagtacaggaaatcttatatttgga
ttaataatctctggtatatcttcatgtcttacatctcttaacttttggacaacaattcat
tttctgagatcttattatctgatattatcttctatcccattatttccttgggctttcttg
attacagctttcatgcttttattaacattaccaatcttatctggtacacttcttttaata
ttgggtgatcttcattctaatacacttttctttgatccaatatttggaggagatcctata
ttctatcaacatttattttggtttttggacatccagaagtttacatattaataattcct
gcatttgggatcatttccataataatttctggtattttacagttaataatctttgctaac
caatcaatgatctttgccatgtcatctatttctcttcttggaggtcttgtttggggacat
catatgtatactgtaggtttagaaagtgatacaagagcttattttacaggagttacaatt
ttaatatccttaccaactggtacaaaaatctttaattggctttttacatatctctcaaat
ccaccattattacaccttagaattacttctgtcttcctctcacatctctttttattaatg
tttacgataggtgggtcaacaggaataattcttggaaatggtgcagtggatctaggatta
catgatacatattatgttgtagcacattttcattttgttctttctttaggagctataatt

gctatcttctctggaataatcttgaatggagaaaagattgttgctactaagaatttatta
ctttcatcctcatgtacactctctctttatcatttacatttaatatttattggtattctt
cttaccttttccccaatgcatttcttaggatttaatgttatgccaagaagaatcccatcc
tttccagattcttttcattcctggaattccctgtcatctattggatcaggaataactttc
ctatctttttctatg
>Isy4_cox1
ttaaatctcttaaccattaatttcctatttcattcactagttaaaaattgtaatcataaa
ggcttaggaatctattatttattatctggattcatctttggaatctccggtacattaata
tcagtccttataagaatagaattatattcttcaggaaataggattatatctccagaaaac
cagaacttctataatataagcattacattgcatggctttcttatgattttctttttagta
atgcctggcttgtttggaggatttggaaattattttgtacctatctttcaagggtctcca
gaagtggtatatcctagagtcaataatttttctatcttaattcttttgctttcatatctt
ttcctaatcctttctttaatctcagaatttggaggtggtacagggtggacgctctaccca
ccattatccacttcttttatgactttatcaccttcaagtacaggaaatcttatatttgga
ttaataatctctggtatatcttcatgtcttacatctcttaacttttggacaacaattcat
tttctgagatcttattatctgatattatcttctatcccattatttccttgggctttcttg
attacagctttcatgcttttattaacattaccaatcttatctggtacacttcttttaata
ttgggtgatcttcattctaatacacttttctttgatccaatatttggaggagatcctata
ttctatcaacatttattttggttttttggacatccagaagtttacatattaataattcct
gcatttgggatcatttccataataatttctggtattttacagttaataatctttgctaac
caatcaatgatctttgccatgtcatctatttctcttcttggaggtcttgtttggggacat
catatgtatactgtaggtttagaaagtgatacaagagcttattttacaggagttacaatc
ttaatatccttaccaactggtacaaaaatctttaattggctttttacatatctctcaaat
ccaccattattacaccttagaattacttctgtcttcctctcacatctcttttttattaatg
tttacgataggtgggtcaacaggaataattcttggaaatggtgcagtggatctaggatta
catgatacatattatgttgtagcacattttcattttgttctttctttaggagctataatt
gctatcttctctggaataatcttgaatggagaaaagattgttgctactaagaatttatta
ctttcatcctcatgtacactctctctttatcatttacatttaatatttattggtattctt
cttaccttttccccaatgcatttcttaggatttaatgttatgccaagaagaatcccatcc
tttccagattcttttcattcctggaattccctgtcatctattggatcaggaataactttc
ctatctttttctatg
>Isy7_cox1
ataaatctcttaaccattaatttcctatttcattcactagttaaaaattgtaatcataaa
ggcttaggaatctattatttattatctggattcatctttggaatctccggtacattaata
tcagtccttataagaatagaattatattcttcaggaaataggattatatctccagaaaac
cagaacttctataatataagcattacattgcatggctttcttatgattttctttttagta
atgcctggcttgtttggaggatttggaaattattttgtacctatctttcaagggtctcca
gaagtggtatatcctagagtcaataatttttctatcttaattcttttgctttcatatctt
ttcctaatcctttctttaatctcagaatttggaggtggtacaggatggacgctctaccca
ccattatccacttcttttatgactttatcaccttcaagtacaggaaatcttatatttgga
ttaataatctctggtatatcttcatgtcttacatctcttaacttttggacaacaattcat
tttctgagatcttattatctgatattatcttctatcccattatttccttgggctttcttg
attacagctttcatgcttttattaacattaccaatcttatctggtacacttcttttaata
ttgggtgatcttcattctaatacacttttctttgatccaatatttggaggagatcctata
ttctatcaacatttattttggttttttggacatccagaagtttacatattaataattcct
gcatttgggatcatttccataataatttctggtattttacaattaattattttttgctaac
caatcaatgatctttgccatgtcatctatttctcttcttggaggtcttgtttggggacat
catatgtatactgtaggtttagaaagtgatacaagagcttattttacaggagttacaatc
ttaatatccttaccaactggtacaaaaatctttaattggctttttacatatctctcaaat
ccaccattattacaccttagaattacttctgtcttcctctcacatctcttttttattaatg
tttacgataggtgggtcaacaggaataattcttggaaatggtgcagtggatctaggatta
catgatacatattatgttgtagcacattttcattttgttctttctttaggagctataatt
gctatcttctctggaataatcttgaatggagaaaagattgttgctactaagaatttatta
ctttcatcctcatgtacactctctctttatcatttacatttaatatttattggtattctt
cttaccttttccccaatgcatttcttaggatttaatgttatgccaagaagaatcccatcc
tttccagattcttttcattcctggaattccctgtcatctattggatcaggaataactttc
ctatctttttctatg

>Isy8_cox1
ataaatctcttaaccattaatttcctatttcattcactagttaaaaattgtaatcataaa
ggcttaggaatctattatttattatctggattcatctttggaatctccggtacattaata
tcagtccttataagaatagaattatattcttcaggaaataggattatatctccagaaaac
cagaacttctataatataagcattacattgcatggctttcttatgattttctttttagta
atgcctggcttgtttggaggatttggaaattattttgtacctatctttcaagggtctcca
gaagtggtatatcctagagtcaataatttttctatcttaattcttttgctttcatatctt
ttcctaatcctttctttaatctcagaatttggaggtggtacagggtggacgctctaccca
ccattatccacttcttttatgactttatcaccttcaagtacaggaaatcttatatttgga
ttaataatctctggtatatcttcatgtcttacatctcttaacttttggacaacaattcat
tttctgagatcttattatctgatattatcttctatcccattatttccttgggctttcttg
attacagctttcatgcttttattaacattaccaatcttatctggtacacttcttttaata
ttgggtgatcttcattctaatacacttttctttgatccaatatttggaggagatcctata
ttctatcaacatttattttggtttttggacatccagaagtttacatattaataattcct
gcatttgggatcatttccataataatttctggtattttacagttaataatctttgctaac
caatcaatgatctttgccatgtcatctatttctcttcttggaggtcttgtttggggacat
catatgtatactgtaggtttagaaagtgatacaagagcttattttacaggagttacaatt
ttaatatccttaccaactggtacaaaaatctttaattggctttttacatatctctcaaat
ccaccattattacaccttagaattacttctgtcttcctctcacatctctttttattaatg
tttacgataggtgggtcaacaggaataattcttggaaatggtgcagtggatctaggatta
catgatacatattatgttgtagcacattttcattttgttctttctttaggagctataatt
gctatcttctctggaataatcttgaatggagaaaagattgttgctactaagaatttatta
ctttcatcctcatgtacactctctctttatcatttacatttaatatttattggtattctt
cttaccttttccccaatgcatttcttaggatttaatgttatgccaagaagaatcccatcc
tttccagattcttttcattcctggaattccctgtcatctattggatcaggaataactttc
ctatctttttctatg
>KrA1_cox1
ttaaatctcttaaccattaatttcctatttcattcactagttaaaaattgtaatcataaa
ggcttaggaatctattatttattatctggattcatctttggaatctccggtacattaata
tcagtccttataagaatagaattatattcttcaggaaataggattatatctccagaaaac
cagaacttctataatataagcattacattgcatggctttcttatgattttctttttagta
atgcctggcttgtttggaggatttggaaattattttgtacctatctttcaagggtctcca
gaagtggtatatcctagagtcaataatttttctatcttaattcttttgctttcatatctt
ttcctaatcctttctttaatctcagaatttggaggtggtacagggtggacgctctaccca
ccattatccacttcttttatgactttatcaccttcaagtacaggaaatcttatatttgga
ttaataatctctggtatatcttcatgtcttacatctcttaacttttggacaacaattcat
tttctgagatcttattatctgatattatcttctatcccattatttccttgggctttcttg
attacagctttcatgcttttattaacattaccaatcttatctggtacacttcttttaata
ttgggtgatcttcattctaatacacttttctttgatccaatatttggaggagatcctata
ttctatcaacatttattttggtttttggacatccagaagtttacatattaataattcct
gcatttgggatcatttccataataatttctggtattttacagttaataatctttgctaac
caatcaatgatctttgccatgtcatctatttctcttcttggaggtcttgtttggggacat
catatgtatactgtaggtttagaaagtgatacaagagcttattttacaggagttacaatc
ttaatatccttaccaactggtacaaaaatctttaattggctttttacatatctctcaaat
ccaccattattacaccttagaattacttctgtcttcctctcacatctctttttattaatg
tttacgataggtgggtcaacaggaataattcttggaaatggtgcagtggatctaggatta
catgatacatattatgttgtagcacattttcattttgttctttctttaggagctataatt
gctatcttctctggaataatcttgaatggagaaaagattgttgctactaagaatttatta
ctttcatcctcatgtacactctctctttatcatttacatttaatatttattggtattctt
cttaccttttccccaatgcatttcttaggatttaatgttatgccaagaagaatcccatcc
tttccagattcttttcattcctggaattccctgtcatctattggatcaggaataactttc
ctatctttttctatg
>KrA10_cox1
ataaatctcttaaccattaatttcctatttcattcactagttaaaaattgtaatcataaa
ggcttaggaatctattatttattatctggattcatctttggaatctccggtacattaata
tcagtccttataagaatagaattatattcttcaggaaataggattatatctccagaaaac
cagaacttctataatataagcattacattgcatggctttcttatgattttctttttagta

atgcctggcttgtttggaggatttggaaattattttgtacctatctttcaagggtctcca
gaagtggtatatcctagagtcaataatttttctatcttaattcttttgctttcatatctt
ttcctaatcctttctttaatctcagaatttggaggtggtacagggtggacgctctaccca
ccattatccacttcttttatgactttatcaccttcaagtacaggaaatcttatatttgga
ttaataatctctggtatatcttcatgtcttacatctcttaacttttggacaacaattcat
tttctgagatcttattatctgatattatcttctatcccattatttccttgggctttcttg
attacagctttcatgcttttattaacattaccaatcttatctggtacacttcttttaata
ttgggtgatcttcattctaatacacttttctttgatccaatatttggaggagatcctata
ttctatcaacatttattttggtttttggacatccagaagtttacatattaataattcct
gcatttgggatcatttccataataatttctggtattttacagttaataatctttgctaac
caatcaatgatctttgccatgtcatctatttctcttcttggaggtcttgtttggggacat
catatgtatactgtaggtttagaaagtgatacaagagcttattttacaggagttacaatc
ttaatatccttaccaactggtacaaaaatctttaattggctttttacatatctctcaaat
ccaccattattacaccttagaattacttctgtcttcctctcacatctctttttattaatg
tttacgataggtgggtcaacaggaataattcttggaaatggtgcagtggatctaggatta
catgatacatattatgttgtagcacattttcattttgttctttctttaggagctataatt
gctatcttctctggaataatcttgaatggagaaaagattgttgctactaagaatttatta
ctttcatcctcatgtacactctctctttatcatttacatttaatatttattggtattctt
cttaccttttccccaatgcatttcttaggatttaatgttatgccaagaagaatcccatcc
tttccagattcttttcattcctggaattccctgtcatctattggatcaggaataactttc
ctatctttttctatg
>KrA11_cox1
ataaatctcttaaccattaatttcctatttcattcactagttaaaaattgtaatcataaa
ggcttaggaatctattatttattatctggattcatctttggaatctccggtacattaata
tcagtccttataagaatagaattatattcttcaggaaataggattatatctccagaaaac
cagaacttctataatataagcattacattgcatggctttcttatgattttctttttagta
atgcctggcttgtttggaggatttggaaattattttgtacctatctttcaagggtctcca
gaagtggtatatcctagagtcaataatttttctatcttaattcttttgctttcatatctt
ttcctaatcctttctttaatctcagaatttggaggtggtacagggtggacgctctaccca
ccattatccacttcttttatgactttatcaccttcaagtacaggaaatcttatatttgga
ttaataatctctggtatatcttcatgtcttacatctcttaacttttggacaacaattcat
tttctgagatcttattatctgatattatcttctatcccattatttccttgggctttcttg
attacagctttcatgcttttattaacattaccaatcttatctggtacacttcttttaata
ttgggtgatcttcattctaatacacttttctttgatccaatatttggaggagatcctata
ttctatcaacatttattttggtttttggacatccagaagtttacatattaataattcct
gcatttgggatcatttccataataatttctggtattttacagttaataatctttgctaac
caatcaatgatctttgccatgtcatctatttctcttcttggaggtcttgtttggggacat
catatgtatactgtaggtttagaaagtgatacaagagcttattttacaggagttacaatc
ttaatatccttaccaactggtacaaaaatctttaattggctttttacatatctctcaaat
ccaccattattacaccttagaattacttctgtcttcctctcacatctctttttattaatg
tttacgataggtgggtcaacaggaataattcttggaaatggtgcagtggatctaggatta
catgatacatattatgttgtagcacattttcattttgttctttctttaggagctataatt
gctatcttctctggaataatcttgaatggagaaaagattgttgctactaagaatttatta
ctttcatcctcatgtacactctctctttatcatttacatttaatatttattggtattctt
cttaccttttccccaatgcatttcttaggatttaatgttatgccaagaagaatcccatcc
tttccagattcttttcattcctggaattccctgtcatctattggatcaggaataactttc
ctatctttttctatg
>KrA12_cox1
ataaatctcttaaccattaatttcctatttcattcactagttaaaaattgtaatcataaa
ggcttaggaatctattatttattatctggattcatctttggaatctccggtacattaata
tcagtccttataagaatagaattatattcttcaggaaataggattatatctccagaaaac
cagaacttctataatataagcattacattgcatggctttcttatgattttctttttagta
atgcctggcttgtttggaggatttggaaattattttgtacctatctttcaagggtctcca
gaagtggtatatcctagagtcaataatttttctatcttaattcttttgctttcatatctt
ttcctaatcctttctttaatctcagaatttggaggtggtacagggtggacgctctaccca
ccattatccacttcttttatgactttatcaccttcaagtacaggaaatcttatatttgga
ttaataatctctggtatatcttcatgtcttacatctcttaacttttggacaacaattcat

tttctgagatcttattatctgatattatcttctatcccattatttccttgggctttcttg
attacagctttcatgcttttattaacattaccaatcttatctggtacacttcttttaata
ttgggtgatcttcattctaatacacttttctttgatccaatatttggaggagatcctata
ttctatcaacatttattttggtttttggacatccagaagtttacatattaataattcct
gcatttgggatcatttccataataatttctggtattttacagttaataatctttgctaac
caatcaatgatctttgccatgtcatctatttctcttcttggaggtcttgtttggggacat
catatgtatactgtaggtttagaaagtgatacaagagcttattttacaggagttacaatc
ttaatatccttaccaactggtacaaaaatctttaattggctttttacatatctctcaaat
ccaccattattacaccttagaattacttctgtcttcctctcacatctctttttattaatg
tttacgataggtgggtcaacaggaataattcttggaaatggtgcagtggatctaggatta
catgatacatattatgttgtagcacattttcattttgttctttctttaggagctataatt
gctatcttctctggaataatcttgaatggagaaaagattgttgctactaagaatttatta
ctttcatcctcatgtacactctctctttatcatttacatttaatatttattggtattctt
cttaccttttccccaatgcatttcttaggatttaatgttatgccaagaagaatcccatcc
tttccagattcttttcattcctggaattccctgtcatctattggatcaggaataactttc
ctatctttttctatg
>KrA13_cox1
ataaatctcttaaccattaatttcctatttcattcactagttaaaaattgtaatcataaa
ggcttaggaatctattatttattatctggattcatctttggaatctccggtacattaata
tcagtccttataagaatagaattatattcttcaggaaataggattatatctccagaaaac
cagaacttctataatataagcattacattgcatggctttcttatgattttctttttagta
atgcctggcttgtttggaggatttggaaattattttgtacctatctttcaagggtctcca
gaagtggtatatcctagagtcaataatttttctatcttaattcttttgctttcatatctt
ttcctaatcctttctttaatctcagaatttggaggtggtacagggtggacgctctaccca
ccattatccacttcttttatgactttatcaccttcaagtacaggaaatcttatatttgga
ttaataatctctggtatatcttcatgtcttacatctcttaacttttggacaacaattcat
tttctgagatcttattatctgatattatcttctatcccattatttccttgggctttcttg
attacagctttcatgcttttattaacattaccaatcttatctggtacacttcttttaata
ttgggtgatcttcattctaatacacttttctttgatccaatatttggaggagatcctata
ttctatcaacatttattttggtttttggacatccagaagtttacatattaataattcct
gcatttgggatcatttccataataatttctggtattttacagttaataatctttgctaac
caatcaatgatctttgccatgtcatctatttctcttcttggaggtcttgtttggggacat
catatgtatactgtaggtttagaaagtgatacaagagcttattttacaggagttacaatc
ttaatatccttaccaactggtacaaaaatctttaattggctttttacatatctctcaaat
ccaccattattacaccttagaattacttctgtcttcctctcacatctctttttattaatg
tttacgataggtgggtcaacaggaataattcttggaaatggtgcagtggatctaggatta
catgatacatattatgttgtagcacattttcattttgttctttctttaggagctataatt
gctatcttctctggaataatcttgaatggagaaaagattgttgctactaagaatttatta
ctttcatcctcatgtacactctctctttatcatttacatttaatatttattggtattctt
cttaccttttccccaatgcatttcttaggatttaatgttatgccaagaagaatcccatcc
tttccagattcttttcattcctggaattccctgtcatctattggatcaggaataactttc
ctatctttttctatg
>KrA14_cox1
ataaatctcttaaccattaatttcctatttcattcactagttaaaaattgtaatcataaa
ggcttaggaatctattatttattatctggattcatctttggaatctccggtacattaata
tcagtccttataagaatagaattatattcttcaggaaataggattatatctccagaaaac
cagaacttctataatataagcattacattgcatggctttcttatgattttctttttagta
atgcctggcttgtttggaggatttggaaattattttgtacctatctttcaagggtctcca
gaagtggtatatcctagagtcaataatttttctatcttaattcttttgctttcatatctt
ttcctaatcctttctttaatctcagaatttggaggtggtacagggtggacgctctaccca
ccattatccacttcttttatgactttatcaccttcaagtacaggaaatcttatatttgga
ttaataatctctggtatatcttcatgtcttacatctcttaacttttggacaacaattcat
tttctgagatcttattatctgatattatcttctatcccattatttccttgggctttcttg
attacagctttcatgcttttattaacattaccaatcttatctggtacacttcttttaata
ttgggtgatcttcattctaatacacttttctttgatccaatatttggaggagatcctata
ttctatcaacatttattttggtttttggacatccagaagtttacatattaataattcct
gcatttgggatcatttccataataatttctggtattttacagttaataatctttgctaac

caatcaatgatctttgccatgtcatctatttctcttcttggaggtcttgtttggggacat
catatgtatactgtaggtttagaaagtgatacaagagcttattttacaggagttacaatc
ttaatatccttaccaactggtacaaaaatctttaattggctttttacatatctctcaaat
ccaccattattacaccttagaattacttctgtcttcctctcacatctctttttattaatg
tttacgataggtgggtcaacaggaataattcttggaaatggtgcagtggatctaggatta
catgatacatattatgttgtagcacattttcattttgttctttctttaggagctataatt
gctatcttctctggaataatcttgaatggagaaaagattgttgctactaagaatttatta
ctttcatcctcatgtacactctctctttatcatttacatttaatatttattggtattctt
cttaccttttccccaatgcatttcttaggatttaatgttatgccaagaagaatcccatcc
tttccagattcttttcattcctggaattccctgtcatctattggatcaggaataactttc
ctatctttttctatg
>KrA15_cox1
ataaatctcttaaccattaatttcctatttcattcactagttaaaaattgtaatcataaa
ggcttaggaatctattatttattatctggattcatctttggaatctccggtacattaata
tcagtccttataagaatagaattatattcttcaggaaataggattatatctccagaaaac
cagaacttctataatataagcattacattgcatggctttcttatgattttctttttagta
atgcctggcttgtttggaggatttggaaattattttgtacctatctttcaagggtctcca
gaagtggtatatcctagagtcaataatttttctatcttaattcttttgctttcatatctt
ttcctaatcctttctttaatctcagaatttggaggtggtacagggtggacgctctaccca
ccattatccacttcttttatgactttatcaccttcaagtacaggaaatcttatatttgga
ttaataatctctggtatatcttcatgtcttacatctcttaacttttggacaacaattcat
tttctgagatcttattatctgatattatcttctatcccattatttccttgggctttcttg
attacagctttcatgcttttattaacattaccaatcttatctggtacacttcttttaata
ttgggtgatcttcattctaatacacttttctttgatccaatatttggaggagatcctata
ttctatcaacatttattttggtttttggacatccagaagtttacatattaataattcct
gcatttgggatcatttccataataatttctggtattttacagttaataatctttgctaac
caatcaatgatctttgccatgtcatctatttctcttcttggaggtcttgtttggggacat
catatgtatactgtaggtttagaaagtgatacaagagcttattttacaggagttacaatc
ttaatatccttaccaactggtacaaaaatctttaattggctttttacatatctctcaaat
ccaccattattacaccttagaattacttctgtcttcctctcacatctctttttattaatg
tttacgataggtgggtcaacaggaataattcttggaaatggtgcagtggatctaggatta
catgatacatattatgttgtagcacattttcattttgttctttctttaggagctataatt
gctatcttctctggaataatcttgaatggagaaaagattgttgctactaagaatttatta
ctttcatcctcatgtacactctctctttatcatttacatttaatatttattggtattctt
cttaccttttccccaatgcatttcttaggatttaatgttatgccaagaagaatcccatcc
tttccagattcttttcattcctggaattccctgtcatctattggatcaggaataactttc
ctatctttttctatg
>KrA2_cox1
ttaaatctcttaaccattaatttcctatttcattcactagttaaaaattgtaatcataaa
ggcttaggaatctattatttattatctggattcatctttggaatctccggtacattaata
tcagtccttataagaatagaattatattcttcaggaaataggattatatctccagaaaac
cagaacttctataatataagcattacattgcatggctttcttatgattttctttttagta
atgcctggcttgtttggaggatttggaaattattttgtacctatctttcaagggtctcca
gaagtggtatatcctagagtcaataatttttctatcttaattcttttgctttcatatctt
ttcctaatcctttctttaatctcagaatttggaggtggtacagggtggacgctctaccca
ccattatccacttcttttatgactttatcaccttcaagtacaggaaatcttatatttgga
ttaataatctctggtatatcttcatgtcttacatctcttaacttttggacaacaattcat
tttctgagatcttattatctgatattatcttctatcccattatttccttgggctttcttg
attacagctttcatgcttttattaacattaccaatcttatctggtacacttcttttaata
ttgggtgatcttcattctaatacacttttctttgatccaatatttggaggagatcctata
ttctatcaacatttattttggtttttggacatccagaagtttacatattaataattcct
gcatttgggatcatttccataataatttctggtattttacagttaataatctttgctaac
caatcaatgatctttgccatgtcatctatttctcttcttggaggtcttgtttggggacat
catatgtatactgtaggtttagaaagtgatacaagagcttattttacaggagttacaatc
ttaatatccttaccaactggtacaaaaatctttaattggctttttacatatctctcaaat
ccaccattattacaccttagaattacttctgtcttcctctcacatctctttttattaatg
tttacgataggtgggtcaacaggaataattcttggaaatggtgcagtggatctaggatta

catgatacatattatgttgtagcacattttcattttgttctttctttaggagctataatt
gctatcttctctggaataatcttgaatggagaaaagattgttgctactaagaatttatta
ctttcatcctcatgtacactctctctttatcatttacatttaatatttattggtattctt
cttaccttttccccaatgcatttcttaggatttaatgttatgccaagaagaatcccatcc
tttccagattcttttcattcctggaattccctgtcatctattggatcaggaataactttc
ctatctttttctatg
>KrA3_cox1
ataaatctcttaaccattaatttcctatttcattcactagttaaaaattgtaatcataaa
ggcttaggaatctattatttattatctggattcatctttggaatctccggtacattaata
tcagtccttataagaatagaattatattcttcaggaaataggattatatctccagaaaac
cagaacttctataatataagcattacattgcatggctttcttatgattttctttttagta
atgcctggcttgtttggaggatttggaaattattttgtacctatctttcaagggtctcca
gaagtggtatatcctagagtcaataatttttctatcttaattcttttgctttcatatctt
ttcctaatccctttctttaatctcagaatttggaggtggtacagggtggacgctctaccca
ccattatccacttcttttatgactttatcaccttcaagtacaggaaatcttatatttgga
ttaataatctctggtatatcttcatgtcttacatctcttaacttttggacaacaattcat
tttctgagatcttattatctgatattatcttctatcccattatttccttgggctttcttg
attacagctttcatgcttttattaacattaccaatcttatctggtacacttcttttaata
ttgggtgatcttcattctaatacacttttctttgatccaatatttggaggagatcctata
ttctatcaacatttattttggttttttggacatccagaagtttacatattaataattcct
gcatttgggatcatttccataataatttctggtattttacagttaataatctttgctaac
caatcaatgatctttgccatgtcatctatttctcttcttggaggtcttgtttggggacat
catatgtatactgtaggtttagaaagtgatacaagagcttattttacaggagttacaatc
ttaatatccttaccaactggtacaaaaatctttaattggctttttacatatctctcaaat
ccaccattattacaccttagaattacttctgtcttcctctcacatctctttttattaatg
tttacgataggtgggtcaacaggaataattcttggaaatggtgcagtggatctaggatta
catgatacatattatgttgtagcacattttcattttgttctttctttaggagctataatt
gctatcttctctggaataatcttgaatggagaaaagattgttgctactaagaatttatta
ctttcatcctcatgtacactctctctttatcatttacatttaatatttattggtattctt
cttaccttttccccaatgcatttcttaggatttaatgttatgccaagaagaatcccatcc
tttccagattcttttcattcctggaattccctgtcatctattggatcaggaataactttc
ctatctttttctatg
>KrA4_cox1
ataaatctcttaaccattaatttcctatttcattcactagttaaaaattgtaatcataaa
ggcttaggaatctattatttattatctggattcatctttggaatctccggtacattaata
tcagtccttataagaatagaattatattcttcaggaaataggattatatctccagaaaac
cagaacttctataatataagcattacattgcatggctttcttatgattttctttttagta
atgcctggcttgtttggaggatttggaaattattttgtacctatctttcaagggtctcca
gaagtggtatatcctagagtcaataatttttctatcttaattcttttgctttcatatctt
ttcctaatccctttctttaatctcagaatttggaggtggtacagggtggacgctctaccca
ccattatccacttcttttatgactttatcaccttcaagtacaggaaatcttatatttgga
ttaataatctctggtatatcttcatgtcttacatctcttaacttttggacaacaattcat
tttctgagatcttattatctgatattatcttctatcccattatttccttgggctttcttg
attacagctttcatgcttttattaacattaccaatcttatctggtacacttcttttaata
ttgggtgatcttcattctaatacacttttctttgatccaatatttggaggagatcctata
ttctatcaacatttattttggttttttggacatccagaagtttacatattaataattcct
gcatttgggatcatttccataataatttctggtattttacagttaataatctttgctaac
caatcaatgatctttgccatgtcatctatttctcttcttggaggtcttgtttggggacat
catatgtatactgtaggtttagaaagtgatacaagagcttattttacaggagttacaatc
ttaatatccttaccaactggtacaaaaatctttaattggctttttacatatctctcaaat
ccaccattattacaccttagaattacttctgtcttcctctcacatctctttttattaatg
tttacgataggtgggtcaacaggaataattcttggaaatggtgcagtggatctaggatta
catgatacatattatgttgtagcacattttcattttgttctttctttaggagctataatt
gctatcttctctggaataatcttgaatggagaaaagattgttgctactaagaatttatta
ctttcatcctcatgtacactctctctttatcatttacatttaatatttattggtattctt
cttaccttttccccaatgcatttcttaggatttaatgttatgccaagaagaatcccatcc
tttccagattcttttcattcctggaattccctgtcatctattggatcaggaataactttc

ctatctttttctatg
>KrA5_cox1
ataaatctcttaaccattaatttcctatttcattcactagttaaaaattgtaatcataaa
ggcttaggaatctattatttattatctggattcatctttggaatctccggtacattaata
tcagtccttataagaatagaattatattcttcaggaaataggattatatctccagaaaac
cagaacttctataatataagcattacattgcatggctttcttatgattttctttttagta
atgcctggcttgtttggaggatttggaaattattttgtacctatctttcaagggtctcca
gaagtggtatatcctagagtcaataatttttctatcttaattcttttgctttcatatctt
ttcctaatcctttctttaatctcagaatttggaggtggtacagggtggacgctctaccca
ccattatccacttcttttatgactttatcaccttcaagtacaggaaatcttatatttgga
ttaataatctctggtatatcttcatgtcttacatctcttaacttttggacaacaattcat
tttctgagatcttattatctgatattatcttctatcccattatttccttgggctttcttg
attacagctttcatgcttttattaacattaccaatcttatctggtacacttcttttaata
ttgggtgatcttcattctaatacacttttctttgatccaatatttggaggagatcctata
ttctatcaacatttattttggttttttggacatccagaagtttacatattaataattcct
gcatttgggatcatttccataataatttctggtattttacagttaataatctttgctaac
caatcaatgatctttgccatgtcatctatttctcttcttggaggtcttgtttggggacat
catatgtatactgtaggtttagaaagtgatacaagagcttattttacaggagttacaatc
ttaatatccttaccaactggtacaaaaatctttaattggctttttacatatctctcaaat
ccaccattattacaccttagaattacttctgtcttcctctcacatctctttttattaatg
tttacgataggtgggtcaacaggaataattcttggaaatggtgcagtggatctaggatta
catgatacatattatgttgtagcacattttcattttgttctttctttaggagctataatt
gctatcttctctggaataatcttgaatggagaaaagattgttgctactaagaatttatta
ctttcatcctcatgtacactctctctttatcatttacatttaatatttattggtattctt
cttaccttttccccaatgcatttcttaggatttaatgttatgccaagaagaatcccatcc
tttccagattcttttcattcctggaattccctgtcatctattggatcaggaataactttc
ctatctttttctatg
>KrA6_cox1
ataaatctcttaaccattaatttcctatttcattcactagttaaaaattgtaatcataaa
ggcttaggaatctattatttattatctggattcatctttggaatctccggtacattaata
tcagtccttataagaatagaattatattcttcaggaaataggattatatctccagaaaac
cagaacttctataatataagcattacattgcatggctttcttatgattttctttttagta
atgcctggcttgtttggaggatttggaaattattttgtacctatctttcaagggtctcca
gaagtggtatatcctagagtcaataatttttctatcttaattcttttgctttcatatctt
ttcctaatcctttctttaatctcagaatttggaggtggtacagggtggacgctctaccca
ccattatccacttcttttatgactttatcaccttcaagtacaggaaatcttatatttgga
ttaataatctctggtatatcttcatgtcttacatctcttaacttttggacaacaattcat
tttctgagatcttattatctgatattatcttctatcccattatttccttgggctttcttg
attacagctttcatgcttttattaacattaccaatcttatctggtacacttcttttaata
ttgggtgatcttcattctaatacacttttctttgatccaatatttggaggagatcctata
ttctatcaacatttattttggttttttggacatccagaagtttacatattaataattcct
gcatttgggatcatttccataataatttctggtattttacagttaataatctttgctaac
caatcaatgatctttgccatgtcatctatttctcttcttggaggtcttgtttggggacat
catatgtatactgtaggtttagaaagtgatacaagagcttattttacaggagttacaatc
ttaatatccttaccaactggtacaaaaatctttaattggctttttacatatctctcaaat
ccaccattattacaccttagaattacttctgtcttcctctcacatctctttttattaatg
tttacgataggtgggtcaacaggaataattcttggaaatggtgcagtggatctaggatta
catgatacatattatgttgtagcacattttcattttgttctttctttaggagctataatt
gctatcttctctggaataatcttgaatggagaaaagattgttgctactaagaatttatta
ctttcatcctcatgtacactctctctttatcatttacatttaatatttattggtattctt
cttaccttttccccaatgcatttcttaggatttaatgttatgccaagaagaatcccatcc
tttccagattcttttcattcctggaattccctgtcatctattggatcaggaataactttc
ctatctttttctatg
>KrA7_cox1
ataaatctcttaaccattaatttcctatttcattcactagttaaaaattgtaatcataaa
ggcttaggaatctattatttattatctggattcatctttggaatctccggtacattaata
tcagtccttataagaatagaattatattcttcaggaaataggattatatctccagaaaac

cagaacttctataatataagcattacattgcatggctttcttatgattttctttttagta
atgcctggcttgtttggaggatttggaaattattttgtacctatctttcaagggtctcca
gaagtggtatatcctagagtcaataatttttctatcttaattcttttgctttcatatctt
ttcctaatcctttctttaatctcagaatttggaggtggtacagggtggacgctctaccca
ccattatccacttcttttatgactttatcaccttcaagtacaggaaatcttatatttgga
ttaataatctctggtatatcttcatgtcttacatctcttaacttttggacaacaattcat
tttctgagatcttattatctgatattatcttctatcccattatttccttgggctttcttg
attacagctttcatgcttttattaacattaccaatcttatctggtacacttcttttaata
ttgggtgatcttcattctaatacacttttctttgatccaatatttggaggagatcctata
ttctatcaacatttattttggtttttggacatccagaagtttacatattaataattcct
gcatttgggatcatttccataataatttctggtattttacagttaataatctttgctaac
caatcaatgatctttgccatgtcatctatttctcttcttggaggtcttgtttggggacat
catatgtatactgtaggtttagaaagtgatacaagagcttattttacaggagttacaatc
ttaatatccttaccaactggtacaaaaatctttaattggctttttacatatctctcaaat
ccaccattattacaccttagaattacttctgtcttcctctcacatctcttttattaatg
tttacgataggtgggtcaacaggaataattcttggaaatggtgcagtggatctaggatta
catgatacatattatgttgtagcacattttcattttgttctttctttaggagctataatt
gctatcttctctggaataatcttgaatggagaaaagattgttgctactaagaatttatta
ctttcatcctcatgtacactctctctttatcatttacatttaatatttattggtattctt
cttacctttccccaatgcatttcttaggatttaatgttatgccaagaagaatcccatcc
tttccagattcttttcattcctggaattccctgtcatctattggatcaggaataactttc
ctatctttttctatg
>KrA8_cox1
ataaatctcttaaccattaatttcctatttcattcactagttaaaaattgtaatcataaa
ggcttaggaatctattatttattatctggattcatctttggaatctccggtacattaata
tcagtccttataagaatagaattatattcttcaggaaataggattatatctccagaaaac
cagaacttctataatataagcattacattgcatggctttcttatgattttctttttagta
atgcctggcttgtttggaggatttggaaattattttgtacctatctttcaagggtctcca
gaagtggtatatcctagagtcaataatttttctatcttaattcttttgctttcatatctt
ttcctaatcctttctttaatctcagaatttggaggtggtacagggtggacgctctaccca
ccattatccacttcttttatgactttatcaccttcaagtacaggaaatcttatatttgga
ttaataatctctggtatatcttcatgtcttacatctcttaacttttggacaacaattcat
tttctgagatcttattatctgatattatcttctatcccattatttccttgggctttcttg
attacagctttcatgcttttattaacattaccaatcttatctggtacacttcttttaata
ttgggtgatcttcattctaatacacttttctttgatccaatatttggaggagatcctata
ttctatcaacatttattttggtttttggacatccagaagtttacatattaataattcct
gcatttgggatcatttccataataatttctggtattttacagttaataatctttgctaac
caatcaatgatctttgccatgtcatctatttctcttcttggaggtcttgtttggggacat
catatgtatactgtaggtttagaaagtgatacaagagcttattttacaggagttacaatc
ttaatatccttaccaactggtacaaaaatctttaattggctttttacatatctctcaaat
ccaccattattacaccttagaattacttctgtcttcctctcacatctcttttattaatg
tttacgataggtgggtcaacaggaataattcttggaaatggtgcagtggatctaggatta
catgatacatattatgttgtagcacattttcattttgttctttctttaggagctataatt
gctatcttctctggaataatcttgaatggagaaaagattgttgctactaagaatttatta
ctttcatcctcatgtacactctctctttatcatttacatttaatatttattggtattctt
cttacctttccccaatgcatttcttaggatttaatgttatgccaagaagaatcccatcc
tttccagattcttttcattcctggaattccctgtcatctattggatcaggaataactttc
ctatctttttctatg
>KrA9_cox1
ataaatctcttaaccattaatttcctatttcattcactagttaaaaattgtaatcataaa
ggcttaggaatctattatttattatctggattcatctttggaatctccggtacattaata
tcagtccttataagaatagaattatattcttcaggaaataggattatatctccagaaaac
cagaacttctataatataagcattacattgcatggctttcttatgattttctttttagta
atgcctggcttgtttggaggatttggaaattattttgtacctatctttcaagggtctcca
gaagtggtatatcctagagtcaataatttttctatcttaattcttttgctttcatatctt
ttcctaatcctttctttaatctcagaatttggaggtggtacagggtggacgctctaccca
ccattatccacttcttttatgactttatcaccttcaagtacaggaaatcttatatttgga

ttaataatctctggtatatcttcatgtcttacatctcttaacttttggacaacaattcat
tttctgagatcttattatctgatattatcttctatcccattatttccttgggctttcttg
attacagctttcatgcttttattaacattaccaatcttatctggtacacttcttttaata
ttgggtgatcttcattctaatacacttttctttgatccaatatttggaggagatcctata
ttctatcaacatttattttggttttttggacatccagaagtttacatattaataattcct
gcatttgggatcatttccataataatttctggtattttacagttaataatctttgctaac
caatcaatgatctttgccatgtcatctatttctcttcttggaggtcttgtttggggacat
catatgtatactgtaggtttagaaagtgatacaagagcttattttacaggagttacaatc
ttaatatccttaccaactggtacaaaaatctttaattggctttttacatatctctcaaat
ccaccattattacaccttagaattacttctgtcttcctctcacatctctttttattaatg
tttacgataggtgggtcaacaggaataattcttggaaatggtgcagtggatctaggatta
catgatacatattatgttgtagcacattttcattttgttctttctttaggagctataatt
gctatcttctctggaataatcttgaatggagaaaagattgttgctactaagaatttatta
ctttcatcctcatgtacactctctctttatcatttacatttaatatttattggtattctt
cttaccttttccccaatgcatttcttaggatttaatgttatgccaagaagaatcccatcc
tttccagattcttttcattcctggaattccctgtcatctattggatcaggaataactttc
ctatctttttctatg
>KrC1_cox1
ttaaatctcttaaccattaatttcctatttcattcactagttaaaaattgtaatcataaa
ggcttaggaatctattatttattatctggattcatctttggaatctccggtacattaata
tcagtccttataagaatagaattatattcttcaggaaataggattatatctccagaaaac
cagaacttctataatataagcattacattgcatggctttcttatgattttctttttagta
atgcctggcttgtttggaggatttggaaattattttgtacctatctttcaagggtctcca
gaagtggtatatcctagagtcaataatttttctatcttaattcttttgctttcatatctt
ttcctaatcctttctttaatctcagaatttggaggtggtacagggtggacgctctaccca
ccattatccacttcttttatgactttatcaccttcaagtacaggaaatcttatatttgga
ttaataatctctggtatatcttcatgtcttacatctcttaacttttggacaacaattcat
tttctgagatcttattatctgatattatcttctatcccattatttccttgggctttcttg
attacagctttcatgcttttattaacattaccaatcttatctggtacacttcttttaata
ttgggtgatcttcattctaatacacttttctttgatccaatatttggaggagatcctata
ttctatcaacatttattttggttttttggacatccagaagtttacatattaataattcct
gcatttgggatcatttccataataatttctggtattttacagttaataatctttgctaac
caatcaatgatctttgccatgtcatctatttctcttcttggaggtcttgtttggggacat
catatgtatactgtaggtttagaaagtgatacaagagcttattttacaggagttacaatc
ttaatatccttaccaactggtacaaaaatctttaattggctttttacatatctctcaaat
ccaccattattacaccttagaattacttctgtcttcctctcacatctctttttattaatg
tttacgataggtgggtcaacaggaataattcttggaaatggtgcagtggatctaggatta
catgatacatattatgttgtagcacattttcattttgttctttctttaggagctataatt
gctatcttctctggaataatcttgaatggagaaaagattgttgctactaagaatttatta
ctttcatcctcatgtacactctctctttatcatttacatttaatatttattggtattctt
cttaccttttccccaatgcatttcttaggatttaatgttatgccaagaagaatcccatcc
tttccagattcttttcattcctggaattccctgtcatctattggatcaggaataactttc
ctatctttttctatg
>KrC10_cox1
ataaatctcttaaccattaatttcctatttcattcactagttaaaaattgtaatcataaa
ggcttaggaatctattatttattatctggattcatctttggaatctccggtacattaata
tcagtccttataagaatagaattatattcttcaggaaataggattatatctccagaaaac
cagaacttctataatataagcattacattgcatggctttcttatgattttctttttagta
atgcctggcttgtttggaggatttggaaattattttgtacctatctttcaagggtctcca
gaagtggtatatcctagagtcaataatttttctatcttaattcttttgctttcatatctt
ttcctaatcctttctttaatctcagaatttggaggtggtacagggtggacgctctaccca
ccattatccacttcttttatgactttatcaccttcaagtacaggaaatcttatatttgga
ttaataatctctggtatatcttcatgtcttacatctcttaacttttggacaacaattcat
tttctgagatcttattatctgatattatcttctatcccattatttccttgggctttcttg
attacagctttcatgcttttattaacattaccaatcttatctggtacacttcttttaata
ttgggtgatcttcattctaatacacttttctttgatccaatatttggaggagatcctata
ttctatcaacatttattttggttttttggacatccagaagtttacatattaataattcct

gcatttgggatcatttccataataatttctggtattttacagttaataatctttgctaac
caatcaatgatctttgccatgtcatctatttctcttcttggaggtcttgtttggggacat
catatgtatactgtaggtttagaaagtgatacaagagcttattttacaggagttacaatc
ttaatatccttaccaactggtacaaaaatctttaattggctttttacatatctctcaaat
ccaccattattacaccttagaattacttctgtcttcctctcacatctctttttattaatg
tttacgataggtgggtcaacaggaataattcttggaaatggtgcagtggatctaggatta
catgatacatattatgttgtagcacattttcattttgttctttctttaggagctataatt
gctatcttctctggaataatcttgaatggagaaaagattgttgctactaagaatttatta
ctttcatcctcatgtacactctctctttatcatttacatttaatatttattggtattctt
cttacctttccccaatgcatttcttaggatttaatgttatgccaagaagaatcccatcc
tttccagattcttttcattcctggaattccctgtcatctattggatcaggaataactttc
ctatctttttctatg
>KrC11_cox1
ataaatctcttaaccattaatttcctatttcattcactagttaaaaattgtaatcataaa
ggcttaggaatctattatttattatctggattcatctttggaatctccggtacattaata
tcagtccttataagaatagaattatattcttcaggaaataggattatatctccagaaaac
cagaacttctataatataagcattacattgcatggctttcttatgattttctttttagta
atgcctggcttgtttggaggatttggaaattattttgtacctatctttcaagggtctcca
gaagtggtatatcctagagtcaataatttttctatcttaattcttttgctttcatatctt
ttcctaatcctttctttaatctcagaatttggaggtggtacagggtggacgctctaccca
ccattatccacttcttttatgactttatcaccttcaagtacaggaaatcttatatttgga
ttaataatctctggtatatcttcatgtcttacatctcttaacttttggacaacaattcat
tttctgagatcttattatctgatattatcttctatcccattatttccttgggctttcttg
attacagctttcatgcttttattaacattaccaatcttatctggtacacttcttttaata
ttgggtgatcttcattctaatacacttttctttgatccaatatttggaggagatcctata
ttctatcaacatttattttggtttttggacatccagaagtttacatattaataattcct
gcatttgggatcatttccataataatttctggtattttacagttaataatctttgctaac
caatcaatgatctttgccatgtcatctatttctcttcttggaggtcttgtttggggacat
catatgtatactgtaggtttagaaagtgatacaagagcttattttacaggagttacaatc
ttaatatccttaccaactggtacaaaaatctttaattggctttttacatatctctcaaat
ccaccattattacaccttagaattacttctgtcttcctctcacatctctttttattaatg
tttacgataggtgggtcaacaggaataattcttggaaatggtgcagtggatctaggatta
catgatacatattatgttgtagcacattttcattttgttctttctttaggagctataatt
gctatcttctctggaataatcttgaatggagaaaagattgttgctactaagaatttatta
ctttcatcctcatgtacactctctctttatcatttacatttaatatttattggtattctt
cttacctttccccaatgcatttcttaggatttaatgttatgccaagaagaatcccatcc
tttccagattcttttcattcctggaattccctgtcatctattggatcaggaataactttc
ctatctttttctatg
>KrC12_cox1
ataaatctcttaaccattaatttcctatttcattcactagttaaaaattgtaatcataaa
ggcttaggaatctattatttattatctggattcatctttggaatctccggtacattaata
tcagtccttataagaatagaattatattcttcaggaaataggattatatctccagaaaac
cagaacttctataatataagcattacattgcatggctttcttatgattttctttttagta
atgcctggcttgtttggaggatttggaaattattttgtacctatctttcaagggtctcca
gaagtggtatatcctagagtcaataatttttctatcttaattcttttgctttcatatctt
ttcctaatcctttctttaatctcagaatttggaggtggtacagggtggacgctctaccca
ccattatccacttcttttatgactttatcaccttcaagtacaggaaatcttatatttgga
ttaataatctctggtatatcttcatgtcttacatctcttaacttttggacaacaattcat
tttctgagatcttattatctgatattatcttctatcccattatttccttgggctttcttg
attacagctttcatgcttttattaacattaccaatcttatctggtacacttcttttaata
ttgggtgatcttcattctaatacacttttctttgatccaatatttggaggagatcctata
ttctatcaacatttattttggtttttggacatccagaagtttacatattaataattcct
gcatttgggatcatttccataataatttctggtattttacagttaataatctttgctaac
caatcaatgatctttgccatgtcatctatttctcttcttggaggtcttgtttggggacat
catatgtatactgtaggtttagaaagtgatacaagagcttattttacaggagttacaatc
ttaatatccttaccaactggtacaaaaatctttaattggctttttacatatctctcaaat
ccaccattattacaccttagaattacttctgtcttcctctcacatctctttttattaatg

tttacgataggtgggtcaacaggaataattcttggaaatggtgcagtggatctaggatta
catgatacatattatgttgtagcacattttcattttgttctttctttaggagctataatt
gctatcttctctggaataatcttgaatggagaaaagattgttgctactaagaatttatta
ctttcatcctcatgtacactctctctttatcatttacatttaatatttattggtattctt
cttaccttttccccaatgcatttcttaggatttaatgttatgccaagaagaatcccatcc
tttccagattcttttcattcctggaattccctgtcatctattggatcaggaataactttc
ctatctttttctatg
>KrC13_cox1
ataaatctcttaaccattaatttcctatttcattcactagttaaaaattgtaatcataaa
ggcttaggaatctattatttattatctggattcatctttggaatctccggtacattaata
tcagtccttataagaatagaattatattcttcaggaaataggattatatctccagaaaac
cagaacttctataatataagcattacattgcatggctttcttatgattttctttttagta
atgcctggcttgtttggaggatttggaaattattttgtacctatctttcaagggtctcca
gaagtggtatatcctagagtcaataatttttctatcttaattcttttgctttcatatctt
ttcctaatcctttctttaatctcagaatttggaggtggtacagggtggacgctctaccca
ccattatccacttcttttatgactttatcaccttcaagtacaggaaatcttatatttgga
ttaataatctctggtatatcttcatgtcttacatctcttaacttttggacaacaattcat
tttctgagatcttattatctgatattatcttctatcccattatttccttgggctttcttg
attacagctttcatgcttttattaacattaccaatcttatctggtacacttcttttaata
ttgggtgatcttcattctaatacacttttctttgatccaatatttggaggagatcctata
ttctatcaacatttattttggtttttggacatccagaagtttacatattaataattcct
gcatttgggatcatttccataataatttctggtattttacagttaataatctttgctaac
caatcaatgatctttgccatgtcatctatttctcttcttggaggtcttgtttggggacat
catatgtatactgtaggtttagaaagtgatacaagagcttattttacaggagttacaatc
ttaatatccttaccaactggtacaaaaatctttaattggctttttacatatctctcaaat
ccaccattattacaccttagaattacttctgtcttcctctcacatctctttttattaatg
tttacgataggtgggtcaacaggaataattcttggaaatggtgcagtggatctaggatta
catgatacatattatgttgtagcacattttcattttgttctttctttaggagctataatt
gctatcttctctggaataatcttgaatggagaaaagattgttgctactaagaatttatta
ctttcatcctcatgtacactctctctttatcatttacatttaatatttattggtattctt
cttaccttttccccaatgcatttcttaggatttaatgttatgccaagaagaatcccatcc
tttccagattcttttcattcctggaattccctgtcatctattggatcaggaataactttc
ctatctttttctatg
>KrC15_cox1
ataaatctcttaaccattaatttcctatttcattcactagttaaaaattgtaatcataaa
ggcttaggaatctattatttattatctggattcatctttggaatctccggtacattaata
tcagtccttataagaatagaattatattcttcaggaaataggattatatctccagaaaac
cagaacttctataatataagcattacattgcatggctttcttatgattttctttttagta
atgcctggcttgtttggaggatttggaaattattttgtacctatctttcaagggtctcca
gaagtggtatatcctagagtcaataatttttctatcttaattcttttgctttcatatctt
ttcctaatcctttctttaatctcagaatttggaggtggtacagggtggacgctctaccca
ccattatccacttcttttatgactttatcaccttcaagtacaggaaatcttatatttgga
ttaataatctctggtatatcttcatgtcttacatctcttaacttttggacaacaattcat
tttctgagatcttattatctgatattatcttctatcccattatttccttgggctttcttg
attacagctttcatgcttttattaacattaccaatcttatctggtacacttcttttaata
ttgggtgatcttcattctaatacacttttctttgatccaatatttggaggagatcctata
ttctatcaacatttattttggtttttggacatccagaagtttacatattaataattcct
gcatttgggatcatttccataataatttctggtattttacagttaataatctttgctaac
caatcaatgatctttgccatgtcatctatttctcttcttggaggtcttgtttggggacat
catatgtatactgtaggtttagaaagtgatacaagagcttattttacaggagttacaatc
ttaatatccttaccaactggtacaaaaatctttaattggctttttacatatctctcaaat
ccaccattattacaccttagaattacttctgtcttcctctcacatctctttttattaatg
tttacgataggtgggtcaacaggaataattcttggaaatggtgcagtggatctaggatta
catgatacatattatgttgtagcacattttcattttgttctttctttaggagctataatt
gctatcttctctggaataatcttgaatggagaaaagattgttgctactaagaatttatta
ctttcatcctcatgtacactctctctttatcatttacatttaatatttattggtattctt
cttaccttttccccaatgcatttcttaggatttaatgttatgccaagaagaatcccatcc

tttccagattcttttcattcctggaattccctgtcatctattggatcaggaataactttc
ctatctttttctatg
>KrC2_cox1
ataaatctcttaaccattaatttcctatttcattcactagttaaaaattgtaatcataaa
ggcttaggaatctattatttattatctggattcatctttggaatctccggtacattaata
tcagtccttataagaatagaattatattcttcaggaaataggattatatctccagaaaac
cagaacttctataatataagcattacattgcatggctttcttatgattttctttttagta
atgcctggcttgtttggaggatttggaaattattttgtacctatctttcaagggtctcca
gaagtggtatatcctagagtcaataatttttctatcttaattcttttgctttcatatctt
ttcctaatcctttctttaatctcagaatttggaggtggtacagggtggacgctctaccca
ccattatccacttcttttatgactttatcaccttcaagtacaggaaatcttatatttgga
ttaataatctctggtatatcttcatgtcttacatctcttaacttttggacaacaattcat
tttctgagatcttattatctgatattatcttctatcccattatttccttgggctttcttg
attacagctttcatgcttttattaacattaccaatcttatctggtacacttcttttaata
ttgggtgatcttcattctaatacacttttctttgatccaatatttggaggagatcctata
ttctatcaacatttattttggtttttggacatccagaagtttacatattaataattcct
gcatttgggatcatttccataataatttctggtattttacagttaataatctttgctaac
caatcaatgatctttgccatgtcatctatttctcttcttggaggtcttgtttggggacat
catatgtatactgtaggtttagaaagtgatacaagagcttattttacaggagttacaatc
ttaatatccttaccaactggtacaaaaatctttaattggctttttacatatctctcaaat
ccaccattattacaccttagaattacttctgtcttcctctcacatctcttttttattaatg
tttacgataggtgggtcaacaggaataattcttggaaatggtgcagtggatctaggatta
catgatacatattatgttgtagcacattttcattttgttctttctttaggagctataatt
gctatcttctctggaataatcttgaatggagaaaagattgttgctactaagaatttatta
ctttcatcctcatgtacactctctctttatcatttacatttaatatttattggtattctt
cttaccttttccccaatgcatttcttaggatttaatgttatgccaagaagaatcccatcc
tttccagattcttttcattcctggaattccctgtcatctattggatcaggaataactttc
ctatctttttctatg
>KrC3_cox1
ataaatctcttaaccattaatttcctatttcattcactagttaaaaattgtaatcataaa
ggcttaggaatctattatttattatctggattcatctttggaatctccggtacattaata
tcagtccttataagaatagaattatattcttcaggaaataggattatatctccagaaaac
cagaacttctataatataagcattacattgcatggctttcttatgattttctttttagta
atgcctggcttgtttggaggatttggaaattattttgtacctatctttcaagggtctcca
gaagtggtatatcctagagtcaataatttttctatcttaattcttttgctttcatatctt
ttcctaatcctttctttaatctcagaatttggaggtggtacagggtggacgctctaccca
ccattatccacttcttttatgactttatcaccttcaagtacaggaaatcttatatttgga
ttaataatctctggtatatcttcatgtcttacatctcttaacttttggacaacaattcat
tttctgagatcttattatctgatattatcttctatcccattatttccttgggctttcttg
attacagctttcatgcttttattaacattaccaatcttatctggtacacttcttttaata
ttgggtgatcttcattctaatacacttttctttgatccaatatttggaggagatcctata
ttctatcaacatttattttggtttttggacatccagaagtttacatattaataattcct
gcatttgggatcatttccataataatttctggtattttacagttaataatctttgctaac
caatcaatgatctttgccatgtcatctatttctcttcttggaggtcttgtttggggacat
catatgtatactgtaggtttagaaagtgatacaagagcttattttacaggagttacaatc
ttaatatccttaccaactggtacaaaaatctttaattggctttttacatatctctcaaat
ccaccattattacaccttagaattacttctgtcttcctctcacatctcttttttattaatg
tttacgataggtgggtcaacaggaataattcttggaaatggtgcagtggatctaggatta
catgatacatattatgttgtagcacattttcattttgttctttctttaggagctataatt
gctatcttctctggaataatcttgaatggagaaaagattgttgctactaagaatttatta
ctttcatcctcatgtacactctctctttatcatttacatttaatatttattggtattctt
cttaccttttccccaatgcatttcttaggatttaatgttatgccaagaagaatcccatcc
tttccagattcttttcattcctggaattccctgtcatctattggatcaggaataactttc
ctatctttttctatg
>KrC4_cox1
ataaatctcttaaccattaatttcctatttcattcactagttaaaaattgtaatcataaa
ggcttaggaatctattatttattatctggattcatctttggaatctccggtacattaata

tcagtccttataagaatagaattatattcttcaggaaataggattatatctccagaaaac
cagaacttctataatataagcattacattgcatggctttcttatgattttctttttagta
atgcctggcttgtttggaggatttggaaattattttgtacctatctttcaagggtctcca
gaagtggtatatcctagagtcaataatttttctatcttaattcttttgctttcatatctt
ttcctaatcctttctttaatctcagaatttggaggtggtacagggtggacgctctaccca
ccattatccacttcttttatgactttatcaccttcaagtacaggaaatcttatatttgga
ttaataatctctggtatatcttcatgtcttacatctcttaacttttggacaacaattcat
tttctgagatcttattatctgatattatcttctatcccattatttccttgggctttcttg
attacagctttcatgcttttattaacattaccaatcttatctggtacacttcttttaata
ttgggtgatcttcattctaatacacttttctttgatccaatatttggaggagatcctata
ttctatcaacatttattttggtttttggacatccagaagtttacatattaataattcct
gcatttgggatcatttccataataatttctggtattttacagttaataatctttgctaac
caatcaatgatctttgccatgtcatctatttctcttcttggaggtcttgtttggggacat
catatgtatactgtaggtttagaaagtgatacaagagcttattttacaggagttacaatc
ttaatatccttaccaactggtacaaaaatctttaattggctttttacatatctctcaaat
ccaccattattacaccttagaattacttctgtcttcctctcacatctctttttattaatg
tttacgataggtgggtcaacaggaataattcttggaaatggtgcagtggatctaggatta
catgatacatattatgttgtagcacattttcattttgttctttctttaggagctataatt
gctatcttctctggaataatcttgaatggagaaaagattgttgctactaagaatttatta
ctttcatcctcatgtacactctctctttatcatttacatttaatatttattggtattctt
cttaccttttccccaatgcatttcttaggatttaatgttatgccaagaagaatcccatcc
tttccagattcttttcattcctggaattccctgtcatctattggatcaggaataactttc
ctatctttttctatg
>KrC5_cox1
ataaatctcttaaccattaatttcctatttcattcactagttaaaaattgtaatcataaa
ggcttaggaatctattatttattatctggattcatctttggaatctccggtacattaata
tcagtccttataagaatagaattatattcttcaggaaataggattatatctccagaaaac
cagaacttctataatataagcattacattgcatggctttcttatgattttctttttagta
atgcctggcttgtttggaggatttggaaattattttgtacctatctttcaagggtctcca
gaagtggtatatcctagagtcaataatttttctatcttaattcttttgctttcatatctt
ttcctaatcctttctttaatctcagaatttggaggtggtacagggtggacgctctaccca
ccattatccacttcttttatgactttatcaccttcaagtacaggaaatcttatatttgga
ttaataatctctggtatatcttcatgtcttacatctcttaacttttggacaacaattcat
tttctgagatcttattatctgatattatcttctatcccattatttccttgggctttcttg
attacagctttcatgcttttattaacattaccaatcttatctggtacacttcttttaata
ttgggtgatcttcattctaatacacttttctttgatccaatatttggaggagatcctata
ttctatcaacatttattttggtttttggacatccagaagtttacatattaataattcct
gcatttgggatcatttccataataatttctggtattttacagttaataatctttgctaac
caatcaatgatctttgccatgtcatctatttctcttcttggaggtcttgtttggggacat
catatgtatactgtaggtttagaaagtgatacaagagcttattttacaggagttacaatc
ttaatatccttaccaactggtacaaaaatctttaattggctttttacatatctctcaaat
ccaccattattacaccttagaattacttctgtcttcctctcacatctctttttattaatg
tttacgataggtgggtcaacaggaataattcttggaaatggtgcagtggatctaggatta
catgatacatattatgttgtagcacattttcattttgttctttctttaggagctataatt
gctatcttctctggaataatcttgaatggagaaaagattgttgctactaagaatttatta
ctttcatcctcatgtacactctctctttatcatttacatttaatatttattggtattctt
cttaccttttccccaatgcatttcttaggatttaatgttatgccaagaagaatcccatcc
tttccagattcttttcattcctggaattccctgtcatctattggatcaggaataactttc
ctatctttttctatg
>KrC6_cox1
ataaatctcttaaccattaatttcctatttcattcactagttaaaaattgtaatcataaa
ggcttaggaatctattatttattatctggattcatctttggaatctccggtacattaata
tcagtccttataagaatagaattatattcttcaggaaataggattatatctccagaaaac
cagaacttctataatataagcattacattgcatggctttcttatgattttctttttagta
atgcctggcttgtttggaggatttggaaattattttgtacctatctttcaagggtctcca
gaagtggtatatcctagagtcaataatttttctatcttaattcttttgctttcatatctt
ttcctaatcctttctttaatctcagaatttggaggtggtacagggtggacgctctaccca

ccattatccacttcttttatgactttatcaccttcaagtacaggaaatcttatatttgga
ttaataatctctggtatatcttcatgtcttacatctcttaacttttggacaacaattcat
tttctgagatcttattatctgatattatcttctatcccattatttccttgggctttcttg
attacagctttcatgcttttattaacattaccaatcttatctggtacacttcttttaata
ttgggtgatcttcattctaatacacttttctttgatccaatatttggaggagatcctata
ttctatcaacatttattttggtttttggacatccagaagtttacatattaataattcct
gcatttgggatcatttccataataatttctggtattttacagttaataatctttgctaac
caatcaatgatctttgccatgtcatctatttctcttcttggaggtcttgtttggggacat
catatgtatactgtaggtttagaaagtgatacaagagcttattttacaggagttacaatc
ttaatatccttaccaactggtacaaaaatctttaattggctttttacatatctctcaaat
ccaccattattacaccttagaattacttctgtcttcctctcacatctcttttattaatg
tttacgataggtgggtcaacaggaataattcttggaaatggtgcagtggatctaggatta
catgatacatattatgttgtagcacattttcattttgttctttctttaggagctataatt
gctatcttctctggaataatcttgaatggagaaaagattgttgctactaagaatttatta
ctttcatcctcatgtacactctctctttatcatttacatttaatatttattggtattctt
cttaccttttccccaatgcatttcttaggatttaatgttatgccaagaagaatcccatcc
tttccagattcttttcattcctggaattccctgtcatctattggatcaggaataactttc
ctatctttttctatg
>KrC7_cox1
ataaatctcttaaccattaatttcctatttcattcactagttaaaaattgtaatcataaa
ggcttaggaatctattatttattatctggattcatctttggaatctccggtacattaata
tcagtccttataagaatagaattatattcttcaggaaataggattatatctccagaaaac
cagaacttctataatataagcattacattgcatggctttcttatgattttctttttagta
atgcctggcttgtttggaggatttggaaattattttgtacctatctttcaagggtctcca
gaagtggtatatcctagagtcaataatttttctatcttaattcttttgctttcatatctt
ttcctaatcctttctttaatctcagaatttggaggtggtacagggtggacgctctaccca
ccattatccacttcttttatgactttatcaccttcaagtacaggaaatcttatatttgga
ttaataatctctggtatatcttcatgtcttacatctcttaacttttggacaacaattcat
tttctgagatcttattatctgatattatcttctatcccattatttccttgggctttcttg
attacagctttcatgcttttattaacattaccaatcttatctggtacacttcttttaata
ttgggtgatcttcattctaatacacttttctttgatccaatatttggaggagatcctata
ttctatcaacatttattttggtttttggacatccagaagtttacatattaataattcct
gcatttgggatcatttccataataatttctggtattttacagttaataatctttgctaac
caatcaatgatctttgccatgtcatctatttctcttcttggaggtcttgtttggggacat
catatgtatactgtaggtttagaaagtgatacaagagcttattttacaggagttacaatc
ttaatatccttaccaactggtacaaaaatctttaattggctttttacatatctctcaaat
ccaccattattacaccttagaattacttctgtcttcctctcacatctcttttattaatg
tttacgataggtgggtcaacaggaataattcttggaaatggtgcagtggatctaggatta
catgatacatattatgttgtagcacattttcattttgttctttctttaggagctataatt
gctatcttctctggaataatcttgaatggagaaaagattgttgctactaagaatttatta
ctttcatcctcatgtacactctctctttatcatttacatttaatatttattggtattctt
cttaccttttccccaatgcatttcttaggatttaatgttatgccaagaagaatcccatcc
tttccagattcttttcattcctggaattccctgtcatctattggatcaggaataactttc
ctatctttttctatg
>KrC8_cox1
ataaatctcttaaccattaatttcctatttcattcactagttaaaaattgtaatcataaa
ggcttaggaatctattatttattatctggattcatctttggaatctccggtacattaata
tcagtccttataagaatagaattatattcttcaggaaataggattatatctccagaaaac
cagaacttctataatataagcattacattgcatggctttcttatgattttctttttagta
atgcctggcttgtttggaggatttggaaattattttgtacctatctttcaagggtctcca
gaagtggtatatcctagagtcaataatttttctatcttaattcttttgctttcatatctt
ttcctaatcctttctttaatctcagaatttggaggtggtacagggtggacgctctaccca
ccattatccacttcttttatgactttatcaccttcaagtacaggaaatcttatatttgga
ttaataatctctggtatatcttcatgtcttacatctcttaacttttggacaacaattcat
tttctgagatcttattatctgatattatcttctatcccattatttccttgggctttcttg
attacagctttcatgcttttattaacattaccaatcttatctggtacacttcttttaata
ttgggtgatcttcattctaatacacttttctttgatccaatatttggaggagatcctata

ttctatcaacatttattttggttttttggacatccagaagtttacatattaataattcct
gcatttgggatcatttccataataatttctggtattttacagttaataatctttgctaac
caatcaatgatctttgccatgtcatctatttctcttcttggaggtcttgtttggggacat
catatgtatactgtaggtttagaaagtgatacaagagcttattttacaggagttacaatc
ttaatatccttaccaactggtacaaaaatctttaattggctttttacatatctctcaaat
ccaccattattacaccttagaattacttctgtcttcctctcacatctctttttattaatg
tttacgataggtgggtcaacaggaataattcttggaaatggtgcagtggatctaggatta
catgatacatattatgttgtagcacattttcattttgttctttctttaggagctataatt
gctatcttctctggaataatcttgaatggagaaaagattgttgctactaagaatttatta
ctttcatcctcatgtacactctctctttatcatttacatttaatatttattggtattctt
cttaccttttccccaatgcatttcttaggatttaatgttatgccaagaagaatcccatcc
tttccagattcttttcattcctggaattccctgtcatctattggatcaggaataactttc
ctatctttttctatg
>KrC9_cox1
ataaatctcttaaccattaatttcctatttcattcactagttaaaaattgtaatcataaa
ggcttaggaatctattatttattatctggattcatctttggaatctccggtacattaata
tcagtccttataagaatagaattatattcttcaggaaataggattatatctccagaaaac
cagaacttctataatataagcattacattgcatggctttcttatgattttctttttagta
atgcctggcttgtttggaggatttggaaattattttgtacctatctttcaagggtctcca
gaagtggtatatcctagagtcaataatttttctatcttaattcttttgctttcatatctt
ttcctaatcctttctttaatctcagaatttggaggtggtacagggtggacgctctaccca
ccattatccacttcttttatgactttatcaccttcaagtacaggaaatcttatatttgga
ttaataatctctggtatatcttcatgtcttacatctcttaacttttggacaacaattcat
tttctgagatcttattatctgatattatcttctatcccattatttccttgggctttcttg
attacagctttcatgcttttattaacattaccaatcttatctggtacacttcttttaata
ttgggtgatcttcattctaatacacttttctttgatccaatatttggaggagatcctata
ttctatcaacatttattttggttttttggacatccagaagtttacatattaataattcct
gcatttgggatcatttccataataatttctggtattttacagttaataatctttgctaac
caatcaatgatctttgccatgtcatctatttctcttcttggaggtcttgtttggggacat
catatgtatactgtaggtttagaaagtgatacaagagcttattttacaggagttacaatc
ttaatatccttaccaactggtacaaaaatctttaattggctttttacatatctctcaaat
ccaccattattacaccttagaattacttctgtcttcctctcacatctctttttattaatg
tttacgataggtgggtcaacaggaataattcttggaaatggtgcagtggatctaggatta
catgatacatattatgttgtagcacattttcattttgttctttctttaggagctataatt
gctatcttctctggaataatcttgaatggagaaaagattgttgctactaagaatttatta
ctttcatcctcatgtacactctctctttatcatttacatttaatatttattggtattctt
cttaccttttccccaatgcatttcttaggatttaatgttatgccaagaagaatcccatcc
tttccagattcttttcattcctggaattccctgtcatctattggatcaggaataactttc
ctatctttttctatg
>KrD1_cox1
ttaaatctcttaaccattaatttcctatttcattcactagttaaaaattgtaatcataaa
ggcttaggaatctattatttattatctggattcatctttggaatctccggtacattaata
tcagtccttataagaatagaattatattcttcaggaaataggattatatctccagaaaac
cagaacttctataatataagcattacattgcatggctttcttatgattttctttttagta
atgcctggcttgtttggaggatttggaaattattttgtacctatctttcaagggtctcca
gaagtggtatatcctagagtcaataatttttctatcttaattcttttgctttcatatctt
ttcctaatcctttctttaatctcagaatttggaggtggtacagggtggacgctctaccca
ccattatccacttcttttatgactttatcaccttcaagtacaggaaatcttatatttgga
ttaataatctctggtatatcttcatgtcttacatctcttaacttttggacaacaattcat
tttctgagatcttattatctgatattatcttctatcccattatttccttgggctttcttg
attacagctttcatgcttttattaacattaccaatcttatctggtacacttcttttaata
ttgggtgatcttcattctaatacacttttctttgatccaatatttggaggagatcctata
ttctatcaacatttattttggttttttggacatccagaagtttacatattaataattcct
gcatttgggatcatttccataataatttctggtattttacagttaataatctttgctaac
caatcaatgatctttgccatgtcatctatttctcttcttggaggtcttgtttggggacat
catatgtatactgtaggtttagaaagtgatacaagagcttattttacaggagttacaatc
ttaatatccttaccaactggtacaaaaatctttaattggctttttacatatctctcaaat

ccaccattattacaccttagaattacttctgtcttcctctcacatctctttttattaatg
tttacgataggtgggtcaacaggaataattcttggaaatggtgcagtggatctaggatta
catgatacatattatgttgtagcacattttcattttgttctttctttaggagctataatt
gctatcttctctggaataatcttgaatggagaaaagattgttgctactaagaatttatta
ctttcatcctcatgtacactctctctttatcatttacatttaatatttattggtattctt
cttaccttttccccaatgcatttcttaggatttaatgttatgccaagaagaatcccatcc
tttccagattcttttcattcctggaattccctgtcatctattggatcaggaataactttc
ctatctttttctatg
>KrD11_cox1
ataaatctcttaaccattaatttcctatttcattcactagttaaaaattgtaatcataaa
ggcttaggaatctattatttattatctggattcatctttggaatctccggtacattaata
tcagtccttataagaatagaattatattcttcaggaaataggattatatctccagaaaac
cagaacttctataatataagcattacattgcatggctttcttatgattttctttttagta
atgcctggcttgtttggaggatttggaaattattttgtacctatctttcaagggtctcca
gaagtggtatatcctagagtcaataatttttctatcttaattcttttgctttcatatctt
ttcctaatcctttctttaatctcagaatttggaggtggtacagggtggacgctctaccca
ccattatccacttcttttatgactttatcaccttcaagtacaggaaatcttatatttgga
ttaataatctctggtatatcttcatgtcttacatctcttaacttttggacaacaattcat
tttctgagatcttattatctgatattatcttctatcccattatttccttgggctttcttg
attacagctttcatgcttttattaacattaccaatcttatctggtacacttcttttaata
ttgggtgatcttcattctaatacacttttctttgatccaatatttggaggagatcctata
ttctatcaacatttattttggtttttggacatccagaagtttacatattaataattcct
gcatttgggatcatttccataataatttctggtattttacagttaataatctttgctaac
caatcaatgatctttgccatgtcatctatttctcttcttggaggtcttgtttggggacat
catatgtatactgtaggtttagaaagtgatacaagagcttattttacaggagttacaatc
ttaatatccttaccaactggtacaaaaatctttaattggctttttacatatctctcaaat
ccaccattattacaccttagaattacttctgtcttcctctcacatctctttttattaatg
tttacgataggtgggtcaacaggaataattcttggaaatggtgcagtggatctaggatta
catgatacatattatgttgtagcacattttcattttgttctttctttaggagctataatt
gctatcttctctggaataatcttgaatggagaaaagattgttgctactaagaatttatta
ctttcatcctcatgtacactctctctttatcatttacatttaatatttattggtattctt
cttaccttttccccaatgcatttcttaggatttaatgttatgccaagaagaatcccatcc
tttccagattcttttcattcctggaattccctgtcatctattggatcaggaataactttc
ctatctttttctatg
>KrD13_cox1
ataaatctcttaaccattaatttcctatttcattcactagttaaaaattgtaatcataaa
ggcttaggaatctattatttattatctggattcatctttggaatctccggtacattaata
tcagtccttataagaatagaattatattcttcaggaaataggattatatctccagaaaac
cagaacttctataatataagcattacattgcatggctttcttatgattttctttttagta
atgcctggcttgtttggaggatttggaaattattttgtacctatctttcaagggtctcca
gaagtggtatatcctagagtcaataatttttctatcttaattcttttgctttcatatctt
ttcctaatcctttctttaatctcagaatttggaggtggtacagggtggacgctctaccca
ccattatccacttcttttatgactttatcaccttcaagtacaggaaatcttatatttgga
ttaataatctctggtatatcttcatgtcttacatctcttaacttttggacaacaattcat
tttctgagatcttattatctgatattatcttctatcccattatttccttgggctttcttg
attacagctttcatgcttttattaacattaccaatcttatctggtacacttcttttaata
ttgggtgatcttcattctaatacacttttctttgatccaatatttggaggagatcctata
ttctatcaacatttattttggtttttggacatccagaagtttacatattaataattcct
gcatttgggatcatttccataataatttctggtattttacagttaataatctttgctaac
caatcaatgatctttgccatgtcatctatttctcttcttggaggtcttgtttggggacat
catatgtatactgtaggtttagaaagtgatacaagagcttattttacaggagttacaatc
ttaatatccttaccaactggtacaaaaatctttaattggctttttacatatctctcaaat
ccaccattattacaccttagaattacttctgtcttcctctcacatctctttttattaatg
tttacgataggtgggtcaacaggaataattcttggaaatggtgcagtggatctaggatta
catgatacatattatgttgtagcacattttcattttgttctttctttaggagctataatt
gctatcttctctggaataatcttgaatggagaaaagattgttgctactaagaatttatta
ctttcatcctcatgtacactctctctttatcatttacatttaatatttattggtattctt

cttaccttttccccaatgcatttcttaggatttaatgttatgccaagaagaatcccatcc
tttccagattcttttcattcctggaattccctgtcatctattggatcaggaataactttc
ctatctttttctatg
>KrD3_cox1
ataaatctcttaaccattaatttcctatttcattcactagttaaaaattgtaatcataaa
ggcttaggaatctattatttattatctggattcatctttggaatctccggtacattaata
tcagtccttataagaatagaattatattcttcaggaaataggattatatctccagaaaac
cagaacttctataatataagcattacattgcatggctttcttatgattttctttttagta
atgcctggcttgtttggaggatttggaaattattttgtacctatctttcaagggtctcca
gaagtggtatatcctagagtcaataatttttctatcttaattcttttgctttcatatctt
ttcctaatccctttctttaatctcagaatttggaggtggtacagggtggacgctctaccca
ccattatccacttcttttatgactttatcaccttcaagtacaggaaatcttatatttgga
ttaataatctctggtatatcttcatgtcttacatctcttaacttttggacaacaattcat
tttctgagatcttattatctgatattatcttctatcccattatttccttgggctttcttg
attacagctttcatgcttttattaacattaccaatcttatctggtacacttcttttaata
ttgggtgatcttcattctaatacacttttctttgatccaatatttggaggagatcctata
ttctatcaacatttattttggtttttggacatccagaagtttacatattaataattcct
gcatttgggatcatttccataataatttctggtattttacagttaataatctttgctaac
caatcaatgatctttgccatgtcatctatttctcttcttggaggtcttgtttggggacat
catatgtatactgtaggtttagaaagtgatacaagagcttattttacaggagttacaatc
ttaatatccttaccaactggtacaaaaaatctttaattggctttttacatatctctcaaat
ccaccattattacaccttagaattacttctgtcttcctctcacatctctttttattaatg
tttacgataggtgggtcaacaggaataattcttggaaatggtgcagtggatctaggatta
catgatacatattatgttgtagcacattttcattttgttctttctttaggagctataatt
gctatcttctctggaataatcttgaatggagaaaagattgttgctactaagaatttatta
ctttcatcctcatgtacactctctctttatcatttacatttaatatttattggtattctt
cttaccttttccccaatgcatttcttaggatttaatgttatgccaagaagaatcccatcc
tttccagattcttttcattcctggaattccctgtcatctattggatcaggaataactttc
ctatctttttctatg
>KrD8_cox1
ataaatctcttaaccattaatttcctatttcattcactagttaaaaattgtaatcataaa
ggcttaggaatctattatttattatctggattcatctttggaatctccggtacattaata
tcagtccttataagaatagaattatattcttcaggaaataggattatatctccagaaaac
cagaacttctataatataagcattacattgcatggctttcttatgattttctttttagta
atgcctggcttgtttggaggatttggaaattattttgtacctatctttcaagggtctcca
gaagtggtatatcctagagtcaataatttttctatcttaattcttttgctttcatatctt
ttcctaatccctttctttaatctcagaatttggaggtggtacagggtggacgctctaccca
ccattatccacttcttttatgactttatcaccttcaagtgtaggaaatcttatatttgga
ttaataatctctggtatatcttcatgtcttacatctcttaacttttggacaacaattcat
tttctgagatcttattatctgatattatcttctatcccattatttccttgggctttcttg
attacagctttcatgcttttattaacattaccaatcttatctggtacacttcttttaata
ttgggtgatcttcattctaatacacttttctttgatccaatatttggaggagatcctata
ttctatcaacatttattttggtttttggacatccagaagtttacatattaataattcct
gcatttgggatcatttccataataatttctggtattttacagttaataatctttgctaac
caatcaatgatctttgccatgtcatctatttctcttcttggaggtcttgtttggggacat
catatgtatactgtaggtttagaaagtgatacaagagcttattttacaggagttacaatc
ttaatatccttaccaactggtacaaaaaatctttaattggctttttacatatctctcaaat
ccaccattattacaccttagaattacttctgtcttcctctcacatctctttttattaatg
tttacgataggtgggtcaacaggaataattcttggaaatggtgcagtggatctaggatta
catgatacatattatgttgtagcacattttcattttgttctttctttaggagctataatt
gctatcttctctggaataatcttgaatggagaaaagattgttgctactaagaatttatta
ctttcatcctcatgtacactctctctttatcatttacatttaatatttattggtattctt
cttaccttttccccaatgcatttcttaggatttaatgttatgccaagaagaatcccatcc
tttccagattcttttcattcctggaattccctgtcatctattggatcaggaataactttc
ctatctttttctatg
>KrD9_cox1
ataaatctcttaaccattaatttcctatttcattcactagttaaaaattgtaatcataaa

ggcttaggaatctattatttattatctggattcatctttggaatctccggtacattaata
tcagtccttataagaatagaattatattcttcaggaaataggattatatctccagaaaac
cagaacttctataatataagcattacattgcatggctttcttatgattttctttttagta
atgcctggcttgtttggaggatttggaaattattttgtacctatctttcaagggtctcca
gaagtggtatatcctagagtcaataatttttctatcttaattcttttgctttcatatctt
ttcctaatcctttctttaatctcagaatttggaggtggtacagggtggacgctctaccca
ccattatccacttcttttatgactttatcaccttcaagtacaggaaatcttatatttgga
ttaataatctctggtatatcttcatgtcttacatctcttaacttttggacaacaattcat
tttctgagatcttattatctgatattatcttctgtcccattatttccttgggctttcttg
attacagctttcatgcttttattaacattaccagtcttatctggtacacttcttttagta
ttgggtgatcttcattcaaatacacttttctttgatccaatatttggaggagatcctata
ttctatcaacatttattttggtttttggacatccagaagtttacatattaataattcct
gcatttgggatcatttccatagtaatttctggggtttcacagttaataatctttgctaac
caatcaatgatctttgccatgtcatctatttctcttcttggaggtcttgtttggggacat
catatgtatactgtaggtttagaaagtgatacaagagcttattttacaggagttacaatc
ttaatatccttaccaactggtacaaaaatctttaattggctttttacatatctctcaaat
ccaccattattacaccttagaattacttctgtcttcctctcacatctctttttattaatg
tttacgataggtgggtcaacaggaataattcttggaaatggtgcagtggatctaggatta
catgatacatattatgttgtagcacattttcattttgttctttctttaggagctataatt
gctatcttctctggaataatcttgaatggagaaaagattgttgctactaagaatttatta
ctttcatcctcatgtacactctctctttatcatttacatttaatatttattggtattctt
cttaccttttccccaatgcatttcttaggatttaatgttatgccaagaagaatcccatcc
tttccagattcttttcattcctggaattccctgtcatctattggatcaggaataactttc
ctatctttttctatg
>KrE1_cox1
ataaatctcttaaccattaatttcctatttcattcactagttaaaaattgtaatcataaa
ggcttaggaatctattatttattatctggattcatctttggaatctccggtacattaata
tcagtccttataagaatagaattatattcttcaggaaataggattatatctccagaaaac
cagaacttctataatataagcattacattgcatggctttcttatgattttctttttagta
atgcctggcttgtttggaggatttggaaattattttgtacctatctttcaagggtctcca
gaagtggtatatcctagagtcaataatttttctatcttaattcttttgctttcatatctt
ttcctaatcctttctttaatctcagaatttggaggtggtacagggtggacgctctaccca
ccattatccacttcttttatgactttatcaccttcaagtacaggaaatcttatatttgga
ttaataatctctggtatatcttcatgtcttacatctcttaacttttggacaacaattcat
tttctgagatcttattatctgatattatcttctatcccattatttccttgggctttcttg
attacagctttcatgcttttattaacattaccaatcttatctggtacacttcttttaata
ttgggtgatcttcattctaatacacttttctttgatccaatatttggaggagatcctata
ttctatcaacatttattttggtttttggacatccagaagtttacatattaataattcct
gcatttgggatcatttccataataatttctggtattttacagttaataatctttgctaac
caatcaatgatctttgccatgtcatctatttctcttcttggaggtcttgtttggggacat
catatgtatactgtaggtttagaaagtgatacaagagcttattttacaggagttacaatc
ttaatatccttaccaactggtacaaaaatctttaattggctttttacatatctctcaaat
ccaccattattacaccttagaattacttctgtcttcctctcacatctctttttattaatg
tttacgataggtgggtcaacaggaataattcttggaaatggtgcagtggatctaggatta
catgatacatattatgttgtagcacattttcattttgttctttctttaggagctataatt
gctatcttctctggaataatcttgaatggagaaaagattgttgctactaagaatttatta
ctttcatcctcatgtacactctctctttatcatttacatttaatatttattggtattctt
cttaccttttccccaatgcatttcttaggatttaatgttatgccaagaagaatcccatcc
tttccagattcttttcattcctggaattccctgtcatctattggatcaggaataactttc
ctatctttttctatg
>KrE10_cox1
ataaatctcttaaccattaatttcctatttcattcactagttaaaaattgtaatcataaa
ggcttaggaatctattatttattatctggattcatctttggaatctccggtacattaata
tcagtccttataagaatagaattatattcttcaggaaataggattatatctccagaaaac
cagaacttctataatataagcattacattgcatggctttcttatgattttctttttagta
atgcctggcttgtttggaggatttggaaattattttgtacctatctttcaagggtctcca
gaagtggtatatcctagagtcaataatttttctatcttaattcttttgctttcatatctt

ttcctaatcctttctttaatctcagaatttggaggtggtacagggtggacgctctaccca
ccattatccacttcttttatgactttatcaccttcaagtacaggaaatcttatatttgga
ttaataatctctggtatatcttcatgtcttacatctcttaacttttggacaacaattcat
tttctgagatcttattatctgatattatcttctatcccattatttccttgggctttcttg
attacagctttcatgcttttattaacattaccaatcttatctggtacacttcttttaata
ttgggtgatcttcattctaatacacttttctttgatccaatatttggaggagatcctata
ttctatcaacatttattttggtttttggacatccagaagtttacatattaataattcct
gcatttgggatcatttccataataatttctggtattttacagttaataatctttgctaac
caatcaatgatctttgccatgtcatctatttctcttcttggaggtcttgtttggggacat
catatgtatactgtaggtttagaaagtgatacaagagcttattttacaggagttacaatc
ttaatatccttaccaactggtacaaaaatctttaattggctttttacatatctctcaaat
ccaccattattacaccttagaattacttctgtcttcctctcacatctcttttattaatg
tttacgataggtgggtcaacaggaataattcttggaaatggtgcagtggatctaggatta
catgatacatattatgttgtagcacattttcattttgttctttctttaggagctataatt
gctatcttctctggaataatcttgaatggagaaaagattgttgctactaagaatttatta
ctttcatcctcatgtacactctctctttatcatttacatttaatatttattggtattctt
cttaccttttccccaatgcatttcttaggatttaatgttatgccaagaagaatcccatcc
tttccagattcttttcattcctggaattccctgtcatctattggatcaggaataactttc
ctatctttttctatg
>KrE11_cox1
ataaatctcttaaccattaatttcctatttcattcactagttaaaaattgtaatcataaa
ggcttaggaatctattatttattatctggattcatctttggaatctccggtacattaata
tcagtccttataagaatagaattatattcttcaggaaataggattatatctccagaaaac
cagaacttctataatataagcattacattgcatggctttcttatgattttctttttagta
atgcctggcttgtttggaggatttggaaattattttgtacctatctttcaagggtctcca
gaagtggtatatcctagagtcaataatttttctatcttaattcttttgctttcatatctt
ttcctaatcctttctttaatctcagaatttggaggtggtacagggtggacgctctaccca
ccattatccacttcttttatgactttatcaccttcaagtacaggaaatcttatatttgga
ttaataatctctggtatatcttcatgtcttacatctcttaacttttggacaacaattcat
tttctgagatcttattatctgatattatcttctatcccattatttccttgggctttcttg
attacagctttcatgcttttattaacattaccaatcttatctggtacacttcttttaata
ttgggtgatcttcattctaatacacttttctttgatccaatatttggaggagatcctata
ttctatcaacatttattttggtttttggacatccagaagtttacatattaataattcct
gcatttgggatcatttccataataatttctggtattttacagttaataatctttgctaac
caatcaatgatctttgccatgtcatctatttctcttcttggaggtcttgtttggggacat
catatgtatactgtaggtttagaaagtgatacaagagcttattttacaggagttacaatc
ttaatatccttaccaactggtacaaaaatctttaattggctttttacatatctctcaaat
ccaccattattacaccttagaattacttctgtcttcctctcacatctcttttattaatg
tttacgataggtgggtcaacaggaataattcttggaaatggtgcagtggatctaggatta
catgatacatattatgttgtagcacattttcattttgttctttctttaggagctataatt
gctatcttctctggaataatcttgaatggagaaaagattgttgctactaagaatttatta
ctttcatcctcatgtacactctctctttatcatttacatttaatatttattggtattctt
cttaccttttccccaatgcatttcttaggatttaatgttatgccaagaagaatcccatcc
tttccagattcttttcattcctggaattccctgtcatctattggatcaggaataactttc
ctatctttttctatg
>KrE12_cox1
ataaatctcttaaccattaatttcctatttcattcactagttaaaaattgtaatcataaa
ggcttaggaatctattatttattatctggattcatctttggaatctccggtacattaata
tcagtccttataagaatagaattatattcttcaggaaataggattatatctccagaaaac
cagaacttctataatataagcattacattgcatggctttcttatgattttctttttagta
atgcctggcttgtttggaggatttggaaattattttgtacctatctttcaagggtctcca
gaagtggtatatcctagagtcaataatttttctatcttaattcttttgctttcatatctt
ttcctaatcctttctttaatctcagaatttggaggtggtacagggtggacgctctaccca
ccattatccacttcttttatgactttatcaccttcaagtacaggaaatcttatatttgga
ttaataatctctggtatatcttcatgtcttacatctcttaacttttggacaacaattcat
tttctgagatcttattatctgatattatcttctatcccattatttccttgggctttcttg
attacagctttcatgcttttattaacattaccaatcttatctggtacacttcttttaata

ttgggtgatcttcattctaatacacttttctttgatccaatatttggaggagatcctata
ttctatcaacatttattttggttttttggacatccagaagtttacatattaataattcct
gcatttgggatcatttccataataatttctggtattttacagttaataatctttgctaac
caatcaatgatctttgccatgtcatctatttctcttcttggaggtcttgtttggggacat
catatgtatactgtaggtttagaaagtgatacaagagcttattttacaggagttacaatc
ttaatatccttaccaactggtacaaaaatctttaattggctttttacatatctctcaaat
ccaccattattacaccttagaattacttctgtcttcctctcacatctctttttattaatg
tttacgataggtgggtcaacaggaataattcttggaaatggtgcagtggatctaggatta
catgatacatattatgttgtagcacattttcattttgttctttctttaggagctataatt
gctatcttctctggaataatcttgaatggagaaaagattgttgctactaagaatttatta
ctttcatcctcatgtacactctctctttatcatttacatttaatatttattggtattctt
cttacctttccccaatgcatttcttaggatttaatgttatgccaagaagaatcccatcc
tttccagattcttttcattcctggaattccctgtcatctattggatcaggaataactttc
ctatctttttctatg
>KrE13_cox1
ataaatctcttaaccattaatttcctatttcattcactagttaaaaattgtaatcataaa
ggcttaggaatctattatttattatctggattcatctttggaatctccggtacattaata
tcagtccttataagaatagaattatattcttcaggaaataggattatatctccagaaaac
cagaacttctataatataagcattacattgcatggctttcttatgattttctttttagta
atgcctggcttgtttggaggatttggaaattattttgtacctatctttcaagggtctcca
gaagtggtatatcctagagtcaataatttttctatcttaattcttttgctttcatatctt
ttcctaatcctttctttaatctcagaatttggaggtggtacagggtggacgctctaccca
ccattatccacttcttttatgactttatcaccttcaagtacaggaaatcttatatttgga
ttaataatctctggtatatcttcatgtcttacatctcttaacttttggacaacaattcat
tttctgagatcttattatctgatattatcttctatcccattatttccttgggctttcttg
attacagctttcatgcttttattaacattaccaatcttatctggtacacttcttttaata
ttgggtgatcttcattctaatacacttttctttgatccaatatttggaggagatcctata
ttctatcaacatttattttggttttttggacatccagaagtttacatattaataattcct
gcatttgggatcatttccataataatttctggtattttacagttaataatctttgctaac
caatcaatgatctttgccatgtcatctatttctcttcttggaggtcttgtttggggacat
catatgtatactgtaggtttagaaagtgatacaagagcttattttacaggagttacaatc
ttaatatccttaccaactggtacaaaaatctttaattggctttttacatatctctcaaat
ccaccattattacaccttagaattacttctgtcttcctctcacatctctttttattaatg
tttacgataggtgggtcaacaggaataattcttggaaatggtgcagtggatctaggatta
catgatacatattatgttgtagcacattttcattttgttctttctttaggagctataatt
gctatcttctctggaataatcttgaatggagaaaagattgttgctactaagaatttatta
ctttcatcctcatgtacactctctctttatcatttacatttaatatttattggtattctt
cttacctttccccaatgcatttcttaggatttaatgttatgccaagaagaatcccatcc
tttccagattcttttcattcctggaattccctgtcatctattggatcaggaataactttc
ctatctttttctatg
>KrE14_cox1
ataaatctcttaaccattaatttcctatttcattcactagttaaaaattgtaatcataaa
ggcttaggaatctattatttattatctggattcatctttggaatctccggtacattaata
tcagtccttataagaatagaattatattcttcaggaaataggattatatctccagaaaac
cagaacttctataatataagcattacattgcatggctttcttatgattttctttttagta
atgcctggcttgtttggaggatttggaaattattttgtacctatctttcaagggtctcca
gaagtggtatatcctagagtcaataatttttctatcttaattcttttgctttcatatctt
ttcctaatcctttctttaatctcagaatttggaggtggtacagggtggacgctctaccca
ccattatccacttcttttatgactttatcaccttcaagtacaggaaatcttatatttgga
ttaataatctctggtatatcttcatgtcttacatctcttaacttttggacaacaattcat
tttctgagatcttattatctgatattatcttctatcccattatttccttgggctttcttg
attacagctttcatgcttttattaacattaccaatcttatctggtacacttcttttaata
ttgggtgatcttcattctaatacacttttctttgatccaatatttggaggagatcctata
ttctatcaacatttattttggttttttggacatccagaagtttacatattaataattcct
gcatttgggatcatttccataataatttctggtattttacagttaataatctttgctaac
caatcaatgatctttgccatgtcatctatttctcttcttggaggtcttgtttggggacat
catatgtatactgtaggtttagaaagtgatacaagagcttattttacaggagttacaatc

ttaatatccttaccaactggtacaaaaatctttaattggctttttacatatctctcaaat
ccaccattattacaccttagaattacttctgtcttcctctcacatctctttttattaatg
tttacgataggtgggtcaacaggaataattcttggaaatggtgcagtggatctaggatta
catgatacatattatgttgtagcacattttcattttgttctttctttaggagctataatt
gctatcttctctggaataatcttgaatggagaaaagattgttgctactaagaatttatta
ctttcatcctcatgtacactctctctttatcatttacatttaatatttattggtattctt
cttacctttccccaatgcatttcttaggatttaatgttatgccaagaagaatcccatcc
tttccagattcttttcattcctggaattccctgtcatctattggatcaggaataactttc
ctatctttttctatg
>KrE15_cox1
ataaatctcttaaccattaatttcctatttcattcactagttaaaaattgtaatcataaa
ggcttaggaatctattatttattatctggattcatctttggaatctccggtacattaata
tcagtccttataagaatagaattatattcttcaggaaataggattatatctccagaaaac
cagaacttctataatataagcattacattgcatggctttcttatgattttctttttagta
atgcctggcttgtttggaggatttggaaattattttgtacctatctttcaagggtctcca
gaagtggtatatcctagagtcaataatttttctatcttaattcttttgctttcatatctt
ttcctaatcctttctttaatctcagaatttggaggtggtacagggtggacgctctaccca
ccattatccacttcttttatgactttatcaccttcaagtacaggaaatcttatatttgga
ttaataatctctggtatatcttcatgtcttacatctcttaacttttggacaacaattcat
tttctgagatcttattatctgatattatcttctatcccattatttccttgggctttcttg
attacagctttcatgcttttattaacattaccaatcttatctggtacacttcttttaata
ttgggtgatcttcattctaatacacttttctttgatccaatatttggaggagatcctata
ttctatcaacatttattttggtttttggacatccagaagtttacatattaataattcct
gcatttgggatcatttccataataatttctggtattttacagttaataatctttgctaac
caatcaatgatctttgccatgtcatctatttctcttcttggaggtcttgtttggggacat
catatgtatactgtaggtttagaaagtgatacaagagcttattttacaggagttacaatc
ttaatatccttaccaactggtacaaaaatctttaattggctttttacatatctctcaaat
ccaccattattacaccttagaattacttctgtcttcctctcacatctctttttattaatg
tttacgataggtgggtcaacaggaataattcttggaaatggtgcagtggatctaggatta
catgatacatattatgttgtagcacattttcattttgttctttctttaggagctataatt
gctatcttctctggaataatcttgaatggagaaaagattgttgctactaagaatttatta
ctttcatcctcatgtacactctctctttatcatttacatttaatatttattggtattctt
cttacctttccccaatgcatttcttaggatttaatgttatgccaagaagaatcccatcc
tttccagattcttttcattcctggaattccctgtcatctattggatcaggaataactttc
ctatctttttctatg
>KrE2_cox1
ataaatctcttaaccattaatttcctatttcattcactagttaaaaattgtaatcataaa
ggcttaggaatctattatttattatctggattcatctttggaatctccggtacattaata
tcagtccttataagaatagaattatattcttcaggaaataggattatatctccagaaaac
cagaacttctataatataagcattacattgcatggctttcttatgattttctttttagta
atgcctggcttgtttggaggatttggaaattattttgtacctatctttcaagggtctcca
gaagtggtatatcctagagtcaataatttttctatcttaattcttttgctttcatatctt
ttcctaatcctttctttaatctcagaatttggaggtggtacagggtggacgctctaccca
ccattatccacttcttttatgactttatcaccttcaagtacaggaaatcttatatttgga
ttaataatctctggtatatcttcatgtcttacatctcttaacttttggacaacaattcat
tttctgagatcttattatctgatattatcttctatcccattatttccttgggctttcttg
attacagctttcatgcttttattaacattaccaatcttatctggtacacttcttttaata
ttgggtgatcttcattctaatacacttttctttgatccaatatttggaggagatcctata
ttctatcaacatttattttggtttttggacatccagaagtttacatattaataattcct
gcatttgggatcatttccataataatttctggtattttacagttaataatctttgctaac
caatcaatgatctttgccatgtcatctatttctcttcttggaggtcttgtttggggacat
catatgtatactgtaggtttagaaagtgatacaagagcttattttacaggagttacaatc
ttaatatccttaccaactggtacaaaaatctttaattggctttttacatatctctcaaat
ccaccattattacaccttagaattacttctgtcttcctctcacatctctttttattaatg
tttacgataggtgggtcaacaggaataattcttggaaatggtgcagtggatctaggatta
catgatacatattatgttgtagcacattttcattttgttctttctttaggagctataatt
gctatcttctctggaataatcttgaatggagaaaagattgttgctactaagaatttatta

ctttcatcctcatgtacactctctctttatcatttacatttaatatttattggtattctt
cttaccttttccccaatgcatttcttaggatttaatgttatgccaagaagaatcccatcc
tttccagattcttttcattcctggaattccctgtcatctattggatcaggaataactttc
ctatctttttctatg
>KrE3_cox1
ataaatctcttaaccattaatttcctatttcattcactagttaaaaattgtaatcataaa
ggcttaggaatctattatttattatctggattcatctttggaatctccggtacattaata
tcagtccttataagaatagaattatattcttcaggaaataggattatatctccagaaaac
cagaacttctataatataagcattacattgcatggctttcttatgattttctttttagta
atgcctggcttgtttggaggatttggaaattattttgtacctatctttcaagggtctcca
gaagtggtatatcctagagtcaataatttttctatcttaattcttttgctttcatatctt
ttcctaatcctttctttaatctcagaatttggaggtggtacagggtggacgctctaccca
ccattatccacttcttttatgactttatcaccttcaagtacaggaaatcttatatttgga
ttaataatctctggtatatcttcatgtcttacatctcttaacttttggacaacaattcat
tttctgagatcttattatctgatattatcttctatcccattatttccttgggctttcttg
attacagctttcatgcttttattaacattaccaatcttatctggtacacttcttttaata
ttgggtgatcttcattctaatacacttttctttgatccaatatttggaggagatcctata
ttctatcaacatttattttggtttttggacatccagaagtttacatattaataattcct
gcatttgggatcatttccataataatttctggtattttacagttaataatctttgctaac
caatcaatgatctttgccatgtcatctatttctcttcttggaggtcttgtttggggacat
catatgtatactgtaggtttagaaagtgatacaagagcttattttacaggagttacaatc
ttaatatccttaccaactggtacaaaaatctttaattggctttttacatatctctcaaat
ccaccattattacaccttagaattacttctgtcttcctctcacatctctttttattaatg
tttacgataggtgggtcaacaggaataattcttggaaatggtgcagtggatctaggatta
catgatacatattatgttgtagcacattttcattttgttctttctttaggagctataatt
gctatcttctctggaataatcttgaatggagaaaagattgttgctactaagaatttatta
ctttcatcctcatgtacactctctctttatcatttacatttaatatttattggtattctt
cttaccttttccccaatgcatttcttaggatttaatgttatgccaagaagaatcccatcc
tttccagattcttttcattcctggaattccctgtcatctattggatcaggaataactttc
ctatctttttctatg
>KrE4_cox1
ataaatctcttaaccattaatttcctatttcattcactagttaaaaattgtaatcataaa
ggcttaggaatctattatttattatctggattcatctttggaatctccggtacattaata
tcagtccttataagaatagaattatattcttcaggaaataggattatatctccagaaaac
cagaacttctataatataagcattacattgcatggctttcttatgattttctttttagta
atgcctggcttgtttggaggatttggaaattattttgtacctatctttcaagggtctcca
gaagtggtatatcctagagtcaataatttttctatcttaattcttttgctttcatatctt
ttcctaatcctttctttaatctcagaatttggaggtggtacagggtggacgctctaccca
ccattatccacttcttttatgactttatcaccttcaagtacaggaaatcttatatttgga
ttaataatctctggtatatcttcatgtcttacatctcttaacttttggacaacaattcat
tttctgagatcttattatctgatattatcttctatcccattatttccttgggctttcttg
attacagctttcatgcttttattaacattaccaatcttatctggtacacttcttttaata
ttgggtgatcttcattctaatacacttttctttgatccaatatttggaggagatcctata
ttctatcaacatttattttggtttttggacatccagaagtttacatattaataattcct
gcatttgggatcatttccataataatttctggtattttacagttaataatctttgctaac
caatcaatgatctttgccatgtcatctatttctcttcttggaggtcttgtttggggacat
catatgtatactgtaggtttagaaagtgatacaagagcttattttacaggagttacaatc
ttaatatccttaccaactggtacaaaaatctttaattggctttttacatatctctcaaat
ccaccattattacaccttagaattacttctgtcttcctctcacatctctttttattaatg
tttacgataggtgggtcaacaggaataattcttggaaatggtgcagtggatctaggatta
catgatacatattatgttgtagcacattttcattttgttctttctttaggagctataatt
gctatcttctctggaataatcttgaatggagaaaagattgttgctactaagaatttatta
ctttcatcctcatgtacactctctctttatcatttacatttaatatttattggtattctt
cttaccttttccccaatgcatttcttaggatttaatgttatgccaagaagaatcccatcc
tttccagattcttttcattcctggaattccctgtcatctattggatcaggaataactttc
ctatctttttctatg
>KrE5_cox1

ttaaatctcttaaccattaatttcctatttcattcactagttaaaaattgtaatcataaa
ggcttaggaatctattatttattatctggattcatctttggaatctccggtacattaata
tcagtccttataagaatagaattatattcttcaggaaataggattatatctccagaaaac
cagaacttctataatataagcattacattgcatggctttcttatgattttctttttagta
atgcctggcttgtttggaggatttggaaattattttgtacctatctttcaagggtctcca
gaagtggtatatcctagagtcaataatttttctatcttaattcttttgctttcatatctt
ttcctaatcctttctttaatctcagaatttggaggtggtacagggtggacgctctaccca
ccattatccacttcttttatgactttatcaccttcaagtacaggaaatcttatatttgga
ttaataatctctggtatatcttcatgtcttacatctcttaacttttggacaacaattcat
tttctgagatcttattatctgatattatcttctatcccattatttccttgggctttcttg
attacagctttcatgcttttattaacattaccaatcttatctggtacacttcttttaata
ttgggtgatcttcattctaatacacttttctttgatccaatatttggaggagatcctata
ttctatcaacatttattttggtttttggacatccagaagtttacatattaataattcct
gcatttgggatcatttccataataatttctggtattttacagttaataatctttgctaac
caatcaatgatctttgccatgtcatctatttctcttcttggaggtcttgtttggggacat
catatgtatactgtaggtttagaaagtgatacaagagcttattttacaggagttacaatc
ttaatatccttaccaactggtacaaaaatctttaattggctttttacatatctctcaaat
ccaccattattacaccttagaattacttctgtcttcctctcacatctctttttattaatg
tttacgataggtgggtcaacaggaataattcttggaaatggtgcagtggatctaggatta
catgatacatattatgttgtagcacattttcattttgttctttctttaggagctataatt
gctatcttctctggaataatcttgaatggagaaaagattgttgctactaagaatttatta
ctttcatcctcatgtacactctctctttatcatttacatttaatatttattggtattctt
cttacctttccccaatgcatttcttaggatttaatgttatgccaagaagaatcccatcc
tttccagattcttttcattcctggaattccctgtcatctattggatcaggaataactttc
ctatctttttctatg
>KrE6_cox1
ataaatctcttaaccattaatttcctatttcattcactagttaaaaattgtaatcataaa
ggcttaggaatctattatttattatctggattcatctttggaatctccggtacattaata
tcagtccttataagaatagaattatattcttcaggaaataggattatatctccagaaaac
cagaacttctataatataagcattacattgcatggctttcttatgattttctttttagta
atgcctggcttgtttggaggatttggaaattattttgtacctatctttcaagggtctcca
gaagtggtatatcctagagtcaataatttttctatcttaattcttttgctttcatatctt
ttcctaatcctttctttaatctcagaatttggaggtggtacagggtggacgctctaccca
ccattatccacttcttttatgactttatcaccttcaagtacaggaaatcttatatttgga
ttaataatctctggtatatcttcatgtcttacatctcttaacttttggacaacaattcat
tttctgagatcttattatctgatattatcttctatcccattatttccttgggctttcttg
attacagctttcatgcttttattaacattaccaatcttatctggtacacttcttttaata
ttgggtgatcttcattctaatacacttttctttgatccaatatttggaggagatcctata
ttctatcaacatttattttggtttttggacatccagaagtttacatattaataattcct
gcatttgggatcatttccataataatttctggtattttacagttaataatctttgctaac
caatcaatgatctttgccatgtcatctatttctcttcttggaggtcttgtttggggacat
catatgtatactgtaggtttagaaagtgatacaagagcttattttacaggagttacaatc
ttaatatccttaccaactggtacaaaaatctttaattggctttttacatatctctcaaat
ccaccattattacaccttagaattacttctgtcttcctctcacatctctttttattaatg
tttacgataggtgggtcaacaggaataattcttggaaatggtgcagtggatctaggatta
catgatacatattatgttgtagcacattttcattttgttctttctttaggagctataatt
gctatcttctctggaataatcttgaatggagaaaagattgttgctactaagaatttatta
ctttcatcctcatgtacactctctctttatcatttacatttaatatttattggtattctt
cttacctttccccaatgcatttcttaggatttaatgttatgccaagaagaatcccatcc
tttccagattcttttcattcctggaattccctgtcatctattggatcaggaataactttc
ctatctttttctatg
>KrE7_cox1
ataaatctcttaaccattaatttcctatttcattcactagttaaaaattgtaatcataaa
ggcttaggaatctattatttattatctggattcatctttggaatctccggtacattaata
tcagtccttataagaatagaattatattcttcaggaaataggattatatctccagaaaac
cagaacttctataatataagcattacattgcatggctttcttatgattttctttttagta
atgcctggcttgtttggaggatttggaaattattttgtacctatctttcaagggtctcca

gaagtggtatatcctagagtcaataatttttctatcttaattcttttgctttcatatctt
ttcctaatcctttctttaatctcagaatttggaggtggtacagggtggacgctctaccca
ccattatccacttcttttatgactttatcaccttcaagtacaggaaatcttatatttgga
ttaataatctctggtatatcttcatgtcttacatctcttaacttttggacaacaattcat
tttctgagatcttattatctgatattatcttctatcccattatttccttgggctttcttg
attacagctttcatgcttttattaacattaccaatcttatctggtacacttcttttaata
ttgggtgatcttcattctaatacacttttctttgatccaatatttggaggagatcctata
ttctatcaacatttattttggttttttggacatccagaagtttacatattaataattcct
gcatttgggatcatttccataataatttctggtattttacagttaataatctttgctaac
caatcaatgatctttgccatgtcatctatttctcttcttggaggtcttgtttggggacat
catatgtatactgtaggtttagaaagtgatacaagagcttattttacaggagttacaatc
ttaatatccttaccaactggtacaaaaatctttaattggctttttacatatctctcaaat
ccaccattattacaccttagaattacttctgtcttcctctcacatctctttttattaatg
tttacgataggtgggtcaacaggaataattcttggaaatggtgcagtggatctaggatta
catgatacatattatgttgtagcacattttcattttgttctttctttaggagctataatt
gctatcttctctggaataatcttgaatggagaaaagattgttgctactaagaatttatta
ctttcatcctcatgtacactctctctttatcatttacatttaatatttattggtattctt
cttaccttttccccaatgcatttcttaggatttaatgttatgccaagaagaatcccatcc
tttccagattcttttcattcctggaattccctgtcatctattggatcaggaataactttc
ctatctttttctatg
>KrE8_cox1
ataaatctcttaaccattaatttcctatttcattcactagttaaaaattgtaatcataaa
ggcttaggaatctattatttattatctggattcatctttggaatctccggtacattaata
tcagtccttataagaatagaattatattcttcaggaaataggattatatctccagaaaac
cagaacttctataatataagcattacattgcatggctttcttatgattttctttttagta
atgcctggcttgtttggaggatttggaaattattttgtacctatctttcaagggtctcca
gaagtggtatatcctagagtcaataatttttctatcttaattcttttgctttcatatctt
ttcctaatcctttctttaatctcagaatttggaggtggtacagggtggacgctctaccca
ccattatccacttcttttatgactttatcaccttcaagtacaggaaatcttatatttgga
ttaataatctctggtatatcttcatgtcttacatctcttaacttttggacaacaattcat
tttctgagatcttattatctgatattatcttctatcccattatttccttgggctttcttg
attacagctttcatgcttttattaacattaccaatcttatctggtacacttcttttaata
ttgggtgatcttcattctaatacacttttctttgatccaatatttggaggagatcctata
ttctatcaacatttattttggttttttggacatccagaagtttacatattaataattcct
gcatttgggatcatttccataataatttctggtattttacagttaataatctttgctaac
caatcaatgatctttgccatgtcatctatttctcttcttggaggtcttgtttggggacat
catatgtatactgtaggtttagaaagtgatacaagagcttattttacaggagttacaatc
ttaatatccttaccaactggtacaaaaatctttaattggctttttacatatctctcaaat
ccaccattattacaccttagaattacttctgtcttcctctcacatctctttttattaatg
tttacgataggtgggtcaacaggaataattcttggaaatggtgcagtggatctaggatta
catgatacatattatgttgtagcacattttcattttgttctttctttaggagctataatt
gctatcttctctggaataatcttgaatggagaaaagattgttgctactaagaatttatta
ctttcatcctcatgtacactctctctttatcatttacatttaatatttattggtattctt
cttaccttttccccaatgcatttcttaggatttaatgttatgccaagaagaatcccatcc
tttccagattcttttcattcctggaattccctgtcatctattggatcaggaataactttc
ctatctttttctatg
>KrE9_cox1
ataaatctcttaaccattaatttcctatttcattcactagttaaaaattgtaatcataaa
ggcttaggaatctattatttattatctggattcatctttggaatctccggtacattaata
tcagtccttataagaatagaattatattcttcaggaaataggattatatctccagaaaac
cagaacttctataatataagcattacattgcatggctttcttatgattttctttttagta
atgcctggcttgtttggaggatttggaaattattttgtacctatctttcaagggtctcca
gaagtggtatatcctagagtcaataatttttctatcttaattcttttgctttcatatctt
ttcctaatcctttctttaatctcagaatttggaggtggtacagggtggacgctctaccca
ccattatccacttcttttatgactttatcaccttcaagtacaggaaatcttatatttgga
ttaataatctctggtatatcttcatgtcttacatctcttaacttttggacaacaattcat
tttctgagatcttattatctgatattatcttctatcccattatttccttgggctttcttg

attacagctttcatgcttttattaacattaccaatcttatctggtacacttcttttaata
ttgggtgatcttcattctaatacacttttctttgatccaatatttggaggagatcctata
ttctatcaacatttattttggtttttggacatccagaagtttacatattaataattcct
gcatttgggatcatttccataataatttctggtattttacagttaataatctttgctaac
caatcaatgatctttgccatgtcatctatttctcttcttggaggtcttgtttggggacat
catatgtatactgtaggtttagaaagtgatacaagagcttattttacaggagttacaatc
ttaatatccttaccaactggtacaaaaatctttaattggctttttacatatctctcaaat
ccaccattattacaccttagaattacttctgtcttcctctcacatctctttttattaatg
tttacgataggtgggtcaacaggaataattcttggaaatggtgcagtggatctaggatta
catgatacatattatgttgtagcacattttcattttgttctttctttaggagctataatt
gctatcttctctggaataatcttgaatggagaaaagattgttgctactaagaatttatta
ctttcatcctcatgtacactctctctttatcatttacatttaatatttattggtattctt
cttaccttttccccaatgcatttcttaggatttaatgttatgccaagaagaatcccatcc
tttccagattcttttcattcctggaattccctgtcatctattggatcaggaataactttc
ctatctttttctatg
>Mz10_cox1
ataaatctcttaaccattaatttcctatttcattcactagttaaaaattgtaatcataaa
ggcttaggaatctattatttattatctggattcatctttggaatctccggtacattaata
tcagtccttataagaatagaattatattcttcaggaaataggattatatctccagaaaac
cagaacttctataatataagcattacattgcatggctttcttatgattttctttttagta
atgcctggcttgtttggaggatttggaaattattttgtacctatctttcaagggtctcca
gaagtggtatatcctagagtcaataatttttctatcttaattcttttgctttcatatctt
ttcctaatcctttctttaatctcagaatttggaggtggtacagggtggacgctctaccca
ccattatccacttcttttatgactttatcaccttcaagtacaggaaatcttatatttgga
ttaataatctctggtatatcttcatgtcttacatctcttaacttttggacaacaattcat
tttctgagatcttattatctgatattatcttctatcccattatttccttgggctttcttg
attacagctttcatgcttttattaacattaccaatcttatctggtacacttcttttaata
ttgggtgatcttcattctaatacacttttctttgatccaatatttggaggagatcctata
ttctatcaacatttattttggtttttggacatccagaagtttacatattaataattcct
gcatttgggatcatttccataataatttctggtattttacagttaataatctttgctaac
caatcaatgatctttgccatgtcatctatttctcttcttggaggtcttgtttggggacat
catatgtatactgtaggtttagaaagtgatacaagagcttattttacaggagttacaatc
ttaatatccttaccaactggtacaaaaatctttaattggctttttacatatctctcaaat
ccaccattattacaccttagaattacttctgtcttcctctcacatctctttttattaatg
tttacgataggtgggtcaacaggaataattcttggaaatggtgcagtggatctaggatta
catgatacatattatgttgtagcacattttcattttgttctttctttaggagctataatt
gctatcttctctggaataatcttgaatggagaaaagattgttgctactaagaatttatta
ctttcatcctcatgtacactctctctttatcatttacatttaatatttattggtattctt
cttaccttttccccaatgcatttcttaggatttaatgttatgccaagaagaatcccatcc
tttccagattcttttcattcctggaattccctgtcatctattggatcaggaataactttc
ctatctttttctatg
>Mz5_cox1
ataaatctcttaaccattaatttcctatttcattcactagttaaaaattgtaatcataaa
ggcttaggaatctattatttattatctggattcatctttggaatctccggtacattaata
tcagtccttataagaatagaattatattcttcaggaaataggattatatctccagaaaac
cagaacttctataatataagcattacattgcatggctttcttatgattttctttttagta
atgcctggcttgtttggaggatttggaaattattttgtacctatctttcaagggtctcca
gaagtggtatatcctagagtcaataatttttctatcttaattcttttgctttcatatctt
ttcctaatcctttctttaatctcagaatttggaggtggtacagggtggacgctctaccca
ccattatccacttcttttatgactttatcaccttcaagtacaggaaatcttatatttgga
ttaataatctctggtatatcttcatgtcttacatctcttaacttttggacaacaattcat
tttctgagatcttattatctgatattatcttctatcccattatttccttgggctttcttg
attacagctttcatgcttttattaacattaccaatcttatctggtacacttcttttaata
ttgggtgatcttcattctaatacacttttctttgatccaatatttggaggagatcctata
ttctatcaacatttattttggtttttggacatccagaagtttacatattaataattcct
gcatttgggatcatttccataataatttctggtattttacagttaataatctttgctaac
caatcaatgatctttgccatgtcatctatttctcttcttggaggtcttgtttggggacat

catatgtatactgtaggtttagaaagtgatacaagagcttattttacaggagttacaatc
ttaatatccttaccaactggtacaaaaatctttaattggctttttacatatctctcaaat
ccaccattattacaccttagaattacttctgtcttcctctcacatctctttttattaatg
tttacgataggtgggtcaacaggaataattcttggaaatggtgcagtggatctaggatta
catgatacatattatgttgtagcacattttcattttgttctttctttaggagctataatt
gctatcttctctggaataatcttgaatggagaaaagattgttgctactaagaatttatta
ctttcatcctcatgtacactctctctttatcatttacatttaatatttattggtattctt
cttacctttccccaatgcatttcttaggatttaatgttatgccaagaagaatcccatcc
tttccagattcttttcattcctggaattccctgtcatctattggatcaggaataactttc
ctatctttttctatg
>Mz6_cox1
ataaatctcttaaccattaatttcctatttcattcactagttaaaaattgtaatcataaa
ggcttaggaatctattatttattatctggattcatctttggaatctccggtacattaata
tcagtccttataagaatagaattatattcttcaggaaataggattatatctccagaaaac
cagaacttctataatataagcattacattgcatggctttcttatgattttctttttagta
atgcctggcttgtttggaggatttggaaattattttgtacctatctttcaagggtctcca
gaagtggtatatcctagagtcaataatttttctatcttaattcttttgctttcatatctt
ttcctaatcctttctttaatctcagaatttggaggtggtacagggtggacgctctaccca
ccattatccacttcttttatgactttatcaccttcaagtacaggaaatcttatatttgga
ttaataatctctggtatatcttcatgtcttacatctcttaacttttggacaacaattcat
tttctgagatcttattatctgatattatcttctatcccattatttccttgggctttcttg
attacagctttcatgcttttattaacattaccaatcttatctggtacacttcttttaata
ttgggtgatcttcattctaatacacttttctttgatccaatatttggaggagatcctata
ttctatcaacatttattttggtttttggacatccagaagtttacatattaataattcct
gcatttgggatcatttccataataatttctggtattttacagttaataatctttgctaac
caatcaatgatctttgccatgtcatctatttctcttcttggaggtcttgtttggggacat
catatgtatactgtaggtttagaaagtgatacaagagcttattttacaggagttacaatc
ttaatatccttaccaactggtacaaaaatctttaattggctttttacatatctctcaaat
ccaccattattacaccttagaattacttctgtcttcctctcacatctctttttattaatg
tttacgataggtgggtcaacaggaataattcttggaaatggtgcagtggatctaggatta
catgatacatattatgttgtagcacattttcattttgttctttctttaggagctataatt
gctatcttctctggaataatcttgaatggagaaaagattgttgctactaagaatttatta
ctttcatcctcatgtacactctctctttatcatttacatttaatatttattggtattctt
cttacctttccccaatgcatttcttaggatttaatgttatgccaagaagaatcccatcc
tttccagattcttttcattcctggaattccctgtcatctattggatcaggaataactttc
ctatctttttctatg
>Mz7_cox1
ataaatctcttaaccattaatttcctatttcattcactagttaaaaattgtaatcataaa
ggcttaggaatctattatttattatctggattcatctttggaatctccggtacattaata
tcagtccttataagaatagaattatattcttcaggaaataggattatatctccagaaaac
cagaacttctataatataagcattacattgcatggctttcttatgattttctttttagta
atgcctggcttgtttggaggatttggaaattattttgtacctatctttcaagggtctcca
gaagtggtatatcctagagtcaataatttttctatcttaattcttttgctttcatatctt
ttcctaatcctttctttaatctcagaatttggaggtggtacagggtggacgctctaccca
ccattatccacttcttttatgactttatcaccttcaagtacaggaaatcttatatttgga
ttaataatctctggtatatcttcatgtcttacatctcttaacttttggacaacaattcat
tttctgagatcttattatctgatattatcttctatcccattatttccttgggctttcttg
attacagctttcatgcttttattaacattaccaatcttatctggtacacttcttttaata
ttgggtgatcttcattctaatacacttttctttgatccaatatttggaggagatcctata
ttctatcaacatttattttggtttttggacatccagaagtttacatattaataattcct
gcatttgggatcatttccataataatttctggtattttacagttaataatctttgctaac
caatcaatgatctttgccatgtcatctatttctcttcttggaggtcttgtttggggacat
catatgtatactgtaggtttagaaagtgatacaagagcttattttacaggagttacaatc
ttaatatccttaccaactggtacaaaaatctttaattggctttttacatatctctcaaat
ccaccattattacaccttagaattacttctgtcttcctctcacatctctttttattaatg
tttacgataggtgggtcaacaggaataattcttggaaatggtgcagtggatctaggatta
catgatacatattatgttgtagcacattttcattttgttctttctttaggagctataatt

gctatcttctctggaataatcttgaatggagaaaagattgttgctactaagaatttatta
ctttcatcctcatgtacactctctctttatcatttacatttaatatttattggtattctt
cttaccttttccccaatgcatttcttaggatttaatgttatgccaagaagaatcccatcc
tttccagattcttttcattcctggaattccctgtcatctattggatcaggaataactttc
ctatctttttctatg
>Mz8_cox1
ttaaatctcttaaccattaatttcctatttcattcactagttaaaaattgtaatcataaa
ggcttaggaatctattatttattatctggattcatctttggaatctccggtacattaata
tcagtccttataagaatagaattatattcttcaggaaataggattatatctccagaaaac
cagaacttctataatataagcattacattgcatggctttcttatgattttctttttagta
atgcctggcttgtttggaggatttggaaattattttgtacctatctttcaagggtctcca
gaagtggtatatcctagagtcaataatttttctatcttaattcttttgctttcatatctt
ttcctaatcctttctttaatctcagaatttggaggtggtacagggtggacgctctaccca
ccattatccacttcttttatgactttatcaccttcaagtacaggaaatcttatatttgga
ttaataatctctggtatatcttcatgtcttacatctcttaacttttggacaacaattcat
tttctgagatcttattatctgatattatcttctatcccattatttccttgggctttcttg
attacagctttcatgcttttattaacattaccaatcttatctggtacacttcttttaata
ttgggtgatcttcattctaatacacttttctttgatccaatatttggaggagatcctata
ttctatcaacatttattttggtttttggacatccagaagtttacatattaataattcct
gcatttgggatcatttccataataatttctggtattttacagttaataatctttgctaac
caatcaatgatctttgccatgtcatctatttctcttcttggaggtcttgtttggggacat
catatgtatactgtaggtttagaaagtgatacaagagcttattttacaggagttacaatc
ttaatatccttaccaactggtacaaaaatctttaattggctttttacatatctctcaaat
ccaccattattacaccttagaattacttctgtcttcctctcacatctctttttattaatg
tttacgataggtgggtcaacaggaataattcttggaaatggtgcagtggatctaggatta
catgatacatattatgttgtagcacattttcattttgttctttctttaggagctataatt
gctatcttctctggaataatcttgaatggagaaaagattgttgctactaagaatttatta
ctttcatcctcatgtacactctctctttatcatttacatttaatatttattggtattctt
cttaccttttccccaatgcatttcttaggatttaatgttatgccaagaagaatcccatcc
tttccagattcttttcattcctggaattccctgtcatctattggatcaggaataactttc
ctatctttttctatg
>Mz9_cox1
ttaaatctcttaaccattaatttcctatttcattcactagttaaaaattgtaatcataaa
ggcttaggaatctattatttattatctggattcatctttggaatctccggtacattaata
tcagtccttataagaatagaattatattcttcaggaaataggattatatctccagaaaac
cagaacttctataatataagcattacattgcatggctttcttatgattttctttttagta
atgcctggcttgtttggaggatttggaaattattttgtacctatctttcaagggtctcca
gaagtggtatatcctagagtcaataatttttctatcttaattcttttgctttcatatctt
ttcctaatcctttctttaatctcagaatttggaggtggtacagggtggacgctctaccca
ccattatccacttcttttatgactttatcaccttcaagtacaggaaatcttatatttgga
ttaataatctctggtatatcttcatgtcttacatctcttaacttttggacaacaattcat
tttctgagatcttattatctgatattatcttctatcccattatttccttgggctttcttg
attacagctttcatgcttttattaacattaccaatcttatctggtacacttcttttaata
ttgggtgatcttcattctaatacacttttctttgatccaatatttggaggagatcctata
ttctatcaacatttattttggtttttggacatccagaagtttacatattaataattcct
gcatttgggatcatttccataataatttctggtattttacagttaataatctttgctaac
caatcaatgatctttgccatgtcatctatttctcttcttggaggtcttgtttggggacat
catatgtatactgtaggtttagaaagtgatacaagagcttattttacaggagttacaatc
ttaatatccttaccaactggtacaaaaatctttaattggctttttacatatctctcaaat
ccaccattattacaccttagaattacttctgtcttcctctcacatctctttttattaatg
tttacgataggtgggtcaacaggaataattcttggaaatggtgcagtggatctaggatta
catgatacatattatgttgtagcacattttcattttgttctttctttaggagctataatt
gctatcttctctggaataatcttgaatggagaaaagattgttgctactaagaatttatta
ctttcatcctcatgtacactctctctttatcatttacatttaatatttattggtattctt
cttaccttttccccaatgcatttcttaggatttaatgttatgccaagaagaatcccatcc
tttccagattcttttcattcctggaattccctgtcatctattggatcaggaataactttc
ctatctttttctatg

>MzC1_cox1
ataaatctcttaaccattaatttcctatttcattcactagttaaaaattgtaatcataaa
ggcttaggaatctattatttattatctggattcatctttggaatctccggtacattaata
tcagtccttataagaatagaattatattcttcaggaaataggattatatctccagaaaac
cagaacttctataatataagcattacattgcatggctttcttatgattttctttttagta
atgcctggcttgtttggaggatttggaaattattttgtacctatctttcaagggtctcca
gaagtggtatatcctagagtcaataatttttctatcttaattcttttgctttcatatctt
ttcctaatcctttctttaatctcagaatttggaggtggtacagggtggacgctctaccca
ccattatccacttcttttatgactttatcaccttcaagtacaggaaatcttatatttgga
ttaataatctctggtatatcttcatgtcttacatctcttaacttttggacaacaattcat
tttctgagatcttattatctgatattatcttctatcccattatttccttgggctttcttg
attacagctttcatgcttttattaacattaccaatcttatctggtacacttcttttaata
ttgggtgatcttcattctaatacacttttctttgatccaatatttggaggagatcctata
ttctatcaacatttattttggtttttggacatccagaagtttacatattaataattcct
gcatttgggatcatttccataataatttctggtattttacagttaataatctttgctaac
caatcaatgatctttgccatgtcatctatttctcttcttggaggtcttgtttggggacat
catatgtatactgtaggtttagaaagtgatacaagagcttattttacaggagttacaatc
ttaatatccttaccaactggtacaaaaatctttaattggctttttacatatctctcaaat
ccaccattattacaccttagaattacttctgtcttcctctcacatctctttttattaatg
tttacgataggtgggtcaacaggaataattcttggaaatggtgcagtggatctaggatta
catgatacatattatgttgtagcacattttcattttgttctttctttaggagctataatt
gctatcttctctggaataatcttgaatggagaaaagattgttgctactaagaatttatta
ctttcatcctcatgtacactctctctttatcatttacatttaatatttattggtattctt
cttaccttttccccaatgcatttcttaggatttaatgttatgccaagaagaatcccatcc
tttccagattcttttcattcctggaattccctgtcatctattggatcaggaataactttc
ctatctttttctatg
>MzC1GaII_cox1
ttaaatctcttaaccattaatttcctatttcattcactagttaaaaattgtaatcataaa
ggcttaggaatctattatttattatctggattcatctttggaatctccggtacattaata
tcagtccttataagaatagaattatattcttcaggaaataggattatatctccagaaaac
cagaacttctataatataagcattacattgcatggctttcttatgattttctttttagta
atgcctggcttgtttggaggatttggaaattattttgtacctatctttcaagggtctcca
gaagtggtatatcctagagtcaataatttttctatcttaattcttttgctttcatatctt
ttcctaatcctttctttaatctcagaatttggaggtggtacagggtggacgctctaccca
ccattatccacttcttttatgactttatcaccttcaagtacaggaaatcttatatttgga
ttaataatctctggtatatcttcatgtcttacatctcttaacttttggacaacaattcat
tttctgagatcttattatctgatattatcttctatcccattatttccttgggctttcttg
attacagctttcatgcttttattaacattaccaatcttatctggtacacttcttttaata
ttgggtgatcttcattctaatacacttttctttgatccaatatttggaggagatcctata
ttctatcaacatttattttggtttttggacatccagaagtttacatattaataattcct
gcatttgggatcatttccataataatttctggtattttacagttaataatctttgctaac
caatcaatgatctttgccatgtcatctatttctcttcttggaggtcttgtttggggacat
catatgtatactgtaggtttagaaagtgatacaagagcttattttacaggagttacaatc
ttaatatccttaccaactggtacaaaaatctttaattggctttttacatatctctcaaat
ccaccattattacaccttagaattacttctgtcttcctctcacatctctttttattaatg
tttacgataggtgggtcaacaggaataattcttggaaatggtgcagtggatctaggatta
catgatacatattatgttgtagcacattttcattttgttctttctttaggagctataatt
gctatcttctctggaataatcttgaatggagaaaagattgttgctactaagaatttatta
ctttcatcctcatgtacactctctctttatcatttacatttaatatttattggtattctt
cttaccttttccccaatgcatttcttaggatttaatgttatgccaagaagaatcccatcc
tttccagattcttttcattcctggaattccctgtcatctattggatcaggaataactttc
ctatctttttctatg
>MzC2_cox1
ataaatctcttaaccattaatttcctatttcattcactagttaaaaattgtaatcataaa
ggcttaggaatctattatttattatctggattcatctttggaatctccggtacattaata
tcagtccttataagaatagaattatattcttcaggaaataggattatatctccagaaaac
cagaacttctataatataagcattacattgcatggctttcttatgattttctttttagta

atgcctggcttgtttggaggatttggaaattattttgtacctatctttcaagggtctcca
gaagtggtatatcctagagtcaataatttttctatcttaattcttttgctttcatatctt
ttcctaatcctttctttaatctcagaatttggaggtggtacagggtggacgctctaccca
ccattatccacttcttttatgactttatcaccttcaagtacaggaaatcttatatttgga
ttaataatctctggtatatcttcatgtcttacatctcttaacttttggacaacaattcat
tttctgagatcttattatctgatattatcttctatcccattatttccttgggctttcttg
attacagctttcatgcttttattaacattaccaatcttatctggtacacttcttttaata
ttgggtgatcttcattctaatacacttttctttgatccaatatttggaggagatcctata
ttctatcaacatttattttggtttttggacatccagaagtttacatattaataattcct
gcatttgggatcatttccataataatttctggtattttacagttaataatctttgctaac
caatcaatgatctttgccatgtcatctatttctcttcttggaggtcttgtttggggacat
catatgtatactgtaggtttagaaagtgatacaagagcttattttacaggagttacaatc
ttaatatccttaccaactggtacaaaaatctttaattggctttttacatatctctcaaat
ccaccattattacaccttagaattacttctgtcttcctctcacatctcttttattaatg
tttacgataggtgggtcaacaggaataattcttggaaatggtgcagtggatctaggatta
catgatacatattatgttgtagcacattttcattttgttctttctttaggagctataatt
gctatcttctctggaataatcttgaatggagaaaagattgttgctactaagaatttatta
ctttcatcctcatgtacactctctctttatcatttacatttaatatttattggtattctt
cttaccttttccccaatgcatttcttaggatttaatgttatgccaagaagaatcccatcc
tttccagattcttttcattcctggaattccctgtcatctattggatcaggaataactttc
ctatctttttctatg
>MzC2GaII_cox1
ataaatctcttaaccattaatttcctatttcattcactagttaaaaattgtaatcataaa
ggcttaggaatctattatttattatctggattcatctttggaatctccggtacattaata
tcagtccttataagaatagaattatattcttcaggaaataggattatatctccagaaaac
cagaacttctataatataagcattacattgcatggctttcttatgattttctttttagta
atgcctggcttgtttggaggatttggaaattattttgtacctatctttcaagggtctcca
gaagtggtatatcctagagtcaataatttttctatcttaattcttttgctttcatatctt
ttcctaatcctttctttaatctcagaatttggaggtggtacagggtggacgctctaccca
ccattatccacttcttttatgactttatcaccttcaagtacaggaaatcttatatttgga
ttaataatctctggtatatcttcatgtcttacatctcttaacttttggacaacaattcat
tttctgagatcttattatctgatattatcttctatcccattatttccttgggctttcttg
attacagctttcatgcttttattaacattaccaatcttatctggtacacttcttttaata
ttgggtgatcttcattctaatacacttttctttgatccaatatttggaggagatcctata
ttctatcaacatttattttggtttttggacatccagaagtttacatattaataattcct
gcatttgggatcatttccataataatttctggtattttacagttaataatctttgctaac
caatcaatgatctttgccatgtcatctatttctcttcttggaggtcttgtttggggacat
catatgtatactgtaggtttagaaagtgatacaagagcttattttacaggagttacaatc
ttaatatccttaccaactggtacaaaaatctttaattggctttttacatatctctcaaat
ccaccattattacaccttagaattacttctgtcttcctctcacatctcttttattaatg
tttacgataggtgggtcaacaggaataattcttggaaatggtgcagtggatctaggatta
catgatacatattatgttgtagcacattttcattttgttctttctttaggagctataatt
gctatcttctctggaataatcttgaatggagaaaagattgttgctactaagaatttatta
ctttcatcctcatgtacactctctctttatcatttacatttaatatttattggtattctt
cttaccttttccccaatgcatttcttaggatttaatgttatgccaagaagaatcccatcc
tttccagattcttttcattcctggaattccctgtcatctattggatcaggaataactttc
ctatctttttctatg
>MzC3_cox1
ttaaatctcttaaccattaatttcctatttcattcactagttaaaaattgtaatcataaa
ggcttaggaatctattatttattatctggattcatctttggaatctccggtacattaata
tcagtccttataagaatagaattatattcttcaggaaataggattatatctccagaaaac
cagaacttctataatataagcattacattgcatggctttcttatgattttctttttagta
atgcctggcttgtttggaggatttggaaattattttgtacctatctttcaagggtctcca
gaagtggtatatcctagagtcaataatttttctatcttaattcttttgctttcatatctt
ttcctaatcctttctttaatctcagaatttggaggtggtacagggtggacgctctaccca
ccattatccacttcttttatgactttatcaccttcaagtacaggaaatcttatatttgga
ttaataatctctggtatatcttcatgtcttacatctcttaacttttggacaacaattcat

tttctgagatcttattatctgatattatcttctatcccattatttccttgggctttcttg
attacagctttcatgcttttattaacattaccaatcttatctggtacacttcttttaata
ttgggtgatcttcattctaatacacttttctttgatccaatatttggaggagatcctata
ttctatcaacatttattttggtttttggacatccagaagtttacatattaataattcct
gcatttgggatcatttccataataatttctggtattttacagttaataatctttgctaac
caatcaatgatctttgccatgtcatctatttctcttcttggaggtcttgtttggggacat
catatgtatactgtaggtttagaaagtgatacaagagcttattttacaggagttacaatc
ttaatatccttaccaactggtacaaaaatctttaattggctttttacatatctctcaaat
ccaccattattacaccttagaattacttctgtcttcctctcacatctctttttattaatg
tttacgataggtgggtcaacaggaataattcttggaaatggtgcagtggatctaggatta
catgatacatattatgttgtagcacattttcattttgttctttctttaggagctataatt
gctatcttctctggaataatcttgaatggagaaaagattgttgctactaagaatttatta
ctttcatcctcatgtacactctctctttatcatttacatttaatatttattggtattctt
cttaccttttccccaatgcatttcttaggatttaatgttatgccaagaagaatcccatcc
tttccagattcttttcattcctggaattccctgtcatctattggatcaggaataactttc
ctatctttttctatg
>MzC4_cox1
ataaatctcttaaccattaatttcctatttcattcactagttaaaaattgtaatcataaa
ggcttaggaatctattatttattatctggattcatctttggaatctccggtacattaata
tcagtccttataagaatagaattatattcttcaggaaataggattatatctccagaaaac
cagaacttctataatataagcattacattgcatggctttcttatgattttcttttagta
atgcctggcttgtttggaggatttggaaattattttgtacctatctttcaagggtctcca
gaagtggtatatcctagagtcaataatttttctatcttaattcttttgctttcatatctt
ttcctaatcctttctttaatctcagaatttggaggtggtacagggtggacgctctaccca
ccattatccacttcttttatgactttatcaccttcaagtacaggaaatcttatatttgga
ttaataatctctggtatatcttcatgtcttacatctcttaacttttggacaacaattcat
tttctgagatcttattatctgatattatcttctatcccattatttccttgggctttcttg
attacagctttcatgcttttattaacattaccaatcttatctggtacacttcttttaata
ttgggtgatcttcattctaatacacttttctttgatccaatatttggaggagatcctata
ttctatcaacatttattttggtttttggacatccagaagtttacatattaataattcct
gcatttgggatcatttccataataatttctggtattttacagttaataatctttgctaac
caatcaatgatctttgccatgtcatctatttctcttcttggaggtcttgtttggggacat
catatgtatactgtaggtttagaaagtgatacaagagcttattttacaggagttacaatc
ttaatatccttaccaactggtacaaaaatctttaattggctttttacatatctctcaaat
ccaccattattacaccttagaattacttctgtcttcctctcacatctctttttattaatg
tttacgataggtgggtcaacaggaataattcttggaaatggtgcagtggatctaggatta
catgatacatattatgttgtagcacattttcattttgttctttctttaggagctataatt
gctatcttctctggaataatcttgaatggagaaaagattgttgctactaagaatttatta
ctttcatcctcatgtacactctctctttatcatttacatttaatatttattggtattctt
cttaccttttccccaatgcatttcttaggatttaatgttatgccaagaagaatcccatcc
tttccagattcttttcattcctggaattccctgtcatctattggatcaggaataactttc
ctatctttttctatg
>Od10_cox1
ataaatctcttaaccattaatttcctatttcattcactagttaaaaattgtaatcataaa
ggcttaggaatctattatttattatctggattcatctttggaatctccggtacattaata
tcagtccttataagaatagaattatattcttcaggaaataggattatatctccagaaaac
cagaacttctataatataagcattacattgcatggctttcttatgattttcttttagta
atgcctggcttgtttggaggatttggaaattattttgtacctatctttcaagggtctcca
gaagtggtatatcctagagtcaataatttttctatcttaattcttttgctttcatatctt
ttcctaatcctttctttaatctcagaatttggaggtggtacagggtggacgctctaccca
ccattatccacttcttttatgactttatcaccttcaagtacaggaaatcttatatttgga
ttaataatctctggtatatcttcatgtcttacatctcttaacttttggacaacaattcat
tttctgagatcttattatctgatattatcttctatcccattatttccttgggctttcttg
attacagctttcatgcttttattaacattaccagtcttatctggtacacttcttttaata
ttgggtgatcttcattctaatacacttttctttgatccaatatttggaggagatcctata
ttctatcaacatttattttggtttttggacatccagaagtttacatattaataattcct
gcatttgggatcatttccataataatttctggtattttacagttaataatctttgctaac

caatcaatgatctttgccatgtcatctatttctcttcttggaggtcttgtttggggacat
catatgtatactgtaggtttagaaagtgatacaagagcttattttacaggagttacaatc
ttaatatccttaccaactggtacaaaaatctttaattggctttttacatatctctcaaat
ccaccattattacaccttagaattacttctgtcttcctctcacatctctttttattaatg
tttacgataggtgggtcaacaggaataattcttggaaatggtgcagtggatctaggatta
catgatacatattatgttgtagcacattttcattttgttctttctttaggagctataatt
gctatcttctctggaataatcttgaatggagaaaagattgttgctactaagaatttatta
ctttcatcctcatgtacactctctctttatcatttacatttaatatttattggtattctt
cttacctttccccaatgcatttcttaggatttaatgttatgccaagaagaatcccatcc
tttccagattcttttcattcctggaattccctgtcatctattggatcaggaataactttc
ctatctttttctatg
>Od11_cox1
ataaatctcttaaccattaatttcctatttcattcactagttaaaaattgtaatcataaa
ggcttaggaatctattatttattatctggattcatctttggaatctccggtacattaata
tcagtccttataagaatagaattatattcttcaggaaataggattatatctccagaaaac
cagaacttctataatataagcattacattgcatggctttcttatgattttctttttagta
atgcctggcttgtttggaggatttggaaattattttgtacctatctttcaagggtctcca
gaagtggtatatcctagagtcaataatttttctatcttaattcttttgctttcatatctt
ttcctaatcctttctttaatctcagaatttggaggtggtacagggtggacgctctaccca
ccattatccacttcttttatgactttatcaccttcaagtacaggaaatcttatatttgga
ttaataatctctggtatatcttcatgtcttacatctcttaacttttggacaacaattcat
tttctgagatcttattatctgatattatcttctatcccattatttccttgggctttcttg
attacagctttcatgcttttattaacattaccaatcttatctggtacacttcttttaata
ttgggtgatcttcattctaatacacttttctttgatccaatatttggaggagatcctata
ttctatcaacatttattttggtttttggacatccagaagtttacatattaataattcct
gcatttgggatcatttccataataatttctggtattttacagttaataatctttgctaac
caatcaatgatctttgccatgtcatctatttctcttcttggaggtcttgtttggggacat
catatgtatactgtaggtttagaaagtgatacaagagcttattttacaggagttacaatc
ttaatatccttaccaactggtacaaaaatctttaattggctttttacatatctctcaaat
ccaccattattacaccttagaattacttctgtcttcctctcacatctctttttattaatg
tttacgataggtgggtcaacaggaataattcttggaaatggtgcagtggatctaggatta
catgatacatattatgttgtagcacattttcattttgttctttctttaggagctataatt
gctatcttctctggaataatcttgaatggagaaaagattgttgctactaagaatttatta
ctttcatcctcatgtacactctctctttatcatttacatttaatatttattggtattctt
cttacctttccccaatgcatttcttaggatttaatgttatgccaagaagaatcccatcc
tttccagattcttttcattcctggaattccctgtcatctattggatcaggaataactttc
ctatctttttctatg
>Od12_cox1
ataaatctcttaaccattaatttcctatttcattcactagttaaaaattgtaatcataaa
ggcttaggaatctattatttattatctggattcatctttggaatctccggtacattaata
tcagtccttataagaatagaattatattcttcaggaaataggattatatctccagaaaac
cagaacttctataatataagcattacattgcatggctttcttatgattttctttttagta
atgcctggcttgtttggaggatttggaaattattttgtacctatctttcaagggtctcca
gaagtggtatatcctagagtcaataatttttctatcttaattcttttgctttcatatctt
ttcctaatcctttctttaatctcagaatttggaggtggtacagggtggacgctctaccca
ccattatccacttcttttatgactttatcaccttcaagtacaggaaatcttatatttgga
ttaataatctctggtatatcttcatgtcttacatctcttaacttttggacaacaattcat
tttctgagatcttattatctgatattatcttctatcccattatttccttgggctttcttg
attacagctttcatgcttttattaacattaccaatcttatctggtacacttcttttaata
ttgggtgatcttcattctaatacacttttctttgatccaatatttggaggagatcctata
ttctatcaacatttattttggtttttggacatccagaagtttacatattaataattcct
gcatttgggatcatttccataataatttctggtattttacagttaataatctttgctaac
caatcaatgatctttgccatgtcatctatttctcttcttggaggtcttgtttggggacat
catatgtatactgtaggtttagaaagtgatacaagagcttattttacaggagttacaatc
ttaatatccttaccaactggtacaaaaatctttaattggctttttacatatctctcaaat
ccaccattattacaccttagaattacttctgtcttcctctcacatctctttttattaatg
tttacgataggtgggtcaacaggaataattcttggaaatggtgcagtggatctaggatta

catgatacatattatgttgtagcacattttcattttgttctttctttaggagctataatt
gctatcttctctggaataatcttgaatggagaaaagattgttgctactaagaatttatta
ctttcatcctcatgtacactctctctttatcatttacatttaatatttattggtattctt
cttaccttttccccaatgcatttcttaggatttaatgttatgccaagaagaatcccatcc
tttccagattcttttcattcctggaattccctgtcatctattggatcaggaataactttc
ctatctttttctatg
>Od13_cox1
ataaatctcttaaccattaatttcctatttcattcactagttaaaaattgtaatcataaa
ggcttaggaatctattatttattatctggattcatctttggaatctccggtacattaata
tcagtccttataagaatagaattatattcttcaggaaataggattatatctccagaaaac
cagaacttctataatataagcattacattgcatggctttcttatgattttctttttagta
atgcctggcttgtttggaggatttggaaattattttgtacctatctttcaagggtctcca
gaagtggtatatcctagagtcaataatttttctatcttaattcttttgctttcatatctt
ttcctaatcctttctttaatctcagaatttggaggtggtacagggtggacgctctaccca
ccattatccacttcttttatgactttatcaccttcaagtacaggaaatcttatatttgga
ttaataatctctggtatatcttcatgtcttacatctcttaacttttggacaacaattcat
tttctgagatcttattatctgatattatcttctatcccattatttccttgggctttcttg
attacagctttcatgcttttattaacattaccaatcttatctggtacacttcttttaata
ttgggtgatcttcattctaatacacttttctttgatccaatatttggaggagatcctata
ttctatcaacatttattttggttttttggacatccagaagtttacatattaataattcct
gcatttgggatcatttccataataatttctggtattttacagttaataatctttgctaac
caatcaatgatctttgccatgtcatctatttctcttcttggaggtcttgtttggggacat
catatgtatactgtaggtttagaaagtgatacaagagcttattttacaggagttacaatc
ttaatatccttaccaactggtacaaaaaatctttaattggctttttacatatctctcaaat
ccaccattattacaccttagaattacttctgtcttcctctcacatctctttttattaatg
tttacgataggtgggtcaacaggaataattcttggaaatggtgcagtggatctaggatta
catgatacatattatgttgtagcacattttcattttgttctttctttaggagctataatt
gctatcttctctggaataatcttgaatggagaaaagattgttgctactaagaatttatta
ctttcatcctcatgtacactctctctttatcatttacatttaatatttattggtattctt
cttaccttttccccaatgcatttcttaggatttaatgttatgccaagaagaatcccatcc
tttccagattcttttcattcctggaattccctgtcatctattggatcaggaataactttc
ctatctttttctatg
>Od14_cox1
ataaatctcttaaccattaatttcctatttcattcactagttaaaaattgtaatcataaa
ggcttaggaatctattatttattatctggattcatctttggaatctccggtacattaata
tcagtccttataagaatagaattatattcttcaggaaataggattatatctccagaaaac
cagaacttctataatataagcattacattgcatggctttcttatgattttctttttagta
atgcctggcttgtttggaggatttggaaattattttgtacctatctttcaagggtctcca
gaagtggtatatcctagagtcaataatttttctatcttaattcttttgctttcatatctt
ttcctaatcctttctttaatctcagaatttggaggtggtacagggtggacgctctaccca
ccattatccacttcttttatgactttatcaccttcaagtacaggaaatcttatatttgga
ttaataatctctggtatatcttcatgtcttacatctcttaacttttggacaacaattcat
tttctgagatcttattatctgatattatcttctatcccattatttccttgggctttcttg
attacagctttcatgcttttattaacattaccaatcttatctggtacacttcttttaata
ttgggtgatcttcattctaatacacttttctttgatccaatatttggaggagatcctata
ttctatcaacatttattttggttttttggacatccagaagtttacatattaataattcct
gcatttgggatcatttccataataatttctggtattttacagttaataatctttgctaac
caatcaatgatctttgccatgtcatctatttctcttcttggaggtcttgtttggggacat
catatgtatactgtaggtttagaaagtgatacaagagcttattttacaggagttacaatc
ttaatatccttaccaactggtacaaaaaatctttaattggctttttacatatctctcaaat
ccaccattattacaccttagaattacttctgtcttcctctcacatctctttttattaatg
tttacgataggtgggtcaacaggaataattcttggaaatggtgcagtggatctaggatta
catgatacatattatgttgtagcacattttcattttgttctttctttaggagctataatt
gctatcttctctggaataatcttgaatggagaaaagattgttgctactaagaatttatta
ctttcatcctcatgtacactctctctttatcatttacatttaatatttattggtattctt
cttaccttttccccaatgcatttcttaggatttaatgttatgccaagaagaatcccatcc
tttccagattcttttcattcctggaattccctgtcatctattggatcaggaataactttc

ctatctttttctatg
>Od15_cox1
ataaatctcttaaccattaatttcctatttcattcactagttaaaaattgtaatcataaa
ggcttaggaatctattatttattatctggattcatctttggaatctccggtacattaata
tcagtccttataagaatagaattatattcttcaggaaataggattatatctccagaaaac
cagaacttctataatataagcattacattgcatggctttcttatgattttctttttagta
atgcctggcttgtttggaggatttggaaattattttgtacctatctttcaagggtctcca
gaagtggtatatcctagagtcaataatttttctatcttaattcttttgctttcatatctt
ttcctaatcctttctttaatctcagaatttggaggtggtacagggtggacgctctaccca
ccattatccacttcttttatgactttatcaccttcaagtacaggaaatcttatatttgga
ttaataatctctggtatatcttcatgtcttacatctcttaacttttggacaacaattcat
tttctgagatcttattatctgatattatcttctatcccattatttccttgggctttcttg
attacagctttcatgcttttattaacattaccaatcttatctggtacacttcttttaata
ttgggtgatcttcattctaatacacttttctttgatccaatatttggaggagatcctata
ttctatcaacatttattttggtttttggacatccagaagtttacatattaataattcct
gcatttgggatcatttccataataatttctggtattttacagttaataatctttgctaac
caatcaatgatctttgccatgtcatctatttctcttcttggaggtcttgtttggggacat
catatgtatactgtaggtttagaaagtgatacaagagcttattttacaggagttacaatc
ttaatatccttaccaactggtacaaaaatctttaattggctttttacatatctctcaaat
ccaccattattacaccttagaattacttctgtcttcctctcacatctctttttattaatg
tttacgataggtgggtcaacaggaataattcttggaaatggtgcagtggatctaggatta
catgatacatattatgttgtagcacattttcattttgttctttctttaggagctataatt
gctatcttctctggaataatcttgaatggagaaaagattgttgctactaagaatttatta
ctttcatcctcatgtacactctctctttatcatttacatttaatatttattggtattctt
cttaccttttccccaatgcatttcttaggatttaatgttatgccaagaagaatcccatcc
tttccagattcttttcattcctggaattccctgtcatctattggatcaggaataactttc
ctatctttttctatg
>Od18_cox1
ataaatctcttaaccattaatttcctatttcattcactagttaaaaattgtaatcataaa
ggcttaggaatctattatttattatctggattcatctttggaatctccggtacattaata
tcagtccttataagaatagaattatattcttcaggaaataggattatatctccagaaaac
cagaacttctataatataagcattacattgcatggctttcttatgattttctttttagta
atgcctggcttgtttggaggatttggaaattattttgtacctatctttcaagggtctcca
gaagtggtatatcctagagtcaataatttttctatcttaattcttttgctttcatatctt
ttcctaatcctttctttaatctcagaatttggaggtggtacagggtggacgctctaccca
ccattatccacttcttttatgactttatcaccttcaagtacaggaaatcttatatttgga
ttaataatctctggtatatcttcatgtcttacatctcttaacttttggacaacaattcat
tttctgagatcttattatctgatattatcttctatcccattatttccttgggctttcttg
attacagctttcatgcttttattaacattaccaatcttatctggtacacttcttttaata
ttgggtgatcttcattctaatacacttttctttgatccaatatttggaggagatcctata
ttctatcaacatttattttggtttttggacatccagaagtttacatattaataattcct
gcatttgggatcatttccataataatttctggtattttacagttaataatctttgctaac
caatcaatgatctttgccatgtcatctatttctcttcttggaggtcttgtttggggacat
catatgtatactgtaggtttagaaagtgatacaagagcttattttacaggagttacaatc
ttaatatccttaccaactggtacaaaaatctttaattggctttttacatatctctcaaat
ccaccattattacaccttagaattacttctgtcttcctctcacatctctttttattaatg
tttacgataggtgggtcaacaggaataattcttggaaatggtgcagtggatctaggatta
catgatacatattatgttgtagcacattttcattttgttctttctttaggagctataatt
gctatcttctctggaataatcttgaatggagaaaagattgttgctactaagaatttatta
ctttcatcctcatgtacactctctctttatcatttacatttaatatttattggtattctt
cttaccttttccccaatgcatttcttaggatttaatgttatgccaagaagaatcccatcc
tttccagattcttttcattcctggaattccctgtcatctattggatcaggaataactttc
ctatctttttctatg
>Od19_cox1
ataaatctcttaaccattaatttcctatttcattcactagttaaaaattgtaatcataaa
ggcttaggaatctattatttattatctggattcatctttggaatctccggtacattaata
tcagtccttataagaatagaattatattcttcaggaaataggattatatctccagaaaac

cagaacttctataatataagcattacattgcatggctttcttatgattttctttttagta
atgcctggcttgtttggaggatttggaaattattttgtacctatctttcaagggtctcca
gaagtggtatatcctagagtcaataatttttctatcttaattcttttgctttcatatctt
ttcctaatcctttctttaatctcagaatttggaggtggtacagggtggacgctctaccca
ccattatccacttcttttatgactttatcaccttcaagtacaggaaatcttatatttgga
ttaataatctctggtatatcttcatgtcttacatctcttaacttttggacaacaattcat
tttctgagatcttattatctgatattatcttctatcccattatttccttgggctttcttg
attacagctttcatgcttttattaacattaccaatcttatctggtacacttcttttaata
ttgggtgatcttcattctaatacacttttctttgatccaatatttggaggagatcctata
ttctatcaacatttattttggtttttggacatccagaagtttacatattaataattcct
gcatttgggatcatttccataataatttctggtattttacagttaataatctttgctaac
caatcaatgatctttgccatgtcatctatttctcttcttggaggtcttgtttggggacat
catatgtatactgtaggtttagaaagtgatacaagagcttattttacaggagttacaatc
ttaatatccttaccaactggtacaaaaatctttaattggctttttacatatctctcaaat
ccaccattattacaccttagaattacttctgtcttcctctcacatctcttttattaatg
tttacgataggtgggtcaacaggaataattcttggaaatggtgcagtggatctaggatta
catgatacatattatgttgtagcacattttcattttgttctttctttaggagctataatt
gctatcttctctggaataatcttgaatggagaaaagattgttgctactaagaatttatta
ctttcatcctcatgtacactctctctttatcatttacatttaatatttattggtattctt
cttacctttccccaatgcatttcttaggatttaatgttatgccaagaagaatcccatcc
tttccagattcttttcattcctggaattccctgtcatctattggatcaggaataactttc
ctatctttttctatg
>Od2_cox1
ttaaatctcttaaccattaatttcctatttcattcactagttaaaaaattgtaatcataaa
ggcttaggaatctattatttattatctggattcatctttggaatctccggtacattaata
tcagtccttataagaatagaattatattcttcaggaaataggattatatctccagaaaac
cagaacttctataatataagcattacattgcatggctttcttatgattttctttttagta
atgcctggcttgtttggaggatttggaaattattttgtacctatctttcaagggtctcca
gaagtggtatatcctagagtcaataatttttctatcttaattcttttgctttcatatctt
ttcctaatcctttctttaatctcagaatttggaggtggtacagggtggacgctctaccca
ccattatccacttcttttatgactttatcaccttcaagtacaggaaatcttatatttgga
ttaataatctctggtatatcttcatgtcttacatctcttaacttttggacaacaattcat
tttctgagatcttattatctgatattatcttctatcccattatttccttgggctttcttg
attacagctttcatgcttttattaacattaccaatcttatctggtacacttcttttaata
ttgggtgatcttcattctaatacacttttctttgatccaatatttggaggagatcctata
ttctatcaacatttattttggtttttggacatccagaagtttacatattaataattcct
gcatttgggatcatttccataataatttctggtattttacagttaataatctttgctaac
caatcaatgatctttgccatgtcatctatttctcttcttggaggtcttgtttggggacat
catatgtatactgtaggtttagaaagtgatacaagagcttattttacaggagttacaatc
ttaatatccttaccaactggtacaaaaatctttaattggctttttacatatctctcaaat
ccaccattattacaccttagaattacttctgtcttcctctcacatctcttttattaatg
tttacgataggtgggtcaacaggaataattcttggaaatggtgcagtggatctaggatta
catgatacatattatgttgtagcacattttcattttgttctttctttaggagctataatt
gctatcttctctggaataatcttgaatggagaaaagattgttgctactaagaatttatta
ctttcatcctcatgtacactctctctttatcatttacatttaatatttattggtattctt
cttacctttccccaatgcatttcttaggatttaatgttatgccaagaagaatcccatcc
tttccagattcttttcattcctggaattccctgtcatctattggatcaggaataactttc
ctatctttttctatg
>Od22_cox1
ttaaatctcttaaccattaatttcctatttcattcactagttaaaaaattgtaatcataaa
ggcttaggaatctattatttattatctggattcatctttggaatctccggtacattaata
tcagtccttataagaatagaattatattcttcaggaaataggattatatctccagaaaac
cagaacttctataatataagcattacattgcatggctttcttatgattttctttttagta
atgcctggcttgtttggaggatttggaaattattttgtacctatctttcaagggtctcca
gaagtggtatatcctagagtcaataatttttctatcttaattcttttgctttcatatctt
ttcctaatcctttctttaatctcagaatttggaggtggtacagggtggacgctctaccca
ccattatccacttcttttatgactttatcaccttcaagtacaggaaatcttatatttgga

ttaataatctctggtatatcttcatgtcttacatctcttaacttttggacaacaattcat
tttctgagatcttattatctgatattatcttctatcccattatttccttgggctttcttg
attacagctttcatgcttttattaacattaccaatcttatctggtacacttcttttaata
ttgggtgatcttcattctaatacacttttctttgatccaatatttggaggagatcctata
ttctatcaacatttattttggttttttggacatccagaagtttacatattaataattcct
gcatttgggatcatttccataataatttctggtattttacagttaataatctttgctaac
caatcaatgatctttgccatgtcatctatttctcttcttggaggtcttgtttggggacat
catatgtatactgtaggtttagaaagtgatacaagagcttattttacaggagttacaatc
ttaatatccttaccaactggtacaaaaatctttaattggctttttacatatctctcaaat
ccaccattattacaccttagaattacttctgtcttcctctcacatctctttttattaatg
tttacgataggtgggtcaacaggaataattcttggaaatggtgcagtggatctaggatta
catgatacatattatgttgtagcacattttcattttgttctttctttaggagctataatt
gctatcttctctggaataatcttgaatggagaaaagattgttgctactaagaatttatta
ctttcatcctcatgtacactctctctttatcatttacatttaatatttattggtattctt
cttaccttttccccaatgcatttcttaggatttaatgttatgccaagaagaatcccatcc
tttccagattcttttcattcctggaattccctgtcatctattggatcaggaataactttc
ctatctttttctatg
>Od23_cox1
ataaatctcttaaccattaatttcctatttcattcactagttaaaaattgtaatcataaa
ggcttaggaatctattatttattatctggattcatctttggaatctccggtacattaata
tcagtccttataagaatagaattatattcttcaggaaataggattatatctccagaaaac
cagaacttctataatataagcattacattgcatggctttcttatgattttctttttagta
atgcctggcttgtttggaggatttggaaattattttgtacctatctttcaagggtctcca
gaagtggtatatcctagagtcaataatttttctatcttaattcttttgctttcatatctt
ttcctaatcctttctttaatctcagaatttggaggtggtacagggtggacgctctaccca
ccattatccacttcttttatgactttatcaccttcaagtacaggaaatcttatatttgga
ttaataatctctggtatatcttcatgtcttacatctcttaacttttggacaacaattcat
tttctgagatcttattatctgatattatcttctatcccattatttccttgggctttcttg
attacagctttcatgcttttattaacattaccaatcttatctggtacacttcttttaata
ttgggtgatcttcattctaatacacttttctttgatccaatatttggaggagatcctata
ttctatcaacatttattttggttttttggacatccagaagtttacatattaataattcct
gcatttgggatcatttccataataatttctggtattttacagttaataatctttgctaac
caatcaatgatctttgccatgtcatctatttctcttcttggaggtcttgtttggggacat
catatgtatactgtaggtttagaaagtgatacaagagcttattttacaggagttacaatc
ttaatatccttaccaactggtacaaaaatctttaattggctttttacatatctctcaaat
ccaccattattacaccttagaattacttctgtcttcctctcacatctctttttattaatg
tttacgataggtgggtcaacaggaataattcttggaaatggtgcagtggatctaggatta
catgatacatattatgttgtagcacattttcattttgttctttctttaggagctataatt
gctatcttctctggaataatcttgaatggagaaaagattgttgctactaagaatttatta
ctttcatcctcatgtacactctctctttatcatttacatttaatatttattggtattctt
cttaccttttccccaatgcatttcttaggatttaatgttatgccaagaagaatcccatcc
tttccagattcttttcattcctggaattccctgtcatctattggatcaggaataactttc
ctatctttttctatg
>Od24_cox1
ttaaatctcttaaccattaatttcctatttcattcactagttaaaaattgtaatcataaa
ggcttaggaatctattatttattatctggattcatctttggaatctccggtacattaata
tcagtccttataagaatagaattatattcttcaggaaataggattatatctccagaaaac
cagaacttctataatataagcattacattgcatggctttcttatgattttctttttagta
atgcctggcttgtttggaggatttggaaattattttgtacctatctttcaagggtctcca
gaagtggtatatcctagagtcaataatttttctatcttaattcttttgctttcatatctt
ttcctaatcctttctttaatctcagaatttggaggtggtacagggtggacgctctaccca
ccattatccacttcttttatgactttatcaccttcaagtacaggaaatcttatatttgga
ttaataatctctggtatatcttcatgtcttacatctcttaacttttggacaacaattcat
tttctgagatcttattatctgatattatcttctatcccattatttccttgggctttcttg
attacagctttcatgcttttattaacattaccaatcttatctggtacacttcttttaata
ttgggtgatcttcattctaatacacttttctttgatccaatatttggaggagatcctata
ttctatcaacatttattttggttttttggacatccagaagtttacatattaataattcct

gcatttgggatcatttccataataatttctggtattttacagttaataatctttgctaac
caatcaatgatctttgccatgtcatctatttctcttcttggaggtcttgtttggggacat
catatgtatactgtaggtttagaaagtgatacaagagcttattttacaggagttacaatc
ttaatatccttaccaactggtacaaaaatctttaattggctttttacatatctctcaaat
ccaccattattacaccttagaattacttctgtcttcctctcacatctctttttattaatg
tttacgataggtgggtcaacaggaataattcttggaaatggtgcagtggatctaggatta
catgatacatattatgttgtagcacattttcattttgttctttctttaggagctataatt
gctatcttctctggaataatcttgaatggagaaaagattgttgctactaagaatttatta
ctttcatcctcatgtacactctctctttatcatttacatttaatatttattggtattctt
cttacctttcccccaatgcatttcttaggatttaatgttatgccaagaagaatcccatcc
tttccagattcttttcattcctggaattccctgtcatctattggatcaggaataactttc
ctatctttttctatg
>Od25_cox1
ataaatctcttaaccattaatttcctatttcattcactagttaaaaattgtaatcataaa
ggcttaggaatctattatttattatctggattcatctttggaatctccggtacattaata
tcagtccttataagaatagaattatattcttcaggaaataggattatatctccagaaaac
cagaacttctataatataagcattacattgcatggctttcttatgattttctttttagta
atgcctggcttgtttggaggatttggaaattattttgtacctatctttcaagggtctcca
gaagtggtatatcctagagtcaataatttttctatcttaattcttttgctttcatatctt
ttcctaatcctttctttaatctcagaatttggaggtggtacagggtggacgctctaccca
ccattatccacttcttttatgactttatcaccttcaagtacaggaaatcttatatttgga
ttaataatctctggtatatcttcatgtcttacatctcttaacttttggacaacaattcat
tttctgagatcttattatctgatattatcttctatcccattatttccttgggctttcttg
attacagctttcatgcttttattaacattaccaatcttatctggtacacttcttttaata
ttgggtgatcttcattctaatacacttttctttgatccaatatttggaggagatcctata
ttctatcaacatttattttggtttttggacatccagaagtttacatattaataattcct
gcatttgggatcatttccataataatttctggtattttacagttaataatctttgctaac
caatcaatgatctttgccatgtcatctatttctcttcttggaggtcttgtttggggacat
catatgtatactgtaggtttagaaagtgatacaagagcttattttacaggagttacaatc
ttaatatccttaccaactggtacaaaaatctttaattggctttttacatatctctcaaat
ccaccattattacaccttagaattacttctgtcttcctctcacatctctttttattaatg
tttacgataggtgggtcaacaggaataattcttggaaatggtgcagtggatctaggatta
catgatacatattatgttgtagcacattttcattttgttctttctttaggagctataatt
gctatcttctctggaataatcttgaatggagaaaagattgttgctactaagaatttatta
ctttcatcctcatgtacactctctctttatcatttacatttaatatttattggtattctt
cttacctttcccccaatgcatttcttaggatttaatgttatgccaagaagaatcccatcc
tttccagattcttttcattcctggaattccctgtcatctattggatcaggaataactttc
ctatctttttctatg
>Od26_cox1
ataaatctcttaaccattaatttcctatttcattcactagttaaaaattgtaatcataaa
ggcttaggaatctattatttattatctggattcatctttggaatctccggtacattaata
tcagtccttataagaatagaattatattcttcaggaaataggattatatctccagaaaac
cagaacttctataatataagcattacattgcatggctttcttatgattttctttttagta
atgcctggcttgtttggaggatttggaaattattttgtacctatctttcaagggtctcca
gaagtggtatatcctagagtcaataatttttctatcttaattcttttgctttcatatctt
ttcctaatcctttctttaatctcagaatttggaggtggtacagggtggacgctctaccca
ccattatccacttcttttatgactttatcaccttcaagtacaggaaatcttatatttgga
ttaataatctctggtatatcttcatgtcttacatctcttaacttttggacaacaattcat
tttctgagatcttattatctgatattatcttctatcccattatttccttgggctttcttg
attacagctttcatgcttttattaacattaccaatcttatctggtacacttcttttaata
ttgggtgatcttcattctaatacacttttctttgatccaatatttggaggagatcctata
ttctatcaacatttattttggtttttggacatccagaagtttacatattaataattcct
gcatttgggatcatttccataataatttctggtattttacagttaataatctttgctaac
caatcaatgatctttgccatgtcatctatttctcttcttggaggtcttgtttggggacat
catatgtatactgtaggtttagaaagtgatacaagagcttattttacaggagttacaatc
ttaatatccttaccaactggtacaaaaatctttaattggctttttacatatctctcaaat
ccaccattattacaccttagaattacttctgtcttcctctcacatctctttttattaatg

tttacgataggtgggtcaacaggaataattcttggaaatggtgcagtggatctaggatta
catgatacatattatgttgtagcacattttcattttgttctttctttaggagctataatt
gctatcttctctggaataatcttgaatggagaaaagattgttgctactaagaatttatta
ctttcatcctcatgtacactctctctttatcatttacatttaatatttattggtattctt
cttaccttttccccaatgcatttcttaggatttaatgttatgccaagaagaatcccatcc
tttccagattcttttcattcctggaattccctgtcatctattggatcaggaataactttc
ctatctttttctatg
>Od27_cox1
ataaatctcttaaccattaatttcctatttcattcactagttaaaaattgtaatcataaa
ggcttaggaatctattatttattatctggattcatctttggaatctccggtacattaata
tcagtccttataagaatagaattatattcttcaggaaataggattatatctccagaaaac
cagaacttctataatataagcattacattgcatggctttcttatgattttctttttagta
atgcctggcttgtttggaggatttggaaattattttgtacctatctttcaagggtctcca
gaagtggtatatcctagagtcaataatttttctatcttaattcttttgctttcatatctt
ttcctaatcctttctttaatctcagaatttggaggtggtacagggtggacgctctaccca
ccattatccacttcttttatgactttatcaccttcaagtacaggaaatcttatatttgga
ttaataatctctggtatatcttcatgtcttacatctcttaacttttggacaacaattcat
tttctgagatcttattatctgatattatcttctatcccattatttccttgggctttcttg
attacagctttcatgcttttattaacattaccaatcttatctggtacacttcttttaata
ttgggtgatcttcattctaatacacttttctttgatccaatatttggaggagatcctata
ttctatcaacatttattttggtttttggacatccagaagtttacatattaataattcct
gcatttgggatcatttccataataatttctggtattttacagttaataatctttgctaac
caatcaatgatctttgccatgtcatctatttctcttcttggaggtcttgtttggggacat
catatgtatactgtaggtttagaaagtgatacaagagcttattttacaggagttacaatc
ttaatatccttaccaactggtacaaaaatctttaattggctttttacatatctctcaaat
ccaccattattacaccttagaattacttctgtcttcctctcacatctctttttattaatg
tttacgataggtgggtcaacaggaataattcttggaaatggtgcagtggatctaggatta
catgatacatattatgttgtagcacattttcattttgttctttctttaggagctataatt
gctatcttctctggaataatcttgaatggagaaaagattgttgctactaagaatttatta
ctttcatcctcatgtacactctctctttatcatttacatttaatatttattggtattctt
cttaccttttccccaatgcatttcttaggatttaatgttatgccaagaagaatcccatcc
tttccagattcttttcattcctggaattccctgtcatctattggatcaggaataactttc
ctatctttttctatg
>Od4_cox1
ataaatctcttaaccattaatttcctatttcattcactagttaaaaattgtaatcataaa
ggcttaggaatctattatttattatctggattcatctttggaatctccggtacattaata
tcagtccttataagaatagaattatattcttcaggaaataggattatatctccagaaaac
cagaacttctataatataagcattacattgcatggctttcttatgattttctttttagta
atgcctggcttgtttggaggatttggaaattattttgtacctatctttcaagggtctcca
gaagtggtatatcctagagtcaataatttttctatcttaattcttttgctttcatatctt
ttcctaatcctttctttaatctcagaatttggaggtggtacagggtggacgctctaccca
ccattatccacttcttttatgactttatcaccttcaagtacaggaaatcttatatttgga
ttaataatctctggtatatcttcatgtcttacatctcttaacttttggacaacaattcat
tttctgagatcttattatctgatattatcttctatcccattatttccttgggctttcttg
attacagctttcatgcttttattaacattaccaatcttatctggtacacttcttttaata
ttgggtgatcttcattctaatacacttttctttgatccaatatttggaggagatcctata
ttctatcaacatttattttggtttttggacatccagaagtttacatattaataattcct
gcatttgggatcatttccataataatttctggtattttacagttaataatctttgctaac
caatcaatgatctttgccatgtcatctatttctcttcttggaggtcttgtttggggacat
catatgtatactgtaggtttagaaagtgatacaagagcttattttacaggagttacaatc
ttaatatccttaccaactggtacaaaaatctttaattggctttttacatatctctcaaat
ccaccattattacaccttagaattacttctgtcttcctctcacatctctttttattaatg
tttacgataggtgggtcaacaggaataattcttggaaatggtgcagtggatctaggatta
catgatacatattatgttgtagcacattttcattttgttctttctttaggagctataatt
gctatcttctctggaataatcttgaatggagaaaagattgttgctactaagaatttatta
ctttcatcctcatgtacactctctctttatcatttacatttaatatttattggtattctt
cttaccttttccccaatgcatttcttaggatttaatgttatgccaagaagaatcccatcc

tttccagattcttttcattcctggaattccctgtcatctattggatcaggaataactttc
ctatctttttctatg
>Od6_cox1
ataaatctcttaaccattaatttcctatttcattcactagttaaaaattgtaatcataaa
ggcttaggaatctattatttattatctggattcatctttggaatctccggtacattaata
tcagtccttataagaatagaattatattcttcaggaaataggattatatctccagaaaac
cagaacttctataatataagcattacattgcatggctttcttatgattttctttttagta
atgcctggcttgtttggaggatttggaaattattttgtacctatctttcaagggtctcca
gaagtggtatatcctagagtcaataatttttctatcttaattcttttgctttcatatctt
ttcctaatcctttctttaatctcagaatttggaggtggtacagggtggacgctctaccca
ccattatccacttcttttatgactttatcaccttcaagtacaggaaatcttatatttgga
ttaataatctctggtatatcttcatgtcttacatctcttaacttttggacaacaattcat
tttctgagatcttattatctgatattatcttctatcccattatttccttgggctttcttg
attacagctttcatgcttttattaacattaccaatcttatctggtacacttcttttaata
ttgggtgatcttcattctaatacacttttctttgatccaatatttggaggagatcctata
ttctatcaacatttattttggtttttggacatccagaagtttacatattaataattcct
gcatttgggatcatttccataataatttctggtattttacagttaataatctttgctaac
caatcaatgatctttgccatgtcatctatttctcttcttggaggtcttgtttggggacat
catatgtatactgtaggtttagaaagtgatacaagagcttattttacaggagttacaatc
ttaatatccttaccaactggtacaaaaatctttaattggctttttacatatctctcaaat
ccaccattattacaccttagaattacttctgtcttcctctcacatctcttttattaatg
tttacgataggtgggtcaacaggaataattcttggaaatggtgcagtggatctaggatta
catgatacatattatgttgtagcacattttcattttgttctttctttaggagctataatt
gctatcttctctggaataatcttgaatggagaaaagattgttgctactaagaatttatta
ctttcatcctcatgtacactctctctttatcatttacatttaatatttattggtattctt
cttaccttttccccaatgcatttcttaggatttaatgttatgccaagaagaatcccatcc
tttccagattcttttcattcctggaattccctgtcatctattggatcaggaataactttc
ctatctttttctatg
>Od8_cox1
ataaatctcttaaccattaatttcctatttcattcactagttaaaaattgtaatcataaa
ggcttaggaatctattatttattatctggattcatctttggaatctccggtacattaata
tcagtccttataagaatagaattatattcttcaggaaataggattatatctccagaaaac
cagaacttctataatataagcattacattgcatggctttcttatgattttctttttagta
atgcctggcttgtttggaggatttggaaattattttgtacctatctttcaagggtctcca
gaagtggtatatcctagagtcaataatttttctatcttaattcttttgctttcatatctt
ttcctaatcctttctttaatctcagaatttggaggtggtacagggtggacgctctaccca
ccattatccacttcttttatgactttatcaccttcaagtacaggaaatcttatatttgga
ttaataatctctggtatatcttcatgtcttacatctcttaacttttggacaacaattcat
tttctgagatcttattatctgatattatcttctatcccattatttccttgggctttcttg
attacagctttcatgcttttattaacattaccaatcttatctggtacacttcttttaata
ttgggtgatcttcattctaatacacttttctttgatccaatatttggaggagatcctata
ttctatcaacatttattttggtttttggacatccagaagtttacatattaataattcct
gcatttgggatcatttccataataatttctggtattttacagttaataatctttgctaac
caatcaatgatctttgccatgtcatctatttctcttcttggaggtcttgtttggggacat
catatgtatactgtaggtttagaaagtgatacaagagcttattttacaggagttacaatc
ttaatatccttaccaactggtacaaaaatctttaattggctttttacatatctctcaaat
ccaccattattacaccttagaattacttctgtcttcctctcacatctcttttattaatg
tttacgataggtgggtcaacaggaataattcttggaaatggtgcagtggatctaggatta
catgatacatattatgttgtagcacattttcattttgttctttctttaggagctataatt
gctatcttctctggaataatcttgaatggagaaaagattgttgctactaagaatttatta
ctttcatcctcatgtacactctctctttatcatttacatttaatatttattggtattctt
cttaccttttccccaatgcatttcttaggatttaatgttatgccaagaagaatcccatcc
tttccagattcttttcattcctggaattccctgtcatctattggatcaggaataactttc
ctatctttttctatg
>Od9_cox1
ttaaatctcttaaccattaatttcctatttcattcactagttaaaaattgtaatcataaa
ggcttaggaatctattatttattatctggattcatctttggaatctccggtacattaata

tcagtccttataagaatagaattatattcttcaggaaataggattatatctccagaaaac
cagaacttctataatataagcattacattgcatggctttcttatgattttctttttagta
atgcctggcttgtttggaggatttggaaattattttgtacctatctttcaagggtctcca
gaagtggtatatcctagagtcaataatttttctatcttaattcttttgctttcatatctt
ttcctaatcctttctttaatctcagaatttggaggtggtacagggtggacgctctaccca
ccattatccacttcttttatgactttatcaccttcaagtacaggaaatcttatatttgga
ttaataatctctggtatatcttcatgtcttacatctcttaacttttggacaacaattcat
tttctgagatcttattatctgatattatcttctatcccattatttccttgggctttcttg
attacagctttcatgcttttattaacattaccaatcttatctggtacacttcttttaata
ttgggtgatcttcattctaatacacttttctttgatccaatatttggaggagatcctata
ttctatcaacatttattttggtttttggacatccagaagtttacatattaataattcct
gcatttgggatcatttccataataatttctggtattttacagttaataatctttgctaac
caatcaatgatctttgccatgtcatctatttctcttcttggaggtcttgtttggggacat
catatgtatactgtaggtttagaaagtgatacaagagcttattttacaggagttacaatc
ttaatatccttaccaactggtacaaaaatctttaattggctttttacatatctctcaaat
ccaccattattacaccttagaattacttctgtcttcctctcacatctctttttattaatg
tttacgataggtgggtcaacaggaataattcttggaaatggtgcagtggatctaggatta
catgatacatattatgttgtagcacattttcattttgttctttctttaggagctataatt
gctatcttctctggaataatcttgaatggagaaaagattgttgctactaagaatttatta
ctttcatcctcatgtacactctctctttatcatttacatttaatatttattggtattctt
cttaccttttccccaatgcatttcttaggatttaatgttatgccaagaagaatcccatcc
tttccagattcttttcattcctggaattccctgtcatctattggatcaggaataactttc
ctatctttttctatg
>Ss5_cox1
ataaatctcttaaccattaatttcctatttcattcactagttaaaaattgtaatcataaa
ggcttaggaatctattatttattatctggattcatctttggaatctccggtacattaata
tcagtccttataagaatagaattatattcttcaggaaataggattatatctccagaaaac
cagaacttctataatataagcattacattgcatggctttcttatgattttctttttagta
atgcctggcttgtttggaggatttggaaattattttgtacctatctttcaagggtctcca
gaagtggtatatcctagagtcaataatttttctatcttaattcttttgctttcatatctt
ttcctaatcctttctttaatctcagaatttggaggtggtacagggtggacgctctaccca
ccattatccacttcttttatgactttatcaccttcaagtacaggaaatcttatatttgga
ttaataatctctggtatatcttcatgtcttacatctcttaacttttggacaacaattcat
tttctgagatcttattatctgatattatcttctatcccattatttccttgggctttcttg
attacagctttcatgcttttattaacattaccaatcttatctggtacacttcttttaata
ttgggtgatcttcattctaatacacttttctttgatccaatatttggaggagatcctata
ttctatcaacatttattttggtttttggacatccagaagtttacatattaataattcct
gcatttgggatcatttccataataatttctggtattttacagttaataatctttgctaac
caatcaatgatctttgccatgtcatctatttctcttcttggaggtcttgtttggggacat
catatgtatactgtaggtttagaaagtgatacaagagcttattttacaggagttacaatc
ttaatatccttaccaactggtacaaaaatctttaattggctttttacatatctctcaaat
ccaccattattacaccttagaattacttctgtcttcctctcacatctctttttattaatg
tttacgataggtgggtcaacaggaataattcttggaaatggtgcagtggatctaggatta
catgatacatattatgttgtagcacattttcattttgttctttctttaggagctataatt
gctatcttctctggaataatcttgaatggagaaaagattgttgctactaagaatttatta
ctttcatcctcatgtacactctctctttatcatttacatttaatatttattggtattctt
cttaccttttccccaatgcatttcttaggatttaatgttatgccaagaagaatcccatcc
tttccagattcttttcattcctggaattccctgtcatctattggatcaggaataactttc
ctatctttttctatg
>Ss6_cox1
ataaatctcttaaccattaatttcctatttcattcactagttaaaaattgtaatcataaa
ggcttaggaatctattatttattatctggattcatctttggaatctccggtacattaata
tcagtccttataagaatagaattatattcttcaggaaataggattatatctccagaaaac
cagaacttctataatataagcattacattgcatggctttcttatgattttctttttagta
atgcctggcttgtttggaggatttggaaattattttgtacctatctttcaagggtctcca
gaagtggtatatcctagagtcaataatttttctatcttaattcttttgctttcatatctt
ttcctaatcctttctttaatctcagaatttggaggtggtacagggtggacgctctaccca

ccattatccacttcttttatgactttatcaccttcaagtacaggaaatcttatatttgga
ttaataatctctggtatatcttcatgtcttacatctcttaacttttggacaacaattcat
tttctgagatcttattatctgatattatcttctatcccattatttccttgggctttcttg
attacagctttcatgcttttattaacattaccaatcttatctggtacacttcttttaata
ttgggtgatcttcattctaatacacttttctttgatccaatatttggaggagatcctata
ttctatcaacatttattttggtttttggacatccagaagtttacatattaataattcct
gcatttgggatcatttccataataatttctggtattttacagttaataatctttgctaac
caatcaatgatctttgccatgtcatctatttctcttcttggaggtcttgtttggggacat
catatgtatactgtaggtttagaaagtgatacaagagcttattttacaggagttacaatc
ttaatatccttaccaactggtacaaaaatctttaattggctttttacatatctctcaaat
ccaccattattacaccttagaattacttctgtcttcctctcacatctctttttattaatg
tttacgataggtgggtcaacaggaataattcttggaaatggtgcagtggatctaggatta
catgatacatattatgttgtagcacattttcattttgttctttctttaggagctataatt
gctatcttctctggaataatcttgaatggagaaaagattgttgctactaagaatttatta
ctttcatcctcatgtacactctctctttatcatttacatttaatatttattggtattctt
cttaccttttccccaatgcatttcttaggatttaatgttatgccaagaagaatcccatcc
tttccagattcttttcattcctggaattccctgtcatctattggatcaggaataactttc
ctatctttttctatg
>Ss7_cox1
ataaatctcttaaccattaatttcctatttcattcactagttaaaaaattgtaatcataaa
ggcttaggaatctattatttattatctggattcatctttggaatctccggtacattaata
tcagtccttataagaatagaattatattcttcaggaaataggattatatctccagaaaac
cagaacttctataatataagcattacattgcatggctttcttatgattttctttttagta
atgcctggcttgtttggaggatttggaaattattttgtacctatctttcaagggtctcca
gaagtggtatatcctagagtcaataatttttctatcttaattcttttgctttcatatctt
ttcctaatcctttctttaatctcagaatttggaggtggtacagggtggacgctctaccca
ccattatccacttcttttatgactttatcaccttcaagtacaggaaatcttatatttgga
ttaataatctctggtatatcttcatgtcttacatctcttaacttttggacaacaattcat
tttctgagatcttattatctgatattatcttctatcccattatttccttgggctttcttg
attacagctttcatgcttttattaacattaccaatcttatctggtacacttcttttaata
ttgggtgatcttcattctaatacacttttctttgatccaatatttggaggagatcctata
ttctatcaacatttattttggtttttggacatccagaagtttacatattaataattcct
gcatttgggatcatttccataataatttctggtattttacagttaataatctttgctaac
caatcaatgatctttgccatgtcatctatttctcttcttggaggtcttgtttggggacat
catatgtatactgtaggtttagaaagtgatacaagagcttattttacaggagttacaatc
ttaatatccttaccaactggtacaaaaatctttaattggctttttacatatctctcaaat
ccaccattattacaccttagaattacttctgtcttcctctcacatctctttttattaatg
tttacgataggtgggtcaacaggaataattcttggaaatggtgcagtggatctaggatta
catgatacatattatgttgtagcacattttcattttgttctttctttaggagctataatt
gctatcttctctggaataatcttgaatggagaaaagattgttgctactaagaatttatta
ctttcatcctcatgtacactctctctttatcatttacatttaatatttattggtattctt
cttaccttttccccaatgcatttcttaggatttaatgttatgccaagaagaatcccatcc
tttccagattcttttcattcctggaattccctgtcatctattggatcaggaataactttc
ctatctttttctatg
>Ss8_cox1
ataaatctcttaaccattaatttcctatttcattcactagttaaaaaattgtaatcataaa
ggcttaggaatctattatttattatctggattcatctttggaatctccggtacattaata
tcagtccttataagaatagaattatattcttcaggaaataggattatatctccagaaaac
cagaacttctataatataagcattacattgcatggctttcttatgattttctttttagta
atgcctggcttgtttggaggatttggaaattattttgtacctatctttcaagggtctcca
gaagtggtatatcctagagtcaataatttttctatcttaattcttttgctttcatatctt
ttcctaatcctttctttaatctcagaatttggaggtggtacagggtggacgctctaccca
ccattatccacttcttttatgactttatcaccttcaagtacaggaaatcttatatttgga
ttaataatctctggtatatcttcatgtcttacatctcttaacttttggacaacaattcat
tttctgagatcttattatctgatattatcttctatcccattatttccttgggctttcttg
attacagctttcatgcttttattaacattaccaatcttatctggtacacttcttttaata
ttgggtgatcttcattctaatacacttttctttgatccaatatttggaggagatcctata

ttctatcaacatttattttggttttttggacatccagaagtttacatattaataattcct
gcatttgggatcatttccataataatttctggtattttacagttaataatctttgctaac
caatcaatgatctttgccatgtcatctatttctcttcttggaggtcttgtttggggacat
catatgtatactgtaggtttagaaagtgatacaagagcttattttacaggagttacaatc
ttaatatccttaccaactggtacaaaaatctttaattggctttttacatatctctcaaat
ccaccattattacaccttagaattacttctgtcttcctctcacatctctttttattaatg
tttacgataggtgggtcaacaggaataattcttggaaatggtgcagtggatctaggatta
catgatacatattatgttgtagcacattttcattttgttctttctttaggagctataatt
gctatcttctctggaataatcttgaatggagaaaagattgttgctactaagaatttatta
ctttcatcctcatgtacactctctctttatcatttacatttaatatttattggtattctt
cttaccttttccccaatgcatttcttaggatttaatgttatgccaagaagaatcccatcc
tttccagattcttttcattcctggaattccctgtcatctattggatcaggaataactttc
ctatctttttctatg
>ohdo3_cox1
ataaatctcttaaccattaatttcctatttcattcactagttaaaaattgtaatcataaa
ggcttaggaatctattatttattatctggattcatctttggaatctccggtacattaata
tcagtccttataagaatagaattatattcttcaggaaataggattatatctccagaaaac
cagaacttctataatataagcattacattgcatggctttcttatgattttctttttagta
atgcctggcttgtttggaggatttggaaattattttgtacctatctttcaagggtctcca
gaagtggtatatcctagagtcaataatttttctatcttaattcttttgctttcatatctt
ttcctaatcctttctttaatctcagaatttggaggtggtacagggtggacgctctaccca
ccattatccacttcttttatgactttatcaccttcaagtacaggaaatcttatatttgga
ttaataatctctggtatatcttcatgtcttacatctcttaacttttggacaacaattcat
tttctgagatcttattatctgatattatcttctatcccattatttccttgggctttcttg
attacagctttcatgcttttattaacattaccaatcttatctggtacacttcttttaata
ttgggtgatcttcattctaatacacttttctttgatccaatatttggaggagatcctata
ttctatcaacatttattttggttttttggacatccagaagtttacatattaataattcct
gcatttgggatcatttccataataatttctggtattttacagttaataatctttgctaac
caatcaatgatctttgccatgtcatctatttctcttcttggaggtcttgtttggggacat
catatgtatactgtaggtttagaaagtgatacaagagcttattttacaggagttacaatc
ttaatatccttaccaactggtacaaaaatctttaattggctttttacatatctctcaaat
ccaccattattacaccttagaattacttctgtcttcctctcacatctctttttattaatg
tttacgataggtgggtcaacaggaataattcttggaaatggtgcagtggatctaggatta
catgatacatattatgttgtagcacattttcattttgttctttctttaggagctataatt
gctatcttctctggaataatcttgaatggagaaaagattgttgctactaagaatttatta
ctttcatcctcatgtacactctctctttatcatttacatttaatatttattggtattctt
cttaccttttccccaatgcatttcttaggatttaatgttatgccaagaagaatcccatcc
tttccagattcttttcattcctggaattccctgtcatctattggatcaggaataactttc
ctatctttttctatg
>ohdo5_cox1
ataaatctcttaaccattaatttcctatttcattcactagttaaaaattgtaatcataaa
ggcttaggaatctattatttattatctggattcatctttggaatctccggtacattaata
tcagtccttataagaatagaattatattcttcaggaaataggattatatctccagaaaac
cagaacttctataatataagcattacattgcatggctttcttatgattttctttttagta
atgcctggcttgtttggaggatttggaaattattttgtacctatctttcaagggtctcca
gaagtggtatatcctagagtcaataatttttctatcttaattcttttgctttcatatctt
ttcctaatcctttctttaatctcagaatttggaggtggtacagggtggacgctctaccca
ccattatccacttcttttatgactttatcaccttcaagtacaggaaatcttatatttgga
ttaataatctctggtatatcttcatgtcttacatctcttaacttttggacaacaattcat
tttctgagatcttattatctgatattatcttctatcccattatttccttgggctttcttg
attacagctttcatgcttttattaacattaccaatcttatctggtacacttcttttaata
ttgggtgatcttcattctaatacacttttctttgatccaatatttggaggagatcctata
ttctatcaacatttattttggttttttggacatccagaagtttacatattaataattcct
gcatttgggatcatttccataataatttctggtattttacagttaataatctttgctaac
caatcaatgatctttgccatgtcatctatttctcttcttggaggtcttgtttggggacat
catatgtatactgtaggtttagaaagtgatacaagagcttattttacaggagttacaatc
ttaatatccttaccaactggtacaaaaatctttaattggctttttacatatctctcaaat

ccaccattattacaccttagaattacttctgtcttcctctcacatctctttttattaatg
tttacgataggtgggtcaacaggaataattcttggaaatggtgcagtggatctaggatta
catgatacatattatgttgtagcacattttcattttgttctttctttaggagctataatt
gctatcttctctggaataatcttgaatggagaaaagattgttgctactaagaatttatta
ctttcatcctcatgtacactctctctttatcatttacatttaatatttattggtattctt
cttaccttttccccaatgcatttcttaggatttaatgttatgccaagaagaatcccatcc
tttccagattcttttcattcctggaattccctgtcatctattggatcaggaataactttc
ctatctttttctatg
>ohdo7_cox1
ataaatctcttaaccattaatttcctatttcattcactagttaaaaattgtaatcataaa
ggcttaggaatctattatttattatctggattcatctttggaatctccggtacattaata
tcagtccttataagaatagaattatattcttcaggaaataggattatatctccagaaaac
cagaacttctataatataagcattacattgcatggctttcttatgattttctttttagta
atgcctggcttgtttggaggatttggaaattattttgtacctatctttcaagggtctcca
gaagtggtatatcctagagtcaataatttttctatcttaattcttttgctttcatatctt
ttcctaatcctttctttaatctcagaatttggaggtggtacagggtggacgctctaccca
ccattatccacttcttttatgactttatcaccttcaagtacaggaaatcttatatttgga
ttaataatctctggtatatcttcatgtcttacatctcttaacttttggacaacaattcat
tttctgagatcttattatctgatattatcttctatcccattatttccttgggctttcttg
attacagctttcatgcttttattaacattaccaatcttatctggtacacttcttttaata
ttgggtgatcttcattctaatacacttttctttgatccaatatttggaggagatcctata
ttctatcaacatttattttggtttttggacatccagaagtttacatattaataattcct
gcatttgggatcatttccataataatttctggtattttacagttaataatctttgctaac
caatcaatgatctttgccatgtcatctatttctcttcttggaggtcttgtttggggacat
catatgtatactgtaggtttagaaagtgatacaagagcttattttacaggagttacaatc
ttaatatccttaccaactggtacaaaaatctttaattggctttttacatatctctcaaat
ccaccattattacaccttagaattacttctgtcttcctctcacatctctttttattaatg
tttacgataggtgggtcaacaggaataattcttggaaatggtgcagtggatctaggatta
catgatacatattatgttgtagcacattttcattttgttctttctttaggagctataatt
gctatcttctctggaataatcttgaatggagaaaagattgttgctactaagaatttatta
ctttcatcctcatgtacactctctctttatcatttacatttaatatttattggtattctt
cttaccttttccccaatgcatttcttaggatttaatgttatgccaagaagaatcccatcc
tttccagattcttttcattcctggaattccctgtcatctattggatcaggaataactttc
ctatctttttctatg
>sesoko1_cox1
ttaaatctcttaaccattaatttcctatttcattcactagttaaaaattgtaatcataaa
ggcttaggaatctattatttattatctggattcatctttggaatctccggtacattaata
tcagtccttataagaatagaattatattcttcaggaaataggattatatctccagaaaac
cagaacttctataatataagcattacattgcatggctttcttatgattttctttttagta
atgcctggcttgtttggaggatttggaaattattttgtacctatctttcaagggtctcca
gaagtggtatatcctagagtcaataatttttctatcttaattcttttgctttcatatctt
ttcctaatcctttctttaatctcagaatttggaggtggtacagggtggacgctctaccca
ccattatccacttcttttatgactttatcaccttcaagtacaggaaatcttatatttgga
ttaataatctctggtatatcttcatgtcttacatctcttaacttttggacaacaattcat
tttctgagatcttattatctgatattatcttctatcccattatttccttgggctttcttg
attacagctttcatgcttttattaacattaccaatcttatctggtacacttcttttaata
ttgggtgatcttcattctaatacacttttctttgatccaatatttggaggagatcctata
ttctatcaacatttattttggtttttggacatccagaagtttacatattaataattcct
gcatttgggatcatttccataataatttctggtattttacagttaataatctttgctaac
caatcaatgatctttgccatgtcatctatttctcttcttggaggtcttgtttggggacat
catatgtatactgtaggtttagaaagtgatacaagagcttattttacaggagttacaatc
ttaatatccttaccaactggtacaaaaatctttaattggctttttacatatctctcaaat
ccaccattattacaccttagaattacttctgtcttcctctcacatctctttttattaatg
tttacgataggtgggtcaacaggaataattcttggaaatggtgcagtggatctaggatta
catgatacatattatgttgtagcacattttcattttgttctttctttaggagctataatt
gctatcttctctggaataatcttgaatggagaaaagattgttgctactaagaatttatta
ctttcatcctcatgtacactctctctttatcatttacatttaatatttattggtattctt

cttaccttttccccaatgcatttcttaggatttaatgttatgccaagaagaatcccatcc
tttccagattcttttcattcctggaattccctgtcatctattggatcaggaataactttc
ctatctttttctatg
>sesoko2_cox1
ttaaatctcttaaccattaatttcctatttcattcactagttaaaaattgtaatcataaa
ggcttaggaatctattatttattatctggattcatctttggaatctccggtacattaata
tcagtccttataagaatagaattatattcttcaggaaataggattatatctccagaaaac
cagaacttctataatataagcattacattgcatggctttcttatgattttctttttagta
atgcctggcttgtttggaggatttggaaattattttgtacctatctttcaagggtctcca
gaagtggtatatcctagagtcaataatttttctatcttaattcttttgctttcatatctt
ttcctaatcctttctttaatctcagaatttggaggtggtacagggtggacgctctaccca
ccattatccacttcttttatgactttatcaccttcaagtacaggaaatcttatatttgga
ttaataatctctggtatatcttcatgtcttacatctcttaacttttggacaacaattcat
tttctgagatcttattatctgatattatcttctatcccattatttccttgggctttcttg
attacagctttcatgcttttattaacattaccaatcttatctggtacacttcttttaata
ttgggtgatcttcattctaatacacttttctttgatccaatatttggaggagatcctata
ttctatcaacatttattttggtttttggacatccagaagtttacatattaataattcct
gcatttgggatcatttccataataatttctggtattttacagttaataatctttgctaac
caatcaatgatctttgccatgtcatctatttctcttcttggaggtcttgtttggggacat
catatgtatactgtaggtttagaaagtgatacaagagcttattttacaggagttacaatc
ttaatatccttaccaactggtacaaaaatctttaattggctttttacatatctctcaaat
ccaccattattacaccttagaattacttctgtcttcctctcacatctctttttattaatg
tttacgataggtgggtcaacaggaataattcttggaaatggtgcagtggatctaggatta
catgatacatattatgttgtagcacattttcattttgttctttctttaggagctataatt
gctatcttctctggaataatcttgaatggagaaaagattgttgctactaagaatttatta
ctttcatcctcatgtacactctctctttatcatttacatttaatatttattggtattctt
cttaccttttccccaatgcatttcttaggatttaatgttatgccaagaagaatcccatcc
tttccagattcttttcattcctggaattccctgtcatctattggatcaggaataactttc
ctatctttttctatg
>sesoko3_cox1
ttaaatctcttaaccattaatttcctatttcattcactagttaaaaattgtaatcataaa
ggcttaggaatctattatttattatctggattcatctttggaatctccggtacattaata
tcagtccttataagaatagaattatattcttcaggaaataggattatatctccagaaaac
cagaacttctataatataagcattacattgcatggctttcttatgattttctttttagta
atgcctggcttgtttggaggatttggaaattattttgtacctatctttcaagggtctcca
gaagtggtatatcctagagtcaataatttttctatcttaattcttttgctttcatatctt
ttcctaatcctttctttaatctcagaatttggaggtggtacagggtggacgctctaccca
ccattatccacttcttttatgactttatcaccttcaagtacaggaaatcttatatttgga
ttaataatctctggtatatcttcatgtcttacatctcttaacttttggacaacaattcat
tttctgagatcttattatctgatattatcttctatcccattatttccttgggctttcttg
attacagctttcatgcttttattaacattaccaatcttatctggtacacttcttttaata
ttgggtgatcttcattctaatacacttttctttgatccaatatttggaggagatcctata
ttctatcaacatttattttggtttttggacatccagaagtttacatattaataattcct
gcatttgggatcatttccataataatttctggtattttacagttaataatctttgctaac
caatcaatgatctttgccatgtcatctatttctcttcttggaggtcttgtttggggacat
catatgtatactgtaggtttagaaagtgatacaagagcttattttacaggagttacaatc
ttaatatccttaccaactggtacaaaaatctttaattggctttttacatatctctcaaat
ccaccattattacaccttagaattacttctgtcttcctctcacatctctttttattaatg
tttacgataggtgggtcaacaggaataattcttggaaatggtgcagtggatctaggatta
catgatacatattatgttgtagcacattttcattttgttctttctttaggagctataatt
gctatcttctctggaataatcttgaatggagaaaagattgttgctactaagaatttatta
ctttcatcctcatgtacactctctctttatcatttacatttaatatttattggtattctt
cttaccttttccccaatgcatttcttaggatttaatgttatgccaagaagaatcccatcc
tttccagattcttttcattcctggaattccctgtcatctattggatcaggaataactttc
ctatctttttctatg
>sesoko4_cox1
ataaatctcttaaccattaatttcctatttcattcactagttaaaaattgtaatcataaa

ggcttaggaatctattatttattatctggattcatctttggaatctccggtacattaata
tcagtccttataagaatagaattatattcttcaggaaataggattatatctccagaaaac
cagaacttctataatataagcattacattgcatggctttcttatgattttctttttagta
atgcctggcttgtttggaggatttggaaattattttgtacctatctttcaagggtctcca
gaagtggtatatcctagagtcaataatttttctatcttaattcttttgctttcatatctt
ttcctaatcctttctttaatctcagaatttggaggtggtacagggtggacgctctaccca
ccattatccacttcttttatgactttatcaccttcaagtacaggaaatcttatatttgga
ttaataatctctggtatatcttcatgtcttacatctcttaacttttggacaacaattcat
tttctgagatcttattatctgatattatcttctatcccattatttccttgggctttcttg
attacagctttcatgcttttattaacattaccaatcttatctggtacacttcttttaata
ttgggtgatcttcattctaatacacttttctttgatccaatatttggaggagatcctata
ttctatcaacatttattttggtttttggacatccagaagtttacatattaataattcct
gcatttgggatcatttccataataatttctggtattttacagttaataatctttgctaac
caatcaatgatctttgccatgtcatctatttctcttcttggaggtcttgtttggggacat
catatgtatactgtaggtttagaaagtgatacaagagcttattttacaggagttacaatc
ttaatatccttaccaactggtacaaaaatctttaattggctttttacatatctctcaaat
ccaccattattacaccttagaattacttctgtcttcctctcacatctctttttattaatg
tttacgataggtgggtcaacaggaataattcttggaaatggtgcagtggatctaggatta
catgatacatattatgttgtagcacattttcattttgttctttctttaggagctataatt
gctatcttctctggaataatcttgaatggagaaaagattgttgctactaagaatttatta
ctttcatcctcatgtacactctctctttatcatttacatttaatatttattggtattctt
cttaccttttccccaatgcatttcttaggatttaatgttatgccaagaagaatcccatcc
tttccagattcttttcattcctggaattccctgtcatctattggatcaggaataactttc
ctatctttttctatg
>REF_DNA_Cox1_LC002801.1
ATAAATCTCTTAACCATTAATTTCCTATTTCATTCACTAGTTAAAAATTGTAATCATAAA
GGCTTAGGAATCTATTATTTATTATCTGGATTCATCTTTGGAATCTCCGGTACATTAATA
TCAGTCCTTATAAGAATAGAATTATATTCTTCAGGAAATAGGATTATATCTCCAGAAAAC
CAGAACTTCTATAATATAAGCATTACATTGCATGGCTTTCTTATGATTTTCTTTTTAGTA
ATGCCTGGCTTGTTTGGAGGATTTGGAAATTATTTTGTACCTATCTTTCAAGGGTCTCCA
GAAGTGGTATATCCTAGAGTCAATAATTTTTCTATCTTAATTCTTTTCCTTTCATATCTT
TTCCTAATCCTTTCTTTAATCTCAGAATTTGGAGGTGGTACAGGGTGGACGCTCTACCCA
CCATTATCCACTTCTTTTATGACTTTATCACCTTCAAGTACAGGAAATCTTATATTTGGA
TTAATAATCTCTGGTATATCTTCATCTCTTACATCTCTTAACTTTTGGACAACAATTCAT
TTTCTGAGATCTTATTATCTGATATTATCTTCTATCCCATTATTTCCTTGGGCTTTCTTG
ATTACAGCTTTCATGCTTTTATTAACATTACCAATCTTATCTGGTACACTTCTTTTAATA
TTGGGTGATCTTCATTCAAATACACTTTTCTTTGATCCAATATTTGGAGGAGATCCTATA
TTCTATCAACATTTATTTTGGTTTTTGGACATCCAGAAGTTTACATATTAATAATTCCT
GCATTTGGGATCATTTCCATAATAATTTCTGGGATTTTACAGTTAATAATCTTTGCTAAC
CAATCAATGATCTTTGCCATGTCATCTATTTCTCTTCTTGGAGGTCTTGTTTGGGGACAT
CATATGTATACTGTAGGTTTAGAAAGTGATACAAGAGCTTATTTTACAGGAGTTACAATC
TTAATATCCTTACCAACTGGTACAAAAATCTTTAATTGGCTTTTTACATATCTCTCCAAT
CCACCATTATTACACCTTAGAATTACTTCTGTCTTCCTCTCACATCTCTTTTTATTAATG
TTTACGGTAGGTGGGTCAACAGGAATAATTCTTGGAAATGGTGCAGTGGATCTAGGATTA
CATGATACATATTATGTTGTAGCACATTTTCATTTTGTTCTTTCTTTAGGAGCTATAATT
GCTATCTTCTCTGGAATAATCTTGAATGGAGAAAAGATTGTTGCTACTAAGAATTTATTA
CTTTCATCCTCATGTACACTCTCTCTTTATCATTTACATTTAATATTTATTGGTATTCTT
CTTACCTTTTCCCCAATGCATTTCTTAGGATTTAATCTTATGCCAAGAAGAATCCCATCC
TTTCCAGATTCTTTTCATTCCTGGAATTCCCTGTCATCTATTGGATCAGGAATAACTTTC
CTATCTTTTTCTACT
>REF_RNA_Cox1_symbB1.comp234_c0_seq1
ataaatctcttaaccattaatttcctatttcattcactagttaaaaattgtaatcataaa
cgcttaggaatctattatttattatctggattcatctttggaatctccggtacattaata
tcagtccttatgagaatagaattatattcttcaggaaataggattatatctccagaaaac
cagaacttctataatgtaagcattacattgcatggccttcttatgattttctttttagta
atgcctggcttgtttggaggatttggaaattattttgtacctatctttcaagggtctcca
gaagtggtatatcctagagtcaataatttttctatcttaattcttttcctttcatatctt

ttcctaatcctttctttaatctcagaatttggaggtggtacagggtggacgctctaccca
ccattatccacttcttttatgactttatcaccttcaagtgtaggaaatcttatatttgga
ttaataatctctggtatatcttcatctcttacatctcttaacttttgggtaacaattcat
tttctgagatcttattctctgatattatcttctgtcccattatttccttgggctttcttg
attacagctttcatgcttttattaacattaccagtcttatctggtacacttcttttagta
ttgggtgatcttcattcaaatacacttttctttgatccagtatttggaggagatcctgta
ctctatcaacatttattttggtttttggacatccagaagtttacatattaataattcct
gcatttggggtcatttccatagtaatttctggggtttcacagttaataatctttgctaac
caatcaatgatctttgccatgtcatctatttctcttcttggaggtcttgtttggggacat
catatgtatactgtaggtttagaaagtgatacaagagcttattttacaggagttacaatc
ttaatatccttaccaactggtacaaaaatctttaattggctttttacatatctctccaat
ccaccattattacaccttagaattacttctgtcttcctctcacatctctttttattaatg
tttacggtaggtgggtcaacaggagtaattcttggaaatggtgcagtggatctagcatta
catgatacatattatgttgtagcacattttcattttgttctttctttaggagctgtaatt
gctatcttctctggaatagtcttgaatggagaaaagattgttgctactaagagtttatta
ctttcatcctcatgtacactctctctttatcatttacatttagtatttgttggtattctt
cttaccttttccccaatgcatttcttaggatttaatcttatgccaagaagaatcccatcc
tttccagattcttttcattcctggaattccctgtcatctattggatcaggaataactctc
ctatctttttctatg

>Hd1_cox3
attaatacagaattattattttcttatctgtatctattggtattttggaaatcattacat
ttagaagttacatcagatttattttgttttcttattaatactcaccatttattaatcttt
gctcttattataattctatttatctttacaggatttaatatttattgttggactggaatt
tacttctcatagaattattttcttatattatttcatcttaattttggattaatttatagt
tttcatatttggtctagagatttattaagagaattcactaaaaaatatgaagtcttatta
ataatctttttcttctttttgggggttttcttgtttctgaagctctattatttgtatcc
ttcttttggacatcttttcatttattatcttctccaactttagggatgtgggtgtgggaa
ggtttctatctggaggatccttgtgaattaacttttgctaatacacttcttttatcaaat
gctgctatatctttaggaaatgcttttattaatttagaaatttcatcagaatatattatt
ttcttcactttatggtcattctttttgtcttctctttttattagtttgcagattaaagaa
tttcgcattctcgcattatcaattaatgattcactttatagttctcttttcttttttctt
acaggattacatttctttcatctatctattggtcttattcttctaatttatttctattat
gtgggtttctttgtttactttatgctttcatggacttcttttaataaaatat
>Hd2_cox3
attaatacagaattattattttcttatctgtatctattggtattttggaaatcattacat
ttagaagttacatcagatttattttgttttcttattaatactcaccatttattaatcttt
gctcttattataattctatttatctttacaggatttaatatttattgttggactggaatt
tacttctcatagaattattttcttatattatttcatcttaattttggattaatttatagt
tttcatatttggtctagagatttattaagagaattcactaaaaaatatgaagtcttatta
ataatctttttcttctttttgggggttttcttgtttctgaagctctattatttgtatcc
ttcttttggacatcttttcatttattatcttctccaactttagggatgtgggtgtgggaa
ggtttctatctggaggatccttgtgaattaacttttgctaatacacttcttttatcaaat
gctgctatatctttaggaaatgcttttattaatttagaaatttcatcagaatatattatt
ttcttcactttatggtcattctttttgtcttctctttttattagtttgcagattaaagaa
tttcgcattctcgcattatcaattaatgattcactttatagttctcttttcttttttctt
acaggattacatttctttcatctatctattggtcttattcttctaatttatttctattat
gtgggtttctttgtttactttatgctttcatggacttcttttaataaaatat
>Hd3_cox3
attaatacagaattattattttcttatctgtatctattggtattttggaaatcattacat
ttagaagttacatcagatttattttgttttcttattaatactcaccatttattaatcttt
gctcttattataattctatttatctttacaggatttaatatttattgttggactggaatt
tacttctcatagaattattttcttatattatttcatcttaattttggattaatttatagt
tttcatatttggtctagagatttattaagagaattcactaaaaaatatgaagtcttatta
ataatctttttcttctttttgggggttttcttgtttctgaagctctattatttgtatcc
ttcttttggacatcttttcatttattatcttctccaactttagggatgtgggtgtgggaa
ggtttctatctggaggatccttgtgaattaacttttgctaatacacttcttttatcaaat
gctgctatatctttaggaaatgcttttattaatttagaaatttcatcagaatatattatt
ttcttcactttatggtcattctttttgtcttctctttttattagtttgcagattaaagaa
tttcgcattctcgcattatcaattaatgattcactttatagttctcttttcttttttctt
acaggattacatttctttcatctatctattggtcttattcttctaatttatttctattat
gtgggtttctttgtttactttatgctttcatggacttcttttaataaaatat
>Hd4_cox3
attaatacagaattattattttcttatctgtatctattggtattttggaaatcattacat
ttagaagttacatcagatttattttgttttcttattaatactcaccatttattaatcttt
gctcttattataattctatttatctttacaggatttaatatttattgttggactggaatt
tacttctcatagaattattttcttatattatttcatcttaattttggattaatttatagt
tttcatatttggtctagagatttattaagagaattcactaaaaaatatgaagtcttatta
ataatctttttcttctttttgggggttttcttgtttctgaagctctattatttgtatcc
ttcttttggacatcttttcatttattatcttctccaactttagggatgtgggtgtgggaa
ggtttctatctggaggatccttgtgaattaacttttgctaatacacttcttttatcaaat
gctgctatatctttaggaaatgcttttattaatttagaaatttcatcagaatatattatt
ttcttcactttatggtcattctttttgtcttctctttttattagtttgcagattaaagaa
tttcgcattctcgcattatcaattaatgattcactttatagttctcttttcttttttctt
acaggattacatttctttcatctatctattggtcttattcttctaatttatttctattat
gtgggtttctttgtttactttatgctttcatggacttcttttaataaaatat
>Hd5_cox3

attaatacagaattattattttcttatctgtatctattggtattttggaaatcattacat
ttagaagttacatcagatttattttgttttcttattaatactcaccatttattaatcttt
gctcttattataattctatttatctttacaggatttaatatttattgttggactggaatt
tacttctcatagaattattttcttatattatttcatcttaattttggattaatttatagt
tttcatatttggtctagagatttattaagagaattcactaaaaaatatgaagtcttatta
ataatctttttttcttctttttgggggttttcttgtttctgaagctctattatttgtatcc
ttcttttggacatcttttcatttattatcttctccaacggctgggatgtgggtgtgggaa
ggtttctatctggaggatccttgtgaattaacttttgctaatacacttcttttatcaaat
gctgctatatctttaggaaatgcttttattaatttagaaatttcatcagaatatattatt
ttcttcactttatggtcattctttttgtcttctctttttattagtttgcagattaaagaa
tttcgcattctcgcattatcaattaatgattcactttatagttctcttttcttttttctt
acaggattacatttctttcatctatctattggtcttattcttctaatttatttctattat
gtgggtttctttgtttactttatgctttcatggacttcttttaataaaatat
>Hd6_cox3
attaatacagaattattattttcttatctgtatctattggtattttggaaatcattacat
ttagaagttacatcagatttattttgttttcttattaatactcaccatttattaatcttt
gctcttattataattctatttatctttacaggatttaatatttattgttggactggaatt
tacttctcatagaattattttcttatattatttcatcttaattttggattaatttatagt
tttcatatttggtctagagatttattaagagaattcactaaaaaatatgaagtcttatta
ataatctttttttcttctttttgggggttttcttgtttctgaagctctattatttgtatcc
ttcttttggacatcttttcatttattatcttctccaactttagggatgtgggtgtgggaa
ggtttctatctggaggatccttgtgaattaacttttgctaatacacttcttttatcaaat
gctgctatatctttaggaaatgcttttattaatttagaaatttcatcagaatatattatt
ttcttcactttatggtcattctttttgtcttctctttttattagtttgcagattaaagaa
tttcgcattctcgcattatcaattaatgattcactttatagttctcttttcttttttctt
acaggattacatttctttcatctatctattggtcttattcttctaatttatttctattat
gtgggtttctttgtttactttatgctttcatggacttcttttaataaaatat
>Hd7_cox3
attaatacagaattattattttattatctgtatctattggtattttggaaatcattacat
ttagaagttacatcagattcattttgttttcttattaatactcaccatttattaatcttt
gctcttattataattctatttatctttacaggatttaatatttattgttggactggaatt
tacttctcatagaattattttcttatattatttcatcttaattttggattaatttatagt
tttcatatttggtctagagatttattaagagaattcactaaaaaatatgaagtcttatta
ataatctttttttcttctttttgggggttttcttgtttctgaagctctattatttgtatcc
ttcttttggacatcttttcatttattatcttctccaactttagggatgtgggtgtgggaa
ggtttctatctggaggatccttgtgaattaacttttgctaatacacttcttttatcaaat
gctgctatatctttaggaagtgcttttattagtttagaaatttcatcagaatatattatt
ttcttcactttatggtcattctttttgtcttctctttttattagtttgcagattaaagaa
tttcgcattctcgcattatcaattaatgattcactttatagttctcttttcttttttctt
acaggattacatttctttcatctatctattggtcttattcttctaatttatttctattat
gtgggtttctttgtttactttatgctttcatggacttcttttaataaaatat
>Hd8_cox3
attaatacagaattattattttcttatctgtatctattggtattttggaaatcattacat
ttagaagttacatcagatttattttgttttcttattaatactcaccatttattaatcttt
gctcttattataattctatttatctttacaggatttaatatttattgttggactggaatt
tacttctcatagaattattttcttatattatttcatcttaattttggattaatttatagt
tttcatatttggtctagagatttattaagagaattcactaaaaaatatgaagtcttatta
ataatctttttttcttctttttgggggttttcttgtttctgaagctctattatttgtatcc
ttcttttggacatcttttcatttattatcttctccaactttagggatgtgggtgtgggaa
ggtttctatctggaggatccttgtgaattaacttttgctaatacacttcttttatcaaat
gctgctatatctttaggaaatgcttttattaatttagaaatttcatcagaatatattatt
ttcttcactttatggtcattctttttgtcttctctttttattagtttgcagattaaagaa
tttcgcattctcgcattatcaattaatgattcactttatagttctcttttcttttttctt
acaggattacatttctttcatctatctattggtcttattcttctaatttatttctattat
gtgggtttctttgtttactttatgctttcatggacttcttttaataaaatat
>Hd9_cox3
attaatacagaattattattttcttatctgtatctattggtattttggaaatcattacat

ttagaagttacatcagatttattttgttttcttattaatactcaccatttattaatcttt
gctcttattataattctatttatctttacaggatttaatatttattgttggactggaatt
tacttctcatagaattattttcttatattatttcatcttaattttggattaatttatagt
tttcatatttggtctagagatttattaagagaattcactaaaaaatatgaagtcttatta
ataatctttttcttctttttgggggttttcttgtttctgaagctctattatttgtatcc
ttcttttggacatcttttcatttattatcttctccaactttagggatgtgggtgtgggaa
ggtttctatctggaggatccttgtgaattaacttttgctaatacacttcttttatcaaat
gctgctatatctttaggaaatgcttttattaatttagaaatttcatcagaatatattatt
ttcttcactttatggtcattctttttgtcttctctttttattagtttgcagattaaagaa
tttcgcattctcgcattatcaattaatgattcactttatagttctcttttctttttctt
acaggattacatttctttcatctatctattggtcttattcttctaatttatttctattat
gtgggtttctttgtttactttatgctttcatggacttcttttaataaaatat
>IS1_cox3
attaatacagaattattattttattatctgtatctattggtattttggaaatcattacat
ttagaagttgcatcaggttcattttgttttctaattaatactcaccatttattttccttt
gctcttattataattctatttatctttacaggatttaatatttattgttgggctggtatt
tacttctcatataattattctcttatattatttcatcttaattttggattaatttatagt
tttcatatttggtctagagatttattaagagaattaagtaaaaaatatgaagtcttatta
ataatctttttattctttttggtggttttctagtttctgaagctctattatttgtatcc
ttcttttgggcatcttttcatttattatcttctccaacggctgggatgtggctgtgggaa
gctttctatctggaggatccttgtgaattaacttttgctaatacacttcttttatctaat
gctgctatatctttaggaaatgcttttattaatgtagaaatttcatcagaatatattatt
ttcttcgctttatggtcattctttttgtcttctctttttattagtttgcagattaaagaa
tttcgtattctcgcattatcaattaatgattcactttatagttctcttttctttttctt
acaggattacatttctttcatctatctattggtcttattcttctaatttatttctattat
gtgggtttctttgtttactttatgctttcatggacttcttttaataaaatat
>IS10_cox3
attaatacagaattattattttcttatctgtatctattggtattttggaaatcattacat
ttagaagttacatcagatttattttgttttcttattaatactcaccatttattaatcttt
gctcttattataattctatttatctttacaggatttaatatttattgttggactggaatt
tacttctcatagaattattttcttatattatttcatcttaattttggattaatttatagt
tttcatatttggtctagagatttattaagagaattcactaaaaaatatgaagtcttatta
ataatctttttcttctttttgggggttttcttgtttctgaagctctattatttgtatcc
ttcttttggacatcttttcatttattatcttctccaactttagggatgtgggtgtgggaa
ggtttctatctggaggatccttgtgaattaacttttgctaatacacttcttttatcaaat
gctgctatatctttaggaaatgcttttattaatttagaaatttcatcagaatatattatt
ttcttcactttatggtcattctttttgtcttctctttttattagtttgcagattaaagaa
tttcgcattctcgcattatcaattaatgattcactttatagttctcttttctttttctt
acaggattacatttctttcatctatctattggtcttattcttctaatttatttctattat
gtgggtttctttgtttactttatgctttcatggacttcttttaataaaatat
>IS2_cox3
attaatacagaattattattttcttatctgtatctattggtattttggaaatcattacat
ttagaagttacatcagatttattttgttttcttattaatactcaccatttattaatcttt
gctcttattataattctatttatctttacaggatttaatatttattgttggactggaatt
tacttctcatagaattattttcttatattatttcatcttaattttggattaatttatagt
tttcatatttggtctagagatttattaagagaattcactaaaaaatatgaagtcttatta
ataatctttttcttctttttgggggttttcttgtttctgaagctctattatttgtatcc
ttcttttggacatcttttcatttattatcttctccaactttagggatgtgggtgtgggaa
ggtttctatctggaggatccttgtgaattaacttttgctaatacacttcttttatcaaat
gctgctatatctttaggaaatgcttttattaatttagaaatttcatcagaatatattatt
ttcttcactttatggtcattctttttgtcttctctttttattagtttgcagattaaagaa
tttcgcattctcgcattatcaattaatgattcactttatagttctcttttctttttctt
acaggattacatttctttcatctatctattggtcttattcttctaatttatttctattat
gtgggtttctttgtttactttatgctttcatggacttcttttaataaaatat
>IS3_cox3
attaatacagaattattattttcttatctgtatctattggtattttggaaatcattacat
ttagaagttacatcagatttattttgttttcttattaatgctcactatttattaatcttt

gctcttgttgtaattctatttatctttacaggatttaatatttattgttgggctggaatt
tacttctcatggagttattctcttatattatttcatcttaattttggattaatttatagt
tttcatatttggtctagagatttattaagagaattcactaaaaaatatgaaatcttatta
ataatctttttattctttttggggggttttctagtttctgaagctctattatttgtatcc
ttcttttggacatcttttcatttattatcttctccaacggctgggatgtggctgtgggaa
gctttctatctggaggatccttgtgaattaacttttgctaatacacttcttttatcaaat
gctgctgtatctttaggaggtgcttttattagtttagaaatttcatcaggatatattatt
ttcttcgctttatggtcattctttttgtcttctctttttattagtttgcagattaaagaa
tttcgcgttctcgcattatcaattaatgattcactttatagttctcttttcttttttctt
acaggattacatttctttcatctatctattggtcttattcttctaatttatttctattat
gtgggtttctttgtttactttatgctttcatggacttcttttaataaaatat
>IS4_cox3
attaatacagaattattattttcttatctgtatctattggtattttggaaatcattacat
ttagaagttacatcagatttattttgttttcttattaatactcaccatttattaatcttt
gctcttattataattctatttatctttacaggatttaatatttattgttggactggtatt
tacttctcatagaattattttcttatattatttcatcttaattttggattaatttatagt
tttcatatttggtctagagatttattaagagaattcactaaaaaatatgaagtcttatta
ataatctttttcttctttttggggggttttcttgtttctgaagctctattatttgtatcc
ttcttttggacatcttttcatttattatcttctccaactttagggatgtgggtgtgggaa
ggtttctatctggaggatccttgtgaattaacttttgctaatacacttcttttatctaat
gctgctatatctttaggaaatgcttttattaatttagaaatttcatcagaatatattatt
ttcttcactttatggtcattctttttgtcttctctttttattagtttgcagattaaagaa
tttcgcattctcgcattatcaattaatgattcactttatagttctcttttcttttttctt
acaggattacatttctttcatctatctattggtcttattcttctaatttatttctattat
gtgggtttctttgtttactttatgctttcatggacttcttttaataaaatat
>IS5_cox3
attaatacagaattattattttcttatctgtatctattggtattttggaaatcattacat
ttagaagttacatcagatttattttgttttcttattaatactcaccatttattaatcttt
gctcttattataattctatttatctttacaggatttaatatttattgttggactggaatt
tacttctcatagaattattttcttatattatttcatcttaattttggattaatttatagt
tttcatatttggtctagagatttattaagagaattcactaaaaaatatgaagtcttatta
ataatctttttcttctttttggggggttttcttgtttctgaagctctattatttgtatcc
ttcttttggacatcttttcatttattatcttctccaactttagggatgtgggtgtgggaa
ggtttctatctggaggatccttgtgaattaacttttgctaatacacttcttttatcaaat
gctgctatatctttaggaaatgcttttattaatttagaaatttcatcagaatatattatt
ttcttcactttatggtcattctttttgtcttctctttttattagtttgcagattaaagaa
tttcgcattctcgcattatcaattaatgattcactttatagttctcttttcttttttctt
acaggattacatttctttcatctatctattggtcttattcttctaatttatttctattat
gtgggtttctttgtttactttatgctttcatggacttcttttaataaaatat
>IS6_cox3
attaatacagaattattattttcttatctgtatctattggtattttggaaatcattacat
ttagaagttacatcagatttattttgttttcttattaatactcaccatttattaatcttt
gctcttattataattctatttatctttacaggatttaatatttattgttggactggaatt
tacttctcatagaattattttcttatattatttcatcttaattttggattaatttatagt
tttcatatttggtctagagatttattaagagaattcactaaaaaatatgaagtcttatta
ataatctttttcttctttttggggggttttcttgtttctgaagctctattatttgtatcc
ttcttttggacatcttttcatttattatcttctccaactttagggatgtgggtgtgggaa
ggtttctatctggaggatccttgtgaattaacttttgctaatacacttcttttatcaaat
gctgctatatctttaggaaatgcttttattaatttagaaatttcatcagaatatattatt
ttcttcactttatggtcattctttttgtcttctctttttattagtttgcagattaaagaa
tttcgcattctcgcattatcaattaatgattcactttatagttctcttttcttttttctt
acaggattacatttctttcatctatctattggtcttattcttctaatttatttctattat
gtgggtttctttgtttactttatgctttcatggacttcttttaataaaatat
>IS7_cox3
attaatacagaattattattttcttatctatatctattggtattttggaaatcattacat
ttagaagttacatcagatttattttgttttctaattaatactcaccatttattaatcttt
gctcttattataattctatttatctttacaggatttaatatttattgttggactggaatt

tacttctcatagaattattttcttatattatttcatcttaattttggattaatttatagt
tttcatatttggtctagagatttattaagagaattcagtaaaaaatatgaagtcttatta
ataatctttttcttctttttggggggttttctagtttctgaagctctattatttgtatcc
ttcttttggacatcttttcatttattatcttctccaactttagggatgtgggtgtgggaa
ggtttctatctggaggatccttgtgaattaacttttgctaatacacttcttttatctaat
gctgctatatctttaggaaatgcttttattaatttagaaatttcatcagaatatattatt
ttcttcactttatggtcattctttttgtcttctctttttattagtttgcagattaaagaa
tttcgcattctcgcattatcaattaatgattcactttatagttctcttttcttttttctt
acaggattacatttctttcatctatctattggtcttcttcttctaatttatttctattat
gtgggtttctttgtttactttatgctttcatggacttcttttaataaaaatat
>IS8_cox3
attaatacagaattattattttcttatctgtatctattggtattttggaaatcattacat
ttagaagttacatcagatttattttgttttcttattaatactcaccatttattaatcttt
gctcttattataattctatttatctttacaggatttaatatttattgttggactggaatt
tacttctcatagaattattttcttatattatttcatcttaattttggattaatttatagt
tttcatatttggtctagagatttattaagagaattcactaaaaaatatgaagtcttatta
ataatctttttcttctttttggggggttttcttgtttctgaagctctattatttgtatcc
ttcttttggacatcttttcatttattatcttctccaactttagggatgtgggtgtgggaa
ggtttctatctggaggatccttgtgaattaacttttgctaatacacttcttttatcaaat
gctgctatatctttaggaaatgcttttattaatttagaaatttcatcagaatatattatt
ttcttcactttatggtcattctttttgtcttctctttttattagtttgcagattaaagaa
tttcgcattctcgcattatcaattaatgattcactttatagttctcttttcttttttctt
acaggattacatttctttcatctatctattggtcttattcttctaatttatttctattat
gtgggtttctttgtttactttatgctttcatggacttcttttaataaaaatat
>IS9_cox3
attaatacagaattattattttcttatctgtatctattggtattttggaaatcattacat
ttagaagttacatcagatttattttgttttcttattaatactcaccatttattaatcttt
gctcttattataattctatttatctttacaggatttaatatttattgttggactggaatt
tacttctcatataattattctcttatattatttcatcttaattttggattaatttatagt
tttcatatttggtctagagatttattaagagaattcactaaaaaatatgaagtcttatta
ataatctttttcttctttttggggggttttcttgtttctgaagctctattatttgtatcc
ttcttttggacatcttttcatttattatcttctccaacggctgggatgtggctgtgggaa
gctttctatctggaggatccttgtgaattaacttttgctaatacacttcttttatcaaat
gctgctatatctttaggaaatgcttttattaatttagaaatttcatcagaatatattatt
ttattcgctttatggtcattatttttgtcttctctttttattagtttgcagattaaagaa
tttcgcattctcgcattatcaattaatgattcactttatagttctcttttcttttttctt
acaggattacatttctttcatctatctattggtcttattcttctaatttatttctattat
gtgggtttctttgtttactttatgctttcatggacttcttttaataaaaatat
>Ik1_cox3
attaatacagaattattattttcttatctgtatctattggtattttggaaatcattacat
ttagaagttacatcagatttattttgttttcttattaatactcaccatttattaatcttt
gctcttattataattctatttatctttacaggatttaatatttattgttggactggaatt
tacttctcatagaattattttcttatattatttcatcttaattttggattaatttatagt
tttcatatttggtctagagatttattaagagaattcactaaaaaatatgaagtcttatta
ataatctttttcttctttttggggggttttcttgtttctgaagctctattatttgtatcc
ttcttttggacatcttttcatttattatcttctccaactttagggatgtgggtgtgggaa
ggtttctatctggaggatccttgtgaattaacttttgctaatacacttcttttatcaaat
gctgctatatctttaggaaatgcttttattaatttagaaatttcatcagaatatattatt
ttcttcactttatggtcattctttttgtcttctctttttattagtttgcagattaaagaa
tttcgcattctcgcattatcaattaatgattcactttatagttctcttttcttttttctt
acaggattacatttctttcatctatctattggtcttattcttctaatttatttctattat
gtgggtttctttgtttactttatgctttcatggacttcttttaataaaaatat
>Ik2_cox3
attaatacagaattattattttcttatctgtatctattggtattttggaaatcattacat
ttagaagttacatcagatttattttgttttcttattaatactcaccatttattaatcttt
gctcttattataattctatttatctttacaggatttaatatttattgttggactggaatt
tacttctcatagaattattctcttatattatttcatcttaattttggattaatttatagt

tttcatatttggtctagagatttattaagagaattcactaaaaaatatgaagtcttatta
ataatcttttttcttcttttggggggttttcttgtttctgaagctctattatttgtatcc
ttcttttggacatcttttcatttattatcttctccaacggcagggatgtgggtgtgggaa
ggtttctatctggaggatccttgtgaattaacttttgctaatacacttcttttatcaaat
gctgctatatctttaggaaatgcttttattaatttagaaatttcatcagaatatattatt
ttcttcactttatggtcattctttttgtcttctctttttattagtttgcagattaaagaa
tttcgcattctcgcattatcaattaatgattcactttatagttctcttttcttttttctt
acaggattacatttctttcatctatctattggtcttattcttctaatttatttctattat
gtgggtttctttgtttactttatgctttcatggacttcttttaataaaatat
>Ik3_cox3
attaatacagaattattattttcttatctgtatctattggtattttggaaatcattacat
ttagaagttacatcagatttattttgttttcttattaatactcaccatttattaatcttt
gctcttattataattctatttatctttacaggatttaatatttattgttggactggaatt
tacttctcatagaattattttcttatattatttcatcttaattttggattaatttatagt
tttcatatttggtctagagatttattaagagaattcactaaaaaatatgaagtcttatta
ataatcttttttcttcttttggggggttttcttgtttctgaagctctattatttgtatcc
ttcttttggacatcttttcatttattatcttctccaactttagggatgtgggtgtgggaa
ggtttctatctggaggatccttgtgaattaacttttgctaatacacttcttttatcaaat
gctgctatatctttaggaaatgcttttattaatttagaaatttcatcagaatatattatt
ttcttcactttatggtcattctttttgtcttctctttttattagtttgcagattaaagaa
tttcgcattctcgcattatcaattaatgattcactttatagttctcttttcttttttctt
acaggattacatttctttcatctatctattggtcttattcttctaatttatttctattat
gtgggtttctttgtttactttatgctttcatggacttcttttaataaaatat
>Ik4_cox3
attaatacagaattattattttcttatctgtatctattggtattttggaaatcattacat
ttagaagttacatcagatttattttgttttcttattaatactcaccatttattaatcttt
gctcttattataattctatttatctttacaggatttaatatttattgttggactggaatt
tacttctcatagaattattttcttatattatttcatcttaattttggattaatttatagt
tttcatatttggtctagagatttattaagagaattcactaaaaaatatgaagtcttatta
ataatcttttttcttcttttggggggttttcttgtttctgaagctctattatttgtatcc
ttcttttggacatcttttcatttattatcttctccaactttagggatgtgggtgtgggaa
ggtttctatctggaggatccttgtgaattaacttttgctaatacacttcttttatcaaat
gctgctatatctttaggaaatgcttttattaatttagaaatttcatcagaatatattatt
ttcttcactttatggtcattctttttgtcttctctttttattagtttgcagattaaagaa
tttcgcattctcgcattatcaattaatgattcactttatagttctcttttcttttttctt
acaggattacatttctttcatctatctattggtcttattcttctaatttatttctattat
gtgggtttctttgtttactttatgctttcatggacttcttttaataaaatat
>Ik5_cox3
attaatacagaattattattttcttatctgtatctattggtattttggaaatcattacat
ttagaagttacatcagatttattttgttttcttattaatactcaccatttattaatcttt
gctcttattataattctatttatctttacaggatttaatatttattgttggactggaatt
tacttctcatagaattattttcttatattatttcatcttaattttggattaatttatagt
tttcatatttggtctagagatttattaagagaattcactaaaaaatatgaagtcttatta
ataatcttttttcttcttttggggggttttcttgtttctgaagctctattatttgtatcc
ttcttttggacatcttttcatttattatcttctccaactttagggatgtgggtgtgggaa
ggtttctatctggaggatccttgtgaattaacttttgctaatacacttcttttatcaaat
gctgctatatctttaggaaatgcttttattaatttagaaatttcatcagaatatattatt
ttcttcactttatggtcattctttttgtcttctctttttattagtttgcagattaaagaa
tttcgcattctcgcattatcaattaatgattcactttatagttctcttttcttttttctt
acaggattacatttctttcatctatctattggtcttattcttctaatttatttctattat
gtgggtttctttgtttactttatgctttcatggacttcttttaataaaatat
>Ik6_cox3
attaatacagaattattattttcttatctgtatctattggtattttggaaatcattacat
ttagaagttacatcagatttattttgttttcttattaatactcaccatttattaatcttt
gctcttattataattctatttatctttacaggatttaatatttattgttggactggaatt
tacttctcatagaattattttcttatattatttcatcttaattttggattaatttatagt
tttcatatttggtctagagatttattaagagaattcactaaaaaatatgaagtcttatta

ataatctttttcttctttttggggggttttcttgtttctgaagctctattatttgtatcc
ttcttttggacatcttttcatttattatcttctccaacttcagggatgtgggtgtgggaa
ggtttctatctggaggatccttgtgaattaacttttgctaatacacttcttttatcaaat
gctgctatatctttaggaaatgcttttattaatttagaaatttcatcagaatatattatt
ttcttcactttatggtcattctttttgtcttctctttttattagtttgcagattaaagaa
tttcgcattctcgcattatcaattaatgattcactttatagttctcttttcttttttctt
acaggattacatttctttcatctatctattggtcttattcttctaatttatttctattat
gtgggtttctttgtttactttatgctttcatggacttcttttaataaaatat
>Ik7_cox3
attaatacagaattattattttcttatctgtatctattggtattttggaaatcattacat
ttagaagttacatcagatttattttgttttcttattaatactcaccatttattaatcttt
gctcttattataattctatttatctttacaggatttaatatttattgttggactggaatt
tacttctcatagaattattttcttatattatttcatcttaattttggattaatttatagt
tttcatatttggtctagagatttattaagagaattcactaaaaaatatgaagtcttatta
ataatctttttcttctttttggggggttttcttgtttctgaagctctattatttgtatcc
ttcttttggacatcttttcatttattatcttctccaactttagggatgtgggtgtgggaa
ggtttctatctggaggatccttgtgaattaacttttgctaatacacttcttttatcaaat
gctgctatatctttaggaaatgcttttattaatttagaaatttcatcagaatatattatt
ttcttcactttatggtcattctttttgtcttctctttttattagtttgcagattaaagaa
tttcgcattctcgcattatcaattaatgattcactttatagttctcttttcttttttctt
acaggattacatttctttcatctatctattggtcttattcttctaatttatttctattat
gtgggtttctttgtttactttatgctttcatggacttcttttaataaaatat
>Ik8_cox3
attaatacagaattattattttcttatctgtatctattggtattttggaaatcattacat
ttagaagttacatcagatttattttgttttcttattaatactcaccatttattaatcttt
gctcttattataattctatttatctttacaggatttaatatttattgttggactggaatt
tacttctcatagaattattttcttatattatttcatcttaattttggattaatttatagt
tttcatatttggtctagagatttattaagagaattcactaaaaaatatgaagtcttatta
ataatctttttcttctttttggggggttttcttgtttctgaagctctattatttgtatcc
ttcttttggacatcttttcatttattatcttctccaactttagggatgtgggtgtgggaa
ggtttctatctggaggatccttgtgaattaacttttgctaatacacttcttttatcaaat
gctgctatatctttaggaaatgcttttattaatttagaaatttcatcagaatatattatt
ttcttcactttatggtcattctttttgtcttctctttttattagtttgcagattaaagaa
tttcgcattctcgcattatcaattaatgattcactttatagttctcttttcttttttctt
acaggattacatttctttcatctatctattggtcttattcttctaatttatttctattat
gtgggtttctttgtttactttatgctttcatggacttcttttaataaaatat
>Ik9_cox3
attaatacagaattattattttcttatctgtatctattggtattttggaaatcattacat
ttagaagttacatcagatttattttgttttcttattaatactcaccatttattaatcttt
gctcttattataattctatttatctttacaggatttaatatttattgttggactggaatt
tacttctcatagaattattttcttatattatttcatcttaattttggattaatttatagt
tttcatatttggtctagagatttattaagagaattcactaaaaaatatgaagtcttatta
ataatctttttcttctttttggggggttttcttgtttctgaagctctattatttgtatcc
ttcttttggacatcttttcatttattatcttctccaactttagggatgtgggtgtgggaa
ggtttctatctggaggatccttgtgaattaacttttgctaatacacttcttttatcaaat
gctgctatatctttaggaaatgcttttattaatttagaaatttcatcagaatatattatt
ttcttcactttatggtcattctttttgtcttctctttttattagtttgcagattaaagaa
tttcgcattctcgcattatcaattaatgattcactttatagttctcttttcttttttctt
acaggattacatttctttcatctatctattggtcttattcttctaatttatttctattat
gtgggtttctttgtttactttatgctttcatggacttcttttaataaaatat
>Irm10_cox3
attaatacagaattattattttcttatctgtatctattggtattttggaaatcattacat
ttagaagttacatcagatttattttgttttcttattaatactcaccatttattaatcttt
gctcttattataattctatttatctttacaggatttaatatttattgttggactggaatt
tacttctcatagaattattttcttatattatttcatcttaattttggattaatttatagt
tttcatatttggtctagagatttattaagagaattcactaaaaaatatgaagtcttatta
ataatctttttcttctttttggggggttttcttgtttctgaagctctattatttgtatcc

ttcttttggacatcttttcatttattatcttctccaactttagggatgtgggtgtgggaa
ggtttctatctggaggatccttgtgaattaacttttgctaatacacttcttttatcaaat
gctgctatatctttaggaaatgcttttattaatttagaaatttcatcagaatatattatt
ttcttcactttatggtcattctttttgtcttctcttttattagtttgcagattaaagaa
tttcgcattctcgcattatcaattaatgattcactttatagttctcttttctttttctt
acaggattacatttctttcatctatctattggtcttattcttctaatttatttctattat
gtgggtttctttgtttactttatgctttcatggacttcttttaataaaatat
>Irm17_cox3
attaatacagaattattattttcttatctgtatctattggtattttggaaatcattacat
ttagaagttacatcagatttattttgttttcttattaatactcaccatttattaatcttt
gctcttattataattctatttatctttacaggatttaatatttattgttggactggaatt
tacttctcatagaattattttcttatattatttcatcttaattttggattaatttatagt
tttcatatttggtctagagatttattaagagaattcactaaaaaatatgaagtcttatta
ataatctttttcttctttttgggggttttcttgtttctgaagctctattatttgtatcc
ttcttttggacatcttttcatttattatcttctccaactttagggatgtgggtgtgggaa
ggtttctatctggaggatccttgtgaattaacttttgctaatacacttcttttatcaaat
gctgctatatctttaggaaatgcttttattaatttagaaatttcatcagaatatattatt
ttcttcactttatggtcattctttttgtcttctcttttattagtttgcagattaaagaa
tttcgcattctcgcattatcaattaatgattcactttatagttctcttttctttttctt
acaggattacatttctttcatctatctattggtcttattcttctaatttatttctattat
gtgggtttctttgtttactttatgctttcatggacttcttttaataaaatat
>Irm2_cox3
attaatacagaattattattttcttatctgtatctattggtattttggaaatcattacat
ttagaagttacatcagatttattttgttttcttattaatactcaccatttattaatcttt
gctcttattataattctatttatctttacaggatttaatatttattgttggactggaatt
tacttctcatagaattattttcttatattatttcatcttaattttggattaatttatagt
tttcatatttggtctagagatttattaagagaattcactaaaaaatatgaagtcttatta
ataatctttttcttctttttgggggttttcttgtttctgaagctctattatttgtatcc
ttcttttggacatcttttcatttattatcttctccaactttagggatgtgggtgtgggaa
ggtttctatctggaggatccttgtgaattaacttttgctaatacacttcttttatcaaat
gctgctatatctttaggaaatgcttttattaatttagaaatttcatcagaatatattatt
ttcttcactttatggtcattctttttgtcttctcttttattagtttgcagattaaagaa
tttcgcattctcgcattatcaattaatgattcactttatagttctcttttctttttctt
acaggattacatttctttcatctatctattggtcttattcttctaatttatttctattat
gtgggtttctttgtttactttatgctttcatggacttcttttaataaaatat
>Irm21_cox3
attaatacagaattattattttcttatctatatctattggtattttggaaatcattacat
ttagaagttacatcagatttattttgttttcttattaatactcaccatttattaatcttt
gctcttattataattctatttatctttacaggatttaatatttattgttggactggaatt
tacttctcatagaattattttcttatattatttcatcttaattttggattaatttatagt
tttcatatttggtctagagatttattaagagaattcactaaaaaatatgaagtcttatta
ataatctttttcttctttttgggggttttcttgtttctgaagctctattatttgtatcc
ttcttttggacatcttttcatttattatcttctccaactttagggatgtgggtgtgggaa
ggtttctatctggaggatccttgtgaattaacttttgctaatacacttcttttatcaaat
gctgctatatctttaggaaatgcttttattaatttagaaatttcatcagaatatattatt
ttcttcactttatggtcattctttttgtcttctcttttattagtttgcagattaaagaa
tttcgcattctcgcattatcaattaatgattcactttatagttctcttttctttttctt
acaggattacatttctttcatctatctattggtcttattcttctaatttatttctattat
gtgggtttctttgtttactttatgctttcatggacttcttttaataaaatat
>Irm22_cox3
attaatacagaattattattttcttatctgtatctattggtattttggaaatcattacat
ttagaagttacatcagatttattttgttttcttattaatactcaccatttattaatcttt
gctcttattataattctatttatctttacaggatttaatatttattgttggactggaatt
tacttctcatggagttattctcttatattatttcatcttaattttggattaatttatagt
tttcatatttggtctagagatttattaagagaattcactaaaaaatatgaaatcttatta
ataatctttttcttctttttgggggttttcttgtttctgaagctctattatttgtatcc
ttcttttggacatcttttcatttattatcttctccaacggctgggatgtggctgtgggaa

gctttctatctggaggatccttgtgaattaacttttgctaatacacttcttttatcaaat
gctgctgtatctttaggaggtgcttttattagtttagaaatttcatcagaatatattatt
ttcttcactttatggtcattctttttgtcttctcttttattagtttgcagattaaagaa
tttcgcattctcgcattatcaattaatgattcactttatagttctcttttcttttttctt
acaggattacatttctttcatctatctattggtcttattcttctaatttatttctattat
gtgggtttctttgtttactttatgctttcatggacttcttttaataaaatat
>Irm23_cox3
attaatacagaattattattttcttatctgtatctattggtattttggaaatcattacat
ttagaagttacatcagatttattttgttttcttattaatactcaccatttattaatcttt
gctcttattataattctatttatctttacaggatttaatatttattgttggactggaatt
tacttctcatagaattattttcttatattatttcatcttaattttggattaatttatagt
tttcatatttggtctagagatttattaagagaattcactaaaaaatatgaagtcttatta
ataatctttttcttctttttggggggttttcttgtttctgaagctctattatttgtatcc
ttcttttggacatcttttcatttattatcttctccaactttagggatgtgggtgtgggaa
ggtttctatctggaggatccttgtgaattaacttttgctaatacacttcttttatcaaat
gctgctatatctttaggaaatgcttttattaatttagaaatttcatcagaatatattatt
ttcttcactttatggtcattctttttgtcttctcttttattagtttgcagattaaagaa
tttcgcattctcgcattatcaattaatgattcactttatagttctcttttcttttttctt
acaggattacatttctttcatctatctattggtcttattcttctaatttatttctattat
gtgggtttctttgtttactttatgctttcatggacttcttttaataaaatat
>Irm24_cox3
attaatacagaattattattttcttatctgtatctattggtattttggaaatcattacat
ttagaagttacatcagatttattttgttttcttattaatactcactatttattaatcttt
gctcttgttgtaattctatttatctttacaggatttaatatttattgttggactggaatt
tacttctcatagaattattttcttatattatttcatcttaattttggattaatttatagt
tttcatatttggtctagagatttattaagagaattcactaaaaaaatatgaagtcttatta
ataatctttttcttctttttggggggttttcttgtttctgaagctctattatttgtatcc
ttcttttggacatcttttcatttattatcttctccaacggctgggatgtgggtgtgggaa
ggtttctatctggaggatccttgtgaattaacttttgctaatacacttcttttatcaaat
gctgctatatctttaggaaatgcttttattaatttagaaatttcatcagaatatattatt
ttcttcactttatggtcattctttttgtcttctcttttattagtttgcagattaaagaa
tttcgcgttctcgcattatcaattaatgattcactttatagttctcttttcttttttctt
acaggattacatttctttcatctatctattggtcttattcttctaatttatttctattat
gtgggtttctttgtttactttatgctttcatggacttcttttaataaaatat
>Irm25_cox3
attaatacagaattattattttcttatctgtatctattggtattttggaaatcattacat
ttagaagttacatcagatttattttgttttcttattaatactcaccatttattaatcttt
gctcttattataattctatttatctttacaggatttaatatttattgttggactggaatt
tacttctcatagaattattttcttatattatttcatcttaattttggattaatttatagt
tttcatatttggtctagagatttattaagagaattcactaaaaaaatatgaagtcttatta
ataatctttttcttctttttggggggttttcttgtttctgaagctctattatttgtatcc
ttcttttggacatcttttcatttattatcttctccaactttagggatgtgggtgtgggaa
ggtttctatctggaggatccttgtgaattaacttttgctaatacacttcttttatcaaat
gctgctatatctttaggaaatgcttttattaatttagaaatttcatcagaatatattatt
ttcttcactttatggtcattctttttgtcttctcttttattagtttgcagattaaagaa
tttcgcattctcgcattatcaattaatgattcactttatagttctcttttcttttttctt
acaggattacatttctttcatctatctattggtcttattcttctaatttatttctattat
gtgggtttctttgtttactttatgctttcatggacttcttttaataaaatat
>Irm26_cox3
attaatacagaattattattttcttatctgtatctattggtattttggaaatcattacat
ttagaagttacatcagatttattttgttttcttattaatactcaccatttattaatcttt
gctcttattataattctatttatctttacaggatttaatatttattgttggactggaatt
tacttctcatagaattattttcttatattatttcatcttaattttggattaatttatagt
tttcatatttggtctagagatttattaagagaattcactaaaaaaatatgaaatcttatta
ataatctttttcttctttttggggggttttcttgtttctgaagctctattatttgtatcc
ttcttttggacatcttttcatttattatcttctccaactttagggatgtgggtgtgggaa
ggtttctatctggaggatccttgtgaattaacttttgctaatacacttcttttatcaaat

gctgctatatctttaggaaatgcttttattaatttagaaatttcatcagaatatattatt
ttcttcactttatggtcattctttttgtcttctctttttattagtttgcagattaaagaa
tttcgcattctcgcattatcaattaatgattcactttatagttctcttttcttttttctt
acaggattacatttctttcatctatctattggtcttattcttctaatttatttctattat
gtgggtttctttgtttactttatgctttcatggacttcttttaataaaatat
>Irm27_cox3
attaatacagaattattattttattatctgtatctattggtattttggaaatcattacat
ttagaagttacatcagattaattttgttttctaattaatactcaccatttattttccttt
gctcttattataattctatttatctttacaggatttaatatttattgttgggctggaatt
tacttctcatggagttattctcttatattatttcatcttaattttggattaatttatagt
tttcatatttggtctagagatttattaagagaattcactaaaaaatatgaaatcttatta
atggtcctttttcttctttttgggggttttctagtttctgaagctctattatttgtatcc
ttcttttgggcatcttttcatttattatcttctccaacggctgggatgtggctgtgggaa
gctttctatctggaggatccttgtgaattaacttttgctaatacacttcttttatcaaat
gctgctgtatctttaggaggtgcttttattagtttagaaatttcatcaggatatattatt
ttcttcgctttatggtcattctttttgtcttctctttttattagtttgcagattaaagaa
tttcgcgttctcgcattatcaattaatgattcactttatagttctcttttcttttttctt
acaggattacatttctttcatctatctattggtcttattcttctaatttatttctattat
gtgggtttctttgtttactttatgctttcatggacttcttttaataaaatat
>Irm3_cox3
attaatacagaattattattttcttatctgtatctattggtattttggaaatcattacat
ttagaagttacatcagattaattttgttttctaattaatactcaccatttattttccttt
gctcttattataattctatttatctttacaggatttaatatttattgttggactggtatt
tacttctcatataattattctcttatattatttcatcttaattttggattaatttatagt
tttcatatttggtctagagatttattaagagaattcactaaaaaatatgaaatcttatta
atggtcctttttcttctttttgggggttttctagtttctgaagctctattatttgtatcc
ttcttttggacatcttttcatttattatcttctccaactttagggatgtggctgtgggaa
gctttctatctggaggatccttgtgaattaacttttgctaatacacttcttttatctaat
gctgctatatctttaggaaatgcttttattaatgtagaaatttcatcagaatatattatt
ttcttcactttatggtcattctttttgtcttctctttttattagtttgcagattaaagaa
tttcgtattctcgcattatcaattaatgattcactttatagttctcttttcttttttctt
acaggattacatttctttcatctatctattggtcttcttcttctaatttatttctattat
gtgggtttctttgtttactttatgctttcatggacttcttttaataaaatat
>Irm4_cox3
attaatacagaattattattttcttatctgtatctattggtattttggaaatcattacat
ttagaagttacatcagatttattttgttttctaattaatactcaccatttattaatcttt
gctcttattataattctatttatctttacaggatttaatatttattgttggactggtatt
tacttctcatataattattttcttatattatttcatcttaatttaggattaatttatagt
tttaatatttggtctagagatttattaagagaattaagtaaaaaatatgaagtcttatta
ataatctttttattctttttgggggttttcttgtttctgaagctctattatttgtatcc
ttcttttggacatcttttcatttattatcttctccaactttagggatgtgggtgtgggaa
ggtttctatctggaggatccttgtgaattaacttttgctaatacacttcttttatcaaat
gctgctatatctttaggaaatgcttttattaatgtagaaatttcatcagaatatattatt
ttcttcactttatggtcattatttttgtcttctctttttattagtttgcagattaaagaa
tttcgtattctcgcattatcaattaatgattcactttatagttctcttttcttttttctt
acaggattacatttctttcatctatctattggtcttattcttctaatttatttctattat
gtgggtttctttgtttactttatgctttcatggacttcttttaataaaatat
>Irm5_cox3
attaatacagaattattattttcttatctgtatctattggtattttggaaatcattacat
ttagaagttacatcagatttattttgttttcttattaatactcaccatttattaatcttt
gctcttattataattctatttatctttacaggatttaatatttattgttggactggaatt
tacttctcatagaattattttcttatattatttcatcttaattttggattaatttatagt
tttcatatttggtctagagatttattaagagaattcactaaaaaatatgaagtcttatta
ataatctttttcttctttttgggggttttcttgtttctgaagctctattatttgtatcc
ttcttttggacatcttttcatttattatcttctccaactttagggatgtgggtgtgggaa
ggtttctatctggaggatccttgtgaattaacttttgctaatacacttcttttatcaaat
gctgctatatctttaggaaatgcttttattaatttagaaatttcatcagaatatattatt

ttcttcactttatggtcattctttttgtcttctcttttattagtttgcagattaaagaa
tttcgcattctcgcattatcaattaatgattcactttatagttctcttttctttttctt
acaggattacatttctttcatctatctattggtcttattcttctaatttatttctattat
gtgggtttctttgtttactttatgctttcatggacttcttttaataaaatat
>Irm7_cox3
attaatacagaattattattttcttatctgtatctattggtattttggaaatcattacat
ttagaagttacatcagatttattttgttttcttattaatactcaccatttattaatcttt
gctcttattataattctatttatctttacaggatttaatatttattgttggactggaatt
tacttctcatagaattattttcttatattatttcatcttaattttggattaatttatagt
tttcatatttggtctagagatttattaagagaattcactaaaaaaatatgaagtcttatta
ataatctttttcttctttttggggggttttcttgtttctgaagctctattatttgtatcc
ttcttttggacatcttttcatttattatcttctccaactttagggatgtgggtgtgggaa
ggtttctatctggaggatccttgtgaattaacttttgctaatacacttcttttatctaat
gctgctatatctttaggaaatgcttttattaatttagaaatttcatcagaatatattatt
ttcttcactttatggtcattctttttgtcttctcttttattagtttgcagattaaagaa
tttcgcattctcgcattatcaattaatgattcactttatagttctcttttctttttctt
acaggattacatttctttcatctatctattggtcttattcttctaatttatttctattat
gtgggtttctttgtttactttatgctttcatggacttcttttaataaaatat
>Irm9_cox3
attaatacagaattattattttcttatctgtatctattggtattttggaaatcattacat
ttagaagttacatcagatttattttgttttctaattaatactcaccatttattttccttt
gctcttattataattctatttatctttacaggatttaatatttattgttggactggtatt
tacttctcatataattattctcttatattatttcatcttaatttaggattaattccttat
tttaatatttggtctagagatttattaagagaattaagtaaaaaaatatgaagtcttatta
atggtctttttattctttttggggggttttctagtttctgaagctctattatttgtatcc
ttcttttggacatcttttcatttattatcttctccaactttagggatgtggctgtgggaa
gctttctatctggaggatccttgtgaattaacttttgctaatacacttcttttatctaat
gctgctgtatctttaggaggtgcttttattagtttagaaatttcatcaggatatattatt
ttcttcgctttatggtcattctttttgtcttctcttttattagtttgcagattaaagaa
tttcgtattctcgcattatcaattaatgattcactttatagttctcttttctttttctt
acaggattacatttctttcatctatctattggtcttcttcttctaatttatttctattat
gtgggtttctttgtttactttatgctttcatggacttcttttaataaaatat
>Isy12_cox3
attaatacagaattattattttcttatctatatctattggtattttggaaatcattacat
ttagaagttacatcagatttattttgttttcttattaatactcaccatttattaatcttt
gctcttattataattctatttatctttacaggatttaatatttattgttggactggaatt
tacttctcatagaattattttcttatattatttcatcttaattttggattaatttatagt
tttcatatttggtctagagatttattaagagaattcactaaaaaaatatgaagtcttatta
ataatctttttcttctttttggggggttttctagtttctgaagctctattatttgtatcc
ttcttttggacatcttttcatttattatcttctccaactttagggatgtgggtgtgggaa
ggtttctatctggaggatccttgtgaattaacttttgctaatacacttcttttatcaaat
gctgctatatctttaggaaatgcttttattaatttagaaatttcatcagaatatattatt
ttcttcactttatggtcattctttttgtcttctcttttattagtttgcagattaaagaa
tttcgcattctcgcattatcaattaatgattcactttatagttctcttttctttttctt
acaggattacatttctttcatctatctattggtcttattcttctaatttatttctattat
gtgggtttctttgtttactttatgctttcatggacttcttttaataaaatat
>Isy15_cox3
attaatacagaattattattttcttatctgtatctattggtattttggaaatcattacat
ttagaagttacatcagattaattttgttttctaattaatactcaccatttattttacttt
gctcttattataattctatttatctttacaggatttaatatttattgttggactggtatt
tacttctcatataattattctcttatattatttcatcttaatttaggattaattccttat
tttaatatttggtctagagatttattaagagaattcagtaaaaaaatatgaagtcttatta
ataatctttttattctttttggggggttttctagtttctgaagctctattatttgtatcc
ttcttttggacatcttttcatttattatcttctccaactttagggatgtgggtgtgggaa
gctttctatctggaggatccttgtgaattaacttttgctaatacacttcttttatctaat
gctgctatatctttaggaaatgcttttattaatgtagaaatttcatcagaatatattatt
ttcttcactttatggtcattatttttgtcttctcttttattagtttgcagattaaagaa

tttcgtattctcgcattatcaattaatgattcactttatagttctcttttcttttttctt
acaggattacatttctttcatctatctattggtcttcttcttctaatttatttctattat
gtgggtttctttgtttactttatgctttcatggacttcttttaataaaatat
>Isy16_cox3
attaatacagaattattattttcttatctgtatctattggtattttggaaatcattacat
ttagaagttacatcagatttattttgttttcttattaatactcaccatttattaatcttt
gctcttattataattctatttatctttacaggatttaatatttattgttggactggaatt
tacttctcatagaattattttcttatattatttcatcttaattttggattaatttatagt
tttcatatttggtctagagatttattaagagaattcactaaaaaatatgaagtcttatta
ataatctttttcttctttttgggggttttcttgtttctgaagctctattatttgtatcc
ttcttttggacatcttttcatttattatcttctccaactttagggatgtgggtgtgggaa
ggtttctatctggaggatccttgtgaattaacttttgctaatacacttcttttatcaaat
gctgctatatctttaggaaatgcttttattaatttagaaatttcatcagaatatattatt
ttcttcactttatggtcattctttttgtcttctctttttattagtttgcagattaaagaa
tttcgcattctcgcattatcaattaatgattcactttatagttctcttttcttttttctt
acaggattacatttctttcatctatctattggtcttattcttctaatttatttctattat
gtgggtttctttgtttactttatgctttcatggacttcttttaataaaatat
>Isy17_cox3
attaatacagaattattattttcttatctatatctattggtattttggaaatcattacat
ttagaagttacatcagatttattttgttttcttattaatactcaccatttattaatcttt
gctcttattataattctatttatctttacaggatttaatatttattgttggactggaatt
tacttctcatagaattattttcttatattatttcatcttaattttggattaatttatagt
tttcatatttggtctagagatttattaagagaattcactaaaaaatatgaagtcttatta
ataatctttttcttctttttgggggttttcttgtttctgaagctctattatttgtatcc
ttcttttggacatcttttcatttattatcttctccaactttagggatgtgggtgtgggaa
ggtttctatctggaggatccttgtgaattaacttttgctaatacacttcttttatcaaat
gctgctatatctttaggaaatgcttttattaatttagaaatttcatcagaatatattatt
ttcttcactttatggtcattctttttgtcttctctttttattagtttgcagattaaagaa
tttcgcattctcgcattatcaattaatgattcactttatagttctcttttcttttttctt
acaggattacatttctttcatctatctattggtcttattcttctaatttatttctattat
gtgggtttctttgtttactttatgctttcatggacttcttttaataaaatat
>Isy18_cox3
attaatacagaattattattttcttatctgtatctattggtattttggaaatcattacat
ttagaagttacatcagatttattttgttttcttattaatactcaccatttattaatcttt
gctcttattataattctatttatctttacaggatttaatatttattgttggactggaatt
tacttctcatagaattattttcttatattatttcatcttaattttggattaatttatagt
tttcatatttggtctagagatttattaagagaattcactaaaaaatatgaagtcttatta
atggtcctttttcttctttttgggggttttctagtttctgaagctctattatttgtatcc
ttcttttggacatcttttcatttattatcttctccaactttagggatgtgggtgtgggaa
ggtttctatctggaggatccttgtgaattaacttttgctaatacacttcttttatcaaat
gctgctatatctttaggaaatgcttttattaatttagaaatttcatcagaatatattatt
ttcttcactttatggtcattctttttgtcttctctttttattagtttgcagattaaagaa
tttcgcattctcgcattatcaattaatgattcactttatagttctcttttcttttttctt
acaggattacatttctttcatctatctattggtcttattcttctaatttatttctattat
gtgggtttctttgtttactttatgctttcatggacttcttttaataaaatat
>Isy21_cox3
attaatacagaattattattttcttatctatatctattggtattttggaaatcattacat
ttagaagttgcatcagatttattttgttttcttattaatactcaccatttattaatcttt
gctcttattataattctatttatctttacaggatttaatatttattgttggactggaatt
tacttctcatagaattattttcttatattatttcatcttaattttggattaatttatagt
tttcatatttggtctagagatttattaagagaattcactaaaaaatatgaagtcttatta
ataatctttttcttctttttgggggttttctagtttctgaagctctattatttgtatcc
ttcttttggacatcttttcatttattatcttctccaactttagggatgtgggtgtgggaa
ggtttctatctggaggatccttgtgaattaacttttgctaatacacttcttttatcaaat
gctgctatatctttaggaaatgcttttattaatttagaaatttcatcagaatatattatt
ttcttcactttatggtcattctttttgtcttctctttttattagtttgcagattaaagaa
tttcgcattctcgcattatcaattaatgattcactttatagttctcttttcttttttctt

acaggattacatttctttcatctatctattggtcttattcttctaatttatttctattat
gtgggtttctttgtttactttatgctttcatggacttcttttaataaaatat
>Isy22_cox3
attaatacagaattattatttcttatctgtatctattggtattttggaaatcattacat
ttagaagttacatcagatttattttgttttcttattaatactcaccatttattaatcttt
gctcttattataattctatttatctttacaggatttaatatttattgttggactggaatt
tacttctcatagaattattttcttatattatttcatcttaattttggattaatttatagt
tttcatatttggtctagagatttattaagagagaattcactaaaaaaatatgaagtcttatta
ataatctttttcttctttttggggggttttcttgtttctgaagctctattatttgtatcc
ttcttttggacatcttttcatttattatcttctccaactttagggatgtgggtgtgggaa
ggtttctatctggaggatccttgtgaattaacttttgctaatacacttcttttatcaaat
gctgctatatctttaggaaatgcttttattaatttagaaatttcatcagaatatattatt
ttcttcactttatggtcattctttttgtcttctcttttattagtttgcagattaaagaa
tttcgcattctcgcattatcaattaatgattcactttatagttctcttttcttttttctt
acaggattacatttctttcatctatctattggtcttattcttctaatttatttctattat
gtgggtttctttgtttactttatgctttcatggacttcttttaataaaatat
>Isy23_cox3
attaatacagaattattatttcttatctatatctattggtattttggaaatcattacat
ttagaagttacatcagatttattttgttttcttattaatactcaccatttattaatcttt
gctcttattataattctatttatctttacaggatttaatatttattgttggactggaatt
tacttctcatagaattattttcttatattatttcatcttaattttggattaatttatagt
tttcatatttggtctagagatttattaagagagaattcactaaaaaaatatgaagtcttatta
ataatctttttcttctttttggggggttttcttgtttctgaagctctattatttgtatcc
ttcttttggacatcttttcatttattatcttctccaactttagggatgtgggtgtgggaa
ggtttctatctggaggatccttgtgaattaacttttgctaatacacttcttttatcaaat
gctgctatatctttaggaaatgcttttattaatttagaaatttcatcagaatatattatt
ttcttcactttatggtcattctttttgtcttctcttttattagtttgcagattaaagaa
tttcgcattctcgcattatcaattaatgattcactttatagttctcttttcttttttctt
acaggattacatttctttcatctatctattggtcttattcttctaatttatttctattat
gtgggtttctttgtttactttatgctttcatggacttcttttaataaaatat
>Isy24_cox3
attaatacagaattattatttcttatctgtatctattggtattttggaaatcattacat
ttagaagttacatcagatttattttgttttcttattaatactcaccatttattaatcttt
gctcttattataattctatttatctttacaggatttaatatttattgttggactggaatt
tacttctcatagaattattttcttatattatttcatcttaattttggattaatttatagt
tttcatatttggtctagagatttattaagagagaattcactaaaaaaatatgaagtcttatta
ataatctttttcttctttttggggggttttcttgtttctgaagctctattatttgtatcc
ttcttttggacatcttttcatttattatcttctccaactttagggatgtgggtgtgggaa
ggtttctatctggaggatccttgtgaattaacttttgctaatacacttcttttatcaaat
gctgctatatctttaggaaatgcttttattaatttagaaatttcatcagaatatattatt
ttcttcactttatggtcattctttttgtcttctcttttattagtttgcagattaaagaa
tttcgcattctcgcattatcaattaatgattcactttatagttctcttttcttttttctt
acaggattacatttctttcatctatctattggtcttattcttctaatttatttctattat
gtgggtttctttgtttactttatgctttcatggacttcttttaataaaatat
>Isy25_cox3
attaatacagaattattatttcttatctatatctattggtattttggaaatcattacat
ttagaagttacatcagatttattttgttttcttattaatactcaccatttattaatcttt
gctcttattataattctatttatctttacaggatttaatatttattgttggactggaatt
tacttctcatagaattattttcttatattatttcatcttaattttggattaatttatagt
tttcatatttggtctagagatttattaagagagaattcactaaaaaaatatgaagtcttatta
ataatctttttcttctttttggggggttttctagtttctgaagctctattatttgtatcc
ttcttttggacatcttttcatttattatcttctccaactttagggatgtgggtgtgggaa
ggtttctatctggaggatccttgtgaattaacttttgctaatacacttcttttatcaaat
gctgctatatctttaggaaatgcttttattaatttagaaatttcatcagaatatattatt
ttcttcactttatggtcattctttttgtcttctcttttattagtttgcagattaaagaa
tttcgcattctcgcattatcaattaatgattcactttatagttctcttttcttttttctt
acaggattacatttctttcatctatctattggtcttattcttctaatttatttctattat

gtgggtttctttgtttactttatgctttcatggacttcttttaataaaatat
>Isy26_cox3
attaatacagaattattatttcttatctatatctattggtattttggaaatcattacat
ttagaagttacatcagatttattttgttttcttattaatactcaccatttattaatcttt
gctcttattataattctatttatctttacaggatttaatatttattgttggactggaatt
tacttctcatagaattattttcttatattatttcatcttaattttggattaatttatagt
tttcatatttggtctagagatttattaagagaattcactaaaaaatatgaagtcttatta
ataatcttttttcttcttttgggggttttctagtttctgaagctctattatttgtatcc
ttcttttggacatcttttcatttattatcttctccaactttagggatgtgggtgtgggaa
ggtttctatctggaggatccttgtgaattaacttttgctaatacacttcttttatcaaat
gctgctatatctttaggaaatgcttttattaatttagaaatttcatcagaatatattatt
ttcttcactttatggtcattctttttgtcttctcttttattagtttgcagattaaagaa
tttcgcattctcgcattatcaattaatgattcactttatagttctcttttctttttctt
acaggattacatttctttcatctatctattggtcttattcttctaatttatttctattat
gtgggtttctttgtttactttatgctttcatggacttcttttaataaaatat
>Isy27_cox3
attaatacagaattattatttcttatctatatctattggtattttggaaatcattacat
ttagaagttacatcagatttattttgttttcttattaatactcaccatttattaatcttt
gctcttattataattctatttatctttacaggatttaatatttattgttggactggaatt
tacttctcatagaattattttcttatattatttcatcttaattttggattaatttatagt
tttcatatttggtctagagatttattaagagaattcactaaaaaatatgaagtcttatta
ataatcttttttcttcttttgggggttttcttgtttctgaagctctattatttgtatcc
ttcttttggacatcttttcatttattatcttctccaactttagggatgtgggtgtgggaa
ggtttctatctggaggatccttgtgaattaacttttgctaatacacttcttttatcaaat
gctgctatatctttaggaaatgcttttattaatttagaaatttcatcagaatatattatt
ttcttcactttatggtcattctttttgtcttctcttttattagtttgcagattaaagaa
tttcgcattctcgcattatcaattaatgattcactttatagttctcttttctttttctt
acaggattacatttctttcatctatctattggtcttattcttctaatttatttctattat
gtgggtttctttgtttactttatgctttcatggacttcttttaataaaatat
>Isy4_cox3
attaatacagaattattatttcttatctgtatctattggtattttggaaatcattacat
ttagaagttacatcagatttattttgttttcttattaatactcaccatttattaatcttt
gctcttattataattctatttatctttacaggatttaatatttattgttggactggaatt
tacttctcatagaattattttcttatattatttcatcttaattttggattaatttatagt
tttcatatttggtctagagatttattaagagaattcactaaaaaatatgaagtcttatta
ataatcttttttcttcttttgggggttttcttgtttctgaagctctattatttgtatcc
ttcttttggacatcttttcatttattatcttctccaactttagggatgtgggtgtgggaa
ggtttctatctggaggatccttgtgaattaacttttgctaatacacttcttttatcaaat
gctgctatatctttaggaaatgcttttattaatttagaaatttcatcagaatatattatt
ttcttcactttatggtcattctttttgtcttctcttttattagtttgcagattaaagaa
tttcgcattctcgcattatcaattaatgattcactttatagttctcttttctttttctt
acaggattacatttctttcatctatctattggtcttattcttctaatttatttctattat
gtgggtttctttgtttactttatgctttcatggacttcttttaataaaatat
>Isy7_cox3
attaatacagaattattatttcttatctgtatctattggtattttggaaatcattacat
ttagaagttacatcagatttattttgttttcttattaatactcaccatttattaatcttt
gctcttattataattctatttatctttacaggatttaatatttattgttggactggaatt
tacttctcatagaattattttcttatattatttcatcttaattttggattaatttatagt
tttcatatttggtctagagatttattaagagaattcactaaaaaatatgaagtcttatta
ataatcttttttcttcttttgggggttttcttgtttctgaagctctattatttgtatcc
ttcttttggacatcttttcatttattatcttctccaactttagggatgtgggtgtgggaa
ggtttctatctggaggatccttgtgaattaacttttgctaatacacttcttttatcaaat
gctgctatatctttaggaaatgcttttattaatttagaaatttcatcagaatatattatt
ttcttcactttatggtcattctttttgtcttctcttttattagtttgcagattaaagaa
tttcgcattctcgcattatcaattaatgattcactttatagttctcttttctttttctt
acaggattacatttctttcatctatctattggtcttattcttctaatttatttctattat
gtgggtttctttgtttactttatgctttcatggacttcttttaataaaatat

>Isy8_cox3
attaatacagaattattattttcttatctatatctattggtattttggaaatcattacat
ttagaagttacatcagatttattttgttttcttattaatactcaccatttattaatcttt
gctcttattataattctatttatctttacaggatttaatatttattgttggactggaatt
tacttctcatagaattattttcttatattatttcatcttaattttggattaatttatagt
tttcatatttggtctagagatttattaagagaattcactaaaaaatatgaagtcttatta
ataatctttttcttctttttgggggttttctagtttctgaagctctattatttgtatcc
ttcttttggacatcttttcatttattatcttctccaactttagggatgtgggtgtgggaa
ggtttctatctggaggatccttgtgaattaacttttgctaatacacttcttttatcaaat
gctgctatatctttaggaaatgcttttattaatttagaaatttcatcagaatatattatt
ttcttcactttatggtcattctttttgtcttctctttttattagtttgcagattaaagaa
tttcgcattctcgcattatcaattaatgattcactttatagttctcttttcttttttctt
acaggattacatttctttcatctatctattggtcttattcttctaatttatttctattat
gtgggtttctttgtttactttatgctttcatggacttcttttaataaaatat
>KrA1_cox3
attaatacagaattattattttcttatctgtatctattggtattttggaaatcattacat
ttagaagttacatcagatttattttgttttcttattaatactcaccatttattaatcttt
gctcttattataattctatttatctttacaggatttaatatttattgttggactggaatt
tacttctcatagaattattttcttatattatttcatcttaattttggattaatttatagt
tttcatatttggtctagagatttattaagagaattcactaaaaaatatgaagtcttatta
ataatctttttcttctttttgggggttttcttgtttctgaagctctattatttgtatcc
ttcttttggacatcttttcatttattatcttctccaactttagggatgtgggtgtgggaa
ggtttctatctggaggatccttgtgaattaacttttgctaatacacttcttttatcaaat
gctgctatatctttaggaaatgcttttattaatttagaaatttcatcagaatatattatt
ttcttcactttatggtcattctttttgtcttctctttttattagtttgcagattaaagaa
tttcgcattctcgcattatcaattaatgattcactttatagttctcttttcttttttctt
acaggattacatttctttcatctatctattggtcttattcttctaatttatttctattat
gtgggtttctttgtttactttatgctttcatggacttcttttaataaaatat
>KrA10_cox3
attaatacagaattattattttcttatctgtatctattggtattttggaaatcattacat
ttagaagttacatcagatttattttgttttcttattaatactcaccatttattaatcttt
gctcttattataattctatttatctttacaggatttaatatttattgttggactggaatt
tacttctcatagaattattttcttatattatttcatcttaattttggattaatttatagt
tttcatatttggtctagagatttattaagagaattcactaaaaaatatgaagtcttatta
ataatctttttcttctttttgggggttttcttgtttctgaagctctattatttgtatcc
ttcttttggacatcttttcatttattatcttctccaactttagggatgtgggtgtgggaa
ggtttctatctggaggatccttgtgaattaacttttgctaatacacttcttttatcaaat
gctgctatatctttaggaaatgcttttattaatttagaaatttcatcagaatatattatt
ttcttcactttatggtcattctttttgtcttctctttttattagtttgcagattaaagaa
tttcgcattctcgcattatcaattaatgattcactttatagttctcttttcttttttctt
acaggattacatttctttcatctatctattggtcttattcttctaatttatttctattat
gtgggtttctttgtttactttatgctttcatggacttcttttaataaaatat
>KrA11_cox3
attaatacagaattattattttcttatctgtatctattggtattttggaaatcattacat
ttagaagttacatcagatttattttgttttcttattaatactcaccatttattaatcttt
gctcttattataattctatttatctttacaggatttaatatttattgttggactggaatt
tacttctcatagaattattttcttatattatttcatcttaattttggattaatttatagt
tttcatatttggtctagagatttattaagagaattcactaaaaaatatgaagtcttatta
ataatctttttcttctttttgggggttttcttgtttctgaagctctattatttgtatcc
ttcttttggacatcttttcatttattatcttctccaactttagggatgtgggtgtgggaa
ggtttctatctggaggatccttgtgaattaacttttgctaatacacttcttttatcaaat
gctgctatatctttaggaaatgcttttattaatttagaaatttcatcagaatatattatt
ttcttcactttatggtcattctttttgtcttctctttttattagtttgcagattaaagaa
tttcgcattctcgcattatcaattaatgattcactttatagttctcttttcttttttctt
acaggattacatttctttcatctatctattggtcttattcttctaatttatttctattat
gtgggtttctttgtttactttatgctttcatggacttcttttaataaaatat
>KrA12_cox3

attaatacagaattattatttcttatctgtatctattggtattttggaaatcattacat
ttagaagttacatcagatttattttgttttcttattaatactcaccatttattaatcttt
gctcttattataattctatttatctttacaggatttaatatttattgttggactggaatt
tacttctcatagaattattttcttatattatttcatcttaattttggattaatttatagt
tttcatatttggtctagagatttattaagagaattcactaaaaaatatgaagtcttatta
ataatctttttcttctttttgggggttttcttgtttctgaagctctattatttgtatcc
ttcttttggacatcttttcatttattatcttctccaactttagggatgtgggtgtgggaa
ggtttctatctggaggatccttgtgaattaacttttgctaatacacttcttttatcaaat
gctgctatatctttaggaaatgcttttattaatttagaaatttcatcagaatatattatt
ttcttcactttatggtcattctttttgtcttctctttttattagtttgcagattaaagaa
tttcgcattctcgcattatcaattaatgattcactttatagttctcttttcttttttctt
acaggattacatttctttcatctatctattggtcttattcttctaatttatttctattat
gtgggtttctttgtttactttatgctttcatggacttcttttaataaaatat
>KrA13_cox3
attaatacagaattattatttcttatctgtatctattggtattttggaaatcattacat
ttagaagttacatcagatttattttgttttcttattaatactcaccatttattaatcttt
gctcttattataattctatttatctttacaggatttaatatttattgttggactggaatt
tacttctcatagaattattttcttatattatttcatcttaattttggattaatttatagt
tttcatatttggtctagagatttattaagagaattcactaaaaaatatgaagtcttatta
ataatctttttcttctttttgggggttttcttgtttctgaagctctattatttgtatcc
ttcttttggacatcttttcatttattatcttctccaactttagggatgtgggtgtgggaa
ggtttctatctggaggatccttgtgaattaacttttgctaatacacttcttttatcaaat
gctgctatatctttaggaaatgcttttattaatttagaaatttcatcagaatatattatt
ttcttcactttatggtcattctttttgtcttctctttttattagtttgcagattaaagaa
tttcgcattctcgcattatcaattaatgattcactttatagttctcttttcttttttctt
acaggattacatttctttcatctatctattggtcttattcttctaatttatttctattat
gtgggtttctttgtttactttatgctttcatggacttcttttaataaaatat
>KrA14_cox3
attaatacagaattattatttcttatctgtatctattggtattttggaaatcattacat
ttagaagttacatcagatttattttgttttcttattaatactcaccatttattaatcttt
gctcttattataattctatttatctttacaggatttaatatttattgttggactggaatt
tacttctcatagaattattttcttatattatttcatcttaattttggattaatttatagt
tttcatatttggtctagagatttattaagagaattcactaaaaaatatgaagtcttatta
ataatctttttcttctttttgggggttttcttgtttctgaagctctattatttgtatcc
ttcttttggacatcttttcatttattatcttctccaactttagggatgtgggtgtgggaa
ggtttctatctggaggatccttgtgaattaacttttgctaatacacttcttttatcaaat
gctgctatatctttaggaaatgcttttattaatttagaaatttcatcagaatatattatt
ttcttcactttatggtcattctttttgtcttctctttttattagtttgcagattaaagaa
tttcgcattctcgcattatcaattaatgattcactttatagttctcttttcttttttctt
acaggattacatttctttcatctatctattggtcttattcttctaatttatttctattat
gtgggtttctttgtttactttatgctttcatggacttcttttaataaaatat
>KrA15_cox3
attaatacagaattattatttcttatctgtatctattggtattttggaaatcattacat
ttagaagttacatcagatttattttgttttcttattaatactcaccatttattaatcttt
gctcttattataattctatttatctttacaggatttaatatttattgttggactggaatt
tacttctcatagaattattttcttatattatttcatcttaattttggattaatttatagt
tttcatatttggtctagagatttattaagagaattcactaaaaaatatgaagtcttatta
ataatctttttcttctttttgggggttttcttgtttctgaagctctattatttgtatcc
ttcttttggacatcttttcatttattatcttctccaactttagggatgtgggtgtgggaa
ggtttctatctggaggatccttgtgaattaacttttgctaatacacttcttttatcaaat
gctgctatatctttaggaaatgcttttattaatttagaaatttcatcagaatatattatt
ttcttcactttatggtcattctttttgtcttctctttttattagtttgcagattaaagaa
tttcgcattctcgcattatcaattaatgattcactttatagttctcttttcttttttctt
acaggattacatttctttcatctatctattggtcttattcttctaatttatttctattat
gtgggtttctttgtttactttatgctttcatggacttcttttaataaaatat
>KrA2_cox3
attaatacagaattattatttcttatctgtatctattggtattttggaaatcattacat

ttagaagttacatcagatttattttgttttcttattaatactcaccatttattaatcttt
gctcttattataattctatttatctttacaggatttaatatttattgttggactggaatt
tacttctcatagaattattttcttatattatttcatcttaattttggattaatttatagt
tttcatatttggtctagagatttattaagagaattcactaaaaaatatgaagtcttatta
ataatctttttcttctttttgggggttttcttgtttctgaagctctattatttgtatcc
ttcttttggacatcttttcatttattatcttctccaactttagggatgtgggtgtgggaa
ggtttctatctggaggatccttgtgaattaacttttgctaatacacttcttttatcaaat
gctgctatatctttaggaaatgcttttattaatttagaaatttcatcagaatatattatt
ttcttcactttatggtcattctttttgtcttctctttttattagtttgcagattaaagaa
tttcgcattctcgcattatcaattaatgattcactttatagttctcttttcttttttctt
acaggattacatttctttcatctatctattggtcttattcttctaatttatttctattat
gtgggtttctttgtttactttatgctttcatggacttcttttaataaaatat
>KrA3_cox3
attaatacagaattattattttcttatctgtatctattggtattttggaaatcattacat
ttagaagttacatcagatttattttgttttcttattaatactcaccatttattaatcttt
gctcttattataattctatttatctttacaggatttaatatttattgttggactggaatt
tacttctcatagaattattttcttatattatttcatcttaattttggattaatttatagt
tttcatatttggtctagagatttattaagagaattcactaaaaaatatgaagtcttatta
ataatctttttcttctttttgggggttttcttgtttctgaagctctattatttgtatcc
ttcttttggacatcttttcatctattatcttctccaactttagggatgtgggtgtgggaa
ggtttctatctggaggatccttgtgaattaacttttgctaatacacttcttttatcaaat
gctgctatatctttaggaaatgcttttattaatttagaaatttcatcagaatatattatt
ttcttcactttatggtcattctttttgtcttctctttttattagtttgcagattaaagaa
tttcgcattctcgcattatcaattaatgattcactttatagttctcttttcttttttctt
acaggattacatttctttcatctatctattggtcttattcttctaatttatttctattat
gtgggtttctttgtttactttatgctttcatggacttcttttaataaaatat
>KrA4_cox3
attaatacagaattattattttcttatctgtatctattggtattttggaaatcattacat
ttagaagttacatcagatttattttgttttcttattaatactcaccatttattaatcttt
gctcttattataattctatttatctttacaggatttaatatttattgttggactggaatt
tacttctcatagaattattttcttatattatttcatcttaattttggattaatttatagt
tttcatatttggtctagagatttattaagagaattcactaaaaaatatgaagtcttatta
ataatctttttcttctttttgggggttttcttgtttctgaagctctattatttgtatcc
ttcttttggacatcttttcatttattatcttctccaactttagggatgtgggtgtgggaa
ggtttctatctggaggatccttgtgaattaacttttgctaatacacttcttttatcaaat
gctgctatatctttaggaaatgcttttattaatttagaaatttcatcagaatatattatt
ttcttcactttatggtcattctttttgtcttctctttttattagtttgcagattaaagaa
tttcgcattctcgcattatcaattaatgattcactttatagttctcttttcttttttctt
acaggattacatttctttcatctatctattggtcttattcttctaatttatttctattat
gtgggtttctttgtttactttatgctttcatggacttcttttaataaaatat
>KrA5_cox3
attaatacagaattattattttcttatctgtatctattggtattttggaaatcattacat
ttagaagttacatcagatttattttgttttcttattaatactcaccatttattaatcttt
gctcttattataattctatttatctttacaggatttaatatttattgttggactggaatt
tacttctcatagaattattttcttatattatttcatcttaattttggattaatttatagt
tttcatatttggtctagagatttattaagagaattcactaaaaaatatgaagtcttatta
ataatctttttcttctttttgggggttttcttgtttctgaagctctattatttgtatcc
ttcttttggacatcttttcatttattatcttctccaactttagggatgtgggtgtgggaa
ggtttctatctggaggatccttgtgaattaacttttgctaatacacttcttttatcaaat
gctgctatatctttaggaaatgcttttattaatttagaaatttcatcagaatatattatt
ttcttcactttatggtcattctttttgtcttctctttttattagtttgcagattaaagaa
tttcgcattctcgcattatcaattaatgattcactttatagttctcttttcttttttctt
acaggattacatttctttcatctatctattggtcttattcttctaatttatttctattat
gtgggtttctttgtttactttatgctttcatggacttcttttaataaaatat
>KrA6_cox3
attaatacagaattattattttcttatctgtatctattggtattttggaaatcattacat
ttagaagttacatcagatttattttgttttcttattaatactcaccatttattaatcttt

gctcttattataattctatttatctttacaggatttaatatttattgttggactggaatt
tacttctcatagaattattttcttatattatttcatcttaattttggattaatttatagt
tttcatatttggtctagagatttattaagagaattcactaaaaaatatgaagtcttatta
ataatcttttttcttcttttttgggggttttcttgtttctgaagctctattatttgtatcc
ttcttttggacatcttttcatttattatcttctccaactttagggatgtgggtgtgggaa
ggtttctatctggaggatccttgtgaattaacttttgctaatacacttcttttatcaaat
gctgctatatctttaggaaatgcttttattaatttagaaatttcatcagaatatattatt
ttcttcactttatggtcattctttttgtcttctctttttattagtttgcagattaaagaa
tttcgcattctcgcattatcaattaatgattcactttatagttctcttttcttttttctt
acaggattacatttctttcatctatctattggtcttattcttctaatttatttctattat
gtgggtttctttgtttactttatgctttcatggacttcttttaataaaatat
>KrA7_cox3
attaatacagaattattattttcttatctgtatctattggtattttggaaatcattacat
ttagaagttacatcagatttattttgttttcttattaatactcaccatttattaatcttt
gctcttattataattctatttatctttacaggatttaatatttattgttggactggaatt
tacttctcatagaattattttcttatattatttcatcttaattttggattaatttatagt
tttcatatttggtctagagatttattaagagaattcactaaaaaatatgaagtcttatta
ataatcttttttcttcttttttgggggttttcttgtttctgaagctctattatttgtatcc
ttcttttggacatcttttcatttattatcttctccaactttagggatgtgggtgtgggaa
ggtttctatctggaggatccttgtgaattaacttttgctaatacacttcttttatcaaat
gctgctatatctttaggaaatgcttttattaatttagaaatttcatcagaatatattatt
ttcttcactttatggtcattctttttgtcttctctttttattagtttgcagattaaagaa
tttcgcattctcgcattatcaattaatgattcactttatagttctcttttcttttttctt
acaggattacatttctttcatctatctattggtcttattcttctaatttatttctattat
gtgggtttctttgtttactttatgctttcatggacttcttttaataaaatat
>KrA8_cox3
attaatacagaattattattttcttatctgtatctattggtattttggaaatcattacat
ttagaagttacatcagatttattttgttttcttattaatactcaccatttattaatcttt
gctcttattataattctatttatctttacaggatttaatatttattgttggactggaatt
tacttctcatagaattattttcttatattatttcatcttaattttggattaatttatagt
tttcatatttggtctagagatttattaagagaattcactaaaaaatatgaagtcttatta
ataatcttttttcttcttttttgggggttttcttgtttctgaagctctattatttgtatcc
ttcttttggacatcttttcatttattatcttctccaactttagggatgtgggtgtgggaa
ggtttctatctggaggatccttgtgaattaacttttgctaatacacttcttttatcaaat
gctgctatatctttaggaaatgcttttattaatttagaaatttcatcagaatatattatt
ttcttcactttatggtcattctttttgtcttctctttttattagtttgcagattaaagaa
tttcgcattctcgcattatcaattaatgattcactttatagttctcttttcttttttctt
acaggattacatttctttcatctatctattggtcttattcttctaatttatttctattat
gtgggtttctttgtttactttatgctttcatggacttcttttaataaaatat
>KrA9_cox3
attaatacagaattattattttcttatctgtatctattggtattttggaaatcattacat
ttagaagttacatcagatttattttgttttcttattaatactcaccatttattaatcttt
gctcttattataattctatttatctttacaggatttaatatttattgttggactggaatt
tacttctcatagaattattttcttatattatttcatcttaattttggattaatttatagt
tttcatatttggtctagagatttattaagagaattcactaaaaaatatgaagtcttatta
ataatcttttttcttcttttttgggggttttcttgtttctgaagctctattatttgtatcc
ttcttttggacatcttttcatttattatcttctccaactttagggatgtgggtgtgggaa
ggtttctatctggaggatccttgtgaattaacttttgctaatacacttcttttatcaaat
gctgctatatctttaggaaatgcttttattaatttagaaatttcatcagaatatattatt
ttcttcactttatggtcattctttttgtcttctctttttattagtttgcagattaaagaa
tttcgcattctcgcattatcaattaatgattcactttatagttctcttttcttttttctt
acaggattacatttctttcatctatctattggtcttattcttctaatttatttctattat
gtgggtttctttgtttactttatgctttcatggacttcttttaataaaatat
>KrC1_cox3
attaatacagaattattattttcttatctgtatctattggtattttggaaatcattacat
ttagaagttacatcagatttattttgttttcttattaatactcaccatttattaatcttt
gctcttattataattctatttatctttacaggatttaatatttattgttggactggaatt

tacttctcatagaattattttcttatattatttcatcttaattttggattaatttatagt
tttcatatttggtctagagatttattaagagaattcactaaaaaaatatgaagtcttatta
ataatcttttttcttctttttgggggttttcttgtttctgaagctctattatttgtatcc
ttcttttggacatcttttcatttattatcttctccaactttagggatgtgggtgtgggaa
ggtttctatctggaggatccttgtgaattaacttttgctaatacacttcttttatcaaat
gctgctatatctttaggaaatgcttttattaatttagaaatttcatcagaatatattatt
ttcttcactttatggtcattctttttgtcttctcttttattagtttgcagattaaagaa
tttcgcattctcgcattatcaattaatgattcactttatagttctcttttcttttttctt
acaggattacatttctttcatctatctattggtcttattcttctaatttatttctattat
gtgggtttctttgtttactttatgctttcatggacttcttttaataaaatat
>KrC10_cox3
attaatacagaattattatttcttatctgtatctattggtattttggaaatcattacat
ttagaagttacatcagatttattttgttttcttattaatactcaccatttattaatcttt
gctcttattataattctatttatctttacaggatttaatatttattgttggactggaatt
tacttctcatagaattattttcttatattatttcatcttaattttggattaatttatagt
tttcatatttggtctagagatttattaagagaattcactaaaaaaatatgaagtcttatta
ataatcttttttcttctttttgggggttttcttgtttctgaagctctattatttgtatcc
ttcttttggacatcttttcatttattatcttctccaactttagggatgtgggtgtgggaa
ggtttctatctggaggatccttgtgaattaacttttgctaatacacttcttttatcaaat
gctgctatatctttaggaaatgcttttattaatttagaaatttcatcagaatatattatt
ttcttcactttatggtcattctttttgtcttctcttttattagtttgcagattaaagaa
tttcgcattctcgcattatcaattaatgattcactttatagttctcttttcttttttctt
acaggattacatttctttcatctatctattggtcttattcttctaatttatttctattat
gtgggtttctttgtttactttatgctttcatggacttcttttaataaaatat
>KrC11_cox3
attaatacagaattattatttcttatctgtatctattggtattttggaaatcattacat
ttagaagttacatcagatttattttgttttcttattaatactcaccatttattaatcttt
gctcttattataattctatttatctttacaggatttaatatttattgttggactggaatt
tacttctcatagaattattttcttatattatttcatcttaattttggattaatttatagt
tttcatatttggtctagagatttattaagagaattcactaaaaaaatatgaagtcttatta
ataatcttttttcttctttttgggggttttcttgtttctgaagctctattatttgtatcc
ttcttttggacatcttttcatttattatcttctccaactttagggatgtgggtgtgggaa
ggtttctatctggaggatccttgtgaattaacttttgctaatacacttcttttatcaaat
gctgctatatctttaggaaatgcttttattaatttagaaatttcatcagaatatattatt
ttcttcactttatggtcattctttttgtcttctcttttattagtttgcagattaaagaa
tttcgcattctcgcattatcaattaatgattcactttatagttctcttttcttttttctt
acaggattacatttctttcatctatctattggtcttattcttctaatttatttctattat
gtgggtttctttgtttactttatgctttcatggacttcttttaataaaatat
>KrC12_cox3
attaatacagaattattatttcttatctgtatctattggtattttggaaatcattacat
ttagaagttacatcagatttattttgttttcttattaatactcaccatttattaatcttt
gctcttattataattctatttatctttacaggatttaatatttattgttggactggaatt
tacttctcatagaattattttcttatattatttcatcttaattttggattaatttatagt
tttcatatttggtctagagatttattaagagaattcactaaaaaaatatgaagtcttatta
ataatcttttttcttctttttgggggttttcttgtttctgaagctctattatttgtatcc
ttcttttggacatcttttcatttattatcttctccaactttagggatgtgggtgtgggaa
ggtttctatctggaggatccttgtgaattaacttttgctaatacacttcttttatcaaat
gctgctatatctttaggaaatgcttttattaatttagaaatttcatcagaatatattatt
ttcttcactttatggtcattctttttgtcttctcttttattagtttgcagattaaagaa
tttcgcattctcgcattatcaattaatgattcactttatagttctcttttcttttttctt
acaggattacatttctttcatctatctattggtcttattcttctaatttatttctattat
gtgggtttctttgtttactttatgctttcatggacttcttttaataaaatat
>KrC13_cox3
attaatacagaattattatttcttatctgtatctattggtattttggaaatcattacat
ttagaagttacatcagatttattttgttttcttattaatactcaccatttattaatcttt
gctcttattataattctatttatctttacaggatttaatatttattgttggactggaatt
tacttctcatagaattattttcttatattatttcatcttaattttggattaatttatagt

tttcatatttggtctagagatttattaagagaattcactaaaaaatatgaagtcttatta
ataatctttttcttcttttggggggtttcttgtttctgaagctctattatttgtatcc
ttcttttggacatcttttcatttattatcttctccaactttagggatgtgggtgtgggaa
ggtttctatctggaggatccttgtgaattaacttttgctaatacacttcttttatcaaat
gctgctatatctttaggaaatgcttttattaatttagaaatttcatcagaatatattatt
ttcttcactttatggtcattctttttgtcttctcttttattagtttgcagattaaagaa
tttcgcattctcgcattatcaattaatgattcactttatagttctcttttcttttttctt
acaggattacatttctttcatctatctattggtcttattcttctaatttatttctattat
gtgggtttctttgtttactttatgctttcatggacttcttttaataaaatat
>KrC15_cox3
attaatacagaattattattttcttatctgtatctattggtattttggaaatcattacat
ttagaagttacatcagatttattttgttttcttattaatactcaccatttattaatcttt
gctcttattataattctatttatctttacaggatttaatatttattgttggactggaatt
tacttctcatagaattattttcttatattatttcatcttaattttggattaatttatagt
tttcatatttggtctagagatttattaagagaattcactaaaaaatatgaagtcttatta
ataatctttttcttcttttggggggtttcttgtttctgaagctctattatttgtatcc
ttcttttggacatcttttcatttattatcttctccaactttagggatgtgggtgtgggaa
ggtttctatctggaggatccttgtgaattaacttttgctaatacacttcttttatcaaat
gctgctatatctttaggaaatgcttttattaatttagaaatttcatcagaatatattatt
ttcttcactttatggtcattctttttgtcttctcttttattagtttgcagattaaagaa
tttcgcattctcgcattatcaattaatgattcactttatagttctcttttcttttttctt
acaggattacatttctttcatctatctattggtcttattcttctaatttatttctattat
gtgggtttctttgtttactttatgctttcatggacttcttttaataaaatat
>KrC2_cox3
attaatacagaattattattttcttatctgtatctattggtattttggaaatcattacat
ttagaagttacatcagatttattttgttttcttattaatactcaccatttattaatcttt
gctcttattataattctatttatctttacaggatttaatatttattgttggactggaatt
tacttctcatagaattattttcttatattatttcatcttaattttggattaatttatagt
tttcatatttggtctagagatttattaagagaattcactaaaaaatatgaagtcttatta
ataatctttttcttcttttggggggtttcttgtttctgaagctctattatttgtatcc
ttcttttggacatcttttcatttattatcttctccaactttagggatgtgggtgtgggaa
ggtttctatctggaggatccttgtgaattaacttttgctaatacacttcttttatcaaat
gctgctatatctttaggaaatgcttttattaatttagaaatttcatcagaatatattatt
ttcttcactttatggtcattctttttgtcttctcttttattagtttgcagattaaagaa
tttcgcattctcgcattatcaattaatgattcactttatagttctcttttcttttttctt
acaggattacatttctttcatctatctattggtcttattcttctaatttatttctattat
gtgggtttctttgtttactttatgctttcatggacttcttttaataaaatat
>KrC3_cox3
attaatacagaattattattttcttatctgtatctattggtattttggaaatcattacat
ttagaagttacatcagatttattttgttttcttattaatactcaccatttattaatcttt
gctcttattataattctatttatctttacaggatttaatatttattgttggactggaatt
tacttctcatagaattattttcttatattatttcatcttaattttggattaatttatagt
tttcatatttggtctagagatttattaagagaattcactaaaaaatatgaagtcttatta
ataatctttttcttcttttggggggtttcttgtttctgaagctctattatttgtatcc
ttcttttggacatcttttcatttattatcttctccaactttagggatgtgggtgtgggaa
ggtttctatctggaggatccttgtgaattaacttttgctaatacacttcttttatcaaat
gctgctatatctttaggaaatgcttttattaatttagaaatttcatcagaatatattatt
ttcttcactttatggtcattctttttgtcttctcttttattagtttgcagattaaagaa
tttcgcattctcgcattatcaattaatgattcactttatagttctcttttcttttttctt
acaggattacatttctttcatctatctattggtcttattcttctaatttatttctattat
gtgggtttctttgtttactttatgctttcatggacttcttttaataaaatat
>KrC4_cox3
attaatacagaattattattttcttatctgtatctattggtattttggaaatcattacat
ttagaagttacatcagatttattttgttttcttattaatactcaccatttattaatcttt
gctcttattataattctatttatctttacaggatttaatatttattgttggactggaatt
tacttctcatagaattattttcttatattatttcatcttaattttggattaatttatagt
tttcatatttggtctagagatttattaagagaattcactaaaaaatatgaagtcttatta

ataatctttttcttctttttggggggttttcttgtttctgaagctctattatttgtatcc
ttcttttggacatcttttcatttattatcttctccaactttagggatgtgggtgtgggaa
ggtttctatctggaggatccttgtgaattaacttttgctaatacacttcttttatcaaat
gctgctatatctttaggaaatgcttttattaatttagaaatttcatcagaatatattatt
ttcttcactttatggtcattctttttgtcttctctttttattagtttgcagattaaagaa
tttcgcattctcgcattatcaattaatgattcactttatagttctcttttcttttttctt
acaggattacatttctttcatctatctattggtcttattcttctaatttatttctattat
gtgggtttctttgtttactttatgctttcatggacttcttttaataaaatat
>KrC5_cox3
attaatacagaattattattttcttatctgtatctattggtattttggaaatcattacat
ttagaagttacatcagatttattttgttttcttattaatactcaccatttattaatcttt
gctcttattataattctatttatctttacaggatttaatatttattgttggactggaatt
tacttctcatagaattattttcttatattatttcatcttaattttggattaatttatagt
tttcatatttggtctagagatttattaagagaattcactaaaaaatatgaagtcttatta
ataatctttttcttctttttggggggttttcttgtttctgaagctctattatttgtatcc
ttcttttggacatcttttcatttattatcttctccaactttagggatgtgggtgtgggaa
ggtttctatctggaggatccttgtgaattaacttttgctaatacacttcttttatcaaat
gctgctatatctttaggaaatgcttttattaatttagaaatttcatcagaatatattatt
ttcttcactttatggtcattctttttgtcttctctttttattagtttgcagattaaagaa
tttcgcattctcgcattatcaattaatgattcactttatagttctcttttcttttttctt
acaggattacatttctttcatctatctattggtcttattcttctaatttatttctattat
gtgggtttctttgtttactttatgctttcatggacttcttttaataaaatat
>KrC6_cox3
attaatacagaattattattttcttatctgtatctattggtattttggaaatcattacat
ttagaagttacatcagatttattttgttttcttattaatactcaccatttattaatcttt
gctcttattataattctatttatctttacaggatttaatatttattgttggactggaatt
tacttctcatagaattattttcttatattatttcatcttaattttggattaatttatagt
tttcatatttggtctagagatttattaagagaattcactaaaaaatatgaagtcttatta
ataatctttttcttctttttggggggttttcttgtttctgaagctctattatttgtatcc
ttcttttggacatcttttcatctattatcttctccaactttagggatgtgggtgtgggaa
ggtttctatctggaggatccttgtgaattaacttttgctaatacacttcttttatcaaat
gctgctatatctttaggaaatgcttttattaatttagaaatttcatcagaatatattatt
ttcttcactttatggtcattctttttgtcttctctttttattagtttgcagattaaagaa
tttcgcattctcgcattatcaattaatgattcactttatagttctcttttcttttttctt
acaggattacatttctttcatctatctattggtcttattcttctaatttatttctattat
gtgggtttctttgtttactttatgctttcatggacttcttttaataaaatat
>KrC7_cox3
attaatacagaattattattttcttatctgtatctattggtattttggaaatcattacat
ttagaagttacatcagatttattttgttttcttattaatactcaccatttattaatcttt
gctcttattataattctatttatctttacaggatttaatatttattgttggactggaatt
tacttctcatagaattattttcttatattatttcatcttaattttggattaatttatagt
tttcatatttggtctagagatttattaagagaattcactaaaaaatatgaagtcttatta
ataatctttttcttctttttggggggttttcttgtttctgaagctctattatttgtatcc
ttcttttggacatcttttcatttattatcttctccaactttagggatgtgggtgtgggaa
ggtttctatctggaggatccttgtgaattaacttttgctaatacacttcttttatcaaat
gctgctatatctttaggaaatgcttttattaatttagaaatttcatcagaatatattatt
ttcttcactttatggtcattctttttgtcttctctttttattagtttgcagattaaagaa
tttcgcattctcgcattatcaattaatgattcactttatagttctcttttcttttttctt
acaggattacatttctttcatctatctattggtcttattcttctaatttatttctattat
gtgggtttctttgtttactttatgctttcatggacttcttttaataaaatat
>KrC8_cox3
attaatacagaattattattttcttatctgtatctattggtattttggaaatcattacat
ttagaagttacatcagatttattttgttttcttattaatactcaccatttattaatcttt
gctcttattataattctatttatctttacaggatttaatatttattgttggactggaatt
tacttctcatagaattattttcttatattatttcatcttaattttggattaatttatagt
tttcatatttggtctagagatttattaagagaattcactaaaaaatatgaagtcttatta
ataatctttttcttctttttggggggttttcttgtttctgaagctctattatttgtatcc

ttcttttggacatcttttcatttattatcttctccaactttagggatgtgggtgtgggaa
ggtttctatctggaggatccttgtgaattaacttttgctaatacacttcttttatcaaat
gctgctatatctttaggaaatgcttttattaatttagaaatttcatcagaatatattatt
ttcttcactttatggtcattctttttgtcttctcttttattagtttgcagattaaagaa
tttcgcattctcgcattatcaattaatgattcactttatagttctcttttcttttttctt
acaggattacatttctttcatctatctattggtcttattcttctaatttatttctattat
gtgggtttctttgtttactttatgctttcatggacttcttttaataaaatat
>KrC9_cox3
attaatacagaattattattttcttatctgtatctattggtattttggaaatcattacat
ttagaagttacatcagatttattttgttttcttattaatactcaccatttattaatcttt
gctcttattataattctatttatctttacaggatttaatatttattgttggactggaatt
tacttctcatagaattattttcttatattatttcatcttaattttggattaatttatagt
tttcatatttggtctagagatttattaagagaattcactaaaaaaatatgaagtcttatta
ataatcttttttcttctttttgggggttttcttgtttctgaagctctattatttgtatcc
ttcttttggacatcttttcatttattatcttctccaactttagggatgtgggtgtgggaa
ggtttctatctggaggatccttgtgaattaacttttgctaatacacttcttttatcaaat
gctgctatatctttaggaaatgcttttattaatttagaaatttcatcagaatatattatt
ttcttcactttatggtcattctttttgtcttctcttttattagtttgcagattaaagaa
tttcgcattctcgcattatcaattaatgattcactttatagttctcttttcttttttctt
acaggattacatttctttcatctatctattggtcttattcttctaatttatttctattat
gtgggtttctttgtttactttatgctttcatggacttcttttaataaaatat
>KrD1_cox3
attaatacagaattattattttcttatctgtatctattggtattttggaaatcattacat
ttagaagttacatcagatttattttgttttcttattaatactcaccatttattaatcttt
gctcttattataattctatttatctttacaggatttaatatttattgttggactggaatt
tacttctcatagaattattttcttatattatttcatcttaattttggattaatttatagt
tttcatatttggtctagagatttattaagagaattcactaaaaaaatatgaagtcttatta
ataatcttttttcttctttttgggggttttcttgtttctgaagctctattatttgtatcc
ttcttttggacatcttttcatttattatcttctccaactttagggatgtgggtgtgggaa
ggtttctatctggaggatccttgtgaattaacttttgctaatacacttcttttatcaaat
gctgctatatctttaggaaatgcttttattaatttagaaatttcatcagaatatattatt
ttcttcactttatggtcattctttttgtcttctcttttattagtttgcagattaaagaa
tttcgcattctcgcattatcaattaatgattcactttatagttctcttttcttttttctt
acaggattacatttctttcatctatctattggtcttattcttctaatttatttctattat
gtgggtttctttgtttactttatgctttcatggacttcttttaataaaatat
>KrD11_cox3
attaatacagaattattattttcttatctgtatctattggtattttggaaatcattacat
ttagaagttacatcagatttattttgttttcttattaatactcaccatttattaatcttt
gctcttattataattctatttatctttacaggatttaatatttattgttggactggaatt
tacttctcatagaattattttcttatattatttcatcttaattttggattaatttatagt
tttcatatttggtctagagatttattaagagaattcactaaaaaaatatgaagtcttatta
ataatcttttttcttctttttgggggttttcttgtttctgaagctctattatttgtatcc
ttcttttggacatcttttcatttattatcttctccaactttagggatgtgggtgtgggaa
ggtttctatctggaggatccttgtgaattaacttttgctaatacacttcttttatcaaat
gctgctatatctttaggaaatgcttttattaatttagaaatttcatcagaatatattatt
ttcttcactttatggtcattctttttgtcttctcttttattagtttgcagattaaagaa
tttcgcattctcgcattatcaattaatgattcactttatagttctcttttcttttttctt
acaggattacatttctttcatctatctattggtcttattcttctaatttatttctattat
gtgggtttctttgtttactttatgctttcatggacttcttttaataaaatat
>KrD13_cox3
attaatacagaattattattttcttatctgtatctattggtattttggaaatcattacat
ttagaagttacatcagatttattttgttttcttattaatactcaccatttattaatcttt
gctcttattataattctatttatctttacaggatttaatatttattgttggactggaatt
tacttctcatagaattattttcttatattatttcatcttaattttggattaatttatagt
tttcatatttggtctagagatttattaagagaattcactaaaaaaatatgaagtcttatta
ataatcttttttcttctttttgggggttttcttgtttctgaagctctattatttgtatcc
ttcttttggacatcttttcatttattatcttctccaactttagggatgtgggtgtgggaa

ggtttctatctggaggatccttgtgaattaacttttgctaatacacttcttttatcaaat
gctgctatatctttaggaaatgcttttattaatttagaaatttcatcagaatatattatt
ttcttcactttatggtcattctttttgtcttctcttttttattagtttgcagattaaagaa
tttcgcattctcgcattatcaattaatgattcactttatagttctcttttcttttttctt
acaggattacatttctttcatctatctattggtcttattcttctaatttatttctattat
gtgggtttctttgtttactttatgctttcatggacttcttttaataaaatat
>KrD3_cox3
attaatacagaattattattttcttatctgtatctattggtattttggaaatcattacat
ttagaagttacatcagatttattttgttttcttattaatactcaccatttattaatcttt
gctcttattataattctatttatctttacaggatttaatatttattgttggactggaatt
tacttctcatagaattattttcttatattatttcatcttaattttggattaatttatagt
tttcatatttggtctagagatttattaagagaattcactaaaaaaatatgaagtcttatta
ataatctttttcttctttttgggggttttcttgtttctgaagctctattatttgtatcc
ttcttttggacatcttttcatttattatcttctccaactttagggatgtgggtgtgggaa
ggtttctatctggaggatccttgtgaattaacttttgctaatacacttcttttatcaaat
gctgctatatctttaggaaatgcttttattaatttagaaatttcatcagaatatattatt
ttcttcactttatggtcattctttttgtcttctcttttttattagtttgcagattaaagaa
tttcgcattctcgcattatcaattaatgattcactttatagttctcttttcttttttctt
acaggattacatttctttcatctatctattggtcttattcttctaatttatttctattat
gtgggtttctttgtttactttatgctttcatggacttcttttaataaaatat
>KrD8_cox3
attaatacagaattattattttcttatctgtatctattggtattttggaaatcattacat
ttagaagttacatcagatttattttgttttcttattaatactcaccatttattaatcttt
gctcttattataattctatttatctttacaggatttaatatttattgttggactggaatt
tacttctcatagaattattttcttatattatttcatcttaattttggattaatttatagt
tttcatatttggtctagagatttattaagagaattcactaaaaaaatatgaagtcttatta
atggtcctttttcttcttttgggggttttcttgtttctgaagctctattatttgtatcc
ttcttttggacatcttttcatttattatcttctccaactttagggatgtgggtgtgggaa
ggtttctatctggaggatccttgtgaattaacttttgctaatacacttcttttatcaaat
gctgctatatctttaggaaatgcttttattaatttagaaatttcatcagaatatattatt
ttcttcactttatggtcattctttttgtcttctcttttttattagtttgcagattaaagaa
tttcgcattctcgcattatcaattaatgattcactttatagttctcttttcttttttctt
acaggattacatttctttcatctatctattggtcttattcttctaatttatttctattat
gtgggtttctttgtttactttatgctttcatggacttcttttaataaaatat
>KrD9_cox3
attaatacagaattattattttcttatctgtatctattggtattttggaaatcattacat
ttagaagttacatcagatttattttgttttcttattaatactcaccatttattaatcttt
gctcttattataattctatttatctttacaggatttaatatttattgttggactggaatt
tacttctcatagaattattttcttatattatttcatcttaattttggattaatttatagt
tttcatatttggtctagagatttattaagagaattcactaaaaaaatatgaagtcttatta
ataatctttttcttctttttgggggttttcttgtttctgaagctctattatttgtatcc
ttcttttggacatcttttcatttattatcttctccaactttagggatgtgggtgtgggaa
ggtttctatctggaggatccttgtgaattaacttttgctaatacacttcttttatcaaat
gctgctatatctttaggaaatgcttttattaatttagaaatttcatcagaatatattatt
ttcttcactttatggtcattctttttgtcttctcttttttattagtttgcagattaaagaa
tttcgcattctcgcattatcaattaatgattcactttatagttctcttttcttttttctt
acaggattacatttctttcatctatctattggtcttattcttctaatttatttctattat
gtgggtttctttgtttactttatgctttcatggacttcttttaataaaatat
>KrE1_cox3
attaatacagaattattattttcttatctgtatctattggtattttggaaatcattacat
ttagaagttacatcagatttattttgttttcttattaatactcaccatttattaatcttt
gctcttattataattctatttatctttacaggatttaatatttattgttggactggaatt
tacttctcatagaattattttcttatattatttcatcttaattttggattaatttatagt
tttcatatttggtctagagatttattaagagaattcactaaaaaaatatgaagtcttatta
ataatctttttcttctttttgggggttttcttgtttctgaagctctattatttgtatcc
ttcttttggacatcttttcatttattatcttctccaactttagggatgtgggtgtgggaa
ggtttctatctggaggatccttgtgaattaacttttgctaatacacttcttttatcaaat

gctgctatatctttaggaaatgcttttattaatttagaaatttcatcagaatatattatt
ttcttcactttatggtcattctttttgtcttctctttttattagtttgcagattaaagaa
tttcgcattctcgcattatcaattaatgattcactttatagttctcttttcttttttctt
acaggattacatttctttcatctatctattggtcttattcttctaatttatttctattat
gtgggtttctttgtttactttatgctttcatggacttcttttaataaaatat
>KrE10_cox3
attaatacagaattattattttcttatctgtatctattggtattttggaaatcattacat
ttagaagttacatcagatttattttgttttcttattaatactcaccatttattaatcttt
gctcttattataattctatttatctttacaggatttaatatttattgttggactggaatt
tacttctcatagaattattttcttatattatttcatcttaattttggattaatttatagt
tttcatatttggtctagagatttattaagagaattcactaaaaaatatgaagtcttatta
ataatctttttcttctttttgggggttttcttgtttctgaagctctattatttgtatcc
ttcttttggacatcttttcatttattatcttctccaactttagggatgtgggtgtgggaa
ggtttctatctggaggatccttgtgaattaacttttgctaatacacttcttttatcaaat
gctgctatatctttaggaaatgcttttattaatttagaaatttcatcagaatatattatt
ttcttcactttatggtcattctttttgtcttctctttttattagtttgcagattaaagaa
tttcgcattctcgcattatcaattaatgattcactttatagttctcttttcttttttctt
acaggattacatttctttcatctatctattggtcttattcttctaatttatttctattat
gtgggtttctttgtttactttatgctttcatggacttcttttaataaaatat
>KrE11_cox3
attaatacagaattattattttcttatctgtatctattggtattttggaaatcattacat
ttagaagttacatcagatttattttgttttcttattaatactcaccatttattaatcttt
gctcttattataattctatttatctttacaggatttaatatttattgttggactggaatt
tacttctcatagaattattttcttatattatttcatcttaattttggattaatttatagt
tttcatatttggtctagagatttattaagagaattcactaaaaaatatgaagtcttatta
ataatctttttcttctttttgggggttttcttgtttctgaagctctattatttgtatcc
ttcttttggacatcttttcatttattatcttctccaactttagggatgtgggtgtgggaa
ggtttctatctggaggatccttgtgaattaacttttgctaatacacttcttttatcaaat
gctgctatatctttaggaaatgcttttattaatttagaaatttcatcagaatatattatt
ttcttcactttatggtcattctttttgtcttctctttttattagtttgcagattaaagaa
tttcgcattctcgcattatcaattaatgattcactttatagttctcttttcttttttctt
acaggattacatttctttcatctatctattggtcttattcttctaatttatttctattat
gtgggtttctttgtttactttatgctttcatggacttcttttaataaaatat
>KrE12_cox3
attaatacagaattattattttcttatctgtatctattggtattttggaaatcattacat
ttagaagttacatcagatttattttgttttcttattaatactcaccatttattaatcttt
gctcttattataattctatttatctttacaggatttaatatttattgttggactggaatt
tacttctcatagaattattttcttatattatttcatcttaattttggattaatttatagt
tttcatatttggtctagagatttattaagagaattcactaaaaaatatgaagtcttatta
ataatctttttcttctttttgggggttttcttgtttctgaagctctattatttgtatcc
ttcttttggacatcttttcatttattatcttctccaactttagggatgtgggtgtgggaa
ggtttctatctggaggatccttgtgaattaacttttgctaatacacttcttttatcaaat
gctgctatatctttaggaaatgcttttattaatttagaaatttcatcagaatatattatt
ttcttcactttatggtcattctttttgtcttctctttttattagtttgcagattaaagaa
tttcgcattctcgcattatcaattaatgattcactttatagttctcttttcttttttctt
acaggattacatttctttcatctatctattggtcttattcttctaatttatttctattat
gtgggtttctttgtttactttatgctttcatggacttcttttaataaaatat
>KrE13_cox3
attaatacagaattattattttcttatctgtatctattggtattttggaaatcattacat
ttagaagttacatcagatttattttgttttcttattaatactcaccatttattaatcttt
gctcttattataattctatttatctttacaggatttaatatttattgttggactggaatt
tacttctcatagaattattttcttatattatttcatcttaattttggattaatttatagt
tttcatatttggtctagagatttattaagagaattcactaaaaaatatgaagtcttatta
ataatctttttcttctttttgggggttttcttgtttctgaagctctattatttgtatcc
ttcttttggacatcttttcatttattatcttctccaactttagggatgtgggtgtgggaa
ggtttctatctggaggatccttgtgaattaacttttgctaatacacttcttttatcaaat
gctgctatatctttaggaaatgcttttattaatttagaaatttcatcagaatatattatt

ttcttcactttatggtcattctttttgtcttctcttttattagtttgcagattaaagaa
tttcgcattctcgcattatcaattaatgattcactttatagttctcttttctttttttctt
acaggattacatttctttcatctatctattggtcttattcttctaatttatttctattat
gtgggtttctttgtttactttatgctttcatggacttcttttaataaaatat
>KrE14_cox3
attaatacagaattattattttcttatctgtatctattggtattttggaaatcattacat
ttagaagttacatcagatttattttgttttcttattaatactcaccatttattaatcttt
gctcttattataattctatttatctttacaggatttaatatttattgttggactggaatt
tacttctcatagaattattttcttatattatttcatcttaattttggattaatttatagt
tttcatatttggtctagagatttattaagagaattcactaaaaaaatatgaagtcttatta
ataatctttttcttctttttggggggttttcttgtttctgaagctctattatttgtatcc
ttcttttggacatcttttcatttattatcttctccaactttagggatgtgggtgtgggaa
ggtttctatctggaggatccttgtgaattaacttttgctaatacacttcttttatcaaat
gctgctatatctttaggaaatgcttttattaatttagaaatttcatcagaatatattatt
ttcttcactttatggtcattctttttgtcttctcttttattagtttgcagattaaagaa
tttcgcattctcgcattatcaattaatgattcactttatagttctcttttctttttttctt
acaggattacatttctttcatctatctattggtcttattcttctaatttatttctattat
gtgggtttctttgtttactttatgctttcatggacttcttttaataaaatat
>KrE15_cox3
attaatacagaattattattttcttatctgtatctattggtattttggaaatcattacat
ttagaagttacatcagatttattttgttttcttattaatactcaccatttattaatcttt
gctcttattataattctatttatctttacaggatttaatatttattgttggactggaatt
tacttctcatagaattattttcttatattatttcatcttaattttggattaatttatagt
tttcatatttggtctagagatttattaagagaattcactaaaaaaatatgaagtcttatta
ataatctttttcttctttttggggggttttcttgtttctgaagctctattatttgtatcc
ttcttttggacatcttttcatttattatcttctccaactttagggatgtgggtgtgggaa
ggtttctatctggaggatccttgtgaattaacttttgctaatacacttcttttatcaaat
gctgctatatctttaggaaatgcttttattaatttagaaatttcatcagaatatattatt
ttcttcactttatggtcattctttttgtcttctcttttattagtttgcagattaaagaa
tttcgcattctcgcattatcaattaatgattcactttatagttctcttttctttttttctt
acaggattacatttctttcatctatctattggtcttattcttctaatttatttctattat
gtgggtttctttgtttactttatgctttcatggacttcttttaataaaatat
>KrE2_cox3
attaatacagaattattattttcttatctgtatctattggtattttggaaatcattacat
ttagaagttacatcagatttattttgttttcttattaatactcaccatttattaatcttt
gctcttattataattctatttatctttacaggatttaatatttattgttggactggaatt
tacttctcatagaattattttcttatattatttcatcttaattttggattaatttatagt
tttcatatttggtctagagatttattaagagaattcactaaaaaaatatgaagtcttatta
ataatctttttcttctttttggggggttttcttgtttctgaagctctattatttgtatcc
ttcttttggacatcttttcatttattatcttctccaactttagggatgtgggtgtgggaa
ggtttctatctggaggatccttgtgaattaacttttgctaatacacttcttttatcaaat
gctgctatatctttaggaaatgcttttattaatttagaaatttcatcagaatatattatt
ttcttcactttatggtcattctttttgtcttctcttttattagtttgcagattaaagaa
tttcgcattctcgcattatcaattaatgattcactttatagttctcttttctttttttctt
acaggattacatttctttcatctatctattggtcttattcttctaatttatttctattat
gtgggtttctttgtttactttatgctttcatggacttcttttaataaaatat
>KrE3_cox3
attaatacagaattattattttcttatctgtatctattggtattttggaaatcattacat
ttagaagttacatcagatttattttgttttcttattaatactcaccatttattaatcttt
gctcttattataattctatttatctttacaggatttaatatttattgttggactggaatt
tacttctcatagaattattttcttatattatttcatcttaattttggattaatttatagt
tttcatatttggtctagagatttattaagagaattcactaaaaaaatatgaagtcttatta
ataatctttttcttctttttggggggttttcttgtttctgaagctctattatttgtatcc
ttcttttggacatcttttcatttattatcttctccaactttagggatgtgggtgtgggaa
ggtttctatctggaggatccttgtgaattaacttttgctaatacacttcttttatcaaat
gctgctatatctttaggaaatgcttttattaatttagaaatttcatcagaatatattatt
ttcttcactttatggtcattctttttgtcttctcttttattagtttgcagattaaagaa

tttcgcattctcgcattatcaattaatgattcactttatagttctcttttcttttttctt
acaggattacatttctttcatctatctattggtcttattcttctaatttatttctattat
gtgggtttctttgtttactttatgctttcatggacttcttttaataaaatat
>KrE4_cox3
attaatacagaattattattttcttatctgtatctattggtattttggaaatcattacat
ttagaagttacatcagatttattttgttttcttattaatactcaccatttattaatcttt
gctcttattataattctatttatctttacaggatttaatatttattgttggactggaatt
tacttctcatagaattattttcttatattatttcatcttaattttggattaatttatagt
tttcatatttggtctagagatttattaagagaattcactaaaaaatatgaagtcttatta
ataatcttttttcttctttttgggggttttcttgtttctgaagctctattatttgtatcc
ttcttttggacatcttttcatttattatcttctccaactttagggatgtgggtgtgggaa
ggtttctatctggaggatccttgtgaattaacttttgctaatacacttcttttatcaaat
gctgctatatctttaggaaatgcttttattaatttagaaatttcatcagaatatattatt
ttcttcactttatggtcattctttttgtcttctctttttattagtttgcagattaaagaa
tttcgcattctcgcattatcaattaatgattcactttatagttctcttttcttttttctt
acaggattacatttctttcatctatctattggtcttattcttctaatttatttctattat
gtgggtttctttgtttactttatgctttcatggacttcttttaataaaatat
>KrE5_cox3
attaatacagaattattattttcttatctgtatctattggtattttggaaatcattacat
ttagaagttacatcagatttattttgttttcttattaatactcaccatttattaatcttt
gctcttattataattctatttatctttacaggatttaatatttattgttggactggaatt
tacttctcatagaattattttcttatattatttcatcttaattttggattaatttatagt
tttcatatttggtctagagatttattaagagaattcactaaaaaatatgaagtcttatta
ataatcttttttcttctttttgggggttttcttgtttctgaagctctattatttgtatcc
ttcttttggacatcttttcatttattatcttctccaactttagggatgtgggtgtgggaa
ggtttctatctggaggatccttgtgaattaacttttgctaatacacttcttttatcaaat
gctgctatatctttaggaaatgcttttattaatttagaaatttcatcagaatatattatt
ttcttcactttatggtcattctttttgtcttctctttttattagtttgcagattaaagaa
tttcgcattctcgcattatcaattaatgattcactttatagttctcttttcttttttctt
acaggattacatttctttcatctatctattggtcttattcttctaatttatttctattat
gtgggtttctttgtttactttatgctttcatggacttcttttaataaaatat
>KrE6_cox3
attaatacagaattattattttcttatctgtatctattggtattttggaaatcattacat
ttagaagttacatcagatttattttgttttcttattaatactcaccatttattaatcttt
gctcttattataattctatttatctttacaggatttaatatttattgttggactggaatt
tacttctcatagaattattttcttatattatttcatcttaattttggattaatttatagt
tttcatatttggtctagagatttattaagagaattcactaaaaaatatgaagtcttatta
ataatcttttttcttctttttgggggttttcttgtttctgaagctctattatttgtatcc
ttcttttggacatcttttcatttattatcttctccaactttagggatgtgggtgtgggaa
ggtttctatctggaggatccttgtgaattaacttttgctaatacacttcttttatcaaat
gctgctatatctttaggaaatgcttttattaatttagaaatttcatcagaatatattatt
ttcttcactttatggtcattctttttgtcttctctttttattagtttgcagattaaagaa
tttcgcattctcgcattatcaattaatgattcactttatagttctcttttcttttttctt
acaggattacatttctttcatctatctattggtcttattcttctaatttatttctattat
gtgggtttctttgtttactttatgctttcatggacttcttttaataaaatat
>KrE7_cox3
attaatacagaattattattttcttatctgtatctattggtattttggaaatcattacat
ttagaagttacatcagatttattttgttttcttattaatactcaccatttattaatcttt
gctcttattataattctatttatctttacaggatttaatatttattgttggactggaatt
tacttctcatagaattattttcttatattatttcatcttaattttggattaatttatagt
tttcatatttggtctagagatttattaagagaattcactaaaaaatatgaagtcttatta
ataatcttttttcttctttttgggggttttcttgtttctgaagctctattatttgtatcc
ttcttttggacatcttttcatttattatcttctccaactttagggatgtgggtgtgggaa
ggtttctatctggaggatccttgtgaattaacttttgctaatacacttcttttatcaaat
gctgctatatctttaggaaatgcttttattaatttagaaatttcatcagaatatattatt
ttcttcactttatggtcattctttttgtcttctctttttattagtttgcagattaaagaa
tttcgcattctcgcattatcaattaatgattcactttatagttctcttttcttttttctt

acaggattacatttctttcatctatctattggtcttattcttctaatttatttctattat
gtgggtttctttgtttactttatgctttcatggacttcttttaataaaatat
>KrE8_cox3
attaatacagaattattattttcttatctgtatctattggtattttggaaatcattacat
ttagaagttacatcagatttattttgttttcttattaatactcaccatttattaatcttt
gctcttattataattctatttatctttacaggatttaatatttattgttggactggaatt
tacttctcatagaattattttcttatattatttcatcttaattttggattaatttatagt
tttcatatttggtctagagatttattaagagaattcactaaaaaatatgaagtcttatta
ataatctttttcttctttttgggggttttcttgtttctgaagctctattatttgtatcc
ttcttttggacatcttttcatttattatcttctccaactttagggatgtgggtgtgggaa
ggtttctatctggaggatccttgtgaattaacttttgctaatacacttcttttatcaaat
gctgctatatctttaggaaatgcttttattaatttagaaatttcatcagaatatattatt
ttcttcactttatggtcattctttttgtcttctctttttattagtttgcagattaaagaa
tttcgcattctcgcattatcaattaatgattcactttatagttctcttttctttttctt
acaggattacatttctttcatctatctattggtcttattcttctaatttatttctattat
gtgggtttctttgtttactttatgctttcatggacttcttttaataaaatat
>KrE9_cox3
attaatacagaattattattttcttatctgtatctattggtattttggaaatcattacat
ttagaagttacatcagatttattttgttttcttattaatactcaccatttattaatcttt
gctcttattataattctatttatctttacaggatttaatatttattgttggactggaatt
tacttctcatagaattattttcttatattatttcatcttaattttggattaatttatagt
tttcatatttggtctagagatttattaagagaattcactaaaaaatatgaagtcttatta
ataatctttttcttctttttgggggttttcttgtttctgaagctctattatttgtatcc
ttcttttggacatcttttcatttattatcttctccaactttagggatgtgggtgtgggaa
ggtttctatctggaggatccttgtgaattaacttttgctaatacacttcttttatcaaat
gctgctatatctttaggaaatgcttttattaatttagaaatttcatcagaatatattatt
ttcttcactttatggtcattctttttgtcttctctttttattagtttgcagattaaagaa
tttcgcattctcgcattatcaattaatgattcactttatagttctcttttctttttctt
acaggattacatttctttcatctatctattggtcttattcttctaatttatttctattat
gtgggtttctttgtttactttatgctttcatggacttcttttaataaaatat
>Mz10_cox3
attaatacagaattattattttcttatctgtatctattggtattttggaaatcattacat
ttagaagttacatcagatttattttgttttcttattaatactcaccatttattaatcttt
gctcttattataattctatttatctttacaggatttaatatttattgttggactggaatt
tacttctcatagaattattttcttatattatttcatcttaattttggattaatttatagt
tttcatatttggtctagagatttattaagagaattcactaaaaaatatgaagtcttatta
ataatctttttcttctttttgggggttttcttgtttctgaagctctattatttgtatcc
ttcttttggacatcttttcatttattatcttctccaactttagggatgtgggtgtgggaa
ggtttctatctggaggatccttgtgaattaacttttgctaatacacttcttttatcaaat
gctgctatatctttaggaaatgcttttattaatttagaaatttcatcagaatatattatt
ttcttcactttatggtcattctttttgtcttctctttttattagtttgcagattaaagaa
tttcgcattctcgcattatcaattaatgattcactttatagttctcttttctttttctt
acaggattacatttctttcatctatctattggtcttattcttctaatttatttctattat
gtgggtttctttgtttactttatgctttcatggacttcttttaataaaatat
>Mz5_cox3
attaatacagaattattattttcttatctgtatctattggtattttggaaatcattacat
ttagaagttacatcagatttattttgttttcttattaatactcaccatttattaatcttt
gctcttattataattctatttatctttacaggatttaatatttattgttggactggaatt
tacttctcatagaattattttcttatattatttcatcttaattttggattaatttatagt
tttcatatttggtctagagatttattaagagaattcactaaaaaatatgaagtcttatta
ataatctttttcttctttttgggggttttcttgtttctgaagctctattatttgtatcc
ttcttttggacatcttttcatttattatcttctccaactttagggatgtgggtgtgggaa
ggtttctatctggaggatccttgtgaattaacttttgctaatacacttcttttatcaaat
gctgctatatctttaggaaatgcttttattaatttagaaatttcatcagaatatattatt
ttcttcactttatggtcattctttttgtcttctctttttattagtttgcagattaaagaa
tttcgcattctcgcattatcaattaatgattcactttatagttctcttttctttttctt
acaggattacatttctttcatctatctattggtcttattcttctaatttatttctattat

gtgggtttctttgtttactttatgctttcatggacttcttttaataaaatat
>Mz6_cox3
attaatacagaattattattttcttatctgtatctattggtattttggaaatcattacat
ttagaagttacatcagatttattttgttttcttattaatactcaccatttattaatcttt
gctcttattataattctatttatctttacaggatttaatatttattgttggactggaatt
tacttctcatagaattattttcttatattatttcatcttaattttggattaatttatagt
tttcatatttggtctagagatttattaagagaattcactaaaaaatatgaagtcttatta
ataatctttttcttctttttggggggttttcttgtttctgaagctctattatttgtatcc
ttcttttggacatcttttcatttattatcttctccaactttagggatgtgggtgtgggaa
ggtttctatctggaggatccttgtgaattaacttttgctaatacacttcttttatcaaat
gctgctatatctttaggaaatgcttttattaatttagaaatttcatcagaatatattatt
ttcttcactttatggtcattctttttgtcttctctttttattagtttgcagattaaagaa
tttcgcattctcgcattatcaattaatgattcactttatagttctcttttcttttttctt
acaggattacatttctttcatctatctattggtcttattcttctaatttatttctattat
gtgggtttctttgtttactttatgctttcatggacttcttttaataaaatat
>Mz7_cox3
attaatacagaattattattttcttatctgtatctattggtattttggaaatcattacat
ttagaagttacatcagatttattttgttttcttattaatactcaccatttattaatcttt
gctcttattataattctatttatctttacaggatttaatatttattgttggactggaatt
tacttctcatagaattattttcttatattatttcatcttaattttggattaatttatagt
tttcatatttggtctagagatttattaagagaattcactaaaaaatatgaagtcttatta
ataatctttttcttctttttggggggttttcttgtttctgaagctctattatttgtatcc
ttcttttggacatcttttcatttattatcttctccaactttagggatgtgggtgtgggaa
ggtttctatctggaggatccttgtgaattaacttttgctaatacacttcttttatcaaat
gctgctatatctttaggaaatgcttttattaatttagaaatttcatcagaatatattatt
ttcttcactttatggtcattctttttgtcttctctttttattagtttgcagattaaagaa
tttcgcattctcgcattatcaattaatgattcactttatagttctcttttcttttttctt
acaggattacatttctttcatctatctattggtcttattcttctaatttatttctattat
gtgggtttctttgtttactttatgctttcatggacttcttttaataaaatat
>Mz8_cox3
attaatacagaattattattttcttatctgtatctattggtattttggaaatcattacat
ttagaagttacatcagatttattttgttttcttattaatactcaccatttattaatcttt
gctcttattataattctatttatctttacaggatttaatatttattgttggactggaatt
tacttctcatagaattattttcttatattatttcatcttaattttggattaatttatagt
tttcatatttggtctagagatttattaagagaattcactaaaaaatatgaagtcttatta
ataatctttttcttctttttggggggttttcttgtttctgaagctctattatttgtatcc
ttcttttggacatcttttcatttattatcttctccaactttagggatgtgggtgtgggaa
ggtttctatctggaggatccttgtgaattaacttttgctaatacacttcttttatcaaat
gctgctatatctttaggaaatgcttttattaatttagaaatttcatcagaatatattatt
ttcttcactttatggtcattctttttgtcttctctttttattagtttgcagattaaagaa
tttcgcattctcgcattatcaattaatgattcactttatagttctcttttcttttttctt
acaggattacatttctttcatctatctattggtcttattcttctaatttatttctattat
gtgggtttctttgtttactttatgctttcatggacttcttttaataaaatat
>Mz9_cox3
attaatacagaattattattttcttatctgtatctattggtattttggaaatcattacat
ttagaagttacatcagatttattttgttttcttattaatactcaccatttattaatcttt
gctcttattataattctatttatctttacaggatttaatatttattgttggactggaatt
tacttctcatagaattattttcttatattatttcatcttaattttggattaatttatagt
tttcatatttggtctagagatttattaagagaattcactaaaaaatatgaagtcttatta
ataatctttttcttctttttggggggttttcttgtttctgaagctctattatttgtatcc
ttcttttggacatcttttcatttattatcttctccaactttagggatgtgggtgtgggaa
ggtttctatctggaggatccttgtgaattaacttttgctaatacacttcttttatcaaat
gctgctatatctttaggaaatgcttttattaatttagaaatttcatcagaatatattatt
ttcttcactttatggtcattctttttgtcttctctttttattagtttgcagattaaagaa
tttcgcattctcgcattatcaattaatgattcactttatagttctcttttcttttttctt
acaggattacatttctttcatctatctattggtcttattcttctaatttatttctattat
gtgggtttctttgtttactttatgctttcatggacttcttttaataaaatat

>MzC1_cox3
attaatacagaattattattttcttatctgtatctattggtattttggaaatcattacat
ttagaagttacatcagatttattttgttttcttattaatactcaccatttattaatcttt
gctcttattataattctatttatctttacaggatttaatatttattgttggactggaatt
tacttctcatagaattattttcttatattatttcatcttaattttggattaatttatagt
tttcatatttggtctagagatttattaagagaattcactaaaaaatatgaagtcttatta
ataatcttttttcttctttttgggggttttcttgtttctgaagctctattatttgtatcc
ttcttttggacatcttttcatttattatcttctccaactttagggatgtgggtgtgggaa
ggtttctatctggaggatccttgtgaattaacttttgctaatacacttcttttatcaaat
gctgctatatctttaggaaatgcttttattaatttagaaatttcatcagaatatattatt
ttcttcactttatggtcattctttttgtcttctctttttattagtttgcagattaaagaa
tttcgcattctcgcattatcaattaatgattcactttatagttctcttttcttttttctt
acaggattacatttctttcatctatctattggtcttattcttctaatttatttctattat
gtgggtttctttgtttactttatgctttcatggacttcttttaataaaatat
>MzC1GaII_cox3
attaatacagaattattattttcttatctgtatctattggtattttggaaatcattacat
ttagaagttacatcagatttattttgttttcttattaatactcaccatttattaatcttt
gctcttattataattctatttatctttacaggatttaatatttattgttggactggaatt
tacttctcatagaattattttcttatattatttcatcttaattttggattaatttatagt
tttcatatttggtctagagatttattaagagaattcactaaaaaatatgaagtcttatta
ataatcttttttcttctttttgggggttttcttgtttctgaagctctattatttgtatcc
ttcttttggacatcttttcatttattatcttctccaactttagggatgtgggtgtgggaa
ggtttctatctggaggatccttgtgaattaacttttgctaatacacttcttttatcaaat
gctgctatatctttaggaaatgcttttattaatttagaaatttcatcagaatatattatt
ttcttcactttatggtcattctttttgtcttctctttttattagtttgcagattaaagaa
tttcgcattctcgcattatcaattaatgattcactttatagttctcttttcttttttctt
acaggattacatttctttcatctatctattggtcttattcttctaatttatttctattat
gtgggtttctttgtttactttatgctttcatggacttcttttaataaaatat
>MzC2_cox3
attaatacagaattattattttcttatctgtatctattggtattttggaaatcattacat
ttagaagttacatcagatttattttgttttcttattaatactcaccatttattaatcttt
gctcttattataattctatttatctttacaggatttaatatttattgttggactggaatt
tacttctcatagaattattttcttatattatttcatcttaattttggattaatttatagt
tttcatatttggtctagagatttattaagagaattcactaaaaaatatgaagtcttatta
ataatcttttttcttctttttgggggttttcttgtttctgaagctctattatttgtatcc
ttcttttggacatcttttcatttattatcttctccaactttagggatgtgggtgtgggaa
ggtttctatctggaggatccttgtgaattaacttttgctaatacacttcttttatcaaat
gctgctatatctttaggaaatgcttttattaatttagaaatttcatcagaatatattatt
ttcttcactttatggtcattctttttgtcttctctttttattagtttgcagattaaagaa
tttcgcattctcgcattatcaattaatgattcactttatagttctcttttcttttttctt
acaggattacatttctttcatctatctattggtcttattcttctaatttatttctattat
gtgggtttctttgtttactttatgctttcatggacttcttttaataaaatat
>MzC2GaII_cox3
attaatacagaattattattttcttatctgtatctattggtattttggaaatcattacat
ttagaagttacatcagatttattttgttttcttattaatactcaccatttattaatcttt
gctcttattataattctatttatctttacaggatttaatatttattgttggactggaatt
tacttctcatagaattattttcttatattatttcatcttaattttggattaatttatagt
tttcatatttggtctagagatttattaagagaattcactaaaaaatatgaagtcttatta
ataatcttttttcttctttttgggggttttcttgtttctgaagctctattatttgtatcc
ttcttttggacatcttttcatttattatcttctccaactttagggatgtgggtgtgggaa
ggtttctatctggaggatccttgtgaattaacttttgctaatacacttcttttatcaaat
gctgctatatctttaggaaatgcttttattaatttagaaatttcatcagaatatattatt
ttcttcactttatggtcattctttttgtcttctctttttattagtttgcagattaaagaa
tttcgcattctcgcattatcaattaatgattcactttatagttctcttttcttttttctt
acaggattacatttctttcatctatctattggtcttattcttctaatttatttctattat
gtgggtttctttgtttactttatgctttcatggacttcttttaataaaatat
>MzC3_cox3

attaatacagaattattattttcttatctgtatctattggtattttggaaatcattacat
ttagaagttacatcagatttattttgttttcttattaatactcaccatttattaatcttt
gctcttattataattctatttatctttacaggatttaatatttattgttggactggaatt
tacttctcatagaattattttcttatattatttcatcttaattttggattaatttatagt
tttcatatttggtctagagatttattaagagaattcactaaaaaatatgaagtcttatta
ataatcttttttcttcttttttgggggttttcttgtttctgaagctctattatttgtatcc
ttcttttggacatcttttcatttattatcttctccaactttagggatgtgggtgtgggaa
ggtttctatctggaggatccttgtgaattaacttttgctaatacacttcttttatcaaat
gctgctatatctttaggaaatgcttttattaatttagaaatttcatcagaatatattatt
ttcttcactttatggtcattctttttgtcttctctttttattagtttgcagattaaagaa
tttcgcattctcgcattatcaattaatgattcactttatagttctcttttcttttttctt
acaggattacatttctttcatctatctattggtcttattcttctaatttatttctattat
gtgggtttctttgtttactttatgctttcatggacttcttttaataaaatat
>MzC4_cox3
attaatacagaattattattttcttatctgtatctattggtattttggaaatcattacat
ttagaagttacatcagatttattttgttttcttattaatactcaccatttattaatcttt
gctcttattataattctatttatctttacaggatttaatatttattgttggactggaatt
tacttctcatagaattattttcttatattatttcatcttaattttggattaatttatagt
tttcatatttggtctagagatttattaagagaattcactaaaaaatatgaagtcttatta
ataatcttttttcttcttttttgggggttttcttgtttctgaagctctattatttgtatcc
ttcttttggacatcttttcatttattatcttctccaactttagggatgtgggtgtgggaa
ggtttctatctggaggatccttgtgaattaacttttgctaatacacttcttttatcaaat
gctgctatatctttaggaaatgcttttattaatttagaaatttcatcagaatatattatt
ttcttcactttatggtcattctttttgtcttctctttttattagtttgcagattaaagaa
tttcgcattctcgcattatcaattaatgattcactttatagttctcttttcttttttctt
acaggattacatttctttcatctatctattggtcttattcttctaatttatttctattat
gtgggtttctttgtttactttatgctttcatggacttcttttaataaaatat
>Od10_cox3
attaatacagaattattattttcttatctgtatctattggtattttggaaatcattacat
ttagaagttacatcaggttcattttgtcttcttattaatactcaccatttattaatcttt
gctcttattataattctatttatctttacaggatttaatatttattgttgggctggaatt
tacttctcatagagttattttcttatattatttcatcttaattttggattaatttatagt
tttcatatttggtctagagatttattaagagaattcactaaaaaatatgaagtcttatta
ataatcttttttcttcttttttgggggttttcttgtttctgaagctctattatttgtatcc
ttcttttggacatcttttcatttattatcttctccaactttagggatgtgggtgtgggaa
ggtttctatctggaggatccttgtgaattaacttttgctaatacacttcttttatcaaat
gctgctatatctttaggaaatgcttttattaatttagaaatttcatcagaatatattatt
ttcttcactttatggtcattctttttgtcttctctttttattagtttgcagattaaagaa
tttcgcattctcgcattatcaattaatgattcactttatagttctcttttcttttttctt
acaggattacatttctttcatctatctattggtcttattcttctaatttatttctattat
gtgggtttctttgtttactttatgctttcatggacttcttttaataaaatat
>Od11_cox3
attaatacagaattattattttcttatctgtatctattggtattttggaaatcattacat
ttagaagttacatcagatttattttgttttcttattaatactcaccatttattaatcttt
gctcttattataattctatttatctttacaggatttaatatttattgttggactggaatt
tacttctcatagaattattttcttatattatttcatcttaattttggattaatttatagt
tttcatatttggtctagagatttattaagagaattcactaaaaaatatgaagtcttatta
ataatcttttttcttcttttttgggggttttcttgtttctgaagctctattatttgtatcc
ttcttttggacatcttttcatttattatcttctccaactttagggatgtgggtgtgggaa
ggtttctatctggaggatccttgtgaattaacttttgctaatacacttcttttatcaaat
gctgctatatctttaggaaatgcttttattaatttagaaatttcatcagaatatattatt
ttcttcactttatggtcattctttttgtcttctctttttattagtttgcagattaaagaa
tttcgcattctcgcattatcaattaatgattcactttatagttctcttttcttttttctt
acaggattacatttctttcatctatctattggtcttattcttctaatttatttctattat
gtgggtttctttgtttactttatgctttcatggacttcttttaataaaatat
>Od12_cox3
attaatacagaattattattttcttatctgtatctattggtattttggaaatcattacat

ttagaagttacatcagatttattttgttttcttattaatactcaccatttattaatcttt
gctcttattataattctatttatctttacaggatttaatatttattgttggactggaatt
tacttctcatagaattattttcttatattatttcatcttaattttggattaatttatagt
tttcatatttggtctagagatttattaagagaattcactaaaaaaatatgaagtcttatta
ataatcttttttcttctttttggggggttttcttgtttctgaagctctattatttgtatcc
ttcttttggacatcttttcatttattatcttctccaactttagggatgtgggtgtgggaa
ggtttctatctggaggatccttgtgaattaacttttgctaatacacttcttttatcaaat
gctgctatatctttaggaaatgcttttattaatttagaaatttcatcagaatatattatt
ttcttcactttatggtcattctttttgtcttctctttttattagtttgcagattaaagaa
tttcgcattctcgcattatcaattaatgattcactttatagttctcttttcttttttctt
acaggattacatttctttcatctatctattggtcttattcttctaatttatttctattat
gtgggtttctttgtttactttatgctttcatggacttcttttaataaaaatat
>Od13_cox3
attaatacagaattattattttcttatctgtatctattggtattttggaaatcattacat
ttagaagttacatcagatttattttgttttcttattaatactcaccatttattaatcttt
gctcttattataattctatttatctttacaggatttaatatttattgttggactggaatt
tacttctcatagaattattttcttatattatttcatcttaattttggattaatttatagt
tttcatatttggtctagagatttattaagagaattcactaaaaaaatatgaagtcttatta
ataatcttttttcttctttttggggggttttcttgtttctgaagctctattatttgtatcc
ttcttttggacatcttttcatttattatcttctccaactttagggatgtgggtgtgggaa
ggtttctatctggaggatccttgtgaattaacttttgctaatacacttcttttatcaaat
gctgctatatctttaggaaatgcttttattaatttagaaatttcatcagaatatattatt
ttcttcactttatggtcattctttttgtcttctctttttattagtttgcagattaaagaa
tttcgcattctcgcattatcaattaatgattcactttatagttctcttttcttttttctt
acaggattacatttctttcatctatctattggtcttattcttctaatttatttctattat
gtgggtttctttgtttactttatgctttcatggacttcttttaataaaaatat
>Od14_cox3
attaatacagaattattattttcttatctgtatctattggtattttggaaatcattacat
ttagaagttgcatcaggttcattttgtcttcttattaatgctcactatttattaatcttt
gctcttgttgtaattctatttatctttacaggatttaatatttattgttggactggaatt
tacttctcatagaattattttcttatattatttcatcttaattttggattaatttatagt
tttcatatttggtctagagatttattaagagaattcactaaaaaaatatgaagtcttatta
ataatcttttttcttctttttggggggttttcttgtttctgaagctctattatttgtatcc
ttcttttggacatcttttcatttattatcttctccaactttagggatgtgggtgtgggaa
ggtttctatctggaggatccttgtgaattaacttttgctaatacacttcttttatcaaat
gctgctatatctttaggaaatgcttttattaatttagaaatttcatcagaatatattatt
ttcttcactttatggtcattctttttgtcttctctttttattagtttgcagattaaagaa
tttcgcattctcgcattatcaattaatgattcactttatagttctcttttcttttttctt
acaggattacatttctttcatctatctattggtcttattcttctaatttatttctattat
gtgggtttctttgtttactttatgctttcatggacttcttttaataaaaatat
>Od15_cox3
attaatacagaattattattttcttatctgtatctattggtattttggaaatcattacat
ttagaagttacatcagatttattttgttttcttattaatactcaccatttattaatcttt
gctcttattataattctatttatctttacaggatttaatatttattgttggactggaatt
tacttctcatagaattattttcttatattatttcatcttaattttggattaatttatagt
tttcatatttggtctagagatttattaagagaattcactaaaaaaatatgaagtcttatta
ataatcttttttcttctttttggggggttttcttgtttctgaagctctattatttgtatcc
ttcttttggacatcttttcatttattatcttctccaactttagggatgtgggtgtgggaa
ggtttctatctggaggatccttgtgaattaacttttgctaatacacttcttttatcaaat
gctgctatatctttaggaaatgcttttattaatttagaaatttcatcagaatatattatt
ttcttcactttatggtcattctttttgtcttctctttttattagtttgcagattaaagaa
tttcgcattctcgcattatcaattaatgattcactttatagttctcttttcttttttctt
acaggattacatttctttcatctatctattggtcttattcttctaatttatttctattat
gtgggtttctttgtttactttatgctttcatggacttcttttaataaaaatat
>Od18_cox3
attaatacagaattattattttattatctgtatctattggtattttggaaatcattacat
ttagaagttacatcagatttattttgttttcttattaatactcaccatttattaatcttt

gctcttattataattctatttatctttacaggatttaatatttattgttggactggaatt
tacttctcatagaattattttcttatattatttcatcttaattttggattaatttatagt
tttcatatttggtctagagatttattaagagaattcactaaaaaatatgaagtcttatta
ataatcttttttcttctttttggggggttttcttgtttctgaagctctattatttgtatcc
ttcttttggacatcttttcatttattatcttctccaactttagggatgtgggtgtgggaa
ggtttctatctggaggatccttgtgaattaacttttgctaatacacttcttttatcaaat
gctgctatatctttaggaaatgcttttattaatttagaaatttcatcagaatatattatt
ttcttcactttatggtcattctttttgtcttctctttttattagtttgcagattaaagaa
tttcgcattctcgcattatcaattaatgattcactttatagttctcttttcttttttctt
acaggattacatttctttcatctatctattggtcttattcttctaatttatttctattat
gtgggtttctttgtttactttatgctttcatggacttcttttaataaaatat
>Od19_cox3
attaatacagaattattattttcttatctgtatctattggtattttggaaatcattacat
ttagaagttacatcaggttcattttgttttcttattaatactcaccatttattaatcttt
gctcttattataattctatttatctttacaggatttaatatttattgttggactggaatt
tacttctcatagaattattttcttatattatttcatcttaattttggattaatttatagt
tttcatatttggtctagagatttattaagagaattcactaaaaaatatgaagtcttatta
ataatcttttttcttctttttggggggttttcttgtttctgaagctctattatttgtatcc
ttcttttggacatcttttcatttattatcttctccaactttagggatgtgggtgtgggaa
ggtttctatctggaggatccttgtgaattaacttttgctaatacacttcttttatcaaat
gctgctatatctttaggaaatgcttttattaatttagaaatttcatcagaatatattatt
ttcttcactttatggtcattctttttgtcttctctttttattagtttgcagattaaagaa
tttcgcattctcgcattatcaattaatgattcactttatagttctcttttcttttttctt
acaggattacatttctttcatctatctattggtcttattcttctaatttatttctattat
gtgggtttctttgtttactttatgctttcatggacttcttttaataaaatat
>Od2_cox3
attaatacagaattattattttcttatctgtatctattggtattttggaaatcattacat
ttagaagttacatcagatttattttgttttcttattaatactcaccatttattaatcttt
gctcttattataattctatttatctttacaggatttaatatttattgttggactggaatt
tacttctcatagaattattttcttatattatttcatcttaattttggattaatttatagt
tttcatatttggtctagagatttattaagagaattcactaaaaaatatgaagtcttatta
ataatcttttttcttctttttggggggttttcttgtttctgaagctctattatttgtatcc
ttcttttggacatcttttcatttattatcttctccaactttagggatgtgggtgtgggaa
ggtttctatctggaggatccttgtgaattaacttttgctaatacacttcttttatcaaat
gctgctatatctttaggaaatgcttttattaatttagaaatttcatcagaatatattatt
ttcttcactttatggtcattctttttgtcttctctttttattagtttgcagattaaagaa
tttcgcattctcgcattatcaattaatgattcactttatagttctcttttcttttttctt
acaggattacatttctttcatctatctattggtcttattcttctaatttatttctattat
gtgggtttctttgtttactttatgctttcatggacttcttttaataaaatat
>Od22_cox3
attaatacagaattattattttcttatctgtatctattggtattttggaaatcattacat
ttagaagttacatcagatttattttgttttcttattaatactcaccatttattaatcttt
gctcttattataattctatttatctttacaggatttaatatttattgttggactggaatt
tacttctcatagaattattttcttatattatttcatcttaattttggattaatttatagt
tttcatatttggtctagagatttattaagagaattcactaaaaaatatgaagtcttatta
ataatcttttttcttctttttggggggttttcttgtttctgaagctctattatttgtatcc
ttcttttggacatcttttcatttattatcttctccaactttagggatgtgggtgtgggaa
ggtttctatctggaggatccttgtgaattaacttttgctaatacacttcttttatcaaat
gctgctatatctttaggaaatgcttttattaatttagaaatttcatcagaatatattatt
ttcttcactttatggtcattctttttgtcttctctttttattagtttgcagattaaagaa
tttcgcattctcgcattatcaattaatgattcactttatagttctcttttcttttttctt
acaggattacatttctttcatctatctattggtcttattcttctaatttatttctattat
gtgggtttctttgtttactttatgctttcatggacttcttttaataaaatat
>Od23_cox3
attaatacagaattattattttcttatctgtatctattggtattttggaaatcattacat
ttagaagttacatcagatttattttgttttcttattaatactcaccatttattaatcttt
gctcttattataattctatttatctttacaggatttaatatttattgttggactggaatt

tacttctcatagaattattttcttatattatttcatcttaattttggattaatttatagt
tttcatatttggtctagagatttattaagagaattcactaaaaaatatgaagtcttatta
ataatctttttcttctttttgggggttttcttgtttctgaagctctattatttgtatcc
ttcttttggacatcttttcatttattatcttctccaactttagggatgtgggtgtgggaa
ggtttctatctggaggatccttgtgaattaacttttgctaatacacttcttttatcaaat
gctgctatatctttaggaaatgcttttattaatttagaaatttcatcagaatatattatt
ttcttcactttatggtcattctttttgtcttctctttttattagtttgcagattaaagaa
tttcgcattctcgcattatcaattaatgattcactttatagttctcttttcttttttctt
acaggattacatttctttcatctatctattggtcttattcttctaatttatttctattat
gtgggtttctttgtttactttatgctttcatggacttcttttaataaaatat
>Od24_cox3
attaatacagaattattattttcttatctgtatctattggtattttggaaatcattacat
ttagaagttacatcagatttattttgttttcttattaatactcactatttattaatcttt
gctcttgttgtaattctatttatctttacaggatttaatatttattgttgggctggaatt
tacttctcatggagttattctcttatattatttcatcttaattttggattaatttatagt
tttcatatttggtctagagatttattaagagaattcactaaaaaatatgaagtcttatta
ataatctttttcttctttttgggggttttcttgtttctgaagctctattatttgtatcc
ttcttttggacatcttttcatttattatcttctccaactttagggatgtgggtgtgggaa
ggtttctatctggaggatccttgtgaattaacttttgctaatacacttcttttatcaaat
gctgctatatctttaggaaatgcttttattaatttagaaatttcatcagaatatattatt
ttcttcactttatggtcattctttttgtcttctctttttattagtttgcagattaaagaa
tttcgcattctcgcattatcaattaatgattcactttatagttctcttttcttttttctt
acaggattacatttctttcatctatctattggtcttattcttctaatttatttctattat
gtgggtttctttgtttactttatgctttcatggacttcttttaataaaatat
>Od25_cox3
attaatacagaattattattttcttatctgtatctattggtattttggaaatcattacat
ttagaagttacatcagatttattttgttttcttattaatactcaccatttattaatcttt
gctcttattataattctatttatctttacaggatttaatatttattgttggactggaatt
tacttctcatagaattattttcttatattatttcatcttaattttggattaatttatagt
tttcatatttggtctagagatttattaagagaattcactaaaaaatatgaagtcttatta
ataatctttttcttctttttgggggttttcttgtttctgaagctctattatttgtatcc
ttcttttggacatcttttcatttattatcttctccaactttagggatgtgggtgtgggaa
ggtttctatctggaggatccttgtgaattaacttttgctaatacacttcttttatcaaat
gctgctatatctttaggaaatgcttttattaatttagaaatttcatcagaatatattatt
ttcttcactttatggtcattctttttgtcttctctttttattagtttgcagattaaagaa
tttcgcattctcgcattatcaattaatgattcactttatagttctcttttcttttttctt
acaggattacatttctttcatctatctattggtcttattcttctaatttatttctattat
gtgggtttctttgtttactttatgctttcatggacttcttttaataaaatat
>Od26_cox3
attaatacagaattattattttcttatctgtatctattggtattttggaaatcattacat
ttagaagttacatcagatttattttgttttcttattaatactcaccatttattaatcttt
gctcttattataattctatttatctttacaggatttaatatttattgttggactggaatt
tacttctcatagaattattttcttatattatttcatcttaattttggattaatttatagt
tttcatatttggtctagagatttattaagagaattcactaaaaaatatgaagtcttatta
ataatctttttcttctttttgggggttttcttgtttctgaagctctattatttgtatcc
ttcttttggacatcttttcatttattatcttctccaactttagggatgtgggtgtgggaa
ggtttctatctggaggatccttgtgaattaacttttgctaatacacttcttttatcaaat
gctgctatatctttaggaaatgcttttattaatttagaaatttcatcagaatatattatt
ttcttcactttatggtcattctttttgtcttctctttttattagtttgcagattaaagaa
tttcgcattctcgcattatcaattaatgattcactttatagttctcttttcttttttctt
acaggattacatttctttcatctatctattggtcttattcttctaatttatttctattat
gtgggtttctttgtttactttatgctttcatggacttcttttaataaaatat
>Od27_cox3
attaatacagaattattattttcttatctgtatctattggtattttggaaatcattacat
ttagaagttacatcagatttattttgttttcttattaatactcaccatttattaatcttt
gctcttattataattctatttatctttacaggatttaatatttattgttggactggaatt
tacttctcatagaattattttcttatattatttcatcttaattttggattaatttatagt

tttcatatttggtctagagatttattaagagaattcactaaaaaatatgaagtcttatta
ataatctttttcttcttttggggggttttcttgtttctgaagctctattatttgtatcc
ttcttttggacatcttttcatttattatcttctccaactttagggatgtgggtgtgggaa
ggtttctatctggaggatccttgtgaattaacttttgctaatacacttcttttatcaaat
gctgctatatctttaggaaatgcttttattaatttagaaatttcatcagaatatattatt
ttcttcactttatggtcattctttttgtcttctctttttattagtttgcagattaaagaa
tttcgcattctcgcattatcaattaatgattcactttatagttctcttttcttttttctt
acaggattacatttctttcatctatctattggtcttattcttctaatttatttctattat
gtgggtttctttgtttactttatgctttcatggacttcttttaataaaatat
>Od4_cox3
attaatacagaattattattttcttatctgtatctattggtattttggaaatcattacat
ttagaagttacatcagatttattttgttttcttattaatactcaccatttattaatcttt
gctcttattataattctatttatctttacaggatttaatatttattgttggactggaatt
tacttctcatagaattattttcttatattatttcatcttaattttggattaatttatagt
tttcatatttggtctagagatttattaagagaattcactaaaaaatatgaagtcttatta
ataatctttttcttcttttggggggttttcttgtttctgaagctctattatttgtatcc
ttcttttggacatcttttcatttattatcttctccaactttagggatgtgggtgtgggaa
ggtttctatctggaggatccttgtgaattaacttttgctaatacacttcttttatcaaat
gctgctatatctttaggaaatgcttttattaatttagaaatttcatcagaatatattatt
ttcttcactttatggtcattctttttgtcttctctttttattagtttgcagattaaagaa
tttcgcattctcgcattatcaattaatgattcactttatagttctcttttcttttttctt
acaggattacatttctttcatctatctattggtcttattcttctaatttatttctattat
gtgggtttctttgtttactttatgctttcatggacttcttttaataaaatat
>Od6_cox3
attaatacagaattattattttcttatctgtatctattggtattttggaaatcattacat
ttagaagttacatcagatttattttgttttcttattaatactcaccatttattaatcttt
gctcttattataattctatttatctttacaggatttaatatttattgttggactggaatt
tacttctcatagaattattttcttatattatttcatcttaattttggattaatttatagt
tttcatatttggtctagagatttattaagagaattcactaaaaaatatgaagtcttatta
ataatctttttcttcttttggggggttttcttgtttctgaagctctattatttgtatcc
ttcttttggacatcttttcatttattatcttctccaactttagggatgtgggtgtgggaa
ggtttctatctggaggatccttgtgaattaacttttgctaatacacttcttttatcaaat
gctgctatatctttaggaaatgcttttattaatttagaaatttcatcagaatatattatt
ttcttcactttatggtcattctttttgtcttctctttttattagtttgcagattaaagaa
tttcgcattctcgcattatcaattaatgattcactttatagttctcttttcttttttctt
acaggattacatttctttcatctatctattggtcttattcttctaatttatttctattat
gtgggtttctttgtttactttatgctttcatggacttcttttaataaaatat
>Od8_cox3
attaatacagaattattattttcttatctgtatctattggtattttggaaatcattacat
ttagaagttacatcagatttattttgttttcttattaatactcaccatttattaatcttt
gctcttattataattctatttatctttacaggatttaatatttattgttggactggaatt
tacttctcatagaattattttcttatattatttcatcttaattttggattaatttatagt
tttcatatttggtctagagatttattaagagaattcactaaaaaatatgaagtcttatta
ataatctttttcttcttttggggggttttcttgtttctgaagctctattatttgtatcc
ttcttttggacatcttttcatttattatcttctccaactttagggatgtgggtgtgggaa
ggtttctatctggaggatccttgtgaattaacttttgctaatacacttcttttatcaaat
gctgctatatctttaggaaatgcttttattaatttagaaatttcatcagaatatattatt
ttcttcactttatggtcattctttttgtcttctctttttattagtttgcagattaaagaa
tttcgcattctcgcattatcaattaatgattcactttatagttctcttttcttttttctt
acaggattacatttctttcatctatctattggtcttattcttctaatttatttctattat
gtgggtttctttgtttactttatgctttcatggacttcttttaataaaatat
>Od9_cox3
attaatacagaattattattttcttatctgtatctattggtattttggaaatcattacat
ttagaagttacatcagatttattttgttttcttattaatactcaccatttattaatcttt
gctcttattataattctatttatctttacaggatttaatatttattgttggactggaatt
tacttctcatagaattattttcttatattatttcatcttaattttggattaatttatagt
tttcatatttggtctagagatttattaagagaattcactaaaaaatatgaagtcttatta

ataatcttttttcttctttttgggggttttcttgtttctgaagctctattatttgtatcc
ttcttttggacatcttttcatttattatcttctccaactttagggatgtgggtgtgggaa
ggtttctatctggaggatccttgtgaattaacttttgctaatacacttcttttatcaaat
gctgctatatctttaggaaatgcttttattaatttagaaatttcatcagaatatattatt
ttcttcactttatggtcattctttttgtcttctcttttattagtttgcagattaaagaa
tttcgcattctcgcattatcaattaatgattcactttatagttctcttttcttttttctt
acaggattacatttctttcatctatctattggtcttattcttctaatttatttctattat
gtgggtttctttgtttactttatgctttcatggacttcttttaataaaatat
>Ss5_cox3
attaatacagaattattatttcttatctgtatctattggtattttggaaatcattacat
ttagaagttacatcagatttattttgttttcttattaatactcaccatttattaatcttt
gctcttattataattctatttatctttacaggatttaatatttattgttggactggaatt
tacttctcatagaattattttcttatattatttcatcttaattttggattaatttatagt
tttcatatttggtctagagatttattaagagaattcactaaaaaatatgaagtcttatta
ataatcttttttcttctttttgggggttttcttgtttctgaagctctattatttgtatcc
ttcttttggacatcttttcatttattatcttctccaactttagggatgtgggtgtgggaa
ggtttctatctggaggatccttgtgaattaacttttgctaatacacttcttttatcaaat
gctgctatatctttaggaaatgcttttattaatttagaaatttcatcagaatatattatt
ttcttcactttatggtcattctttttgtcttctcttttattagtttgcagattaaagaa
tttcgcattctcgcattatcaattaatgattcactttatagttctcttttcttttttctt
acaggattacatttctttcatctatctattggtcttattcttctaatttatttctattat
gtgggtttctttgtttactttatgctttcatggacttcttttaataaaatat
>Ss6_cox3
attaatacagaattattatttcttatctgtatctattggtattttggaaatcattacat
ttagaagttacatcagatttattttgttttcttattaatactcaccatttattaatcttt
gctcttattataattctatttatctttacaggatttaatatttattgttggactggaatt
tacttctcatagaattattttcttatattatttcatcttaattttggattaatttatagt
tttcatatttggtctagagatttattaagagaattcactaaaaaatatgaagtcttatta
ataatcttttttcttctttttgggggttttcttgtttctgaagctctattatttgtatcc
ttcttttggacatcttttcatttattatcttctccaactttagggatgtgggtgtgggaa
ggtttctatctggaggatccttgtgaattaacttttgctaatacacttcttttatcaaat
gctgctatatctttaggaaatgcttttattaatttagaaatttcatcagaatatattatt
ttcttcactttatggtcattctttttgtcttctcttttattagtttgcagattaaagaa
tttcgcattctcgcattatcaattaatgattcactttatagttctcttttcttttttctt
acaggattacatttctttcatctatctattggtcttattcttctaatttatttctattat
gtgggtttctttgtttactttatgctttcatggacttcttttaataaaatat
>Ss7_cox3
attaatacagaattattatttcttatctgtatctattggtattttggaaatcattacat
ttagaagttacatcagatttattttgttttcttattaatactcaccatttattaatcttt
gctcttattataattctatttatctttacaggatttaatatttattgttggactggaatt
tacttctcatagaattattttcttatattatttcatcttaattttggattaatttatagt
tttcatatttggtctagagatttattaagagaattcactaaaaaatatgaagtcttatta
ataatcttttttcttctttttgggggttttcttgtttctgaagctctattatttgtatcc
ttcttttggacatcttttcatttattatcttctccaactttagggatgtgggtgtgggaa
ggtttctatctggaggatccttgtgaattaacttttgctaatacacttcttttatcaaat
gctgctatatctttaggaaatgcttttattaatttagaaatttcatcagaatatattatt
ttcttcactttatggtcattctttttgtcttctcttttattagtttgcagattaaagaa
tttcgcattctcgcattatcaattaatgattcactttatagttctcttttcttttttctt
acaggattacatttctttcatctatctattggtcttattcttctaatttatttctattat
gtgggtttctttgtttactttatgctttcatggacttcttttaataaaatat
>Ss8_cox3
attaatacagaattattatttcttatctgtatctattggtattttggaaatcattacat
ttagaagttacatcagatttattttgttttcttattaatactcaccatttattaatcttt
gctcttattataattctatttatctttacaggatttaatatttattgttggactggaatt
tacttctcatagaattattttcttatattatttcatcttaattttggattaatttatagt
tttcatatttggtctagagatttattaagagaattcactaaaaaatatgaagtcttatta
ataatcttttttcttctttttgggggttttcttgtttctgaagctctattatttgtatcc

ttcttttggacatcttttcatttattatcttctccaactttagggatgtgggtgtgggaa
ggtttctatctggaggatccttgtgaattaacttttgctaatacacttcttttatcaaat
gctgctatatctttaggaaatgcttttattaatttagaaatttcatcagaatatattatt
ttcttcactttatggtcattctttttgtcttctcttttattagtttgcagattaaagaa
tttcgcattctcgcattatcaattaatgattcactttatagttctcttttcttttttctt
acaggattacatttctttcatctatctattggtcttattcttctaatttatttctattat
gtgggtttctttgtttactttatgctttcatggacttcttttaataaaatat
>ohdo3_cox3
attaatacagaattattattttcttatctgtatctattggtattttggaaatcattacat
ttagaagttacatcagatttattttgttttcttattaatactcaccatttattaatcttt
gctcttattataattctatttatctttacaggatttaatatttattgttggactggaatt
tacttctcatagaattattttcttatattatttcatcttaattttggattaatttatagt
tttcatatttggtctagagatttattaagagaattcactaaaaaaatatgaagtcttatta
ataatctttttcttctttttgggggttttcttgtttctgaagctctattatttgtatcc
ttcttttggacatcttttcatttattatcttctccaactttagggatgtgggtgtgggaa
ggtttctatctggaggatccttgtgaattaacttttgctaatacacttcttttatcaaat
gctgctatatctttaggaaatgcttttattaatttagaaatttcatcagaatatattatt
ttcttcactttatggtcattctttttgtcttctcttttattagtttgcagattaaagaa
tttcgcattctcgcattatcaattaatgattcactttatagttctcttttcttttttctt
acaggattacatttctttcatctatctattggtcttattcttctaatttatttctattat
gtgggtttctttgtttactttatgctttcatggacttcttttaataaaatat
>ohdo5_cox3
attaatacagaattattattttcttatctgtatctattggtattttggaaatcattacat
ttagaagttacatcagatttattttgttttcttattaatactcaccatttattaatcttt
gctcttattataattctatttatctttacaggatttaatatttattgttggactggaatt
tacttctcatagaattattttcttatattatttcatcttaattttggattaatttatagt
tttcatatttggtctagagatttattaagagaattcactaaaaaaatatgaagtcttatta
ataatctttttcttctttttgggggttttcttgtttctgaagctctattatttgtatcc
ttcttttggacatcttttcatttattatcttctccaactttagggatgtgggtgtgggaa
ggtttctatctggaggatccttgtgaattaacttttgctaatacacttcttttatcaaat
gctgctatatctttaggaaatgcttttattaatttagaaatttcatcagaatatattatt
ttcttcactttatggtcattctttttgtcttctcttttattagtttgcagattaaagaa
tttcgcattctcgcattatcaattaatgattcactttatagttctcttttcttttttctt
acaggattacatttctttcatctatctattggtcttattcttctaatttatttctattat
gtgggtttctttgtttactttatgctttcatggacttcttttaataaaatat
>ohdo7_cox3
attaatacagaattattattttcttatctgtatctattggtattttggaaatcattacat
ttagaagttacatcagatttattttgttttcttattaatactcaccatttattaatcttt
gctcttattataattctatttatctttacaggatttaatatttattgttggactggaatt
tacttctcatagaattattttcttatattatttcatcttaattttggattaatttatagt
tttcatatttggtctagagatttattaagagaattcactaaaaaaatatgaagtcttatta
ataatctttttcttctttttgggggttttcttgtttctgaagctctattatttgtatcc
ttcttttggacatcttttcatttattatcttctccaactttagggatgtgggtgtgggaa
ggtttctatctggaggatccttgtgaattaacttttgctaatacacttcttttatcaaat
gctgctatatctttaggaaatgcttttattaatttagaaatttcatcagaatatattatt
ttcttcactttatggtcattctttttgtcttctcttttattagtttgcagattaaagaa
tttcgcattctcgcattatcaattaatgattcactttatagttctcttttcttttttctt
acaggattacatttctttcatctatctattggtcttattcttctaatttatttctattat
gtgggtttctttgtttactttatgctttcatggacttcttttaataaaatat
>sesoko1_cox3
attaatacagaattattattttcttatctgtatctattggtattttggaaatcattacat
ttagaagttacatcagatttattttgttttcttattaatactcaccatttattaatcttt
gctcttattataattctatttatctttacaggatttaatatttattgttggactggaatt
tacttctcatagaattattttcttatattatttcatcttaattttggattaatttatagt
tttcatatttggtctagagatttattaagagaattcactaaaaaaatatgaagtcttatta
ataatctttttcttctttttgggggttttcttgtttctgaagctctattatttgtatcc
ttcttttggacatcttttcatttattatcttctccaactttagggatgtgggtgtgggaa

ggtttctatctggaggatccttgtgaattaacttttgctaatacacttcttttatcaaat
gctgctatatctttaggaaatgcttttattaatttagaaatttcatcagaatatattatt
ttcttcactttatggtcattctttttgtcttctcttttttattagtttgcagattaaagaa
tttcgcattctcgcattatcaattaatgattcactttatagttctcttttctttttcttt
acaggattacatttctttcatctatctattggtcttattcttctaatttatttctattat
gtgggtttctttgtttactttatgctttcatggacttcttttaataaaatat
>sesoko2_cox3
attaatacagaattattattttcttatctgtatctattggtattttggaaatcattacat
ttagaagttacatcagatttattttgttttcttattaatactcaccatttattaatcttt
gctcttattataattctatttatctttacaggatttaatatttattgttggactggaatt
tacttctcatagaattattttcttatattatttcatcttaattttggattaatttatagt
tttcatatttggtctagagatttattaagagaattcactaaaaaatatgaagtcttatta
ataatctttttttcttctttttggggggttttcttgtttctgaagctctattatttgtatcc
ttcttttggacatcttttcatttattatcttctccaactttagggatgtgggtgtgggaa
ggtttctatctggaggatccttgtgaattaacttttgctaatacacttcttttatcaaat
gctgctatatctttaggaaatgcttttattaatttagaaatttcatcagaatatattatt
ttcttcactttatggtcattctttttgtcttctcttttttattagtttgcagattaaagaa
tttcgcattctcgcattatcaattaatgattcactttatagttctcttttctttttcttt
acaggattacatttctttcatctatctattggtcttattcttctaatttatttctattat
gtgggtttctttgtttactttatgctttcatggacttcttttaataaaatat
>sesoko3_cox3
attaatacagaattattattttcttatctgtatctattggtattttggaaatcattacat
ttagaagttacatcagatttattttgttttcttattaatactcaccatttattaatcttt
gctcttattataattctatttatctttacaggatttaatatttattgttggactggaatt
tacttctcatagaattattttcttatattatttcatcttaattttggattaatttatagt
tttcatatttggtctagagatttattaagagaattcactaaaaaatatgaagtcttatta
ataatctttttttcttctttttggggggttttcttgtttctgaagctctattatttgtatcc
ttcttttggacatcttttcatttattatcttctccaactttagggatgtgggtgtgggaa
ggtttctatctggaggatccttgtgaattaacttttgctaatacacttcttttatcaaat
gctgctatatctttaggaaatgcttttattaatttagaaatttcatcagaatatattatt
ttcttcactttatggtcattctttttgtcttctcttttttattagtttgcagattaaagaa
tttcgcattctcgcattatcaattaatgattcactttatagttctcttttctttttcttt
acaggattacatttctttcatctatctattggtcttattcttctaatttatttctattat
gtgggtttctttgtttactttatgctttcatggacttcttttaataaaatat
>sesoko4_cox3
attaatacagaattattattttcttatctgtatctattggtattttggaaatcattacat
ttagaagttacatcagatttattttgttttcttattaatactcaccatttattaatcttt
gctcttattataattctatttatctttacaggatttaatatttattgttggactggaatt
tacttctcatagaattattttcttatattatttcatcttaattttggattaatttatagt
tttcatatttggtctagagatttattaagagaattcactaaaaaatatgaagtcttatta
ataatctttttttcttctttttggggggttttcttgtttctgaagctctattatttgtatcc
ttcttttggacatcttttcatttattatcttctccaactttagggatgtgggtgtgggaa
ggtttctatctggaggatccttgtgaattaacttttgctaatacacttcttttatcaaat
gctgctatatctttaggaaatgcttttattaatttagaaatttcatcagaatatattatt
ttcttcactttatggtcattctttttgtcttctcttttttattagtttgcagattaaagaa
tttcgcattctcgcattatcaattaatgattcactttatagttctcttttctttttcttt
acaggattacatttctttcatctatctattggtcttattcttctaatttatttctattat
gtgggtttctttgtttactttatgctttcatggacttcttttaataaaatat
>REF_DNA_Cox3_LC002802.1
ATTAATACAGAATTATTATTTTATTATCTGTATCTATTGGTATTTTGGAAATCATTACAT
TTAGAAGTTACATCAGATTTATTTTGTTTTCTTATTAATACTCACCATTTATTAATCTTT
GCTCTTATTATAATTCTATTTATCTTTACAGGATTTAATATTTATTGTTGGACTGGAATT
TACTTCTCATAGAATTATTTTCTTATATTATTTCATCTTAATTTTGGATTAATTTATAGT
TTTCATATTTGGTCTAGAGATTTATTAAGAGAATTCACTAAAAAATATGAAGTCTTATTA
ATAATCTTTTTTTCTTCTTTTTGGGGGTTTTCTAGTTTCTGAAGCTCTATTATTTGTATCC
TTCTTTTGGACATCTTTTCATTTATTATCTTCTCCAACGGCTGGGATGTGGCTGTGGGAA
GCTTTCTATCTGGAGGATCCTTGTGAATTAACTTTTGCTAATACACTTCTTTTATCAAAT

GCTGCTATATCTTTAGGAAATGCTTTTATTAATTTAGAAATTTCATCAGAATATATTATT
TTCTTCACTTTATGGTCATTCTTTTTGTCTTCTCTTTTTATTAGTTTGCAGATTAAAGAA
TTTCGCATTCTCGCATTATCAATTAATGATTCACTTTATAGTTCTCTTTTCTTTTTTCTT
ACAGGATTACATTTCTTTCATCTATCTATTGGTCTTATTCTTCTAATTTATTTCTATTAT
GTGGGTTTCTTTGTTTACTTTATGCTTTCATGGACTTCTTTTAATAAAATAT
>REF_RNA_Cox3_symbB1.comp4_c1_seq1_symbB1.EST_k37c20_2341
ATTAATACAGAATTATTATTTTATTATCTGTATCTATTGGTATTTTGGAAATCATTACAT
TTAGAAGTTGCATCAGGTTCATTTTGTCTTCTTATTAATGCTCACTATTTATTAATCTTT
GCTCTTGTTGTAATTCTATTTATCTTTACAGGATTTAATATTTATTGTTGGGCTGGAATT
TACTTCTCATGGAGTTATTCTCTTATATTATTTCATCTTAATTTTGGATTAATTTATAGT
TTTCATATTTGGTCTAGAGATTTATTAAGAGAATTCACTAAAAAATATGAAATCTTATTA
ATGGTCCTTTTTCTTCTTTTTGGGGGTTTTCTAGTTTCTGAAGCTCTATTATTTGTATCC
TTCTTTTGGGCATCTTTTCATTTATTATCTTCTCCAACGGCTGGGATGTGGCTGTGGGAA
GCTTTCTATCTGGAGGATCCTTGTGAATTAACTTTTGCTAATACACTTCTTTTATCAAAT
GCTGCTGTATCTTTAGGAGGTGCTTTTATTAGTTTAGAAATTTCATCAGGATATATTATT
TTCTTCGCTTTATGGTCATTCTTTTTGTCTTCTCTTTTTATTAGTTTGCAGATTAAAGAA
TTTCGCGTTCTCGCATTATCAATTAATGATTCACTTTATAGTTCTCTTTTCTTTTTTCTT
ACAGGATTACATTTCTTTCATCTATCTATTGGTCTTATTCTTCTAATTTATTTCTATTAT
GTGGGTTTCTTTGTTTACTTTATGCTTTCATGGACTTCTTTTAATAAAATAT

>Hd1_cob
atgaaatctcatttacaatcatatccttgtcctctgatcataaattatttttggaatctt
ggtttttattagggattactattttattacaaattatatctggaatcttcttaggttta
cattatacatcagatattaattcagcatattttagtattttctttattattagagaaata
tattatggatggtgtttacgttatcttcattctaatggttcatcatttgtctttcttttg
atatttctacatcttggaagagctatatcttatggttcatatttttataatccaaatact
tggttttctggaattattattatcttcttcttaatgggaacagcatttatgggttatgtg
ttacctttaggacaaatgagtttatgggggggttacagtaattacaaatttattatctgca
tttccatctttaatagaatggctttgtggaggacattacatttacaatcctacatttaag
aggttctttgtctttcattttctatttccatttcttctttgtggttttcttgtttatcat
atttttaatctacattttctatcttctaataatcctttaaggaattccactaataataaa
atagcatttttccctttcattattagtaaagatttatatggaaagatattaattctctat
ctatatcttcttcaaattcatttcggtttctcttctttctcacatccagataatgcatta
gaagcatgtggattacttactcctttacatatagtacctgaatggtatttcttatgccaa
tatgctatgttaaaagctgtgccaaataaaaatgcaggattcattatattatttacttct
atcttcatcttattttactttatgagaagtctttcaatctcattctattttatcgtgtgg
gtaagctctagatttaatagtttctttgtaattctttggtttttagttttcatatcctta
atttggataggtggtcaatttcctgtagacaactttctatcttatggtcgtatcttgaca
ttacattattattatcttcttatctgtatcttattatcttag
>Hd2_cob
atgaaatctcatttacaatcatatccttgtcctctgatcataaattatttttggaatctt
ggtttttattagggattactattttattacaaattatatctggaatcttcttaggttta
cattatacatcagatattaattcagcatattttagtattttctttattattagagaaata
tattatggatggtgtttacgttatcttcattctaatggttcatcatttgtctttcttttg
atatttctacatcttggaagagctatatcttatggttcatatttttataatccaaatact
tggttttctggaattattattatcttcttcttaatgggaacagcatttatgggttatgtg
ttacctttaggacaaatgagtttatgggggggttacagtaattacaaatttattatctgca
tttccatctttaatagaatggctttgtggaggacattacatttacaatcctacatttaag
aggttctttgtctttcattttctatttccatttcttctttgtggttttcttgtttatcat
atttttaatctacattttctatcttctaataatcctttaaggaattccactaataataaa
atagcatttttccctttcattattagtaaagatttatatggaaagatattaattctctat
ctatatcttcttcaaattcatttcggtttctcttctttctcacatccagataatgcatta
gaagcatgtggattacttactcctttacatatagtacctgaatggtatttcttatgccaa
tatgctatgttaaaagctgtgccaaataaaaatgcaggattcattatattatttacttct
atcttcatcttattttactttatgagaagtctttcagtatctttctattttatcgtgtgg
gtaagctctagatttaatagtttctttgtaattctttggtttttagttttcatatcctta
atttggataggtggtcaatttcctgtagacaactttctatcttatggtcgtatcttgaca
ttacattattattatcttcttatctgtatcttattatcttag
>Hd3_cob
atgaaatctcatttacaatcatatccttgtcctctgatcataaattatttttggaatctt
ggtttttattagggattactattttattacaaattatatctggaatcttcttaggttta
cattatacatcagatattaattcagcatattttagtattttctttattattagagaaata
tattatggatggtgtttacgttatcttcattctaatggttcatcatttgtctttcttttg
atatttctacatcttggaagagctatatcttatggttcatatttttataatccaaatact
tggttttctggaattattattatcttcttcttaatgggaacagcatttatgggttatgtg
ttacctttaggacaaatgagtttatgggggggttacagtaattacaaatttattatctgca
tttccatctttaatagaatggctttgtggaggacattacatttacaatcctacatttaag
aggttctttgtctttcattttctatttccatttcttctttgtggttttcttgtttatcat
atttttaatctacattttctatcttctaataatcctttaaggaattccactaataataaa
atagcatttttccctttcattattagtaaagatttatatggaaagatattaattctctat
ctatatcttcttcaaattcatttcggtttctcttctttctcacatccagataatgcatta
gaagcatgtggattacttactcctttacatatagtacctgaatggtatttcttatgccaa
tatgctatgttaaaagctgtgccaaataaaaatgcaggattcattatattatttacttct
atcttcatcttattttactttatgagaagtctttcaatctcattctattttatcgtgtgg
gtaagctctagatttaatagtttctttgtaattctttggtttttagttttcatatcctta
atttggataggtggtcaatttcctgtagacaactttctatcttatggtcgtatcttgaca
ttacattattattatcttcttatctgtatcttattatcttag

>Hd4_cob
atgaaatctcatttacaatcatatccttgtcctctgatcataaattatttttggaatctt
ggtttttattagggattactattttattacaaattatatctggaatcttcttaggttta
cattatacatcagatattaattcagcatattttagtattttctttattattagagaaata
tattatggatggtgtttacgttatcttcattctaatggttcatcatttgtctttcttttg
atatttctacatcttggaagagctatatcttatggttcatatttttataatccaaatact
tggttttctggaattattattatcttcttcttaatgggaacagcatttatgggttatgtg
ttacctttaggacaaatgagtttatggggggttacagtaattacaaatttattatctgca
tttccatctttaatagaatggctttgtggaggacattacatttacaatcctacatttaag
aggttctttgtctttcattttctatttccatttcttctttgtggttttcttgtttatcat
atttttaatctacattttctatcttctaataatcctttaaggaattccactaataataaa
atagcatttttccctttcattattagtaaagatttatatggaaagatattaattctctat
ctatatcttcttcaaattcatttcggtttctcttctttctcacatccagataatgcatta
gaagcatgtggattacttactcctttacatatagtacctgaatggtatttcttatgccaa
tatgctatgttaaaagctgtgccaaataaaaatgcaggattcattatattatttacttct
atcttcatcttattttactttatgagaagtctttcaatctcattctattttatcgtgtgg
gtaagctctagatttaatagtttctttgtaattctttggtttttagttttcatatcctta
atttggataggtggtcaatttcctgtagacaactttctatcttatggtcgtatcttgaca
ttacattattattatcttcttatctgtatcttattatcttag
>Hd5_cob
atgaaatctcatttacaatcatatccttgtcctctgatcataaattatttttggaatctt
ggtttttattagggattactattttattacaaattatatctggaatcttcttaggttta
cattatacatcagatattaattcagcatattttagtattttctttattattagagaaata
tattatggatggtgtttacgttatcttcattctaatggttcatcatttgtctttcttttg
atatttctacatcttggaagagctatatcttatggttcatatttttataatccaaatact
tggttttctggaattattattatcttcttcttaatgggaacagcatttatgggttatgtg
ttacctttaggacaaatgagtttatggggggttacagtaattacaaatttattatctgca
tttccatctttaatagaatggctttgtggaggacattacatttacaatcctacatttaag
aggttctttgtctttcattttctatttccatttcttctttgtggttttcttgtttatcat
atttttaatctacattttctatcttctaataatcctttaaggaattccactaataataaa
atagcatttttccctttcattattagtaaagatttatatggaaagatattaattctctat
ctatatcttcttcaaattcatttcggtttctcttctttctcacatccagataatgcatta
gaagcatgtggattacttactcctttacatatagtacctgaatggtatttcttatgccaa
tatgctatgttaaaagctgtgccaaataaaaatgcaggattcattatattatttacttct
atcttcatcttattttattttatgagaagtctttcagtatctttctattttatcgtgtgg
gtaagctctagatttaatagtttctttgtaattctttggtttttagttttcatatcctta
atttggataggtggtcaatttcctgtagacaactttctatcttatggtcgtatcttgaca
ttacattattattatcttcttatctgtatcttattctcttag
>Hd6_cob
atgaaatctcatttacaatcatatccttgtcctctgatcataaattatttttggaatctt
ggtttttattagggattactattttattacaaattatatctggaatcttcttaggttta
cattatacatcagatattaattcagcatattttagtattttctttattattagagaaata
tattatggatggtgtttacgttatcttcattctaatggttcatcatttgtctttcttttg
atatttctacatcttggaagagctatatcttatggttcatatttttataatccaaatact
tggttttctggaattattattatcttcttcttaatgggaacagcatttatgggttatgtg
ttacctttaggacaaatgagtttatggggggttacagtaattacaaatttattatctgca
tttccatctttaatagaatggctttgtggaggacattacatttacaatcctacatttaag
aggttctttgtctttcattttctatttccatttcttctttgtggttttcttgtttatcat
atttttaatctacattttctatcttctaataatcctttaaggaattccactaataataaa
atagcatttttccctttcattattagtaaagatttatatggaaagatattaattctctat
ctatatcttcttcaaattcatttcggtttctcttctttctcacatccagataatgcatta
gaagcatgtggattacttactcctttacatatagtacctgaatggtatttcttatgccaa
tatgctatgttaaaagctgtgccaaataaaaatgcaggattcattatattatttacttct
atcttcatcttattttactttatgagaagtctttcagtatctttctattttatcgtgtgg
gtaagctctagatttaatagtttctttgtaattctttggtttttagttttcatatcctta
atttggataggtggtcaatttcctgtagacaactttctatcttatggtcgtatcttgaca
ttacattattattatcttcttatctgtatcttattatcttag

>Hd7_cob
atgaaatctcatttacaatcatatccttgtcctctgatcataaattatttttggaatctt
ggtttttattagggattactattttattacaaattatatctggaatcttcttaggttta
cattatacatcagatattaattcagcatattttagtattttctttattattagagaaata
tattatggatggtgtttacgttatcttcattctaatggttcatcatttgtctttctttg
atatttctacatcttggaagagctatatcttatggttcatatttttataatccaaatact
tggttttctggaattattattatcttcttcttaatgggaacagcatttatgggttatgtg
ttacctttaggacaaatgagtttatggggggttacagtaattacaaatttattatctgca
tttccatctttaatagaatggctttgtggaggacattacatttacaatcctacatttaag
aggttctttgtctttcattttctatttccatttcttctttgtggttttcttgttcatcat
atttttaatctacattttctatcttctaataatcctttaaggaattccactaataataaa
atagcatttttccctttcattattagtaaagatttatatggaaagatattaattctctat
ctatatcttcttcaaattcatttcggtttctcttctctctcacatccagataatgcattg
gaagcttgtggattacttactcctttacatatagtacctgaatggtatttcttatgccaa
tatgctatgttaaaagctgtgccaaataaaaatgcaggattcattgtcttactaacttct
atctttgtattattttattttatgagaagtctttcagtatctttctattttatcgtgtgg
gtaagctctagatttaatagtttctttgtaattctttggtttttagttttcatatcctta
atttggataggtggtcaatttcctgtagacaactttctatcttatggtcgtatcttgaca
ttacattattattatcttcttatctgtatcttattatcttag
>Hd8_cob
atgaaatctcatttacaatcatatccttgtcctctgatcataaattatttttggaatctt
ggtttttattagggattactattttattacaaattatatctggaatcttcttaggttta
cattatacatcagatattaattcagcatattttagtattttctttattattagagaaata
tattatggatggtgtttacgttatcttcattctaatggttcatcatttgtctttctttg
atatttctacatcttggaagagctatatcttatggttcatatttttataatccaaatact
tggttttctggaattattattatcttcttcttaatgggaacagcatttatgggttatgtg
ttacctttaggacaaatgagtttatggggggttacagtaattacaaatttattatctgca
tttccatctttaatagaatggctttgtggaggacattacatttacaatcctacatttaag
aggttctttgtctttcattttctatttccatttcttctttgtggttttcttgtttatcat
atttttaatctacattttctatcttctaataatcctttaaggaattccactaataataaa
atagcatttttccctttcattattagtaaagatttatatggaaagatattaattctctat
ctatatcttcttcaaattcatttcggtttctcttctttctcacatccagataatgcatta
gaagcatgtggattacttactcctttacatatagtacctgaatggtatttcttatgccaa
tatgctatgttaaaagctgtgccaaataaaaatgcaggattcattatattatttacttct
atcttcatcttattttactttatgagaagtctttcagtatctttctattttatcgtgtgg
gtaagctctagatttaatagtttctttgtaattctttggtttttagttttcatatcctta
atttggataggtggtcaatttcctgtagacaactttctatcttatggtcgtatcttgaca
ttacattattattatcttcttatctgtatcttattatcttag
>Hd9_cob
atgaaatctcatttacaatcatatccttgtcctctgatcataaattatttttggaatctt
ggtttttattagggattactattttattacaaattatatctggaatcttcttaggttta
cattatacatcagatattaattcagcatattttagtattttctttattattagagaaata
tattatggatggtgtttacgttatcttcattctaatggttcatcatttgtctttctttg
atatttctacatcttggaagagctatatcttatggttcatatttttataatccaaatact
tggttttctggaattattattatcttcttcttaatgggaacagcatttatgggttatgtg
ttacctttaggacaaatgagtttatggggggttacagtaattacaaatttattatctgca
tttccatctttaatagaatggctttgtggaggacattacatttacaatcctacatttaag
aggttctttgtctttcattttctatttccatttcttctttgtggttttcttgtttatcat
atttttaatctacattttctatcttctaataatcctttaaggaattccactaataataaa
atagcatttttccctttcattattagtaaagatttatatggaaagatattaattctctat
ctatatcttcttcaaattcatttcggtttctcttctttctcacatccagataatgcatta
gaagcatgtggattacttactcctttacatatagtacctgaatggtatttcttatgccaa
tatgctatgttaaaagctgtgccaaataaaaatgcaggattcattatattatttacttct
atcttcatcttattttactttatgagaagtctttcaatctcattctattttatcgtgtgg
gtaagctctagatttaatagtttctttgtaattctttggtttttagttttcatatcctta
atttggataggtggtcaatttcctgtagacaactttctatcttatggtcgtatcttgaca
ttacattattattatcttcttatctgtatcttattctcttag

>IS1_cob
atgaaatctcatttacaatcatatccttgtcctcttatcataaattatttatggaatctt
ggtttttattaggggttgctattttattacaaattatatctggaatcttcttaggttta
cattatacatcagatattaattcagcatattttagtattttctttattattagagaagta
tattatggatggtgtttacgttattttcattctaatggttcatcatttgtctttctttt
atatttctacatcttggaagagctatatcttatggttcatatttttataatccaaatact
tggttttctggaattattattatcttcttattaatggcaacagcatttatgggttatgtc
ttacctttaggacaaatgagtttatggggggttacagtaattacaaatttattatctgca
tttccatctttaatagaatggctttgtggaggacattatgtttacaatcttacattgaag
aggttctttgtctttcattttctatttccatttcttctttgtggttttcttctttatcat
atttttaatctacattttctatcttctaataatcctttaaggaattccactaataataaa
atagtatttttccctttcattattagtaaagatctctatggaaagatattaattccctat
ctatattttcttcagattcattttggattctcttctctctcacatccagataatgcattg
gaagcttgtgcattacgtacacctttacacatagtacctgaatggtatttcttatgccaa
tatgctatgttaaaagctgtgccaaataaaaatgcaggattcattatattactaacttct
atctttgtattattttattttatgagaagtctttcagtatctttctattttatcgtgtgg
gtaagctctagatttaatagtttctttgtaattctttggtttttagttttcatatcctta
atttggataggtggtcaatttcctgtcgacaactttctatcttatggtcgtatcttgaca
ttatattattattattttcttatctgtatcttattatcttag
>IS10_cob
atgaaatctcatttacaatcatatccttgtcctctgatcataaattatttttggaatctt
ggtttttattagggattactattttattacaaattatatctggaatcttcttaggttta
cattatacatcagatattaattcagcatattttagtattttctttattattagagaaata
tattatggatggtgtttacgttatcttcattctaatggttcatcatttgtctttcttttg
atatttctacatcttggaagagctatatcttatggttcatatttttataatccaaatact
tggttttctggaattattattatcttcttcttaatgggaacagcatttatgggttatgtg
ttacctttaggacaaatgagtttatggggggttacagtaattacaaatttattatctgca
tttccatctttaatagaatggctttgtggaggacattacatttacaatcctacatttaag
aggttctttgtctttcattttctatttccatttcttctttgtggttttcttgtttatcat
atttttaatctacattttctatcttctaataatcctttaaggaattccactaataataaa
atagcatttttccctttcattattagtaaagatttatatggaaagatattaattctctat
ctatatcttcttcaaattcatttcggtttctcttctttctcacatccagataatgcatta
gaagcatgtggattacttactcctttacatatagtacctgaatggtatttcttatgccaa
tatgctatgttaaaagctgtgccaaataaaaatgcaggattcattatattatttacttct
atcttcatcttattttactttatgagaagtctttcaatctcattctattttatcgtgtgg
gtaagctctagatttaatagtttctttgtaattctttggtttttagttttcatatcctta
atttggataggtggtcaatttcctgtagacaactttctatcttatggtcgtatcttgaca
ttacattattattatcttcttatctgtatcttattatcttag
>IS2_cob
atgaaatctcatttacaatcatatccttgtcctctgatcataaattatttttggaatctt
ggtttttattagggattactattttattacaaattatatctggaatcttcttaggttta
cattatacatcagatattaattcagcatattttagtattttctttattattagagaaata
tattatggatggtgtttacgttatcttcattctaatggttcatcatttgtctttcttttg
atatttctacatcttggaagagctatatcttatggttcatatttttataatccaaatact
tggttttctggaattattattatcttcttcttaatgggaacagcatttatgggttatgtg
ttacctttaggacaaatgagtttatggggggttacagtaattacaaatttattatctgca
tttccatctttaatagaatggctttgtggaggacattacatttacaatcctacatttaag
aggttctttgtctttcattttctatttccatttcttctttgtggttttcttgtttatcat
atttttaatctacattttctatcttctaataatcctttaaggaattccactaataataaa
atagcatttttccctttcattattagtaaagatttatatggaaagatattaattctctat
ctatatcttcttcaaattcatttcggtttctcttctttctcacatccagataatgcatta
gaagcatgtggattacttactcctttacatatagtacctgaatggtatttcttatgccaa
tatgctatgttaaaagctgtgccaaataaaaatgcaggattcattatattatttacttct
atcttcatcttattttactttatgagaagtctttcaatctcattctattttatcgtgtgg
gtaagctctagatttaatagtttctttgtaattctttggtttttagttttcatatcctta
atttggataggtggtcaatttcctgtagacaactttctatcttatggtcgtatcttgaca
ttacattattattatcttcttatctgtatcttattatcttag

>IS3_cob
atgaaatctcatttacaatcatatccttgtcctctgatcataaattatttttggaatctt
ggtttttattagggattactattttattacaaattatatctggaatcttcttaggttta
cattatacatcagatattaattcagcatattttagtattttctttattattagagaaata
tattatggatggtctttacgttatcttcattctaatggttcatcatttgtctttcttttt
atatttctacatcttggaagagctatatcttatggttcatatttttataatccaaatact
tggttttctggaattattattgttttcttcttgatgggaatagcatttatgggttatgtg
ttacctttaggacaaatgagtttatggggggttacagtaattacaaatttattatctcca
tttccatcttttatagaatggctttgtggaggacattacattcacaatcctacattgaag
aggttctttgtctttcattttctatttccatttcttctttgtggttttcttctttatcat
atttttaatctacattttctatcttctaataatccattaagtaattccactaataataaa
atagcatttttgcctttcattattagtaaagatttatatggaaagatattaattctctat
ctatattttcttcaaattcattttggtttctcttctctctcacatccagataatgcattg
gaagcttgtgcattacgtacacctttacacattgtacctgaatggtatttcctatgccaa
tatgctatgttaaaagctgtgccaaataaaaatgcaggattcattgtcttactaacttct
atctttgtattattttattttatgagaagtctttcagtatctttctattttatcgtgtgg
gtaagctctagatttaatagtttctttgtaattctttggtttttagttttcatatcctta
atttggataggtggtcaatttcctgtagacaactttctatcttatggtcgtatcttgaca
ttacattattattatcttcttatctgtatcttattatcttag
>IS4_cob
atgaaatctcatttacaatcatatccttgtcctctgatcataaattatttttggaatctt
ggtttttattagggattactattttattacaaattatatctggaatcttcttaggttta
cattatacatcagatattaattcagcatattttagtattttctttattattagagaaata
tattatggatggtgtttacgttatcttcattctaatggttcatcatttgtctttcttttg
atatttctacatcttggaagagctatatcttatggttcatatttttataatccaaatact
tggttttctggaattattattatcttcttattaatgggaacagcatttatgggttatgtg
ttacctttaggacaaatgagtttatggggtgttacagtaattacaaatttattatctcca
tttccatcttttatagaatggctttgtggaggagattatgtttacaatcttacattgaag
aggttctttgtctttcattttctatttccatttcttctttgtggttttcttgtttatcat
atttttaatctacattttctatcttctaataatcctttaaggaattccactaataataaa
atagcatttttccctttcattattagtaaagatttatatggaaagatattaattctctat
ctatatcttcttcaaattcatttcggtttctcttctttctcacatccagataatgcatta
gaagcatgtggattacttactcctttacatatagtacctgaatggtatttcttatgccaa
tatgctatgttaaaagctgtgccaaataaaaatgcaggattcattatattatttacttct
atcttcatcttattttattttatgagaagtctttcagtatctttctattttatcgtgtgg
gtaagctctagatttaatagtttctttgtaattctttggtttttagttttcatatcctta
atttggataggtggtcaatttcctgtagacaactttctatcttatggtcgtatcttgaca
ttacattattattatcttcttatctgtatcttattatcttag
>IS5_cob
atgaaatctcatttacaatcatatccttgtcctctgatcataaattatttttggaatctt
ggtttttattagggattactattttattacaaattatatctggaatcttcttaggttta
cattatacatcagatattaattcagcatattttagtattttctttattattagagaaata
tattatggatggtgtttacgttatcttcattctaatggttcatcatttgtctttcttttg
atatttctacatcttggaagagctatatcttatggttcatatttttataatccaaatact
tggttttctggaattattattatcttcttcttaatgggaacagcatttatgggttatgtg
ttacctttaggacaaatgagtttatggggggttacagtaattacaaatttattatctgca
tttccatctttaatagaatggctttgtggaggacattacatttacaatcctacatttaag
aggttctttgtctttcattttctatttccatttcttctttgtggttttcttgtttatcat
atttttaatctacattttctatcttctaataatcctttaaggaattccactaataataaa
atagcatttttccctttcattattagtaaagatttatatggaaagatattaattctctat
ctatatcttcttcaaattcatttcggtttctcttctttctcacatccagataatgcatta
gaagcatgtggattacttactcctttacatatagtacctgaatggtatttcttatgccaa
tatgctatgttaaaagctgtgccaaataaaaatgcaggattcattatattatttacttct
atcttcatcttattttactttatgagaagtctttcagtatctttctattttatcgtgtgg
gtaagctctagatttaatagtttctttgtaattctttggtttttagttttcatatcctta
atttggataggtggtcaatttcctgtagacaactttctatcttatggtcgtatcttgaca
ttacattattattatcttcttatctgtatcttattatcttag

>IS6_cob
atgaaatctcatttacaatcatatccttgtcctctgatcataaattatttttggaatctt
ggtttttattagggattactattttattacaaattatatctggaatcttcttaggttta
cattatacatcagatattaattcagcatattttagtattttctttattattagagaaata
tattatggatggtgtttacgttatcttcattctaatggttcatcatttgtctttcttttg
atatttctacatcttggaagagctatatcttatggttcatatttttataatccaaatact
tggttttctggaattattattatcttcttcttaatgggaacagcatttatgggttatgtg
ttacctttaggacaaatgagtttatggggggttacagtaattacaaatttattatctgca
tttccatctttaatagaatggctttgtggaggacattacatttacaatcctacatttaag
aggttctttgtctttcattttctatttccatttcttctttgtggttttcttgtttatcat
atttttaatctacattttctatcttctaataatcctttaaggaattccactaataataaa
atagcatttttccctttcattattagtaaagatttatatggaaagatattaattctctat
ctatatcttcttcaaattcatttcggtttctcttctttctcacatccagataatgcatta
gaagcatgtggattacttactcctttacatatagtacctgaatggtatttcttatgccaa
tatgctatgttaaaagctgtgccaaataaaaatgcaggattcattatattatttacttct
atcttcatcttattttactttatgagaagtctttcagtatctttctattttatcgtgtgg
gtaagctctagatttaatagtttctttgtaattctttggtttttagttttcatatcctta
atttggataggtggtcaatttcctgtagacaactttctatcttatggtcgtatcttgaca
ttacattattattatcttcttatctgtatcttattatcttag
>IS7_cob
atgaaatctcatttacaatcatatccttgtcctctgatcataaattatttttggaatctt
ggtttttattagggattactattttattacaaattatatctggaatcttcttaggttta
cattatacatcagatattaattcagcatattttagtattttctttattattagagaaata
tattatggatggtgtttacgttatcttcattctaatggttcatcatttgtttttcttttt
atatttctacatcttggaagagctatatcttatggttcatatttttataatccaaatact
tggttttctggaattattattatcttcttcttaatgggaacagcatttatgggttatgtg
ttacctttaggacaaatgagtttatggggagttacagtaattacaaatttattatctgca
tttccatctttaatagaatggctttgtggaggacattacatttacaatcctacattgaag
aggttctttgtctttcattttctatttccatttattctttgtggttttcttctttatcat
atttttaatctacattttctatcttctaataatcctttaaggaattccactaataataaa
atagcatttttccctttcattattagtaaagatttatatggaaagatattaattctctat
ctatatcttcttcaaattcatttcggtttctcttctttctcacatccagataatgcatta
gaagcatgtggattacgtactcctttacatatagtacctgaatggtatttcttatgccaa
tatgctatgttaaaagctgtgccaaataaaaatgcaggattcattatattatttacttct
atcttcatcttattttactttatgagaagtctttcagtatctttctattttatcgtgtgg
gtaagctctagatttaatagtttctttgtaattctttggtttttagttttcatatcctta
atttggataggtggtcaatttcctgtagacaactttctatcttatggtcgtatcttgaca
ttacattattattatcttcttatctgtatcttattatcttag
>IS8_cob
atgaaatctcatttacaatcatatccttgtcctctgatcataaattatttttggaatctt
ggtttttattagggattactattttattacaaattatatctggaatcttcttaggttta
cattatacatcagatattaattcagcatattttagtattttctttattattagagaaata
tattatggatggtgtttacgttatcttcattctaatggttcatcatttgtctttcttttg
atatttctacatcttggaagagctatatcttatggttcatatttttataatccaaatact
tggttttctggaattattattatcttcttcttaatgggaacagcatttatgggttatgtg
ttacctttaggacaaatgagtttatggggggttacagtaattacaaatttattatctgca
tttccatctttaatagaatggctttgtggaggacattacatttacaatcctacatttaag
aggttctttgtctttcattttctatttccatttcttctttgtggttttcttgtttatcat
atttttaatctacattttctatcttctaataatcctttaaggaattccactaataataaa
atagcatttttccctttcattattagtaaagatttatatggaaagatattaattctctat
ctatatcttcttcaaattcatttcggtttctcttctttctcacatccagataatgcatta
gaagcatgtggattacttactcctttacatatagtacctgaatggtatttcttatgccaa
tatgctatgttaaaagctgtgccaaataaaaatgcaggattcattatattatttacttct
atcttcatcttattttactttatgagaagtctttcaatctcattctattttatcgtgtgg
gtaagctctagatttaatagtttctttgtaattctttggtttttagttttcatatcctta
atttggataggtggtcaatttcctgtagacaactttctatcttatggtcgtatcttgaca
ttacattattattatcttcttatctgtatcttattatcttag

>IS9_cob
atgaaatctcatttacaatcatatccttgtcctctgatcataaattatttttggaatctt
ggtttttattagggattactattttattacaaattatatctggaatcttcttaggttta
cattatacatcagatattaattcagcatattttagtattttctttattattagagaaata
tattatggatggtgtttacgttatcttcattctaatggttcatcatttgtctttcttttg
atatttctacatcttggaagagctatatcttatggttcatatttttataatccaaatact
tggttttctggaattattattatcttcttcttaatgggaacagcatttatgggttatgtg
ttacctttaggacaaatgagtttatggggggttacagtaattacaaatttattatctgca
tttccatctttaatagaatggctttgtggaggacattacatttacaatcctacatttaag
aggttctttgtctttcattttctatttccatttcttctttgtggttttcttgtttatcat
atttttaatctacattttctatcttctaataatcctttaaggaattccactaataataaa
atagcatttttccctttcattattagtaaagatttatatggaaagatattaattctctat
ctatatcttcttcaaattcatttcggtttctcttctttctcacatccagataatgcatta
gaagcatgtggattacttactcctttacatatagtacctgaatggtatttcttatgccaa
tatgctatgttaaaagctgtgccaaataaaaatgcaggattcattatattatttacttct
atcttcatcttattttactttatgagaagtctttcagtatctttctattttatcgtgtgg
gtaagctctagatttaatagtttctttgtaattctttggtttttagttttcatatcctta
atttggataggtggtcaatttcctgtagacaactttctatcttatggtcgtatcttgaca
ttacattattattatcttcttatctgtatcttattatcttag
>Ik1_cob
atgaaatctcatttacaatcatatccttgtcctctgatcataaattatttttggaatctt
ggtttttattagggattactattttattacaaattatatctggaatcttcttaggttta
cattatacatcagatattaattcagcatattttagtattttctttattattagagaaata
tattatggatggtgtttacgttatcttcattctaatggttcatcatttgtctttcttttg
atatttctacatcttggaagagctatatcttatggttcatatttttataatccaaatact
tggttttctggaattattattatcttcttcttaatgggaacagcatttatgggttatgtg
ttacctttaggacaaatgagtttatggggggttacagtaattacaaatttattatctgca
tttccatctttaatagaatggctttgtggaggacattacatttacaatcctacatttaag
aggttctttgtctttcattttctatttccatttcttctttgtggttttcttgtttatcat
atttttaatctacattttctatcttctaataatcctttaaggaattccactaataataaa
atagcatttttccctttcattattagtaaagatttatatggaaagatattaattctctat
ctatatcttcttcaaattcatttcggtttctcttctttctcacatccagataatgcatta
gaagcatgtggattacttactcctttacatatagtacctgaatggtatttcttatgccaa
tatgctatgttaaaagctgtgccaaataaaaatgcaggattcattatattatttacttct
atcttcatcttattttactttatgagaagtctttcagtatctttctattttatcgtgtgg
gtaagctctagatttaatagtttctttgtaattctttggtttttagttttcatatcctta
atttggataggtggtcaatttcctgtagacaactttctatcttatggtcgtatcttgaca
ttacattattattatcttcttatctgtatcttattatcttag
>Ik2_cob
atgaaatctcatttacaatcatatccttgtcctctgatcataaattatttttggaatctt
ggtttttattagggattactattttattacaaattatatctggaatcttcttaggttta
cattatacatcagatattaattcagcatattttagtattttctttattattagagaaata
tattatggatggtgtttacgttatcttcattctaatggttcatcatttgtctttcttttg
atatttctacatcttggaagagctatatcttatggttcatatttttataatccaaatact
tggttttctggaattattattatcttcttcttaatgggaacagcatttatgggttatgtg
ttacctttaggacaaatgagtttatggggggttacagtaattacaaatttattatctgca
tttccatctttaatagaatggctttgtggaggacattacatttacaatcctacatttaag
aggttctttgtctttcattttctatttccatttcttctttgtggttttcttgtttatcat
atttttaatctacattttctatcttctaataatcctttaaggaattccactaataataaa
atagcatttttccctttcattattagtaaagatttatatggaaagatattaattctctat
ctatatcttcttcaaattcatttcggtttctcttctttctcacatccagataatgcatta
gaagcatgtggattacttactcctttacatatagtacctgaatggtatttcttatgccaa
tatgctatgttaaaagctgtgccaaataaaaatgcaggattcattatattatttacttct
atcttcatcttattttactttatgagaagtctttcagtatctttctattttatcgtgtgg
gtaagctctagatttaatagtttctttgtaattctttggtttttagttttcatatcctta
atttggataggtggtcaatttcctgtagacaactttctatcttatggtcgtatcttgaca
ttacattattattatcttcttatctgtatcttattatcttag

>Ik3_cob
atgaaatctcatttacaatcatatccttgtcctctgatcataaattatttttggaatctt
ggtttttattagggattactattttattacaaattatatctggaatcttcttaggttta
cattatacatcagatattaattcagcatattttagtattttctttattattagagaaata
tattatggatggtgtttacgttatcttcattctaatggttcatcatttgtctttcttttg
atatttctacatcttggaagagctatatcttatggttcatatttttataatccaaatact
tggttttctggaattattattatcttcttcttaatgggaacagcatttatgggttatgtg
ttacctttaggacaaatgagtttatggggggttacagtaattacaaatttattatctgca
tttccatctttaatagaatggctttgtggaggacattacatttacaatcctacatttaag
aggttctttgtctttcattttctatttccatttcttctttgtggttttcttgtttatcat
atttttaatctacattttctatcttctaataatcctttaaggaattccactaataataaa
atagcatttttccctttcattattagtaaagatttatatggaaagatattaattctctat
ctatatcttcttcaaattcatttcggtttctcttctttctcacatccagataatgcatta
gaagcatgtggattacttactcctttacatatagtacctgaatggtatttcttatgccaa
tatgctatgttaaaagctgtgccaaataaaaatgcaggattcattatattatttacttct
atcttcatcttattttactttatgagaagtctttcagtatctttctattttatcgtgtgg
gtaagctctagatttaatagtttctttgtaattctttggtttttagttttcatatcctta
atttggataggtggtcaatttcctgtagacaactttctatcttatggtcgtatcttgaca
ttacattattattatcttcttatctgtatcttattatcttag
>Ik4_cob
atgaaatctcatttacaatcatatccttgtcctctgatcataaattatttttggaatctt
ggtttttattagggattactattttattacaaattatatctggaatcttcttaggttta
cattatacatcagatattaattcagcatattttagtattttctttattattagagaaata
tattatggatggtgtttacgttatcttcattctaatggttcatcatttgtctttcttttg
atatttctacatcttggaagagctatatcttatggttcatatttttataatccaaatact
tggttttctggaattattattatcttcttcttaatgggaacagcatttatgggttatgtg
ttacctttaggacaaatgagtttatggggggttacagtaattacaaatttattatctgca
tttccatctttaatagaatggctttgtggaggacattacatttacaatcctacatttaag
aggttctttgtctttcattttctatttccatttcttctttgtggttttcttgtttatcat
atttttaatctacattttctatcttctaataatcctttaaggaattccactaataataaa
atagcatttttccctttcattattagtaaagatttatatggaaagatattaattctctat
ctatatcttcttcaaattcatttcggtttctcttctttctcacatccagataatgcatta
gaagcatgtggattacttactcctttacatatagtacctgaatggtatttcttatgccaa
tatgctatgttaaaagctgtgccaaataaaaatgcaggattcattatattatttacttct
atcttcatcttattttactttatgagaagtctttcagtatctttctattttatcgtgtgg
gtaagctctagatttaatagtttctttgtaattctttggtttttagttttcatatcctta
atttggataggtggtcaatttcctgtagacaactttctatcttatggtcgtatcttgaca
ttacattattattatcttcttatctgtatcttattatcttag
>Ik5_cob
atgaaatctcatttacaatcatatccttgtcctctgatcataaattatttttggaatctt
ggtttttattagggattactattttattacaaattatatctggaatcttcttaggttta
cattatacatcagatattaattcagcatattttagtattttctttattattagagaaata
tattatggatggtgtttacgttatcttcattctaatggttcatcatttgtctttcttttg
atatttctacatcttggaagagctatatcttatggttcatatttttataatccaaatact
tggttttctggaattattattatcttcttcttaatgggaacagcatttatgggttatgtg
ttacctttaggacaaatgagtttatggggggttacagtaattacaaatttattatctgca
tttccatctttaatagaatggctttgtggaggacattacatttacaatcctacatttaag
aggttctttgtctttcattttctatttccatttcttctttgtggttttcttgtttatcat
atttttaatctacattttctatcttctaataatcctttaaggaattccactaataataaa
atagcatttttccctttcattattagtaaagatttatatggaaagatattaattctctat
ctatatcttcttcaaattcatttcggtttctcttctttctcacatccagataatgcatta
gaagcatgtggattacttactcctttacatatagtacctgaatggtatttcttatgccaa
tatgctatgttaaaagctgtgccaaataaaaatgcaggattcattatattatttacttct
atcttcatcttattttactttatgagaagtctttcaatctcattctattttatcgtgtgg
gtaagctctagatttaatagtttctttgtaattctttggtttttagttttcatatcctta
atttggataggtggtcaatttcctgtagacaactttctatcttatggtcgtatcttgaca
ttacattattattatcttcttatctgtatcttattatcttag

>Ik6_cob
atgaaatctcatttacaatcatatccttgtcctctgatcataaattatttttggaatctt
ggtttttattagggattactattttattacaaattatatctggaatcttcttaggttta
cattatacatcagatattaattcagcatattttagtattttctttattattagagaaata
tattatggatggtgtttacgttatcttcattctaatggttcatcatttgtctttcttttg
atatttctacatcttggaagagctatatcttatggttcatatttttataatccaaatact
tggttttctggaattattattatcttcttcttaatgggaacagcatttatgggttatgtg
ttacctttaggacaaatgagtttatggggggttacagtaattacaaatttattatctgca
tttccatctttaatagaatggctttgtggaggacattacatttacaatcctacatttaag
aggttctttgtctttcattttctatttccatttcttctttgtggttttcttgtttatcat
atttttaatctacattttctatcttctaataatcctttaaggaattccactaataataaa
atagcatttttccctttcattattagtaaagatttatatggaaagatattaattctctat
ctatatcttcttcaaattcatttcggtttctcttctttctcacatccagataatgcatta
gaagcatgtggattacttactcctttacatatagtacctgaatggtatttcttatgccaa
tatgctatgttaaaagctgtgccaaataaaaatgcaggattcattatattatttacttct
atcttcatcttattttactttatgagaagtctttcagtctcattctattttatcgtgtgg
gtaagctctagatttaatagtttctttgtaattctttggtttttagttttcatatcctta
atttggataggtggtcaatttcctgtagacaactttctatcttatggtcgtatcttgaca
ttacattattattatcttcttatctgtatcttattatcttag
>Ik7_cob
atgaaatctcatttacaatcatatccttgtcctctgatcataaattatttttggaatctt
ggtttttattagggattactattttattacaaattatatctggaatcttcttaggttta
cattatacatcagatattaattcagcatattttagtattttctttattattagagaaata
tattatggatggtgtttacgttatcttcattctaatggttcatcatttgtctttcttttg
atatttctacatcttggaagagctatatcttatggttcatatttttataatccaaatact
tggttttctggaattattattatcttcttcttaatgggaacagcatttatgggttatgtg
ttacctttaggacaaatgagtttatggggggttacagtaattacaaatttattatctgca
tttccatctttaatagaatggctttgtggaggacattacatttacaatcctacatttaag
aggttctttgtctttcattttctatttccatttcttctttgtggttttcttgtttatcat
atttttaatctacattttctatcttctaataatcctttaaggaattccactaataataaa
atagcatttttccctttcattattagtaaagatttatatggaaagatattaattctctat
ctatatcttcttcaaattcatttcggtttctcttctttctcacatccagataatgcatta
gaagcatgtggattacttactcctttacatatagtacctgaatggtatttcttatgccaa
tatgctatgttaaaagctgtgccaaataaaaatgcaggattcattatattatttacttct
atcttcatcttattttactttatgagaagtctttcagtatctttctattttatcgtgtgg
gtaagctctagatttaatagtttctttgtaattctttggtttttagttttcatatcctta
atttggataggtggtcaatttcctgtagacaactttctatcttatggtcgtatcttgaca
ttacattattattatcttcttatctgtatcttattatcttag
>Ik8_cob
atgaaatctcatttacaatcatatccttgtcctctgatcataaattatttttggaatctt
ggtttttattagggattactattttattacaaattatatctggaatcttcttaggttta
cattatacatcagatattaattcagcatattttagtattttctttattattagagaaata
tattatggatggtgtttacgttatcttcattctaatggttcatcatttgtctttcttttg
atatttctacatcttggaagagctatatcttatggttcatatttttataatccaaatact
tggttttctggaattattattatcttcttcttaatgggaacagcatttatgggttatgtg
ttacctttaggacaaatgagtttatggggggttacagtaattacaaatttattatctgca
tttccatctttaatagaatggctttgtggaggacattacatttacaatcctacatttaag
aggttctttgtctttcattttctatttccatttcttctttgtggttttcttgtttatcat
atttttaatctacattttctatcttctaataatcctttaaggaattccactaataataaa
atagcatttttccctttcattattagtaaagatttatatggaaagatattaattctctat
ctatatcttcttcaaattcatttcggtttctcttctttctcacatccagataatgcatta
gaagcatgtggattacttactcctttacatatagtacctgaatggtatttcttatgccaa
tatgctatgttaaaagctgtgccaaataaaaatgcaggattcattatattatttacttct
atcttcatcttattttactttatgagaagtctttcagtatctttctattttatcgtgtgg
gtaagctctagatttaatagtttctttgtaattctttggtttttagttttcatatcctta
atttggataggtggtcaatttcctgtagacaactttctatcttatggtcgtatcttgaca
ttacattattattatcttcttatctgtatcttattatcttag

>Ik9_cob
atgaaatctcatttacaatcatatccttgtcctctgatcataaattatttttggaatctt
ggtttttattagggattactattttattacaaattatatctggaatcttcttaggttta
cattatacatcagatattaattcagcatattttagtattttctttattattagagaaata
tattatggatggtgtttacgttatcttcattctaatggttcatcatttgtctttcttttg
atatttctacatcttggaagagctatatcttatggttcatatttttataatccaaatact
tggttttctggaattattattatcttcttcttaatgggaacagcatttatgggttatgtg
ttacctttaggacaaatgagtttatggggggttacagtaattacaaatttattatctgca
tttccatctttaatagaatggctttgtggaggacattacatttacaatcctacatttaag
aggttctttgtctttcattttctatttccatttcttctttgtggttttcttgtttatcat
atttttaatctacattttctatcttctaataatcctttaaggaattccactaataataaa
atagcattttgcctttcattattagtaaagatttatatggaaagatattaattctctat
ctatatcttcttcaaattcatttcggtttctcttctttctcacatccagataatgcatta
gaagcatgtggattacttactcctttacatatagtacctgaatggtatttcttatgccaa
tatgctatgttaaaagctgtgccaaataaaaatgcaggattcattatattatttacttct
atcttcatcttattttactttatgagaagtctttcagtatctttctattttatcgtgtgg
gtaagctctagatttaatagtttctttgtaattctttggtttttagttttcatatcctta
atttggataggtggtcaatttcctgtagacaactttctatcttatggtcgtatcttgaca
ttacattattattatcttcttatctgtatcttattatcttag
>Irm10_cob
atgaaatctcatttacaatcatatccttgtcctctgatcataaattatttttggaatctt
ggtttttattagggattactattttattacaaattatatctggaatcttcttaggttta
cattatacatcagatattaattcagcatattttagtattttctttattattagagaaata
tattatggatggtgtttacgttatcttcattctaatggttcatcatttgtctttcttttg
atatttctacatcttggaagagctatatcttatggttcatatttttataatccaaatact
tggttttctggaattattattatcttcttcttaatgggaacagcatttatgggttatgtg
ttacctttaggacaaatgagtttatggggggttacagtaattacaaatttattatctgca
tttccatctttaatagaatggctttgtggaggacattacatttacaatcctacatttaag
aggttctttgtctttcattttctatttccatttcttctttgtggttttcttgtttatcat
atttttaatctacattttctatcttctaataatcctttaaggaattccactaataataaa
atagcatttttccctttcattattagtaaagatttatatggaaagatattaattctctat
ctatatcttcttcaaattcatttcggtttctcttctttctcacatccagataatgcatta
gaagcatgtggattacttactcctttacatatagtacctgaatggtatttcttatgccaa
tatgctatgttaaaagctgtgccaaataaaaatgcaggattcattatattatttacttct
atcttcatcttattttactttatgagaagtctttcaatctcattctattttatcgtgtgg
gtaagctctagatttaatagtttctttgtaattctttggtttttagttttcatatcctta
atttggataggtggtcaatttcctgtagacaactttctatcttatggtcgtatcttgaca
ttacattattattatcttcttatctgtatcttattatcttag
>Irm17_cob
atgaaatctcatttacaatcatatccttgtcctctgatcataaattatttttggaatctt
ggtttttattagggattactattttattacaaattatatctggaatcttcttaggttta
cattatacatcagatattaattcagcatattttagtattttctttattattagagaaata
tattatggatggtgtttacgttatcttcattctaatggttcatcatttgtctttcttttg
atatttctacatcttggaagagctatatcttatggttcatatttttataatccaaatact
tggttttctggaattattattatcttcttcttaatgggaacagcatttatgggttatgtg
ttacctttaggacaaatgagtttatggggggttacagtaattacaaatttattatctgca
tttccatctttaatagaatggctttgtggaggacattacatttacaatcctacatttaag
aggttctttgtctttcattttctatttccatttcttctttgtggttttcttgtttatcat
atttttaatctacattttctatcttctaataatcctttaaggaattccactaataataaa
atagcatttttccctttcattattagtaaagatttatatggaaagatattaattctctat
ctatatcttcttcaaattcatttcggtttctcttctttctcacatccagataatgcatta
gaagcatgtggattacttactcctttacatatagtacctgaatggtatttcttatgccaa
tatgctatgttaaaagctgtgccaaataaaaatgcaggattcattatattatttacttct
atcttcatcttattttactttatgagaagtctttcaatctcattctattttatcgtgtgg
gtaagctctagatttaatagtttctttgtaattctttggtttttagttttcatatcctta
atttggataggtggtcaatttcctgtagacaactttctatcttatggtcgtatcttgaca
ttacattattattatcttcttatctgtatcttattatcttag

>Irm2_cob
atgaaatctcatttacaatcatatccttgtcctctgatcataaattatttttggaatctt
ggtttttattagggattactattttattacaaattatatctggaatcttcttaggttta
cattatacatcagatattaattcagcatattttagtattttctttattattagagaaata
tattatggatggtgtttacgttatcttcattctaatggttcatcatttgtctttcttttg
atatttctacatcttggaagagctatatcttatggttcatatttttataatccaaatact
tggttttctggaattattattatcttcttcttaatgggaacagcatttatgggttatgtg
ttacctttaggacaaatgagtttatggggggttacagtaattacaaatttattatctgca
tttccatctttaatagaatggctttgtggaggacattacatttacaatcctacatttaag
aggttctttgtctttcattttctatttccatttcttctttgtggttttcttgtttatcat
atttttaatctacattttctatcttctaataatcctttaaggaattccactaataataaa
atagcatttttccctttcattattagtaaagatttatatggaaagatattaattctctat
ctatatcttcttcaaattcatttcggtttctcttctttctcacatccagataatgcatta
gaagcatgtggattacttactcctttacatatagtacctgaatggtatttcttatgccaa
tatgctatgttaaaagctgtgccaaataaaaatgcaggattcattatattatttacttct
atcttcatcttattttactttatgagaagtctttcaatctcattctattttatcgtgtgg
gtaagctctagatttaatagtttctttgtaattctttggtttttagttttcatatcctta
atttggataggtggtcaatttcctgtagacaactttctatcttatggtcgtatcttgaca
ttacattattattatcttcttatctgtatcttattatcttag
>Irm21_cob
atgaaatctcatttacaatcatatccttgtcctctgatcataaattatttttggaatctt
ggtttttattagggattactattttattacaaattatatctggaatcttcttaggttta
cattatacatcagatattaattcagcatattttagtattttctttattattagagaaata
tattatggatggtgtttacgttatcttcattctaatggttcatcatttgtttttcttttg
atatttctacatcttggaagagctatatcttatggttcatatttttataatccaaatact
tggttttctggaattattattatcttcttcttaatgggaacagcatttatgggttatgtg
ttacctttaggacaaatgagtttatggggagttacagtaattacaaatttattatctgca
tttccatctttaatagaatggctttgtggaggacattacatttacaatcctacatttaag
aggttctttgtctttcattttctatttccatttcttctttgtggttttcttgtttatcat
atttttaatctacattttctatcttctaataatcctttaaggaattccactaataataaa
atagcatttttccctttcattattagtaaagatttatatggaaagatattaattctctat
ctatatcttcttcaaattcatttcggtttctcttctttctcacatccagataatgcatta
gaagcatgtggattacttactcctttacatatagtacctgaatggtatttcttatgccaa
tatgctatgttaaaagctgtgccaaataaaaatgcaggattcattatattatttacttct
atcttcatcttattttactttatgagaagtctttcagtatctttctattttatcgtgtgg
gtaagctctagatttaatagtttctttgtaattctttggtttttagttttcatatcctta
atttggataggtggtcaatttcctgtagacaactttctatcttatggtcgtatcttgaca
ttacattattattatcttcttatctgtatcttattatcttag
>Irm22_cob
atgaaatctcatttacaatcatatccttgtcctctgatcataaattatttttggaatctt
ggtttttattagggattactattttattacaaattatatctggaatcttcttaggttta
cattatacatcagatattaattcagcatattttagtattttctttattattagagaaata
tattatggatggtgtttacgttatcttcattctaatggttcatcatttgtctttcttttg
atatttctacatcttggaagagctatatcttatggttcatatttttataatccaaatact
tggttttctggaattattattgttttcttcttgatgggaacagcatttatgggttatgtg
ttacctttaggacaaatgagtttatggggggttacagtaattacaaatttattatctgca
tttccatctttaatagaatggctttgtggaggacattacatttacaatcctacatttaag
aggttctttgtctttcattttctatttccatttcttctttgtggttttcttctttatcat
atttttaatctacattttctatcttctaataatcctttaaggaattccactaataataaa
atagcatttttccctttcattattagtaaagatttctatggaatgatattaattccctat
ctatatcttcttcagattcattttggattctcttctctctcacatccagataatgcatta
gaagcatgtggattacttactcctttacatatagtacctgaatggtatttcttatgccaa
tatgctatgttaaaagctgtgccaaataaaaatgcaggattcattatattatttacttct
atctttgtattattttattttatgagaagtctttcagtatctttctattttatcgtgtgg
gtaagctctagatttaatagtttctttgtaattctttggtttttagttttcatatcctta
atttggataggtggtcaatttcctgtagacaactttctatcttatggtcgtatcttgaca
ttacattattattatcttcttatctgtatcttattatcttag

>Irm23_cob
atgaaatctcatttacaatcatatccttgtcctctgatcataaattatttttggaatctt
ggtttttattagggattactattttattacaaattatatctggaatcttcttaggttta
cattatacatcagatattaattcagcatattttagtattttctttattattagagaaata
tattatggatggtgtttacgttatcttcattctaatggttcatcatttgtctttcttttg
atatttctacatcttggaagagctatatcttatggttcatatttttataatccaaatact
tggttttctggaattattattatcttcttcttaatgggaacagcatttatgggttatgtg
ttacctttaggacaaatgagtttatggggggttacagtaattacaaatttattatctgca
tttccatctttaatagaatggctttgtggaggacattacatttacaatcctacatttaag
aggttctttgtctttcattttctatttccatttcttctttgtggttttcttgtttatcat
atttttaatctacattttctatcttctaataatcctttaaggaattccactaataataaa
atagcatttttccctttcattattagtaaagatttatatggaaagatattaattctctat
ctatatcttcttcaaattcatttcggtttctcttctttctcacatccagataatgcatta
gaagcatgtggattacttactcctttacatatagtacctgaatggtatttcttatgccaa
tatgctatgttaaaagctgtgccaaataaaaatgcaggattcattatattatttacttct
atcttcatcttattttactttatgagaagtctttcaatctcattctattttatcgtgtgg
gtaagctctagatttaatagtttctttgtaattctttggtttttagttttcatatcctta
atttggataggtggtcaatttcctgtagacaactttctatcttatggtcgtatcttgaca
ttacattattattatcttcttatctgtatcttattatcttag
>Irm24_cob
atgaaatctcatttacaatcatatccttgtcctctgatcataaattatttttggaatctt
ggtttttattagggattactattttattacaaattatatctggaatcttcttaggttta
cattatacatcagatattaattcagcatattttagtattttctttattattagagaaata
tattatggatggtgtttacgttatcttcattctaatggttcatcatttgtctttcttttt
atatttctacatcttggaagagctatatcttatggttcatatttttataatccaaatact
tggttttctggaattattattatcttcttcttaatgggaacagcatttatgggttatgtg
ttacctttaggacaaatgagtttatggggggttacagtaattacaaatttattatctgca
tttccatctttaatagaatggctttgtggaggatattgcattcacaatcctacatttaag
aggttctttgtctttcattttctacttccatttcttctttgtggttttcttctttatcat
atttttaatctacattttctatcttctaataatcctttaaggaattccactaataataaa
atagcatttttccctttcattattagtaaagatttatatggaaagatattaatttcctat
ctatatcttcttcaaattcatttcggtttctcttctttctcacatccagataatgcatta
gaagcatgtggattacttactcctttacatatagtacctgaatggtatttcttatgccaa
tatgctatgttaaaagctgtgccaaataaaaatgcaggattcattgtcttactaacttct
atctttgtattattttattttatgagaagtctttcagtatctttctattttatcgtgtgg
gtaagctctagatttaatagtttctttgtaattctttggtttttagttttcatatcctta
atttggataggtggtcaatttcctgtagacaactttctatcttatggtcgtatcttgaca
ttatattattattattttcttatctgtatcttattatcttag
>Irm25_cob
atgaaatctcatttacaatcatatccttgtcctctgatcataaattatttttggaatctt
ggtttttattagggattactattttattacaaattatatctggaatcttcttaggttta
cattatacatcagatattaattcagcatattttagtattttctttattattagagaaata
tattatggatggtgtttacgttatcttcattctaatggttcatcatttgtctttcttttg
atatttctacatcttggaagagctatatcttatggttcatatttttataatccaaatact
tggttttctggaattattattatcttcttcttaatgggaacagcatttatgggttatgtg
ttacctttaggacaaatgagtttatggggggttacagtaattacaaatttattatctgca
tttccatctttaatagaatggctttgtggaggacattacatttacaatcctacatttaag
aggttctttgtctttcattttctatttccatttcttctttgtggttttcttgtttatcat
atttttaatctacattttctatcttctaataatcctttaaggaattccactaataataaa
atagcatttttccctttcattattagtaaagatttatatggaaagatattaattctctat
ctatatcttcttcaaattcatttcggtttctcttctttctcacatccagataatgcatta
gaagcatgtggattacttactcctttacatatagtacctgaatggtatttcttatgccaa
tatgctatgttaaaagctgtgccaaataaaaatgcaggattcattgtcttactaacttct
atctttgtattattttattttatgagaagtctttcagtatctttctattttatcgtgtgg
gtaagctctagatttaatagtttctttgtaattctttggtttttagttttcatatcctta
atttggataggtggtcaatttcctgtagacaactttctatcttatggtcgtatcttgaca
ttacattattattatcttcttatctgtatcttattatcttag

>Irm26_cob
atgaaatctcatttacaatcatatccttgtcctctgatcataaattatttttggaatctt
ggtttttattagggattactattttattacaaattatatctggaatcttcttaggttta
cattatacatcagatattaattcagcatattttagtattttctttattattagagaaata
tattatggatggtgtttacgttatcttcattctaatggttcatcatttgtctttcttttg
atatttctacatcttggaagagctatatcttatggttcatatttttataatccaaatact
tggttttctggaattattattatcttcttcttaatgggaacagcatttatgggttatgtg
ttacctttaggacaaatgagtttatggggggttacagtaattacaaatttattatctgca
tttccatctttaatagaatggctttgtggaggacattacatttacaatcctacatttaag
aggttctttgtctttcattttctatttccatttcttctttgtggttttcttgtttatcat
atttttaatctacattttctatcttctaataatcctttaaggaattccactaataataaa
atagcatttttccctttcattattagtaaagatttatatggaaagatattaattctctat
ctatatcttcttcaaattcatttcggtttctcttctttctcacatccagataatgcatta
gaagcatgtggattacttactcctttacatatagtacctgaatggtatttcttatgccaa
tatgctatgttaaaagctgtgccaaataaaaatgcaggattcattatattatttacttct
atcttcatcttattttactttatgagaagtctttcagtatctttctattttatcgtgtgg
gtaagctctagatttaatagtttctttgtaattctttggtttttagttttcatatcctta
atttggataggtggtcaatttcctgtagacaactttctatcttatggtcgtatcttgaca
ttacattattattatcttcttatctgtatcttattatcttag
>Irm27_cob
atgaaatctcatttacaatcatatccttgtcctctgatcataaattatttatggaatctt
ggtttttattagggattactattttattacaaattatatctggtatcttcttaggttta
cattatacatcagaaattaattcagcatattttagtattttctttattattagagaaata
tattatggatggtctttacgttatcttcattctaatggttcatcatttgtctttcttttt
atatttctacatcttggaagagctatatcttatggttcatatttttataatccaaatact
tggttttctggaattattattgttttcttcttgatgggaatagcatttatgggttatgtg
ttacctttaggacaaatgagtttatggggggttacagtaattacaaatttattatctcca
tttccatcttttatagaatggctttgtggaggagattatgtttacaatcttacattgaag
aggttctttgtctttcattttctatttccatttcttctttgtggttttcttctttatcat
atttttaatctacattttctatcttctaataatccattaagtaattccactaataataaa
atagcatttttgcctttcattattagtaaagatctctatggaatgatattaatttcctat
ctatattttcttcagattcattttggattctcttctctctcacatccagataatgcattg
gaagcttgtgcattacgtacacctttacatatagtacctgaatggtatttcttatgccaa
tatgctatgttaaaagctgtgccaaataaaaatgcaggattcattgtcttactaacttct
atctttgtattattttattttatgagaagtctttcagtatctttctattttatcgtgtgg
gtaagctctagatttaatagtttctttgtaattctttggtttttagttttcatatcctta
atttggataggtggtcaatttcctgtagacaactttctatcttatggtcgtatcttgaca
ttacattattattatcttcttatctgtatcttattatcttag
>Irm3_cob
atgaaatctcatttacaatcatatccttgtcctcttatcataaattatttatggaatctt
ggtttttattaggggttgctattttattacaaattatatctggtatcttcttaggttta
cattatacatcagaaattaattcagcatattttagtattttctttattattagagaaata
tattatggatggtgtttacgttattttcattctaatggttcatcatttgtctttcttttt
atatttctacatcttggaagagctatatcttatggttcatatttttataatccaaatact
tggttttctggaattattattatcttcttattaatggcaacagcatttatgggttatgtc
ttacctttaggacaaatgagtttatggggtgttacagtaattacaaatttattatctcca
tttccatcttttatagaatggctttgtggaggagattatgtttacaatcttacattgaag
aggttctttgtctttcattttctatttccatttcttctttgtggttttcttctttatcat
atttttaatctacattttctatcttctaataatccattaagtaattccactaataataaa
atagtatttttccctttcattattagtaaagatttctatggaaagatattaattctctat
ctatatcttcttcaaattcatttcggtttctcttctttctcacatccagataatgcatta
gaagcatgtggattacttactcctttacatatagtacctgaatggtatttcttatgccaa
tatgctatgttaaaagctgtgccaaataaaaatgcaggattcattatattatttacttct
atcttcatcttattttactttatgagaagtctttcagtatctttctattttatcgtgtgg
gtaagctctagatttaatagtttctttgtaattctttggtttttagttttcatatcctta
atttggataggtggtcaatttcctgtagacaactttctatcttatggtcgtatcttgaca
ttacattattattatcttcttatctgtatcttattatcttag

>Irm4_cob
atgaaatctcatttacaatcatatccttgtcctcttatcataaattatttttggaatctt
ggtttttattagggattactattttattacaaattatatctggtatcttcttaggttta
cattatacatcagaaattaattcagcatattttagtattttctttattattagagaaata
tattatggatggtctttacgttatcttcattctaatggttcatcatttgtctttcttttg
atatttctacatcttggaagagctatatcttatggttcatatttttataatccaaatact
tggttttctggaattattattatcttcttattaatggcaacagcatttatgggttatgtc
ttacctttaggacaaatgagtttatggggtgttacagtaattacaaatttattatctcca
tttccatcttttatagaatggctttgtggaggacattatgtttacaatcttacattgaag
aggttctttgtctttcattttctatttccatttcttctttgtggttttcttgtttatcat
atttttaatctacattttctatcttctaataatccattaagtaattccactaataataaa
atagtatttttccctttcattattagtaaagatttatatggaaagatattaattctctat
ctatatcttcttcaaattcatttcggtttctcttctttctcacatccagataatgcatta
gaagcatgtggattacttactcctttacatatagtacctgaatggtatttcttatgccaa
tatgctatgttaaaagctgtgccaaataaaaatgcaggattcattatattatttacttct
atcttcatcttattttactttatgagaagtctttcagtatctttctattttatcgtgtgg
gtaagctctagatttaatagtttctttgtaattctttggtttttagttttcatatcctta
atttggataggtggtcaatttcctgtagacaactttctatcttatggtcgtatcttgaca
ttacattattattatcttcttatctgtatcttattatcttag
>Irm5_cob
atgaaatctcatttacaatcatatccttgtcctctgatcataaattatttttggaatctt
ggtttttattagggattactattttattacaaattatatctggaatcttcttaggttta
cattatacatcagatattaattcagcatattttagtattttctttattattagagaaata
tattatggatggtgtttacgttatcttcattctaatggttcatcatttgtctttcttttg
atatttctacatcttggaagagctatatcttatggttcatatttttataatccaaatact
tggttttctggaattattattatcttcttcttaatgggaacagcatttatgggttatgtg
ttacctttaggacaaatgagtttatgggggggttacagtaattacaaatttattatctgca
tttccatctttaatagaatggctttgtggaggacattacatttacaatcctacatttaag
aggttctttgtctttcattttctatttccatttcttctttgtggttttcttgtttatcat
atttttaatctacattttctatcttctaataatcctttaaggaattccactaataataaa
atagcatttttccctttcattattagtaaagatttatatggaaagatattaattctctat
ctatatcttcttcaaattcatttcggtttctcttctttctcacatccagataatgcatta
gaagcatgtggattacttactcctttacatatagtacctgaatggtatttcttatgccaa
tatgctatgttaaaagctgtgccaaataaaaatgcaggattcattatattatttacttct
atcttcatcttattttactttatgagaagtctttcaatctcattctattttatcgtgtgg
gtaagctctagatttaatagtttctttgtaattctttggtttttagttttcatatcctta
atttggataggtggtcaatttcctgtagacaactttctatcttatggtcgtatcttgaca
ttacattattattatcttcttatctgtatcttattatcttag
>Irm7_cob
atgaaatctcatttacaatcatatccttgtcctctgatcataaattatttttggaatctt
ggtttttattagggattactattttattacaaattatatctggaatcttcttaggttta
cattatacatcagatattaattcagcatattttagtattttctttattattagagaaata
tattatggatggtgtttacgttatcttcattctaatggttcatcatttgtctttcttttg
atatttctacatcttggaagagctatatcttatggttcatatttttataatccaaatact
tggttttctggaattattattatcttcttcttaatgggaacagcatttatgggttatgtg
ttacctttaggacaaatgagtttatggggtgttacagtaattacaaatttattatctgca
tttccatctttaatagaatggctttgtggaggacattacatttacaatcctacatttaag
aggttctttgtctttcattttctatttccatttcttctttgtggttttcttgtttatcat
atttttaatctacattttctatcttctaataatcctttaaggaattccactaataataaa
atagcatttttccctttcattattagtaaagatttatatggaaagatattaattctctat
ctatatcttcttcaaattcatttcggtttctcttctttctcacatccagataatgcatta
gaagcatgtggattacttactcctttacatatagtacctgaatggtatttcttatgccaa
tatgctatgttaaaagctgtgccaaataaaaatgcaggattcattgtcttactaacttct
atctttgtattattttattttatgagaagtctttcagtatctttctattttatcgtgtgg
gtaagctctagatttaatagtttctttgtaattctttggtttttagttttcatatcctta
atttggataggtggtcaatttcctgtagacaactttctatcttatggtcgtatcttgaca
ttacattattattatcttcttatctgtatcttattatcttag

>Irm9_cob
atgaaatctcatttacaatcatatccttgtcctcttatcataaattatttatggaatctt
ggtttttattaggggttgctattttattacaaattatatctggtatcttcttaggttta
cattatacatcagaaattaattcagcatattttagtattttctttattattagagaaata
tattatggatggtctttacgttattttcattctaatggttcatcatttgtctttcttttt
atatttctacatcttggaagagctatatcttatggttcatatttttataatccaaatact
tggttttctggaattattattatcttcttattaatggcaacagcatttatgggttatgtc
ttacctttaggacaaatgagtttatggggtgttacagtaattacaaatttattatctcca
tttccatcttttatagaatggctttgtggaggagattatgtttacaatcttacattgaag
aggttctttgtctttcattttctatttccatttcttctttgtggttttcttctttatcat
atttttaatctacattttctatcttctaataatccattaagtaattccactaataataaa
atagtatttttccctttcattattagtaaagatttctatggaaagatattaattctctat
ctatatcttcttcaaattcatttcggtttctcttctttctcacatccagataatgcatta
gaagcatgtggattacttactcctttacatatagtacctgaatggtatttcttatgccaa
tatgctatgttaaaagctgtgccaaataaaaatgcaggattcattgtcttactaacttct
atctttgtattattttattttatgagaagtctttcagtatctttctattttatcgtgtgg
gtaagctctagatttaatagtttctttgtaattctttggtttttagttttcatatcctta
atttggataggtggtcaatttcctgtcgacaactttctatcttatgctcgtatcttgaca
ttatattattattattttcttatctgtatcttattatcttag
>Isy12_cob
atgaaatctcatttacaatcatatccttgtcctctgatcataaattatttttggaatctt
ggtttttattagggattactattttattacaaattatatctggaatcttcttaggttta
cattatacatcagatattaattcagcatattttagtattttctttattattagagaaata
tattatggatggtgtttacgttatcttcattctaatggttcatcatttgtttttcttttt
atatttctacatcttggaagagctatatcttatggttcatatttttataatccaaatact
tggttttctggaattattattatcttcttcttaatgggaacagcatttatgggttatgtg
ttacctttaggacaaatgagtttatggggagttacagtaattacaaatttattatctgca
tttccatctttaatagaatggctttgtggaggacattacatttacaatcctacatttaag
aggttctttgtctttcattttctatttccatttcttctttgtggttttcttctttatcat
atttttaatctacattttctatcttctaataatcctttaaggaattccactaataataaa
atagcatttttccctttcattattagtaaagatttatatggaaagatattaattctctat
ctatatcttcttcaaattcatttcggtttctcttctttctcacatccagataatgcatta
gaagcatgtggattacttactcctttacatatagtacctgaatggtatttcttatgccaa
tatgctatgttaaaagctgtgccaaataaaaatgcaggattcattatattatttacttct
atcttcatcttattttactttatgagaagtctttcaatctcattctattttatcgtgtgg
gtaagctctagatttaatagtttctttgtaattctttggtttttagttttcatatcctta
atttggataggtggtcaatttcctgtagacaactttctatcttatggtcgtatcttgaca
ttacattattattattttcttatctgtatcttattatcttag
>Isy15_cob
atgaaatctcatttacaatcatatccttgtcctcttatcataaattatttatggaatctt
ggtttttattagggattactattttattacaaattatatctggtatcttcttaggttta
cattatacatcagaaattaattcagcatattttagtattttctttattattagagaaata
tattatggatggtctttacgttatcttcattctaatggttcatcatttgtctttcttttt
atatttctacatcttggaagagctatatcttatggttcatatttttataatccaaatact
tggttttctggaattattattatcttcttattaatggcaacagcatttatgggttatgtc
ttacctttaggacaaatgagtttatggggtgttacagtaattacaaatttattatctcca
tttccatcttttatagaatggctttgtggaggagattatgtttacaatcttacattgaag
aggttctttgtctttcattttctatttccatttattctttgtggttttcttgtttatcat
atttttaatctacattttctatcttctaataatccattaagtaattccactaataataaa
atagtatttttccctttcattattagtaaagatttctatggaaagatattaattctctat
ctatatcttcttcaaattcatttcggtttctcttctttctcacatccagataatgcatta
gaagcatgtggattacttactcctttacatatagtacctgaatggtatttcttatgccaa
tatgctatgttaaaagctgtgccaaataaaaatgcaggattcattgtcttactaacttct
atctttgtattattttattttatgagaagtctttcagtatctttctattttatcgtgtgg
gtaagctctagatttaatagtttctttgtaattctttggtttttagttttcatatcctta
atttggataggtggtcaatttcctgtagacaactttctatcttatggtcgtatcttgaca
ttacattattattatcttcttatctgtatcttattatcttag

>Isy16_cob
atgaaatctcatttacaatcatatccttgtcctctgatcataaattatttttggaatctt
ggtttttattagggattactattttattacaaattatatctggaatcttcttaggttta
cattatacatcagatattaattcagcatattttagtattttctttattattagagaaata
tattatggatggtgtttacgttatcttcattctaatggttcatcatttgtctttcttttg
atatttctacatcttggaagagctatatcttatggttcatatttttataatccaaatact
tggttttctggaattattattatcttcttcttaatgggaacagcatttatgggttatgtg
ttacctttaggacaaatgagtttatggggggttacagtaattacaaatttattatctgca
tttccatctttaatagaatggctttgtggaggacattacatttacaatcctacatttaag
aggttctttgtctttcattttctatttccatttcttctttgtggttttcttgtttatcat
atttttaatctacattttctatcttctaataatcctttaaggaattccactaataataaa
atagcatttttccctttcattattagtaaagatttatatggaaagatattaattctctat
ctatatcttcttcaaattcatttcggtttctcttctttctcacatccagataatgcatta
gaagcatgtggattacttactcctttacatatagtacctgaatggtatttcttatgccaa
tatgctatgttaaaagctgtgccaaataaaaatgcaggattcattatattatttacttct
atcttcatcttattttactttatgagaagtctttcaatctcattctattttatcgtgtgg
gtaagctctagatttaatagtttctttgtaattctttggtttttagttttcatatccttt
atttggataggtggtcaatttcctgtagacaactttctatcttatggtcgtatcttgaca
ttacattattattatcttcttatctgtatcttattatcttag
>Isy17_cob
atgaaatctcatttacaatcatatccttgtcctctgatcataaattatttttggaatctt
ggtttttattagggattactattttattacaaattatatctggaatcttcttaggttta
cattatacatcagatattaattcagcatattttagtattttctttattattagagaaata
tattatggatggtgtttacgttatcttcattctaatggttcatcatttgtttttcttttg
atatttctacatcttggaagagctatatcttatggttcatatttttataatccaaatact
tggttttctggaattattattatcttcttcttaatgggaacagcatttatgggttatgtg
ttacctttaggacaaatgagtttatggggagttacagtaattacaaatttattatctgca
tttccatctttaatagaatggctttgtggaggacattacatttacaatcctacatttaag
aggttctttgtctttcattttctatttccatttcttctttgtggttttcttgtttatcat
atttttaatctacattttctatcttctaataatcctttaaggaattccactaataataaa
atagcatttttccctttcattattagtaaagatttatatggaaagatattaattctctat
ctatatcttcttcaaattcatttcggtttctcttctttctcacatccagataatgcatta
gaagcatgtggattacttactcctttacatatagtacctgaatggtatttcttatgccaa
tatgctatgttaaaagctgtgccaaataaaaatgcaggattcattatattatttacttct
atcttcatcttattttactttatgagaagtctttcaatctcattctattttatcgtgtgg
gtaagctctagatttaatagtttctttgtaattctttggtttttagttttcatatcctta
atttggataggtggtcaatttcctgtagacaactttctatcttatggtcgtatcttgaca
ttacattattattatcttcttatctgtatcttattatcttag
>Isy18_cob
atgaaatctcatttacaatcatatccttgtcctctgatcataaattatttttggaatctt
ggtttttattagggattactattttattacaaattatatctggaatcttcttaggttta
cattatacatcagatattaattcagcatattttagtattttctttattattagagaaata
tattatggatggtgtttacgttatcttcattctaatggttcatcatttgtctttcttttt
atatttctacatcttggaagagctatatcttatggttcatatttttataatccaaatact
tggttttctggaattattattatcttcttcttaatgggaacagcatttatgggttatgtg
ttacctttaggacaaatgagtttatggggggttacagtaattacaaatttattatctgca
tttccatctttaatagaatggctttgtggaggacattacatttacaatcctacatttaag
aggttctttgtctttcattttctatttccatttcttctttgtggttttcttgtttatcat
atttttaatctacattttctatcttctaataatcctttaaggaattccactaataataaa
atagcatttttccctttcattattagtaaagatttatatggaaagatattaattctctat
ctatatcttcttcaaattcatttcggtttctcttctttctcacatccagataatgcatta
gaagcatgtggattacttactcctttacatatagtacctgaatggtatttcttatgccaa
tatgctatgttaaaagctgtgccaaataaaaatgcaggattcattatattatttacttct
atcttcatcttattttactttatgagaagtctttcagtatctttctattttatcgtgtgg
gtaagctctagatttaatagtttctttgtaattctttggtttttagttttcatatcctta
atttggataggtggtcaatttcctgtagacaactttctatcttatggtcgtatcttgaca
ttacattattattatcttcttatctgtatcttattatcttag

>Isy21_cob
atgaaatctcatttacaatcatatccttgtcctctgatcataaattatttttggaatctt
ggtttttattagggattactattttattacaaattatatctggaatcttcttaggttta
cattatacatcagatattaattcagcatattttagtattttctttattattagagaaata
tattatggatggtgtttacgttatcttcattctaatggttcatcatttgtttttcttttt
atatttctacatcttggaagagctatatcttatggttcatatttttataatccaaatact
tggttttctggaattattattatcttcttcttaatgggaacagcatttatgggttatgtg
ttacctttaggacaaatgagtttatggggagttacagtaattacaaatttattatctgca
tttccatctttaatagaatggctttgtggaggacattacatttacaatcctacatttaag
aggttctttgtctttcattttctatttccatttcttctttgtggttttcttgtttatcat
atttttaatctacattttctatcttctaataatcctttaaggaattccactaataataaa
atagcatttttccctttcattattagtaaagatttatatggaaagatattaattctctat
ctatatcttcttcaaattcatttcggtttctcttctttctcacatccagataatgcatta
gaagcatgtggattacttactcctttacatatagtacctgaatggtatttcttatgccaa
tatgctatgttaaaagctgtgccaaataaaaatgcaggattcattatattatttacttct
atcttcatcttattttactttatgagaagtctttcaatctcattctattttatcgtgtgg
gtaagctctagatttaatagtttctttgtaattctttggtttttagttttcatatcctta
atttggataggtggtcaatttcctgtagacaactttctatcttatggtcgtatcttgaca
ttacattattattatcttcttatctgtatcttattatcttag
>Isy22_cob
atgaaatctcatttacaatcatatccttgtcctctgatcataaattatttttggaatctt
ggtttttattagggattactattttattacaaattatatctggaatcttcttaggttta
cattatacatcagatattaattcagcatattttagtattttctttattattagagaaata
tattatggatggtgtttacgttatcttcattctaatggttcatcatttgtctttcttttg
atatttctacatcttggaagagctatatcttatggttcatatttttataatccaaatact
tggttttctggaattattattatcttcttcttaatgggaacagcatttatgggttatgtg
ttacctttaggacaaatgagtttatggggggttacagtaattacaaatttattatctgca
tttccatctttaatagaatggctttgtggaggacattacatttacaatcctacatttaag
aggttctttgtctttcattttctatttccatttcttctttgtggttttcttgtttatcat
atttttaatctacattttctatcttctaataatcctttaaggaattccactaataataaa
atagcatttttccctttcattattagtaaagatttatatggaaagatattaattctctat
ctatatcttcttcaaattcatttcggtttctcttctttctcacatccagataatgcatta
gaagcatgtggattacttactcctttacatatagtacctgaatggtatttcttatgccaa
tatgctatgttaaaagctgtgccaaataaaaatgcaggattcattatattatttacttct
atcttcatcttattttactttatgagaagtctttcaatctcattctattttatcgtgtgg
gtaagctctagatttaatagtttctttgtaattctttggtttttagttttcatatcctta
atttggataggtggtcaatttcctgtagacaactttctatcttatggtcgtatcttgaca
ttacattattattatcttcttatctgtatcttattatcttag
>Isy23_cob
atgaaatctcatttacaatcatatccttgtcctctgatcataaattatttttggaatctt
ggtttttattagggattactattttattacaaattatatctggaatcttcttaggttta
cattatacatcagatattaattcagcatattttagtattttctttattattagagaaata
tattatggatggtgtttacgttatcttcattctaatggttcatcatttgtttttcttttg
atatttctacatcttggaagagctatatcttatggttcatatttttataatccaaatact
tggttttctggaattattattatcttcttcttaatgggaacagcatttatgggttatgtg
ttacctttaggacaaatgagtttatggggagttacagtaattacaaatttattatctgca
tttccatctttaatagaatggctttgtggaggacattacatttacaatcctacatttaag
aggttctttgtctttcattttctatttccatttcttctttgtggttttcttgtttatcat
atttttaatctacattttctatcttctaataatcctttaaggaattccactaataataaa
atagcatttttccctttcattattagtaaagatttatatggaaagatattaattctctat
ctatatcttcttcaaattcatttcggtttctcttctttctcacatccagataatgcatta
gaagcatgtggattacttactcctttacatatagtacctgaatggtatttcttatgccaa
tatgctatgttaaaagctgtgccaaataaaaatgcaggattcattatattatttacttct
atcttcatcttattttactttatgagaagtctttcagtatctttctattttatcgtgtgg
gtaagctctagatttaatagtttctttgtaattctttggtttttagttttcatatcctta
atttggataggtggtcaatttcctgtagacaactttctatcttatggtcgtatcttgaca
ttacattattattatcttcttatctgtatcttattatcttag

>Isy24_cob
atgaaatctcatttacaatcatatccttgtcctctgatcataaattatttttggaatctt
ggtttttattagggattactattttattacaaattatatctggaatcttcttaggttta
cattatacatcagatattaattcagcatattttagtattttctttattattagagaaata
tattatggatggtgtttacgttatcttcattctaatggttcatcatttgtctttctttg
atatttctacatcttggaagagctatatcttatggttcatatttttataatccaaatact
tggttttctggaattattattatcttcttcttaatgggaacagcatttatgggttatgtg
ttacctttaggacaaatgagtttatggggggttacagtaattacaaatttattatctgca
tttccatctttaatagaatggctttgtggaggacattacatttacaatcctacatttaag
aggttctttgtctttcattttctatttccatttcttctttgtggttttcttgtttatcat
atttttaatctacattttctatcttctaataatcctttaaggaattccactaataataaa
atagcatttttccctttcattattagtaaagatttatatggaaagatattaattctctat
ctatatcttcttcaaattcatttcggtttctcttctttctcacatccagataatgcatta
gaagcatgtggattacttactcctttacatatagtacctgaatggtatttcttatgccaa
tatgctatgttaaaagctgtgccaaataaaaatgcaggattcattatattatttacttct
atcttcatcttattttactttatgagaagtctttcaatctcattctattttatcgtgtgg
gtaagctctagatttaatagtttctttgtaattctttggtttttagttttcatatcctta
atttggataggtggtcaatttcctgtagacaactttctatcttatggtcgtatcttgaca
ttacattattattatcttcttatctgtatcttattatcttag
>Isy25_cob
atgaaatctcatttacaatcatatccttgtcctctgatcataaattatttttggaatctt
ggtttttattagggattactattttattacaaattatatctggaatcttcttaggttta
cattatacatcagatattaattcagcatattttagtattttctttattattagagaaata
tattatggatggtgtttacgttatcttcattctaatggttcatcatttgtttttcttttt
atatttctacatcttggaagagctatatcttatggttcatatttttataatccaaatact
tggttttctggaattattattatcttcttcttaatgggaacagcatttatgggttatgtg
ttacctttaggacaaatgagtttatggggagttacagtaattacaaatttattatctgca
tttccatctttaatagaatggctttgtggaggacattacatttacaatcctacatttaag
aggttctttgtctttcattttctatttccatttcttctttgtggttttcttctttatcat
atttttaatctacattttctatcttctaataatcctttaaggaattccactaataataaa
atagcatttttccctttcattattagtaaagatttatatggaaagatattaattctctat
ctatatcttcttcaaattcatttcggtttctcttctttctcacatccagataatgcatta
gaagcatgtggattacttactcctttacatatagtacctgaatggtatttcttatgccaa
tatgctatgttaaaagctgtgccaaataaaaatgcaggattcattatattatttacttct
atcttcatcttattttactttatgagaagtctttcaatctcattctattttatcgtgtgg
gtaagctctagatttaatagtttctttgtaattctttggtttttagttttcatatcctta
atttggataggtggtcaatttcctgtagacaactttctatcttatggtcgtatcttgaca
ttacattattattatcttcttatctgtatcttattatcttag
>Isy26_cob
atgaaatctcatttacaatcatatccttgtcctctgatcataaattatttttggaatctt
ggtttttattagggattactattttattacaaattatatctggaatcttcttaggttta
cattatacatcagatattaattcagcatattttagtattttctttattattagagaaata
tattatggatggtgtttacgttatcttcattctaatggttcatcatttgtttttcttttt
atatttctacatcttggaagagctatatcttatggttcatatttttataatccaaatact
tggttttctggaattattattatcttcttcttaatgggaacagcatttatgggttatgtg
ttacctttaggacaaatgagtttatggggagttacagtaattacaaatttattatctgca
tttccatctttaatagaatggctttgtggaggacattacatttacaatcctacatttaag
aggttctttgtctttcattttctatttccatttcttctttgtggttttcttctttatcat
atttttaatctacattttctatcttctaataatcctttaaggaattccactaataataaa
atagcatttttccctttcattattagtaaagatttatatggaaagatattaattctctat
ctatatcttcttcaaattcatttcggtttctcttctttctcacatccagataatgcatta
gaagcatgtggattacttactcctttacatatagtacctgaatggtatttcttatgccaa
tatgctatgttaaaagctgtgccaaataaaaatgcaggattcattatattatttacttct
atcttcatcttattttactttatgagaagtctttcagtatcattctattttatcgtgtgg
gtaagctctagatttaatagtttctttgtaattctttggtttttagttttcatatcctta
atttggataggtggtcaatttcctgtagacaactttctatcttatggtcgtatcttgaca
ttacattattattatcttcttatctgtatcttattatcttag

>Isy27_cob
atgaaatctcatttacaatcatatccttgtcctctgatcataaattatttttggaatctt
ggttttttattagggattactattttattacaaattatatctggaatcttcttaggttta
cattatacatcagatattaattcagcatattttagtattttctttattattagagaaata
tattatggatggtgtttacgttatcttcattctaatggttcatcatttgtttttctttg
atatttctacatcttggaagagctatatcttatggttcatatttttataatccaaatact
tggttttctggaattattattatcttcttcttaatgggaacagcatttatgggttatgtg
ttacctttaggacaaatgagtttatggggagttacagtaattacaaatttattatctgca
tttccatctttaatagaatggctttgtggaggacattacatttacaatcctacatttaag
aggttctttgtctttcattttctatttccatttcttctttgtggttttcttgtttatcat
atttttaatctacattttctatcttctaataatcctttaaggaattccactaataataaa
atagcatttttccctttcattattagtaaagatttatatggaaagatattaattctctat
ctatatcttcttcaaattcatttcggtttctcttctttctcacatccagataatgcatta
gaagcatgtggattacttactcctttacatatagtacctgaatggtatttcttatgccaa
tatgctatgttaaaagctgtgccaaataaaaatgcaggattcattatattatttacttct
atcttcatcttattttactttatgagaagtctttcagtatctttctattttatcgtgtgg
gtaagctctagatttaatagtttctttgtaattctttggtttttagttttcatatcctta
atttggataggtggtcaatttcctgtagacaactttctatcttatggtcgtatcttgaca
ttacattattattatcttcttatctgtatcttattatcttag
>Isy4_cob
atgaaatctcatttacaatcatatccttgtcctctgatcataaattatttttggaatctt
ggttttttattagggattactattttattacaaattatatctggaatcttcttaggttta
cattatacatcagatattaattcagcatattttagtattttctttattattagagaaata
tattatggatggtgtttacgttatcttcattctaatggttcatcatttgtctttctttg
atatttctacatcttggaagagctatatcttatggttcatatttttataatccaaatact
tggttttctggaattattattatcttcttcttaatgggaacagcatttatgggttatgtg
ttacctttaggacaaatgagtttatggggggttacagtaattacaaatttattatctgca
tttccatctttaatagaatggctttgtggaggacattacatttacaatcctacatttaag
aggttctttgtctttcattttctatttccatttcttctttgtggttttcttgtttatcat
atttttaatctacattttctatcttctaataatcctttaaggaattccactaataataaa
atagcatttttccctttcattattagtaaagatttatatggaaagatattaattctctat
ctatatcttcttcaaattcatttcggtttctcttctttctcacatccagataatgcatta
gaagcatgtggattacttactcctttacatatagtacctgaatggtatttcttatgccaa
tatgctatgttaaaagctgtgccaaataaaaatgcaggattcattatattatttacttct
atcttcatcttattttactttatgagaagtctttcagtatctttctattttatcgtgtgg
gtaagctctagatttaatagtttctttgtaattctttggtttttagttttcatatcctta
atttggataggtggtcaatttcctgtagacaactttctatcttatggtcgtatcttgaca
ttacattattattatcttcttatctgtatcttattatcttag
>Isy7_cob
atgaaatctcatttacaatcatatccttgtcctcttatcataaattatttatggaatctt
ggttttttattagggattactattttattacaaattatatctggaatcttcttaggttta
cattatacatcagatattaattcagcatattttagtattttctttattattagagaaata
tattatggatggtgtttacgttatcttcattctaatggttcatcatttgtctttctttg
atatttctacatcttggaagagctatatcttatggttcatatttttataatccaaatact
tggttttctggaattattattatcttcttcttaatgggaacagcatttatgggttatgtg
ttacctttaggacaaatgagtttatggggggttacagtaattacaaatttattatctgca
tttccatctttaatagaatggctttgtggaggacattacatttacaatcctacatttaag
aggttctttgtctttcattttctatttccatttcttctttgtggttttcttgtttatcat
atttttaatctacattttctatcttctaataatcctttaaggaattccactaataataaa
atagcatttttccctttcattattagtaaagatttatatggaaagatattaattctctat
ctatatcttcttcaaattcatttcggtttctcttctttctcacatccagataatgcatta
gaagcatgtggattacttactcctttacatatagtacctgaatggtatttcttatgccaa
tatgctatgttaaaagctgtgccaaataaaaatgcaggattcattatattatttacttct
atcttcatcttattttactttatgagaagtctttcaatctcattctattttatcgtgtgg
gtaagctctagatttaatagtttctttgtaattctttggtttttagttttcatatcctta
atttggataggtggtcaatttcctgtagacaactttctatcttatggtcgtatcttgaca
ttacattattattatcttcttatctgtatcttattatcttag

>Isy8_cob
atgaaatctcatttacaatcatatccttgtcctctgatcataaattatttttggaatctt
ggttttttattagggattactattttattacaaattatatctggaatcttcttaggttta
cattatacatcagatattaattcagcatattttagtattttctttattattagagaaata
tattatggatggtgtttacgttatcttcattctaatggttcatcatttgtttttcttttt
atatttctacatcttggaagagctatatcttatggttcatatttttataatccaaatact
tggttttctggaattattattatcttcttcttaatgggaacagcatttatgggttatgtg
ttacctttaggacaaatgagtttatggggagttacagtaattacaaatttattatctgca
tttccatctttaatagaatggctttgtggaggacattacatttacaatcctacatttaag
aggttctttgtctttcattttctatttccatttcttctttgtggttttcttctttatcat
atttttaatctacattttctatcttctaataatcctttaaggaattccactaataataaa
atagcatttttccctttcattattagtaaagatttatatggaaagatattaattctctat
ctatatcttcttcaaattcatttcggtttctcttctttctcacatccagataatgcatta
gaagcatgtggattacttactcctttacatatagtacctgaatggtatttcttatgccaa
tatgctatgttaaaagctgtgccaaataaaaatgcaggattcattatattatttacttct
atcttcatcttattttactttatgagaagtctttcaatctcattctattttatcgtgtgg
gtaagctctagatttaatagtttctttgtaattctttggtttttagttttcatatcctta
atttggataggtggtcaatttcctgtagacaactttctatcttatggtcgtatcttgaca
ttacattattattatcttcttatctgtatcttattatcttag
>KrA1_cob
atgaaatctcatttacaatcatatccttgtcctctgatcataaattatttttggaatctt
ggttttttattagggattactattttattacaaattatatctggaatcttcttaggttta
cattatacatcagatattaattcagcatattttagtattttctttattattagagaaata
tattatggatggtgtttacgttatcttcattctaatggttcatcatttgtctttcttttg
atatttctacatcttggaagagctatatcttatggttcatatttttataatccaaatact
tggttttctggaattattattatcttcttcttaatgggaacagcatttatgggttatgtg
ttacctttaggacaaatgagtttatgggggggttacagtaattacaaatttattatctgca
tttccatctttaatagaatggctttgtggaggacattacatttacaatcctacatttaag
aggttctttgtctttcattttctatttccatttcttctttgtggttttcttgtttatcat
atttttaatctacattttctatcttctaataatcctttaaggaattccactaataataaa
atagcatttttccctttcattattagtaaagatttatatggaaagatattaattctctat
ctatatcttcttcaaattcatttcggtttctcttctttctcacatccagataatgcatta
gaagcatgtggattacttactcctttacatatagtacctgaatggtatttcttatgccaa
tatgctatgttaaaagctgtgccaaataaaaatgcaggattcattatattatttacttct
atcttcatcttattttactttatgagaagtctttcaatctcattctattttatcgtgtgg
gtaagctctagatttaatagtttctttgtaattctttggtttttagttttcatatcctta
atttggataggtggtcaatttcctgtagacaactttctatcttatggtcgtatcttgaca
ttacattattattatcttcttatctgtatcttattatcttag
>KrA10_cob
atgaaatctcatttacaatcatatccttgtcctctgatcataaattatttttggaatctt
ggttttttattagggattactattttattacaaattatatctggaatcttcttaggttta
cattatacatcagatattaattcagcatattttagtattttctttattattagagaaata
tattatggatggtgtttacgttatcttcattctaatggttcatcatttgtctttcttttg
atatttctacatcttggaagagctatatcttatggttcatatttttataatccaaatact
tggttttctggaattattattatcttcttcttaatgggaacagcatttatgggttatgtg
ttacctttaggacaaatgagtttatgggggggttacagtaattacaaatttattatctgca
tttccatctttaatagaatggctttgtggaggacattacatttacaatcctacatttaag
aggttctttgtctttcattttctatttccatttcttctttgtggttttcttgtttatcat
atttttaatctacattttctatcttctaataatcctttaaggaattccactaataataaa
atagcatttttccctttcattattagtaaagatttatatggaaagatattaattctctat
ctatatcttcttcaaattcatttcggtttctcttctttctcacatccagataatgcatta
gaagcatgtggattacttactcctttacatatagtacctgaatggtatttcttatgccaa
tatgctatgttaaaagctgtgccaaataaaaatgcaggattcattatattatttacttct
atcttcatcttattttactttatgagaagtctttcaatctcattctattttatcgtgtgg
gtaagctctagatttaatagtttctttgtaattctttggtttttagttttcatatcctta
atttggataggtggtcaatttcctgtagacaactttctatcttatggtcgtatcttgaca
ttacattattattatcttcttatctgtatcttattatcttag

>KrA11_cob
atgaaatctcatttacaatcatatccttgtcctctgatcataaattatttttggaatctt
ggtttttattagggattactattttattacaaattatatctggaatcttcttaggttta
cattatacatcagatattaattcagcatattttagtattttctttattattagagaaata
tattatggatggtgtttacgttatcttcattctaatggttcatcatttgtctttcttttg
atatttctacatcttggaagagctatatcttatggttcatatttttataatccaaatact
tggttttctggaattattattatcttcttcttaatgggaacagcatttatgggttatgtg
ttacctttaggacaaatgagtttatggggggttacagtaattacaaatttattatctgca
tttccatctttaatagaatggctttgtggaggacattacatttacaatcctacatttaag
aggttctttgtctttcattttctatttccatttcttctttgtggttttcttgtttatcat
atttttaatctacattttctatcttctaataatcctttaaggaattccactaataataaa
atagcatttttccctttcattattagtaaagatttatatggaaagatattaattctctat
ctatatcttcttcaaattcatttcggtttctcttctttctcacatccagataatgcatta
gaagcatgtggattacttactcctttacatatagtacctgaatggtatttcttatgccaa
tatgctatgttaaaagctgtgccaaataaaaatgcaggattcattatattatttacttct
atcttcatcttattttactttatgagaagtctttcaatctcattctattttatcgtgtgg
gtaagctctagatttaatagtttctttgtaattctttggtttttagttttcatatcctta
atttggataggtggtcaatttcctgtagacaactttctatcttatggtcgtatcttgaca
ttacattattattatcttcttatctgtatcttattatcttag
>KrA12_cob
atgaaatctcatttacaatcatatccttgtcctctgatcataaattatttttggaatctt
ggtttttattagggattactattttattacaaattatatctggaatcttcttaggttta
cattatacatcagatattaattcagcatattttagtattttctttattattagagaaata
tattatggatggtgtttacgttatcttcattctaatggttcatcatttgtctttcttttg
atatttctacatcttggaagagctatatcttatggttcatatttttataatccaaatact
tggttttctggaattattattatcttcttcttaatgggaacagcatttatgggttatgtg
ttacctttaggacaaatgagtttatggggggttacagtaattacaaatttattatctgca
tttccatctttaatagaatggctttgtggaggacattacatttacaatcctacatttaag
aggttctttgtctttcattttctatttccatttcttctttgtggttttcttgtttatcat
atttttaatctacattttctatcttctaataatcctttaaggaattccactaataataaa
atagcatttttccctttcattattagtaaagatttatatggaaagatattaattctctat
ctatatcttcttcaaattcatttcggtttctcttctttctcacatccagataatgcatta
gaagcatgtggattacttactcctttacatatagtacctgaatggtatttcttatgccaa
tatgctatgttaaaagctgtgccaaataaaaatgcaggattcattatattatttacttct
atcttcatcttattttactttatgagaagtctttcaatctcattctattttatcgtgtgg
gtaagctctagatttaatagtttctttgtaattctttggtttttagttttcatatcctta
atttggataggtggtcaatttcctgtagacaactttctatcttatggtcgtatcttgaca
ttacattattattatcttcttatctgtatcttattatcttag
>KrA13_cob
atgaaatctcatttacaatcatatccttgtcctctgatcataaattatttttggaatctt
ggtttttattagggattactattttattacaaattatatctggaatcttcttaggttta
cattatacatcagatattaattcagcatattttagtattttctttattattagagaaata
tattatggatggtgtttacgttatcttcattctaatggttcatcatttgtctttcttttg
atatttctacatcttggaagagctatatcttatggttcatatttttataatccaaatact
tggttttctggaattattattatcttcttcttaatgggaacagcatttatgggttatgtg
ttacctttaggacaaatgagtttatggggggttacagtaattacaaatttattatctgca
tttccatctttaatagaatggctttgtggaggacattacatttacaatcctacatttaag
aggttctttgtctttcattttctatttccatttcttctttgtggttttcttgtttatcat
atttttaatctacattttctatcttctaataatcctttaaggaattccactaataataaa
atagcatttttccctttcattattagtaaagatttatatggaaagatattaattctctat
ctatatcttcttcaaattcatttcggtttctcttctttctcacatccagataatgcatta
gaagcatgtggattacttactcctttacatatagtacctgaatggtatttcttatgccaa
tatgctatgttaaaagctgtgccaaataaaaatgcaggattcattatattatttacttct
atcttcatcttattttactttatgagaagtctttcagtatctttctattttatcgtgtgg
gtaagctctagatttaatagtttctttgtaattctttggtttttagttttcatatcctta
atttggataggtggtcaatttcctgtagacaactttctatcttatggtcgtatcttgaca
ttacattattattatcttcttatctgtatcttattatcttag

>KrA14_cob
atgaaatctcatttacaatcatatccttgtcctctgatcataaattatttttggaatctt
ggtttttattagggattactattttattacaaattatatctggaatcttcttaggttta
cattatacatcagatattaattcagcatattttagtattttctttattattagagaaata
tattatggatggtgtttacgttatcttcattctaatggttcatcatttgtctttcttttg
atatttctacatcttggaagagctatatcttatggttcatatttttataatccaaatact
tggttttctggaattattattatcttcttcttaatgggaacagcatttatgggttatgtg
ttacctttaggacaaatgagtttatggggggttacagtaattacaaatttattatctgca
tttccatctttaatagaatggctttgtggaggacattacatttacaatcctacatttaag
aggttctttgtctttcattttctatttccatttcttctttgtggttttcttgtttatcat
atttttaatctacattttctatcttctaataatcctttaaggaattccactaataataaa
atagcatttttccctttcattattagtaaagatttatatggaaagatattaattctctat
ctatatcttcttcaaattcatttcggtttctcttctttctcacatccagataatgcatta
gaagcatgtggattacttactcctttacatatagtacctgaatggtatttcttatgccaa
tatgctatgttaaaagctgtgccaaataaaaatgcaggattcattatattatttacttct
atcttcatcttattttactttatgagaagtctttcaatctcattctattttatcgtgtgg
gtaagctctagatttaatagtttctttgtaattctttggtttttagttttcatatcctta
atttggataggtggtcaatttcctgtagacaactttctatcttatggtcgtatcttgaca
ttacattattattatcttcttatctgtatcttattatcttag
>KrA15_cob
atgaaatctcatttacaatcatatccttgtcctctgatcataaattatttttggaatctt
ggtttttattagggattactattttattacaaattatatctggaatcttcttaggttta
cattatacatcagatattaattcagcatattttagtattttctttattattagagaaata
tattatggatggtgtttacgttatcttcattctaatggttcatcatttgtctttcttttg
atatttctacatcttggaagagctatatcttatggttcatatttttataatccaaatact
tggttttctggaattattattatcttcttcttaatgggaacagcatttatgggttatgtg
ttacctttaggacaaatgagtttatggggggttacagtaattacaaatttattatctgca
tttccatctttaatagaatggctttgtggaggacattacatttacaatcctacatttaag
aggttctttgtctttcattttctatttccatttcttctttgtggttttcttgtttatcat
atttttaatctacattttctatcttctaataatcctttaaggaattccactaataataaa
atagcatttttccctttcattattagtaaagatttatatggaaagatattaattctctat
ctatatcttcttcaaattcatttcggtttctcttctttctcacatccagataatgcatta
gaagcatgtggattacttactcctttacatatagtacctgaatggtatttcttatgccaa
tatgctatgttaaaagctgtgccaaataaaaatgcaggattcattatattatttacttct
atcttcatcttattttactttatgagaagtctttcaatctcattctattttatcgtgtgg
gtaagctctagatttaatagtttctttgtaattctttggtttttagttttcatatcctta
atttggataggtggtcaatttcctgtagacaactttctatcttatggtcgtatcttgaca
ttacattattattatcttcttatctgtatcttattatcttag
>KrA2_cob
atgaaatctcatttacaatcatatccttgtcctctgatcataaattatttttggaatctt
ggtttttattagggattactattttattacaaattatatctggaatcttcttaggttta
cattatacatcagatattaattcagcatattttagtattttctttattattagagaaata
tattatggatggtgtttacgttatcttcattctaatggttcatcatttgtctttcttttg
atatttctacatcttggaagagctatatcttatggttcatatttttataatccaaatact
tggttttctggaattattattatcttcttcttaatgggaacagcatttatgggttatgtg
ttacctttaggacaaatgagtttatggggggttacagtaattacaaatttattatctgca
tttccatctttaatagaatggctttgtggaggacattacatttacaatcctacatttaag
aggttctttgtctttcattttctatttccatttcttctttgtggttttcttgtttatcat
atttttaatctacattttctatcttctaataatcctttaaggaattccactaataataaa
atagcatttttccctttcattattagtaaagatttatatggaaagatattaattctctat
ctatatcttcttcaaattcatttcggtttctcttctttctcacatccagataatgcatta
gaagcatgtggattacttactcctttacatatagtacctgaatggtatttcttatgccaa
tatgctatgttaaaagctgtgccaaataaaaatgcaggattcattatattatttacttct
atcttcatcttattttactttatgagaagtctttcaatctcattctattttatcgtgtgg
gtaagctctagatttaatagtttctttgtaattctttggtttttagttttcatatcctta
atttggataggtggtcaatttcctgtagacaactttctatcttatggtcgtatcttgaca
ttacattattattatcttcttatctgtatcttattatcttag

>KrA3_cob
atgaaatctcatttacaatcatatccttgtcctctgatcataaattatttttggaatctt
ggtttttattagggattactattttattacaaattatatctggaatcttcttaggttta
cattatacatcagatattaattcagcatattttagtattttctttattattagagaaata
tattatggatggtgtttacgttatcttcattctaatggttcatcatttgtctttcttttg
atatttctacatcttggaagagctatatcttatggttcatatttttataatccaaatact
tggttttctggaattattattatcttcttcttaatgggaacagcatttatgggttatgtg
ttacctttaggacaaatgagtttatggggggttacagtaattacaaatttattatctgca
tttccatctttaatagaatggctttgtggaggacattacatttacaatcctacatttaag
aggttctttgtctttcattttctatttccatttcttctttgtggttttcttgtttatcat
atttttaatctacattttctatcttctaataatcctttaaggaattccactaataataaa
atagcatttttccctttcattattagtaaagatttatatggaaagatattaattctctat
ctatatcttcttcaaattcatttcggtttctcttctttctcacatccagataatgcatta
gaagcatgtggattacttactcctttacatatagtacctgaatggtatttcttatgccaa
tatgctatgttaaaagctgtgccaaataaaaatgcaggattcattatattatttacttct
atcttcatcttattttactttatgagaagtctttcaatctcattctattttatcgtgtgg
gtaagctctagatttaatagtttctttgtaattctttggtttttagttttcatatcctta
atttggataggtggtcaatttcctgtagacaactttctatcttatggtcgtatcttgaca
ttacattattattatcttcttatctgtatcttattatcttag
>KrA4_cob
atgaaatctcatttacaatcatatccttgtcctctgatcataaattatttttggaatctt
ggtttttattagggattactattttattacaaattatatctggaatcttcttaggttta
cattatacatcagatattaattcagcatattttagtattttctttattattagagaaata
tattatggatggtgtttacgttatcttcattctaatggttcatcatttgtctttcttttg
atatttctacatcttggaagagctatatcttatggttcatatttttataatccaaatact
tggttttctggaattattattatcttcttcttaatgggaacagcatttatgggttatgtg
ttacctttaggacaaatgagtttatggggggttacagtaattacaaatttattatctgca
tttccatctttaatagaatggctttgtggaggacattacatttacaatcctacatttaag
aggttctttgtctttcattttctatttccatttcttctttgtggttttcttgtttatcat
atttttaatctacattttctatcttctaataatcctttaaggaattccactaataataaa
atagcatttttccctttcattattagtaaagatttatatggaaagatattaattctctat
ctatatcttcttcaaattcatttcggtttctcttctttctcacatccagataatgcatta
gaagcatgtggattacttactcctttacatatagtacctgaatggtatttcttatgccaa
tatgctatgttaaaagctgtgccaaataaaaatgcaggattcattatattatttacttct
atcttcatcttattttactttatgagaagtctttcaatctcattctattttatcgtgtgg
gtaagctctagatttaatagtttctttgtaattctttggtttttagttttcatatcctta
atttggataggtggtcaatttcctgtagacaactttctatcttatggtcgtatcttgaca
ttacattattattatcttcttatctgtatcttattatcttag
>KrA5_cob
atgaaatctcatttacaatcatatccttgtcctctgatcataaattatttttggaatctt
ggtttttattagggattactattttattacaaattatatctggaatcttcttaggttta
cattatacatcagatattaattcagcatattttagtattttctttattattagagaaata
tattatggatggtgtttacgttatcttcattctaatggttcatcatttgtctttcttttg
atatttctacatcttggaagagctatatcttatggttcatatttttataatccaaatact
tggttttctggaattattattatcttcttcttaatgggaacagcatttatgggttatgtg
ttacctttaggacaaatgagtttatggggggttacagtaattacaaatttattatctgca
tttccatctttaatagaatggctttgtggaggacattacatttacaatcctacatttaag
aggttctttgtctttcattttctatttccatttcttctttgtggttttcttgtttatcat
atttttaatctacattttctatcttctaataatcctttaaggaattccactaataataaa
atagcatttttccctttcattattagtaaagatttatatggaaagatattaattctctat
ctatatcttcttcaaattcatttcggtttctcttctttctcacatccagataatgcatta
gaagcatgtggattacttactcctttacatatagtacctgaatggtatttcttatgccaa
tatgctatgttaaaagctgtgccaaataaaaatgcaggattcattatattatttacttct
atcttcatcttattttactttatgagaagtctttcagtatctttctattttatcgtgtgg
gtaagctctagatttaatagtttctttgtaattctttggtttttagttttcatatcctta
atttggataggtggtcaatttcctgtagacaactttctatcttatggtcgtatcttgaca
ttacattattattatcttcttatctgtatcttattatcttag

>KrA6_cob
atgaaatctcatttacaatcatatccttgtcctctgatcataaattatttttggaatctt
ggtttttattagggattactattttattacaaattatatctggaatcttcttaggttta
cattatacatcagatattaattcagcatattttagtattttctttattattagagaaata
tattatggatggtgtttacgttatcttcattctaatggttcatcatttgtctttcttttg
atatttctacatcttggaagagctatatcttatggttcatatttttataatccaaatact
tggttttctggaattattattatcttcttcttaatgggaacagcatttatgggttatgtg
ttacctttaggacaaatgagtttatggggggttacagtaattacaaatttattatctgca
tttccatctttaatagaatggctttgtggaggacattacatttacaatcctacatttaag
aggttctttgtctttcattttctatttccatttcttctttgtggttttcttgtttatcat
atttttaatctacattttctatcttctaataatcctttaaggaattccactaataataaa
atagcatttttccctttcattattagtaaagatttatatggaaagatattaattctctat
ctatatcttcttcaaattcatttcggtttctcttctttctcacatccagataatgcatta
gaagcatgtggattacttactcctttacatatagtacctgaatggtatttcttatgccaa
tatgctatgttaaaagctgtgccaaataaaaatgcaggattcattatattatttacttct
atcttcatcttattttactttatgagaagtctttcaatctcattctattttatcgtgtgg
gtaagctctagatttaatagtttctttgtaattctttggtttttagttttcatatcctta
atttggataggtggtcaatttcctgtagacaactttctatcttatggtcgtatcttgaca
ttacattattattatcttcttatctgtatcttattatcttag
>KrA7_cob
atgaaatctcatttacaatcatatccttgtcctctgatcataaattatttttggaatctt
ggtttttattagggattactattttattacaaattatatctggaatcttcttaggttta
cattatacatcagatattaattcagcatattttagtattttctttattattagagaaata
tattatggatggtgtttacgttatcttcattctaatggttcatcatttgtctttcttttg
atatttctacatcttggaagagctatatcttatggttcatatttttataatccaaatact
tggttttctggaattattattatcttcttcttaatgggaacagcatttatgggttatgtg
ttacctttaggacaaatgagtttatggggggttacagtaattacaaatttattatctgca
tttccatctttaatagaatggctttgtggaggacattacatttacaatcctacatttaag
aggttctttgtctttcattttctatttccatttcttctttgtggttttcttgtttatcat
atttttaatctacattttctatcttctaataatcctttaaggaattccactaataataaa
atagcatttttccctttcattattagtaaagatttatatggaaagatattaattctctat
ctatatcttcttcaaattcatttcggtttctcttctttctcacatccagataatgcatta
gaagcatgtggattacttactcctttacatatagtacctgaatggtatttcttatgccaa
tatgctatgttaaaagctgtgccaaataaaaatgcaggattcattatattatttacttct
atcttcatcttattttactttatgagaagtctttcaatctcattctattttatcgtgtgg
gtaagctctagatttaatagtttctttgtaattctttggtttttagttttcatatcctta
atttggataggtggtcaatttcctgtagacaactttctatcttatggtcgtatcttgaca
ttacattattattatcttcttatctgtatcttattatcttag
>KrA8_cob
atgaaatctcatttacaatcatatccttgtcctctgatcataaattatttttggaatctt
ggtttttattagggattactattttattacaaattatatctggaatcttcttaggttta
cattatacatcagatattaattcagcatattttagtattttctttattattagagaaata
tattatggatggtgtttacgttatcttcattctaatggttcatcatttgtctttcttttg
atatttctacatcttggaagagctatatcttatggttcatatttttataatccaaatact
tggttttctggaattattattatcttcttcttaatgggaacagcatttatgggttatgtg
ttacctttaggacaaatgagtttatggggggttacagtaattacaaatttattatctgca
tttccatctttaatagaatggctttgtggaggacattacatttacaatcctacatttaag
aggttctttgtctttcattttctatttccatttcttctttgtggttttcttgtttatcat
atttttaatctacattttctatcttctaataatcctttaaggaattccactaataataaa
atagcatttttccctttcattattagtaaagatttatatggaaagatattaattctctat
ctatatcttcttcaaattcatttcggtttctcttctttctcacatccagataatgcatta
gaagcatgtggattacttactcctttacatatagtacctgaatggtatttcttatgccaa
tatgctatgttaaaagctgtgccaaataaaaatgcaggattcattatattatttacttct
atcttcatcttattttactttatgagaagtctttcaatctcattctattttatcgtgtgg
gtaagctctagatttaatagtttctttgtaattctttggtttttagttttcatatcctta
atttggataggtggtcaatttcctgtagacaactttctatcttatggtcgtatcttgaca
ttacattattattatcttcttatctgtatcttattatcttag

>KrA9_cob
atgaaatctcatttacaatcatatccttgtcctctgatcataaattatttttggaatctt
ggtttttattagggattactattttattacaaattatatctggaatcttcttaggttta
cattatacatcagatattaattcagcatattttagtattttctttattattagagaaata
tattatggatggtgtttacgttatcttcattctaatggttcatcatttgtctttcttttg
atatttctacatcttggaagagctatatcttatggttcatatttttataatccaaatact
tggttttctggaattattattatcttcttcttaatgggaacagcatttatgggttatgtg
ttacctttaggacaaatgagtttatggggggttacagtaattacaaatttattatctgca
tttccatctttaatagaatggctttgtggaggacattacatttacaatcctacatttaag
aggttctttgtctttcattttctatttccatttcttctttgtggttttcttgtttatcat
atttttaatctacattttctatcttctaataatcctttaaggaattccactaataataaa
atagcatttttccctttcattattagtaaagatttatatggaaagatattaattctctat
ctatatcttcttcaaattcatttcggtttctcttctttctcacatccagataatgcatta
gaagcatgtggattacttactcctttacatatagtacctgaatggtatttcttatgccaa
tatgctatgttaaaagctgtgccaaataaaaatgcaggattcattatattatttacttct
atcttcatcttattttactttatgagaagtctttcaatctcattctattttatcgtgtgg
gtaagctctagatttaatagtttctttgtaattctttggtttttagttttcatatcctta
atttggataggtggtcaatttcctgtagacaactttctatcttatggtcgtatcttgaca
ttacattattattatcttcttatctgtatcttattatcttag
>KrC1_cob
atgaaatctcatttacaatcatatccttgtcctctgatcataaattatttttggaatctt
ggtttttattagggattactattttattacaaattatatctggaatcttcttaggttta
cattatacatcagatattaattcagcatattttagtattttctttattattagagaaata
tattatggatggtgtttacgttatcttcattctaatggttcatcatttgtctttcttttg
atatttctacatcttggaagagctatatcttatggttcatatttttataatccaaatact
tggttttctggaattattattatcttcttcttaatgggaacagcatttatgggttatgtg
ttacctttaggacaaatgagtttatggggggttacagtaattacaaatttattatctgca
tttccatctttaatagaatggctttgtggaggacattacatttacaatcctacatttaag
aggttctttgtctttcattttctatttccatttcttctttgtggttttcttgtttatcat
atttttaatctacattttctatcttctaataatcctttaaggaattccactaataataaa
atagcatttttccctttcattattagtaaagatttatatggaaagatattaattctctat
ctatatcttcttcaaattcatttcggtttctcttctttctcacatccagataatgcatta
gaagcatgtggattacttactcctttacatatagtacctgaatggtatttcttatgccaa
tatgctatgttaaaagctgtgccaaataaaaatgcaggattcattatattatttacttct
atcttcatcttattttactttatgagaagtctttcaatctcattctattttatcgtgtgg
gtaagctctagatttaatagtttctttgtaattctttggtttttagttttcatatcctta
atttggataggtggtcaatttcctgtagacaactttctatcttatggtcgtatcttgaca
ttacattattattatcttcttatctgtatcttattatcttag
>KrC10_cob
atgaaatctcatttacaatcatatccttgtcctctgatcataaattatttttggaatctt
ggtttttattagggattactattttattacaaattatatctggaatcttcttaggttta
cattatacatcagatattaattcagcatattttagtattttctttattattagagaaata
tattatggatggtgtttacgttatcttcattctaatggttcatcatttgtctttcttttg
atatttctacatcttggaagagctatatcttatggttcatatttttataatccaaatact
tggttttctggaattattattatcttcttcttaatgggaacagcatttatgggttatgtg
ttacctttaggacaaatgagtttatggggggttacagtaattacaaatttattatctgca
tttccatctttaatagaatggctttgtggaggacattacatttacaatcctacatttaag
aggttctttgtctttcattttctatttccatttcttctttgtggttttcttgtttatcat
atttttaatctacattttctatcttctaataatcctttaaggaattccactaataataaa
atagcatttttccctttcattattagtaaagatttatatggaaagatattaattctctat
ctatatcttcttcaaattcatttcggtttctcttctttctcacatccagataatgcatta
gaagcatgtggattacttactcctttacatatagtacctgaatggtatttcttatgccaa
tatgctatgttaaaagctgtgccaaataaaaatgcaggattcattatattatttacttct
atcttcatcttattttactttatgagaagtctttcaatctcattctattttatcgtgtgg
gtaagctctagatttaatagtttctttgtaattctttggtttttagttttcatatcctta
atttggataggtggtcaatttcctgtagacaactttctatcttatggtcgtatcttgaca
ttacattattattatcttcttatctgtatcttattatcttag

>KrC11_cob
atgaaatctcatttacaatcatatccttgtcctctgatcataaattatttttggaatctt
ggtttttattagggattactattttattacaaattatatctggaatcttcttaggttta
cattatacatcagatattaattcagcatattttagtattttctttattattagagaaata
tattatggatggtgtttacgttatcttcattctaatggttcatcatttgtctttcttttg
atatttctacatcttggaagagctatatcttatggttcatatttttataatccaaatact
tggttttctggaattattattatcttcttcttaatgggaacagcatttatgggttatgtg
ttacctttaggacaaatgagtttatggggggttacagtaattacaaatttattatctgca
tttccatctttaatagaatggctttgtggaggacattacatttacaatcctacatttaag
aggttctttgtctttcattttctatttccatttcttctttgtggttttcttgtttatcat
atttttaatctacattttctatcttctaataatcctttaaggaattccactaataataaa
atagcatttttccctttcattattagtaaagatttatatggaaagatattaattctctat
ctatatcttcttcaaattcatttcggtttctcttctttctcacatccagataatgcatta
gaagcatgtggattacttactcctttacatatagtacctgaatggtatttcttatgccaa
tatgctatgttaaaagctgtgccaaataaaaatgcaggattcattatattatttacttct
atcttcatcttattttactttatgagaagtctttcaatctcattctattttatcgtgtgg
gtaagctctagatttaatagtttctttgtaattctttggtttttagttttcatatcctta
atttggataggtggtcaatttcctgtagacaactttctatcttatggtcgtatcttgaca
ttacattattattatcttcttatctgtatcttattatcttag
>KrC12_cob
atgaaatctcatttacaatcatatccttgtcctctgatcataaattatttttggaatctt
ggtttttattagggattactattttattacaaattatatctggaatcttcttaggttta
cattatacatcagatattaattcagcatattttagtattttctttattattagagaaata
tattatggatggtgtttacgttatcttcattctaatggttcatcatttgtctttcttttg
atatttctacatcttggaagagctatatcttatggttcatatttttataatccaaatact
tggttttctggaattattattatcttcttcttaatgggaacagcatttatgggttatgtg
ttacctttaggacaaatgagtttatggggggttacagtaattacaaatttattatctgca
tttccatctttaatagaatggctttgtggaggacattacatttacaatcctacatttaag
aggttctttgtctttcattttctatttccatttcttctttgtggttttcttgtttatcat
atttttaatctacattttctatcttctaataatcctttaaggaattccactaataataaa
atagcatttttccctttcattattagtaaagatttatatggaaagatattaattctctat
ctatatcttcttcaaattcatttcggtttctcttctttctcacatccagataatgcatta
gaagcatgtggattacttactcctttacatatagtacctgaatggtatttcttatgccaa
tatgctatgttaaaagctgtgccaaataaaaatgcaggattcattatattatttacttct
atcttcatcttattttactttatgagaagtctttcaatctcattctattttatcgtgtgg
gtaagctctagatttaatagtttctttgtaattctttggtttttagttttcatatcctta
atttggataggtggtcaatttcctgtagacaactttctatcttatggtcgtatcttgaca
ttacattattattatcttcttatctgtatcttattatcttag
>KrC13_cob
atgaaatctcatttacaatcatatccttgtcctctgatcataaattatttttggaatctt
ggtttttattagggattactattttattacaaattatatctggaatcttcttaggttta
cattatacatcagatattaattcagcatattttagtattttctttattattagagaaata
tattatggatggtgtttacgttatcttcattctaatggttcatcatttgtctttcttttg
atatttctacatcttggaagagctatatcttatggttcatatttttataatccaaatact
tggttttctggaattattattatcttcttcttaatgggaacagcatttatgggttatgtg
ttacctttaggacaaatgagtttatggggggttacagtaattacaaatttattatctgca
tttccatctttaatagaatggctttgtggaggacattacatttacaatcctacatttaag
aggttctttgtctttcattttctatttccatttcttctttgtggttttcttgtttatcat
atttttaatctacattttctatcttctaataatcctttaaggaattccactaataataaa
atagcatttttccctttcattattagtaaagatttatatggaaagatattaattctctat
ctatatcttcttcaaattcatttcggtttctcttctttctcacatccagataatgcatta
gaagcatgtggattacttactcctttacatatagtacctgaatggtatttcttatgccaa
tatgctatgttaaaagctgtgccaaataaaaatgcaggattcattatattatttacttct
atcttcatcttattttactttatgagaagtctttcaatctcattctattttatcgtgtgg
gtaagctctagatttaatagtttctttgtaattctttggtttttagttttcatatcctta
atttggataggtggtcaatttcctgtagacaactttctatcttatggtcgtatcttgaca
ttacattattattatcttcttatctgtatcttattatcttag

>KrC15_cob
atgaaatctcatttacaatcatatccttgtcctctgatcataaattatttttggaatctt
ggtttttattagggattactattttattacaaattatatctggaatcttcttaggttta
cattatacatcagatattaattcagcatattttagtattttctttattattagagaaata
tattatggatggtgtttacgttatcttcattctaatggttcatcatttgtctttcttttg
atatttctacatcttggaagagctatatcttatggttcatatttttataatccaaatact
tggttttctggaattattattatcttcttcttaatgggaacagcatttatgggttatgtg
ttacctttaggacaaatgagtttatggggggttacagtaattacaaatttattatctgca
tttccatctttaatagaatggctttgtggaggacattacatttacaatcctacatttaag
aggttctttgtctttcattttctatttccatttcttctttgtggttttcttgtttatcat
atttttaatctacattttctatcttctaataatcctttaaggaattccactaataataaa
atagcatttttccctttcattattagtaaagatttatatggaaagatattaattctctat
ctatatcttcttcaaattcatttcggtttctcttctttctcacatccagataatgcatta
gaagcatgtggattacttactcctttacatatagtacctgaatggtatttcttatgccaa
tatgctatgttaaaagctgtgccaaataaaaatgcaggattcattatattatttacttct
atcttcatcttattttactttatgagaagtctttcaatctcattctattttatcgtgtgg
gtaagctctagatttaatagtttctttgtaattctttggtttttagttttcatatcctta
atttggataggtggtcaatttcctgtagacaactttctatcttatggtcgtatcttgaca
ttacattattattatcttcttatctgtatcttattatcttag
>KrC2_cob
atgaaatctcatttacaatcatatccttgtcctctgatcataaattatttttggaatctt
ggtttttattagggattactattttattacaaattatatctggaatcttcttaggttta
cattatacatcagatattaattcagcatattttagtattttctttattattagagaaata
tattatggatggtgtttacgttatcttcattctaatggttcatcatttgtctttcttttg
atatttctacatcttggaagagctatatcttatggttcatatttttataatccaaatact
tggttttctggaattattattatcttcttcttaatgggaacagcatttatgggttatgtg
ttacctttaggacaaatgagtttatggggggttacagtaattacaaatttattatctgca
tttccatctttaatagaatggctttgtggaggacattacatttacaatcctacatttaag
aggttctttgtctttcattttctatttccatttcttctttgtggttttcttgtttatcat
atttttaatctacattttctatcttctaataatcctttaaggaattccactaataataaa
atagcatttttccctttcattattagtaaagatttatatggaaagatattaattctctat
ctatatcttcttcaaattcatttcggtttctcttctttctcacatccagataatgcatta
gaagcatgtggattacttactcctttacatatagtacctgaatggtatttcttatgccaa
tatgctatgttaaaagctgtgccaaataaaaatgcaggattcattatattatttacttct
atcttcatcttattttactttatgagaagtctttcaatatctttctattttatcgtgtgg
gtaagctctagatttaatagtttctttgtaattctttggtttttagttttcatatcctta
atttggataggtggtcaatttcctgtagacaactttctatcttatggtcgtatcttgaca
ttacattattattatcttcttatctgtatcttattatcttag
>KrC3_cob
atgaaatctcatttacaatcatatccttgtcctctgatcataaattatttttggaatctt
ggtttttattagggattactattttattacaaattatatctggaatcttcttaggttta
cattatacatcagatattaattcagcatattttagtattttctttattattagagaaata
tattatggatggtgtttacgttatcttcattctaatggttcatcatttgtctttcttttg
atatttctacatcttggaagagctatatcttatggttcatatttttataatccaaatact
tggttttctggaattattattatcttcttcttaatgggaacagcatttatgggttatgtg
ttacctttaggacaaatgagtttatggggggttacagtaattacaaatttattatctgca
tttccatctttaatagaatggctttgtggaggacattacatttacaatcctacatttaag
aggttctttgtctttcattttctatttccatttcttctttgtggttttcttgtttatcat
atttttaatctacattttctatcttctaataatcctttaaggaattccactaataataaa
atagcatttttccctttcattattagtaaagatttatatggaaagatattaattctctat
ctatatcttcttcaaattcatttcggtttctcttctttctcacatccagataatgcatta
gaagcatgtggattacttactcctttacatatagtacctgaatggtatttcttatgccaa
tatgctatgttaaaagctgtgccaaataaaaatgcaggattcattatattatttacttct
atcttcatcttattttactttatgagaagtctttcaatctcattctattttatcgtgtgg
gtaagctctagatttaatagtttctttgtaattctttggtttttagttttcatatcctta
atttggataggtggtcaatttcctgtagacaactttctatcttatggtcgtatcttgaca
ttacattattattatcttcttatctgtatcttattatcttag

>KrC4_cob
atgaaatctcatttacaatcatatccttgtcctctgatcataaattatttttggaatctt
ggtttttattagggattactattttattacaaattatatctggaatcttcttaggttta
cattatacatcagatattaattcagcatattttagtattttctttattattagagaaata
tattatggatggtgtttacgttatcttcattctaatggttcatcatttgtctttcttttg
atatttctacatcttggaagagctatatcttatggttcatatttttataatccaaatact
tggttttctggaattattattatcttcttcttaatgggaacagcatttatgggttatgtg
ttacctttaggacaaatgagtttatgggggggttacagtaattacaaatttattatctgca
tttccatctttaatagaatggctttgtggaggacattacatttacaatcctacatttaag
aggttctttgtctttcattttctatttccatttcttctttgtggttttcttgtttatcat
atttttaatctacattttctatcttctaataatcctttaaggaattccactaataataaa
atagcatttttccctttcattattagtaaagatttatatggaaagatattaattctctat
ctatatcttcttcaaattcatttcggtttctcttctttctcacatccagataatgcatta
gaagcatgtggattacttactcctttacatatagtacctgaatggtatttcttatgccaa
tatgctatgttaaaagctgtgccaaataaaaatgcaggattcattatattatttacttct
atcttcatcttattttactttatgagaagtctttcaatatctttctattttatcgtgtgg
gtaagctctagatttaatagtttctttgtaattctttggtttttagttttcatatcctta
atttggataggtggtcaatttcctgtagacaactttctatcttatggtcgtatcttgaca
ttacattattattatcttcttatctgtatcttattatcttag
>KrC5_cob
atgaaatctcatttacaatcatatccttgtcctctgatcataaattatttttggaatctt
ggtttttattagggattactattttattacaaattatatctggaatcttcttaggttta
cattatacatcagatattaattcagcatattttagtattttctttattattagagaaata
tattatggatggtgtttacgttatcttcattctaatggttcatcatttgtctttcttttg
atatttctacatcttggaagagctatatcttatggttcatatttttataatccaaatact
tggttttctggaattattattatcttcttcttaatgggaacagcatttatgggttatgtg
ttacctttaggacaaatgagtttatgggggggttacagtaattacaaatttattatctgca
tttccatctttaatagaatggctttgtggaggacattacatttacaatcctacatttaag
aggttctttgtctttcattttctatttccatttcttctttgtggttttcttgtttatcat
atttttaatctacattttctatcttctaataatcctttaaggaattccactaataataaa
atagcatttttccctttcattattagtaaagatttatatggaaagatattaattctctat
ctatatcttcttcaaattcatttcggtttctcttctttctcacatccagataatgcatta
gaagcatgtggattacttactcctttacatatagtacctgaatggtatttcttatgccaa
tatgctatgttaaaagctgtgccaaataaaaatgcaggattcattatattatttacttct
atcttcatcttattttactttatgagaagtctttcaatctcattctattttatcgtgtgg
gtaagctctagatttaatagtttctttgtaattctttggtttttagttttcatatcctta
atttggataggtggtcaatttcctgtagacaactttctatcttatggtcgtatcttgaca
ttacattattattatcttcttatctgtatcttattatcttag
>KrC6_cob
atgaaatctcatttacaatcatatccttgtcctctgatcataaattatttttggaatctt
ggtttttattagggattactattttattacaaattatatctggaatcttcttaggttta
cattatacatcagatattaattcagcatattttagtattttctttattattagagaaata
tattatggatggtgtttacgttatcttcattctaatggttcatcatttgtctttcttttg
atatttctacatcttggaagagctatatcttatggttcatatttttataatccaaatact
tggttttctggaattattattatcttcttcttaatgggaacagcatttatgggttatgtg
ttacctttaggacaaatgagtttatgggggggttacagtaattacaaatttattatctgca
tttccatctttaatagaatggctttgtggaggacattacatttacaatcctacatttaag
aggttctttgtctttcattttctatttccatttcttctttgtggttttcttgtttatcat
atttttaatctacattttctatcttctaataatcctttaaggaattccactaataataaa
atagcatttttccctttcattattagtaaagatttatatggaaagatattaattctctat
ctatatcttcttcaaattcatttcggtttctcttctttctcacatccagataatgcatta
gaagcatgtggattacttactcctttacatatagtacctgaatggtatttcttatgccaa
tatgctatgttaaaagctgtgccaaataaaaatgcaggattcattatattatttacttct
atcttcatcttattttactttatgagaagtctttcagtatctttctattttatcgtgtgg
gtaagctctagatttaatagtttctttgtaattctttggtttttagttttcatatcctta
atttggataggtggtcaatttcctgtagacaactttctatcttatggtcgtatcttgaca
ttacattattattatcttcttatctgtatcttattatcttag

>KrC7_cob
atgaaatctcatttacaatcatatccttgtcctctgatcataaattatttttggaatctt
ggtttttattagggattactattttattacaaattatatctggaatcttcttaggttta
cattatacatcagatattaattcagcatattttagtattttctttattattagagaaata
tattatggatggtgtttacgttatcttcattctaatggttcatcatttgtctttcttttg
atatttctacatcttggaagagctatatcttatggttcatatttttataatccaaatact
tggttttctggaattattattatcttcttcttaatgggaacagcatttatgggttatgtg
ttacctttaggacaaatgagtttatggggggttacagtaattacaaatttattatctgca
tttccatctttaatagaatggctttgtggaggacattacatttacaatcctacatttaag
aggttctttgtctttcattttctatttccatttcttctttgtggttttcttgtttatcat
atttttaatctacattttctatcttctaataatcctttaaggaattccactaataataaa
atagcatttttccctttcattattagtaaagatttatatggaaagatattaattctctat
ctatatcttcttcaaattcatttcggtttctcttctttctcacatccagataatgcatta
gaagcatgtggattacttactcctttacatatagtacctgaatggtatttcttatgccaa
tatgctatgttaaaagctgtgccaaataaaaatgcaggattcattgtcttactaacttct
atctttgtattattttattttatgagaagtctttcagtatctttctattttatcgtgtgg
gtaagctctagatttaatagtttctttgtaattctttggtttttagttttcatatcctta
atttggataggtggtcaatttcctgtagacaactttctatcttatggtcgtatcttgaca
ttacattattattatcttcttatctgtatcttattatcttag
>KrC8_cob
atgaaatctcatttacaatcatatccttgtcctctgatcataaattatttttggaatctt
ggtttttattagggattactattttattacaaattatatctggaatcttcttaggttta
cattatacatcagatattaattcagcatattttagtattttctttattattagagaaata
tattatggatggtgtttacgttatcttcattctaatggttcatcatttgtctttcttttg
atatttctacatcttggaagagctatatcttatggttcatatttttataatccaaatact
tggttttctggaattattattatcttcttcttaatgggaacagcatttatgggttatgtg
ttacctttaggacaaatgagtttatggggggttacagtaattacaaatttattatctgca
tttccatctttaatagaatggctttgtggaggacattacatttacaatcctacatttaag
aggttctttgtctttcattttctatttccatttcttctttgtggttttcttgtttatcat
atttttaatctacattttctatcttctaataatcctttaaggaattccactaataataaa
atagcatttttccctttcattattagtaaagatttatatggaaagatattaattctctat
ctatatcttcttcaaattcatttcggtttctcttctttctcacatccagataatgcatta
gaagcatgtggattacttactcctttacatatagtacctgaatggtatttcttatgccaa
tatgctatgttaaaagctgtgccaaataaaaatgcaggattcattatattatttacttct
atcttcatcttattttactttatgagaagtctttcaatctcattctattttatcgtgtgg
gtaagctctagatttaatagtttctttgtaattctttggtttttagttttcatatcctta
atttggataggtggtcaatttcctgtagacaactttctatcttatggtcgtatcttgaca
ttacattattattatcttcttatctgtatcttattatcttag
>KrC9_cob
atgaaatctcatttacaatcatatccttgtcctctgatcataaattatttttggaatctt
ggtttttattagggattactattttattacaaattatatctggaatcttcttaggttta
cattatacatcagatattaattcagcatattttagtattttctttattattagagaaata
tattatggatggtgtttacgttatcttcattctaatggttcatcatttgtctttcttttg
atatttctacatcttggaagagctatatcttatggttcatatttttataatccaaatact
tggttttctggaattattattatcttcttcttaatgggaacagcatttatgggttatgtg
ttacctttaggacaaatgagtttatggggggttacagtaattacaaatttattatctgca
tttccatctttaatagaatggctttgtggaggacattacatttacaatcctacatttaag
aggttctttgtctttcattttctatttccatttcttctttgtggttttcttgtttatcat
atttttaatctacattttctatcttctaataatcctttaaggaattccactaataataaa
atagcatttttccctttcattattagtaaagatttatatggaaagatattaattctctat
ctatatcttcttcaaattcatttcggtttctcttctttctcacatccagataatgcatta
gaagcatgtggattacttactcctttacatatagtacctgaatggtatttcttatgccaa
tatgctatgttaaaagctgtgccaaataaaaatgcaggattcattatattatttacttct
atcttcatcttattttactttatgagaagtctttcaatctcattctattttatcgtgtgg
gtaagctctagatttaatagtttctttgtaattctttggtttttagttttcatatcctta
atttggataggtggtcaatttcctgtagacaactttctatcttatggtcgtatcttgaca
ttacattattattatcttcttatctgtatcttattatcttag

>KrD1_cob
atgaaatctcatttacaatcatatccttgtcctctgatcataaattatttttggaatctt
ggtttttattagggattactattttattacaaattatatctggaatcttcttaggttta
cattatacatcagatattaattcagcatattttagtattttctttattattagagaaata
tattatggatggtgtttacgttatcttcattctaatggttcatcatttgtctttcttttg
atatttctacatcttggaagagctatatcttatggttcatatttttataatccaaatact
tggttttctggaattattattatcttcttcttaatgggaacagcatttatgggttatgtg
ttacctttaggacaaatgagtttatgggggggttacagtaattacaaatttattatctgca
tttccatctttaatagaatggctttgtggaggacattacatttacaatcctacatttaag
aggttctttgtctttcattttctatttccatttcttctttgtggttttcttgtttatcat
atttttaatctacattttctatcttctaataatcctttaaggaattccactaataataaa
atagcatttttccctttcattattagtaaagatttatatggaaagatattaattctctat
ctatatcttcttcaaattcatttcggtttctcttctttctcacatccagataatgcatta
gaagcatgtggattacttactcctttacatatagtacctgaatggtatttcttatgccaa
tatgctatgttaaaagctgtgccaaataaaaatgcaggattcattatattatttacttct
atcttcatcttattttactttatgagaagtctttcagtatctttctattttatcgtgtgg
gtaagctctagatttaatagtttctttgtaattctttggtttttagttttcatatcctta
atttggataggtggtcaatttcctgtagacaactttctatcttatggtcgtatcttgaca
ttacattattattatcttcttatctgtatcttattatcttag
>KrD11_cob
atgaaatctcatttacaatcatatccttgtcctctgatcataaattatttttggaatctt
ggtttttattagggattactattttattacaaattatatctggaatcttcttaggttta
cattatacatcagatattaattcagcatattttagtattttctttattattagagaaata
tattatggatggtgtttacgttatcttcattctaatggttcatcatttgtctttcttttg
atatttctacatcttggaagagctatatcttatggttcatatttttataatccaaatact
tggttttctggaattattattatcttcttcttaatgggaacagcatttatgggttatgtg
ttacctttaggacaaatgagtttatgggggggttacagtaattacaaatttattatctgca
tttccatctttaatagaatggctttgtggaggacattacatttacaatcctacatttaag
aggttctttgtctttcattttctatttccatttcttctttgtggttttcttgtttatcat
atttttaatctacattttctatcttctaataatcctttaaggaattccactaataataaa
atagcatttttccctttcattattagtaaagatttatatggaaagatattaattctctat
ctatatcttcttcaaattcatttcggtttctcttctttctcacatccagataatgcatta
gaagcatgtggattacttactcctttacatatagtacctgaatggtatttcttatgccaa
tatgctatgttaaaagctgtgccaaataaaaatgcaggattcattatattatttacttct
atcttcatcttattttactttatgagaagtctttcaatctcattctattttatcgtgtgg
gtaagctctagatttaatagtttctttgtaattctttggtttttagttttcatatcctta
atttggataggtggtcaatttcctgtagacaactttctatcttatggtcgtatcttgaca
ttacattattattatcttcttatctgtatcttattatcttag
>KrD13_cob
atgaaatctcatttacaatcatatccttgtcctctgatcataaattatttttggaatctt
ggtttttattagggattactattttattacaaattatatctggaatcttcttaggttta
cattatacatcagatattaattcagcatattttagtattttctttattattagagaaata
tattatggatggtgtttacgttatcttcattctaatggttcatcatttgtctttcttttg
atatttctacatcttggaagagctatatcttatggttcatatttttataatccaaatact
tggttttctggaattattattatcttcttcttaatgggaacagcatttatgggttatgtg
ttacctttaggacaaatgagtttatgggggggttacagtaattacaaatttattatctgca
tttccatctttaatagaatggctttgtggaggacattacatttacaatcctacatttaag
aggttctttgtctttcattttctatttccatttcttctttgtggttttcttgtttatcat
atttttaatctacattttctatcttctaataatcctttaaggaattccactaataataaa
atagcatttttccctttcattattagtaaagatttatatggaaagatattaattctctat
ctatatcttcttcaaattcatttcggtttctcttctttctcacatccagataatgcatta
gaagcatgtggattacttactcctttacatatagtacctgaatggtatttcttatgccaa
tatgctatgttaaaagctgtgccaaataaaaatgcaggattcattatattatttacttct
atcttcatcttattttactttatgagaagtctttcaatctcattctattttatcgtgtgg
gtaagctctagatttaatagtttctttgtaattctttggtttttagttttcatatcctta
atttggataggtggtcaatttcctgtagacaactttctatcttatggtcgtatcttgaca
ttacattattattatcttcttatctgtatcttattatcttag

>KrD3_cob
atgaaatctcatttacaatcatatccttgtcctctgatcataaattatttttggaatctt
ggttttttattagggattactattttattacaaattatatctggaatcttcttaggttta
cattatacatcagatattaattcagcatattttagtattttctttattattagagaaata
tattatggatggtgtttacgttatcttcattctaatggttcatcatttgtctttcttttg
atatttctacatcttggaagagctatatcttatggttcatatttttataatccaaatact
tggttttctggaattattattatcttcttcttaatgggaacagcatttatgggttatgtg
ttacctttaggacaaatgagtttatggggggttacagtaattacaaatttattatctgca
tttccatctttaatagaatggctttgtggaggacattacatttacaatcctacatttaag
aggttctttgtctttcattttctatttccatttcttctttgtggttttcttgtttatcat
atttttaatctacattttctatcttctaataatcctttaaggaattccactaataataaa
atagcatttttccctttcattattagtaaagatttatatggaaagatattaattctctat
ctatatcttcttcaaattcatttcggtttctcttctttctcacatccagataatgcatta
gaagcatgtggattacttactcctttacatatagtacctgaatggtatttcttatgccaa
tatgctatgttaaaagctgtgccaaataaaaatgcaggattcattatattatttacttct
atcttcatcttattttactttatgagaagtctttcaatctcattctattttatcgtgtgg
gtaagctctagatttaatagtttctttgtaattctttggtttttagttttcatatcctta
atttggataggtggtcaatttcctgtagacaactttctatcttatggtcgtatcttgaca
ttacattattattatcttcttatctgtatcttattatcttag
>KrD8_cob
atgaaatctcatttacaatcatatccttgtcctctgatcataaattatttttggaatctt
ggttttttattagggattactattttattacaaattatatctggaatcttcttaggttta
cattatacatcagatattaattcagcatattttagtattttctttattattagagaaata
tattatggatggtgtttacgttatcttcattctaatggttcatcatttgtctttcttttg
atatttctacatcttggaagagctatatcttatggttcatatttttataatccaaatact
tggttttctggaattattattatcttcttcttaatgggaacagcatttatgggttatgtg
ttacctttaggacaaatgagtttatggggggttacagtaattacaaatttattatctgca
tttccatctttaatagaatggctttgtggaggacattacatttacaatcctacatttaag
aggttctttgtctttcattttctatttccatttcttctttgtggttttcttgtttatcat
atttttaatctacattttctatcttctaataatcctttaaggaattccactaataataaa
atagcatttttccctttcattattagtaaagatttatatggaaagatattaattctctat
ctatatcttcttcaaattcatttcggtttctcttctttctcacatccagataatgcatta
gaagcatgtggattacttactcctttacatatagtacctgaatggtatttcttatgccaa
tatgctatgttaaaagctgtgccaaataaaaatgcaggattcattatattatttacttct
atcttcatcttattttactttatgagaagtctttcaatctcattctattttatcgtgtgg
gtaagctctagatttaatagtttctttgtaattctttggtttttagttttcatatcctta
atttggataggtggtcaatttcctgtagacaactttctatcttatggtcgtatcttgaca
ttacattattattatcttcttatctgtatcttattatcttag
>KrD9_cob
atgaaatctcatttacaatcatatccttgtcctctgatcataaattatttttggaatctt
ggttttttattagggattactattttattacaaattatatctggaatcttcttaggttta
cattatacatcagatattaattcagcatattttagtattttctttattattagagaaata
tattatggatggtgtttacgttattttcattctaatggttcatcatttgtctttcttttg
atatttctacatcttggaagagctatatcttatggttcatatttttataatccaaatact
tggttttctggaattattattatcttcttcttaatgggaacagcatttatgggttatgtg
ttacctttaggacaaatgagtttatggggggttacagtaattacaaatttattatctgca
tttccatctttagtagaatggctttgtggaggatattgcattcacaatcctacatttaag
aggttctttgtctttcattttctacttccatttcttctttgtggttttcttgtttatcat
atttttaatctacattttctatcttctaataatcctttaaggaattccactaataataaa
atagcatttttccctttcattattagtaaagatttatatggaaagatattaattctctat
ctatatcttcttcaaattcatttcggtttctcttctttctcacatccagataatgcatta
gaagcatgtggattacttactcctttacatatagtacctgaatggtatttcttatgccaa
tatgctatgttaaaagctgtgccaaataaaaatgcaggattcattatattatttacttct
atcttcatcttattttactttatgagaagtctttcagtatctttctattttatcgtgtgg
gtaagctctagatttaatagtttctttgtaattctttggtttttagttttcatatcctta
atttggataggtggtcaatttcctgtagacaactttctatcttatggtcgtatcttgaca
ttacattattattatcttcttatctgtatcttattatcttag

>KrE1_cob
atgaaatctcatttacaatcatatccttgtcctctgatcataaattatttttggaatctt
ggtttttattagggattactattttattacaaattatatctggaatcttcttaggttta
cattatacatcagatattaattcagcatattttagtattttctttattattagagaaata
tattatggatggtgtttacgttatcttcattctaatggttcatcatttgtctttcttttg
atatttctacatcttggaagagctatatcttatggttcatatttttataatccaaatact
tggttttctggaattattattatcttcttcttaatgggaacagcatttatgggttatgtg
ttacctttaggacaaatgagtttatggggggttacagtaattacaaatttattatctgca
tttccatctttaatagaatggctttgtggaggacattacatttacaatcctacatttaag
aggttctttgtctttcattttctatttccatttcttctttgtggttttcttgtttatcat
atttttaatctacattttctatcttctaataatcctttaaggaattccactaataataaa
atagcatttttccctttcattattagtaaagatttatatggaaagatattaattctctat
ctatatcttcttcaaattcatttcggtttctcttctttctcacatccagataatgcatta
gaagcatgtggattacttactcctttacatatagtacctgaatggtatttcttatgccaa
tatgctatgttaaaagctgtgccaaataaaaatgcaggattcattatattatttacttct
atcttcatcttattttactttatgagaagtctttcaatctcattctattttatcgtgtgg
gtaagctctagatttaatagtttctttgtaattctttggtttttagttttcatatcctta
atttggataggtggtcaatttcctgtagacaactttctatcttatggtcgtatcttgaca
ttacattattattatcttcttatctgtatcttattatcttag
>KrE10_cob
atgaaatctcatttacaatcatatccttgtcctctgatcataaattatttttggaatctt
ggtttttattagggattactattttattacaaattatatctggaatcttcttaggttta
cattatacatcagatattaattcagcatattttagtattttctttattattagagaaata
tattatggatggtgtttacgttatcttcattctaatggttcatcatttgtctttcttttg
atatttctacatcttggaagagctatatcttatggttcatatttttataatccaaatact
tggttttctggaattattattatcttcttcttaatgggaacagcatttatgggttatgtg
ttacctttaggacaaatgagtttatggggggttacagtaattacaaatttattatctgca
tttccatctttaatagaatggctttgtggaggacattacatttacaatcctacatttaag
aggttctttgtctttcattttctatttccatttcttctttgtggttttcttgtttatcat
atttttaatctacattttctatcttctaataatcctttaaggaattccactaataataaa
atagcatttttccctttcattattagtaaagatttatatggaaagatattaattctctat
ctatatcttcttcaaattcatttcggtttctcttctttctcacatccagataatgcatta
gaagcatgtggattacttactcctttacatatagtacctgaatggtatttcttatgccaa
tatgctatgttaaaagctgtgccaaataaaaatgcaggattcattgtcttactaacttct
atctttgtattattttattttatgagaagtctttcagtatctttctattttatcgtgtgg
gtaagctctagatttaatagtttctttgtaattctttggtttttagttttcatatcctta
atttggataggtggtcaatttcctgtagacaactttctatcttatggtcgtatcttgaca
ttacattattattatcttcttatctgtatcttattatcttag
>KrE11_cob
atgaaatctcatttacaatcatatccttgtcctctgatcataaattatttttggaatctt
ggtttttattagggattactattttattacaaattatatctggaatcttcttaggttta
cattatacatcagatattaattcagcatattttagtattttctttattattagagaaata
tattatggatggtgtttacgttatcttcattctaatggttcatcatttgtctttcttttg
atatttctacatcttggaagagctatatcttatggttcatatttttataatccaaatact
tggttttctggaattattattatcttcttcttaatgggaacagcatttatgggttatgtg
ttacctttaggacaaatgagtttatggggggttacagtaattacaaatttattatctgca
tttccatctttaatagaatggctttgtggaggacattacatttacaatcctacatttaag
aggttctttgtctttcattttctatttccatttcttctttgtggttttcttgtttatcat
atttttaatctacattttctatcttctaataatcctttaaggaattccactaataataaa
atagcatttttccctttcattattagtaaagatttatatggaaagatattaattctctat
ctatatcttcttcaaattcatttcggtttctcttctttctcacatccagataatgcatta
gaagcatgtggattacttactcctttacatatagtacctgaatggtatttcttatgccaa
tatgctatgttaaaagctgtgccaaataaaaatgcaggattcattatattatttacttct
atcttcatcttattttactttatgagaagtctttcaatctcattctattttatcgtgtgg
gtaagctctagatttaatagtttctttgtaattctttggtttttagttttcatatcctta
atttggataggtggtcaatttcctgtagacaactttctatcttatggtcgtatcttgaca
ttacattattattatcttcttatctgtatcttattatcttag

>KrE12_cob
atgaaatctcatttacaatcatatccttgtcctctgatcataaattatttttggaatctt
ggtttttattagggattactattttattacaaattatatctggaatcttcttaggttta
cattatacatcagatattaattcagcatattttagtattttctttattattagagaaata
tattatggatggtgtttacgttatcttcattctaatggttcatcatttgtctttcttttg
atatttctacatcttggaagagctatatcttatggttcatatttttataatccaaatact
tggttttctggaattattattatcttcttcttaatgggaacagcatttatgggttatgtg
ttacctttaggacaaatgagtttatggggggttacagtaattacaaatttattatctgca
tttccatctttaatagaatggctttgtggaggacattacatttacaatcctacatttaag
aggttctttgtctttcattttctatttccatttcttctttgtggttttcttgtttatcat
atttttaatctacattttctatcttctaataatcctttaaggaattccactaataataaa
atagcatttttccctttcattattagtaaagatttatatggaaagatattaattctctat
ctatatcttcttcaaattcatttcggtttctcttctttctcacatccagataatgcatta
gaagcatgtggattacttactcctttacatatagtacctgaatggtatttcttatgccaa
tatgctatgttaaaagctgtgccaaataaaaatgcaggattcattatattatttacttct
atcttcatcttattttactttatgagaagtctttcaatctcattctattttatcgtgtgg
gtaagctctagatttaatagtttctttgtaattctttggtttttagttttcatatcctta
atttggataggtggtcaatttcctgtagacaactttctatcttatggtcgtatcttgaca
ttacattattattatcttcttatctgtatcttattatcttag
>KrE13_cob
atgaaatctcatttacaatcatatccttgtcctctgatcataaattatttttggaatctt
ggtttttattagggattactattttattacaaattatatctggaatcttcttaggttta
cattatacatcagatattaattcagcatattttagtattttctttattattagagaaata
tattatggatggtgtttacgttatcttcattctaatggttcatcatttgtctttcttttg
atatttctacatcttggaagagctatatcttatggttcatatttttataatccaaatact
tggttttctggaattattattatcttcttcttaatgggaacagcatttatgggttatgtg
ttacctttaggacaaatgagtttatggggggttacagtaattacaaatttattatctgca
tttccatctttaatagaatggctttgtggaggacattacatttacaatcctacatttaag
aggttctttgtctttcattttctatttccatttcttctttgtggttttcttgtttatcat
atttttaatctacattttctatcttctaataatcctttaaggaattccactaataataaa
atagcatttttccctttcattattagtaaagatttatatggaaagatattaattctctat
ctatatcttcttcaaattcatttcggtttctcttctttctcacatccagataatgcatta
gaagcatgtggattacttactcctttacatatagtacctgaatggtatttcttatgccaa
tatgctatgttaaaagctgtgccaaataaaaatgcaggattcattatattatttacttct
atcttcatcttattttactttatgagaagtctttcaatctcattctattttatcgtgtgg
gtaagctctagatttaatagtttctttgtaattctttggtttttagttttcatatcctta
atttggataggtggtcaatttcctgtagacaactttctatcttatggtcgtatcttgaca
ttacattattattatcttcttatctgtatcttattatcttag
>KrE14_cob
atgaaatctcatttacaatcatatccttgtcctctgatcataaattatttttggaatctt
ggtttttattagggattactattttattacaaattatatctggaatcttcttaggttta
cattatacatcagatattaattcagcatattttagtattttctttattattagagaaata
tattatggatggtgtttacgttatcttcattctaatggttcatcatttgtctttcttttg
atatttctacatcttggaagagctatatcttatggttcatatttttataatccaaatact
tggttttctggaattattattatcttcttcttaatgggaacagcatttatgggttatgtg
ttacctttaggacaaatgagtttatggggggttacagtaattacaaatttattatctgca
tttccatctttaatagaatggctttgtggaggacattacatttacaatcctacatttaag
aggttctttgtctttcattttctatttccatttcttctttgtggttttcttgtttatcat
atttttaatctacattttctatcttctaataatcctttaaggaattccactaataataaa
atagcatttttccctttcattattagtaaagatttatatggaaagatattaattctctat
ctatatcttcttcaaattcatttcggtttctcttctttctcacatccagataatgcatta
gaagcatgtggattacttactcctttacatatagtacctgaatggtatttcttatgccaa
tatgctatgttaaaagctgtgccaaataaaaatgcaggattcattatattatttacttct
atctttgtattattttattttatgagaagtctttcagtatctttctattttatcgtgtgg
gtaagctctagatttaatagtttctttgtaattctttggtttttagttttcatatcctta
atttggataggtggtcaatttcctgtagacaactttctatcttatggtcgtatcttgaca
ttacattattattatcttcttatctgtatcttattatcttag

>KrE15_cob
atgaaatctcatttacaatcatatccttgtcctctgatcataaattatttttggaatctt
ggtttttattagggattactattttattacaaattatatctggaatcttcttaggttta
cattatacatcagatattaattcagcatattttagtattttctttattattagagaaata
tattatggatggtgtttacgttatcttcattctaatggttcatcatttgtctttctttg
atatttctacatcttggaagagctatatcttatggttcatatttttataatccaaatact
tggttttctggaattattattatcttcttcttaatgggaacagcatttatgggttatgtg
ttacctttaggacaaatgagtttatggggggttacagtaattacaaatttattatctgca
tttccatctttaatagaatggctttgtggaggacattacatttacaatcctacatttaag
aggttctttgtctttcattttctatttccatttcttctttgtggttttcttgtttatcat
atttttaatctacattttctatcttctaataatcctttaaggaattccactaataataaa
atagcatttttccctttcattattagtaaagatttatatggaaagatattaattctctat
ctatatcttcttcaaattcatttcggtttctcttctttctcacatccagataatgcatta
gaagcatgtggattacttactcctttacatatagtacctgaatggtatttcttatgccaa
tatgctatgttaaaagctgtgccaaataaaaatgcaggattcattatattatttacttct
atcttcatcttattttactttatgagaagtctttcagtatctttctattttatcgtgtgg
gtaagctctagatttaatagtttctttgtaattctttggtttttagttttcatatcctta
atttggataggtggtcaatttcctgtagacaactttctatcttatggtcgtatcttgaca
ttacattattattatcttcttatctgtatcttattatcttag
>KrE2_cob
atgaaatctcatttacaatcatatccttgtcctctgatcataaattatttttggaatctt
ggtttttattagggattactattttattacaaattatatctggaatcttcttaggttta
cattatacatcagatattaattcagcatattttagtattttctttattattagagaaata
tattatggatggtgtttacgttatcttcattctaatggttcatcatttgtctttctttg
atatttctacatcttggaagagctatatcttatggttcatatttttataatccaaatact
tggttttctggaattattattatcttcttcttaatgggaacagcatttatgggttatgtg
ttacctttaggacaaatgagtttatggggggttacagtaattacaaatttattatctgca
tttccatctttaatagaatggctttgtggaggacattacatttacaatcctacatttaag
aggttctttgtctttcattttctatttccatttcttctttgtggttttcttgtttatcat
atttttaatctacattttctatcttctaataatcctttaaggaattccactaataataaa
atagcatttttccctttcattattagtaaagatttatatggaaagatattaattctctat
ctatatcttcttcaaattcatttcggtttctcttctttctcacatccagataatgcatta
gaagcatgtggattacttactcctttacatatagtacctgaatggtatttcttatgccaa
tatgctatgttaaaagctgtgccaaataaaaatgcaggattcattatattatttacttct
atcttcatcttattttactttatgagaagtctttcaatctcattctattttatcgtgtgg
gtaagctctagatttaatagtttctttgtaattctttggtttttagttttcatatcctta
atttggataggtggtcaatttcctgtagacaactttctatcttatggtcgtatcttgaca
ttacattattattatcttcttatctgtatcttattatcttag
>KrE3_cob
atgaaatctcatttacaatcatatccttgtcctctgatcataaattatttttggaatctt
ggtttttattagggattactattttattacaaattatatctggaatcttcttaggttta
cattatacatcagatattaattcagcatattttagtattttctttattattagagaaata
tattatggatggtgtttacgttatcttcattctaatggttcatcatttgtctttctttg
atatttctacatcttggaagagctatatcttatggttcatatttttataatccaaatact
tggttttctggaattattattatcttcttcttaatgggaacagcatttatgggttatgtg
ttacctttaggacaaatgagtttatggggggttacagtaattacaaatttattatctgca
tttccatctttaatagaatggctttgtggaggacattacatttacaatcctacatttaag
aggttctttgtctttcattttctatttccatttcttctttgtggttttcttgtttatcat
atttttaatctacattttctatcttctaataatcctttaaggaattccactaataataaa
atagcatttttccctttcattattagtaaagatttatatggaaagatattaattctctat
ctatatcttcttcaaattcatttcggtttctcttctttctcacatccagataatgcatta
gaagcatgtggattacttactcctttacatatagtacctgaatggtatttcttatgccaa
tatgctatgttaaaagctgtgccaaataaaaatgcaggattcattatattatttacttct
atcttcatcttattttactttatgagaagtctttcaatctcattctattttatcgtgtgg
gtaagctctagatttaatagtttctttgtaattctttggtttttagttttcatatcctta
atttggataggtggtcaatttcctgtagacaactttctatcttatggtcgtatcttgaca
ttacattattattatcttcttatctgtatcttattatcttag

>KrE4_cob
atgaaatctcatttacaatcatatccttgtcctctgatcataaattatttttggaatctt
ggtttttattagggattactattttattacaaattatatctggaatcttcttaggttta
cattatacatcagatattaattcagcatattttagtattttctttattattagagaaata
tattatggatggtgtttacgttatcttcattctaatggttcatcatttgtctttctttg
atatttctacatcttggaagagctatatcttatggttcatatttttataatccaaatact
tggttttctggaattattattatcttcttcttaatgggaacagcatttatgggttatgtg
ttacctttaggacaaatgagtttatggggggttacagtaattacaaatttattatctgca
tttccatctttaatagaatggctttgtggaggacattacatttacaatcctacatttaag
aggttctttgtctttcattttctatttccatttcttctttgtggttttcttgtttatcat
atttttaatctacattttctatcttctaataatcctttaaggaattccactaataataaa
atagcatttttccctttcattattagtaaagatttatatggaaagatattaattctctat
ctatatcttcttcaaattcatttcggtttctcttctttctcacatccagataatgcatta
gaagcatgtggattacttactcctttacatatagtacctgaatggtatttcttatgccaa
tatgctatgttaaaagctgtgccaaataaaaatgcaggattcattatattatttacttct
atcttcatcttattttactttatgagaagtctttcaatctcattctattttatcgtgtgg
gtaagctctagatttaatagtttctttgtaattctttggtttttagttttcatatcctta
atttggataggtggtcaatttcctgtagacaactttctatcttatggtcgtatcttgaca
ttacattattattatcttcttatctgtatcttattatcttag
>KrE5_cob
atgaaatctcatttacaatcatatccttgtcctctgatcataaattatttttggaatctt
ggtttttattagggattactattttattacaaattatatctggaatcttcttaggttta
cattatacatcagatattaattcagcatattttagtattttctttattattagagaaata
tattatggatggtgtttacgttatcttcattctaatggttcatcatttgtctttctttg
atatttctacatcttggaagagctatatcttatggttcatatttttataatccaaatact
tggttttctggaattattattatcttcttcttaatgggaacagcatttatgggttatgtg
ttacctttaggacaaatgagtttatggggggttacagtaattacaaatttattatctgca
tttccatctttaatagaatggctttgtggaggacattacatttacaatcctacatttaag
aggttctttgtctttcattttctatttccatttcttctttgtggttttcttgtttatcat
atttttaatctacattttctatcttctaataatcctttaaggaattccactaataataaa
atagcatttttccctttcattattagtaaagatttatatggaaagatattaattctctat
ctatatcttcttcaaattcatttcggtttctcttctttctcacatccagataatgcatta
gaagcatgtggattacttactcctttacatatagtacctgaatggtatttcttatgccaa
tatgctatgttaaaagctgtgccaaataaaaatgcaggattcattatattatttacttct
atcttcatcttattttactttatgagaagtctttcaatctcattctattttatcgtgtgg
gtaagctctagatttaatagtttctttgtaattctttggtttttagttttcatatcctta
atttggataggtggtcaatttcctgtagacaactttctatcttatggtcgtatcttgaca
ttacattattattatcttcttatctgtatcttattatcttag
>KrE6_cob
atgaaatctcatttacaatcatatccttgtcctctgatcataaattatttttggaatctt
ggtttttattagggattactattttattacaaattatatctggaatcttcttaggttta
cattatacatcagatattaattcagcatattttagtattttctttattattagagaaata
tattatggatggtgtttacgttatcttcattctaatggttcatcatttgtctttctttg
atatttctacatcttggaagagctatatcttatggttcatatttttataatccaaatact
tggttttctggaattattattatcttcttcttaatgggaacagcatttatgggttatgtg
ttacctttaggacaaatgagtttatggggggttacagtaattacaaatttattatctgca
tttccatctttaatagaatggctttgtggaggacattacatttacaatcctacatttaag
aggttctttgtctttcattttctatttccatttcttctttgtggttttcttgtttatcat
atttttaatctacattttctatcttctaataatcctttaaggaattccactaataataaa
atagcatttttccctttcattattagtaaagatttatatggaaagatattaattctctat
ctatatcttcttcaaattcatttcggtttctcttctttctcacatccagataatgcatta
gaagcatgtggattacttactcctttacatatagtacctgaatggtatttcttatgccaa
tatgctatgttaaaagctgtgccaaataaaaatgcaggattcattatattatttacttct
atcttcatcttattttactttatgagaagtctttcaatctcattctattttatcgtgtgg
gtaagctctagatttaatagtttctttgtaattctttggtttttagttttcatatcctta
atttggataggtggtcaatttcctgtagacaactttctatcttatggtcgtatcttgaca
ttacattattattatcttcttatctgtatcttattatcttag

>KrE7_cob
atgaaatctcatttacaatcatatccttgtcctctgatcataaattatttttggaatctt
ggtttttattagggattactattttattacaaattatatctggaatcttcttaggttta
cattatacatcagatattaattcagcatattttagtattttctttattattagagaaata
tattatggatggtgtttacgttatcttcattctaatggttcatcatttgtctttcttttg
atatttctacatcttggaagagctatatcttatggttcatatttttataatccaaatact
tggttttctggaattattattatcttcttcttaatgggaacagcatttatgggttatgtg
ttacctttaggacaaatgagtttatggggggttacagtaattacaaatttattatctgca
tttccatctttaatagaatggctttgtggaggacattacatttacaatcctacatttaag
aggttctttgtctttcattttctatttccatttcttctttgtggttttcttgtttatcat
atttttaatctacattttctatcttctaataatcctttaaggaattccactaataataaa
atagcatttttccctttcattattagtaaagatttatatggaaagatattaattctctat
ctatatcttcttcaaattcatttcggtttctcttctttctcacatccagataatgcatta
gaagcatgtggattacttactcctttacatatagtacctgaatggtatttcttatgccaa
tatgctatgttaaaagctgtgccaaataaaaatgcaggattcattatattatttacttct
atcttcatcttattttactttatgagaagtctttcaatctcattctattttatcgtgtgg
gtaagctctagatttaatagtttctttgtaattctttggtttttagttttcatatcctta
atttggataggtggtcaatttcctgtagacaactttctatcttatggtcgtatcttgaca
ttacattattattatcttcttatctgtatcttattatcttag
>KrE8_cob
atgaaatctcatttacaatcatatccttgtcctctgatcataaattatttttggaatctt
ggtttttattagggattactattttattacaaattatatctggaatcttcttaggttta
cattatacatcagatattaattcagcatattttagtattttctttattattagagaaata
tattatggatggtgtttacgttatcttcattctaatggttcatcatttgtctttcttttg
atatttctacatcttggaagagctatatcttatggttcatatttttataatccaaatact
tggttttctggaattattattatcttcttcttaatgggaacagcatttatgggttatgtg
ttacctttaggacaaatgagtttatggggggttacagtaattacaaatttattatctgca
tttccatctttaatagaatggctttgtggaggacattacatttacaatcctacatttaag
aggttctttgtctttcattttctatttccatttcttctttgtggttttcttgtttatcat
atttttaatctacattttctatcttctaataatcctttaaggaattccactaataataaa
atagcatttttccctttcattattagtaaagatttatatggaaagatattaattctctat
ctatatcttcttcaaattcatttcggtttctcttctttctcacatccagataatgcatta
gaagcatgtggattacttactcctttacatatagtacctgaatggtatttcttatgccaa
tatgctatgttaaaagctgtgccaaataaaaatgcaggattcattatattatttacttct
atcttcatcttattttactttatgagaagtctttcaatctcattctattttatcgtgtgg
gtaagctctagatttaatagtttctttgtaattctttggtttttagttttcatatcctta
atttggataggtggtcaatttcctgtagacaactttctatcttatggtcgtatcttgaca
ttacattattattatcttcttatctgtatcttattatcttag
>KrE9_cob
atgaaatctcatttacaatcatatccttgtcctctgatcataaattatttttggaatctt
ggtttttattagggattactattttattacaaattatatctggaatcttcttaggttta
cattatacatcagatattaattcagcatattttagtattttctttattattagagaaata
tattatggatggtgtttacgttatcttcattctaatggttcatcatttgtctttcttttg
atatttctacatcttggaagagctatatcttatggttcatatttttataatccaaatact
tggttttctggaattattattgttttcttcttaatgggaacagcatttatgggttatgtg
ttacctttaggacaaatgagtttatggggggttacagtaattacaaatttattatctgca
tttccatctttaatagaatggctttgtggaggacattacatttacaatcctacatttaag
aggttctttgtctttcattttctatttccatttcttctttgtggttttcttgtttatcat
atttttaatctacattttctatcttctaataatcctttaaggaattccactaataataaa
atagcatttttccctttcattattagtaaagatttatatggaaagatattaattctctat
ctatatcttcttcaaattcatttcggtttctcttctttctcacatccagataatgcatta
gaagcatgtggattacttactcctttacatatagtacctgaatggtatttcttatgccaa
tatgctatgttaaaagctgtgccaaataaaaatgcaggattcattatattatttacttct
atcttcatcttattttactttatgagaagtctttcagtatctttctattttatcgtgtgg
gtaagctctagatttaatagtttctttgtaattctttggtttttagttttcatatcctta
atttggataggtggtcaatttcctgtagacaactttctatcttatggtcgtatcttgaca
ttacattattattatcttcttatctgtatcttattatcttag

>Mz10_cob
atgaaatctcatttacaatcatatccttgtcctctgatcataaattatttttggaatctt
ggtttttattagggattactattttattacaaattatatctggaatcttcttaggttta
cattatacatcagatattaattcagcatattttagtattttctttattattagagaaata
tattatggatggtgtttacgttatcttcattctaatggttcatcatttgtctttcttttg
atatttctacatcttggaagagctatatcttatggttcatatttttataatccaaatact
tggttttctggaattattattatcttcttcttaatgggaacagcatttatgggttatgtg
ttacctttaggacaaatgagtttatggggggttacagtaattacaaatttattatctgca
tttccatctttaatagaatggctttgtggaggacattacatttacaatcctacatttaag
aggttctttgtctttcattttctatttccatttcttctttgtggttttcttgtttatcat
atttttaatctacattttctatcttctaataatcctttaaggaattccactaataataaa
atagcatttttccctttcattattagtaaagatttatatggaaagatattaattctctat
ctatatcttcttcaaattcatttcggtttctcttctttctcacatccagataatgcatta
gaagcatgtggattacttactcctttacatatagtacctgaatggtatttcttatgccaa
tatgctatgttaaaagctgtgccaaataaaaatgcaggattcattatattatttacttct
atcttcatcttattttactttatgagaagtctttcaatctcattctattttatcgtgtgg
gtaagctctagatttaatagtttctttgtaattctttggtttttagttttcatatcctta
atttggataggtggtcaatttcctgtagacaactttctatcttatggtcgtatcttgaca
ttacattattattatcttcttatctgtatcttattatcttag
>Mz5_cob
atgaaatctcatttacaatcatatccttgtcctctgatcataaattatttttggaatctt
ggtttttattagggattactattttattacaaattatatctggaatcttcttaggttta
cattatacatcagatattaattcagcatattttagtattttctttattattagagaaata
tattatggatggtgtttacgttatcttcattctaatggttcatcatttgtctttcttttg
atatttctacatcttggaagagctatatcttatggttcatatttttataatccaaatact
tggttttctggaattattattatcttcttcttaatgggaacagcatttatgggttatgtg
ttacctttaggacaaatgagtttatggggggttacagtaattacaaatttattatctgca
tttccatctttaatagaatggctttgtggaggacattacatttacaatcctacatttaag
aggttctttgtctttcattttctatttccatttcttctttgtggttttcttgtttatcat
atttttaatctacattttctatcttctaataatcctttaaggaattccactaataataaa
atagcatttttccctttcattattagtaaagatttatatggaaagatattaattctctat
ctatatcttcttcaaattcatttcggtttctcttctttctcacatccagataatgcatta
gaagcatgtggattacttactcctttacatatagtacctgaatggtatttcttatgccaa
tatgctatgttaaaagctgtgccaaataaaaatgcaggattcattatattatttacttct
atcttcatcttattttactttatgagaagtctttcaatctcattctattttatcgtgtgg
gtaagctctagatttaatagtttctttgtaattctttggtttttagttttcatatcctta
atttggataggtggtcaatttcctgtagacaactttctatcttatggtcgtatcttgaca
ttacattattattatcttcttatctgtatcttattatcttag
>Mz6_cob
atgaaatctcatttacaatcatatccttgtcctctgatcataaattatttttggaatctt
ggtttttattagggattactattttattacaaattatatctggaatcttcttaggttta
cattatacatcagatattaattcagcatattttagtattttctttattattagagaaata
tattatggatggtgtttacgttatcttcattctaatggttcatcatttgtctttcttttg
atatttctacatcttggaagagctatatcttatggttcatatttttataatccaaatact
tggttttctggaattattattatcttcttcttaatgggaacagcatttatgggttatgtg
ttacctttaggacaaatgagtttatggggggttacagtaattacaaatttattatctgca
tttccatctttaatagaatggctttgtggaggacattacatttacaatcctacatttaag
aggttctttgtctttcattttctatttccatttcttctttgtggttttcttgtttatcat
atttttaatctacattttctatcttctaataatcctttaaggaattccactaataataaa
atagcatttttccctttcattattagtaaagatttatatggaaagatattaattctctat
ctatatcttcttcaaattcatttcggtttctcttctttctcacatccagataatgcatta
gaagcatgtggattacttactcctttacatatagtacctgaatggtatttcttatgccaa
tatgctatgttaaaagctgtgccaaataaaaatgcaggattcattatattatttacttct
atcttcatcttattttactttatgagaagtctttcaatctcattctattttatcgtgtgg
gtaagctctagatttaatagtttctttgtaattctttggtttttagttttcatatcctta
atttggataggtggtcaatttcctgtagacaactttctatcttatggtcgtatcttgaca
ttacattattattatcttcttatctgtatcttattatcttag

>Mz7_cob
atgaaatctcatttacaatcatatccttgtcctctgatcataaattatttttggaatctt
ggtttttattagggattactattttattacaaattatatctggaatcttcttaggttta
cattatacatcagatattaattcagcatattttagtattttctttattattagagaaata
tattatggatggtgtttacgttatcttcattctaatggttcatcatttgtctttcttttg
atatttctacatcttggaagagctatatcttatggttcatatttttataatccaaatact
tggttttctggaattattattatcttcttcttaatgggaacagcatttatgggttatgtg
ttacctttaggacaaatgagtttatggggggttacagtaattacaaatttattatctgca
tttccatctttaatagaatggctttgtggaggacattacatttacaatcctacatttaag
aggttctttgtctttcattttctatttccatttcttctttgtggttttcttgtttatcat
atttttaatctacattttctatcttctaataatcctttaaggaattccactaataataaa
atagcatttttccctttcattattagtaaagatttatatggaaagatattaattctctat
ctatatcttcttcaaattcatttcggtttctcttctttctcacatccagataatgcatta
gaagcatgtggattacttactcctttacatatagtacctgaatggtatttcttatgccaa
tatgctatgttaaaagctgtgccaaataaaaatgcaggattcattatattatttacttct
atcttcatcttattttactttatgagaagtctttcaatctctttctattttatcgtgtgg
gtaagctctagatttaatagtttctttgtaattctttggtttttagttttcatatcctta
atttggataggtggtcaatttcctgtagacaactttctatcttatggtcgtatcttgaca
ttacattattattatcttcttatctgtatcttattatcttag
>Mz8_cob
atgaaatctcatttacaatcatatccttgtcctctgatcataaattatttttggaatctt
ggtttttattagggattactattttattacaaattatatctggaatcttcttaggttta
cattatacatcagatattaattcagcatattttagtattttctttattattagagaaata
tattatggatggtgtttacgttatcttcattctaatggttcatcatttgtctttcttttg
atatttctacatcttggaagagctatatcttatggttcatatttttataatccaaatact
tggttttctggaattattattatcttcttcttaatgggaacagcatttatgggttatgtg
ttacctttaggacaaatgagtttatggggggttacagtaattacaaatttattatctgca
tttccatctttaatagaatggctttgtggaggacattacatttacaatcctacatttaag
aggttctttgtctttcattttctatttccatttcttctttgtggttttcttgtttatcat
atttttaatctacattttctatcttctaataatcctttaaggaattccactaataataaa
atagcatttttccctttcattattagtaaagatttatatggaaagatattaattctctat
ctatatcttcttcaaattcatttcggtttctcttctttctcacatccagataatgcatta
gaagcatgtggattacttactcctttacatatagtacctgaatggtatttcttatgccaa
tatgctatgttaaaagctgtgccaaataaaaatgcaggattcattatattatttacttct
atcttcatcttattttactttatgagaagtctttcaatctcattctattttatcgtgtgg
gtaagctctagatttaatagtttctttgtaattctttggtttttagttttcatatcctta
atttggataggtggtcaatttcctgtagacaactttctatcttatggtcgtatcttgaca
ttacattattattatcttcttatctgtatcttattatcttag
>Mz9_cob
atgaaatctcatttacaatcatatccttgtcctctgatcataaattatttttggaatctt
ggtttttattagggattactattttattacaaattatatctggaatcttcttaggttta
cattatacatcagatattaattcagcatattttagtattttctttattattagagaaata
tattatggatggtgtttacgttatcttcattctaatggttcatcatttgtctttcttttg
atatttctacatcttggaagagctatatcttatggttcatatttttataatccaaatact
tggttttctggaattattattatcttcttcttaatgggaacagcatttatgggttatgtg
ttacctttaggacaaatgagtttatggggggttacagtaattacaaatttattatctgca
tttccatctttaatagaatggctttgtggaggacattacatttacaatcctacatttaag
aggttctttgtctttcattttctatttccatttcttctttgtggttttcttgtttatcat
atttttaatctacattttctatcttctaataatcctttaaggaattccactaataataaa
atagcatttttccctttcattattagtaaagatttatatggaaagatattaattctctat
ctatatcttcttcaaattcatttcggtttctcttctttctcacatccagataatgcatta
gaagcatgtggattacttactcctttacatatagtacctgaatggtatttcttatgccaa
tatgctatgttaaaagctgtgccaaataaaaatgcaggattcattatattatttacttct
atcttcatcttattttactttatgagaagtctttcaatctcattctattttatcgtgtgg
gtaagctctagatttaatagtttctttgtaattctttggtttttagttttcatatcctta
atttggataggtggtcaatttcctgtagacaactttctatcttatggtcgtatcttgaca
ttacattattattatcttcttatctgtatcttattatcttag

>MzC1_cob
atgaaatctcatttacaatcatatccttgtcctctgatcataaattatttttggaatctt
ggtttttattagggattactattttattacaaattatatctggaatcttcttaggttta
cattatacatcagatattaattcagcatattttagtattttctttattattagagaaata
tattatggatggtgtttacgttatcttcattctaatggttcatcatttgtctttcttttg
atatttctacatcttggaagagctatatcttatggttcatatttttataatccaaatact
tggttttctggaattattattatcttcttcttaatgggaacagcatttatgggttatgtg
ttacctttaggacaaatgagtttatggggggttacagtaattacaaatttattatctgca
tttccatctttaatagaatggctttgtggaggacattacatttacaatcctacatttaag
aggttctttgtctttcattttctatttccatttcttctttgtggttttcttgtttatcat
atttttaatctacattttctatcttctaataatcctttaaggaattccactaataataaa
atagcatttttccctttcattattagtaaagatttatatggaaagatattaattctctat
ctatatcttcttcaaattcatttcggtttctcttctttctcacatccagataatgcatta
gaagcatgtggattacttactcctttacatatagtacctgaatggtatttcttatgccaa
tatgctatgttaaaagctgtgccaaataaaaatgcaggattcattatattatttacttct
atcttcatcttattttactttatgagaagtctttcaatctcattctattttatcgtgtgg
gtaagctctagatttaatagtttctttgtaattctttggtttttagttttcatatcctta
atttggataggtggtcaatttcctgtagacaactttctatcttatggtcgtatcttgaca
ttacattattattatcttcttatctgtatcttattatcttag
>MzC1GaII_cob
atgaaatctcatttacaatcatatccttgtcctctgatcataaattatttttggaatctt
ggtttttattagggattactattttattacaaattatatctggaatcttcttaggttta
cattatacatcagatattaattcagcatattttagtattttctttattattagagaaata
tattatggatggtgtttacgttatcttcattctaatggttcatcatttgtctttcttttg
atatttctacatcttggaagagctatatcttatggttcatatttttataatccaaatact
tggttttctggaattattattatcttcttcttaatgggaacagcatttatgggttatgtg
ttacctttaggacaaatgagtttatggggggttacagtaattacaaatttattatctgca
tttccatctttaatagaatggctttgtggaggacattacatttacaatcctacatttaag
aggttctttgtctttcattttctatttccatttcttctttgtggttttcttgtttatcat
atttttaatctacattttctatcttctaataatcctttaaggaattccactaataataaa
atagcatttttccctttcattattagtaaagatttatatggaaagatattaattctctat
ctatatcttcttcaaattcatttcggtttctcttctttctcacatccagataatgcatta
gaagcatgtggattacttactcctttacatatagtacctgaatggtatttcttatgccaa
tatgctatgttaaaagctgtgccaaataaaaatgcaggattcattatattatttacttct
atcttcatcttattttactttatgagaagtctttcaatctcattctattttatcgtgtgg
gtaagctctagatttaatagtttctttgtaattctttggtttttagttttcatatccttt
atttggataggtggtcaatttcctgtagacaactttctatcttatggtcgtatcttgaca
ttacattattattatcttcttatctgtatcttattatcttag
>MzC2_cob
atgaaatctcatttacaatcatatccttgtcctctgatcataaattatttttggaatctt
ggtttttattagggattactattttattacaaattatatctggaatcttcttaggttta
cattatacatcagatattaattcagcatattttagtattttctttattattagagaaata
tattatggatggtgtttacgttatcttcattctaatggttcatcatttgtctttcttttg
atatttctacatcttggaagagctatatcttatggttcatatttttataatccaaatact
tggttttctggaattattattatcttcttcttaatgggaacagcatttatgggttatgtg
ttacctttaggacaaatgagtttatggggggttacagtaattacaaatttattatctgca
tttccatctttaatagaatggctttgtggaggacattacatttacaatcctacatttaag
aggttctttgtctttcattttctatttccatttcttctttgtggttttcttgtttatcat
atttttaatctacattttctatcttctaataatcctttaaggaattccactaataataaa
atagcatttttccctttcattattagtaaagatttatatggaaagatattaattctctat
ctatatcttcttcaaattcatttcggtttctcttctttctcacatccagataatgcatta
gaagcatgtggattacttactcctttacatatagtacctgaatggtatttcttatgccaa
tatgctatgttaaaagctgtgccaaataaaaatgcaggattcattatattatttacttct
atcttcatcttattttactttatgagaagtctttcaatctcattctattttatcgtgtgg
gtaagctctagatttaatagtttctttgtaattctttggtttttagttttcatatcctta
atttggataggtggtcaatttcctgtagacaactttctatcttatggtcgtatcttgaca
ttacattattattatcttcttatctgtatcttattctcttag

>MzC2GaII_cob
atgaaatctcatttacaatcatatccttgtcctctgatcataaattatttttggaatctt
ggtttttattagggattactattttattacaaattatatctggaatcttcttaggttta
cattatacatcagatattaattcagcatattttagtattttctttattattagagaaata
tattatggatggtgtttacgttatcttcattctaatggttcatcatttgtctttcttttg
atatttctacatcttggaagagctatatcttatggttcatatttttataatccaaatact
tggttttctggaattattattatcttcttcttaatgggaacagcatttatgggttatgtg
ttacctttaggacaaatgagtttatgggggggttacagtaattacaaatttattatctgca
tttccatctttaatagaatggctttgtggaggacattacatttacaatcctacatttaag
aggttctttgtctttcattttctatttccatttcttctttgtggttttcttgtttatcat
atttttaatctacattttctatcttctaataatcctttaaggaattccactaataataaa
atagcatttttccctttcattattagtaaagatttatatggaaagatattaattctctat
ctatatcttcttcaaattcatttcggtttctcttctttctcacatccagataatgcatta
gaagcatgtggattacttactcctttacatatagtacctgaatggtatttcttatgccaa
tatgctatgttaaaagctgtgccaaataaaaatgcaggattcattatattatttacttct
atcttcatcttattttactttatgagaagtctttcaatctcattctattttatcgtgtgg
gtaagctctagatttaatagtttctttgtaattctttggtttttagttttcatatcctta
atttggataggtggtcaatttcctgtagacaactttctatcttatggtcgtatcttgaca
ttacattattattatcttcttatctgtatcttattatcttag
>MzC3_cob
atgaaatctcatttacaatcatatccttgtcctctgatcataaattatttttggaatctt
ggtttttattagggattactattttattacaaattatatctggaatcttcttaggttta
cattatacatcagatattaattcagcatattttagtattttctttattattagagaaata
tattatggatggtgtttacgttatcttcattctaatggttcatcatttgtctttcttttg
atatttctacatcttggaagagctatatcttatggttcatatttttataatccaaatact
tggttttctggaattattattatcttcttcttaatgggaacagcatttatgggttatgtg
ttacctttaggacaaatgagtttatgggggggttacagtaattacaaatttattatctgca
tttccatctttaatagaatggctttgtggaggacattacatttacaatcctacatttaag
aggttctttgtctttcattttctatttccatttcttctttgtggttttcttgtttatcat
atttttaatctacattttctatcttctaataatcctttaaggaattccactaataataaa
atagcatttttccctttcattattagtaaagatttatatggaaagatattaattctctat
ctatatcttcttcaaattcatttcggtttctcttctttctcacatccagataatgcatta
gaagcatgtggattacttactcctttacatatagtacctgaatggtatttcttatgccaa
tatgctatgttaaaagctgtgccaaataaaaatgcaggattcattatattatttacttct
atcttcatcttattttactttatgagaagtctttcaatctcattctattttatcgtgtgg
gtaagctctagatttaatagtttctttgtaattctttggtttttagttttcatatcctta
atttggataggtggtcaatttcctgtagacaactttctatcttatggtcgtatcttgaca
ttacattattattatcttcttatctgtatcttattatcttag
>MzC4_cob
atgaaatctcatttacaatcatatccttgtcctctgatcataaattatttttggaatctt
ggtttttattagggattactattttattacaaattatatctggaatcttcttaggttta
cattatacatcagatattaattcagcatattttagtattttctttattattagagaaata
tattatggatggtgtttacgttatcttcattctaatggttcatcatttgtctttcttttg
atatttctacatcttggaagagctatatcttatggttcatatttttataatccaaatact
tggttttctggaattattattatcttcttcttaatgggaacagcatttatgggttatgtg
ttacctttaggacaaatgagtttatgggggggttacagtaattacaaatttattatctgca
tttccatctttaatagaatggctttgtggaggacattacatttacaatcctacatttaag
aggttctttgtctttcattttctatttccatttcttctttgtggttttcttgtttatcat
atttttaatctacattttctatcttctaataatcctttaaggaattccactaataataaa
atagcatttttccctttcattattagtaaagatttatatggaaagatattaattctctat
ctatatcttcttcaaattcatttcggtttctcttctttctcacatccagataatgcatta
gaagcatgtggattacttactcctttacatatagtacctgaatggtatttcttatgccaa
tatgctatgttaaaagctgtgccaaataaaaatgcaggattcattatattatttacttct
atcttcatcttattttactttatgagaagtctttcagtatctttctattttatcgtgtgg
gtaagctctagatttaatagtttctttgtaattctttggtttttagttttcatatcctta
atttggataggtggtcaatttcctgtagacaactttctatcttatggtcgtatcttgaca
ttacattattattatcttcttatctgtatcttattatcttag

>Od10_cob
atgaaatctcatttacaatcatatccttgtcctctgatcataaattatttttggaatctt
ggtttttattagggattactattttattacaaattatatctggaatcttcttaggttta
cattatacatcagatattaattcagcatattttagtattttctttattattagagaagta
tattatggatggtgtttacgttattttcattctaatggttcatcatttgtctttcttttg
atatttctacatcttggaagagctatatcttatggttcatatttttataatccaaatact
tggttttctggaattattattatcttcttcttaatgggaacagcatttatgggttatgtg
ttacctttaggacaaatgagtttatgggggggttacagtaattacaaatttattatctgca
tttccatctttaatagaatggctttgtggaggacattacatttacaatcctacatttaag
aggttctttgtctttcattttctatttccatttcttctttgtggttttcttgtttatcat
atttttaatctacattttctatcttctaataatcctttaaggaattccactaataataaa
atagcatttttccctttcattattagtaaagatttatatggaaagatattaattctctat
ctatatcttcttcaaattcatttcggtttctcttctttctcacatccagataatgcatta
gaagcatgtggattacttactcctttacatatagtacctgaatggtatttcttatgccaa
tatgctatgttaaaagctgtgccaaataaaaatgcaggattcattatattatttacttct
atcttcatcttattttactttatgagaagtctttcagtatctttctattttatcgtgtgg
gtaagctctagatttaatagtttctttgtaattctttggtttttagttttcatatcctta
atttggataggtggtcaatttcctgtagacaactttctatcttatggtcgtatcttgaca
ttacattattattatcttcttatctgtatcttattatcttag
>Od11_cob
atgaaatctcatttacaatcatatccttgtcctctgatcataaattatttttggaatctt
ggtttttattagggattactattttattacaaattatatctggaatcttcttaggttta
cattatacatcagatattaattcagcatattttagtattttctttattattagagaaata
tattatggatggtgtttacgttatcttcattctaatggttcatcatttgtctttcttttg
atatttctacatcttggaagagctatatcttatggttcatatttttataatccaaatact
tggttttctggaattattattatcttcttcttaatgggaacagcatttatgggttatgtg
ttacctttaggacaaatgagtttatgggggggttacagtaattacaaatttattatctgca
tttccatctttaatagaatggctttgtggaggacattacatttacaatcctacatttaag
aggttctttgtctttcattttctatttccatttcttctttgtggttttcttgtttatcat
atttttaatctacattttctatcttctaataatcctttaaggaattccactaataataaa
atagcatttttccctttcattattagtaaagatttatatggaaagatattaattctctat
ctatatcttcttcaaattcatttcggtttctcttctttctcacatccagataatgcatta
gaagcatgtggattacttactcctttacatatagtacctgaatggtatttcttatgccaa
tatgctatgttaaaagctgtgccaaataaaaatgcaggattcattatattatttacttct
atcttcatcttattttactttatgagaagtctttcaatctcattctattttatcgtgtgg
gtaagctctagatttaatagtttctttgtaattctttggtttttagttttcatatcctta
atttggataggtggtcaatttcctgtagacaactttctatcttatggtcgtatcttgaca
ttacattattattatcttcttatctgtatcttattatcttag
>Od12_cob
atgaaatctcatttacaatcatatccttgtcctctgatcataaattatttttggaatctt
ggtttttattagggattactattttattacaaattatatctggaatcttcttaggttta
cattatacatcagatattaattcagcatattttagtattttctttattattagagaaata
tattatggatggtgtttacgttatcttcattctaatggttcatcatttgtctttcttttg
atatttctacatcttggaagagctatatcttatggttcatatttttataatccaaatact
tggttttctggaattattattatcttcttcttaatgggaacagcatttatgggttatgtg
ttacctttaggacaaatgagtttatgggggggttacagtaattacaaatttattatctgca
tttccatctttaatagaatggctttgtggaggacattacatttacaatcctacatttaag
aggttctttgtctttcattttctatttccatttcttctttgtggttttcttgtttatcat
atttttaatctacattttctatcttctaataatcctttaaggaattccactaataataaa
atagcatttttccctttcattattagtaaagatttatatggaaagatattaattctctat
ctatatcttcttcaaattcatttcggtttctcttctttctcacatccagataatgcatta
gaagcatgtggattacttactcctttacatatagtacctgaatggtatttcttatgccaa
tatgctatgttaaaagctgtgccaaataaaaatgcaggattcattatattatttacttct
atcttcatcttattttactttatgagaagtctttcaatctcattctattttatcgtgtgg
gtaagctctagatttaatagtttctttgtaattctttggtttttagttttcatatcctta
atttggataggtggtcaatttcctgtagacaactttctatcttatggtcgtatcttgaca
ttacattattattatcttcttatctgtatcttattatcttag

>Od13_cob
atgaaatctcatttacaatcatatccttgtcctctgatcataaattatttttggaatctt
ggtttttattagggattactattttattacaaattatatctggaatcttcttaggttta
cattatacatcagatattaattcagcatattttagtattttctttattattagagaaata
tattatggatggtgtttacgttatcttcattctaatggttcatcatttgtctttcttttg
atatttctacatcttggaagagctatatcttatggttcatatttttataatccaaatact
tggttttctggaattattattatcttcttcttaatgggaacagcatttatgggttatgtg
ttacctttaggacaaatgagtttatggggggttacagtaattacaaatttattatctgca
tttccatctttaatagaatggctttgtggaggacattacatttacaatcctacatttaag
aggttctttgtctttcattttctatttccatttcttctttgtggttttcttgtttatcat
atttttaatctacattttctatcttctaataatcctttaaggaattccactaataataaa
atagcatttttccctttcattattagtaaagatttatatggaaagatattaattctctat
ctatatcttcttcaaattcatttcggtttctcttctttctcacatccagataatgcatta
gaagcatgtggattacttactcctttacatatagtacctgaatggtatttcttatgccaa
tatgctatgttaaaagctgtgccaaataaaaatgcaggattcattatattatttacttct
atcttcatcttattttactttatgagaagtctttcaatctcattctattttatcgtgtgg
gtaagctctagatttaatagtttctttgtaattctttggtttttagttttcatatccttt
atttggataggtggtcaatttcctgtagacaactttctatcttatggtcgtatcttgaca
ttacattattattatcttcttatctgtatcttattatcttag
>Od14_cob
atgaaatctcatttacaatcatatccttgtcctctgatcataaattatttttggaatctt
ggtttttattaggggttgctattttattacaaattatatctggaatcttcttaggttta
cattatacatcagatattaattcagcatattttagtattttctttattattagagaaata
tattatggatggtgtttacgttatcttcattctaatggttcatcatttgtctttcttttg
atatttctacatcttggaagagctatatcttatggttcatatttttataatccaaatact
tggttttctggaattattattatcttcttcttaatgggaacagcatttatgggttatgtg
ttacctttaggacaaatgagtttatggggggttacagtaattacaaatttattatctgca
tttccatctttaatagaatggctttgtggaggacattacatttacaatcctacatttaag
aggttctttgtctttcattttctatttccatttcttctttgtggttttcttgtttatcat
atttttaatctacattttctatcttctaataatcctttaaggaattccactaataataaa
atagcatttttccctttcattattagtaaagatttatatggaaagatattaattctctat
ctatatcttcttcaaattcatttcggtttctcttctttctcacatccagataatgcatta
gaagcatgtggattacttactcctttacatatagtacctgaatggtatttcttatgccaa
tatgctatgttaaaagctgtgccaaataaaaatgcaggattcattatattatttacttct
atcttcatcttattttactttatgagaagtctttcagtatctttctattttatcgtgtgg
gtaagctctagatttaatagtttctttgtaattctttggtttttagttttcatatcctta
atttggataggtggtcaatttcctgtagacaactttctatcttatggtcgtatcttgaca
ttacattattattatcttcttatctgtatcttattatcttag
>Od15_cob
atgaaatctcatttacaatcatatccttgtcctctgatcataaattatttttggaatctt
ggtttttattagggattactattttattacaaattatatctggaatcttcttaggttta
cattatacatcagatattaattcagcatattttagtattttctttattattagagaaata
tattatggatggtgtttacgttatcttcattctaatggttcatcatttgtctttcttttg
atatttctacatcttggaagagctatatcttatggttcatatttttataatccaaatact
tggttttctggaattattattatcttcttcttaatgggaacagcatttatgggttatgtg
ttacctttaggacaaatgagtttatggggggttacagtaattacaaatttattatctgca
tttccatctttaatagaatggctttgtggaggacattacatttacaatcctacatttaag
aggttctttgtctttcattttctatttccatttcttctttgtggttttcttgtttatcat
atttttaatctacattttctatcttctaataatcctttaaggaattccactaataataaa
atagcatttttccctttcattattagtaaagatttatatggaaagatattaattctctat
ctatatcttcttcaaattcatttcggtttctcttctttctcacatccagataatgcatta
gaagcatgtggattacttactcctttacatatagtacctgaatggtatttcttatgccaa
tatgctatgttaaaagctgtgccaaataaaaatgcaggattcattatattatttacttct
atcttcatcttattttactttatgagaagtctttcagtatctttctattttatcgtgtgg
gtaagctctagatttaatagtttctttgtaattctttggtttttagttttcatatcctta
atttggataggtggtcaatttcctgtagacaactttctatcttatggtcgtatcttgaca
ttacattattattatcttcttatctgtatcttattatcttag

>Od18_cob
atgaaatctcatttacaatcatatccttgtcctctgatcataaattatttttggaatctt
ggtttttattagggattactattttattacaaattatatctggaatcttcttaggttta
cattatacatcagatattaattcagcatattttagtattttctttattattagagaaata
tattatggatggtgtttacgttatcttcattctaatggttcatcatttgtctttcttttg
atatttctacatcttggaagagctatatcttatggttcatatttttataatccaaatact
tggttttctggaattattattatcttcttcttaatgggaacagcatttatgggttatgtg
ttacctttaggacaaatgagtttatgggggggttacagtaattacaaatttattatctgca
tttccatctttaatagaatggctttgtggaggacattacatttacaatcctacatttaag
aggttctttgtctttcattttctatttccatttcttctttgtggttttcttgtttatcat
atttttaatctacattttctatcttctaataatcctttaaggaattccactaataataaa
atagcatttttccctttcattattagtaaagatttatatggaaagatattaattctctat
ctatatcttcttcaaattcatttcggtttctcttctttctcacatccagataatgcatta
gaagcatgtggattacttactcctttacacattgtacctgaatggtatttcttatgccaa
tatgctatgttaaaagctgtgccaaataaaaatgcaggattcattatattacttacttct
atctttgtattattttattttatgagaagtctttcagtatctttctattttatcgtgtgg
gtaagctctagatttaatagtttctttgtaattctttggtttttagttttcatatcctta
atttggataggtggtcaatttcctgtagacaactttctatcttatgctcgtatcttgaca
ttatattattattattttcttatctgtatcttattatcttag
>Od19_cob
atgaaatctcatttacaatcatatccttgtcctctgatcataaattatttttggaatctt
ggtttttattagggattactattttattacaaattatatctggaatcttcttaggttta
cattatacatcagatattaattcagcatattttagtattttctttattattagagaaata
tattatggatggtgtttacgttatcttcattctaatggttcatcatttgtctttcttttg
atatttctacatcttggaagagctatatcttatggttcatatttttataatccaaatact
tggttttctggaattattattatcttcttcttaatgggaacagcatttatgggttatgtg
ttacctttaggacaaatgagtttatgggggggttacagtaattacaaatttattatctgca
tttccatctttaatagaatggctttgtggaggacattacatttacaatcctacatttaag
aggttctttgtctttcattttctatttccatttcttctttgtggttttcttgtttatcat
atttttaatctacattttctatcttctaataatcctttaaggaattccactaataataaa
atagcatttttccctttcattattagtaaagatttatatggaaagatattaattctctat
ctatatcttcttcaaattcatttcggtttctcttctttctcacatccagataatgcatta
gaagcatgtggattacttactcctttacatatagtacctgaatggtatttcttatgccaa
tatgctatgttaaaagctgtgccaaataaaaatgcaggattcattatattatttacttct
atcttcatcttattttactttatgagaagtctttcaatctcattctattttatcgtgtgg
gtaagctctagatttaatagtttctttgtaattctttggtttttagttttcatatcctta
atttggataggtggtcaatttcctgtagacaactttctatcttatggtcgtatcttgaca
ttacattattattatcttcttatctgtatcttattatcttag
>Od2_cob
atgaaatctcatttacaatcatatccttgtcctctgatcataaattatttttggaatctt
ggtttttattagggattactattttattacaaattatatctggaatcttcttaggttta
cattatacatcagatattaattcagcatattttagtattttctttattattagagaaata
tattatggatggtgtttacgttatcttcattctaatggttcatcatttgtctttcttttg
atatttctacatcttggaagagctatatcttatggttcatatttttataatccaaatact
tggttttctggaattattattatcttcttcttaatgggaacagcatttatgggttatgtg
ttacctttaggacaaatgagtttatgggggggttacagtaattacaaatttattatctgca
tttccatctttaatagaatggctttgtggaggacattacatttacaatcctacatttaag
aggttctttgtctttcattttctatttccatttcttctttgtggttttcttgtttatcat
atttttaatctacattttctatcttctaataatcctttaaggaattccactaataataaa
atagcatttttccctttcattattagtaaagatttatatggaaagatattaattctctat
ctatatcttcttcaaattcatttcggtttctcttctttctcacatccagataatgcatta
gaagcatgtggattacttactcctttacatatagtacctgaatggtatttcttatgccaa
tatgctatgttaaaagctgtgccaaataaaaatgcaggattcattatattatttacttct
atcttcatcttattttactttatgagaagtctttcaatctcattctattttatcgtgtgg
gtaagctctagatttaatagtttctttgtaattctttggtttttagttttcatatcctta
atttggataggtggtcaatttcctgtagacaactttctatcttatggtcgtatcttgaca
ttacattattattatcttcttatctgtatcttattctcttag

>Od22_cob
atgaaatctcatttacaatcatatccttgtcctctgatcataaattatttttggaatctt
ggttttttattagggattactattttattacaaattatatctggaatcttcttaggttta
cattatacatcagatattaattcagcatattttagtattttctttattattagagaaata
tattatggatggtgtttacgttatcttcattctaatggttcatcatttgtctttcttttg
atatttctacatcttggaagagctatatcttatggttcatatttttataatccaaatact
tggttttctggaattattattatcttcttcttaatgggaacagcatttatgggttatgtg
ttacctttaggacaaatgagtttatggggggttacagtaattacaaatttattatctgca
tttccatctttaatagaatggctttgtggaggacattacatttacaatcctacatttaag
aggttctttgtctttcattttctatttccatttcttctttgtggttttcttgtttatcat
atttttaatctacattttctatcttctaataatcctttaaggaattccactaataataaa
atagcatttttccctttcattattagtaaagatttatatggaaagatattaattctctat
ctatatcttcttcaaattcatttcggtttctcttctttctcacatccagataatgcatta
gaagcatgtggattacttactcctttacatatagtacctgaatggtatttcttatgccaa
tatgctatgttaaaagctgtgccaaataaaaatgcaggattcattatattatttacttct
atcttcatcttattttactttatgagaagtctttcaatctcattctattttatcgtgtgg
gtaagctctagatttaatagtttctttgtaattctttggtttttagttttcatatcctta
atttggataggtggtcaatttcctgtagacaactttctatcttatggtcgtatcttgaca
ttacattattattatcttcttatctgtatcttattatcttag
>Od23_cob
atgaaatctcatttacaatcatatccttgtcctctgatcataaattatttttggaatctt
ggttttttattagggattactattttattacaaattatatctggaatcttcttaggttta
cattatacatcagatattaattcagcatattttagtattttctttattattagagaaata
tattatggatggtgtttacgttatcttcattctaatggttcatcatttgtctttcttttg
atatttctacatcttggaagagctatatcttatggttcatatttttataatccaaatact
tggttttctggaattattattatcttcttcttaatgggaacagcatttatgggttatgtg
ttacctttaggacaaatgagtttatggggggttacagtaattacaaatttattatctgca
tttccatctttaatagaatggctttgtggaggacattacatttacaatcctacatttaag
aggttctttgtctttcattttctatttccatttcttctttgtggttttcttgtttatcat
atttttaatctacattttctatcttctaataatcctttaaggaattccactaataataaa
atagcatttttccctttcattattagtaaagatttatatggaaagatattaattctctat
ctatatcttcttcaaattcatttcggtttctcttctttctcacatccagataatgcatta
gaagcatgtggattacttactcctttacatatagtacctgaatggtatttcttatgccaa
tatgctatgttaaaagctgtgccaaataaaaatgcaggattcattatattatttacttct
atcttcatcttattttactttatgagaagtctttcaatctcattctattttatcgtgtgg
gtaagctctagatttaatagtttctttgtaattctttggtttttagttttcatatcctta
atttggataggtggtcaatttcctgtagacaactttctatcttatggtcgtatcttgaca
ttacattattattatcttcttatctgtatcttattatcttag
>Od24_cob
atgaaatctcatttacaatcatatccttgtcctctgatcataaattatttttggaatctt
ggttttttattagggattactattttattacaaattatatctggaatcttcttaggttta
cattatacatcagatattaattcagcatattttagtattttctttattattagagaaata
tattatggatggtgtttacgttatcttcattctaatggttcatcatttgtctttcttttg
atatttctacatcttggaagagctatatcttatggttcatatttttataatccaaatact
tggttttctggaattattattatcttcttcttaatgggaacagcatttatgggttatgtg
ttacctttaggacaaatgagtttatggggggttacagtaattacaaatttattatctgca
tttccatctttaatagaatggctttgtggaggacattacatttacaatcctacatttaag
aggttctttgtctttcattttctatttccatttcttctttgtggttttcttgtttatcat
atttttaatctacattttctatcttctaataatcctttaaggaattccactaataataaa
atagcatttttccctttcattattagtaaagatttatatggaaagatattaattctctat
ctatatcttcttcaaattcatttcggtttctcttctctctcacatccagataatgcatta
gaagcatgtggattacttactcctttacatatagtacctgaatggtatttcttatgccaa
tatgctatgttaaaagctgtgccaaataaaaatgcaggattcattatattatttacttct
atcttcatcttattttactttatgagaagtctttcagtatctttctattttatcgtgtgg
gtaagctctagatttaatagtttctttgtaattctttggtttttagttttcatatcctta
atttggataggtggtcaatttcctgtagacaactttctatcttatggtcgtatcttgaca
ttacattattattatcttcttatctgtatcttattatcttag

>Od25_cob
atgaaatctcatttacaatcatatccttgtcctctgatcataaattatttttggaatctt
ggtttttattagggattactattttattacaaattatatctggaatcttcttaggttta
cattatacatcagatattaattcagcatattttagtattttctttattattagagaaata
tattatggatggtgtttacgttatcttcattctaatggttcatcatttgtctttcttttg
atatttctacatcttggaagagctatatcttatggttcatatttttataatccaaatact
tggttttctggaattattattatcttcttcttaatgggaacagcatttatgggttatgtg
ttacctttaggacaaatgagtttatggggggttacagtaattacaaatttattatctgca
tttccatctttaatagaatggctttgtggaggacattacatttacaatcctacatttaag
aggttctttgtctttcattttctatttccatttcttctttgtggttttcttgtttatcat
atttttaatctacattttctatcttctaataatcctttaaggaattccactaataataaa
atagcatttttccctttcattattagtaaagatttatatggaaagatattaattctctat
ctatatcttcttcaaattcatttcggtttctcttctttctcacatccagataatgcatta
gaagcatgtggattacttactcctttacatatagtacctgaatggtatttcttatgccaa
tatgctatgttaaaagctgtgccaaataaaaatgcaggattcattatattatttacttct
atcttcatcttattttactttatgagaagtctttcaatctcattctattttatcgtgtgg
gtaagctctagatttaatagtttctttgtaattctttggtttttagttttcatatcctta
atttggataggtggtcaatttcctgtagacaactttctatcttatggtcgtatcttgaca
ttacattattattatcttcttatctgtatcttattatcttag
>Od26_cob
atgaaatctcatttacaatcatatccttgtcctctgatcataaattatttttggaatctt
ggtttttattagggattactattttattacaaattatatctggaatcttcttaggttta
cattatacatcagatattaattcagcatattttagtattttctttattattagagaaata
tattatggatggtgtttacgttatcttcattctaatggttcatcatttgtctttcttttg
atatttctacatcttggaagagctatatcttatggttcatatttttataatccaaatact
tggttttctggaattattattatcttcttcttaatgggaacagcatttatgggttatgtg
ttacctttaggacaaatgagtttatggggggttacagtaattacaaatttattatctgca
tttccatctttaatagaatggctttgtggaggacattacatttacaatcctacatttaag
aggttctttgtctttcattttctatttccatttcttctttgtggttttcttgtttatcat
atttttaatctacattttctatcttctaataatcctttaaggaattccactaataataaa
atagcatttttccctttcattattagtaaagatttatatggaaagatattaattctctat
ctatatcttcttcaaattcatttcggtttctcttctttctcacatccagataatgcatta
gaagcatgtggattacttactcctttacatatagtacctgaatggtatttcttatgccaa
tatgctatgttaaaagctgtgccaaataaaaatgcaggattcattatattatttacttct
atcttcatcttattttactttatgagaagtctttcaatctcattctattttatcgtgtgg
gtaagctctagatttaatagtttctttgtaattctttggtttttagttttcatatcctta
atttggataggtggtcaatttcctgtagacaactttctatcttatggtcgtatcttgaca
ttacattattattatcttcttatctgtatcttattatcttag
>Od27_cob
atgaaatctcatttacaatcatatccttgtcctctgatcataaattatttttggaatctt
ggtttttattagggattactattttattacaaattatatctggaatcttcttaggttta
cattatacatcagatattaattcagcatattttagtattttctttattattagagaaata
tattatggatggtgtttacgttatcttcattctaatggttcatcatttgtctttcttttg
atatttctacatcttggaagagctatatcttatggttcatatttttataatccaaatact
tggttttctggaattattattatcttcttcttaatgggaacagcatttatgggttatgtg
ttacctttaggacaaatgagtttatggggggttacagtaattacaaatttattatctgca
tttccatctttaatagaatggctttgtggaggacattacatttacaatcctacatttaag
aggttctttgtctttcattttctatttccatttcttctttgtggttttcttgtttatcat
atttttaatctacattttctatcttctaataatcctttaaggaattccactaataataaa
atagcatttttccctttcattattagtaaagatttatatggaaagatattaattctctat
ctatatcttcttcaaattcatttcggtttctcttctttctcacatccagataatgcatta
gaagcatgtggattacttactcctttacatatagtacctgaatggtatttcttatgccaa
tatgctatgttaaaagctgtgccaaataaaaatgcaggattcattatattatttacttct
atcttcatcttattttactttatgagaagtctttcagtatctttctattttatcgtgtgg
gtaagctctagatttaatagtttctttgtaattctttggtttttagttttcatatcctta
atttggataggtggtcaatttcctgtagacaactttctatcttatggtcgtatcttgaca
ttacattattattatcttcttatctgtatcttattatcttag

>Od4_cob
atgaaatctcatttacaatcatatccttgtcctctgatcataaattatttttggaatctt
ggtttttattagggattactattttattacaaattatatctggaatcttcttaggttta
cattatacatcagatattaattcagcatattttagtattttctttattattagagaaata
tattatggatggtgtttacgttatcttcattctaatggttcatcatttgtctttcttttg
atatttctacatcttggaagagctatatcttatggttcatatttttataatccaaatact
tggttttctggaattattattatcttcttcttaatgggaacagcatttatgggttatgtg
ttacctttaggacaaatgagtttatggggggttacagtaattacaaatttattatctgca
tttccatctttaatagaatggctttgtggaggacattacatttacaatcctacatttaag
aggttctttgtctttcattttctatttccatttcttctttgtggttttcttgtttatcat
atttttaatctacattttctatcttctaataatcctttaaggaattccactaataataaa
atagcatttttccctttcattattagtaaagatttatatggaaagatattaattctctat
ctatatcttcttcaaattcatttcggtttctcttctttctcacatccagataatgcatta
gaagcatgtggattacttactcctttacatatagtacctgaatggtatttcttatgccaa
tatgctatgttaaaagctgtgccaaataaaaatgcaggattcattatattatttacttct
atcttcatcttattttactttatgagaagtctttcaatctcattctattttatcgtgtgg
gtaagctctagatttaatagtttctttgtaattctttggtttttagttttcatatcctta
atttggataggtggtcaatttcctgtagacaactttctatcttatggtcgtatcttgaca
ttacattattattatcttcttatctgtatcttattatcttag
>Od6_cob
atgaaatctcatttacaatcatatccttgtcctctgatcataaattatttttggaatctt
ggtttttattagggattactattttattacaaattatatctggaatcttcttaggttta
cattatacatcagatattaattcagcatattttagtattttctttattattagagaaata
tattatggatggtgtttacgttatcttcattctaatggttcatcatttgtctttcttttg
atatttctacatcttggaagagctatatcttatggttcatatttttataatccaaatact
tggttttctggaattattattatcttcttcttaatgggaacagcatttatgggttatgtg
ttacctttaggacaaatgagtttatggggggttacagtaattacaaatttattatctgca
tttccatctttaatagaatggctttgtggaggacattacatttacaatcctacatttaag
aggttctttgtctttcattttctatttccatttcttctttgtggttttcttgtttatcat
atttttaatctacattttctatcttctaataatcctttaaggaattccactaataataaa
atagcatttttccctttcattattagtaaagatttatatggaaagatattaattctctat
ctatatcttcttcaaattcatttcggtttctcttctttctcacatccagataatgcatta
gaagcatgtggattacttactcctttacatatagtacctgaatggtatttcttatgccaa
tatgctatgttaaaagctgtgccaaataaaaatgcaggattcattatattatttacttct
atcttcatcttattttactttatgagaagtctttcaatctcattctattttatcgtgtgg
gtaagctctagatttaatagtttctttgtaattctttggtttttagttttcatatcctta
atttggataggtggtcaatttcctgtagacaactttctatcttatggtcgtatcttgaca
ttacattattattatcttcttatctgtatcttattatcttag
>Od8_cob
atgaaatctcatttacaatcatatccttgtcctctgatcataaattatttttggaatctt
ggtttttattagggattactattttattacaaattatatctggaatcttcttaggttta
cattatacatcagatattaattcagcatattttagtattttctttattattagagaaata
tattatggatggtgtttacgttatcttcattctaatggttcatcatttgtctttcttttg
atatttctacatcttggaagagctatatcttatggttcatatttttataatccaaatact
tggttttctggaattattattatcttcttcttaatgggaacagcatttatgggttatgtg
ttacctttaggacaaatgagtttatggggggttacagtaattacaaatttattatctgca
tttccatctttaatagaatggctttgtggaggacattacatttacaatcctacatttaag
aggttctttgtctttcattttctatttccatttcttctttgtggttttcttgtttatcat
atttttaatctacattttctatcttctaataatcctttaaggaattccactaataataaa
atagcatttttccctttcattattagtaaagatttatatggaaagatattaattctctat
ctatatcttcttcaaattcatttcggtttctcttctttctcacatccagataatgcatta
gaagcatgtggattacttactcctttacatatagtacctgaatggtatttcttatgccaa
tatgctatgttaaaagctgtgccaaataaaaatgcaggattcattatattatttacttct
atcttcatcttattttactttatgagaagtctttcaatctcattctattttatcgtgtgg
gtaagctctagatttaatagtttctttgtaattctttggtttttagttttcatatcctta
atttggataggtggtcaatttcctgtagacaactttctatcttatggtcgtatcttgaca
ttacattattattatcttcttatctgtatcttattctcttag

>Od9_cob
atgaaatctcatttacaatcatatccttgtcctctgatcataaattatttttggaatctt
ggtttttattagggattactattttattacaaattatatctggaatcttcttaggttta
cattatacatcagatattaattcagcatattttagtattttctttattattagagaaata
tattatggatggtgtttacgttatcttcattctaatggttcatcatttgtctttcttttg
atatttctacatcttggaagagctatatcttatggttcatatttttataatccaaatact
tggttttctggaattattattatcttcttcttaatgggaacagcatttatgggttatgtg
ttacctttaggacaaatgagtttatggggggttacagtaattacaaatttattatctgca
tttccatctttaatagaatggctttgtggaggacattacatttacaatcctacatttaag
aggttctttgtctttcattttctatttccatttcttctttgtggttttcttgtttatcat
atttttaatctacattttctatcttctaataatcctttaaggaattccactaataataaa
atagcatttttccctttcattattagtaaagatttatatggaaagatattaattctctat
ctatatcttcttcaaattcatttcggtttctcttctttctcacatccagataatgcatta
gaagcatgtggattacttactcctttacatatagtacctgaatggtatttcttatgccaa
tatgctatgttaaaagctgtgccaaataaaaatgcaggattcattatattatttacttct
atcttcatcttattttactttatgagaagtctttcaatctcattctattttatcgtgtgg
gtaagctctagatttaatagtttctttgtaattctttggtttttagttttcatatcctta
atttggataggtggtcaatttcctgtagacaactttctatcttatggtcgtatcttgaca
ttacattattattatcttcttatctgtatcttattatcttag
>Ss5_cob
atgaaatctcatttacaatcatatccttgtcctctgatcataaattatttttggaatctt
ggtttttattagggattactattttattacaaattatatctggaatcttcttaggttta
cattatacatcagatattaattcagcatattttagtattttctttattattagagaaata
tattatggatggtgtttacgttatcttcattctaatggttcatcatttgtctttcttttg
atatttctacatcttggaagagctatatcttatggttcatatttttataatccaaatact
tggttttctggaattattattatcttcttcttaatgggaacagcatttatgggttatgtg
ttacctttaggacaaatgagtttatggggggttacagtaattacaaatttattatctgca
tttccatctttaatagaatggctttgtggaggacattacatttacaatcctacatttaag
aggttctttgtctttcattttctatttccatttcttctttgtggttttcttgtttatcat
atttttaatctacattttctatcttctaataatcctttaaggaattccactaataataaa
atagcatttttccctttcattattagtaaagatttatatggaaagatattaattctctat
ctatatcttcttcaaattcatttcggtttctcttctttctcacatccagataatgcatta
gaagcatgtggattacttactcctttacatatagtacctgaatggtatttcttatgccaa
tatgctatgttaaaagctgtgccaaataaaaatgcaggattcattatattatttacttct
atcttcatcttattttactttatgagaagtctttcaatctcattctattttatcgtgtgg
gtaagctctagatttaatagtttctttgtaattctttggtttttagttttcatatcctta
atttggataggtggtcaatttcctgtagacaactttctatcttatggtcgtatcttgaca
ttacattattattatcttcttatctgtatcttattctcttag
>Ss6_cob
atgaaatctcatttacaatcatatccttgtcctctgatcataaattatttttggaatctt
ggtttttattagggattactattttattacaaattatatctggaatcttcttaggttta
cattatacatcagatattaattcagcatattttagtattttctttattattagagaaata
tattatggatggtgtttacgttatcttcattctaatggttcatcatttgtctttcttttg
atatttctacatcttggaagagctatatcttatggttcatatttttataatccaaatact
tggttttctggaattattattatcttcttcttaatgggaacagcatttatgggttatgtg
ttacctttaggacaaatgagtttatggggggttacagtaattacaaatttattatctgca
tttccatctttaatagaatggctttgtggaggacattacatttacaatcctacatttaag
aggttctttgtctttcattttctatttccatttcttctttgtggttttcttgtttatcat
atttttaatctacattttctatcttctaataatcctttaaggaattccactaataataaa
atagcatttttccctttcattattagtaaagatttatatggaaagatattaattctctat
ctatatcttcttcaaattcatttcggtttctcttctttctcacatccagataatgcatta
gaagcatgtggattacttactcctttacatatagtacctgaatggtatttcttatgccaa
tatgctatgttaaaagctgtgccaaataaaaatgcaggattcattatattatttacttct
atcttcatcttattttactttatgagaagtctttcaatatctttctattttatcgtgtgg
gtaagctctagatttaatagtttctttgtaattctttggtttttagttttcatatcctta
atttggataggtggtcaatttcctgtagacaactttctatcttatggtcgtatcttgaca
ttacattattattatcttcttatctgtatcttattatcttag

>Ss7_cob
atgaaatctcatttacaatcatatccttgtcctctgatcataaattatttttggaatctt
ggtttttattagggattactattttattacaaattatatctggaatcttcttaggttta
cattatacatcagatattaattcagcatattttagtattttctttattattagagaaata
tattatggatggtgtttacgttatcttcattctaatggttcatcatttgtctttcttttg
atatttctacatcttggaagagctatatcttatggttcatatttttataatccaaatact
tggttttctggaattattattatcttcttcttaatgggaacagcatttatgggttatgtg
ttacctttaggacaaatgagtttatggggggttacagtaattacaaatttattatctgca
tttccatctttaatagaatggctttgtggaggacattacatttacaatcctacatttaag
aggttctttgtctttcattttctatttccatttcttctttgtggttttcttgtttatcat
atttttaatctacattttctatcttctaataatcctttaaggaattccactaataataaa
atagcatttttccctttcattattagtaaagatttatatggaaagatattaattctctat
ctatatcttcttcaaattcatttcggtttctcttctttctcacatccagataatgcatta
gaagcatgtggattacttactcctttacatatagtacctgaatggtatttcttatgccaa
tatgctatgttaaaagctgtgccaaataaaaatgcaggattcattatattatttacttct
atcttcatcttattttactttatgagaagtctttcaatctcattctattttatcgtgtgg
gtaagctctagatttaatagtttctttgtaattctttggtttttagttttcatatcctta
atttggataggtggtcaatttcctgtagacaactttctatcttatggtcgtatcttgaca
ttacattattattatcttcttatctgtatcttattatcttag
>Ss8_cob
atgaaatctcatttacaatcatatccttgtcctctgatcataaattatttttggaatctt
ggtttttattagggattactattttattacaaattatatctggaatcttcttaggttta
cattatacatcagatattaattcagcatattttagtattttctttattattagagaaata
tattatggatggtgtttacgttatcttcattctaatggttcatcatttgtctttcttttg
atatttctacatcttggaagagctatatcttatggttcatatttttataatccaaatact
tggttttctggaattattattatcttcttcttaatgggaacagcatttatgggttatgtg
ttacctttaggacaaatgagtttatggggggttacagtaattacaaatttattatctgca
tttccatctttaatagaatggctttgtggaggacattacatttacaatcctacatttaag
aggttctttgtctttcattttctatttccatttcttctttgtggttttcttgtttatcat
atttttaatctacattttctatcttctaataatcctttaaggaattccactaataataaa
atagcatttttccctttcattattagtaaagatttatatggaaagatattaattctctat
ctatatcttcttcaaattcatttcggtttctcttctttctcacatccagataatgcatta
gaagcatgtggattacttactcctttacatatagtacctgaatggtatttcttatgccaa
tatgctatgttaaaagctgtgccaaataaaaatgcaggattcattatattatttacttct
atcttcatcttattttactttatgagaagtctttcaatctcattctattttatcgtgtgg
gtaagctctagatttaatagtttctttgtaattctttggtttttagttttcatatcctta
atttggataggtggtcaatttcctgtagacaactttctatcttatggtcgtatcttgaca
ttacattattattatcttcttatctgtatcttattatcttag
>ohdo3_cob
atgaaatctcatttacaatcatatccttgtcctctgatcataaattatttttggaatctt
ggtttttattagggattactattttattacaaattatatctggaatcttcttaggttta
cattatacatcagatattaattcagcatattttagtattttctttattattagagaaata
tattatggatggtgtttacgttatcttcattctaatggttcatcatttgtctttcttttg
atatttctacatcttggaagagctatatcttatggttcatatttttataatccaaatact
tggttttctggaattattattatcttcttcttaatgggaacagcatttatgggttatgtg
ttacctttaggacaaatgagtttatggggagttacagtaattacaaatttattatctgca
tttccatctttaatagaatggctttgtggaggacattacatttacaatcctacatttaag
aggttctttgtctttcattttctatttccatttcttctttgtggttttcttgtttatcat
atttttaatctacattttctatcttctaataatcctttaaggaattccactaataataaa
atagcatttttccctttcattattagtaaagatttatatggaaagatattaattctctat
ctatatcttcttcaaattcatttcggtttctcttctttctcacatccagataatgcatta
gaagcatgtggattacttactcctttacatatagtacctgaatggtatttcttatgccaa
tatgctatgttaaaagctgtgccaaataaaaatgcaggattcattatattatttacttct
atcttcatcttattttactttatgagaagtctttcagtatctttctattttatcgtgtgg
gtaagctctagatttaatagtttctttgtaattctttggtttttagttttcatatcctta
atttggataggtggtcaatttcctgtagacaactttctatcttatggtcgtatcttgaca
ttacattattattatcttcttatctgtatcttattatcttag

>ohdo5_cob
atgaaatctcatttacaatcatatccttgtcctctgatcataaattatttttggaatctt
ggtttttattagggattactattttattacaaattatatctggaatcttcttaggttta
cattatacatcagatattaattcagcatattttagtattttctttattattagagaaata
tattatggatggtgtttacgttatcttcattctaatggttcatcatttgtctttcttttg
atatttctacatcttggaagagctatatcttatggttcatatttttataatccaaatact
tggttttctggaattattattatcttcttcttaatgggaacagcatttatgggttatgtg
ttacctttaggacaaatgagtttatggggggttacagtaattacaaatttattatctgca
tttccatctttaatagaatggctttgtggaggacattacatttacaatcctacatttaag
aggttctttgtctttcattttctatttccatttcttctttgtggttttcttgtttatcat
atttttaatctacattttctatcttctaataatcctttaaggaattccactaataataaa
atagcatttttccctttcattattagtaaagatttatatggaaagatattaattctctat
ctatatcttcttcaaattcatttcggtttctcttctttctcacatccagataatgcatta
gaagcatgtggattacttactcctttacatatagtacctgaatggtatttcttatgccaa
tatgctatgttaaaagctgtgccaaataaaaatgcaggattcattatattatttacttct
atcttcatcttattttactttatgagaagtctttcagtatctttctattttatcgtgtgg
gtaagctctagatttaatagtttctttgtaattctttggtttttagttttcatatcctta
atttggataggtggtcaatttcctgtagacaactttctatcttatggtcgtatcttgaca
ttatattattattattttcttatctgtatcttattatcttag
>ohdo7_cob
atgaaatctcatttacaatcatatccttgtcctctgatcataaattatttttggaatctt
ggtttttattagggattactattttattacaaattatatctggaatcttcttaggttta
cattatacatcagatattaattcagcatattttagtattttctttattattagagaaata
tattatggatggtgtttacgttatcttcattctaatggttcatcatttgtctttcttttg
atatttctacatcttggaagagctatatcttatggttcatatttttataatccaaatact
tggttttctggaattattattatcttcttcttaatgggaacagcatttatgggttatgtg
ttacctttaggacaaatgagtttatggggggttacagtaattacaaatttattatctgca
tttccatctttaatagaatggctttgtggaggacattacatttacaatcctacatttaag
aggttctttgtctttcattttctatttccatttcttctttgtggttttcttgtttatcat
atttttaatctacattttctatcttctaataatcctttaaggaattccactaataataaa
atagcatttttccctttcattattagtaaagatttatatggaaagatattaattctctat
ctatatcttcttcaaattcatttcggtttctcttctttctcacatccagataatgcatta
gaagcatgtggattacttactcctttacatatagtacctgaatggtatttcttatgccaa
tatgctatgttaaaagctgtgccaaataaaaatgcaggattcattatattatttacttct
atcttcatcttattttactttatgagaagtctttcagtatctttctattttatcgtgtgg
gtaagctctagatttaatagtttctttgtaattctttggtttttagttttcatatcctta
atttggataggtggtcaatttcctgtagacaactttctatcttatggtcgtatcttgaca
ttacattattattatcttcttatctgtatcttattatcttag
>sesoko1_cob
atgaaatctcatttacaatcatatccttgtcctctgatcataaattatttttggaatctt
ggtttttattagggattactattttattacaaattatatctggaatcttcttaggttta
cattatacatcagatattaattcagcatattttagtattttctttattattagagaaata
tattatggatggtgtttacgttatcttcattctaatggttcatcatttgtctttcttttg
atatttctacatcttggaagagctatatcttatggttcatatttttataatccaaatact
tggttttctggaattattattatcttcttcttaatgggaacagcatttatgggttatgtg
ttacctttaggacaaatgagtttatggggggttacagtaattacaaatttattatctgca
tttccatctttaatagaatggctttgtggaggacattacatttacaatcctacatttaag
aggttctttgtctttcattttctatttccatttcttctttgtggttttcttgtttatcat
atttttaatctacattttctatcttctaataatcctttaaggaattccactaataataaa
atagcatttttccctttcattattagtaaagatttatatggaaagatattaattctctat
ctatatcttcttcaaattcatttcggtttctcttctttctcacatccagataatgcatta
gaagcatgtggattacttactcctttacatatagtacctgaatggtatttcttatgccaa
tatgctatgttaaaagctgtgccaaataaaaatgcaggattcattatattatttacttct
atcttcatcttattttactttatgagaagtctttcagtatctttctattttatcgtgtgg
gtaagctctagatttaatagtttctttgtaattctttggtttttagttttcatatccttt
atttggataggtggtcaatttcctgtagacaactttctatcttatggtcgtatcttgaca
ttatattattattattttcttatctgtatcttattatcttag

>sesoko2_cob
atgaaatctcatttacaatcatatccttgtcctctgatcataaattatttttggaatctt
ggtttttattagggattactattttattacaaattatatctggaatcttcttaggttta
cattatacatcagatattaattcagcatattttagtattttctttattattagagaaata
tattatggatggtgtttacgttatcttcattctaatggttcatcatttgtctttcttttg
atatttctacatcttggaagagctatatcttatggttcatatttttataatccaaatact
tggttttctggaattattattatcttcttcttaatgggaacagcatttatgggttatgtg
ttacctttaggacaaatgagtttatgggggttacagtaattacaaatttattatctgca
tttccatctttaatagaatggctttgtggaggacattacatttacaatcctacatttaag
aggttctttgtctttcattttctatttccatttcttctttgtggttttcttgtttatcat
atttttaatctacattttctatcttctaataatcctttaaggaattccactaataataaa
atagcatttttccctttcattattagtaaagatttatatggaaagatattaattctctat
ctatatcttcttcaaattcatttcggtttctcttctttctcacatccagataatgcatta
gaagcatgtggattacttactcctttacatatagtacctgaatggtatttcttatgccaa
tatgctatgttaaaagctgtgccaaataaaaatgcaggattcattatattatttacttct
atcttcatcttattttactttatgagaagtctttcagtatctttctattttatcgtgtgg
gtaagctctagatttaatagtttctttgtaattctttggtttttagttttcatatctttt
atttggataggtggtcaatttcctgtagacaactttctatcttatggtcgtatcttgaca
ttatattattattattttcttatctgtatcttattatcttag
>sesoko3_cob
atgaaatctcatttacaatcatatccttgtcctctgatcataaattatttttggaatctt
ggtttttattagggattactattttattacaaattatatctggaatcttcttaggttta
cattatacatcagatattaattcagcatattttagtattttctttattattagagaaata
tattatggatggtgtttacgttatcttcattctaatggttcatcatttgtctttcttttg
atatttctacatcttggaagagctatatcttatggttcatatttttataatccaaatact
tggttttctggaattattattatcttcttcttaatgggaacagcatttatgggttatgtg
ttacctttaggacaaatgagtttatgggggttacagtaattacaaatttattatctgca
tttccatctttaatagaatggctttgtggaggacattacatttacaatcctacatttaag
aggttctttgtctttcattttctatttccatttcttctttgtggttttcttgtttatcat
atttttaatctacattttctatcttctaataatcctttaaggaattccactaataataaa
atagcatttttccctttcattattagtaaagatttatatggaaagatattaattctctat
ctatatcttcttcaaattcatttcggtttctcttctttctcacatccagataatgcatta
gaagcatgtggattacttactcctttacatatagtacctgaatggtatttcttatgccaa
tatgctatgttaaaagctgtgccaaataaaaatgcaggattcattatattatttacttct
atcttcatcttattttactttatgagaagtctttcagtatctttctattttatcgtgtgg
gtaagctctagatttaatagtttctttgtaattctttggtttttagttttcatatccttt
atttggataggtggtcaatttcctgtagacaactttctatcttatggtcgtatcttgaca
ttacattattattatcttcttatctgtatcttattatcttag
>sesoko4_cob
atgaaatctcatttacaatcatatccttgtcctctgatcataaattatttttggaatctt
ggtttttattagggattactattttattacaaattatatctggaatcttcttaggttta
cattatacatcagatattaattcagcatattttagtattttctttattattagagaaata
tattatggatggtgtttacgttatcttcattctaatggttcatcatttgtctttcttttg
atatttctacatcttggaagagctatatcttatggttcatatttttataatccaaatact
tggttttctggaattattattatcttcttcttaatgggaacagcatttatgggttatgtg
ttacctttaggacaaatgagtttatgggggttacagtaattacaaatttattatctgca
tttccatctttaatagaatggctttgtggaggacattacatttacaatcctacatttaag
aggttctttgtctttcattttctatttccatttcttctttgtggttttcttgtttatcat
atttttaatctacattttctatcttctaataatcctttaaggaattccactaataataaa
atagcatttttccctttcattattagtaaagatttatatggaaagatattaattctctat
ctatatcttcttcaaattcatttcggtttctcttctttctcacatccagataatgcatta
gaagcatgtggattacttactcctttacatatagtacctgaatggtatttcttatgccaa
tatgctatgttaaaagctgtgccaaataaaaatgcaggattcattatattatttacttct
atcttcatcttattttactttatgagaagtctttcagtatctttctattttatcgtgtgg
gtaagctctagatttaatagtttctttgtaattctttggtttttagttttcatatcctta
atttggataggtggtcaatttcctgtagacaactttctatcttatggtcgtatcttgaca
ttacattattattatcttcttatctgtatcttattatcttag

>REF_DNA_Cob_LC002802.1
ATGAAATCTCATTTACAATCATATCCTTGTCCTCTGATCATAAATTATTTTTGGAATCTT
GGTTTTTTATTAGGGATTACTATTTTATTACAAATTATATCTGGAATCTTCTTAGGTTTA
CATTATACATCAGATATTAATTCAGCATATTTTAGTATTTTCTTTATTATTAGAGAAATA
TATTATGGATGGTGTTTACGTTATCTTCATTCTAATGGTTCATCATTTGTCTTTCTTTTT
ATATTTCTACATCTTGGAAGAGCTATATCTTATGGTTCATATTTTTATAATCCAAATACT
TGGTTTTCTGGAATTATTATTATTTTCTTCTTGATGGGAACAGCATTTATGGGTTATGTG
TTACCTTTAGGACAAATGAGTTTATGGGGGGTTACAGTAATTACAAATTTATTATCTGCA
TTTCCATCTTTAATAGAATGGCTTTGTGGAGGACATTACATTTACAATCCTACATTTAAG
AGGTTCTTTGTCTTTCATTTTCTATTTCCATTTCTTCTTTGTGGTTTTCTTCTTTATCAT
ATTTTTAATCTACATTTTCTATCTTCTAATAATCCTTTAAGGAATTCCACTAATAATAAA
ATAGCATTTTTGCCTTTCATTATTAGTAAAGATTTCTATGGAATGATATTAATTTCCTAT
CTATATCTTCTTCAGATTCATTTTGGATTCTCTTCTTTCTCACATCCAGATAATGCATTG
GAAGCTTGTGGATTACTTACACCTTTACACATTGTACCTGAATGGTATTTTCTATGCCAA
TATGCTATGTTAAAAGCTGTACCCAATAAAAATGCAGGATTCATTGTCTTACTAACTTCT
ATCTTTGTATTATTTTATTTTATGAGAAGTCTTTCAATATCTTTCTATTTTATCGTGTGG
GTAAGCTCTAGATTTAATAGTTTCTTTGTAATTCTTTGGTTTTTAGTTTTCATATCCTTA
ATTTGGATAGGTGGTCAATTTCCTGTCGACAACTTTCTATCTTATGGTCGTATCTTGACA
TTATATTATTATTATTTTCTTATCTGTATCTTATTATCTTAG
>REF_RNA_Cob_symbB1.EST_k37c20_4808
ATGAAATCTCATTTACAATCATATCCTTGTCCTCTGATCATAAATTATTTTTGGAATCTT
GGTTTTTTATTAGGGGTTGCTATTTTATTACAAATTATATCTGGAATCTTCTTAGGTTTA
CATTATACATCAGATATTAATTCAGCATATTTTAGTATTTTCTTTATTATTAGAGAAGTA
TATTATGGATGGTGTTTACGTTATTTTCATTCTAATGGTTCATCATTTGTCTTTCTTTTT
ATATTTCTACATCTTGGAAGAGCTATATCTTATGGTTCATATTTTTATAATCCAAATACT
TGGTTTTCTGGAATTATTATTGTTTTCTTCTTGATGGGAATAGCATTTATGGGTTATGTG
TTACCTTTAGGACAAATGAGTTTATGGGGGGTTACAGTAATTACAAATTTATTATCTGCA
TTTCCATCTTTAGTAGAATGGCTTTGTGGAGGATATTGCATTCACAATCCTACATTTAAG
AGGTTCTTTGTCTTTCATTTTCTACTTCCATTTCTTCTTTGTGGTTTTCTTCTTTATCAT
ATTTTTAGTCTACATTTTCTATCTTCTAATAATCCTTTAAGGAATTCCACTAATAATAAA
ATAGCATTTTTGCCTTTCATTATTAGTAAAGATCTCTATGGAATGATATTAATTTCCTAT
CTATATTTTCTTCAGATTCATTTTGGATTCTCTTCTCTCTCACATCCAGATAATGCATTG
GAAGCTTGTGCATTACGTACACCTTTACACATTGTACCTGAATGGTATTTTCTATGCCAA
TATGCTATGTTAAAAGCTGTACCCAATAAAAATGCAGGATTCATTGTCTTACTAACTTCT
ATCTTTGTATTATTTTATTTTATGAGAAGTCTTTCAGTATCTTTCTATTTTATCGTGTGG
GTAAGCTCTAGATTTAATAGTTTCTTTGTAATTCTTTGGTTTTTAGTTTTCATATCCTTA
ATTTGGATAGGTGGTCAATTTCCTGTCGACAACTTTCTATCTTATGCTCGTATCTTGACA
TTATATTATTATTATTTTCTTATCTGTATCTTATTATCTTAG

Dataset S2. The nucleotide sequences of Symbiodiniciae plastid (pt) genomes from 150 individuals of *Acropora digitifera* and the reference sequences from *Breviolum minutum*

>Hd1_psbA
atgaagaacacatcttactatcaactcaacttacttggtaatgtcataggattcgtacta
tccacaacaaatcgtctctacattgggtgctttggtatcctaatgttccctcttttaact
ttagcaactattgcttatatcacagcttttattcttgcacctgcagtagatattgatgga
ataagagaaccagtagctggctcacttctttatggcaataacattataacaggagctgta
ataccaagttctaatgctattggggttcatttctatccagtttgggagtcaaatggattt
gatgagtgcttatataatggtggtacatatcaatttgtagtacttcattttatgcttggt
gttgcttgttggatgggtagagaatgggaattcagtttcagattaggtatgagaccatgg
atatttgtagctttctcagcacctgttgtagcagcttttgcagtatttgttgtttatcca
attggtcaagctagcttttccgatggaatgcctttaggaataagtggaacttttaatttt
atgcttgtgttccaagcagaacataacattttaatgcatccattccatatcctaggagta
gctggtgtatttggaggttccttatttagtgcaatgcatggttcattagttacatcttca
cttcttgcagaaagtgcaggagatattagcctaaatgttggatataagtttggtcaagaa
gatgaaacttatagcatatcagcagcccatggttattttggtagactcatatttcaatat
gcttccttcaataactctcgtagtcttcacttcttcctagcagcttggccagttattggt
atttggttcacagcacttggagtaagtacaatggctttcaacttaaatggtttaaacttc
aaccaatccatcctagattccagtggccatctaatcttaagttgggcagatattgtgaat
cgtgctgatctaggtatggaagtgatgcatgaaagaaatgctcacaatttcccattagat
ctagcataa
>Hd2_psbA
atgaagaacacatcttactatcaactcaacttacttggtaatgtcataggattcgtacta
tccacaacaaatcgtctctacattgggtgctttggtatcctaatgttccctcttttaact
ttagcaactattgcttatatcacagcttttattcttgcacctgcagtagatattgatgga
ataagagaaccagtagctggctcacttctttatggcaataacattataacaggagctgta
ataccaagttctaatgctattggggttcatttctatccagtttgggagtcaaatggattt
gatgagtgcttatataatggtggtacatatcaatttgtagtacttcattttatgcttggt
gttgcttgttggatgggtagagaatgggaattcagtttcagattaggtatgagaccatgg
atatttgtagctttctcagcacctgttgtagcagcttttgcagtatttgttgtttatcca
attggtcaagctagcttttccgatggaatgcctttaggaataagtggaacttttaatttt
atgcttgtgttccaagcagaacataacattttaatgcatccattccatatcctaggagta
gctggtgtatttggaggttccttatttagtgcaatgcatggttcattagttacatcttca
cttcttgcagaaagtgcaggagatattagcctaaatgttggatataagtttggtcaagaa
gatgaaacttatagcatatcagcagcccatggttattttggtagactcatatttcaatat
gcttccttcaataactctcgtagtcttcacttcttcctagcagcttggccagttattggt
atttggttcacagcacttggagtaagtacaatggctttcaacttaaatggtttaaacttc
aaccaatccatcctagattccagtggccatctaatcttaagttgggcagatattgtgaat
cgtgctgatctaggtatggaagtgatgcatgaaagaaatgctcacaatttcccattagat
ctagcataa
>Hd3_psbA
atgaagaacacatcttactatcaactcaacttacttggtaatgtcataggattcgtgcta
tccacaacaaatcgtctctatattggatgctttggaatcctcatgttccctcttttaact
ttagcaactattgcttatatcacagcttttattcttgcacctgcagtagatattgatgga
ataagagaaccagtagctggctcacttctttatggcaacaacattataacaggagctgta
ataccaagttctaatgctattggggttcatttctatccagtttgggagtcaaatggtttc
gatgagtgcttatataatggtggaacatatcaattcgtagtccttcattttatgcttggt
gttgcttgttggatgggtagagaatgggaattcagtttcagattaggtatgagaccatgg
atatttgtagctttctcagcacctgttattgcagcctttgcagtatttgttgtttatcca
attggtcaagctagcttttccgatggaatgcctttaggaataagtggaacttttaatttt
atgcttgtgttccaagcagaacataacattttaatgcatccattccatatcctaggagta
gctggtgtatttggaggttccttatttagtgcaatgcatggttcattagttacatcttca
cttcttgcagaaagtgcaggagatattagcctcaatgttggatataagtttggtcaagaa
gatgaaacttatagcatatcagcagcccatggttattttggtagactcatatttcaatat
gcttccttcaataactctcgtagtcttcacttcttcctagcagcttggccagttattggt
atttggttcacagcccttggagtaagtacaatggctttcaacttaaatggtttaaacttc
aaccaatccatcctagattccagtggccatctaatcttaagttgggcagatattgtgaat
cgtgctgatctaggtatggaagtgatgcatgaaagaaatgctcacaatttcccattagat
ctagcataa

>Hd4_psbA
atgaagaacacatcttactatcaactcaacttacttggtaatgtcataggattcgtacta
tccacaacaaatcgtctctacattgggtgctttggtatcctaatgttccctcttttaact
ttagctactattgcttatatcaccgcttttattcttgcacctgcagtagatattgatgga
ataagagaaccagtagctggctcacttctttatggcaataacattataacaggagctgta
ataccaagttctaatgctattggggttcatttctatccagtttgggagtcaaatgaattt
gatgagtgcttatataatggtggtacatatcaatttgtagtacttcattttatgcttggt
gttgcttgttggatgggtagagaatgggaattcagtttcagattaggtatgagaccatgg
atatttgtagctttctcagcacctgttgtagcagcttttgcagtatttgttgtttatcca
attggtcaagctagcttttccgatggaatgcctttaggaataagtggaacttttaatttt
atgcttgttttccaagcagaacataacattttaatgcatccattccatatcctaggagta
gctggtgtatttggaggttccttatttagtgcaatgcatggttcattagttacatcttca
cttcttgcagaaagtgcaggagatattagcctaaatgttggatataagtttggtcaagaa
gatgaaacttatagcatatcagcagcccatggttattttggtagactcatatttcaatat
gcttccttcaataactctcgtagtcttcacttcttcctagcagcttggccagttattggt
atttggttcacagcacttggagtaagtacaatggctttcaacttaaatggtttaaacttc
aaccaatccatcctagattccagtggccatctaatcttaagttgggcagatattgtgaat
cgtgctgatctaggtatggaagtgatgcatgaaagaaatgctcacaatttcccattagat
ctagcataa
>Hd5_psbA
atgaagaacacatcttactatcaactcaacttacttggtaatgtcataggattcgtacta
tccacaacaaatcgtctctacattgggtgctttggtatcctaatgttccctcttttaact
ttagcaactattgcttatatcacagcttttattcttgcacctgcagtagatattgatgga
ataagagaaccagtagctggctcacttctttatggcaataacattataacaggagctgta
ataccaagttctaatgctattggggttcatttctatccagtttgggagtcaaatggattt
gatgagtgcttatataatggtggtacatatcaatttgtagtacttcattttatgcttggt
gttgcttgttggatgggtagagaatgggaattcagtttcagattaggtatgagaccatgg
atatttgtagctttctcagcacctgttgtagcagcttttgcagtatttgttgtttatcca
attggtcaagctagcttttccgatggaatgcctttaggaataagtggaacttttaatttt
atgcttgtgttccaagcagaacataacattttaatgcatccattccatatcctaggagta
gctggtgtatttggaggttccttatttagtgcaatgcatggttcattagttacatcttca
cttcttgcagaaagtgcaggagatattagcctaaatgttggatataagtttggtcaagaa
gatgaaacttatagcatatcagcagcccatggttattttggtagactcatatttcaatat
gcttccttcaataactctcgtagtcttcacttcttcctagcagcttggccagttattggt
atttggttcacagcacttggagtaagtacaatggctttcaacttaaatggtttaaacttc
aaccaatccatcctagattccagtggccatctaatcttaagttgggcagatattgtgaat
cgtgctgatctaggtatggaagtgatgcatgaaagaaatgctcacaatttcccattagat
ctagcataa
>Hd6_psbA
atgaagaacacatcttactatcaactcaacttacttggtaatgtcataggattcgtacta
tccacaacaaatcgtctctacattgggtgctttggtatcctcatgtttcctcttttaact
ttagctactattgcttatatcacagcttttattcttgcacctgcagtagatattgatgga
ataagagaaccagtagctggctcacttctttatggcaataacattataacaggagctgta
ataccaagttctaatgctattggggttcatttctatccagtttgggagtcaaatggattt
gatgagtgcttatataatggtggtacatatcaatttgtagtacttcattttatgcttggt
gttgcttgttggatgggtagagaatgggaattcagtttcagattaggtatgagaccatgg
atatttgtagctttctcagcacctgttgtagcagcttttgcagtatttgttgtttatcca
attggtcaagctagcttttccgatggaatgcctttaggaataagtggaacttttaatttt
atgcttgttttccaagcagagcataacatcttaatgcatccattccatattctaggagta
gctggtgtatttggaggttccttatttagtgcaatgcatggttcattagttacatcttca
cttcttgcagaaagtgcaggagatattagcctaaatgttggatataagtttggtcaagaa
gatgaaacttatagcatatcagcagcccatggttattttggtagactcatatttcaatat
gcttccttcaataactctcgtagtcttcacttcttcctagcagcttggccagttattggt
atttggttcacagcacttggagtaagtacaatggctttcaacttaaatggtttaaacttc
aaccaatccatcctagattccagtggccatctaatcttaagttgggcagatattgtgaat
cgtgctgatctaggtatggaagtgatgcatgaaagaaatgctcacaatttcccattagat
ctagcataa

>Hd7_psbA
atgaagaatagatcttactatcaactcaacttacttggtaatgtcataggattcgtacta
tccacaacaaatcgtctctatattggatgctttggaatcctaatgtttcctcttttaact
ttagcaactattgcttatatcaccgcttttattcttgcacctgcagtagatattgatgga
ataagagaaccagtagctggctcacttctttatggcaataacattataacaggagctgta
ataccaagttctaatgctattggggttcatttctatccagtttgggagtcaaatggattt
gatgagtgcttatataatggtggtacatatcaattcgtagtccttcattttatgcttggt
gttgcttgttggatgggtagagaatgggaattcagtttcagattaggtatgagaccatgg
atatttgtagctttctcagcacctgttgtagcagcttttgcagtatttgttgtttatcca
attggtcaagctagcttttctgatggaatgcctttaggaataagtggtacttttaatttt
atgcttgttttccaagcagagcataacattttaatgcatccattccatatcctaggagta
gctggtgtatttggaggttccttatttagtgcaatgcatggttcattagttacatcttca
cttcttgcagaaagtgcaggagatattagcctaaatgttggatataagtttggtcaagaa
gatgaaacttatagcatatcagcagcccatggttattttggtagactcatatttcaatat
gcttccttcaataactctcgtagtcttcacttctgcctagcagcttggccagttattggt
atttggttcactgcccttggagtaagtacaatggctttcaacttaaatggtttaaacttc
aaccaatccatcctagattccagtggccatctaatcttaagttgggcagatattgtgaat
cgtgctgatctaggtatggaagtgatgcatgaaagaaatgctcacaatttcccattagat
ctagcataa
>Hd8_psbA
atgaagaatagatcttactatcaactcaacttacttggtaatgtcataggattcgtacta
tccacaacaaatcgtctctacattgggtgctttggtatcctaatgttccctcttttaact
ttagcaactattgcttatatcacagcttttattcttgcacctgcagtagatattgatgga
ataagagaaccagtagctggctcacttctttatggcaataacattataacaggagctgta
ataccaagttctaatgctattggggttcatttctatccagtttgggagtcaaatggattt
gatgagtgcttatataatggtggtacatatcaatttgtagtacttcattttatgcttggt
gttgcttgttggatgggtagagaatgggaattcagtttcagattaggtatgagaccatgg
atatttgtagctttctcagcacctgttgtagcagcttttgcagtatttgttgtttatcca
attggtcaagctagcttttccgatggaatgcctttaggaataagtggaacttttaatttt
atgcttgtgttccaagcagaacataacattttaatgcatccattccatattctaggagta
gctggagtatttggaggttccttatttagtgcaatgcatggttcattagttacatcttca
cttcttgcagaaagtgcaggagatattagcctaaatgttggatataagtttggtcaagaa
gatgaaacttatagcatatcagcagcccatggttattttggtagactcatatttcaatat
gcttccttcaataactctcgtagtcttcacttcttcctagcagcttggccagttattggt
atttggttcacagcacttggagtaagtacaatggctttcaacttaaatggtttaaacttc
aaccaatccatcctagattccagtggccatctaatcttaagttgggcagatattgtgaat
cgtgctgatctaggtatggaagtgatgcatgaaagaaatgctcacaatttcccattagat
ctagcataa
>Hd9_psbA
atgaagaatagatcttactatcaactcaacttacttggtaatgtcataggatttgtgcta
tccacaacaaatcgtctctatattggatgctttggaatcctcatgttccctcttttaact
ttagcaactattgcttatatcacagcttttattcttgcacctgcagtagatattgatgga
ataagagaaccagtagctggctcacttctttatggcaacaacattataacaggagctgta
atcccgagttctaatgctattggtgttcatttctatccagtctgggaatcaaaccatttc
gatgagtgcttatataatggtggaacatatcaatttgtagtacttcattttatgcttggt
gttgcttgttggatgggtagagaatgggaattcagtttcagattaggtatgagaccatgg
atatttgtagctttctcagcacctgttgtagcagcttttgcagtatttgttgtttatcca
attggtcaagctagcttttctgatggaatgcctttaggaataagtggaacttttaatttt
atgcttgtgttccaagcagagcataacattttaatgcatccattccatattctaggagta
gctggagtatttggtggatcattgttcagtgcaatgcatggttcattagttacatcttca
cttcttgcagaaagtgcaggagatattagcctcaatgttggatataagtttggtcaagaa
gatgaaacttatagcatatcagcagctcatggttattttggtagactcatatttcaatat
gcttccttcaataactctcgtagtcttcacttcttcctagcagcttggccagttattggt
atttggttcactgcccttggagtaagtacaatggctttcaacttaaatggtttaaacttc
aaccaatccatcctagattccagtggccatctaatcttaagttgggcagatattgtgaat
cgtgctgatctaggtatggaagtgatgcatgaaagaaatgctcacaatttcccattagat
ctagcataa

>IS1_psbA
atgaagaatagatcttactatcaactcaacttacttggtaatgtcataggatttgtgcta
tccacaacaaatcgtctctatattggatgctttggaatcctcatgtttcctcttttaact
ttagctactattgcttatatcaccgctttcattcttgcacctgcagtagatattgatgga
ataagagaaccagtagctggctcacttctttatggcaacaacattataacaggagctgta
atcccgagttctaatgctattggtgttcatttctatccagtctgggaatcaaaccatttc
gatgagtgcttatataatggtggaacatatcaattcgtagtccttcattttatgcttggt
gttgcttgttggatgggtagagaatgggaattcagttttagattaggtatgagaccatgg
atatttgtagctttctcagcacctgttattgcagcctttgcagtatttgttgtttacccg
atcggtcaagctagcttttctgatggaatgcctttaggaataagtggtacttttaatttt
atgcttgttttccaagcagagcataacatcttaatgcatccattccacatcttaggagta
gctggagtatttggtggatcattatttagtgcaatgcatggttccctagttacatcttca
cttcttgcagaaagtgcaggagatattagcctcaatgttggttataagtttggacaagaa
gatgaaacttatagcatatcagccgctcatggttattttggtagactcatatttcaatat
gcttccttcaataactctcgtagccttcacttctttctagcagcttggccagttattggt
atttggttcactgcccttggagtaagtacaatggctttcaacttaaatggtttaaacttc
aaccaatccatcctagattccagtggccatctaatcttaagttgggcagatattgtgaat
cgtgctgatctaggtatggaagtaatgcatgaaagaaacgctcataatttcccattagat
ctagcataa
>IS2_psbA
atgaagaacacatcttactatcaactcaacttacttggtaatgtcataggattcgtacta
tccacaacaaatcgtctctacattgggtgctttggtatcctaatgttccctcttttaact
ttagcaactattgcttatatcacagcttttattcttgcacctgcagtagatattgatgga
ataagagaaccagtagctggatcacttctttatggcaataacattataacaggagctgta
ataccaagttctaatgctattggggttcatttctatccagtttgggagtcaaatggattt
gatgagtgcttatataatggtggtacatatcaattcgtagtccttcattttatgcttggt
gttgcttgttggatgggtagagaatgggaattcagtttcagattaggtatgagaccatgg
atatttgtagctttctcagcacctgttgtagcagcttttgcagtatttgttgtttatcca
attggtcaagctagcttttccgatggaatgcctttaggaataagtggaacttttaatttt
atgcttgtgttccaggcagaacataacattttaatgcatccattccacatcttaggagta
gctggtgtatttggaggttccttatttagtgcaatgcatggttcattagttacatcttca
cttcttgcagaaagtgcaggagatattagcctaaatgttggatataagtttggtcaagaa
gatgaaacttatagcatatcagcagcccatggttattttggtagactcatatttcaatat
gcttccttcaataactctcgtagtcttcacttcttcctagcagcttggccagttattggt
atttggttcacagcacttggagtaagtacaatggctttcaacttaaatggtttaaacttc
aaccaatccatcctagattccagtggccatctaatcttaagttgggcagatattgtgaat
cgtgctgatctaggtatggaagtgatgcatgaaagaaatgctcacaatttcccattagat
ctagcataa
>IS3_psbA
atgaagaatagatcttactatcaactcaacttacttggtaatgtcataggatttgtacta
tctacaacaaatcgtctctacattggatgctttggtatcctaatgtttcctcttttaact
ttagcaactattgcttatatcgcagctttcattcttgcacctgcagtagatattgatgga
ataagagaaccagtagctggatcacttctttatggtaataacattataactggagccgta
attccaagttctaatgctattggagttcatttttatccagtttgggagtcaaatggattc
gatgagtgcttatataatggtggaacatatcaatttgtagtacttcatttcatgcttggt
gtggcttgttggatgggtagagaatgggaattcagtttcagattaggtatgagaccatgg
atatttgtagctttctcagcacctgttgtagcagcttttgcagtatttgtagtctatcca
attgggcaagctagcttttccgatggaatgcctttaggaataagtggaacctttaatttt
atgcttgtattccaggcagagcataacatcttaatgcatccattccacatcttaggagta
gctggagtatttggtggatcattatttagtgcaatgcatggttcattagttacatcttca
cttcttgcagaaagtgcaggagatattagcctcaatgttggttataagtttggacaagaa
gatgaaacttatagcatatcagcagctcatggttattttggtagacttatattccaatat
gcctccttcaataactctcgtagtcttcatttcttcttagcagcttggccagttattggt
atttggtttactgcacttggagtaagcacaatggctttcaacttaaatggtttaaacttc
aaccaatccatcctagattccagtggccatctaattttaagctgggcagatattgtgaat
cgtgctgatctaggtatggaagtaatgcatgaaagaaacgctcataatttcccattagat
ctagcataa

>IS4_psbA
atgaagaacacatcttactatcaactcaacttacttggtaatgtcataggattcgtacta
tctacaacaaatcgtctctacattgggtgctttggtatcctaatgttccctcttttaact
ttagcaactattgcttatatcgcagctttcattcttgcacctgcagtagatattgatgga
ataagagaaccagtagctggatcacttctttatggtaataacattataactggagccgta
attccaagttctaatgctattggagttcatttttatccagtttgggagtcaaatggattc
gatgagtgcttatataatggtggaacatatcaatttgtagtacttcatttcatgcttggt
gtggcttgttggatgggtagagaatgggaattcagtttcagattaggtatgagaccatgg
atatttgtagctttctcagcacctgttgtagcagcttttgcagtatttgtagtctatcca
attgggcaagctagcttttccgatggaatgcctttaggaataagtggaacctttaatttt
atgcttgtattccaggcagagcataacattttaatgcatccattccacatcttaggagta
gctggtgtatttggaggatccttatttagtgcaatgcatggttccctagtaacatcttcc
cttcttgcagaaactgcaggagatattagcctaaatgttggttataagtttggacaagaa
gatgaaacttatagcatatcagcagcccatggttattttggtagacttatattccaatat
gcctccttcaataactctcgtagtcttcatttcttcttagcagcttggccagttattggt
atttggtttacagcacttggagtaagcacaatggctttcaacttaaatggtttaaacttc
aaccaatccatcctagattccagtggccatctaattttaagctgggcagatattgtgaat
cgtgctgatctaggtatggaagtaatgcatgaaagaaacgctcataatttcccattagat
ctagcataa
>IS5_psbA
atgaagaacacatcttactatcaactcaacttacttggtaatgtcataggattcgtacta
tccacaacaaatcgtctctacattgggtgctttggtatcctaatgttccctcttttaact
ttagcaactattgcttatatcacagcttttattcttgcacctgcagtagatattgatgga
ataagagaaccagtagctggctcacttctttatggcaataacattataacaggagctgta
ataccaagttctaatgctattggggttcatttctatccagtttgggagtcaaaccatttc
gatgagtgcttatataatggtggtacatatcaatttgtagtacttcattttatgcttggt
gttgcttgttggatgggtagagaatgggaattcagttttagattaggtatgagaccatgg
atatttgtagctttctcagcacctgttgtagcagcttttgcagtatttgttgtttatcca
attggtcaagctagcttttccgatggaatgcctttaggaataagtggaacttttaatttt
atgcttgtgttccaagcagaacataacattttaatgcatccattccatattctaggagta
gctggtgtatttggaggttccttatttagtgcaatgcatggttcattagttacatcttca
cttcttgcagaaagtgcaggagatattagcctcaatgttggatataagtttggtcaagaa
gatgaaacttatagcatatcagcagctcatggttattttggtagactcatatttcaatat
gcttccttcaataactctcgtagtcttcacttcttcctagcagcttggccagttattggt
atttggttcacagcacttggagtaagtacaatggctttcaacttaaatggtttaaacttc
aaccaatccatcctagattccagtggccatctaatcttaagttgggcagatattgtgaat
cgtgctgatctaggtatggaagtgatgcatgaaagaaatgctcacaatttcccattagat
ctagcataa
>IS6_psbA
atgaagaatagatcttactatcaactcaacttacttggtaatgtcataggattcgtacta
tccacaacaaatcgtctctacattgggtgctttggtatcctaatgttccctcttttaact
ttagcaactattgcttatatcacagcttttattcttgcacctgcagtagatattgatgga
ataagagaaccagtagctggctcacttctttatggcaataacattataacaggagctgta
ataccaagttctaatgctattggtgttcatttctatccagtttgggagtcaaatggattt
gatgagtgcttatataatggtggtacatatcaatttgtagtacttcattttatgcttggt
gttgcttgttggatgggtagagaatgggaattcagtttcagattaggtatgagaccatgg
atatttgtagctttctcagcacctgttgtagcagcttttgcagtatttgttgtttatcca
attggtcaagctagcttttccgatggaatgcctttaggaataagtggaacttttaatttt
atgcttgtgttccaagcagaacataacattttaatgcatccattccatatcctaggagta
gctggtgtatttggaggttccttatttagtgcaatgcatggttcattagttacatcttca
cttcttgcagaaagtgcaggagatattagcctaaatgttggatataagtttggtcaagaa
gatgaaacttatagcatatcagcagcccatggttattttggtagactcatatttcaatat
gcttccttcaataactctcgtagtcttcacttcttcctagcagcttggccagttattggt
atttggttcacagcacttggagtaagtacaatggctttcaacttaaatggtttaaacttc
aaccaatccatcctagattccagtggccatctaatcttaagttgggcagatattgtgaat
cgtgctgatctaggtatggaagtgatgcatgaaagaaatgctcacaatttcccattagat
ctagcataa

>IS7_psbA
atgaagaatagatcttactatcaactcaacttacttggtaatgtcataggattcgtgcta
tccacaacaaatcgtctctacattgggtgctttggaatcctcatgtttcctcttttaact
ttagctactattgcttatatcaccgctttcattcttgcacctgcagtagatattgatgga
ataagagaaccagtagctggatcacttctttatggcaacaacattataacaggagctgta
atcccgagttctaatgctattggtgttcatttctatccagtctgggagtcaaatggattc
gatgagtgcttatataatggtggtacatatcaatttgtagtacttcattttatgcttggt
gttgcttgttggatgggtagagaatgggaattcagtttcagattaggtatgagaccatgg
atatttgtagctttctcagcacctgttgtagcagcctttgcagtatttgtagtctatcca
attgggcaagctagcttttccgatggaatgcctttaggaataagtggaacctttaatttt
atgcttgtattccaggcagaacataacattttaatgcatccattccacatcttaggagta
gctggtgtatttggtggatcattatttagtgcaatgcatggttcattagttacatcttca
cttcttgcagaaagtgcaggagatattagcctaaatgttggatataagtttggtcaagaa
gatgaaacttatagcatatcagccgctcatggttattttggtagacttatattccaatat
gcctccttcaataactctcgtagtcttcatttcttcttagcagcttggccagttattggt
atttggttcacagcacttggagtaagtacaatggctttcaacttaaatggtttaaacttc
aaccaatccatcctagattccagtggccatctaattttaagctgggcagatattgtgaat
cgtgctgatctaggtatggaagtgatgcatgaaagaaacgctcacaatttcccattagat
ctagcataa
>IS8_psbA
atgaagaacacatcttactatcaactcaacttacttggtaatgtcataggattcgtacta
tccacaacaaatcgtctctacattgggtgctttggtatcctaatgttccctcttttaact
ttagcaactattgcttatatcacagcttttattcttgcacctgcagtagatattgatgga
ataagagaaccagtagctggctcacttctttatggcaataacattataacaggagctgta
ataccaagttctaatgctattggggttcatttctatccagtttgggagtcaaaccatttt
gatgagtgcttatataatggtggtacatatcaatttgtagtacttcattttatgcttggt
gttgcttgttggatgggtagagaatgggaattcagtttcagattaggtatgagaccatgg
atatttgtagctttctcagcacctgttgtagcagcttttgcagtatttgttgtttatcca
attggtcaagctagcttttccgatggaatgcctttaggaataagtggaacttttaatttt
atgcttgtgttccaagcagaacataacattttaatgcatccattccatatcctaggagta
gctggtgtatttggaggttccttatttagtgcaatgcatggttcattagttacatcttca
cttcttgcagaaagtgcaggagatattagcctaaatgttggatataagtttggtcaagaa
gatgaaacttatagcatatcagcagcccatggttattttggtagactcatatttcaatat
gcttccttcaataactctcgtagtcttcacttcttcctagcagcttggccagttattggt
atttggttcacagcacttggagtaagtacaatggctttcaacttaaatggtttaaacttc
aaccaatccatcctagattccagtggccatctaatcttaagttcggcagatattgtgaat
cgtgctgatctaggtatggaagtgatgcatgaaagaaatgctcacaatttcccattagat
ctagcataa
>IS9_psbA
atgaagaacacatcttactatcaactcaacttacttggtaatgtcataggattcgtacta
tccacaacaaatcgtctctacattgggtgctttggtatcctaatgttccctcttttaact
ttagcaactattgcttatatcgcagcttttattcttgcacctgcagtagatattgatgga
ataagagaaccagtagctggatcacttctttatggtaataacattataacaggagctgta
ataccaagttctaatgctattggggttcatttttatccagtttgggagtcaaatggattc
gatgagtgcttatataatggtggaacatatcaatttgtagtacttcattttatgcttggt
gttgcttgttggatgggtagagaatgggaattcagtttcagattaggtatgagaccatgg
atatttgtagctttctcagcacctgttgtagcagcttttgcagtatttgttgtttatcca
attggtcaagctagcttttccgatggaatgcctttaggaataagtggaacttttaatttt
atgcttgtattccaggcagaacataacattttaatgcatccattccatattctaggagta
gctggagtatttggtggatcattgttcagtgcaatgcatggttccctagtaacatcttcc
cttcttgcagaaactgcaggagatattagcctcaatgttggttataagtttggacaagaa
gatgaaacttatagcatatcagcagctcatggttattttggtagacttatattccaatat
gcctccttcaataactctcgtagtcttcatttcttcttagcagcttggccagttattggt
atttggtttacagcacttggagtaagcacaatggctttcaacttaaatggtttaaacttc
aaccaatccatcctagattccagtggccatctaattttaagctgggcagatattgtgaat
cgtgctgatctaggtatggaagtaatgcatgaaagaaacgctcataatttcccattagat
ctagcataa

>Ik1_psbA
atgaagaatagatcttactatcaactcaacttacttggtaatgtcataggatttgtgcta
tccacaacaaatcgtctctacattgggtgctttggtatcctaatgttccctcttttaact
ttagcaactattgcttatatcacagcttttattcttgcacctgcagtagatattgatgga
ataagagaaccagtagctggctcacttctttatggcaataacattataacaggagctgta
ataccaagttctaatgctattggggttcatttctatccagtctgggaatcaaaccatttc
gatgagtgcttatataatggtggtacatatcaatttgtagtacttcattttatgcttggt
gttgcttgttggatgggtagagaatgggaattcagttttagattaggtatgagaccatgg
atatttgtagctttctcagcacctgttgtagcagcttttgcagtatttgttgtttatcca
attggtcaagctagcttttccgatggaatgcctttaggaataagtggaacttttaatttt
atgcttgtgttccaagcagaacataacattttaatgcatccattccatatcctaggagta
gctggtgtatttggaggttccttatttagtgcaatgcatggttcattagttacatcttca
cttcttgcagaaagtgcaggagatattagcctaaatgttggatataagtttggtcaagaa
gatgaaacttatagcatatcagcagcccatggttattttggtagactcatatttcaatat
gcttccttcaataactctcgtagtcttcacttcttcctagcagcttggccagttattggt
atttggttcacagcacttggagtaagtacaatggctttcaacttaaatggtttaaacttc
aaccaatccatcctagattccagtggccatctaatcttaagttgggcagatattgtgaat
cgtgctgatctaggtatggaagtgatgcatgaaagaaatgctcacaatttcccattagat
ctagcataa
>Ik2_psbA
atgaagaacacatcttactatcaactcaacttacttggtaatgtcataggattcgtgcta
tccacaacaaatcgtctctatattggatgctttggaatcctcatgtttcctcttttaact
ttagcaactattgcttatatcacagcttttattcttgcacctgcagtagatattgatgga
ataagagaaccagtagctggctcacttctttatggcaataacattataacaggagctgta
ataccaagttctaatgctattggggttcatttctatccagtttgggagtcaaatggattt
gatgagtgcttatataatggtggtacatatcaatttgtagtacttcattttatgcttggt
gttgcttgttggatgggtagagaatgggaattcagtttcagattaggtatgagaccatgg
atatttgtagctttctcagcacctgttgtagcagcttttgcagtatttgttgtttatcca
attggtcaagctagcttttccgatggaatgcctttaggaataagtggaacttttaatttt
atgcttgtgttccaagcagaacataacattttaatgcatccattccatattctaggagta
gctggtgtatttggaggttccttatttagtgcaatgcatggttcattagttacatcttca
cttcttgcagaaagtgcaggagatattagcctaaatgttggatataagtttggtcaagaa
gatgaaacttatagcatatcagcagcccatggttattttggtagactcatatttcaatat
gcttccttcaataactctcgtagtcttcacttcttcctagcagcttggccagttattggt
atttggttcacagcacttggagtaagtacaatggctttcaacttaaatggtttaaacttc
aaccaatccatcctagattccagtggccatctaatcttaagttgggcagatattgtgaat
cgtgctgatctaggtatggaagtgatgcatgaaagaaatgctcacaatttcccattagat
ctagcataa
>Ik3_psbA
atgaagaacacatcttactatcaactcaacttacttggtaatgtcataggattcgtacta
tccacaacaaatcgtctctacattgggtgctttggtatcctaatgttccctcttttaact
ttagcaactattgcttatatcacagcttttattcttgcacctgcagtagatattgatgga
ataagagaaccagtagctggctcacttctttatggcaataacattataacaggagctgta
ataccaagttctaatgctattggggttcatttctatccagtttgggagtcaaaccatttc
gatgagtgcttatataatggtggtacatatcaatttgtagtacttcattttatgcttggt
gttgcttgttggatgggtagagaatgggaattcagtttcagattaggtatgagaccatgg
atatttgtagctttctcagcacctgttgtagcagcttttgcagtatttgttgtttatccg
atcggtcaagctagcttttctgatggaatgcctttaggaataagtggaacttttaatttt
atgcttgtgttccaagcagaacataacattttaatgcatccattccatatcctaggagta
gctggtgtatttggaggttccttatttagtgcaatgcatggttcattagttacatcttca
cttcttgcagaaagtgcaggagatattagcctaaatgttggatataagtttggtcaagaa
gatgaaacttatagcatatcagcagcccatggttattttggtagactcatatttcaatat
gcttccttcaataactctcgtagtcttcacttcttcctagcagcttggccagttattggt
atttggttcacagcacttggagtaagtacaatggctttcaacttaaatggtttaaacttc
aaccaatccatcctagattccagtggccatctaatcttaagttgggcagatattgtgaat
cgtgctgatctaggtatggaagtgatgcatgaaagaaatgctcacaatttcccattagat
ctagcataa

>Ik4_psbA
atgaagaacacatcttactatcaactcaacttacttggtaatgtcataggattcgtacta
tccacaacaaatcgtctctatattggatgctttggaatcctcatgtttcctcttttaact
ttagctactattgcttatatcacagcttttattcttgcacctgcagtagatattgatgga
ataagagaaccagtagctggctcacttctttatggcaataacattataacaggagctgta
atcccgagttctaatgctattggggttcatttctatccagtttgggagtcaaatggattt
gatgagtgcttatataatggtggtacatatcaatttgtagtacttcattttatgcttggt
gttgcttgttggatgggtagagaatgggaattcagtttcagattaggtatgagaccatgg
atatttgtagctttctcagcacctgttgtagcagcttttgcagtatttgttgtttatcca
attggtcaagctagcttttccgatggaatgcctttaggaataagtggaacttttaatttt
atgcttgtgttccaagcagaacataacattttaatgcatccattccatatcctaggagta
gctggtgtatttggaggttccttatttagtgcaatgcatggttcattagttacatcttca
cttcttgcagaaagtgcaggagatattagcctaaatgttggatataagtttggtcaagaa
gatgaaacttatagcatatcagcagcccatggttattttggtagactcatatttcaatat
gcttccttcaataactctcgtagtcttcacttcttcctagcagcttggccagttattggt
atttggttcacagcacttggagtaagtacaatggctttcaacttaaatggtttaaacttc
aaccaatccatcctagattccagtggccatctaatcttaagttgggcagatattgtgaat
cgtgctgatctaggtatggaagtgatgcatgaaagaaatgctcacaatttcccattagat
ctagcataa
>Ik5_psbA
atgaagaacacatcttactatcaactcaacttacttggtaatgtcataggattcgtacta
tccacaacaaatcgtctctacattgggtgctttggtatcctaatgttccctcttttaact
ttagcaactattgcttatatcaccgcttttattcttgcacctgcagtagatattgatgga
ataagagaaccagtagctggctcacttctttatggcaataacattataacaggagctgta
ataccaagttctaatgctattggggttcatttctatccagtttgggagtcaaatggattt
gatgagtgcttatataatggtggtacatatcaatttgtagtacttcattttatgcttggt
gttgcttgttggatgggtagagaatgggaattcagttttagattaggtatgagaccatgg
atatttgtagctttctcagcacctgttgtagcagcttttgcagtatttgttgtttatcca
attggtcaagctagcttttccgatggaatgcctttaggaataagtggaacttttaatttt
atgcttgtgttccaagcagaacataacattttaatgcatccattccatatcctaggagta
gctggtgtatttggaggttccttatttagtgcaatgcatggttcattagttacatcttca
cttcttgcagaaagtgcaggagatattagcctaaatgttggatataagtttggtcaagaa
gatgaaacttatagcatatcagcagcccatggttattttggtagactcatatttcaatat
gcttccttcaataactctcgtagtcttcacttcttcctagcagcttggccagttattggt
atttggttcacagcacttggagtaagtacaatggctttcaacttaaatggtttaaacttc
aaccaatccatcctagattccagtggccatctaatcttaagttgggcagatattgtgaat
cgtgctgatctaggtatggaagtgatgcatgaaagaaatgctcacaatttcccattagat
ctagcataa
>Ik6_psbA
atgaagaatagatcttactatcaactcaacttacttggtaatgtcataggatttgtgcta
tccacaacaaatcgtctctacattgggtgctttggtatcctaatgtttcctcttttaact
ttatctactattgcttatatcaccgctttcattcttgcacctgcagtagatattgatgga
ataagagaaccagtagctggctcacttctttatggcaataacattataacaggagctgta
ataccaagttctaatgctattggggttcatttctatccagtctgggaatcaaaccatttc
gatgagtgcttatataatggtggaacatatcaattcgtagtccttcattttatgcttggt
gttgcttgttggatgggtagagaatgggaattcagttttagattaggtatgagaccatgg
atatttgtagctttctcagcacctgttattgcagcctttgcagtatttgttgtttacccg
atcggtcaagctagcttttctgatggaatgcctttaggaataagtggtacttttaatttt
atgcttgtgttccaagcagaacataacattttaatgcatccattccatatcctaggagta
gctggtgtatttggaggttccttatttagtgcaatgcatggttcattagttacatcttca
cttcttgcagaaagtgcaggagatattagcctcaatgttggatataagtttggtcaagaa
gatgaaacttatagcatatcagcagcccatggttattttggtagactcatatttcaatat
gcttccttcaataactctcgtagtcttcacttcttcctagcagcttggccagttattggt
atttggttcacagcacttggagtaagtacaatggctttcaacttaaatggtttaaacttc
aaccaatccatcctagattccagtggccatctaatcttaagttgggcagatattgtgaat
cgtgctgatctaggtatggaagtgatgcgtgaaagaaatgctcataatttcccattagat
ctagcataa

>Ik7_psbA
atgaagaacacatcttactatcaactcaacttacttggtaatgtcataggattcgtacta
tccacaacaaatcgtctctacattgggtgctttggtatcctaatgttccctcttttaact
ttagctactattgcttatatcacagcttttattcttgcacctgcagtagatattgatgga
ataagagaaccagtagctggctcacttctttatggcaataacattataacaggagctgta
ataccaagttctaatgctattggggttcatttctatccagtctgggaatcaaaccatttc
gatgagtgcttatataatggtggtacatatcaatttgtagtacttcattttatgcttggt
gttgcttgttggatgggtagagaatgggaattcagtttcagattaggtatgagaccatgg
atatttgtagctttctcagcacctgttgtagcagcttttgcagtatttgttgtttacccg
attggtcaagctagcttttctgatggaatgcctttaggaataagtggaacttttaatttt
atgcttgtgttccaagcagaacataacattttaatgcatccattccatatcctaggagta
gctggtgtatttggaggttccttatttagtgcaatgcatggttcattagttacatcttca
cttcttgcagaaagtgcaggagatattagcctcaatgttggatataagtttggtcaagaa
gatgaaacttatagcatatcagcagcccatggttattttggtagactcatatttcaatat
gcttccttcaataactctcgtagccttcacttctttctagcagcttggccagttattggt
atttggttcactgcacttggagtaagtacaatggctttcaacttaaatggtttaaacttc
aaccaatccatcctagattccagtggccatctaatcttaagttgggcagatattgtgaat
cgtgctgatctaggtatggaagtgatgcatgaaagaaatgctcacaatttcccattagat
ctagcataa
>Ik8_psbA
atgaagaatagatcttactatcaactcaacttacttggtaatgtcataggatttgtgcta
tccacaacaaatcgtctctatattggatgctttggaatcctcatgtttcctcttttaact
ttagctactattgcttatatcacagcttttattcttgcacctgcagtagatattgatgga
ataagagaaccagtagctggctcacttctttatggcaacaacattataacaggagctgta
ataccaagttctaatgctattggggttcatttctatccagtttgggagtcaaaccatttc
gatgagtgcttatataatggtggaacatatcaattcgtagtccttcattttatgcttggt
gttgcttgttggatgggtagagaatgggaattcagttttagattaggtatgagaccatgg
atatttgtagctttctcagcacctgttattgcagcctttgcagtatttgttgtttacccg
atcggtcaagctagcttttctgatggaatgcctttaggaataagtggtacttttaatttt
atgcttgttttccaagcagaacataacattttaatgcatccattccatatcctaggagta
gctggagtatttggtggttccttgttcagtgcaatgcatggttcattagttacatcttca
cttcttgcagaaagtgcaggagatattagcctcaatgttggatataagtttggtcaagaa
gatgaaacttatagcatatcagcagcccatggttattttggtagactcatatttcaatat
gcttccttcaataactctcgtagtcttcacttcttcctagcagcttggccagttattggt
atttggttcacagcacttggagtaagtacaatggctttcaacttaaatggtttaaacttc
aaccaatccatcctagattccagtggccatctaatcttaagttgggcagatattgtgaat
cgtgctgatctaggtatggaagtgatgcatgaaagaaatgctcacaatttcccattagat
ctagcataa
>Ik9_psbA
atgaagaacacatcttactatcaactcaacttacttggtaatgtcataggattcgtacta
tccacaacaaatcgtctctatattggatgctttggaatcctcatgtttcctcttttaact
ttagcaactattgcttatatcacagcttttattcttgcacctgcagtagatattgatgga
ataagagaaccagtagctggctcacttctttatggcaataacattataacaggagctgta
ataccaagttctaatgctattggggttcatttctatccagtctgggaatcaaaccatttc
gatgagtgcttatataatggtggtacatatcaatttgtagtacttcattttatgcttggt
gttgcttgttggatgggtagagaatgggaattcagtttcagattaggtatgagaccatgg
atatttgtagctttctcagcacctgttattgcagcctttgcagtatttgttgtttacccg
atcggtcaagctagcttttccgatggaatgcctttaggaataagtggaacttttaatttt
atgcttgtgttccaagcagaacataacattttaatgcatccattccatatcctaggagta
gctggtgtatttggaggatccttatttagtgcaatgcatggttcattagttacatcttca
cttcttgcagaaagtgcaggagatattagcctaaatgttggatataagtttggtcaagaa
gatgaaacttatagcatatcagcagcccatggttattttggtagactcatatttcaatat
gcttccttcaataactctcgtagtcttcacttcttcctagcagcttggccagttattggt
atttggttcacagcccttggagtaagtacaatggctttcaacttaaatggtttaaacttc
aaccaatccatcctagattccagtggccatctaatcttaagttgggcagatattgtgaat
cgtgctgatctaggtatggaagtgatgcatgaaagaaatgctcacaatttcccattagat
ctagcataa

>Irm10_psbA
atgaagaacacatcttactatcaactcaacttacttggtaatgtcataggattcgtacta
tccacaacaaatcgtctctacattgggtgctttggtatcctaatgttccctcttttaact
ttagcaactattgcttatatcacagcttttattcttgcacctgcagtagatattgatgga
ataagagaaccagtagctggctcacttctttatggcaataacattataacaggagctgta
ataccaagttctaatgctattggggttcatttctatccagtttgggagtcaaatggattt
gatgagtgcttatataatggtggtacatatcaatttgtagtacttcattttatgcttggt
gttgcttgttggatgggtagagaatgggaattcagttttagattaggtatgagaccatgg
atatttgtagctttctcagcacctgttattgcagcttttgcagtatttgttgtttatcca
attggtcaagctagcttttccgatggaatgcctttaggaataagtggaacttttaatttt
atgcttgtgttccaagcagaacataacattttaatgcatccattccatatcctaggagta
gctggagtatttggtggatcattgttcagtgcaatgcatggttcattagttacatcttca
cttcttgcagaaagtgcaggagatattagcctaaatgttggatataagtttggtcaagaa
gatgaaacttatagcatatcagccgctcatggttattttggtagactcatatttcaatat
gcttccttcaataactctcgtagtcttcacttcttcctagcagcttggccagttattggt
atttggttcacagcacttggagtaagtacaatggctttcaacttaaatggtttaaacttc
aaccaatccatcctagattccagtggccatctaatcttaagttgggcagatattgtgaat
cgtgctgatctaggtatggaagtgatgcgtgaaagaaatgctcataatttcccattagat
ctagcataa
>Irm17_psbA
atgaagaacacatcttactatcaactcaacttacttggtaatgtcataggattcgtacta
tccacaacaaatcgtctctacattgggtgctttggtatcctaatgttccctcttttaact
ttagcaactattgcttatatcaccgctttcattcttgcacctgcagtagatattgatgga
ataagagaaccagtagctggctcacttctttatggcaataacattataacaggagctgta
ataccaagttctaatgctattggggttcatttctatccagtttgggagtcaaatggattt
gatgagtgcttatataatggtggtacatatcaatttgtagtacttcattttatgcttggt
gttgcttgttggatgggtagagaatgggaattcagtttcagattaggtatgagaccatgg
atatttgtagctttctcagcacctgttgtagcagcttttgcagtatttgttgtttatcca
attggtcaagctagcttttccgatggaatgcctttaggaataagtggaacttttaatttt
atgcttgtgttccaagcagaacataacattttaatgcatccattccatatcctaggagta
gctggtgtatttggaggttccttatttagtgcaatgcatggttcattagttacatcttca
cttcttgcagaaagtgcaggagatattagcctaaatgttggatataagtttggtcaagaa
gatgaaacttatagcatatcagcagcccatggttattttggtagactcatatttcaatat
gcttccttcaataactctcgtagtcttcacttcttcctagcagcttggccagttattggt
atttggttcacagcacttggagtaagtacaatggctttcaacttaaatggtttaaacttc
aaccaatccatcctagattccagtggccatctaatcttaagttgggcagatattgtgaat
cgtgctgatctaggtatggaagtgatgcatgaaagaaatgctcacaatttcccattagat
ctagcataa
>Irm2_psbA
atgaagaacacatcttactatcaactcaacttacttggtaatgtcataggattcgtacta
tccacaacaaatcgtctctacattgggtgctttggtatcctaatgttccctcttttaact
ttagcaactattgcttatatcacagcttttattcttgcacctgcagtagatattgatgga
ataagagaaccagtagctggctcacttctttatggcaataacattataacaggagctgta
ataccaagttctaatgctattggggttcatttctatccagtttgggagtcaaatggattt
gatgagtgcttatataatggtggtacatatcaatttgtagtacttcattttatgcttggt
gttgcttgttggatgggtagagaatgggaattcagtttcagattaggtatgagaccatgg
atatttgtagctttctcagcacctgttgtagcagcttttgcagtatttgttgtttatcca
attggtcaagctagcttttccgatggaatgcctttaggaataagtggaacttttaatttt
atgcttgtgttccaagcagaacataacattttaatgcatccattccatatcctaggagta
gctggtgtatttggaggttccttatttagtgcaatgcatggttcattagttacatcttca
cttcttgcagaaagtgcaggagatattagcctaaatgttggatataagtttggtcaagaa
gatgaaacttatagcatatcagccgctcatggttattttggtagactcatatttcaatat
gcttccttcaataactctcgtagtcttcacttcttcctagcagcttggccagttattggt
atttggttcacagcacttggagtaagtacaatggctttcaacttaaatggtttaaacttc
aaccaatccatcctagattccagtggccatctaatcttaagttgggcagatattgtgaat
cgtgctgatctaggtatggaagtgatgcatgaaagaaatgctcacaatttcccattagat
ctagcataa

>Irm21_psbA
atgaagaacacatcttactatcaactcaacttacttggtaatgtcataggattcgtacta
tccacaacaaatcgtctctacattgggtgctttggtatcctaatgttccctcttttaact
ttagcaactattgcttatatcacagcttttattcttgcacctgcagtagatattgatgga
ataagagaaccagtagctggctcacttctttatggcaataacattataacaggagctgta
ataccaagttctaatgctattggggttcatttctatccagtttgggagtcaaatggattt
gatgagtgcttatataatggtggtacatatcaatttgtagtacttcattttatgcttggt
gttgcttgttggatgggtagagaatgggaattcagttttagattaggtatgagaccatgg
atatttgtagctttctcagcacctgttattgcagcctttgcagtatttgttgtttacccg
atcggtcaagctagcttttccgatggaatgcctttaggaataagtggaacttttaatttt
atgcttgtgttccaagcagaacataacattttaatgcatccattccatattctaggagta
gctggagtatttggtggatcattgttcagtgcaatgcatggttcattagttacatcttca
cttcttgcagaaagtgcaggagatattagcctaaatgttggatataagtttggtcaagaa
gatgaaacttatagcatatcagtagcccatggttattttggtagactcatatttcaatat
gcttccttcaataactctcgtagtcttcacttcttcctagcagcttggccagttattggt
atttggttcacagcacttggagtaagtacaatggctttcaacttaaatggtttaaacttc
aaccaatccatcctagattccagtggccatctaatcttaagttgggcagatattgtgaat
cgtgctgatctaggtatggaagtgatgcatgaaagaaatgctcacaatttcccattagat
ctagcataa
>Irm22_psbA
atgaagaatagatcttactatcaactcaacttacttggtaatgtcataggattcgtgcta
tccacaacaaatcgtctctacattgggtgctttggtatcctaatgttccctcttttaact
ttagcaactattgcttatatcacagcttttattcttgcacctgcagtagatattgatgga
ataagagaaccagtagctggctcacttctttatggcaataacattataacaggagctgta
atcccgagttctaatgctattggtgttcatttctatccagtctgggaatcaaaccatttc
gatgagtgcttatataatggtggaacatatcaattcgtagtccttcattttatgcttggt
gttgcttgttggatgggtagagaatgggaattcagttttagattaggtatgagaccatgg
atatttgtagctttctcagcacctgttattgcagcctttgcagtatttgttgtttacccg
atcggtcaagctagcttttctgatggaatgcctttaggaataagtggtacttttaatttt
atgcttgtgttccaagcagaacataacattttaatgcatccattccatatcctaggagta
gctggtgtatttggtggatcattgttcagtgcaatgcatggttcattagttacatcttca
cttcttgcagaaagtgcaggagatattagcctcaatgttggatataagtttggtcaagaa
gatgaaacttatagcatatcagcagcccatggttattttggtagactcatatttcaatat
gcttccttcaataactctcgtagtcttcacttcttcctagcagcttggccagttattggt
atttggttcactgcccttggagtaagtacaatggctttcaacttaaatggtttaaacttc
aaccaatccatcctagattccagtggccatctaatcttaagttgggcagatattgtgaat
cgtgctgatctaggtatggaagtgatgcatgaaagaaatgctcataatttcccattagat
ctagcataa
>Irm23_psbA
atgaagaatagatcttactatcaactcaacttacttggtaatgtcataggattcgtacta
tccacaacaaatcgtctctacattgggtgctttggtatcctaatgttccctcttttaact
ttagctactattgcttatatcacagcttttattcttgcacctgcagtagatattgatgga
ataagagaaccagtagctggctcacttctttatggcaataacattataacaggagctgta
ataccaagttctaatgctattggggttcatttctatccagtctgggaatcaaaccatttc
gatgagtgcttatataatggtggaacatatcaattcgtagtacttcattttatgcttggt
gttgcttgttggatgggtagagaatgggaattcagtttcagattaggtatgagaccatgg
atatttgtagctttctcagcacctgttattgcagcctttgcagtatttgttgtttatcca
attggtcaagctagcttttccgatggaatgcctttaggaataagtggaacttttaatttt
atgcttgttttccaagcagagcataacatcttaatgcatccattccatattctaggagta
gctggtgtatttggaggttccttatttagtgcaatgcatggttcattagttacatcttca
cttcttgcagaaagtgcaggagatattagcctaaatgttggatataagtttggtcaagaa
gatgaaacttatagcatatcagccgctcatggttattttggtagactcatatttcaatat
gcttccttcaataactctcgtagccttcacttctttctagcagcttggccagttattggt
atttggttcactgcccttggagtaagtacaatggctttcaacttaaatggtttaaacttc
aaccaatccatcctagattccagtggccatctaatcttaagttgggcagatattgtgaat
cgtgctgatctaggtatggaagtgatgcgtgaaagaaatgctcataatttcccattagat
ctagcataa

>Irm24_psbA
atgaagaatagatcttactatcaactcaacttacttggtaatgtcataggatttgtgcta
tccacaacaaatcgtctctatattggatgctttggaatcctcatgtttcctcttttaact
ttagctactattgcttatatcaccgctttcattcttgcacctgcagtagatattgatgga
ataagagaaccagtagctggctcacttctttatggcaacaacattataacaggagctgta
atcccgagttctaatgctattggtgttcatttctatccagtctgggaatcaaaccatttc
gatgagtgcttatataatggtggtacatatcaatttgtagtacttcattttatgcttggt
gttgcttgttggatgggtagagaatgggaattcagtttcagattaggtatgagaccatgg
atatttgtagctttctcagcacctgttgtagcagcttttgcagtatttgttgtttatcca
attggtcaagctagcttttctgatggaatgcctttaggaataagtggtacttttaatttt
atgcttgtgttccaagcagaacataacattttaatgcatccattccatatcctaggagta
gctggtgtatttggaggatccttatttagtgcaatgcatggttcattagttacatcttca
cttcttgcagaaagtgcaggagatattagcctaaatgttggatataagtttggtcaagaa
gatgaaacttatagcatatcagcagcccatggttattttggtagactcatatttcaatat
gcttccttcaataactctcgtagccttcacttctttctagcagcttggccagttattggt
atttggttcactgcccttggagtaagtacaatggctttcaacttaaatggtttaaacttc
aaccaatccatcctagattccagtggccatctaatcttaagttgggcagatattgtgaat
cgtgctgatctaggtatggaagtgatgcgtgaaagaaatgctcataatttcccattagat
ctagcataa
>Irm25_psbA
atgaagaatagatcttactatcaactcaacttacttggtaatgtcataggatttgtgcta
tccacaacaaatcgtctctatattggatgctttggaatcctcatgtttcctcttttaact
ttagctactattgcttatatcaccgctttcattcttgcacctgcagtagatattgatgga
ataagagaaccagtagctggctcacttctttatggcaacaacattataacaggagctgta
atcccgagttctaatgctattggtgttcatttctatccagtctgggaatcaaaccatttc
gatgagtgcttatataatggtggaacatatcaattcgtagtccttcattttatgcttggt
gttgcttgttggatgggtagagaatgggaattcagttttagattaggtatgagaccatgg
atatttgtagctttctcagcacctgttattgcagcctttgcagtatttgttgtttacccg
atcggtcaagctagcttttccgatggaatgcctttaggaataagtggtacttttaatttt
atgcttgtgttccaagcagagcataacattttaatgcatccattccatatcctaggagta
gctggagtatttggtggatcattgttcagtgcaatgcatggttcattagttacatcttca
cttcttgcagaaagtgcaggagatattagcctaaatgttggatataagtttggtcaagaa
gatgaaacttatagcatatcagcagcccatggttattttggtagactcatatttcaatat
gcttccttcaataactctcgtagccttcacttctttctagcagcttggccagttattggt
atttggttcactgcccttggagtaagtacaatggctttcaacttaaatggtttaaacttc
aaccaatccatcctagattccagtggccatctaatcttaagttgggcagatattgtgaat
cgtgctgatctaggtatggaagtgatgcgtgaaagaaatgctcataatttcccattagat
ctagcataa
>Irm26_psbA
atgaagaacacatcttactatcaactcaacttacttggtaatgtcataggattcgtacta
tccacaacaaatcgtctctacattgggtgctttggtatcctaatgttccctcttttaact
ttagctactattgcttatatcaccgctttcattcttgcacctgcagtagatattgatgga
ataagagaaccagtagctggctcacttctttatggcaataacattataacaggagctgta
ataccaagttctaatgctattggggttcatttctatccagtttgggagtcaaatggattt
gatgagtgcttatataatggtggtacatatcaatttgtagtacttcattttatgcttggt
gttgcttgttggatgggtagagaatgggaattcagtttcagattaggtatgagaccatgg
atatttgtagctttctcagcacctgttgtagcagcttttgcagtatttgttgtttatcca
attggtcaagctagcttttccgatggaatgcctttaggaataagtggaacttttaatttt
atgcttgtgttccaagcagaacataacatcttaatgcatccattccatattctaggagta
gctggagtatttggtggatcattgttcagtgcaatgcatggttcattagttacatcttca
cttcttgcagaaagtgcaggagatattagcctaaatgttggatataagtttggtcaagaa
gatgaaacttatagcatatcagcagcccatggttattttggtagactcatatttcaatat
gcttccttcaataactctcgtagtcttcacttcttcctagcagcttggccagttattggt
atttggttcacagcacttggagtaagtacaatggctttcaacttaaatggtttaaacttc
aaccaatccatcctagattccagtggccatctaatcttaagttgggcagatattgtgaat
cgtgctgatctaggtatggaagtgatgcatgaaagaaatgctcacaatttcccattagat
ctagcataa

>Irm27_psbA
atgaagaatagatcttactatcaactcaacttacttggtaatgtcataggatttgtgcta
tccacaacaaatcgtctctatattggatgctttggaatcctcatgtttcctcttttaact
ttagctactattgcttatatcaccgctttcattcttgcacctgcagtagatattgatgga
ataagagaaccagtagctggctcacttctttatggcaacaacattataacaggagctgta
atcccgagttctaatgctattggtgttcatttctatccagtctgggaatcaaaccatttc
gatgagtgcttatataatggtggaacatatcaatttgtagtacttcatttcatgcttggt
gtggcttgttggatgggtagagaatgggaattcagtttcagattaggtatgagaccatgg
atatttgtagctttctcagcacctgttattgcagcctttgcagtatttgtagtctaccca
attgggcaagctagcttttccgatggaatgcctttaggaataagtggaacctttaatttt
atgcttgtattccaggcagagcataacatcttaatgcatccattccatattctaggagta
gctggagtatttggtggatcattatttagtgcaatgcatggttccctagtaacatcttcc
cttcttgcagaaactgcaggagatattagcctcaatgttggttataagtttggacaagaa
gatgaaacttatagcatatcagcagctcatggttattttggtagactcatatttcaatat
gcttccttcaataactctcgtagccttcacttctttctagcagcttggccagttattggt
atttggttcactgcccttggagtaagtacaatggctttcaacttaaatggtttaaacttc
aaccaatccatcctagattccagtggccatctaatcttaagttgggcagatattgtgaat
cgtgctgatctaggtatggaagtgatgcgtgaaagaaatgctcataatttcccattagat
ctagcataa
>Irm3_psbA
atgaagaatagatcttactatcaactcaacttacttggtaatgtcataggattcgtacta
tctacaacaaatcgtctctacattggatgctttggtatcctaatgtttcctcttttaact
ttagcaactattgcttatatcgcagctttcattcttgcacctgcagtagatattgatgga
ataagagaaccagtagctggatcacttctttatggtaataacattataactggagccgta
attccaagttctaatgctattggagttcatttttatccagtttgggagtcaaatggattc
gatgagtgcttatataatggtggaacatatcaatttgtagtacttcatttcatgcttggt
gtggcttgttggatgggtagagaatgggaattcagtttcagattaggtatgagaccatgg
atatttgtagctttctcagcacctgttattgcagcctttgcagtatttgttgtttacccg
atcggtcaagctagcttttccgatggaatgcctttaggaataagtggaacctttaatttt
atgcttgtattccaggcagagcataacatcttaatgcatccattccacatcttaggagta
gctggagtatttggtggatcattatttagtgcaatgcatggttccctagtaacatcttca
cttcttgcagaaagtgcaggagatattagcctcaatgttggttataagtttggacaagaa
gatgaaacttatagcatatcagcagctcatggttattttggtagacttatattccaatat
gcctccttcaataactctcgtagtcttcatttctttctagcagcttggccagttattggt
atttggtttacagcacttggagtaagtacaatggctttcaacttaaatggtttaaacttc
aaccaatccatcctagattccagtggccatctaattttaagctgggcagatattgtgaat
cgtgctgatctaggtatggaagtaatgcatgaaagaaacgctcataatttcccattagat
ctagcataa
>Irm4_psbA
atgaagaatagatcttactatcaactcaacttacttggcaatgtcataggattcgtacta
tctacaacaaatcgtctctacattggatgctttggtatcctaatgtttcctcttttaact
ttagcaactattgcttatatcgcagctttcattcttgcacctgcagtagatattgatgga
ataagagaaccagtagctggatcacttctttatggcaataacattataacaggagctgta
ataccaagttctaatgctattggggttcatttctatccagtctgggaatcaaaccatttc
gatgagtgcttatataatggtggtacatatcaatttgtagtacttcatttcatgcttggt
gtggcttgttggatgggtagagaatgggaattcagtttcagattaggtatgagaccatgg
atatttgtagctttctcagcacctgttgtagcagcttttgcagtatttgtagtctatcca
attgggcaagctagcttttccgatggaatgcctttaggaataagtggaacctttaatttt
atgcttgttttccaagcagagcataacatcttaatgcatccattccatattctaggagta
gctggagtatttggtggatcattgttcagtgcaatgcatggttcattagttacatcttca
cttcttgcagaaactgcaggagatattagcctcaatgttggttataagtttggacaagaa
gatgaaacttatagcatatcagcagcccatggttattttggtagacttatatttcaatat
gcctccttcaataactctcgtagtcttcacttcttcctagcagcttggccagttattggt
atttggttcacagcacttggagtaagtacaatggctttcaacttaaatggtttaaacttc
aaccaatccatcctagattccagtggccatctaatcttaagttgggcagatattgtgaat
cgtgctgatctaggtatggaagtgatgcgtgaaagaaatgctcataatttcccattagat
ctagcataa

>Irm5_psbA
atgaagaacacatcttactatcaactcaacttacttggtaatgtcataggattcgtacta
tccacaacaaatcgtctctacattgggtgctttggtatcctaatgttccctcttttaact
ttagcaactattgcttatatcacagcttttattcttgcacctgcagtagatattgatgga
ataagagaaccagtagctggctcacttctttatggcaataacattataacaggagctgta
ataccaagttctaatgctattggggttcatttctatccagtttgggagtcaaatggattt
gatgagtgcttatataatggtggtacatatcaatttgtagtacttcattttatgcttggt
gttgcttgttggatgggtagagaatgggaattcagtttcagattaggtatgagaccatgg
atatttgtagctttctcagcacctgttgtagcagcttttgcagtatttgttgtttatcca
attggtcaagctagcttttccgatggaatgcctttaggaataagtggaacttttaatttt
atgcttgtgttccaagcagaacataacattttaatgcatccattccatatcctaggagta
gctggtgtatttggaggttccttatttagtgcaatgcatggttcattagttacatcttca
cttcttgcagaaagtgcaggagatattagcctaaatgttggatataagtttggtcaagaa
gatgaaacttatagcatatcagcagcccatggttattttggtagactcatatttcaatat
gcttccttcaataactctcgtagtcttcacttcttcctagcagcttggccagttattggt
atttggttcacagcacttggagtaagtacaatggctttcaacttaaatggtttaaacttc
aaccaatccatcctagattccagtggccatctaatcttaagttgggcagatattgtgaat
cgtgctgatctaggtatggaagtgatgcatgaaagaaatgctcacaatttcccattagat
ctagcataa
>Irm7_psbA
atgaagaatagatcttactatcaactcaacttacttggtaatgtcataggattcgtacta
tccacaacaaatcgtctctacattgggtgctttggtatcctaatgttccctcttttaact
ttagcaactattgcttatatcacagcttttattcttgcacctgcagtagatattgatgga
ataagagaaccagtagctggctcacttctttatggcaacaacattataacaggagctgta
atcccgagttctaatgctattggtgttcatttctatccagtctgggaatcaaaccatttc
gatgagtgcttatataatggtggaacatatcaattcgtagtccttcattttatgcttggt
gttgcttgttggatgggtagagaatgggaattcagttttagattaggtatgagaccatgg
atatttgtagctttctcagcacctgttattgcagcctttgcagtatttgttgtttacccg
atcggtcaagctagcttttctgatggaatgcctttaggaataagtggtacttttaatttt
atgcttgtgttccaagcagaacataacattttaatgcatccattccatatcctaggagta
gctggagtatttggtggatccttattcagtgcaatgcatggttcattagttacatcttca
cttcttgcagaaagtgcaggagatattagcctcaatgttggatataagtttggtcaagaa
gatgaaacttatagcatatcagccgctcatggttattttggtagactcatatttcaatat
gcttccttcaataactctcgtagccttcacttcttcctagcagcttggccagttattggt
atttggttcacagcacttggagtaagtacaatggctttcaacttaaatggtttaaacttc
aaccaatccatcctagattccagtggccatctaatcttaagttgggcagatattgtgaat
cgtgctgatctaggtatggaagtgatgcatgaaagaaatgctcacaatttcccattagat
ctagcataa
>Irm9_psbA
atgaagaatagatcttactatcaactcaacttacttggtaatgtcataggatttgtacta
tctacaacaaatcgtctctacattggatgctttggtatcctcatgtttcctcttttaact
ttagcaactattgcttatatcgcagctttcattcttgcacctgcagtagatattgatgga
ataagagaaccagtagctggatcacttctttatggtaataacattataacaggagctgta
atcccgagttctaatgctattggtgttcatttctatccagtctgggaatcaaaccatttc
gatgagtgcttatataatggtggaacatatcaattcgtagtccttcattttatgcttggt
gttgcttgttggatgggtagagaatgggaattcagtttcagattaggtatgagaccatgg
atatttgtagctttctcagcacctgttgtagcagcttttgcagtatttgttgtttacccg
atcggtcaagctagcttttctgatggaatgcctttaggaataagtggaacctttaatttt
atgcttgtattccaggcagagcataacatcttaatgcatccattccacatcttaggagta
gctggagtatttggtggatcattatttagtgcaatgcatggttccctagtaacatcttcc
cttcttgcagaaagtgcaggagatattagcctcaatgttggttataagtttggacaagaa
gatgaaacttatagcatatcagcagctcatggttattttggtagacttatattccaatat
gcttccttcaataactctcgtagtcttcatttcttcttagcagcttggccagttattggt
atttggtttacagcacttggagtaagcacaatggctttcaacttaaatggtttaaacttc
aaccaatccatcctagattccagtggccatctaatcttaagttgggcagatattgtgaat
cgtgctgatctaggtatggaagtaatgcatgaaagaaacgctcataatttcccattagat
ctagcataa

>Isy12_psbA
atgaagaatagatcttactatcaactcaacttacttggtaatgtcataggattcgtacta
tccacaacaaatcgtctctacattgggtgctttggtatcctaatgttccctcttttaact
ttagcaactattgcttatatcacagcttttattcttgcacctgcagtagatattgatgga
ataagagaaccagtagctggctcacttctttatggcaataacattataacaggagctgta
ataccaagttctaatgctattggggttcatttctatccagtttgggagtcaaaccatttc
gatgagtgcttatataatggtggaacatatcaattcgtagtccttcattttatgcttggt
gttgcttgttggatgggtagagaatgggaattcagtttcagattaggtatgagaccatgg
atatttgtagctttctcagcacctgttgtagcagcttttgcagtatttgttgtttatcca
attggtcaagctagcttttccgatggaatgcctttaggaataagtggaacttttaatttt
atgcttgtgttccaagcagaacataacattttaatgcatccattccatattctaggagta
gctggagtatttggtggatcattatttagtgcaatgcatggttcattagttacatcttca
cttcttgcagaaagtgcaggagatattagcctaaatgttggatataagtttggtcaagaa
gatgaaacttatagcatatcagccgctcatggttattttggtagactcatatttcaatat
gcttccttcaataactctcgtagccttcacttctttctagcagcttggccagttattggt
atttggttcactgcccttggagtaagtacaatggctttcaacttaaatggtttaaacttc
aaccaatccatcctagattccagtggccatctaatcttaagttgggcagatattgtgaat
cgtgctgatctaggtatggaagtgatgcatgaaagaaatgctcacaatttcccattagat
ctagcataa
>Isy15_psbA
atgaagaacacatcttactatcaactcaacttacttggtaatgtcataggattcgtgcta
tccacaacaaatcgtctctacattgggtgctttggtatcctaatgttccctcttttaact
ttagcaactattgcttatatcgcagctttcattcttgcacctgcagtagatattgatgga
ataagagaaccagtagctggatcacttctttatggtaataacattataacaggagccgta
attccaagttctaatgctattggagttcatttttatccagtttgggagtcaaatggattc
gatgagtgcttatataatggtggaacatatcaatttgtagtacttcatttcatgcttggt
gtggcttgttggatgggtagagaatgggaattcagtttcagattaggtatgagaccatgg
atatttgtagctttctcagcacctgttattgcagcctttgcagtatttgttgtttacccg
atcggtcaagctagcttttctgatggaatgcctttaggaataagtggaacctttaatttt
atgcttgtattccaggcagagcatagcatcttaatgcatccattccacatcttaggagta
gctggagtatttggtggatcattatttagtgcaatgcatggttccctagtaacatcttcc
cttcttgcagaaactgcaggagatattagcctcaatgttggttataagtttggacaagaa
gatgaaacttatagcatatcagcagctcatggttattttggtagacttatattccaatat
gcctccttcaataactctcgtagtcttcacttctttctagcagcttggccagttattggt
atttggtttacagcacttggagtaagcacaatggctttcaacttaaatggtttaaacttc
aaccaatccatcctagattccagtggccatctaattttaagctgggcagatattgtgaat
cgtgctgatctaggtatggaagtgatgcgtgaaagaaatgctcataatttcccattagat
ctagcataa
>Isy16_psbA
atgaagaacacatcttactatcaactcaacttacttggtaatgtcataggattcgtacta
tccacaacaaatcgtctctacattgggtgctttggtatcctaatgttccctcttttaact
ttagcaactattgcttatatcacagcttttattcttgcacctgcagtagatattgatgga
ataagagaaccagtagctggctcacttctttatggcaataacattataacaggagctgta
ataccaagttctaatgctattggggttcatttctatccagtttgggagtcaaatggattt
gatgagtgcttatataatggtggtacatatcaatttgtagtacttcattttatgcttggt
gttgcttgttggatgggtagagaatgggaattcagtttcagattaggtatgagaccatgg
atatttgtagctttctcagcacctgttgtagcagcttttgcagtatttgttgtttatcca
attggtcaagctagcttttccgatggaatgcctttaggaataagtggaacttttaatttt
atgcttgtgttccaagcagaacataacattttaatgcatccattccatatcctaggagta
gctggtgtatttggaggttccttatttagtgcaatgcatggttcattagttacatcttca
cttcttgcagaaagtgcaggagatattagcctaaatgttggatataagtttggtcaagaa
gatgaaacttatagcatatcagcagcccatggttattttggtagactcatatttcaatat
gcttccttcaataactctcgtagtcttcacttcttcctagcagcttggccagttattggt
atttggttcacagcacttggagtaagtacaatggctttcaacttaaatggtttaaacttc
aaccaatccatcctagattccagtggccatctaatcttaagttgggcagatattgtgaat
cgtgctgatctaggtatggaagtgatgcatgaaagaaatgctcacaatttcccattagat
ctagcataa

>Isy17_psbA
atgaagaacacatcttactatcaactcaacttacttggtaatgtcataggattcgtacta
tccacaacaaatcgtctctacattgggtgctttggtatcctaatgttccctcttttaact
ttagcaactattgcttatatcacagcttttattcttgcacctgcagtagatattgatgga
ataagagaaccagtagctggctcacttctttatggcaataacattataacaggagctgta
ataccaagttctaatgctattggggttcatttctatccagtttgggagtcaaatggattt
gatgagtgcttatataatggtggtacatatcaatttgtagtacttcattttatgcttggt
gttgcttgttggatgggtagagaatgggaattcagtttcagattaggtatgagaccatgg
atatttgtagctttctcagcacctgttgtagcagcttttgcagtatttgttgtttatcca
attggtcaagctagcttttccgatggaatgcctttaggaataagtggaacttttaatttt
atgcttgtgttccaagcagaacataacattttaatgcatccattccatatcctaggagta
gctggtgtatttggtggatcattgttcagtgcaatgcatggttcattagttacatcttca
cttcttgcagaaagtgcaggagatattagcctaaatgttggatataagtttggtcaagaa
gatgaaacttatagcatatcagcagcccatggttattttggtagactcatatttcaatat
gcttccttcaataactctcgtagtcttcacttcttcctagcagcttggccagttattggt
atttggttcacagcacttggagtaagtacaatggctttcaacttaaatggtttaaacttc
aaccaatccatcctagattccagtggccatctaatcttaagttgggcagatattgtgaat
cgtgctgatctaggtatggaagtgatgcatgaaagaaatgctcacaatttcccattagat
ctagcataa
>Isy18_psbA
atgaagaacacatcttactatcaactcaacttacttggtaatgtcataggatttgtgcta
tccacaacaaatcgtctctatattggatgctttggaatcctcatgttccctcttttaact
ttagcaactattgcttatatcacagcttttattcttgcacctgcagtagatattgatgga
ataagagaaccagtagctggctcacttctttatggcaataacattataacaggagctgta
ataccaagttctaatgctattggggttcatttctatccagtctgggaatcaaaccatttc
gatgagtgcttatataatggtggaacatatcaattcgtagtccttcattttatgcttggt
gttgcttgttggatgggtagagaatgggaattcagttttagattaggtatgagaccatgg
atatttgtagctttctcagcacctgttattgcagcctttgcagtatttgttgtttacccg
atcggtcaagctagcttttctgatggaatgcctttaggaataagtggaacttttaatttt
atgcttgtgttccaagcagaacataacattttaatgcatccattccatatcctaggagta
gctggtgtatttggaggttccttatttagtgcaatgcatggttcattagttacatcttca
cttcttgcagaaagtgcaggagatattagcctaaatgttggatataagtttggtcaagaa
gatgaaacttatagcatatcagcagcccatggttattttggtagactcatatttcaatat
gcttccttcaataactctcgtagtcttcacttcttcctagcagcttggccagttattggt
atttggttcacagcacttggagtaagtacaatggctttcaacttaaatggtttaaacttc
aaccaatccatcctagattccagtggccatctaatcttaagttgggcagatattgtgaat
cgtgctgatctaggtatggaagtgatgcatgaaagaaatgctcacaatttcccattagat
ctagcataa
>Isy21_psbA
atgaagaacacatcttactataaactcaacttacttggtaatgtcataggattcgtgcta
tccacaacaaatcgtctctacattggatgctttggaatcctcatgtttcctcttttaact
ttagcaactattgcttatatcacagcttttattcttgcacctgcagtagatattgatgga
ataagagaaccagtagctggctcacttctttatggcaataacattataacaggagctgta
ataccaagttctaatgctattggggttcatttctatccagtttgggagtcaaaccatttc
gatgagtgcttatataatggtggtacatatcaatttgtagtacttcattttatgcttggt
gttgcttgttggatgggtagagaatgggaattcagtttcagattaggtatgagaccatgg
atatttgtagctttctcagcacctgttgtagcagcttttgcagtatttgttgtttatcca
attggtcaagctagcttttccgatggaatgcctttaggaataagtggaacttttaatttt
atgcttgtgttccaagcagaacataacattttaatgcatccattccatatcctaggagta
gctggtgtatttggaggttcattatttagtgcaatgcatggttcattagttacatcttca
cttcttgcagaaagtgcaggagatattagcctaaatgttggatataagtttggtcaagaa
gatgaaacttatagcatatcagcagcccatggttattttggtagactcatatttcaatat
gcttccttcaataactctcgtagtcttcacttcttcctagcagcttggccagttattggt
atttggttcacagcacttggagtaagtacaatggctttcaacttaaatggtttaaacttc
aaccaatccatcctagattccagtggccatctaatcttaagttgggcagatattgtgaat
cgtgctgatctaggtatggaagtgatgcatgaaagaaatgctcacaatttcccattagat
ctagcataa

>Isy22_psbA
atgaagaacacatcttactatcaactcaacttacttggtaatgtcataggattcgtacta
tccacaacaaatcgtctctacattgggtgctttggtatcctaatgttccctcttttaact
ttagcaactattgcttatatcacagcttttattcttgcacctgcagtagatattgatgga
ataagagaaccagtagctggctcacttctttatggcaataacattataacaggagctgta
ataccaagttctaatgctattggggttcatttctatccagtttgggagtcaaatggattt
gatgagtgcttatataatggtggtacatatcaatttgtagtacttcattttatgcttggt
gttgcttgttggatgggtagagaatgggaattcagtttcagattaggtatgagaccatgg
atatttgtagctttctcagcacctgttgtagcagcttttgcagtatttgttgtttatcca
attggtcaagctagcttttccgatggaatgcctttaggaataagtggaacttttaatttt
atgcttgtgttccaagcagaacataacattttaatgcatccattccatatcctaggagta
gctggtgtatttggaggttcattatttagtgcaatgcatggttcattagttacatcttca
cttcttgcagaaagtgcaggagatattagcctaaatgttggatataagtttggtcaagaa
gatgaaacttatagcatatcagcagcccatggttattttggtagactcatatttcaatat
gcttccttcaataactctcgtagtcttcacttctttctagcagcttggccagttattggt
atttggttcacagcacttggagtaagtacaatggctttcaacttaaatggtttaaacttc
aaccaatccatcctagattccagtggccatctaatcttaagttgggcagatattgtgaat
cgtgctgatctaggtatggaagtgatgcatgaaagaaatgctcacaatttcccattagat
ctagcataa
>Isy23_psbA
atgaagaatagatcttactataaactcaacttacttggtaatgtcataggattcgtgcta
tccacaacaaatcgtctctacattgggtgctttggtatcctaatgttccctcttttaact
ttagcaactattgcttatatcacagcttttattcttgcacctgcagtagatattgatgga
ataagagaaccagtagctggctcacttctttatggcaataacattataacaggagctgta
ataccaagttctaatgctattggggttcatttctatccagtttgggagtcaaatggattt
gatgagtgcttatataatggtggtacatatcaatttgtagtacttcattttatgcttggt
gttgcttgttggatgggtagagaatgggaattcagtttcagattaggtatgagaccatgg
atatttgtagctttctcagcacctgttgtagcagcttttgcagtatttgttgtttatcca
attggtcaagctagcttttccgatggaatgcctttaggaataagtggaacttttaatttt
atgcttgtgttccaagcagaacataacattttaatgcatccattccatatcctaggagta
gctggagtatttggtggatcattgttcagtgcaatgcatggttcattagttacatcttca
cttcttgcagaaagtgcaggagatattagcctaaatgttggatataagtttggtcaagaa
gatgaaacttatagcatatcagcagcccatggttattttggtagactcatatttcaatat
gcttccttcaataactctcgtagtcttcacttcttcctagcagcttggccagttattggt
atttggttcacagcacttggagtaagtacaatggctttcaacttaaatggtttaaacttc
aaccaatccatcctagattccagtggccatctaatcttaagttgggcagatattgtgaat
cgtgctgatctaggtatggaagtgatgcatgaaagaaatgctcacaatttcccattagat
ctagcataa
>Isy24_psbA
atgaagaatagatcttactatcaactcaacttacttggtaatgtcataggattcgtacta
tccacaacaaatcgtctctacattgggtgctttggtatcctaatgttccctcttttaact
ttagctactattgcttatatcaccgctttcattcttgcacctgcagtagatattgatgga
ataagagaaccagtagctggctcacttctttatggcaataacattataacaggagctgta
ataccaagttctaatgctattggggttcatttctatccagtttgggagtcaaaccatttc
gatgagtgcttatataatggtggtacatatcaatttgtagtacttcattttatgcttggt
gttgcttgttggatgggtagagaatgggaattcagtttcagattaggtatgagaccatgg
atatttgtagctttctcagcacctgttgtagcagcttttgcagtatttgttgtttatcca
attggtcaagctagcttttccgatggaatgcctttaggaataagtggtacttttaatttt
atgcttgttttccaagcagagcataacatcttaatgcatccattccatatcctaggagta
gctggtgtatttggaggttccttatttagtgcaatgcatggttcattagttacatcttca
cttcttgcagaaagtgcaggagatattagcctaaatgttggatataagtttggtcaagaa
gatgaaacttatagcatatcagcagcccatggttattttggtagactcatatttcaatat
gcttccttcaataactctcgtagtcttcacttcttcctagcagcttggccagttattggt
atttggttcacagcacttggagtaagtacaatggctttcaacttaaatggtttaaacttc
aaccaatccatcctagattccagtggccatctaatcttaagttgggcagatattgtgaat
cgtgctgatctaggtatggaagtgatgcatgaaagaaatgctcacaatttcccattagat
ctagcataa

>Isy25_psbA
atgaagaacacatcttactataaactcaacttacttggtaatgtcataggattcgtgcta
tccacaacaaatcgtctctacattgggtgctttggtatcctaatgttccctcttttaact
ttagcaactattgcttatatcaccgcttttattcttgcacctgcagtagatattgatgga
ataagagaaccagtagctggctcacttctttatggcaataacattataacaggagctgta
ataccaagttctaatgctattggggttcatttctatccagtttgggagtcaaatggattt
gatgagtgcttatataatggtggtacatatcaatttgtagtacttcattttatgcttggt
gttgcttgttggatgggtagagaatgggaattcagtttcagattaggtatgagaccatgg
atatttgtagctttctcagcacctgttgtagcagcttttgcagtatttgttgtttatcca
attggtcaagctagcttttccgatggaatgcctttaggaataagtggtacttttaatttt
atgcttgtgttccaagcagaacataacattttaatgcatccattccatatcctaggagta
gctggtgtatttggaggttcattatttagtgcaatgcatggttcattagttacatcttca
cttcttgcagaaagtgcaggagatattagcctaaatgttggatataagtttggtcaagaa
gatgaaacttatagcatatcagccgctcatggttattttggtagactcatatttcaatat
gcttccttcaataactctcgtagccttcacttctttctagcagcttggccagttattggt
atttggttcacagcacttggagtaagtacaatggctttcaacttaaatggtttaaacttc
aaccaatccatcctagattccagtggccatctaatcttaagttgggcagatattgtgaat
cgtgctgatctaggtatggaagtgatgcatgaaagaaatgctcacaatttcccattagat
ctagcataa
>Isy26_psbA
atgaagaatagatcttactatcaactcaacttacttggtaatgtcataggattcgtgcta
tccacaacaaatcgtctctacattgggtgctttggtatcctaatgttccctcttttaact
ttagcaactattgcttatatcacagcttttattcttgcacctgcagtagatattgatgga
ataagagaaccagtagctggctcacttctttatggcaataacattataacaggagctgta
ataccaagttctaatgctattggggttcatttctatccagtttgggagtcaaatggattt
gatgagtgcttatataatggtggtacatatcaatttgtagtacttcattttatgcttggt
gttgcttgttggatgggtagagaatgggaattcagtttcagattaggtatgagaccatgg
atatttgtagctttctcagcacctgttgtagcagcttttgcagtatttgttgtttatcca
attggtcaagctagcttttccgatggaatgcctttaggaataagtggaacttttaatttt
atgcttgtgttccaagcagaacataacattttaatgcatccattccatatcctaggagta
gctggtgtatttggaggttcattatttagtgcaatgcatggttcattagttacatcttca
cttcttgcagaaagtgcaggagatattagcctaaatgttggatataagtttggtcaagaa
gatgaaacttatagcatatcagcagcccatggttattttggtagactcatatttcaatat
gcttccttcaataactctcgtagtcttcacttcttcctagcagcttggccagttattggt
atttggttcacagcacttggagtaagtacaatggctttcaacttaaatggtttaaacttc
aaccaatccatcctagattccagtggccatctaatcttaagttgggcagatattgtgaat
cgtgctgatctaggtatggaagtgatgcatgaaagaaatgctcacaatttcccattagat
ctagcataa
>Isy27_psbA
atgaagaacacatcttactataaactcaacttacttggtaatgtcataggattcgtgcta
tccacaacaaatcgtctctacattgggtgctttggtatcctaatgttccctcttttaact
ttagcaactattgcttatatcacagcttttattcttgcacctgcagtagatattgatgga
ataagagaaccagtagctggctcacttctttatggcaataacattataacaggagctgta
ataccaagttctaatgctattggggttcatttctatccagtttgggagtcaaaccatttc
gatgagtgcttatataatggtggtacatatcaatttgtagtacttcattttatgcttggt
gttgcttgttggatgggtagagaatgggaattcagtttcagattaggtatgagaccatgg
atatttgtagctttctcagcacctgttgtagcagcttttgcagtatttgttgtttatcca
attggtcaagctagcttttccgatggaatgcctttaggaataagtggaacttttaatttt
atgcttgtgttccaagcagaacataacattttaatgcatccattccatatcctaggagta
gctggtgtatttggaggttcattatttagtgcaatgcatggttcattagttacatcttca
cttcttgcagaaagtgcaggagatattagcctaaatgttggatataagtttggtcaagaa
gatgaaacttatagcatatcagcagcccatggttattttggtagactcatatttcaatat
gcttccttcaataactctcgtagtcttcacttcttcctagcagcttggccagttattggt
atttggttcacagcacttggagtaagtacaatggctttcaacttaaatggtttaaacttc
aaccaatccatcctagattccagtggccatctaatcttaagttgggcagatattgtgaat
cgtgctgatctaggtatggaagtgatgcatgaaagaaatgctcacaatttcccattagat
ctagcataa

>Isy4_psbA
atgaagaacacatcttactatcaactcaacttacttggtaatgtcataggattcgtacta
tccacaacaaatcgtctctacattgggtgctttggtatcctaatgttccctcttttaact
ttagcaactattgcttatatcacagcttttattcttgcacctgcagtagatattgatgga
ataagagaaccagtagctggctcacttctttatggcaataacattataacaggagctgta
ataccaagttctaatgctattggggttcatttctatccagtttgggagtcaaatggattt
gatgagtgcttatataatggtggtacatatcaatttgtagtacttcattttatgcttggt
gttgcttgttggatgggtagagaatgggaattcagtttcagattaggtatgagaccatgg
atatttgtagctttctcagcacctgttgtagcagcttttgcagtatttgttgtttatcca
attggtcaagctagcttttccgatggaatgcctttaggaataagtggaacttttaatttt
atgcttgtgttccaagcagaacataacattttaatgcatccattccatatcctaggagta
gctggtgtatttggaggttccttatttagtgcaatgcatggttcattagttacatcttca
cttcttgcagaaagtgcaggagatattagcctaaatgttggatataagtttggtcaagaa
gatgaaacttatagcatatcagcagcccatggttattttggtagactcatatttcaatat
gcttccttcaataactctcgtagtcttcacttcttcctagcagcttggccagttattggt
atttggttcacagcacttggagtaagtacaatggctttcaacttaaatggtttaaacttc
aaccaatccatcctagattccagtggccatctaatcttaagttgggcagatattgtgaat
cgtgctgatctaggtatggaagtgatgcatgaaagaaatgctcacaatttcccattagat
ctagcataa
>Isy7_psbA
atgaagaacacatcttactatcaactcaacttacttggtaatgtcataggattcgtacta
tccacaacaaatcgtctctacattgggtgctttggtatcctaatgttccctcttttaact
ttagcaactattgcttatatcgcagcttttattcttgcacctgcagtagatattgatgga
ataagagaaccagtagctggatcacttctttatggtaataacattataactggagccgta
attccaagttctaatgctattggagttcatttttatccagtttgggagtcaaatggattt
gatgagtgcttatataatggtggtacatatcaatttgtagtacttcatttcatgcttggt
gttgcttgttggatgggtagagaatgggaattcagtttcagattaggtatgagaccatgg
atatttgtagctttctcagcacctgttgtagcagcttttgcagtatttgttgtttatcca
attggtcaagctagcttttccgatggaatgcctttaggaataagtggaacctttaatttt
atgcttgtgttccaggcagaacataacattttaatgcatccattccacatcttaggagta
gctggtgtatttggaggttccttatttagtgcaatgcatggttccctagtaacatcttcc
cttcttgcagaaactgcaggagatattagcctcaatgttggttataagtttggacaagaa
gatgaaacttatagcatatcagcagcccatggttattttggtagactcatatttcaatat
gcttccttcaataactctcgtagtcttcatttcttcttagcagcttggccagttattggt
atttggtttacagcacttggagtaagcacaatggctttcaacttaaatggtttaaacttc
aaccaatccatcctagattccagtggccatctaattttaagctgggcagatattgtgaat
cgtgctgatctaggtatggaagtaatgcatgaaagaaacgctcacaatttcccattagat
ctagcataa
>Isy8_psbA
atgaagaacacatcttactatcaactcaacttacttggtaatgtcataggattcgtgcta
tccacaacaaatcgtctctacattgggtgctttggtatcctaatgttccctcttttaact
ttagcaactattgcttatatcacagcttttattcttgcacctgcagtagatattgatgga
ataagagaaccagtagctggctcacttctttatggcaataacattataacaggagctgta
ataccaagttctaatgctattggggttcatttctatccagtttgggagtcaaatggattt
gatgagtgcttatataatggtggtacatatcaatttgtagtacttcattttatgcttggt
gttgcttgttggatgggtagagaatgggaattcagtttcagattaggtatgagaccatgg
atatttgtagctttctcagcacctgttgtagcagcttttgcagtatttgttgtttatcca
attggtcaagctagcttttccgatggaatgcctttaggaataagtggaacttttaatttt
atgcttgtgttccaagcagaacataacattttaatgcatccattccatatcctaggagta
gctggtgtatttggaggttcattatttagtgcaatgcatggttcattagttacatcttca
cttcttgcagaaagtgcaggagatattagcctaaatgttggatataagtttggtcaagaa
gatgaaacttatagcatatcagcagcccatggttattttggtagactcatatttcaatat
gcttccttcaataactctcgtagtcttcacttcttcctagcagcttggccagttattggt
atttggttcacagcacttggagtaagtacaatggctttcaacttaaatggtttaaacttc
aaccaatccatcctagattccagtggccatctaatcttaagttgggcagatattgtgaat
cgtgctgatctaggtatggaagtgatgcatgaaagaaatgctcacaatttcccattagat
ctagcataa

>KrA1_psbA
atgaagaacacatcttactatcaactcaacttacttggtaatgtcataggattcgtacta
tccacaacaaatcgtctctacattgggtgctttggtatcctaatgttccctcttttaact
ttagcaactattgcttatatcacagcttttattcttgcacctgcagtagatattgatgga
ataagagaaccagtagctggctcacttctttatggcaataacattataacaggagctgta
ataccaagttctaatgctattggggttcatttctatccagtttgggagtcaaatggattt
gatgagtgcttatataatggtggtacatatcaatttgtagtacttcattttatgcttggt
gttgcttgttggatgggtagagaatgggaattcagtttcagattaggtatgagaccatgg
atatttgtagctttctcagcacctgttgtagcagcttttgcagtatttgttgtttatcca
attggtcaagctagcttttccgatggaatgcctttaggaataagtggaacttttaatttt
atgcttgtgttccaagcagaacataacattttaatgcatccattccatatcctaggagta
gctggtgtatttggaggttccttatttagtgcaatgcatggttcattagttacatcttca
cttcttgcagaaagtgcaggagatattagcctaaatgttggatataagtttggtcaagaa
gatgaaacttatagcatatcagcagcccatggttattttggtagactcatatttcaatat
gcttccttcaataactctcgtagtcttcacttcttcctagcagcttggccagttattggt
atttggttcacagcacttggagtaagtacaatggctttcaacttaaatggtttaaacttc
aaccaatccatcctagattccagtggccatctaatcttaagttgggcagatattgtgaat
cgtgctgatctaggtatggaagtgatgcatgaaagaaatgctcacaatttcccattagat
ctagcataa
>KrA10_psbA
atgaagaacacatcttactatcaactcaacttacttggtaatgtcataggattcgtacta
tccacaacaaatcgtctctacattgggtgctttggtatcctaatgttccctcttttaact
ttagcaactattgcttatatcacagcttttattcttgcacctgcagtagatattgatgga
ataagagaaccagtagctggctcacttctttatggcaataacattataacaggagctgta
ataccaagttctaatgctattggggttcatttctatccagtttgggagtcaaatggattt
gatgagtgcttatataatggtggtacatatcaatttgtagtacttcattttatgcttggt
gttgcttgttggatgggtagagaatgggaattcagtttcagattaggtatgagaccatgg
atatttgtagctttctcagcacctgttgtagcagcttttgcagtatttgttgtttatcca
attggtcaagctagcttttccgatggaatgcctttaggaataagtggaacttttaatttt
atgcttgtgttccaagcagaacataacattttaatgcatccattccatatcctaggagta
gctggtgtatttggaggttccttatttagtgcaatgcatggttcattagttacatcttca
cttcttgcagaaagtgcaggagatattagcctaaatgttggatataagtttggtcaagaa
gatgaaacttatagcatatcagcagcccatggttattttggtagactcatatttcaatat
gcttccttcaataactctcgtagtcttcacttcttcctagcagcttggccagttattggt
atttggttcacagcacttggagtaagtacaatggctttcaacttaaatggtttaaacttc
aaccaatccatcctagattccagtggccatctaatcttaagttgggcagatattgtgaat
cgtgctgatctaggtatggaagtgatgcatgaaagaaatgctcacaatttcccattagat
ctagcataa
>KrA11_psbA
atgaagaacacatcttactatcaactcaacttacttggtaatgtcataggattcgtacta
tccacaacaaatcgtctctacattgggtgctttggtatcctaatgttccctcttttaact
ttagcaactattgcttatatcacagcttttattcttgcacctgcagtagatattgatgga
ataagagaaccagtagctggctcacttctttatggcaataacattataacaggagctgta
ataccaagttctaatgctattggggttcatttctatccagtttgggagtcaaatggattt
gatgagtgcttatataatggtggtacatatcaattcgtagtccttcattttatgcttggt
gttgcttgttggatgggtagagaatgggaattcagtttcagattaggtatgagaccatgg
atatttgtagctttctcagcacctgttgtagcagcttttgcagtatttgttgtttatcca
attggtcaagctagcttttccgatggaatgcctttaggaataagtggaacttttaatttt
atgcttgtgttccaagcagaacataacattttaatgcatccattccatattctaggagta
gctggagtatttggtggatcattgttcagtgcaatgcatggttcattagttacatcttca
cttcttgcagaaagtgcaggagatattagcctaaatgttggatataagtttggtcaagaa
gatgaaacttatagcatatcagccgctcatggttattttggtagactcatatttcaatat
gcttccttcaataactctcgtagtcttcacttcttcctagcagcttggccagttattggt
atttggttcacagcacttggagtaagtacaatggctttcaacttaaatggtttaaacttc
aaccaatccatcctagattccagtggccatctaatcttaagttgggcagatattgtgaat
cgtgctgatctaggtatggaagtgatgcatgaaagaaatgctcacaatttcccattagat
ctagcataa

>KrA12_psbA
atgaagaatacatcttactatcaactcaacttacttggtaatgtcataggattcgtacta
tccacaacaaatcgtctctacattgggtgctttggtatcctaatgttccctcttttaact
ttagcaactattgcttatatcaccgctttcattcttgcacctgcagtagatattgatgga
ataagagaaccagtagctggctcacttctttatggcaacaacattataacaggagctgta
ataccaagttctaatgctattggggttcatttctatccagtttgggagtcaaatggattt
gatgagtgcttatataatggtggaacatatcaattcgtagtccttcattttatgcttggt
gttgcttgttggatgggtagagaatgggaattcagttttagattaggtatgagaccatgg
atatttgtagctttctcagcacctgttgtagcagcttttgcagtatttgttgtttatcca
attggtcaagctagcttttccgatggaatgcctttaggaataagtggaacttttaatttt
atgcttgtgttccaagcagaacataacattttaatgcatccattccatatcctaggagta
gctggtgtatttggtggatcattgttcagtgcaatgcatggttcattagttacatcttca
cttcttgcagaaagtgcaggagatattagcctcaatgttggatataagtttggtcaagaa
gatgaaacttatagcatatcagcagcccatggttattttggtagactcatatttcaatat
gcttccttcaataactctcgtagtcttcacttctttctagcagcttggccagttattggt
atttggttcactgcccttggagtaagtacaatggctttcaacttaaatggtttaaacttc
aaccaatccatcctagattccagtggccatctaatcttaagttgggcagatattgtgaat
cgtgctgatctaggtatggaagtgatgcgtgaaagaaatgctcataatttcccattagat
ctagcataa
>KrA13_psbA
atgaagaatagatcttactatcaactcaacttacttggtaatgtcataggattcgtacta
tccacaacaaatcgtctctacattgggtgctttggtatcctaatgttccctcttttaact
ttagcaactattgcttatatcacagcttttattcttgcacctgcagtagatattgatgga
ataagagaaccagtagctggctcacttctttatggcaataacattataacaggagctgta
ataccaagttctaatgctattggggttcatttctatccagtttgggagtcaaaccatttc
gatgagtgcttatataatggtggaacatatcaattcgtagtccttcattttatgcttggt
gttgcttgttggatgggtagagaatgggaattcagttttagattaggtatgagaccatgg
atatttgtagctttctcagcacctgttgtagcagcttttgcagtatttgttgtttatcca
attggtcaagctagcttttccgatggaatgcctttaggaataagtggaacttttaatttt
atgcttgtgttccaagcagaacataacattttaatgcatccattccatatcctaggagta
gctggtgtatttggaggttccttatttagtgcaatgcatggttcattagttacatcttca
cttcttgcagaaagtgcaggagatattagcctaaatgttggatataagtttggtcaagaa
gatgaaacttatagcatatcagcagcccatggttattttggtagactcatatttcaatat
gcttccttcaataactctcgtagtcttcacttcttcctagcagcttggccagttattggt
atttggttcacagcacttggagtaagtacaatggctttcaacttaaatggtttaaacttc
aaccaatccatcctagattccagtggccatctaatcttaagttgggcagatattgtgaat
cgtgctgatctaggtatggaagtgatgcatgaaagaaatgctcacaatttcccattagat
ctagcataa
>KrA14_psbA
atgaagaacacatcttactatcaactcaacttacttggtaatgtcataggattcgtacta
tccacaacaaatcgtctctacattgggtgctttggtatcctaatgttccctcttttaact
ttagcaactattgcttatatcacagcttttattcttgcacctgcagtagatattgatgga
ataagagaaccagtagctggctcacttctttatggcaataacattataacaggagctgta
ataccaagttctaatgctattggggttcatttctatccagtttgggagtcaaatggattt
gatgagtgcttatataatggtggtacatatcaatttgtagtacttcattttatgcttggt
gttgcttgttggatgggtagagaatgggaattcagtttcagattaggtatgagaccatgg
atatttgtagctttctcagcacctgttgtagcagcttttgcagtatttgttgtttatcca
attggtcaagctagcttttccgatggaatgcctttaggaataagtggaacttttaatttt
atgcttgtgttccaagcagaacataacattttaatgcatccattccatattctaggagta
gctggtgtatttggaggttccttatttagtgcaatgcatggttcattagttacatcttca
cttcttgcagaaagtgcaggagatattagcctaaatgttggatataagtttggtcaagaa
gatgaaacttatagcatatcagccgctcatggttattttggtagactcatatttcaatat
gcttccttcaataactctcgtagtcttcacttcttcctagcagcttggccagttattggt
atttggttcacagcacttggagtaagtacaatggctttcaacttaaatggtttaaacttc
aaccaatccatcctagattccagtggccatctaatcttaagttgggcagatattgtgaat
cgtgctgatctaggtatggaagtgatgcatgaaagaaatgctcacaatttcccattagat
ctagcataa

>KrA15_psbA
atgaagaatagatcttactatcaactcaacttacttggtaatgtcataggatttgtgcta
tccacaacaaatcgtctctacattgggtgctttggtatcctaatgttccctcttttaact
ttagcaactattgcttatatcacagcttttattcttgcacctgcagtagatattgatgga
ataagagaaccagtagctggctcacttctttatggcaataacattataacaggagctgta
ataccaagttctaatgctattggggttcatttctatccagtttgggagtcaaatggattt
gatgagtgcttatataatggtggtacatatcaatttgtagtacttcattttatgcttggt
gttgcttgttggatgggtagagaatgggaattcagtttcagattaggtatgagaccatgg
atatttgtagctttctcagcacctgttgtagcagcctttgcagtatttgttgtttacccg
atcggtcaagctagcttttccgatggaatgcctttaggaataagtggaacttttaatttt
atgcttgtgttccaagcagaacataacattttaatgcatccattccatatcctaggagta
gctggtgtatttggaggttccttatttagtgcaatgcatggttcattagttacatcttca
cttcttgcagaaagtgcaggagatattagcctaaatgttggatataagtttggtcaagaa
gatgaaacttatagcatatcagcagcccatggttattttggtagactcatatttcaatat
gcttccttcaataactctcgtagccttcacttctttctagcagcttggccagttattggt
atttggttcactgcccttggagtaagtacaatggctttcaacttaaatggtttaaacttc
aaccaatccatcctagattccagtggccatctaatcttaagttgggcagatattgtgaat
cgtgctgatctaggtatggaagtgatgcatgaaagaaatgctcacaatttcccattagat
ctagcataa
>KrA2_psbA
atgaagaacacatcttactatcaactcaacttacttggtaatgtcataggattcgtacta
tccacaacaaatcgtctctacattgggtgctttggtatcctaatgttccctcttttaact
ttagcaactattgcttatatcacagcttttattcttgcacctgcagtagatattgatgga
ataagagaaccagtagctggctcacttctttatggcaataacattataacaggagctgta
ataccaagttctaatgctattggggttcatttctatccagtttgggagtcaaatggattt
gatgagtgcttatataatggtggtacatatcaatttgtagtacttcattttatgcttggt
gttgcttgttggatgggtagagaatgggaattcagtttcagattaggtatgagaccatgg
atatttgtagctttctcagcacctgttgtagcagcttttgcagtatttgttgtttatcca
attggtcaagctagcttttccgatggaatgcctttaggaataagtggaacttttaatttt
atgcttgtgttccaagcagaacataacattttaatgcatccattccatatcctaggagta
gctggtgtatttggaggttccttatttagtgcaatgcatggttcattagttacatcttca
cttcttgcagaaagtgcaggagatattagcctaaatgttggatataagtttggtcaagaa
gatgaaacttatagcatatcagcagcccatggttattttggtagactcatatttcaatat
gcttccttcaataactctcgtagtcttcacttcttcctagcagcttggccagttattggt
atttggttcacagcacttggagtaagtacaatggctttcaacttaaatggtttaaacttc
aaccaatccatcctagattccagtggccatctaatcttaagttgggcagatattgtgaat
cgtgctgatctaggtatggaagtgatgcatgaaagaaatgctcacaatttcccattagat
ctagcataa
>KrA3_psbA
atgaagaacacatcttactatcaactcaacttacttggtaatgtcataggattcgtacta
tccacaacaaatcgtctctacattgggtgctttggtatcctaatgttccctcttttaact
ttagcaactattgcttatatcacagcttttattcttgcacctgcagtagatattgatgga
ataagagaaccagtagctggctcacttctttatggcaataacattataacaggagctgta
ataccaagttctaatgctattggggttcatttctatccagtttgggagtcaaatggattt
gatgagtgcttatataatggtggtacatatcaatttgtagtacttcattttatgcttggt
gttgcttgttggatgggtagagaatgggaattcagtttcagattaggtatgagaccatgg
atatttgtagctttctcagcacctgttgtagcagcttttgcagtatttgttgtttatcca
attggtcaagctagcttttccgatggaatgcctttaggaataagtggaacttttaatttt
atgcttgtgttccaagcagaacataacattttaatgcatccattccatatcctaggagta
gctggtgtatttggaggttccttatttagtgcaatgcatggttcattagttacatcttca
cttcttgcagaaagtgcaggagatattagcctaaatgttggatataagtttggtcaagaa
gatgaaacttatagcatatcagcagcccatggttattttggtagactcatatttcaatat
gcttccttcaataactctcgtagtcttcacttcttcctagcagcttggccagttattggt
atttggttcacagcacttggagtaagtacaatggctttcaacttaaatggtttaaacttc
aaccaatccatcctagattccagtggccatctaatcttaagttgggcagatattgtgaat
cgtgctgatctaggtatggaagtgatgcatgaaagaaatgctcacaatttcccattagat
ctagcataa

>KrA4_psbA
atgaagaacacatcttactatcaactcaacttacttggtaatgtcataggattcgtacta
tccacaacaaatcgtctctacattgggtgctttggtatcctaatgttccctcttttaact
ttagcaactattgcttatatcaccgctttcattcttgcacctgcagtagatattgatgga
ataagagaaccagtagctggctcacttctttatggcaataacattataacaggagctgta
ataccaagttctaatgctattggtgttcatttctatccagtctgggaatcaaaccatttc
gatgagtgcttatataatggtggaacatatcaattcgtagtacttcattttatgcttggt
gttgcttgttggatgggtagagaatgggaattcagttttagattaggtatgagaccatgg
atatttgtagctttctcagcacctgttgtagcagcttttgcagtatttgttgtttatcca
attggtcaagctagcttttccgatggaatgcctttaggaataagtggtacttttaatttt
atgcttgtgttccaagcagaacataacattttaatgcatccattccatatcctaggagta
gctggtgtatttggaggttccttatttagtgcaatgcatggttcattagttacatcttca
cttcttgcagaaagtgcaggagatattagcctaaatgttggatataagtttggtcaagaa
gatgaaacttatagcatatcagcagcccatggttattttggtagactcatatttcaatat
gcttccttcaataactctcgtagtcttcacttctttctagcagcttggccagttattggt
atttggttcacagcacttggagtaagtacaatggctttcaacttaaatggtttaaacttc
aaccaatccatcctagattccagtggccatctaatcttaagttgggcagatattgtgaat
cgtgctgatctaggtatggaagtgatgcatgaaagaaatgctcacaatttcccattagat
ctagcataa
>KrA5_psbA
atgaagaatagatcttactatcaactcaacttacttggtaatgtcataggattcgtacta
tccacaacaaatcgtctctacattgggtgctttggtatcctaatgttccctcttttaact
ttagcaactattgcttatatcacagcttttattcttgcacctgcagtagatattgatgga
ataagagaaccagtagctggctcacttctttatggcaataacattataacaggagctgta
ataccaagttctaatgctattggggttcatttctatccagtttgggagtcaaatggattt
gatgagtgcttatataatggtggtacatatcaatttgtagtacttcattttatgcttggt
gttgcttgttggatgggtagagaatgggaattcagtttcagattaggtatgagaccatgg
atatttgtagctttctcagcacctgttgtagcagcttttgcagtatttgttgtttatcca
attggtcaagctagcttttccgatggaatgcctttaggaataagtggaacttttaatttt
atgcttgttttccaagcagagcataacatcttaatgcatccattccatattctaggagta
gctggagtatttggtggatcattgttcagtgcaatgcatggttcattagttacatcttca
cttcttgcagaaagtgcaggagatattagcctaaatgttggatataagtttggtcaagaa
gatgaaacttatagcatatcagcagcccatggttattttggtagactcatatttcaatat
gcttccttcaataactctcgtagccttcacttctttctagcagcttggccagttattggt
atttggttcactgcccttggagtaagtacaatggctttcaacttaaatggtttaaacttc
aaccaatccatcctagattccagtggccatctaatcttaagttgggcagatattgtgaat
cgtgctgatctaggtatggaagtgatgcatgaaagaaatgctcacaatttcccattagat
ctagcataa
>KrA6_psbA
atgaagaacacatcttactatcaactcaacttacttggtaatgtcataggattcgtacta
tccacaacaaatcgtctctacattgggtgctttggtatcctaatgttccctcttttaact
ttagcaactattgcttatatcacagcttttattcttgcacctgcagtagatattgatgga
ataagagaaccagtagctggctcacttctttatggcaataacattataacaggagctgta
ataccaagttctaatgctattggggttcatttctatccagtttgggagtcaaatggattt
gatgagtgcttatataatggtggtacatatcaatttgtagtacttcattttatgcttggt
gttgcttgttggatgggtagagaatgggaattcagtttcagattaggtatgagaccatgg
atatttgtagctttctcagcacctgttgtagcagcttttgcagtatttgttgtttatcca
attggtcaagctagcttttccgatggaatgcctttaggaataagtggaacttttaatttt
atgcttgtgttccaagcagaacataacattttaatgcatccattccatatcctaggagta
gctggtgtatttggaggttccttatttagtgcaatgcatggttcattagttacatcttca
cttcttgcagaaagtgcaggagatattagcctaaatgttggatataagtttggtcaagaa
gatgaaacttatagcatatcagcagcccatggttattttggtagactcatatttcaatat
gcttccttcaataactctcgtagtcttcacttcttcctagcagcttggccagttattggt
atttggttcacagcacttggagtaagtacaatggctttcaacttaaatggtttaaacttc
aaccaatccatcctagattccagtggccatctaatcttaagttgggcagatattgtgaat
cgtgctgatctaggtatggaagtgatgcatgaaagaaatgctcacaatttcccattagat
ctagcataa

>KrA7_psbA
atgaagaacacatcttactatcaactcaacttacttggtaatgtcataggattcgtacta
tccacaacaaatcgtctctacattgggtgctttggtatcctaatgttccctcttttaact
ttagcaactattgcttatatcacagctttcattcttgcacctgcagtagatattgatgga
ataagagaaccagtagctggctcacttctttatggcaacaacattataacaggagctgta
ataccaagttctaatgctattggggttcatttctatccagtttgggagtcaaatggattt
gatgagtgcttatataatggtggtacatatcaatttgtagtacttcattttatgcttggt
gttgcttgttggatgggtagagaatgggaattcagtttcagattaggtatgagaccatgg
atatttgtagctttctcagcacctgttgtagcagcttttgcagtatttgttgtttacccg
atcggtcaagctagcttttccgatggaatgcctttaggaataagtggaacttttaatttt
atgcttgtgttccaagcagaacataacattttaatgcatccattccatatcctaggagta
gctggagtatttggaggttccttatttagtgcaatgcatggttcattagttacatcttca
cttcttgcagaaagtgcaggagatattagcctcaatgttggatataagtttggtcaagaa
gatgaaacttatagcatatcagccgctcatggttattttggtagactcatatttcaatat
gcttccttcaataactctcgtagtcttcacttcttcctagcagcttggccagttattggt
atttggttcacagcacttggagtaagtacaatggctttcaacttaaatggtttaaacttc
aaccaatccatcctagattccagtggccatctaatcttaagttgggcagatattgtgaat
cgtgctgatctaggtatggaagtgatgcatgaaagaaatgctcacaatttcccattagat
ctagcataa
>KrA8_psbA
atgaagaatagatcttactatcaactcaacttacttggtaatgtcataggattcgtacta
tccacaacaaatcgtctctacattgggtgctttggtatcctaatgttccctcttttaact
ttagcaactattgcttatatcacagcttttattcttgcacctgcagtagatattgatgga
ataagagaaccagtagctggctcacttctttatggcaataacattataacaggagctgta
ataccaagttctaatgctattggggttcatttctatccagtttgggagtcaaatggattt
gatgagtgcttatataatggtggtacatatcaatttgtagtacttcattttatgcttggt
gttgcttgttggatgggtagagaatgggaattcagtttcagattaggtatgagaccatgg
atatttgtagctttctcagcacctgttattgcagcctttgcagtatttgttgtttacccg
atcggtcaagctagcttttctgatggaatgcctttaggaataagtggaacttttaatttt
atgcttgtgttccaagcagaacataacattttaatgcatccattccatatcctaggagta
gctggtgtatttggtggatcattgttcagtgcaatgcatggttcattagttacatcttca
cttcttgcagaaagtgcaggagatattagcctaaatgttggatataagtttggtcaagaa
gatgaaacttatagcatatcagcagcccatggttattttggtagactcatatttcaatat
gcttccttcaataactctcgtagtcttcacttcttcctagcagcttggccagttattggt
atttggttcacagcacttggagtaagtacaatggctttcaacttaaatggtttaaacttc
aaccaatccatcctagattccagtggccatctaatcttaagttgggcagatattgtgaat
cgtgctgatctaggtatggaagtgatgcgtgaaagaaatgctcacaatttcccattagat
ctagcataa
>KrA9_psbA
atgaagaacacatcttactatcaactcaacttacttggtaatgtcataggattcgtacta
tccacaacaaatcgtctctacattgggtgctttggtatcctaatgttccctcttttaact
ttagcaactattgcttatatcacagcttttattcttgcacctgcagtagatattgatgga
ataagagaaccagtagctggctcacttctttatggcaataacattataacaggagctgta
ataccaagttctaatgctattggggttcatttctatccagtttgggagtcaaatggattt
gatgagtgcttatataatggtggtacatatcaatttgtagtacttcattttatgcttggt
gttgcttgttggatgggtagagaatgggaattcagtttcagattaggtatgagaccatgg
atatttgtagctttctcagcacctgttgtagcagcttttgcagtatttgttgtttatcca
attggtcaagctagcttttccgatggaatgcctttaggaataagtggaacttttaatttt
atgcttgtgttccaagcagaacataacattttaatgcatccattccatatcctaggagta
gctggtgtatttggaggttccttatttagtgcaatgcatggttcattagttacatcttca
cttcttgcagaaagtgcaggagatattagcctaaatgttggatataagtttggtcaagaa
gatgaaacttatagcatatcagcagcccatggttattttggtagactcatatttcaatat
gcttccttcaataactctcgtagtcttcacttcttcctagcagcttggccagttattggt
atttggttcacagcacttggagtaagtacaatggctttcaacttaaatggtttaaacttc
aaccaatccatcctagattccagtggccatctaatcttaagttgggcagatattgtgaat
cgtgctgatctaggtatggaagtgatgcatgaaagaaatgctcacaatttcccattagat
ctagcataa

>KrC1_psbA
atgaagaacacatcttactatcaactcaacttacttggtaatgtcataggattcgtacta
tccacaacaaatcgtctctacattgggtgctttggtatcctaatgttccctcttttaact
ttagcaactattgcttatatcacagcttttattcttgcacctgcagtagatattgatgga
ataagagaaccagtagctggctcacttctttatggcaataacattataacaggagctgta
ataccaagttctaatgctattggggttcatttctatccagtttgggagtcaaatggattt
gatgagtgcttatataatggtggtacatatcaatttgtagtacttcattttatgcttggt
gttgcttgttggatgggtagagaatgggaattcagtttcagattaggtatgagaccatgg
atatttgtagctttctcagcacctgttgtagcagcttttgcagtatttgttgtttatcca
attggtcaagctagcttttccgatggaatgcctttaggaataagtggaacttttaatttt
atgcttgtgttccaagcagaacataacattttaatgcatccattccatatcctaggagta
gctggtgtatttggaggttccttatttagtgcaatgcatggttcattagttacatcttca
cttcttgcagaaagtgcaggagatattagcctaaatgttggatataagtttggtcaagaa
gatgaaacttatagcatatcagcagcccatggttattttggtagactcatatttcaatat
gcttccttcaataactctcgtagtcttcacttcttcctagcagcttggccagttattggt
atttggttcacagcacttggagtaagtacaatggctttcaacttaaatggtttaaacttc
aaccaatccatcctagattccagtggccatctaatcttaagttgggcagatattgtgaat
cgtgctgatctaggtatggaagtgatgcatgaaagaaatgctcacaatttcccattagat
ctagcataa
>KrC10_psbA
atgaagaacacatcttactatcaactcaacttacttggtaatgtcataggattcgtacta
tccacaacaaatcgtctctacattgggtgctttggtatcctaatgttccctcttttaact
ttagcaactattgcttatatcacagcttttattcttgcacctgcagtagatattgatgga
ataagagaaccagtagctggctcacttctttatggcaataacattataacaggagctgta
ataccaagttctaatgctattggggttcatttctatccagtttgggagtcaaatggattt
gatgagtgcttatataatggtggtacatatcaatttgtagtacttcattttatgcttggt
gttgcttgttggatgggtagagaatgggaattcagtttcagattaggtatgagaccatgg
atatttgtagctttctcagcacctgttgtagcagcttttgcagtatttgttgtttatcca
attggtcaagctagcttttccgatggaatgcctttaggaataagtggaacttttaatttt
atgcttgtgttccaagcagaacataacattttaatgcatccattccatatcctaggagta
gctggtgtatttggaggttccttatttagtgcaatgcatggttcattagttacatcttca
cttcttgcagaaagtgcaggagatattagcctaaatgttggatataagtttggtcaagaa
gatgaaacttatagcatatcagcagcccatggttattttggtagactcatatttcaatat
gcttccttcaataactctcgtagtcttcacttcttcctagcagcttggccagttattggt
atttggttcacagcacttggagtaagtacaatggctttcaacttaaatggtttaaacttc
aaccaatccatcctagattccagtggccatctaatcttaagttgggcagatattgtgaat
cgtgctgatctaggtatggaagtgatgcatgaaagaaatgctcacaatttcccattagat
ctagcataa
>KrC11_psbA
atgaagaacacatcttactatcaactcaacttacttggtaatgtcataggattcgtacta
tccacaacaaatcgtctctacattgggtgctttggtatcctaatgttccctcttttaact
ttagcaactattgcttatatcacagcttttattcttgcacctgcagtagatattgatgga
ataagagaaccagtagctggctcacttctttatggcaataacattataacaggagctgta
ataccaagttctaatgctattggggttcatttctatccagtttgggagtcaaatggattt
gatgagtgcttatataatggtggtacatatcaatttgtagtacttcattttatgcttggt
gttgcttgttggatgggtagagaatgggaattcagtttcagattaggtatgagaccatgg
atatttgtagctttctcagcacctgttgtagcagcttttgcagtatttgttgtttatcca
attggtcaagctagcttttccgatggaatgcctttaggaataagtggaacttttaatttt
atgcttgtgttccaagcagaacataacattttaatgcatccattccatatcctaggagta
gctggtgtatttggaggttccttatttagtgcaatgcatggttcattagttacatcttca
cttcttgcagaaagtgcaggagatattagcctaaatgttggatataagtttggtcaagaa
gatgaaacttatagcatatcagcagcccatggttattttggtagactcatatttcaatat
gcttccttcaataactctcgtagtcttcacttcttcctagcagcttggccagttattggt
atttggttcacagcacttggagtaagtacaatggctttcaacttaaatggtttaaacttc
aaccaatccatcctagattccagtggccatctaatcttaagttgggcagatattgtgaat
cgtgctgatctaggtatggaagtgatgcatgaaagaaatgctcacaatttcccattagat
ctagcataa

>KrC12_psbA
atgaagaacacatcttactatcaactcaacttacttggtaatgtcataggattcgtacta
tccacaacaaatcgtctctacattgggtgctttggtatcctaatgttccctcttttaact
ttagcaactattgcttatatcacagcttttattcttgcacctgcagtagatattgatgga
ataagagaaccagtagctggctcacttctttatggcaataacattataacaggagctgta
ataccaagttctaatgctattggggttcatttctatccagtttgggagtcaaatggattt
gatgagtgcttatataatggtggtacatatcaatttgtagtacttcattttatgcttggt
gttgcttgttggatgggtagagaatgggaattcagtttcagattaggtatgagaccatgg
atatttgtagctttctcagcacctgttgtagcagcttttgcagtatttgttgtttatcca
attggtcaagctagcttttccgatggaatgcctttaggaataagtggaacttttaatttt
atgcttgtgttccaagcagaacataacattttaatgcatccattccatatcctaggagta
gctggtgtatttggaggttccttatttagtgcaatgcatggttcattagttacatcttca
cttcttgcagaaagtgcaggagatattagcctaaatgttggatataagtttggtcaagaa
gatgaaacttatagcatatcagcagcccatggttattttggtagactcatatttcaatat
gcttccttcaataactctcgtagtcttcacttcttcctagcagcttggccagttattggt
atttggttcacagcacttggagtaagtacaatggctttcaacttaaatggtttaaacttc
aaccaatccatcctagattccagtggccatctaatcttaagttgggcagatattgtgaat
cgtgctgatctaggtatggaagtgatgcatgaaagaaatgctcacaatttcccattagat
ctagcataa
>KrC13_psbA
atgaagaacacatcttactatcaactcaacttacttggtaatgtcataggattcgtacta
tccacaacaaatcgtctctacattgggtgctttggtatcctaatgttccctcttttaact
ttagcaactattgcttatatcacagcttttattcttgcacctgcagtagatattgatgga
ataagagaaccagtagctggctcacttctttatggcaataacattataacaggagctgta
ataccaagttctaatgctattggggttcatttctatccagtttgggagtcaaatggattt
gatgagtgcttatataatggtggtacatatcaatttgtagtacttcattttatgcttggt
gttgcttgttggatgggtagagaatgggaattcagtttcagattaggtatgagaccatgg
atatttgtagctttctcagcacctgttgtagcagcttttgcagtatttgttgtttatcca
attggtcaagctagcttttccgatggaatgcctttaggaataagtggaacttttaatttt
atgcttgttttccaagcagagcataacatcttaatgcatccattccatattctaggagta
gctggagtatttggtggatcattgttcagtgcaatgcatggttcattagttacatcttca
cttcttgcagaaagtgcaggagatattagcctaaatgttggatataagtttggtcaagaa
gatgaaacttatagcatatcagcagcccatggttattttggtagactcatatttcaatat
gcttccttcaataactctcgtagtcttcacttcttcctagcagcttggccagttattggt
atttggttcacagcacttggagtaagtacaatggctttcaacttaaatggtttaaacttc
aaccaatccatcctagattccagtggccatctaatcttaagttgggcagatattgtgaat
cgtgctgatctaggtatggaagtgatgcatgaaagaaatgctcacaatttcccattagat
ctagcataa
>KrC14_psbA
atgaagaacacatcttactatcaactcaacttacttggtaatgtcataggattcgtacta
tccacaacaaatcgtctctacattgggtgctttggtatcctaatgttccctcttttaact
ttagcaactattgcttatatcacagcttttattcttgcacctgcagtagatattgatgga
ataagagaaccagtagctggctcacttctttatggcaataacattataacaggagctgta
ataccaagttctaatgctattggggttcatttctatccagtttgggagtcaaatggattt
gatgagtgcttatataatggtggtacatatcaatttgtagtacttcattttatgcttggt
gttgcttgttggatgggtagagaatgggaattcagtttcagattaggtatgagaccatgg
atatttgtagctttctcagcacctgttattgcagcctttgcagtatttgttgtttatcca
attggtcaagctagcttttccgatggaatgcctttaggaataagtggaacttttaatttt
atgcttgtgttccaagcagaacataacattttaatgcatccattccatatcctaggagta
gctggagtatttggtggatcattgttcagtgcaatgcatggttcattagttacatcttca
cttcttgcagaaagtgcaggagatattagcctaaatgttggatataagtttggtcaagaa
gatgaaacttatagcatatcagcagcccatggttattttggtagactcatatttcaatat
gcttccttcaataactctcgtagtcttcacttcttcctagcagcttggccagttattggt
atttggttcacagcacttggagtaagtacaatggctttcaacttaaatggtttaaacttc
aaccaatccatcctagattccagtggccatctaatcttaagttgggcagatattgtgaat
cgtgctgatctaggtatggaagtgatgcatgaaagaaatgctcacaatttcccattagat
ctagcataa

>KrC15_psbA
atgaagaacacatcttactatcaactcaacttacttggtaatgtcataggattcgtacta
tccacaacaaatcgtctctacattgggtgctttggtatcctaatgttccctcttttaact
ttagcaactattgcttatatcacagcttttattcttgcacctgcagtagatattgatgga
ataagagaaccagtagctggctcacttctttatggcaataacattataacaggagctgta
ataccaagttctaatgctattggggttcatttctatccagtttgggagtcaaatggattt
gatgagtgcttatataatggtggtacatatcaatttgtagtacttcattttatgcttggt
gttgcttgttggatgggtagagaatgggaattcagtttcagattaggtatgagaccatgg
atatttgtagctttctcagcacctgttgtagcagcttttgcagtatttgttgtttatcca
attggtcaagctagcttttccgatggaatgcctttaggaataagtggaacttttaatttt
atgcttgtgttccaagcagaacataacattttaatgcatccattccatatcctaggagta
gctggtgtatttggaggttccttatttagtgcaatgcatggttcattagttacatcttca
cttcttgcagaaagtgcaggagatattagcctaaatgttggatataagtttggtcaagaa
gatgaaacttatagcatatcagcagcccatggttattttggtagactcatatttcaatat
gcttccttcaataactctcgtagtcttcacttcttcctagcagcttggccagttattggt
atttggttcacagcacttggagtaagtacaatggctttcaacttaaatggtttaaacttc
aaccaatccatcctagattccagtggccatctaatcttaagttgggcagatattgtgaat
cgtgctgatctaggtatggaagtgatgcatgaaagaaatgctcacaatttcccattagat
ctagcataa
>KrC2_psbA
atgaagaacacatcttactatcaactcaacttacttggtaatgtcataggattcgtacta
tccacaacaaatcgtctctacattgggtgctttggtatcctaatgttccctcttttaact
ttagcaactattgcttatatcacagcttttattcttgcacctgcagtagatattgatgga
ataagagaaccagtagctggctcacttctttatggcaataacattataacaggagctgta
ataccaagttctaatgctattggggttcatttctatccagtttgggagtcaaatggattt
gatgagtgcttatataatggtggtacatatcaatttgtagtacttcattttatgcttggt
gttgcttgttggatgggtagagaatgggaattcagtttcagattaggtatgagaccatgg
atatttgtagctttctcagcacctgttgtagcagcttttgcagtatttgttgtttatcca
attggtcaagctagcttttccgatggaatgcctttaggaataagtggaacttttaatttt
atgcttgtgttccaagcagaacataacattttaatgcatccattccatatcctaggagta
gctggtgtatttggaggttccttatttagtgcaatgcatggttcattagttacatcttca
cttcttgcagaaagtgcaggagatattagcctaaatgttggatataagtttggtcaagaa
gatgaaacatatagcatatcagccgcccatggttattttggtagactcatatttcaatat
gcttccttcaataactctcgtagtcttcacttcttcctagcagcttggccagttattggt
atttggttcacagcacttggagtaagtacaatggctttcaacttaaatggtttaaacttc
aaccaatccatcctagattccagtggccatctaatcttaagttgggcagatattgtgaat
agtgctgatctaggtatggaagtgatgcatgaaagaaatgctcacaatttcccattagat
ctagcataa
>KrC3_psbA
atgaagaacacatcttactatcaactcaacttacttggtaatgtcataggattcgtacta
tccacaacaaatcgtctctacattgggtgctttggtatcctaatgttccctcttttaact
ttagcaactattgcttatatcacagcttttattcttgcacctgcagtagatattgatgga
ataagagaaccagtagctggctcacttctttatggcaataacattataacaggagctgta
ataccaagttctaatgctattggggttcatttctatccagtttgggagtcaaatggattt
gatgagtgcttatataatggtggtacatatcaatttgtagtacttcattttatgcttggt
gttgcttgttggatgggtagagaatgggaattcagtttcagattaggtatgagaccatgg
atatttgtagctttctcagcacctgttgtagcagcttttgcagtatttgttgtttatcca
attggtcaagctagcttttccgatggaatgcctttaggaataagtggaacttttaatttt
atgcttgtgttccaagcagaacataacattttaatgcatccattccatatcctaggagta
gctggtgtatttggaggttccttatttagtgcaatgcatggttcattagttacatcttca
cttcttgcagaaagtgcaggagatattagcctaaatgttggatataagtttggtcaagaa
gatgaaacatatagcatatcagcagcccatggttattttggtagactcatatttcaatat
gcttccttcaataactctcgtagtcttcacttcttcctagcagcttggccagttattggt
atttggttcacagcacttggagtaagtacaatggctttcaacttaaatggtttaaacttc
aaccaatccatcctagattccagtggccatctaatcttaagttgggcagatattgtgaat
cgtgctgatctaggtatggaagtgatgcatgaaagaaatgctcacaatttcccattagat
ctagcataa

>KrC4_psbA
atgaagaacacatcttactatcaactcaacttacttggtaatgtcataggattcgtacta
tccacaacaaatcgtctctacattgggtgctttggtatcctaatgttccctcttttaact
ttagcaactattgcttatatcacagcttttattcttgcacctgcagtagatattgatgga
ataagagaaccagtagctggctcacttctttatggcaataacattataacaggagctgta
ataccaagttctaatgctattggggttcatttctatccagtttgggagtcaaatggattt
gatgagtgcttatataatggtggtacatatcaatttgtagtacttcattttatgcttggt
gttgcttgttggatgggtagagaatgggaattcagtttcagattaggtatgagaccatgg
atatttgtagctttctcagcacctgttgtagcagcttttgcagtatttgttgtttatcca
attggtcaagctagcttttccgatggaatgcctttaggaataagtggaacttttaatttt
atgcttgtgttccaagcagaacataacattttaatgcatccattccatatcctaggagta
gctggtgtatttggaggttccttatttagtgcaatgcatggttcattagttacatcttca
cttcttgcagaaagtgcaggagatattagcctaaatgttggatataagtttggtcaagaa
gatgaaacttatagcatatcagcagcccatggttattttggtagactcatatttcaatat
gcttccttcaataactctcgtagtcttcacttcttcctagcagcttggccagttattggt
atttggttcacagcacttggagtaagtacaatggctttcaacttaaatggtttaaacttc
aaccaatccatcctagattccagtggccatctaatcttaagttgggcagatattgtgaat
cgtgctgatctaggtatggaagtgatgcatgaaagaaatgctcacaatttcccattagat
ctagcataa
>KrC5_psbA
atgaagaacacatcttactatcaactcaacttacttggtaatgtcataggattcgtacta
tccacaacaaatcgtctctacattgggtgctttggtatcctaatgttccctcttttaact
ttagcaactattgcttatatcacagcttttattcttgcacctgcagtagatattgatgga
ataagagaaccagtagctggctcacttctttatggcaataacattataacaggagctgta
ataccaagttctaatgctattggggttcatttctatccagtttgggagtcaaatggattt
gatgagtgcttatataatggtggtacatatcaatttgtagtacttcattttatgcttggt
gttgcttgttggatgggtagagaatgggaattcagtttcagattaggtatgagaccatgg
atatttgtagctttctcagcacctgttgtagcagcttttgcagtatttgttgtttatcca
attggtcaagctagcttttccgatggaatgcctttaggaataagtggaacttttaatttt
atgcttgtgttccaagcagaacataacattttaatgcatccattccatatcctaggagta
gctggtgtatttggaggttccttatttagtgcaatgcatggttcattagttacatcttca
cttcttgcagaaagtgcaggagatattagcctaaatgttggatataagtttggtcaagaa
gatgaaacttatagcatatcagcagcccatggttattttggtagactcatatttcaatat
gcttccttcaataactctcgtagtcttcacttcttcctagcagcttggccagttattggt
atttggttcacagcacttggagtaagtacaatggctttcaacttaaatggtttaaacttc
aaccaatccatcctagattccagtggccatctaatcttaagttgggcagatattgtgaat
cgtgctgatctaggtatggaagtgatgcatgaaagaaatgctcacaatttcccattagat
ctagcataa
>KrC6_psbA
atgaagaacacatcttactatcaactcaacttacttggtaatgtcataggattcgtacta
tccacaacaaatcgtctctacattgggtgctttggtatcctaatgttccctcttttaact
ttagcaactattgcttatatcacagcttttattcttgcacctgcagtagatattgatgga
ataagagaaccagtagctggctcacttctttatggcaataacattataacaggagctgta
ataccaagttctaatgctattggggttcatttctatccagtttgggagtcaaatggattt
gatgagtgcttatataatggtggtacatatcaatttgtagtacttcattttatgcttggt
gttgcttgttggatgggtagagaatgggaattcagtttcagattaggtatgagaccatgg
atatttgtagctttctcagcacctgttgtagcagcttttgcagtatttgttgtttatcca
attggtcaagctagcttttccgatggaatgcctttaggaataagtggaacttttaatttt
atgcttgtgttccaagcagaacataacattttaatgcatccattccatatcctaggagta
gctggtgtatttggaggttccttatttagtgcaatgcatggttcattagttacatcttca
cttcttgcagaaagtgcaggagatattagcctaaatgttggatataagtttggtcaagaa
gatgaaacttatagcatatcagcagcccatggttattttggtagactcatatttcaatat
gcttccttcaataactctcgtagtcttcacttcttcctagcagcttggccagttattggt
atttggttcacagcacttggagtaagtacaatggctttcaacttaaatggtttaaacttc
aaccaatccatcctagattccagtggccatctaatcttaagttgggcagatattgtgaat
cgtgctgatctaggtatggaagtgatgcatgaaagaaatgctcacaatttcccattagat
ctagcataa

>KrC7_psbA
atgaagaacacatcttactatcaactcaacttacttggtaatgtcataggattcgtacta
tccacaacaaatcgtctctacattgggtgctttggtatcctaatgttccctcttttaact
ttagcaactattgcttatatcacagcttttattcttgcacctgcagtagatattgatgga
ataagagaaccagtagctggctcacttctttatggcaataacattataacaggagctgta
ataccaagttctaatgctattggggttcatttctatccagtttgggagtcaaatggattt
gatgagtgcttatataatggtggtacatatcaatttgtagtccttcattttatgcttggt
gttgcttgttggatgggtagagaatgggaattcagtttcagattaggtatgagaccatgg
atatttgtagctttctcagcacctgttgtagcagcttttgcagtatttgttgtttatcca
attggtcaagctagcttttccgatggaatgcctttaggaataagtggaacttttaatttt
atgcttgtgttccaagcagaacataacattttaatgcatccattccatatcctaggagta
gctggtgtatttggaggttccttatttagtgcaatgcatggttcattagttacatcttca
cttcttgcagaaagtgcaggagatattagcctaaatgttggatataagtttggtcaagaa
gatgaaacttatagcatatcagcagcccatggttattttggtagactcatatttcaatat
gcttccttcaataactctcgtagtcttcacttcttcctagcagcttggccagttattggt
atttggttcacagcacttggagtaagtacaatggctttcaacttaaatggtttaaacttc
aaccaatccatcctagattccagtggccatctaatcttaagttgggcagatattgtgaat
cgtgctgatctaggtatggaagtgatgcatgaaagaaatgctcacaatttcccattagat
ctagcataa
>KrC8_psbA
atgaagaacacatcttactatcaactcaacttacttggtaatgtcataggattcgtacta
tccacaacaaatcgtctctacattgggtgctttggtatcctaatgttccctcttttaact
ttagcaactattgcttatatcacagcttttattcttgcacctgcagtagatattgatgga
ataagagaaccagtagctggctcacttctttatggcaataacattataacaggagctgta
ataccaagttctaatgctattggggttcatttctatccagtttgggagtcaaaccatttc
gatgagtgcttatataatggtggtacatatcaatttgtagtacttcattttatgcttggt
gttgcttgttggatgggtagagaatgggaattcagtttcagattaggtatgagaccatgg
atatttgtagctttctcagcacctgttgtagcagcttttgcagtatttgttgtttatcca
attggtcaagctagcttttccgatggaatgcctttaggaataagtggaacttttaatttt
atgcttgtgttccaagcagaacataacattttaatgcatccattccatatcctaggagta
gctggtgtatttggaggttccttatttagtgcaatgcatggttcattagttacatcttca
cttcttgcagaaagtgcaggagatattagcctaaatgttggatataagtttggtcaagaa
gatgaaacttatagcatatcagcagcccatggttattttggtagactcatatttcaatat
gcttccttcaataactctcgtagtcttcacttcttcctagcagcttggccagttattggt
atttggttcacagcacttggagtaagtacaatggctttcaacttaaatggtttaaacttc
aaccaatccatcctagattccagtggccatctaatcttaagttgggcagatattgtgaat
cgtgctgatctaggtatggaagtgatgcatgaaagaaatgctcacaatttcccattagat
ctagcataa
>KrC9_psbA
atgaagaacacatcttactatcaactcaacttacttggtaatgtcataggattcgtacta
tccacaacaaatcgtctctacattgggtgctttggtatcctaatgttccctcttttaact
ttagcaactattgcttatatcacagcttttattcttgcacctgcagtagatattgatgga
ataagagaaccagtagctggctcacttctttatggcaataacattataacaggagctgta
ataccaagttctaatgctattggggttcatttctatccagtttgggagtcaaatggattt
gatgagtgcttatataatggtggtacatatcaatttgtagtacttcattttatgcttggt
gttgcttgttggatgggtagagaatgggaattcagtttcagattaggtatgagaccatgg
atatttgtagctttctcagcacctgttgtagcagcttttgcagtatttgttgtttatcca
attggtcaagctagcttttccgatggaatgcctttaggaataagtggaacttttaatttt
atgcttgtgttccaagcagaacataacattttaatgcatccattccatatcctaggagta
gctggtgtatttggaggttccttatttagtgcaatgcatggttcattagttacatcttca
cttcttgcagaaagtgcaggagatattagcctaaatgttggatataagtttggtcaagaa
gatgaaacttatagcatatcagcagcccatggttattttggtagactcatatttcaatat
gcttccttcaataactctcgtagtcttcacttcttcctagcagcttggccagttattggt
atttggttcacagcacttggagtaagtacaatggctttcaacttaaatggtttaaacttc
aaccaatccatcctagattccagtggccatctaatcttaagttgggcagatattgtgaat
cgtgctgatctaggtatggaagtgatgcatgaaagaaatgctcacaatttcccattagat
ctagcataa

>KrD1_psbA
atgaagaacacatcttactatcaactcaacttacttggtaatgtcataggattcgtacta
tccacaacaaatcgtctctacattgggtgctttggtatcctaatgttccctcttttaact
ttagcaactattgcttatatcacagcttttattcttgcacctgcagtagatattgatgga
ataagagaaccagtagctggctcacttctttatggcaataacattataacaggagctgta
ataccaagttctaatgctattggggttcatttctatccagtttgggagtcaaatcatttt
gatgagtgcttatataatggtggaacatatcaattcgtagtccttcattttatgcttggt
gttgcttgttggatgggtagagaatgggaattcagtttcagattaggtatgagaccatgg
atatttgtagctttctcagcacctgttgtagcagcttttgcagtatttgttgtttatcca
attggtcaagctagcttttccgatggaatgcctttaggaataagtggtacttttaatttt
atgcttgtgttccaagcagaacataacattttaatgcatccattccatatcctaggagta
gctggtgtatttggaggttccttatttagtgcaatgcatggttcattagttacatcttca
cttcttgcagaaagtgcaggagatattagcctaaatgttggatataagtttggtcaagaa
gatgaaacttatagcatatcagcagcccatggttattttggtagactcatatttcaatat
gcttccttcaataactctcgtagtcttcacttcttcctagcagcttggccagttattggt
atttggttcacagcacttggagtaagtacaatggctttcaacttaaatggtttaaacttc
aaccaatccatcctagattccagtggccatctaatcttaagttgggcagatattgtgaat
cgtgctgatctaggtatggaagtgatgcatgaaagaaatgctcacaatttcccattagat
ctagcataa
>KrD11_psbA
atgaagaacacatcttactatcaactcaacttacttggtaatgtcataggattcgtacta
tccacaacaaatcgtctctacattgggtgctttggtatcctaatgttccctcttttaact
ttagcaactattgcttatatcacagcttttattcttgcacctgcagtagatattgatgga
ataagagaaccagtagctggctcacttctttatggcaataacattataacaggagctgta
ataccaagttctaatgctattggggttcatttctatccagtttgggagtcaaatggattt
gatgagtgcttatataatggtggtacatatcaatttgtagtacttcattttatgcttggt
gttgcttgttggatgggtagagaatgggaattcagtttcagattaggtatgagaccatgg
atatttgtagctttctcagcacctgttgtagcagcttttgcagtatttgttgtttatcca
attggtcaagctagcttttccgatggaatgcctttaggaataagtggaacttttaatttt
atgcttgttttccaagcagaacataacattttaatgcatccattccatatcctaggagta
gctggtgtatttggaggttccttatttagtgcaatgcatggttcattagttacatcttca
cttcttgcagaaagtgcaggagatattagcctaaatgttggatataagtttggtcaagaa
gatgaaacttatagcatatcagcagcccatggttattttggtagactcatatttcaatat
gcttccttcaataactctcgtagtcttcacttcttcctagcagcttggccagttattggt
atttggttcacagcacttggagtaagtacaatggctttcaacttaaatggtttaaacttc
aaccaatccatcctagattccagtggccatctaatcttaagttgggcagatattgtgaat
cgtgctgatctaggtatggaagtgatgcatgaaagaaatgctcataatttcccattagat
ctagcataa
>KrD13_psbA
atgaagaacacatcttactatcaactcaacttacttggtaatgtcataggattcgtacta
tccacaacaaatcgtctctacattgggtgctttggaatcctaatgttccctcttttaact
ttagcaactattgcttatatcacagcttttattcttgcacctgcagtagatattgatgga
ataagagaaccagtagctggctcacttctttatggcaataacattataacaggagctgta
ataccaagttctaatgctattggggttcatttctatccagtttgggagtcaaatggattt
gatgagtgcttatataatggtggtacatatcaatttgtagtacttcattttatgcttggt
gttgcttgttggatgggtagagaatgggaattcagtttcagattaggtatgagaccatgg
atatttgtagctttctcagcacctgttgtagcagcttttgcagtatttgttgtttatcca
attggtcaagctagcttttccgatggaatgcctttaggaataagtggaacttttaatttt
atgcttgtgttccaagcagaacataacattttaatgcatccattccatatcctaggagta
gctggtgtatttggaggttccttatttagtgcaatgcatggttcattagttacatcttca
cttcttgcagaaagtgcaggagatattagcctaaatgttggatataagtttggtcaagaa
gatgaaacttatagcatatcagcagcccatggttattttggtagactcatatttcaatat
gcttccttcaataactctcgtagtcttcacttcttcctagcagcttggccagttattggt
atttggttcacagcacttggagtaagtacaatggctttcaacttaaatggtttaaacttc
aaccaatccatcctagattccagtggccatctaatcttaagttgggcagatattgtgaat
cgtgctgatctaggtatggaagtgatgcatgaaagaaatgctcacaatttcccattagat
ctagcataa

>KrD3_psbA
atgaagaatagatcttactatcaactcaacttacttggtaatttcataggattcgtacta
tccacaacaaatcgtctctacattggatgctttggtatcctaatgttccctcttttaact
ttagcaactattgcttatatcacagcttttattcttgcacctgcagtagatattgatgga
ataagagaaccagtagctggctcacttctttatggcaataacattataacaggagctgta
ataccaagttctaatgctattggggttcatttctatccagtttgggagtcaaatggtttt
gatgagtgcttatataatggtggtacatatcaatttgtagtacttcattttatgcttggt
gttgcttgttggatgggtagagaatgggaattcagtttcagattaggtatgagaccatgg
atatttgtagctttctcagcacctgttgtagcagcttttgcagtatttgttgtttatcca
attggtcaagctagcttttccgatggaatgcctttaggaataagtggaacttttaatttt
atgcttgtgttccaagcagaacataacattttaatgcatccattccatatcctaggagta
gctggtgtatttggatgttccttatttagtgcaatgcatggttcattagttacatcttca
cttcttgcagaaagtgcaggagatattagcctaaatgttggatataagtttggtcaagaa
gatgaaacttatagcatatcagcagcccatggttattttggtagactcatatttcaatat
gcttccttcaataactctcgtagtcttcacttcttcctagcagcttggccagttattggt
atttggttcacagcacttggagtaagtacaatggctttcaacttaaatggtttaaacttc
aaccaatccatcctagattccagtggccatctaatcttaagttgggcagatattgtgaat
cgtgctgatctaggtatggaagtgatgcatgaaagaaatgctcacaatttcccattagat
ctagcataa
>KrD8_psbA
atgaagaacacatcttactatcaactcaacttacttggtaatgtcataggattcgtacta
tccacaacaaatcgtctctacattggatgctttggaatcctaatgtttcctcttttaact
ttagctactattgcttatatcaccgctttcattcttgcacctgcagtagatattgatgga
ataagagaaccagtagctggctcacttctttatggcaataacattataacaggagctgta
ataccaagttctaatgctattggggttcatttctatccagtttgggagtcaaaccatttc
gatgagtgcttatataatggtggaacatatcaattcgtagtccttcattttatgcttggt
gttgcttgttggatgggtagagaatgggaattcagtttcagattaggtatgagaccatgg
atatttgtagctttctcagcacctgttgtagcagcttttgcagtatttgttgtttatcca
attggtcaagctagcttttccgatggaatgcctttaggaataagtggaacttttaatttt
atgcttgtgttccaagcagaacataacattttaatgcatccattccatatcctaggagta
gctggtgtatttggaggttccttatttagtgcaatgcatggttcattagttacatcttca
cttcttgcagaaagtgcaggagatattagcctcaatgttggatataagtttggtcaagaa
gatgaaacttatagcatatcagccgctcatggttattttggtagactcatatttcaatat
gcttccttcaataactctcgtagccttcacttcttcctagcagcttggccagttattggt
atttggttcacagcacttggagtaagtacaatggctttcaacttaaatggtttaaacttc
aaccaatccatcctagattccagtggccatctaatcttaagttgggcagatattgtgaat
cgtgctgatctaggtatggaagtgatgcatgaaagaaatgctcacaatttcccattagat
ctagcataa
>KrD9_psbA
atgaagaatagatcttactatcaactcaacttacttggtaatgtcataggatttgtgcta
tccacaacaaatcgtctctatattgggtgctttggtatcctaatgttccctcttttaact
ttagcaactattgcttatatcacagcttttattcttgcacctgcagtagatattgatgga
ataagagaaccagtagctggctcacttctttatggcaacaacattataacaggagctgta
ataccaagttctaatgctattggggttcatttctatccagtttgggagtcaaatcgattt
gatgagtgcttatataatggtggtacatatcaattcgtagtccttcattttatgcttggt
gttgcttgttggatgggtagagaatgggaattcagttttagattaggtatgagaccatgg
atatttgtagctttctcagcacctgttattgcagcctttgcagtatttgttgtttacccg
atcggtcaagctagcttttccgatggaatgcctttaggaataagtggaacttttagtttt
atgcttgtgttccaagcagaacataacattttaatgcatccattccatatcctaggagta
gctggtgtatttggtggatcattgttcagtgcaatgcatggttcattagttacatcttca
cttcttgcagaaagtgcaggagatattagcctaaatgttggatataagtttggtcaagaa
gatgaaacttatagcatatcagccgctcatggttattttggtagactcatatttcaatat
gcttccttcaataactctcgtagtcttcacttcttcctagcagcttggccagttattggt
atttggttcacagcacttggagtaagtacaatggctttcaacttaaatggtttaaacttc
aaccaatccatcctagattccagtggccatctaatcttaagttgggcagatattgtgaat
cgtgctgatctaggtatggaagtgatgcatgaaagaaatgctcacaatttcccattagat
ctagcataa

>KrE1_psbA
atgaagaacacatcttactatcaactcaacttacttggtaatgtcataggattcgtacta
tccacaacaaatcgtctctacattgggtgctttggtatcctaatgttccctcttttaact
ttagcaactattgcttatatcacagcttttattcttgcacctgcagtagatattgatgga
ataagagaaccagtagctggctcacttctttatggcaataacattataacaggagctgta
ataccaagttctaatgctattggggttcatttctatccagtttgggagtcaaatggattt
gatgagtgcttatataatggtggtacatatcaatttgtagtacttcattttatgcttggt
gttgcttgttggatgggtagagaatgggaattcagtttcagattaggtatgagaccatgg
atatttgtagctttctcagcacctgttgtagcagcttttgcagtatttgttgtttatcca
attggtcaagctagcttttccgatggaatgcctttaggaataagtggaacttttaatttt
atgcttgtgttccaagcagaacataacattttaatgcatccattccatatcctaggagta
gctggtgtatttggaggttccttatttagtgcaatgcatggttcattagttacatcttca
cttcttgcagaaagtgcaggagatattagcctaaatgttggatataagtttggtcaagaa
gatgaaacttatagcatatcagcagcccatggttattttggtagactcatatttcaatat
gcttccttcaataactctcgtagtcttcacttcttcctagcagcttggccagttattggt
atttggttcacagcacttggagtaagtacaatggctttcaacttaaatggtttaaacttc
aaccaatccatcctagattccagtggccatctaatcttaagttgggcagatattgtgaat
cgtgctgatctaggtatggaagtgatgcatgaaagaaatgctcacaatttcccattagat
ctagcataa
>KrE10_psbA
atgaagaacacatcttactatcaactcaacttacttggtaatgtcataggattcgtacta
tccacaacaaatcgtctctacattgggtgctttggtatcctaatgttccctcttttaact
ttagcaactattgcttatatcacagcttttattcttgcacctgcagtagatattgatgga
ataagagaaccagtagctggctcacttctttatggcaataacattataacaggagctgta
ataccaagttctaatgctattggggttcatttctatccagtttgggagtcaaatggattt
gatgagtgcttatataatggtggtacatatcaatttgtagtacttcattttatgcttggt
gttgcttgttggatgggtagagaatgggaattcagttttagattaggtatgagaccatgg
atatttgtagctttctcagcacctgttgtagcagcttttgcagtatttgttgtttatcca
attggtcaagctagcttttctgatggaatgcctttaggaataagtggaacttttaatttt
atgcttgtgttccaagcagaacataacattttaatgcatccattccatatcctaggagta
gctggtgtatttggaggttccttatttagtgcaatgcatggttcattagttacatcttca
cttcttgcagaaagtgcaggagatattagcctaaatgttggatataagtttggtcaagaa
gatgaaacttatagcatatcagccgctcatggttattttggtagactcatatttcaatat
gcttccttcaataactctcgtagtcttcacttcttcctagcagcttggccagttattggt
atttggttcactgcccttggagtaagtacaatggctttcaacttaaatggtttaaacttc
aaccaatccatcctagattccagtggccatctaatcttaagttgggcagatattgtgaat
cgtgctgatctaggtatggaagtgatgcatgaaagaaatgctcacaatttcccattagat
ctagcataa
>KrE11_psbA
atgaagaacacatcttactatcaactcaacttacttggtaatgtcataggattcgtacta
tccacaacaaatcgtctctacattgggtgctttggtatcctaatgttccctcttttaact
ttagctactattgcttatatcaccgctttcattcttgcacctgcagtagatattgatgga
ataagagaaccagtagctggctcacttctttatggcaacaacattataacaggagctgta
ataccaagttctaatgctattggggttcatttctatccagtttgggagtcaaatggattt
gatgagtgcttatataatggtggtacatatcaatttgtagtacttcattttatgcttggt
gttgcttgttggatgggtagagaatgggaattcagtttcagattaggtatgagaccatgg
atatttgtagctttctcagcacctgttgtagcagcttttgcagtatttgttgtttatcca
attggtcaagctagcttttccgatggaatgcctttaggaataagtggaacttttaatttt
atgcttgtgttccaagcagaacataacattttaatgcatccattccatatcctaggagta
gctggtgtatttggaggttccttatttagtgcaatgcatggttcattagttacatcttca
cttcttgcagaaagtgcaggagatattagcctaaatgttggatataagtttggtcaagaa
gatgaaacttatagcatatcagcagcccatggttattttggtagactcatatttcaatat
gcttccttcaataactctcgtagtcttcacttctttctagcagcttggccagttattggt
atttggttcacagcacttggagtaagtacaatggctttcaacttaaatggtttaaacttc
aaccaatccatcctagattccagtggccatctaatcttaagttgggcagatattgtgaat
cgtgctgatctaggtatggaagtgatgcatgaaagaaatgctcacaatttcccattagat
ctagcataa

>KrE12_psbA
atgaagaacacatcttactatcaactcaacttacttggtaatgtcataggattcgtacta
tccacaacaaatcgtctctacattgggtgctttggtatcctaatgttccctcttttaact
ttagcaactattgcttatatcacagcttttattcttgcacctgcagtagatattgatgga
ataagagaaccagtagctggctcacttctttatggcaataacattataacaggagctgta
ataccaagttctaatgctattggggttcatttctatccagtttgggagtcaaatggattt
gatgagtgcttatataatggtggtacatatcaatttgtagtacttcattttatgcttggt
gttgcttgttggatgggtagagaatgggaattcagtttcagattaggtatgagaccatgg
atatttgtagctttctcagcacctgttgtagcagcttttgcagtatttgttgtttatcca
attggtcaagctagcttttccgatggaatgcctttaggaataagtggaacttttaatttt
atgcttgtgttccaagcagaacataacatcttaatgcatccattccatatcctaggagta
gctggtgtatttggaggttccttatttagtgcaatgcatggttcattagttacatcttca
cttcttgcagaaagtgcaggagatattagcctaaatgttggatataagtttggtcaagaa
gatgaaacttatagcatatcagcagcccatggttattttggtagactcatatttcaatat
gcttccttcaataactctcgtagtcttcacttcttcctagcagcttggccagttattggt
atttggttcacagcacttggagtaagtacaatggctttcaacttaaatggtttaaacttc
aaccaatccatcctagattccagtggccatctaatcttaagttgggcagatattgtgaat
cgtgctgatctaggtatggaagtgatgcatgaaagaaatgctcacaatttcccattagat
ctagcataa
>KrE13_psbA
atgaagaacacatcttactatcaactcaacttacttggtaatgtcataggattcgtacta
tccacaacaaatcgtctctacattgggtgctttggtatcctaatgttccctcttttaact
ttagcaactattgcttatatcacagcttttattcttgcacctgcagtagatattgatgga
ataagagaaccagtagctggctcacttctttatggcaataacattataacaggagctgta
ataccaagttctaatgctattggggttcatttctatccagtttgggagtcaaatggattt
gatgagtgcttatataatggtggtacatatcaatttgtagtacttcattttatgcttggt
gttgcttgttggatgggtagagaatgggaattcagtttcagattaggtatgagaccatgg
atatttgtagctttctcagcacctgttgtagcagcttttgcagtatttgttgtttatcca
attggtcaagctagcttttccgatggaatgcctttaggaataagtggaacttttaatttt
atgcttgtgttccaagcagaacataacattttaatgcatccattccatatcctaggagta
gctggtgtatttggaggttccttatttagtgcaatgcatggttcattagttacatcttca
cttcttgcagaaagtgcaggagatattagcctaaatgttggatataagtttggtcaagaa
gatgaaacttatagcatatcagcagcccatggttattttggtagactcatatttcaatat
gcttccttcaataactctcgtagtcttcacttcttcctagcagcttggccagttattggt
atttggttcacagcacttggagtaagtacaatggctttcaacttaaatggtttaaacttc
aaccaatccatcctagattccagtggccatctaatcttaagttgggcagatattgtgaat
cgtgctgatctaggtatggaagtgatgcatgaaagaaatgctcacaatttcccattagat
ctagcataa
>KrE14_psbA
atgaagaatagatcttactatcaactcaacttacttggtaatgtcataggattcgtacta
tccacaacaaatcgtctctacattgggtgctttggtatcctaatgttccctcttttaact
ttagcaactattgcttatatcacagcttttattcttgcacctgcagtagatattgatgga
ataagagaaccagtagctggctcacttctttatggcaataacattataacaggagctgta
ataccaagttctaatgctattggggttcatttctatccagtttgggagtcaaaccatttc
gatgagtgcttatataatggtggaacatatcaattcgtagtccttcattttatgcttggt
gttgcttgttggatgggtagagaatgggaattcagtttcagattaggtatgagaccatgg
atatttgtagctttctcagcacctgttgtagcagcttttgcagtatttgttgtttatcca
attggtcaagctagcttttccgatggaatgcctttaggaataagtggaacttttaatttt
atgcttgttttccaagcagagcataacatcttaatgcatccattccatattctaggagta
gctggagtatttggtggatcattgttcagtgcaatgcatggttcattagttacatcttca
cttcttgcagaaagtgcaggagatattagcctaaatgttggatataagtttggtcaagaa
gatgaaacttatagcatatcagccgctcatggttattttggtagactcatatttcaatat
gcttccttcaataactctcgtagtcttcacttcttcctagcagcttggccagttattggt
atttggttcacagcacttggagtaagtacaatggctttcaacttaaatggtttaaacttc
aaccaatccatcctagattccagtggccatctaatcttaagttgggcagatattgtgaat
cgtgctgatctaggtatggaagtgatgcatgaaagaaatgctcacaatttcccattagat
ctagcataa

>KrE15_psbA
atgaagaacacatcttactatcaactcaacttacttggtaatgtcataggattcgtacta
tccacaacaaatcgtctctacattgggtgctttggtatcctaatgttccctcttttaact
ttagcaactattgcttatatcacagcttttattcttgcacctgcagtagatattgatgga
ataagagaaccagtagctggctcacttctttatggcaataacattataacaggagctgta
ataccaagttctaatgctattggggttcatttctatccagtttgggagtcaaatggattt
gatgagtgcttatataatggtggtacatatcaatttgtagtacttcattttatgcttggt
gttgcttgttggatgggtagagaatgggaattcagttttagattaggtatgagaccatgg
atatttgtagctttctcagcacctgttgtagcagcttttgcagtatttgttgtttatcca
attggtcaagctagcttttccgatggaatgcctttaggaataagtggaacttttaatttt
atgcttgtgttccaagcagaacataacattttaatgcatccattccatatcctaggagta
gctggtgtatttggaggttccttatttagtgcaatgcatggttcattagttacatcttca
cttcttgcagaaagtgcaggagatattagcctaaatgttggatataagtttggtcaagaa
gatgaaacttatagcatatcagcagcccatggttattttggtagactcatatttcaatat
gcttccttcaataactctcgtagtcttcacttcttcctagcagcttggccagttattggt
atttggttcactgcccttggagtaagtacaatggctttcaacttaaatggtttaaacttc
aaccaatccatcctagattccagtggccatctaatcttaagttgggcagatattgtgaat
cgtgctgatctaggtatggaagtgatgcatgaaagaaatgctcacaatttcccattagat
ctagcataa
>KrE2_psbA
atgaagaacacatcttactatcaactcaacttacttggtaatgtcataggattcgtacta
tccacaacaaatcgtctctacattgggtgctttggtatcctaatgttccctcttttaact
ttagcaactattgcttatatcacagcttttattcttgcacctgcagtagatattgatgga
ataagagaaccagtagctggctcacttctttatggcaataacattataacaggagctgta
ataccaagttctaatgctattggggttcatttctatccagtttgggagtcaaatggattt
gatgagtgcttatataatggtggtacatatcaatttgtagtacttcattttatgcttggt
gttgcttgttggatgggtagagaatgggaattcagtttcagattaggtatgagaccatgg
atatttgtagctttctcagcacctgttgtagcagcttttgcagtatttgttgtttatcca
attggtcaagctagcttttccgatggaatgcctttaggaataagtggaacttttaatttt
atgcttgtgttccaagcagaacataacattttaatgcatccattccatatcctaggagta
gctggtgtatttggaggttccttatttagtgcaatgcatggttcattagttacatcttca
cttcttgcagaaagtgcaggagatattagcctaaatgttggatataagtttggtcaagaa
gatgaaacttatagcatatcagcagcccatggttattttggtagactcatatttcaatat
gcttccttcaataactctcgtagtcttcacttcttcctagcagcttggccagttattggt
atttggttcacagcacttggagtaagtacaatggctttcaacttaaatggtttaaacttc
aaccaatccatcctagattccagtggccatctaatcttaagttgggcagatattgtgaat
cgtgctgatctaggtatggaagtgatgcatgaaagaaatgctcacaatttcccattagat
ctagcataa
>KrE3_psbA
atgaagaacacatcttactatcaactcaacttacttggtaatgtcataggattcgtacta
tccacaacaaatcgtctctacattgggtgctttggtatcctaatgttccctcttttaact
ttagcaactattgcttatatcacagcttttattcttgcacctgcagtagatattgatgga
ataagagaaccagtagctggatcacttctttatggcaataacattataacaggagctgta
ataccaagttctaatgctattggtgttcatttctatccagtctgggaatcaaaccatttc
gatgagtgcttatataatggtggaacatatcaattcgtagtccttcattttatgcttggt
gttgcttgttggatgggtagagaatgggaattcagtttcagattaggtatgagaccatgg
atatttgtagctttctcagcacctgttgtagcagcttttgcagtatttgttgtttatcca
attggtcaagctagcttttccgatggaatgcctttaggaataagtggaacttttaatttt
atgcttgtgttccaagcagaacataacattttaatgcatccattccatatcctaggagta
gctggtgtatttggaggttccttatttagtgcaatgcatggttcattagttacatcttca
cttcttgcagaaagtgcaggagatattagcctaaatgttggatataagtttggtcaagaa
gatgaaacttatagcatatcagcagcccatggttattttggtagactcatatttcaatat
gcttccttcaataactctcgtagtcttcacttcttcctagcagcttggccagttattggt
atttggttcactgcccttggagtaagtacaatggctttcaacttaaatggtttaaacttc
aaccaatccatcctagattccagtggccatctaatcttaagttgggcagatattgtgaat
cgtgctgatctaggtatggaagtgatgcatgaaagaaatgctcacaatttcccattagat
ctagcataa

>KrE4_psbA
atgaagaacacatcttactatcaactcaacttacttggtaatgtcataggattcgtacta
tccacaacaaatcgtctctacattgggtgctttggtatcctaatgttccctcttttaact
ttagcaactattgcttatatcacagcttttattcttgcacctgcagtagatattgatgga
ataagagaaccagtagctggctcacttctttatggcaataacattataacaggagctgta
atcccaagttctaatgctattggggttcatttctatccagtttgggagtcaaatggattt
gatgagtgcttatataatggtggtacatatcaatttgtagtacttcattttatgcttggt
gttgcttgttggatgggtagagaatgggaattcagtttcagattaggtatgagaccatgg
atatttgtagctttctcagcacctgttattgcagcctttgcagtatttgttgtttatcca
attggtcaagctagcttttccgatggaatgcctttaggaataagtggaacttttaatttt
atgcttgtgttccaagcagaacataacatcttaatgcatccattccatattctaggagta
gctggtgtatttggaggttccttatttagtgcaatgcatggttcattagttacatcttca
cttcttgcagaaagtgcaggagatattagcctaaatgttggatataagtttggtcaagaa
gatgaaacttatagcatatcagcagcccatggttattttggtagactcatatttcaatat
gcttccttcaataactctcgtagtcttcacttcttcctagcagcttggccagttattggt
atttggttcactgcccttggagtaagtacaatggctttcaacttaaatggtttaaacttc
aaccaatccatcctagattccagtggccatctaatcttaagttgggcagatattgtgaat
cgtgctgatctaggtatggaagtgatgcatgaaagaaatgctcataatttcccattagat
ctagcataa
>KrE5_psbA
atgaagaacacatcttactatcaactcaacttacttggtaatgtcataggattcgtacta
tccacaacaaatcgtctctacattgggtgctttggtatcctaatgttccctcttttaact
ttagcaactattgcttatatcacagcttttattcttgcacctgcagtagatattgatgga
ataagagaaccagtagctggctcacttctttatggcaataacattataacaggagctgta
ataccaagttctaatgctattggggttcatttctatccagtttgggagtcaaatggattt
gatgagtgcttatataatggtggtacatatcaatttgtagtacttcattttatgcttggt
gttgcttgttggatgggtagagaatgggaattcagtttcagattaggtatgagaccatgg
atatttgtagctttctcagcacctgttgtagcagcttttgcagtatttgttgtttatcca
attggtcaagctagcttttccgatggaatgcctttaggaataagtggaacttttaatttt
atgcttgtgttccaagcagaacataacattttaatgcatccattccatatcctaggagta
gctggtgtatttggaggttccttatttagtgcaatgcatggttcattagttacatcttca
cttcttgcagaaagtgcaggagatattagcctaaatgttggatataagtttggtcaagaa
gatgaaacttatagcatatcagcagcccatggttattttggtagactcatatttcaatat
gcttccttcaataactctcgtagtcttcacttcttcctagcagcttggccagttattggt
atttggttcacagcacttggagtaagtacaatggctttcaacttaaatggtttaaacttc
aaccaatccatcctagattccagtggccatctaatcttaagttgggcagatattgtgaat
cgtgctgatctaggtatggaagtgatgcatgaaagaaatgctcacaatttcccattagat
ctagcataa
>KrE6_psbA
atgaagaacacatcttactatcaactcaacttacttggtaatgtcataggattcgtacta
tccacaacaaatcgtctctacattgggtgctttggtatcctaatgttccctcttttaact
ttagcaactattgcttatatcacagcttttattcttgcacctgcagtagatattgatgga
ataagagaaccagtagctggctcacttctttatggcaataacattataacaggagctgta
ataccaagttctaatgctattggggttcatttctatccagtttgggagtcaaatggattt
gatgagtgcttatataatggtggtacatatcaatttgtagtacttcattttatgcttggt
gttgcttgttggatgggtagagaatgggaattcagtttcagattaggtatgagaccatgg
atatttgtagctttctcagcacctgttgtagcagcttttgcagtatttgttgtttatcca
attggtcaagctagcttttccgatggaatgcctttaggaataagtggaacttttaatttt
atgcttgtgttccaagcagaacataacattttaatgcatccattccatatcctaggagta
gctggtgtatttggaggttccttatttagtgcaatgcatggttcattagttacatcttca
cttcttgcagaaagtgcaggagatattagcctaaatgttggatataagtttggtcaagaa
gatgaaacttatagcatatcagcagcccatggttattttggtagactcatatttcaatat
gcttccttcaataactctcgtagtcttcacttcttcctagcagcttggccagttattggt
atttggttcacagcacttggagtaagtacaatggctttcaacttaaatggtttaaacttc
aaccaatccatcctagattccagtggccatctaatcttaagttgggcagatattgtgaat
cgtgctgatctaggtatggaagtgatgcatgaaagaaatgctcacaatttcccattagat
ctagcataa

>KrE7_psbA
atgaagaacacatcttactatcaactcaacttacttggtaatgtcataggattcgtacta
tccacaacaaatcgtctctacattgggtgctttggtatcctaatgttccctcttttaact
ttagcaactattgcttatatcacagcttttattcttgcacctgcagtagatattgatgga
ataagagaaccagtagctggctcacttctttatggcaataacattataacaggagctgta
ataccaagttctaatgctattggggttcatttctatccagtttgggagtcaaaccatttc
gatgagtgcttatataatggtggtacatatcaatttgtagtacttcattttatgcttggt
gttgcttgttggatgggtagagaatgggaattcagtttcagattaggtatgagaccatgg
atatttgtagctttctcagcacctgttgtagcagcttttgcagtatttgttgtttatcca
attggtcaagctagcttttccgatggaatgcctttaggaataagtggaacttttaatttt
atgcttgtgttccaagcagaacataacattttaatgcatccattccatatcctaggagta
gctggtgtatttggtggatcattgttcagtgcaatgcatggttcattagttacatcttca
cttcttgcagaaagtgcaggagatattagcctaaatgttggatataagtttggtcaagaa
gatgaaacttatagcatatcagcagcccatggttattttggtagactcatatttcaatat
gcttccttcaataactctcgtagccttcacttctttctagcagcttggccagttattggt
atttggttcactgcccttggagtaagtacaatggctttcaacttaaatggtttaaacttc
aaccaatccatcctagattccagtggccatctaatcttaagttgggcagatattgtgaat
cgtgctgatctaggtatggaagtgatgcatgaaagaaatgctcacaatttcccattagat
ctagcataa
>KrE8_psbA
atgaagaacacatcttactatcaactcaacttacttggtaatgtcataggattcgtacta
tccacaacaaatcgtctctacattgggtgctttggtatcctaatgttccctcttttaact
ttagcaactattgcttatatcacagcttttattcttgcacctgcagtagatattgatgga
ataagagaaccagtagctggctcacttctttatggcaataacattataacaggagctgta
ataccaagttctaatgctattggggttcatttctatccagtttgggagtcaaatggattt
gatgagtgcttatataatggtggtacatatcaatttgtagtacttcattttatgcttggt
gttgcttgttggatgggtagagaatgggaattcagtttcagattaggtatgagaccatgg
atatttgtagctttctcagcacctgttgtagcagcttttgcagtatttgttgtttatcca
attggtcaagctagcttttccgatggaatgcctttaggaataagtggaacttttaatttt
atgcttgtgttccaagcagaacataacattttaatgcatccattccatatcctaggagta
gctggtgtatttggaggttccttatttagtgcaatgcatggttcattagttacatcttca
cttcttgcagaaagtgcaggagatattagcctaaatgttggatataagtttggtcaagaa
gatgaaacttatagcatatcagcagcccatggttattttggtagactcatatttcaatat
gcttccttcaataactctcgtagtcttcacttcttcctagcagcttggccagttattggt
atttggttcacagcacttggagtaagtacaatggctttcaacttaaatggtttaaacttc
aaccaatccatcctagattccagtggccatctaatcttaagttgggcagatattgtgaat
cgtgctgatctaggtatggaagtgatgcatgaaagaaatgctcacaatttcccattagat
ctagcataa
>KrE9_psbA
atgaagaacacatcttactatcaactcaacttacttggtaatgtcataggattcgtacta
tccacaacaaatcgtctctacattgggtgctttggtatcctaatgttccctcttttaact
ttagcaactattgcttatatcacagcttttattcttgcacctgcagtagatattgatgga
ataagagaaccagtagctggctcacttctttatggcaataacattataacaggagctgta
ataccaagttctaatgctattggggttcatttctatccagtttgggagtcaaatggattt
gatgagtgcttatataatggtggtacatatcaatttgtagtacttcattttatgcttggt
gttgcttgttggatgggtagagaatgggaattcagtttcagattaggtatgagaccatgg
atatttgtagctttctcagcacctgttgtagcagcttttgcagtatttgttgtttatcca
attggtcaagctagcttttccgatggaatgcctttaggaataagtggaacttttaatttt
atgcttgtgttccaagcagaacataacattttaatgcatccattccatatcctaggagta
gctggtgtatttggaggttccttatttagtgcaatgcatggttcattagttacatcttca
cttcttgcagaaagtgcaggagatattagcctcaatgttggatataagtttggtcaagaa
gatgaaacttatagcatatcagccgctcatggttattttggtagactcatatttcaatat
gcttccttcaataactctcgtagccttcacttctttctagcagcttggccagttattggt
atttggttcacagcacttggagtaagtacaatggctttcaacttaaatggtttaaacttc
aaccaatccatcctagattccagtggccatctaatcttaagttgggcagatattgtgaat
cgtgctgatctaggtatggaagtgatgcatgaaagaaatgctcacaatttcccattagat
ctagcataa

>Mz10_psbA
atgaagaatagatcttactatcaactcaacttacttggtaatgtcataggattcgtacta
tccacaacaaatcgtctctacattgggtgctttggtatcctaatgttccctcttttaact
ttagctactattgcttatatcaccgctttcattcttgcacctgcagtagatattgatgga
ataagagaaccagtagctggctcacttctttatggcaataacattataacaggagctgta
ataccaagttctaatgctattggtgttcatttctatccagtctgggaatcaaaccatttc
gatgagtgcttatataatggtggtacatatcaatttgtagtccttcattttatgcttggt
gttgcttgttggatgggtagagaatgggaattcagtttcagattaggtatgagaccatgg
atatttgtagctttctcagcacctgttgtagcagcttttgcagtatttgttgtttatcca
attggtcaagctagcttttccgatggaatgcctttaggaataagtggaacttttaatttt
atgcttgttttccaagcagaacataacattttaatgcatccattccatatcctaggagta
gctggtgtatttggaggttccttatttagtgcaatgcatggttcattagttacatcttca
cttcttgcagaaagtgcaggagatattagcctaaatgttggatataagtttggtcaagaa
gatgaaacttatagcatatcagcagcccatggttattttggtagactcatatttcaatat
gcttccttcaataactctcgtagccttcacttcttcctagcagcttggccagttattggt
atttggttcacagcacttggagtaagtacaatggctttcaacttaaatggtttaaacttc
aaccaatccatcctagattccagtggccatctaatcttaagttgggcagatattgtgaat
cgtgctgatctaggtatggaagtgatgcgtgaaagaaatgctcataatttcccattagat
ctagcataa
>Mz5_psbA
atgaagaacacatcttactatcaactcaacttacttggtaatgtcataggattcgtacta
tccacaacaaatcgtctctacattgggtgctttggtatcctaatgttccctcttttaact
ttagcaactattgcttatatcacagcttttattcttgcacctgcagtagatattgatgga
ataagagaaccagtagctggctcacttctttatggcaataacattataacaggagctgta
ataccaagttctaatgctattggggttcatttctatccagtttgggagtcaaaccatttc
gatgagtgcttatataatggtggaacatatcaattcgtagtacttcattttatgcttggt
gttgcttgttggatgggtagagaatgggaattcagtttcagattaggtatgagaccatgg
atatttgtagctttctcagcacctgttgtagcagcttttgcagtatttgttgtttatcca
attggtcaagctagcttttccgatggaatgcctttaggaataagtggaacttttaatttt
atgcttgtgttccaagcagaacataacattttaatgcatccattccatatcctaggagta
gctggtgtatttggaggttccttatttagtgcaatgcatggttcattagttacatcttca
cttcttgcagaaagtgcaggagatattagcctaaatgttggatataagtttggtcaagaa
gatgaaacttatagcatatcagcagcccatggttattttggtagactcatatttcaatat
gcttccttcaataactctcgtagtcttcacttcttcctagcagcttggccagttattggt
atttggttcacagcacttggagtaagtacaatggctttcaacttaaatggtttaaacttc
aaccaatccatcctagattccagtggccatctaatcttaagttgggcagatattgtgaat
cgtgctgatctaggtatggaagtgatgcatgaaagaaatgctcacaatttcccattagat
ctagcataa
>Mz6_psbA
atgaagaacacatcttactatcaactcaacttacttggtaatgtcataggattcgtacta
tccacaacaaatcgtctctacattgggtgctttggtatcctaatgttccctcttttaact
ttagcaactattgcttatatcacagcttttattcttgcacctgcagtagatattgatgga
ataagagaaccagtagctggctcacttctttatggcaataacattataacaggagctgta
ataccaagttctaatgctattggggttcatttctatccagtttgggagtcaaatcaattt
gatgagtgcttatataatggtggtacatatcaatttgtagtacttcattttatgcttggt
gttgcttgttggatgggtagagaatgggaattcagtttcagattaggtatgagaccatgg
atatttgtagctttctcagcacctgttgtagcagcttttgcagtatttgttgtttatcca
attggtcaagctagcttttccgatggaatgcctttaggaataagtggtacttttaatttt
atgcttgtgttccaagcagagcataacattttaatgcatccattccatatcctaggagta
gctggtgtatttggaggttccttatttagtgcaatgcatggttcattagttacatcttca
cttcttgcagaaagtgcaggagatattagcctaaatgttggatataagtttggtcaagaa
gatgaaacttatagcatatcagcagcccatggttattttggtagactcatatttcaatat
gcttccttcaataactctcgtagtcttcacttcttcctagcagcttggccagttattggt
atttggttcacagcacttggagtaagtacaatggctttcaacttaaatggtttaaacttc
aaccaatccatcctagattccagtggccatctaatcttaagttgggcagatattgtgaat
cgtgctgatctaggtatggaagtgatgcatgaaagaaatgctcacaatttcccattagat
ctagcataa

>Mz7_psbA
atgaagaacacatcttactatcaactcaacttacttggtaatgtcataggattcgtacta
tccacaacaaatcgtctctacattgggtgctttggtatcctaatgttccctcttttaact
ttagctactattgcttatatcaccgctttcattcttgcacctgcagtagatattgatgga
ataagagaaccagtagctggctcacttctttatggcaacaacattataacaggagctgta
atcccgagttctaatgctattggtgttcatttctatccagtctgggaatcaaaccatttc
gatgagtgcttatataatggtggtacatatcaatttgtagtacttcattttatgcttggt
gttgcttgttggatgggtagagaatgggaattcagtttcagattaggtatgagaccatgg
atatttgtagctttctcagcacctgttgtagcagcttttgcagtatttgttgtttatcca
attggtcaagctagcttttccgatggaatgcctttaggaataagtggaacttttaatttt
atgcttgtgttccaagcagaacataacattttaatgcatccattccatatcctaggagta
gctggtgtatttggaggttccttatttagtgcaatgcatggttcattagttacatcttca
cttcttgcagaaagtgcaggagatattagcctaaatgttggatataagtttggtcaagaa
gatgaaacttatagcatatcagcagcccatggttattttggtagactcatatttcaatat
gcttccttcaataactctcgtagtcttcacttcttcctagcagcttggccagttattggt
atttggttcacagcacttggagtaagtacaatggctttcaacttaaatggtttaaacttc
aaccaatccatcctagattccagtggccatctaatcttaagttgggcagatattgtgaat
cgtgctgatctaggtatggaagtgatgcgtgaaagaaatgctcataatttcccattagat
ctagcataa
>Mz8_psbA
atgaagaacacatcttactatcaactcaacttacttggtaatgtcataggattcgtacta
tccacaacaaatcgtctctacattgggtgctttggtatcctaatgttccctcttttaact
ttagcaactattgcttatatcaccgcttttattcttgcacctgcagtagatattgatgga
ataagagaaccagtagctggctcacttctttatggcaacaacattataacaggagctgta
atcccgagttctaatgctattggtgttcatttctatccagtctgggaatcaaaccatttc
gatgagtgcttatataatggtggaacatatcaattcgtagtccttcattttatgcttggt
gttgcttgttggatgggtagagaatgggaattcagtttcagattaggtatgagaccatgg
atatttgtagctttctcagcacctgttgtagcagcttttgcagtatttgttgtttacccg
atcggtcaagctagcttttctgatggaatgcctttaggaataagtggtacttttaatttt
atgcttgttttccaagcagagcataacatcttaatgcatccattccatatcctaggagta
gctggtgtatttggaggttccttatttagtgcaatgcatggttcattagttacatcttca
cttcttgcagaaagtgcaggagatattagcctcaatgttggatataagtttggtcaagaa
gatgaaacttatagcatatcagcagctcatggttattttggtagactcatatttcaatat
gcttccttcaataactctcgtagtcttcacttcttcctagcagcttggccagttattggt
atttggttcactgcccttggagtaagtacaatggctttcaacttaaatggtttaaacttc
aaccaatccatcctagattccagtggccatctaatcttaagttgggcagatattgtgaat
cgtgctgatctaggtatggaagtgatgcatgaaagaaatgctcacaatttcccattagat
ctagcataa
>Mz9_psbA
atgaagaatagatcttactatcaactcaacttacttggtaatgtcataggattcgtacta
tccacaacaaatcgtctctacattgggtgctttggtatcctaatgtttcctcttttaact
ttagctactattgcttatatcaccgctttcattcttgcacctgcagtagatattgatgga
ataagagaaccagtagctggctcacttctttatggcaataacattataacaggagctgta
ataccaagttctaatgctattggggttcatttctatccagtttgggagtcaaatggattt
gatgagtgcttatataatggtggtacatatcaatttgtagtacttcattttatgcttggt
gttgcttgttggatgggtagagaatgggaattcagttttagattaggtatgagaccatgg
atatttgtagctttctcagcacctgttgtagcagcttttgcagtatttgttgtttatcca
attggtcaagctagcttttctgatggaatgcctttaggaataagtggaacttttaatttt
atgcttgtgttccaagcagaacataacattttaatgcatccattccatattctaggagta
gctggagtatttggtggatcattgttcagtgcaatgcatggttcattagttacatcttca
cttcttgcagaaagtgcaggagatattagcctcaatgttggatataagtttggtcaagaa
gatgaaacttatagcatatcagcagcccatggttattttggtagactcatatttcaatat
gcttccttcaataactctcgtagtcttcacttcttcctagcagcttggccagttattggt
atttggttcacagcacttggagtaagtacaatggctttcaacttaaatggtttaaacttc
aaccaatccatcctagattccagtggccatctaatcttaagttgggcagatattgtgaat
cgtgctgatctaggtatggaagtgatgcgtgaaagaaatgctcataatttcccattagat
ctagcataa

>MzC1_psbA
atgaagaacacatcttactatcaactcaacttacttggtaatgtcataggattcgtacta
tccacaacaaatcgtctctacattgggtgctttggtatcctaatgttccctcttttaact
ttagcaactattgcttatatcacagcttttattcttgcacctgcagtagatattgatgga
ataagagaaccagtagctggctcacttctttatggcaataacattataacaggagctgta
ataccaagttctaatgctattggtgttcatttctatccagtttgggagtcaaatggattt
gatgagtgcttatataatggtggtacatatcaatttgtagtacttcattttatgcttggt
gttgcttgttggatgggtagagaatgggaattcagtttcagattaggtatgagaccatgg
atatttgtagctttctcagcacctgttgtagcagcttttgcagtatttgttgtttatcca
attggtcaagctagcttttccgatggaatgcctttaggaataagtggaacttttaatttt
atgcttgtgttccaagcagaacataacattttaatgcatccattccatatcctaggagta
gctggtgtatttggaggttccttatttagtgcaatgcatggttcattagttacatcttca
cttcttgcagaaagtgcaggagatattagcctcaatgttggatataagtttggtcaagaa
gatgaaacttatagcatatcagcagcccatggttattttggtagactcatatttcaatat
gcttccttcaataactctcgtagtcttcacttcttcctagcagcttggccagttattggt
atttggttcacagcacttggagtaagtacaatggctttcaacttaaatggtttaaacttc
aaccaatccatcctagattccagtggccatctaatcttaagttgggcagatattgtgaat
cgtgctgatctaggtatggaagtgatgcatgaaagaaatgctcacaatttcccattagat
ctagcataa
>MzC1GaII_psbA
atgaagaacacatcttactatcaactcaacttacttggtaatgtcataggattcgtacta
tccacaacaaatcgtctctacattgggtgctttggtatcctaatgttccctcttttaact
ttagcaactattgcttatatcacagcttttattcttgcacctgcagtagatattgatgga
ataagagaaccagtagctggctcacttctttatggcaataacattataacaggagctgta
ataccaagttctaatgctattggggttcatttctatccagtttgggagtcaaatggattt
gatgagtgcttatataatggtggtacatatcaatttgtagtacttcattttatgcttggt
gttgcttgttggatgggtagagaatgggaattcagtttcagattaggtatgagaccatgg
atatttgtagctttctcagcacctgttgtagcagcttttgcagtatttgttgtttatcca
attggtcaagctagcttttccgatggaatgcctttaggaataagtggaacttttaatttt
atgcttgtgttccaagcagaacataacattttaatgcatccattccatatcctaggagta
gctggtgtatttggaggttccttatttagtgcaatgcatggttcattagttacatcttca
cttcttgcagaaagtgcaggagatattagcctaaatgttggatataagtttggtcaagaa
gatgaaacttatagcatatcagcagcccatggttattttggtagactcatatttcaatat
gcttccttcaataactctcgtagtcttcacttcttcctagcagcttggccagttattggt
atttggttcacagcacttggagtaagtacaatggctttcaacttaaatggtttaaacttc
aaccaatccatcctagattccagtggccatctaatcttaagttgggcagatattgtgaat
cgtgctgatctaggtatggaagtgatgcatgaaagaaatgctcacaatttcccattagat
ctagcataa
>MzC2_psbA
atgaagaacacatcttactatcaactcaacttacttggtaatgtcataggattcgtacta
tccacaacaaatcgtctctacattgggtgctttggtatcctaatgttccctcttttaact
ttagcaactattgcttatatcacagcttttattcttgcacctgcagtagatattgatgga
ataagagaaccagtagctggctcacttctttatggcaataacattataacaggagctgta
ataccaagttctaatgctattggggttcatttctatccagtttgggagtcaaatggattt
gatgagtgcttatataatggtggtacatatcaatttgtagtacttcattttatgcttggt
gttgcttgttggatgggtagagaatgggaattcagtttcagattaggtatgagaccatgg
atatttgtagctttctcagcacctgttgtagcagcttttgcagtatttgttgtttatcca
attggtcaagctagcttttccgatggaatgcctttaggaataagtggaacttttaatttt
atgcttgtgttccaagcagaacataacattttaatgcatccattccatatcctaggagta
gctggtgtatttggaggttccttatttagtgcaatgcatggttcattagttacatcttca
cttcttgcagaaagtgcaggagatattagcctaaatgttggatataagtttggtcaagaa
gatgaaacttatagcatatcagcagcccatggttattttggtagactcatatttcaatat
gcttccttcaataactctcgtagtcttcacttcttcctagcagcttggccagttattggt
atttggttcacagcacttggagtaagtacaatggctttcaacttaaatggtttaaacttc
aaccaatccatcctagattccagtggccatctaatcttaagttgggcagatattgtgaat
cgtgctgatctaggtatggaagtgatgcatgaaagaaatgctcacaatttcccattagat
ctagcataa

>MzC2GaII_psbA
atgaagaacacatcttactatcaactcaacttacttggtaatgtcataggattcgtacta
tccacaacaaatcgtctctacattgggtgctttggtatcctaatgttccctcttttaact
ttagcaactattgcttatatcacagcttttattcttgcacctgcagtagatattgatgga
ataagagaaccagtagctggctcacttctttatggcaataacattataacaggagctgta
ataccaagttctaatgctattggggttcatttctatccagtttgggagtcaaatggattt
gatgagtgcttatataatggtggtacatatcaatttgtagtacttcattttatgcttggt
gttgcttgttggatgggtagagaatgggaattcagtttcagattaggtatgagaccatgg
atatttgtagctttctcagcacctgttgtagcagcttttgcagtatttgttgtttatcca
attggtcaagctagcttttccgatggaatgcctttaggaataagtggaacttttaatttt
atgcttgtgttccaagcagaacataacattttaatgcatccattccatatcctaggagta
gctggtgtatttggaggttccttatttagtgcaatgcatggttcattagttacatcttca
cttcttgcagaaagtgcaggagatattagcctaaatgttggatataagtttggtcaagaa
gatgaaacttatagcatatcagcagcccatggttattttggtagactcatatttcaatat
gcttccttcaataactctcgtagtcttcacttcttcctagcagcttggccagttattggt
atttggttcacagcacttggagtaagtacaatggctttcaacttaaatggtttaaacttc
aaccaatccatcctagattccagtggccatctaatcttaagttgggcagatattgtgaat
cgtgctgatctaggtatggaagtgatgcatgaaagaaatgctcacaatttcccattagat
ctagcataa
>MzC3_psbA
atgaagaacacatcttactatcaactcaacttacttggtaatgtcataggattcgtacta
tccacaacaaatcgtctctacattgggtgctttggtatcctaatgttccctcttttaact
ttagcaactattgcttatatcacagcttttattcttgcacctgcagtagatattgatgga
ataagagaaccagtagctggctcacttctttatggcaataacattataacaggagctgta
ataccaagttctaatgctattggggttcatttctatccagtttgggagtcaaaccatttc
gatgagtgcttatataatggtggtacatatcaatttgtagtacttcattttatgcttggt
gttgcttgttggatgggtagagaatgggaattcagttttagattaggtatgagaccatgg
atatttgtagctttctcagcacctgttgtagcagcttttgcagtatttgttgtttatcca
attggtcaagctagcttttccgatggaatgcctttaggaataagtggaacttttaatttt
atgcttgtgttccaagcagaacataacattttaatgcatccattccatatcctaggagta
gctggtgtatttggaggttccttatttagtgcaatgcatggttcattagttacatcttca
cttcttgcagaaagtgcaggagatattagcctaaatgttggatataagtttggtcaagaa
gatgaaacttatagcatatcagcagcccatggttattttggtagactcatatttcaatat
gcttccttcaataactctcgtagtcttcacttcttcctagcagcttggccagttattggt
atttggttcacagcacttggagtaagtacaatggctttcaacttaaatggtttaaacttc
aaccaatccatcctagattccagtggccatctaatcttaagttgggcagatattgtgaat
cgtgctgatctaggtatggaagtgatgcatgaaagaaatgctcacaatttcccattagat
ctagcataa
>MzC4_psbA
atgaagaacacatcttactatcaactcaacttacttggtaatgtcataggattcgtacta
tccacaacaaatcgtctctacattgggtgctttggtatcctaatgttccctcttttaact
ttagcaactattgcttatatcacagcttttattcttgcacctgcagtagatattgatgga
ataagagaaccagtagctggctcacttctttatggcaataacattataacaggagctgta
ataccaagttctaatgctattggggttcatttctatccagtttgggagtcaaatggattt
gatgagtgcttatataatggtggtacatatcaatttgtagtacttcattttatgcttggt
gttgcttgttggatgggtagagaatgggaattcagtttcagattaggtatgagaccatgg
atatttgtagctttctcagcacctgttgtagcagcttttgcagtatttgttgtttatcca
attggtcaagctagcttttccgatggaatgcctttaggaataagtggaacttttaatttt
atgcttgtgttccaagcagaacataacattttaatgcatccattccatatcctaggagta
gctggtgtatttggaggttccttatttagtgcaatgcatggttcattagttacatcttca
cttcttgcagaaagtgcaggagatattagcctaaatgttggatataagtttggtcaagaa
gatgaaacttatagcatatcagccgctcatggttattttggtagactcatatttcaatat
gcttccttcaataactctcgtagtcttcacttcttcctagcagcttggccagttattggt
atttggttcacagcacttggagtaagtacaatggctttcaacttaaatggtttaaacttc
aaccaatccatcctagattccagtggccatctaatcttaagttgggcagatattgtgaat
cgtgctgatctaggtatggaagtgatgcatgaaagaaatgctcacaatttcccattagat
ctagcataa

>Od10_psbA
atgaagaacacatcttactatcaactcaacttacttggtaatgtcataggattcgtacta
tccacaacaaatcgtctctacattgggtgctttggtatcctaatgttccctcttttaact
ttagcaactattgcttatatcacagcttttattcttgcacctgcagtagatattgatgga
ataagagaaccagtagctggctcacttctttatggcaataacattataacaggagctgta
ataccaagttctaatgctattggggttcatttctatccagtttgggagtcaaatggattt
gatgagtgcttatataatggtggtacatatcaatttgtagtacttcattttatgcttggt
gttgcttgttggatgggtagagaatgggaattcagtttcagattaggtatgagaccatgg
atatttgtagctttctcagcacctgttgtagcagcttttgcagtatttgttgtttatcca
attggtcaagctagcttttccgatggaatgcctttaggaataagtggaacttttaatttt
atgcttgtgttccaagcagaacataacattttaatgcatccattccatatcctaggagta
gctggtgtatttggaggttccttatttagtgcaatgcatggttcattagttacatcttca
cttcttgcagaaagtgcaggagatattagcctaaatgttggatataagtttggtcaagaa
gatgaaacttatagcatatcagcagcccatggttattttggtagactcatatttcaatat
gcttccttcaataactctcgtagtcttcacttcttcctagcagcttggccagttattggt
atttggttcacagcacttggagtaagtacaatggctttcaacttaaatggtttaaacttc
aaccaatccatcctagattccagtggccatctaatcttaagttgggcagatattgtgaat
cgtgctgatctaggtatggaagtgatgcatgaaagaaatgctcacaatttcccattagat
ctagcataa
>Od11_psbA
atgaagaacacatcttactatcaactcaacttacttggtaatgtcataggattcgtacta
tccacaacaaatcgtctctacattgggtgctttggtatcctaatgttccctcttttaact
ttagcaactattgcttatatcacagcttttattcttgcacctgcagtagatattgatgga
ataagagaaccagtagctggctcacttctttatggcaataacattataacaggagctgta
ataccaagttctaatgctattggggttcatttctatccagtttgggagtcaaatggattt
gatgagtgcttatataatggtggtacatatcaatttgtagtacttcattttatgcttggt
gttgcttgttggatgggtagagaatgggaattcagtttcagattaggtatgagaccatgg
atatttgtagctttctcagcacctgttgtagcagcttttgcagtatttgttgtttatcca
attggtcaagctagcttttccgatggaatgcctttaggaataagtggaacttttaatttt
atgcttgtgttccaagcagaacataacattttaatgcatccattccatatcctaggagta
gctggtgtatttggaggttccttatttagtgcaatgcatggttcattagttacatcttca
cttcttgcagaaagtgcaggagatattagcctaaatgttggatataagtttggtcaagaa
gatgaaacttatagcatatcagcagcccatggttattttggtagactcatatttcaatat
gcttccttcaataactctcgtagtcttcacttcttcctagcagcttggccagttattggt
atttggttcacagcacttggagtaagtacaatggctttcaacttaaatggtttaaacttc
aaccaatccatcctagattccagtggccatctaatcttaagttgggcagatattgtgaat
cgtgctgatctaggtatggaagtgatgcatgaaagaaatgctcacaatttcccattagat
ctagcataa
>Od12_psbA
atgaagaacacatcttactatcaactcaacttacttggtaatgtcataggattcgtacta
tccacaacaaatcgtctctacattgggtgctttggtatcctaatgttccctcttttaact
ttagcaactattgcttatatcacagcttttattcttgcacctgcagtagatattgatgga
ataagagaaccagtagctggctcacttctttatggcaataacattataacaggagctgta
ataccaagttctaatgctattggggttcatttctatccggtttgggagtcaaatggattt
gatgagtgcttatataatggtggtacatatcaatttgtagtacttcattttatgcttggt
gttgcttgttggatgggtagagaatgggaattcagtttcagattaggtatgagaccatgg
atatttgtagctttctcagcacctgttattgcagcttttgcagtatttgttgtttatcca
attggtcaagctagcttttccgatggaatgcctttaggaataagtggaacttttaatttt
atgcttgtgttccaagcagaacataacattttaatgcatccattccatatcctaggagta
gctggtgtatttggaggttccttatttagtgcaatgcatggttcattagttacatcttca
cttcttgcagaaagtgcaggagatattagcctaaatgttggatataagtttggtcaagaa
gatgaaacttatagcatatcagcagcccatggttattttggtagactcatatttcaatat
gcttccttcaataactctcgtagtcttcacttcttcctagcagcttggccagttattggt
atttggttcacagcacttggagtaagtacaatggctttcaacttaaatggtttaaacttc
aaccaatccatcctagattccagtggccatctaatcttaagttgggcagatattgtgaat
cgtgctgatctaggtatggaagtgatgcatgaaagaaatgctcacaatttcccattagat
ctagcataa

>Od13_psbA
atgaagaacacatcttactatcaactcaacttacttggtaatgtcataggattcgtacta
tccacaacaaatcgtctctacattgggtgctttggtatcctaatgttccctcttttaact
ttagcaactattgcttatatcacagcttttattcttgcacctgcagtagatattgatgga
ataagagaaccagtagctggctcacttctttatggcaacaacattataacaggagctgta
ataccaagttctaatgctattggggttcatttctatccggtttgggagtcaaatggattt
gatgagtgcttatataatggtggtacatatcaatttgtagtacttcattttatgcttggt
gttgcttgttggatgggtagagaatgggaattcagtttcagattaggtatgagaccatgg
atatttgtagctttctcagcacctgttgtagcagcttttgcagtatttgttgtttatcca
attggtcaagctagcttttccgatggaatgcctttaggaataagtggaacttttaatttt
atgcttgtgttccaagcagaacataacattttaatgcatccattccatatcctaggagta
gctggtgtatttggaggttccttatttagtgcaatgcatggttcattagttacatcttca
cttcttgcagaaagtgcaggagatattagcctaaatgttggatataagtttggtcaagaa
gatgaaacttatagcatatcagcagcccatggttattttggtagactcatatttcaatat
gcttccttcaataactctcgtagtcttcacttcttcctagcagcttggccagttattggt
atttggttcacagcacttggagtaagtacaatggctttcaacttaaatggtttaaacttc
aaccaatccatcctagattccagtggccatctaatcttaagttgggcagatattgtgaat
cgtgctgatctaggtatggaagtgatgcatgaaagaaatgctcacaatttcccattagat
ctagcataa
>Od14_psbA
atgaagaacacatcttactatcaactcaacttacttggtaatgtcataggattcgtacta
tccacaacaaatcgtctctacattgggtgctttggtatcctaatgttccctcttttaact
ttagcaactattgcttatatcacagcttttattcttgcacctgcagtagatattgatgga
ataagagaaccagtagctggctcacttctttatggcaataacattataacaggagctgta
ataccaagttctaatgctattggggttcatttctatccggtttgggagtcaaatggattt
gatgagtgcttatataatggtggaacatatcaatttgtagtacttcattttatgcttggt
gttgcttgttggatgggtagagaatgggaattcagtttcagattaggtatgagaccatgg
atatttgtagctttctcagcacctgttgtagcagcttttgcagtatttgttgtttatcca
attggtcaagctagcttttccgatggaatgcctttaggaataagtggaacttttaatttt
atgcttgtgttccaagcagaacataacattttaatgcatccattccatatcctaggagta
gctggtgtatttggaggttccttatttagtgcaatgcatggttcattagttacatcttca
cttcttgcagaaagtgcaggagatattagcctaaatgttggatataagtttggtcaagaa
gatgaaacttatagcatatcagcagcccatggttattttggtagactcatatttcaatat
gcttccttcaataactctcgtagtcttcacttcttcctagcagcttggccagttattggt
atttggttcacagcacttggagtaagtacaatggctttcaacttaaatggtttaaacttc
aaccaatccatcctagattccagtggccatctaatcttaagttgggcagatattgtgaat
cgtgctgatctaggtatggaagtgatgcatgaaagaaatgctcacaatttcccattagat
ctagcataa
>Od15_psbA
atgaagaacacatcttactatcaactcaacttacttggtaatgtcataggattcgtacta
tccacaacaaatcgtctctacattgggtgctttggtatcctaatgttccctcttttaact
ttagcaactattgcttatatcacagcttttattcttgcacctgcagtagatattgatgga
ataagagaaccagtagctggctcacttctttatggcaataacattataacaggagctgta
ataccaagttctaatgctattggggttcatttctatccagtttgggagtcaaatggattt
gatgagtgcttatataatggtggtacatatcaatttgtagtacttcattttatgcttggt
gttgcttgttggatgggtagagaatgggaattcagtttcagattaggtatgagaccatgg
atatttgtagctttctcagcacctgttgtagcagcttttgcagtatttgttgtttatcca
attggtcaagctagcttttccgatggaatgcctttaggaataagtggaacttttaatttt
atgcttgtgttccaagcagaacataacattttaatgcatccattccatatcctaggagta
gctggtgtatttggaggttccttatttagtgcaatgcatggttcattagttacatcttca
cttcttgcagaaagtgcaggagatattagcctaaatgttggatataagtttggtcaagaa
gatgaaacttatagcatatcagcagcccatggttattttggtagactcatatttcaatat
gcttccttcaataactctcgtagtcttcacttcttcctagcagcttggccagttattggt
atttggttcacagcacttggagtaagtacaatggctttcaacttaaatggtttaaacttc
aaccaatccatcctagattccagtggccatctaatcttaagttgggcagatattgtgaat
cgtgctgatctaggtatggaagtgatgcatgaaagaaatgctcacaatttcccattagat
ctagcataa

>Od18_psbA
atgaagaacacatcttactatcaactcaacttacttggtaatgtcataggattcgtacta
tccacaacaaatcgtctctacattgggtgctttggtatcctaatgttccctcttttaact
ttagcaactattgcttatatcacagcttttattcttgcacctgcagtagatattgatgga
ataagagaaccagtagctggctcacttctttatggcaataacattataacaggagctgta
ataccaagttctaatgctattggggttcatttctatccggtttgggagtcaaatggattt
gatgagtgcttatataatggtggtacatatcaatttgtagtacttcattttatgcttggt
gttgcttgttggatgggtagagaatgggaattcagtttcagattaggtatgagaccatgg
atatttgtagctttctcagcacctgttgtagcagcttttgcagtatttgttgtttatcca
attggtcaagctagcttttccgatggaatgcctttaggaataagtggaacttttaatttt
atgcttgtgttccaagcagaacataacattttaatgcatccattccatatcctaggagta
gctggtgtatttggaggttccttatttagtgcaatgcatggttcattagttacatcttca
cttcttgcagaaagtgcaggagatattagcctaaatgttggatataagtttggtcaagaa
gatgaaacttatagcatatcagcagcccatggttattttggtagactcatatttcaatat
gcttccttcaataactctcgtagtcttcacttcttcctagcagcttggccagttattggt
atttggttcacagcacttggagtaagtacaatggctttcaacttaaatggtttaaacttc
aaccaatccatcctagattccagtggccatctaatcttaagttgggcagatattgtgaat
cgtgctgatctaggtatggaagtgatgcgtgaaagaaatgctcataatttcccattagat
ctagcataa
>Od19_psbA
atgaagaacacatcttactatcaactcaacttacttggtaatgtcataggattcgtacta
tccacaacaaatcgtctctacattgggtgctttggtatcctaatgttccctcttttaact
ttagcaactattgcttatatcacagcttttattcttgcacctgcagtagatattgatgga
ataagagaaccagtagctggctcacttctttatggcaataacattataacaggagctgta
ataccaagttctaatgctattggggttcatttctatccagtttgggagtcaaatggattt
gatgagtgcttatataatggtggtacatatcaatttgtagtacttcattttatgcttggt
gttgcttgttggatgggtagagaatgggaattcagtttcagattaggtatgagaccatgg
atatttgtagctttctcagcacctgttgtagcagcttttgcagtatttgttgtttatcca
attggtcaagctagcttttccgatggaatgcctttaggaataagtggaacttttaatttt
atgcttgtgttccaagcagaacataacattttaatgcatccattccatatcctaggagta
gctggtgtatttggaggttccttatttagtgcaatgcatggttcattagttacatcttca
cttcttgcagaaagtgcaggagatattagcctaaatgttggatataagtttggtcaagaa
gatgaaacttatagcatatcagcagcccatggttattttggtagactcatatttcaatat
gcttccttcaataactctcgtagtcttcacttcttcctagcagcttggccagttattggt
atttggttcacagcacttggagtaagtacaatggctttcaacttaaatggtttaaacttc
aaccaatccatcctagattccagtggccatctaatcttaagttgggcagatattgtgaat
cgtgctgatctaggtatggaagtgatgcatgaaagaaatgctcacaatttcccattagat
ctagcataa
>Od2_psbA
atgaagaacacatcttactatcaactcaacttacttggtaatgtcataggattcgtacta
tccacaacaaatcgtctctacattgggtgctttggtatcctaatgttccctcttttaact
ttagcaactattgcttatatcacagcttttattcttgcacctgcagtagatattgatgga
ataagagaaccagtagctggctcacttctttatggcaataacattataacaggagctgta
ataccaagttctaatgctattggggttcatttctatccggtttgggagtcaaatggattt
gatgagtgcttatataatggtggtacatatcaatttgtagtacttcattttatgcttggt
gttgcttgttggatgggtagagaatgggaattcagtttcagattaggtatgagaccatgg
atatttgtagctttctcagcacctgttgtagcagcttttgcagtatttgttgtttatcca
attggtcaagctagcttttccgatggaatgcctttaggaataagtggaacttttaatttt
atgcttgtgttccaagcagaacataacattttaatgcatccattccatatcctaggagta
gctggtgtatttggaggttccttatttagtgcaatgcatggttcattagttacatcttca
cttcttgcagaaagtgcaggagatattagcctaaatgttggatataagtttggtcaagaa
gatgaaacttatagcatatcagcagcccatggttattttggtagactcatatttcaatat
gcttccttcaataactctcgtagtcttcacttcttcctagcagcttggccagttattggt
atttggttcacagcacttggagtaagtacaatggctttcaacttaaatggtttaaacttc
aaccaatccatcctagattccagtggccatctaatcttaagttgggcagatattgtgaat
cgtgctgatctaggtatggaagtgatgcatgaaagaaatgctcacaatttcccattagat
ctagcataa

>Od22_psbA
atgaagaacacatcttactatcaactcaacttacttggtaatgtcataggattcgtacta
tccacaacaaatcgtctctacattgggtgctttggtatcctaatgttccctcttttaact
ttagcaactattgcttatatcacagcttttattcttgcacctgcagtagatattgatgga
ataagagaaccagtagctggctcacttctttatggcaataacattataacaggagctgta
ataccaagttctaatgctattggggttcatttctatccagtttgggagtcaaatggattt
gatgagtgcttatataatggtggtacatatcaatttgtagtacttcattttatgcttggt
gttgcttgttggatgtgtagagaatgggaattcagtttcagattaggtatgagaccatgg
atatttgtagctttctcagcacctgttgtagcagcttttgcagtatttgttgtttacccg
atcggtcaagctagcttttctgatggaatgcctttaggaataagtggaacttttaatttt
atgcttgtgttccaagcagaacataacattttaatgcatccattccatatcctaggagta
gctggtgtatttggaggttccttatttagtgcaatgcatggttcattagttacatcttca
cttcttgcagaaagtgcaggagatattagcctaaatgttggatataagtttggtcaagaa
gatgaaacttatagcatatcagcagcccatggttattttggtagactcatatttcaatat
gcttccttcaataactctcgtagtcttcacttcttcctagcagcttggccagttattggt
atttggttcacagcacttggagtaagtacaatggctttcaacttaaatggtttaaacttc
aaccaatccatcctagattccagtggccatctaatcttaagttgggcagatattgtgaat
cgtgctgatctaggtatggaagtgatgcatgaaagaaatgctcacaatttcccattagat
ctagcataa
>Od23_psbA
atgaagaacacatcttactatcaactcaacttacttggtaatgtcataggattcgtacta
tccacaacaaatcgtctctacattgggtgctttggtatcctaatgttccctcttttaact
ttagcaactattgcttatatcacagcttttattcttgcacctgcagtagatattgatgga
ataagagaaccagtagctggctcacttctttatggcaataacattataacaggagctgta
ataccaagttctaatgctattggggttcatttctatccagtttgggagtcaaatggattt
gatgagtgcttatataatggtggtacatatcaatttgtagtacttcattttatgcttggt
gttgcttgttggatgggtagagaatgggaattcagtttcagattaggtatgagaccatgg
atatttgtagctttctcagcacctgttgtagcagcttttgcagtatttgttgtttatcca
attggtcaagctagcttttccgatggaatgcctttaggaataagtggaacttttaatttt
atgcttgtgttccaagcagaacataacattttaatgcatccattccatatcctaggagta
gctggtgtatttggaggttccttatttagtgcaatgcatggttcattagttacatcttca
cttcttgcagaaagtgcaggagatattagcctaaatgttggatataagtttggtcaagaa
gatgaaacttatagcatatcagcagcccatggttattttggtagactcatatttcaatat
gcttccttcaataactctcgtagtcttcacttcttcctagcagcttggccagttattggt
atttggttcacagcacttggagtaagtacaatggctttcaacttaaatggtttaaacttc
aaccaatccatcctagattccagtggccatctaatcttaagttgggcagatattgtgaat
cgtgctgatctaggtatggaagtgatgcatgaaagaaatgctcacaatttcccattagat
ctagcataa
>Od24_psbA
atgaagaatagatcttactatcaactcaacttacttggtaatgtcataggattcgtacta
tccacaacaaatcgtctctacattgggtgctttggtatcctaatgttccctcttttaact
ttagcaactattgcttatatcacagcttttattcttgcacctgcagtagatattgatgga
ataagagaaccagtagctggctcacttctttatggcaataacattataacaggagctgta
ataccaagttctaatgctattggtgttcatttctatccagtctgggaatcaaaccatttc
gatgagtgcttatataatggtggaacatatcaattcgtagtccttcattttatgcttggt
gttgcttgttggatgggtagagaatgggaattcagtttcagattaggtatgagaccatgg
atatttgtagctttctcagcacctgttgtagcagcttttgcagtatttgttgtttacccg
atcggtcaagctagcttttctgatggaatgcctttaggaataagtggtacttttaatttt
atgcttgttttccaagcagaacataacattttaatgcatccattccatatcctaggagta
gctggtgtatttggaggttccttattcagtgcaatgcatggttcattagttacatcttca
cttcttgcagaaagtgcaggagatattagcctaaatgttggatataagtttggtcaagaa
gatgaaacttatagcatatcagcagcccatggttattttggtagactcatatttcaatat
gcttccttcaataactctcgtagccttcacttctttctagcagcttggccagttattggt
atttggttcactgcccttggagtaagtacaatggctttcaacttaaatggtttaaacttc
aaccaatccatcctagattccagtggccatctaatcttaagttgggcagatattgtgaat
cgtgctgatctaggtatggaagtgatgcatgaaagaaatgctcacaatttcccattagat
ctagcataa

>Od25_psbA
atgaagaacacatcttactatcaactcaacttacttggtaatgtcataggattcgtacta
tccacaacaaatcgtctctacattgggtgctttggtatcctaatgttccctcttttaact
ttagcaactattgcttatatcacagcttttattcttgcacctgcagtagatattgatgga
ataagagaaccagtagctggctcacttctttatggcaataacattataacaggagctgta
ataccaagttctaatgctattggggttcatttctatccagtttgggagtcaaatggattt
gatgagtgcttatataatggtggtacatatcaatttgtagtacttcattttatgcttggt
gttgcttgttggatgggtagagaatgggaattcagtttcagattaggtatgagaccatgg
atatttgtagctttctcagcacctgttgtagcagcttttgcagtatttgttgtttatcca
attggtcaagctagcttttccgatggaatgcctttaggaataagtggaacttttaatttt
atgcttgtgttccaagcagaacataacattttaatgcatccattccatatcctaggagta
gctggtgtatttggaggttccttatttagtgcaatgcatggttcattagttacatcttca
cttcttgcagaaagtgcaggagatattagcctaaatgttggatataagtttggtcaagaa
gatgaaacttatagcatatcagcagcccatggttattttggtagactcatatttcaatat
gcttccttcaataactctcgtagtcttcacttcttcctagcagcttggccagttattggt
atttggttcacagcacttggagtaagtacaatggctttcaacttaaatggtttaaacttc
aaccaatccatcctagattccagtggccatctaatcttaagttgggcagatattgtgaat
cgtgctgatctaggtatggaagtgatgcatgaaagaaatgctcacaatttcccattagat
ctagcataa
>Od26_psbA
atgaagaacacatcttactatcaactcaacttacttggtaatgtcataggattcgtacta
tccacaacaaatcgtctctacattgggtgctttggtatcctaatgttccctcttttaact
ttagcaactattgcttatatcacagcttttattcttgcacctgcagtagatattgatgga
ataagagaaccagtagctggctcacttctttatggcaataacattataacaggagctgta
ataccaagttctaatgctattggggttcatttctatccagtttgggagtcaaatggattt
gatgagtgcttatataatggtggtacatatcaatttgtagtacttcattttatgcttggt
gttgcttgttggatgggtagagaatgggaattcagtttcagattaggtatgagaccatgg
atatttgtagctttctcagcacctgttgtagcagcttttgcagtatttgttgtttatcca
attggtcaagctagcttttccgatggaatgcctttaggaataagtggtacttttaatttt
atgcttgttttccaagcagaacataacattttaatgcatccattccatatcctaggagta
gctggtgtatttggaggttccttatttagtgcaatgcatggttcattagttacatcttca
cttcttgcagaaagtgcaggagatattagcctaaatgttggatataagtttggtcaagaa
gatgaaacttatagcatatcagcagcccatggttattttggtagactcatatttcaatat
gcttccttcaataactctcgtagtcttcacttcttcctagcagcttggccagttattggt
atttggttcacagcacttggagtaagtacaatggctttcaacttaaatggtttaaacttc
aaccaatccatcctagattccagtggccatctaatcttaagttgggcagatattgtgaat
cgtgctgatctaggtatggaagtgatgcatgaaagaaatgctcacaatttcccattagat
ctagcataa
>Od27_psbA
atgaagaacacatcttactatcaactcaacttacttggtaatgtcataggattcgtacta
tccacaacaaatcgtctctacattgggtgctttggtatcctaatgttccctcttttaact
ttagcaactattgcttatatcacagcttttattcttgcacctgcagtagatattgatgga
ataagagaaccagtagctggctcacttctttatggcaataacattataacaggagctgta
ataccaagttctaatgctattggggttcatttctatccggtttgggagtcaaatggattt
gatgagtgcttatataatggtggtacatatcaatttgtagtacttcattttatgcttggt
gttgcttgttggatgggtagagaatgggaattcagtttcagattaggtatgagaccatgg
atatttgtagctttctcagcacctgttgtagcagcttttgcagtatttgttgtttatcca
attggtcaagctagcttttccgatggaatgcctttaggaataagtggaacttttaatttt
atgcttgtgttccaagcagaacataacattttaatgcatccattccatatcctaggagta
gctggtgtatttggaggttccttatttagtgcaatgcatggttcattagttacatcttca
cttcttgcagaaagtgcaggagatattagcctaaatgttggatataagtttggtcaagaa
gatgaaacttatagcatatcagcagcccatggttattttggtagactcatatttcaatat
gcttccttcaataactctcgtagtcttcacttcttcctagcagcttggccagttattggt
atttggttcacagcacttggagtaagtacaatggctttcaacttaaatggtttaaacttc
aaccaatccatcctagattccagtggccatctaatcttaagttgggcagatattgtgaat
cgtgctgatctaggtatggaagtgatgcatgaaagaaatgctcacaatttcccattagat
ctagcataa

>Od4_psbA
atgaagaacacatcttactatcaactcaacttacttggtaatgtcataggattcgtacta
tccacaacaaatcgtctctacattgggtgctttggtatcctaatgttccctcttttaact
ttagcaactattgcttatatcacagcttttattcttgcacctgcagtagatattgatgga
ataagagaaccagtagctggctcacttctttatggcaataacattataacaggagctgta
ataccaagttctaatgctattggggttcatttctatccagtttgggagtcaaatggattt
gatgagtgcttatataatggtggtacatatcaatttgtagtacttcattttatgcttggt
gttgcttgttggatgggtagagaatgggaattcagtttcagattaggtatgagaccatgg
atatttgtagctttctcagcacctgttgtagcagcttttgcagtatttgttgtttatcca
attggtcaagctagcttttccgatggaatgcctttaggaataagtggaacttttaatttt
atgcttgtgttccaagcagaacataacattttaatgcatccattccatatcctaggagta
gctggtgtatttggaggttccttatttagtgcaatgcatggttcattagttacatcttca
cttcttgcagaaagtgcaggagatattagcctaaatgttggatataagtttggtcaagaa
gatgaaacttatagcatatcagcagcccatggttattttggtagactcatatttcaatat
gcttccttcaataactctcgtagtcttcacttcttcctagcagcttggccagttattggt
atttggttcacagcacttggagtaagtacaatggctttcaacttaaatggtttaaacttc
aaccaatccatcctagattccagtggccatctaatcttaagttgggcagatattgtgaat
cgtgctgatctaggtatggaagtgatgcatgaaagaaatgctcacaatttcccattagat
ctagcataa
>Od6_psbA
atgaagaacacatcttactatcaactcaacttacttggtaatgtcataggattcgtacta
tccacaacaaatcgtctctacattgggtgctttggtatcctaatgttccctcttttaact
ttagcaactattgcttatatcacagcttttattcttgcacctgcagtagatattgatgga
ataagagaaccagtagctggctcacttctttatggcaataacattataacaggagctgta
ataccaagttctaatgctattggggttcatttctatccagtttgggagtcaaatggattt
gatgagtgcttatataatggtggtacatatcaattcgtagtccttcattttatgcttggt
gttgcttgttggatgggtagagaatgggaattcagtttcagattaggtatgagaccatgg
atatttgtagctttctcagcacctgttgtagcagcttttgcagtatttgttgtttatcca
attggtcaagctagcttttccgatggaatgcctttaggaataagtggaacttttaatttt
atgcttgtgttccaagcagaacataacattttaatgcatccattccatatcctaggagta
gctggtgtatttggaggttccttatttagtgcaatgcatggttcattagttacatcttca
cttcttgcagaaagtgcaggagatattagcctaaatgttggatataagtttggtcaagaa
gatgaaacttatagcatatcagcagcccatggttattttggtagactcatatttcaatat
gcttccttcaataactctcgtagtcttcacttcttcctagcagcttggccagttattggt
atttggttcacagcacttggagtaagtacaatggctttcaacttaaatggtttaaacttc
aaccaatccatcctagattccagtggccatctaatcttaagttgggcagatattgtgaat
cgtgctgatctaggtatggaagtgatgcatgaaagaaatgctcacaatttcccattagat
ctagcataa
>Od8_psbA
atgaagaacacatcttactatcaactcaacttacttggtaatgtcataggattcgtacta
tccacaacaaatcgtctctacattgggtgctttggtatcctaatgttccctcttttaact
ttagcaactattgcttatatcacagcttttattcttgcacctgcagtagatattgatgga
ataagagaaccagtagctggctcacttctttatggcaataacattataacaggagctgta
ataccaagttctaatgctattggggttcatttctatccagtttgggagtcaaatggattt
gatgagtgcttatataatggtggtacatatcaatttgtagtacttcattttatgcttggt
gttgcttgttggatgggtagagaatgggaattcagtttcagattaggtatgagaccatgg
atatttgtagctttctcagcacctgttgtagcagcttttgcagtatttgttgtttatcca
attggtcaagctagcttttccgatggaatgcctttaggaataagtggaacttttaatttt
atgcttgtgttccaagcagaacataacattttaatgcatccattccatatcctaggagta
gctggtgtatttggaggttccttatttagtgcaatgcatggttcattagttacatcttca
cttcttgcagaaagtgcaggagatattagcctaaatgttggatataagtttggtcaagaa
gatgaaacttatagcatatcagcagcccatggttattttggtagactcatatttcaatat
gcttccttcaataactctcgtagtcttcacttcttcctagcagcttggccagttattggt
atttggttcacagcacttggagtaagtacaatggctttcaacttaaatggtttaaacttc
aaccaatccatcctagattccagtggccatctaatcttaagttgggcagatattgtgaat
cgtgctgatctaggtatggaagtgatgcatgaaagaaatgctcacaatttcccattagat
ctagcataa

>Od9_psbA
atgaagaacacatcttactatcaactcaacttacttggtaatgtcataggattcgtacta
tccacaacaaatcgtctctacattgggtgctttggtatcctaatgttccctcttttaact
ttagcaactattgcttatatcacagcttttattcttgcacctgcagtagatattgatgga
ataagagaaccagtagctggctcacttctttatggcaataacattataacaggagctgta
ataccaagttctaatgctattggggttcatttctatccagtttgggagtcaaatggattt
gatgagtgcttatataatggtggtacatatcaatttgtagtacttcattttatgcttggt
gttgcttgttggatgggtagagaatgggaattcagtttcagattaggtatgagaccatgg
atatttgtagctttctcagcacctgttgtagcagcttttgcagtatttgttgtttatcca
attggtcaagctagcttttccgatggaatgcctttaggaataagtggaacttttaatttt
atgcttgtgttccaagcagaacataacattttaatgcatccattccatatcctaggagta
gctggtgtatttggaggttccttatttagtgcaatgcatggttcattagttacatcttca
cttcttgcagaaagtgcaggagatattagcctaaatgttggatataagtttggtcaagaa
gatgaaacttatagcatatcagcagcccatggttattttggtagactcatatttcaatat
gcttccttcaataactctcgtagtcttcacttcttcctagcagcttggccagttattggt
atttggttcacagcacttggagtaagtacaatggctttcaacttaaatggtttaaacttc
aaccaatccatcctagattccagtggccatctaatcttaagttgggcagatattgtgaat
cgtgctgatctaggtatggaagtgatgcatgaaagaaatgctcacaatttcccattagat
ctagcataa
>Ss5_psbA
atgaagaacacatcttactatcaactcaacttacttggtaatgtcataggattcgtacta
tccacaacaaatcgtctctacattgggtgctttggtatcctaatgttccctcttttaact
ttagcaactattgcttatatcacagcttttattcttgcacctgcagtagatattgatgga
ataagagaaccagtagctggctcacttctttatggcaataacattataacaggagctgta
ataccaagttctaatgctattggggttcatttctatccagtttgggagtcaaatggattt
gatgagtgcttatataatggtggtacatatcaatttgtagtacttcattttatgcttggt
gttgcttgttggatgggtagagaatgggaattcagtttcagattaggtatgagaccatgg
atatttgtagctttctcagcacctgttgtagcagcttttgcagtatttgttgtttatcca
attggtcaagctagcttttccgatggaatgcctttaggaataagtggaacttttaatttt
atgcttgtgttccaagcagaacataacattttaatgcatccattccatatcctaggagta
gctggtgtatttggaggttccttatttagtgcaatgcatggttcattagttacatcttca
cttcttgcagaaagtgcaggagatattagcctaaatgttggatataagtttggtcaagaa
gatgaaacttatagcatatcagcagcccatggttattttggtagactcatatttcaatat
gcttccttcaataactctcgtagtcttcacttcttcctagcagcttggccagttattggt
atttggttcacagcacttggagtaagtacaatggctttcaacttaaatggtttaaacttc
aaccaatccatcctagattccagtggccatctaatcttaagttgggcagatattgtgaat
cgtgctgatctaggtatggaagtgatgcatgaaagaaatgctcacaatttcccattagat
ctagcataa
>Ss6_psbA
atgaagaatagatcttactatcaactcaacttacttggtaatgtcataggatttgtgcta
tccacaacaaatcgtctctacattgggtgctttggtatcctaatgttccctcttttaact
ttagcaactattgcttatatcacagcttttattcttgcacctgcagtagatattgatgga
ataagagaaccagtagctggctcacttctttatggcaataacattataacaggagctgta
ataccaagttctaatgctattggggttcatttctatccagtttgggagtcaaatggattt
gatgagtgcttatataatggtggtacatatcaatttgtagtacttcattttatgcttggt
gttgcttgttggatgggtagagaatgggaattcagtttcagattaggtatgagaccatgg
atatttgtagctttctcagcacctgttgtagcagcttttgcagtatttgttgtttacccg
atcggtcaagctagcttttctgatggaatgcctttaggaataagtggaacttttaatttt
atgcttgtgttccaagcagaacataacattttaatgcatccattccatatcctaggagta
gctggtgtatttggaggttccttatttagtgcaatgcatggttcattagttacatcttca
cttcttgcagaaagtgcaggagatattagcctaaatgttggatataagtttggtcaagaa
gatgaaacttatagcatatcagcagcccatggttattttggtagactcatatttcaatat
gcttccttcaataactctcgtagtcttcacttcttcctagcagcttggccagttattggt
atttggttcacagcacttggagtaagtacaatggctttcaacttaaatggtttaaacttc
aaccaatccatcctagattccagtggccatctaatcttaagttgggcagatattgtgaat
cgtgctgatctaggtatggaagtgatgcgtgaaagaaatgctcacaatttcccattagat
ctagcataa

>Ss7_psbA
atgaagaacacatcttactatcaactcaacttacttggtaatgtcataggattcgtacta
tccacaacaaatcgtctctacattgggtgctttggtatcctaatgttccctcttttaact
ttagcaactattgcttatatcacagcttttattcttgcacctgcagtagatattgatgga
ataagagaaccagtagctggctcacttctttatggcaataacattataacaggagctgta
ataccaagttctaatgctattggggttcatttctatccagtttgggagtcaaatggattt
gatgagtgcttatataatggtggtacatatcaatttgtagtacttcattttatgcttggt
gttgcttgttggatgggtagagaatgggaattcagtttcagattaggtatgagaccatgg
atatttgtagctttctcagcacctgttgtagcagcttttgcagtatttgttgtttatcca
attggtcaagctagcttttccgatggaatgcctttaggaataagtggaacttttaatttt
atgcttgtgttccaagcagaacataacattttaatgcatccattccatatcctaggagta
gctggtgtatttggaggttccttatttagtgcaatgcatggttcattagttacatcttca
cttcttgcagaaagtgcaggagatattagcctaaatgttggatataagtttggtcaagaa
gatgaaacttatagcatatcagcagcccatggttattttggtagactcatatttcaatat
gcttccttcaataactctcgtagtcttcacttcttcctagcagcttggccagttattggt
atttggttcacagcacttggagtaagtacaatggctttcaacttaaatggtttaaacttc
aaccaatccatcctagattccagtggccatctaatcttaagttgggcagatattgtgaat
cgtgctgatctaggtatggaagtgatgcatgaaagaaatgctcacaatttcccattagat
ctagcataa
>Ss8_psbA
atgaagaacacatcttactatcaactcaacttacttggtaatgtcataggattcgtacta
tccacaacaaatcgtctctacattgggtgctttggtatcctaatgttccctcttttaact
ttagcaactattgcttatatcaccgcttttattcttgcacctgcagtagatattgatgga
ataagagaaccagtagctggctcacttctttatggcaataacattataacaggagctgta
ataccaagttctaatgctattggggttcatttctatccagtttgggagtcaaatggattt
gatgagtgcttatataatggtggtacatatcaatttgtagtacttcattttatgcttggt
gttgcttgttggatgggtagagaatgggaattcagtttcagattaggtatgagaccatgg
atatttgtagctttctcagcacctgttgtagcagcttttgcagtatttgttgtttatcca
attggtcaagctagcttttccgatggaatgcctttaggaataagtggaacttttaatttt
atgcttgtgttccaagcagaacataacattttaatgcatccattccatatcctaggagta
gctggtgtatttggaggatccttatttagtgcaatgcatggttcattagttacatcttca
cttcttgcagaaagtgcaggagatattagcctaaatgttggatataagtttggtcaagaa
gatgaaacttatagcatatcagcagcccatggttattttggtagactcatatttcaatat
gcttccttcaataactctcgtagtcttcacttcttcctagcagcttggccagttattggt
atttggttcacagcccttggagtaagtacaatggctttcaacttaaatggtttaaacttc
aaccaatccatcctagattccagtggccatctaatcttaagttgggcagatattgtgaat
cgtgctgatctaggtatggaagtgatgcatgaaagaaatgctcataatttcccattagat
ctagcataa
>ohdo1_psbA
atgaagaacacatcttactatcaactcaacttacttggtaatgtcataggattcgtgcta
tccacaacaaatcgtctctacattgggtgctttggtatcctaatgttccctcttttaact
ttagcaactattgcttatatcacagcttttattcttgcacctgcagtagatattgatgga
ataagagaaccagtagctggctcacttctttatggcaataacattataacaggagctgta
ataccaagttctaatgctattggggttcatttctatccagtttgggagtcaaatggattt
gatgagtgcttatataatggtggtacatatcaatttgtagtacttcattttatgcttggt
gttgcttgttggatgggtagagaatgggaattcagtttcagattaggtatgagaccatgg
atatttgtagctttctcagcacctgttgtagcagcttttgcagtatttgttgtttatcca
attggtcaagctagcttttccgatggaatgcctttaggaataagtggaacttttaatttt
atgcttgtgttccaagcagaacataacattttaatgcatccattccatatcctaggagta
gctggtgtatttggaggttcattatttagtgcaatgcatggttcattagttacatcttca
cttcttgcagaaagtgcaggagatattagcctaaatgttggatataagtttggtcaagaa
gatgaaacttatagcatatcagcagcccatggttattttggtagactcatatttcaatat
gcttccttcaataactctcgtagtcttcacttcttcctagcagcttggccagttattggt
atttggttcacagcacttggagtaagtacaatggctttcaacttaaatggtttaaacttc
aaccaatccatcctagattccagtggccatctaatcttaagttgggcagatattgtgaat
cgtgctgatctaggtatggaagtgatgcatgaaagaaatgctcacaatttcccattagat
ctagcataa

>ohdo3_psbA
atgaagaacacatcttactatcaactcaacttacttggtaatgtcataggattcgtacta
tccacaacaaatcgtctctatattggatgctttggaatcctcatgttccctcttttaact
ttagcaactattgcttatatcacagcttttattcttgcacctgcagtagatattgatgga
ataagagaaccagtagctggctcacttctttatggcaataacattataacaggagctgta
ataccaagttctaatgctattggggttcatttctatccagtttgggagtcaaaccatttc
gatgagtgcttatataatggtggtacatatcaatttgtagtacttcattttatgcttggt
gttgcttgttggatgggtagagaatgggaattcagtttcagattaggtatgagaccatgg
atatttgtagctttctcagcacctgttgtagcagcttttgcagtatttgttgtttatcca
attggtcaagctagcttttccgatggaatgcctttaggaataagtggaacttttaatttt
atgcttgtgttccaagcagaacataacattttaatgcatccattccatatcctaggagta
gctggtgtatttggaggttccttatttagtgcaatgcatggttcattagttacatcttca
cttcttgcagaaagtgcaggagatattagcctaaatgttggatataagtttggtcaagaa
gatgaaacttatagcatatcagcagcccatggttattttggtagactcatatttcaatat
gcttccttcaataactctcgtagtcttcacttcttcctagcagcttggccagttattggt
atttggttcacagcacttggagtaagtacaatggctttcaacttaaatggtttaaacttc
aaccaatccatcctagattccagtggccatctaatcttaagttgggcagatattgtgaat
cgtgctgatctaggtatggaagtgatgcatgaaagaaatgctcacaatttcccattagat
ctagcataa
>ohdo5_psbA
atgaagaacacatcttactatcaactcaacttacttggtaatgtcataggattcgtacta
tccacaacaaatcgtctctacattgggtgctttggtatcctaatgttccctcttttaact
ttagcaactattgcttatatcacagcttttattcttgcacctgcagtagatattgatgga
ataagagaaccagtagctggctcacttctttatggcaataacattataacaggagctgta
atcccgagttctaatgctattggtgttcatttctatccagtctgggaatcaaaccatttc
gatgagtgcttatataatggtggtacatatcaatttgtagtacttcattttatgcttggt
gttgcttgttggatgggtagagaatgggaattcagtttcagattaggtatgagaccatgg
atatttgtagctttctcagcacctgttgtagcagcttttgcagtatttgttgtttatcca
attggtcaagctagcttttccgatggaatgcctttaggaataagtggaacttttgatttt
atgcttgtgttccaagcagaacataacattttaatgcatccattccatatcctaggagta
gctggtgtatttggaggttccttatttagtgcaatgcatggttcattagttacatcttca
cttcttgcagaaagtgcaggagatattagcctaaatgttggatataagtttggtcaagaa
gatgaaacttatagcatatcagcagcccatggttattttggtagactcatatttcaatat
gcttccttcaataactctcgtagtcttcacttcttcctagcagcttggccagttattggt
atttggttcacagcacttggagtaagtacaatggctttcaacttaaatggtttaaacttc
aaccaatccatcctagattccagtggccatctaatcttaagttgggcagatattgtgaat
cgtgctgatctaggtatggaagtgatgcatgaaagaaatgctcacaatttcccattagat
ctagcataa
>ohdo7_psbA
atgaagaacacatcttactatcaactcaacttacttggtaatgtcataggattcgtacta
tccacaacaaatcgtctctacattgggtgctttggtatcctaatgttccctcttttaact
ttagcaactattgcttatatcacagcttttattcttgcacctgcagtagatattgatgga
ataagagaaccagtagctggctcacttctttatggcaataacattataacaggagctgta
ataccaagttctaatgctattggggttcatttctatccagtttgggagtcaaatggattt
gatgagtgcttatataatggtggtacatatcaatttgtagtacttcattttatgcttggt
gttgcttgttggatgggtagagaatgggaattcagtttcagattaggtatgagaccatgg
atatttgtagctttctcagcacctgttgtagcagcttttgcagtatttgttgtttatcca
attggtcaagctagcttttccgatggaatgcctttaggaataagtggaacttttaatttt
atgcttgtgttccaagcagaacataacattttaatgcatccattccatatcctaggagta
gctggtgtatttggaggttccttatttagtgcaatgcatggttcattagttacatcttca
cttcttgcagaaagtgcaggagatattagcctaaatgttggatataagtttggtcaagaa
gatgaaacttatagcatatcagcagcccatggttattttggtagactcatatttcaatat
gcttccttcaataactctcgtagtcttcacttcttcctagcagcttggccagttattggt
atttggttcacagcacttggagtaagtacaatggctttcaacttaaatggtttaaacttc
aaccaatccatcctagattccagtggccatctaatcttaagttgggcagatattgtgaat
cgtgctgatctaggtatggaagtgatgcatgaaagaaatgctcacaatttcccattagat
ctagcataa

>sesoko1_psbA
atgaagaacacatcttactatcaactcaacttacttggtaatgtcataggattcgtacta
tccacaacaaatcgtctctacattgggtgctttggtatcctaatgttccctcttttaact
ttagcaactattgcttatatcacagcttttattcttgcacctgcagtagatattgatgga
ataagagaaccagtagctggctcacttctttatggcaataacattataacaggagctgta
ataccaagttctaatgctattggggttcatttctatccagtttgggagtcaaatggattt
gatgagtgcttatataatggtggtacatatcaatttgtagtacttcattttatgcttggt
gttgcttgttggatgggtagagaatgggaattcagtttcagattaggtatgagaccatgg
atatttgtagctttctcagcacctgttgtagcagcttttgcagtatttgttgtttatcca
attggtcaagctagcttttccgatggaatgcctttaggaataagtggaacttttaatttt
atgcttgtgttccaagcagaacataacattttaatgcatccattccatatcctaggagta
gctggtgtatttggaggttccttatttagtgcaatgcatggttcattagttacatcttca
cttcttgcagaaagtgcaggagatattagcctaaatgttggatataagtttggtcaagaa
gatgaaacttatagcatatcagcagcccatggttattttggtagactcatatttcaatat
gcttccttcaataactctcgtagtcttcacttcttcctagcagcttggccagttattggt
atttggttcacagcacttggagtaagtacaatggctttcaacttaaatggtttaaacttc
aaccaatccatcctagattccagtggccatctaatcttaagttgggcagatattgtgaat
cgtgctgatctaggtatggaagtgatgcatgaaagaaatgctcacaatttcccattagat
ctagcataa
>sesoko3_psbA
atgaagaacacatcttactatcaactcaacttacttggtaatgtcataggattcgtacta
tccacaacaaatcgtctctacattgggtgctttggtatcctaatgttccctcttttaact
ttagcaactattgcttatatcacagcttttattcttgcacctgcagtagatattgatgga
ataagagaaccagtagctggctcacttctttatggcaataacattataacaggagctgta
ataccaagttctaatgctattggggttcatttctatccagtttgggagtcaaatggattt
gatgagtgcttatataatggtggtacatatcaatttgtagtacttcattttatgcttggt
gttgcttgttggatgggtagagaatgggaattcagtttcagattaggtatgagaccatgg
atatttgtagctttctcagcacctgttgtagcagcttttgcagtatttgttgtttatcca
attggtcaagctagcttttccgatggaatgcctttaggaataagtggaacttttaatttt
atgcttgtgttccaagcagaacataacattttaatgcatccattccatatcctaggagta
gctggtgtatttggaggttccttatttagtgcaatgcatggttcattagttacatcttca
cttcttgcagaaagtgcaggagatattagcctaaatgttggatataagtttggtcaagaa
gatgaaacttatagcatatcagcagcccatggttattttggtagactcatatttcaatat
gcttccttcaataactctcgtagtcttcacttcttcctagcagcttggccagttattggt
atttggttcacagcacttggagtaagtacaatggctttcaacttaaatggtttaaacttc
aaccaatccatcctagattccagtggccatctaatcttaagttgggcagatattgtgaat
cgtgctgatctaggtatggaagtgatgcatgaaagaaatgctcacaatttcccattagat
ctagcataa
>sesoko4_psbA
atgaagaacacatcttactatcaactcaacttacttggtaatgtcataggattcgtacta
tccacaacaaatcgtctctacattgggtgctttggtatcctaatgttccctcttttaact
ttagcaactattgcttatatcacagcttttattcttgcacctgcagtagatattgatgga
ataagagaaccagtagctggctcacttctttatggcaataacattataacaggagctgta
ataccaagttctaatgctattggggttcatttctatccagtttgggagtcaaatggattt
gatgagtgcttatataatggtggtacatatcaatttgtagtacttcattttatgcttggt
gttgcttgttggatgggtagagaatgggaattcagtttcagattaggtatgagaccatgg
atatttgtagctttctcagcacctgttgtagcagcttttgcagtatttgttgtttatcca
attggtcaagctagcttttccgatggaatgcctttaggaataagtggaacttttaatttt
atgcttgtgttccaagcagaacataacattttaatgcatccattccatatcctaggagta
gctggtgtatttggaggttccttatttagtgcaatgcatggttcattagttacatcttca
cttcttgcagaaagtgcaggagatattagcctaaatgttggatataagtttggtcaagaa
gatgaaacttatagcatatcagcagcccatggttattttggtagactcatatttcaatat
gcttccttcaataactctcgtagtcttcacttcttcctagcagcttggccagttattggt
atttggttcacagcacttggagtaagtacaatggctttcaacttaaatggtttaaacttc
aaccaatccatcctagattccagtggccatctaatcttaagttgggcagatattgtgaat
cgtgctgatctaggtatggaagtgatgcatgaaagaaatgctcacaatttcccattagat
ctagcataa

```
>REF_DNA_psbA_JX094320.1_JX094319.1
ATGAAGAATAGATCTTACTATCAACTCAACTTACTTGGTAATGTCATAGGATTTGTGCTA
TCCACAACAAATCGTCTCTATATTGGATGCTTTGGAATCCTCATGTTTCCTCTTTTAACT
TTAGCTACTATTGCTTATATCACCGCTTTCATTCTTGCACCTGCAGTAGATATTGATGGA
ATAAGAGAACCAGTAGCTGGCTCACTTCTTTATGGCAACAACATTATAACAGGAGCTGTA
ATCCCGAGTTCTAATGCTATTGGTGTTCATTTCTATCCAGTCTGGGAATCAAACCATTTC
GATGAGTGCTTATATAATGGTGGAACATATCAATTCGTAGTCCTTCATTTTATGCTTGGT
GTTGCTTGTTGGATGGGTAGAGAATGGGAATTCAGTTTTAGATTAGGTATGAGACCATGG
ATATTTGTAGCTTTCTCAGCACCTGTTATTGCAGCCTTTGCAGTATTTGTTGTTTACCCG
ATCGGTCAAGCTAGCTTTTCTGATGGAATGCCTTTAGGAATAAGTGGTACTTTTAATTTT
ATGCTTGTTTTCCAAGCAGAGCATAACATCTTAATGCATCCATTCCATATTCTAGGAGTA
GCTGGAGTATTTGGTGGATCATTGTTCAGTGCAATGCATGGTTCATTAGTTACATCTTCA
CTTCTTGCAGAAAGTGCAGGAGATATTAGCCTCAATGTTGGATATAAGTTTGGTCAAGAA
GATGAAACTTATAGCATATCAGCCGCTCATGGTTATTTTGGTAGACTCATATTTCAATAT
GCTTCCTTCAATAACTCTCGTAGCCTTCACTTCTTTCTAGCAGCTTGGCCAGTTATTGGT
ATTTGGTTCACTGCCCTTGGAGTAAGTACAATGGCTTTCAACTTAAATGGTTTAAACTTC
AACCAATCCATCCTAGATTCCAGTGGCCATCTAATCTTAAGTTGGGCAGATATTGTGAAT
CGTGCTGATCTAGGTATGGAAGTGATGCATGAAAGAAATGCTCATAATTTCCCATTAGAT
CTAGCATAA
>REF_RNA_psbA_symbB1.comp0_c0_seq1
atgaagaatagatcttactatcaactcaacttacttggtaatgtcatgggatttgtgcta
tccacaacaaatcgtctctatattggatgctttggaatcctcatgtttcctcttttaact
ttagctactattgcttatatcaccgctttcattcttgcacctgcagtagatattgatgga
ataagagaaccagtagctggctcacttctttatggcaacaacattataacaggagctgta
atcccgagttctaatgctattggtgttcatttctatccagtctgggaatcaaaccatttc
gatgagtgcttatataatggtggaacatatcaattcgtagtccttcattttatgcttggt
gttgcttgttggatgggtagagaatgggaattcagttttagattaggtatgagaccatgg
atatttgtagctttctcagcacctgttgttgcagcctttgcagtatttgttgtttacccg
atcggtcaagctagcttttctgatggaatgcctttaggaataagtggtacttttaatttt
atgcttgttttccaagcagagcataacatcttaatgcatccattccatattctaggagta
gctggagtatttggtggatcattgttcagtgcaatgcatggttcattagttacatcttca
cttcttgcagaaactgcaggagatattagcctcaatgttggatataagtttggtcaagaa
gatgaaacttatagcatatcagccgctcatggttattttggtagactcatatttcaatat
gcttccttcaataactctcgtagccttcacttctttctagcagcttggccagttattggt
atttggttcactgcccttggagtaagtacaatggctttcaacttaaatggtttaaacttc
aaccaatccatcctagattccagtggccatctaatcttaagttgggcagatattgtgaat
cgtgctgatctaggtatggaagtgatgcatgaaagaaatgctcataatttcccattagat
ctagcataa
```

>Hd1_psbB
atgcaaataactgatgctttaccttggtttagggtacatatagttattcttaatgatcca
ggacgtctcattagctcacatattatgcacacagctttagtagcaggttggtcagctctc
atgcttttatatgagcttatcaccatagatcctacagatcctgtgtataatccaatttgg
agacaagcagcttacacactcccatttatctcacgtattggtgttattcgctctcttttt
agttggtcacttggcatagatcctacatccaatctaatctggacttatgaaacaatgaac
atagcccatattctattatctggtttattaattcttgcatcattttggcattgggcatat
tgggacttagatctattcttcacctcaacattaacactagaccttaatcaaatcctcagt
attcacctcacactagcatcatctctctgtctgggatttggtttagctcatctaactggt
tttcttggtccaggaatgtggactagtgattccctcaatcttgtaggctctattagattc
gtaaaagcctccttcaatctccttgcttatgcacgattagcttatggtgtcatatcatca
catcacatcatctctggattgcttggcacttccataggattatggcatatcacattacgt
ccattagcatacctctacaatctattaagcatgggaaaggttgagtccattctttcaagt
agtattacagctgtattcttcactgcattccttatttcagcccttatgtggtatggctct
gcacacaccacacaagaactcttcggtcctactagatactcatgggacaatgcttattac
tctcttgatatcagaacctcttgctcaaatgcctggaacactcttccagacaagttagtc
ctttatgattatattggctctaaccctgctaaaggtggcttatttcgttcaggaccaatg
cttaaagctgatggtcttgttcaaaactggttgggccatgcatgcttctccatgggtaca
ttatccttaagcatcagaaggatgcctgctttctttgaaaccttcccagtaatcctcatt
gaccaaacaagtacagtgagagcagacattgctttcagacggtccacatctacttatagc
atggaagaatcccaaatacaagtatacttctctggtggttgtttaaatggtacagaatat
tctacaccttcccttgtaaaagcttatgcaagaaaagctcagtttggtcagatatttaca
tttgataaaaagacatccagattgactgatggtgtatttagaacatctgctcgaggttgg
tattcattctcacacatagctttagctttctcctttttctttggccatttgtggcatgct
agtcgtgctatcttccaagacatttggacaggtgtaacatttgaatcacaagcaaaacaa
gaatatggtagaaatgaaaagctaggagataagacatcttcaactaaatccattgtttaa
>Hd2_psbB
atgcaaataactgatgctttaccttggtttagggtacatatagttattcttaatgatcca
ggacgtctcattagctcacatattatgcacacagctttagtagcaggttggtcagctctc
atgcttttatatgagcttatcaccatagatcctacagatcctgtgtataatccaatttgg
agacaagcagcttacacactcccatttatctcacgtattggtgttattcgctctcttttt
agttggtcacttggcatagatcctacatccaatctaatctggacttatgaaacaatgaac
atagcccatattctattatctggtttattaattcttgcatcattttggcattgggcatat
tgggacttagatctattcttcacctcaacattaacactagaccttaatcaaatcctcagt
attcacctcacactagcatcatctctctgtctgggatttggtttagctcatctaactggt
tttcttggtccaggaatgtggactagtgattccctcaatcttgtaggctctattagattc
gtaaaagcctccttcaatctccttgcttatgcacgattagcttatggtgtcatatcatca
catcacatcatctctggattgcttggcacttccataggattatggcatatcacattacgt
ccattagcatacctctacaatctattaagcatgggaaaggttgagtccattctttcaagt
agtattacagctgtattcttcactgcattccttatttcagcccttatgtggtatggctct
gcacacaccacacaagaactcttcggtcctactagatactcatgggacaatgcttattac
tctcttgatatcagaacctcttgctcaaatgcctggaacactcttccagacaagttagtc
ctttatgattatattggatctaaccctgctaaaggtggcttatttcgttcaggaccaatg
cttaaagctgatggtcttgttcaaaactggttgggccatgcatgcttctccatgggtaca
ttatccttaagcatcagaaggatgcctgctttctttgaaaccttcccagtaatcctcatt
gaccaaacaagtacagtgagagcagacattgctttcagacggtccacatctacttatagc
atggaagaatcccaaatacaagtatacttctctggtggttgtttaaatggtacagaatat
tctacaccttcccttgtaaaagcttatgcaagaaaagctcagtttggtcagatatttaca
tttgataaaaagacatccagattgactgatggtgtatttagaacatctgctcgaggttgg
tattcattctcacacatagctttagctttctcctttttctttggccatttgtggcatgct
agtcgtgctatcttccaagacatttggacaggtgtaacatttgaatcacaagcaaaacaa
gaatatggtagaaatgaaaagctaggagataagacatcttcaactaaatccattgtttaa
>Hd3_psbB
atgcaaataactgatgctttaccttggtttagggtacatatagttattcttaatgatcca
ggacgtctcattagctcacatattatgcacacagctttagtagcaggggtggtcagctctc
atgcttttatatgagcttatcaccatagatcctacagatcctgtgtataatccaatttgg
agacaagcagcttacacactcccatttatctctcgtattggtgttattcgctctcttttc

agttggtcacttggcatagatcctacatccaatctaatctggacttatgaaacaatgaac
atagcccatattctattatctggtttattaattcttgcatcattttggcattgggcatat
tgggacttagatctattcttcacctcaacattaacactagaccttaatcaaatcctcagt
attcacctcacactagcatcatctctctgtctgggatttggtttagctcatctaactggt
tttcttggtccaggaatgtggactagtgattccctcaatcttgtaggctctattagattc
gtaaaagcctccttcaatctccttgcttatgcacgattagcttatggtgtcatatcatca
catcacatcatctctggattgcttggcacttccataggattatggcatatcacattacgt
ccattagcatacctctacaatctattaagcatgggaaaggttgagtccattctttcaagt
agtattacagctgtattcttcactgcattccttatttcagccctgatgtggtatggttct
gcacataccacacaagaactattcggtcctactagatactcatgggacaatgcttattac
tctcttgatatcagaacctcttgctcaaatgcctggaacactcttccagacaagttagta
ctttatgattatattggctctaaccctgctaaaggtggcttatttcgttcaggaccaatg
cttaaagctgatggtcttgttcaaaactggttgggccatgcatgcttctccatgggtaca
ttatccttaagcatcagaaggatgcctgctttctttgaaaccttcccagtaatcctcatt
gaccaaacaagtacagtgagagcagacattgctttcagacggtccacatctacttatagc
atggaagaatcccaaatacaagtatacttctctggtggttgtttaaatggtacagaatat
tctacaccttcccttgtaaaagcttatgcaagaaaagctcagtttggtcagatatttaca
tttgataaaaagacatccagattgactgatggtgtatttagaacatcttctcgaggttgg
tattcattctcacacatagctttagctttctcctttttctttggccatttgtggcatgct
agtcgtgctatcttcaaagacatttggacaggtgtaacatttgaatctcaagcaaaacaa
gaatatggtagaaatgaaaagctaggagataagacatcttcaactaaatccattgtttaa
>Hd4_psbB
atgcaaataactgatgctttaccttggtttagggtacatatagttattcttaatgatcca
ggacgtctcattagctcacatattatgcacacagctttagtagcaggttggtcagctctc
atgcttttatatgagcttatcaccatagatcctacagatcctgtgtataatccaatttgg
agacaagcagcttacacactcccatttatctcacgtattggtgttattcgctctcttttt
agttggtcacttggcatagatcctacatccaatctaatctggacttatgaaacaatgaac
atagcccatattctattatctggtttattaattcttgcatcattttggcattgggcatat
tgggacttagatctattcttcacctcaacattaacactagaccttaatcaaatcctcagt
attcacctcacactagcatcatctctctgtctgggatttggtttagctcatctaacaggt
tttcttggtccaggaatgtggactagtgattccctcaatcttgtaggctctattagattc
gtaaaagcctccttcaatctccttgcttatgcacgattagcttatggtgtcatatcatca
catcacatcatctctggattgcttggcacttccataggattatggcatatcacattacgt
ccattagcatacctctacaatctattaagcatgggaaaggttgagtccattctttcaagt
agtattacagctgtattcttcactgcattccttatttcagcccttatgtggtatggctct
gcacacaccacacaagaactcttcggtcctactagatactcatgggacaatgcttattac
tctcttgatatcagaacctcttgctcaaatgcctggaacactcttccagacaagttagtc
ctttatgattatattggatctaaccctgctaaaggtggcttatttcgttcaggaccaatg
cttaaagctgatggtcttgttcaaaactggttgggccatgcatgcttctccatgggtaca
ttatccttaagcatcagaaggatgcctgctttctttgaaaccttcccagtaatcctcatt
gaccaaacaagtacagtgagagcagacattgctttcagacggtccacatctacttatagc
atggaagaatcccaaatacaagtatacttctctggtggttgtttaaatggtacagaatat
tctacaccttcccttgtaaaagcttatgcaagaaaagctcagtttggtcagatatttaca
tttgataaaaagacatccagattgactgatggtgtatttagaacatctgctcgaggttgg
tattcattctcacacatagctttagctttctcctttttctttggccatttgtggcatgct
agtcgtgctatcttccaagacatttggacaggtgtaacatttgaatcacaagcaaaacaa
gaatatggtagaaatgaaaagctaggagataagacatcttcaactaaatccattgtttaa
>Hd5_psbB
atgcaaataactgatgctttaccttggtttagggtacatatagttattcttaatgatcca
ggacgtctcattagctcacatattatgcacacagctttagtagcaggttggtcagctctc
atgcttttatatgagcttatcaccatagatcctacagatcctgtgtataatccaatttgg
agacaagcagcttacacactcccatttatctcacgtattggtgttattcgctctcttttt
agttggtcacttggcatagatcctacatccaatctaatctggacttatgaaacaatgaac
atagcccatattctattatctggtttattaattcttgcatcattttggcattgggcatat
tgggacttagatctattcttcacctcaacattaacactagaccttaatcaaatcctcagt
attcacctcacactagcatcatctctctgtctgggatttggtttagctcatctaactggt
tttcttggtccaggaatgtggactagtgattccctcaatcttgtaggctctattagattc

gtaaaagcctccttcaatctccttgcttatgcacgattagcttatggtgtcatatcatca
catcacatcatctctggattgcttggcacttccataggattatggcatatcacattacgt
ccattagcatacctctacaatctattaagcatgggaaaggttgagtccattctttcaagt
agtattacagctgtattcttcactgcattccttatttcagcccttatgtggtatggctct
gcacacaccacacaagaactcttcggtcctactagatactcatgggacaatgcttattac
tctcttgatatcagaacctcttgctcaaatgcctggaacactcttccagacaagttagtc
ctttatgattatattggatctaaccctgctaaaggtggcttatttcgttcaggaccaatg
cttaaagctgatggtcttgttcaaaactggttgggccatgcatgcttctccatgggtaca
ttatccttaagcatcagaaggatgcctgctttctttgaaaccttcccagtaatcctcatt
gaccaaacaagtacagtgagagcagacattgctttcagacggtccacatctacttatagc
atggaagaatcccaaatacaagtatacttctctggtggttgtttaaatggtacagaatat
tctacaccttcccttgtaaaagcttatgcaagaaaagctcagtttggtcagatatttaca
tttgataaaaagacatccagattgactgatggtgtatttagaacatctgctcgaggttgg
tattcattctcacacatagctttagctttctcctttttctttggccatttgtggcatgct
agtcgtgctatcttccaagacatttggacaggtgtaacatttgaatcacaagcaaaacaa
gaatatggtagaaatgaaaagctaggagataagacatcttcaactaaatccattgtttaa
>Hd6_psbB
atgcaaataactgatgctttaccttggtttagggtacatatagttattcttaatgatcca
ggacgtctcattagctcacatattatgcacacagctttagtagcaggttggtcagctctc
atgcttttatatgagcttatcaccatagatcctacagatcctgtgtataatccaatttgg
agacaagcagcttacacactcccatttatctctcgtattggtgttattcgctctcttttt
agttggtcacttggcatagatcctacatccaatctaatctggacttatgaaacaatgaac
atagcccatattctattatctggtttattaattcttgcatcattttggcattgggcatat
tgggacttagatctattcttcacctcaacattaacactagaccttaatcaaatcctcagt
attcacctcacactagcatcatctctctgtctgggatttggtttagctcatctaactggt
tttcttggtccaggaatgtggactagtgattccctcaatcttgtaggctctattagattc
gtaaaagcctccttcaatctccttgcttatgcacgattagcttatggtgtcatatcatca
catcacatcatctctggattgcttggcacttccataggattatggcatatcacattacgt
ccattagcatacctctacaatctattaagcatgggaaaggttgagtccattctttcaagt
agtattacagctgtattcttcactgcattccttatttcagcccttatgtggtatggctct
gcacacaccacacaagaactcttcggtcctactagatactcatgggacaatgcttattac
tctcttgatatcagaacctcttgctcaaatgcctggaacactcttccagacaagttagtc
ctttatgattatattggatctaaccctgctaaaggtggcttatttcgttcaggaccaatg
cttaaagctgatggtcttgttcaaaactggttgggccatgcatgtttctccatgggtaca
ttatccttaagcatcagaaggatgcctgctttctttgaaaccttcccagtaatcctcatt
gaccaaacaagtacagtgagagcagacattgctttcagacggtccacatctacttatagc
atggaagaatcccaaatacaagtatacttctctggtggttgtttaaatggtacagaatat
tctacaccttcccttgtaaaagcttatgcaagaaaagctcagtttggtcagatatttaca
tttgataaaaagacatccagattgactgatggtgtatttagaacatctgctcgaggttgg
tattcattctcacacataggtttagctttcctattcttctttggccatttgtggcatgct
agtcgtgctatcttcaaagacatttggacaggtgtaacatttgaatctcaagcaaaacaa
gaatatggtagaaatgaaaagctaggagataagacatcttcaactaaatccattgtttaa
>Hd7_psbB
atgcaaataactgatgctttaccttggtttagggtacatatagttattcttaatgatcca
ggacgtctcattagctctcacattatgcacacagctttagtagcaggttggtcagctctc
atgcttttatatgagcttatcaccatagatcctacagatcctgtgtataatccaatttgg
agacaagcagcttacacactcccatttatctcacgtattggtgttattcgctctcttttc
agttggtcacttggcatagatcctacatccaatctaatctggacttatgaaacaatgaac
atagcccatattctattatctggtttattaattcttgcatcattttggcattgggcatat
tgggacttagatctattcttcacctcaacattaacactagaccttaatcaaatcctcagt
attcacctcacactagcatcatctctctgtctgggatttggtttagctcatctaactggt
tttcttggtccaggaatgtggactagtgattccctcaatcttgtaggctctattagattc
gtaaaagcctccttcaatctccttgcttatgcacgattagcttatggtgtcatatcatca
catcacatcatctctggattgcttggcacttccataggattatggcatatcacattacgt
ccattagcatacctctacaatctattaagcatgggaaaggttgagtccattctttcaagt
agtattacagctgtattcttcactgcattccttatttcagcccttatgtggtatggctct
gcacacaccacacaagaactcttcggtcctactagatactcatgggacaatgcttattac

tctcttgatatcagaacctcttgctcaaatgcctggaacactcttccagacaagttagtc
ctttatgattatattggatctaaccctgctaaaggtggcttatttcgttcaggaccaatg
cttaaagctgatggtcttgttcaaaactggttaggtcatgcatgtttctccatgggtaca
ttatccttaagcatcagaaggatgcctgctttctttgaaaccttcccagtaatcctcatt
gaccaaacaagtacagtgagagcagacattgctttcagacggtccacatctacttatagc
atggaagaatcccaaatacaagtatacttctctggtggttgtttaaatggtacagaatat
tctacaccttcccttgtaaaagcttatgcaagaaaagctcagtttggtcagatatttaca
tttgataaaaagacatccagattgactgatggtgtatttagaacatctgctcgaggttgg
tattcattctcacacatagctttagctttctcctttttctttggccatttgtggcatgct
agtcgtgctatcttccaagacatttggacaggtgtaacatttgaatcacaagcaaaacaa
gaatatggtagaaatgaaaagctaggagataagacatcttcaactaaatccattgtttaa
>Hd8_psbB
atgcaaataactgatgctttaccttggtttagggtacatatagttattcttaatgatcca
ggacgtctcattagctcacatattatgcacacagctttagtagcaggttggtcagctctc
atgcttttatatgagcttatcaccatagatcctacagatcctgtgtataatccaatttgg
agacaagcagcttacacactcccatttatctcacgtattggtgttattcgctctctttt
agttggtcacttggcatagatcctacatccaatctaatctggacttatgaaacaatgaac
atagcccatattctattatctggtttattaattcttgcatcattttggcattgggcatat
tgggacttagatctattcttcacctcaacattaacactagaccttaatcaaatcctcagt
attcacctcacactagcatcatctctctgtctgggatttggtttagctcatctaactggt
tttcttggtccaggaatgtggactagtgattccctcaatcttgtaggctctattagattc
gtaaaagcctccttcaatctccttgcttatgcacgattagcttatggtgtcatatcatca
catcacatcatctctggattgcttggcacttccataggattatggcatatcacattacgt
ccattagcatacctctacaatctattaagcatgggaaaggttgagtccattctttcaagt
agtattacagctgtattcttcactgcattccttatttcagcccttatgtggtatggctct
gcacacaccacacaagaactcttcggtcctactagatactcatgggacaatgcttattac
tctcttgatatcagaacctcttgctcaaatgcctggaacactcttccagacaagttagtc
ctttatgattatattggatctaaccctgctaaaggtggcttatttcgttcaggaccaatg
cttaaagctgatggtcttgttcaaaactggttgggccatgcatgcttctccatgggtaca
ttatccttaagcatcagaaggatgcctgctttctttgaaaccttcccagtaatcctcatt
gaccaaacaagtacagtgagagcagacattgctttcagacggtccacatctacttatagc
atggaagaatcccaaatacaagtatacttctctggtggttgtttaaatggtacagaatat
tctacaccttcccttgtaaaagcttatgcaagaaaagctcagtttggtcagatatttaca
tttgataaaaagacatccagattgactgatggtgtatttagaacatctgctcgaggttgg
tattcattctcacacatagctttagctttctcctttttctttggccatttgtggcatgct
agtcgtgctatcttccaagacatttggacaggtgtaacatttgaatcacaagcaaaacaa
gaatatggtagaaatgaaaagctaggagataagacatcttcaactaaatccattgtttaa
>Hd9_psbB
atgcaaataactgatgctttaccttggtttagggtacatatagttattcttaatgatcca
ggacgtctcattagctctcacattatgcacacagctttagtagctgggtggtcagctctc
atgcttttatatgagcttatcaccatagatcctacagatcctgtgtataatccaatttgg
agacaagcagcttacacactcccatttatctcacgtatcggtgttattcgctctcttttc
agttggtcacttggcatagatcctacatccaatctaatctggacttatgaaacaatgaac
atagcccatattctattatctggtttattaattcttgcatcattttggcattgggcatat
tgggacttagatctattcttcacctcaacattaacactagaccttaatcaaatcctcagt
attcacctcacactagcatcatctctctgtctgggatttggtttagctcatctaacaggt
tttcttggtccaggaatgtggactagtgattccctcaatcttgtaggctctattagattc
gtaaaagcctccttcaatatcctttcttatgcacgattagcttatggtgtcatatcatca
catcacatcatctctggattgcttggcacttccataggattatggcatatcacattacgt
ccattagcatacctctacaatctattaagcatgggaaaggttgagtccattctttcaagt
agtattacagctgtattcttcactgcattccttatttcagccctgatgtggtatggttct
gcacataccacacaagaactattcggtcctactagatactcatgggacaatgcttattac
tctcttgatatcagaacctcttgctcaaatgcctggaacactcttccagacaagttagtc
ctttatgattatattggatctaaccctgctaaaggtggcttatttcgttcaggaccaatg
cttaaagctgatggtcttgttcaaaactggttaggtcatgcatgtttctccatgggtaca
ttatccttaagcatcagaaggatgcctgctttctttgaaaccttcccagtaatcctcatt
gaccaaacaagtacagtgagagcagacattgcttttagacggtccacatctacttatagc

atggaagaatcccaaatacaagtatacttctctggtggttgtttaaatggtacagaatat
tctacaccttcccttgtaaaagcttatgcaagaaaagctcagtttggtcagatatttaca
tttgataaaaagacatccagattgactgatggtgtatttagaacatctgctcgaggttgg
tattcattctcacacataggtttagctttcctattcttctttggccatttgtggcatgct
agtcgtgctatcttcaaagacatttggacaggtgtaacatttgaatctcaagcaaaacaa
gaatatggtagaaatgaaaagctaggagataagacatcttcaactaaatccattgtttaa
>IS1_psbB
atgcaaataactgatgctttaccttggtttagagtacatatagttgttcttaatgatcca
ggacgtctcattagttcacacattatgcacacagctttagtagctgggtggtcagctctc
atgcttttatatgagcttatcaccatagatcctacagatcctgtgtataatccaatttgg
agacaagcagcttacacactcccatttatctctcgtatcggtgttattcgctctcttttc
agttggtcacttggcatagatcctacatccaatctaatctggacttatgaaacaatgaac
atagcccatattctattatcaggtttattaatccttgcagcattttggcattgggcatat
tgggacttagatctattcttcaccccaagattaacactagaccttaatcaaatcttcagt
attcatctcacacttgcatcctctctctgtctgggatttggtttagctcatctaacaggt
tttcttggtccaggaatgtggactagtgattccctcaatcttgtaggctctattagattc
gtaaaagcctccttcaatatcctttcttatgcacgattagcttatggtgtcatatcatca
catcacatcatctctggattgcttggcacttccataggattatggcatatcacattacgt
ccattagcatatctctacaatctattaagtatgggaaagattgagtccattctttcaagt
agtattacagctgtattcttcactgcattccttatttcagccctgatgtggtatggttct
gcacataccacacaagaactcttcggtcctactagatactcatgggacaatgcttattac
tctcttgatatcagaacctcttgctcaaatgcctggaacactcttccagacaagttagta
ctttatgattatattggatctaaccctgctaaaggtggtttattccgttcaggacctatg
cttaaagctgatggtcttgttcaaaactggttaggtcatgcatgtttctccatgggtaca
ttatccttaagcatcagaaggatgcctgctttctttgaaaccttcccagtaatcctcatt
gaccaaacaagtacagtgagagcagacattgctttcagacgctctacatccacttatagc
atggaagaatcacaaatacaagtatacttctctggtggttgtttaaatggtacagaatat
tctacaccttctctagttaaagcttatgcaagaaaagctcagtttggtcaaatatttaca
tttgataaaaagacatctagattgactgatggtgtatttagaacatcttctcgaggttgg
tattcattctcacacatagctttagctttcctcttcttcttcggtcatttgtggcatgct
agtcgtgctatattcaaagacatttggacaggtgtaacatttgaatctcaagcaaaacaa
gaatatggtagaaatgaaaagctaggagataagacatcttcaactaaatccattgtttaa
>IS2_psbB
atgcaaataactgatgctttaccttggtttagggtacatatagttattcttaatgatcca
ggacgtctcattagctcacatattatgcacacagctttagtagcaggttggtcagctctc
atgcttttatatgagcttatcaccatagatcctacagatcctgtgtataatccaatttgg
agacaagcagcttacacactcccatttatctcacgtattggtgttattcgctctcttttt
aattggtcacttggcatagatcctacatccaatctaatctggacttatgaaacaatgaac
atagcccatattctattatctggtttattaattcttgcatcattttggcattgggcatat
tgggacttagatctattcttcacctcaacattaacactagaccttaatcaaatcctcagt
attcacctcacactagcatcatctctctgtctgggatttggtttagctcatctaactggt
tttcttggtccaggaatgtggactagtgattccctcaatcttgtaggctctattagattc
gtaaaagcctccttcaatctccttgcttatgcacgattagcttatggtgtcatatcatca
catcacatcatctctggattgcttggcacttccataggattatggcatatcacattacgt
ccattagcatacctctacaatctattaagcatgggaaagattgagtccattctttcaagt
agtattacagctgtattcttcactgcattccttatttcagcccttatgtggtatggctct
gcacacaccacacaagaactcttcggtcctactagatactcatgggacaatgcttattac
tctcttgatatcagaacctcttgctcaaatgcctggaacactcttccagacaagttagtc
ctttatgattatattggatctaaccctgctaaaggtggcttatttcgttcaggaccaatg
cttaaagctgatggtcttgttcaaaactggttgggccatgcatgcttctccatgggtaca
ttatccttaagcatcagaaggatgcctgctttctttgaaaccttcccagtaatcctcatt
gaccaaacaagtacagtgagagcagacattgctttcagacggtccacatctacttatagc
atggaagaatcccaaatacaagtatacttctctggtggttgtttaaatggtacagaatat
tctacaccttcccttgtaaaagcttatgcaagaaaagctcagtttggtcagatatttaca
tttgataaaaagacatccagattgactgatggtgtatttagaacatctgctcgaggttgg
tattcattctcacacatagctttagctttctcctttttctttggccatttgtggcatgct
agtcgtgctatcttccaagacatttggacaggtgtaacatttgaatcacaagcaaaacaa

gaatatggtagaaatgaaaagctaggagataagacatcttcaactaaatccattgtttaa
>IS3_psbB
atgcaaataactgatgctttaccttggtttagagttcatatagttgttcttaatgatcca
ggacgtctcattagttcacacattatgcacaccgctttagtagctgggtggtcggctcta
atgcttttatacgagcttatcaccatagatcctacagaccctgtgtataacccaatttgg
agacaagccgcttatacactcccatttatctcacgtattggtgttattcgctctcttttt
aattggtcaattgctatagatcctacttctaatctaatctggacttatgaaacaattaac
acagcccacattctattatcaggtttattaatccttgcagcattttggcattgggcatat
tgggacttagatctattcttcaccccaagattaacactagaccttaatcaaatcttcagt
attcatctcacacttgcatcctctctctgtttaggatatggtttagctcatttaactggc
ttttttggtccaggaatgtggactagtgattctcttaatcttgttggctctgttagattc
gtaaaagcctcctttaatctccttgcttatgcacgattagcttatggtgtcatatcatca
catcacatcatctctggattgcttggcacttctataggattatggcatatcacattacgt
ccattagcatatctctacaatctattaagtatgggaaagattgagtccattctttcaagt
agtattacagctgtattcttcactgcattccttatatcagctcttatgtggtatggatct
gcacacaccacacaagaactcttcggtcctactagatactcatgggacaatgcttattac
tctcttgatatcagaacctcttgctcaaatgcctggaacactctcccagataagttagtc
ctttatgattatattggatctaaccctgctaaaggtggtttattccgttcaggacctatg
cttaaagctgatggacttgttcaaaactggttggggcacgcatgcttctcgatgggtaca
ttatccctaagcatcagaaggatgcctgctttctttgaaagcttcccagtaatcctcatt
gaccaaacaagtacagtgagagcagacattgctttcagacgctctacatccacttatagc
atggaagaatcacaaatacaagtatacttctctggtggttgtttaaatggtacagaatat
tctacaccttctctagttaaagcttatgcaagaaaagctcagtttggtcaaatatttaca
tttgataaaaagacatctagattgactgatggtgtatttagaacatctgctcgaggttgg
tattcattctcacacatagctttagctttcctcttcttcttcggtcatctgtggcatgct
agtcgtgctatattccaagacatttggacaggtgtaagctttgaatcacaagcaaagcaa
gaatatggtagaaatgaaaagctaggagataagacatcttcaactaaatccattgtttaa
>IS4_psbB
atgcaaataactgatgctttaccttggtttagagttcatatagttattcttaatgatcca
ggacgtctcattagctcacatattatgcacaccgctttagtagctgggtggtcggctcta
atgcttttatacgagcttatcaccatagatcctacagaccctgtgtataacccaatttgg
agacaagccgcttatacactcccatttatctcacgtattggtgttattcgctctcttttt
aattggtcaattggtatagatcctacttctaatctaatatggacttatgaaacaattaac
acagcccacattctattatctggtttattaattcttgcagcattttggcattgggcatat
tgggacttagatctattcttcaccccaagattaacactagaccttaatcaaatcttcagt
attcatctcacacttgcatcctctctctgtttaggatatggtttagctcatttaactggc
ttttttggtccaggaatgtggactagtgattctcttaatcttgttggctctgttagattc
gtaaaagcctcctttaatctccttgcttatgcacgattagcttatggtgtcatatcatca
catcacatcatctctggattgcttggcacttctataggattatggcatatcacattacgt
ccattagcatatctctacaatctattaagtatgggaaagattgagtccattctttcaagt
agtattacagctgtattcttcactgcattccttatatcagctcttatgtggtatggatct
gcacacaccacacaagaactcttcggtcctactagatactcatgggacaatgcttattac
tctcttgatatcagaacctcttgctcaaatgcctggaacactctcccagataagttagtc
ctttatgattatattggctctaaccctgctaaaggtggtttatttcgttcaggaccaatg
cttaaagctgatggtcttgttcaaaactggttgggccatgcatgcttctccatgggtaca
ttatccttaagcatcagaaggatgcctgctttctttgaaaccttcccagtaatcctcatt
gaccaaacaagtacagtgagagcagacattgctttcagacgctctacatccacttatagc
atggaagaatcacaaatacaagtatacttctctggtggttgtttaaatggtacagaatat
tctacaccttcccttgttaaagcttatgcaagaaaagctcagtttggtcaaatatttaca
tttgataaaaagacatctagattgactgatggtgtatttagaacatctgctcgaggttgg
tattcattctcacacatagctttagctttcctcttcttcttcggtcatctgtggcatgct
agtcgtgctatattccaagacatttggacaggtgtaagctttgaatcacaagcaaagcaa
gaatatggtagaaatgaaaagctaggagataagacatcttcaactaaatccattgtttaa
>IS5_psbB
atgcaaataactgatgctttaccttggtttagggtacatatagttattcttaatgatcca
ggacgtctcattagctcacatattatgcacacagctttagtagcaggttggtcagctctc
atgcttttatatgagcttatcaccatagatcctacagatcctgtgtataatccaatttgg

agacaagcagcttacacactcccatttatctcacgtattggtgttattcgctctcttttt
agttggtcacttggcatagatcctacatccaatctaatctggacttatgaaacaatgaac
atagcccatattctattatctggtttattaattcttgcatcattttggcattgggcatat
tgggacttagatctattcttcacctcaacattaacactagaccttaatcaaatcctcagt
attcacctcacactagcatcatctctctgtctgggatttggtttagctcatctaactggt
tttcttggtccaggaatgtggactagtgattccctcaatcttgtaggctctattagattc
gtaaaagcctccttcaatctccttgcttatgcacgattagcttatggtgtcatatcatca
catcacatcatctctggattgcttggcacttccataggattatggcatatcacattacgt
ccattagcatacctctacaatctattaagcatgggaaaggttgagtccattctttcaagt
agtattacagctgtattcttcactgcattccttatttcagcccttatgtggtatggctct
gcacacaccacacaagaactcttcggtcctactagatactcatgggacaatgcttattac
tctcttgatatcagaacctcttgctcaaatgcctggaacactcttccagacaagttagtc
ctttatgattatattggatctaaccctgctaaaggtggcttatttcgttcaggaccaatg
cttaaagctgatggtcttgttcaaaactggttgggccatgcatgcttctccatgggtaca
ttatccttaagcatcagaaggatgcctgctttctttgaaaccttcccagtaatcctcatt
gaccaaacaagtacagtgagagcagacattgcttttagacggtccacatctacttatagc
atggaagaatcccaaatacaagtatacttctctggtggttgtttaaatggtacagaatat
tctacaccttcccttgtaaaagcttatgcaagaaaagctcagtttggtcagatatttaca
tttgataaaaagacatccagattgactgatggtgtatttagaacatctgctcgaggttgg
tattcattctcacacatagctttagctttctcctttttctttggccatttgtggcatgct
agtcgtgctatcttccaagacatttggacaggtgtaacatttgaatcacaagcaaaacaa
gaatatggtagaaatgaaaagctaggagataagacatcttcaactaaatccattgtttaa
>IS6_psbB
atgcaaataactgatgctttaccttggtttagggtacatatagttattcttaatgatcca
ggacgtctcattagctcacatattatgcacacagctttagtagcaggttggtcagctctc
atgcttttatatgagcttatcaccatagatcctacagatcctgtgtataatccaatttgg
agacaagcagcttacacactcccatttatctcacgtattggtgttattcgctctcttttt
agttggtcacttggcatagatcctacatccaatctaatctggacttatgaaacaatgaac
atagcccatattctattatctggtttattaattcttgcatcattttggcattgggcatat
tgggacttagatctattcttcacctcaacattaacactagaccttaatcaaatcctcagt
attcacctcacactagcatcatctctctgtctgggatttggtttagctcatctaacaggt
tttcttggtccaggaatgtggactagtgattccctcaatcttgtaggctctattagattc
gtaaaagcctccttcaatctccttgcttatgcacgattagcttatggtgtcatatcatca
catcacatcatctctggattgcttggcacttccataggattatggcatatcacattacgt
ccattagcatacctctacaatctattaagcatgggaaaggttgagtccattctttcaagt
agtattacagctgtattcttcactgcattccttatttcagcccttatgtggtatggctct
gcacacaccacacaagaactcttcggtcctactagatactcatgggacaatgcttattac
tctcttgatatcagaacctcttgctcaaatgcctggaacactcttccagacaagttagtc
ctttatgattatattggatctaaccctgctaaaggtggcttatttcgttcaggaccaatg
cttaaagctgatggtcttgttcaaaactggttgggccatgcatgcttctccatgggtaca
ttatccttaagcatcagaaggatgcctgctttctttgaaaccttcccagtaatcctcatt
gaccaaacaagtacagtgagagcagacattgctttcagacggtccacatctacttatagc
atggaagaatcccaaatacaagtatacttctctggtggttgtttaaatggtacagaatat
tctacaccttcccttgtaaaagcttatgcaagaaaagctcagtttggtcagatatttaca
tttgataaaaagacatccagattgactgatggtgtatttagaacatctgctcgaggttgg
tattcattctcacacatagctttagctttctcctttttctttggccatttgtggcatgct
agtcgtgctatcttccaagacatttggacaggtgtaacatttgaatcacaagcaaaacaa
gaatatggtagaaatgaaaagctaggagataagacatcttcaactaaatccattgtttaa
>IS7_psbB
atgcaaataactgatgctttaccttggtttagagttcatatagttattcttaatgatcca
ggacgtctcattagctcacatattatgcacacagctttagtagcaggttggtcggctcta
atgcttttatacgagcttatcaccatagatcctacagaccctgtgtataacccaatttgg
agacaagccgcttacacactcccatttatctcacgtattggtgttattcgctctcttttt
aattggtcaattggtatagatcctacatccaatctaatctggacttatgaaacaatgaac
atagcccatattctattatctggtttattaattcttgcatcattttggcattgggcatat
tgggacttagatctattcttcacctcaacattaacactagaccttaatcaaatcctcagt
attcacctcacacttgcatcctctctctgtttaggatatggtttagctcatttaactggc

ttttttggtccaggaatgtggactagtgattctcttaatcttgtaggctctattagattc
gtaaaagcctccttcaatatcctttcttatgcacgattagcttatggtgtcatatcatca
catcacatcatctctggattgcttggcacttctataggattatggcatatcacattacgt
ccattagcatatctctacaatctattaagtatgggaaagattgagtccattctttcaagt
agtattacagctgtattcttcactgcattccttatatcagctcttatgtggtatggatct
gcacacaccacacaagaactcttcggtcctactagatactcatgggacaatgcttattac
tctcttgatatcagaacctcttgctcaaatgcctggaacactcttccagacaagttagtc
ctttatgattatattggatctaaccctgctaaaggtggcttatttcgttcaggaccaatg
cttaaagctgatggtcttgttcaaaactggttgggtcatgcatgcttctccatgggtaca
ttatccctaagcatcagaaggatgcctgctttctttgaaagcttcccagtaatcctcatt
gaccaaacaagtacagtgagagcagacattgcttttagacggtccacatctacttatagc
atggaagaatcccaaatacaagtatacttctctggtggttgtttaaatggtacagaatat
tctacaccttcccttgtaaaagcttatgcaagaaaagctcagtttggtcagatatttaca
tttgataaaaagacatctagattgactgatggtgtatttagaacatctgctcgaggttgg
tattcattctcacacatagctttagctttcctcttcttcttcggccatctgtggcatgct
agtcgtgctatattccaagacatttggacaggtgtaacatttgaatcacaagcaaaacaa
gaatatggtagaaatgaaaagctaggagataagacatcttcaactaaatccattgtttaa
>IS8_psbB
atgcaaataactgatgctttaccttggtttagggtacatatagttattcttaatgatcca
ggacgtctcattagctcacatattatgcacacagctttagtagcaggttggtcagctctc
atgcttttatatgagcttatcaccatagatcctacagatcctgtgtataatccaatttgg
agacaagcagcttacacactcccatttatctctcgtattggtgttattcgctctcttttt
agttggtcacttggcatagatcctacatccaatctaatctggacttatgaaacaatgaac
atagcccatattctattatctggtttattaattcttgcatcattttggcattgggcatat
tgggacttagatctattcttcacctcaacattaacactagaccttaatcaaatcctcagt
attcacctcacactagcatcatctctctgtctgggatttggtttagctcatctaactggt
tttcttggtccaggaatgtggactagtgattccctcaatcttgtaggctctattagattc
gtaaaagcctccttcaatctccttgcttatgcacgattagcttatggtgtcatatcatca
catcacatcatctctggattgcttggcacttccataggattatggcatatcacattacgt
ccattagcatacctctacaatctattaagcatgggaaaggttgagtccattctttcaagt
agtattacagctgtattcttcactgcattccttatttcagcccttatgtggtatggctct
gcacacaccacacaagaactcttcggtcctactagatactcatgggacaatgcttattac
tctcttgatatcagaacctcttgctcaaatgcctggaacactcttccagacaagttagtc
ctttatgattatattggatctaaccctgctaaaggtggcttatttcgttcaggaccaatg
cttaaagctgatggtcttgttcaaaactggttgggccatgcatgcttctccatgggtaca
ttatccttaagcatcagaaggatgcctgctttctttgaaaccttcccagtaatcctcatt
gaccaaacaagtacagtgagagcagacattgctttcagacggtccacatctacttatagc
atggaagaatcccaaatacaagtatacttctctggtggttgtttaaatggtacagaatat
tctacaccttcccttgtaaaagcttatgcaagaaaagctcagtttggtcagatatttaca
tttgataaaaagacatccagattgactgatggtgtatttagaacatctgctcgaggttgg
tattcattctcacacatagctttagctttctcctttttctttggccatttgtggcatgct
agtcgtgctatcttcaaagacatttggacaggtgtaacatttgaatctcaagcaaaacaa
gaatatggtagaaatgaaaagctaggagataagacatcttcaactaaatccattgtttaa
>IS9_psbB
atgcaaataactgatgctttaccttggtttagggtacatatagttattcttaatgatcca
ggacgtctcattagctcacacattatgcacacagctttagtagcaggttggtcagctctc
atgcttttatatgagcttatcaccatagatcctacagaccctgtgtataacccaatttgg
agacaagccgcttacacactcccatttatctcacgtattggtgttattcgctctcttttt
aattggtcaattggtatagatcctacatccaatctaatctggacttatgaaacaatgaac
atagcccatattctattatctggtttattaattcttgcatcattttggcattgggcatat
tgggacttagatctattcttcacctcaacattaacactagaccttaatcaaatcctcagt
attcatctcacacttgcatcctctctctgtttaggatatggtttagctcatttaactggc
ttttttggtccaggaatgtggactagtgattccctcaatcttgtaggctctattagattc
gtaaaagcctccttcaatctccttgcttatgcacgattagcttatggtgtcatatcatca
catcacatcatctctggattgcttggcacttctataggattatggcatatcacattacgt
ccattagcatacctctacaatctattaagcatgggaaaggttgagtccattctttcaagt
agtattacagctgtattcttcactgcattccttatttcagcccttatgtggtatggctct

gcacacaccacacaagaactcttcggtcctactagatactcatgggacaatgcttattac
tctcttgatatcagaacctcttgctcaaatgcctggaacactcttccagacaagttagtc
ctttatgattatattggatctaaccctgctaaaggtggtttattccgttcaggacctatg
cttaaagctgatggtcttgttcaaaactggttgggccatgcatgcttctccatgggtaca
ttatccttaagcatcagaaggatgcctgctttctttgaaaccttcccagtaatcctcatt
gaccaaacaagtacagtgagagcagacattgctttcagacgctctacatccacttatagc
atggaagaatcacaaatacaagtatacttctctggtggttgtttaaatggtacagaatat
tctacaccttcccttgtaaaagcttatgcaagaaaagctcagtttggtcagatatttaca
tttgataaaaagacatctagattgactgatggtgtatttagaacatctgctcgaggttgg
tattcattctcacacatagctttagctttcctcttcttctttggccatttgtggcatgct
agtcgtgctatcttcaaagacatttggacaggtgtaacatttgaatcacaagcaaaacaa
gaatatggtagaaatgaaaagctaggagataagacatcttcaactaaatccattgtttaa
>Ik1_psbB
atgcaaataactgatgctttaccttggtttagggtacatatagttattcttaatgatcca
ggacgtctcattagctcacatattatgcacacagctttagtagcaggttggtcagctctc
atgcttttatatgagcttatcaccatagatcctacagatcctgtgtataatccaatttgg
agacaagcagcttacacactcccatttatctcacgtattggtgttattcgctctcttttt
agttggtcacttggcatagatcctacatccaatctaatctggacttatgaaacaatgaac
atagcccatattctattatctggtttattaattcttgcatcattttggcattgggcatat
tgggacttagatctattcttcacctcaacattaacactagaccttaatcaaatcctcagt
attcacctcacactagcatcatctctctgtctgggatttggtttagctcatctaactggt
tttcttggtccaggaatgtggactagtgattccctcaatcttgtaggctctattagattc
gtaaaagcctccttcaatctccttgcttatgcacaattagcttatggtgtcatatcatca
catcacatcatctctggattgcttggcacttccataggattatggcatatcacattacgt
ccattagcatacctctacaatctattaagcatgggaaaggttgagtccattctttcaagt
agtattacagctgtattcttcactgcattccttatttcagccctgatgtggtatggttct
gcacataccacacaagaactattcggtcctactagatactcatgggacaatgcttattac
tctcttgatatcagaacctcttgctcaaatgcctggaacactcttccagacaagttagtc
ctttatgattatattggatctaaccctgctaaaggtggcttatttcgttcaggaccaatg
cttaaagctgatggtcttgttcaaaactggttgggccatgcatgcttctccatgggtaca
ttatccttaagcatcagaaggatgcctgctttctttgaaaccttcccagtaatcctcatt
gaccaaacaagtacagtgagagcagacattgctttcagacggtccacatctacttatagc
atggaagaatcccaaatacaagtatacttctctggtggttgtttaaatggtacagaatat
tctacaccttcccttgtaaaagcttatgcaagaaaagctcagtttggtcagatatttaca
tttgataaaaagacatccagattgactgatggtgtatttagaacatcttctcgaggttgg
tattcattctcacacatagctttagctttctcctttttctttggccatttgtggcatgct
agtcgtgctatcttcaaagacatttggacaggtgtaacatttgaatctcaagcaaaacaa
gaatatggtagaaatgaaaagctaggagataagacatcttcaactaaatccattgtttaa
>Ik2_psbB
atgcaaataactgatgctttaccttggtttagggtacatatagttattcttaatgatcca
ggacgtctcattagctcacatattatgcacacagctttagtagcaggttggtcagctctc
atgcttttatatgagcttatcaccatagatcctacagatcctgtgtataatccaatttgg
agacaagcagcttacacactcccatttatctcacgtattggtgttattcgctctcttttt
agttggtcacttggcatagatcctacatccaatctaatctggacttatgaaacaatgaac
atagcccatattctattatctggtttattaattcttgcatcattttggcattgggcatat
tgggacttagatctattcttcacctcaacattaacactagaccttaatcaaatcctcagt
attcacctcacactagcatcatctctctgtctgggatttggtttagctcatctaactggt
tttcttggtccaggaatgtggactagtgattccctcaatcttgtaggctctattagattc
gtaaaagcctccttcaatctccttgcttatgcacgattagcttatggtgtcatatcatca
catcacatcatctctggattgcttggcacttccataggattatggcatatcacattacgt
ccattagcatacctctacaatctattaagcatgggaaaggttgagtccattctttcaagt
agtattacagctgtattcttcactgcattccttatttcagcccttatgtggtatggctct
gcacacaccacacaagaactcttcggtcctactagatactcatgggacaatgcttattac
tctcttgatatcagaacctcttgctcaaatgcctggaacactcttccagacaagttagtc
ctttatgattatattggatctaaccctgctaaaggtggcttatttcgttcaggaccaatg
cttaaagctgatggtcttgttcaaaactggttgggccatgcatgcttctccatgggtaca
ttatccttaagcatcagaaggatgcctgctttctttgaaaccttcccagtaatcctcatt

gaccaaacaagtacagtgagagcagacattgcttttagacggtccacatctacttatagc
atggaagaatcccaaatacaagtatacttctctggtggttgtttaaatggtacagaatat
tctacaccttcccttgtaaaagcttatgcaagaaaagctcagtttggtcagatatttaca
tttgataaaaagacatccagattgactgatggtgtatttagaacatctgctcgaggttgg
tattcattctcacacatagctttagctttctcctttttctttggccatttgtggcatgct
agtcgtgctatcttcaaagacatttggacaggtgtaacatttgaatcacaagcaaaacaa
gaatatggtagaaatgaaaagctaggagataagacatcttcaactaaatccattgtttaa
>Ik3_psbB
atgcaaataactgatgctttaccttggtttagggtacatatagttattcttaatgatcca
ggacgtctcattagctcacatattatgcacacagctttagtagcaggttggtcagctctc
atgcttttatatgagcttatcaccatagatcctacagatcctgtgtataatccaatttgg
agacaagcagcttacacactcccatttatctcacgtattggtgttattcgctctcttttc
agttggtcacttggcatagatcctacatccaatctaatctggacttatgaaacaatgaac
atagcccatattctattatctggtttattaattcttgcatcattttggcattgggcatat
tgggacttagatctattcttcacctcaacattaacactagaccttaatcaaatcctcagt
attcacctcacactagcatcatctctctgtctgggatttggtttagctcatctaacaggt
tttcttggtccaggaatgtggactagtgattccctcaatcttgtaggctctattagattc
gtaaaagcctccttcaatctccttgcttatgcacgattagcttatggtgtcatatcatca
catcacatcatctctggattgcttggcacttccataggattatggcatatcacattacgt
ccattagcatacctctacaatctattaagcatgggaaaggttgagtccattctttcaagt
agtattacagctgtattcttcactgcattccttatttcagccctgatgtggtatggctct
gcacacaccacacaagaactcttcggtcctactagatactcatgggacaatgcttattac
tctcttgatatcagaacctcttgctcaaatgcctggaacactcttccagacaagttagtc
ctttatgattatattggatctaaccctgctaaaggtggcttatttcgttcaggaccaatg
cttaaagctgatggtcttgttcaaaactggttgggccatgcatgcttctccatgggtaca
ttatccttaagcatcagaaggatgcctgctttctttgaaaccttcccagtaatcctcatt
gaccaaacaagtacagtgagagcagacattgctttcagacggtccacatctacttatagc
atggaagaatcccaaatacaagtatacttctctggtggttgtttaaatggtacagaatat
tctacaccttcccttgtaaaagcttatgcaagaaaagctcagtttggtcagatatttaca
tttgataaaaagacatccagattgactgatggtgtatttagaacatcttctcgaggttgg
tattcattctcacacataggtttagctttcctattcttctttggccatttgtggcatgct
agtcgtgctatcttcaaagacatttggacaggtgtaacatttgaatctcaagcaaaacaa
gaatatggtagaaatgaaaagctaggagataagacatcttcaactaaatccattgtttaa
>Ik4_psbB
atgcaaataactgatgctttaccttggtttagggtacatatagttattcttaatgatcca
ggacgtctcattagctcacatattatgcacacagctttagtagcaggttggtcagctctc
atgcttttatatgagcttatcaccatagatcctacagatcctgtgtataatccaatttgg
agacaagcagcttacacactcccatttatctcacgtattggtgttattcgctctcttttt
agttggtcacttggcatagatcctacatccaatctaatctggacttatgaaacaatgaac
atagcccatattctattatctggtttattaattcttgcatcattttggcattgggcatat
tgggacttagatctattcttcacctcaacattaacactagaccttaatcaaatcctcagt
attcacctcacactagcatcatctctctgtctgggatttggtttagctcatctaactggt
tttcttggtccaggaatgtggactagtgattccctcaatcttgtaggctctattagattc
gtaaaagcctccttcaatctccttgcttatgcacgattagcttatggtgtcatatcatca
catcacatcatctctggattgcttggcacttccataggattatggcatatcacattacgt
ccattagcatacctctacaatctattaagcatgggaaaggttgagtccattctttcaagt
agtattacagctgtattcttcactgcattccttatttcagcccttatgtggtatggctct
gcacacaccacacaagaactcttcggtcctactagatactcatgggacaatgcttattac
tctcttgatatcagaacctcttgctcaaatgcctggaacactcttccagacaagttagta
ctttatgattatattggctctaaccctgctaaaggtggcttatttcgttcaggaccaatg
cttaaagctgatggtcttgttcaaaactggttgggccatgcatgtttctccatgggtaca
ttatccttaagcatcagaaggatgcctgctttctttgaaaccttcccagtaatcctcatt
gaccaaacaagtacagtgagagcagacattgctttcagacggtccacatctacttatagc
atggaagaatcccaaatacaagtatacttctctggtggttgtttaaatggtacagaatat
tctacaccttcccttgtaaaagcttatgcaagaaaagctcagtttggtcagatatttaca
tttgataaaaagacatccagattgactgatggtgtatttagaacatctgctcgaggttgg
tattcattctcacacataggtttagctttcctatttttctttggccatttgtggcatgct

agtcgtgctatcttccaagacatttggacaggtgtaacatttgaatcacaagcaaaacaa
gaatatggtagaaatgaaaagctaggagataagacatcttcaactaaatccattgtttaa
>Ik5_psbB
atgcaaataactgatgctttaccttggtttagggtacatatagttattcttaatgatcca
ggacgtctcattagctcacatattatgcacacagctttagtagcaggttggtcagctctc
atgcttttatatgagcttatcaccatagatcctacagatcctgtgtataatccaatttgg
agacaagcagcttacacactcccatttatctcacgtattggtgttattcgctctcttttt
agttggtcacttggcatagatcctacatccaatctaatctggacttatgaaacaatgaac
atagcccatattctattatctggtttattaattcttgcatcattttggcattgggcatat
tgggacttagatctattcttcacctcaacattaacactagaccttaatcaaatcctcagt
attcacctcacactagcatcatctctctgtctgggatttggtttagctcatctaactggt
tttcttggtccaggaatgtggactagtgattccctcaatcttgtaggctctattagattc
gtaaaagcctccttcaatctccttgcttatgcacgattagcttatggtgtcatatcatca
catcacatcatctctggattgcttggcacttccataggattatggcatatcacattacgt
ccattagcatacctctacaatctattaagcatgggaaaggttgagtccattctttcaagt
agtattacagctgtattcttcactgcattccttatttcagccctgatgtggtatggctct
gcacacaccacacaagaactcttcggtcctactagatactcatgggacaatgcttattac
tctcttgatatcagaacctcttgctcaaatgcctggaacactcttccagacaagttagtc
ctttatgattatattggatctaaccctgctaaaggtggcttatttcgttcaggaccaatg
cttaaagctgatggtcttgttcaaaactggttgggccatgcatgcttctccatgggtaca
ttatccttaagcatcagaaggatgcctgctttctttgaaaccttcccagtaatcctcatt
gaccaaacaagtacagtgagagcagacattgctttcagacggtccacatctacttatagc
atggaagaatcccaaatacaagtatacttctctggtggttgtttaaatggtacagaatat
tctacaccttcccttgtaaaagcttatgcaagaaaagctcagtttggtcagatatttaca
tttgataaaaagacatccagattgactgatggtgtatttagaacatctgctcgaggttgg
tattcattctcacacatagctttagctttctcctttttctttggccatttgtggcatgct
agtcgtgctatcttcaaagacatttggacaggtgtaacatttgaatcacaagcaaaacaa
gaatatggtagaaatgaaaagctaggagataagacatcttcaactaaatccattgtttaa
>Ik6_psbB
atgcaaataactgatgctttaccttggtttagggtacatatagttattcttaatgatcca
ggacgtctcattagctctcacattatgcatacagctttagtagctgggtggtcagctctc
atgcttttatatgagcttatcaccatagatcctacagatcctgtgtataatccaatttgg
agacaagcagcttacacactcccatttatctctcgtatcggtgttattcgctctcttttc
agttggtcacttggcatagatcctacatccaatctaatctggacttatgaaacaatgaac
atagcccatattctattatctggtttattaattcttgcatcattttggcattgggcatat
tgggacttagatctattcttcacctcaacattaacactagaccttaatcaaatcctcagt
attcacctcacactagcatcatctctctgtctgggatttggtttagctcatctaactggt
tttcttggtccaggaatgtggactagtgattccctcaatcttgtaggctctattagattc
gtaaaagcctccttcaatctccttgcttatgcacgattagcttatggtgtcatatcatca
catcacatcatctctggattgcttggcacttccataggattatggcatatcacattacgt
ccattagcatacctctacaatctattaagcatgggaaaggttgagtccattctttcaagt
agtattacagctgtattcttcactgcattccttatttcagccctgatgtggtatggttct
gcacataccacacaagaactattcggtcctactagatactcatgggacaatgcttattac
tctcttgatatcagaacctcttgctcaaatgcctggaacactcttccagacaagttagta
ctttatgattatattggctctaaccctgctaaaggtggcttatttcgttcaggaccaatg
cttaaagctgatggtcttgttcaaaactggttgggccatgcatgcttctccatgggtaca
ttatccttaagcatcagaaggatgcctgctttctttgaaaccttcccagtaatcctcatt
gaccaaacaagtacagtgagagcagacattgctttcagacggtccacatctacttatagc
atggaagaatcccaaatacaagtatacttctctggtggttgtttaaatggtacagaatat
tctacaccttcccttgtaaaagcttatgcaagaaaagctcagtttggtcagatatttaca
tttgataaaaagacatccagattgactgatggtgtatttagaacatcttctcgaggttgg
tattcattctcacacataggtttagctttcctattcttctttggccatttgtggcatgct
agtcgtgctatcttcaaagacatttggacaggtgtaacatttgaatctcaagcaaaacaa
gaatatggtagaaatgaaaagctaggagataagacatcttcaactaaatccattgtttaa
>Ik7_psbB
atgcaaataactgatgctttaccttggtttagggtacatatagttattcttaatgatcca
ggacgtctcattagctcacatattatgcacacagctttagtagcagggtggtcagctctc

atgcttttatatgagcttatcaccatagatcctacagatcctgtgtataatccaatttgg
agacaagcagcttacacactcccatttatctcacgtattggtgttattcgctctcttttt
agttggtcacttggcatagatcctacatccaatctaatctggacttatgaaacaatgaac
atagcccatattctattatctggtttattaattcttgcatcattttggcattgggcatat
tgggacttagatctattcttcacctcaacattaacactagaccttaatcaaatcctcagt
attcacctcacactagcatcatctctctgtctgggatttggtttagctcatctaactggt
tttcttggtccaggaatgtggactagtgattccctcaatcttgtaggctctattagattc
gtaaaagcctccttcaatatccttcttatgcacgattagcttatggtgtcatatcatca
catcacatcatctctggattgcttggcacttccataggattatggcatatcacattacgt
ccattagcatacctctacaatctattaagcatgggaaaggttgagtccattctttcaagt
agtattacagctgtattcttcactgcattccttatttcagcccttatgtggtatggctct
gcacacaccacacaagaactcttcggtcctactagatactcatgggacaatgcttattac
tctcttgatatcagaacctcttgctcaaatgcctggaacactcttccagacaagttagtc
ctttatgattatattggatctaaccctgctaaaggtggcttatttcgttcaggaccaatg
cttaaagctgatggtcttgttcaaaactggttgggccatgcatgcttctccatgggtaca
ttatccttaagcatcagaaggatgcctgctttctttgaaaccttcccagtaatcctcatt
gaccaaacaagtacagtgagagcagacattgctttcagacggtccacatctacttatagc
atggaagaatcccaaatacaagtatacttctctggtggttgtttaaatggtacagaatat
tctacaccttcccttgtaaaagcttatgcaagaaaagctcagtttggtcagatatttaca
tttgataaaaagacatccagattgactgatggtgtatttagaacatcttctcgaggttgg
tattcattctcacacatagctttagctttctccttttctttggccatttgtggcatgct
agtcgtgctatcttccaagacatttggacaggtgtaacatttgaatcacaagcaaaacaa
gaatatggtagaaatgaaaagctaggagataagacatcttcaactaaatccattgtttaa
>Ik8_psbB
atgcaaataactgatgctttaccttggtttagggtacatatagttattcttaatgatcca
ggacgtctcattagctcacatattatgcacacagctttagtagcaggttggtcagctctc
atgcttttatatgagcttatcaccatagatcctacagatcctgtgtataatccaatttgg
agacaagcagcttacacactcccatttatctctcgtatcggtgttattcgctctcttttc
agttggtcacttggcatagatcctacatccaatctaatctggacttatgaaacaatgaac
atagcccatattctattatctggtttattaattcttgcatcattttggcattgggcatat
tgggacttagatctattcttcacctcaacattaacactagaccttaatcaaatcctcagt
attcacctcacactagcatcatctctctgtctgggatttggtttagctcatctaactggt
tttcttggtccaggaatgtggactagtgattccctcaatcttgtaggctctattagattc
gtaaaagcctccttcaatatccttcttatgcacgattagcttatggtgtcatatcatca
catcacatcatctctggattgcttggcacttccataggattatggcatatcacattacgt
ccattagcatacctctacaatctattaagcatgggaaaggttgagtccattctttcaagt
agtattacagctgtattcttcactgcattccttatttcagcccttatgtggtatggctct
gcacacaccacacaagaactcttcggtcctactagatactcatgggacaatgattattac
tctcttgatatcagaacctcttgctcaaatgcctggaacactcttccagacaagttagtc
ctttatgattatattggatctaaccctgctaaaggtggcttatttcgttcaggaccaatg
cttaaagctgatggtcttgttcgaaactggttgggccatgcatgcttctccatgggtaca
ttatccttaagcatcagaaggatgcctgctttctttgaaaccttcccagtaatcctcatt
gaccaaacaagtacagtgagagcagacattgctttaagacggtccacatctacttatagc
atggaagaatcccaaatacaagtatacttctctggtggttgtttaaatggtacagaatat
tctacaccttcccttgtaaaagcttatgcaagaaaagctcagtttggtcagatatttaca
tttgataaaaagacatccagattgactgatggtgtatttagaacatcttctcgaggttgg
tattcattctcacacatagctttagctttcttcttttctttggccatttgtggcatgct
agtcgtgctatcttcaaagacatttggacaggtgtaacatttgaatcacaagcaaaacaa
gaatatggtagaaatgaaaagctaggagataagacatcttcaactaaatccattgtttaa
>Ik9_psbB
atgcaaataactgatgctttaccttggtttagggtacatatagttattcttaatgatcca
ggacgtctcattagctctcacattatgcatacagctttagtagctggttggtcagctctc
atgcttttatatgagcttatcaccatagatcctacagatcctgtgtataatccaatttgg
agacaagcagcttacacactcccatttatctctcgtatcggtgttattcgctctcttttc
agttggtcacttggcatagatcctacatccaatctaatctggacttatgaaacaatgaac
atagcccatattctattatctggtttattaattcttgcatcattttggcattgggcatat
tgggacttagatctattcttcacctcaacattaacactagaccttaatcaaatcctcagt

attcacctcacactagcatcatctctctgtctgggatttggtttagctcatctaactggt
tttcttggtccaggaatgtggactagtgattccctcaatcttgtaggctctattagattc
gtaaaagcctccttcaatatcctttcttatgcacgattagcttatggtgtcatatcatca
catcacatcatctctggattgcttggcacttccataggattatggcatatcacattacgt
ccattagcatacctctacaatctattaagcatgggaaaggttgagtccattctttcaagt
agtattacagctgtattcttcactgcattccttatttcagcccttatgtggtatggctct
gcacacaccacacaagaactattcggtcctactagatactcatgggacaatgcttattac
tctcttgatatcagaacctcttgctcaaatgcctggaacactcttccagacaagttagtc
ctttatgattatattggatctaaccctgctaaaggtggcttatttcgttcaggaccaatg
cttaaagctgatggtcttgttcaaaactggttgggccatgcatgtttctccatgggtaca
ttatccttaagcatcagaaggatgcctgctttctttgaaaccttcccagtaatcctcatt
gaccaaacaagtacagtgagagcagacattgctttcagacggtccacatctacttatagc
atggaagaatcccaaatacaagtatacttctctggtggttgtttaaatggtacagaatat
tctacaccttcccttgtaaaagcttatgcaagaaaagctcagtttggtcagatatttaca
tttgataaaaagacatccagattgactgatggtgtatttagaacatctgctcgaggttgg
tattcattctcacacatagctttagctttcctattcttctttggccatttgtggcatgct
agtcgtgctatcttccaagacatttggacaggtgtaacatttgaatcacaagcaaaacaa
gaatatggtagaaatgaaaagctaggagataagacatcttcaactaaatccattgtttaa
>Irm10_psbB
atgcaaataactgatgctttaccttggtttagggtacatatagttattcttaatgatcca
ggacgtctcattagctcacatattatgcacacagctttagtagcaggttggtcagctctc
atgcttttatatgagcttatcaccatagatcctacagatcctgtgtataatccaatttgg
agacaagcagcttacacactcccatttatctcacgtattggtgttattcgctctcttttt
agttggtcacttggcatagatcctacatccaatctaatctggacttatgaaacaatgaac
atagcccatattctattatctggtttattaattcttgcatcattttggcattgggcatat
tgggacttagatctattcttcacctcaacattaacactagaccttaatcaaatcctcagt
attcacctcacactagcatcatctctctgtctgggatttggtttagctcatctaactggt
tttcttggtccaggaatgtggactagtgattccctcaatcttgtaggctctattagattc
gtaaaagcctccttcaatctccttgcttatgcacgattagcttatggtgtcatatcatca
catcacatcatctctggattgcttggcacttccataggattatggcatatcacattacgt
ccattagcatacctctacaatctattaagcatgggaaaggttgagtccattctttcaagt
agtattacagctgtattcttcactgcattccttatttcagcccttatgtggtatggctct
gcacacaccacacaagaactattcggtcctactagatactcatgggacaatgcttattac
tctcttgatatcagaacctcttgctcaaatgcctgggacactcttccagacaagttagtc
ctttatgattatattggctctaaccctgctaaaggtggcttatttcgttcaggaccaatg
cttaaagctgatggtcttgttcaaaactggttgggccatgcatgcttctccatgggtaca
ttatccttaagcatcagaaggatgcctgctttctttgaaaccttcccagtaatcctcatt
gaccaaacaagtacagtgagagcagacattgctttcagacggtccacatctacttatagc
atggaagaatcccaaatacaagtatacttctctggtggttgtttaaatggtacagaatat
tctacaccttcccttgtaaaagcttatgcaagaaaagctcagtttggtcagatatttaca
tttgataaaaagacatccagattgactgatggtgtatttagaacatctgctcgaggttgg
tattcattctcacacatagctttagctttctcctttttctttggccatttgtggcatgct
agtcgtgctatcttccaagacatttggacaggtgtaacatttgaatcacaagcaaaacaa
gaatatggtagaaatgaaaagctaggagataagacatcttcaactaaatccattgtttaa
>Irm17_psbB
atgcaaataactgatgttttaccttggtttagggtacatatagttattcttaatgatcca
ggacgtctcattagctcacatattatgcacacagctttagtagcaggttggtcagctctc
atgcttttatatgagcttatcaccatagatcctacagatcctgtgtataatccaatttgg
agacaagcagcttacacactcccatttatctcacgtattggtgttattcgctctcttttt
agttggtcacttggcatagatcctacatccaatctaatctggacttatgaaacaatgaac
atagcccatattctattatctggtttattaattcttgcatcattttggcattgggcatat
tgggacttagatctattcttcacctcaacattaacactagaccttaatcaaatcctcagt
attcacctcacactagcatcatctctctgtctgggatttggtttagctcatctaactggt
tttcttggtccaggaatgtggactagtgattccctcaatcttgtaggctctattagattc
gtaaaagcctccttcaatctccttgcttatgcacgattagcttatggtgtcatatcatca
catcacatcatctctggattgcttggcacttccataggattatggcatatcacattacgt
ccattagcatacctctacaatctattaagcatgggaaaggttgagtccattctttcaagt

agtattacagctgtattcttcactgcattccttatttcagcccttatgtggtatggctct
gcacacaccacacaagaactcttcggtcctactagatactcatgggacaatgcttattac
tctcttgatatcagaacctcttgctcaaatgcctggaacactcttccagacaagttagtc
ctttatgattatattggatctaaccctgctaaaggtggcttatttcgttcaggaccaatg
cttaaagctgatggtcttgttcaaaactggttgggccatgcatgcttctccatgggtaca
ttatccttaagcatcagaaggatgcctgctttctttgaaaccttcccagtaatcctcatt
gaccaaacaagtacagtgagagcagacattgctttcagacggtccacatctacttatagc
atggaagaatcccaaatacaagtatacttctctggtggttgtttaaatggtacagaatat
tctacaccttcccttgtaaaagcttatgcaagaaaagctcagtttggtcagatatttaca
tttgataaaaagacatccagattgactgatggtgtatttagaacatctgctcgaggttgg
tattcattctcacacatagctttagctttctcctttttctttggccatttgtggcatgct
agtcgtgctatcttccaagacatttggacaggtgtaacatttgaatcacaagcaaaacaa
gaatatggtagaaatgaaaagctaggagataagacatcttcaactaaatccattgtttaa
>Irm2_psbB
atgcaaataactgatgctttaccttggtttagggtacatatagttattcttaatgatcca
ggacgtctcattagctcacatattatgcacacagctttagtagcaggttggtcagctctc
atgcttttatatgagcttatcaccatagatcctacagatcctgtgtataatccaatttgg
agacaagcagcttacacactcccatttatctcacgtattggtgttattcgctctcttttt
agttggtcacttggcatagatcctacatccaatctaatctggacttatgaaacaatgaac
atagcccatattctattatctggtttattaattcttgcatcattttggcattgggcatat
tgggacttagatctattcttcacctcaacattaacactagaccttaatcaaatcctcagt
attcacctcacactagcatcatctctctgtctgggatttggtttagctcatctaactggt
tttcttggtccaggaatgtggactagtgattccctcaatcttgtaggctctattagattc
gtaaaagcctccttcaatctccttgcttatgcacgattagcttatggtgtcatatcatca
catcacatcatctctggattgcttggcacttccataggattatggcatatcacattacgt
ccattagcatacctctacaatctattaagcatgggaaaggttgagtccattctttcaagt
agtattacagctgtattcttcactgcattccttatttcagcccttatgtggtatggctct
gcacacaccacacaagaactcttcggtcctactagatactcatgggacaatgcttattac
tctcttgatatcagaacctcttgctcaaatgcctggaacactcttccagacaagttagtc
ctttatgattatattggatctaaccctgctaaaggtggcttatttcgttcaggaccaatg
cttaaagctgatggtcttgttcaaaactggttgggccatgcatgcttctccatgggtaca
ttatccttaagcatcagaaggatgcctgctttctttgaaaccttcccagtaatcctcatt
gaccaaacaagtacagtgagagcagacattgctttcagacggtccacatctacttatagc
atggaagaatcccaaatacaagtatacttctctggtggttgtttaaatggtacagaatat
tctacaccttcccttgtaaaagcttatgcaagaaaagctcagtttggtcagatatttaca
tttgataaaaagacatccagattgactgatggtgtatttagaacatctgctcgaggttgg
tattcattctcacacatagctttagctttctcctttttctttggccatttgtggcatgct
agtcgtgctatcttccaagacatttggacaggtgtaacatttgaatcacaagcaaaacaa
gaatatggtagaaatgaaaagctaggagataagacatcttcaactaaatccattgtttaa
>Irm21_psbB
atgcaaataactgatgctttaccttggtttagggtacatatagttattcttaatgatcca
ggacgtctcattagctcacatattatgcacacagctttagtagcaggttggtcagctctc
atgcttttatatgagcttatcaccatagatcctacagatcctgtgtataatccaatttgg
agacaagcagcttacacactcccatttatctctcgtatcggtgttattcgctctcttttt
agttggtcacttggcatagatcctacatccaatctaatctggacttatgaaacaatgaac
atagcccatattctattatctggtttattaattcttgcatcattttggcattgggcatat
tgggacttagatctattcttcacctcaacattaacactagaccttaatcaaatcctcagt
attcacctcacactagcatcatctctctgtctgggatttggtttagctcatctaactggt
tttcttggtccaggaatgtggactagtgattccctcaatcttgtaggctctattagattc
gtaaaagcctccttcaatctccttgcttatgcacgattagcttatggtgtcatatcatca
catcacatcatctctggattgcttggcacttccataggattatggcatatcacattacgt
ccattagcatacctctacaatctattaagcatgggaaaggttgagtccattctttcaagt
agtattacagctgtattcttcactgcattccttatttcagccctgatgtggtatggttct
gcacacaccacacaagaactcttcggtcctactagatactcatgggacaatgcttattac
tctcttgatatcagaacctcttgctcaaatgcctggaacactcttccagacaagttagtc
ctttatgattatattggatctaaccctgctaaaggtggcttatttcgttcaggaccaatg
cttaaagctgatggtcttgttcaaaactggttgggccatgcatgcttctccatgggtaca

ttatccttaagcatcagaaggatgcctgctttctttgaaaccttcccagtaatcctcatt
gaccaaacaagtacagtgagagcagacattgctttcagacggtccacatctacttatagc
atggaagaatcccaaatacaagtatacttctctggtggttgtttaaatggtacagaatat
tctacaccttcccttgtaaaagcttatgcaagaaaagctcagtttggtcagatatttaca
tttgataaaaagacatccagattgactgatggtgtatttagaacatctgctcgaggttgg
tattcattctcacacatagctttagctttctccttttcttggccatttgtggcatgct
agtcgtgctatcttccaagacatttggacaggtgtaacatttgaatcacaagcaaaacaa
gaatatggtagaaatgaaaagctaggagataagacatcttcaactaaatccattgtttaa
>Irm22_psbB
atgcaaataactgatgctttaccttggtttagggtacatatagttattcttaatgatcca
ggacgtctcattagctctcacattatgcatacagctttagtagctgggtggtcagctctc
atgcttttatatgagcttatcaccatagatcctacagatcctgtgtataatccaatttgg
agacaagcagcttacacactcccatttatctctcgtatcggtgttattcgctctcttttc
agttggtcacttggcatagatcctacatccaatctaatctggacttatgaaacaatgaac
atagcccatattctattatctggtttattaattcttgcatcattttggcattgggcatat
tgggacttagatctattcttcacctcaacattaacactagaccttaatcaaatcctcagt
attcacctcacactagcatcatctctctgtctgggatttggtttagctcatctaactggt
tttcttggtccaggaatgtggactagtgattccctcaatcttgtaggctctattagattc
gtaaaagcctccttcaatctccttgcttatgcacgattagcttatggtgtcatatcatca
catcacatcatctctggattgcttggcacttccataggattatggcatatcacattacgt
ccattagcatacctctacaatctattaagcatgggaaaggttgagtccattctttcaagt
agtattacagctgtattcttcactgcattccttatttcagccctgatgtggtatggttct
gcacataccacacaagaactattcggtcctactagatactcatgggacaatgcttattac
tctcttgatatcagaacctcttgctcaaatgcctggaacactcttccagacaagttagta
ctttatgattatattggatctaaccctgctaaaggtggcttatttcgttcaggaccaatg
cttaaagctgatggtcttgttcaaaactggttgggccatgcatgtttctccatgggtaca
ttatccttaagcatcagaaggatgcctgctttctttgaaaccttcccagtaatcctcatt
gaccaaacaagtacagtgagagcagacattgctttcagacggtccacatctacttatagc
atggaagaatcccaaatacaagtatacttctctggtggttgtttaaatggtacagaatat
tctacaccttcccttgtaaaagcttatgcaagaaaagctcagtttggtcagatatttaca
tttgataaaaagacatccagattgactgatggtgtatttagaacatctgctcgaggttgg
tattcattctcacacatagctttagctttctccttttcttggccatttgtggcatgct
agtcgtgctatcttccaagacatttggacaggtgtaacatttgaatcacaagcaaaacaa
gaatatggtagaaatgaaaagctaggagataagacatcttcaactaaatccattgtttaa
>Irm23_psbB
atgcaaataactgatgctttaccttggtttagggtacatatagttattcttaatgatcca
ggacgtctcattagctcacatattatgcacacagctttagtagcaggttggtcagctctc
atgcttttatatgagcttatcaccatagatcctacagatcctgtgtataatccaatttgg
agacaagcagcttacacactcccatttatctctcgtatcggtgttattcgctctcttttc
agttggtcacttggcatagatcctacatccaatctaatctggacttatgaaacaatgaac
atagcccatattctattatctggtttattaattcttgcatcattttggcattgggcatat
tgggacttagatctattcttcacctcaacattaacactagaccttaatcaaatcctcagt
attcacctcacactagcatcatctctctgtctgggatttggtttagctcatctaactggt
tttcttggtccaggaatgtggactagtgattccctcaatcttgtaggctctattagattc
gtaaaagcctccttcaatctccttgcttatgcacgattagcttatggtgtcatatcatca
catcacatcatctctggattgcttggcacttccataggattatggcatatcacattacgt
ccattagcatacctctacaatctattaagcatgggaaaggttgagtccattctttcaagt
agtattacagctgtattcttcactgcattccttatttcagcccttatgtggtatggctct
gcacacaccacacaagaactcttcggtcctactagatactcatgggacaatgcttattac
tctcttgatatcagaacctcttgctcaaatgcctggaacactcttccagacaagttagtc
ctttatgattatattggatctaaccctgctaaaggtggcttatttcgttcaggaccaatg
cttaaagctgatggtcttgttcaaaactggttgggccatgcatgcttctccatgggtaca
ttatccttaagcatcagaaggatgcctgctttctttgaaaccttcccagtaatcctcatt
gaccaaacaagtacagtgagagcagacattgcttttagacggtccacatctacttatagc
atggaagaatcccaaatacaagtatacttctctggtggttgtttaaatggtacagaatat
tctacaccttcccttgtaaaagcttatgcaagaaaagctcagtttggtcagatatttaca
tttgataaaaagacatccagattgactgatggtgtatttagaacatctgctcgaggttgg

tattcattctcacacatagctttagctttctcctttttctttggccatttgtggcatgct
agtcgtgctatcttccaagacatttggacaggtgtaacatttgaatcacaagcaaaacaa
gaatatggtagaaatgaaaagctaggagataagacatcttcaactaaatccattgtttaa
>Irm24_psbB
atgcaaataactgatgctttaccttggtttagggtacatatagttattcttaatgatcca
ggacgtctcattagctcacatattatgcacacagctttagtagcaggttggtcagctctc
atgcttttatatgagcttatcaccatagatcctacagatcctgtgtataatccaatttgg
agacaagcagcttacacactcccatttatctctcgtatcggtgttattcgctctcttttc
agttggtcacttggcatagatcctacatccaatctaatctggacttatgaaacaatgaac
atagcccatattctattatctggtttattaattcttgcatcattttggcattgggcatat
tgggacttagatctattcttcacctcaacattaacactagaccttaatcaaatcctcagt
attcacctcacactagcatcatctctctgtctgggatttggtttagctcatctaacaggt
tttcttggtccaggaatgtggactagtgattccctcaatcttgtaggctctattagattc
gtaaaagcctccttcaatatccttcttatgcacgattagcttatggtgtcatatcatca
catcacatcatctctggattgcttggcacttccataggattatggcatatcacattacgt
ccattagcatacctctacaatctattaagcatgggaaaggttgagtccattctttcaagt
agtattacagctgtattcttcactgcattccttatttcagcccttatgtggtatggctct
gcacacaccacacaagaactattcggtcctactagatactcatgggacaatgcttattac
tctcttgatatcagaacctcttgctcaaatgcctggaacactcttccagacaagttagta
ctttatgattatattggctctaaccctgctaaaggtggcttatttcgttcaggaccaatg
cttaaagctgatggtcttgttcaaaactggttaggtcatgcatgtttctccatgggtaca
ttatccttaagcatcagaaggatgcctgctttctttgaaaccttcccagtaatcctcatt
gaccaaacaagtacagtgagagcagacattgcttttagacggtccacatctacttatagc
atggaagaatcccaaatacaagtatacttctctggtggttgtttaaatggtacagaatat
tctacaccttcccttgtaaaagcttatgcaagaaaagctcagtttggtcagatatttaca
tttgataaaaagacatccagattgactgatggtgtatttagaacatctgctcgaggttgg
tattcattctcacacatagctttagctttctcctttttctttggccatttgtggcatgct
agtcgtgctatcttcaaagacatttggacaggtgtaacatttgaatctcaagcaaaacaa
gaatatggtagaaatgaaaagctaggagataagacatcttcaactaaatccattgtttaa
>Irm25_psbB
atgcaaataactgatgctttaccttggtttagggtacatatagttattcttaatgatcca
ggacgtctcattagctctcacattatgcatacagctttagtagcaggttggtcagctctc
atgcttttatatgagcttatcaccatagatcctacagatcctgtgtataatccaatttgg
agacaagcagcttacacactcccatttatctcacgtattggtgttattcgctctcttttc
agttggtcacttggcatagatcctacatccaatctaatctggacttatgaaacaatgaac
atagcccatattctattatctggtttattaattcttgcatcattttggcattgggcatat
tgggacttagatctattcttcacctcaacattaacactagaccttaatcaaatcctcagt
attcacctcacactagcatcatctctctgtctgggatttggtttagctcatctaacaggt
tttcttggtccaggaatgtggactagtgattccctcaatcttgtaggctctattagattc
gtaaaagcctccttcaatctccttgcttatgcacgattagcttatggtgtcatatcatca
catcacatcatctctggattgcttggcacttccataggattatggcatatcacattacgt
ccattagcatacctctacaatctattaagcatgggaaaggttgagtccattctttcaagt
agtattacagctgtattcttcactgcattccttatttcagccctgatgtggtatggttct
gcacataccacacaagaactattcggtcctactagatactcatgggacaatgcttattac
tctcttgatatcagaacctcttgctcaaatgcctggaacactcttccagacaagttagtc
ctttatgattatattggatctaaccctgctaaaggtggcttatttcgttcaggaccaatg
cttaaagctgatggtcttgttcaaaactggttgggccatgcatgcttctccatgggtaca
ttatccttaagcatcagaaggatgcctgctttctttgaaaccttcccagtaatcctcatt
gaccaaacaagtacagtgagagcagacattgcttttagacggtccacatctacttatagc
atggaagaatcccaaatacaagtatacttctctggtggttgtttaaatggtacagaatat
tctacaccttcccttgtaaaagcttatgcaagaaaagctcagtttggtcagatatttaca
tttgataaaaagacatccagattgactgatggtgtatttagaacatcttctcgaggttgg
tattcattctcacacataggtttagctttcctattcttctttggccatttgtggcatgct
agtcgtgctatcttccaagacatttggacaggtgtaacatttgaatcacaagcaaaacaa
gaatatggtagaaatgaaaagctaggagataagacatcttcaactaaatccattgtttaa
>Irm26_psbB
atgcaaataactgatgctttaccttggtttagggtacatatagttattcttaatgatcca

ggacgtctcattagctcacatattatgcacacagctttagtagcaggttggtcagctctc
atgcttttatatgagcttatcaccatagatcctacagatcctgtgtataatccaatttgg
agacaagcagcttacacactcccatttatctcacgtattggtgttattcgctctcttttt
agttggtcacttggcatagatcctacatccaatctaatctggacttatgaaacaatgaac
atagcccatattctattatctggtttattaattcttgcatcattttggcattgggcatat
tgggacttagatctattcttcacctcaacattaacactagaccttaatcaaatcctcagt
attcacctcacactagcatcatctctctgtctgggatttggtttagctcatctaactggt
tttcttggtccaggaatgtggactagtgattccctcaatcttgtaggctctattagattc
gtaaaagcctccttcaatctccttgcttatgcacgattagcttatggtgtcatatcatca
catcacatcatctctggattgcttggcacttccataggattatggcatatcacattacgt
ccattagcatacctctacaatctattaagcatgggaaaggttgagtccattctttcaagt
agtattacagctgtattcttcactgcattccttatttcagccctgatgtggtatggttct
gcacataccacacaagaactcttcggtcctactagatactcatgggacaatgcttattac
tctcttgatatcagaacctcttgctcaaatgcctggaacactcttccagacaagttagta
ctttatgattatattggctctaaccctgctaaaggtggcttatttcgttcaggaccaatg
cttaaagctgatggtcttgttcaaaactggttgggccatgcatgcttctccatgggtaca
ttatccttaagcatcagaaggatgcctgctttctttgaaaccttcccagtaatcctcatt
gaccaaacaagtacagtgagagcagacattgctttcagacggtccacatctacttatagc
atggaagaatcccaaatacaagtatacttctctggtggttgtttaaatggtacagaatat
tctacaccttcccttgtaaaagcttatgcaagaaaagctcagtttggtcagatatttaca
tttgataaaaagacatccagattgactgatggtgtatttagaacatctgctcgaggttgg
tattcattctcacacataggtttagctttcctattcttctttggccatttgtggcatgct
agtcgtgctatcttccaagacatttggacaggtgtaacatttgaatcacaagcaaaacaa
gaatatggtagaaatgaaaagctaggagataagacatcttcaactaaatccattgtttaa
>Irm27_psbB
atgcaaataactgatgctttaccttggtttagagttcatatagttgttcttaatgatcca
ggacgtctcattagttcacacattatgcatacagctttagtagctgggtggtcagctctc
atgcttttatatgagcttatcaccatagatcctacagatcctgtgtataatccaatttgg
agacaagcagcttacacactcccatttatctctcgtatcggtgttattcgctctcttttc
agttggtcacttggcatagatcctacatccaatctaatctggacttatgaaacaatgaac
atagcccatattctattatctggtttattaattcttgcatcattttggcattgggcatat
tgggacttagatctattcttcacctcaacattaacactagaccttaatcaaatcctcagt
attcacctcacactagcatcatctctctgtctgggatttggtttagctcatctaacaggt
tttcttggtccaggaatgtggactagtgattccctcaatcttgtaggctctattagattc
gtaaaagcctccttcaatatcctttcttatgcacgattagcttatggtgtcatatcatca
catcacatcatctctggattgcttggcacttctataggattatggcatatcacattacgt
ccattagcatatctctacaatctattaagcatgggaaaggttgagtccattctttcaagt
agtattacagctgtattcttcactgcattccttatttcagccctgatgtggtatggttct
gcacataccacacaagaactattcggtcctactagatactcatgggacaatgcttattac
tctcttgatatcagaacctcttgctcaaatgcctggaacactcttccagacaagttagta
ctttatgattatattggctctaaccctgctaaaggtggcttatttcgttcaggaccaatg
cttaaagctgatggtcttgttcaaaactggttaggtcatgcatgtttctccatgggtaca
ttatccttaagcatcagaaggatgcctgctttctttgaaaccttcccagtaatcctcatt
gaccaaacaagtacagtgagagcagacattgcttttagacggtccacatctacttatagc
atggaagaatcccaaatacaagtatacttctctggtggttgtttaaatggtacagaatat
tctacaccttcccttgtaaaagcttatgcaagaaaagctcagtttggtcagatatttaca
tttgataaaaagacatccagattgactgatggtgtatttagaacatcttctcgaggttgg
tattcattctcacacatagctttagctttcctcttcttcttcggtcatctgtggcatgct
agtcgtgctatattccaagacatttggacaggtgtaacatttgaatctcaagcaaaacaa
gaatatggtagaaatgaaaagctaggagataagacatcttcaactaaatccattgtttaa
>Irm3_psbB
atgcaaataactgatgctttaccttggtttagagttcatatagttgttcttaatgatcca
ggacgtctcattagttcacacattatgcacaccgctttagtagctgggtggtcggctcta
atgcttttatacgagcttatcaccatagatcctacagaccctgtgtataacccaatttgg
agacaagccgcttatacactcccatttatctcacgtattggtgttattcgctctcttttc
agttggtcacttggcatagatcctacatccaatctaatatggacttatgaaacaattaac
acagcccacattctattatcaggtttattaatccttgcagcattttggcattgggcatat

tgggacttagatctattcttcaccccaagattaacactagaccttaatcaaatcttcagt
attcatctcacacttgcatcctctctctgtttaggatatggtttagctcatttaactggc
ttttttggtccaggaatgtggactagtgattctcttaatcttgttggctctgttagattc
gtaaaagcctcctttaatctccttgcttatgcgagattgtcttatggtgtcatatcatca
catcacatcatctctggattgcttggcacttctataggattatggcatatcacattacgt
ccattagcatatctctacaatctattaagtatgggaaagattgagtccattctttcaagt
agtattacagctgtattcttcactgcattccttatatcagctcttatgtggtatggatct
gcacacaccacacaagaactcttcggtcctactagatactcatgggacaatgcttattac
tctcttgatatcagaacctcttgctcaaatgcctggaacactctcccagataagttagtc
ctttatgattatattggatctaaccctgctaaaggtggtttattccgttcaggacctatg
cttaaagctgatggacttgttcaaaactggttggggcacgcatgcttctcgatgggtaca
ttatccctaagcatcagaaggatgcctgctttctttgaaagcttcccagtaatcctcatt
gaccaaacaagtacagtgagagcagacattgctttcagacgctctacatccacttatagc
atggaagaatcacaaatacaagtatacttctctggtggttgtttaaatggtacagaatat
tctacaccttctctagttaaagcttatgcaagaaaagctcagtttggtcaaatatttaca
tttgataaaaagacatctagattgactgatggtgtatttagaacatctgctcgaggttgg
tattcattctcacacatagctttagctttcctcttcttcttcggtcatctgtggcatgct
agtcgtgctatattccaagacatttggacaggtgtaagctttgaatcacaagcaaagcaa
gaatatggtagaaatgaaaagctaggagataagacatcttcaactaaatccattgtttaa
>Irm4_psbB
atgcaaataactgatgctttaccttggtttagggtacatatagttattcttaatgatcca
ggacgtctcattagttcacatattatgcacaccgctttagtagctgggtggtcggctcta
atgcttttatatgagcttatcaccatagatcctacagatcctgtgtataatccaatttgg
agacaagcagcttacacactcccatttatctcacgtattggtgttattcgctctcttttt
aattggtcaattggtatagatcctacttctaatctaatctggacttatgaaacaatgaac
atagcccatattctattatcaggtttattaatccttgcagcattttggcattgggcatat
tgggacttagatctattcttcaccccaagattaacactagaccttaatcaaatcttcagt
attcatctcacacttgcatcctctctctgtttaggatatggtttagctcatttaactggc
tttcttggtccaggaatgtggactagtgattccctcaatcttgtaggctctattagattc
gtaaaagcctccttcaatctccttgcttatgcacgattagcttatggtgtcatatcatca
catcacatcatctctggattgcttggcacttctataggattatggcatatcacattacgt
ccattagcatatctctacaatctattaagtatgggaaaggttgagtccattctttcaagt
agtattacagctgtattcttcactgcattccttatttcagccctgatgtggtatggttct
gcacataccacacaagaactattcggtcctactagatactcatgggacaatgcttattac
tctcttgatatcagaacctcttgctcaaatgcctggaacactctcccagataagttagtc
ctttatgattatattggatctaaccctgctaaaggtggtttattccgttcaggacctatg
cttaaagctgatggacttgttcaaaactggttgggccatgcatgcttctccatgggtaca
ttatccttaagcatcagaaggatgcctgctttctttgaaagcttcccagtaatcctcatt
gaccaaacaagtacagtgagagcagacattgctttcagacgctctacatccacttatagc
atggaagaatcacaaatacaagtatacttctctggtggttgtttaaatggtacagaatat
tctacaccttcccttgtaaaagcttatgcaagaaaagctcagtttggtcagatatttaca
tttgataaaaagacatccagattgactgatggtgtatttagaacatctgctcgaggttgg
tattcattctcacacatagctttagctttctcctttttcttcggccatctgtggcatgct
agtcgtgctatattccaagacatttggacaggtgtaacatttgaatcacaagcaaagcaa
gaatatggcgcaaatgaaaagttaggagacaagacctcttcaactaaatccattgtttaa
>Irm5_psbB
atgcaaataactgatgctttaccttggtttagggtacatatagttattcttaatgatcca
ggacgtctcattagctcacatattatgcacacagctttagtagcaggttggtcagctctc
atgcttttatatgagcttatcaccatagatcctacagatcctgtgtataatccaatttgg
agacaagcagcttacacactcccatttatctcacgtattggtgttattcgctctcttttt
agttggtcacttggcatagatcctacatccaatctaatctggacttatgaaacaatgaac
atagcccatattctattatctggtttattaattcttgcatcattttggcattgggcatat
tgggacttagatctattcttcacctcaacattaacactagaccttaatcaaatcctcagt
attcacctcacactagcatcatctctctgtctgggatttggtttagctcatctaacaggt
tttcttggtccaggaatgtggactagtgattccctcaatcttgtaggctctattagattc
gtaaaagcctccttcaatctccttgcttatgcacgattagcttatggtgtcatatcatca
catcacatcatctctggattgcttggcacttccataggattatggcatatcacattacgt

ccattagcatacctctacaatctattaagcatgggaaaggttgagtccattctttcaagt
agtattacagctgtattcttcactgcattccttatttcagcccttatgtggtatggctct
gcacacaccacacaagaactcttcggtcctactagatactcatgggacaatgcttattac
tctcttgatatcagaacctcttgctcaaatgcctggaacactcttccagacaagttagtc
ctttatgattatattggatctaaccctgctaaaggtggcttatttcgttcaggaccaatg
cttaaagctgatggtcttgttcaaaactggttgggccatgcatgcttctccatgggtaca
ttatccttaagcatcagaaggatgcctgctttctttgaaaccttcccagtaatcctcatt
gaccaaacaagtacagtgagagcagacattgctttcagacggtccacatctacttatagc
atggaagaatcccaaatacaagtatacttctctggtggttgtttaaatggtacagaatat
tctacaccttcccttgtaaaagcttatgcaagaaaagctcagtttggtcagatatttaca
tttgataaaaagacatccagattgactgatggtgtatttagaacatctgctcgaggttgg
tattcattctcacacatagctttagctttctcctttttctttggccatttgtggcatgct
agtcgtgctatcttccaagacatttggacaggtgtaacatttgaatcacaagcaaaacaa
gaatatggtagaaatgaaaagctaggagataagacatcttcaactaaatccattgtttaa
>Irm7_psbB
atgcaaataactgatgctttaccttggtttagggtacatatagttattcttaatgatcca
ggacgtctcattagctcacatattatgcacacagctttagtagctgggtggtcagctctc
atgcttttatatgagcttatcaccatagatcctacagatcctgtgtataatccaatttgg
agacaagcagcttacacactcccatttatctctcgtatcggtgttattcgctctcttttc
agttggtcacttggcatagatcctacatccaatctaatctggacttatgaaacaatgaac
atagcccatattctattatctggtttattaattcttgcatcattttggcattgggcatat
tgggacttagatctattcttcacctcaacattaacactagaccttaatcaaatcctcagt
attcacctcacactagcatcatctctctgtctgggatttggtttagctcatctaactggt
tttcttggtccaggaatgtggactagtgattccctcaatcttgtaggctctattagattc
gtaaaagcctccttcaatatcctttcttatgcacgattagcttatggtgtcatatcatca
catcacatcatctctggattgcttggcacttccataggattatggcatatcacattacgt
ccattagcatacctctacaatctattaagcatgggaaaggttgagtccattctttcaagt
agtattacagctgtattcttcactgcattccttatttcagcccttatgtggtatggctct
gcacacaccacacaagaactcttcggtcctactagatactcatgggacaatgcttattac
tctcttgatatcagaacctcttgctcaaatgcctggaacactcttccagacaagttagtc
ctttatgattatattggatctaaccctgctaaaggtggcttatttcgttcaggaccaatg
cttaaagctgatggtcttgttcaaaactggttaggtcatgcatgtttctccatgggtaca
ttatccttaagcatcagaaggatgcctgctttctttgaaaccttcccagtaatcctcatt
gaccaaacaagtacagtgagagcagacattgcttttagacggtccacatctacttatagc
atggaagaatcccaaatacaagtatacttctctggtggttgtttaaatggtacagaatat
tctacaccttcccttgtaaaagcttatgcaagaaaagctcagtttggtcagatatttaca
tttgataaaaagacatccagattgactgatggtgtatttagaacatcttctcgaggttgg
tattcattctcacacataggtttagctttcctattcttctttggccatttgtggcatgct
agtcgtgctatcttcaaagacatttggacaggtgtaacatttgaatctcaagcaaaacaa
gaatatggtagaaatgaaaagctaggagataagacatcttcaactaaatccattgtttaa
>Irm9_psbB
atgcaaataactgatgctttaccttggtttagagttcatatagttgttcttaatgatcca
ggacgtctcattagctctcacattatgcatacagctttagtagctgggtggtcggctcta
atgcttttatacgagcttatcaccatagatcctacagaccctgtgtataacccaatttgg
agacaagccgcttatacactcccatttatctcacgtattggtgttattcgctctcttttt
aattggtcaattggtatagatcctacttctaatctaatatggacttatgaaacaattaac
acagcccacattctattatcaggtttattaatccttgcagcattttggcattgggcatat
tgggacttagatctattcttcaccccaagattaacactagaccttaatcaaatcctcagt
attcacctcacactagcatcatctctctgtctgggatttggtttagctcatctaacaggt
tttcttggtccaggaatgtggactagtgattccctcaatcttgtaggctctattagattc
gtaaaagcctccttcaatatcctttcttatgcacgattagcttatggtgtcatatcatca
catcacatcatctctggattgcttggcacttccataggattatggcatatcacattacgt
ccattagcatacctctacaatctattaagcatgggaaaggttgagtccattctttcaagt
agtattacagctgtattcttcactgcattccttatttcagccctgatgtggtatggttct
gcacataccacacaagaactcttcggtcctactagatactcatgggacaatgcttattac
tctcttgatatcagaacctcttgctcaaatgcctggaacactctcccagataagttagta
ctttatgattatattggctctaaccctgctaaaggtggcttatttcgttcaggaccaatg

cttaaagctgatggtcttgttcaaaactggttaggtcatgcatgtttctccatgggtaca
ttatccttaagcatcagaaggatgcctgctttctttgaaaccttcccagtaatcctcatt
gaccaaacaagtacagtgagagcagacattgctttcagacgctctacatccacttatagc
atggaagaatcacaaatacaagtatacttctctggtggttgtttaaatggtacagaatat
tctacaccttcccttgtaaaagcttatgcaagaaaagctcagtttggtcagatatttaca
tttgataaaaagacatccagattgactgatggtgtatttagaacatcttctcgaggttgg
tattcattctcacacataggtttagctttcctcttcttcttcggtcatctgtggcatgct
agtcgtgctatattccaagacatttggacaggtgtaagctttgaatcacaagcaaagcaa
gaatatggtagaaatgaaaagctaggagataagacatcttcaactaaatccattgtttaa
>Isy12_psbB
atgcaaataactgatgctttaccttggtttagggtacatatagttattcttaatgatcca
ggacgtctcattagctcacatattatgcacacagctttagtagcaggttggtcagctctc
atgcttttatatgagcttatcaccatagatcctacagatcctgtgtataatccaatttgg
agacaagcagcttacacactcccatttatctcacgtattggtgttattcgctctcttttt
agttggtcacttggcatagatcctacatccaatctaatctggacttatgaaacaatgaac
atagcccatattctattatctggtttattaattcttgcatcattttggcattgggcatat
tgggacttagatctattcttcacctcaacattaacactagaccttaatcaaatcctcagt
attcacctcacactagcatcatctctctgtctgggatttggtttagctcatctaacaggt
tttcttggtccaggaatgtggactagtgattccctcaatcttgtaggctctattagattc
gtaaaagcctccttcaatatcctttcttatgcacgattagcttatggtgtcatatcatca
catcacatcatctctggattgcttggcacttccataggattatggcatatcacattacgt
ccattagcatacctctacaatctattaagcatgggaaaggttgagtccattctttcaagt
agtattacagctgtattcttcactgcattccttattttagcccttatgtggtatggctct
gcacacaccacacaagaactcttcggtcctactagatactcatgggacaatgcttattac
tctcttgatatcagaacctcttgctcaaatgcctggaacactcttccagacaagttagtc
ctttatgattatattggatctaaccctgctaaaggtggcttatttcgttcaggaccaatg
cttaaagctgatggtcttgttcaaaactggttgggccatgcatgcttctccatgggtaca
ttatccttaagcatcagaaggatgcctgctttctttgaaaccttcccagtaatcctcatt
gaccaaacaagtacagtgagagcagacattgctttcagacggtccacatctacttatagc
atggaagaatcccaaatacaagtatacttctctggtggttgtttaaatggtacagaatat
tctacaccttcccttgtaaaagcttatgcaagaaaagctcagtttggtcagatatttaca
tttgataaaaagacatccagattgactgatggtgtatttagaacatctgctcgaggttgg
tattcattctcacacatagctttagctttctcctttttctttggccatttgtggcatgct
agtcgtgctatcttccaagacatttggacaggtgtaacatttgaatcacaagcaaaacaa
gaatatggtagaaatgaaaagctaggagataagacatcttcaactaaatccattgtttaa
>Isy15_psbB
atgcaaataactgatgctttaccttggtttagagttcatatagttgttcttaatgatcca
ggacgtctcattagttcacacattatgcacaccgctttagtagctgggtggtcggctcta
atgcttttatacgagcttatcaccatagatcctacagaccctgtgtataacccaatttgg
agacaagccgcttatacactcccatttatctctcgtatcggtgttattcgctctcttttc
agttggtcacttggcatagatcctacatccaatctaatctggacttatgaaacaattaac
acagcccacattctattatcaggtttattaatccttgcagcattttggcattgggcatat
tgggacttagatctattcttcaccccaagattaacactagaccttaatcaaatcttcagt
attcatctcacacttgcatcctctctctgtttaggatatggtttagctcatttaactggc
ttttttggtccaggaatgtggactagtgattctcttaatcttgttggctctgttagattc
gtaaaagcctcctttaatctccttgcttatgcgagattgtcttatggtgttataccagct
catcatataatctctggattgcttggcacttctataggattatggcatatcacattacgt
ccattagcatatctctacaatctattaagtatgggaaagattgagtccattctttcaagt
agtattacagctgtattcttcactgcattccttatatcagctcttatgtggtatggatct
gcacacaccacacaagaactcttcggtcctactagatactcatgggacaatgcttattac
tctcttgatatcagaacctcttgctcaaatgcctggaacactctcccagataagttagtc
ctttatgattatattggatctaaccctgctaaaggtggtttattccgttcaggacctatg
cttaaagctgatggacttgttcaaaactggttggggcacgcatgcttctcgatgggtaca
ttatccctaagcatcagaaggatgcctgctttctttgaaagcttcccagtaatcctcatt
gaccaaacaagtacagtgagagcagacattgctttcagacggtccacatctacttatagc
atggaagaatcacaaatacaagtatacttctctggtggttgtttaaatggtacagaatat
tctacaccttctctagttaaagcttatgcaagaaaagctcagtttggtcaaatatttaca

tttgataaaaagacatccagattgactgatggtgtatttagaacatctgctcgaggttgg
tattcattctcacacatagctttagctttcctcttcttcttcggtcatctgtggcatgct
agtcgtgctatattccaagacatttggacaggtgtaagctttgaatcacaagcaaagcaa
gaatatggtagaaatgaaaagctaggagataagacatcttcaactaaatccattgtttaa
>Isy16_psbB
atgcaaataactgctgttttaccttggtttagggtacatatagttattcttaatgatcca
ggacgtctcattagctcacatattatgcacacagctttagtagcaggttggtcagctctc
atgcttttatatgagcttatcaccatagatcctacagatcctgtgtataatccaatttgg
agacaagcagcttacacactcccatttatctcacgtattggtgttattcgctctcttttt
agttggtcacttggcatagatcctacatccaatctaatctggacttatgaaacaatgaac
atagcccatattctattatctggtttattaattcttgcatcattttggcattgggcatat
tgggacttagatctattcttcacctcaacattaacactagaccttaatcaaatcctcagt
attcacctcacactagcatcatctctctgtctgggatttggtttagctcatctaactggt
tttcttggtccaggaatgtggactagtgattccctcaatcttgtaggctctattagattc
gtaaaagcctccttcaatctccttgcttatgcacgattagcttatggtgtcatatcatca
catcacatcatctctggattgcttggcacttccataggattatggcatatcacattacgt
ccattagcatacctctacaatctattaagcatgggaaaggttgagtccattctttcaagt
agtattacagctgtattcttcactgcattccttatttcagcccttatgtggtatggctct
gcacacaccacacaagaactcttcggtcctactagatactcatgggacaatgcttattac
tctcttgatatcagaacctcttgctcaaatgcctggaacactcttccagacaagttagtc
ctttatgattatattggatctaaccctgctaaaggtggcttatttcgttcaggaccaatg
cttaaagctgatggtcttgttcaaaactggttgggccatgcatgcttctccatgggtaca
ttatccttaagcatcagaaggatgcctgctttctttgaaaccttcccagtaatcctcatt
gaccaaacaagtacagtgagagcagacattgctttcagacggtccacatctacttatagc
atggaagaatcccaaatacaagtatacttctctggtggttgtttaaatggtacagaatat
tctacaccttcccttgtaaaagcttatgcaagaaaagctcagtttggtcagatatttaca
tttgataaaaagacatccagattgactgatggtgtatttagaacatctgctcgaggttgg
tattcattctcacacatagctttagctttctcctttttctttggccatttgtggcatgct
agtcgtgctatcttccaagacatttggacaggtgtaacatttgaatcacaagcaaaacaa
gaatatggtagaaatgaaaagctaggagataagacatcttcaactaaatccattgtttaa
>Isy17_psbB
atgcaaataactgatgctttaccttggtttagggtacatatagttattcttaatgatcca
ggacgtctcattagctcacatattatgcacacagctttagtagcaggttggtcagctctc
atgcttttatatgagcttatcaccatagatcctacagatcctgtgtataatccaatttgg
agacaagcagcttacacactcccatttatctcacgtattggtgttattcgctctcttttt
agttggtcacttggcatagatcctacatccaatctaatctggacttatgaaacaatgaac
atagcccatattctattatctggtttattaattcttgcatcattttggcattgggcatat
tgggacttagatctattcttcacctcaacattaacactagaccttaatcaaatcctcagt
attcacctcacactagcatcatctctctgtctgggatttggtttagctcatctaactggt
tttcttggtccaggaatgtggactagtgattccctcaatcttgtaggctctattagattc
gtaaaagcctccttcaatctccttgcttatgcacgattagcttatggtgtcatatcatca
catcacatcatctctggattgcttggcacttccataggattatggcatatcacattacgt
ccattagcatacctctacaatctattaagcatgggaaaggttgagtccattctttcaagt
agtattacagctgtattcttcactgcattccttatttcagcccttatgtggtatggctct
gcacacaccacacaagaactcttcggtcctactagatactcatgggacaatgcttattac
tctcttgatatcagaacctcttgctcaaatgcctggaacactcttccagacaagttagtc
ctttatgattatattggatctaaccctgctaaaggtggcttatttcgttcaggaccaatg
cttaaagctgatggtcttgttcaaaactggttgggccatgcatgcttctccatgggtaca
ttatccttaagcatcagaaggatgcctgctttctttgaaaccttcccagtaatcctcatt
gaccaaacaagtacagtgagagcagacattgctttcagacggtccacatctacttatagc
atggaagaatcccaaatacaagtatacttctctggtggttgtttaaatggtacagaatat
tctacaccttcccttgtaaaagcttatgcaagaaaagctcagtttggtcagatatttaca
tttgataaaaagacatccagattgactgatggtgtatttagaacatctgctcgaggttgg
tattcattctcacacatagctttagctttctcctttttctttggccatttgtggcatgct
agtcgtgctatcttccaagacatttggacaggtgtaacatttgaatcacaagcaaaacaa
gaatatggtagaaatgaaaagctaggagataagacatcttcaactaaatccattgtttaa
>Isy18_psbB

atgcaaataactgatgctttaccttggtttagggtacatatagttattcttaatgatcca
ggacgtctcattagctcacatattatgcacacagctttagtagcaggttggtcagctctc
atgcttttatatgagcttatcaccatagatcctacagatcctgtgtataatccaatttgg
agacaagcagcttacacactcccatttatctcacgtattggtgttattcgctctcttttt
agttggtcacttggcatagatcctacatccaatctaatctggacttatgaaacaatgaac
atagcccatattctattatctggtttattaattcttgcatcattttggcattgggcatat
tgggacttagatctattcttcacctcaacattaacactagaccttaatcaaatcctcagt
attcacctcacactagcatcatctctctgtctgggatttggtttagctcatctaacaggt
tttcttggtccaggaatgtggactagtgattccctcaatcttgtaggctctattagattc
gtaaaagcctccttcaatatcctttcttatgcactattagcttatggtgtcatatcatca
catcacatcatctctggattgcttggcacttccataggattatggcatatcacattacgt
ccattagcatacctctacaatctattaagcatgggaaaggttgagtccattctttcaagt
agtattacagctgtattcttcactgcattccttatttcagcccttatgtggtatggctct
gcacacaccacacaagaactcttcggtcctactagatactcatgggacaatgcttattac
tctcttgatatcagaacctcttgctcaaatgcctggaacactcttccagacaagttagtc
ctttatgattatattggatctaaccctgctaaaggtggcttatttcgttcaggaccaatg
cttaaagctgatggtcttgttcaaaactggttgggccatgcatgcttctccatgggtaca
ttatccttaagcatcagaaggatgcctgctttctttgaaaccttcccagtaatcctcatt
gaccaaacaagtacagtgagagcagacattgctttcagacggtccacatctacttatagc
atggaagaatcccaaatacaagtatacttctctggtggttgtttaaatggtacagaatat
tctacaccttcccttgtaaaagcttatgcaagaaaagctcagtttggtcagatatttaca
tttgataaaaagacatccagattgactgatggtgtatttagaacatctgctcgaggttgg
tattcattctcacacatagctttagctttctcctttttctttggccatttgtggcatgct
agtcgtgctatcttcaaagacatttggacaggtgtaacatttgaatctcaagcaaaacaa
gaatatggtagaaatgaaaagctaggagataagacatcttcaactaaatccattgtttaa
>Isy21_psbB
atgcaaataactgatgctttaccttggtttagggtacatatagttattcttaatgatcca
ggacgtctcattagctcacatattatgcacacagctttagtagcaggttggtcagctctc
atgcttttatatgagcttatcaccatagatcctacagatcctgtgtataatccaatttgg
agacaagcagcttacacactcccatttatctcacgtattggtgttattcgctctcttttc
agttggtcacttggcatagatcctacatccaatctaatctggacttatgaaa-aatgaac
atagcccatattctattatctggtttattaattcttgcatcattttggcattgggcatat
tgggacttagatctattcttcacctcaacattaacactagaccttaatcaaatcctcagt
attcacctcacactagcatcatctctctgtctgggatttggtttagctcatctaactggt
tttcttggtccaggaatgtggactagtgattccctcaatcttgtaggctctattagattc
gtaaaagcctccttcaatctccttgcttatgcacgattagcttatggtgtcatatcatca
catcacatcatctctggattgcttggcacttccataggattatggcatatcacattacgt
ccattagcatacctctacaatctattaagcatgggaaaggttgagtccattctttcaagt
agtattacagctgtattcttcactgcattccttatttcagcccttatgtggtatggctct
gcacacaccacacaagaactcttcggtcctactagatactcatgggacaatgcttattac
tctcttgatatcagaacctcttgctcaaatgcctggaacactcttccagacaagttagtc
ctttatgattatattggatctaaccctgctaaaggtggcttatttcgttcaggaccaatg
cttaaagctgatggtcttgttcaaaactggttgggccatgcatgcttctccatgggtaca
ttatccttaagcatcagaaggatgcctgctttctttgaaaccttcccagtaatcctcatt
gaccaaacaagtacagtgagagcagacattgctttcagacggtccacatctacttatagc
atggaagaatcccaaatacaagtatacttctctggtggttgtttaaatggtacagaatat
tctacaccttcccttgtaaaagcttatgcaagaaaagctcagtttggtcagatatttaca
tttgataaaaagacatccagattgactgatggtgtatttagaacatctgctcgaggttgg
tattcattctcacacatagctttagctttctcctttttctttggccatttgtggcatgct
agtcgtgctatcttccaagacatttggacaggtgtaacatttgaatcacaagcaaaacaa
gaatatggtagaaatgaaaagctaggagataagacatcttcaactaaatccattgtttaa
>Isy22_psbB
atgcaaataactgatgctttaccttggtttagggtacatatagttattcttaatgatcca
ggacgtctcattagctcacatattatgcacacagctttagtagcaggttggtcagctctc
atgcttttatatgagcttatcaccatagatcctacagatcctgtgtataatccaatttgg
agacaagcagcttacacactcccatttatctcacgtattggtgttattcgctctcttttt
agttggtcacttggcatagatcctacatccaatctaatctggacttatgaaacaatgaac

atagcccatattctattatctggtttattaattcttgcatcattttggcattgggcatat
tgggacttagatctattcttcacctcaacattaacactagaccttaatcaaatcctcagt
attcacctcacactagcatcatctctctgtctgggatttggtttagctcatctaactggt
tttcttggtccaggaatgtggactagtgattccctcaatcttgtaggctctattagattc
gtaaaagcctccttcaatatcctttcttatgcacgattagcttatggtgtcatatcatca
catcacatcatctctggattgcttggcacttccataggattatggcatatcacattacgt
ccattagcatacctctacaatctattaagcatgggaaaggttgagtccattctttcaagt
agtattacagctgtattcttcactgcattccttatttcagcccttatgtggtatggctct
gcacacaccacacaagaactcttcggtcctactagatactcatgggacaatgcttattac
tctcttgatatcagaacctcttgctcaaatgcctggaacactcttccagacaagttagtc
ctttatgattatattggatctaaccctgctaaaggtggcttatttcgttcaggaccaatg
cttaaagctgatggtcttgttcaaaactggttgggccatgcatgcttctccatgggtaca
ttatccttaagcatcagaaggatgcctgctttctttgaaaccttcccagtaatcctcatt
gaccaaacaagtacagtgagagcagacattgctttcagacggtccacatctacttatagc
atggaagaatcccaaatacaagtatacttctctggtggttgtttaaatggtacagaatat
tctacaccttcccttgtaaaagcttatgcaagaaaagctcagtttggtcagatatttaca
tttgataaaaagacatccagattgactgatggtgtatttagaacatcttctcgaggttgg
tattcattctcacacatagctttagctttctcctttttctttggccatttgtggcatgct
agtcgtgctatcttccaagacatttggacaggtgtaacatttgaatcacaagcaaaacaa
gaatatggtagaaatgaaaagctaggagataagacatcttcaactaaatccattgtttaa
>Isy23_psbB
atgcaaataactgatgttttaccttggtttagggtacatatagttattcttaatgatcca
ggacgtctcattagctcacatattatgcacacagctttagtagcaggttggtcagctctc
atgcttttatatgagcttatcaccatagatcctacagatcctgtgtataatccaatttgg
agacaagcagcttacacactcccatttatctcacgtattggtgttattcgctctcttttt
agttggtcacttggcatagatcctacatccaatctaatctggacttatgaaacaatgaac
atagcccatattctattatctggtttattaattcttgcatcattttggcattgggcatat
tgggacttagatctattcttcacctcaacattaacactagaccttaatcaaatcctcagt
attcacctcacactagcatcatctctctgtctgggatttggtttagctcatctaactggt
tttcttggtccaggaatgtggactagtgattccctcaatcttgtaggctctattagattc
gtaaaagcctccttcaatctccttgcttatgcacgattagcttatggtgtcatatcatca
catcacatcatctctggattgcttggcacttccataggattatggcatatcacattacgt
ccattagcatacctctacaatctattaagcatgggaaaggttgagtccattctttcaagt
agtattacagctgtattcttcactgcattccttatttcagcccttatgtggtatggctct
gcacacaccacacaagaactcttcggtcctactagatactcatgggacaatgcttattac
tctcttgatatcagaacctcttgctcaaatgcctggaacactcttccagacaagttagtc
ctttatgattatattggatctaaccctgctaaaggtggcttatttagttcaggaccaatg
cttaaagctgatggtcttgttcaaaactggttgggccatgcatgcttctccatgggtaca
ttatccttaagcatcagaaggatgcctgctttctttgaaaccttcccagtaatcctcatt
gaccaaacaagtacagtgagagcagacattgctttcagacggtccacatctacttatagc
atggaagaatcccaaatacaagtatacttctctggtggttgtttaaatggtacagaatat
tctacaccttcccttgtaaaagcttatgcaagaaaagctcagtttggtcagatatttaca
tttgataaaaagacatccagattgactgatggtgtatttagaacatctgctcgaggttgg
tattcattctcacacatagctttagctttctcctttttctttggccatttgtggcatgct
agtcgtgctatcttccaagacatttggacaggtgtaacatttgaatcacaagcaaaacaa
gaatatggtagaaatgaaaagctaggagataagacatcttcaactaaatccattgtttaa
>Isy24_psbB
atgcaaataactgctgctttaccttggtttagggtacatatagttattcttaatgatcca
ggacgtctcattagctcacacattatgcacacagctttagtagcaggttggtcagctctc
atgcttttatatgagcttatcaccatagatcctacagatcctgtgtataatccaatttgg
agacaagcagcttacacactcccatttatctcacgtattggtgttattcgctctcttttt
agttggtcacttggcatagatcctacatccaatctaatctggacttatgaaacaatgaac
atagcccatattctattatctggtttattaattcttgcatcattttggcattgggcatat
tgggacttagatctattcttcacctcaacattaacactagaccttaatcaaatcctcagt
attcacctcacactagcatcatctctctgtctgggatttggtttagctcatctaactggt
tttcttggtccaggaatgtggactagtgattccctcaatcttgtaggctctattagattc
gtaaaagcctccttcaatctccttgcttatgcacgattagcttatggtgtcatatcatca

catcacatcatctctggattgcttggcacttccataggattatggcatatcacattacgt
ccattagcatacctctacaatctattaagcatgggaaaggttgagtccattctttcaagt
agtattacagctgtattcttcactgcattccttatttcagcccttatgtggtatggttct
gcacataccacacaagaactcttcggtcctactagatactcatgggacaatgcttattac
tctcttgatatcagaacctcttgctcaaatgcctggaacactcttccagacaagttagtc
ctttatgattatattggatctaaccctgctaaaggtggcttatttcgttcaggaccaatg
cttaaagctgatggtcttgttcaaaactggttgggccatgcatgcttctccatgggtaca
ttatccttaagcatcagaaggatgcctgctttctttgaaaccttcccagtaatcctcatt
gaccaaacaagtacagtgagagcagacattgctttcagacggtccacatctacttatagc
atggaagaatcccaaatacaagtatacttctctggtggttgtttaaatggtacagaatat
tctacaccttcccttgtaaaagcttatgcaagaaaagctcagtttggtcagatatttaca
tttgataaaaagacatccagattgactgatggtgtatttagaacatctgctcgaggttgg
tattcattctcacacatagctttagctttctcctttttctttggccatttgtggcatgct
agtcgtgctatcttccaagacatttggacaggtgtaacatttgaatcacaagcaaaacaa
gaatatggtagaaatgaaaagctaggagataagacatcttcaactaaatccattgtttaa
>Isy25_psbB
atgcaaataactgatgctttaccttggtttagggtacatatagttattcttaatgatcca
ggacgtctcattagctcacacattatgcacacagctttagtagcaggttggtcagctctc
atgcttttatatgagcttatcaccatagatcctacagatcctgtgtataatccaatttgg
agacaagcagcttacacactcccatttatctcacgtattggtgttattcgctctcttttt
agttggtcacttggcatagatcctacatccaatctaatctggacttatgaaacaatgaac
atagcccatattctattatctggtttattaattcttgcatcattttggcattgggcatat
tgggacttagatctattcttcacctcaacattaacactagaccttaatcaaatcctcagt
attcacctcacactagcatcatctctctgtctgggatttggtttagctcatctaactggt
tttcttggtccaggaatgtggactagtgattccctcaatcttgtaggctctattagattc
gtaaaagcctccttcaatctccttgcttatgcacgattagcttatggtgtcatatcatca
catcacatcatctctggattgcttggcacttccataggattatagcatatcacattacgt
ccattagcatacctctacaatctattaagcatgggaaaggttgagtccattctttcaagt
agtattacagctgtattcttcactgcattccttatttcagcccttatgtggtatggctct
gcacataccacacaagaactattcggtcctactagatactcatgggacaatgcttattac
tctcttgatatcagaacctcttgctcaaatgcctggaacactcttccagacaagttagtc
ctttatgattatattggatctaaccctgctaaaggtggcttatttagttcaggaccaatg
cttaaagctgatggtcttgttcaaaactggttgggccatgcatgcttctccatgggtaca
ttatccttaagcatcagaaggatgcctgctttctttgaaaccttcccagtaatcctcatt
gaccaaacaagtacagtgagagcagacattgctttcagacggtccacatctacttatagc
atggaagaatcccaaatacaagtatacttctctggtggttgtttaaatggtacagaatat
tctacaccttcccttgtaaaagcttatgcaagaaaagctcagtttggtcagatatttaca
tttgataaaaagacatccagattgactgatggtgtatttagaacatctgctcgaggttgg
tattcattctcacacatagctttagctttctcctttttctttggccatttgtggcatgct
agtcgtgctatcttccaagacatttggacaggtgtaacatttgaatcacaagcaaaacaa
gaatatggtagaaatgaaaagctaggagataagacatcttcaactaaatccattgtttaa
>Isy26_psbB
atgcaaataactgatgctttaccttggtttagggtacatatagttattcttaatgatcca
ggacgtctcattagctcacatattatgcacacagctttagtagcaggttggtcagctctc
atgcttttatatgagcttatcaccatagatcctacagatcctgtgtataatccaatttgg
agacaagcagcttacacactcccatttatctcacgtattggtgttattcgctctcttttt
agttggtcacttggcatagatcctacatccaatctaatctggacttatgaaacaatgaac
atagcccatattctattatctggtttattaattcttgcatcattttggcattgggcatat
tgggacttagatctattcttcacctcaacattaacactagaccttaatcaaatcctcagt
attcacctcacactagcatcatctctctgtctgggatttggtttagctcatctaactggt
tttcttggtccaggaatgtggactagtgattccctcaatcttgtaggctctattagattc
gtaaaagcctccttcaatctccttgcttatgcacgattagcttatggtgtcatatcatca
catcacatcatctctggattgcttggcacttccataggattatggcatatcacattacgt
ccattagcatacctctacaatctattaagcatgggaaaggttgagtccattctttcaagt
agtattacagctgtattcttcactgcattccttatttcagccctgatgtggtatggttct
gcacacaccacacaagaactcttcggtcctactagatactcatgggacaatgcttattac
tctcttgatatcagaacctcttgctcaaatgcctggaacactcttccagacaagttagtc

ctttatgattatattggatctaaccctgctaaaggtggcttatttagttcaggaccaatg
cttaaagctgatggtcttgttcaaaactggttgggccatgcatgcttctccatgggtaca
ttatccttaagcatcagaaggatgcctgctttctttgaaaccttcccagtaatcctcatt
gaccaaacaagtacagtgagagcagacattgctttcagacggtccacatctacttatagc
atggaagaatcccaaatacaagtatacttctctggtggttgtttaaatggtacagaatat
tctacaccttcccttgtaaaagcttatgcaagaaaagctcagtttggtcagatatttaca
tttgataaaaagacatccagattgactgatggtgtatttagaacatctgctcgaggttgg
tattcattctcacacatagctttagctttctcctttttctttggccatttgtggcatgct
agtcgtgctatcttccaagacatttggacaggtgtaacatttgaatcacaagcaaaacaa
gaatatggtagaaatgaaaagctaggagataagacatcttcaactaaatccattgtttaa
>Isy27_psbB
atgcaaataactgatgctttaccttggtttagggtacatatagttattcttaatgatcca
ggacgtctcattagctcacatattatgcacacagctttagtagcaggttggtcagctctc
atgcttttatatgagcttatcaccatagatcctacagatcctgtgtataatccaatttgg
agacaagcagcttacacactcccatttatctcacgtattggtgttattcgctctcttttt
agttggtcacttggcatagatcctacatccaatctaatctggacttatgaaacaatgaac
atagcccatattctattatctggtttattaattcttgcatcattttggcattgggcatat
tgggacttagatctattcttcacctcaacattaacactagaccttaatcaaatcctcagt
attcacctcacactagcatcatctctctgtctgggatttggtttagctcatctaactggt
tttcttggtccaggaatgtggactagtgattccctcaatcttgtaggctctattagattc
gtaaaagcctccttcaatctccttgcttatgcacgattagcttatggtgtcatatcatca
catcacatcatctctggattgcttggcacttccataggattatggcatatcacattacgt
ccattagcatacctctacaatctattaagcatgggaaaggttgagtccattctttcaagt
agtattacagctgtattcttcactgcattccttatttcagcccttatgtggtatggctct
gcacacaccacacaagaactcttcggtcctactagatactcatgggacaatgcttattac
tctcttgatatcagaacctcttgctcaaatgcctggaacactcttccagacaagttagtc
ctttatgattatattggatctaaccctgctaaaggtggcttatttagttcaggaccaatg
cttaaagctgatggtcttgttcaaaactggttgggccatgcatgcttctccatgggtaca
ttatccttaagcatcagaaggatgcctgctttctttgaaaccttcccagtaatcctcatt
gaccaaacaagtacagtgagagcagacattgctttcagacggtccacatctacttatagc
atggaagaatcccaaatacaagtatacttctctggtggttgtttaaatggtacagaatat
tctacaccttcccttgtaaaagcttatgcaagaaaagctcagtttggtcagatatttaca
tttgataaaaagacatccagattgactgatggtgtatttagaacatctgctcgaggttgg
tattcattctcacacatagctttagctttctcctttttctttggccatttgtggcatgct
agtcgtgctatcttccaagacatttggacaggtgtaacatttgaatcacaagcaaaacaa
gaatatggtagaaatgaaaagctaggagataagacatcttcaactaaatccattgtttaa
>Isy4_psbB
atgcaaataactgatgctttaccttggtttagggtacatatagttattcttaatgatcca
ggacgtctcattagctcacatattatgcacacagctttagtagcaggttggtcagctctc
atgcttttatatgagcttatcaccatagatcctacagatcctgtgtataatccaatttgg
agacaagcagcttacacactcccatttatctcacgtattggtgttattcgctctcttttt
agttggtcacttggcatagatcctacatccaatctaatctggacttatgaaacaatgaac
atagcccatattctattatctggtttattaattcttgcatcattttggcattgggcatat
tgggacttagatctattcttcacctcaacattaacactagaccttaatcaaatcctcagt
attcacctcacactagcatcatctctctgtctgggatttggtttagctcatctaactggt
tttcttggtccaggaatgtggactagtgattccctcaatcttgtaggctctattagattc
gtaaaagcctccttcaatctccttgcttatgcacgattagcttatggtgtcatatcatca
catcacatcatctctggattgcttggcacttccataggattatggcatatcacattacgt
ccattagcatacctctacaatctattaagcatgggaaaggttgagtccattctttcaagt
agtattacagctgtattcttcactgcattccttatttcagcccttatgtggtatggctct
gcacacaccacacaagaactcttcggtcctactagatactcatgggacaatgcttattac
tctcttgatatcagaacctcttgctcaaatgcctggaacactcttccagacaagttagtc
ctttatgattatattggatctaaccctgctaaaggtggcttatttcgttcaggaccaatg
cttaaagctgatggtcttgttcaaaactggttgggccatgcatgcttctccatgggtaca
ttatccttaagcatcagaaggatgcctgctttctttgaaaccttcccagtaatcctcatt
gaccaaacaagtacagtgagagcagacattgctttcagacggtccacatctacttatagc
atggaagaatcccaaatacaagtatacttctctggtggttgtttaaatggtacagaatat

tctacaccttcccttgtaaaagcttatgcaagaaaagctcagtttggtcagatatttaca
tttgataaaaagacatccagattgactgatggtgtatttagaacatctgctcgaggttgg
tattcattctcacacatagctttagctttctccttttcttggccatttgtggcatgct
agtcgtgctatcttccaagacatttggacaggtgtaacatttgaatcacaagcaaaacaa
gaatatggtagaaatgaaaagctaggagataagacatcttcaactaaatccattgtttaa
>Isy7_psbB
atgcaaataactgctgttttaccttggtttagggtacatatagttattcttaatgatcca
ggacgtctcattagctcacatattatgcacacagctttagtagcaggttggtcagctctc
atgcttttatatgagcttatcaccatagatcctacagaccctgtgtataacccaatttgg
agacaagccgcttatacactcccatttatctcacgtattggtgttattcgctctcttttt
aattggtcaattggtatagatcctacatccaatctaatctggacttatgaaacaatgaac
atagcccatattctattatctggtttattaattcttgcatcattttggcattgggcatat
tgggacttagatctattcttcacctcaacattaacactagaccttaatcaaatcctcagt
attcacctcacacttgcatcctctctctgtctgggatttggtttagctcatctaactggt
tttcttggtccaggaatgtggactagtgattctcttaatcttgtaggctctattagattc
gtaaaagcctccttcaatctccttgcttatgcacgattagcttatggtgtcatatcatca
catcacatcatctctggattgcttggcacttccataggattatggcatatcacattacgt
ccattagcatatctctacaatctattaagcatgggaaaggttgagtccattctttcaagt
agtattacagctgtattcttcactgcattccttatatcagctcttatgtggtatggctct
gcacacaccacacaagaactcttcggtcctactagatactcatgggacaatgcttattac
tctcttgatatcagaacctcttgctcaaatgcctggaacactcttccagacaagttagtc
ctttatgattatattggatctaaccctgctaaaggtggcttatttcgttcaggaccaatg
cttaaagctgatggtcttgttcaaaactggttgggccatgcatgcttctccatgggtaca
ttatccttaagcatcagaaggatgcctgctttctttgaaaccttcccagtaatcctcatt
gaccaaacaagtacagtgagagcagacattgctttcagacggtccacatctacttatagc
atggaagaatcccaaatacaagtatacttctctggtggttgtttaaatggtacagaatat
tctacaccttcccttgtaaaagcttatgcaagaaaagctcagtttggtcagatatttaca
tttgataaaaagacatccagattgactgatggtgtatttagaacatctgctcgaggttgg
tattcattctcacacatagctttagctttctccttttcttggccatttgtggcatgct
agtcgtgctatcttccaagacatttggacaggtgtaacatttgaatcacaagcaaaacaa
gaatatggtagaaatgaaaagctaggagataagacatcttcaactaaatccattgtttaa
>Isy8_psbB
atgcaaataactgatgctttaccttggtttagggtacatatagttattcttaatgatcca
ggacgtctcattagctcacatattatgcacacagctttagtagcaggttggtcagctctc
atgcttttatatgagcttatcaccatagatcctacagatcctgtgtataatccaatttgg
agacaagcagcttacacactcccatttatctcacgtattggtgttattcgctctcttttt
agttggtcacttggcatagatcctacatccaatctaatctggacttatgaaacaatgaac
atagcccatattctattatctggtttattaattcttgcatcattttggcattgggcatat
tgggacttagatctattcttcacctcaacattaacactagaccttaatcaaatcctcagt
attcacctcacactagcatcatctctctgtctgggatttggtttagctcatctaactggt
tttcttggtccaggaatgtggactagtgattccctcaatcttgtaggctctattagattc
gtaaaagcctccttcaatctccttgcttatgcacgattagcttatggtgtcatatcatca
catcacatcatctctggattgcttggcacttccataggattatggcatatcacattacgt
ccattagcatacctctacaatctattaagcatgggaaaggttgagtccattctttcaagt
agtattacagctgtattcttcactgcattccttattttagcccttatgtggtatggctct
gcacacaccacacaagaactcttcggtcctactagatactcatgggacaatgcttattac
tctcttgatatcagaacctcttgctcaaatgcctggaacactcttccagacaagttagtc
ctttatgattatattggatctaaccctgctaaaggtggcttatttagttcaggaccaatg
cttaaagctgatggtcttgttcaaaactggttgggccatgcatgcttctccatgggtaca
ttatccttaagcatcagaaggatgcctgctttctttgaaaccttcccagtaatcctcatt
gaccaaacaagtacagtgagagcagacattgctttcagacggtccacatctacttatagc
atggaagaatcccaaatacaagtatacttctctggtggttgtttaaatggtacagaatat
tctacaccttcccttgtaaaagcttatgcaagaaaagctcagtttggtcagatatttaca
tttgataaaaagacatccagattgactgatggtgtatttagaacatctgctcgaggttgg
tattcattctcacacatagctttagctttctccttttcttggccatttgtggcatgct
agtcgtgctatcttccaagacatttggacaggtgtaacatttgaatcacaagcaaaacaa
gaatatggtagaaatgaaaagctaggagataagacatcttcaactaaatccattgtttaa

>KrA1_psbB
atgcaaataactgatgctttaccttggtttagggtacatatagttattcttaatgatcca
ggacgtctcattagctcacatattatgcacacagctttagtagcaggttggtcagctctc
atgcttttatatgagcttatcaccatagatcctacagatcctgtgtataatccaatttgg
agacaagcagcttacacactcccatttatctcacgtattggtgttattcgctctcttttt
agttggtcacttggcatagatcctacatccaatctaatctggacttatgaaacaatgaac
atagcccatattctattatctggtttattaattcttgcatcattttggcattgggcatat
tgggacttagatctattcttcacctcaacattaacactagaccttaatcaaatcctcagt
attcacctcacactagcatcatctctctgtctgggatttggtttagctcatctaactggt
tttcttggtccaggaatgtggactagtgattccctcaatcttgtaggctctattagattc
gtaaaagcctccttcaatctccttgcttatgcacgattagcttatggtgtcatatcatca
catcacatcatctctggattgcttggcacttccataggattatggcatatcacattacgt
ccattagcatacctctacaatctattaagcatgggaaaggttgagtccattctttcaagt
agtattacagctgtattcttcactgcattccttatttcagcccttatgtggtatggctct
gcacacaccacacaagaactcttcggtcctactagatactcatgggacaatgcttattac
tctcttgatatcagaacctcttgctcaaatgcctggaacactcttccagacaagttagtc
ctttatgattatattggatctaaccctgctaaaggtggcttatttcgttcaggaccaatg
cttaaagctgatggtcttgttcaaaactggttgggccatgcatgcttctccatgggtaca
ttatccttaagcatcagaaggatgcctgctttctttgaaaccttcccagtaatcctcatt
gaccaaacaagtacagtgagagcagacattgctttcagacggtccacatctacttatagc
atggaagaatcccaaatacaagtatacttctctggtggttgtttaaatggtacagaatat
tctacaccttcccttgtaaaagcttatgcaagaaaagctcagtttggtcagatatttaca
tttgataaaaagacatccagattgactgatggtgtatttagaacatctgctcgaggttgg
tattcattctcacacatagctttagctttctcctttttctttggccatttgtggcatgct
agtcgtgctatcttccaagacatttggacaggtgtaacatttgaatcacaagcaaaacaa
gaatatggtagaaatgaaaagctaggagataagacatcttcaactaaatccattgtttaa
>KrA10_psbB
atgcaaataactgatgttttaccttggtttagggtacatatagttattcttaatgatcca
ggacgtctcattagctcacatattatgcacacagctttagtagcaggttggtcagctctc
atgcttttatatgagcttatcaccatagatcctacagatcctgtgtataatccaatttgg
agacaagcagcttacacactcccatttatctcacgtattggtgttattcgctctcttttt
agttggtcacttggcatagatcctacatccaatctaatctggacttatgaaacaatgaac
atagcccatattctattatctggtttattaattcttgcatcattttggcattgggcatat
tgggacttagatctattcttcacctcaacattaacactagaccttaatcaaatcctcagt
attcacctcacactagcatcatctctctgtctgggatttggtttagctcatctaactggt
tttcttggtccaggaatgtggactagtgattccctcaatcttgtaggctctattagattc
gtaaaagcctccttcaatctccttgcttatgcacgattagcttatggtgtcatatcatca
catcacatcatctctggattgcttggcacttccataggattatggcatatcacattacgt
ccattagcatacctctacaatctattaagcatgggaaaggttgagtccattctttcaagt
agtattacagctgtattcttcactgcattccttatttcagcccttatgtggtatggctct
gcacacaccacacaagaactcttcggtcctactagatactcatgggacaatgcttattac
tctcttgatatcagaacctcttgctcaaatgcctggaacactcttccagacaagttagtc
ctttatgattatattggatctaaccctgctaaaggtggcttatttcgttcaggaccaatg
cttaaagctgatggtcttgttcaaaactggttgggccatgcatgcttctccatgggtaca
ttatccttaagcatcagaaggatgcctgctttctttgaaaccttcccagtaatcctcatt
gaccaaacaagtacagtgagagcagacattgctttcagacggtccacatctacttatagc
atggaagaatcccaaatacaagtatacttctctggtggttgtttaaatggtacagaatat
tctacaccttcccttgtaaaagcttatgcaagaaaagctcagtttggtcagatatttaca
tttgataaaaagacatccagattgactgatggtgtatttagaacatctgctcgaggttgg
tattcattctcacacatagctttagctttctcctttttctttggccatttgtggcatgct
agtcgtgctatcttccaagacatttggacaggtgtaacatttgaatcacaagcaaaacaa
gaatatggtagaaatgaaaagctaggagataagacatcttcaactaaatccattgtttaa
>KrA11_psbB
atgcaaataactgatgctttaccttggtttagggtacatatagttattcttaatgatcca
ggacgtctcattagctcacatattatgcacacagctttagtagcaggttggtcagctctc
atgcttttatatgagcttatcaccatagatcctacagatcctgtgtataatccaatttgg
agacaagcagcttacacactcccatttatctcacgtattggtgttattcgctctcttttt

agttggtcacttggcatagatcctacatccaatctaatctggacttatgaaacaatgaac
atagcccatattctattatctggtttattaattcttgcatcattttggcattgggcatat
tgggacttagatctattcttcacctcaacattaacactagaccttaatcaaatcctcagt
attcacctcacactagcatcatctctctgtctgggatttggtttagctcatctaactggt
tttcttggtccaggaatgtggactagtgattccctcaatcttgtaggctctattagattc
gtaaaagcctccttcaatctccttgcttatgcacgattagcttatggtgtcatatcatca
catcacatcatctctggattgcttggcacttccataggattatggcatatcacattacgt
ccattagcatacctctacaatctattaagcatgggaaaggttgagtccattctttcaagt
agtattacagctgtattcttcactgcattccttatttcagcccttatgtggtatggctct
gcacacaccacacaagaactcttcggtcctactagatactcatgggacaatgcttattac
tctcttgatatcagaacctcttgctcaaatgcctggaacactcttccagacaagttagtc
ctttatgattatattggatctaaccctgctaaaggtggcttatttcgttcaggaccaatg
cttaaagctgatggtcttgttcaaaactggttgggccatgcatgcttctccatgggtaca
ttatccttaagcatcagaaggatgcctgctttctttgaaaccttcccagtaatcctcatt
gaccaaacaagtacagtgagagcagacattgctttcagacggtccacatctacttatagc
atggaagaatcccaaatacaagtatacttctctggtggttgtttaaatggtacagaatat
tctacaccttcccttgtaaaagcttatgcaagaaaagctcagtttggtcagatatttaca
tttgataaaaagacatccagattgactgatggtgtatttagaacatctgctcgaggttgg
tattcattctcacacatagctttagctttctcctttttctttggccatttgtggcatgct
agtcgtgctatcttccaagacatttggacaggtgtaacatttgaatcacaagcaaaacaa
gaatatggtagaaatgaaaagctaggagataagacatcttcaactaaatccattgtttaa
>KrA12_psbB
atgcaaataactgatgctttaccttggtttagggtacatatagttattcttaatgatcca
ggacgtctcattagctcacatattatgcacacagctttagtagctgggtggtcagctctc
atgcttttatatgagcttatcaccatagatcctacagatcctgtgtataatccaatttgg
agacaagcagcttacacactcccatttatctcacgtattggtgttattcgctctcttttt
agttggtcacttggcatagatcctacatccaatctaatctggacttatgaaacaatgaac
atagcccatattctattatctggtttattaattcttgcatcattttggcattgggcatat
tgggacttagatctattcttcacctcaacattaacactagaccttaatcaaatcctcagt
attcacctcacactagcatcatctctctgtctgggatttggtttagctcatctaacaggt
tttcttggtccaggaatgtggactagtgattccctcaatcttgtaggctctattagattc
gtaaaagcctccttcaatatcctttcttatgcacgattagcttatggtgtcatatcatca
catcacatcatctctggattgcttggcacttccataggattatggcatatcacattacgt
ccattagcatacctctacaatctattaagcatgggaaaggttgagtccattctttcaagt
agtattacagctgtattcttcactgcattccttatttcagccctgatgtggtatggttct
gcacataccacacaagaactcttcggtcctactagatactcatgggacaatgcttattac
tctcttgatatcagaacctcttgctcaaatgcctggaacactcttccagacaagttagtc
ctttatgattatattggatctaaccctgctaaaggtggcttatttcgttcaggaccaatg
cttaaagctgatggtcttgttcaaaactggttgggccatgcatgcttctccatgggtaca
ttatccttaagcatcagaaggatgcctgctttctttgaaaccttcccagtaatcctcatt
gaccaaacaagtacagtgagagcagacattgctttcagacggtccacatctacttatagc
atggaagaatcccaaatacaagtatacttctctggtggttgtttaaatggtacagaatat
tctacaccttcccttgtaaaagcttatgcaagaaaagctcagtttggtcagatatttaca
tttgataaaaagacatccagattgactgatggtgtatttagaacatctgctcgaggttgg
tattcattctcacacatagctttagctttctcctttttctttggccatttgtggcatgct
agtcgtgctatcttccaagacatttggacaggtgtaacatttgaatcacaagcaaaacaa
gaatatggtagaaatgaaaagctaggagataagacatcttcaactaaatccattgtttaa
>KrA13_psbB
atgcaaataactgatgttttaccttggtttagggtacatatagttattcttaatgatcca
ggacgtctcattagctcacatattatgcacacagctttagtagcaggttggtcagctctc
atgcttttatatgagcttatcaccatagatcctacagatcctgtgtataatccaatttgg
agacaagcagcttacacactcccatttatctctcgtatcggtgttattcgctctcttttt
agttggtcacttggcatagatcctacatccaatctaatctggacttatgaaacaatgaac
atagcccatattctattatctggtttattaattcttgcatcattttggcattgggcatat
tgggacttagatctattcttcacctcaacattaacactagaccttaatcaaatcctcagt
attcacctcacactagcatcatctctctgtctgggatttggtttagctcatctaactggt
tttcttggtccaggaatgtggactagtgattccctcaatcttgtaggctctattagattc

gtaaaagcctccttcaatctccttgcttatgcacgattagcttatggtgtcatatcatca
catcacatcatctctggattgcttggcacttccataggattatggcatatcacattacgt
ccattagcatacctctacaatctattaagcatgggaaaggttgagtccattctttcaagt
agtattacagctgtattcttcactgcattccttatttcagcccttatgtggtatggctct
gcacacaccacacaagaactcttcggtcctactagatactcatgggacaatgcttattac
tctcttgatatcagaacctcttgctcaaatgcctggaacactcttccagacaagttagta
ctttatgattatattggctctaaccctgctaaaggtggcttatttcgttcaggaccaatg
cttaaagctgatggtcttgttcaaaactggttgggccatgcatgcttctccatgggtaca
ttatccttaagcatcagaaggatgcctgctttctttgaaaccttcccagtaatcctcatt
gaccaaacaagtacagtgagagcagacattgctttcagacggtccacatctacttatagc
atggaagaatcccaaatacaagtatacttctctggtggttgtttaaatggtacagaatat
tctacaccttcccttgtaaaagcttatgcaagaaaagctcagtttggtcagatatttaca
tttgataaaaagacatccagattgactgatggtgtatttagaacatctgctcgaggttgg
tattcattctcacacatagctttagctttctcctttttctttggccatttgtggcatgct
agtcgtgctatcttccaagacatttggacaggtgtaacatttgaatcacaagcaaaacaa
gaatatggtagaaatgaaaagctaggagataagacatcttcaactaaatccattgtttaa
>KrA14_psbB
atgcaaataactgatgctttaccttggtttagggtacatatagttattcttaatgatcca
ggacgtctcattagctcacatattatgcacacagctttagtagcaggttggtcagctctc
atgcttttatatgagcttatcaccatagatcctacagatcctgtgtataatccaatttgg
agacaagcagcttacacactcccatttatctcacgtattggtgttattcgctctcttttt
agttggtcacttggcatagatcctacatccaatctaatctggacttatgaaacaatgaac
atagcccatattctattatctggtttattaattcttgcatcattttggcattgggcatat
tgggacttagatctattcttcacctcaacattaacactagaccttaatcaaatcctcagt
attcacctcacactagcatcatctctctgtctgggatttggtttagctcatctaactggt
tttcttggtccaggaatgtggactagtgattccctcaatcttgtaggctctattagattc
gtaaaagcctccttcaatctcctttcttatgcacgattagcttatggtgtcatatcatca
catcacatcatctctggattgcttggcacttccataggattatggcatatcacattacgt
ccattagcatacctctacaatctattaagcatgggaaaggttgagtccattctttcaagt
agtattacagctgtattcttcactgcattccttatttcagcccttatgtggtatggctct
gcacacaccacacaagaactcttcggtcctactagatactcatgggacaatgcttattac
tctcttgatatcagaacctcttgctcaaatgcctggaacactcttccagacaagttagta
ctttatgattatattggatctaaccctgctaaaggtggcttatttcgttcaggaccaatg
cttaaagctgatggtcttgttcaaaactggttgggccatgcatgtttctccatgggtaca
ttatccttaagcatcagaaggatgcctgctttctttgaaaccttcccagtaatcctcatt
gaccaaacaagtacagtgagagcagacattgctttcagacggtccacatctacttatagc
atggaagaatcccaaatacaagtatacttctctggtggttgtttaaatggtacagaatat
tctacaccttcccttgtaaaagcttatgcaagaaaagctcagtttggtcagatatttaca
tttgataaaaagacatccagattgactgatggtgtatttagaacatctgctcgaggttgg
tattcattctcacacatagctttagctttctcctttttctttggccatttgtggcatgct
agtcgtgctatcttcaaagacatttggacaggtgtaacatttgaatcacaagcaaaacaa
gaatatggtagaaatgaaaagctaggagataagacatcttcaactaaatccattgtttaa
>KrA15_psbB
atgcaaataactgatgctttaccttggtttagggtacatatagttattcttaatgatcca
ggacgtctcattagctcacatattatgcacacagctttagtagcaggttggtcagctctc
atgcttttatatgagcttatcaccatagatcctacagatcctgtgtataatccaatttgg
agacaagcagcttacacactcccatttatctcacgtattggtgttattcgctctcttttt
agttggtcacttggcatagatcctacatccaatctaatctggacttatgaaacaatgaac
atagcccatattctattatctggtttattaattcttgcatcattttggcattgggcatat
tgggacttagatctattcttcacctcaacattaacactagaccttaatcaaatcctcagt
attcacctcacactagcatcatctctctgtctgggatttggtttagctcatctaacaggt
tttcttggtccaggaatgtggactagtgattccctcaatcttgtaggctctattagattc
gtaaaagcctccttcaatctccttgcttatgcacgattagcttatggtgtcatatcatca
catcacatcatctctggattgcttggcacttccataggattatggcatatcacattacgt
ccattagcatacctctacaatctattaagcatgggaaaggttgagtccattctttcaagt
agtattacagctgtattcttcactgcattccttatttcagcccttatgtggtatggctct
gcacacaccacacaagaactcttcggtcctactagatactcatgggacaatgcttattac

tctcttgatatcagaacctcttgctcaaatgcctggaacactcttccagacaagttagtc
cttgatgattatattggctctaaccctgctaaaggtggcttatttcgttcaggaccaatg
cttaaagctgatggtcttgttcaaaactggttaggtcatgcatgtttctccatgggtaca
ttatccttaagcatcagaaggatgcctgctttctttgaaaccttcccagtaatcctcatt
gaccaaacaagtacagtgagagcagacattgctttcagacggtccacatctacttatagc
atggaagaatcccaaatacaagtatacttctctggtggttgtttaaatggtacagaatat
tctacaccttcccttgtaaaagcttatgcaagaaaagctcagtttggtcagatatttaca
tttgataaaaagacatccagattgactgatggtgtatttagaacatctgctcgaggttgg
tattcattctcacacatagctttagctttcctattcttctttggccatttgtggcatgct
agtcgtgctatcttccaagacatttggacaggtgtaacatttgaatcacaagcaaaacaa
gaatatggtagaaatgaaaagctaggagataagacatcttcaactaaatccattgtttaa
>KrA2_psbB
atgcaaataactgatgctttaccttggtttagggtacatatagttattcttaatgatcca
ggacgtctcattagctcacatattatgcacacagctttagtagcaggttggtcagctctc
atgcttttatatgagcttatcaccatagatcctacagatcctgtgtataatccaatttgg
agacaagcagcttacacactcccatttatctcacgtattggtgttattcgctctcttttt
agttggtcacttggcatagatcctacatccaatctaatctggacttatgaaacaatgaac
atagcccatattctattatctggtttattaattcttgcatcattttggcattgggcatat
tgggacttagatctattcttcacctcaacattaacactagaccttaatcaaatcctcagt
attcacctcacactagcatcatctctctgtctgggatttggtttagctcatctaactggt
tttcttggtccaggaatgtggactagtgattccctcaatcttgtaggctctattagattc
gtaaaagcctccttcaatctccttgcttatgcacgattagcttatggtgtcatatcatca
catcacatcatctctggattgcttggcacttccataggattatggcatatcacattacgt
ccattagcatacctctacaatctattaagcatgggaaaggttgagtccattctttcaagt
agtattacagctgtattcttcactgcattccttatttcagcccttatgtggtatggctct
gcacacaccacacaagaactcttcggtcctactagatactcatgggacaatgcttattac
tctcttgatatcagaacctcttgctcaaatgcctggaacactcttccagacaagttagtc
ctttatgattatattggatctaaccctgctaaaggtggcttatttcgttcaggaccaatg
cttaaagctgatggtcttgttcaaaactggttgggccatgcatgcttctccatgggtaca
ttatccttaagcatcagaaggatgcctgctttctttgaaaccttcccagtaatcctcatt
gaccaaacaagtacagtgagagcagacattgctttcagacggtccacatctacttatagc
atggaagaatcccaaatacaagtatacttctctggtggttgtttaaatggtacagaatat
tctacaccttcccttgtaaaagcttatgcaagaaaagctcagtttggtcagatatttaca
tttgataaaaagacatccagattgactgatggtgtatttagaacatctgctcgaggttgg
tattcattctcacacatagctttagctttctcctttttctttggccatttgtggcatgct
agtcgtgctatcttccaagacatttggacaggtgtaacatttgaatcacaagcaaaacaa
gaatatggtagaaatgaaaagctaggagataagacatcttcaactaaatccattgtttaa
>KrA3_psbB
atgcaaataactgatgctttaccttggtttagggtacatatagttattcttaatgatcca
ggacgtctcattagctcacatattatgcacacagctttagtagcaggttggtcagctctc
atgcttttatatgagcttatcaccatagatcctacagatcctgtgtataatccaatttgg
agacaagcagcttacacactcccatttatctcacgtattggtgttattcgctctcttttt
agttggtcacttggcatagatcctacatccaatctaatctggacttatgaaacaatgaac
atagcccatattctattatctggtttattaattcttgcatcattttggcattgggcatat
tgggacttagatctattcttcacctcaacattaacactagaccttaatcaaatcctcagt
attcacctcacactagcatcatctctctgtctgggatttggtttagctcatctaactggt
tttcttggtccaggaatgtggactagtgattccctcaatcttgtaggctctattagattc
gtaaaagcctccttcaatctccttgcttatgcacgattagcttatggtgtcatatcatca
catcacatcatctctggattgcttggcacttccataggattatggcatatcacattacgt
ccattagcatacctctacaatctattaagcatgggaaaggttgagtccattctttcaagt
agtattacagctgtattcttcactgcattccttatttcagcccttatgtggtatggctct
gcacacaccacacaagaactcttcggtcctactagatactcatgggacaatgcttattac
tctcttgatatcagaacctcttgctcaaatgcctggaacactcttccagacaagttagtc
ctttatgattatattggatctaaccctgctaaaggtggcttatttcgttcaggaccaatg
cttaaagctgatggtcttgttcaaaactggttgggccatgcatgcttctccatgggtaca
ttatccttaagcatcagaaggatgcctgctttctttgaaaccttcccagtaatcctcatt
gaccaaacaagtacagtgagagcagacattgctttcagacggtccacatctacttatagc

atggaagaatcccaaatacaagtatacttctctggtggttgtttaaatggtacagaatat
tctacaccttcccttgtaaaagcttatgcaagaaaagctcagtttggtcagatatttaca
tttgataaaaagacatccagattgactgatggtgtatttagaacatctgctcgaggttgg
tattcattctcacacatagctttagctttctcctttttctttggccatttgtggcatgct
agtcgtgctatcttccaagacatttggacaggtgtaacatttgaatcacaagcaaaacaa
gaatatggtagaaatgaaaagctaggagataagacatcttcaactaaatccattgtttaa
>KrA4_psbB
atgcaaataactgatgctttaccttggtttagggtacatatagttattcttaatgatcca
ggacgtctcattagctcacatattatgcacacagctttagtagcaggttggtcagctctc
atgcttttatatgagcttatcaccatagatcctacagatcctgtgtataatccaatttgg
agacaagcagcttacacactcccatttatctcacgtattggtgttattcgctctcttttt
agttggtcacttggcatagatcctacatccaatctaatctggacttatgaaacaatgaac
atagcccatattctattatctggtttattaattcttgcatcattttggcattgggcatat
tgggacttagatctattcttcacctcaacattaacactagaccttaatcaaatcctcagt
attcacctcacactagcatcatctctctgtctgggatttggtttagctcatctaacaggt
tttcttggtccaggaatgtggactagtgattccctcaatcttgtaggctctattagattc
gtaaaagcctccttcaatctccttgcttatgcacgattagcttatggtgtcatatcatca
catcacatcatctctggattgcttggcacttccataggattatggcatatcacattacgt
ccattagcatacctctacaatctattaagcatgggaaaggttgagtccattctttcaagt
agtattacagctgtattcttcactgcattccttatttcagcccttatgtggtatggctct
gcacacaccacacaagaactcttcggtcctactagatactcatgggacaatgcttattac
tctcttgatatcagaacctcttgctcaaatgcctggaacactcttccagacaagttagtc
ctttatgattatattggatctaaccctgctaaaggtggcttatttcgttcaggaccaatg
cttaaagctgatggtcttgttcaaaactggttgggccatgcatgcttctccatgggtaca
ttatccttaagcatcagaaggatgcctgctttctttgaaaccttcccagtaatcctcatt
gaccaaacaagtacagtgagagcagacattgctttcagacggtccacatctacttatagc
atggaagaatcccaaatacaagtatacttctctggtggttgtttaaatggtacagaatat
tctacaccttcccttgtaaaagcttatgcaagaaaagctcagtttggtcagatatttaca
tttgataaaaagacatccagattgactgatggtgtatttagaacatctgctcgaggttgg
tattcattctcacacatagctttagctttctcctttttctttggccatttgtggcatgct
agtcgtgctatcttccaagacatttggacaggtgtaacatttgaatcacaagcaaaacaa
gaatatggtagaaatgaaaagctaggagataagacatcttcaactaaatccattgtttaa
>KrA5_psbB
atgcaaataactgatgctttaccttggtttagggtacatatagttattcttaatgatcca
ggacgtctcattagctcacatattatgcacacagctttagtagcaggttggtcagctctc
atgcttttatatgagcttatcaccatagatcctacagatcctgtgtataatccaatttgg
agacaagcagcttacacactcccatttatctcacgtattggtgttattcgctctcttttt
agttggtcacttggcatagatcctacatccaatctaatctggacttatgaaacaatgaac
atagcccatattctattatctggtttattaattcttgcatcattttggcattgggcatat
tgggacttagatctattcttcacctcaacattaacactagaccttaatcaaatcctcagt
attcacctcacactagcatcatctctctgtctgggatttggtttagctcatctaactggt
tttcttggtccaggaatgtggactagtgattccctcaatcttgtaggctctattagattc
gtaaaagcctccttcaatctccttgcttatgcacgattagcttatggtgtcatatcatca
catcacatcatctctggattgcttggcacttccataggattatggcatatcacattacgt
ccattagcatacctctacaatctattaagcatgggaaaggttgagtccattctttcaagt
agtattacagctgtattcttcactgcattccttatttcagcccttatgtggtatggctct
gcacacaccacacaagaactcttcggtcctactagatactcatgggacaatgcttattac
tctcttgatatcagaacctcttgctcaaatgcctggaacactcttccagacaagttagtc
ctttatgattatattggatctaaccctgctaaaggtggcttatttcgttcaggaccaatg
cttaaagctgatggtcttgttcaaaactggttgggccatgcatgcttctccatgggtaca
ttatccttaagcatcagaaggatgcctgctttctttgaaaccttcccagtaatcctcatt
gaccaaacaagtacagtgagagcagacattgctttcagacggtccacatctacttatagc
atggaagaatcccaaatacaagtatacttctctggtggttgtttaaatggtacagaatat
tctacaccttcccttgtaaaagcttatgcaagaaaagctcagtttggtcagatatttaca
tttgataaaaagacatccagattgactgatggtgtatttagaacatctgctcgaggttgg
tattcattctcacacatagctttagctttctcctttttctttggccatttgtggcatgct
agtcgtgctatcttccaagacatttggacaggtgtaacatttgaatcacaagcaaaacaa

gaatatggtagaaatgaaaagctaggagataagacatcttcaactaaatccattgtttaa
>KrA6_psbB
atgcaaataactgctgttttaccttggtttagggtacatatagttattcttaatgatcca
ggacgtctcattagctcacatattatgcacacagctttagtagcaggttggtcagcactc
atgcttttatatgagcttatcaccatagatcctacagatcctgtgtataatccaatttgg
agacaagcagcttacacactcccatttatctcacgtattggtgttattcgctctcttttt
agttggtcacttggcatagatcctacatccaatctaatctggacttatgaaacaatgaac
atagcccatattctattatctggtttattaattcttgcatcattttggcattgggcatat
tgggacttagatctattcttcacctcaacattaacactagaccttaatcaaatcctcagt
attcacctcacactagcatcatctctctgtctgggatttggtttagctcatctaactggt
tttcttggtccaggaatgtggactagtgattccctcaatcttgtaggctctattagattc
gtaaaagcctccttcaatctccttgcttatgcacgattagcttatggtgtcatatcatca
catcacatcatctctggattgcttggcacttccataggattatggcatatcacattacgt
ccattagcatacctctacaatctattaagcatgggaaaggttgagtccattctttcaagt
agtattacagctgtattcttcactgcattccttatttcagcccttatgtggtatggctct
gcacacaccacacaagaactcttcggtcctactagatactcatgggacaatgcttattac
tctcttgatatcagaacctcttgctcaaatgcctggaacactcttccagacaagttagtc
ctttatgattatattggatctaaccctgctaaaggtggcttatttcgttcaggaccaatg
cttaaagctgatggtcttgttcaaaactggttgggccatgcatgcttctccatgggtaca
ttatccttaagcatcagaaggatgcctgctttctttgaaaccttcccagtaatcctcatt
gaccaaacaagtacagtgagagcagacattgctttcagacggtccacatctacttatagc
atggaagaatcccaaatacaagtatacttctctggtggttgtttaaatggtacagaatat
tctacaccttcccttgtaaaagcttatgcaagaaaagctcagtttggtcagatatttaca
tttgataaaaagacatccagattgactgatggtgtatttagaacatctgctcgaggttgg
tattcattctcacacatagctttagctttctcctttttctttggccatttgtggcatgct
agtcgtgctatcttccaagacatttggacaggtgtaacatttgaatcacaagcaaaacaa
gaatatggtagaaatgaaaagctaggagataagacatcttcaactaaatccattgtttaa
>KrA7_psbB
atgcaaataactgatgctttaccttggtttagggtacatatagttattcttaatgatcca
ggacgtctcattagctcacatattatgcacacagctttagtagcaggttggtcagctctc
atgcttttatatgagcttatcaccatagatcctacagatcctgtgtataatccaatttgg
agacaagcagcttacacactcccatttatctcacgtattggtgttattcgctctcttttt
agttggtcacttggcatagatcctacatccaatctaatctggacttatgaaacaatgaac
atagcccatattctattatctggtttattaattcttgcatcattttggcattgggcatat
tgggacttagatctattcttcacctcaacattaacactagaccttaatcaaatcctcagt
attcacctcacactagcatcatctctctgtctgggatttggtttagctcatctaactggt
tttcttggtccaggaatgtggactagtgattccctcaatcttgtaggctctattagattc
gtaaaagcctccttcaatctccttgcttatgcacgattagcttatggtgtcatatcatca
catcacatcatctctggattgcttggcacttccataggattatggcatatcacattacgt
ccattagcatacctctacaatctattaagcatgggaaaggttgagtccattctttcaagt
agtattacagctgtattcttcactgcattccttatttcagcccttatgtggtatggctct
gcacacaccacacaagaactcttcggtcctactagatactcatgggacaatgcttattac
tctcttgatatcagaacctcttgctcaaatgcctggaacactcttccagacaagttagtc
ctttatgattatattggatctaaccctgctaaaggtggcttatttcgttcaggaccaatg
cttaaagctgatggtcttgttcaaaactggttaggccatgcatgcttctccatgggtaca
ttatccttaagcatcagaaggatgcctgctttctttgaaaccttcccagtaatcctcatt
gaccaaacaagtacagtgagagcagacattgcttttagacggtccacatctacttatagc
atggaagaatcccaaatacaagtatacttctctggtggttgtttaaatggtacagaatat
tctacaccttcccttgtaaaagcttatgcaagaaaagctcagtttggtcagatatttaca
tttgataaaaagacatccagattgactgatggtgtatttagaacatctgctcgaggttgg
tattcattctcacacatagctttagctttctcctttttctttggccatttgtggcatgct
agtcgtgctatcttccaagacatttggacaggtgtaacatttgaatctcaagcaaaacaa
gaatatggtagaaatgaaaagctaggagataagacatcttcaactaaatccattgtttaa
>KrA8_psbB
atgcaaataactgctgttttaccttggtttagggtacatatagttattcttaatgatcca
ggacgtctcattagctcacatattatgcacacagctttagtagcaggttggtcagctctc
atgcttttatatgagcttatcaccatagatcctacagatcctgtgtataatccaatttgg

agacaagcagcttacacactcccatttatctcacgtatcggtgttattcgctctctttttt
agttggtcacttggcatagatcctacatccaatctaatctggacttatgaaacaatgaac
atagcccatattctattatctggtttattaattcttgcatcattttggcattgggcatat
tgggacttagatctattcttcacctcaacattaacactagaccttaatcaaatcctcagt
attcacctcacactagcatcatctctctgtctgggatttggtttagctcatctaactggt
tttcttggtccaggaatgtggactagtgattccctcaatcttgtaggctctattagattc
gtaaaagcctccttcaatctccttgcttatgcacgattagcttatggtgtcatatcatca
catcacatcatctctggattgcttggcacttccataggattatggcatatcacattacgt
ccattagcatacctctacaatctattaagcatgggaaaggttgagtccattctttcaagt
agtattacagctgtattcttcactgcattccttatttcagcccttatgtggtatggctct
gcacacaccacacaagaactcttcggtcctactagatactcatgggacaatgcttattac
tctcttgatatcagaacctcttgctcaaatgcctggaacactcttccagacaagttagtc
ctttatgattatattggatctaaccctgctaaaggtggcttatttcgttcaggaccaatg
cttaaagctgatggtcttgttcaaaactggttgggccatgcatgcttctccatgggtaca
ttatccttaagcatcagaaggatgcctgctttctttgaaaccttcccagtaatcctcatt
gaccaaacaagtacagtgagagcagacattgctttcagacggtccacatctacttatagc
atggaagaatcccaaatacaagtatacttctctggtggttgtttaaatggtacagaatat
tctacaccttcccttgtaaaagcttatgcaagaaaagctcagtttggtcagatatttaca
tttgataaaaagacatccagattgactgatggtgtatttagaacatctgctcgaggttgg
tattcattctcacacatagctttagctttctcctttttctttggccatttgtggcatgct
agtcgtgctatcttccaagacatttggacaggtgtaacatttgaatcacaagcaaaacaa
gaatatggtagaaatgaaaagctaggagataagacatcttcaactaaatccattgtttaa
>KrA9_psbB
atgcaaataactgatgctttaccttggtttagggtacatatagttattcttaatgatcca
ggacgtctcattagctcacatattatgcacacagctttagtagcaggttggtcagctctc
atgcttttatatgagcttatcaccatagatcctacagatcctgtgtataatccaatttgg
agacaagcagcttacacactcccatttatctcacgtattggtgttattcgctctctttttt
agttggtcacttggcatagatcctacatccaatctaatctggacttatgaaacaatgaac
atagcccatattctattatctggtttattaattcttgcatcattttggcattgggcatat
tgggacttagatctattcttcacctcaacattaacactagaccttaatcaaatcctcagt
attcacctcacactagcatcatctctctgtctgggatttggtttagctcatctaactggt
tttcttggtccaggaatgtggactagtgattccctcaatcttgtaggctctattagattc
gtaaaagcctccttcaatctccttgcttatgcacgattagcttatggtgtcatatcatca
catcacatcatctctggattgcttggcacttccataggattatggcatatcacattacgt
ccattagcatacctctacaatctattaagcatgggaaaggttgagtccattctttcaagt
agtattacagctgtattcttcactgcattccttatttcagcccttatgtggtatggctct
gcacacaccacacaagaactcttcggtcctactagatactcatgggacaatgcttattac
tctcttgatatcagaacctcttgctcaaatgcctggaacactcttccagacaagttagtc
ctttatgattatattggatctaaccctgctaaaggtggcttatttcgttcaggaccaatg
cttaaagctgatggtcttgttcaaaactggttgggccatgcatgcttctccatgggtaca
ttatccttaagcatcagaaggatgcctgctttctttgaaaccttcccagtaatcctcatt
gaccaaacaagtacagtgagagcagacattgctttcagacggtccacatctacttatagc
atggaagaatcccaaatacaagtatacttctctggtggttgtttaaatggtacagaatat
tctacaccttcccttgtaaaagcttatgcaagaaaagctcagtttggtcagatatttaca
tttgataaaaagacatccagattgactgatggtgtatttagaacatctgctcgaggttgg
tattcattctcacacatagctttagctttctcctttttctttggccatttgtggcatgct
agtcgtgctatcttccaagacatttggacaggtgtaacatttgaatcacaagcaaaacaa
gaatatggtagaaatgaaaagctaggagataagacatcttcaactaaatccattgtttaa
>KrC1_psbB
atgcaaataactgatgctttaccttggtttagggtacatatagttattcttaatgatcca
ggacgtctcattagctctcacattatgcacacagctttagtagcaggttggtcagctctc
atgcttttatatgagcttatcaccatagatcctacagatcctgtgtataatccaatttgg
agacaagcagcttacacactcccatttatctcacgtattggtgttattcgctctctttttt
agttggtcacttggcatagatcctacatccaatctaatctggacttatgaaacaatgaac
atagcccatattctattatctggtttattaattcttgcatcattttggcattgggcatat
tgggacttagatctattcttcacctcaacattaacactagaccttaatcaaatcctcagt
attcacctcacactagcatcatctctctgtctgggatttggtttagctcatctaactggt

tttcttggtccaggaatgtggactagtgattccctcaatcttgtaggctctattagattc
gtaaaagcctccttcaatctccttgcttatgcacgattagcttatggtgtcatatcatca
catcacatcatctctggattgcttggcacttccataggattatggcatatcacattacgt
ccattagcatacctctacaatctattaagcatgggaaaggttgagtccattctttcaagt
agtattacagctgtattcttcactgcattccttatttcagcccttatgtggtatggctct
gcacacaccacacaagaactcttcggtcctactagatactcatgggacaatgcttattac
tctcttgatatcagaacctcttgctcaaatgcctggaacactcttccagacaagttagtc
ctttatgattatattggatctaaccctgctaaaggtggcttatttcgttcaggaccaatg
cttaaagctgatggtcttgttcaaaactggttgggccatgcatgcttctccatgggtaca
ttatccttaagcatcagaaggatgcctgctttctttgaaaccttcccagtaatcctcatt
gaccaaacaagtacagtgagagcagacattgctttcagacggtccacatctacttatagc
atggaagaatcccaaatacaagtatacttctctggtggttgtttaaatggtacagaatat
tctacaccttcccttgtaaaagcttatgcaagaaaagctcagtttggtcagatatttaca
tttgataaaaagacatccagattgactgatggtgtatttagaacatctgctcgaggttgg
tattcattctcacacatagctttagctttctccttttctttggccatttgtggcatgct
agtcgtgctatcttccaagacatttggacaggtgtaacatttgaatcacaagcaaaacaa
gaatatggtagaaatgaaaagctaggagataagacatcttcaactaaatccattgtttaa
>KrC10_psbB
atgcaaataactgctgttttaccttggtttagggtacatatagttattcttaatgatcca
ggacgtctcattagctcacatattatgcacacagctttagtagcaggttggtcagctctc
atgcttttatatgagcttatcaccatagatcctacagatcctgtgtataatccaatttgg
agacaagcagcttacacactcccatttatctcacgtattggtgttattcgctctcttttt
agttggtcacttggcatagatcctacatccaatctaatctggacttatgaaacaatgaac
atagcccatattctattatctggtttattaattcttgcatcattttggcattgggcatat
tgggacttagatctattcttcacctcaacattaacactagaccttaatcaaatcctcagt
attcacctcacactagcatcatctctctgtctgggatttggtttagctcatctaactggt
tttcttggtccaggaatgtggactagtgattccctcaatcttgtaggctctattagattc
gtaaaagcctccttcaatctccttgcttatgcacgattagcttatggtgtcatatcatca
catcacatcatctctggattgcttggcacttccataggattatggcatatcacattacgt
ccattagcatacctctacaatctattaagcatgggaaaggttgagtccattctttcaagt
agtattacagctgtattcttcactgcattccttatttcagcccttatgtggtatggctct
gcacacaccacacaagaactcttcggtcctactagatactcatgggacaatgcttattac
tctcttgatatcagaacctcttgctcaaatgcctggaacactcttccagacaagttagtc
ctttatgattatattggatctaaccctgctaaaggtggcttatttcgttcaggaccaatg
cttaaagctgatggtcttgttcaaaactggttgggccatgcatgcttctccatgggtaca
ttatccttaagcatcagaaggatgcctgctttctttgaaaccttcccagtaatcctcatt
gaccaaacaagtacagtgagagcagacattgctttcagacggtccacatctacttatagc
atggaagaatcccaaatacaagtatacttctctggtggttgtttaaatggtacagaatat
tctacaccttcccttgtaaaagcttatgcaagaaaagctcagtttggtcagatatttaca
tttgataaaaagacatccagattgactgatggtgtatttagaacatctgctcgaggttgg
tattcattctcacacataggtttagctttcctattcttctttggccatttgtggcatgct
agtcgtgctatcttcaaagacatttggacaggtgtaacatttgaatcacaagcaaaacaa
gaatatggtagaaatgaaaagctaggagataagacatcttcaactaaatccattgtttaa
>KrC11_psbB
atgcaaataactgctgttttaccttggtttagggtacatatagttattcttaatgatcca
ggacgtctcattagctcacatattatgcacacagctttagtagcaggttggtcagctctc
atgcttttatatgagcttatcaccatagatcctacagatcctgtgtataatccaatttgg
agacaagcagcttacacactcccatttatctcacgtattggtgttattcgctctcttttt
agttggtcacttggcatagatcctacatccaatctaatctggacttatgaaacaatgaac
atagcccatattctattatctggtttattaattcttgcatcattttggcattgggcatat
tgggacttagatctattcttcacctcaacattaacactagaccttaatcaaatcctcagt
attcacctcacactagcatcatctctctgtctgggatttggtttagctcatctaactggt
tttcttggtccaggaatgtggactagtgattccctcaatcttgtaggctctattagattc
gtaaaagcctccttcaatctccttgcttatgcacgattagcttatggtgtcatatcatca
catcacatcatctctggattgcttggcacttccataggattatggcatatcacattacgt
ccattagcatacctctacaatctattaagcatgggaaaggttgagtccattctttcaagt
agtattacagctgtattcttcactgcattccttatttcagcccttatgtggtatggctct

gcacacaccacacaagaactcttcggtcctactagatactcatgggacaatgcttattac
tctcttgatatcagaacctcttgctcaaatgcctggaacactcttccagacaagttagtc
ctttatgattatattggatctaaccctgctaaaggtggcttatttcgttcaggaccaatg
cttaaagctgatggtcttgttcaaaactggttgggccatgcatgcttctccatgggtaca
ttatccttaagcatcagaaggatgcctgctttctttgaaaccttcccagtaatcctcatt
gaccaaacaagtacagtgagagcagacattgctttcagacggtccacatctacttatagc
atggaagaatcccaaatacaagtatacttctctggtggttgtttaaatggtacagaatat
tctacaccttcccttgtaaaagcttatgcaagaaaagctcagtttggtcagatatttaca
tttgataaaaagacatccagattgactgatggtgtatttagaacatctgctcgaggttgg
tattcattctcacacatagctttagctttctcctttttctttggccatttgtggcatgct
agtcgtgctatcttccaagacatttggacaggtgtaacatttgaatcacaagcaaaacaa
gaatatggtagaaatgaaaagctaggagataagacatcttcaactaaatccattgtttaa
>KrC12_psbB
atgcaaataactgatgctttaccttggtttagggtacatatagttattcttaatgatcca
ggacgtctcattagctcacatattatgcacacagctttagtagcaggttggtcagctctc
atgcttttatatgagcttatcaccatagatcctacagatcctgtgtataatccaatttgg
agacaagcagcttacacactcccatttatctcacgtattggtgttattcgctctcttttt
agttggtcacttggcatagatcctacatccaatctaatctggacttatgaaacaatgaac
atagcccatattctattatctggtttattaattcttgcatcattttggcattgggcatat
tgggacttagatctattcttcacctcaacattaacactagaccttaatcaaatcctcagt
attcacctcacactagcatcatctctctgtctgggatttggtttagctcatctaactggt
tttcttggtccaggaatgtggactagtgattccctcaatcttgtaggctctattagattc
gtaaaagcctccttcaatatccttgcttatgcacgattagcttatggtgtcatatcatca
catcacatcatctctggattgcttggcacttccataggattatggcatatcacattacgt
ccattagcatacctctacaatctattaagcatgggaaaggttgagtccattctttcaagt
agtattacagctgtattcttcactgcattccttatttcagcccttatgtggtatggctct
gcacacaccacacaagaactcttcggtcctactagatactcatgggacaatgcttattac
tctcttgatatcagaacctcttgctcaaatgcctggaacactcttccagacaagttagtc
ctttatgattatattggatctaaccctgctaaaggtggcttatttcgttcaggaccaatg
cttaaagctgatggtcttgttcaaaactggttgggccatgcatgcttctccatgggtaca
ttatccttaagcatcagaaggatgcctgctttctttgaaaccttcccagtaatcctcatt
gaccaaacaagtacagtgagagcagacattgctttcagacggtccacatctacttatagc
atggaagaatcccaaatacaagtatacttctctggtggttgtttaaatggtacagaatat
tctacaccttcccttgtaaaagcttatgcaagaaaagctcagtttggtcagatatttaca
tttgataaaaagacatccagattgactgatggtgtatttagaacatctgctcgaggttgg
tattcattctcacacatagctttagctttctcctttttctttggccatttgtggcatgct
agtcgtgctatcttccaagacatttggacaggtgtaacatttgaatcacaagcaaaacaa
gaatatggtagaaatgaaaagctaggagataagacatcttcaactaaatccattgtttaa
>KrC13_psbB
atgcaaataactgatgctttaccttggtttagggtacatatagttattcttaatgatcca
ggacgtctcattagctcacatattatgcacacagctttagtagcaggttggtcagctctc
atgcttttatatgagcttatcaccatagatcctacagatcctgtgtataatccaatttgg
agacaagcagcttacacactcccatttatctcacgtattggtgttattcgctctcttttc
agttggtcacttggcatagatcctacatccaatctaatctggacttatgaaacaatgaac
atagcccatattctattatctggtttattaattcttgcatcattttggcattgggcatat
tgggacttagatctattcttcacctcaacattaacactagaccttaatcaaatcctcagt
attcacctcacactagcatcatctctctgtctgggatttggtttagctcatctaactggt
tttcttggtccaggaatgtggactagtgattccctcaatcttgtaggctctattagattc
gtaaaagcctccttcaatctccttgcttatgcacgattagcttatggtgtcatatcatca
catcacatcatctctggattgcttggcacttccataggattatggcatatcacattacgt
ccattagcatacctctacaatctattaagcatgggaaaggttgagtccattctttcaagt
agtattacagctgtattcttcactgcattccttatttcagcccttatgtggtatggctct
gcacacaccacacaagaactcttcggtcctactagatactcatgggacaatgcttattac
tctcttgatatcagaacctcttgctcaaatgcctggaacactcttccagacaagttagtc
ctttatgattatattggatctaaccctgctaaaggtggcttatttcgttcaggaccaatg
cttaaagctgatggtcttgttcaaaactggttgggccatgcatgcttctccatgggtaca
ttatccttaagcatcagaaggatgcctgctttctttgaaaccttcccagtaatcctcatt

gaccaaacaagtacagtgagagcagacattgctttcagacggtccacatctacttatagc
atggaagaatcccaaatacaagtatacttctctggtggttgtttaaatggtacagaatat
tctacaccttcccttgtaaaagcttatgcaagaaaagctcagtttggtcagatatttaca
tttgataaaaagacatccagattgactgatggtgtatttagaacatctgctcgaggttgg
tattcattctcacacatagctttagctttctcctttttctttggccatttgtggcatgct
agtcgtgctatcttccaagacatttggacaggtgtaacatttgaatcacaagcaaaacaa
gaatatggtagaaatgaaaagctaggagataagacatcttcaactaaatccattgtttaa
>KrC14_psbB
atgcaaataactgatgctttaccttggtttagggtacatatagttattcttaatgatcca
ggacgtctcattagctcacatattatgcacacagctttagtagcaggttggtcagctctc
atgcttttatatgagcttatcaccatagatcctacagatcctgtgtataatccaatttgg
agacaagcagcttacacactcccatttatctcacgtattggtgttattcgctctcttttt
agttggtcacttggcatagatcctacatccaatctaatctggacttatgaaacaatgaac
atagcccatattctattatctggtttattaattcttgcatcattttggcattgggcatat
tgggacttagatctattcttcacctcaacattaacactagaccttaatcaaatcctcagt
attcacctcacactagcatcatctctctgtctgggatttggtttagctcatctaactggt
tttcttggtccaggaatgtggactagtgattccctcaatcttgtaggctctattagattc
gtaaaagcctccttcaatctccttgcttatgcacgattagcttatggtgtcatatcatca
catcacatcatctctggattgcttggcacttccataggattatggcatatcacattacgt
ccattagcatacctctacaatctattaagcatgggaaaggttgagtccattctttcaagt
agtattacagctgtattcttcactgcattccttatttcagcccttatgtggtatggctct
gcacacaccacacaagaactcttcggtcctactagatactcatgggacaatgcttattac
tctcttgatatcagaacctcttgctcaaatgcctggaacactcttccagacaagttagtc
ctttatgattatattggatctaaccctgctaaaggtggcttatttcgttcaggaccaatg
cttaaagctgatggtcttgttcaaaactggttgggccatgcatgcttctccatgggtaca
ttatccttaagcatcagaaggatgcctgctttctttgaaaccttcccagtaatcctcatt
gaccaaacaagtacagtgagagcagacattgctttcagacggtccacatctacttatagc
atggaagaatcccaaatacaagtatacttctctggtggttgtttaaatggtacagaatat
tctacaccttcccttgtaaaagcttatgcaagaaaagctcagtttggtcagatatttaca
tttgataaaaagacatccagattgactgatggtgtatttagaacatctgctcgaggttgg
tattcattctcacacatagctttagctttctcctttttctttggccatttgtggcatgct
agtcgtgctatcttccaagacatttggacaggtgtaacatttgaatcacaagcaaaacaa
gaatatggtagaaatgaaaagctaggagataagacatcttcaactaaatccattgtttaa
>KrC15_psbB
atgcaaataactgatgctttaccttggtttagggtacatatagttattcttaatgatcca
ggacgtctcattagctcacatattatgcacacagctttagtagcaggttggtcagctctc
atgcttttatatgagcttatcaccatagatcctacagatcctgtgtataatccaatttgg
agacaagcagcttacacactcccatttatctcacgtattggtgttattcgctctcttttt
agttggtcacttggcatagatcctacatccaatctaatctggacttatgaaacaatgaac
atagcccatattctattatctggtttattaattcttgcatcattttggcattgggcatat
tgggacttagatctattcttcacctcaacattaacactagaccttaatcaaatcctcagt
attcacctcacactagcatcatctctctgtctgggatttggtttagctcatctaactggt
tttcttggtccaggaatgtggactagtgattccctcaatcttgtaggctctattagattc
gtaaaagcctccttcaatctccttgcttatgcacgattagcttatggtgtcatatcatca
catcacatcatctctggattgcttggcacttccataggattatggcatatcacattacgt
ccattagcatacctctacaatctattaagcatgggaaaggttgagtccattctttcaagt
agtattacagctgtattcttcactgcattccttatttcagcccttatgtggtatggctct
gcacacaccacacaagaactcttcggtcctactagatactcatgggacaatgcttattac
tctcttgatatcagaacctcttgctcaaatgcctggaacactcttccagacaagttagtc
ctttatgattatattggatctaaccctgctaaaggtggcttatttcgttcaggaccaatg
cttaaagctgatggtcttgttcaaaactggttgggccatgcatgcttctccatgggtaca
ttatccttaagcatcagaaggatgcctgctttctttgaaaccttcccagtaatcctcatt
gaccaaacaagtacagtgagagcagacattgctttcagacggtccacatctacttatagc
atggaagaatcccaaatacaagtatacttctctggtggttgtttaaatggtacagaatat
tctacaccttcccttgtaaaagcttatgcaagaaaagctcagtttggtcagatatttaca
tttgataaaaagacatccagattgactgatggtgtatttagaacatctgctcgaggttgg
tattcattctcacacatagctttagctttctcctttttctttggccatttgtggcatgct

agtcgtgctatcttccaagacatttggacaggtgtaacatttgaatcacaagcaaaacaa
gaatatggtagaaatgaaaagctaggagataagacatcttcaactaaatccattgtttaa
>KrC2_psbB
atgcaaataactgatgctttaccttggtttagggtacatatagttattcttaatgatcca
ggacgtctcattagctcacatattatgcacacagctttagtagcaggttggtcagctctc
atgcttttatatgagcttatcaccatagatcctacagatcctgtgtataatccaatttgg
agacaagcagcttacacactcccatttatctcacgtattggtgttattcgctctcttttt
agttggtcacttggcatagatcctacatccaatctaatctggacttatgaaacaatgaac
atagcccatattctattatctggtttattaattcttgcatcattttggcattgggcatat
tgggacttagatctattcttcacctcaacattaacactagaccttaatcaaatcctcagt
attcacctcacactagcatcatctctctgtctgggatttggtttagctcatctaactggt
tttcttggtccaggaatgtggactagtgattccctcaatcttgtaggctctattagattc
gtaaaagcctccttcaatctccttgcttatgcacgattagcttatggtgtcatatcatca
catcacatcatctctggattgcttggcacttccataggattatggcatatcacattacgt
ccattagcatacctctacaatctattaagcatgggaaaggttgagtccattctttcaagt
agtattacagctgtattcttcactgcattccttatttcagcccttatgtggtatggctct
gcacacaccacacaagaactcttcggtcctactagatactcatgggacaatgcttattac
tctcttgatatcagaacctcttgctcaaatgcctggaacactcttccagacaagttagtc
ctttatgattatattggatctaaccctgctaaaggtggcttatttcgttcaggaccaatg
cttaaagctgatggtcttgttcaaaactggttgggccatgcatgcttctccatgggtaca
ttatccttaagcatcagaaggatgcctgctttctttgaaaccttcccagtaatcctcatt
gaccaaacaagtacagtgagagcagacattgctttcagacggtccacatctacttatagc
atggaagaatcccaaatacaagtatacttctctggtggttgtttaaatggtacagaatat
tctacaccttcccttgtaaaagcttatgcaagaaaagctcagtttggtcagatatttaca
tttgataaaaagacatccagattgactgatggtgtatttagaacatctgctcgaggttgg
tattcattctcacacatagctttagctttctcctttttctttggccatttgtggcatgct
agtcgtgctatcttccaagacatttggacaggtgtaacatttgaatcacaagcaaaacaa
gaatatggtagaaatgaaaagctaggagataagacatcttcaactaaatccattgtttaa
>KrC3_psbB
atgcaaataactgatgctttaccttggtttagggtacatatagttattcttaatgatcca
ggacgtctcattagctcacatattatgcacacagctttagtagcaggttggtcagctctc
atgcttttatatgagcttatcaccatagatcctacagatcctgtgtataatccaatttgg
agacaagcagcttacacactcccatttatctcacgtattggtgttattcgctctcttttt
agttggtcacttggcatagatcctacatccaatctaatctggacttatgaaacaatgaac
atagcccatattctattatctggtttattaattcttgcatcattttggcattgggcatat
tgggacttagatctattcttcacctcaacattaacactagaccttaatcaaatcctcagt
attcacctcacactagcatcatctctctgtctgggatttggtttagctcatctaactggt
tttcttggtccaggaatgtggactagtgattccctcaatcttgtaggctctattagattc
gtaaaagcctccttcaatctccttgcttatgcacgattagcttatggtgtcatatcatca
catcacatcatctctggattgcttggcacttccataggattatggcatatcacattacgt
ccattagcatacctctacaatctattaagcatgggaaaggttgagtccattctttcaagt
agtattacagctgtattcttcactgcattccttatttcagcccttatgtggtatggctct
gcacacaccacacaagaactcttcggtcctactagatactcatgggacaatgcttattac
tctcttgatatcagaacctcttgctcaaatgcctggaacactcttccagacaagttagtc
ctttatgattatattggatctaaccctgctaaaggtggcttatttcgttcaggaccaatg
cttaaagctgatggtcttgttcaaaactggttgggccatgcatgcttctccatgggtaca
ttatccttaagcatcagaaggatgcctgctttctttgaaaccttcccagtaatcctcatt
gaccaaacaagtacagtgagagcagacattgctttcagacggtccacatctacttatagc
atggaagaatcccaaatacaagtatacttctctggtggttgtttaaatggtacagaatat
tctacaccttcccttgtaaaagcttatgcaagaaaagctcagtttggtcagatatttaca
tttgataaaaagacatccagattgactgatggtgtatttagaacatctgctcgaggttgg
tattcattctcacacatagctttagctttctcctttttctttggccatttgtggcatgct
agtcgtgctatcttccaagacatttggacaggtgtaacatttgaatcacaagcaaaacaa
gaatatggtagaaatgaaaagctaggagataagacatcttcaactaaatccattgtttaa
>KrC4_psbB
atgcaaataactgatgctttaccttggtttagggtacatatagttattcttaatgatcca
ggacgtctcattagctcacatattatgcacacagctttagtagcaggttggtcagctctc

atgcttttatatgagcttatcaccatagatcctacagatcctgtgtataatccaatttgg
agacaagcagcttacacactcccatttatctcacgtattggtgttattcgctctcttttt
agttggtcacttggcatagatcctacatccaatctaatctggacttatgaaacaatgaac
atagcccatattctattatctggtttattaattcttgcatcattttggcattgggcatat
tgggacttagatctattcttcacctcaacattaacactagaccttaatcaaatcctcagt
attcacctcacactagcatcatctctctgtctgggatttggtttagctcatctaactggt
tttcttggtccaggaatgtggactagtgattccctcaatcttgtaggctctattagattc
gtaaaagcctccttcaatctccttgcttatgcacgattagcttatggtgtcatatcatca
catcacatcatctctggattgcttggcacttccataggattatggcatatcacattacgt
ccattagcatacctctacaatctattaagcatgggaaaggttgagtccattctttcaagt
agtattacagctgtattcttcactgcattccttatttcagcccttatgtggtatggctct
gcacacaccacacaagaactcttcggtcctactagatactcatgggacaatgcttattac
tctcttgatatcagaacctcttgctcaaatgcctggaacactcttccagacaagttagtc
ctttatgattatattggatctaaccctgctaaaggtggcttatttcgttcaggaccaatg
cttaaagctgatggtcttgttcaaaactggttgggccatgcatgcttctccatgggtaca
ttatccttaagcatcagaaggatgcctgctttctttgaaaccttcccagtaatcctcatt
gaccaaacaagtacagtgagagcagacattgctttcagacggtccacatctacttatagc
atggaagaatcccaaatacaagtatacttctctggtggttgtttaaatggtacagaatat
tctacaccttcccttgtaaaagcttatgcaagaaaagctcagtttggtcagatatttaca
tttgataaaaagacatccagattgactgatggtgtatttagaacatctgctcgaggttgg
tattcattctcacacatagctttagctttctcctttttctttggccatttgtggcatgct
agtcgtgctatcttccaagacatttggacaggtgtaacatttgaatcacaagcaaaacaa
gaatatggtagaaatgaaaagctaggagataagacatcttcaactaaatccattgtttaa
>KrC5_psbB
atgcaaataactgctgttttaccttggtttagggtacatatagttattcttaatgatcca
ggacgtctcattagctcacatattatgcacacagctttagtagcaggttggtcagctctc
atgcttttatatgagcttatcaccatagatcctacagatcctgtgtataatccaatttgg
agacaagcagcttacacactcccatttatctcacgtattggtgttattcgctctcttttt
agttggtcacttggcatagatcctacatccaatctaatctggacttatgaaacaatgaac
atagcccatattctattatctggtttattaattcttgcatcattttggcattgggcatat
tgggacttagatctattcttcacctcaacattaacactagaccttaatcaaatcctcagt
attcacctcacactagcatcatctctctgtctgggatttggtttagctcatctaactggt
tttcttggtccaggaatgtggactagtgattccctcaatcttgtaggctctattagattc
gtaaaagcctccttcaatctccttgcttatgcacgattagcttatggtgtcatatcatca
catcacatcatctctggattgcttggcacttccataggattatggcatatcacattacgt
ccattagcatacctctacaatctattaagcatgggaaaggttgagtccattctttcaagt
agtattacagctgtattcttcactgcattccttatttcagcccttatgtggtatggctct
gcacacaccacacaagaactcttcggtcctactagatactcatgggacaatgcttattac
tctcttgatatcagaacctcttgctcaaatgcctggaacactcttccagacaagttagtc
ctttatgattatattggatctaaccctgctaaaggtggcttatttcgttcaggaccaatg
cttaaagctgatggtcttgttcaaaactggttgggccatgcatgcttctccatgggtaca
ttatccttaagcatcagaaggatgcctgctttctttgaaaccttcccagtaatcctcatt
gaccaaacaagtacagtgagagcagacattgctttcagacggtccacatctacttatagc
atggaagaatcccaaatacaagtatacttctctggtggttgtttaaatggtacagaatat
tctacaccttcccttgtaaaagcttatgcaagaaaagctcagtttggtcagatatttaca
tttgataaaaagacatccagattgactgatggtgtatttagaacatctgctcgaggttgg
tattcattctcacacatagctttagctttctcctttttctttggccatttgtggcatgct
agtcgtgctatcttccaagacatttggacaggtgtaacatttgaatcacaagcaaaacaa
gaatatggtagaaatgaaaagctaggagataagacatcttcaactaaatccattgtttaa
>KrC6_psbB
atgcaaataactgatgctttaccttggtttagggtacatatagttattcttaatgatcca
ggacgtctcattagctcacatattatgcacacagctttagtagcaggttggtcagctctc
atgcttttatatgagcttatcaccatagatcctacagatcctgtgtataatccaatttgg
agacaagcagcttacacactcccatttatctcacgtattggtgttattcgctctcttttt
agttggtcacttggcatagatcctacatccaatctaatctggacttatgaaacaatgaac
atagcccatattctattatctggtttattaattcttgcatcattttggcattgggcatat
tgggacttagatctatccttcacctcaacattaacactagaccttaatcaaatcctcagt

attcacctcacactagcatcatctctctgtctgggatttggtttagctcatctaactggt
tttcttggtccaggaatgtggactagtgattccctcaatcttgtaggctctattagattc
gtaaaagcctccttcaatctccttgcttatgcacgattagcttatggtgtcatatcatca
catcacatcatctctggattgcttggcacttccataggattatggcatatcacattacgt
ccattagcatacctctacaatctattaagcatgggaaaggttgagtccattctttcaagt
agtattacagctgtattcttcactgcattccttatttcagcccttatgtggtatggctct
gcacacaccacacaagaactcttcggtcctactagatactcatgggacaatgcttattac
tctcttgatatcagaacctcttgctcaaatgcctggaacactcttccagacaagttagtc
ctttatgattatattggatctaaccctgctaaaggtggcttatttcgttcaggaccaatg
cttaaagctgatggtcttgttcaaaactggttgggccatgcatgcttctccatgggtaca
ttatccttaagcatcagaaggatgcctgctttctttgaaaccttcccagtaatcctcatt
gaccaaacaagtacagtgagagcagacattgctttcagacggtccacatctacttatagc
atggaagaatcccaaatacaagtatacttctctggtggttgtttaaatggtacagaatat
tctacaccttcccttgtaaaagcttatgcaagaaaagctcagtttggtcagatatttaca
tttgataaaaagacatccagattgactgatggtgtatttagaacatctgctcgaggttgg
tattcattctcacacatagctttagctttctcctttttctttggccatttgtggcatgct
agtcgtgctatcttccaagacatttggacaggtgtaacatttgaatcacaagcaaaacaa
gaatatggtagaaatgaaaagctaggagataagacatcttcaactaaatccattgtttaa
>KrC7_psbB
atgcaaataactgatgctttaccttggtttagggtacatatagttattcttaatgatcca
ggacgtctcattagctcacatattatgcacacagctttagtagcaggttggtcagctctc
atgcttttatatgagcttatcaccatagatcctacagatcctgtgtataatccaatttgg
agacaagcagcttacacactcccatttatctcacgtattggtgttattcgctctcttttt
agttggtcacttggcatagatcctacatccaatctaatctggacttatgaaacaatgaac
atagcccatattctattatctggtttattaattcttgcatcattttggcattgggcatat
tgggacttagatctattcttcacctcaacattaacactagaccttaatcaaatcctcagt
attcacctcacactagcatcatctctctgtctgggatttggtttagctcatctaactggt
tttcttggtccaggaatgtggactagtgattccctcaatcttgtaggctctattagattc
gtaaaagcctccttcaatctccttgcttatgcacgattagcttatggtgtcatatcatca
catcacatcatctctggattgcttggcacttccataggattatggcatatcacattacgt
ccattagcatacctctacaatctattaagcatgggaaaggttgagtccattctttcaagt
agtattacagctgtattcttcactgcattccttatttcagcccttatgtggtatggctct
gcacacaccacacaagaactcttcggtcctactagatactcatgggacaatgcttattac
tctcttgatatcagaacctcttgctcaaatgcctggaacactcttccagacaagttagtc
ctttatgattatattggatctaaccctgctaaaggtggcttatttcgttcaggaccaatg
cttaaagctgatggtcttgttcaaaactggttgggccatgcatgcttctccatgggtaca
ttatccttaagcatcagaaggatgcctgctttctttgaaaccttcccagtaatcctcatt
gaccaaacaagtacagtgagagcagacattgctttcagacggtccacatctacttatagc
atggaagaatcccaaatacaagtatacttctctggtggttgtttaaatggtacagaatat
tctacaccttcccttgtaaaagcttatgcaagaaaagctcagtttggtcagatatttaca
tttgataaaaagacatccagattgactgatggtgtatttagaacatctgctcgaggttgg
tattcattctcacacatagctttagctttctcctttttctttggccatttgtggcatgct
agtcgtgctatcttccaagacatttggacaggtgtaacatttgaatcacaagcaaaacaa
gaatatggtagaaatgaaaagctaggagataagacatcttcaactaaatccattgtttaa
>KrC8_psbB
atgcaaataactgatgctttaccttggtttagggtacatatagttattcttaatgatcca
ggacgtctcattagctcacatattatgcacacagctttagtagcaggttggtcagctctc
atgcttttatatgagcttatcaccatagatcctacagatcctgtgtataatccaatttgg
agacaagcagcttacacactcccatttatctcacgtattggtgttattcgctctcttttt
agttggtcacttggcatagatcctacatccaatctaatctggacttatgaaacaatgaac
atagcccatattctattatctggtttattaattcttgcatcattttggcattgggcatat
tgggacttagatctattcttcacctcaacattaacactagaccttaatcaaatcctcagt
attcacctcacactagcatcatctctctgtctgggatttggtttagctcatctaactggt
tttcttggtccaggaatgtggactagtgattccctcaatcttgtaggctctattagattc
gtaaaagcctccttcaatctccttgcttatgcacgattagcttatggtgtcatatcatca
catcacatcatctctggattgcttggcacttccataggattatggcatatcacattacgt
ccattagcatacctctacaatctattaagcatgggaaaggttgagtccattctttcaagt

agtattacagctgtattcttcactgcattccttatttcagcccttatgtggtatggctct
gcacacaccacacaagaactcttcggtcctactagatactcatgggacaatgcttattac
tctcttgatatcagaacctcttgctcaaatgcctggaacactcttccagacaagttagtc
ctttatgattatattggatctaaccctgctaaaggtggcttatttcgttcaggaccaatg
cttaaagctgatggtcttgttcaaaactggttgggccatgcatgcttctccatgggtaca
ttatccttaagcatcagaaggatgcctgctttctttgaaaccttcccagtaatcctcatt
gaccaaacaagtacagtgagagcagacattgctttcagacggtccacatctacttatagc
atggaagaatcccaaatacaagtatacttctctggtggttgtttaaatggtacagaatat
tctacaccttcccttgtaaaagcttatgcaagaaaagctcagtttggtcagatatttaca
tttgataaaaagacatccagattgactgatggtgtatttagaacatctgctcgaggttgg
tattcattctcacacatagctttagctttctcctttttctttggccatttgtggcatgct
agtcgtgctatcttccaagacatttggacaggtgtaacatttgaatcacaagcaaaacaa
gaatatggtagaaatgaaaagctaggagataagacatcttcaactaaatccattgtttaa
>KrC9_psbB
atgcaaataactgatgctttaccttggtttagggtacatatagttattcttaatgatcca
ggacgtctcattagctcacatattatgcacacagctttagtagcaggttggtcagctctc
atgcttttatatgagcttatcaccatagatcctacagatcctgtgtataatccaatttgg
agacaagcagcttacacactcccatttatctcacgtattggtgttattcgctctcttttt
agttggtcacttggcatagatcctacatccaatctaatctggacttatgaaacaatgaac
atagcccatattctattatctggtttattaattcttgcatcattttggcattgggcatat
tgggacttagatctattcttcacctcaacattaacactagaccttaatcaaatcctcagt
attcacctcacactagcatcatctctctgtctgggatttggtttagctcatctaactggt
tttcttggtccaggaatgtggactagtgattccctcaatcttgtaggctctattagattc
gtaaaagcctccttcaatctccttgcttatgcacgattagcttatggtgtcatatcatca
catcacatcatctctggattgcttggcacttccataggattatggcatatcacattacgt
ccattagcatacctctacaatctattaagcatgggaaaggttgagtccattctttcaagt
agtattacagctgtattcttcactgcattccttatttcagcccttatgtggtatggctct
gcacataccacacaagaactattcggtcctactagatactcatgggacaatgcttattac
tctcttgatatcagaacctcttgctcaaatgcctggaacactcttccagacaagttagtc
ctttatgattatattggatctaaccctgctaaaggtggcttatttcgttcaggaccaatg
cttaaagctgatggtcttgttcaaaactggttgggccatgcatgcttctccatgggtaca
ttatccttaagcatcagaaggatgcctgctttctttgaaaccttcccagtaatcctcatt
gaccaaacaagtacagtgagagcagacattgctttcagacggtccacatctacttatagc
atggaagaatcccaaatacaagtatacttctctggtggttgtttaaatggtacagaatat
tctacaccttcccttgtaaaagcttatgcaagaaaagctcagtttggtcagatatttaca
tttgataaaaagacatccagattgactgatggtgtatttagaacatctgctcgaggttgg
tattcattctcacacatagctttagctttctcctttttctttggccatttgtggcatgct
agtcgtgctatcttccaagacatttggacaggtgtaacatttgaatcacaagcaaaacaa
gaatatggtagaaatgaaaagctaggagataagacatcttcaactaaatccattgtttaa
>KrD1_psbB
atgcaaataactgatgctttaccttggtttagggtacatatagttattcttaatgatcca
ggacgtctcattagctcacatattatgcacacagctttagtagcaggttggtcagctctc
atgcttttatatgagcttatcaccatagatcctacagatcctgtgtataatccaatttgg
agacaagcagcttacacactcccatttatctctcgtattggtgttattcgctctcttttt
agttggtcacttggcatagatcctacatccaatctaatctggacttatgaaacaatgaac
atagcccatattctattatctggtttattaattcttgcatcattttggcattgggcatat
tgggacttagatctattcttcacctcaacattaacactagaccttaatcaaatcctcagt
attcacctcacactagcatcatctctctgtctgggatttggtttagctcatctaactggt
tttcttggtccaggaatgtggactagtgattccctcaatcttgtaggctctattagattc
gtaaaagcctccttcaatctccttgcttatgcacgattagcttatggtgtcatatcatca
catcacatcatctctggattgcttggcacttccataggattatggcatatcacattacgt
ccattagcatacctctacaatctattaagcatgggaaaggttgagtccattctttcaagt
agtattacagctgtattcttcactgcattccttatttcagcccttatgtggtatggctct
gcacacaccacacaagaactcttcggtcctactagatactcatgggacaatgcttattac
tctcttgatatcagaacctcttgctcaaatgcctggaacactcttccagacaagttagtc
ctttatgattatattggatctaaccctgctaaaggtggcttatttcgttcaggaccaatg
cttaaagctgatggtcttgttcaaaactggttgggccatgcatgcttctccatgggtaca

ttatccttaagcatcagaaggatgcctgctttctttgaaaccttcccagtaatcctcatt
gaccaaacaagtacagtgagagcagacattgctttcagacggtccacatctacttatagc
atggaagaatcccaaatacaagtatacttctctggtggttgtttaaatggtacagaatat
tctacaccttcccttgtaaaagcttatgcaagaaaagctcagtttggtcagatatttaca
tttgataaaaagacatccagattgactgatggtgtatttagaacatctgctcgaggttgg
tattcattctcacacatagctttagctttctcctttttctttggccatttgtggcatgct
agtcgtgctatcttccaagacatttggacaggtgtaacatttgaatcacaagcaaaacaa
gaatatggtagaaatgaaaagctaggagataagacatcttcaactaaatccattgtttaa
>KrD11_psbB
atgcaaataactgatgctttaccttggtttagggtacatatagttattcttaatgatcca
ggacgtctcattagctcacatattatgcacacagctttagtagcaggttggtcagctctc
atgcttttatatgagcttatcaccatagatcctacagatcctgtgtataatccaatttgg
agacaagcagcttacacactcccatttatctcacgtattggtgttattcgctctcttttt
agttggtcacttggcatagatcctacatccaatctaatctggacttatgaaacaatgaac
atagcccatattctattatctggtttattaattcttgcatcattttggcattgggcatat
tgggacttagatctattcttcacctcaacattaacactagaccttaatcaaatcctcagt
attcacctcacactagcatcatctctctgtctgggatttggtttagctcatctaactggt
tttcttggtccaggaatgtggactagtgattccctcaatcttgtaggctctattagattc
gtaaaagcctccttcaatctccttgcttatgcacgattagcttatggtgtcatatcatca
catcacatcatctctggattgcttggcacttccataggattatggcatatcacattacgt
ccattagcatacctctacaatctattaagcatgggaaaggttgagtccattctttcaagt
agtattacagctgtattcttcactgcattccttatttcagcccttatgtggtatggctct
gcacacaccacacaagaactcttcggtcctactagatactcatgggacaatgcttattac
tctcttgatatcagaacctcttgctcaaatgcctggaacactcttccagacaagttagtc
ctttatgattatattggatctaaccctgctaaaggtggcttatttcgttcaggaccaatg
cttaaagctgatggtcttgttcaaaactggttaggtcatgcatgtttctccatgggtaca
ttatccttaagcatcagaaggatgcctgctttctttgaaaccttcccagtaatcctcatt
gaccaaacaagtacagtgagagcagacattgctttcagacggtccacatctacttatagc
atggaagaatcccaaatacaagtatacttctctggtggttgtttaaatggtacagaatat
tctacaccttcccttgtaaaagcttatgcaagaaaagctcagtttggtcagatatttaca
tttgataaaaagacatccagattgactgatggtgtatttagaacatctgctcgaggttgg
tattcattctcacacatagctttagctttcttctttttctttggccatttgtggcatgct
agtcgtgctatcttcaaagacatttggacaggtgtaacatttgaatctcaagcaaaacaa
gaatatggtagaaatgaaaagctaggagataagacatcttcaactaaatccattgtttaa
>KrD13_psbB
atgcaaataactgatgttttaccttggtttagggtacatatagttattcttaatgatcca
ggacgtctcattagctcacatattatgcacacagctttagtagcaggttggtcagctctc
atgcttttatatgagcttatcaccatagatcctacagatcctgtgtataatccaatttgg
agacaagcagcttacacactcccatttatctcacgtattggtgttattcgctctcttttt
agttggtcacttggcatagatcctacatccaatctaatctggacttatgaaacaatgaac
atagcccatattctattatctggtttattaattcttgcatcattttggcattgggcatat
tgggacttagatctattcttcacctcaacattaacactagaccttaatcaaatcctcagt
attcacctcacactagcatcatctctctgtctgggatttggtttagctcatctaactggt
tttcttggtccaggaatgtggactagtgattccctcaatcttgtaggctctattagattc
gtaaaagcctccttcaatctccttgcttatgcacgattagcttatggtgtcatatcatca
catcacatcatctctggattgcttggcacttccataggattatggcatatcacattacgt
ccattagcatacctctacaatctattaagcatgggaaaggttgagtccattctttcaagt
agtattacagctgtattcttcactgcattccttatttcagcccttatgtggtatggctct
gcacacaccacacaagaactcttcggtcctactagatactcatgggacaatgcttattac
tctcttgatatcagaacctcttgctcaaatgcctggaacactcttccagacaagttagtc
ctttatgattatattggatctaaccctgctaaaggtggcttatttcgttcaggaccaatg
cttaaagctgatggtcttgttcaaaactggttgggccatgcatgcttctccatgggtaca
ttatccttaagcatcagaaggatgcctgctttctttgaaaccttcccagtaatcctcatt
gaccaaacaagtacagtgagagcagacattgctttcagacggtccacatctacttatagc
atggaagaatcccaaatacaagtatacttctctggtggttgtttaaatggtacagaatat
tctacaccttcccttgtaaaagcttatgcaagaaaagctcagtttggtcagatatttaca
tttgataaaaagacatccagattgactgatggtgtatttagaacatctgctcgaggttgg

tattcattctcacacatagctttagctttctccttttctttggccatttgtggcatgct
agtcgtgctatcttccaagacatttggacaggtgtaacatttgaatcacaagcaaaacaa
gaatatggtagaaatgaaaagctaggagataagacatcttcaactaaatccattgtttaa
>KrD3_psbB
atgcaaataactgatgctttaccttggtttagggtacatatagttattcttaatgatcca
ggacgtctcattagctcacatattatgcacacagctttagtagcaggttggtcagctctc
atgcttttatatgagcttatcaccatagatcctacagatcctgtgtataatccaatttgg
agacaagcagcttacacactcccatttatctctcgtattggtgttattcgctctcttttt
agttggtcacttggcatagatcctacatccaatctaatctggacttatgaaacaatgaac
atagcccatattctattatctggtttattaattcttgcatcattttggcattgggcatat
tgggacttagatctattcttcacctcaacattaacactagaccttaatcaaatcctcagt
attcacctcacactagcatcatctctctgtctgggatttggtttagctcatctaactggt
tttcttggtccaggaatgtggactagtgattccctcaatcttgtaggctctattagattc
gtaaaagcctccttcaatctccttgcttatgcacgattagcttatggtgtcatatcatca
catcacatcatctctggattgcttggcacttccataggattatggcatatcacattacgt
ccattagcatacctctacaatctattaagcatgggaaaggttgagtccattctttcaagt
agtattacagctgtattcttcactgcattccttatttcagcccttatgtggtatggctct
gcacacaccacacaagaactcttcggtcctactagatactcatgggacaatgcttattac
tctcttgatatcagaacctcttgctcaaatgcctggaacactcttccagacaagttagtc
ctttatgattatattggatctaaccctgctaaaggtggcttatttcgttcaggaccaatg
cttaaagctgatggtcttgttcaaaactggttgggccatgcatgcttctccatgggtaca
ttatccttaagcatcagaaggatgcctgctttctttgaaaccttcccagtaatcctcatt
gaccaaacaagtacagtgagagcagacattgctttcagacggtccacatctacttatagc
atggaagaatcccaaatacaagtatacttctctggtggttgtttaaatggtacagaatat
tctacaccttcccttgtaaaagcttatgcaagaaaagctcagtttggtcagatatttaca
tttgataaaaagacatccagattgactgatggtgtatttagaacatctgctcgaggttgg
tattcattctcacacatagctttagctttcctattcttctttggccatttgtggcatgct
agtcgtgctatcttccaagacatttggacaggtgtaacatttgaatcacaagcaaaacaa
gaatatggtagaaatgaaaagctaggagataagacatcttcaactaaatccattgtttaa
>KrD8_psbB
atgcaaataactgatgctttaccttggtttagggtacatatagttattcttaatgatcca
ggacgtctcattagctcacatattatgcacacagctttagtagcaggttggtcagctctc
atgcttttatatgagcttatcaccatagatcctacagatcctgtgtataatccaatttgg
agacaagcagcttacacactcccatttatctctcgtatcggtgttattcgctctcttttc
agttggtcacttggcatagatcctacatccaatctaatctggacttatgaaacaatgaac
atagcccatattctattatctggtttattaattcttgcatcattttggcattgggcatat
tgggacttagatctattcttcacctcaacattaacactagaccttaatcaaatcctcagt
attcacctcacactagcatcatctctctgtctgggatttggtttagctcatctaactggt
tttcttggtccaggaatgtggactagtgattccctcaatcttgtaggctctattagattc
gtaaaagcctccttcaatctccttgcttatgcacgattagcttatggtgtcatatcatca
catcacatcatctctggattgcttggcacttccataggattatggcatatcacattacgt
ccattagcatacctctacaatctattaagcatgggaaaggttgagtccattctttcaagt
agtattacagctgtattcttcactgcattccttatttcagccctgatgtggtatggttct
gcacataccacacaagaactcttcggtcctactagatactcatgggacaatgcttattac
tctcttgatatcagaacctcttgctcaaatgcctggaacactcttccagacaagttagtc
ctttatgattatattggatctaaccctgctaaaggtggcttatttcgttcaggaccaatg
cttaaagctgatggtcttgttcaaaactggttaggtcatgcatgtttctccatgggtaca
ttatccttaatcatcagaaggatgcctgctttctttgaaaccttcccagtaatcctcatt
gaccaaacaagtacagtgagagcagacattgctttcagacggtccacatctacttatagc
atggaagaatcccaaatacaagtatacttctctggtggttgtttaaatggtacagaatat
tctacaccttcccttgtaaaagcttatgcaagaaaagctcagtttggtcagatatttaca
tttgataaaaagacatccagattgactgatggtgtatttagaacatcttctcgaggttgg
tattcattctcacacatagctttagctttctccttttctttggccatttgtggcatgct
agtcgtgctatcttccaagacatttggacaggtgtaacatttgaatcacaagcaaaacaa
gaatatggtagaaatgaaaagctaggagataagacatcttcaactaaatccattgtttaa
>KrD9_psbB
atgcaaataactgatgctttaccttggtttagggtacatatagttattcttaatgatcca

ggacgtctcattagctctcacattatgcatacagctttagtagctgggtggtcagctctc
atgcttttatatgagcttatcaccatagatcctacagatcctgtgtataatccaatttgg
agacaagcagcttacacactcccatttatctcacgtattggtgttattcgctctctttttt
agttggtcacttggcatagatcctacatccaatctaatctggacttatgaaacaatgaac
atagcccatattctattatctggtttattaattcttgcatcattttggcattgggcatat
tgggacttagatctattcttcacctcaacattaacactagaccttaatcaaatcctcagt
attcacctcacactagcatcatctctctgtctgggatttggtttagctcatctaacaggt
tttcttggtccaggaatgtggactagtgattccctcaatcttgtaggctctattagattc
gtaaaagcctccttcaatatcctttcttatgcacgattagcttatggtgtcatatcatca
catcacatcatctctggattgcttggcacttccataggattatggcatatcacattacgt
ccattagcatacctctacaatctattaagcatgggaaaggttgagtccattctttcaagt
agtattacagctgtattcttcactgcattccttatttcagcccttatgtggtatggctct
gcacacaccacacaagaactcttcggtcctactagatactcatgggacaatgcttattac
tctcttgatatcagaacctcttgctcaaatgcctggaacactcttccagacaagttagtc
ctttatgattatattggatctaaccctgctaaaggtggcttatttcgttcaggaccaatg
cttaaagctgatggtcttgttcaaaactggttaggtcatgcatgtttctccatgggtaca
ttatccttaagcatcagaaggatgcctgctttctttgaaaccttcccagtaatcctcatt
gaccaaacaagtacagtgagagcagacattgctttcagacggtccacatctacttatagc
atggaagaatcccaaatacaagtatacttctctggtggttgtttaaatggtacagaatat
tctacaccttcccttgtaaaagcttatgcaagaaaagctcagtttggtcagatatttaca
tttgataaaaagacatccagattgactgatggtgtatttagaacatctgctcgaggttgg
tattcattctcacacatagctttagctttctcctttttctttggccatttgtggcatgct
agtcgtgctatcttcaaagacatttggacaggtgtaacatttgaatcacaagcaaaacaa
gaatatggtagaaatgaaaagctaggagataagacatcttcaactaaatccattgtttaa
>KrE1_psbB
atgcaaataactgatgctttaccttggtttagggtacatatagttattcttaatgatcca
ggacgtctcattagctcacatattatgcacacagctttagtagcaggttggtcagctctc
atgcttttatatgagcttatcaccatagatcctacagatcctgtgtataatccaatttgg
agacaagcagcttacacactcccatttatctcacgtattggtgttattcgctctctttttt
agttggtcacttggcatagatcctacatccaatctaatctggacttatgaaacaatgaac
atagcccatattctattatctggtttattaattcttgcatcattttggcattgggcatat
tgggacttagatctattcttcacctcaacattaacactagaccttaatcaaatcctcagt
attcacctcacactagcatcatctctctgtctgggatttggtttagctcatctaactggt
tttcttggtccaggaatgtggactagtgattccctcaatcttgtaggctctattagattc
gtaaaagcctccttcaatctccttgcttatgcacgattagcttatggtgtcatatcatca
catcacatcatctctggattgcttggcacttccataggattatggcatatcacattacgt
ccattagcatacctctacaatctattaagcatgggaaaggttgagtccattctttcaagt
agtattacagctgtattcttcactgcattccttatttcagcccttatgtggtatggctct
gcacacaccacacaagaactcttcggtcctactagatactcatgggacaatgcttattac
tctcttgatatcagaacctcttgctcaaatgcctggaacactcttccagacaagttagtc
ctttatgattatattggatctaaccctgctaaaggtggcttatttcgttcaggaccaatg
cttaaagctgatggtcttgttcaaaactggttgggccatgcatgcttctccatgggtaca
ttatccttaagcatcagaaggatgcctgctttctttgaaaccttcccagtaatcctcatt
gaccaaacaagtacagtgagagcagacattgctttcagacggtccacatctacttatagc
atggaagaatcccaaatacaagtatacttctctggtggttgtttaaatggtacagaatat
tctacaccttcccttgtaaaagcttatgcaagaaaagctcagtttggtcagatatttaca
tttgataaaaagacatccagattgactgatggtgtatttagaacatctgctcgaggttgg
tattcattctcacacatagctttagctttctcctttttctttggccatttgtggcatgct
agtcgtgctatcttccaagacatttggacaggtgtaacatttgaatcacaagcaaaacaa
gaatatggtagaaatgaaaagctaggagataagacatcttcaactaaatccattgtttaa
>KrE10_psbB
atgcaaataactgatgctttaccttggtttagggtacatatagttattcttaatgatcca
ggacgtctcattagctcacatattatgcacacagctttagtagcaggttggtcagctctc
atgcttttatatgagcttatcaccatagatcctacagatcctgtgtataatccaatttgg
agacaagcagcttacacactcccatttatctcacgtattggtgttattcgctctctttttt
agttggtcacttggcatagatcctacatccaatctaatctggacttatgaaacaatgaac
atagcccatattctattatctggtttattaattcttgcatcattttggcattgggcatat

tgggacttagatctattcttcacctcaacattaacactagaccttaatcaaatcctcagt
attcacctcacactagcatcatctctctgtctgggatttggtttagctcatctaactggt
tttcttggtccaggaatgtggactagtgattccctcaatcttgtaggctctattagattc
gtaaaagcctccttcaatctccttgcttatgcacgattagcttatggtgtcatatcatca
catcacatcatctctggattgcttggcacttccataggattatggcatatcacattacgt
ccattagcatacctctacaatctattaagcatgggaaaggttgagtccattctttcaagt
agtattacagctgtattcttcactgcattccttatttcagcccttatgtggtatggctct
gcacacaccacacaagaactcttcggtcctactagatactcatgggacaatgcttattac
tctcttgatatcagaacctcttgctcaaatgcctggaacactcttccagacaagttagtc
ctttatgattatattggatctaaccctgctaaaggtggcttatttcgttcaggaccaatg
cttaaagctgatggtcttgttcaaaactggttgggccatgcatgcttctccatgggtaca
ttatccttaagcatcagaaggatgcctgctttctttgaaaccttcccagtaatcctcatt
gaccaaacaagtacagtgagagcagacattgcttttagacggtccacatctacttatagc
atggaagaatcccaaatacaagtatacttctctggtggttgtttaaatggtacagaatat
tctacaccttcccttgtaaaagcttatgcaagaaaagctcagtttggtcagatatttaca
tttgataaaaagacatccagattgactgatggtgtatttagaacatctgctcgaggttgg
tattcattctcacacatagctttagctttctcctttttctttggccatttgtggcatgct
agtcgtgctatcttccaagacatttggacaggtgtaacatttgaatcacaagcaaaacaa
gaatatggtagaaatgaaaagctaggagataagacatcttcaactaaatccattgtttaa
>KrE11_psbB
atgcaaataactgatgctttaccttggtttagggtacatatagttattcttaatgatcca
ggacgtctcattagctctcatattatgcacacagctttagtagcaggttggtcagctctc
atgcttttatatgagcttatcaccatagatcctacagatcctgtgtataatccaatttgg
agacaagcagcttacacactcccatttatctcacgtattggtgttattcgctctcttttt
agttggtcacttggcatagatcctacatccaatctaatctggacttatgaaacaatgaac
atagcccatattctattatctggtttattaattcttgcatcattttggcattgggcatat
tgggacttagatctattcttcacctcaacattaacactagaccttaatcaaatcctcagt
attcacctcacactagcatcatctctctgtctgggatttggtttagctcatctaactggt
tttcttggtccaggaatgtggactagtgattccctcaatcttgtaggctctattagattc
gtaaaagcctccttcaatctccttgcttatgcacgattagcttatggtgtcatatcatca
catcacatcatctctggattgcttggcacttccataggattatggcatatcacattacgt
ccattagcatacctctacaatctattaagcatgggaaaggttgagtccattctttcaagt
agtattacagctgtattcttcactgcattccttatttcagccctgatgtggtatggttct
gcacataccacacaagaactattcggtcctactagatactcatgggacaatgcttattac
tctcttgatatcagaacctcttgctcaaatgcctggaacactcttccagacaagttagta
ctttatgattatattggctctaaccctgctaaaggtggcttatttcgttcaggaccaatg
cttaaagctgatggtcttgttcaaaactggttgggccatgcatgcttctccatgggtaca
ttatccttaagcatcagaaggatgcctgctttctttgaaaccttcccagtaatcctcatt
gaccaaacaagtacagtgagagcagacattgctttcagacggtccacatctacttatagc
atggaagaatcccaaatacaagtatacttctctggtggttgtttaaatggtacagaatat
tctacaccttcccttgtaaaagcttatgcaagaaaagctcagtttggtcagatatttaca
tttgataaaaagacatccagattgactgatggtgtatttagaacatctgctcgaggttgg
tattcattctcacacatagctttagctttctcctttttctttggccatttgtggcatgct
agtcgtgctatcttccaagacatttggacaggtgtaacatttgaatcacaagcaaaacaa
gaatatggtagaaatgaaaagctaggagataagacatcttcaactaaatccattgtttaa
>KrE12_psbB
atgcaaataactgctgttttaccttggtttagggtacatatagttattcttaatgatcca
ggacgtctcattagctcacatattatgcacacagctttagtagcagggtggtcagctctc
atgcttttatatgagcttatcaccatagatcctacagatcctgtgtataatccaatttgg
agacaagcagcttacacactcccatttatctcacgtattggtgttattcgctctcttttt
agttggtcacttggcatagatcctacatccaatctaatctggacttatgaaacaatgaac
atagcccatattctattatctggtttattaattcttgcatcattttggcattgggcatat
tgggacttagatctattcttcacctcaacattaacactagaccttaatcaaatcctcagt
attcacctcacactagcatcatctctctgtctgggatttggtttagctcatctaactggt
tttcttggtccaggaatgtggactagtgattccctcaatcttgtaggctctattagattc
gtaaaagcctccttcaatctccttgcttatgcacgattagcttatggtgtcatatcatca
catcacatcatctctggattgcttggcacttccataggattatggcatatcacattacgt

ccattagcatacctctacaatctattaagcatgggaaaggttgagtccattctttcaagt
agtattacagctgtattcttcactgcattccttatttcagcccttatgtggtatggctct
gcacacaccacacaagaactcttcggtcctactagatactcatgggacaatgcttattac
tctcttgatatcagaacctcttgctcaaatgcctggaacactcttccagacaagttagtc
ctttatgattatattggatctaaccctgctaaaggtggcttatttcgttcaggaccaatg
cttaaagctgatggtcttgttcaaaactggttgggccatgcatgcttctccatgggtaca
ttatccttaagcatcagaaggatgcctgctttctttgaaaccttcccagtaatcctcatt
gaccaaacaagtacagtgagagcagacattgctttcagacggtccacatctacttatagc
atggaagaatcccaaatacaagtatacttctctggtggttgtttaaatggtacagaatat
tctacaccttcccttgtaaaagcttatgcaagaaaagctcagtttggtcagatatttaca
tttgataaaaagacatccagattgactgatggtgtatttagaacatctgctcgaggttgg
tattcattctcacacatagctttagctttctcctttttctttggccatttgtggcatgct
agtcgtgctatcttccaagacatttggacaggtgtaacatttgaatcacaagcaaaacaa
gaatatggtagaaatgaaaagctaggagataagacatcttcaactaaatccattgtttaa
>KrE13_psbB
atgcaaataactgatgttttaccttggtttagggtacatatagttattcttaatgatcca
ggacgtctcattagctcacatattatgcacacagctttagtagcaggttggtcagctctc
atgcttttatatgagcttatcaccatagatcctacagatcctgtgtataatccaatttgg
agacaagcagcttacacactcccatttatctcacgtattggtgttattcgctctcttttt
agttggtcacttggcatagatcctacatccaatctaatctggacttatgaaacaatgaac
atagcccatattctattatctggtttattaattcttgcatcattttggcattgggcatat
tgggacttagatctattcttcacctcaacattaacactagaccttaatcaaatcctcagt
attcacctcacactagcatcatctctctgtctgggatttggtttagctcatctaactggt
tttcttggtccaggaatgtggactagtgattccctcaatcttgtaggctctattagattc
gtaaaagcctccttcaatctccttgcttatgcacgattagcttatggtgtcatatcatca
catcacatcatctctggattgcttggcacttccataggattatggcatatcacattacgt
ccattagcatacctctacaatctattaagcatgggaaaggttgagtccattctttcaagt
agtattacagctgtattcttcactgcattccttatttcagcccttatgtggtatggctct
gcacacaccacacaagaactcttcggtcctactagatactcatgggacaatgcttattac
tctcttgatatcagaacctcttgctcaaatgcctggaacactcttccagacaagttagtc
ctttatgattatattggatctaaccctgctaaaggtggcttatttcgttcaggaccaatg
cttaaagctgatggtcttgttcaaaactggttgggccatgcatgcttctccatgggtaca
ttatccttaagcatcagaaggatgcctgctttctttgaaaccttcccagtaatcctcatt
gaccaaacaagtacagtgagagcagacattgctttcagacggtccacatctacttatagc
atggaagaatcccaaatacaagtatacttctctggtggttgtttaaatggtacagaatat
tctacaccttcccttgtaaaagcttatgcaagaaaagctcagtttggtcagatatttaca
tttgataaaaagacatccagattgactgatggtgtatttagaacatctgctcgaggttgg
tattcattctcacacatagctttagctttctcctttttctttggccatttgtggcatgct
agtcgtgctatcttcaaagacatttggacaggtgtaacatttgaatcacaagcaaaacaa
gaatatggtagaaatgaaaagctaggagataagacatcttcaactaaatccattgtttaa
>KrE14_psbB
atgcaaataactgatgctttaccttggtttagggtacatatagttattcttaatgatcca
ggacgtctcattagctcacatattatgcacacagctttagtagcaggttggtcagctctc
atgcttttatatgagcttatcaccatagatcctacagatcctgtgtataatccaatttgg
agacaagcagcttacacactcccatttatctcacgtattggtgttattcgctctcttttt
agttggtcacttggcatagatcctacatccaatctaatctggacttatgaaacaatgaac
atagcccatattctattatctggtttattaattcttgcatcattttggcattgggcatat
tgggacttagatctattcttcacctcaacattaacactagaccttaatcaaatcctcagt
attcacctcacactagcatcatctctctgtctgggatttggtttagctcatctaactggt
tttcttggtccaggaatgtggactagtgattccctcaatcttgtaggctctattagattc
gtaaaagcctccttcaatctccttgcttatgcacgattagcttatggtgtcatatcatca
catcacatcatctctggattgcttggcacttccataggattatggcatatcacattacgt
ccattagcatacctctacaatctattaagcatgggaaaggttgagtccattctttcaagt
agtattacagctgtattcttcactgcattccttatttcagcccttatgtggtatggctct
gcacacaccacacaagaactcttcggtcctactagatactcatgggacaatgcttattac
tctcttgatatcagaacctcttgctcaaatgcctggaacactcttccagacaagttagta
ctttatgattatattggctctaaccctgctaaaggtggcttatttcgttcaggaccaatg

cttaaagctgatggtcttgttcaaaactggttgggccatgcatgcttctccatgggtaca
ttatccttaagcatcagaaggatgcctgctttctttgaaaccttcccagtaatcctcatt
gaccaaacaagtacagtgagagcagacattgctttcagacggtccacatctacttatagc
atggaagaatcccaaatacaagtatacttctctggtggttgtttaaatggtacagaatat
tctacaccttcccttgtaaaagcttatgcaagaaaagctcagtttggtcagatatttaca
tttgataaaaagacatccagattgactgatggtgtatttagaacatctgctcgaggttgg
tattcattctcacacatagctttagctttctcctttttctttggccatttgtggcatgct
agtcgtgctatcttcaaagacatttggacaggtgtaacatttgaatcacaagcaaaacaa
gaatatggtagaaatgaaaagctaggagataagacatcttcaactaaatccattgtttaa
>KrE15_psbB
atgcaaataactgatgctttaccttggtttagggtacatatagttattcttaatgatcca
ggacgtctcattagctcacatattatgcacacagctttagtagcaggttggtcagctctc
atgcttttatatgagcttatcaccatagatcctacagatcctgtgtataatccaatttgg
agacaagcagcttacacactcccatttatctcacgtattggtgttattcgctctcttttt
agttggtcacttggcatagatcctacatccaatctaatctggacttatgaaacaatgaac
atagcccatattctattatctggtttattaattcttgcatcattttggcattgggcatat
tgggacttagatctattcttcacctcaacattaacactagaccttaatcaaatcctcagt
attcacctcacactagcatcatctctctgtctgggatttggtttagctcatctaactggt
tttcttggtccaggaatgtggactagtgattccctcaatcttgtaggctctattagattc
gtaaaagcctccttcaatctccttgcttatgcacgattagcttatggtgtcatatcatca
catcacatcatctctggattgcttggcacttccataggattatggcatatcacattacgt
ccattagcatacctctacaatctattaagcatgggaaaggttgagtccattctttcaagt
agtattacagctgtattcttcactgcattccttatttcagcccttatgtggtatggctct
gcacacaccacacaagaactcttcggtcctactagatactcatgggacaatgcttattac
tctcttgatatcagaacctcttgctcaaatgcctggaacactcttccagacaagttagtc
ctttatgattatattggatctaaccctgctaaaggtggcttatttcgttcaggaccaatg
cttaaagctgatggtcttgttcaaaactggttgggccatgcatgcttctccatgggtaca
ttatccttaagcatcagaaggatgcctgctttctttgaaaccttcccagtaatcctcatt
gaccaaacaagtacagtgagagcagacattgctttcagacggtccacatctacttatagc
atggaagaatcccaaatacaagtatacttctctggtggttgtttaaatggtacagaatat
tctacaccttcccttgtaaaagcttatgcaagaaaagctcagtttggtcagatatttaca
tttgataaaaagacatccagattgactgatggtgtatttagaacatctgctcgaggttgg
tattcattctcacacatagctttagctttctcctttttctttggccatttgtggcatgct
agtcgtgctatcttccaagacatttggacaggtgtaacatttgaatcacaagcaaaacaa
gaatatggtagaaatgaaaagctaggagataagacatcttcaactaaatccattgtttaa
>KrE2_psbB
atgcaaataactgatgctttaccttggtttagggtacatatagttattcttaatgatcca
ggacgtctcattagctcacatattatgcacacagctttagtagcaggttggtcagctctc
atgcttttatatgagcttatcaccatagatcctacagatcctgtgtataatccaatttgg
agacaagcagcttacacactcccatttatctcacgtattggtgttattcgctctcttttt
agttggtcacttggcatagatcctacatccaatctaatctggacttatgaaacaatgaac
atagcccatattctattatctggtttattaattcttgcatcattttggcattgggcatat
tgggacttagatctattcttcacctcaacattaacactagaccttaatcaaatcctcagt
attcacctcacactagcatcatctctctgtctgggatttggtttagctcatctaactggt
tttcttggtccaggaatgtggactagtgattccctcaatcttgtaggctctattagattc
gtaaaagcctccttcaatctccttgcttatgcacgattagcttatggtgtcatatcatca
catcacatcatctctggattgcttggcacttccataggattatggcatatcacattacgt
ccattagcatacctctacaatctattaagcatgggaaaggttgagtccattctttcaagt
agtattacagctgtattcttcactgcattccttatttcagcccttatgtggtatggctct
gcacacaccacacaagaactcttcggtcctactagatactcatgggacaatgcttattac
tctcttgatatcagaacctcttgctcaaatgcctggaacactcttccagacaagttagtc
ctttatgattatattggatctaaccctgctaaaggtggcttatttcgttcaggaccaatg
cttaaagctgatggtcttgttcaaaactggttgggccatgcatgcttctccatgggtaca
ttatccttaagcatcagaaggatgcctgctttctttgaaaccttcccagtaatcctcatt
gaccaaacaagtacagtgagagcagacattgctttcagacggtccacatctacttatagc
atggaagaatcccaaatacaagtatacttctctggtggttgtttaaatggtacagaatat
tctacaccttcccttgtaaaagcttatgcaagaaaagctcagtttggtcagatatttaca

tttgataaaaagacatccagattgactgatggtgtatttagaacatctgctcgaggttgg
tattcattctcacacatagctttagctttctcctttttctttggccatttgtggcatgct
agtcgtgctatcttccaagacatttggacaggtgtaacatttgaatcacaagcaaaacaa
gaatatggtagaaatgaaaagctaggagataagacatcttcaactaaatccattgtttaa
>KrE3_psbB
atgcaaataactgatgctttaccttggtttagggtacatatagttattcttaatgatcca
ggacgtctcattagctcacatattatgcacacagctttagtagcaggttggtcagctctc
atgcttttatatgagcttatcaccatagatcctacagatcctgtgtataatccaatttgg
agacaagcagcttacacactcccatttatctcacgtattggtgttattcgctctctttt
agttggtcacttggcatagatcctacatccaatctaatctggacttatgaaacaatgaac
atagcccatattctattatctggtttattaattcttgcatcattttggcattgggcatat
tgggacttagatctattcttcacctcaacattaacactagaccttaatcaaatcctcagt
attcacctcacactagcatcatctctctgtctgggatttggtttagctcatctaacaggt
tttcttggtccaggaatgtggactagtgattccctcaatcttgtaggctctattagattc
gtaaaagcctccttcaatctccttgcttatgcacgattagcttatggtgtcatatcatca
catcacatcatctctggattgcttggcacttccataggattatggcatatcacattacgt
ccattagcatacctctacaatctattaagcatgggaaaggttgagtccattctttcaagt
agtattacagctgtattcttcactgcattccttatttcagcccttatgtggtatggctct
gcacacaccacacaagaactcttcggtcctactagatactcatgggacaatgcttattac
tctcttgatatcagaacctcttgctcaaatgcctggaacactcttccagacaagttactc
ctttatgattatattggatctaaccctgctaaaggtggcttatttcgttcaggaccaatg
cttaaagctgatggtcttgttcaaaactggttgggccatgcatgcttctccatgggtaca
ttatccttaagcatcagaaggatgcctgctttctttgaaaccttcccagtaatcctcatt
gaccaaacaagtacagtgagagcagacattgctttcagacggtccacatctacttatagc
atggaagaatcccaaatacaagtatacttctctggtggttgtttaaatggtacagaatat
tctacaccttcccttgtaaaagcttatgcaagaaaagctcagtttggtcagatatttaca
tttgataaaaagacatccagattgactgatggtgtatttagaacatctgctcgaggttgg
tattcattctcacacatagctttagctttctcctttttctttggccatttgtggcatgct
agtcgtgctatcttccaagacatttggacaggtgtaacatttgaatcacaagcaaaacaa
gaatatggtagaaatgaaaagctaggagataagacatcttcaactaaatccattgtttaa
>KrE4_psbB
atgcaaataactgatgctttaccttggtttagggtacatatagttattcttaatgatcca
ggacgtctcattagctcacatattatgcacacagctttagtagcaggttggtcagctctc
atgcttttatatgagcttatcaccatagatcctacagatcctgtgtataatccaatttgg
agacaagcagcttacacactcccatttatctcacgtattggtgttattcgctctctttt
agttggtcacttggcatagatcctacatccaatctaatctggacttatgaaacaatgaac
atagcccatattctattatctggtttattaattcttgcatcattttggcattgggcatat
tgggacttagatctattcttcacctcaacattaacactagaccttaatcaaatcctcagt
attcacctcacactagcatcatctctctgtctgggatttggtttagctcatctaactggt
tttcttggtccaggaatgtggactagtgattccctcaatcttgtaggctctattagattc
gtaaaagcctccttcaatctccttgcttatgcacgattagcttatggtgtcatatcatca
catcacatcatctctggattgcttggcacttccataggattatggcatatcacattacgt
ccattagcatacctctacaatctattaagcatgggaaaggttgagtccattctttcaagt
agtattacagctgtattcttcactgcattccttatttcagcccttatgtggtatggctct
gcacacaccacacaagaactcttcggtcctactagatactcatgggacaatgcttattac
tctcttgatatcagaacctcttgctcaaatgcctggaacactcttccagacaagttagtc
ctttatgattatattggatctaaccctgctaaaggtggcttatttcgttcaggaccaatg
cttaaagctgatggtcttgttcaaaactggttgggccatgcatgcttctccatgggtaca
ttatccttaagcatcagaaggatgcctgctttctttgaaaccttcccagtaatcctcatt
gaccaaacaagtacagtgagagcagacattgctttcagacggtccacatctacttatagc
atggaagaatcccaaatacaagtatacttctctggtggttgtttaaatggtacagaatat
tctacaccttcccttgtaaaagcttatgcaagaaaagctcagtttggtcagatatttaca
tttgataaaaagacatccagattgactgatggtgtatttagaacatctgctcgaggttgg
tattcattctcacacatagctttagctttctcctttttctttggccatttgtggcatgct
agtcgtgctatcttccaagacatttggacaggtgtaacatttgaatcacaagcaaaacaa
gaatatggtagaaatgaaaagctaggagataagacatcttcaactaaatccattgtttaa
>KrE5_psbB

atgcaaataactgatgttttaccttggtttagggtacatatagttattcttaatgatcca
ggacgtctcattagctcacatattatgcacacagctttagtagcaggttggtcagctctc
atgcttttatatgagcttatcaccatagatcctacagatcctgtgtataatccaatttgg
agacaagcagcttacacactcccatttatctcacgtattggtgttattcgctctcttttt
agttggtcacttggcatagatcctacatccaatctaatctggacttatgaaacaatgaac
atagcccatattctattatctggtttattaattcttgcatcattttggcattgggcatat
tgggacttagatctattcttcacctcaacattaacactagaccttaatcaaatcctcagt
attcacctcacactagcatcatctctctgtctgggatttggtttagctcatctaactggt
tttcttggtccaggaatgtggactagtgattccctcaatcttgtaggctctattagattc
gtaaaagcctccttcaatctccttgcttatgcacgattagcttatggtgtcatatcatca
catcacatcatctctggattgcttggcacttccataggattatggcatatcacattacgt
ccattagcatacctctacaatctattaagcatgggaaaggttgagtccattctttcaagt
agtattacagctgtattcttcactgcattccttatttcagcccttatgtggtatggctct
gcacacaccacacaagaactcttcggtcctactagatactcatgggacaatgcttattac
tctcttgatatcagaacctcttgctcaaatgcctggaacactcttccagacaagttagtc
ctttatgattatattggatctaaccctgctaaaggtggcttatttcgttcaggaccaatg
cttaaagctgatggtcttgttcaaaactggttgggccatgcatgcttctccatgggtaca
ttatccttaagcatcagaaggatgcctgctttctttgaaaccttcccagtaatcctcatt
gaccaaacaagtacagtgagagcagacattgctttcagacggtccacatctacttatagc
atggaagaatcccaaatacaagtatacttctctggtggttgtttaaatggtacagaatat
tctacaccttcccttgtaaaagcttatgcaagaaaagctcagtttggtcagatatttaca
tttgataaaaagacatccagattgactgatggtgtatttagaacatctgctcgaggttgg
tattcattctcacacatagctttagctttctcctttttctttggccatttgtggcatgct
agtcgtgctatcttccaagacatttggacaggtgtaacatttgaatcacaagcaaaacaa
gaatatggtagaaatgaaaagctaggagataagacatcttcaactaaatccattgtttaa
>KrE6_psbB
atgcaaataactgatgctttaccttggtttagggtacatatagttattcttaatgatcca
ggacgtctcattagctcacatattatgcacacagctttagtagcaggttggtcagctctc
atgcttttatatgagcttatcaccatagatcctacagatcctgtgtataatccaatttgg
agacaagcagcttacacactcccatttatctcacgtatcggtgttattcgctctcttttt
agttggtcacttggcatagatcctacatccaatctaatctggacttatgaaacaatgaac
atagcccatattctattatctggtttattaattcttgcatcattttggcattgggcatat
tgggacttagatctattcttcacctcaacattaacactagaccttaatcaaatcctcagt
attcacctcacactagcatcatctctctgtctgggatttggtttagctcatctaactggt
tttcttggtccaggaatgtggactagtgattccctcaatcttgtaggctctattagattc
gtaaaagcctccttcaatctccttgcttatgcacgattagcttatggtgtcatatcatca
catcacatcatctctggattgcttggcacttccataggattatggcatatcacattacgt
ccattagcatacctctacaatctattaagcatgggaaaggttgagtccattctttcaagt
agtattacagctgtattcttcactgcattccttatttcagcccttatgtggtatggctct
gcacacaccacacaagaactcttcggtcctactagatactcatgggacaatgcttattac
tatcttgatatcagaacctcttgctcaaatgcctggaacactcttccagacaagttagtc
ctttatgattatattggatctaaccctgctaaaggtggcttatttcgttcaggaccaatg
cttaaagctgatggtcttgttcaaaactggttgggccatgcatgcttctccatgggtaca
ttatccttaagcatcagaaggatgcctgctttctttgaaaccttcccagtaatcctcatt
gaccaaacaagtacagtgagagcagacattgctttcagacggtccacatctacttatagc
atggaagaatcccaaatacaagtatacttctctggtggttgtttaaatggtacagaatat
tctacaccttcccttgtaaaagcttatgcaagaaaagctcagtttggtcagatatttaca
tttgataaaaagacatccagattgactgatggtgtatttagaacatctgctcgaggttgg
tattcattctcacacatagctttagctttctcctttttctttggccatttgtggcatgct
agtcgtgctatcttccaagacatttggacaggtgtaacatttgaatcacaagcaaaacaa
gaatatggtagaaatgaaaagctaggagataagacatcttcaactaaatccattgtttaa
>KrE7_psbB
atgcaaataactgatgctttaccttggtttagggtacatatagttattcttaatgatcca
ggacgtctcattagctcacatattatgcacacagctttagtagcaggttggtcagctctc
atgcttttatatgagcttatcaccatagatcctacagatcctgtgtataatccaatttgg
agacaagcagcttacacactcccatttatctcacgtattggtgttattcgctctcttttt
agttggtcacttggcatagatcctacatccaatctaatctggacttatgaaacaatgaac

atagcccatattctattatctggtttattaattcttgcatcattttggcattgggcatat
tgggacttagatctattcttcacctcaacattaacactagaccttaatcaaatcctcagt
attcacctcacactagcatcatctctctgtctgggatttggtttagctcatctaactggt
tttcttggtccaggaatgtggactagtgattccctcaatcttgtaggctctattagattc
gtaaaagcctccttcaatctccttgcttatgcacgattagcttatggtgtcatatcatca
catcacatcatctctggattgcttggcacttccataggattatggcatatcacattacgt
ccattagcatacctctacaatctattaagcatgggaaaggttgagtccattctttcaagt
agtattacagctgtattcttcactgcattccttatttcagcccttatgtggtatggctct
gcacacaccacacaagaactcttcggtcctactagatactcatgggacaatgcttattac
tctcttgatatcagaacctcttgctcaaatgcctggaacactcttccagacaagttagta
ctttatgattatattggctctaaccctgctaaaggtggcttatttcgttcaggaccaatg
cttaaagctgatggtcttgttcaaaactggttgggccatgcatgcttctccatgggtaca
ttatccttaagcatcagaaggatgcctgctttctttgaaaccttcccagtaatcctcatt
gaccaaacaagtacagtgagagcagacattgcttttagacggtccacatctacttatagc
atggaagaatcccaaatacaagtatacttctctggtggttgtttaaatggtacagaatat
tctacaccttcccttgtaaaagcttatgcaagaaaagctcagtttggtcagatatttaca
tttgataaaaagacatccagattgactgatggtgtatttagaacatctgctcgaggttgg
tattcattctcacacatagctttagctttctcctttttctttggccatttgtggcatgct
agtcgtgctatcttccaagacatttggacaggtgtaacatttgaatcacaagcaaaacaa
gaatatggtagaaatgaaaagctaggagataagacatcttcaactaaatccattgtttaa
>KrE8_psbB
atgcaaataactgatgctttaccttggtttagggtacatatagttattcttaatgatcca
ggacgtctcattagctcacatattatgcacacagctttagtagcaggttggtcagctctc
atgcttttatatgagcttatcaccatagatcctacagatcctgtgtataatccaatttgg
agacaagcagcttacacactcccatttatctcacgtattggtgttattcgctctcttttt
agttggtcacttggcatagatcctacatccaatctaatctggacttatgaaacaatgaac
atagcccatattctattatctggtttattaattcttgcatcattttggcattgggcatat
tgggacttagatctattcttcacctcaacattaacactagaccttaatcaaatcctcagt
attcacctcacactagcatcatctctctgtctgggatttggtttagctcatctaactggt
tttcttggtccaggaatgtggactagtgattccctcaatcttgtaggctctattagattc
gtaaaagcctccttcaatctccttgcttatgcacgattagcttatggtgtcatatcatca
catcacatcatctctggattgcttggcacttccataggattatggcatatcacattacgt
ccattagcatacctctacaatctattaagcatgggaaaggttgagtccattctttcaagt
agtattacagctgtattcttcactgcattccttatttcagcccttatgtggtatggctct
gcacacaccacacaagaactcttcggtcctactagatactcatgggacaatgcttattac
tctcttgatatcagaacctcttgctcaaatgcctggaacactcttccagacaagttagtc
ctttatgattatattggatctaaccctgctaaaggtggcttatttcgttcaggaccaatg
cttaaagctgatggtcttgttcaaaactggttgggccatgcatgcttctccatgggtaca
ttatccttaagcatcagaaggatgcctgctttctttgaaaccttcccagtaatcctcatt
gaccaaacaagtacagtgagagcagacattgctttcagacggtccacatctacttatagc
atggaagaatcccaaatacaagtatacttctctggtggttgtttaaatggtacagaatat
tctacaccttcccttgtaaaagcttatgcaagaaaagctcagtttggtcagatatttaca
tttgataaaaagacatccagattgactgatggtgtatttagaacatctgctcgaggttgg
tattcattctcacacatagctttagctttctcctttttctttggccatttgtggcatgct
agtcgtgctatcttccaagacatttggacaggtgtaacatttgaatcacaagcaaaacaa
gaatatggtagaaatgaaaagctaggagataagacatcttcaactaaatccattgtttaa
>KrE9_psbB
atgcaaataactgatgttttaccttggtttagggtacatatagttattcttaatgatcca
ggacgtctcattagctcacatattatgcacacagctttagtagcaggttggtcagctctc
atgcttttatatgagcttatcaccatagatcctacagatcctgtgtataatccaatttgg
agacaagcagcttacacactcccatttatctcacgtattggtgttattcgctctcttttt
agttggtcacttggcatagatcctacatccaatctaatctggacttatgaaacaatgaac
atagcccatattctattatctggtttattaattcttgcatcattttggcattgggcatat
tgggacttagatctattcttcacctcaacattaacactagaccttaatcaaatcctcagt
attcacctcacactagcatcatctctctgtctgggatttggtttagctcatctaactggt
tttcttggtccaggaatgtggactagtgattccctcaatcttgtaggctctattagattc
gtaaaagcctccttcaatctccttgcttatgcacgattagcttatggtgtcatatcatca

catcacatcatctctggattgcttggcacttccataggattatggcatatcacattacgt
ccattagcatacctctacaatctattaagcatgggaaaggttgagtccattctttcaagt
agtattacagctgtattcttcactgcattccttatttcagcccttatgtggtatggctct
gcacataccacacaagaactattcggtcctactagatactcatgggacaatgcttattac
tctcttgatatcagaacctcttgctcaaatgcctggaacactcttccagacaagttagtc
ctttatgattatattggatctaaccctgctaaaggtggcttatttcgttcaggaccaatg
cttaaagctgatggtcttgttcaaaactggttgggccatgcatgcttctccatgggtaca
ttatccttaagcatcagaaggatgcctgctttctttgaaaccttcccagtaatcctcatt
gaccaaacaagtacagtgagagcagacattgctttcagacggtccacatctacttatagc
atggaagaatcccaaatacaagtatacttctctggtggttgtttaaatggtacagaatat
tctacaccttcccttgtaaaagcttatgcaagaaaagctcagtttggtcagatatttaca
tttgataaaaagacatccagattgactgatggtgtatttagaacatctgctcgaggttgg
tattcattctcacacataggtttagctttcctattcttctttggccatttgtggcatgct
agtcgtgctatcttccaagacatttggacaggtgtaacatttgaatcacaagcaaaacaa
gaatatggtagaaatgaaaagctaggagataagacatcttcaactaaatccattgtttaa
>Mz10_psbB
atgcaaataactgatgctttaccttggtttagggtacatatagttattcttaatgatcca
ggacgtctcattagctctcatattatgcacacagctttagtagcaggttggtcagctctc
atgcttttatatgagcttatcaccatagatcctacagatcctgtgtataatccaatttgg
agacaagcagcttacacactcccatttatctcacgtatcggtgttattcgctctcttttt
agttggtcacttggcatagatcctacatccaatctaatctggacttatgaaacaatgaac
atagcccatattctattatctggtttattaattcttgcatcattttggcattgggcatat
tgggacttagatctattcttcacctcaacattaacactagaccttaatcaaatcctcagt
attcacctcacactagcatcatctctctgtctgggatttggtttagctcatctaactggt
tttcttggtccaggaatgtggactagtgattccctcaatcttgtaggctctattagattc
gtaaaagcctccttcaatctccttgcttatgcacgattagcttatggtgtcatatcatca
catcacatcatctctggattgcttggcacttccataggattatggcatatcacattacgt
ccattagcatacctctacaatctattaagcatgggaaaggttgagtccattctttcaagt
agtattacagctgtattcttcactgcattccttatttcagcccttatgtggtatggctct
gcacacaccacacaagaactcttcggtcctactagatactcatgggacaatgcttattac
tctcttgatatcagaacctcttgctcaaatgcctggaacactcttccagacaagttagtc
ctttatgattatattggatctaaccctgctaaaggtggcttatttcgttcaggaccaatg
cttaaagctgatggtcttgttcaaaactggttgggccatgcatgcttctccatgggtaca
ttatccttaagcatcagaaggatgcctgctttctttgaaaccttcccagtaatcctcatt
gaccaaacaagtacagtgagagcagacattgctttcagacggtccacatctacttatagc
atggaagaatcccaaatacaagtatacttctctggtggttgtttaaatggtacagaatat
tctacaccttcccttgtaaaagcttatgcaagaaaagctcagtttggtcagatatttaca
tttgataaaaagacatccagattgactgatggtgtatttagaacatctgctcgaggttgg
tattcattctcacacatagctttagctttctcctttttctttggccatttgtggcatgct
agtcgtgctatcttccaagacatttggacaggtgtaacatttgaatctcaagcaaaacaa
gaatatggtagaaatgaaaagctaggagataagacatcttcaactaaatccattgtttaa
>Mz5_psbB
atgcaaataactgatgctttaccttggtttagggtacatatagttattcttaatgatcca
ggacgtctcattagctcacatattatgcacacagctttagtagcaggttggtcagctctc
atgcttttatatgagcttatcaccatagatcctacagatcctgtgtataatccaatttgg
agacaagcagcttacacactcccatttatctcacgtattggtgttattcgctctcttttt
agttggtcacttggcatagatcctacatccaatctaatctggacttatgaaacaatgaac
atagcccatattctattatctggtttattaattcttgcatcattttggcattgggcatat
tgggacttagatctattcttcacctcaacattaacactagaccttaatcaaatcctcagt
attcacctcacactagcatcatctctctgtctgggatttggtttagctcatctaactggt
tttcttggtccaggaatgtggactagtgattccctcaatcttgtaggctctattagattc
gtaaaagcctccttcaatctccttgcttatgcacgattagcttatggtgtcatatcatca
catcacatcatctctggattgcttggcacttccataggattatggcatatcacattacgt
ccattagcatacctctacaatctattaagcatgggaaaggttgagtccattctttcaagt
agtattacagctgtattcttcactgcattccttatttcagcccttatgtggtatggctct
gcacacaccacacaagaactcttcggtcctactagatactcatgggacaatgcttattac
tctcttgatatcagaacctcttgctcaaatgcctggaacactcttccagacaagttagtc

ctttatgattatattggatctaaccctgctaaaggtggcttatttcgttcaggaccaatg
cttaaagctgatggtcttgttcaaaactggttgggccatgcatgcttctccatgggtaca
ttatccttaagcatcagaaggatgcctgctttctttgaaaccttcccagtaatcctcatt
gaccaaacaagtacagtgagagcagacattgctttcagacggtccacatctacttatagc
atggaagaatcccaaatacaagtatacttctctggtggttgtttaaatggtacagaatat
tctacaccttcccttgtaaaagcttatgcaagaaaagctcagtttggtcagatatttaca
tttgataaaaagacatccagattgactgatggtgtatttagaacatctgctcgaggttgg
tattcattctcacacatagctttagctttctcctttttctttggccatttgtggcatgct
agtcgtgctatcttccaagacatttggacaggtgtaacatttgaatcacaagcaaaacaa
gaatatggtagaaatgaaaagctaggagataagacatcttcaactaaatccattgtttaa
>Mz6_psbB
atgcaaataactgatgctttaccttggtttagggtacatatagttattcttaatgatcca
ggacgtctcattagctcacatattatgcacacagctttagtagcaggttggtcagctctc
atgcttttatatgagcttatcaccatagatcctacagatcctgtgtataatccaatttgg
agacaagcagcttacacactcccatttatctcacgtattggtgttattcgctctcttttt
agttggtcacttggcatagatcctacatccaatctaatctggacttatgaaacaatgaac
atagcccatattctattatctggtttattaattcttgcatcattttggcattgggcatat
tgggacttagatctattcttcacctcaacattaacactagaccttaatcaaatcctcagt
attcacctcacactagcatcatctctctgtctgggatttggtttagctcatctaactggt
tttcttggtccaggaatgtggactagtgattccctcaatcttgtaggctctattagattc
gtaaaagcctccttcaatctccttgcttatgcacgattagcttatggtgtcatatcatca
catcacatcatctctggattgcttggcacttccataggattatggcatatcacattacgt
ccattagcatacctctacaatctattaagcatgggaaaggttgagtccattctttcaagt
agtattacagctgtattcttcactgcattccttatttcagcccttatgtggtatggctct
gcacacaccacacaagaactcttcggtcctactagatactcatgggacaatgcttattac
tctcttgatatcagaacctcttgctcaaatgcctggaacactcttccagacaagttagtc
ctttatgattatattggatctaaccctgctaaaggtggcttatttcgttcaggaccaatg
cttaaagctgatggtcttgttcaaaactggttgggccatgcatgcttctccatgggtaca
ttatccttaagcatcagaaggatgcctgctttctttgaaaccttcccagtaatcctcatt
gaccaaacaagtacagtgagagcagacattgctttcagacggtccacatctacttatagc
atggaagaatcccaaatacaagtatacttctctggtggttgtttaaatggtacagaatat
tctacaccttcccttgtaaaagcttatgcaagaaaagctcagtttggtcagatatttaca
tttgataaaaagacatccagattgactgatggtgtatttagaacatctgctcgaggttgg
tattcattctcacacatagctttagctttcctattcttctttggccatttgtggcatgct
agtcgtgctatcttccaagacatttggacaggtgtaacatttgaatcacaagcaaaacaa
gaatatggtagaaatgaaaagctaggagataagacatcttcaactaaatccattgtttaa
>Mz7_psbB
atgcaaataactgatgctttaccttggtttagggtacatatagttattcttaatgatcca
ggacgtctcattagctcacatattatgcacacagctttagtagctgggtggtcagctctc
atgcttttatatgagcttatcaccatagatcctacagatcctgtgtataatccaatttgg
agacaagcagcttacacactcccatttatctcacgtattggtgttattcgctctcttttt
agttggtcacttggcatagatcctacatccaatctaatctggacttatgaaacaatgaac
atagcccatattctattatctggtttattaattcttgcatcattttggcattgggcatat
tgggacttagatctattcttcacctcaacattaacactagaccttaatcaaatcctcagt
attcacctcacactagcatcatctctctgtctgggatttggtttagctcatctaactggt
tttcttggtccaggaatgtggactagtgattccctcaatcttgtaggctctattagattc
gtaaaagcctccttcaatctccttgcttatgcacgattagcttatggtgtcatatcatca
catcacatcatctctggattgcttggcacttccataggattatggcatatcacattacgt
ccattagcatacctctacaatctattaagcatgggaaaggttgagtccattctttcaagt
agtattacagctgtattcttcactgcattccttatttcagcccttatgtggtatggctct
gcacacaccacacaagaactcttcggtcctactagatactcatgggacaatgcttattac
tctcttgatatcagaacctcttgctcaaatgcctggaacactcttccagacaagttagta
ctttatgattatattggatctaaccctgctaaaggtggcttatttcgttcaggaccaatg
cttaaagctgatggtcttgttcaaaactggttgggccatgcatgcttctccatgggtaca
ttatccttaagcatcagaaggatgcctgctttctttgaaaccttcccagtaatcctcatt
gaccaaacaagtacagtgagagcagacattgctttcagacggtccacatctacttatagc
atggaagaatcccaaatacaagtatacttctatggtggttgtttaaatggtacagaatat

tctacaccttcccttgtaaaagcttatgcaagaaaagctcagtttggtcagatatttaca
tttgataaaaagacatccagattgactgatggtgtatttagaacatctgctcgaggttgg
tattcattctcacacatagctttagctttctcctttttctttggccatttgtggcatgct
agtcgtgctatcttcaaagacatttggacaggtgtaacatttgaatcacaagcaaaacaa
gaatatggtagaaatgaaaagctaggagataagacatcttcaactaaatccattgtttaa
>Mz8_psbB
atgcaaataactgatgctttaccttggtttagggtacatatagttattcttaatgatcca
ggacgtctcattagctcacatattatgcacacagctttagtagcaggttggtcagctctc
atgcttttatatgagcttatcaccatagatcctacagatcctgtgtataatccaatttgg
agacaagcagcttacacactcccatttatctcacgtattggtgttattcgctctcttttt
agttggtcacttggcatagatcctacatccaatctaatctggacttatgaaacaatgaac
atagcccatattctattatctggtttattaattcttgcatcattttggcattgggcatat
tgggacttagatctattcttcacctcaacattaacactagaccttaatcaaatcctcagt
attcacctcacactagcatcatctctctgtctgggatttggtttagctcatctaacaggt
tttcttggtccaggaatgtggactagtgattccctcaatcttgtaggctctattagattc
gtaaaagcctccttcaatctccttgcttatgcacgattagcttatggtgtcatatcatca
catcacatcatctctggattgcttggcacttccataggattatggcatatcacattacgt
ccattagcatacctctacaatctattaagcatgggaaaggttgagtccattctttcaagt
agtattacagctgtattcttcactgcattccttatttcagcccttatgtggtatggctct
gcacacaccacacaagaactattcggtcctactagatactcatgggacaatgcttattac
tctcttgatatcagaacctcttgctcaaatgcctggaacactcttccagacaagttagtc
ctttatgattatattggatctaaccctgctaaaggtggcttatttcgttcaggaccaatg
cttaaagctgatggtcttgttcaaaactggttaggtcatgcatgcttctccatgggtaca
ttatccttaagcatcagaaggatgcctgctttctttgaaaccttcccagtaatcctcatt
gaccaaacaagtacagtgagagcagacattgctttcagacggtccacatctacttatagc
atggaagaatcccaaatacaagtatacttctctggtggttgtttaaatggtacagaatat
tctacaccttcccttgtaaaagcttatgcaagaaaagctcagtttggtcagatatttaca
tttgataaaaagacatccagattgactgatggtgtatttagaacatctgctcgaggttgg
tattcattctcacacatagctttagctttctcctttttctttggccatttgtggcatgct
agtcgtgctatcttcaaagacatttggacaggtgtaacatttgaatctcaagcaaaacaa
gaatatggtagaaatgaaaagctaggagataagacatcttcaactaaatccattgtttaa
>Mz9_psbB
atgcaaataactgatgctttaccttggtttagggtacatatagttattcttaatgatcca
ggacgtctcattagctcacatattatgcacacagctttagtagcaggttggtcagctctc
atgcttttatatgagcttatcaccatagatcctacagatcctgtgtataatccaatttgg
agacaagcagcttacacactcccatttatctctcgtatcggtgttattcgctctcttttc
agttggtcacttggcatagatcctacatccaatctaatctggacttatgaaacaatgaac
atagcccatattctattatctggtttattaattcttgcatcattttggcattgggcatat
tgggacttagatctattcttcacctcaacattaacactagaccttaatcaaatcctcagt
attcacctcacactagcatcatctctctgtctgggatttggtttagctcatctaacaggt
tttcttggtccaggaatgtggactagtgattccctcaatcttgtaggctctattagattc
gtaaaagcctccttcaatatcctttcttatgcacgattagcttatggtgtcatatcatca
catcacatcatctctggattgcttggcacttccataggattatggcatatcacattacgt
ccattagcatacctctacaatctattaagcatgggaaaggttgagtccattctttcaagt
agtattacagctgtattcttcactgcattccttatttcagcccttatgtggtatggctct
gcacacaccacacaagaactattcggtcctactagatactcatgggacaatgcttattac
tctcttgatatcagaacctcttgctcaaatgcctggaacactcttccagacaagttagtc
ctttatgattatattggatctaaccctgctaaaggtggcttatttcgttcaggaccaatg
cttaaagctgatggtcttgttcaaaactggttaggtcatgcatgtttctccatgggtaca
ttatccttaagcatcagaaggatgcctgctttctttgaaaccttcccagtaatcctcatt
gaccaaacaagtacagtgagagcagacattgctttcagacggtccacatctacttatagc
atggaagaatcccaaatacaagtatacttctctggtggttgtttaaatggtacagaatat
tctacaccttcccttgtaaaagcttatgcaagaaaagctcagtttggtcagatatttaca
tttgataaaaagacatccagattgactgatggtgtatttagaacatctgctcgaggttgg
tattcattctcacacatagctttagctttctcctttttctttggccatttgtggcatgct
agtcgtgctatcttcaaagacatttggacaggtgtaacatttgaatcacaagcaaaacaa
gaatatggtagaaatgaaaagctaggagataagacatcttcaactaaatccattgtttaa

>MzC1_psbB
atgcaaataactgatgctttaccttggtttagggtacatatagttattcttaatgatcca
ggacgtctcattagctcacatattatgcacacagctttagtagcaggttggtcagctctc
atgcttttatatgagcttatcaccatagatcctacagatcctgtgtataatccaatttgg
agacaagcagcttacacactcccatttatctcacgtattggtgttattcgctctcttttt
agttggtcacttggcatagatcctacatccaatctaatctggacttatgaaacaatgaac
atagcccatattctattatctggtttattaattcttgcatcattttggcattgggcatat
tgggacttagatctattcttcacctcaacattaacactagaccttaatcaaatcctcagt
attcacctcacactagcatcatctctctgtctgggatttggtttagctcatctaactggt
tttcttggtccaggaatgtggactagtgattccctcaatcttgtaggctctattagattc
gtaaaagcctccttcaatctccttgcttatgcacgattagcttatggtgtcatatcatca
catcacatcatctctggattgcttggcacttccataggattatggcatatcacattacgt
ccattagcatacctctacaatctattaagcatgggaaaggttgagtccattctttcaagt
agtattacagctgtattcttcactgcattccttatttcagcccttatgtggtatggctct
gcacacaccacacaagaactcttcggtcctactagatactcatgggacaatgcttattac
tctcttgatatcagaacctcttgctcaaatgcctggaacactcttccagacaagttagtc
ctttatgattatattggatctaaccctgctaaaggtggcttatttcgttcaggaccaatg
cttaaagctgatggtcttgttcaaaactggttgggccatgcatgcttctccatgggtaca
ttatccttaagcatcagaaggatgcctgctttctttgaaaccttcccagtaatcctcatt
gaccaaacaagtacagtgagagcagacattgctttcagacggtccacatctacttatagc
atggaagaatcccaaatacaagtatacttctctggtggttgtttaaatggtacagaatat
tctacaccttcccttgtaaaagcttatgcaagaaaagctcagtttggtcagatatttaca
tttgataaaaagacatccagattgactgatggtgtatttagaacatctgctcgaggttgg
tattcattctcacacatagctttagctttctcctttttctttggccatttgtggcatgct
agtcgtgctatcttccaagacatttggacaggtgtaacatttgaatcacaagcaaaacaa
gaatatggtagaaatgaaaagctaggagataagacatcttcaactaaatccattgtttaa
>MzC1GaII_psbB
atgcaaataactgatgttttaccttggtttagggtacatatagttattcttaatgatcca
ggacgtctcattagctcacatattatgcacacagctttagtagcaggttggtcagctctc
atgcttttatatgagcttatcaccatagatcctacagatcctgtgtataatccaatttgg
agacaagcagcttacacactcccatttatctcacgtattggtgttattcgctctcttttt
agttggtcacttggcatagatcctacatccaatctaatctggacttatgaaacaatgaac
atagcccatattctattatctggtttattaattcttgcatcattttggcattgggcatat
tgggacttagatctattcttcacctcaacattaacactagaccttaatcaaatcctcagt
attcacctcacactagcatcatctctctgtctgggatttggtttagctcatctaactggt
tttcttggtccaggaatgtggactagtgattccctcaatcttgtaggctctattagattc
gtaaaagcctccttcaatctccttgcttatgcacgattagcttatggtgtcatatcatca
catcacatcatctctggattgcttggcacttccataggattatggcatatcacattacgt
ccattagcatacctctacaatctattaagcatgggaaaggttgagtccattctttcaagt
agtattacagctgtattcttcactgcattccttatttcagcccttatgtggtatggctct
gcacacaccacacaagaactcttcggtcctactagatactcatgggacaatgcttattac
tctcttgatatcagaacctcttgctcaaatgcctggaacactcttccagacaagttagtc
ctttatgattatattggatctaaccctgctaaaggtggcttatttcgttcaggaccaatg
cttaaagctgatggtcttgttcaaaactggttgggccatgcatgcttctccatgggtaca
ttatccttaagcatcagaaggatgcctgctttctttgaaaccttcccagtaatcctcatt
gaccaaacaagtacagtgagagcagacattgctttcagacggtccacatctacttatagc
atggaagaatcccaaatacaagtatacttctctggtggttgtttaaatggtacagaatat
tctacaccttcccttgtaaaagcttatgcaagaaaagctcagtttggtcagatatttaca
tttgataaaaagacatccagattgactgatggtgtatttagaacatctgctcgaggttgg
tattcattctcacacatagctttagctttctcctttttctttggccatttgtggcatgct
agtcgtgctatcttccaagacatttggacaggtgtaacatttgaatcacaagcaaaacaa
gaatatggtagaaatgaaaagctaggagataagacatcttcaactaaatccattgtttaa
>MzC2_psbB
atgcaaataactgatgctttaccttggtttagggtacatatagttattcttaatgatcca
ggacgtctcattagctcacatattatgcacacagctttagtagcaggttggtcagctctc
atgcttttatatgagcttatcaccatagatcctacagatcctgtgtataatccaatttgg
agacaagcagcttacacactcccatttatctcacgtattggtgttattcgctctcttttt

agttggtcacttggcatagatcctacatccaatctaatctggacttatgaaacaatgaac
atagcccatattctattatctggtttattaattcttgcatcattttggcattgggcatat
tgggacttagatctattcttcacctcaacattaacactagaccttaatcaaatcctcagt
attcacctcacactagcatcatctctctgtctgggatttggtttagctcatctaactggt
tttcttggtccaggaatgtggactagtgattccctcaatcttgtaggctctattagattc
gtaaaagcctccttcaatctccttgcttatgcacgattagcttatggtgtcatatcatca
catcacatcatctctggattgcttggcacttccataggattatggcatatcacattacgt
ccattagcatacctctacaatctattaagcatgggaaaggttgagtccattctttcaagt
agtattacagctgtattcttcactgcattccttatttcagcccttatgtggtatggctct
gcacacaccacacaagaactcttcggtcctactagatactcatgggacaatgcttattac
tctcttgatatcagaacctcttgctcaaatgcctggaacactcttccagacaagttagtc
ctttatgattatattggatctaaccctgctaaaggtggcttatttcgttcaggaccaatg
cttaaagctgatggtcttgttcaaaactggttgggccatgcatgcttctccatgggtaca
ttatccttaagcatcagaaggatgcctgctttctttgaaaccttcccagtaatcctcatt
gaccaaacaagtacagtgagagcagacattgctttcagacggtccacatctacttatagc
atggaagaatcccaaatacaagtatacttctctggtggttgtttaaatggtacagaatat
tctacaccttcccttgtaaaagcttatgcaagaaaagctcagtttggtcagatatttaca
tttgataaaaagacatccagattgactgatggtgtatttagaacatctgctcgaggttgg
tattcattctcacacatagctttagctttctccttttttctttggccatttgtggcatgct
agtcgtgctatcttccaagacatttggacaggtgtaacatttgaatcacaagcaaaacaa
gaatatggtagaaatgaaaagctaggagataagacatcttcaactaaatccattgtttaa
>MzC2GaII_psbB
atgcaaataactgatgctttaccttggtttagggtacatatagttattcttaatgatcca
ggacgtctcattagctcacatattatgcacacagctttagtagcaggttggtcagctctc
atgcttttatatgagcttatcaccatagatcctacagatcctgtgtataatccaatttgg
agacaagcagcttacacactcccatttatctcacgtattggtgttattcgctctcttttt
agttggtcacttggcatagatcctacatccaatctaatctggacttatgaaacaatgaac
atagcccatattctattatctggtttattaattcttgcatcattttggcattgggcatat
tgggacttagatctattcttcacctcaacattaacactagaccttaatcaaatcctcagt
attcacctcacactagcatcatctctctgtctgggatttggtttagctcatctaactggt
tttcttggtccaggaatgtggactagtgattccctcaatcttgtaggctctattagattc
gtaaaagcctccttcaatctccttgcttatgcacgattagcttatggtgtcatatcatca
catcacatcatctctggattgcttggcacttccataggattatggcatatcacattacgt
ccattagcatacctctacaatctattaagcatgggaaaggttgagtccattctttcaagt
agtattacagctgtattcttcactgcattccttatttcagcccttatgtggtatggctct
gcacacaccacacaagaactcttcggtcctactagatactcatgggacaatgcttattac
tctcttgatatcagaacctcttgctcaaatgcctggaacactcttccagacaagttagtc
ctttatgattatattggatctaaccctgctaaaggtggcttatttcgttcaggaccaatg
cttaaagctgatggtcttgttcaaaactggttgggccatgcatgcttctccatgggtaca
ttatccttaagcatcagaaggatgcctgctttctttgaaaccttcccagtaatcctcatt
gaccaaacaagtacagtgagagcagacattgctttcagacggtccacatctacttatagc
atggaagaatcccaaatacaagtatacttctctggtggttgtttaaatggtacagaatat
tctacaccttcccttgtaaaagcttatgcaagaaaagctcagtttggtcagatatttaca
tttgataaaaagacatccagattgactgatggtgtatttagaacatctgctcgaggttgg
tattcattctcacacatagctttagctttctccttttttctttggccatttgtggcatgct
agtcgtgctatcttccaagacatttggacaggtgtaacatttgaatcacaagcaaaacaa
gaatatggtagaaatgaaaagctaggagataagacatcttcaactaaatccattgtttaa
>MzC3_psbB
atgcaaataactgatgctttaccttggtttagggtacatatagttattcttaatgatcca
ggacgtctcattagctcacatattatgcacacagctttagtagcaggttggtcagctctc
atgcttttatatgagcttatcaccatagatcctacagatcctgtgtataatccaatttgg
agacaagcagcttacacactcccatttatctcacgtattggtgttattcgctctcttttc
agttggtcacttggcatagatcctacatccaatctaatctggacttatgaaacaatgaac
atagcccatattctattatctggtttattaattcttgcatcattttggcattgggcatat
tgggacttagatctattcttcacctcaacattaacactagaccttaatcaaatcctcagt
attcacctcacactagcatcatctctctgtctgggatttggtttagctcatctaactggt
tttcttggtccaggaatgtggactagtgattccctcaatcttgtaggctctattagattc

gtaaaagcctccttcaatctccttgcttatgcacgattagcttatggtgtcatatcatca
catcacatcatctctggattgcttggcacttccataggattatggcatatcacattacgt
ccattagcatacctctacaatctattaagcatgggaaaggttgagtccattctttcaagt
agtattacagctgtattcttcactgcattccttatttcagcccttatgtggtatggctct
gcacacaccacacaagaactcttcggtcctactagatactcatgggacaatgcttattac
tctcttgatatcagaacctcttgctcaaatgcctggaacactcttccagacaagttagtc
ctttatgattatattggatctaaccctgctaaaggtggcttatttcgttcaggaccaatg
cttaaagctgatggtcttgttcaaaactggttgggccatgcatgcttctccatgggtaca
ttatccttaagcatcagaaggatgcctgctttctttgaaaccttcccagtaatcctcatt
gaccaaacaagtacagtgagagcagacattgctttcagacggtccacatctacttatagc
atggaagaatcccaaatacaagtatacttctctggtggttgtttaaatggtacagaatat
tctacaccttcccttgtaaaagcttatgcaagaaaagctcagtttggtcagatatttaca
tttgataaaaagacatccagattgactgatggtgtatttagaacatctgctcgaggttgg
tattcattctcacacatagctttagctttctcctttttctttggccatttgtggcatgct
agtcgtgctatcttccaagacatttggacaggtgtaacatttgaatcacaagcaaaacaa
gaatatggtagaaatgaaaagctaggagataagacatcttcaactaaatccattgtttaa
>MzC4_psbB
atgcaaataactgatgttttaccttggtttagggtacatatagttattcttaatgatcca
ggacgtctcattagctcacatattatgcacacagctttagtagcaggttggtcagctctc
atgcttttatatgagcttatcaccatagatcctacagatcctgtgtataatccaatttgg
agacaagcagcttacacactcccatttatctcacgtattggtgttattcgctctcttttt
agttggtcacttggcatagatcctacatccaatctaatctggacttatgaaacaatgaac
atagcccatattctattatctggtttattaattcttgcatcattttggcattgggcatat
tgggacttagatctattcttcacctcaacattaacactagaccttaatcaaatcctcagt
attcacctcacactagcatcatctctctgtctgggatttggtttagctcatctaactggt
tttcttggtccaggaatgtggactagtgattccctcaatcttgtaggctctattagattc
gtaaaagcctccttcaatctccttgcttatgcacgattagcttatggtgtcatatcatca
catcacatcatctctggattgcttggcacttccataggattatggcatatcacattacgt
ccattagcatacctctacaatctattaagcatgggaaaggttgagtccattctttcaagt
agtattacagctgtattcttcactgcattccttatttcagcccttatgtggtatggctct
gcacacaccacacaagaactcttcggtcctactagatactcatgggacaatgcttattac
tctcttgatatcagaacctcttgctcaaatgcctggaacactcttccagacaagttagtc
ctttatgattatattggatctaaccctgctaaaggtggcttatttcgttcaggaccaatg
cttaaagctgatggtcttgttcaaaactggttgggccatgcatgcttctccatgggtaca
ttatccttaagcatcagaaggatgcctgctttctttgaaaccttcccagtaatcctcatt
gaccaaacaagtacagtgagagcagacattgctttcagacggtccacatctacttatagc
atggaagaatcccaaatacaagtatacttctctggtggttgtttaaatggtacagaatat
tctacaccttcccttgtaaaagcttatgcaagaaaagctcagtttggtcagatatttaca
tttgataaaaagacatccagattgactgatggtgtatttagaacatctgctcgaggttgg
tattcattctcacacatagctttagctttctcctttttctttggccatttgtggcatgct
agtcgtgctatcttccaagacatttggacaggtgtaacatttgaatcacaagcaaaacaa
gaatatggtagaaatgaaaagctaggagataagacatcttcaactaaatccattgtttaa
>Od10_psbB
atgcaaataactgatgctttaccttggtttagggtacatatagttattcttaatgatcca
ggacgtctcattagctcacatattatgcacacagctttagtagcaggttggtcagctctc
atgcttttatatgagcttatcaccatagatcctacagatcctgtgtataatccaatttgg
agacaagcagcttacacactcccatttatctcacgtattggtgttattcgctctcttttt
agttggtcacttggcatagatcctacatccaatctaatctggacttatgaaacaatgaac
atagcccatattctattatctggtttattaattcttgcatcattttggcattgggcatat
tgggacttagatctattcttcacctcaacattaacactagaccttaatcaaatcctcagt
attcacctcacactagcatcatctctctgtctgggatttggtttagctcatctaactggt
tttcttggtccaggaatgtggactagtgattccctcaatcttgtaggctctattagattc
gtaaaagcctccttcaatctccttgcttatgcacgattagcttatggtgtcatatcatca
catcacatcatctctggattgcttggcacttccataggattatggcatatcacattacgt
ccattagcatacctctacaatctattaagcatgggaaaggttgagtccattctttcaagt
agtattacagctgtattcttcactgcattccttatttcagcccttatgtggtatggctct
gcacacaccacacaagaactcttcggtcctactagatactcatgggacaatgcttattac

tctcttgatatcagaacctcttgctcaaatgcctggaacactcttccagacaagttagtc
ctttatgattatattggatctaaccctgctaaaggtggcttatttcgttcaggaccaatg
cttaaagctgatggtcttgttcaaaactggttgggccatgcatgcttctccatgggtaca
ttatccttaagcatcagaaggatgcctgctttctttgaaaccttcccagtaatcctcatt
gaccaaacaagtacagtgagagcagacattgctttcagacggtccacatctacttatagc
atggaagaatcccaaatacaagtatacttctctggtggttgtttaaatggtacagaatat
tctacaccttcccttgtaaaagcttatgcaagaaaagctcagtttggtcagatatttaca
tttgataaaaagacatccagattgactgatggtgtatttagaacatctgctcgaggttgg
tattcattctcacacatagctttagctttctcctttttctttggccatttgtggcatgct
agtcgtgctatcttccaagacatttggacaggtgtaacatttgaatcacaagcaaaacaa
gaatatggtagaaatgaaaagctaggagataagacatcttcaactaaatccattgtttaa
>Od11_psbB
atgcaaataactgatgttttaccttggtttagggtacatatagttattcttaatgatcca
ggacgtctcattagctcacatattatgcacacagctttagtagcaggttggtcagctctc
atgcttttatatgagcttatcaccatagatcctacagatcctgtgtataatccaatttgg
agacaagcagcttacacactcccatttatctcacgtattggtgttattcgctctcttttt
agttggtcacttggcatagatcctacatccaatctaatctggacttatgaaacaatgaac
atagcccatattctattatctggtttattaattcttgcatcattttggcattgggcatat
tgggacttagatctattcttcacctcaacattaacactagaccttaatcaaatcctcagt
attcacctcacactagcatcatctctctgtctgggatttggtttagctcatctaactggt
tttcttggtccaggaatgtggactagtgattccctcaatcttgtaggctctattagattc
gtaaaagcctccttcaatctccttgcttatgcacgattagcttatggtgtcatatcatca
catcacatcatctctggattgcttggcacttccataggattatggcatatcacattacgt
ccattagcatacctctacaatctattaagcatgggaaaggttgagtccattctttcaagt
agtattacagctgtattcttcactgcattccttatttcagcccttatgtggtatggctct
gcacacaccacacaagaactcttcggtcctactagatactcatgggacaatgcttattac
tctcttgatatcagaacctcttgctcaaatgcctggaacactcttccagacaagttagtc
ctttatgattatattggatctaaccctgctaaaggtggcttatttcgttcaggaccaatg
cttaaagctgatggtcttgttcaaaactggttgggccatgcatgcttctccatgggtaca
ttatccttaagcatcagaaggatgcctgctttctttgaaaccttcccagtaatcctcatt
gaccaaacaagtacagtgagagcagacattgctttcagacggtccacatctacttatagc
atggaagaatcccaaatacaagtatacttctctggtggttgtttaaatggtacagaatat
tctacaccttcccttgtaaaagcttatgcaagaaaagctcagtttggtcagatatttaca
tttgataaaaagacatccagattgactgatggtgtatttagaacatctgctcgaggttgg
tattcattctcacacatagctttagctttctcctttttctttggccatttgtggcatgct
agtcgtgctatcttccaagacatttggacaggtgtaacatttgaatcacaagcaaaacaa
gaatatggtagaaatgaaaagctaggagataagacatcttcaactaaatccattgtttaa
>Od12_psbB
atgcaaataactgatgttttaccttggtttagggtacatatagttattcttaatgatcca
ggacgtctcattagctcacatattatgcacacagctttagtagcaggttggtcagctctc
atgcttttatatgagcttatcaccatagatcctacagatcctgtgtataatccaatttgg
agacaagcagcttacacactcccatttatctcacgtattggtgttattcgctctcttttt
agttggtcacttggcatagatcctacatccaatctaatctggacttatgaaacaatgaac
atagcccatattctattatctggtttattaattcttgcatcattttggcattgggcatat
tgggacttagatctattcttcacctcaacattaacactagaccttaatcaaatcctcagt
attcacctcacactagcatcatctctctgtctgggatttggtttagctcatctaactggt
tttcttggtccaggaatgtggactagtgattccctcaatcttgtaggctctattagattc
gtaaaagcctccttcaatctccttgcttatgcacgattagcttatggtgtcatatcatca
catcacatcatctctggattgcttggcacttccataggattatggcatatcacattacgt
ccattagcatacctctacaatctattaagcatgggaaaggttgagtccattctttcaagt
agtattacagctgtattcttcactgcattccttatttcagcccttatgtggtatggctct
gcacacaccacacaagaactcttcggtcctactagatactcatgggacaatgcttattac
tctcttgatatcagaacctcttgctcaaatgcctggaacactcttccagacaagttagtc
ctttatgattatattggatctaaccctgctaaaggtggcttatttcgttcaggaccaatg
cttaaagctgatggtcttgttcaaaactggttgggccatgcatgcttctccatgggtaca
ttatccttaagcatcagaaggatgcctgctttctttgaaaccttcccagtaatcctcatt
gaccaaacaagtacagtgagagcagacattgctttcagacggtccacatctacttatagc

atggaagaatcccaaatacaagtatacttctctggtggttgtttaaatggtacagaatat
tctacaccttcccttgtaaaagcttatgcaagaaaagctcagtttggtcagatatttaca
tttgataaaaagacatccagattgactgatggtgtatttagaacatctgctcgaggttgg
tattcattctcacacatagctttagctttctcctttttctttggccatttgtggcatgct
agtcgtgctatcttccaagacatttggacaggtgtaacatttgaatcacaagcaaaacaa
gaatatggtagaaatgaaaagctaggagataagacatcttcaactaaatccattgtttaa
>Od13_psbB
atgcaaataactgatgctttaccttggtttagggtacatatagttattcttaatgatcca
ggacgtctcattagctcacatattatgcacacagctttagtagcaggttggtcagctctc
atgcttttatatgagcttatcaccatagatcctacagatcctgtgtataatccaatttgg
agacaagcagcttacacactcccatttatctcacgtattggtgttattcgctctcttttt
agttggtcacttggcatagatcctacatccaatctaatctggacttatgaaacaatgaac
atagcccatattctattatctggtttattaattcttgcatcattttggcattgggcatat
tgggacttagatctattcttcacctcaacattaacactagaccttaatcaaatcctcagt
attcacctcacactagcatcatctctctgtctgggatttggtttagctcatctaactggt
tttcttggtccaggaatgtggactagtgattccctcaatcttgtaggctctattagattc
gtaaaagcctccttcaatctccttgcttatgcacgattagcttatggtgtcatatcatca
catcacatcatctctggattgcttggcacttccataggattatggcatatcacattacgt
ccattagcatacctctacaatctattaagcatgggaaaggttgagtccattctttcaagt
agtattacagctgtattcttcactgcattccttatttcagcccttatgtggtatggctct
gcacacaccacacaagaactcttcggtcctactagatactcatgggacaatgcttattac
tctcttgatatcagaacctcttgctcaaatgcctggaacactcttccagacaagttagtc
ctttatgattatattggatctaaccctgctaaaggtggcttatttcgttcaggaccaatg
cttaaagctgatggtcttgttcaaaactggttgggccatgcatgcttctccatgggtaca
ttatccttaagcatcagaaggatgcctgctttctttgaaaccttcccagtaatcctcatt
gaccaaacaagtacagtgagagcagacattgctttcagacggtccacatctacttatagc
atggaagaatcccaaatacaagtatacttctctggtggttgtttaaatggtacagaatat
tctacaccttcccttgtaaaagcttatgcaagaaaagctcagtttggtcagatatttaca
tttgataaaaagacatccagattgactgatggtgtatttagaacatctgctcgaggttgg
tattcattctcacacatagctttagctttctcctttttctttggccatttgtggcatgct
agtcgtgctatcttccaagacatttggacaggtgtaacatttgaatcacaagcaaaacaa
gaatatggtagaaatgaaaagctaggagataagacatcttcaactaaatccattgtttaa
>Od14_psbB
atgcaaataactgatgttttaccttggtttagggtacatatagttattcttaatgatcca
ggacgtctcattagctcacatattatgcacacagctttagtagcaggttggtcagctctc
atgcttttatatgagcttatcaccatagatcctacagatcctgtgtataatccaatttgg
agacaagcagcttacacactcccatttatctcacgtattggtgttattcgctctcttttt
agttggtcacttggcatagatcctacatccaatctaatctggacttatgaaacaatgaac
atagcccatattctattatctggtttattaattcttgcatcattttggcattgggcatat
tgggacttagatctattcttcacctcaacattaacactagaccttaatcaaatcctcagt
attcacctcacactagcatcatctctctgtctgggatttggtttagctcatctaactggt
tttcttggtccaggaatgtggactagtgattccctcaatcttgtaggctctattagattc
gtaaaagcctccttcaatctccttgcttatgcacgattagcttatggtgtcatatcatca
catcacatcatctctggattgcttggcacttccataggattatggcatatcacattacgt
ccattagcatacctctacaatctattaagcatgggaaaggttgagtccattctttcaagt
agtattacagctgtattcttcactgcattccttatttcagcccttatgtggtatggctct
gcacacaccacacaagaactcttcggtcctactagatactcatgggacaatgcttattac
tctcttgatatcagaacctcttgctcaaatgcctggaacactcttccagacaagttagtc
ctttatgattatattggatctaaccctgctaaaggtggcttatttcgttcaggaccaatg
cttaaagctgatggtcttgttcaaaactggttgggccatgcatgcttctccatgggtaca
ttatccttaagcatcagaaggatgcctgctttctttgaaaccttcccagtaatcctcatt
gaccaaacaagtacagtgagagcagacattgctttcagacggtccacatctacttatagc
atggaagaatcccaaatacaagtatacttctctggtggttgtttaaatggtacagaatat
tctacaccttcccttgtaaaagcttatgcaagaaaagctcagtttggtcagatatttaca
tttgataaaaagacatccagattgactgatggtgtatttagaacatctgctcgaggttgg
tattcattctcacacatagctttagctttctcctttttctttggccatttgtggcatgct
agtcgtgctatcttccaagacatttggacaggtgtaacatttgaatcacaagcaaaacaa

gaatatggtagaaatgaaaagctaggagataagacatcttcaactaaatccattgtttaa
>Od15_psbB
atgcaaataactgatgttttaccttggtttagggtacatatagttattcttaatgatcca
ggacgtctcattagctcacatattatgcacacagctttagtagcaggttggtcagctctc
atgcttttatatgagcttatcaccatagatcctacagatcctgtgtataatccaatttgg
agacaagcagcttacacactcccatttatctctcgtatcggtgttattcgctctcttttt
agttggtcacttggcatagatcctacatccaatctaatctggacttatgaaacaatgaac
atagcccatattctattatctggtttattaattcttgcatcattttggcattgggcatat
tgggacttagatctattcttcacctcaacattaacactagaccttaatcaaatcctcagt
attcacctcacactagcatcatctctctgtctgggatttggtttagctcatctaactggt
tttcttggtccaggaatgtggactagtgattccctcaatcttgtaggctctattagattc
gtaaaagcctccttcaatctccttgcttatgcacgattagcttatggtgtcatatcatca
catcacatcatctctggattgcttggcacttccataggattatggcatatcacattacgt
ccattagcatacctctacaatctattaagcatgggaaaggttgagtccattctttcaagt
agtattacagctgtattcttcactgcattccttatttcagcccttatgtggtatggctct
gcacacaccacacaagaactcttcggtcctactagatactcatgggacaatgcttattac
tctcttgatatcagaacctcttgctcaaatgcctggaacactcttccagacaagttagtc
ctttatgattatattggatctaaccctgctaaaggtggcttatttcgttcaggaccaatg
cttaaagctgatggtcttgttcaaaactggttgggccatgcatgcttctccatgggtaca
ttatccttaagcatcagaaggatgcctgctttctttgaaaccttcccagtaatcctcatt
gaccaaacaagtacagtgagagcagacattgctttcagacggtccacatctacttatagc
atggaagaatcccaaatacaagtatacttctctggtggttgtttaaatggtacagaatat
tctacaccttcccttgtaaaagcttatgcaagaaaagctcagtttggtcagatatttaca
tttgataaaaagacatccagattgactgatggtgtatttagaacatctgctcgaggttgg
tattcattctcacacatagctttagctttctcctttttctttggccatttgtggcatgct
agtcgtgctatcttccaagacatttggacaggtgtaacatttgaatcacaagcaaaacaa
gaatatggtagaaatgaaaagctaggagataagacatcttcaactaaatccattgtttaa
>Od18_psbB
atgcaaataactgatgctttaccttggtttagggtacatatagttattcttaatgatcca
ggacgtctcattagctcacatattatgcacacagctttagtagcaggttggtcagctctc
atgcttttatatgagcttatcaccatagatcctacagatcctgtgtataatccaatttgg
agacaagcagcttacacactcccatttatctcacgtattggtgttattcgctctcttttt
agttggtcacttggcatagatcctacatccaatctaatctggacttatgaaacaatgaac
atagcccatattctattatctggtttattaattcttgcatcattttggcattgggcatat
tgggacttagatctattcttcacctcaacattaacactagaccttaatcaaatcctcagt
attcacctcacactagcatcatctctctgtctgggatttggtttagctcatctaactggt
tttcttggtccaggaatgtggactagtgattccctcaatcttgtaggctctattagattc
gtaaaagcctccttcaatctccttgcttatgcacgattagcttatggtgtcatatcatca
catcacatcatctctggattgcttggcacttccataggattatggcatatcacattacgt
ccattagcatacctctacaatctattaagcatgggaaaggttgagtccattctttcaagt
agtattacagctgtattcttcactgcattccttatttcagcccttatgtggtatggctct
gcacacaccacacaagaactcttcggtcctactagatactcatgggacaatgcttattac
tctcttgatatcagaacctcttgctcaaatgcctggaacactcttccagacaagttagtc
ctttatgattatattggatctaaccctgctaaaggtggcttatttcgttcaggaccaatg
cttaaagctgatggtcttgttcaaaactggttgggccatgcatgcttctccatgggtaca
ttatccttaagcatcagaaggatgcctgctttctttgaaaccttcccagtaatcctcatt
gaccaaacaagtacagtgagagcagacattgctttcagacggtccacatctacttatagc
atggaagaatcccaaatacaagtatacttctctggtggttgtttaaatggtacagaatat
tctacaccttcccttgtaaaagcttatgcaagaaaagctcagtttggtcagatatttaca
tttgataaaaagacatccagattgactgatggtgtatttagaacatctgctcgaggttgg
tattcattctcacacatagctttagctttctcctttttctttggccatttgtggcatgct
agtcgtgctatcttccaagacatttggacaggtgtaacatttgaatcacaagcaaaacaa
gaatatggtagaaatgaaaagctaggagataagacatcttcaactaaatccattgtttaa
>Od19_psbB
atgcaaataactgctgttttaccttggtttagggtacatatagttattcttaatgatcca
ggacgtctcattagctcacatattatgcacacagctttagtagcaggttggtcagctctc
atgcttttatatgagcttatcaccatagatcctacagatcctgtgtataatccaatttgg

agacaagcagcttacacactcccatttatctcacgtattggtgttattcgctctcttttt
agttggtcacttggcatagatcctacatccaatctaatctggacttatgaaacaatgaac
atagcccatattctattatctggtttattaattcttgcatcattttggcattgggcatat
tgggacttagatctattcttcacctcaacattaacactagaccttaatcaaatcctcagt
attcacctcacactagcatcatctctctgtctgggatttggtttagctcatctaactggt
tttcttggtccaggaatgtggactagtgattccctcaatcttgtaggctctattagattc
gtaaaagcctccttcaatctccttgcttatgcacgattagcttatggtgtcatatcatca
catcacatcatctctggattgcttggcacttccataggattatggcatatcacattacgt
ccattagcatacctctacaatctattaagcatgggaaaggttgagtccattctttcaagt
agtattacagctgtattcttcactgcattccttatttcagcccttatgtggtatggctct
gcacacaccacacaagaactcttcggtcctactagatactcatgggacaatgcttattac
tctcttgatatcagaacctcttgctcaaatgcctggaacactcttccagacaagttagtc
ctttatgattatattggatctaaccctgctaaaggtggcttatttcgttcaggaccaatg
cttaaagctgatggtcttgttcaaaactggttgggccatgcatgcttctccatgggtaca
ttatccttaagcatcagaaggatgcctgctttctttgaaaccttcccagtaatcctcatt
gaccaaacaagtacagtgagagcagacattgctttcagacggtccacatctacttatagc
atggaagaatcccaaatacaagtatacttctctggtggttgtttaaatggtacagaatat
tctacaccttcccttgtaaaagcttatgcaagaaaagctcagtttggtcagatatttaca
tttgataaaaagacatccagattgactgatggtgtatttagaacatctgctcgaggttgg
tattcattctcacacatagctttagctttctcctttttctttggccatttgtggcatgct
agtcgtgctatcttccaagacatttggacaggtgtaacatttgaatcacaagcaaaacaa
gaatatggtagaaatgaaaagctaggagataagacatcttcaactaaatccattgtttaa
>Od2_psbB
atgcaaataactgatgctttaccttggtttagggtacatatagttattcttaatgatcca
ggacgtctcattagctcacatattatgcacacagctttagtagcaggttggtcagctctc
atgcttttatatgagcttatcaccatagatcctacagatcctgtgtataatccaatttgg
agacaagcagcttacacactcccatttatctcacgtattggtgttattcgctctcttttt
agttggtcacttggcatagatcctacatccaatctaatctggacttatgaaacaatgaac
atagcccatattctattatctggtttattaattcttgcatcattttggcattgggcatat
tgggacttagatctattcttcacctcaacattaacactagaccttaatcaaatcctcagt
attcacctcacactagcatcatctctctgtctgggatttggtttagctcatctaactggt
tttcttggtccaggaatgtggactagtgattccctcaatcttgtaggctctattagattc
gtaaaagcctccttcaatctccttgcttatgcacgattagcttatggtgtcatatcatca
catcacatcatctctggattgcttggcacttccataggattatggcatatcacattacgt
ccattagcatacctctacaatctattaagcatgggaaaggttgagtccattctttcaagt
agtattacagctgtattcttcactgcattccttatttcagcccttatgtggtatggctct
gcacacaccacacaagaactcttcggtcctactagatactcatgggacaatgcttattac
tctcttgatatcagaacctcttgctcaaatgcctggaacactcttccagacaagttagtc
ctttatgattatattggatctaaccctgctaaaggtggcttatttcgttcaggaccaatg
cttaaagctgatggtcttgttcaaaactggttgggccatgcatgcttctccatgggtaca
ttatccttaagcatcagaaggatgcctgctttctttgaaaccttcccagtaatcctcatt
gaccaaacaagtacagtgagagcagacattgctttcagacggtccacatctacttatagc
atggaagaatcccaaatacaagtatacttctctggtggttgtttaaatggtacagaatat
tctacaccttcccttgtaaaagcttatgcaagaaaagctcagtttggtcagatatttaca
tttgataaaaagacatccagattgactgatggtgtatttagaacatctgctcgaggttgg
tattcattctcacacatagctttagctttctcctttttctttggccatttgtggcatgct
agtcgtgctatcttccaagacatttggacaggtgtaacatttgaatcacaagcaaaacaa
gaatatggtagaaatgaaaagctaggagataagacatcttcaactaaatccattgtttaa
>Od22_psbB
atgcaaataactgatgctttaccttggtttagggtacatatagttattcttaatgatcca
ggacgtctcattagctcacatattatgcacacagctttagtagcaggttggtcagctctc
atgcttttatatgagcttatcaccatagatcctacagatcctgtgtataatccaatttgg
agacaagcagcttacacactcccatttatctcacgtattggtgttattcgctctcttttt
agttggtcacttggcatagatcctacatccaatctaatctggacttatgaaacaatgaac
atagcccatattctattatctggtttattaattcttgcatcattttggcattgggcatat
tgggacttagatctattcttcacctcaacattaacactagaccttaatcaaatcctcagt
attcacctcacactagcatcatctctctgtctgggatttggtttagctcatctaactggt

tttcttggtccaggaatgtggactagtgattccctcaatcttgtaggctctattagattc
gtaaaagcctccttcaatctccttgcttatgcacgattagcttatggtgtcatatcatca
catcacatcatctctggattgcttggcacttccataggattatggcatatcacattacgt
ccattagcatacctctacaatctattaagcatgggaaaggttgagtccattctttcaagt
agtattacagctgtattcttcactgcattccttatttcagcccttatgtggtatggctct
gcacacaccacacaagaactcttcggtcctactagatactcatgggacaatgcttattac
tctcttgatatcagaacctcttgctcaaatgcctggaacactcttccagacaagttagtc
ctttatgattatattggatctaaccctgctaaaggtggcttatttcgttcaggaccaatg
cttaaagctgatggtcttgttcaaaactggttgggccatgcatgcttctccatgggtaca
ttatccttaagcatcagaaggatgcctgctttctttgaaaccttcccagtaatcctcatt
gaccaaacaagtacagtgagagcagacattgctttcagacggtccacatctacttatagc
atggaagaatcccaaatacaagtatacttctctggtggttgtttaaatggtacagaatat
tctacaccttcccttgtaaaagcttatgcaagaaaagctcagtttggtcagatatttaca
tttgataaaaagacatccagattgactgatggtgtatttagaacatctgctcgaggttgg
tattcattctcacacatagctttagctttctcctttttctttggccatttgtggcatgct
agtcgtgctatcttccaagacatttggacaggtgtaacatttgaatcacaagcaaaacaa
gaatatggtagaaatgaaaagctaggagataagacatcttcaactaaatccattgtttaa
>Od23_psbB
atgcaaataactgctgttttaccttggtttagggtacatatagttattcttaatgatcca
ggacgtctcattagctcacatattatgcacacagctttagtagcaggttggtcagctctc
atgcttttatatgagcttatcaccatagatcctacagatcctgtgtataatccaatttgg
agacaagcagcttacacactcccatttatctcacgtattggtgttattcgctctcttttt
agttggtcacttggcatagatcctacatccaatctaatctggacttatgaaacaatgaac
atagcccatattctattatctggtttattaattcttgcatcattttggcattgggcatat
tgggacttagatctattcttcacctcaacattaacactagaccttaatcaaatcctcagt
attcacctcacactagcatcatctctctgtctgggatttggtttagctcatctaactggt
tttcttggtccaggaatgtggactagtgattccctcaatcttgtaggctctattagattc
gtaaaagcctccttcaatctccttgcttatgcacgattagcttatggtgtcatatcatca
catcacatcatctctggattgcttggcacttccataggattatggcatatcacattacgt
ccattagcatacctctacaatctattaagcatgggaaaggttgagtccattctttcaagt
agtattacagctgtattcttcactgcattccttatttcagcccttatgtggtatggctct
gcacacaccacacaagaactcttcggtcctactagatactcatgggacaatgcttattac
tctcttgatatcagaacctcttgctcaaatgcctggaacactcttccagacaagttagtc
ctttatgattatattggatctaaccctgctaaaggtggcttatttcgttcaggaccaatg
cttaaagctgatggtcttgttcaaaactggttgggccatgcatgcttctccatgggtaca
ttatccttaagcatcagaaggatgcctgctttctttgaaaccttcccagtaatcctcatt
gaccaaacaagtacagtgagagcagacattgctttcagacggtccacatctacttatagc
atggaagaatcccaaatacaagtatacttctctggtggttgtttaaatggtacagaatat
tctacaccttcccttgtaaaagcttatgcaagaaaagctcagtttggtcagatatttaca
tttgataaaaagacatccagattgactgatggtgtatttagaacatctgctcgaggttgg
tattcattctcacacatagctttagctttctcctttttctttggccatttgtggcatgct
agtcgtgctatcttccaagacatttggacaggtgtaacatttgaatcacaagcaaaacaa
gaatatggtagaaatgaaaagctaggagataagacatcttcaactaaatccattgtttaa
>Od24_psbB
atgcaaataactgatgctttaccttggtttagggtacatatagttattcttaatgatcca
ggacgtctcattagctctcacattatgcatacagctttagtagctgggtggtcagctctc
atgcttttatatgagcttatcaccatagatcctacagatcctgtgtataatccaatttgg
agacaagcagcttacacactcccatttatctcacgtattggtgttattcgctctcttttt
agttggtcacttggcatagatcctacatccaatctaatctggacttatgaaacaatgaac
atagcccatattctattatcttgtttattaattcttgcatcattttggcattgggcatat
tgggacttagatctattcttcacctcaacattaacactagaccttaatcaaatcctcagt
attcacctcacactagcatcatctctctgtctgggatttggtttagctcatctaactggt
tttcttggtccaggaatgtggactagtgattccctcaatcttgtaggctctattagattc
gtaaaagcctccttcaatctccttgcttatgcacgattagcttatggtgtcatatcatca
catcacatcatctctggattgcttggcacttccataggattatggcatatcacattacgt
ccattagcatacctctacaatctattaagcatgggaaaggttgagtccattctttcaagt
agtattacagctgtattcttcactgcattccttatttcagccctgatgtggtatggttct

gcacataccacacaagaactattcggtcctactagatactcatgggacaatgcttattac
tctcttgatatcagaacctcttgctcaaatgcctggaacactcttccagacaagttagtc
ctttatgattatattggatctaaccctgctaaaggtggcttatttcgttcaggaccaatg
cttaaagctgatggtcttgttcaaaactggttgggccatgcatgcttctccatgggtaca
ttatccttaagcatcagaaggatgcctgctttctttgaaaccttcccagtaatcctcatt
gaccaaacaagtacagtgagagcagacattgctttcagacggtccacatctacttatagc
atggaagaatcccaaatacaagtatacttctctggtggttgtttaaatggtacagaatat
tctacaccttcccttgtaaaagcttatgcaagaaaagctcagtttggtcagatatttaca
tttgataaaaagacatccagattgactgatggtgtatttagaacatctgctcgaggttgg
tattcattctcacacatagctttagctttcctattcttctttggccatttgtggcatgct
agtcgtgctatcttccaagacatttggacaggtgtaacatttgaatcacaagcaaaacaa
gaatatggtagaaatgaaaagctaggagataagacatcttcaactaaatccattgtttaa
>Od25_psbB
atgcaaataactgatgttttaccttggtttagggtacatatagttattcttaatgatcca
ggacgtctcattagctcacatattatgcacacagctttagtagcaggttggtcagctctc
atgcttttatatgagcttatcaccatagatcctacagatcctgtgtataatccaatttgg
agacaagcagcttacacactcccatttatctcacgtattggtgttattcgctctcttttt
agttggtcacttggcatagatcctacatccaatctaatctggacttatgaaacaatgaac
atagcccatattctattatctggtttattaattcttgcatcattttggcattgggcatat
tgggacttagatctattcttcacctcaacattaacactagaccttaatcaaatcctcagt
attcacctcacactagcatcatctctctgtctgggatttggtttagctcatctaactggt
tttcttggtccaggaatgtggactagtgattccctcaatcttgtaggctctattagattc
gtaaaagcctccttcaatctccttgcttatgcacgattagcttatggtgtcatatcatca
catcacatcatctctggattgcttggcacttccataggattatggcatatcacattacgt
ccattagcatacctctacaatctattaagcatgggaaaggttgagtccattctttcaagt
agtattacagctgtattcttcactgcattccttatttcagcccttatgtggtatggctct
gcacacaccacacaagaactcttcggtcctactagatactcatgggacaatgcttattac
tctcttgatatcagaacctcttgctcaaatgcctggaacactcttccagacaagttagtc
ctttatgattatattggatctaaccctgctaaaggtggcttatttcgttcaggaccaatg
cttaaagctgatggtcttgttcaaaactggttgggccatgcatgcttctccatgggtaca
ttatccttaagcatcagaaggatgcctgctttctttgaaaccttcccagtaatcctcatt
gaccaaacaagtacagtgagagcagacattgctttcagacggtccacatctacttatagc
atggaagaatcccaaatacaagtatacttctctggtggttgtttaaatggtacagaatat
tctacaccttcccttgtaaaagcttatgcaagaaaagctcagtttggtcagatatttaca
tttgataaaaagacatccagattgactgatggtgtatttagaacatctgctcgaggttgg
tattcattctcacacatagctttagctttctcctttttctttggccatttgtggcatgct
agtcgtgctatcttccaagacatttggacaggtgtaacatttgaatcacaagcaaaacaa
gaatatggtagaaatgaaaagctaggagataagacatcttcaactaaatccattgtttaa
>Od26_psbB
atgcaaataactgatgctttaccttggtttagggtacatatagttattcttaatgatcca
ggacgtctcattagctcacatattatgcacacagctttagtagcaggttggtcagctctc
atgcttttatatgagcttatcaccatagatcctacagatcctgtgtataatccaatttgg
agacaagcagcttacacactcccatttatctcacgtattggtgttattcgctctcttttt
agttggtcacttggcatagatcctacatccaatctaatctggacttatgaaacaatgaac
atagcccatattctattatctggtttattaattcttgcatcattttggcattgggcatat
tgggacttagatctattcttcacctcaacattaacactagaccttaatcaaatcctcagt
attcacctcacactagcatcatctctctgtctgggatttggtttagctcatctaactggt
tttcttggtccaggaatgtggactagtgattccctcaatcttgtaggctctattagattc
gtaaaagcctccttcaatctccttgcttatgcacgattagcttatggtgtcatatcatca
catcacatcatctctggattgcttggcacttccataggattatggcatatcacattacgt
ccattagcatacctctacaatctattaagcatgggaaaggttgagtccattctttcaagt
agtattacagctgtattcttcactgcattccttatttcagcccttatgtggtatggctct
gcacacaccacacaagaactcttcggtcctactagatactcatgggacaatgcttattac
tctcttgatatcagaacctcttgctcaaatgcctggaacactcttccagacaagttagtc
ctttatgattatattggatctaaccctgctaaaggtggcttatttcgttcaggaccaatg
cttaaagctgatggtcttgttcaaaactggttgggccatgcatgcttctccatgggtaca
ttatccttaagcatcagaaggatgcctgctttctttgaaaccttcccagtaatcctcatt

gaccaaacaagtacagtgagagcagacattgctttcagacggtccacatctacttatagc
atggaagaatcccaaatacaagtatacttctctggtggttgtttaaatggtacagaatat
tctacaccttcccttgtaaaagcttatgcaagaaaagctcagtttggtcagatatttaca
tttgataaaaagacatccagattgactgatggtgtatttagaacatctgctcgaggttgg
tattcattctcacacatagctttagctttctcctttttctttggccatttgtggcatgct
agtcgtgctatcttccaagacatttggacaggtgtaacatttgaatcacaagcaaaacaa
gaatatggtagaaatgaaaagctaggagataagacatcttcaactaaatccattgtttaa
>Od27_psbB
atgcaaataactgatgctttaccttggtttagggtacatatagttattcttaatgatcca
ggacgtctcattagctcacatattatgcacacagctttagtagcaggttggtcagctctc
atgcttttatatgagcttatcaccatagatcctacagatcctgtgtataatccaatttgg
agacaagcagcttacacactcccatttatctcacgtattggtgttattcgctctcttttt
agttggtcacttggcatagatcctacatccaatctaatctggacttatgaaacaatgaac
atagcccatattctattatctggtttattaattcttgcatcattttggcattgggcatat
tgggacttagatctattcttcacctcaacattaacactagaccttaatcaaatcctcagt
attcacctcacactagcatcatctctctgtctgggatttggtttagctcatctaactggt
tttcttggtccaggaatgtggactagtgattccctcaatcttgtaggctctattagattc
gtaaaagcctccttcaatctccttgcttatgcacgattagcttatggtgtcatatcatca
catcacatcatctctggattgcttggcacttccataggattatggcatatcacattacgt
ccattagcatacctctacaatctattaagcatgggaaaggttgagtccattctttcaagt
agtattacagctgtattcttcactgcattccttatttcagcccttatgtggtatggctct
gcacacaccacacaagaactcttcggtcctactagatactcatgggacaatgcttattac
tctcttgatatcagaacctcttgctcaaatgcctggaacactcttccagacaagttagtc
ctttatgattatattggatctaaccctgctaaaggtggcttatttcgttcaggaccaatg
cttaaagctgatggtcttgttcaaaactggttgggccatgcatgcttctccatgggtaca
ttatccttaagcatcagaaggatgcctgctttctttgaaaccttcccagtaatcctcatt
gaccaaacaagtacagtgagagcagacattgctttcagacggtccacatctacttatagc
atggaagaatcccaaatacaagtatacttctctggtggttgtttaaatggtacagaatat
tctacaccttcccttgtaaaagcttatgcaagaaaagctcagtttggtcagatatttaca
tttgataaaaagacatccagattgactgatggtgtatttagaacatctgctcgaggttgg
tattcattctcacacatagctttagctttctcctttttctttggccatttgtggcatgct
agtcgtgctatcttccaagacatttggacaggtgtaacatttgaatcacaagcaaaacaa
gaatatggtagaaatgaaaagctaggagataagacatcttcaactaaatccattgtttaa
>Od4_psbB
atgcaaataactgatgctttaccttggtttagggtacatatagttattcttaatgatcca
ggacgtctcattagctcacatattatgcacacagctttagtagcaggttggtcagctctc
atgcttttatatgagcttatcaccatagatcctacagatcctgtgtataatccaatttgg
agacaagcagcttacacactcccatttatctcacgtattggtgttattcgctctcttttt
agttggtcacttggcatagatcctacatccaatctaatctggacttatgaaacaatgaac
atagcccatattctattatctggtttattaattcttgcatcattttggcattgggcatat
tgggacttagatctattcttcacctcaacattaacactagaccttaatcaaatcctcagt
attcacctcacactagcatcatctctctgtctgggatttggtttagctcatctaactggt
tttcttggtccaggaatgtggactagtgattccctcaatcttgtaggctctattagattc
gtaaaagcctccttcaatctccttgcttatgcacgattagcttatggtgtcatatcatca
catcacatcatctctggattgcttggcacttccataggattatggcatatcacattacgt
ccattagcatacctctacaatctattaagcatgggaaaggttgagtccattctttcaagt
agtattacagctgtattcttcactgcattccttatttcagcccttatgtggtatggctct
gcacacaccacacaagaactcttcggtcctactagatactcatgggacaatgcttattac
tctcttgatatcagaacctcttgctcaaatgcctggaacactcttccagacaagttagtc
ctttatgattatattggatctaaccctgctaaaggtggcttatttcgttcaggaccaatg
cttaaagctgatggtcttgttcaaaactggttgggccatgcatgcttctccatgggtaca
ttatccttaagcatcagaaggatgcctgctttctttgaaaccttcccagtaatcctcatt
gaccaaacaagtacagtgagagcagacattgctttcagacggtccacatctacttatagc
atggaagaatcccaaatacaagtatacttctctggtggttgtttaaatggtacagaatat
tctacaccttcccttgtaaaagcttatgcaagaaaagctcagtttggtcagatatttaca
tttgataaaaagacatccagattgactgatggtgtatttagaacatctgctcgaggttgg
tattcattctcacacatagctttagctttctcctttttctttggccatttgtggcatgct

agtcgtgctatcttccaagacatttggacaggtgtaacatttgaatcacaagcaaaacaa
gaatatggtagaaatgaaaagctaggagataagacatcttcaactaaatccattgtttaa
>Od6_psbB
atgcaaataactgatgctttaccttggtttagggtacatatagttattcttaatgatcca
ggacgtctcattagctcacatattatgcacacagctttagtagcaggttggtcagctctc
atgcttttatatgagcttatcaccatagatcctacagatcctgtgtataatccaatttgg
agacaagcagcttacacactcccatttatctcacgtattggtgttattcgctctcttttt
agttggtcacttggcatagatcctacatccaatctaatctggacttatgaaacaatgaac
atagcccatattctattatctggtttattaattcttgcatcattttggcattgggcatat
tgggacttagatctattcttcacctcaacattaacactagaccttaatcaaatcctcagt
attcacctcacactagcatcatctctctgtctgggatttggtttagctcatctaactggt
tttcttggtccaggaatgtggactagtgattccctcaatcttgtaggctctattagattc
gtaaaagcctccttcaatctccttgcttatgcacgattagcttatggtgtcatatcatca
catcacatcatctctggattgcttggcacttccataggattatggcatatcacattacgt
ccattagcatacctctacaatctattaagcatgggaaaggttgagtccattctttcaagt
agtattacagctgtattcttcactgcattccttatttcagcccttatgtggtatggctct
gcacacaccacacaagaactcttcggtcctactagatactcatgggacaatgcttattac
tctcttgatatcagaacctcttgctcaaatgcctggaacactcttccagacaagttagtc
ctttatgattatattggctctaaccctgctaaaggtggcttatttcgttcaggaccaatg
cttaaagctgatggtcttgttcaaaactggttgggccatgcatgcttctccatgggtaca
ttatccttaagcatcagaaggatgcctgctttctttgaaaccttcccagtaatcctcatt
gaccaaacaagtacagtgagagcagacattgctttcagacggtccacatctacttatagc
atggaagaatcccaaatacaagtatacttctctggtggttgtttaaatggtacagaatat
tctacaccttcccttgtaaaagcttatgcaagaaaagctcagtttggtcagatatttaca
tttgataaaaagacatccagattgactgatggtgtatttagaacatctgctcgaggttgg
tattcattctcacacatagctttagctttctcctttttctttggccatttgtggcatgct
agtcgtgctatcttccaagacatttggacaggtgtaacatttgaatcacaagcaaaacaa
gaatatggtagaaatgaaaagctaggagataagacatcttcaactaaatccattgtttaa
>Od8_psbB
atgcaaataactgatgttttaccttggtttagggtacatatagttattcttaatgatcca
ggacgtctcattagctcacatattatgcacacagctttagtagcaggttggtcagctctc
atgcttttatatgagcttatcaccatagatcctacagatcctgtgtataatccaatttgg
agacaagcagcttacacactcccatttatctcacgtattggtgttattcgctctcttttt
agttggtcacttggcatagatcctacatccaatctaatctggacttatgaaacaatgaac
atagcccatattctattatctggtttattaattcttgcatcattttggcattgggcatat
tgggacttagatctattcttcacctcaacattaacactagaccttaatcaaatcctcagt
attcacctcacactagcatcatctctctgtctgggatttggtttagctcatctaactggt
tttcttggtccaggaatgtggactagtgattccctcaatcttgtaggctctattagattc
gtaaaagcctccttcaatctccttgcttatgcacgattagcttatggtgtcatatcatca
catcacatcatctctggattgcttggcacttccataggattatggcatatcacattacgt
ccattagcatacctctacaatctattaagcatgggaaaggttgagtccattctttcaagt
agtattacagctgtattcttcactgcattccttatttcagcccttatgtggtatggctct
gcacacaccacacaagaactcttcggtcctactagatactcatgggacaatgcttattac
tctcttgatatcagaacctcttgctcaaatgcctggaacactcttccagacaagttagtc
ctttatgattatattggatctaaccctgctaaaggtggcttatttcgttcaggaccaatg
cttaaagctgatggtcttgttcaaaactggttgggccatgcatgcttctccatgggtaca
ttatccttaagcatcagaaggatgcctgctttctttgaaaccttcccagtaatcctcatt
gaccaaacaagtacagtgagagcagacattgctttcagacggtccacatctacttatagc
atggaagaatcccaaatacaagtatacttctctggtggttgtttaaatggtacagaatat
tctacaccttcccttgtaaaagcttatgcaagaaaagctcagtttggtcagatatttaca
tttgataaaaagacatccagattgactgatggtgtatttagaacatctgctcgaggttgg
tattcattctcacacatagctttagctttctcctttttctttggccatttgtggcatgct
agtcgtgctatcttccaagacatttggacaggtgtaacatttgaatcacaagcaaaacaa
gaatatggtagaaatgaaaagctaggagataagacatcttcaactaaatccattgtttaa
>Od9_psbB
atgcaaataactgatgttttaccttggtttagggtacatatagttattcttaatgatcca
ggacgtctcattagctcacatattatgcacacagctttagtagcaggttggtcagctctc

atgcttttatatgagcttatcaccatagatcctacagatcctgtgtataatccaatttgg
agacaagcagcttacacactcccatttatctcacgtattggtgttattcgctctcttttt
agttggtcacttggcatagatcctacatccaatctaatctggacttatgaaacaatgaac
atagcccatattctattatctggtttattaattcttgcatcattttggcattgggcatat
tgggacttagatctattcttcacctcaacattaacactagaccttaatcaaatcctcagt
attcacctcacactagcatcatctctctgtctgggatttggtttagctcatctaactggt
tttcttggtccaggaatgtggactagtgattccctcaatcttgtaggctctattagattc
gtaaaagcctccttcaatctccttgcttatgcacgattagcttatggtgtcatatcatca
catcacatcatctctggattgcttggcacttccataggattatggcatatcacattacgt
ccattagcatacctctacaatctattaagcatgggaaaggttgagtccattctttcaagt
agtattacagctgtattcttcactgcattccttatttcagcccttatgtggtatggctct
gcacacaccacacaagaactcttcggtcctactagatactcatgggacaatgcttattac
tctcttgatatcagaacctcttgctcaaatgcctggaacactcttccagacaagttagtc
ctttatgattatattggatctaaccctgctaaaggtggcttatttcgttcaggaccaatg
cttaaagctgatggtcttgttcaaaactggttgggccatgcatgcttctccatgggtaca
ttatccttaagcatcagaaggatgcctgctttctttgaaaccttcccagtaatcctcatt
gaccaaacaagtacagtgagagcagacattgctttcagacggtccacatctacttatagc
atggaagaatcccaaatacaagtatacttctctggtggttgtttaaatggtacagaatat
tctacaccttcccttgtaaaagcttatgcaagaaaagctcagtttggtcagatatttaca
tttgataaaaagacatccagattgactgatggtgtatttagaacatctgctcgaggttgg
tattcattctcacacatagctttagctttctcctttttctttggccatttgtggcatgct
agtcgtgctatcttccaagacatttggacaggtgtaacatttgaatcacaagcaaaacaa
gaatatggtagaaatgaaaagctaggagataagacatcttcaactaaatccattgtttaa
>Ss5_psbB
atgcaaataactgatgctttaccttggtttagggtacatatagttattcttaatgatcca
ggacgtctcattagctcacatattatgcacacagctttagtagcaggttggtcagctctc
atgcttttatatgagcttatcaccatagatcctacagatcctgtgtataatccaatttgg
agacaagcagcttacacactcccatttatctcacgtattggtgttattcgctctcttttt
agttggtcacttggcatagatcctacatccaatctaatctggacttatgaaacaatgaac
atagcccatattctattatctggtttattaattcttgcatcattttggcattgggcatat
tgggacttagatctattcttcacctcaacattaacactagaccttaatcaaatcctcagt
attcacctcacactagcatcatctctctgtctgggatttggtttagctcatctaactggt
tttcttggtccaggaatgtggactagtgattccctcaatcttgtaggctctattagattc
gtaaaagcctccttcaatctccttgcttatgcacgattagcttatggtgtcatatcatca
catcacatcatctctggattgcttggcacttccataggattatggcatatcacattacgt
ccattagcatacctctacaatctattaagcatgggaaaggttgagtccattctttcaagt
agtattacagctgtattcttcactgcattccttatttcagcccttatgtggtatggctct
gcacacaccacacaagaactcttcggtcctactagatactcatgggacaatgcttattac
tctcttgatatcagaacctcttgctcaaatgcctggaacactcttccagacaagttagtc
ctttatgattatattggatctaaccctgctaaaggtggcttatttcgttcaggaccaatg
cttaaagctgatggtcttgttcaaaactggttgggccatgcatgcttctccatgggtaca
ttatccttaagcatcagaaggatgcctgctttctttgaaaccttcccagtaatcctcatt
gaccaaacaagtacagtgagagcagacattgctttcagacggtccacatctacttatagc
atggaagaatcccaaatacaagtatacttctctggtggttgtttaaatggtacagaatat
tctacaccttcccttgtaaaagcttatgcaagaaaagctcagtttggtcagatatttaca
tttgataaaaagacatccagattgactgatggtgtatttagaacatctgctcgaggttgg
tattcattctcacacatagctttagctttctcctttttctttggccatttgtggcatgct
agtcgtgctatcttccaagacatttggacaggtgtaacatttgaatcacaagcaaaacaa
gaatatggtagaaatgaaaagctaggagataagacatcttcaactaaatccattgtttaa
>Ss6_psbB
atgcaaataactgatgctttaccttggtttagggtacatatagttattcttaatgatcca
ggacgtctcattagctctcatattatgcacacagctttagtagcaggttggtcagctctc
atgcttttatatgagcttatcaccatagatcctacagatcctgtgtataatccaatttgg
agacaagcagcttacacactcccatttatctctcgtattggtgttattcgctctcttttt
agttggtcacttggcatagatcctacatccaatctaatctggacttatgaaacaatgaac
atagcccatattctattatctggtttattaattcttgcatcattttggcattgggcatat
tgggacttagatctattcttcacctcaacattaacactagaccttaatcaaatcctcagt

attcacctcacactagcatcatctctctgtctgggatttggtttagctcatctaactggt
tttcttggtccaggaatgtggactagtgattccctcaatcttgtaggctctattagattc
gtaaaagcctccttcaatctccttgcttatgcacgattagcttatggtgtcatatcatca
catcacatcatctctggattgcttggcacttccataggattatggcatatcacattacgt
ccattagcatacctctacaatctattaagcatgggaaaggttgagtccattctttcaagt
agtattacagctgtattcttcactgcattccttatttcagcccttatgtggtatggctct
gcacacaccacacaagaactcttcggtcctactagatactcatgggacaatgcttattac
tctcttgatatcagaacctcttgctcaaatgcctggaacactcttccagacaagttagtc
ctttatgattatattggatctaaccctgctaaaggtggcttatttcgttcaggaccaatg
cttaaagctgatggtcttgttcaaaactggttgggccatgcatgcttctccatgggtaca
ttatccttaagcatcagaaggatgcctgctttctttgaaaccttcccagtaatcctcatt
gaccaaacaagtacagtgagagcagacattgctttcagacggtccacatctacttatagc
atggaagaatcccaaatacaagtatacttctctggtggttgtttaaatggtacagaatat
tctacaccttcccttgtaaaagcttatgcaagaaaagctcagtttggtcagatatttaca
tttgataaaaagacatccagattgactgatggtgtatttagaacatctgctcgaggttgg
tattcattctcacacatagctttagctttcctattcttctttggccatttgtggcatgct
agtcgtgctatcttccaagacatttggacaggtgtaacatttgaatcacaagcaaaacaa
gaatatggtagaaatgaaaagctaggagataagacatcttcaactaaatccattgtttaa
>Ss7_psbB
atgcaaataactgatgctttaccttggtttagggtacatatagttattcttaatgatcca
ggacgtctcattagctcacatattatgcacacagctttagtagcaggttggtcagctctc
atgcttttatatgagcttatcaccatagatcctacagatcctgtgtataatccaatttgg
agacaagcagcttacacactcccatttatctcacgtattggtgttattcgctctcttttt
agttggtcacttggcatagatcctacatccaatctaatctggacttatgaaacaatgaac
atagcccatattctattatctggtttattaattcttgcatcattttggcattgggcatat
tgggacttagatctattcttcacctcaacattaacactagaccttaatcaaatcctcagt
attcacctcacactagcatcatctctctgtctgggatttggtttagctcatctaactggt
tttcttggtccaggaatgtggactagtgattccctcaatcttgtaggctctattagattc
gtaaaagcctccttcaatctccttgcttatgcacgattagcttatggtgtcatatcatca
catcacatcatctctggattgcttggcacttccataggattatggcatatcacattacgt
ccattagcatacctctacaatctattaagcatgggaaaggttgagtccattctttcaagt
agtattacagctgtattcttcactgcattccttatttcagcccttatgtggtatggctct
gcacacaccacacaagaactcttcggtcctactagatactcatgggacaatgcttattac
tctcttgatatcagaacctcttgctcaaatgcctggaacactcttccagacaagttagtc
ctttatgattatattggatctaaccctgctaaaggtggcttatttcgttcaggaccaatg
cttaaagctgatggtcttgttcaaaactggttgggccatgcatgcttctccatgggtaca
ttatccttaagcatcagaaggatgcctgctttctttgaaaccttcccagtaatcctcatt
gaccaaacaagtacagtgagagcagacattgctttcagacggtccacatctacttatagc
atggaagaatcccaaatacaagtatacttctctggtggttgtttaaatggtacagaatat
tctacaccttcccttgtaaaagcttatgcaagaaaagctcagtttggtcagatatttaca
tttgataaaaagacatccagattgactgatggtgtatttagaacatctgctcgaggttgg
tattcattctcacacatagctttagctttctccttttctttggccatttgtggcatgct
agtcgtgctatcttccaagacatttggacaggtgtaacatttgaatcacaagcaaaacaa
gaatatggtagaaatgaaaagctaggagataagacatcttcaactaaatccattgtttaa
>Ss8_psbB
atgcaaataactgatgctttaccttggtttagggtacatatagttattcttaatgatcca
ggacgtctcattagctcacatattatgcacacagctttagtagcaggttggtcagctctc
atgcttttatatgagcttatcaccatagatcctacagatcctgtgtataatccaatttgg
agacaagcagcttacacactcccatttatctcacgtattggtgttattcgctctcttttt
agttggtcacttggcatagatcctacatccaatctaatctggacttatgaaacaatgaac
atagcccatattctattatctggtttattaattcttgcatcattttggcattgggcatat
tgggacttagatctattcttcacctcaacattaacactagaccttaatcaaatcctcagt
attcacctcacactagcatcatctctctgtctgggatttggtttagctcatctaactggt
tttcttggtccaggaatgtggactagtgattccctcaatcttgtaggctctattagattc
gtaaaagcctccttcaatctccttgcttatgcacgattagcttatggtgtcatatcatca
catcacatcatctctggattgcttggcacttccataggattatggcatatcacattacgt
ccattagcatacctctacaatctattaagcatgggaaaggttgagtccattctttcaagt

agtattacagctgtattcttcactgcattccttatttcagcccttatgtggtatggctct
gcacacaccacacaagaactcttcggtcctactagatactcatgggacaatgcttattac
tctcttgatatcagaacctcttgctcaaatgcctggaacactcttccagacaagttagtc
ctttatgattatattggatctaaccctgctaaaggtggcttatttcgttcaggaccaatg
cttaaagctgatggtcttgttcaaaactggttgggccatgcatgcttctccatgggtaca
ttatccttaagcatcagaaggatgcctgctttctttgaaaccttcccagtaatcctcatt
gaccaaacaagtacagtgagagcagacattgctttcagacggtccacatctacttatagc
atggaagaatcccaaatacaagtatacttctctggtggttgtttaaatggtacagaatat
tctacaccttcccttgtaaaagcttatgcaagaaaagctcagtttggtcagatatttaca
tttgataaaaagacatccagattgactgatggtgtatttagaacatctgctcgaggttgg
tattcattctcacacatagctttagctttctcctttttctttggccatttgtggcatgct
agtcgtgctatcttccaagacatttggacaggtgtaacatttgaatcacaagcaaaacaa
gaatatggtagaaatgaaaagctaggagataagacatcttcaactaaatccattgtttaa
>ohdo1_psbB
atgcaaataactgatgctttaccttggtttagggtacatatagttattcttaatgatcca
ggacgtctcattagctcacatattatgcacacagctttagtagcaggttggtcagctctc
atgcttttatatgagcttatcaccatagatcctacagatcctgtgtataatccaatttgg
agacaagcagcttacacactcccatttatctcacgtattggtgttattcgctctcttttt
agttggtcacttggcatagatcctacatccaatctaatctggacttatgaaacaatgaac
atagcccatattctattatctggtttattaattcttgcatcattttggcattgggcatat
tgggacttagatctattcttcacctcaacattaacactagaccttaatcaaatcctcagt
attcacctcacactagcatcatctctctgtctgggatttggtttagctcatctaactggt
tttcttggtccaggaatgtggactagtgattccctcaatcttgtaggctctattagattc
gtaaaagcctccttcaatctccttgcttatgcacgattagcttatggtgtcatatcatca
catcacatcatctctggattgcttggcacttccataggattatggcatatcacattacgt
ccattagcatacctctacaatctattaagcatgggaaaggttgagtccattctttcaagt
agtattacagctgtattcttcactgcattccttatttcagcccttatgtggtatggctct
gcacacaccacacaagaactcttcggtcctactagatactcatgggacaatgcttattac
tctcttgatatcagaacctcttgctcaaatgcctggaacactcttccagacaagttagtc
ctttatgattatattggatctaaccctgctaaaggtggcttatttcgttcaggaccaatg
cttaaagctgatggtcttgttcaaaactggttgggccatgcatgcttctccatgggtaca
ttatccttaagcatcagaaggatgcctgctttctttgaaaccttcccagtaatcctcatt
gaccaaacaagtacagtgagagcagacattgctttcagacggtccacatctacttatagc
atggaagaatcccaaatacaagtatacttctctggtggttgtttaaatggtacagaatat
tctacaccttcccttgtaaaagcttatgcaagaaaagctcagtttggtcagatatttaca
tttgataaaaagacatccagattgactgatggtgtatttagaacatctgctcgaggttgg
tattcattctcacacatagctttagctttctcctttttctttggccatttgtggcatgct
agtcgtgctatcttccaagacatttggacaggtgtaacatttgaatcccaagcaaaacaa
gaatatggtagaaatgaaaagctaggagataagacatcttcaactaaatccattgtttaa
>ohdo3_psbB
atgcaaataactgatgctttaccttggtttagggtacatatagttattcttaatgatcca
ggacgtctcattagctctcatattatgcacacagctttagtagcaggttggtcagctctc
atgcttttatatgagcttatcaccatagatcctacagatcctgtgtataatccaatttgg
agacaagcagcttacacactcccatttatctctcgtattggtgttattcgctctcttttt
agttggtcacttggcatagatcctacatccaatctaatctggacttatgaaacaatgaac
atagcccatattctattatctggtttattaattcttgcatcattttggcattgggcatat
tgggacttagatctattcttcacctcaacattaacactagaccttaatcaaatcctcagt
attcacctcacactagcatcatctctctgtctgggatttggtttagctcatctaactggt
tttcttggtccaggaatgtggactagtgattccctcaatcttgtaggctctattagattc
gtaaaagcctccttcaatctccttgcttatgcacgattagcttatggtgtcatatcatca
catcacatcatctctggattgcttggcacttccataggattatggcatatcacattacgt
ccattagcatacctctacaatctattaagcatgggaaaggttgagtccattctttcaagt
agtattacagctgtattcttcactgcattccttatttcagcccttatgtggtatggctct
gcacacaccacacaagaactcttcggtcctactagatactcatgggacaatgcttattac
tctcttgatatcagaacctcttgctcaaatgcctggaacactcttccagacaagttagtc
ctttatgattatattggatctaaccctgctaaaggtggcttatttcgttcaggaccaatg
cttaaagctgatggtcttgttcaaaactggttgggccatgcatgcttctccatgggtaca

ttatccttaagcatcagaaggatgcctgctttctttgaaaccttcccagtaatcctcatt
gaccaaacaagtacagtgagagcagacattgctttcagacggtccacatctacttatagc
atggaagaatcccaaatacaagtatacttctctggtggttgtttaaatggtacagaatat
tctacaccttcccttgtaaaagcttatgcaagaaaagctcagtttggtcagatatttaca
tttgataaaaagacatccagattgactgatggtgtatttagaacatctgctcgaggttgg
tattcattctcacacatagctttagctttctcctttttctttggccatttgtggcatgct
agtcgtgctatcttccaagacatttggacaggtgtaacatttgaatcacaagcaaaacaa
gaatatggtagaaatgaaaagctaggagataagacatcttcaactaaatccattgtttaa
>ohdo5_psbB
atgcaaataactgctgttttaccttggtttagggtacatatagttattcttaatgatcca
ggacgtctcattagctcacatattatgcacacagctttagtagcaggttggtcagctctc
atgcttttatatgagcttatcaccatagatcctacagatcctgtgtataatccaatttgg
agacaagcagcttacacactcccatttatctcacgtattggtgttattcgctctcttttt
agttggtcacttggcatagatcctacatccaatctaatctggacttatgaaacaatgaac
atagcccatattctattatctggtttattaattcttgcatcattttggcattgggcatat
tgggacttagatctattcttcacctcaacattaacactagaccttaatcaaatcctcagt
attcacctcacactagcatcatctctctgtctgggatttggtttagctcatctaactggt
tttcttggtccaggaatgtggactagtgattccctcaatcttgtaggctctattagattc
gtaaaagcctccttcaatctccttgcttatgcacgattagcttatggtgtcatatcatca
catcacatcatctctggattgcttggcacttccataggattatggcatatcacattacgt
ccattagcatacctctacaatctattaagcatgggaaaggttgagtccattctttcaagt
agtattacagctgtattcttcactgcattccttatttcagcccttatgtggtatggctct
gcacacaccacacaagaactcttcggtcctactagatactcatgggacaatgcttattac
tctcttgatatcagaacctcttgctcaaatgcctggaacactcttccagacaagttagtc
ctttatgattatattggatctaaccctgctaaaggtggcttatttcgttcaggaccaatg
cttaaagctgatggtcttgttcaaaactggttgggccatgcatgcttctccatgggtaca
ttatccttaagcatcagaaggatgcctgctttctttgaaaccttcccagtaatcctcatt
gaccaaacaagtacagtgagagcagacattgctttcagacggtccacatctacttatagc
atggaagaatcccaaatacaagtatacttctctggtggttgtttaaatggtacagaatat
tctacaccttcccttgtaaaagcttatgcaagaaaagctcagtttggtcagatatttaca
tttgataaaaagacatccagattgactgatggtgtatttagaacatctgctcgaggttgg
tattcattctcacacatagctttagctttctcctttttctttggccatttgtggcatgct
agtcgtgctatcttccaagacatttggacaggtgtaacatttgaatcacaagcaaaacaa
gaatatggtagaaatgaaaagctaggagataagacatcttcaactaaatccattgtttaa
>ohdo7_psbB
atgcaaataactgatgttttaccttggtttagggtacatatagttattcttaatgatcca
ggacgtctcattagctcacatattatgcacacagctttagtagcaggttggtcagctctc
atgcttttatatgagcttatcaccatagatcctacagatcctgtgtataatccaatttgg
agacaagcagcttacacactcccatttatctcacgtattggtgttattcgctctcttttt
agttggtcacttggcatagatcctacatccaatctaatctggacttatgaaacaatgaac
atagcccatattctattatctggtttattaattcttgcatcattttggcattgggcatat
tgggacttagatctattcttcacctcaacattaacactagaccttaatcaaatcctcagt
attcacctcacactagcatcatctctctgtctgggatttggtttagctcatctaactggt
tttcttggtccaggaatgtggactagtgattccctcaatcttgtaggctctattagattc
gtaaaagcctccttcaatctccttgcttatgcacgattagcttatggtgtcatatcatca
catcacatcatctctggattgcttggcacttccataggattatggcatatcacattacgt
ccattagcatacctctacaatctattaagcatgggaaaggttgagtccattctttcaagt
agtattacagctgtattcttcactgcattccttatttcagcccttatgtggtatggctct
gcacacaccacacaagaactcttcggtcctactagatactcatgggacaatgcttattac
tctcttgatatcagaacctcttgctcaaatgcctggaacactcttccagacaagttagtc
ctttatgattatattggatctaaccctgctaaaggtggcttatttcgttcaggaccaatg
cttaaagctgatggtcttgttcaaaactggttgggccatgcatgcttctccatgggtaca
ttatccttaagcatcagaaggatgcctgctttctttgaaaccttcccagtaatcctcatt
gaccaaacaagtacagtgagagcagacattgctttcagacggtccacatctacttatagc
atggaagaatcccaaatacaagtatacttctctggtggttgtttaaatggtacagaatat
tctacaccttcccttgtaaaagcttatgcaagaaaagctcagtttggtcagatatttaca
tttgataaaaagacatccagattgactgatggtgtatttagaacatctgctcgaggttgg

tattcattctcacacatagctttagctttctcctttttctttggccatttgtggcatgct
agtcgtgctatcttccaagacatttggacaggtgtaacatttgaatcacaagcaaaacaa
gaatatggtagaaatgaaaagctaggagataagacatcttcaactaaatccattgtttaa
>sesoko1_psbB
atgcaaataactgctgttttaccttggtttagggtacatatagttattcttaatgatcca
ggacgtctcattagctcacatattatgcacacagctttagtagcaggttggtcagctctc
atgcttttatatgagcttatcaccatagatcctacagatcctgtgtataatccaatttgg
agacaagcagcttacacactcccatttatctcacgtattggtgttattcgctctcttttt
agttggtcacttggcatagatcctacatccaatctaatctggacttatgaaacaatgaac
atagcccatattctattatctggtttattaattcttgcatcattttggcattgggcatat
tgggacttagatctattcttcacctcaacattaacactagaccttaatcaaatcctcagt
attcacctcacactagcatcatctctctgtctgggatttggtttagctcatctaactggt
tttcttggtccaggaatgtggactagtgattccctcaatcttgtaggctctattagattc
gtaaaagcctccttcaatctccttgcttatgcacgattagcttatggtgtcatatcatca
catcacatcatctctggattgcttggcacttccataggattatggcatatcacattacgt
ccattagcatacctctacaatctattaagcatgggaaaggttgagtccattctttcaagt
agtattacagctgtattcttcactgcattccttatttcagcccttatgtggtatggctct
gcacacaccacacaagaactcttcggtcctactagatactcatgggacaatgcttattac
tctcttgatatcagaacctcttgctcaaatgcctggaacactcttccagacaagttagtc
ctttatgattatattggatctaaccctgctaaaggtggcttatttcgttcaggaccaatg
cttaaagctgatggtcttgttcaaaactggttgggccatgcatgcttctccatgggtaca
ttatccttaagcatcagaaggatgcctgctttctttgaaaccttcccagtaatcctcatt
gaccaaacaagtacagtgagagcagacattgctttcagacggtccacatctacttatagc
atggaagaatcccaaatacaagtatacttctctggtggttgtttaaatggtacagaatat
tctacaccttcccttgtaaaagcttatgcaagaaaagctcagtttggtcagatatttaca
tttgataaaaagacatccagattgactgatggtgtatttagaacatctgctcgaggttgg
tattcattctcacacatagctttagctttctcctttttctttggccatttgtggcatgct
agtcgtgctatcttccaagacatttggacaggtgtaacatttgaatcacaagcaaaacaa
gaatatggtagaaatgaaaagctaggagataagacatcttcaactaaatccattgtttaa
>sesoko3_psbB
atgcaaataactgctgttttaccttggtttagggtacatatagttattcttaatgatcca
ggacgtctcattagctcacatattatgcacacagctttagtagcaggttggtcagctctc
atgcttttatatgagcttatcaccatagatcctacagatcctgtgtataatccaatttgg
agacaagcagcttacacactcccatttatctcacgtattggtgttattcgctctcttttt
agttggtcacttggcatagatcctacatccaatctaatctggacttatgaaacaatgaac
atagcccatattctattatctggtttattaattcttgcatcattttggcattgggcatat
tgggacttagatctattcttcacctcaacattaacactagaccttaatcaaatcctcagt
attcacctcacactagcatcatctctctgtctgggatttggtttagctcatctaactggt
tttcttggtccaggaatgtggactagtgattccctcaatcttgtaggctctattagattc
gtaaaagcctccttcaatctccttgcttatgcacgattagcttatggtgtcatatcatca
catcacatcatctctggattgcttggcacttccataggattatggcatatcacattacgt
ccattagcatacctctacaatctattaagcatgggaaaggttgagtccattctttcaagt
agtattacagctgtattcttcactgcattccttatttcagcccttatgtggtatggctct
gcacacaccacacaagaactcttcggtcctactagatactcatgggacaatgcttattac
tctcttgatatcagaacctcttgctcaaatgcctggaacactcttccagacaagttagtc
ctttatgattatattggatctaaccctgctaaaggtggcttatttcgttcaggaccaatg
cttaaagctgatggtcttgttcaaaactggttgggccatgcatgcttctccatgggtaca
ttatccttaagcatcagaaggatgcctgctttctttgaaaccttcccagtaatcctcatt
gaccaaacaagtacagtgagagcagacattgctttcagacggtccacatctacttatagc
atggaagaatcccaaatacaagtatacttctctggtggttgtttaaatggtacagaatat
tctacaccttcccttgtaaaagcttatgcaagaaaagctcagtttggtcagatatttaca
tttgataaaaagacatccagattgactgatggtgtatttagaacatctgctcgaggttgg
tattcattctcacacatagctttagctttctcctttttctttggccatttgtggcatgct
agtcgtgctatcttccaagacatttggacaggtgtaacatttgaatcacaagcaaaacaa
gaatatggtagaaatgaaaagctaggagataagacatcttcaactaaatccattgtttaa
>sesoko4_psbB
atgcaaataactgctgttttaccttggtttagggtacatatagttattcttaatgatcca

ggacgtctcattagctcacatattatgcacacagctttagtagcaggttggtcagctctc
atgcttttatatgagcttatcaccatagatcctacagatcctgtgtataatccaatttgg
agacaagcagcttacacactcccatttatctcacgtattggtgttattcgctctcttttt
agttggtcacttggcatagatcctacatccaatctaatctggacttatgaaacaatgaac
atagcccatattctattatctggtttattaattcttgcatcattttggcattgggcatat
tgggacttagatctattcttcacctcaacattaacactagaccttaatcaaatcctcagt
attcacctcacactagcatcatctctctgtctgggatttggtttagctcatctaactggt
tttcttggtccaggaatgtggactagtgattccctcaatcttgtaggctctattagattc
gtaaaagcctccttcaatctccttgcttatgcacgattagcttatggtgtcatatcatca
catcacatcatctctggattgcttggcacttccataggattatggcatatcacattacgt
ccattagcatacctctacaatctattaagcatgggaaaggttgagtccattctttcaagt
agtattacagctgtattcttcactgcattccttatttcagcccttatgtggtatggctct
gcacacaccacacaagaactcttcggtcctactagatactcatgggacaatgcttattac
tctcttgatatcagaacctcttgctcaaatgcctggaacactcttccagacaagttagtc
ctttatgattatattggatctaaccctgctaaaggtggcttatttcgttcaggaccaatg
cttaaagctgatggtcttgttcaaaactggttgggccatgcatgcttctccatgggtaca
ttatccttaagcatcagaaggatgcctgctttctttgaaaccttcccagtaatcctcatt
gaccaaacaagtacagtgagagcagacattgctttcagacggtccacatctacttatagc
atggaagaatcccaaatacaagtatacttctctggtggttgtttaaatggtacagaatat
tctacaccttcccttgtaaaagcttatgcaagaaaagctcagtttggtcagatatttaca
tttgataaaaagacatccagattgactgatggtgtatttagaacatctgctcgaggttgg
tattcattctcacacatagctttagctttctcctttttctttggccatttgtggcatgct
agtcgtgctatcttccaagacatttggacaggtgtaacatttgaatcacaagcaaaacaa
gaatatggtagaaatgaaaagctaggagataagacatcttcaactaaatccattgtttaa
>REF_DNA_psbB_JX094324.1
ATGCAAATAACTGATGCTTTACCTTGGTTTAGGGTACATATAGTTATTCTTAATGATCCA
GGACGTCTCATTAGCTCTCACATTATGCATACAGCTTTAGTAGCTGGGTGGTCAGCTCTC
ATGCTTTTATATGAGCTTATCACCATAGATCCTACAGATCCTGTGTATAATCCAATTTGG
AGACAAGCAGCTTACACACTCCCATTTATCTCTCGTATCGGTGTTATTCGCTCTCTTTTC
AGTTGGTCACTTGGCATAGATCCTACATCCAATCTAATCTGGACTTATGAAACAATGAAC
ATAGCCCATATTCTATTATCTGGTTTATTAATTCTTGCATCATTTTGGCATTGGGCATAT
TGGGACTTAGATCTATTCTTCACCTCAACATTAACACTAGACCTTAATCAAATCCTCAGT
ATTCACCTCACACTAGCATCATCTCTCTGTCTGGGATTTGGTTTAGCTCATCTAACAGGT
TTTCTTGGTCCAGGAATGTGGACTAGTGATTCCCTCAATCTTGTAGGCTCTATTAGATTC
GTAAAAGCCTCCTTCAATATCCTTTCTTATGCACGATTAGCTTATGGTGTCATATCATCA
CATCACATCATCTCTGGATTGCTTGGCACTTCCATAGGATTATGGCATATCACATTACGT
CCATTAGCATACCTCTACAATCTATTAAGCATGGGAAAGGTTGAGTCCATTCTTTCAAGT
AGTATTACAGCTGTATTCTTCACTGCATTCCTTATTTCAGCCCTGATGTGGTATGGTTCT
GCACATACCACACAAGAACTATTCGGTCCTACTAGATACTCATGGGACAATGCTTATTAC
TCTCTTGATATCAGAACCTCTTGCTCAAATGCCTGGAACACTCTTCCAGACAAGTTAGTA
CTTTATGATTATATTGGCTCTAACCCTGCTAAAGGTGGCTTATTTCGTTCAGGACCAATG
CTTAAAGCTGATGGTCTTGTTCAAAACTGGTTAGGTCATGCATGTTTCTCCATGGGTACA
TTATCCTTAAGCATCAGAAGGATGCCTGCTTTCTTTGAAACCTTCCCAGTAATCCTCATT
GACCAAACAAGTACAGTGAGAGCAGACATTGCTTTTAGACGGTCCACATCTACTTATAGC
ATGGAAGAATCCCAAATACAAGTATACTTCTCTGGTGGTTGTTTAAATGGTACAGAATAT
TCTACACCTTCCCTTGTAAAAGCTTATGCAAGAAAAGCTCAGTTTGGTCAGATATTTACA
TTTGATAAAAAGACATCCAGATTGACTGATGGTGTATTTAGAACATCTTCTCGAGGTTGG
TATTCATTCTCACACATAGGTTTAGCTTTCCTATTCTTCTTTGGCCATTTGTGGCATGCT
AGTCGTGCTATCTTCAAAGACATTTGGACAGGTGTAACATTTGAATCTCAAGCAAAACAA
GAATATGGTAGAAATGAAAAGCTAGGAGATAAGACATCTTCAACTAAATCCATTGTTTAA
>REF_RNA_psbB_symbB1.comp28_c0_seq1
atgcaaataactgatgctttaccttggtttagggtacatatagttgttcttaatgatcca
ggacgtctcattagctctcacattatgcatacagctttagtagctgggtggtcagctctc
atgcttttatatgagcttatcaccatagatcctacagatcctgtgtataatccaatttgg
agacaagcagcttacacactcccatttgcctctcgtatcggtgttgttcgctctcttttc
gattggtcacttggcatagatcctacatccaatctaatctggacttatgaaacagtgagc
atagcccatattctattatctggtttattaattcttgcatcattttggcattgggcatat

tgggacttagatctattcttcgcctcaacattaacactagaccttaatcgaatcttcggt
attcacctcacactagcatcatctctctgtctgggatttggtttagctcatctaacaggt
ttttttggtccaggaatgtggactagtgattccctcaatcttgtaggctctattagattc
gtaaaagcctccttcaatatcctttcttatgcacgattagcttatggtgtcataccatca
catcacatcatctctggattgcttggcattttcgtaggattatggcatatcacatcacgt
ccattagcatacctctacaatctattaagcatgggaaagattgagtccgttctttcaagt
agtattgcagctgtattcttcgctgcattccttacttcagccctgatgtggtatggttct
gcacataccacacaagaactattcggtcctactagatactcatgggacaatgcttattac
tctcttgatatcagaacctctcgctcaaatgcctggaacactcttccagacaagttagta
ctttatgattatattggctctaaccctgctaaaggtggcttatttcgttcaggaccaatg
cttaaagctgatggtcttgttcaaaactggttaggtcatgcatgtttctccatgggtaca
ttatccttaagcatcagaaggatgcctgctttctttgaaaccttcccagtaatcctcatt
gaccaaacaggtacagtgagagcagacattgcttttagacggtccacatctacttatagc
gtggaagaatcccaagtacaagtatacttctctggtggttgtttaaatggtacagaatat
tctacaccttcccttgtaaaagcttatgcaagaaaagctcagtttggtcagatatttaca
tttgataaaaagacatccagattgactgatggtgtatttagaacatcttctcgaggttgg
tattcattctcacacataagtttagctttcctattcttctttggccatttgtggcatgct
ggtcgtgctatcttcagagacatttggacaggtgtaacatttgaatctcaagcaaaacaa
gaatatggtagaaatgaaaagctaggagataagacatcttcaactaaatccattgtttaa

>Hd1_psbC
atgcctctcatttattctttaaaacgacgaactctagttggatccaggtatgcttggtgg
tcaggtaatgcaagattcattgagcttagcggtaaattcctaggtgctcatcttgtgcat
acagctcttattctagtttgggctggaacaatgtctttgtttgaattatctcacttcatc
ccagaaaaacctctatatgagcaaggctttattcttcttcctcacttgtttgaattgggt
ggcactttatattcacacttcataataagcatcctacatctcatctctgcaggtatccta
gctcttggaggtatttatcatgctatagttggtgctgaaagattagaagaaacaagcttc
agtgctctatttgcttatgggttacaagatagattccgtattactgctatacttggatca
catttacttactctaggaattggagcagccttactctttgcaaaagcagtctatttaggt
ggtctttatgatacatgggcttgtggtggtggagacatgagattgattgaaagggtagag
cttggtttaaatccttatctactagctcagtacttacttcgtgctccatttgggtcatca
ggatggataattagcatcaataacatggaagatctcattggaggtcattattgggtctca
ttttatctaattctaggcagctgttggcatattcaaacaagagctcttacaattacaaca
agagcttttacatggtcagcagaagcttacctctcttatactttatcagctgtaacttta
tgtggttctatcacagcattattttcttggttcaataatactgcatatccaagtgaattc
tatggacctactggggcagaagcctctcaagcacaaagctttacattcctcatacgagat
tggaaattgggtataaagataacttcatccgaaggtcctacagctcttggtaagtatctc
atgagatctccaacaggtgaaataatatttggtggtgaaagtatgagattctggtctatg
caagcacattggcttgaagctctcagaacatcatttggactggacttatctaagattcaa
tctgatattcaaacttggcaagaaagaagagctgcagagtatatgacccatgcaccacta
gggagcttaaattctgttggtggtgttgctactgaaattaactctgtaaactatgtatct
cctagatcttggttaacatcatctcattggttcttagcattctttattttagttggtcat
tggtggcatggagcaagagcaagagcttcagcattgtctagtgaaacaggcttatctcga
gtatatgaacctgttctttatatgcgacctattgattag
>Hd2_psbC
atgcctctcatttattctttaaaacgacgaactctagttggatccaggtatgcttggtgg
tcaggtaatgcaagattcattgagcttagcggtaaattcctaggtgctcatcttgtgcat
acagctcttattctagtttgggctggaacaatgtctttgtttgaattatctcacttcatc
ccagaaaaacctctatatgagcaaggctttattcttcttcctcacttgtttgaattgggt
ggcactttatattcacacttcataataagcatcctacatctcatctctgcaggtatccta
gctcttggaggtatttatcatgctatagttggtgctgaaagattagaagaaacaagcttc
agtgctctatttgcttatgggttacaagatagattccgtattactgctatacttggatca
catttacttactctaggaattggagcagccttactctttgcaaaagcagtctatttaggt
ggtctttatgatacatgggcttgtggtggtggagacatgagattgattgaaagggtagag
cttggtttaaatccttatctactagctcagtacttacttcgtgctccatttgggtcatca
ggatggataattagcatcaataacatggaagatctcattggaggtcattattgggtctca
ttttatctaattctaggcagctgttggcatattcaaacaagagctcttacaattacaaca
agagcttttacatggtcagcagaagcttacctctcttatactttatcagctgtaacttta
tgtggttctatcacagcattattttcttggttcaataatactgcatatccaagtgaattc
tatggacctactggggcagaagcctctcaagcacaaagctttacattcctcatacgagat
tggaaattgggtataaagataacttcatccgaaggtcctacagctcttggtaagtatctc
atgagatctccaacaggtgaaataatatttggtggtgaaagtatgagattctggtctatg
caagcacattggcttgaagctctcagaacatcatttggattagacttatctaagattcaa
tctgatattcaaacttggcaagaaagaagagctgcagagtatatgacccatgcaccacta
gggagcttaaattctgttggtggtgttgctactgaaattaactctgtaaactatgtatct
cctagatcttggttaacatcatctcactggttcttagcattctttattttagtgggtcat
tggtggcatggagcaagatcaagagcttcagcattatctagtgaaacaggcttatctcga
gtatatgaacctgttctttatatgcgacctattgattag
>Hd3_psbC
atgcctctcatttattctttaaaacgacgaactctagttggatccaggtatgcttggtgg
tcaggtaatgcaagattcattgagcttagcggtaaattcctaggtgctcatcttgttcat
acagctcttattctagtttgggctggaacaatgtctttgtttgaattatctcacttcatc
ccagaaaaacctctatatgagcaaggctttattcttcttcctcacttgtttgaactgggt
ggcactttatattcacacttcataataagcatcctacatctcatctctgcaggtatctta
gctcttggaggtatttatcatgctatagttggtggtgaaagattagaagaaacaagcttc
agtgctctatttgcttatgggttacaagatagattccgtattactgctatacttggatca
catttacttactctaggaattggagcagccttactctttgcaaaagcagtctatttaggt

ggtctttatgatacttgggcttgtggtggtggagacatgagattgattgaaagagtagag
cttggtttaaatccttatctactagctcagtacttacttcgtgctccatttgggtcatca
ggatggataattagcatcaataacatggaagatctcattggaggccattattgggtctca
ttttatctaattctaggtagctgttggcatattcaaacaagagctcttacaatcacaaca
agagcttttacatggtcagcagaagcttacctctcttacactctatcagctgtaacttta
tgtggttctatcacaagtttgttttcttggtacaataatactgcatatccaagtgaattc
tatggacctactggagcagaagcctctcaagcacaaagctttacattcctcatacgagat
tggaaattgggtataaagataacttcatccgaaggtcctacagctcttggtaagtatctc
atgagatctccaacaggtgaaataatatttggtggtgaaagtatgagattctggtctatg
caagcacattggcttgaagctctcagaacatcatttggactggacttatctaagattcaa
tctgatattcaaacttggcaagaaagaagagctgcagagtatatgacccatgcaccacta
gggagcttaaattctgttggtggtgttgctactgaaattaactctgtaaactatgtatct
cctagatcttggttaacatcatctcactggttcttagcattctttattttagttggtcat
tggtggcatggagcaagagcaagagcttcagcattatctagtgaaacaggcttatctcga
gtatatgaacctgtgctttatatgcgacctattgattag
>Hd4_psbC
atgcctctcatttattctttaaaacgacgaactctagttggatccaggtatgcttggtgg
tcaggtaatgcaagattcattgagcttagcggtaaattcctaggtgctcatcttgtgcat
acagctcttattctagtttgggctggaacaatgtctttgtttgaattatctcacttcatc
ccagaaaaacctctatatgagcaaggctttattcttcttcctcacttgtttgaattgggt
ggcactttatattcacacttcataataagcatcctacatctcatctctgcaggtatccta
gctcttggaggtatttatcatgctatagttggtgctgaaagattagaagaaacaagcttc
agtgctctatttgcttatgggttacaagatagattccgtattactgctatacttggatca
catttacttactctaggaattggagcaggcttactctttgcaaaagcagtctatttaggt
ggtctttatgatacatgggcttgtggtggtggagacatgagattgattgaaagggtagag
cttggtttaaatccttatctactagctcagtacttacttcgtgctccatttgggtcatca
ggatggataattagcatcaataacatggaagatctcattggaggtcattattgggtctca
ttttatctaattctaggcagctgttggcatattcaaacaagagctcttacaattacaaca
agagcttttacatggtcagcagaagcttacctctcttatactttatcagctgtaacttta
tgtggttctatcacagcattattttcttggttcaataatactgcatatccaagtgaattc
tatggacctactggggcagaagcctctcaagcacaaagctttacattcctcatacgagat
tggaaattgggtataaagataacttcatccgaaggtcctacagctcttggtaagtatctc
atgagatctccaacaggtgaaataatatttggtggtgaaagtatgagattctggtctatg
caagcacattggcttgaagctctcagaacatcatttggactggacttatctaagattcaa
tctgatattcaaacttggcaagaaagaagagctgcagagtatatgacccatgcaccacta
gggagcttaaattctgttggtggtgttgctactgaaattaactctgtaaactatgtatct
cctagatcttggttaacatcatctcactggttcttagcattctttattttagttggtcat
tggtggcatggagcaagagcaagagcttcagcattatctagtgaaacaggcttatctcga
gtatatgaacctgttctttatatgcgacctattgattag
>Hd5_psbC
atgcctctcatttattctttaaaacgacgaactctagttggatccaggtatgcttggtgg
tcaggtaatgcaagattcattgagcttagtggtaaattcctaggtgctcatcttgtgcat
acagctcttattctagtttgggctggaacaatgtctttgtttgaattatctcacttcatc
ccagaaaaacctctatatgagcaaggctttattcttcttcctcacttgtttgaattgggt
ggcactttatattcacacttcataataagcatcctacatctcatctctgcaggtatccta
gctcttggaggtatttatcatgctatagttggtgctgaaagattagaagaaacaagcttc
agtgctctatttgcttatgggttacaagatagattccgtattactgctatacttggatca
catttacttactctaggaattggagcaggcttactctttgcaaaagcagtctatttaggt
ggtctttatgatacatgggcttgtggtggtggagacatgagattgattgaaagggtagag
cttggtttaaatccttatctactagctcagtacttacttcgtgctccatttgggtcatca
ggatggataattagcatcaataacatggaagatctcattggaggtcattattgggtctca
ttttatctaattctaggcagctgttggcatattcaaacaagagctcttacaattacaaca
agagcttttacatggtcagcagaagcttacctctcttatactttatcagctgtaacttta
tgtggttctatcacagcattattttcttggttcaataatactgcatatccaagtgaattc
tatggacctactggggcagaagcctctcaagcacaaagctttacattcctcatacgagat
tggaaattgggtataaagataagttcatccgaaggtcctacagctcttggtaagtatctc
atgagatctccaacaggtgaaataatatttggtggtgaaagtatgagattctggtctatg

caagcacattggcttgaagctctcagaacatcatttggactggacttatctaagattcaa
tctgatattcaaacttggcaagaaagaagagctgcagagtatatgacccatgcaccacta
gggagcttaaattctgttggtggtgttgctactgaaattaactctgtaaactatgtatct
cctagatcttggttaacatcatctcactggttcttagcattctttattttagtgggtcat
tggtggcatggagcaagagcaagagcttcagcattatctagtgaaacaggtttatctcga
gtatatgaacctgttctttatatgcgacctattgattag
>Hd6_psbC
atgcctctcatttattctttaaaacgacgaactctagttggatccaggtatgcttggtgg
tcaggtaatgcaagattcattgagcttagcggtaaattcctaggtgctcatcttgtgcat
acagctcttattctagtttgggcaggaagaatgtctttgtttgaattatctcacttcatt
ccagaaaaacctctatatgagcaaggctttattcttctacctcacttgtttgaattgggt
ggcactttatattcacacttcataataagcatcctacatctcatctctgcaggtatccta
gctcttggaggtatttatcatgctatagttggtgctgaaagattagaagaaacaagcttc
agtgctctatttgcttatgggttacaagatagattccgtattactgctatacttggttca
catttacttactctaggaattggagcaggcttactctttgcaaaagcagtctatttaggt
ggtctttatgatacatgggcttgtggtggtggagacatgagattgattgaaagagtagag
cttggtttaaatccttatctactagctcaatacttacttcgtgctccatttggttcatca
ggatggataattagcatcaataacatggaagatctcattggaggtcattattgggtctca
ttttatctaattctaggcagctgttggcatattcaaacaagagctcttacaatcacaaca
agagcttttacatggtcagcagaagcttacctctcttacactctatcagctgtaacttta
tgtggttctatcacaagtttgttttcttggttcaataatactgcatatccaagtgaattc
tatggacctactggggcagaagcctctcaagcacaaagctttacattcctcatacgagat
tggaaattgggtataaagataacttcatccgaaggtcctacagctcttggtaagtatctc
atgagatctccaacaggtgaaataatatttggtggtgaaagtatgagattctggtctatg
caagcacattggcttgaagctctcagaacatcatttggattagacttatctaagattcaa
tctgatattcaaacttggcaagaaagaagagctgcagagtacatgacccatgcaccacta
gggagcttaaattctgttggtggtgttgctactgaaattaactctgtaaactatgtatct
cctagatcttggttaacctcctctcattggttcttggccttctttattttagtgggtcat
tggtggcatggagcaagatcaagagctgcagcattgtctagtgaaacaggcttatctcga
gtatatgaacctgttctttatatgcgacctattgattag
>Hd7_psbC
atgcctctcatttattctttaaaacgacgaactctagttggatccaagtatgcttggtgg
tcaggtaatgcaagattcattgagcttagcggtaaattcctaggtgctcatcttgtgcat
acagctcttattctagtttgggcaggaagaatgtctttgtttgaattatctcacttcatt
ccagaaaaacctctatatgagcaaggctttattcttcttcctcacttgtttgaactggga
ggcaatctatattcacacttcataataagcatcctacatctcatctctgcaggtatctta
gctcttggaggtatttatcatgctatagttggtgctgaaagattagaagaaacaagcttc
agtgctctatttgcttatgggttacaagatagattccgtattacagctatacttggttca
catttacttactctaggaattggagcaggcttactctttgcaaaagcagtctatttaggt
ggtctttatgatacatgggcttgtggtggtggagacatgagattgattgaaagagtagag
cttggtttaaatccttatctactagctcaatacttacttcgtgctccatttgggtcatca
ggatggataattagcatcaataacatggaagatctcattggaggtcattattgggtctca
ttttatctaattctaggcagctgttggcatattcaaacaagagctcttacaattacaaca
agagcttttacatggtcagcagaagcttacctctcttatactttatcagctgtaacttta
tgtggttctatcacaagtttgttttcttggtacaataatactgcatatccaagtgaattc
tatggacctactggagcagaagcctctcaagcacaaagctttacattccttatacgagat
tggaaattgggtataaagataacttcatccgaaggtcctacagctcttggtaagtatctc
atgagatctccaacaggtgaaataatatttggtggtgaaagtatgagattctggtctatg
caagcacattggcttgaagctctcagaacatcctttggattagacttatctaagattcaa
tctgatattcaaacttggcaagaaagaagagctgcagagtatatgacccatgcaccacta
gggagcttaaattctgttggtggtgttgctactgaaattaactctgtaaactatgtatct
cctagatcttggttaacatcctctcattggttcttggccttctttattttagtgggtcat
tggtggcatggagcaagatcaagagctgcagcattgtctagtgaaacaggtttatctcga
gtatatgaacctgtgctttatatgcgacctattgattag
>Hd8_psbC
atgcctctcattcattctttaaaacgacgaactctagttggatccaggtatgcttggtgg
tcaggtaatgcaagattcattgagcttagcggtaaattcctaggtgctcatcttgtgcat

acagctcttattctagtttgggctggaacaatgtctttgtttgaattatctcacttcatc
ccagaaaaacctctatatgagcaaggctttattcttcttcctcacttgtttgaattgggt
ggcactttatattcacacttcataataagcatcctacatctcatctctgcaggtatccta
gctcttggaggtatttatcatgctatagttggtgctgaaagattagaagaaacaagcttc
agtgctctatttgcttatgggttacaagatagattccgtattactgctatacttggatca
catttacttactctaggaattggagcagccttactctttgcaaaagcagtctatttaggt
ggtctttatgatacttgggcttgtggtggtggagacatgagattgattgaaagggtagag
cttggtttaaatccttatctactagctcaatacttacttcgtgctccatttggttcatca
ggatggataattagcatcaataacatggaagatctcattggaggtcattattgggtctca
ttttatctaattctaggcagctgttggcatattcaaacaagagctcttacaattacaaca
agagcttttacatggtcagcagaagcttacctctcttatactttatcagctgtaacttta
tgtggttctatcacaggattattttcttggttcaataatactgcatatccaagtgaattc
tatggacctactggggcagaagcctctcaagcacaaagctttacattcctcatacgagat
tggaaattgggtataaagataacttcatccgaaggtcctacagctcttggtaagtatctc
atgagatctccaacaggtgaaataatatttggtggtgaaagtatgagattttgggctatg
caagcacattggcttgaagctttaagaacatcatttggactggacttatctaagattcaa
tctgatattcaaacttggcaagaaagaagagctgcagagtatatgacccatgcaccacta
gggagcttaaattctgttggtggtgttgctactgaaattaactctgtaaactatgtatct
cctagatcttggttaacatcatctcactggttcttggccttctttattttagtgggtcat
tggtggcatggagcaagatcaagagcttcagcattatctagtgaaacaggcttatctcga
gtatatgaacctgttctttatatgcgacctattgattag
>Hd9_psbC
atgcctctcatttattctttaaaacgacgaactctagttggatccaagtatgcttggtgg
tcaggtaatgcaagattcattgagcttagcggtaaattcctaggtgctcatcttgtgcat
acagctcttattctagtttgggcaggaagaatgtctttgtttgaattatctcacttcatt
ccagaaaaacctctatatgagcaaggctttattcttcttcctcacttgtttgaattgggt
ggcactttatattcacacttcataataagcatcctacatctcatctctgcaggtatccta
gctcttggaggtatttatcatgctatagttggtgctgaaagattagaagaaacaagcttc
agtgctctatttgcttatgggttacaagatagattccgtattactgctatacttggatca
catttacttactctaggaattggagcagccttactctttgcaaaagcagtctatttaggt
ggtctttatgatacatgggcttgtggtggtggagacatgagattgattgaaagggtagag
cttggtttaaatccttatctactagctcagtacttacttcgtgctccatttggttcatca
ggatggataattagcatcaataacatggaagatctcattggaggccattattgggtctca
ttttatctaattctaggcagctgttggcatattcaaacaagagctcttacaattacaaca
agagcttttacatggtcagcagaagcttacctctcttatactctatcagctgtaacttta
tgtggttctatcacaagtttgttttcttggtacaataatactgcatatccaagtgaattc
tatggacctactggggcagaagcctctcaagcacaaagctttacattccttatacgagat
tggaaattgggtataaagataagttcatccgaaggtcctacagctcttggtaagtatctc
atgagatctccaacaggtgaaataatatttggtggtgaaagtatgagattctggtctatg
caagcacattggcttgaagctctcagaacatcctttggattagacttatctaagattcaa
tctgatattcaaacttggcaagaaagaagagctgcagagtatatgacccatgcaccacta
gggagcttaaattctgttggtggtgttgctactgaaattaactctgtaaactatgtatct
cctagatcttggttaacatcatctcactggttcttagcattctttattttagttggtcat
tggtggcatggagcaagagcaagagcttcagcattatctagtgaaacaggcttatctcga
gtatatgaacctgttctttatatgcgacctattgattag
>IS1_psbC
atgcctctcatttattctttaaaacgacgaactctagttggatccaagtatgcttggtgg
tcaggtaatgcaagattcattgagctaagtggtaaattcctaggtgctcatcttgttcat
acagctcttattctagtttgggcaggaagaatgtctttgtttgaattatctcacttcatt
ccagaaaaacctctatatgagcaaggttttattcttcttcctcatttgtttgaactagga
ggcaatctatactcacacttcataataagtattctacatcttatctctgctggtattctt
gcacttggaggtatttatcatgctatagttggtggtgaaagactagaagaaacaagctat
agctccctatttgcttatgggttacaagatagattccgtattacagctatacttggttca
catttacttactctaggaattggagcaggcttactctttgcaaaagcagtctatttaggt
ggtctttatgatacttgggcttgtggtggtggagacatgagattgattgaaagggtggag
cttggattaaatccttatctactagctcagtatttacttcgtgctccatttggttcatca
ggatggataattagtatcaataacatggaagatcttattggaggtcattattgggtcgca

ttttatctaattctaggtagctgttggcatattcaaacaagacctcttacaattataaca
agagcttttacatggtcagctgaagcttacctctcttacactctatcagctgtaacttta
tgtggttctatcacaagtttgttttcttggtacaataatactgcatatccaagtgaattc
tatggacctacagcagcagaagcctctcaagcacaaagctttacatttcttatacgagat
tggaaattgggtataaagataagttcatccgaaggtcctacagctcttggtaagtatctc
atgagatctccaacaggtgaaataatatttggtggtgaaagtatgagattctggtctatg
caagcacattggcttgaagctctcagaacatcctttggattagacttatctaagattcaa
tctgatattcaaacttggcaagaaagaagagcagctgagtacatgacacatgctcctcta
ggaagcttaaattctgtcggtggtgttgctactgaaattaactctgtaaactatgtatct
cctagatcttggttaacctcctctcattggttcttggccttctttattttagtgggtcat
tggtggcatggagcaagatcaagagctgcagcattgtctagtgaaacaggtttatctcga
gtatatgaacctgtgctttatatgcgacctattgattag
>IS2_psbC
atgcctctcatttattctttaaaacgacgaactctagttggatccaggtatgcttggtgg
tcaggtaatgcaagattcattgagcttagcggtaaattcctaggtgctcatcttgtgcat
acagctcttattctagtttgggctggaacaatgtctttgtttgaattatctcacttcatc
ccagaaaaacctctatatgagcaaggctttattcttcttcctcacttgtttgaattgggt
ggcactttatattcacacttcataataagcatcctacatctcatctctgcaggtatccta
gctcttggaggtatttatcatgctatagttggtgctgaaagattagaagaaacaagcttc
agttctctatttgcttatgggttacaagatagattccgtattactgctatacttggatca
catttacttactctaggaattggagcagccttactctttgcaaaagcagtctatttaggt
ggtctttatgatacatgggcttgtggtggtggagacatgagattgattgaaagggtagag
cttggtttaaatccttatctactagctcagtacttacttcgtgctccatttgggtcatca
ggatggataattagcatcaataacatggaagatctcattggaggtcattattgggtctca
ttttatctaattctaggcagctgttggcatattcaaacaagagctcttacaattacaaca
agagcttttacatggtcagcagaagcttacctctcttatactttatcagctgtaacttta
tgtggttctatcacagcattattttcttggttcaataatactgcatatccaagtgaattc
tatggacctactggggcagaagcctctcaagcacaaagctttacattcctcatacgagat
tggaaattgggtataaagataacttcatccgaaggtcctacagctcttggtaagtatctc
atgagatctccaacaggtgaaataatatttggtggtgaaagtatgagattctggtctatg
caagcacattggcttgaagctctcagaacatcatttggactggacttatctaagattcaa
tctgatattcaaacttggcaagaaagaagagctgcagagtatatgacccatgcaccacta
gggagcttaaattctgttggtggtgttgctactgaaattaactctgtaaactatgtatct
cctagatcttggttaacatcatcccactggtttttagcattctttattttagttggtcat
tggtggcatggagcaagagcaagagcttcagcattatctagtgaaacaggcttatctcga
gtatatgaacctgttctttatatgcgacctattgattag
>IS3_psbC
atgcctctcatttattctttaaaacgacgaactctagttggatccaggtatgcctggtgg
tctggtaatgcaagatttattgagcttagtgggaagttcctaggtgctcatcttgtacat
gcagctcttattctagtttgggcaggaagaatgtctttgtttgaattatctcacttcatt
ccagaaaaacctctatatgagcaaggttttattcttcttcctcatctttttgaattagga
ggtaatctatactcacacttcataataagtattctacatcttatctctgctggtattctt
gctcttggaggtatttatcatgctatagttggtgctgaaagactagaagaaacaagattc
agtgctatatttgcttatggcttacaagatagattccgtattacagctatactaggttca
catttacttactctaggaattggagcagccttactctttgctaaagcagtctatctaggt
ggtctttatgatacatgggcttgtggtggtggagatatgagattgattgaaagagtagag
cttggtttaaatccttatctactagctcagtatttacttcgtgctccgtttggctcatca
gggtggataattagtatcaataacatggaagatcttattggaggtcattattgggtcgca
ttttatctaattctaggtagctgttggcatattcaaacaagacctcttacaattataaca
agagcttttacatggtcagctgaagcttacctctcttacactttatcagccgtaacttta
tgtgcttctaccacaagcttgttttcctggtataataatactgcttatccaagtgaattc
tatggacctacagcagcagaagcctctcaagcacaaagctttacatttctcatacgagat
tggaagttaggtataaagataacttcatccgaaggtcctacagctcttggtaagtatctc
atgagatctccaacaggtgaaataatatttggtggtgaaagtatgagattttgggctatg
caagcacattggcttgaagctttaagaacatcttttggcttggacttatctaagattcaa
tctgatgttcaaacttggcaagaaagaagagcagctgagtacatgacacatgctcctcta
ggaagcttaaattctgtcggtggtgttgctactgaaattaactctgtaaactatgtatct

cctagatcttggttaacttccacccattggtttttggccttctttattttagttggtcat
tggtggcatggagcaagagcaagggcatcagcattatctagtgaaacaggtttatcgcgt
atatatgaacctgtgctttatatgcgacctattgattag
>IS4_psbC
atgcctctcatttattctttaaaacgacgaactctagttggatccaggtatgcctggtgg
tctggtaatgcaagatttattgagcttagtgggaagttcctaggtgctcatcttgtgcat
gcagctcttattctagtttgggctggaacaatgtctttgtttgaattatctcacttcatt
ccagaaaaacctctatatgagcaaggttttattcttcttcctcatctttttgaattagga
ggtaatctatactcacacttcataataagtattctacatcttatctctgctggtattctt
gcacttggaggtatttatcatgctatagttggtgctgaaagactagaagaaacaagatac
agtgctatatttgcttatggcttacaagatagattccgtattactgctatactaggatca
catttacttactctaggaattggagcagccttactctttgcaaaagcagtctatctaggt
ggtctttatgatacatgggcttgtggtggtggagatatgagattgattgaaagggtggag
cttggattaaatccttatctactagctcagtatttacttcgtgctccgtttggctcatca
gggtggataattagtatcaataacatggaagatcttattggaggtcattattgggtcgca
ttttatctaattctaggcagctgttggcatattcaaacaagagctcttacaattacaaca
agagcttttacatggtcagctgaagcttacctctcttacactttatcagccgtaacttta
tgtgcttctaccacagccttattttcttggttcaataatactgcatatccaagtgaattc
tatggacctacagcggcagaagcctctcaagcacaaagctttacatttctcatacgagat
tggaagttaggtataaagataacttcatccgaaggtcctacagctcttggtaagtatctc
atgagatctccaacaggtgaaataatatttggtggtgaaagtatgagattttgggctatg
caagcacattggcttgaagctttaagaacatcttttggcttggacttatctaagattcaa
tctgatgttcaaacttggcaagaaagaagagcagctgagtacatgacacatgctcctcta
ggaagcttaaattctgtcggtggtgttgctactgaaattaactctgtaaactatgtatct
cctagatcttggttaacatcatctcactggttcttagcattctttattttagttggtcat
tggtggcatggagcaagagcaagggcatcagcattatctagtgaaacaggtttatcgcga
gtatatgaacctgtgctttatatgcgacctattgattag
>IS5_psbC
atgcctctcatttattctttaaaacgacgaactctagttggatccaggtatgcttggtgg
tcaggtaatgcaagattcattgagcttagcggtaaattcctaggtgctcatcttgtgcat
acagctcttattctagtttgggcaggaagaatgtctttgtttgaattatctcacttcatt
ccagaaaaacctctatatgagcaaggctttattcttcttcctcacttgtttgaattggga
ggcactctatattcacacttcataataagcatcctacatctcatctctgcaggtatccta
gctcttggaggtatttatcatgctatagttggtgctgaaagattagaagaaacaagcttc
agtgctctatttgcttatgggttacaagatagattccgtattactgctatacttggatca
catttacttactctaggaattggagcagccttactctttgcaaaagcagtctatttaggt
ggtctttatgatacatgggcttgtggtggtggagacatgagattgattgaaagggtagag
cttggtttaaatccttatctactagctcaatacttacttcgtgctccatttggttcatca
ggatggataattagcatcaataacatggaagatctcattggaggtcattattgggtctca
ttttatctaattctaggtagctgttggcatattcaaacaagagctcttacaattacaaca
agagcttttacatggtcagcagaagcttacctctcttatactctatcagctgtaacttta
tgtggttctatcacagcattattttcttggttcaataatactgcatatccaagtgaattc
tatggacctactggggcagaagcctctcaagcacaaagctttacattcctcatacgagat
tggaaattgggtataaagataacttcatccgaaggtcctacagctcttggtaagtatctc
atgagatctccaacaggtgaaataatatttggtggtgaaagtatgagattctggtctatg
caagcacattggcttgaagctctcagaacatcctttggattagacttatctaagattcaa
tctgatattcaaacttggcaagaaagaagagctgcagagtatatgacccatgcaccacta
gggagcttaaattctgttggtggtgttgctactgaaattaactctgtaaactatgtatct
cctagatcttggttaacctcctctcattggttcttggccttctttattttagtgggtcat
tggtggcatggagcaagagcaagagcttcagcattatctagtgaaacaggcttatctcga
gtatatgaacctgtgctttatatgcgacctattgattag
>IS6_psbC
atgcctctcatttattctttaaaacgacgaactctagttggatccaggtatgcttggtgg
tcaggtaatgcaagattcattgagcttagcggtaaattcctaggtgctcatcttgtgcat
acagctcttattctagtttgggctggaacaatgtctttgtttgaattatctcacttcatc
ccagaaaaacctctatatgagcaaggctttattcttcttcctcacttgtttgaattggga
ggcactttatattcacacttcataataagcatcctacatctcatctctgcaggtatccta

gctcttggaggtatttatcatgctatagttggtgctgaaagattagaagaaacaagctat
agctccctatttgcttatgggttacaagatagattccgtattactgctatacttggatca
catttacttactctaggaattggagcaggcttactctttgcaaaagcagtctatttaggt
ggtctttatgatacttgggcttgtggtggtggagacatgagattgattgaaagggtagag
cttggtttaaatccttatctactagctcaatacttacttcgtgctccatttggttcatca
ggatggataattagcatcaataacatggaagatctcattggaggtcattattgggtctca
ttttatctaattctaggcagctgttggcatattcaaacaagagctcttacaattacaaca
agagcttttacatggtcagcagaagcttacctctcttacactctatcagctgtaacttta
tgtggttctatcacagcattattttcttggttcaataatactgcatatccaagtgaattc
tatggacctactggggcagaagcctctcaagcacaaagctttacattcctcatacgagat
tggaaattgggtataaagataacttcatccgaaggtcctacagctcttggtaagtatctc
atgagatctccaacaggtgaaataatatttggtggtgaaagtatgagattctggtctatg
caagcacattggcttgaagctctcagaacatcatttggactggacttatctaagattcaa
tctgatattcaaacttggcaagaaagaagagctgcagagtatatgacccatgcaccacta
gggagcttaaattctgttggtggtgttgctactgaaattaactctgtaaactatgtatct
cctagatcttggttaacatcatctcactggttcttagcattctttattttagttggtcat
tggtggcatggagcaagagcaagagcttcagcattatctagtgaaacaggcttatctcga
gtatatgaacctgttctttatatgcgacctattgattag
>IS7_psbC
atgcctctcatttattctttaaaacgacgaactctagttggatccaggtatgcctggtgg
tctggtaatgcaagatttattgagcttagtgggaagttcctaggtgctcatcttgtacat
gcagctcttattctagtttgggcaggaagaatgtctttgtttgaattatctcacttcatt
ccagaaaaacctctatatgagcaaggctttattcttcttcctcaccttttgaattgggt
ggcaatctatattcacacttcataataagcatcctacatctcatctctgcaggtatccta
gctcttggaggtatttatcatgctatagttggtgctgaaagactagaagaaacaagattc
agtgctatatttgcttatggcttacaagatagattccgtattactgctatactaggatca
catttacttactctaggaattggagcagccttactctttgctaaagcagtctatctaggt
ggtctttatgatacatgggcttgtggtggtggagacatgagattgattgaaagggtagag
cttggtttaaatccttatctactagctcagtatttacttcgtgctccgtttggctcatca
gggtggataattagcatcaataacatggaagatctcattggaggtcattattgggtctca
ttttatctaattctaggcagctgttggcatattcaaacaagacctcttacaattataaca
agagcttttacatggtcagctgaagcttacctctcttatactttatcagccgtaacttta
tgtggttctatcacaagtttgttttcctggtataataatactgcatatccaagtgaattc
tatggacctactgcagcagaagcctctcaagcacaaagctttacattcctcatacgagat
tggaagttaggtataaagataacttcatccgaaggtcctacagctcttggtaagtatctc
atgagatctccaacaggtgaaataatatttggtggtgaaagtatgagattttgggctatg
caagcacattggcttgaagctctaagaacatcttttggcctggacttatctaagattcaa
tctgatgttcaaacttggcaagaaagaagagcagctgagtacatgacacatgctcctcta
ggaagcttaaattctgtcggtggtgttgctactgaaattaactctgtaaactatgtatct
cctagatcttggttaacatcatctcactggttcttagcattcttcattttagttggtcat
tggtggcatggagcaagagcaagggcatcagcattatctagtgaaacaggcttatctcga
gtatatgaacctgttctttatatgcgacctattgattag
>IS8_psbC
atgcctctcatttattctttaaaacgacgaactctagttggatccaggtatgcttggtgg
tcaggtaatgcaagattcattgagcttagcggtaaattcctaggtgctcatcttgtgcat
acagctcttattctagtttgggctggaacaatgtctttgtttgaattatctcacttcatc
ccagaaaaacctctatatgagcaaggctttattcttcttcctcacttgtttgaattgggt
ggcactttatattcacacttcataataagcatcctacatctcatctctgcaggtatccta
gctcttggaggtatttatcatgctatagttggtgctgaaagattagaagaaacaagctat
agctccctatttgcttatgggttacaagatagattccgtattactgctatacttggatca
catttacttactctaggaattggagcagccttactctttgcaaaagcagtctatttaggt
ggtctttatgatacatgggcttgtggtggtggagacatgagattgattgaaagggtagag
cttggtttaaatccttatctactagctcagtacttacttcgtgctccatttgggtcatca
ggatggataattagcatcaataacatggaagatctcattggaggtcattattgggtctca
ttttatctaattctaggcagctgttggcatattcaaacaagagctcttacaattacaaca
agagcttttacatggtcagcagaagcttacctctcttatactttatcagctgtaacttta
tgtggttctatcacaagtttgttttcttggtacaataatactgcatatccaagtgaattc

tatggacctactggggcagaagcctctcaagcacaaagctttacattcctcatacgagat
tggaaattgggtataaagataacttcatccgaaggtcctacagctcttggtaagtatctc
atgagatctccaacaggtgaaataatatttggtggtgaaagtatgagattctggtctatg
caagcacattggcttgaagctctcagaacatcatttggactggacttatctaagattcaa
tctgatattcaaacttggcaagaaagaagagctgcagagtatatgacccatgcaccacta
gggagcttaaattctgttggtggtgttgctactgaaattaactctgtaaactatgtatct
cctagatcttggttaacatcatctcactggttcttagcattctttattttagttggtcat
tggtggcatggagcaagagcaagagcttcagcattatctagtgaaacaggcttatctcga
gtatatgaacctgttctttatatgcgacctattgattag
>IS9_psbC
atgcctctcatttattctttaaaacgacgaactctagttggatccaggtatgcttggtgg
tctggtaatgcaagatttattgagcttagtgggaagttcctaggtgctcatcttgtacat
gcagctcttattctagtttgggcaggaagaatgtctttgtttgaattatctcacttcatc
ccagaaaaacctctatatgagcaaggttttattcttcttcctcacctttttgaattagga
ggcaatctatattcacacttcataataagcatcctacatctcatctctgcaggtatctta
gctcttggaggtatttatcatgctatagttggtggtgaaagactagaagaaacaagctat
agctccctatttgcttatgggttacaagatagattccgtattacagctatactaggttca
catttacttactctaggaattggagcagccttactctttgcaaaagcagtctatttaggt
ggtctttatgatacatgggcttgtggtggtggagatatgagattgattgaaagggtggag
cttggattaaatccttatctactagctcagtatttacttcgtgctccgtttggctcatca
gggtggataattagtatcaataacatggaagatcttattggaggtcattattgggtcgca
ttttatctaattctaggcagctgttggcatattcaaacaagagctcttacaatcacaaca
agagcttttacatggtcagctgaagcttacctctcttatactttatcagccgtaacttta
tgtggttctaccacaagtttgttttcctggtataataatactgcttatccaagtgaattc
tatggacctactggggcagaagcctctcaagcacaaagctttacatttctcatacgagat
tggaagttaggtataaagataacttcatccgaaggtcctacagctcttggtaagtatctc
atgagatctccaacaggtgaaataatatttggtggtgaaagtatgagattttgggctatg
caagcacattggcttgaagctttaagaacatcttttggcttggacttatctaagattcaa
tctgatgttcaaacttggcaagaaagaagagcagctgagtatatgacacatgctcctcta
ggaagcttaaattctgttggtggtgttgctactgaaattaactctgtaaactatgtatct
cctagatcttggttaacctcctctcattggttcttggccttctttattttagtgggtcat
tggtggcatggagcaagatcaagagctgcagcattgtctagtgaaacaggtttatctcga
gtatatgaacctgtgctttatatgcgacctattgattag
>Ik1_psbC
atgcctctcatttattctttaaaacgacgaactctagttggatccaggtatgcttggtgg
tcaggtaatgcaagattcattgagctaagtggtaaattcctaggtgctcatcttgttcat
acagctcttattctagtttgggcaggaagaatgtctttgtttgaattatctcacttcatt
ccagaaaaacctctatatgagcaaggctttattcttcttcctcacttgtttgaactggga
ggcaatctatattcacacttcataataagcatcctacatctcatctctgcaggtatccta
gctcttggaggtatttatcatgctatagttggtgctgaaagattagaagaaacaagctac
agtgctctatttgcttatgggttacaagatagattccgtattacagctatacttggttca
catttacttactctaggaattggagcagccttactctttgcaaaagcagtctatttaggt
ggtctttatgatacatgggcttgtggtggttgagacatgagattgattgaaagggtagag
cttggtttaaatccttatctactagctcaatacttacttcgtgctccatttggttcatca
ggatggataattagcatcaataacatggaagatctaattggaggccattattgggtctca
ttttatctaattctaggcagctgttggcatattcaaacaagagctcttacaattacaaca
agagcttttacatggtcagcagaagcttacctctcttatactttatcagctgtaacttta
tgtggttctatcacagcattattttcttggttcaataatactgcatatccaagtgaattc
tatggacctactggggcagaagcctctcaagcacaaagctttacattccttatacgagat
tggaaattgggtataaagataacttcatccgaaggtcctacagctcttggtaagtatctc
atgagatctccaacaggtgaaataatatttggtggtgaaagtatgagattctggtctatg
caagcacattggcttgaagctctcagaacatcatttggattagacttatctaagattcaa
tctgatattcaaacttggcaagaaagaagagctgcagagtacatgacccatgcaccacta
gggagcttaaattctgttggtggtgttgctactgaaattaactatgtaaactatgtatct
cctagatcttggttaacctcatctcactggttcttagcattctttattttagttggtcat
tggtggcatggagcaagatcaagagctgcagcattgtctagtgaaacaggtttatctcga
gtatatgaacctgtgctttatatgcgacctattgattag

>Ik2_psbC
atgcctctcatttattctttaaaacgacgaactctagttggatccaggtatgcttggtgg
tcaggtaatgcaagattcattgagcttagcggtaaattcctaggtgctcatcttgtgcat
acagctcttattctagtttgggctggaacaatgtctttgtttgaattatctcacttcatc
ccagaaaaacctctatatgagcaaggctttattcttcttcctcacttgtttgaattgggt
ggcactttatattcacacttcataataagcatcctacatctcatctctgcaggtatccta
gctcttggaggtatttatcatgctatagttggtgctgaaagattagaagaaacaagcttc
agtgctctatttgcttatgggttacaagatagattccgtattactgctatacttggatca
catttacttactctaggaattggagcagccttactctttgcaaaagcagtctatttaggt
ggtctttatgatacatgggcttgtggtggtggagacatgagattgattgaaagggtagag
cttggtttaaatccttatctactagctcagtacttacttcgtgctccatttgggtcatca
ggatggataattagcatcaataacatggaagatctcattggaggtcattattgggtctca
ttttatctaattctaggcagctgttggcatattcaaacaagagctcttacaattacaaca
agagcttttacatggtcagcagaagcttacctctcttatactttatcagctgtaacttta
tgtggttctatcacaagtttgttttcttggtacaataatactgcatatccaagtgaattc
tatggacctactggagcagaagcctctcaagcacaaagctttacattcctcatacgagat
tggaaattgggtataaagataacttcatccgaaggtcctacagctcttggtaagtatctc
atgagatctccaacaggtgaaataatatttggtggtgaaagtatgagattctggtctatg
caagcacattggcttgaagctctcagaacatcatttggactggacttatctaagattcaa
tctgatattcaaacttggcaagaaagaagagctgcagagtatatgacccatgcaccacta
gggagcttaaattctgttggtggtgttgctactgaaattaactctgtaaactatgtatct
cctagatcttggttaacctcctctcattggttcttggcattctttattttagttggtcat
tggtggcatggagcaagagcaagagcttcagcattgtctagtgaaacaggcttatctcga
gtatatgaacctgttctttatatgcgacctattgattag
>Ik3_psbC
atgcctctcatttattctttaaaacgacgaactctagttggatccaggtatgcttggtgg
tcaggtaatgcaagattcattgagcttagcggtaaattcctaggtgctcatcttgtgcat
acagctcttattctagtttgggcagcaacaatgtctttgtttgaattatctcacttcatc
ccagaaaaacctctatatgagcaaggctttattcttcttcctcacttgtttgaattgggt
ggcactttatattcacacttcataataagcatcctacatctcatctctgcaggtatccta
gctcttggaggtatttatcatgctatagttggtggtgaaagactagaagaaacaagcttc
agtgctctatttgcttatgggttacaagatagattccgtattactgctatacttggatca
catttacttactctaggaattggagcagccttactctttgcaaaagcagtctatttaggt
ggtctttatgatacatgggcttgtggtggtggagacatgagattgattgaaagggtagag
cttggtttaaatccttatctactagctcaatacttacttcgtgctccatttgggtcatca
ggatggataattagcatcaataacatggaagatctcattggaggtcattattgggtctca
ttttatctaattctaggtagctgttggcatattcaaacaagagctcttacaattacaaca
agagcttttacatggtcagcagaagcttacctctcttatactttatcagctgtaacttta
tgtggttctatcacagcattattttcttggttcaataatactgcatatccaagtgaattc
tatggacctactggggcagaagcctctcaagcacaaagctttacattcctcatacgagat
tggaaattgggtataaagataacttcatccgaaggtcctacagctcttggtaagtatctc
atgagatctccaacaggtgaaataatatttggtggtgaaagtatgagattctggtctatg
caagcacattggcttgaagctctcagaacatcatttggactggacttatctaagattcaa
tctgatattcaaacttggcaagaaagaagagctgcagagtatatgacccatgcaccacta
gggagcttaaattctgttggtggtgttgctactgaaattaactctgtaaactatgtatct
cctagatcttggttaacatcctctcactggttcttggccttctttattttagtgggtcat
tggtggcatggagcaagatcaagagctgcagcattgtctagtgaaacaggcttatctcga
gtatatgaacctgttctttatatgcgacctattgattag
>Ik4_psbC
atgcctctcatttattctttaaaacgacgaactctagttggatccaggtatgcttggtgg
tcaggtaatgcaagattcattgagctaagtggtaaattcctaggtgctcatcttgtgcat
acagctcttattctagtttgggctggaacaatgtctttgtttgaattatctcacttcatc
ccagaaaaacctctatatgagcaaggctttattcttctacctcatttgtttgaactgggt
ggcactctatattcacacttcataataagcatcctacatctcatctctgcaggtatccta
gctcttggaggtatttatcatgctatagttggtgctgaaagattagaagaaacaagctat
agcgctctatttgcttatgggttacaagatagattccgtattactgctatacttggatca
catttacttactctaggaattggagcagccttactctttgcaaaagcagtctatttaggt

ggtctttatgatacatgggcttgtggtggtggagacatgagattgattgaaagagtagag
cttggtttaaatccttatctactagctcagtacttacttcgtgctccatttgggtcatca
ggatggataattagcatcaataacatggaagatctcattggaggtcattattgggtctca
ttttatctaattctaggcagctgttggcatattcaaacaagagctcttacaattacaaca
agagcttttacatggtcagcagaagcttacctctcttatactttatcagctgtaacttta
tgtggttctatcacagcattattttcttggttcaataatactgcatatccaagtgaattc
tatggacctactggggcagaagcctctcaagcacaaagctttacattcctcatacgagat
tggaaattgggtataaagataacttcatccgaaggtcctacagctcttggtaagtatctc
atgagatctccaacaggtgaaataatatttggtggtgaaagtatgagattctggtctatg
caagcacattggcttgaagctctcagaacatcatttggactggacttatctaagattcaa
tctgatattcaaacttggcaagaaagaagagctgcagagtatatgacccatgcaccacta
gggagcttaaattctgttggtggtgttgctactgaaattaactctgtaaactatgtatct
cctagatcttggttaacctcctctcactggttcttagcattctttattttagttggtcat
tggtggcatggagcaagagcaagagcttcagcattatctagtgaaacaggtttatctcga
gtatatgaacctgtgctttatatgcgacctattgattag
>Ik5_psbC
atgcctctcatttattctttaaaacgacgaactctagttggatccaagtatgcttggtgg
tcaggtaatgcaagattcattgagctaagtggtaaattcctaggtgctcatcttgtgcat
acagctcttattctagtttgggctggaacaatgtctttgtttgaattatctcacttcatt
ccagaaaaacctctatatgagcaaggctttattcttctacctcatttgtttgaactggga
ggcaatctatattcacacttcataataagcatcctacatctcatctctgcaggtatccta
gctcttggaggtatttatcatgctatagttggtgctgaaagattagaagaaacaagcttc
agtgctctatttgcttatgggttacaagatagattccgtattacagctatacttggatca
catttacttactctaggaattggagcagccttactctttgcaaaagcagtctatttaggt
ggtctttatgatacatgggcttgtggtggtggagacatgagattgattgaaagagtagag
cttggtttaaatccttatctactagctcaatacttacttcgtgctccatttgggtcatca
ggatggataattagcatcaataacatggaagatctcattggaggccattattgggtctca
ttttatctaattctaggtagctgttggcatattcaaacaagagctcttacaatcacaaca
agagcttttacatggtcagcagaagcttacctctcttatactttatcagctgtaacttta
tgtggttctatcacaagattgttttcttggtacaataatactgcatatccaagtgaattc
tatggacctactggagcagaagcctctcaagcacaaagctttacattcctcatacgagat
tggaaattgggtataaagataacttcatccgaaggtcctacagctcttggtaagtatctc
atgagatctccaacaggtgaaataatatttggtggtgaaagtatgagattctggtctatg
caagcacattggcttgaagctctcagaacatcatttggactggacttatctaagattcaa
tctgatattcaaacttggcaagaaagaagagctgcagagtatatgacccatgcaccacta
gggagcttaaattctgttggtggtgttgctactgaaattaactctgtaaactatgtatct
cctagatcttggttaacatcatctcactggttcttagcattctttattttagttggtcat
tggtggcatggagcaagagcaagagctgcagcattgtctagtgaaacaggcttatctcga
gtatatgaacctgttctttatatgcgacctattgattag
>Ik6_psbC
atgcctctcatttattctttaaaacgacgaactctagttggatccaggtatgcttggtgg
tcaggtaatgcaagattcattgagcttagcggtaaattcctaggtgctcatcttgttcat
acagctcttattctagtttgggcaggaacaatgtctttgtttgaattatctcacttcatc
ccagaaaaacctctatatgagcaaggctttattcttcttcctcacttgtttgaattgggt
ggcactttatattcacacttcataataagcatcctacatctcatctctgcaggtatctta
gctcttggaggtatttatcatgctatagttggtggtgaaagactagaagaaacaagctat
agctccctatttgcttatgggttacaagatagattccgtattacagctatacttggttca
catttacttactctaggaattggagcagccttactctttgcaaaagcagtctatttaggt
ggtctttatgatacttgggcttgtggtggtggagacatgagattgattgaaagagtagag
cttggtttaaatccttatctactagctcaatacttacttcgtgctccatttgggtcatca
ggatggataattagcatcaataacatggaagatctcattggaggtcattattgggtctca
ttttatctaattctaggtagctgttggcatattcaaacaagagctcttacaattacaaca
agagcttttacatggtcagcagaagcttacctctcttatactttatcagctgtaacttta
tgtggttctatcacagctttgttttcttggtacaataatactgcatatccaagtgaattc
tatggacctactggagcagaagcctctcaagcacaaagctttacattccttatacgagat
tggaaattgggtataaagataagttcatccgaaggtcctacagctcttggtaagtatctc
atgagatctccaacaggtgaaataatatttggtggtgaaagtatgagattctggtctatg

caagcacattggcttgaagctctcagaacatcctttggattagacttatctaagattcaa
tctgatattcaaacttggcaagaaagaagagctgcagagtacatgacccatgcaccacta
gggagcttaaattctgttggtggtgttgctactgaaattaactctgtaaactatgtatct
cctagatcttggttaacctcctctcattggttcttggccttctttattttagtgggtcat
tggtggcatggagcaagatcaagagctgcagcattgtctagtgaaacaggtttatctcga
gtatatgaacctgtgctttatatgcgacctattgattag
>Ik7_psbC
atgcctctcatttattctttaaaacgacgaactctagttggatccaggtatgcttggtgg
tcaggtaatgcaagattcattgagcttagcggtaaattcctaggtgctcatcttgtgcat
acagctcttattctagtttgggcaggaagaatgtctttgtttgaattatctcacttcatc
ccagaaaaacctctatatgagcaaggctttattcttcttcctcacttgtttgaattgggt
ggcactttatattcacacttcataataagcatcctacatctcatctctgcaggtatctta
gctcttggaggtatttatcatgctatagttggtggtgaaagactagaagaaacaagctat
agctccctatttgcttatgggttacaagatagattccgtattacagctatacttggatca
catttacttactctaggaattggagcaggcttactctttgcaaaagcagtctatttaggt
ggtctttatgatacatgggcttgtggtggtggagacatgagattgattgaaagagtagag
cttggtttaaatccttatctactagctcagtacttacttcgtgctccatttgggtcatca
ggatggataattagcatcaataacatggaagatctcattggaggtcattattgggtctca
ttttatctaattctaggtagctgttggcatattcaaacaagagctcttacaatcacaaca
agagcttttacatggtcagcagaagcttacctctcttatactttatcagctgtaacttta
tgtggttctatcacaggtttattttcttggttcaataatactgcatatccaagtgaattc
tatggacctactggggcagaagcctctcaagcacaaagctttacattccttatacgagat
tggaaattgggtataaagataacttcatccgaaggtcctacagctcttggtaagtatctc
atgagatctccaacaggtgaaataatatttggtggtgaaagtatgagattctggtctatg
caagcacattggcttgaagctctcagaacatcatttggactggacttatctaagattcaa
tctgatattcaaacttggcaagaaagaagagctgcagagtacatgacccatgcaccacta
gggagcttaaattctgttggtggtgttgctactgaaattaactctgtaaactatgtatct
cctagatcttggttaacatcatctcactggttcttagcattctttattttagttggtcat
tggtggcatggagcaagatcaagagctgcagcattatctagtgaaacaggcttatctcga
gtatatgaacctgttctttatatgcgacctattgattag
>Ik8_psbC
atgcctctcatttattctttaaaacgacgaactctagttggatccaagtatgcttggtgg
tcaggtaatgcaagattcattgagcttagcggtaaattcctaggtgctcatcttgttcat
acagctcttattctagtttgggctggaacaatgtctttgtttgaattatctcacttcatt
ccagaaaaacctctatatgagcaaggctttattcttctacctcatttgtttgaactgggt
ggcactttatattcacacttcataataagcatcctacatctcatctctgcaggtatctta
gctcttggaggtatttatcatgctatagttggtggtgaaagactagaagaaacaagctat
agctccctatttgcttatgggttacaagatagattccgtattacagctatacttggttca
catttacttactctaggaattggagcaggcttactctttgcaaaagcagtctatttaggt
ggtctttatgatacttgggcttgtggtggtggagacatgagattgattgaaagagtagag
cttggtttaaatccttatctactagctcaatacttacttcgtgctccatttggttcatca
ggatggataattagcatcaataacatggaagatctcattggaggccattattgggtctca
ttttatctaattctaggcagctgttggcatattcaaacaagagctcttacaattacaaca
agagcttttacatggtcagcagaagcttacctctcttatactctatcagctgtaacttta
tgtggttctatcacagcattattttcttggttcaataatactgcatatccaagtgaattc
tatggacctactggagcagaagcctctcaagcacaaagctttacattcctcatacgagat
tggaaattgggtataaagataacttcatccgaaggtcctacagctcttggtaagtatctc
atgagatctccaacaggtgaaataatatttggtggtgaaagtatgagattctggtctatg
caagcacattggcttgaagctctcagaacatcctttggattagacttatctaagattcaa
tctgatattcaaacttggcaagaaagaagagctgcagagtatatgacccatgcaccacta
gggagcttaaattctgttggtggtgttgctactgaaattaactctgtaaactatgtatct
cctagatcttggttaacctcctctcactggttcttggcattctttattttagtgggtcat
tggtggcatggagcaagatcaagagctgcagcattgtctagtgaaacaggtttatctcga
gtatatgaacctgtgctttatatgcgacctattgattag
>Ik9_psbC
atgcctctcatttattctttaaaacgacgaactctagttggatccaggtatgcttggtgg
tcaggtaatgcaagattcattgagcttagcggtaaattcctaggtgctcatcttgtgcat

acagctcttattctagtttgggcaggaagaatgtctttgtttgaattatctcacttcatt
ccagaaaaacctctatatgagcaaggctttattcttctacctcatttgtttgaactggga
ggcaatctatattcacacttcataataagcatcctacatctcatctctgcaggtatccta
gctcttggaggtatttatcatgctatagttggtgctgaaagattagaagaaacaagcttc
agctctctatttgcttatgggttacaagatagattccgtattacagctatacttggttca
catttacttactctaggaattggagcagccttactctttgcaaaagcagtctatttaggt
ggtctttatgatacttgggcttgtggtggtggagacatgagattgattgaaagggtagag
cttggtttaaatccttatctactagctcaatacttacttcgtgctccatttgggtcatca
ggatggataattagcatcaataacatggaagatctcattggaggtcattattgggtctca
ttttatctaattctaggcagctgttggcatattcaaacaagagctcttacaattacaaca
agagcttttacatggtcagcagaagcttacctctcttacactctatcagctgtaacttta
tgtggttctatcacagcattattttcttggttcaataatactgcatatccaagtgaattc
tatggacctactggagcagaagcctctcaagcacaaagctttacattccttatacgagat
tggaaattgggtataaagataacttcatccgaaggtcctacagctcttggtaagtatctc
atgagatctccaacaggtgaaataatatttggtggtgaaagtatgagattctggtctatg
caagcacattggcttgaagctctcagaacatcctttggattagacttatctaagattcaa
tctgatattcaaacttggcaagaaagaagagctgcagagtatatgacccatgcaccacta
gggagcttaaattctgttggtggtgttgctactgaaattaactctgtaaactatgtatct
cctagatcttggttaacatcatctcactggttcttagcattctttattttagtgggtcat
tggtggcatggagcaagagcaagagcttcagcattatctagtgaaacaggcttatctcga
gtatatgaacctgttctttatatgcgacctattgattag
>Irm10_psbC
atgcctctcatttattctttaaaacgacgaactctagttggatccaggtatgcttggtgg
tcaggtaatgcaagattcattgagcttagcggtaaattcctaggtgctcatcttgtgcat
acagctcttattctagtttgggctggaacaatgtctttgtttgaattatctcacttcatc
ccagaaaaacctctatatgagcaaggctttattcttcttcctcacttgtttgaattgggt
ggcactttatattcacacttcataataagcatcctacatctcatctctgcaggtatccta
gctcttggaggtatttatcatgctatagttggtgctgaaagattagaagaaacaagcttc
agtgctctatttgcttatgggttacaagatagattccgtattactgctatacttggatca
catttacttactctaggaattggagcagccttactctttgcaaaagcagtctatttaggt
ggtctttatgatacatgggcttgtggtggtggagacatgagattgattgaaagggtagag
cttggtttaaatccttatctactagctcagtacttacttcgtgctccatttgggtcatca
ggatggataattagcatcaataacatggaagatctcattggaggtcattattgggtctca
ttttatctaattctaggtagctgttggcatattcaaacaagagctcttacaatcacaaca
agagcttttacatggtcagcagaagcttacctctcttatactttatcagctgtaacttta
tgtggttctatcacagcattattttcttggttcaataatactgcatatccaagtgaattc
tatggacctactggggcagaagcctctcaagcacaaagctttacattcctcatacgagat
tggaaattgggtataaagataacttcatccgaaggtcctacagctcttggtaagtatctc
atgagatctccaacaggtgaaataatatttggtggtgaaagtatgagattctggtctatg
caagcacattggcttgaagctctcagaacatcctttggattagacttatctaagattcaa
tctgatattcaaacttggcaagaaagaagagctgcagagtacatgacccatgcaccacta
gggagcttaaattctgttggtggtgttgctactgaaattaactctgtaaactatgtatct
cctagatcttggttaacctcctctcattggttcttggccttctttattttagtgggtcat
tggtggcatggagcaagatcaagagcttcagcattatctagtgaaacaggcttatctcga
gtatatgaacctgttctttatatgcgacctattgattag
>Irm17_psbC
atgcctctcatttattctttaaaacgacgaactctagttggatccaggtatgcttggtgg
tcaggtaatgcaagattcattgagctaagtggtaaattcctaggtgctcatcttgttcat
acagctcttattctagtttgggcaggaagaatgtctttgtttgaattatctcacttcatc
ccagaaaaacctctatatgagcaaggctttattcttcttcctcacttgtttgaattgggt
ggcactttatattcacacttcataataagcatcctacatctcatctctgcaggtatccta
gctcttggaggtatttatcatgctatagttggtgctgaaagattagaagaaacaagctta
agtgctctatttgcttatgggttacaagatagattccgtattactgctatacttggatca
catttacttactctaggaattggagcagccttactctttgcaaaagcagtctatttaggt
ggtctttatgatacatgggcttgtggtggtggagacatgagattgattgaaagggtagag
cttggtttaaatccttatctactagctcagtacttacttcgtgctccatttgggtcatca
ggatggataattagcatcaataacatggaagatctcattggaggtcattattgggtctca

ttttatctaattctaggcagctgttggcatattcaaacaagagctcttacaattacaaca
agagcttttacatggtcagcagaagcttacctctcttatactttatcagctgtaacttta
tgtggttctatcacagcattattttcttggttcaataatactgcatatccaagtgaattc
tatggacctactggggcagaagcctctcaagcacaaagctttacattcctcatacgagat
tggaaattgggtataaagataagttcatccgaaggtcctacagctcttggtaagtatctc
atgagatctccaacaggtgaaataatatttggtggtgaaagtatgagattctggtctatg
caagcacattggcttgaagctctcagaacatcatttggactggacttatctaagattcaa
tctgatattcaaacttggcaagaaagaagagctgcagagtatatgacccatgcaccacta
gggagcttaaattctgttggtggtgttgctactgaaattaactctgtaaactatgtatct
cctagatcttggttaacatcatctcactggttcttagcattctttattttagttggtcat
tggtggcatggagcaagagcaagagcttcagcattatctagtgaaacaggcttatctcga
gtatatgaacctgtgctttatatgcgacctattgattag
>Irm2_psbC
atgcctctcatttattctttaaaacgacgaactctagttggatccaggtatgcttggtgg
tcaggtaatgcaagattcattgagcttagcggtaaattcctaggtgctcatcttgtgcat
acagctcttattctagtttgggctggaacaatgtctttgtttgaattatctcacttcatc
ccagaaaaacctctatatgagcaaggctttattcttcttcctcacttgtttgaattgggt
ggcactttatattcacacttcataataagcatcctacatctcatctctgcaggtatccta
gctcttggaggtatttatcatgctatagttggtgctgaaagattagaagaaacaagcttc
agtgctctatttgcttatgggttacaagatagattccgtattactgctatacttggatca
catttacttactctaggaattggagcagccttactctttgcaaaagcagtctatttaggt
ggtctttatgatacatgggcttgtggtggtggagacatgagattgattgaaagggtagag
cttggtttaaatccttatctactagctcagtacttacttcgtgctccatttgggtcatca
ggatggataattagcatcaataacatggaagatctcattggaggtcattattgggtctca
ttttatctaattctaggcagctgttggcatattcaaacaagagctcttacaattacaaca
agagcttttacatggtcagcagaagcttacctctcttatactttatcagctgtaacttta
tgtggttctatcacagcattattttcttggttcaataatactgcatatccaagtgaattc
tatggacctactggggcagaagcctctcaagcacaaagctttacattcctcatacgagat
tggaaattgggtataaagataacttcatccgaaggtcctacagctcttggtaagtatctc
atgagatctccaacaggtgaaataatatttggtggtgaaagtatgagattctggtctatg
caagcacattggcttgaagctctcagaacatcatttggactggacttatctaagattcaa
tctgatattcaaacttggcaagaaagaagagctgcagagtatatgacccatgcaccacta
gggagcttaaattctgttggtggtgttgctactgaaattaactctgtaaactatgtatct
cctagatcttggttaacatcatctcactggttcttagcattctttattttagttggtcat
tggtggcatggagcaagagcaagagcttcagcattatctagtgaaacaggcttatctcga
gtatatgaacctgttctttatatgcgacctattgattag
>Irm21_psbC
atgcctctcatttattctttaaaacgacgaactctagttggatccaggtatgcttggtgg
tcaggtaatgcaagattcattgagcttagcggtaaattcctaggtgctcatcttgtgcat
acagctcttattctagtttgggctggaacaatgtctttgtttgaattatctcacttcatc
ccagaaaaacctctatatgagcaaggctttattcttcttcctcacttgtttgaattgggt
ggcaatctatattcacacttcataataagcatcctacatctcatctctgcaggtatctta
gctcttggaggtatttatcatgctatagttggtggtgaaagactagaagaaacaagctat
agctccctatttgcttatgggttacaagatagattccgtattacagctatacttggttca
catttacttactctaggaattggagcaggcttactctttgcaaaagcagtctatttaggt
ggtctttatgatacatgggcttgtggtggtggagacatgagattgattgaaagggtagag
cttggtttaaatccttatctactagctcagtacttacttcgtgctccatttgggtcatca
ggatggataattagcatcaataacatggaagatctcattggaggtcattattgggtctca
ttttatctaattctaggcagctgttggcatattcaaacaagagctcttacaattacaaca
agagcttttacatggtcagcagaagcttacctctcttatactttatcagctgtaacttta
tgtggttctatcacaagattattttcttggttcaataatactgcatatccaagtgaattc
tatggacctactggggcagaagcctctcaagcacaaagctttacattcctcatacgagat
tggaaattgggtataaagataacttcatccgaaggtcctacagctcttggtaagtatctc
atgagatctccaacaggtgaaataatatttggtggtgaaagtatgagattctggtctatg
caagcacattggcttgaagctctcagaacatcatttggactggacttatctaagattcaa
tctgatattcaaacttggcaagaaagaagagctgcagagtacatgacccatgcaccacta
gggagcttaaattctgttggtggtgttgctactgaaattaactctgtaaactatgtatct

cctagatcttggttaacctcctctcattggttcttggccttctttattttagttggtcat
tggtggcatggagcaagagcaagagcttcagcattatctagtgaaacaggcttatctcga
gtatatgaacctgttctttatatgcgacctattgattag
>Irm22_psbC
atgcctctcatttattctttaaaacgacgaactctagttggatccaggtatgcttggtgg
tcaggtaatgcaagattcattgagctaagtggtaaattcctaggtgctcatcttgttcat
acagctcttattctagtttgggcaggaagaatgtctttgtttgaattatctcacttcatc
ccagaaaaacctctatatgagcaaggctttattcttcttcctcacttgtttgaattgggt
ggcactttatattcacacttcataataagcatcctacatctcatctctgcaggtatccta
gctcttggaggtatttatcatgctatagttggtggtgaaagactagaagaaacaagctat
agctccctatttgcttatgggttacaagatagattccgtattacagctatacttggttca
catttacttactctaggaattggagcaggcttactctttgcaaaagcagtctatttaggt
ggtctttatgatacatgggcttgtggtggtggagacatgagattgattgaaagggtagag
cttggtttaaatccttatctactagctcaatacttacttcgtgctccatttggttcatca
ggatggataattagcatcaataacatggaagatctaattggaggccattattgggtctca
ttttatctaattctaggtagctgttggcatattcaaacaagagctcttacaatcacaaca
agagcttttacatggtcagcagaagcttacctctcttacactctatcagctgtaacttta
tgtggttctatcacaagtttgttttcttggtacaataatactgcatatccaagtgaattc
tatggacctactggagcagaagcctctcaagcacaaagctttacattccttatacgagat
tggaaattgggtataaagataagttcatccgaaggtcctacagctcttggtaagtatctc
atgagatctccaacaggtgaaataatatttggtggtgaaagtatgagattctggtctatg
caagcacattggcttgaagctctcagaacatcctttggattagacttatctaagattcaa
tctgatattcaaacttggcaagaaagaagagctgcagagtacatgacccatgcaccacta
gggagcttaaattctgttggtggtgttgctactgaaattaactctgtaaactatgtatct
cctagatcttggttaacctcctctcattggttcttggccttctttattttagtgggtcat
tggtggcatggagcaagatcaagagctgcagcattgtctagtgaaacaggtttatctcga
gtatatgaacctgtgctttatatgcgacctattgattag
>Irm23_psbC
atgcctctcatttattctttaaaacgacgaactctagttggatccaggtatgcttggtgg
tcaggtaatgcaagattcattgagcttagcggtaaattcctaggtgctcatcttgtgcat
acagctcttattctagtttgggcaggaagaatgtctttgtttgaattatctcacttcatt
ccagaaaaacctctatatgagcaaggctttattcttctacctcatttgtttgaactggga
ggcaatctatattcacacttcataataagcatcctacatctcatctctgcaggtatccta
gctcttggaggtatttatcatgctatagttggtgctgaaagattagaagaaacaagctat
agctccctatttgcttatgggttacaagatagattccgtattacagctatacttggttca
catttacttactctaggaattggagcagccttactctttgcaaaagcagtctatttaggt
ggtctttatgatacatgggcttgtggtggtggagacatgagattgattgaaagggtagag
cttggtttaaatccttatctactagctcaatacttacttcgtgctccatttggttcatca
ggatggataattagcatcaataacatggaagatctcattggaggtcattattgggtctca
ttttatctaattctaggcagctgttggcatattcaaacaagagctcttacaattacaaca
agagcttttacatggtcagcagaagcttacctctcttacactctatcagctgtaacttta
tgtggttctatcacagcattattttcttggttcaataatactgcatatccaagtgaattc
tatggacctactggggcagaagcctctcaagcacaaagctttacattccttatacgagat
tggaaattgggtataaagataacttcatccgaaggtcctacagctcttggtaagtatctc
atgagatctccaacaggtgaaataatatttggtggtgaaagtatgagattctggtctatg
caagcacattggcttgaagctctcagaacatcatttggactggacttatctaagattcaa
tctgatattcaaacttggcaagaaagaagagctgcagagtatatgacccatgcaccacta
gggagcttaaattctgttggtggtgttgctactgaaattaactctgtaaactatgtatct
cctagatcttggttaacctcctctcattggttcttggccttctttattttagtgggtcat
tggtggcatggagcaagatcaagagctgcagcattgtctagtgaaacaggtttatctcga
gtatatgaacctgtgctttatatgcgacctattgattag
>Irm24_psbC
atgcctctcatttattctttaaaacgacgaactctagttggatccaagtatgcttggtgg
tcaggtaatgcaagattcattgagctaagtggtaaattcctaggtgctcatcttgttcat
acagctcttattctagtttgggcaggaagaatgtctttgtttgaattatctcacttcatt
ccagaaaaacctctatatgagcaaggctttattcttctacctcatttgtttgaactggga
ggcaatctatattcacacttcataataagcatcctacatctcatctctgcaggtatctta

gctcttggaggtatttatcatgctatagttggtggtgaaagactagaagaaacaagctat
agctccctatttgcttatgggttacaagatagattccgtattacagctatacttggttca
catttacttactctaggaattggagcaggcttactctttgcaaaagcagtctatttaggt
ggtctttatgatacatgggcttgtggtggtggagacatgagattgattgaaagggtagag
cttggtttaaatccttatctactagctcagtacttacttcgtgctccatttggttcatca
ggatggataattagcatcaataacatggaagatctaattggaggccattattgggtctca
ttttatctaattctaggtagctgttggcatattcaaacaagagctcttacaatcacaaca
agagcttttacatggtcagcagaagcttacctctcttatactttatcagctgtaacttta
tgtggttctatcacagcattattttcttggttcaataatactgcatatccaagtgaattc
tatggacctactggggcagaagcctctcaagcacaaagctttacattcctcatacgagat
tggaaattgggtataaagataacttcatccgaaggtcctacagctcttggtaagtatctc
atgagatctccaacaggtgaaataatatttggtggtgaaagtatgagattctggtctatg
caagcacattggcttgaagctctcagaacatcctttggattagacttatctaagattcaa
tctgatattcaaacttggcaagaaagaagagctgcagagtatatgacccatgcaccacta
gggagcttaaattctgttggtggtgttgctactgaaattaactctgtaaactatgtatct
cctagatcttggttaacctcctctcattggttcttggccttctttattttagtgggtcat
tggtggcatggagcaagatcaagagctgcagcattgtctagtgaaacaggtttatctcga
gtatatgaacctgtgctttatatgcgacctattgattag
>Irm25_psbC
atgcctctcatttattctttaaaacgacgaactctagttggatccaggtatgcttggtgg
tcaggtaatgcaagattcattgagcttagcggtaaattcctaggtgctcatcttgtgcat
acagctcttattctagtttgggcaggaagaatgtctttgtttgaattatctcacttcatt
ccagaaaaacctctatatgagcaaggctttattcttctacctcatttgtttgaactggga
ggcaatctatattcacacttcataataagcatcctacatctcatctctgcaggtatctta
gctcttggaggtatttatcatgctatagttggtgctgaaagattagaagaaacaagcttc
agtgctctatttgcttatgggttacaagatagattccgtattactgctatacttggatca
catttacttactctaggaattggagcagccttactctttgcaaaagcagtctatttaggt
ggtctttatgatacatgggcttgtggtggtggagacatgagattgattgaaagagtagag
cttggtttaaatccttatctactagctcaatacttacttcgtgctccatttgggtcatca
ggatggataattagcatcaataacatggaagatctcattggaggtcattattgggtctca
ttttatctaattctaggtagctgttggcatattcaaacaagagctcttacaatcacaaca
agagcttttacatggtcagcagaagcttacctctcttacactctatcagctgtaacttta
tgtggttctatcacaagtttgttttcttggtacaataatactgcatatccaagtgaattc
tatggacctactggagcagaagcctctcaagcacaaagctttacattccttatacgagat
tggaaattgggtataaagataagttcatccgaaggtcctacagctcttggtaagtatctc
atgagatctccaacaggtgaaataatatttggtggtgaaagtatgagattctggtctatg
caagcacattggcttgaagctctcagaacatcctttggattagacttatctaagattcaa
tctgatattcaaacttggcaagaaagaagagctgcagagtacatgacccatgcaccacta
gggagcttaaattctgttggtggtgttgctactgaaattaactctgtaaactatgtatct
cctagatcttggttaacctcctctcattggttcttggccttctttattttagttggtcat
tggtggcatggagcaagagcaagagcttcagcattatctagtgaaacaggcttatctcga
gtatatgaacctgttctttatatgcgacctattgattag
>Irm26_psbC
atgcctctcatttattctttaaaacgacgaactctagttggatccaagtatgcttggtgg
tcaggtaatgcaagattcattgagctaagcggtaaattcctaggtgctcatcttgtgcat
acagctcttattctagtttgggctggaacaatgtctttgtttgaattatctcacttcatc
ccagaaaaacctctatatgagcaaggctttattcttctacctcatttgtttgaactggga
ggcaatctatattcacacttcataataagcatcctacatctcatctctgcaggtatccta
gctcttggaggtatttatcatgctatagttggtgctgaaagattagaagaaacaagcttc
agtgctctatttgcttatgggttacaagatagattccgtattactgctatacttggatca
catttacttactctaggaattggagcagccttactctttgcaaaagcagtctatttaggt
ggtctttatgatacatgggcttgtggtggtggagacatgagattgattgaaagggtagag
cttggtttaaatccttatctactagctcagtacttacttcgtgctccatttgggtcatca
ggatggataattagcatcaataacatggaagatctaattggaggccattattgggtctca
ttttatctaattctaggtagctgttggcatattcaaacaagagctcttacaatcacaaca
agagcttttacatggtcagcagaagcttacctctcttatactttatcagctgtaacttta
tgtggttctatcacagcattattttcttggttcaataatactgcatatccaagtgaattc

tatggacctactggggcagaagcctctcaagcacaaagctttacattccttatacgagat
tggaaattgggtataaagataacttcatccgaaggtcctacagctcttggtaagtatctc
atgagatctccaacaggtgaaataatatttggtggtgaaagtatgagattctggtctatg
caagcacattggcttgaagctctcagaacatcctttggattagacttatctaagattcaa
tctgatattcaaacttggcaagaaagaagagctgcagagtatatgacccatgcaccacta
gggagcttaaattctgttggtggtgttgctactgaaattaactctgtaaactatgtatct
cctagatcttggttaacctcctctcattggttcttggccttctttattttagtgggtcat
tggtggcatggagcaagatcaagagctgcagcattgtctagtgaaacaggcttatctcga
gtatatgaacctgttctttatatgcgacctattgattag
>Irm27_psbC
atgcctctcatttattctttaaaacgacgaactctagttggatccaagtatgcttggtgg
tcaggtaatgcaagatttattgagcttagtgggaagttcctaggtgctcatcttgttcat
gcagctcttattctagtttgggcaggaagaatgtctttgtttgaattatctcacttcatt
ccagaaaaacctctatatgagcaaggctttattcttctacctcatttgtttgaactggga
ggcaatctatattcacacttcataataagcatcctacatctcatctctgcaggtatctta
gctcttggaggtatttatcatgctatagttggtggtgaaagactagaagaaacaagctat
agctccctatttgcttatgggttacaagatagattccgtattacagctatacttggttca
catttacttactctaggaattggagcaggcttactctttgcaaaagcagtctatttaggt
ggtctttatgatacttgggcttgtggtggtggagacatgagattgattgaaagagtagag
cttggtttaaatccttatctactagctcaatacttacttcgtgctccatttggttcatca
ggatggataattagcatcaataacatggaagatctaattggaggccattattgggtctca
ttttatctaattctaggtagctgttggcatattcaaacaagagctcttacaatcacaaca
agagcttttacatggtcagcagaagcttacctctcttacactctatcagctgtaacttta
tgtggttctatcacaagtttgttttcttggtacaataatactgcatatccaagtgaattc
tatggacctactggagcagaagcctctcaagcacaaagctttacattccttatacgagat
tggaaattgggtataaagataagttcatccgaaggtcctacagctcttggtaagtatctc
atgagatctccaacaggtgaaataatatttggtggtgaaagtatgagattttgggctatg
caagcacattggcttgaagctttaagaacatcttttggcttggacttatctaagattcaa
tctgatgttcaaacttggcaagaaagaagagcagctgagtacatgacacatgcaccacta
gggagcttaaattctgttggtggtgttgctactgaaattaactctgtaaactatgtatct
cctagatcttggttaacctcctctcattggttcttggccttctttattttagttggtcat
tggtggcatggagcaagagcaagggcatcagcattatctagtgaaacaggtttatcgcgt
atatatgaacctgtactttacatgagacctattgattag
>Irm3_psbC
atgcctctcatttattctttaaaacgacgaactctagttggatccaggtatgcctggtgg
tctggtaatgcaagatttattgagcttagtgggaagttcctaggtgctcatcttgtacat
gcagctcttattctagtttgggcaggaagaatgtctttgtttgaattatctcacttcatt
ccagaaaaacctctatatgagcaaggttttattcttcttcctcatctttttgaattagga
ggtaatctatactcacacttcataataagtattctacatcttatctctgctggtattctt
gcacttggaggtatttatcatgctatagttggtgctgaaagactagaagaaacaagattc
agtgctatatttgcttatggcttacaagatagattccgtattacagctatactaggttca
catttacttactctaggaattggagcagccttactctttgctaaagcagtctatctaggt
ggtctttatgatacatgggcttgtggtggtggagatatgagattgattgaaagggtggag
cttggattaaatccttatctactagctcagtatttacttcgtgctccgtttggctcatca
gggtggataattagtatcaataacatggaagatcttattggaggtcattattgggtcgca
ttttatctaattctaggtagctgttggcatattcaaacaagacctcttacaattataaca
agagcttttacatggtcagctgaagcttacctctcttacactttatcagccgtaacttta
tgtgcttctaccacaagcttgttttcctggtataataatactgcttatccaagtgaattc
tatggacctacagcagcagaagcctctcaagcacaaagctttacatttctcatacgagat
tggaagttaggtataaagataacttcatccgaaggtcctacagctcttggtaagtatctc
atgagatctccaacaggtgaaataatatttggtggtgaaagtatgagattttgggctatg
caagcacattggcttgaagctttaagaacatcttttggcttggacttatctaagattcaa
tctgatgttcaaacttggcaagaaagaagagcagctgagtacatgacacatgctcctcta
ggaagcttaaattctgtcggtggtgttgctactgaaattaactctgtaaactatgtatct
cctagatcttggttaacttcctcccattggtttttggccttctttattttagttggtcat
tggtggcatggagcaagagcaagggcatcagcattatctagtgaaacaggtttatcgcgt
atatatgaacctgtactttatatgcgacctattgattag

>Irm4_psbC
atgcctctcatttattctttaaaacgacgaactctagttggatccaggtatgcctggtgg
tctggtaatgcaagatttattgagcttagtgggaaattcctaggtgctcatcttgtgcat
acagctcttattctagtttgggctggaacaatgtctttgtttgaattatctcacttcatt
ccagaaaaacctctatatgagcaaggttttattcttcttcctcatctttttgaattagga
ggtaatctatactcacacttcataataagtatcctacatcttatctctgctggtattctt
gcacttggaggtatttatcatgctatagttggtgctgaaagactagaagaaacaagattc
agtgctatatttgcttatggcttacaagatagattccgtattacagctatactaggttca
catttacttactctaggaattggagcagccttactctttgctaaagcagtctatttaggt
ggtctttatgatacatgggcttgtggtggtggagacatgagattgattgaaagggtagag
cttggtttaaatccttatctactagctcaatacttacttcgtgctccatttgggtcatca
ggatggataattagcatcaataacatggaagatctcattggaggtcattattgggtcgca
ttttatctaattctaggtagctgttggcatattcaaacaagacctcttacaattataaca
agagcttttacatggtcagctgaagcttacctctcttacactttatcagccgtaacttta
tgtgcttctaccacaagcttgttttcctggtacaataatactgcttatccaagtgaattc
tatggacctacagcggcagaagcctctcaagcacaaagctttacatttctcatacgagat
tggaagttaggtataaagataacttcatccgaaggtcctacagctcttggtaagtatctc
atgagatctccaacaggtgaaataatatttggtggtgaaagtatgagattctggtctatg
caagcacattggcttgaagctctcagaacatcttttggcctggacttatctaagattcaa
tctgatgttcaaacttggcaagaaagaagagcagctgagtacatgacacatgctcctcta
ggaagcttaaattctgtcggtggtgttgctactgaaattaactctgtaaactatgtatct
cctagatcttggttaacttcatcccactggtttttagcattctttattttagttggtcat
tggtggcatggagcaagatcaagagctgcagcattgtctagtgaaacaggtttatctcga
gtatatgaacctgtgctttatatgcgacctattgattag
>Irm5_psbC
atgcctctcatttattctttaaaacgacgaactctagttggatccaggtatgcttggtgg
tcaggtaatgcaagattcattgagcttagcggtaaattcctaggtgctcatcttgtgcat
acagctcttattctagtttgggctggaacaatgtctttgtttgaattatctcacttcatc
ccagaaaaacctctatatgagcaaggctttattcttcttcctcacttgtttgaattgggt
ggcactttatattcacacttcataataagcatcctacatctcatctctgcaggtatccta
gctcttggaggtatttatcatgctatagttggtgctgaaagattagaagaaacaagcttc
agtgctctatttgcttatgggttacaagatagattccgtattactgctatacttggatca
catttacttactctaggaattggagcagccttactctttgcaaaagcagtctatttaggt
ggtctttatgatacatgggcttgtggtggtggagacatgagattgattgaaagggtagag
cttggtttaaatccttatctactagctcagtacttacttcgtgctccatttgggtcatca
ggatggataattagcatcaataacatggaagatctcattggaggtcattattgggtctca
ttttatctaattctaggtagctgttggcatattcaaacaagagctcttacaattacaaca
agagcttttacatggtcagcagaagcttacctctcttatactttatcagctgtaacttta
tgtggttctatcacagcattattttcttggttcaataatactgcatatccaagtgaattc
tatggacctactggggcagaagcctctcaagcacaaagctttacattcctcatacgagat
tggaaattgggtataaagataacttcatccgaaggtcctacagctcttggtaagtatctc
atgagatctccaacaggtgaaataatatttggtggtgaaagtatgagattctggtctatg
caagcacattggcttgaagctctcagaacatcatttggactggacttatctaagattcaa
tctgatattcaaacttggcaagaaagaagagctgcagagtatatgacccatgcaccacta
gggagcttaaattctgttggtggtgttgctactgaaattaactctgtaaactatgtatct
cctagatcttggttaacatcatctcactggttcttagcattctttattttagttggtcat
tggtggcatggagcaagagcaagagcttcagcattatctagtgaaacaggcttatctcga
gtatatgaacctgttctttatatgcgacctattgattag
>Irm7_psbC
atgcctctcatttattctttaaaacgacgaactctagttggatccaggtatgcttggtgg
tcaggtaatgcaagattcattgagcttagcggtaaattcctaggtgctcatcttgtgcat
acagctcttattctagtttgggcaggaagaatgtctttgtttgaattatctcacttcatt
ccagaaaaacctctatatgagcaaggctttattcttctacctcatttgtttgaactggga
ggcaatctatattcacacttcataataagcatcctacatctcatctctgcaggtatctta
gctcttggaggtatttatcatgctatagttggtggtgaaagactagaagaaacaagctat
agctccctatttgcttatgggttacaagatagattccgtattacagctatacttggttca
catttacttactctaggaattggagcaggcttactctttgcaaaagcagtctatttaggt

ggtctttatgatacttgggcttgtggtggtggagacatgagattgattgaaagagtagag
cttggtttaaatccttatctactagctcaatacttacttcgtgctccatttggttcatca
ggatggataattagcatcaataacatggaagatctaattggaggccattattgggtctca
ttttatctaattctaggtagctgttggcatattcaaacaagagctcttacaatcacaaca
agagcttttacatggtcagcagaagcttacctctcttatactttatcagctgtaacttta
tgtggttctatcacagcattattttcttggttcaataatactgcatatccaagtgaattc
tatggacctactggggcagaagcctctcaagcacaaagctttacattcctcatacgagat
tggaaattgggtataaagataagttcatccgaaggtcctacagctcttggtaagtatctc
atgagatctccaacaggtgaaataatatttggtggtgaaagtatgagattctggtctatg
caagcacattggcttgaagctctcagaacatcctttggattagacttatctaagattcaa
tctgatattcaaacttggcaagaaagaagagctgcagagtatatgacccatgcaccacta
gggagcttaaattctgttggtggtgttgctactgaaattaactctgtaaactatgtatct
cctagatcttggttaacatcatctcactggttcttagcattctttattttagttggtcat
tggtggcatggagcaagagcaagagctgcagcattgtctagtgaaacaggtttatctcga
gtatatgaacctgtgctttatatgcgacctattgattag
>Irm9_psbC
atgcctctcatttattctttaaaacgacgaactctagttggatccaggtatgcctggtgg
tctggtaatgcaagatttattgagcttagtgggaagttcctaggtgctcatcttgtacat
gcagctcttattctagtttgggcaggaagaatgtctttgtttgaattatctcacttcatt
ccagaaaaacctctatatgagcaaggctttattcttctacctcatttgtttgaactggga
ggcaatctatattcacacttcataataagtattctacatcttatctctgctggtattctt
gcacttggaggtatttatcatgctatagttggtgctgaaagactagaagaaacaagctat
agctccctatttgcttatgggttacaagatagattccgtattacagctatacttggttca
catttacttactctaggaattggagcaggcttactctttgcaaaagcagtctatttaggt
ggtctttatgatacttgggcttgtggtggtggagacatgagattgattgaaagagtagag
cttggtttaaatccttatctactagctcaatacttacttcgtgctccatttggttcatca
ggatggataattagcatcaataacatggaagatctaattggaggccattattgggtctca
ttttatctaattctaggtagctgttggcatattcaaacaagagctcttacaattacaaca
agagcttttacatggtcagcagaagcttacctctcttatactttatcagccgtaacttta
tgtgcttctaccacaagcttgttttcctggtataataatactgcttatccaagtgaattc
tatggacctacagcagcagaagcctctcaagcacaaagctttacattccttatacgagat
tggaaattgggtataaagataagttcatccgaaggtcctacagctcttggtaagtatctc
atgagatctccaacaggtgaaataatatttggtggtgaaagtatgagattctggtctatg
caagcacattggcttgaagctctcagaacatcctttggattagacttatctaagattcaa
tctgatattcaaacttggcaagaaagaagagctgcagagtacatgacccatgcaccacta
gggagcttaaattctgttggtggtgttgctactgaaattaactctgtaaactatgtatct
cctagatcttggttaacctcctctcattggttcttggccttctttattttagtgggtcat
tggtggcatggagcaagatcaagagctgcagcattgtctagtgaaacaggtttatctcga
gtatatgaacctgtgctttatatgcgacctattgattag
>Isy12_psbC
atgcctctcatttattctttaaaacgacgaactctagttggatccaggtatgcttggtgg
tcaggtaatgcaagattcattgagcttagcggtaaattcctaggtgctcatcttgtgcat
acagctcttattctagtttgggctggaacaatgtctttgtttgaattatctcacttcatt
ccagaaaaacctctatatgagcaaggctttattcttctacctcatttgtttgaactggga
ggcaatctatattcacacttcataataagcatcctacatctcatctctgcaggtatctta
gctcttggaggtatttatcatgctatagttggtgctgaaagattagaagaaacaagcttc
agtgctctatttgcttatgggttacaagatagattccgtattactgctatacttggttca
catttacttactctaggaattggagcaggcttactctttgcaaaagcagtctatttaggt
ggtctttatgatacatgggcttgtggtggtggagacatgagattgattgaaagggtagag
cttggtttaaatccttatctactagctcagtacttacttcgtgctccatttgggtcatca
ggatggataattagcatcaataacatggaagatctcattggaggtcattattgggtctca
ttttatctaattctaggcagctgttggcatattcaaacaagagctcttacaatcacaaca
agagcttttacatggtcagcagaagcttacctctcttatactttatcagctgtaacttta
tgtggttctatcacaagtttgttttcttggtacaataatactgcatatccaagtgaattc
tatggacctactggagcagaagcctctcaagcacaaagctttacattcctcatacgagat
tggaaattgggtataaagataacttcatccgaaggtcctacagctcttggtaagtatctc
atgagatctccaacaggtgaaataatatttggtggtgaaagtatgagattctggtctatg

caagcacattggcttgaagctctcagaacatcatttggactggacttatctaagattcaa
tctgatattcaaacttggcaagaaagaagagctgcagagtatatgacccatgcaccacta
gggagcttaaattctgttggtggtgttgctactgaaattaactctgtaaactatgtatct
cctagatcttggttaacctcctctcattggttcttggccttctttattttagtgggtcat
tggtggcatggagcaagatcaagagctgcagcattgtctagtgaaacaggcttatctcga
gtatatgaacctgttctttatatgcgacctattgattag
>Isy15_psbC
atgcctcttctttattctttaaaacgacgaactctagttggatccaggtatgcctggtgg
tctggtaatgcaagatttattgagcttagtgggaagttcctaggtgctcatcttgtacat
gcagctcttattctagtttgggcaggaagaatgtctttgtttgaattatctcacttcatt
ccagaaaaacctctatatgagcaaggctttattcttcttcctcacttgtttgaattgggt
ggtaatctatactcacacttcataataagtattctacatcttatctctgctggtattctt
gcacttggaggtatttatcatgctatagttggtgctgaaagactagaagaaacaagattc
agtgctatatttgcttatggcttacaagatagattccgtattacagctatactaggttca
catttacttactctaggaattggagcagccttactctttgctaaagcagtctatctaggt
ggtctttatgatacatgggcttgtggtggtggagatatgagattgattgaaagggtggag
cttggattaaatccttatctactagctcagtatttacttcgtgctccgtttggctcatca
gggtggataattagtatcaataacatggaagatcttattggaggtcattattgggtcgca
ttttatctaattctaggtagctgttggcatattcaaacaagacctcttacaattataaca
agagcttttacatggtcagctgaagcttacctctcttatactttatcagccgtaacttta
cgtgcttctaccacaagcttgttttcctggtataataatactgcttatccaagtgaattc
tatggacctacagcagcagaagcctctcaagcacaaagctttacatttctcatacgagat
tggaagttaggtataaagataacttcatccgaaggtcctacagctcttggtaagtatctc
atgagatctccaacaggtgaaataatatttggtggtgaaagtatgagattttgggctatg
caagcacattggcttgaagctttaagaacatcttttggcctggacttatctaagattcaa
tctgatgttcaaacttggcaagaaagaagagcagctgagtacatgacacatgctcctcta
ggaagcttaaattctgtcggtggtgttgctactgaaattaactctgtaaactatgtatct
cctagatcttggttaacttcaacccactggtttttggccttctttattttagttggtcat
tggtggcatggagcaagagcaagggcatcagcattatctagtgaaacaggcttatcgcgt
atatatgaacctgttctttatatgcgacctattgattag
>Isy16_psbC
atgcctctcatttattctttaaaacgacgaactctagttggatccaggtatgcttggtgg
tcaggtaatgcaagattcattgagcttagcggtaaattcctaggtgctcatcttgtgcat
acagctcttattctagtttgggctggaacaatgtctttgtttgaattatctcacttcatc
ccagaaaaacctctatatgagcaaggctttattcttcttcctcacttgtttgaattgggt
ggcactttatattcacacttcataataagcatcctacatctcatctctgcaggtatccta
gctcttggaggtatttatcatgctatagttggtgctgaaagattagaagaaacaagcttc
agtgctctatttgcttatgggttacaagatagattccgtattactgctatacttggatca
catttacttactctaggaattggagcagccttactctttgcaaaagcagtctatttaggt
ggtctttatgatacatgggcttgtggtggtggagacatgagattgattgaaagggtagag
cttggtttaaatccttatctactagctcagtacttacttcgtgctccatttgggtcatca
ggatggataattagcatcaataacatggaagatctcattggaggtcattattgggtctca
ttttatctaattctaggcagctgttggcatattcaaacaagagctcttacaattacaaca
agagcttttacatggtcagcagaagcttacctctcttatactttatcagctgtaacttta
tgtggttctatcacagcattattttcttggttcaataatactgcatatccaagtgaattc
tatggacctactggggcagaagcctctcaagcacaaagctttacattcctcatacgagat
tggaaattgggtataaagataacttcatccgaaggtcctacagctcttggtaagtatctc
atgagatctccaacaggtgaaataatatttggtggtgaaagtatgagattctggtctatg
caagcacattggcttgaagctctcagaacatcatttggactggacttatctaagattcaa
tctgatattcaaacttggcaagaaagaagagctgcagagtatatgacccatgcaccacta
gggagcttaaattctgttggtggtgttgctactgaaattaactctgtaaactatgtatct
cctagatcttggttaacatcatctcactggttcttagcattctttattttagttggtcat
tggtggcatggagcaagagcaagagcttcagcattatctagtgaaacaggcttatctcga
gtatatgaacctgttctttatatgcgacctattgattag
>Isy17_psbC
atgcctctcatttattctttaaaacgacgaactctagttggatccaggtatgcttggtgg
tcaggtaatgcaagattcattgagcttagcggtaaattcctaggtgctcatcttgtgcat

acagctcttattctagtttgggctggaacaatgtctttgtttgaattatctcacttcatc
ccagaaaaacctctatatgagcaaggctttattcttcttcctcacttgtttgaattgggt
ggcactttatattcacacttcataataagcatcctacatctcatctctgcaggtatccta
gctcttggaggtatttatcatgctatagttggtgctgaaagattagaagaaacaagcttc
agtgctctatttgcttatgggttacaagatagattccgtattactgctatacttggttca
catttacttactctaggaattggagcaggcttactctttgcaaaagcagtctatttaggt
ggtctttatgatacttgggcttgtggtggtggagacatgagattgattgaaagggtagag
cttggtttaaatccttatctactagctcagtacttacttcgtgctccatttgggtcatca
ggatggataattagcatcaataacatggaagatctcattggaggtcattattgggtctca
ttttatctaattctaggcagctgttggcatattcaaacaagagctcttacaattacaaca
agagcttttacatggtcagcagaagcttacctctcttatactttatcagctgtaactttta
tgtggttctatcacagcattattttcttggttcaataatactgcatatccaagtgaattc
tatggacctactggggcagaagcctctcaagcacaaagctttacattcctcatacgagat
tggaaattgggtataaagataacttcatccgaaggtcctacagctcttggtaagtatctc
atgagatctccaacaggtgaaataatatttggtggtgaaagtatgagattctggtctatg
caagcacattggcttgaagctctcagaacatcatttggactggacttatctaagattcaa
tctgatattcaaacttggcaagaaagaagagctgcagagtatatgacccatgcaccacta
gggagcttaaattctgttggtggtgttgctactgaaattaactctgtaaactatgtatct
cctagatcttggttaacatcatctcactggttcttagcattctttattttagttggtcat
tggtggcatggagcaagagcaagagcttcagcattatctagtgaaacaggcttatctcga
gtatatgaacctgttctttatatgcgacctattgattag
>Isy18_psbC
atgcctctcatttattctttaaaacgacgaactctagttggatccaggtatgcttggtgg
tcaggtaatgcaagattcattgagcttagcggtaaattcctaggtgctcatcttgtgcat
acagctcttattctagtttgggctggaacaatgtctttgtttgaattatctcacttcatc
ccagaaaaacctctatatgagcaaggctttattcttcttcctcatttgtttgaattggga
ggcactttatattcacacttcataataagcatcctacatctcatctctgcaggtatctta
gctcttggaggtatttatcatgctatagttggtgctgaaagattagaagaaacaagcttc
agtgctctatttgcttataggttacaagatagattccgtattactgctatacttggatca
catttacttactctaggaattggagcaggcttactctttgcaaaagcagtctatttaggt
ggtctttatgatacatgggcttgtggtggtggagacatgagattgattgaaagggtagag
cttggtttaaatccttatctactagctcagtacttacttcgtgctccatttggttcatca
ggatggataattagcatcaataacatggaagatctcattggaggtcattattgggtctca
ttttatctaattctaggcagctgttggcatattcaaacaagagctcttacaattacaaca
agagcttttacatggtcagcagaagcttacctctcttatactttatcagctgtaactttta
tgtggttctatcacagcattattttcttggttcaataatactgcatatccaagtgaattc
tatggacctactggggcagaagcctctcaagcacaaagctttacattcctcatacgagat
tggaaattgggtataaagataacttcatccgaaggtcctacagctcttggtaagtatctc
atgagatctccaacaggtgaaataatatttggtggtgaaagtatgagattctggtctatg
caagcacattggcttgaagctctcagaacatcctttggattagacttatctaagattcaa
tctgatattcaaacttggcaagaaagaagagctgcagagtatatgacccatgcaccacta
gggagcttaaattctgttggtggtgttgctactgaaattaactctgtaaactatgtatct
cctagatcttggttaacctcctctcattggttcttggccttctttattttagtgggtcat
tggtggcatggagcaagatcaagagctgcagcattatctagtgaaacaggcttatctcga
gtatatgaacctgttctttatatgcgacctattgattag
>Isy21_psbC
atgcctctcatttattctttaaaacgacgaactctagttggatccaggtatgcttggtgg
tcaggtaatgcaagattcattgagcttagcggtaaattcctaggtgctcatcttgtgcat
acagctcttattctagtttgggctggaacaatgtctttgtttgaattatctcacttcatc
ccagaaaaacctctatatgagcaaggctttattcttcttcctcacttgtttgaattgggt
ggcaatctatattcacacttcataataagcatcctacatctcatctctgcaggtatccta
gctcttggaggtatttatcatgctatagttggtgctgaaagattagaagaaacaagcttc
agtgctctatttgcttatgggttacaagatagattccgtattactgctatacttggatca
catttacttactctaggaattggagcagccttactctttgcaaaagcagtctatttaggt
ggtctttatgatacatgggcttgtggtggtggagacatgagattgattgaaagggtagag
cttggtttaaatccttatctactagctcagtacttacttcgtgctccatttgggtcatca
ggatggataattagcatcaataacatggaagatctcattggaggtcattattgggtctca

ttttatctaattctaggcagctgttggcatattcaaacaagagctcttacaatcacaaca
agagcttttacatggtcagcagaagcttacctctcttatactttatcagctgtaacttta
tgtggttctatcacagcattattttcttggtacaataatactgcatatccaagtgaattc
tatggacctactggagcagaagcctctcaagcacaaagctttacattcctcatacgagat
tggaaattgggtataaagataacttcatccgaaggtcctacagctcttggtaagtatctc
atgagatctccaacaggtgaaataatatttggtggtgaaagtatgagattctggtctatg
caagcacattggcttgaagctctcagaacatcatttggactggacttatctaagattcaa
tctgatattcaaacttggcaagaaagaagagctgcagagtatatgacccatgcaccacta
gggagcttaaattctgttggtggtgttgctactgaaattaactctgtaaactatgtatct
cctagatcttggttaacatcatctcactggttcttagcattcttcattttagttggtcat
tggtggcatggagcaagagcaagagcttcagcattatctagtgaaacaggcttatctcga
gtatatgaacctgttctttatatgcgacctattgattag
>Isy22_psbC
atgcctctcatttattctttaaaacgacgaactctagttggatccaggtatgcttggtgg
tcaggtaatgcaagattcattgagcttagcggtaaattcctaggtgctcatcttgtgcat
acagctcttattctagtttgggctggaacaatgtctttgtttgaattatctcacttcatt
ccagaaaaacctctatatgagcaaggctttattcttcttcctcacttgtttgaattgggt
ggcaatctatattcacacttcataataagcatcctacatctcatctctgcaggtatccta
gctcttggaggtatttatcatgctatagttggtgctgaaagattagaagaaacaagctat
agtgctctatttgcttatgggttacaagatagattccgtattactgctatacttggttca
catttacttactctaggaattggagcaggcttactctttgcaaaagcagtctatttaggt
ggtctttatgatacatgggcttgtggtggtggagacatgagattgattgaaagggtagag
cttggtttaaatccttatctactagctcagtacttacttcgtgctccatttgggtcatca
ggatggataattagcatcaataacatggaagatctcattggaggtcattattgggtctca
ttttatctaattctaggcagctgttggcatattcaaacaagagctcttacaattacaaca
agagcttttacatggtcagcagaagcttacctctcttatactttatcagctgtaacttta
tgtggttctatcacagctttattttcttggttcaataatactgcatatccaagtgaattc
tatggacctactggggcagaagcctctcaagcacaaagctttacattcctcatacgagat
tggaaattgggtataaagataacttcatccgaaggtcctacagctcttggtaagtatctc
atgagatctccaacaggtgaaataatatttggtggtgaaagtatgagattctggtctatg
caagcacattggcttgaagctctcagaacatcatttggactggacttatctaagattcaa
tctgatattcaaacttggcaagaaagaagagctgcagagtatatgacccatgcaccacta
gggagcttaaattctgttggtggtgttgctactgaaattaactctgtaaactatgtatct
cctagatcttggttaacatcatctcactggttcttagcattcttcattttagttggtcat
tggtggcatggagcaagagcaagagcttcagcattatctagtgaaacaggcttatctcga
gtatatgaacctgtgctttatatgcgacctattgattag
>Isy23_psbC
atgcctcttcttcattctttaaaacgacgaactctagttggatccaggtatgcttggtgg
tcaggtaatgcaagattcattgagcttagcggtaaattcctaggtgctcatcttgtgcat
acagctcttattctagtttgggctggaacaatgtctttgtttgaattatctcacttcatc
ccagaaaaacctctatatgagcaaggctttattcttcttcctcacttgtttgaattgggt
ggcactttatattcacacttcataataagcatcctacatctcatctctgcaggtatccta
gctcttggaggtatttatcatgctatagttggtgctgaaagattagaagaaacaagcttc
agtgctctatttgcttatgggttacaagatagattccgtattactgctatacttggatca
catttacttactctaggaattggagcagccttactctttgcaaaagcagtctatttaggt
ggtctttatgatacatgggcttgtggtggtggagacatgagattgattgaaagggtagag
cttggtttaaatccttatctactagctcagtacttacttcgtgctccatttgggtcatca
ggatggataattagcatcaataacatggaagatctcattggaggtcattattgggtctca
ttttatctaattctaggcagctgttggcatattcaaacaagagctcttacaatcacaaca
agagcttttacatggtcagcagaagcttacctctcttatactttatcagctgtaacttta
tgtggttctatcacagcattattttcttggtacaataatactgcatatccaagtgaattc
tatggacctactggagcagaagcctctcaagcacaaagctttacattcctcatacgagat
tggaaattgggtataaagataagttcatccgaaggtcctacagctcttggtaagtatctc
atgagatctccaacaggtgaaataatatttggtggtgaaagtatgagattctggtctatg
caagcacattggcttgaagctctcagaacatcatttggactggacttatctaagattcaa
tctgatattcaaacttggcaagaaagaagagctgcagagtatatgacccatgcaccacta
gggagcttaaattctgttggtggtgttgctactgaaattaactctgtaaactatgtatct

cctagatcttggttaacatcatctcactggttcttagcattcttcattttagttggtcat
tggtggcatggagcaagagcaagagcttcagcattatctagtgaaacaggcttatctcga
gtatatgaacctgttctttatatgcgacctattgattag
>Isy24_psbC
atgcctctcattcattctttaaaacgacgaactctagttggatccaggtatgcttggtgg
tcaggtaatgcaagattcattgagcttagcggtaaattcctaggtgctcatcttgtgcat
acagctcttattctagtttgggctggaacaatgtctttgtttgaattatctcacttcatc
ccagaaaaacctctatatgagcaaggctttattcttcttcctcacttgtttgaattgggt
ggcactttatattcacacttcataataagcatcctacatctcatctctgcaggtatctta
gctcttggaggtatttatcatgctatagttggtggtgaaagactagaagaaacaagcttc
agtgccctatttgcttatgggttacaagatagattccgtattacagctatacttggttca
catttacttactctaggaattggagcagccttactctttgcaaaagcagtctatttaggt
ggtctttatgatacatgggcttgtggtggtggagacatgagattgattgaaagggtagag
cttggtttaaatccttatctactagctcagtacttacttcgtgctccatttgggtcatca
ggatggataattagcatcaataacatggaagatctcattggaggtcattattgggtctca
ttttatctaattctaggtagctgttggcatattcaaacaagagctcttacaattacaaca
agagcttttacatggtcagcagaagcttacctctcttatactttatcagctgtaacttta
tgtggttctatcacagcattattttcttggttcaataatactgcatatccaagtgaattc
tatggacctactggggcagaagcctctcaagcacaaagctttacattcctcatacgagat
tggaaattgggtataaagataacttcatccgaaggtcctacagctcttggtaagtatctc
atgagatctccaacaggtgaaataatatttggtggtgaaagtatgagattctggtctatg
caagcacattggcttgaagctctcagaacatcatttggactggacttatctaagattcaa
tctgatattcaaacttggcaagaaagaagagctgcagagtatatgacccatgcaccacta
gggagcttaaattctgttggtggtgttgctactgaaattaactctgtaaactatgtatct
cctagatcttggttaacatcatctcactggttcttagcattctttattttagttggtcat
tggtggcatggagcaagagcaagagcttcagcattatctagtgaaacaggcttatctcga
gtatatgaacctgttctttatatgcgacctattgattag
>Isy25_psbC
atgcctctcatttattctttaaaacgacgaactctagttggatccaggtatgcttggtgg
tcaggtaatgcaagattcattgagcttagcggtaaattcctaggtgctcatcttgtgcat
acagctcttattctagtttgggctggaacaatgtctttgtttgaattatctcacttcatc
ccagaaaaacctctatatgagcaaggctttattcttcttcctcacttgtttgaattgggt
ggcaatctatattcacacttcataataagcatcctacatctcatctctgcaggtatccta
gctcttggaggtatttatcatgctatagttggtgctgaaagattagaagaaacaagcttc
agtgctctatttgcttatgggttacaagatagattccgtattactgctatacttggatca
catttacttactctaggaattggagcagccttactctttgcaaaagcagtctatttaggt
ggtctttatgatacatgggcttgtggtggtggagacatgagattgattgaaagggtagag
cttggtttaaatccttatctactagctcagtacttacttcgtgctccatttgggtcatca
ggatggataattagcatcaataacatggaagatctcattggaggtcattattgggtctca
ttttatctaattctaggcagctgttggcatattcaaacaagagctcttacaatcacaaca
agagcttttacatggtcagcagaagcttacctctcttatactttatcagctgtaacttta
tgtggttctatcacagcattattttcttggtacaataatactgcatatccaagtgaattc
tatggacctactggagcagaagcctctcaagcacaaagctttacattcctcatacgagat
tggaaattgggtataaagataacttcatccgaaggtcctacagctcttggtaagtatctc
atgagatctccaacaggtgaaataatatttggtggtgaaagtatgagattctggtctatg
caagcacattggcttgaagctctcagaacatcatttggactggacttatctaagattcaa
tctgatattcaaacttggcaagaaagaagagctgcagagtatatgacccatgcaccacta
gggagcttaaattctgttggtggtgttgctactgaaattaactctgtaaactatgtatct
cctagatcttggttaacatcatctcactggttcttagcattcttcattttagttggtcat
tggtggcatggagcaagagcaagagcttcagcattatctagtgaaacaggcttatctcga
gtatatgaacctgttctttatatgcgacctattgattag
>Isy26_psbC
atgcctctcatttattctttaaaacgacgaactctagttggatccaggtatgcttggtgg
tcaggtaatgcaagattcattgagcttagcggtaaattcctaggtgctcatcttgtgcat
acagctcttattctagtttgggctggaacaatgtctttgtttgaattatctcacttcatc
ccagaaaaacctctatatgagcaaggctttattcttcttcctcacttgtttgaattgggt
ggcaatctatattcacacttcataataagcatcctacatctcatctctgcaggtatccta

gctcttggaggtatttatcatgctatagttggtgctgaaagattagaagaaacaagcttc
agtgctctatttgcttatgggttacaagatagattccgtattacagctatacttggatca
catttacttactctaggaattggagcagccttactctttgcaaaagcagtctatttaggt
ggtctttatgatacatgggcttgtggtggtggagacatgagattgattgaaagggtagag
cttggtttaaatccttatctactagctcagtacttacttcgtgctccatttgggtcatca
ggatggataattagcatcaataacatggaagatctaattggaggtcattattgggtctca
ttttatctaattctaggcagctgttggcatattcaaacaagagctcttacaatcacaaca
agagcttttacatggtcagcagaagcttacctctcttatactttatcagctgtaacttta
tgtggttctatcacagcattattttcttggtacaataatactgcatatccaagtgaattc
tatggacctactggagcagaagcctctcaagcacaaagctttacattcctcatacgagat
tggaaattgggtataaagataacttcatccgaaggtcctacagctcttggtaagtatctc
atgagatctccaacaggtgaaataatatttggtggtgaaagtatgagattctggtctatg
caagcacattggcttgaagctctcagaacatcatttggactggacttatctaagattcaa
tctgatattcaaacttggcaagaaagaagagctgcagagtacatgacccatgcaccacta
gggagcttaaattctgttggtggtgttgctactgaaattaactctgtaaactatgtatct
cctagatcttggttaacatcatctcactggttcttagcattcttcattttagttggtcat
tggtggcatggagcaagagcaagagcttcagcattatctagtgaaacaggcttatctcga
gtatatgaacctgttctttatatgcgacctattgattag
>Isy27_psbC
atgcctctcatttattctttaaaacgacgaactctagttggatccaggtatgcttggtgg
tcaggtaatgcaagattcattgagcttagcggtaaattcctaggtgctcatcttgtgcat
acagctcttattctagtttgggctggaacaatgtctttgtttgaattatctcacttcatc
ccagaaaaacctctatatgagcaaggctttattcttcttcctcacttgtttgaattgggt
ggcaatctatattcacacttcataataagcatcctacatctcatctctgcaggtatccta
gctcttggaggtatttatcatgctatagttggtgctgaaagattagaagaaacaagcttc
agtgctctatttgcttatgggttacaagatagattccgtattactgctatacttggatca
catttacttactctaggaattggagcagccttactctttgcaaaagcagtctatttaggt
ggtctttatgatacatgggcttgtggtggtggagacatgagattgattgaaagggtagag
cttggtttaaatccttatctactagctcagtacttacttcgtgctccatttgggtcatca
ggatggataattagcatcaataacatggaagatctcattggaggtcattattgggtctca
ttttatctaattctaggcagctgttggcatattcaaacaagagctcttacaattacaaca
agagcttttacatggtcagcagaagcttacctctcttatactttatcagctgtaacttta
tgtggttctatcacaagtttgttttcttggtacaataatactgcatatccaagtgaattc
tatggacctactggagcagaagcctctcaagcacaaagctttacattccttatacgagat
tggaaattgggtataaagataagttcatccgaaggtcctacagctcttggtaagtatctc
atgagatctccaacaggtgaaataatatttggtggtgaaagtatgagattctggtctatg
caagcacattggcttgaagctctcagaacatcatttggactggacttatctaagattcaa
tctgatattcaaacttggcaagaaagaagagctgcagagtatatgacccatgcaccacta
gggagcttaaattctgttggtggtgttgctactgaaattaactctgtaaactatgtatct
cctagatcttggttaacatcatctcactggttcttagcattcttcattttagttggtcat
tggtggcatggagcaagagcaagagcttcagcattatctagtgaaacaggcttatctcga
gtatatgaacctgttctttatatgcgacctattgattag
>Isy4_psbC
atgcctctcatttattctttaaaacgacgaactctagttggatccaggtatgcttggtgg
tcaggtaatgcaagattcattgagcttagcggtaaattcctaggtgctcatcttgtgcat
acagctcttattctagtttgggctggaacaatgtctttgtttgaattatctcacttcatc
ccagaaaaacctctatatgagcaaggctttattcttcttcctcacttgtttgaattgggt
ggcactttatattcacacttcataataagcatcctacatctcatctctgcaggtatccta
gctcttggaggtatttatcatgctatagttggtgctgaaagattagaagaaacaagcttc
agtgctctatttgcttatgggttacaagatagattccgtattactgctatacttggatca
catttacttactctaggaattggagcagccttactctttgcaaaagcagtctatttaggt
ggtctttatgatacatgggcttgtggtggtggagacatgagattgattgaaagggtagag
cttggtttaaatccttatctactagctcagtacttacttcgtgctccatttgggtcatca
ggatggataattagcatcaataacatggaagatctcattggaggtcattattgggtctca
ttttatctaattctaggcagctgttggcatattcaaacaagagctcttacaattacaaca
agagcttttacatggtcagcagaagcttacctctcttatactttatcagctgtaacttta
tgtggttctatcacagcattattttcttggttcaataatactgcatatccaagtgaattc

tatggacctactggggcagaagcctctcaagcacaaagctttacattcctcatacgagat
tggaaattgggtataaagataacttcatccgaaggtcctacagctcttggtaagtatctc
atgagatctccaacaggtgaaataatatttggtggtgaaagtatgagattctggtctatg
caagcacattggcttgaagctctcagaacatcatttggactggacttatctaagattcaa
tctgatattcaaacttggcaagaaagaagagctgcagagtatatgacccatgcaccacta
gggagcttaaattctgttggtggtgttgctactgaaattaactctgtaaactatgtatct
cctagatcttggttaacatcatctcactggttcttagcattctttattttagttggtcat
tggtggcatggagcaagagcaagagcttcagcattatctagtgaaacaggcttatctcga
gtatatgaacctgttctttatatgcgacctattgattag
>Isy7_psbC
atgcctctcatttattctttaaaacgacgaactctagttggatccaggtatgcctggtgg
tcaggtaatgcaagatttattgagctaagcggtaaattcctaggtgctcatcttgtgcat
acagctcttattctagtttgggctggaacaatgtctttgtttgaattatctcacttcatc
ccagaaaaacctctatatgagcaaggttttattcttcttcctcaccttttttgaattaggt
ggtactttatactcacacttcataataagcatcctacatctcatctctgcaggtatccta
gctcttggaggtatttatcatgctatagttggtgctgaaagactagaagaaacaagattc
agtgctctatttgcttatggcttacaagatagattccgtattactgctatactaggatca
catttacttactctaggaattggagcagccttactctttgcaaaagcagtctatttaggt
ggtctttatgatacatgggcttgtggtggtggagacatgagattgattgaaagggtagag
cttggattaaatccttatctactagctcagtatttacttcgtgctccgtttgggtcatca
gggtggataattagtatcaataacatggaagatctcattggaggtcattattgggtctca
ttttatctaattctaggcagctgttggcatattcaaacaagagctcttacaattacaaca
agagcttttacatggtcagctgaagcttacctctcttacactttatcagccgtaacttta
tgtgcttctaccacagccttattttcctggtttaataatactgcttatccaagtgaattc
tatggacctactggggcagaagcctctcaagcacaaagctttacattcctcatacgagat
tggaaattgggtataaagataacttcatccgaaggtcctacagctcttggtaagtatctc
atgagatctccaacaggtgaaataatatttggtggtgaaagtatgagattttgggctatg
caagcacattggcttgaagctctcagaacatcatttggactggacttatctaagattcaa
tctgatattcaaacttggcaagaaagaagagctgcagagtatatgacccatgcaccacta
ggaagcttaaattctgttggtggtgttgctactgaaattaactctgtaaactatgtatct
cctagatcttggttaacatcatctcactggttcttagcattctttattttagttggtcat
tggtggcatggagcaagagcaagagcttcagcattatctagtgaaacaggcttatcgcgt
atatatgaacctgttctttacatgagacccattgattag
>Isy8_psbC
atgcctctcatttattctttaaaacgacgaactctagttggatccaggtatgcttggtgg
tcaggtaatgcaagattcattgagcttagcggtaaattcctaggtgctcatcttgtgcat
acagctcttattctagtttgggctggaacaatgtctttgtttgaattatctcacttcatc
ccagaaaaacctctatatgagcaaggctttattcttcttcctcacttgtttgaattgggt
ggcaatctatattcacacttcataataagcatcctacatctcatctctgcaggtatccta
gctcttggaggtatttatcatgctatagttggtgctgaaagattagaagaaacaagcttc
agtgctctatttgcttatgggttacaagatagattccgtattactgctatacttggatca
catttacttactctaggaattggagcagccttactctttgcaaaagcagtctatttaggt
ggtctttatgatacatgggcttgtggtggtggagacatgagattgattgaaagggtagag
cttggtttaaatccttatctactagctcagtacttacttcgtgctccatttgggtcatca
ggatggataattagcatcaataacatggaagatctcattggaggtcattattgggtctca
ttttatctaattctaggcagctgttggcatattcaaacaagagctcttacaatcacaaca
agagcttttacatggtcagcagaagcttacctctcttatactttatcagctgtaacttta
tgtggttctatcacagcattattttcttggtacaataatactgcatatccaagtgaattc
tatggacctactggagcagaagcctctcaagcacaaagctttacattcctcatacgagat
tggaaattgggtataaagataacttcatccgaaggtcctacagctcttggtaagtatctc
atgagatctccaacaggtgaaataatatttggtggtgaaagtatgagattctggtctatg
caagcacattggcttgaagctctcagaacatcatttggactggacttatctaagattcaa
tctgatattcaaacttggcaagaaagaagagctgcagagtatatgacccatgcaccacta
gggagcttaaattctgttggtggtgttgctactgaaattaactctgtaaactatgtatct
cctagatcttggttaacatcatctcactggttcttagcattcttcattttagttggtcat
tggtggcatggagcaagagcaagagcttcagcattatctagtgaaacaggcttatctcga
gtatatgaacctgttctttatatgcgacctattgattag

>KrA1_psbC
atgcctctcatttattctttaaaacgacgaactctagttggatccaggtatgcttggtgg
tcaggtaatgcaagattcattgagcttagcggtaaattcctaggtgctcatcttgtgcat
acagctcttattctagtttgggctggaacaatgtctttgtttgaattatctcacttcatc
ccagaaaaacctctatatgagcaaggctttattcttcttcctcacttgtttgaattgggt
ggcactttatattcacacttcataataagcatcctacatctcatctctgcaggtatccta
gctcttggaggtatttatcatgctatagttggtgctgaaagattagaagaaacaagcttc
agtgctctatttgcttatgggttacaagatagattccgtattactgctatacttggatca
catttacttactctaggaattggagcagccttactctttgcaaaagcagtctatttaggt
ggtctttatgatacatgggcttgtggtggtggagacatgagattgattgaaagggtagag
cttggtttaaatccttatctactagctcagtacttacttcgtgctccatttgggtcatca
ggatggataattagcatcaataacatggaagatctcattggaggtcattattgggtctca
ttttatctaattctaggcagctgttggcatattcaaacaagagctcttacaattacaaca
agagcttttacatggtcagcagaagcttacctctcttatactttatcagctgtaacttta
tgtggttctatcacagcattattttcttggttcaataatactgcatatccaagtgaattc
tatggacctactggggcagaagcctctcaagcacaaagctttacattcctcatacgagat
tggaaattgggtataaagataacttcatccgaaggtcctacagctcttggtaagtatctc
atgagatctccaacaggtgaaataatatttggtggtgaaagtatgagattctggtctatg
caagcacattggcttgaagctctcagaacatcatttggactggacttatctaagattcaa
tctgatattcaaacttggcaagaaagaagagctgcagagtatatgacccatgcaccacta
gggagcttaaattctgttggtggtgttgctactgaaattaactctgtaaactatgtatct
cctagatcttggttaacatcatctcactggttcttagcattctttattttagttggtcat
tggtggcatggagcaagagcaagagcttcagcattatctagtgaaacaggcttatctcga
gtatatgaacctgttctttatatgcgacctattgattag
>KrA10_psbC
atgcctctcatttattctttaaaacgacgaactctagttggatccaggtatgcttggtgg
tcaggtaatgcaagattcattgagcttagcggtaaattcctaggtgctcatcttgtgcat
acagctcttattctagtttgggctggaacaatgtctttgtttgaattatctcacttcatc
ccagaaaaacctctatatgagcaaggctttattcttcttcctcacttgtttgaattgggt
ggcactttatattcacacttcataataagcatcctacatctcatctctgcaggtatccta
gctcttggaggtatttatcatgctatagttggtgctgaaagattagaagaaacaagcttc
agtgctctatttgcttatgggttacaagatagattccgtattactgctatacttggatca
catttacttactctaggaattggagcagccttactctttgcaaaagcagtctatttaggt
ggtctttatgatacatgggcttgtggtggtggagacatgagattgattgaaagggtagag
cttggtttaaatccttatctactagctcagtacttacttcgtgctccatttgggtcatca
ggatggataattagcatcaataacatggaagatctcattggaggtcattattgggtctca
ttttatctaattctaggcagctgttggcatattcaaacaagagctcttacaattacaaca
agagcttttacatggtcagcagaagcttacctctcttatactttatcagctgtaacttta
tgtggttctatcacagcattattttcttggttcaataatactgcatatccaagtgaattc
tatggacctactggggcagaagcctctcaagcacaaagctttacattccttatacgagat
tggaaattgggtataaagataacttcatccgaaggtcctacagctcttggtaagtatctc
atgagatctccaacaggtgaaataatatttggtggtgaaagtatgagattctggtctatg
caagcacattggcttgaagctctcagaacatcatttggactggacttatctaagattcaa
tctgatattcaaacttggcaagaaagaagagctgcagagtatatgacccatgcaccacta
gggagcttaaattctgttggtggtgttgctactgaaattaactctgtaaactatgtatct
cctagatcttggttaacatcatctcactggttcttagcattctttattttagttggtcat
tggtggcatggagcaagagcaagagcttcagcattatctagtgaaacaggcttatctcga
gtatatgaacctgttctttatatgcgacctattgattag
>KrA11_psbC
atgcctctcatttattctttaaaacgacgaactctagttggatccaggtatgcttggtgg
tcaggtaatgcaagattcattgagcttagcggtaaattcctaggtgctcatcttgtgcat
acagctcttattctagtttgggctggaacaatgtctttgtttgaattatctcacttcatc
ccagaaaaacctctatatgagcaaggctttattcttcttcctcacttgtttgaactggga
ggcaatctatattcacacttcataataagcatcctacatctcatctctgcaggtatccta
gctcttggaggtatttatcatgctatagttggtgctgaaagattagaagaaacaagcttc
agtgctctatttgcttatgggttacaagatagattccgtattactgctatacttggttca
catttacttactctaggaattggagcagccttactctttgcaaaagcagtctatttaggt

ggtctttatgatacatgggcttgtggtggtggagacatgagattgattgaaagggtagag
cttggtttaaatccttatctactagctcagtacttacttcgtgctccatttgggtcatca
ggatggataattagcatcaataacatggaagatctcattggaggtcattattgggtctca
ttttatctaattctaggcagctgttggcatattcaaacaagagctcttacaattacaaca
agagcttttacatggtcagcagaagcttacctctcttatactttatcagctgtaacttta
tgtggttctatcacagcattattttcttggttcaataatactgcatatccaagtgaattc
tatggacctactggggcagaagcctctcaagcacaaagctttacattcctcatacgagat
tggaaattgggtataaagataacttcatccgaaggtcctacagctcttggtaagtatctc
atgagatctccaacaggtgaaataatatttggtggtgaaagtatgagattctggtctatg
caagcacattggcttgaagctctcagaacatcatttggactggacttatctaagattcaa
tctgatattcaaacttggcaagaaagaagagctgcagagtatatgacccatgcaccacta
gggagcttaaattctgttggtggtgttgctactgaaattaactctgtaaactatgtatct
cctagatcttggttaacatcatctcactggttcttagcattctttattttagttggtcat
tggtggcatggagcaagagcaagagcttcagcattatctagtgaaacaggcttatctcga
gtatatgaacctgttctttatatgcgacctattgattag
>KrA12_psbC
atgcctctcatttattctttaaaacgacgaactctagttggatccaggtatgcttggtgg
tcaggtaatgcaagattcattgagctaagtggtaaattcctaggtgctcatcttgttcat
acagctcttattctagtttgggcaggaagaatgtctttgtttgaattatctcacttcatt
ccagaaaaacctctatatgagcaaggctttattcttctacctcatttgtttgaactggga
ggcaatctatattcacacttcataataagcatcctacatctcatctctgcaggtatctta
gctcttggaggtatttatcatgctatagttggtggtgaaagactagaagaaacaagctat
agctccctatttgcttatgggttacaagatagattccgtattacagctatacttggttca
catttacttactctaggaattggagcaggcttactctttgcaaaagcagtctatttaggt
ggtctttatgatacatgggcttgtggtggtggagacatgagattgattgaaagggtagag
cttggtttaaatccttatctactagctcagtacttacttcgtgctccatttgggtcatca
ggatggataattagcatcaataacatggaagatctaattggaggccattattgggtctca
ttttatctaattctaggtagctgttggcatattcaaacaagagctcttacaatcacaaca
agagcttttacatggtcagcagaagcttacctctcttacactctatcagctgtaacttta
tgtggttctatcacaagtttgttttcttggtacaataatactgcatatccaagtgaattc
tatggacctactggagcagaagcctctcaagcacaaagctttacattccttatacgagat
tggaaattgggtataaagataagttcatccgaaggtcctacagctcttggtaagtatctc
atgagatctccaacaggtgaaataatatttggtggtgaaagtatgagattctggtctatg
caagcacattggcttgaagctctcagaacatcctttggattagacttatctaagattcaa
tctgatattcaaacttggcaagaaagaagagctgcagagtacatgacccatgcaccacta
gggagcttaaattctgttggtggtgttgctactgaaattaactctgtaaactatgtatct
cctagatcttggttaacctcctctcactggttcttagcattctttattttagttggtcat
tggtggcatggagcaagagcaagagcttcagcattatctagtgaaacaggcttatctcga
gtatatgaacctgtgctttatatgcgacctattgattag
>KrA13_psbC
atgcctctcatttattctttaaaacgacgaactctagttggatccaagtatgcttggtgg
tcaggtaatgcaagattcattgagcttagcggtaaattcctaggtgctcatcttgtgcat
acagctcttattctagtttgggctggaacaatgtctttgtttgaattatctcacttcatc
ccagaaaaacctctatatgagcaaggctttattcttcttcctcacttgtttgaattgggt
ggcactttatattcacacttcataataagcatcctacatctcatctctgcaggtatccta
gctcttggaggtatttatcatgctatagttggtggtgaaagactagaagaaacaagctat
agctccctatttgcttatgggttacaagatagattccgtattacagctatacttggttca
catttacttactctaggaattggagcaggcttactctttgcaaaagcagtctatttaggt
ggtctttatgatacatgggcttgtggtggtggagacatgagattgattgaaagggtagag
cttggtttaaatccttatctactagctcagtacttacttcgtgctccatttgggtcatca
ggatggataattagcatcaataacatggaagatctcattggaggtcattattgggtctca
ttttatctaattctaggcagctgttggcatattcaaacaagagctcttacaattacaaca
agagcttttacatggtcagcagaagcttacctctcttatactttatcagctgtaacttta
tgtggttctatcacagcattattttcttggttcaataatactgcatatccaagtgaattc
tatggacctactggggcagaagcctctcaagcacaaagctttacattcctcatacgagat
tggaaattgggtataaagataacttcatccgaaggtcctacagctcttggtaagtatctc
atgagatctccaacaggtgaaataatatttggtggtgaaagtatgagattctggtctatg

caagcacattggcttgaagctctcagaacatcatttggactggacttatctaagattcaa
tctgatattcaaacttggcaagaaagaagagctgcagagtatatgacccatgcaccacta
gggagcttaaattctgttggtggtgttgctactgaaattaactctgtaaactatgtatct
cctagatcttggttaacctcctctcattggttcttggccttctttattttagttggtcat
tggtggcatggagcaagagcaagagcttcagcattatctagtgaaacaggcttatctcga
gtatatgaacctgttctttatatgcgacctattgattag
>KrA14_psbC
atgcctcttcttcattctttaaaacgacgaactctagttggatccaggtatgcttggtgg
tcaggtaatgcaagattcattgagcttagcggtaaattcctaggtgctcatcttgtgcat
acagctcttattctagtttgggctggaacaatgtctttgtttgaattatctcacttcatt
ccagaaaaacctctatatgagcaaggctttattcttcttcctcacttgtttgaattgggt
ggcactttatattcacacttcataataagcatcctacatctcatctctgcaggtatccta
gctcttggaggtatttatcatgctatagttggtgctgaaagattagaagaaacaagcttc
agtgctctatttgcttatgggttacaagatagattccgtattactgctatacttggatca
catttacttactctaggaattggagcagccttactctttgcaaaagcagtctatttaggt
ggtctttatgatacatgggcttgtggtggtggagacatgagattgattgaaagggtagag
cttggtttaaatccttatctactagctcagtacttacttcgtgctccatttgggtcatca
ggatggataattagcatcaataacatggaagatctcattggaggtcattattgggtctca
ttttatctaattctaggtagctgttggcatattcaaacaagagctcttacaattacaaca
agagcttttacatggtcagcagaagcttacctctcttatactttatcagctgtaacttta
tgtggttctatcacagcattattttcttggttcaataatactgcatatccaagtgaattc
tatggacctactggggcagaagcctctcaagcacaaagctttacattcctcatacgagat
tggaaattgggtataaagataacttcatccgaaggtcctacagctcttggtaagtatctc
atgagatctccaacaggtgaaataatatttggtggtgaaagtatgagattctggtctatg
caagcacattggcttgaagctctcagaacatcatttggactggacttatctaagattcaa
tctgatattcaaacttggcaagaaagaagagctgcagagtatatgacccatgcaccacta
gggagcttaaattctgttggtggtgttgctactgaaattaactctgtaaactatgtatct
cctagatcttggttaacatcatctcactggttcttagcattctttattttagttggtcat
tggtggcatggagcaagagcaagagctgcagcattgtctagtgaaacaggtttatctcga
gtatatgaacctgtgctttatatgcgacctattgattag
>KrA15_psbC
atgcctctcatttattctttaaaacgacgaactctagttggatccaggtatgcttggtgg
tcaggtaatgcaagattcattgagcttagcggtaaattcctaggtgctcatcttgtgcat
acagctcttattctagtttgggctggaacaatgtctttgtttgaattatctcacttcatc
ccagaaaaacctctatatgagcaaggctttattcttctacctcatttgtttgaactggga
ggcaatctatattcacacttcataataagcatcctacatctcatctctgcaggtatccta
gctcttggaggtatttatcatgctatagttggtgctgaaagactagaagaaacaagctat
agctccctatttgcttatgggttacaagatagattccgtattacagctatacttggatca
catttacttactctaggaattggagcagccttactctttgcaaaagcagtctatttaggt
ggtctttatgatacatgggcttgtggtggtggagacatgagattgattgaaagggtagag
cttggtttaaatccttatctactagctcagtacttacttcgtgctccatttgggtcatca
ggatggataattagcatcaataacatggaagatctaattggaggccattattgggtctca
ttttatctaattctaggtagctgttggcatattcaaacaagagctcttacaatcacaaca
agagcttttacatggtcagcagaagcttacctctcttatactttatcagctgtaacttta
tgtggttctatcacagcattattttcttggttcaataatactgcatatccaagtgaattc
tatggacctactggggcagaagcctctcaagcacaaagctttacattcctcatacgagat
tggaaattgggtataaagataacttcatccgaaggtcctacagctcttggtaagtatctc
atgagatctccaacaggtgaaataatatttggtggtgaaagtatgagattctggtctatg
caagcacattggcttgaagctctcagaacatcatttggactggacttatctaagattcaa
tctgatattcaaacttggcaagaaagaagagctgcagagtatatgacccatgcaccacta
gggagcttaaattctgttggtggtgttgctactgaaattaactctgtaaactatgtatct
cctagatcttggttaacatcatctcactggttcttagcattctttattttagttggtcat
tggtggcatggagcaagagcaagagcttcagcattatctagtgaaacaggcttatctcga
gtatatgaacctgttctttatatgcgacctattgattag
>KrA2_psbC
atgcctctcatttattctttaaaacgacgaactctagttggatccaggtatgcttggtgg
tcaggtaatgcaagattcattgagcttagcggtaaattcctaggtgctcatcttgtgcat

acagctcttattctagtttgggctggaacaatgtctttgtttgaattatctcacttcatc
ccagaaaaacctctatatgagcaaggctttattcttcttcctcacttgtttgaattgggt
ggcactttatattcacacttcataataagcatcctacatctcatctctgcaggtatccta
gctcttggaggtatttatcatgctatagttggtgctgaaagattagaagaaacaagcttc
agtgctctatttgcttatgggttacaagatagattccgtattactgctatacttggatca
catttacttactctaggaattggagcagccttactctttgcaaaagcagtctatttaggt
ggtctttatgatacatgggcttgtggtggtggagacatgagattgattgaaagggtagag
cttggtttaaatccttatctactagctcagtacttacttcgtgctccatttgggtcatca
ggatggataattagcatcaataacatggaagatctcattggaggtcattattgggtctca
ttttatctaattctaggcagctgttggcatattcaaacaagagctcttacaattacaaca
agagcttttacatggtcagcagaagcttacctctcttatactttatcagctgtaacttta
tgtggttctatcacagcattattttcttggttcaataatactgcatatccaagtgaattc
tatggacctactggggcagaagcctctcaagcacaaagctttacattcctcatacgagat
tggaaattgggtataaagataacttcatccgaaggtcctacagctcttggtaagtatctc
atgagatctccaacaggtgaaataatatttggtggtgaaagtatgagattctggtctatg
caagcacattggcttgaagctctcagaacatcatttggactggacttatctaagattcaa
tctgatattcaaacttggcaagaaagaagagctgcagagtatatgacccatgcaccacta
gggagcttaaattctgttggtggtgttgctactgaaattaactctgtaaactatgtatct
cctagatcttggttaacatcatctcactggttcttagcattctttattttagttggtcat
tggtggcatggagcaagagcaagagcttcagcattatctagtgaaacaggcttatctcga
gtatatgaacctgttctttatatgcgacctattgattag
>KrA3_psbC
atgcctctcatttattctttaaaacgacgaactctagttggatccaggtatgcttggtgg
tcaggtaatgcaagattcattgagcttagcggtaaattcctaggtgctcatcttgtgcat
acagctcttattctagtttgggctggaacaatgtctttgtttgaattatctcacttcatc
ccagaaaaacctctatatgagcaaggctttattcttcttcctcacttgtttgaattgggt
ggcactttatattcacacttcataataagcatcctacatctcatctctgcaggtatgcta
gctcttggaggtatttatcatgctatagttggtggtgaaagattagaagaaacaagcttc
agtgctctatttgcttatgggttacaagatagattccgtattactgctatacttggatca
catttacttactctaggaattggagcagccttactctttgcaaaagcagtctatttaggt
ggtctttatgatacatgggcttgtggtggtggagacatgagattgattgaaagggtagag
cttggtttaaatccttatctactagctcagtacttacttcgtgctccatttgggtcatca
ggatggataattagcatcaataacatggaagatctaattggaggtcattattgggtctca
ttttatctaattctaggcagctgttggcatattcaaacaagagctcttacaattacaaca
agagcttttacatggtcagcagaagcttacctctcttatactttatcagctgtaacttta
tgtggttctatcacagcattattttcttggttcaataatactgcatatccaagtgaattc
tatggacctactggggcagaagcctctcaagcacaaagctttacattcctcatacgagat
tggaaattgggtataaagataacttcatccgaaggtcctacagctcttggtaagtatctc
atgagatctccaacaggtgaaataatatttggtggtgaaagtatgagattctggtctatg
caagcacattggcttgaagctctcagaacatcatttggactggacttatctaagattcaa
tctgatattcaaacttggcaagaaagaagagctgcagagtatatgacccatgcaccacta
gggagcttaaattctgttggtggtgttgctactgaaattaactctgtaaactatgtatct
cctagatcttggttaacatcatctcactggttcttagcattctttattttagttggtcat
tggtggcatggagcaagagcaagagcttcagcattatctagtgaaacaggcttatctcga
gtatatgaacctgttctttatatgcgacctattgattag
>KrA4_psbC
atgcctctcatttattctttaaaacgacgaactctagttggatccaggtatgcttggtgg
tcaggtaatgcaagattcattgagcttagcggtaaattcctaggtgctcatcttgtgcat
acagctcttattctagtttgggctggaacaatgtctttgtttgaattatctcacttcatc
ccagaaaaacctctatatgagcaaggctttattcttcttcctcacttgtttgaattgggt
ggcactttatattcacacttcataataagcatcctacatctcatctctgcaggtatccta
gctcttggaggtatttatcatgctatagttggtgctgaaagattagaagaaacaagcttc
agtgctctatttgcttatgggttacaagatagattccgtattactgctatacttggatca
catttacttactctaggaattggagcagccttactctttgcaaaagcagtctatttaggt
ggtctttatgatacttgggcttgtggtggtggagacatgagattgattgaaagagtagag
cttggtttaaatccttatctactagctcaatacttacttcgtgctccatttggttcatca
ggatggataattagcatcaataacatggaagatctaattggaggccattattgggtctca

ttttatctaattctaggtagctgttggcatattcaaacaagagctcttacaatcacaaca
agagcttttacatggtcagcagaagcttacctctcttacactctatcagctgtaacttta
tgtggttctatcacaagtttgttttcttggtacaataatactgcatatccaagtgaattc
tatggacctactggagcagaagcctctcaagcacaaagctttacattccttatacgagat
tggaaattgggtataaagataagttcatccgaaggtcctacagctcttggtaagtatctc
atgagatctccaacaggtgaaataatatttggtggtgaaagtatgagattctggtctatg
caagcacattggcttgaagctctcagaacatcatttggattagacttatctaagattcaa
tctgatattcaaacttggcaagaaagaagagctgcagagtacatgacccatgcaccacta
gggagcttaaattctgttggtggtgttgctactgaaattaactctgtaaactatgtatct
cctagatcttggttaacctcatctcactggttcttagcattctttattttagttggtcat
tggtggcatggagcaagagcaagagcttcagcattatctagtgaaacaggcttatctcga
gtatatgaacctgttctttatatgcgacctattgattag
>KrA5_psbC
atgcctctcatttattctttaaaacgacgaactctagttggatccaagtatgcttggtgg
tcaggtaatgcaagattcattgagctaagtggtaaattcctaggtgctcatcttgtgcat
acagctcttattctagtttgggctggaacaatgtctttgtttgaattatctcacttcatc
ccagaaaaacctctatatgagcaaggctttattcttcttcctcacttgtttgaattgggt
ggcactttatattcacacttcataataagcatcctacatctcatctctgcaggtatccta
gctcttggaggtatttatcatgctatagttggtgctgaaagattagaagaaacaagctat
agctccctatttgcttatgggttacaagatagattccgtattactgctatacttggatca
catttacttactctaggaattggagcagccttactctttgcaaaagcagtctatttaggt
ggtctttatgatacatgggcttgtggtggtggagacatgagattgattgaaagggtagag
cttggtttaaatccttatctactagctcagtacttacttcgtgctccatttgggtcatca
ggatggataattagcatcaataacatggaagatctcattggaggtcattattgggtctca
ttttatctaattctaggcagctgttggcatattcaaacaagagctcttacaattacaaca
agagcttttacatggtcagcagaagcttacctctcttatactttatcagctgtaacttta
tgtggttctatcacaagtttgttttcttggtacaataatactgcatatccaagtgaattc
tatggacctactggggcagaagcctctcaagcacaaagctttacattcctcatacgagat
tggaaattgggtataaagataacttcatccgaaggtcctacagctcttggtaagtatctc
atgagatctccaacaggtgaaataatatttggtggtgaaagtatgagattctggtctatg
caagcacattggcttgaagctctcagaacatcatttggactggacttatctaagattcaa
tctgatattcaaacttggcaagaaagaagagctgcagagtacatgacccatgcaccacta
gggagcttaaattctgttggtggtgttgctactgaaattaactctgtaaactatgtatct
cctagatcttggttaacatcatctcactggttcttagcattctttattttagttggtcat
tggtggcatggagcaagagcaagagcttcagcattatctagtgaaacaggcttatctcga
gtatatgaacctgttctttatatgcgacctattgattag
>KrA6_psbC
atgcctcttcttcattctttaaaacgacgaactctagttggatccaggtatgcttggtgg
tcaggtaatgcaagattcattgagcttagcggtaaattcctaggtgctcatcttgtgcat
acagctcttattctagtttgggctggaacaatgtctttgtttgaattatctcacttcatc
ccagaaaaacctctatatgagcaaggctttattcttcttcctcacttgtttgaattgggt
ggcactttatattcacacttcataataagcatcctacatctcatctctgcaggtatccta
gctcttggaggtatttatcatgctatagttggtggtgaaagactagaagaaacaagctat
agctccctatttgcttatgggttacaagatagattccgtattacagctatacttggttca
catttacttactctaggaattggagcaggcttactctttgcaaaagcagtctatttaggt
ggtctttatgatacatgggcttgtggtggtggagacatgagattgattgaaagggtagag
cttggtttaaatccttatctactagctcagtacttacttcgtgctccatttgggtcatca
ggatggataattagcatcaataacatggaagatctcattggaggtcattattgggtctca
ttttatctaattctaggcagctgttggcatattcaaacaagagctcttacaattacaaca
agagcttttacatggtcagcagaagcttacctctcttatactttatcagctgtaacttta
tgtggttctatcacagcattattttcttggttcaataatactgcatatccaagtgaattc
tatggacctactggggcagaagcctctcaagcacaaagctttacattcctcatacgagat
tggaaattgggtataaagataacttcatccgaaggtcctacagctcttggtaagtatctc
atgagatctccaacaggtgaaataatatttggtggtgaaagtatgagattctggtctatg
caagcacattggcttgaagctctcagaacatcatttggactggacttatctaagattcaa
tctgatattcaaacttggcaagaaagaagagctgcagagtatatgacccatgcaccacta
gggagcttaaattctgttggtggtgttgctactgaaattaactctgtaaactatgtatct

cctagatcttggttaacatcatctcactggttcttagcattctttattttagttggtcat
tggtggcatggagcaagagcaagagcttcagcattatctagtgaaacaggcttatctcga
gtatatgaacctgttctttatatgcgacctattgattag
>KrA7_psbC
atgcctctcatttattctttaaaacgacgaactctagttggatccaggtatgcttggtgg
tcaggtaatgcaagattcattgagcttagcggtaaattcctaggtgctcatcttgtgcat
acagctcttattctagtttgggctggaacaatgtctttgtttgaattatctcacttcatc
ccagaaaaacctctatatgagcaaggctttattcttcttcctcacttgtttgaattgggt
ggcactttatattcacacttcataataagcatcctacatctcatctctgcaggtatccta
gctcttggaggtatttatcatgctatagttggtgctgaaagactagaagaaacaagcttc
agtgctctatttgcttatgggttacaagatagattccgtattactgctatacttggatca
catttacttactctaggaattggagcagccttactctttgcaaaagcagtctatttaggt
ggtctttatgatacatgggcttgtggtggtggagacatgagattgattgaaagggtagag
cttggtttaaatccttatctactagctcaatacttacttcgtgctccatttgggtcatca
ggatggataattagcatcaataacatggaagatctcattggaggtcattattgggtctca
ttttatctaattctaggcagctgttggcatattcaaacaagagctcttacaattacaaca
agagcttttacatggtcagcagaagcttacctctcttatactttatcagctgtaacttta
tgtggttctatcacaagtttgttttcttggtacaataatactgcatatccaagtgaattc
tatggacctactggggcagaagcctctcaagcacaaagctttacattcctcatacgagat
tggaaattgggtataaagataacttcatccgaaggtcctacagctcttggtaagtatctc
atgagatctccaacaggtgaaataatatttggtggtgaaagtatgagattctggtctatg
caagcacattggcttgaagctctcagaacatcatttggactggacttatctaagattcaa
tctgatattcaaacttggcaagaaagaagagctgcagagtatatgacccatgcaccacta
gggagcttaaattctgttggtggtgttgctactgaaattaactctgtaaactatgtatct
cctagatcttggttaacatcatctcactggttcttagcattctttattttagttggtcat
tggtggcatggagcaagagcaagagcttcagcattatctagtgaaacaggtttatctcga
gtatatgaacctgttctttatatgcgacctattgattag
>KrA8_psbC
atgcctctcatttattctttaaaacgacgaactctagttggatccaagtatgcttggtgg
tcaggtaatgcaagattcattgagcttagcggtaaattcctaggtgctcatcttgtgcat
acagctcttattctagtttgggctggaacaatgtctttgtttgaattatctcacttcatc
ccagaaaaacctctatatgagcaaggctttattcttcttcctcacttgtttgaattgggt
ggcactttatattcacacttcataataagcatcctacatctcatctctgcaggtatccta
gctcttggaggtatttatcatgctatagttggtgctgaaagattagaagaaacaagcttc
agtgctctatttgcttatgggttacaagatagattccgtattactgctatacttggatca
catttacttactctaggaattggagcagccttactctttgcaaaagcagtctatttaggt
ggtctttatgatacttgggcttgtggtggtggagacatgagattgattgaaagagtagag
cttggtttaaatccttatctactagctcaatacttacttcgtgctccatttgggtcatca
ggatggataattagcatcaataacatggaagatctcattggaggtcattattgggtctca
ttttatctaattctaggcagctgttggcatattcaaacaagagctcttacaattacaaca
agagcttttacatggtcagcagaagcttacctctcttacactctatcagctgtaacttta
tgtggttctatcacagcattattttcttggttcaataatactgcatatccaagtgaattc
tatggacctactggggcagaagcctctcaagcacaaagctttacattccttatacgagat
tggaaattgggtataaagataagttcatccgaaggtcctacagctcttggtaagtatctc
atgagatctccaacaggtgaaataatatttggtggtgaaagtatgagattctggtctatg
caagcacattggcttgaagctctcagaacatcctttggattagacttatctaagattcaa
tctgatattcaaacttggcaagaaagaagagctgcagagtatatgacccatgcaccacta
gggagcttaaattctgttggtggtgttgctactgaaattaactctgtaaactatgtatct
cctagatcttggttaacatcatctcactggttcttagcattctttattttagttggtcat
tggtggcatggagcaagagcaagagcttcagcattatctagtgaaacaggcttatctcga
gtatatgaacctgttctttatatgcgacctattgattag
>KrA9_psbC
atgcctctcatttattctttaaaacgacgaactctagttggatccaggtatgcttggtgg
tcaggtaatgcaagattcattgagcttagcggtaaattcctaggtgctcatcttgtgcat
acagctcttattctagtttgggctggaacaatgtctttgtttgaattatctcacttcatc
ccagaaaaacctctatatgagcaaggctttattcttcttcctcatttgtttgaattgggt
ggcactttatattcacacttcataataagcatcctacatctcatctctgcaggtatccta

gctcttggaggtatttatcatgctatagttggtgctgaaagattagaagaaacaagcttc
agtgctctatttgcttatgggttacaagatagattccgtattactgctatacttggatca
catttacttactctaggaattggagcagccttactctttgcaaaagcagtctatttaggt
ggtctttatgatacatgggcttgtggtggtggagacatgagattgattgaaagggtagag
cttggtttaaatccttatctactagctcagtacttacttcgtgctccatttgggtcatca
ggatggataattagcatcaataacatggaagatctaattggaggccattattgggtctca
ttttatctaattctaggcagctgttggcatattcaaacaagagctcttacaattacaaca
agagcttttacatggtcagcagaagcttacctctcttatactttatcagctgtaacttta
tgtggttctatcacaagtttgttttcttggtacaataatactgcatatccaagtgaattc
tatggacctactggggcagaagcctctcaagcacaaagctttacattcctcatacgagat
tggaaattgggtataaagataacttcatccgaaggtcctacagctcttggtaagtatctc
atgagatctccaacaggtgaaataatatttggtggtgaaagtatgagattctggtctatg
caagcacattggcttgaagctctcagaacatcatttggactggacttatctaagattcaa
tctgatattcaaacttggcaagaaagaagagctgcagagtatatgacccatgcaccacta
gggagcttaaattctgttggtggtgttgctactgaaattaactctgtaaactatgtatct
cctagatcttggttaacatcatctcactggttcttagcattctttattttagttggtcat
tggtggcatggagcaagagcaagagcttcagcattatctagtgaaacaggcttatctcga
gtatatgaacctgttctttatatgcgacctattgattag
>KrC1_psbC
atgcctctcatttattctttaaaacgacgaactctagttggatccaggtatgcttggtgg
tcaggtaatgcaagattcattgagcttagcggtaaattcctaggtgctcatcttgtgcat
acagctcttattctagtttgggctggaacaatgtctttgtttgaattatctcacttcatc
ccagaaaaacctctatatgagcaaggctttattcttcttcctcacttgtttgaattgggt
ggcactttatattcacacttcataataagcatcctacatctcatctctgcaggtatccta
gctcttggaggtatttatcatgctatagttggtgctgaaagattagaagaaacaagcttc
agtgctctatttgcttatgggttacaagatagattccgtattactgctatacttggatca
catttacttactctaggaattggagcagccttactctttgcaaaagcagtctatttaggt
ggtctttatgatacatgggcttgtggtggtggagacatgagattgattgaaagggtagag
cttggtttaaatccttatctactagctcagtacttacttcgtgctccatttgggtcatca
ggatggataattagcatcaataacatggaagatctcattggaggtcattattgggtctca
ttttatctaattctaggcagctgttggcatattcaaacaagagctcttacaattacaaca
agagcttttacatggtcagcagaagcttacctctcttatactttatcagctgtaacttta
tgtggttctatcacagcattattttcttggttcaataatactgcatatccaagtgaattc
tatggacctactggggcagaagcctctcaagcacaaagctttacattcctcatacgagat
tggaaattgggtataaagataacttcatccgaaggtcctacagctcttggtaagtatctc
atgagatctccaacaggtgaaataatatttggtggtgaaagtatgagattctggtctatg
caagcacattggcttgaagctctcagaacatcatttggactggacttatctaagattcaa
tctgatattcaaacttggcaagaaagaagagctgcagagtatatgacccatgcaccacta
gggagcttaaattctgttggtggtgttgctactgaaattaactctgtaaactatgtatct
cctagatcttggttaacatcatctcactggttcttagcattctttattttagttggtcat
tggtggcatggagcaagagcaagagctgcagcattgtctagtgaaacaggtttatctcga
gtatatgaacctgtgctttatatgcgacctattgattag
>KrC10_psbC
atgcctctcatttattctttaaaacgacgaactctagttggatccaggtatgcttggtgg
tcaggtaatgcaagattcattgagcttagcggtaaattcctaggtgctcatcttgtgcat
acagctcttattctagtttgggctggaacaatgtctttgtttgaattatctcacttcatc
ccagaaaaacctctatatgagcaaggctttattcttcttcctcacttgtttgaattgggt
ggcactttatattcacacttcataataagcatcctacatctcatctctgcaggtatctta
gctcttggaggtatttatcatgctatagttggtgctgaaagattagaagaaacaagcttc
agtgctctatttgcttatgggttacaagatagattccgtattactgctatacttggatca
catttacttactctaggaattggagcagccttactctttgcaaaagcagtctatttaggt
ggtctttatgatacatgggcttgtggtggtggagacatgagattgattgaaagggtagag
cttggtttaaatccttatctactagctcagtacttacttcgtgctccatttgggtcatca
ggatggataattagcatcaataacatggaagatctcattggaggtcattattgggtctca
ttttatctaattctaggcagctgttggcatattcaaacaagagctcttacaattacaaca
agagcttttacatggtcagcagaagcttacctctcttatactttatcagctgtaacttta
tgtggttctatcacagcattattttcttggttcaataatactgcatatccaagtgaattc

tatggacctactggggcagaagcctctcaagcacaaagctttacattcctcatacgagat
tggaaattgggtataaagataacttcatccgaaggtcctacagctcttggtaagtatctc
atgagatctccaacaggtgaaataatatttggtggtgaaagtatgagattctggtctatg
caagcacattggcttgaagctctcagaacatcatttggactggacttatctaagattcaa
tctgatattcaaacttggcaagaaagaagagctgcagagtacatgacccatgcaccacta
gggagcttaaattctgttggtggtgttgctactgaaattaactctgtaaactatgtatct
cctagatcttggttaacatcatctcactggttcttagcattctttattttagttggtcat
tggtggcatggagcaagagcaagagcttcagcattatctagtgaaacaggcttatctcga
gtatatgaacctgttctttatatgcgacctattgattag
>KrC11_psbC
atgcctctcatttattctttaaaacgacgaactctagttggatccaggtatgcttggtgg
tcaggtaatgcaagattcattgagcttagcggtaaattcctaggtgctcatcttgtgcat
acagctcttattctagtttgggctggaacaatgtctttgtttgaattatctcacttcatc
ccagaaaaacctctatatgagcaaggctttattcttcttcctcacttgtttgaattgggt
ggcactttatattcacacttcataataagcatcctacatctcatctctgcaggtatccta
gctcttggaggtatttatcatgctatagttggtgctgaaagattagaagaaacaagcttc
agtgctctatttgcttatgggttacaagatagattccgtattactgctatacttggatca
catttacttactctaggaattggagcagccttactctttgcaaaagcagtctatttaggt
ggtctttatgatacatgggcttgtggtggtggagacatgagattgattgaaagggtagag
cttggtttaaatccttatctactagctcagtacttacttcgtgctccatttgggtcatca
ggatggataattagcatcaataacatggaagatctcattggaggtcattattgggtctca
ttttatctaattctaggcagctgttggcatattcaaacaagagctcttacaattacaaca
agagcttttacatggtcagcagaagcttacctctcttatactttatcagctgtaacttta
tgtggttctatcacagcattattttcttggttcaataatactgcatatccaagtgaattc
tatggacctactggggcagaagcctctcaagcacaaagctttacattcctcatacgagat
tggaaattgggtataaagataacttcatccgaaggtcctacagctcttggtaagtatctc
atgagatctccaacaggtgaaataatatttggtggtgaaagtatgagattctggtctatg
caagcacattggcttgaagctctcagaacatcatttggactggacttatctaagattcaa
tctgatattcaaacttggcaagaaagaagagctgcagagtatatgacccatgcaccacta
gggagcttaaattctgttggtggtgttgctactgaaattaactctgtaaactatgtatct
cctagatcttggttaacatcatctcactggttcttagcattctttattttagttggtcat
tggtggcatggagcaagagcaagagcttcagcattatctagtgaaacaggcttatctcga
gtatatgaacctgttctttatatgcgacctattgattag
>KrC12_psbC
atgcctctcatttattctttaaaacgacgaactctagttggatccaggtatgcttggtgg
tcaggtaatgcaagattcattgagcttagcggtaaattcctaggtgctcatcttgtgcat
acagctcttattctagtttgggctggaacaatgtctttgtttgaattatctcacttcatc
ccagaaaaacctctatatgagcaaggctttattcttcttcctcacttgtttgaattgggt
ggcactttatattcacacttcataataagcatcctacatctcatctctgcaggtatccta
gctcttggaggtatttatcatgctatagttggtgctgaaagattagaagaaacaagctat
agctccctatttgcttatgggttacaagatagattccgtattactgctatacttggatca
catttacttactctaggaattggagcagccttactctttgcaaaagcagtctatttaggt
ggtctttatgatacatgggcttgtggtggtggagacatgagattgattgaaagggtagag
cttggtttaaatccttatctactagctcagtacttacttcgtgctccatttgggtcatca
ggatggataattagcatcaataacatggaagatctcattggaggtcattattgggtctca
ttttatctaattctaggcagctgttggcatattcaaacaagagctcttacaattacaaca
agagcttttacatggtcagcagaagcttacctctcttatactttatcagctgtaacttta
tgtggttctatcacagcattattttcttggttcaataatactgcatatccaagtgaattc
tatggacctactggggcagaagcctctcaagcacaaagctttacattcctcatacgagat
tggaaattgggtataaagataacttcatccgaaggtcctacagctcttggtaagtatctc
atgagatctccaacaggtgaaataatatttggtggtgaaagtatgagattctggtctatg
caagcacattggcttgaagctctcagaacatcatttggactggacttatctaagattcaa
tctgatattcaaacttggcaagaaagaagagctgcagagtatatgacccatgcaccacta
gggagcttaaattctgttggtggtgttgctactgaaattaactctgtaaactatgtatct
cctagatcttggttaacatcatctcactggttcttagcattctttattttagttggtcat
tggtggcatggagcaagagcaagagcttcagcattatctagtgaaacaggcttatctcga
gtatatgaacctgttctttatatgcgacctattgattag

>KrC13_psbC
atgcctctcatttattctttaaaacgacgaactctagttggatccaggtatgcttggtgg
tcaggtaatgcaagattcattgagcttagcggtaaattcctaggtgctcatcttgtgcat
acagctcttattctagtttgggctggaacaatgtctttgtttgaattatctcacttcatc
ccagaaaaacctctatatgagcaaggctttattcttcttcctcacttgtttgaattgggt
ggcactttatattcacacttcataataagcatcctacatctcatctctgcaggtatccta
gctcttggaggtatttatcatgctatagttggtgctgaaagattagaagaaacaagcttc
agttctctatttgcttatgggttacaagatagattccgtattactgctatacttggatca
catttacttactctaggaattggagcagccttactctttgcaaaagcagtctatttaggt
ggtctttatgatacatgggcttgtggtggtggagacatgagattgattgaaagggtagag
cttggtttaaatccttatctactagctcagtacttacttcgtgctccatttgggtcatca
ggatggataattagcatcaataacatggaagatctcattggaggtcattattgggtctca
ttttatctaattctaggcagctgttggcatattcaaacaagagctcttacaattacaaca
agagcttttacatggtcagcagaagcttacctctcttatactttatcagctgtaacttta
tgtggttctatcacagcattattttcttggttcaataatactgcatatccaagtgaattc
tatggacctactggggcagaagcctctcaagcacaaagctttacattcctcatacgagat
tggaaattgggtataaagataacttcatccgaaggtcctacagctcttggtaagtatctc
atgagatctccaacaggtgaaataatatttggtggtgaaagtatgagattctggtctatg
caagcacattggcttgaagctctcagaacatcatttggactggacttatctaagattcaa
tctgatattcaaacttggcaagaaagaagagctgcagagtatatgacccatgcaccacta
gggagcttaaattctgttggtggtgttgctactgaaattaactctgtaaactatgtatct
cctagatcttggttaacatcatctcactggttcttagcattctttattttagttggtcat
tggtggcatggagcaagagcaagagcttcagcattatctagtgaaacaggcttatctcga
gtatatgaacctgttctttatatgcgacctattgattag
>KrC14_psbC
atgcctctcatttattctttaaaacgacgaactctagttggatccaagtatgcttggtgg
tcaggtaatgcaagattcattgagcttagcggtaaattcctaggtgctcatcttgtgcat
acagctcttattctagtttgggctggaacaatgtctttgtttgaattatctcacttcatc
ccagaaaaacctctatatgagcaaggctttattcttctacctcatttgtttgaactggga
ggcaatctatattcacacttcataataagcatcctacatctcatctctgcaggtatccta
gctcttggaggtatttatcatgctatagttggtgctgaaagattagaagaaacaagctat
agctccctatttgcttatgggttacaagatagattccgtattactgctatacttggatca
catttacttactctaggaattggagcagccttactctttgcaaaagcagtctatttaggt
ggtctttatgatacatgggcttgtggtggtggagacatgagattgattgaaagagtagag
cttggtttaaatccttatctactagctcagtacttacttcgtgctccatttgggtcatca
ggatggataattagcatcaataacatggaagatctaattggaggccattattgggtctca
ttttatctaattctaggcagctgttggcatattcaaacaagagctcttacaattacaaca
agagcttttacatggtcagcagaagcttacctctcttatactttatcagctgtaacttta
tgtggttctatcacagcattattttcttggttcaataatactgcatatccaagtgaattc
tatggacctactggggcagaagcctctcaagcacaaagctttacattcctcatacgagat
tggaaattgggtataaagataacttcatccgaaggtcctacagctcttggtaagtatctc
atgagatctccaacaggtgaaataatatttggtggtgaaagtatgagattctggtctatg
caagcacattggcttgaagctctcagaacatcatttggactggacttatctaagattcaa
tctgatattcaaacttggcaagaaagaagagctgcagagtatatgacccatgcaccacta
gggagcttaaattctgttggtggtgttgctactgaaattaactctgtaaactatgtatct
cctagatcttggttaacatcatctcactggttcttagcattctttattttagttggtcat
tggtggcatggagcaagagcaagagcttcagcattatctagtgaaacaggcttatctcga
gtatatgaacctgttctttatatgcgacctattgattag
>KrC15_psbC
atgcctctcatttattctttaaaacgacgaactctagttggatccaggtatgcttggtgg
tcaggtaatgcaagattcattgagcttagcggtaaattcctaggtgctcatcttgtgcat
acagctcttattctagtttgggctggaacaatgtctttgtttgaattatctcacttcatc
ccagaaaaacctctatatgagcaaggctttattcttcttcctcacttgtttgaattgggt
ggcactttatattcacacttcataataagcatcctacatctcatctctgcaggtatccta
gctcttggaggtatttatcatgctatagttggtgctgaaagattagaagaaacaagcttc
agtgctctatttgcttatgggttacaagatagattccgtattactgctatacttggatca
catttacttactctaggaattggagcagccttactctttgcaaaagcagtctatttaggt

ggtctttatgatacatgggcttgtggtggtggagacatgagattgattgaaagggtagag
cttggtttaaatccttatctactagctcagtacttacttcgtgctccatttgggtcatca
ggatggataattagcatcaataacatggaagatctcattggaggtcattattgggtctca
ttttatctaattctaggcagctgttggcatattcaaacaagagctcttacaattacaaca
agagcttttacatggtcagcagaagcttacctctcttatactttatcagctgtaacttta
tgtggttctatcacagcattattttcttggttcaataatactgcatatccaagtgaattc
tatggacctactggggcagaagcctctcaagcacaaagctttacattcctcatacgagat
tggaaattgggtataaagataacttcatccgaaggtcctacagctcttggtaagtatctc
atgagatctccaacaggtgaaataatatttggtggtgaaagtatgagattctggtctatg
caagcacattggcttgaagctctcagaacatcatttggactggacttatctaagattcaa
tctgatattcaaacttggcaagaaagaagagctgcagagtatatgacccatgcaccacta
gggagcttaaattctgttggtggtgttgctactgaaattaactctgtaaactatgtatct
cctagatcttggttaacatcatctcactggttcttagcattctttattttagttggtcat
tggtggcatggagcaagagcaagagcttcagcattatctagtgaaacaggcttatctcga
gtatatgaacctgttctttatatgcgacctattgattag
>KrC2_psbC
atgcctctcatttattctttaaaacgacgaactctagttggatccaggtatgcttggtgg
tcaggtaatgcaagattcattgagcttagcggtaaattcctaggtgctcatcttgtgcat
acagctcttattctagtttgggctggaacaatgtctttgtttgaattatctcacttcatc
ccagaaaaacctctatatgagcaaggctttattcttcttcctcacttgtttgaattgggt
ggcactttatattcacacttcataataagcatcctacatctcatctctgcaggtatccta
gctcttggaggtatttatcatgctatagttggtgctgaaagattagaagaaacaagcttc
agtgctctatttgcttatgggttacaagatagattccgtattactgctatacttggatca
catttacttactctaggaattggagcagccttactctttgcaaaagcagtctatttaggt
ggtctttatgatacatgggcttgtggtggtggagacatgagattgattgaaagggtagag
cttggtttaaatccttatctactagctcagtacttacttcgtgctccatttgggtcatca
ggatggataattagcatcaataacatggaagatctcattggaggtcattattgggtctca
ttttatctaattctaggcagctgttggcatattcaaacaagagctcttacaattacaaca
agagcttttacatggtcagcagaagcttacctctcttatactttatcagctgtaacttta
tgtggttctatcacagcattattttcttggttcaataatactgcatatccaagtgaattc
tatggacctactggagcagaagcctctcaagcacaaagctttacattcctcatacgagat
tggaaattgggtataaagataacttcatccgaaggtcctacagctcttggtaagtatctc
atgagatctccaacaggtgaaataatatttggtggtgaaagtatgagattctggtctatg
caagcacattggcttgaagctctcagaacatcatttggactggacttatctaagattcaa
tctgatattcaaacttggcaagaaagaagagctgcagagtatatgacccatgcaccacta
gggagcttaaattctgttggtggtgttgctactgaaattaactctgtaaactatgtatct
cctagatcttggttaacatcatctcactggttcttagcattctttattttagttggtcat
tggtggcatggagcaagagcaagagcttcagcattatctagtgaaacaggcttatctcga
gtatatgaacctgttctttatatgcgacctattgattag
>KrC3_psbC
atgcctctcatttattctttaaaacgacgaactctagttggatccaggtatgcttggtgg
tcaggtaatgcaagattcattgagcttagcggtaaattcctaggtgctcatcttgtgcat
acagctcttattctagtttgggctggaacaatgtctttgtttgaattatctcacttcatc
ccagaaaaacctctatatgagcaaggctttattcttcttcctcacttgtttgaattgggt
ggcactttatattcacacttcataataagcatcctacatctcatctctgcaggtatccta
gctcttggaggtatttatcatgctatagttggtgctgaaagattagaagaaacaagcttc
agtgctctatttgcttatgggttacaagatagattccgtattactgctatacttggatca
catttacttactctaggaattggagcagccttactctttgcaaaagcagtctatttaggt
ggtctttatgatacatgggcttgtggtggtggagacatgagattgattgaaagggtagag
cttggtttaaatccttatctactagctcagtacttacttcgtgctccatttgggtcatca
ggatggataattagcatcaataacatggaagatctcattggaggtcattattgggtctca
ttttatctaattctaggcagctgttggcatattcaaacaagagctcttacaattacaaca
agagcttttacatggtcagcagaagcttacctctcttatactttatcagctgtaacttta
tgtggttctatcacagcattattttcttggttcaataatactgcatatccaagtgaattc
tatggacctactggggcagaagcctctcaagcacaaagctttacattcctcatacgagat
tggaaattgggtataaagataacttcatccgaaggtcctacagctcttggtaagtatctc
atgagatctccaacaggtgaaataatatttggtggtgaaagtatgagattctggtctatg

caagcacattggcttgaagctctcagaacatcatttggactggacttatctaagattcaa
tctgatattcaaacttggcaagaaagaagagctgcagagtatatgacccatgcaccacta
gggagcttaaattctgttggtggtgttgctactgaaattaactctgtaaactatgtatct
cctagatcttggttaacatcatctcactggttcttagcattctttattttagttggtcat
tggtggcatggagcaagagcaagagcttcagcattatctagtgaaacaggcttatctcga
gtatatgaacctgttctttatatgcgacctattgattag
>KrC4_psbC
atgcctcttcttcattctttaaaacgacgaactctagttggatccaggtatgcttggtgg
tcaggtaatgcaagattcattgagcttagcggtaaattcctaggtgctcatcttgtgcat
acagctcttattctagtttgggctggaacaatgtctttgtttgaattatctcacttcatc
ccagaaaaacctctatatgagcaaggctttattcttcttcctcacttgtttgaattgggt
ggcactttatattcacacttcataataagcatcctacatctcatctctgcaggtatccta
gctcttggaggtatttatcatgctatagttggtgctgaaagattagaagaaacaagcttc
agtgctctatttgcttatgggttacaagatagattccgtattactgctatacttggatca
catttacttactctaggaattggagcagccttactctttgcaaaagcagtctatttaggt
ggtctttatgatacatgggcttgtggtggtggagacatgagattgattgaaagggtagag
cttggtttaaatccttatctactagctcagtacttacttcgtgctccatttgggtcatca
ggatggataattagcatcaataacatggaagatctaattggaggccattattgggtctca
ttttatctaattctaggcagctgttggcatattcaaacaagagctcttacaattacaaca
agagcttttacatggtcagcagaagcttacctctcttatactttatcagctgtaacttta
tgtggttctatcacagcattattttcttggttcaataatactgcatatccaagtgaattc
tatggacctactggggcagaagcctctcaagcacaaagctttacattcctcatacgagat
tggaaattgggtataaagataagttcatccgaaggtcctacagctcttggtaagtatctc
atgagatctccaacaggtgaaataatatttggtggtgaaagtatgagattctggtctatg
caagcacattggcttgaagctctcagaacatcatttggactggacttatctaagattcaa
tctgatattcaaacttggcaagaaagaagagctgcagagtatatgacccatgcaccacta
gggagcttaaattctgttggtggtgttgctactgaaattaactctgtaaactatgtatct
cctagatcttggttaacatcatctcactggttcttagcattctttattttagttggtcat
tggtggcatggagcaagagcaagagcttcagcattatctagtgaaacaggcttatctcga
gtatatgaacctgttctttatatgcgacctattgattag
>KrC5_psbC
atgcctctcatttattctttaaaacgacgaactctagttggatccaggtatgcttggtgg
tcaggtaatgcaagattcattgagcttagcggtaaattcctaggtgctcatcttgtgcat
acagctcttattctagtttgggctggaacaatgtctttgtttgaattatctcacttcatc
ccagaaaaacctctatatgagcaaggctttattcttcttcctcacttgtttgaattgggt
ggcactttatattcacacttcataataagcatcctacatctcatctctgcaggtatccta
gctcttggaggtatttatcatgctatagttggtgctgaaagattagaagaaacaagcttc
agtgctctatttgcttatgggttacaagatagattccgtattactgctatacttggatca
catttacttactctaggaattggagcagccttactctttgcaaaagcagtctatttaggt
ggtctttatgatacatgggcttgtggtggtggagacatgagattgattgaaagggtagag
cttggtttaaatccttatctactagctcagtacttacttcgtgctccatttgggtcatca
ggatggataattagcatcaataacatggaagatctcattggaggtcattattgggtctca
ttttatctaattctaggcagctgttggcatattcaaacaagagctcttacaattacaaca
agagcttttacatggtcagcagaagcttacctctcttatactttatcagctgtaacttta
tgtggttctatcacagcattattttcttggttcaataatactgcatatccaagtgaattc
tatggacctactggggcagaagcctctcaagcacaaagctttacattcctcatacgagat
tggaaattgggtataaagataacttcatccgaaggtcctacagctcttggtaagtatctc
atgagatctccaacaggtgaaataatatttggtggtgaaagtatgagattctggtctatg
caagcacattggcttgaagctctcagaacatcatttggactggacttatctaagattcaa
tctgatattcaaacttggcaagaaagaagagctgcagagtatatgacccatgcaccacta
gggagcttaaattctgttggtggtgttgctactgaaattaactctgtaaactatgtatct
cctagatcttggttaacatcatctcactggttcttagcattctttattttagttggtcat
tggtggcatggagcaagagcaagagcttcagcattatctagtgaaacaggcttatctcga
gtatatgaacctgttctttatatgcgacctattgattag
>KrC6_psbC
atgcctctcattcattctttaaaacgacgaactctagttggatccaggtatgcttggtgg
tcaggtaatgcaagattcattgagcttagcggtaaattcctaggtgctcatcttgtgcat

acagctcttattctagtttgggctggaacaatgtctttgtttgaattatctcacttcatc
ccagaaaaacctctatatgagcaaggctttattcttcttcctcacttgtttgaattgggt
ggcactttatattcacacttcataataagcatcctacatctcatctctgcaggtatccta
gctcttggaggtatttatcatgctatagttggtgctgaaagattagaagaaacaagcttc
agtgctctatttgcttatgggttacaagatagattccgtattactgctatacttggatca
catttacttactctaggaattggagcagccttactctttgcaaaagcagtctatttaggt
ggtctttatgatacatgggcttgtggtggtggagacatgagattgattgaaagggtagag
cttggtttaaatccttatctactagctcagtacttacttcgtgctccatttgggtcatca
ggatggataattagcatcaataacatggaagatctcattggaggtcattattgggtctca
ttttatctaattctaggcagctgttggcatattcaaacaagagctcttacaattacaaca
agagcttttacatggtcagcagaagcttacctctcttatactttatcagctgtaacttta
tgtggttctatcacagcattattttcttggttcaataatactgcatatccaagtgaattc
tatggacctactggggcagaagcctctcaagcacaaagctttacattcctcatacgagat
tggaaattgggtataaagataacttcatccgaaggtcctacagctcttggtaagtatctc
atgagatctccaacaggtgaaataatatttggtggtgaaagtatgagattctggtctatg
caagcacattggcttgaagctctcagaacatcatttggactggacttatctaagattcaa
tctgatattcaaacttggcaagaaagaagagctgcagagtatatgacccatgcaccacta
gggagcttaaattctgttggtggtgttgctactgaaattaactctgtaaactatgtatct
cctagatcttggttaacatcatctcactggttcttagcattctttattttagttggtcat
tggtggcatggagcaagagcaagagcttcagcattatctagtgaaacaggcttatctcga
gtatatgaacctgttctttatatgcgacctattgattag
>KrC7_psbC
atgcctctcatttattctttaaaacgacgaactctagttggatccaggtatgcttggtgg
tcaggtaatgcaagattcattgagcttagcggtaaattcctaggtgctcatcttgtgcat
acagctcttattctagtttgggctggaacaatgtctttgtttgaattatctcacttcatc
ccagaaaaacctctatatgagcaaggctttattcttcttcctcatttgtttgaattgggt
ggcactttatattcacacttcataataagcatcctacatctcatctctgcaggtatccta
gctcttggaggtatttatcatgctatagttggtgctgaaagattagaagaaacaagcttc
agtgctctatttgcttatgggttacaagatagattccgtattactgctatacttggatca
catttacttactctaggaattggagcagccttactctttgcaaaagcagtctatttaggt
ggtctttatgatacttgggcttgtggtggtggagacatgagattgattgaaagggtagag
cttggtttaaatccttatctactagctcagtacttacttcgtgctccatttgggtcatca
ggatggataattagcatcaataacatggaagatctcattggaggtcattattgggtctca
ttttatctaattctaggcagctgttggcatattcaaacaagagctcttacaattacaaca
agagcttttacatggtcagcagaagcttacctctcttatactttatcagctgtaacttta
tgtggttctatcacagcattattttcttggttcaataatattgcatatccaagtgaattc
tatggacctactggggcagaagcctctcaagcacaaagctttacattcctcatacgagat
tggaaattgggtataaagataacttcatccgaaggtcctacagctcttggtaagtatctc
atgagatctccaacaggtgaaataatatttggtggtgaaagtatgagattctggtctatg
caagcacattggcttgaagctctcagaacatcatttggactggacttatctaagattcaa
tctgatattcaaacttggcaagaaagaagagctgcagagtatatgacccatgcaccacta
gggagcttaaattctgttggtggtgttgctactgaaattaactctgtaaactatgtatct
cctagatcttggttaacatcatctcactggttcttagcattctttattttagttggtcat
tggtggcatggagcaagagcaagagcttcagcattatctagtgaaacaggcttatctcga
gtatatgaacctgttctttatatgcgacctattgattag
>KrC8_psbC
atgcctctcatttattctttaaaacgacgaactctagttggatccaggtatgcttggtgg
tcaggtaatgcaagattcattgagcttagcggtaaattcctaggtgctcatcttgtgcat
acagctcttattctagtttgggctggaacaatgtctttgtttgaattatctcacttcatc
ccagaaaaacctctatatgagcaaggctttattcttcttcctcacttgtttgaactgggt
ggcactttatattcacacttcataataagcatcctacatctcatctctgcaggtatccta
gctcttggaggtatttatcatgctatagttggtggtgaaagactagaagaaacaagctat
agctccctatttgcttatgggttacaagatagattccgtattactgctatacttggatca
catttacttactctaggaattggagcagccttactctttgcaaaagcagtctatttaggt
ggtctttatgatacatgggcttgtggtggtggagacatgagattgattgaaagggtagag
cttggtttaaatccttatctactagctcagtacttacttcgtgctccatttgggtcatca
ggatggataattagcatcaataacatggaagatctcattggaggtcattattgggtctca

ttttatctaattctaggcagctgttggcatattcaaacaagagctcttacaattacaaca
agagcttttacatggtcagcagaagcttacctctcttatactttatcagctgtaacttta
tgtggttctatcacagcattattttcttggttcaataatactgcatatccaagtgaattc
tatggacctactggggcagaagcctctcaagcacaaagctttacattcctcatacgagat
tggaaattgggtataaagataacttcatccgaaggtcctacagctcttggtaagtatctc
atgagatctccaacaggtgaaataatatttggtggtgaaagtatgagattctggtctatg
caagcacattggcttgaagctctcagaacatcatttggactggacttatctaagattcaa
tctgatattcaaacttggcaagaaagaagagctgcagagtatatgacccatgcaccacta
gggagcttaaattctgttggtggtgttgctactgaaattaactctgtaaactatgtatct
cctagatcttggttaacatcatctcactggttcttagcattctttattttagttggtcat
tggtggcatggagcaagagcaagagcttcagcattatctagtgaaacaggcttatctcga
gtatatgaacctgttctttatatgcgacctattgattag
>KrC9_psbC
atgcctctcattcattctttaaaacgacgaactctagttggatccaggtatgcttggtgg
tcaggtaatgcaagattcattgagcttagcggtaaattcctaggtgctcatcttgtgcat
acagctcttattctagtttgggctggaacaatgtctttgtttgaattatctcacttcatc
ccagaaaaacctctatatgagcaaggctttattcttcttcctcacttgtttgaattgggt
ggcactttatattcacacttcataataagcatcctacatctcatctctgcaggtatccta
gctcttggaggtatttatcatgctatagttggtgctgaaagattagaagaaacaagctat
agctccctatttgcttatgggttacaagatagattccgtattactgctatacttggatca
catttacttactctaggaattggagcagccttactctttgcaaaagcagtctatttaggt
ggtctttatgatacatgggcttgtggtggtggagacatgagattgattgaaagggtagag
cttggtttaaatccttatctactagctcagtacttacttcgtgctccatttgggtcatca
ggatggataattagcatcaataacatggaagatctcattggaggtcattattgggtctca
ttttatctaattctaggcagctgttggcatattcaaacaagagctcttacaattacaaca
agagcttttacatggtcagcagaagcttacctctcttatactttatcagctgtaacttta
tgtggttctatcacaagtttgttttcttggtacaataatactgcatatccaagtgaattc
tatggacctactggggcagaagcctctcaagcacaaagctttacattcctcatacgagat
tggaaattgggtataaagataacttcatccgaaggtcctacagctcttggtaagtatctc
atgagatctccaacaggtgaaataatatttggtggtgaaagtatgagattctggtctatg
caagcacattggcttgaagctctcagaacatcatttggactggacttatctaagattcaa
tctgatattcaaacttggcaagaaagaagagctgcagagtatatgacccatgcaccacta
gggagcttaaattctgttggtggtgttgctactgaaattaactctgtaaactatgtatct
cctagatcttggttaacatcatctcactggttcttagcattctttattttagttggtcat
tggtggcatggagcaagagcaagagcttcagcattatctagtgaaacaggtttatctcga
gtatatgaacctgttctttatatgcgacctattgattag
>KrD1_psbC
atgcctctcatttattctttaaaacgacgaactctagttggatccaggtatgcttggtgg
tcaggtaatgcaagattcattgagcttagcggtaaattcctaggtgctcatcttgtgcat
acagctcttattctagtttgggctggaacaatgtctttgtttgaattatctcacttcatc
ccagaaaaacctctatatgagcaaggctttattcttcttcctcacttgtttgaattgggt
ggcactttatattcacacttcataataagcatcctacatctcatctctgcaggtatccta
gctcttggaggtatttatcatgctatagttggtgctgaaagattagaagaaacaagcttc
agtgctctatttgcttatgggttacaagatagattccgtattactgctatacttggatca
catttacttactctaggaattggagcagccttactctttgcaaaagcagtctatttaggt
ggtctttatgatacatgggcttgtggtggtggagacatgagattgattgaaagggtagag
cttggtttaaatccttatctactagctcagtacttacttcgtgctccatttgggtcatca
ggatggataattagcatcaataacatggaagatctcattggaggccattattgggtctca
ttttatctaattctaggtagctgttggcatattcaaacaagagctcttacaattacaaca
agagcttttacatggtcagcagaagcttacctctcttatactttatcagctgtaacttta
tgtggttctatcacagcattattttcttggttcaataatactgcatatccaagtgaattc
tatggacctactggggcagaagcctctcaagcacaaagctttacattcctcatacgagat
tggaaattgggtataaagataacttcatccgaaggtcctacagctcttggtaagtatctc
atgagatctccaacaggtgaaataatatttggtggtgaaagtatgagattctggtctatg
caagcacattggcttgaagctctcagaacatcatttggactggacttatctaagattcaa
tctgatattcaaacttggcaagaaagaagagctgcagagtatatgacccatgcaccacta
gggagcttaaattctgttggtggtgttgctactgaaattaactctgtaaactatgtatct

cctagatcttggttaacatcatctcactggttcttagcattctttattttagttggtcat
tggtggcatggagcaagagcaagagcttcagcattatctagtgaaacaggcttatctcga
gtatatgaacctgttctttatatgcgacctattgattag
>KrD11_psbC
atgcctctccttcattctttaaaacgacgaactctagttggatccaggtatgcttggtgg
tcaggtaatgcaagattcattgagcttagcggtaaattcctaggtgctcatcttgttcat
acagctcttattctagtttgggctggaacaatgtctttgtttgaattatctcacttcatt
ccagaaaaacctctatatgagcaaggctttattcttctacctcacttgtttgaattgggt
ggcactttatattcacacttcataataagcatcctacatctcatctctgcaggtatccta
gctcttggaggtatttatcatgctatagttggtggtgaaagactagaagaaacaagctat
agctccctatttgcttatgggttacaagatagattccgtattactgctatacttggatca
catttacttactctaggaattggagcagccttactctttgcaaaagcagtctatttaggt
ggtctttatgatacttgggcttgtggtggtggagacatgagattgattgaaagggtagag
cttggtttaaatccttatctactagctcagtacttacttcgtgctccatttgggtcatca
ggatggataattagcatcaataacatggaagatctaattggaggccattattgggtctca
ttttatctaattctaggtagctgttggcatattcaaacaagagctcttacaattacaaca
agagcttttacatggtcagcagaagcttacctctcttatactttatcagctgtaacttta
tgtggttctatcacagcattattttcttggttcaataatactgcatatccaagtgaattc
tatggacctactggggcagaagcctctcaagcacaaagctttacattcctcatacgagat
tggaaattgggtataaagataacttcatccgaaggtcctacagctcttggtaagtatctc
atgagatctccaacaggtgaaataatatttggtggtgaaagtatgagattctggtctatg
caagcacattggcttgaagctctcagaacatcatttggactggacttatctaagattcaa
tctgatattcaaacttggcaagaaagaagagctgcagagtatatgacccatgcaccacta
gggagcttaaattctgttggtggtgttgctactgaaattaactctgtaaactatgtatct
cctagatcttggttaacatcatctcactggttcttagcattctttattttagttggtcat
tggtggcatggagcaagagcaagagcttcagcattatctagtgaaacaggtttatctcga
gtatatgaacctgtgctttatatgcgacctattgattag
>KrD13_psbC
atgcctctcatttattctttaaaacgacgaactctagttggatccaggtatgcttggtgg
tcaggtaatgcaagattcattgagcttagcggtaaattcctaggtgctcatcttgtgcat
acagctcttattctagtttgggcaggaagaatgtctttgtttgaattatctcacttcatc
ccagaaaaacctctatatgagcaaggctttattcttcttcctcacttgtttgaattgggt
ggcactttatattcacacttcataataagcatcctacatctcatctctgcaggtatccta
gctcttggaggtatttatcatgctatagttggtgctgaaagattagaagaaacaagcttc
agtgctctatttgcttatgggttacaagatagattccgtattactgctatacttggatca
catttacttactctaggaattggagcagccttactctttgcaaaagcagtctatttaggt
ggtctttatgatacatgggcttgtggtggtggagacatgagattgattgaaagggtagag
cttggtttaaatccttatctactagctcagtacttacttcgtgctccatttgggtcatca
ggatggataattagcatcaataacatggaagatctcattggaggtcattattgggtctca
ttttatctaattctaggcagctgttggcatattcaaacaagagctcttacaattacaaca
agagcttttacatggtcagcagaagcttacctctcttatactttatcagctgtaacttta
tgtggttctatcacagcattattttcttggttcaataatactgcatatccaagtgaattc
tatggacctactggggcagaagcctctcaagcacaaagctttacattcctcatacgagat
tggaaattgggtataaagataacttcatccgaaggtcctacagctcttggtaagtatctc
atgagatctccaacaggtgaaataatatttggtggtgaaagtatgagattctggtctatg
caagcacattggcttgaagctctcagaacatcatttggactggacttatctaagattcaa
tctgatattcaaccttggcaagaaagaagagctgcagagtatatgacccatgcaccacta
gggagcttaaattctgttggtggtgttgctactgaaattaactctgtaaactatgtatct
cctagatcttggttaacatcatctcactggttcttagcattctttattttagttggtcat
tggtggcatggagcaagagcaagagcttcagcattatctagtgaaacaggcttatctcga
gtatatgaacctgttctttatatgcgacctattgattag
>KrD3_psbC
atgcctctcatttattctttaaaacgacgaactctagttggatccaggtatgcttggtgg
tcaggtaatgcaagattcattgagcttagcggtaaattcctaggtgctcatcttgtgcat
acagctcttattctagtttgggctggaacaatgtctttgtttgaattatctcacttcatc
ccagaaaaacctctatatgagcaaggctttattcttcttcctcacttgtttgaattgggt
ggcactttatattcacacttcataataagcatcctacatctcatctctgcaggtatctta

gctcttggaggtatttatcatgctatagttggtggtgaaagactagaagaaacaagctat
agctccctatttgcttatgggttacaagatagattccgtattacagctatacttggatca
catttacttactctaggaattggagcagccttactctttgcaaaagcagtctatttaggt
ggtctttatgatacatgggcttgtggtggtggagacatgagattgattgaaagggtagag
cttggtttaaatccttatctactagctcaatacttacttcgtgctccatttggttcatca
ggatggataattagcatcaataacatggaagatctcattggaggtcattattgggtctca
ttttatctaattctaggcagctgttggcatattcaaacaagagctcttacaattacaaca
agagcttttacatggtcagcagaagcttacctctcttatactttatcagctgtaacttta
tgtggttctatcacagcattattttcttggttcaataatactgcatatccaagtgaattc
tatggacctactggggcagaagcctctcaagcacaaagctttacattccttatacgagat
tggaaattgggtataaagataagttcatccgaaggtcctacagctcttggtaagtatctc
atgagatctccaacaggtgaaataatatttggtggtgaaagtatgagattctggtctatg
caagcacattggcttgaagctctcagaacatcctttggattagacttatctaagattcaa
tctgatattcaaacttggcaagaaagaagagctgcagagtatatgacccatgcaccacta
gggagcttaaattctgttggtggtgttgctactgaaattaactctgtaaactatgtatct
cctagatcttggttaacctcctctcattggttcttagcattctttattttagtgggtcat
tggtggcatggagcaagagcaagagcttcagcattatctagtgaaacaggcttatctcga
gtatatgaacctgtgctttatatgcgacctattgattag
>KrD8_psbC
atgcctctcatttattctttaaaacgacgaactctagttggatccaggtatgcttggtgg
tcaggtaatgcaagattcattgagcttagcggtaaattcctaggtgctcatcttgtgcat
acagctcttattctagtttgggctggaacaatgtctttgtttgaattatctcacttcatc
ccagaaaaacctctatatgagcaaggctttattcttcttcctcacttgtttgaattgggt
ggcactttatattcacacttcataataagcatcctacatctcatctctgcaggtatccta
gctcttggaggtatttatcatgctatagttggtggtgaaagactagaagaaacaagctat
agctccctatttgcttatgggttacaagatagattccgtattactgctatacttggatca
catttacttactctaggaattggagcagccttactctttgcaaaagcagtctatttaggt
ggtctttatgatacatgggcttgtggtggtggagacatgagattgattgaaagggtagag
cttggtttaaatccttatctactagctcagtacttacttcgtgctccatttgggtcatca
ggatggataattagcatcaataacatggaagatctaattggaggccattattgggtctca
ttttatctaattctaggtagctgttggcatattcaaacaagagctcttacaattacaaca
agagcttttacatggtcagcagaagcttacctctcttatactttatcagctgtaacttta
tgtggttctatcacaagtttgttttcttggtacaataatactgcatatccaagtgaattc
tatggacctactggagcagaagcctctcaagcacaaagctttacattcctcatacgagat
tggaaattgggtataaagataacttcatccgaaggtcctacagctcttggtaagtatctc
atgagatctccaacaggtgaaataatatttggtggtgaaagtatgagattctggtctatg
caagcacattggcttgaagctctcagaacatcatttggactagacttatctaagattcaa
tctgatattcaaacttggcaagaaagaagagctgcagagtatatgacccatgcaccacta
gggagcttaaattctgttggtggtgttgctactgaaattaactctgtaaactatgtatct
cctagatcttggttaacctcctctcattggttcttggccttctttattttagtgggtcat
tggtggcatggagcaagagcaagagcttcagcattatctagtgaaacaggcttatctcga
gtatatgaacctgttctttatatgcgacctattgattag
>KrD9_psbC
atgcctctcatttattctttaaaacgacgaactctagttggatccaggtatgcttggtgg
tcaggtaatgcaagattcattgagcttagcggtaaattcctaggtgctcatcttgtgcat
acagctcttattctagtttgggcaggaacaatgtctttgtttgaattatctgacttcatt
ccagaaaaacctctatatgagcaaggctttattcttctacctcatttgtttgaactggga
ggcaatctatattcacacttcataataagcatcctacatctcatctctgcaggtatccta
gctcttggaggtatttatcatgctatagttggtggtgaaagactagaagaaacaagctat
agctccctatttgcttatgggttacaagatagattccgtattacagctatacttggttca
catttacttactctaggaattggagcagccttactctttgcaaaagcagtctatttaggt
ggtctttatgatacatgggcttgtggtggtggagacatgagattgattgaaagggtagag
cttggtttaaatccttatctactagctcagtacttacttcgtgctccatttgggtcatca
ggatggataattagcatcaataacatggaagatctaattggaggccattattgggtctca
ttttatctaattctaggtagctgttggcatattcaaacaagagctcttacaatcacaaca
agagcttttacatggtcagcagaagcttacctctcttacactctatcagctgtaacttta
tgtggttctatcacaagtttgttttcttggtacaataatactgcatatccaagtgaattc

tatggacctactggagcagaagcctctcaagcacaaagctttacattccttatacgagat
tggaaattgggtataaagataagttcatccgaaggtcctacagctcttggtaagtatctc
atgagatctccaacaggtgaaataatatttggtggtgaaagtatgagattctggtctatg
caagcacattggcttgaagctctcagaacatcctttggattagacttatctaagattcaa
tctgatattcaaacttggcaagaaagaagagctgcagagtacatgacccatgcaccacta
gggagcttaaattctgttggtggtgttgctactgaaattaactctgtaaactatgtatct
cctagatcttggttaacctcctctcattggttcttggccttctttattttagttggtcat
tggtggcatggagcaagagcaagagcttcagcattatctagtgaaacaggcttatctcga
gtatatgaacctgttctttatatgcgacctattgattag
>KrE1_psbC
atgcctctcatttattctttaaaacgacgaactctagttggatccaggtatgcttggtgg
tcaggtaatgcaagattcattgagcttagcggtaaattcctaggtgctcatcttgtgcat
acagctcttattctagtttgggctggaacaatgtctttgtttgaattatctcacttcatc
ccagaaaaacctctatatgagcaaggctttattcttcttcctcacttgtttgaattgggt
ggcactttatattcacacttcataataagcatcctacatctcatctctgcaggtatccta
gctcttggaggtatttatcatgctatagttggtgctgaaagattagaagaaacaagcttc
agtgctctatttgcttatgggttacaagatagattccgtattactgctatacttggatca
catttacttactctaggaattggagcagccttactctttgcaaaagcagtctatttaggt
ggtctttatgatacatgggcttgtggtggtggagacatgagattgattgaaagggtagag
cttggtttaaatccttatctactagctcagtacttacttcgtgctccatttgggtcatca
ggatggataattagcatcaataacatggaagatctcattggaggtcattattgggtctca
ttttatctaattctaggcagctgttggcatattcaaacaagagctcttacaattacaaca
agagcttttacatggtcagcagaagcttacctctcttatactttatcagctgtaacttta
tgtggttctatcacagcattattttcttggttcaataatactgcatatccaagtgaattc
tatggacctactggggcagaagcctctcaagcacaaagctttacattcctcatacgagat
tggaaattgggtataaagataacttcatccgaaggtcctacagctcttggtaagtatctc
atgagatctccaacaggtgaaataatatttggtggtgaaagtatgagattctggtctatg
caagcacattggcttgaagctctcagaacatcatttggactggacttatctaagattcaa
tctgatattcaaacttggcaagaaagaagagctgcagagtatatgacccatgcaccacta
gggagcttaaattctgttggtggtgttgctactgaaattaactctgtaaactatgtatct
cctagatcttggttaacatcatctcactggttcttagcattctttattttagttggtcat
tggtggcatggagcaagagcaagagcttcagcattatctagtgaaacaggcttatctcga
gtatatgaacctgttctttatatgcgacctattgattag
>KrE10_psbC
atgcctcttcttcattctttaaaacgacgaactctagttggatccaggtatgcttggtgg
tcaggtaatgcaagattcattgagcttagcggtaaattcctaggtgctcatcttgtgcat
acagctcttattctagtttgggctggaacaatgtctttgtttgaattatctcacttcatc
ccagaaaaacctctatatgagcaaggctttattcttctacctcatttgtttgaactggga
ggcaatctatattcacacttcataataagcatcctacatctcatctctgcaggtatccta
gctcttggaggtatttatcatgctatagttggtgctgaaagattagaagaaacaagcttc
agtgctctatttgcttatgggttacaagatagattccgtattactgctatacttggatca
catttacttactctaggaattggagcaggcttactctttgcaaaagcagtctatttaggt
ggtctttatgatacttgggcttgtggtggtggagacatgagattgattgaaagagtagag
cttggtttaaatccttatctactagctcagtacttacttcgtgctccatttgggtcatca
ggatggataattagcatcaataacatggaagatctcattggaggtcattattgggtctca
ttttatctaattctaggcagctgttggcatattcaaacaagagctcttacaattacaaca
agagcttttacatggtcagcagaagcttacctctcttatactttatcagctgtaacttta
tgtggttctatcacagcattattttcttggttcaataatactgcatatccaagtgaattc
tatggacctactggagcagaagcctctcaagcacaaagctttacattccttatacgagat
tggaaattgggtataaagataagttcatccgaaggtcctacagctcttggtaagtatctc
atgagatctccaacaggtgaaataatatttggtggtgaaagtatgagattctggtctatg
caagcacattggcttgaagctctcagaacatcatttggactggacttatctaagattcaa
tctgatattcaaacttggcaagaaagaagagctgcagagtatatgacccatgcaccacta
gggagcttaaattctgttggtggtgttgctactgaaattaactctgtaaactatgtatct
cctagatcttggttaacatcatctcactggttcttagcattctttattttagttggtcat
tggtggcatggagcaagagcaagagcttcagcattatctagtgaaacaggcttatctcga
gtatatgaacctgttctttatatgcgacctattgattag

>KrE11_psbC
atgcctctcatttattctttaaaacgacgaactctagttggatccaggtatgcttggtgg
tcaggtaatgcaagattcattgagcttagcggtaaattcctaggtgctcatcttgtgcat
acagctcttattctagtttgggctggaacaatgtctttgtttgaattatctcacttcatc
ccagaaaaacctctatatgagcaaggctttattcttctacctcatttgtttgaactggga
ggcaatctatattcacacttcataataagcatcctacatctcatctctgcaggtatccta
gctcttggaggtatttatcatgctatagttggtgctgaaagattagaagaaacaagctat
agctccctatttgcttatgggttacaagatagattccgtattactgctatacttggatca
catttacttactctaggaattggagcagccttactctttgcaaaagcagtctatttaggt
ggtctttatgatacatgggcttgtggtggtggagacatgagattgattgaaagggtagag
cttggtttaaatccttatctactagctcagtacttacttcgtgctccatttgggtcatca
ggatggataattagcatcaataacatggaagatctcattggaggtcattattgggtctca
ttttatctaattctaggcagctgttggcatattcaaacaagagctcttacaattacaaca
agagcttttacatggtcagcagaagcttacctctcttacactctatcagctgtaacttta
tgtggttctatcacaagattattttcttggtacaataatactgcatatccaagtgaattc
tatggacctactggggcagaagcctctcaagcacaaagctttacattcctcatacgagat
tggaaattgggtataaagataacttcatccgaaggtcctacagctcttggtaagtatctc
atgagatctccaacaggtgaaataatatttggtggtgaaagtatgagattctggtctatg
caagcacattggcttgaagctctcagaacatcatttggactggacttatctaagattcaa
tctgatattcaaacttggcaagaaagaagagctgcagagtatatgacccatgcaccacta
gggagcttaaattctgttggtggtgttgctactgaaattaactctgtaaactatgtatct
cctagatcttggttaacctcctctcattggttcttggccttctttattttagttggtcat
tggtggcatggagcaagagcaagagcttcagcattatctagtgaaacaggcttatctcga
gtatatgaacctgttctttatatgcgacctattgattag
>KrE12_psbC
atgcctctcatttattctttaaaacgacgaactctagttggatccaggtatgcttggtgg
tcaggtaatgcaagattcattgagcttagcggtaaattcctaggtgctcatcttgtgcat
acagctcttattctagtttgggctggaacaatgtctttgtttgaattatctcacttcatc
ccagaaaaacctctatatgagcaaggctttattcttctacctcatttgtttgaactggga
ggcaatctatattcacacttcataataagcatcctacatctcatctctgcaggtatccta
gctcttggaggtatttatcatgctatagttggtgctgaaagattagaagaaacaagcttc
agtgctctatttgcttatgggttacaagatagattccgtattactgctatacttggatca
catttacttactctaggaattggagcagccttactctttgcaaaagcagtctatttaggt
ggtctttatgatacatgggcttgtggtggtggagacatgagattgattgaaagggtagag
cttggtttaaatccttatctactagctcagtacttacttcgtgctccatttgggtcatca
ggatggataattagcatcaataacatggaagatctcattggaggtcattattgggtctca
ttttatctaattctaggcagctgttggcatattcaaacaagagctcttacaattacaaca
agagcttttacatggtcagcagaagcttacctctcttatactttatcagctgtaacttta
tgtggttctatcacagcattattttcttggttcaataatactgcatatccaagtgaattc
tatggacctactggggcagaagcctctcaagcacaaagctttacattcctcatacgagat
tggaaattgggtataaagataacttcatccgaaggtcctacagctcttggtaagtatctc
atgagatctccaacaggtgaaataatatttggtggtgaaagtatgagattctggtctatg
caagcacattggcttgaagctctcagaacatcatttggactggacttatctaagattcaa
tctgatattcaaacttggcaagaaagaagagctgcagagtatatgacccatgcaccacta
gggagcttaaattctgttggtggtgttgctactgaaattaactctgtaaactatgtatct
cctagatcttggttaacatcatctcactggttcttagcattctttattttagttggtcat
tggtggcatggagcaagagcaagagcttcagcattatctagtgaaacaggcttatctcga
gtatatgaacctgttctttatatgcgacctattgattag
>KrE13_psbC
atgcctctcatttattctttaaaacgacgaactctagttggatccaggtatgcttggtgg
tcaggtaatgcaagattcattgagcttagcggtaaattcctaggtgctcatcttgtgcat
acagctcttattctagtttgggctggaacaatgtctttgtttgaattatctcacttcatc
ccagaaaaacctctatatgagcaaggctttattcttcttcctcacttgtttgaattgggt
ggcactttatattcacacttcataataagcatcctacatctcatctctgcaggtatccta
gctcttggaggtatttatcatgctatagttggtgctgaaagattagaagaaacaagcttc
agtgctctatttgcttatgggttacaagatagattccgtattactgctatacttggatca
catttacttactctaggaattggagcagccttactctttgcaaaagcagtctatttaggt

ggtctttatgatacatgggcttgtggtggtggagacatgagattgattgaaagggtagag
cttggtttaaatccttatctactagctcagtacttacttcgtgctccatttgggtcatca
ggatggataattagcatcaataacatggaagatctcattggaggtcattattgggtctca
ttttatctaattctaggcagctgttggcatattcaaacaagagctcttacaattacaaca
agagcttttacatggtcagcagaagcttacctctcttatactttatcagctgtaacttta
tgtggttctatcacagcattattttcttggttcaataatactgcatatccaagtgaattc
tatggacctactggggcagaagcctctcaagcacaaagctttacattcctcatacgagat
tggaaattgggtataaagataacttcatccgaaggtcctacagctcttggtaagtatctc
atgagatctccaacaggtgaaataatatttggtggtgaaagtatgagattctggtctatg
caagcacattggcttgaagctctcagaacatcatttggactggacttatctaagattcaa
tctgatattcaaacttggcaagaaagaagagctgcagagtatatgacccatgcaccacta
gggagcttaaattctgttggtggtgttgctactgaaattaactctgtaaactatgtatct
cctagatcttggttaacatcatctcactggttcttagcattctttattttagttggtcat
tggtggcatggagcaagagcaagagcttcagcattatctagtgaaacaggcttatctcga
gtatatgaacctgttctttatatgcgacctattgattag
>KrE14_psbC
atgcctctcatttattctttaaaacgacgaactctagttggatccaggtatgcttggtgg
tcaggtaatgcaagattcattgagcttagcggtaaattcctaggtgctcatcttgtgcat
acagctcttattctagtttgggctggaacaatgtctttgtttgaattatctcacttcatc
ccagaaaaacctctatatgagcaaggctttattcttcttcctcacttgtttgaattgggt
ggcactttatattcacacttcataataagcatcctacatctcatctctgcaggtatccta
gctcttggaggtatttatcatgctatagttggtgctgaaagattagaagaaacaagcttc
agtgctctatttgcttatgggttacaagatagattccgtattacagctatacttggttca
catttacttactctaggaattggagcagccttactctttgcaaaagcagtctatttaggt
ggtctttatgatacatgggcttgtggtggtggagacatgagattgattgaaagggtagag
cttggtttaaatccttatctactagctcaatacttacttcgtgctccatttgggtcatca
ggatggataattagcatcaataacatggaagatctcattggaggtcattattgggtctca
ttttatctaattctaggtagctgttggcatattcaaacaagagctcttacaatcacaaca
agagcttttacatggtcagcagaagcttacctctcttatactttatcagctgtaacttta
tgtggttctatcacagcattattttcttggttcaataatactgcatatccaagtgaattc
tatggacctactggggcagaagcctctcaagcacaaagctttacattcctcatacgagat
tggaaattgggtataaagataacttcatccgaaggtcctacagctcttggtaagtatctc
atgagatctccaacaggtgaaataatatttggtggtgaaagtatgagattctggtctatg
caagcacattggcttgaagctctcagaacatcatttggattagacttatctaagattcaa
tctgatattcaaacttggcaagaaagaagagctgcagagtatatgacccatgcaccacta
gggagcttaaattctgttggtggtgttgctactgaaattaactctgtaaactatgtatct
cctagatcttggttaacatcatctcactggttcttagcattctttattttagttggtcat
tggtggcatggagcaagagcaagagcttcagcattatctagtgaaacaggcttatctcga
gtatatgaacctgttctttatatgcgacctattgattag
>KrE15_psbC
atgcctctcatttattctttaaaacgacgaactctagttggatccaggtatgcttggtgg
tcaggtaatgcaagattcattgagcttagcggtaaattcctaggtgctcatcttgtgcat
acagctcttattctagtttgggctggaacaatgtctttgtttgaattatctcacttcatc
ccagaaaaacctctatatgagcaaggctttattcttcttcctcacttgtttgaattgggt
ggcactttatattcacacttcataataagcatcctacatctcatctctgcaggtatccta
gctcttggaggtatttatcatgctatagttggtgctgaaagattagaagaaacaagctat
agctccctatttgcttatgggttacaagatagattccgtattactgctatacttggatca
catttacttactctaggaattggagcagccttactctttgcaaaagcagtctatttaggt
ggtctttatgatacatgggcttgtggtggtggagacatgagattgattgaaagggtagag
cttggtttaaatccttatctactagctcagtacttacttcgtgctccatttgggtcatca
ggatggataattagcatcaataacatggaagatctcattggaggtcattattgggtctca
ttttatctaattctaggcagctgttggcatattcaaacaagagctcttacaatcacaaca
agagcttttacatggtcagcagaagcttacctctcttacactctatcagctgtaacttta
tgtggttctatcacagcattattttcttggttcaataatactgcatatccaagtgaattc
tatggacctactggggcagaagcctctcaagcacaaagctttacattcctcatacgagat
tggaaattgggtataaagataacttcatccgaaggtcctacagctcttggtaagtatctc
atgagatctccaacaggtgaaataatatttggtggtgaaagtatgagattctggtctatg

caagcacattggcttgaagctctcagaacatcatttggactggacttatctaagattcaa
tctgatattcaaacttggcaagaaagaagagctgcagagtatatgacccatgcaccacta
gggagcttaaattctgttggtggtgttgctactgaaattaactctgtaaactatgtatct
cctagatcttggttaacatcatctcactggttcttagcattctttattttagttggtcat
tggtggcatggagcaagagcaagagcttcagcattatctagtgaaacaggcttatctcga
gtatatgaacctgttctttatatgcgacctattgattag
>KrE2_psbC
atgcctctcatttattctttaaaacgacgaactctagttggatccaggtatgcttggtgg
tcaggtaatgcaagattcattgagcttagcggtaaattcctaggtgctcatcttgtgcat
acagctcttattctagtttgggctggaacaatgtctttgtttgaattatctcacttcatc
ccagaaaaacctctatatgagcaaggctttattcttcttcctcacttgtttgaattgggt
ggcactttatattcacacttcataataagcatcctacatctcatctctgcaggtatccta
gctcttggaggtatttatcatgctatagttggtgctgaaagattagaagaaacaagcttc
agtgctctatttgcttatgggttacaagatagattccgtattactgctatacttggatca
catttacttactctaggaattggagcaggcttactctttgcaaaagcagtctatttaggt
ggtctttatgatacatgggcttgtggtggtggagacatgagattgattgaaagggtagag
cttggtttaaatccttatctactagctcagtacttacttcgtgctccatttgggtcatca
ggatggataattagcatcaataacatggaagatctcattggaggtcattattgggtctca
ttttatctaattctaggcagctgttggcatattcaaacaagagctcttacaattacaaca
agagcttttacatggtcagcagaagcttacctctcttatactttatcagctgtaacttta
tgtggttctatcacagcattattttcttggttcaataatactgcatatccaagtgaattc
tatggacctactggggcagaagcctctcaagcacaaagctttacattcctcatacgagat
tggaaattgggtataaagataacttcatccgaaggtcctacagctcttggtaagtatctc
atgagatctccaacaggtgaaataatatttggtggtgaaagtatgagattctggtctatg
caagcacattggcttgaagctctcagaacatcctttggactggacttatctaagattcaa
tctgatattcaaacttggcaagaaagaagagctgcagagtatatgacccatgcaccacta
gggagcttaaattctgttggtggtgttgctactgaaattaactctgtaaactatgtatct
cctagatcttggttaacatcatctcactggttcttagcattctttattttagttggtcat
tggtggcatggagcaagagcaagagcttcagcattatctagtgaaacaggcttatctcga
gtatatgaacctgttctttatatgcgacctattgattag
>KrE3_psbC
atgcctctcatttattctttaaaacgacgaactctagttggatccaggtatgcttggtgg
tcaggtaatgcaagattcattgagcttagcggtaaattcctaggtgctcatcttgtgcat
acagctcttattctagtttgggctggaacaatgtctttgtttgaattatctcacttcatc
ccagaaaaacctctatatgagcaaggctttattcttcttcctcacttgtttgaattggga
ggcactttatattcacacttcataataagcatcctacatctcatctctgcaggtatccta
gctcttggaggtatttatcatgctatagttggtgctgaaagattagaagaaacaagcttc
agtgctctatttgcttatgggttacaagatagattccgtattacagctatacttggttca
catttacttactctaggaattggagcaggcttactctttgcaaaagcagtctatttaggt
ggtctttatgatacatgggcttgtggtggtggagacatgagattgattgaaagggtagag
cttggtttaaatccttatctactagctcagtacttacttcgtgctccatttgggtcatca
ggatggataattagcatcaataacatggaagatctcattggaggtcattattgggtctca
ttttatctaattctaggcagctgttggcatattcaaacaagagctcttacaattacaaca
agagcttttacatggtcagcagaagcttacctctcttatactttatcagctgtaacttta
tgtggttctatcacagcattattttcttggttcaataatactgcatatccaagtgaattc
tatggacctactggggcagaagcctctcaagcacaaagctttacattcctcatacgagat
tggaaattgggtataaagataacttcatccgaaggtcctacagctcttggtaagtatctc
atgagatctccaacaggtgaaataatatttggtggtgaaagtatgagattctggtctatg
caagcacattggcttgaagctctcagaacatcatttggactggacttatctaagattcaa
tctgatattcaaacttggcaagaaagaagagctgcagagtatatgacccatgcaccacta
gggagcttaaattctgttggtggtgttgctactgaaattaactctgtaaactatgtatct
cctagatcttggttaacatcatctcactggttcttagcattctttattttagttggtcat
tggtggcatggagcaagagcaagagcttcagcattatctagtgaaacaggcttatctcga
gtatatgaacctgttctttatatgcgacctattgattag
>KrE4_psbC
atgcctctcatttattctttaaaacgacgaactctagttggatccaggtatgcttggtgg
tcaggtaatgcaagattcattgagcttagcggtaaattcctaggtgctcatcttgtgcat

acagctcttattctagtttgggctggaagaatgtctttgtttgaattatctcacttcatc
ccagaaaaacctctatatgagcaaggctttattcttcttcctcacttgtttgaattgggt
ggcactttatattcacacttcataataagcatcctacatctcatctctgcaggtatccta
gctcttggaggtatttatcatgctatagttggtgctgaaagattagaagaaacaagcttc
agtgctctatttgcttatgggttacaagatagattccgtattactgctatacttggatca
catttacttactctaggaattggagcagccttactctttgcaaaagcagtctatttaggt
ggtctttatgatacttgggcttgtggtggtggagacatgagattgattgaaagggtagag
cttggtttaaatccttatctactagctcagtacttacttcgtgctccatttgggtcatca
ggatggataattagcataaataacatggaagatctaattggaggccattattgggtctca
ttttatctaattctaggcagctgttggcatattcaaacaagagctcttacaattacaaca
agagcttttacatggtcagcagaagcttacctctcttatactttatcagctgtaacttta
tgtggttctatcacagcattattttcttggttcaataatactgcatatccaagtgaattc
tatggacctactggggcagaagcctctcaagcacaaagctttacattcctcatacgagat
tggaaattgggtataaagataacttcatccgaaggtcctacagctcttggtaagtatctc
atgagatctccaacaggtgaaataatatttggtggtgaaagtatgagattctggtctatg
caagcacattggcttgaagctctcagaacatcatttggactggacttatctaagattcaa
tctgatattcaaacttggcaagaaagaagagctgcagagtatatgacccatgcaccacta
gggagcttaaattctgttggtggtgttgctactgaaattaactctgtaaactatgtatct
cctagatcttggttaacatcatctcactggttcttagcattctttattttagttggtcat
tggtggcatggagcaagagcaagagcttcagcattatctagtgaaacaggtttatctcga
gtatatgaacctgttctttatatgcgacctattgattag
>KrE5_psbC
atgcctcttcttcattctttaaaacgacgaactctagttggatccaggtatgcttggtgg
tcaggtaatgcaagattcattgagcttagcggtaaattcctaggtgctcatcttgtgcat
acagctcttattctagtttgggctggaacaatgtctttgtttgaattatctcacttcatc
ccagaaaaacctctatatgagcaaggctttattcttcttcctcacttgtttgaattgggt
ggcactttatattcacacttcataataagcatcctacatctcatctctgcaggtatccta
gctcttggaggtatttatcatgctatagttggtgctgaaagattagaagaaacaagcttc
agtgctctatttgcttatgggttacaagatagattccgtattactgctatacttggatca
catttacttactctaggaattggagcagccttactctttgcaaaagcagtctatttaggt
ggtctttatgatacatgggcttgtggtggtggagacatgagattgattgaaagggtagag
cttggtttaaatccttatctactagctcagtacttacttcgtgctccatttgggtcatca
ggatggataattagcatcaataacatggaagatctcattggaggtcattattgggtctca
ttttatctaattctaggcagctgttggcatattcaaacaagagctcttacaattacaaca
agagcttttacatggtcagcagaagcttacctctcttatactttatcagctgtaacttta
tgtggttctatcacagcattattttcttggttcaataatactgcatatccaagtgaattc
tatggacctactggggcagaagcctctcaagcacaaagctttacattcctcatacgagat
tggaaattgggtataaagataacttcatccgaaggtcctacagctcttggtaagtatctc
atgagatctccaacaggtgaaataatatttggtggtgaaagtatgagattctggtctatg
caagcacattggcttgaagctctcagaacatcatttggactggacttatctaagattcaa
tctgatattcaaacttggcaagaaagaagagctgcagagtatatgacccatgcaccacta
gggagcttaaattctgttggtggtgttgctactgaaattaactctgtaaactatgtatct
cctagatcttggttaacatcatctcactggttcttagcattctttattttagttggtcat
tggtggcatggagcaagagcaagagcttcagcattatctagtgaaacaggcttatctcga
gtatatgaacctgttctttatatgcgacctattgattag
>KrE6_psbC
atgcctctcatttattctttaaaacgacgaactctagttggatccaggtatgcttggtgg
tcaggtaatgcaagattcattgagcttagcggtaaattcctaggtgctcatcttgtgcat
acagctcttattctagtttgggctggaacaatgtctttgtttgaattatctcacttcatc
ccagaaaaacctctatatgagcaaggctttattcttcttcctcacttgtttgaattgggt
ggcactttatattcacacttcataataagcatcctacatctcatctctgcaggtatccta
gctcttggaggtatttatcatgctatagttggtgctgaaagattagaagaaacaagcttc
agtgctctatttgcttatgggttacaagatagattccgtattacagctatacttggttca
catttacttactctaggaattggagcaggcttactctttgcaaaagcagtctatttaggt
ggtctttatgatacatgggcttgtggtggtggagacatgagattgattgaaagggtagag
cttggtttaaatccttatctactagctcagtacttacttcgtgctccatttgggtcatca
ggatggataattagcatcaataacatggaagatctcattggaggtcattattgggtctca

ttttatctaattctaggcagctgttggcatattcaaacaagagctcttacaattacaaca
agagcttttacatggtcagcagaagcttacctctcttatactttatcagctgtaacttta
tgtggttctatcacagcattattttcttggttcaataatactgcatatccaagtgaattc
tatggacctactggggcagaagcctctcaagcacaaagctttacattcctcatacgagat
tggaaattgggtataaagataacttcatccgaaggtcctacagctcttggtaagtatctc
atgagatctccaacaggtgaaataatatttggtggtgaaagtatgagattctggtctatg
caagcacattggcttgaagctctcagaacatcatttggactggacttatctaagattcaa
tctgatattcaaacttggcaagaaagaagagctgcagagtatatgacccatgcaccacta
gggagcttaaattctgttggtggtgttgctactgaaattaactctgtaaactatgtatct
cctagatcttggttaacatcatctcactggttcttagcattctttattttagttggtcat
tggtggcatggagcaagagcaagagcttcagcattatctagtgaaacaggcttatctcga
gtatatgaacctgttctttatatgcgacctattgattag
>KrE7_psbC
atgcctctcatttattctttaaaacgacgaactctagttggatccaagtatgcttggtgg
tcaggtaatgcaagattcattgagctaagcggtaaattcctaggtgctcatcttgtgcat
acagctcttattctagtttgggctggaacaatgtctttgtttgaattatctcacttcatt
ccagaaaaacctctatatgagcaaggctttattcttctacctcatttgtttgaactggga
ggcaatctatattcacacttcataataagcatcctacatctcatctctgcaggtatctta
gctcttggaggtatttatcatgctatagttggtggtgaaagactagaagaaacaagctat
agctccctatttgcttatgggttacaagatagattccgtattacagctatacttggttca
catttacttactctaggaattggagcagccttactctttgcaaaagcagtctatttaggt
ggtctttatgatacatgggcttgtggtggtggagacatgagattgattgaaagagtagag
cttggtttaaatccttatctactagctcagtacttacttcgtgctccatttgggtcatca
ggatggataattagcatcaataacatggaagatctcattggaggccattattgggtctca
ttttatctaattctaggtagctgttggcatattcaaacaagagctcttacaatcacaaca
agagcttttacatggtcagcagaagcttacctctcttatactttatcagctgtaacttta
tgtggttctatcacagcattattttcttggttcaataatactgcatatccaagtgaattc
tatggacctactggggcagaagcctctcaagcacaaagctttacattcctcatacgagat
tggaaattgggtataaagataacttcatccgaaggtcctacagctcttggtaagtatctc
atgagatctccaacaggtgaaataatatttggtggtgaaagtatgagattctggtctatg
caagcacattggcttgaagctctcagaacatcatttggactggacttatctaagattcaa
tctgatattcaaacttggcaagaaagaagagctgcagagtatatgacccatgcaccacta
gggagcttaaattctgttggtggtgttgctactgaaattaactctgtaaactatgtatct
cctagatcttggttaacatcatctcactggttcttagcattctttattttagttggtcat
tggtggcatggagcaagagcaagagcttcagcattatctagtgaaacaggcttatctcga
gtatatgaacctgttctttatatgcgacctattgattag
>KrE8_psbC
atgcctctcattcattctttaaaacgacgaactctagttggatccaggtatgcttggtgg
tcaggtaatgcaagattcattgagcttagcggtaaattcctaggtgctcatcttgtgcat
acagctcttattctagtttgggctggaacaatgtctttgtttgaattatctcacttcatc
ccagaaaaacctctatatgagcaaggctttattcttcttcctcacttgtttgaattgggt
ggcactttatattcacacttcataataagcatcctacatctcatctctgcaggtatccta
gctcttggaggtatttatcatgctatagttggtgctgaaagattagaagaaacaagcttc
agtgctctatttgcttatgggttacaagatagattccgtattactgctatacttggatca
catttacttactctaggaattggagcagccttactctttgcaaaagcagtctatttaggt
ggtctttatgatacatgggcttgtggtggtggagacatgagattgattgaaagggtagag
cttggtttaaatccttatctactagctcagtacttacttcgtgctccatttgggtcatca
ggatggataattagcatcaataacatggaagatctcattggaggtcattattgggtctca
ttttatctaattctaggcagctgttggcatattcaaacaagagctcttacaattacaaca
agagcttttacatggtcagcagaagcttacctctcttatactttatcagctgtaacttta
tgtggttctatcacagcattattttcttggttcaataatactgcatatccaagtgaattc
tatggacctactggggcagaagcctctcaagcacaaagctttacattcctcatacgagat
tggaaattgggtataaagataacttcatccgaaggtcctacagctcttggtaagtatctc
atgagatctccaacaggtgaaataatatttggtggtgaaagtatgagattctggtctatg
caagcacattggcttgaagctctcagaacatcatttggactggacttatctaagattcaa
tctgatattcaaacttggcaagaaagaagagctgcagagtatatgacccatgcaccacta
gggagcttaaattctgttggtggtgttgctactgaaattaactctgtaaactatgtatct

cctagatcttggttaacatcatctcactggttcttagcattctttattttagttggtcat
tggtggcatggagcaagagcaagagcttcagcattatctagtgaaacaggcttatctcga
gtatatgaacctgttctttatatgcgacctattgattag
>KrE9_psbC
atgcctctcatttattctttaaaacgacgaactctagttggatccaggtatgcttggtgg
tcaggtaatgcaagattcattgagcttagcggtaaattcctaggtgctcatcttgtgcat
acagctcttattctagtttgggctggaacaatgtctttgtttgaattatctcacttcatc
ccagaaaaacctctatatgagcaaggctttattcttcttcctcacttgtttgaattgggt
ggcactttatattcacacttcataataagcatcctacatctcatctctgcaggtatccta
gctcttggaggtatttatcatgctatagttggtgctgaaagattagaagaaacaagcttc
agtgctctatttgcttatgggttacaagatagattccgtattactgctatacttggatca
catttacttactctaggaattggagcaggcttactctttgcaaaagcagtctatttaggt
ggtctttatgatacatgggcttgtggtggtggagacatgagattgattgaaagggtagag
cttggtttaaatccttatctactagctcagtacttacttcgtgctccatttgggtcatca
ggatggataattagcatcaataacatggaagatctcattggaggtcattattgggtctca
ttttatctaattctaggcagctgttggcatattcaaacaagagctcttacaattacaaca
agagcttttacatggtcagcagaagcttacctctcttatactttatcagctgtaacttta
tgtggttctatcacagcattattttcttggttcaataatactgcatatccaagtgaattc
tatggacctactggggcagaagcctctcaagcacaaagctttacattccttatacgagat
tggaaattgggtataaagataacttcatccgaaggtcctacagctcttggtaagtatctc
atgagatctccaacaggtgaaataatatttggtggtgaaagtatgagattctggtctatg
caagcacattggcttgaagctctcagaacatcatttggactggacttatctaagattcaa
tctgatattcaaacttggcaagaaagaagagctgcagagtatatgacccatgcaccacta
gggagcttaaattctgttggtggtgttgctactgaaattaactctgtaaactatgtatct
cctagatcttggttaacatcatctcactggttcttagcattctttattttagttggtcat
tggtggcatggagcaagagcaagagcttcagcattatctagtgaaacaggcttatctcga
gtatatgaacctgttctttatatgcgacctattgattag
>Mz10_psbC
atgcctctcatttattctttaaaacgacgaactctagttggatccaagtatgcttggtgg
tcaggtaatgcaagattcattgagcttagcggtaaattcctaggtgctcatcttgtgcat
acagctcttattctagtttgggctggaacaatgtctttgtttgaattatctcacttcatc
ccagaaaaacctctatatgagcaaggctttattcttcttcctcacttgtttgaattgggt
ggcactttatattcacacttcataataagcatcctacatctcatctctgcaggtatccta
gctcttggaggtatttatcatgctatagttggtggtgaaagattagaagaaacaagctac
agtgctctatttgcttatgggttacaagatagattccgtattactgctatacttggatca
catttacttactctaggaattggagcagccttactctttgcaaaagcagtctatttaggt
ggtctttatgatacatgggcttgtggtggtggagacatgagattgattgaaagagtagag
cttggtttaaatccttatctactagctcaatacttacttcgtgctccatttggttcatca
ggatggataattagcatcaataacatggaagatctaattggaggccattattgggtctca
ttttatctaattctaggtagctgttggcatattcaaacaagagctcttacaattacaaca
agagcttttacatggtcagcagaagcttacctctcttatactttatcagctgtaacttta
tgtggttctatcacaagtttgttttcttggtacaataatactgcatatccaagtgaattc
tatggacctactggagcagaagcctctcaagcacaaagctttacattccttatacgagat
tggaaattgggtataaagataacttcatccgaaggtcctacagctcttggtaagtatctc
atgagatctccaacaggtgaaataatatttggtggtgaaagtatgagattctggtctatg
caagcacattggcttgaagctctcagaacatcctttggattagacttatctaagattcaa
tctgatattcaaacttggcaagaaagaagagctgcagagtatatgacccatgcaccacta
gggagcttaaattctgttggtggtgttgctactgaaattaactctgtaaactatgtatct
cctagatcttggttaacctcctctcactggttcttagcattctttattttagttggtcat
tggtggcatggagcaagagcaagagcttcagcattatctagtgaaacaggcttatctcga
gtatatgaacctgttctttatatgcgacctattgattag
>Mz5_psbC
atgcctctcatttattctttaaaacgacgaactctagttggatccaggtatgcttggtgg
tcaggtaatgcaagattcattgagcttagcggtaaattcctaggtgctcatcttgtgcat
acagctcttattctagtttgggctggaacaatgtctttgtttgaattatctcacttcatc
ccagaaaaacctctatatgagcaaggctttattcttcttcctcacttgtttgaattgggt
ggcactttatattcacacttcataataagcatcctacatctcatctctgcaggtatccta

gctcttggaggtatttatcatgctatagttggtgctgaaagattagaagaaacaagcttc
agtgctctatttgcttatgggttacaagatagattccgtattactgctatacttggatca
catttacttactctaggaattggagcagccttactctttgcaaaagcagtctatttaggt
ggtctttatgatacatgggcttgtggtggtggagacatgagattgattgaaagggtagag
cttggtttaaatccttatctactagctcagtacttacttcgtgctccatttgggtcatca
ggatggataattagcatcaataacatggaagatctcattggaggtcattattgggtctca
ttttatctaattctaggcagctgttggcatattcaaacaagagctcttacaattacaaca
agagcttttacatggtcagcagaagcttacctctcttatactttatcagctgtaacttta
tgtggttctatcacagcattattttcttggttcaataatactgcatatccaagtgaattc
tatggacctactggggcagaagcctctcaagcacaaagctttacattccttatacgagat
tggaaattgggtataaagataacttcatccgaaggtcctacagctcttggtaagtatctc
atgagatctccaacaggtgaaataatatttggtggtgaaagtatgagattctggtctatg
caagcacattggcttgaagctctcagaacatcatttggactggacttatctaagattcaa
tctgatattcaaacttggcaagaaagaagagctgcagagtatatgacccatgcaccacta
gggagcttaaattctgttggtggtgttgctactgaaattaactctgtaaactatgtatct
cctagatcttggttaacatcatctcactggttcttagcattctttattttagttggtcat
tggtggcatggagcaagagcaagagcttcagcattatctagtgaaacaggcttatctcga
gtatatgaacctgttctttatatgcgacctattgattag
>Mz6_psbC
atgcctctcatttattctttaaaacgacgaactctagttggatccaggtatgcttggtgg
tcaggtaatgcaagattcattgagcttagcggtaaattcctaggtgctcatcttgtgcat
acagctcttattctagtttgggctggaacaatgtctttgtttgaattatctcacttcatc
ccagaaaaacctctatatgagcaaggctttattcttcttcctcacttgtttgaattgggt
ggcactttatattcacacttcataataagcatcctacatctcatctctgcaggtatccta
gctcttggaggtatttatcatgctatagttggtgctgaaagattagaagaaacaagcttc
agtgccctatttgcttatgggttacaagatagattccgtattactgctatacttggatca
catttacttactctaggaattggagcagccttactctttgcaaaagcagtctatttaggt
ggtctttatgatacatgggcttgtggtggtggagacatgagattgattgaaagggtagag
cttggtttaaatccttatctactagctcagtacttacttcgtgctccatttgggtcatca
ggatggataattagcatcaataacatggaagatctcattggaggtcattattgggtctca
ttttatctaattctaggcagctgttggcatattcaaacaagagctcttacaattacaaca
agagcttttacatggtcagcagaagcttacctctcttatactttatcagctgtaacttta
tgtggttctatcacagcattattttcttggttcaataatactgcatatccaagtgaattc
tatggacctactggggcagaagcctctcaagcacaaagctttacattcctcatacgagat
tggaaattgggtataaagataacttcatccgaaggtcctacagctcttggtaagtatctc
atgagatctccaacaggtgaaataatatttggtggtgaaagtatgagattctggtctatg
caagcacattggcttgaagctctcagaacatcatttggactggacttatctaagattcaa
tctgatattcaaacttggcaagaaagaagagctgcagagtatatgacccatgcaccacta
gggagcttaaattctgttggtggtgttgctactgaaattaactctgtaaactatgtatct
cctagatcttggttaacatcatctcactggttcttagcattctttattttagttggtcat
tggtggcatggagcaagagcaagagcttcagcattatctagtgaaacaggcttatctcga
gtatatgaacctgttctttatatgcgacctattgattag
>Mz7_psbC
atgcctctcatttattctttaaaacgacgaactctagttggatccaggtatgcttggtgg
tcaggtaatgcaagattcattgagctaagtggtaaattcctaggtgctcatcttgtgcat
acagctcttattctagtttgggctggaacaatgtctttgtttgaattatctcacttcatc
ccagaaaaacctctatatgagcaaggctttattcttcttcctcacttgtttgaattgggt
ggcactttatattcacacttcataataagcatcctacatctcatctctgcaggtatccta
gctcttggaggtatttatcatgctatagttggtgctgaaagattagaagaaacaagcttc
agtgctctatttgcttatgggttacaagatagattccgtattactgctatacttggatca
catttacttactctaggaattggagcagccttactctttgcaaaagcagtctatttaggt
ggtctttatgatacttgggcttgtggtggtggagacatgagattgattgaaagggtagag
cttggtttaaatccttatctactagctcagtacttacttcgtgctccatttgggtcatca
ggatggataattagcatcaataacatggaagatctcattggaggtcattattgggtctca
ttttatctaattctaggcagctgttggcatattcaaacaagagctcttacaattacaaca
agagcttttacatggtcagcagaagcttacctctcttatactttatcagctgtaacttta
tgtggttctatcacagcattattttcttggttcaataatactgcatatccaagtgaattc

tatggacctactggggcagaagcctctcaagcacaaagctttacattcctcatacgagat
tggaaattgggtataaagataagttcatccgaaggtcctacagctcttggtaagtatctc
atgagatctccaacaggtgaaataatatttggtggtgaaagtatgagattctggtctatg
caagcacattggcttgaagctctcagaacatcatttggactggacttatctaagattcaa
tctgatattcaaacttggcaagaaagaagagctgcagagtatatgacccatgcaccacta
gggagcttaaattctgttggtggtgttgctactgaaattaactctgtaaactatgtatct
cctagatcttggttaacatcatctcactggttcttagcattctttattttagttggtcat
tggtggcatggagcaagatcaagagctgcagcattgtctagtgaaacaggcttatctcga
gtatatgaacctgttctttatatgcgacctattgattag
>Mz8_psbC
atgcctctcatttattctttaaaacgacgaactctagttggatccaagtatgcttggtgg
tcaggtaatgcaagattcattgagcttagcggtaaattcctaggtgctcatcttgtgcat
acagctcttattctagtttgggctggaagaatgtctttgtttgaattatctcacttcatt
ccagaaaaacctctatatgagcaaggctttattcttctacctcatttgtttgaactggga
ggcaatctatattcacacttcataataagcatcctacatctcatctctgcaggtatctta
gctcttggaggtatttatcatgctatagttggtggtgaaagattagaagaaacaagcttc
agtgctctatttgcttatgggttacaagatagattccgtattacagctatacttggttca
catttacttactctaggaattggagcaggcttactctttgcaaaagcagtctatttaggt
ggtctttatgatacttgggcttgtggtggtggagacatgagattgattgaaagggtagag
cttggtttaaatccttatctactagctcagtacttacttcgtgctccatttgggtcatca
ggatggataattagcatcaataacatggaagatctaattggaggtcattattgggtctca
ttttatctaattctaggcagctgttggcatattcaaacaagagctcttacaattacaaca
agagcttttacatggtcagcagaagcttacctctcttatactttatcagctgtaacttta
tgtggttctatcacaagtttgttttcttggtacaataatactgcatatccaagtgaattc
tatggacctactggagcagaagcctctcaagcacaaagctttacattccttatacgagat
tggaaattgggtataaagataacttcatccgaaggtcctacagctcttggtaagtatctc
atgagatctccaacaggtgaaataatatttggtggtgaaagtatgagattctggtctatg
caagcacattggcttgaagctctcagaacatcatttggactggacttatctaagattcaa
tctgatattcaaacttggcaagaaagaagagctgcagagtatatgacccatgcaccacta
gggagcttaaattctgttggtggtgttgctactgaaattaactctgtaaactatgtatct
cctagatcttggttaacctcatctcactggttcttagcattctttattttagttggtcat
tggtggcatggagcaagagcaagagcttcagcattatctagtgaaacaggcttatctcga
gtatatgaacctgttctttatatgcgacctattgattag
>Mz9_psbC
atgcctctcatttattctttaaaacgacgaactctagttggatccaagtatgcttggtgg
tcaggtaatgcaagattcattgagctaagtggtaaattcctaggtgctcatcttgtgcat
acagctcttattctagtttgggctggaacaatgtctttgtttgaattatctcacttcatt
ccagaaaaacctctatatgagcaaggctttattcttcttcctcacttgtttgaattgggt
ggcactttatattcacacttcataataagcatcctacatctcatctctgcaggtatccta
gctcttggaggtatttatcatgctatagttggtgctgaaagattagaagaaacaagctac
agtgctctatttgcttatgggttacaagatagattccgtattacagctatacttggttca
catttacttactctaggaattggagcagccttactctttgcaaaagcagtctatttaggt
ggtctttatgatacatgggcttgtggtggtggagacatgagattgattgaaagagtagag
cttggtttaaatccttatctactagctcaatacttacttcgtgctccatttggttcatca
ggatggataattagcatcaataacatggaagatctaattggaggccattattgggtctca
ttttatctaattctaggtagctgttggcatattcaaacaagagctcttacaatcacaaca
agagcttttacatggtcagcagaagcttacctctcttacactttatcagctgtaacttta
tgtggttctatcacagcattattttcttggttcaataatactgcatatccaagtgaattc
tatggacctactggggcagaagcctctcaagcacaaagctttacattccttatacgagat
tggaaattgggtataaagataagttcatccgaaggtcctacagctcttggtaagtatctc
atgagatctccaacaggtgaaataatatttggtggtgaaagtatgagattctggtctatg
caagcacattggcttgaagctctcagaacatcatttggactggacttatctaagattcaa
tctgatattcaaacttggcaagaaagaagagctgcagagtatatgacccatgcaccacta
gggagcttaaattctgttggtggtgttgctactgaaattaactctgtaaactatgtatct
cctagatcttggttaacatcatctcactggttcttagcattctttattttagttggtcat
tggtggcatggagcaagatcaagagctgcagcattgtctagtgaaacaggcttatctcga
gtatatgaacctgttctttatatgcgacctattgattag

>MzC1_psbC
atgcctctcatttattctttaaaacgacgaactctagttggatccaggtatgcttggtgg
tcaggtaatgcaagattcattgagcttagcggtaaattcctaggtgctcatcttgtgcat
acagctcttattctagtttgggctggaagaatgtctttgtttgaattatctcacttcatc
ccagaaaaacctctatatgagcaaggctttattcttcttcctcacttgtttgaattgggt
ggcactttatattcacacttcataataagcatcctacatctcatctctgcaggtatccta
gctcttggaggtatttatcatgctatagttggtgctgaaagactagaagaaacaagctat
agctccctatttgcttatgggttacaagatagattccgtattactgctatacttggatca
catttacttactctaggaattggagcaggcttactctttgcaaaagcagtctatttaggt
ggtctttatgatacatgggcttgtggtggtggagacatgagattgattgaaagggtagag
cttggtttaaatccttatctactagctcagtacttacttcgtgctccatttgggtcatca
ggatggataattagcatcaataacatggaagatctcattggaggtcattattgggtctca
ttttatctaattctaggcagctgttggcatattcaaacaagagctcttacaattacaaca
agagcttttacatggtcagcagaagcttacctctcttatactttatcagctgtaacttta
tgtggttctatcacagcattattttcttggttcaataatactgcatatccaagtgaattc
tatggacctactggagcagaagcctctcaagcacaaagctttacattcctcatacgagat
tggaaattgggtataaagataacttcatccgaaggtcctacagctcttggtaagtatctc
atgagatctccaacaggtgaaataatatttggtggtgaaagtatgagattctggtctatg
caagcacattggcttgaagctctcagaacatcatttggactggacttatctaagattcaa
tctgatattcaaacttggcaagaaagaagagctgcagagtatatgacccatgcaccacta
gggagcttaaattctgttggtggtgttgctactgaaattaactctgtaaactatgtatct
cctagatcttggttaacatcatctcactggttcttagcattctttattttagttggtcat
tggtggcatggagcaagagcaagagcttcagcattatctagtgaaacaggcttatctcga
gtatatgaacctgttctttatatgcgacctattgattag
>MzC1GaII_psbC
atgcctctcatttattctttaaaacgacgaactctagttggatccaggtatgcttggtgg
tcaggtaatgcaagattcattgagcttagcggtaaattcctaggtgctcatcttgtgcat
acagctcttattctagtttgggctggaacaatgtctttgtttgaattatctcacttcatc
ccagaaaaacctctatatgagcaaggctttattcttcttcctcacttgtttgaattgggt
ggcactttatattcacacttcataataagcatcctacatctcatctctgcaggtatccta
gctcttggaggtatttatcatgctatagttggtgctgaaagattagaagaaacaagcttc
agtgctctatttgcttatgggttacaagatagattccgtattactgctatacttggatca
catttacttactctaggaattggagcagccttactctttgcaaaagcagtctatttaggt
ggtctttatgatacatgggcttgtggtggtggagacatgagattgattgaaagggtagag
cttggtttaaatccttatctactagctcagtacttacttcgtgctccatttgggtcatca
ggatggataattagcatcaataacatggaagatctcattggaggtcattattgggtctca
ttttatctaattctaggcagctgttggcatattcaaacaagagctcttacaattacaaca
agagcttttacatggtcagcagaagcttacctctcttatactttatcagctgtaacttta
tgtggttctatcacagcattattttcttggttcaataatactgcatatccaagtgaattc
tatggacctactggggcagaagcctctcaagcacaaagctttacattcctcatacgagat
tggaaattgggtataaagataacttcatccgaaggtcctacagctcttggtaagtatctc
atgagatctccaacaggtgaaataatatttggtggtgaaagtatgagattctggtctatg
caagcacattggcttgaagctctcagaacatcatttggactggacttatctaagattcaa
tctgatattcaaacttggcaagaaagaagagctgcagagtatatgacccatgcaccacta
gggagcttaaattctgttggtggtgttgctactgaaattaactctgtaaactatgtatct
cctagatcttggttaacatcatctcactggttcttagcattctttattttagttggtcat
tggtggcatggagcaagagcaagagcttcagcattatctagtgaaacaggcttatctcga
gtatatgaacctgttctttatatgcgacctattgattag
>MzC2_psbC
atgcctctcatttattctttaaaacgacgaactctagttggatccaggtatgcttggtgg
tcaggtaatgcaagattcattgagcttagcggtaaattcctaggtgctcatcttgtgcat
acagctcttattctagtttgggctggaacaatgtctttgtttgaattatctcacttcatc
ccagaaaaacctctatatgagcaaggctttattcttcttcctcacttgtttgaattgggt
ggcactttatattcacacttcataataagcatcctacatctcatctctgcaggtatccta
gctcttggaggtatttatcatgctatagttggtgctgaaagattagaagaaacaagcttc
agtgctctatttgcttatgggttacaagatagattccgtattactgctatacttggatca
catttacttactctaggaattggagcagccttactctttgcaaaagcagtctatttaggt

ggtctttatgatacatgggcttgtggtggtggagacatgagattgattgaaagggtagag
cttggtttaaatccttatctactagctcagtacttacttcgtgctccatttgggtcatca
ggatggataattagcatcaataacatggaagatctcattggaggtcattattgggtctca
ttttatctaattctaggcagctgttggcatattcaaacaagagctcttacaattacaaca
agagcttttacatggtcagcagaagcttacctctcttatactttatcagctgtaacttta
tgtggttctatcacagcattattttcttggttcaataatactgcatatccaagtgaattc
tatggacctactggggcagaagcctctcaagcacaaagctttacattcctcatacgagat
tggaaattgggtataaagataacttcatccgaaggtcctacagctcttggtaagtatctc
atgagatctccaacaggtgaaataatatttggtggtgaaagtatgagattctggtctatg
caagcacattggcttgaagctctcagaacatcatttggactggacttatctaagattcaa
tctgatattcaaacttggcaagaaagaagagctgcagagtatatgacccatgcaccacta
gggagcttaaattctgttggtggtgttgctactgaaattaactctgtaaactatgtatct
cctagatcttggttaacatcatctcactggttcttagcattctttattttagttggtcat
tggtggcatggagcaagagcaagagcttcagcattatctagtgaaacaggcttatctcga
gtatatgaacctgttctttatatgcgacctattgattag
>MzC2GaII_psbC
atgcctctcattcattctttaaaacgacgaactctagttggatccaggtatgcttggtgg
tcaggtaatgcaagattcattgagcttagcggtaaattcctaggtgctcatcttgtgcat
acagctcttattctagtttgggctggaacaatgtctttgtttgaattatctcacttcatc
ccagaaaaacctctatatgagcaaggctttattcttcttcctcacttgtttgaattgggt
ggcactttatattcacacttcataataagcatcctacatctcatctctgcaggtatccta
gctcttggaggtatttatcatgctatagttggtgctgaaagattagaagaaacaagcttc
agtgctctatttgcttatgggttacaagatagattccgtattactgctatacttggatca
catttacttactctaggaattggagcagccttactctttgcaaaagcagtctatttaggt
ggtctttatgatacatgggcttgtggtggtggagacatgagattgattgaaagggtagag
cttggtttaaatccttatctactagctcagtacttacttcgtgctccatttgggtcatca
ggatggataattagcatcaataacatggaagatctcattggaggtcattattgggtctca
ttttatctaattctaggcagctgttggcatattcaaacaagagctcttacaattacaaca
agagcttttacatggtcagcagaagcttacctctcttatactttatcagctgtaacttta
tgtggttctatcacagcattattttcttggttcaataatactgcatatccaagtgaattc
tatggacctactggggcagaagcctctcaagcacaaagctttacattcctcatacgagat
tggaaattgggtataaagataacttcatccgaaggtcctacagctcttggtaagtatctc
atgagatctccaacaggtgaaataatatttggtggtgaaagtatgagattctggtctatg
caagcacattggcttgaagctctcagaacatcatttggactggacttatctaagattcaa
tctgatattcaaacttggcaagaaagaagagctgcagagtatatgacccatgcaccacta
gggagcttaaattctgttggtggtgttgctactgaaattaactctgtaaactatgtatct
cctagatcttggttaacatcatctcactggttcttagcattctttattttagttggtcat
tggtggcatggagcaagagcaagagcttcagcattatctagtgaaacaggcttatctcga
gtatatgaacctgttctttatatgcgacctattgattag
>MzC3_psbC
atgcctctcatttattctttaaaacgacgaactctagttggatccaggtatgcttggtgg
tcaggtaatgcaagattcattgagcttagcggtaaattcctaggtgctcatcttgtgcat
acagctcttattctagtttgggctggaacaatgtctttgtttgaattatctcacttcatc
ccagaaaaacctctatatgagcaaggctttattcttcttcctcacttgtttgaattgggt
ggcactttatattcacacttcataataagcatcctacatctcatctctgcaggtatccta
gctcttggaggtatttatcatgctatagttggtgctgaaagattagaagaaacaagcttc
agtgctctatttgcttatgggttacaagatagattccgtattactgctatacttggatca
catttacttactctaggaattggagcagccttactctttgcaaaagcagtctatttaggt
ggtctttatgatacatgggcttgtggtggtggagacatgagattgattgaaagggtagag
cttggtttaaatccttatctactagctcagtacttacttcgtgctccatttgggtcatca
ggatggataattagcatcaataacatggaagatctcattggaggtcattattgggtctca
ttttatctaattctaggcagctgttggcatattcaaacaagagctcttacaattacaaca
agagcttttacatggtcagcagaagcttacctctcttatactttatcagctgtaacttta
tgtggttctatcacagcattattttcttggttcaataatactgcatatccaagtgaattc
tatggacctactggggcagaagcctctcaagcacaaagctttacattcctcatacgagat
tggaaattgggtataaagataacttcatccgaaggtcctacagctcttggtaagtatctc
atgagatctccaacaggtgaaataatatttggtggtgaaagtatgagattctggtctatg

caagcacattggcttgaagctctcagaacatcatttggactggacttatctaagattcaa
tctgatattcaaacttggcaagaaagaagagctgcagagtatatgacccatgcaccacta
gggagcttaaattctgttggtggtgttgctactgaaattaactctgtaaactatgtatct
cctagatcttggttaacatcatctcactggttcttagcattctttattttagttggtcat
tggtggcatggagcaagagcaagagcttcagcattatctagtgaaacaggcttatctcga
gtatatgaacctgttctttatatgcgacctattgattag
>MzC4_psbC
atgcctctcattcattctttaaaacgacgaactctagttggatccaggtatgcttggtgg
tcaggtaatgcaagattcattgagcttagcggtaaattcctaggtgctcatcttgtgcat
acagctcttattctagtttgggctggaacaatgtctttgtttgaattatctcacttcatc
ccagaaaaacctctatatgagcaaggctttattcttcttcctcacttgtttgaattgggt
ggcactttatattcacacttcataataagcatcctacatctcatctctgcaggtatccta
gctcttggaggtatttatcatgctatagttggtgctgaaagattagaagaaacaagcttc
agtgctctatttgcttatgggttacaagatagattccgtattactgctatacttggatca
catttacttactctaggaattggagcagccttactctttgcaaaagcagtctatttaggt
ggtctttatgatacatgggcttgtggtggtggagacatgagattgattgaaagggtagag
cttggtttaaatccttatctactagctcagtacttacttcgtgctccatttgggtcatca
ggatggataattagcatcaataacatggaagatctcattggaggtcattattgggtctca
ttttatctaattctaggcagctgttggcatattcaaacaagagctcttacaattacaaca
agagcttttacatggtcagcagaagcttacctctcttatactttatcagctgtaacttta
tgtggttctatcacagcattattttcttggttcaataatactgcatatccaagtgaattc
tatggacctactggggcagaagcctctcaagcacaaagctttacattcctcatacgagat
tggaaattgggtataaagataacttcatccgaaggtcctacagctcttggtaagtatctc
atgagatctccaacaggtgaaataatatttggtggtgaaagtatgagattctggtctatg
caagcacattggcttgaagctctcagaacatcatttggactggacttatctaagattcaa
tctgatattcaaacttggcaagaaagaagagctgcagagtatatgacccatgcaccacta
gggagcttaaattctgttggtggtgttgctactgaaattaactctgtaaactatgtatct
cctagatcttggttaacatcatctcactggttcttagcattctttattttagttggtcat
tggtggcatggagcaagagcaagagcttcagcattatctagtgaaacaggcttatctcga
gtatatgaacctgttctttatatgcgacctattgattag
>Od10_psbC
atgcctctcatttattctttaaaacgacgaactctagttggatccaggtatgcttggtgg
tcaggtaatgcaagattcattgagcttagcggtaaattcctaggtgctcatcttgtgcat
acagctcttattctagtttgggctggaacaatgtctttgtttgaattatctcacttcatc
ccagaaaaacctctatatgagcaaggctttattcttcttcctcacttgtttgaattgggt
ggcactttatattcacacttcataataagcatcctacatctcatctctgcaggtatccta
gctcttggaggtatttatcatgctatagttggtgctgaaagattagaagaaacaagcttc
agtgctctatttgcttatgggttacaagatagattccgtattactgctatacttggatca
catttacttactctaggaattggagcagccttactctttgcaaaagcagtctatttaggt
ggtctttatgatacatgggcttgtggtggtggagacatgagattgattgaaagggtagag
cttggtttaaatccttatctactagctcagtacttacttcgtgctccatttgggtcatca
ggatggataattagcatcaataacatggaagatctcattggaggtcattattgggtctca
ttttatctaattctaggcagctgttggcatattcaaacaagagctcttacaattacaaca
agagcttttacatggtcagcagaagcttacctctcttatactttatcagctgtaacttta
tgtggttctatcacagcattattttcttggttcaataatactgcatatccaagtgaattc
tatggacctactggggcagaagcctctcaagcacaaagctttacattcctcatacgagat
tggaaattgggtataaagataacttcatccgaaggtcctacagctcttggtaagtatctc
atgagatctccaacaggtgaaataatatttggtggtgaaagtatgagattctggtctatg
caagcacattggcttgaagctctcagaacatcatttggactggacttatctaagattcaa
tctgatattcaaacttggcaagaaagaagagctgcagagtatatgacccatgcaccacta
gggagcttaaattctgttggtggtgttgctactgaaattaactctgtaaactatgtatct
cctagatcttggttaacatcatctcactggttcttagcattctttattttagttggtcat
tggtggcatggagcaagagcaagagcttcagcattatctagtgaaacaggcttatctcga
gtatatgaacctgttctttatatgcgacctattgattag
>Od11_psbC
atgcctctccttcattctttaaaacgacgaactctagttggatccaggtatgcttggtgg
tcaggtaatgcaagattcattgagcttagcggtaaattcctaggtgctcatcttgtgcat

acagctcttattctagtttgggctggaacaatgtctttgtttgaattatctcacttcatc
ccagaaaaacctctatatgagcaaggctttattcttcttcctcacttgtttgaattgggt
ggcactttatattcacacttcataataagcatcctacatctcatctctgcaggtatccta
gctcttggaggtatttatcatgctatagttggtgctgaaagattagaagaaacaagcttc
agtgctctatttgcttatgggttacaagatagattccgtattactgctatacttggatca
catttacttactctaggaattggagcagccttactctttgcaaaagcagtctatttaggt
ggtctttatgatacatgggcttgtggtggtggagacatgagattgattgaaagggtagag
cttggtttaaatccttatctactagctcagtacttacttcgtgctccatttgggtcatca
ggatggataattagcatcaataacatggaagatctcattggaggtcattattgggtctca
ttttatctaattctaggcagctgttggcatattcaaacaagagctcttacaattacaaca
agagcttttacatggtcagcagaagcttacctctcttatactttatcagctgtaacttta
tgtggttctatcacagcattattttcttggttcaataatactgcatatccaagtgaattc
tatggacctactggggcagaagcctctcaagcacaaagctttacattcctcatacgagat
tggaaattgggtataaagataacttcatccgaaggtcctacagctcttggtaagtatctc
atgagatctccaacaggtgaaataatatttggtggtgaaagtatgagattctggtctatg
caagcacattggcttgaagctctcagaacatcatttggactggacttatctaagattcaa
tctgatattcaaacttggcaagaaagaagagctgcagagtatatgacccatgcaccacta
gggagcttaaattctgttggtggtgttgctactgaaattaactctgtaaactatgtatct
cctagatcttggttaacatcatctcactggttcttagcattctttattttagttggtcat
tggtggcatggagcaagagcaagagcttcagcattatctagtgaaacaggcttatctcga
gtatatgaacctgttctttatatgcgacctattgattag
>Od12_psbC
atgcctctcatttattctttaaaacgacgaactctagttggatccaggtatgcttggtgg
tcaggtaatgcaagattcattgagcttagcggtaaattcctaggtgctcatcttgtgcat
acagctcttattctagtttgggctggaacaatgtctttgtttgaattatctcacttcatc
ccagaaaaacctctatatgagcaaggctttattcttcttcctcacttgtttgaattgggt
ggcactttatattcacacttcataataagcatcctacatctcatctctgcaggtatccta
gctcttggaggtatttatcatgctatagttggtgctgaaagattagaagaaacaagcttc
agtgctctatttgcttatgggttacaagatagattccgtattactgctatacttggatca
catttacttactctaggaattggagcagccttactctttgcaaaagcagtctatttaggt
ggtctttatgatacatgggcttgtggtggtggagacatgagattgattgaaagggtagag
cttggtttaaatccttatctactagctcagtacttacttcgtgctccatttgggtcatca
ggatggataattagcatcaataacatggaagatctcattggaggtcattattgggtctca
ttttatctaattctaggcagctgttggcatattcaaacaagagcttttacaattacaaca
agagcttttacatggtcagcagaagcttacctctcttatactttatcagctgtaacttta
tgtggttctatcacagcattattttcttggttcaataatactgcatatccaagtgaattc
tatggacctactggggcagaagcctctcaagcacaaagctttacattcctcatacgagat
tggaaattgggtataaagataacttcatccgaaggtcctacagctcttggtaagtatctc
atgagatctccaacaggtgaaataatatttggtggtgaaagtatgagattctggtctatg
caagcacattggcttgaagctctcagaacatcatttggactggacttatctaagattcaa
tctgatattcaaacttggcaagaaagaagagctgcagagtatatgacccatgcaccacta
gggagcttaaattctgttggtggtgttgctactgaaattaactctgtaaactatgtatct
cctagatcttggttaacatcatctcactggttcttagcattctttattttagttggtcat
tggtggcatggagcaagagcaagagcttcagcattatctagtgaaacaggcttatctcga
gtatatgaacctgttctttatatgcgacctattgattag
>Od13_psbC
atgcctctcattcattctttaaaacgacgaactctagttggatccaggtatgcttggtgg
tcaggtaatgcaagattcattgagcttagcggtaaattcctaggtgctcatcttgtgcat
acagctcttattctagtttgggctggaacaatgtctttgtttgaattatctcacttcatc
ccagaaaaacctctatatgagcaaggctttattcttcttcctcacttgtttgaattgggt
ggcactttatattcacacttcataataagcatcctacatctcatctctgcaggtatccta
gctcttggaggtatttatcatgctatagttggtgctgaaagattagaagaaacaagcttc
agtgctctatttgcttatgggttacaagatagattccgtattactgctatacttggatca
catttacttactctaggaattggagcagccttactctttgcaaaagcagtctatttaggt
ggtctttatgatacatgggcttgtggtggtggagacatgagattgattgaaagggtagag
cttggtttaaatccttatctactagctcagtacttacttcgtgctccatttgggtcatca
ggatggataattagcatcaataacatggaagatctcattggaggtcattattgggtctca

ttttatctaattctaggcagctgttggcatattcaaacaagagctcttacaattacaaca
agagcttttacatggtcagcagaagcttacctctcttatactttatcagctgtaacttta
tgtggttctatcacagcattattttcttggttcaataatactgcatatccaagtgaattc
tatggacctactggggcagaagcctctcaagcacaaagctttacattcctcatacgagat
tggaaattgggtataaagataacttcatccgaaggtcctacagctcttggtaagtatctc
atgagatctccaacaggtgaaataatatttggtggtgaaagtatgagattctggtctatg
caagcacattggcttgaagctctcagaacatcatttggactggacttatctaagattcaa
tctgatattcaaacttggcaagaaagaagagctgcagagtatatgacccatgcaccacta
gggagcttaaattctgttggtggtgttgctactgaaattaactctgtaaactatgtatct
cctagatcttggttaacatcatctcactggttcttagcattctttattttagttggtcat
tggtggcatggagcaagagcaagagcttcagcattatctagtgaaacaggcttatctcga
gtatatgaacctgttctttatatgcgacctattgattag
>Od14_psbC
atgcctctcattcattctttaaaacgacgaactctagttggatccaggtatgcttggtgg
tcaggtaatgcaagattcattgagcttagcggtaaattcctaggtgctcatcttgtgcat
acagctcttattctagtttgggctggaacaatgtctttgtttgaattatctcacttcatc
ccagaaaaacctctatatgagcaaggctttattcttcttcctcacttgtttgaattgggt
ggcactttatattcacacttcataataagcatcctacatctcatctctgcaggtatccta
gctcttggaggtatttatcatgctatagttggtgctgaaagattagaagaaacaagcttc
agtgctctatttgcttatgggttacaagatagattccgtattactgctatacttggatca
catttacttactctaggaattggagcagccttactctttgcaaaagcagtctatttaggt
ggtctttatgatacatgggcttgtggtggtggagacatgagattgattgaaagggtagag
cttggtttaaatccttatctactagctcagtacttacttcgtgctccatttgggtcatca
ggatggataattagcatcaataacatggaagatctcattggaggtcattattgggtctca
ttttatctaattctaggcagctgttggcatattcaaacaagagctcttacaattacaaca
agagcttttacatggtcagcagaagcttacctctcttatactttatcagctgtaacttta
tgtggttctatcacagcattattttcttggttcaataatactgcatatccaagtgaattc
tatggacctactggggcagaagcctctcaagcacaaagctttacattcctcatacgagat
tggaaattgggtataaagataacttcatccgaaggtcctacagctcttggtaagtatctc
atgagatctccaacaggtgaaataatatttggtggtgaaagtatgagattctggtctatg
caagcacattggcttgaagctctcagaacatcatttggactggacttatctaagattcaa
tctgatattcaaacttggcaagaaagaagagctgcagagtatatgacccatgcaccacta
gggagcttaaattctgttggtggtgttgctactgaaattaactctgtaaactatgtatct
cctagatcttggttaacatcatctcactggttcttagcattctttattttagttggtcat
tggtggcatggagcaagagcaagagcttcagcattatctagtgaaacaggcttatctcga
gtatatgaacctgttctttatatgcgacctattgattag
>Od15_psbC
atgcctctcatttattctttaaaacgacgaactctagttggatccaggtatgcttggtgg
tcaggtaatgcaagattcattgagcttagcggtaaattcctaggtgctcatcttgtgcat
acagctcttattctagtttgggctggaacaatgtctttgtttgaattatctcacttcatc
ccagaaaaacctctatatgagcaaggctttattcttcttcctcacttgtttgaattgggt
ggcactttatattcacacttcataataagcatcctacatctcatctctgcaggtatccta
gctcttggaggtatttatcatgctatagttggtgctgaaagattagaagaaacaagcttc
agtgctctatttgcttatgggttacaagatagattccgtattactgctatacttggatca
catttacttactctaggaattggagcagccttactctttgcaaaagcagtctatttaggt
ggtctttatgatacatgggcttgtggtggtggagacatgagattgattgaaagggtagag
cttggtttaaatccttatctactagctcagtacttacttcgtgctccatttgggtcatca
ggatggataattagcatcaataacatggaagatctcattggaggtcattattgggtctca
ttttatctaattctaggcagctgttggcatattcaaacaagagctcttacaattacaaca
agagcttttacatggtcagcagaagcttacctctcttatactttatcagctgtaacttta
tgtggttctatcacagcattattttcttggttcaataatactgcatatccaagtgaattc
tatggacctactggggcagaagcctctcaagcacaaagctttacattcctcatacgagat
tggaaattgggtataaagataacttcatccgaaggtcctacagctcttggtaagtatctc
atgagatctccaacaggtgaaataatatttggtggtgaaagtatgagattctggtctatg
caagcacattggcttgaagctctcagaacatcatttggactggacttatctaagattcaa
tctgatattcaaacttggcaagaaagaagagctgcagagtatatgacccatgcaccacta
gggagcttaaattctgttggtggtgttgctactgaaattaactctgtaaactatgtatct

cctagatcttggttaacatcatctcactggttcttagcattctttattttagttggtcat
tggtggcatggagcaagagcaagagcttcagcattatctagtgaaacaggcttatctcga
gtatatgaacctgttctttatatgcgacctattgattag
>Od18_psbC
atgcctctcatttattctttaaaacgacgaactctagttggatccaggtatgcttggtgg
tcaggtaatgcaagattcattgagcttagcggtaaattcctaggtgctcatcttgtgcat
acagctcttattctagtttgggctggaacaatgtctttgtttgaattatctcacttcatc
ccagaaaaacctctatatgagcaaggctttattcttcttcctcacttgtttgaattgggt
ggcactttatattcacacttcataataagcatcctacatctcatctctgcaggtatccta
gctcttggaggtatttatcatgctatagttggtgctgaaagattagaagaaacaagcttc
agtgctctatttgcttatgggttacaagatagattccgtattactgctatacttggatca
catttacttactctaggaattggagcagccttactctttgcaaaagcagtctatttaggt
ggtctttatgatacatgggcttgtggtggtggagacatgagattgattgaaagggtagag
cttggtttaaatccttatctactagctcagtacttacttcgtgctccatttgggtcatca
ggatggataattagcatcaataacatggaagatctcattggaggtcattattgggtctca
ttttatctaattctaggcagctgttggcatattcaaacaagagctcttacaattacaaca
agagcttttacatggtcagcagaagcttacctctcttatactttatcagctgtaacttta
tgtggttctatcacagcattattttcttggttcaataatactgcatatccaagtgaattc
tatggacctactggggcagaagcctctcaagcacaaagctttacattcctcatacgagat
tggaaattgggtataaagataacttcatccgaaggtcctacagctcttggtaagtatctc
atgagatctccaacaggtgaaataatatttggtggtgaaagtatgagattctggtctatg
caagcacattggcttgaagctctcagaacatcatttggactggacttatctaagattcaa
tctgatattcaaacttggcaagaaagaagagctgcagagtatatgacccatgcaccacta
gggagcttaaattctgttggtggtgttgctactgaaattaactctgtaaactatgtatct
cctagatcttggttaacatcatctcactggttcttagcattctttattttagttggtcat
tggtggcatggagcaagagcaagagcttcagcattatctagtgaaacaggcttatctcga
gtatatgaacctgttctttatatgcgacctattgattag
>Od19_psbC
atgcctcttcttcattctttaaaacgacgaactctagttggatccaggtatgcttggtgg
tcaggtaatgcaagattcattgagcttagcggtaaattcctaggtgctcatcttgtgcat
acagctcttattctagtttgggctggaacaatgtctttgtttgaattatctcacttcatc
ccagaaaaacctctatatgagcaaggctttattcttcttcctcacttgtttgaattgggt
ggcactttatattcacacttcataataagcatcctacatctcatctctgcaggtatccta
gctcttggaggtatttatcatgctatagttggtgctgaaagattagaagaaacaagcttc
agtgctctatttgcttatgggttacaagatagattccgtattactgctatacttggatca
catttacttactctaggaattggagcagccttactctttgcaaaagcagtctatttaggt
ggtctttatgatacatgggcttgtggtggtggagacatgagattgattgaaagggtagag
cttggtttaaatccttatctactagctcagtacttacttcgtgctccatttgggtcatca
ggatggataattagcatcaataacatggaagatctcattggaggtcattattgggtctca
ttttatctaattctaggcagctgttggcatattcaaacaagagctcttacaattacaaca
agagcttttacatggtcagcagaagcttacctctcttatactttatcagctgtaacttta
tgtggttctatcacagcattattttcttggttcaataatactgcatatccaagtgaattc
tatggacctactggggcagaagcctctcaagcacaaagctttacattcctcatacgagat
tggaaattgggtataaagataacttcatccgaaggtcctacagctcttggtaagtatctc
atgagatctccaacaggtgaaataatatttggtggtgaaagtatgagattctggtctatg
caagcacattggcttgaagctctcagaacatcatttggactggacttatctaagattcaa
tctgatattcaaacttggcaagaaagaagagctgcagagtatatgacccatgcaccacta
gggagcttaaattctgttggtggtgttgctactgaaattaactctgtaaactatgtatct
cctagatcttggttaacatcatctcactggttcttagcattctttattttagttggtcat
tggtggcatggagcaagagcaagagcttcagcattatctagtgaaacaggcttatctcga
gtatatgaacctgttctttatatgcgacctattgattag
>Od2_psbC
atgcctctcatttattctttaaaacgacgaactctagttggatccaggtatgcttggtgg
tcaggtaatgcaagattcattgagcttagcggtaaattcctaggtgctcatcttgtgcat
acagctcttattctagtttgggctggaacaatgtctttgtttgaattatctcacttcatc
ccagaaaaacctctatatgagcaaggctttattcttcttcctcacttgtttgaattgggt
ggcactttatattcacacttcataataagcatcctacatctcatctctgcaggtatccta

gctcttggaggtatttatcatgctatagttggtgctgaaagattagaagaaacaagcttc
agtgctctatttgcttatgggttacaagatagattccgtattactgctatacttggatca
catttacttactctaggaattggagcagccttactctttgcaaaagcagtctatttaggt
ggtctttatgatacatgggcttgtggtggtggagacatgagattgattgaaagggtagag
cttggtttaaatccttatctactagctcagtacttacttcgtgctccatttgggtcatca
ggatggataattagcatcaataacatggaagatctcattggaggtcattattgggtctca
ttttatctaattctaggcagctgttggcatattcaaacaagagctcttacaattacaaca
agagcttttacatggtcagcagaagcttacctctcttatactttatcagctgtaacttta
tgtggttctatcacagcattattttcttggttcaataatactgcatatccaagtgaattc
tatggacctactggggcagaagcctctcaagcacaaagctttacattcctcatacgagat
tggaaattgggtataaagataacttcatccgaaggtcctacagctcttggtaagtatctc
atgagatctccaacaggtgaaataatatttggtggtgaaagtatgagattctggtctatg
caagcacattggcttgaagctctcagaacatcatttggactggacttatctaagattcaa
tctgatattcaaacttggcaagaaagaagagctgcagagtatatgacccatgcaccacta
gggagcttaaattctgttggtggtgttgctactgaaattaactctgtaaactatgtatct
cctagatcttggttaacatcatctcactggttcttagcattctttattttagttggtcat
tggtggcatggagcaagagcaagagcttcagcattatctagtgaaacaggcttatctcga
gtatatgaacctgttctttatatgcgacctattgattag
>Od22_psbC
atgcctcttcttcattctttaaaacgacgaactctagttggatccaggtatgcttggtgg
tcaggtaatgcaagattcattgagcttagcggtaaattcctaggtgctcatcttgtgcat
acagctcttattctagtttgggctggaacaatgtctttgtttgaattatctcacttcatc
ccagaaaaacctctatatgagcaaggctttattcttcttcctcacttgtttgaattgggt
ggcactttatattcacacttcataataagcatcctacatctcatctctgcaggtatctta
gctcttggaggtatttatcatgctatagttggtgctgaaagattagaagaaacaagcttc
agtgctctatttgcttatgggttacaagatagattccgtattactgctatacttggatca
catttacttactctaggaattggagcagccttactctttgcaaaagcagtctatttaggt
ggtctttatgatacatgggcttgtggtggtggagacatgagattgattgaaagggtagag
cttggtttaaatccttatctactagctcagtacttacttcgtgctccatttgggtcatca
ggatggataattagcatcaataacatggaagatctcattggaggtcattattgggtctca
ttttatctaattctaggcagctgttggcatattcaaacaagagctcttacaattacaaca
agagcttttacatggtcagcagaagcttacctctcttatactttatcagctgtaacttta
tgtggttctatcacagcattattttcttggttcaataatactgcatatccaagtgaattc
tatggacctactggggcagaagcctctcaagcacaaagctttacattcctcatacgagat
tggaaattgggtataaagataacttcatccgaaggtcctacagctcttggtaagtatctc
atgagatctccaacaggtgaaataatatttggtggtgaaagtatgagattctggtctatg
caagcacattggcttgaagctctcagaacatcatttggactggacttatctaagattcaa
tctgatattcaaacttggcaagaaagaagagctgcagagtatatgacccatgcaccacta
gggagcttaaattctgttggtggtgttgctactgaaattaactctgtaaactatgtatct
cctagatcttggttaacatcatctcactggttcttagcattctttattttagttggtcat
tggtggcatggagcaagagcaagagcttcagcattatctagtgaaacaggcttatctcga
gtatatgaacctgttctttatatgcgacctattgattag
>Od23_psbC
atgcctctcatttattctttaaaacgacgaactctagttggatccaggtatgcttggtgg
tcaggtaatgcaagattcattgagcttagcggtaaattcctaggtgctcatcttgtgcat
acagctcttattctagtttgggctggaacaatgtctttgtttgaattatctcacttcatc
ccagaaaaacctctatatgagcaaggctttattcttcttcctcacttgtttgaattgggt
ggcactttatattcacacttcataataagcatcctacatctcatctctgcaggtatccta
gctcttggaggtatttatcatgctatagttggtgctgaaagattagaagaaacaagcttc
agtgctctatttgcttatgggttacaagatagattccgtattactgctatacttggatca
catttacttactctaggaattggagcagccttactctttgcaaaagcagtctatttaggt
ggtctttatgatacatgggcttgtggtggtggagacatgagattgattgaaagggtagag
cttggtttaaatccttatctactagctcagtacttacttcgtgctccatttgggtcatca
ggatggataattagcatcaataacatggaagatctcattggaggtcattattgggtctca
ttttatctaattctaggcagctgttggcatattcaaacaagagctcttacaattacaaca
agagcttttacatggtcagcagaagcttacctctcttatactttatcagctgtaacttta
tgtggttctatcacagcattattttcttggttcaataatactgcatatccaagtgaattc

tatggacctactggggcagaagcctctcaagcacaaagctttacattcctcatacgagat
tggaaattgggtataaagataacttcatccgaaggtcctacagctcttggtaagtatctc
atgagatctccaacaggtgaaataatatttggtggtgaaagtatgagattctggtctatg
caagcacattggcttgaagctctcagaacatcatttggactggacttatctaagattcaa
tctgatattcaaacttggcaagaaagaagagctgcagagtatatgacccatgcaccacta
gggagcttaaattctgttggtggtgttgctactgaaattaactctgtaaactatgtatct
cctagatcttggttaacatcatctcactggttcttagcattctttattttagttggtcat
tggtggcatggagcaagagcaagagcttcagcattatctagtgaaacaggcttatctcga
gtatatgaacctgttctttatatgcgacctattgattag
>Od24_psbC
atgcctctcatttattctttaaaacgacgaactctagttggatccaagtatgcttggtgg
tcaggtaatgcaagattcattgagctaagtggtaaattcctaggtgctcatcttgtgcat
acagctcttattctagtttgggctggaacaatgtctttgtttgaattatctcacttcatc
ccagaaaaacctctatatgagcaaggctttattcttctacctcatttgtttgaactggga
ggcaatctatattcacacttcataataagcatcctacatctcatctctgcaggtatctta
gctcttggaggtatttatcatgctatagttggtggtgaaagactagaagaaacaagctat
agctccctatttgcttatgggttacaagatagattccgtattactgctatacttggatca
catttacttactctaggaattggagcagccttactctttgcaaaagcagtctatttaggt
ggtctttatgatacatgggcttgtggtggtggagacatgagattgattgaaagggtagag
cttggtttaaatccttatctactagctcagtacttacttcgtgctccatttgggtcatca
ggatggataattagcatcaataacatggaagatctaattggaggccattattgggtctca
ttttatctaattctaggcagctgttggcatattcaaacaagagctcttacaattacaaca
agagcttttacatggtcagcagaagcttacctctcttatactttatcagctgtaacttta
tgtggttctatcacagcattattttcttggttcaataatactgcatatccaagtgaattc
tatggacctactggggcagaagcctctcaagcacaaagctttacattcctcatacgagat
tggaaattgggtataaagataacttcatccgaaggtcctacagctcttggtaagtatctc
atgagatctccaacaggtgaaataatatttggtggtgaaagtatgagattctggtctatg
caagcacattggcttgaagctctcagaacatcatttggactggacttatctaagattcaa
tctgatattcaaacttggcaagaaagaagagctgcagagtatatgacccatgcaccacta
gggagcttaaattctgttggtggtgttgctactgaaattaactctgtaaactatgtatct
cctagatcttggttaacatcatctcactggttcttagcattctttattttagttggtcat
tggtggcatggagcaagagcaagagctgcagcattgtctagtgaaacaggtttatctcga
gtatatgaacctgttctttatatgcgacctattgattag
>Od25_psbC
atgcctctcatttattctttaaaacgacgaactctagttggatccaggtatgcttggtgg
tcaggtaatgcaagattcattgagcttagcggtaaattcctaggtgctcatcttgtgcat
acagctcttattctagtttgggctggaacaatgtctttgtttgaattatctcacttcatc
ccagaaaaacctctatatgagcaaggctttattcttcttcctcacttgtttgaattgggt
ggcactttatattcacacttcataataagcatcctacatctcatctctgcaggtatccta
gctcttggaggtatttatcatgctatagttggtgctgaaagattagaagaaacaagcttc
agtgctctatttgcttatgggttacaagatagattccgtattactgctatacttggatca
catttacttactctaggaattggagcagccttactctttgcaaaagcagtctatttaggt
ggtctttatgatacatgggcttgtggtggtggagacatgagattgattgaaagggtagag
cttggtttaaatccttatctactagctcagtacttacttcgtgctccatttgggtcatca
ggatggataattagcatcaataacatggaagatctcattggaggtcattattgggtctca
ttttatctaattctaggcagctgttggcatattcaaacaagagctcttacaattacaaca
agagcttttacatggtcagcagaagcttacctctcttatactttatcagctgtaacttta
tgtggttctatcacagcattattttcttggttcaataatactgcatatccaagtgaattc
tatggacctactggggcagaagcctctcaagcacaaagctttacattcctcatacgagat
tggaaattgggtataaagataacttcatccgaaggtcctacagctcttggtaagtatctc
atgagatctccaacaggtggaataatatttggtggtgaaagtatgagattctggtctatg
caagcacattggcttgaagctctcagaacatcatttggactggacttatctaagattcaa
tctgatattcaaacttggcaagaaagaagagctgcagagtatatgacccatgcaccacta
gggagcttaaattctgttggtggtgttgctactgaaattaactctgtaaactatgtatct
cctagatcttggttaacatcatctcactggttcttagcattctttattttagttggtcat
tggtggcatggagcaagagcaagagcttcagcattatctagtgaaacaggcttatctcga
gtatatgaacctgttctttatatgcgacctattgattag

>Od26_psbC
atgcctctcatttattctttaaaacgacgaactctagttggatccaggtatgcttggtgg
tcaggtaatgcaagattcattgagcttagcggtaaattcctaggtgctcatcttgtgcat
acagctcttattctagtttgggctggaacaatgtctttgtttgaattatctcacttcatc
ccagaaaaacctctatatgagcaaggctttattcttcttcctcacttgtttgaattgggt
ggcactttatattcacacttcataataagcatcctacatctcatctctgcaggtatccta
gctcttggaggtatttatcatgctatagttggtgctgaaagattagaagaaacaagcttc
agtgctctatttgcttatgggttacaagatagattccgtattactgctatacttggatca
catttacttactctaggaattggagcagccttactctttgcaaaagcagtctatttaggt
ggtctttatgatacatgggcttgtggtggtggagacatgagattgattgaaagggtagag
cttggtttaaatccttatctactagctcagtacttacttcgtgctccatttgggtcatca
ggatggataattagcatcaataacatggaagatctcattggaggtcattattgggtctca
ttttatctaattctaggcagctgttggcatattcaaacaagagctcttacaattacaaca
agagcttttacatggtcagcagaagcttacctctcttatactttatcagctgtaacttta
tgtggttctatcacagcattattttcttggttcaataatactgcatatccaagtgaattc
tatggacctactggggcagaagcctctcaagcacaaagctttacattcctcatacgagat
tggaaattgggtataaagataacttcatccgaaggtcctacagctcttggtaagtatctc
atgagatctccaacaggtgaaataatatttggtggtgaaagtatgagattctggtctatg
caagcacattggcttgaagctctcagaacatcatttggactggacttatctaagattcaa
tctgatattcaaacttggcaagaaagaagagctgcagagtatatgacccatgcaccacta
gggagcttaaattctgttggtggtgttgctactgaaattaactctgtaaactatgtatct
cctagatcttggttaacatcatctcactggttcttagcattctttattttagttggtcat
tggtggcatggagcaagagcaagagcttcagcattatctagtgaaacaggcttatctcga
gtatatgaacctgttctttatatgcgacctattgattag
>Od27_psbC
atgcctcttcttcattctttaaaacgacgaactctagttggatccaggtatgcttggtgg
tcaggtaatgcaagattcattgagcttagcggtaaattcctaggtgctcatcttgtgcat
acagctcttattctagtttgggctggaacaatgtctttgtttgaattatctcacttcatc
ccagaaaaacctctatatgagcaaggctttattcttcttcctcacttgtttgaattgggt
ggcactttatattcacacttcataataagcatcctacatctcatctctgcaggtatccta
gctcttggaggtatttatcatgctatagttggtgctgaaagattagaagaaacaagcttc
agtgctctatttgcttatgggttacaagatagattccgtattactgctatacttggatca
catttacttactctaggaattggagcagccttactctttgcaaaagcagtctatttaggt
ggtctttatgatacatgggcttgtggtggtggagacatgagattgattgaaagggtagag
cttggtttaaatccttatctactagctcagtacttacttcgtgctccatttgggtcatca
ggatggataattagcatcaataacatggaagatctcattggaggtcattattgggtctca
ttttatctaattctaggcagctgttggcatattcaaacaagagctcttacaattacaaca
agagcttttacatggtcagcagaagcttacctctcttatactttatcagctgtaacttta
tgtggttctatcacagcattattttcttggttcaataatactgcatatccaagtgaattc
tatggacctactggggcagaagcctctcaagcacaaagctttacattcctcatacgagat
tggaaattgggtataaagataacttcatccgaaggtcctacagctcttggtaagtatctc
atgagatctccaacaggtgaaataatatttggtggtgaaagtatgagattctggtctatg
caagcacattggcttgaagctctcagaacatcatttggactggacttatctaagattcaa
tctgatattcaaacttggcaagaaagaagagctgcagagtatatgacccatgcaccacta
gggagcttaaattctgttggtggtgttgctactgaaattaactctgtaaactatgtatct
cctagatcttggttaacatcatctcactggttcttagcattctttattttagttggtcat
tggtggcatggagcaagatcaagagctgcagcattgtctagtgaaacaggcttatctcga
gtatatgaacctgttctttatatgcgacctattgattag
>Od4_psbC
atgcctctcattcattctttaaaacgacgaactctagttggatccaggtatgcttggtgg
tcaggtaatgcaagattcattgagcttagcggtaaattcctaggtgctcatcttgtgcat
acagctcttattctagtttgggctggaacaatgtctttgtttgaattatctcacttcatc
ccagaaaaacctctatatgagcaaggctttattcttcttcctcacttgtttgaattgggt
ggcactttatattcacacttcataataagcatcctacatctcatctctgcaggtatccta
gctcttggaggtatttatcatgctatagttggtgctgaaagattagaagaaacaagcttc
agtgctctatttgcttatgggttacaagatagattccgtattactgctatacttggatca
catttacttactctaggaattggagcagccttactctttgcaaaagcagtctatttaggt

ggtctttatgatacatgggcttgtggtggtggagacatgagattgattgaaagggtagag
cttggtttaaatccttatctactagctcagtacttacttcgtgctccatttgggtcatca
ggatggataattagcatcaataacatggaagatctcattggaggtcattattgggtctca
ttttatctaattctaggcagctgttggcatattcaaacaagagctcttacaattacaaca
agagcttttacatggtcagcagaagcttacctctcttatactttatcagctgtaacttta
tgtggttctatcacagcattattttcttggttcaataatactgcatatccaagtgaattc
tatggacctactggggcagaagcctctcaagcacaaagctttacattcctcatacgagat
tggaaattgggtataaagataacttcatccgaaggtcctacagctcttggtaagtatctc
atgagatctccaacaggtgaaataatatttggtggtgaaagtatgagattctggtctatg
caagcacattggcttgaagctctcagaacatcatttggactggacttatctaagattcaa
tctgatattcaaacttggcaagaaagaagagctgcagagtatatgacccatgcaccacta
gggagcttaaattctgttggtggtgttgctactgaaattaactctgtaaactatgtatct
cctagatcttggttaacatcatctcactggttcttagcattctttattttagttggtcat
tggtggcatggagcaagagcaagagcttcagcattatctagtgaaacaggcttatctcga
gtatatgaacctgttctttatatgcgacctattgattag
>Od6_psbC
atgcctctcatttattctttaaaacgacgaactctagttggatccaggtatgcttggtgg
tcaggtaatgcaagattcattgagcttagcggtaaattcctaggtgctcatcttgtgcat
acagctcttattctagtttgggctggaacaatgtctttgtttgaattatctcacttcatc
ccagaaaaacctctatatgagcaaggctttattcttcttcctcacttgtttgaattgggt
ggcactttatattcacacttcataataagcatcctacatctcatctctgcaggtatccta
gctcttggaggtatttatcatgctatagttggtgctgaaagattagaagaaacaagcttc
agtgctctatttgcttatgggttacaagatagattccgtattactgctatacttggatca
catttacttactctaggaattggagcagccttactctttgcaaaagcagtctatttaggt
ggtctttatgatacatgggcttgtggtggtggagacatgagattgattgaaagggtagag
cttggtttaaatccttatctactagctcagtacttacttcgtgctccatttgggtcatca
ggatggataattagcatcaataacatggaagatctcattggaggtcattattgggtctca
ttttatctaattctaggcagctgttggcatattcaaacaagagctcttacaattacaaca
agagcttttacatggtcagcagaagcttacctctcttatactttatcagctgtaacttta
tgtggttctatcacagcattattttcttggttcaataatactgcatatccaagtgaattc
tatggacctactggggcagaagcctctcaagcacaaagctttacattcctcatacgagat
tggaaattgggtataaagataacttcatccgaaggtcctacagctcttggtaagtatctc
atgagatctccaacaggtgaaataatatttggtggtgaaagtatgagattctggtctatg
caagcacattggcttgaagctctcagaacatcctttggattagacttatctaagattcaa
tctgatattcaaacttggcaagaaagaagagctgcagagtatatgacccatgcaccacta
gggagcttaaattctgttggtggtgttgctactgaaattaactctgtaaactatgtatct
cctagatcttggttaacatcatctcactggttcttagcattctttattttagttggtcat
tggtggcatggagcaagagcaagagcttcagcattatctagtgaaacaggcttatctcga
gtatatgaacctgttctttatatgcgacctattgattag
>Od8_psbC
atgcctctcatttattctttaaaacgacgaactctagttggatccaggtatgcttggtgg
tcaggtaatgcaagattcattgagcttagcggtaaattcctaggtgctcatcttgtgcat
acagctcttattctagtttgggctggaacaatgtctttgtttgaattatctcacttcatc
ccagaaaaacctctatatgagcaaggctttattcttcttcctcacttgtttgaattgggt
ggcactttatattcacacttcataataagcatcctacatctcatctctgcaggtatccta
gctcttggaggtatttatcatgctatagttggtgctgaaagattagaagaaacaagcttc
agtgctctatttgcttatgggttacaagatagattccgtattactgctatacttggatca
catttacttactctaggaattggagcagccttactctttgcaaaagcagtctatttaggt
ggtctttatgatacatgggcttgtggtggtggagacatgagattgattgaaagggtagag
cttggtttaaatccttatctactagctcagtacttacttcgtgctccatttgggtcatca
ggatggataattagcatcaataacatggaagatctcattggaggtcattattgggtctca
ttttatctaattctaggcagctgttggcatattcaaacaagagctcttacaattacaaca
agagcttttacatggtcagcagaagcttacctctcttatactttatcagctgtaacttta
tgtggttctatcacagcattattttcttggttcaataatactgcatatccaagtgaattc
tatggacctactggggcagaagcctctcaagcacaaagctttacattcctcatacgagat
tggaaattgggtataaagataacttcatccgaaggtcctacagctcttggtaagtatctc
atgagatctccaacaggtgaaataatatttggtggtgaaagtatgagattctggtctatg

caagcacattggcttgaagctctcagaacatcatttggactggacttatctaagattcaa
tctgatattcaaacttggcaagaaagaagagctgcagagtatatgacccatgcaccacta
gggagcttaaattctgttggtggtgttgctactgaaattaactctgtaaactatgtatct
cctagatcttggttaacatcatctcactggttcttagcattctttattttagttggtcat
tggtggcatggagcaagagcaagagcttcagcattatctagtgaaacaggcttatctcga
gtatatgaacctgttctttatatgcgacctattgattag
>Od9_psbC
atgcctctcattcattctttaaaacgacgaactctagttggatccaggtatgcttggtgg
tcaggtaatgcaagattcattgagcttagcggtaaattcctaggtgctcatcttgtgcat
acagctcttattctagtttgggctggaacaatgtctttgtttgaattatctcacttcatc
ccagaaaaacctctatatgagcaaggctttattcttcttcctcacttgtttgaattgggt
ggcactttatattcacacttcataataagcatcctacatctcatctctgcaggtatccta
gctcttggaggtatttatcatgctatagttggtgctgaaagattagaagaaacaagcttc
agtgctctatttgcttatgggttacaagatagattccgtattactgctatacttggatca
catttacttactctaggaattggagcagccttactctttgcaaaagcagtctatttaggt
ggtctttatgatacatgggcttgtggtggtggagacatgagattgattgaaagggtagag
cttggtttaaatccttatctactagctcagtacttacttcgtgctccatttgggtcatca
ggatggataattagcatcaataacatggaagatctcattggaggtcattattgggtctca
ttttatctaattctaggcagctgttggcatattcaaacaagagctcttacaattacaaca
agagcttttacatggtcagcagaagcttacctctcttatactttatcagctgtaacttta
tgtggttctatcacagcattattttcttggttcaataatactgcatatccaagtgaattc
tatggacctactggggcagaagcctctcaagcacaaagctttacattcctcatacgagat
tggaaattgggtataaagataacttcatccgaaggtcctacagctcttggtaagtatctc
atgagatctccaacaggtgaaataatatttggtggtgaaagtatgagattctggtctatg
caagcacattggcttgaagctctcagaacatcatttggactggacttatctaagattcaa
tctgatattcaaacttggcaagaaagaagagctgcagagtatatgacccatgcaccacta
gggagcttaaattctgttggtggtgttgctactgaaattaactctgtaaactatgtatct
cctagatcttggttaacatcatctcactggttcttagcattctttattttagttggtcat
tggtggcatggagcaagagcaagagcttcagcattatctagtgaaacaggcttatctcga
gtatatgaacctgttctttatatgcgacctattgattag
>Ss5_psbC
atgcctctcattcattctttaaaacgacgaactctagttggatccaggtatgcttggtgg
tcaggtaatgcaagattcattgagcttagcggtaaattcctaggtgctcatcttgtgcat
acagctcttattctagtttgggctggaacaatgtctttgtttgaattatctcacttcatc
ccagaaaaacctctatatgagcaaggctttattcttcttcctcacttgtttgaactgggt
ggcactttatattcacacttcataataagcatcctacatctcatctctgcaggtatccta
gctcttggaggtatttatcatgctatagttggtgctgaaagattagaagaaacaagcttc
agtgctctatttgcttatgggttacaagatagattccgtattactgctatacttggatca
catttacttactctaggaattggagcagccttactctttgcaaaagcagtctatttaggt
ggtctttatgatacatgggcttgtggtggtggagacatgagattgattgaaagggtagag
cttggtttaaatccttatctactagctcagtacttacttcgtgctccatttgggtcatca
ggatggataattagcatcaataacatggaagatctcattggaggtcattattgggtctca
ttttatctaattctaggaagctgttggcatattcaaacaagagctcttacaattacaaca
agagcttttacatggtcagcagaagcttacctctcttatactttatcagctgtaacttta
tgtggttctatcacagcattattttcttggttcaataatactgcatatccaagtgaattc
tatggacctactggggcagaagcctctcaagcacaaagctttacattcctcatacgagat
tggaaattgggtataaagataacttcatccgaaggtcctacagctcttggtaagtatctc
atgagatctccaacaggtgaaataatatttggtggtgaaagtatgagattctggtctatg
caagcacattggcttgaagctctcagaacatcatttggactggacttatctaagattcaa
tctgatattcaaacttggcaagaaagaagagctgcagagtatatgacccatgcaccacta
gggagcttaaattctgttggtggtgttgctactgaaattaactctgtaaactatgtatct
cctagatcttggttaacatcatctcactggttcttagcattctttattttagttggtcat
tggtggcatggagcaagagcaagagcttcagcattatctagtgaaacaggcttatctcga
gtatatgaacctgttctttatatgcgacctattgattag
>Ss6_psbC
atgcctctcatttattctttaaaacgacgaactctagttggatccaggtatgcttggtgg
tcaggtaatgcaagattcattgagctaagtggtaaattcctaggtgctcatcttgtgcat

acagctcttattctagtttgggctggaacaatgtctttgtttgaattatctcacttcatt
ccagaaaaacctctatatgagcaaggctttattcttctacctcatttgtttgaactggga
ggcaatctatattcacacttcataataagcatcctacatctcatctctgcaggtatccta
gctcttggaggtatttatcatgctatagttggtgctgaaagattagaagaaacaagcttc
agtgctctatttgcttatgggttacaagatagattccgtattactgctatacttggatca
catttacttactctaggaattggagcagccttactctttgcaaaagcagtctatttaggt
ggtctttatgatacttgggcttgtggtggtggagacatgagattgattgaaagggtagag
cttggtttaaatccttatctactagctcagtacttacttcgtgctccatttgggtcatca
ggatggataattagcatcaataacatggaagatctcattggaggtcattattgggtctca
ttttatctaattctaggaagctgttggcatattcaaacaagagctcttacaatcacaaca
agagcttttacatggtcagcagaagcttacctctcttatactttatcagctgtaacttta
tgtggttctatcacaacattattttcttggttcaataatactgcatatccaagtgaattc
tatggacctactggggcagaagcctctcaagcacaaagctttacattcctcatacgagat
tggaaattgggtataaagataacttcatccgaaggtcctacagctcttggtaagtatctc
atgagatctccaacaggtgaaataatatttggtggtgaaagtatgagattctggtctatg
caagcacattggcttgaagctctcagaacatcatttggactggacttatctaagattcaa
tctgatattcaaacttggcaagaaagaagagctgcagagtatatgacccatgcaccacta
gggagcttaaattctgttggtggtgttgctactgaaattaactctgtaaactatgtatct
cctagatcttggttaacatcatctcactggttcttagcattctttattttagttggtcat
tggtggcatggagcaagatcaagagctgcagcattgtctagtgaaacaggtttatctcga
gtatatgaacctgtgctttatatgcgacctattgattag
>Ss7_psbC
atgcctctcatttattctttaaaacgacgaactctagttggatccaggtatgcttggtgg
tcaggtaatgcaagattcattgagcttagcggtaaattcctaggtgctcatcttgtgcat
acagctcttattctagtttgggctggaacaatgtctttgtttgaattatctcacttcatc
ccagaaaaacctctatatgagcaaggctttattcttcttcctcacttgtttgaattgggt
ggcactttatattcacacttcataataagcatcctacatctcatctctgcaggtatccta
gctcttggaggtatttatcatgctatagttggtgctgaaagattagaagaaacaagcttc
agtgctctatttgcttatgggttacaagatagattccgtattactgctatacttggatca
catttacttactctaggaattggagcagccttactctttgcaaaagcagtctatttaggt
ggtctttatgatacatgggcttgtggtggtggagacatgagattgattgaaagggtagag
cttggtttaaatccttatctactagctcagtacttacttcgtgctccatttgggtcatca
ggatggataattagcatcaataacatggaagatctcattggaggtcattattgggtctca
ttttatctaattctaggaagctgttggcatattcaaacaagagctcttacaattacaaca
agagcttttacatggtcagcagaagcttacctctcttatactttatcagctgtaacttta
tgtggttctatcacagcattattttcttggttcaataatactgcatatccaagtgaattc
tatggacctactggggcagaagcctctcaagcacaaagctttacattcctcatacgagat
tggaaattgggtataaagataacttcatccgaaggtcctacagctcttggtaagtatctc
atgagatctccaacaggtgaaataatatttggtggtgaaagtatgagattctggtctatg
caagcacattggcttgaagctctcagaacatcatttggactggacttatctaagattcaa
tctgatattcaaacttggcaagaaagaagagctgcagagtatatgacccatgcaccacta
gggagcttaaattctgttggtggtgttgctactgaaattaactctgtaaactatgtatct
cctagatcttggttaacatcatctcactggttcttagcattctttattttagttggtcat
tggtggcatggagcaagagcaagagcttcagcattatctagtgaaacaggcttatctcga
gtatatgaacctgttctttatatgcgacctattgattag
>Ss8_psbC
atgcctctcatttattctttaaaacgacgaactctagttggatccaggtatgcttggtgg
tcaggtaatgcaagattcattgagcttagcggtaaattcctaggtgctcatcttgtgcat
acagctcttattctagtttgggctggaacaatgtctttgtttgaattatctcacttcatc
ccagaaaaacctctatatgagcaaggctttattcttcttcctcacttgtttgaattgggt
ggcactttatattcacacttcataataagcatcctacatctcatctctgcaggtatccta
gctcttggaggtatttatcatgctatagttggtgctgaaagattagaagaaacaagcttc
agtgctctatttgcttatgggttacaagatagattccgtattactgctatacttggatca
catttacttactctaggaattggagcagccttactctttgcaaaagcagtctatttaggt
ggtctttatgatacatgggcttgtggtggtggagacatgagattgattgaaagggtagag
cttggtttaaatccttatctactagctcagtacttacttcgtgctccatttgggtcatca
ggatggataattagcatcaataacatggaagatctcattggaggtcattattgggtctca

ttttatctaattctaggaagctgttggcatattcaaacaagagctcttacaattacaaca
agagcttttacatggtcagcagaagcttacctctcttatactttatcagctgtaacttta
tgtggttctatcacagcattattttcttggttcaataatactgcatatccaagtgaattc
tatggacctactggggcagaagcctctcaagcacaaagctttacattcctcatacgagat
tggaaattgggtataaagataacttcatccgaaggtcctacagctcttggtaagtatctc
atgagatctccaacaggtgaaataatatttggtggtgaaagtatgagattctggtctatg
caagcacattggcttgaagctctcagaacatcatttggactggacttatctaagattcaa
tctgatattcaaacttggcaagaaagaagagctgcagagtatatgacccatgcaccacta
gggagcttaaattctgttggtggtgttgctactgaaattaactctgtaaactatgtatct
cctagatcttggttaacatcatctcactggttcttagcattctttattttagttggtcat
tggtggcatggagcaagagcaagagcttcagcattatctagtgaaacaggcttatctcga
gtatatgaacctgttctttatatgcgacctattgattag
>ohdo1_psbC
atgcctctcatttattctttaaaacgacgaactctagttggatccaggtatgcttggtgg
tcaggtaatgcaagattcattgagcttagcggtaaattcctaggtgctcatcttgtgcat
acagctcttattctagtttgggctggaacaatgtctttgtttgaattatctcacttcatc
ccagaaaaacctctatatgagcaaggctttattcttcttcctcacttgtttgaattgggt
ggcaatctatattcacacttcataataagcatcctacatctcatctctgcaggtatccta
gctcttggaggtatttatcatgctatagttggtgctgaaagattagaagaaacaagcttc
agtgctctatttgcttatgggttacaagatagattccgtattactgctatacttggatca
catttacttactctaggaattggagcagccttactctttgcaaaagcagtctatttaggt
ggtctttatgatacatgggcttgtggtggtggagacatgagattgattgaaagggtagag
cttggtttaaatccttatctactagctcagtacttacttcgtgctccatttgggtcatca
ggatggataattagcatcaataacatggaagatctcattggaggtcattattgggtctca
ttttatctaattctaggcagctgttggcatattcaaacaagagctcttacaatcacaaca
agagcttttacatggtcagcagaagcttacctctcttatactttatcagctgtaacttta
tgtggttctatcacagcattattttcttggtacaataatactgcatatccaagtgaattc
tatggacctactggagcagaagcctctcaagcacaaagctttacattcctcatacgagat
tggaaattgggtataaagataacttcatccgaaggtcctacagctcttggtaagtatctc
atgagatctccaacaggtgaaataatatttggtggtgaaagtatgagattctggtctatg
caagcacattggcttgaagctctcagaacatcatttggactggacttatctaagattcaa
tctgatattcaaacttggcaagaaagaagagctgcagagtatatgacccatgcaccacta
gggagcttaaattctgttggtggtgttgctactgaaattaactctgtaaactatgtatct
cctagatcttggttaacatcatctcactggttcttagcattcttcattttagttggtcat
tggtggcatggagcaagagcaagagcttcagcattatctagtgaaacaggcttatctcga
gtatatgaacctgttctttatatgcgacctattgattag
>ohdo3_psbC
atgcctctcatttattctttaaaacgacgaactctagttggatccaggtatgcttggtgg
tcaggtaatgcaagattcattgagcttagcggtaaattcctaggtgctcatcttgtgcat
acagctcttattctagtttgggctggaacaatgtctttgtttgaattatctcacttcatc
ccagaaaaacctctatatgagcaaggctttattcttcttcctcacttgtttgaattgggt
ggcactttatattcacacttcataataagcatcctacatctcatctctgcaggtatccta
gctcttggaggtatttatcatgctatagttggtgctgaaagattagaagaaacaagcttc
agtgctctatttgcttatgggttacaagatagattccgtattactgctatacttggatca
catttacttactctaggaattggagcagccttactctttgcaaaagcagtctatttaggt
ggtctttatgatacatgggcttgtggtggtggagacatgagattgattgaaagggtagag
cttggtttaaatccttatctactagctcagtacttacttcgtgctccatttgggtcatca
ggatggataattagcatcaataacatggaagatctcattggaggtcattattgggtctca
ttttatctaattctaggcagctgttggcatattcaaacaagagctcttacaatcacaaca
agagcttttacatggtcagcagaagcttacctctcttatactttatcagctgtaacttta
tgtggttctatcacaagtttgttttcttggtacaataatactgcatatccaagtgaattc
tatggacctactggggcagaagcctctcaagcacaaagctttacattcctcatacgagat
tggaaattgggtataaagataacttcatccgaaggtcctacagctcttggtaagtatctc
atgagatctccaacaggtgaaataatatttggtggtgaaagtatgagattctggtctatg
caagcacattggcttgaagctctcagaacatcatttggactggacttatctaagattcaa
tctgatattcaaacttggcaagaaagaagagctgcagagtatatgacccatgcaccacta
gggagcttaaattctgttggtggtgttgctactgaaattaactctgtaaactatgtatct

cctagatcttggttaacatcatctcactggttcttagcattctttattttagttggtcat
tggtggcatggagcaagagcaagagcttcagcattatctagtgaaacaggcttatctcga
gtatatgaacctgttctttatatgcgacctattgattag
>ohdo5_psbC
atgcctcttcttcattctttaaaacgacgaactctagttggatccaggtatgcttggtgg
tcaggtaatgcaagattcattgagcttagcggtaaattcctaggtgctcatcttgtgcat
acagctcttattctagtttgggctggaacaatgtctttgtttgaattatctcacttcatc
ccagaaaaacctctatatgagcaaggctttattcttcttcctcacttgtttgaattgggt
ggcactttatattcacacttcataataagcatcctacatctcatctctgcaggtatccta
gctcttggaggtatttatcatgctatagttggtgctgaaagattagaagaaacaagcttc
agtgctctatttgcttatgggttacaagatagattccgtattactgctatacttggatca
catttacttactctaggaattggagcagccttactctttgcaaaagcagtctatttaggt
ggtctttatgatacatgggcttgtggtggtggagacatgagattgattgaaagggtagag
cttggtttaaatccttatctactagctcagtacttacttcgtgctccatttgggtcatca
ggatggataattagcatcaataacatggaagatctcattggaggtcattattgggtctca
ttttatctaattctaggcagctgttggcatattcaaacaagagctcttacaattacaaca
agagcttttacatggtcagcagaagcttacctctcttatactttatcagctgtaacttta
tgtggttctatcacagcattattttcttggttcaataatactgcatatccaagtgaattc
tatggacctactggggcagaagcctctcaagcacaaagctttacattcctcatacgagat
tggaaattgggtataaagataacttcatccgaaggtcctacagctcttggtaagtatctc
atgagatctccaacaggtgaaataatatttggtggtgaaagtatgagattctggtctatg
caagcacattggcttgaagctctcagaacatcatttggactggacttatctaagattcaa
tctgatattcaaacttggcaagaaagaagagctgcagagtatatgacccatgcaccacta
gggagcttaaattctgttggtggtgttgctactgaaattaactctgtaaactatgtatct
cctagatcttggttaacatcatctcactggttcttagcattctttattttagttggtcat
tggtggcatggagcaagagcaagagcttcagcattatctagtgaaacaggcttatctcga
gtatatgaacctgttctttatatgcgacctattgattag
>ohdo7_psbC
atgcctctcatttattctttaaaacgacgaactctagttggatccaggtatgcttggtgg
tcaggtaatgcaagattcattgagcttagcggtaaattcctaggtgctcatcttgtgcat
acagctcttattctagtttgggctggaacaatgtctttgtttgaattatctcacttcatc
ccagaaaaacctctatatgagcaaggctttattcttcttcctcacttgtttgaattgggt
ggcactttatattcacacttcataataagcatcctacatctcatctctgcaggtatccta
gctcttggaggtatttatcatgctatagttggtgctgaaagattagaagaaacaagcttc
agtgctctatttgcttatgggttacaagatagattccgtattactgctatacttggatca
catttacttactctaggaattggagcagccttactctttgcaaaagcagtctatttaggt
ggtctttatgatacatgggcttgtggtggtggagacatgagattgattgaaagggtagag
cttggtttaaatccttatctactagctcagtacttacttcgtgctccatttgggtcatca
ggatggataattagcatcaataacatggaagatctcattggaggtcattattgggtctca
ttttatctaattctaggcagctgttggcatattcaaacaagagctcttacaattacaaca
agagcttttacatggtcagcagaagcttacctctcttatactttatcagctgtaacttta
tgtggttctatcacagcattattttcttggttcaataatactgcatatccaagtgaattc
tatggacctactggggcagaagcctctcaagcacaaagctttacattcctcatacgagat
tggaaattgggtataaagataacttcatccgaaggtcctacagctcttggtaagtatctc
atgagatctccaacaggtgaaataatatttggtggtgaaagtatgagattctggtctatg
caagcacattggcttgaagctctcagaacatcatttggactggacttatctaagattcaa
tctgatattcaaacttggcaagaaagaagagctgcagagtatatgacccatgcaccacta
gggagcttaaattctgttggtggtgttgctactgaaattaactctgtaaactatgtatct
cctagatcttggttaacatcatctcactggttcttagcattctttattttagttggtcat
tggtggcatggagcaagagcaagagcttcagcattatctagtgaaacaggcttatctcga
gtatatgaacctgttctttatatgcgacctattgattag
>sesoko1_psbC
atgcctcttcttcattctttaaaacgacgaactctagttggatccaggtatgcttggtgg
tcaggtaatgcaagattcattgagcttagcggtaaattcctaggtgctcatcttgtgcat
acagctcttattctagtttgggctggaacaatgtctttgtttgaattatctcacttcatc
ccagaaaaacctctatatgagcaaggctttattcttcttcctcacttgtttgaattgggt
ggcactttatattcacacttcataataagcatcctacatctcatctctgcaggtatccta

gctcttggaggtatttatcatgctatagttggtgctgaaagattagaagaaacaagcttc
agtgctctatttgcttatgggttacaagatagattccgtattactgctatacttggatca
catttacttactctaggaattggagcagccttactctttgcaaaagcagtctatttaggt
ggtctttatgatacatgggcttgtggtggtggagacatgagattgattgaaagggtagag
cttggtttaaatccttatctactagctcagtacttacttcgtgctccatttgggtcatca
ggatggataattagcatcaataacatggaagatctcattggaggtcattattgggtctca
ttttatctaattctaggcagctgttggcatattcaaacaagagctcttacaattacaaca
agagcttttacatggtcagcagaagcttacctctcttatactttatcagctgtaacttta
tgtggttctatcacagcattattttcttggttcaataatactgcatatccaagtgaattc
tatggacctactggggcagaagcctctcaagcacaaagctttacattcctcatacgagat
tggaaattgggtataaagataacttcatccgaaggtcctacagctcttggtaagtatctc
atgagatctccaacaggtgaaataatatttggtggtgaaagtatgagattctggtctatg
caagcacattggcttgaagctctcagaacatcatttggactggacttatctaagattcaa
tctgatattcaaacttggcaagaaagaagagctgcagagtatatgacccatgcaccacta
gggagcttaaattctgttggtggtgttgctactgaaattaactctgtaaactatgtatct
cctagatcttggttaacatcatctcactggttcttagcattctttattttagttggtcat
tggtggcatggagcaagagcaagagcttcagcattatctagtgaaacaggcttatctcga
gtatatgaacctgttctttatatgcgacctattgattag
>sesoko3_psbC
atgcctctcatttattctttaaaacgacgaactctagttggatccaggtatgcttggtgg
tcaggtaatgcaagattcattgagcttagcggtaaattcctaggtgctcatcttgtgcat
acagctcttattctagtttgggctggaacaatgtctttgtttgaattatctcacttcatc
ccagaaaaacctctatatgagcaaggctttattcttcttcctcacttgtttgaattgggt
ggcactttatattcacacttcataataagcatcctacatctcatctctgcaggtatccta
gctcttggaggtatttatcatgctatagttggtgctgaaagattagaagaaacaagcttc
agtgctctatttgcttatgggttacaagatagattccgtattactgctatacttggatca
catttacttactctaggaattggagcagccttactctttgcaaaagcagtctatttaggt
ggtctttatgatacatgggcttgtggtggtggagacatgagattgattgaaagggtagag
cttggtttaaatccttatctactagctcagtacttacttcgtgctccatttgggtcatca
ggatggataattagcatcaataacatggaagatctcattggaggtcattattgggtctca
ttttatctaattctaggaagctgttggcatattcaaacaagagctcttacaattacaaca
agagcttttacatggtcagcagaagcttacctctcttatactttatcagctgtaacttta
tgtggttctatcacagcattattttcttggttcaataatactgcatatccaagtgaattc
tatggacctactggggcagaagcctctcaagcacaaagctttacattcctcatacgagat
tggaaattgggtataaagataacttcatccgaaggtcctacagctcttggtaagtatctc
atgagatctccaacaggtgaaataatatttggtggtgaaagtatgagattctggtctatg
caagcacattggcttgaagctctcagaacatcatttggactggacttatctaagattcaa
tctgatattcaaacttggcaagaaagaagagctgcagagtatatgacccatgcaccacta
gggagcttaaattctgttggtggtgttgctactgaaattaactctgtaaactatgtatct
cctagatcttggttaacatcatctcactggttcttagcattctttattttagttggtcat
tggtggcatggagcaagagcaagagcttcagcattatctagtgaaacaggcttatctcga
gtatatgaacctgttctttatatgcgacctattgattag
>sesoko4_psbC
atgcctcttcttcattctttaaaacgacgaactctagttggatccaggtatgcttggtgg
tcaggtaatgcaagattcattgagcttagcggtaaattcctaggtgctcatcttgtgcat
acagctcttattctagtttgggctggaacaatgtctttgtttgaattatctcacttcatc
ccagaaaaacctctatatgagcaaggctttattcttcttcctcacttgtttgaattgggt
ggcactttatattcacacttcataataagcatcctacatctcatctctgcaggtatccta
gctcttggaggtatttatcatgctatagttggtgctgaaagattagaagaaacaagcttc
agtgctctatttgcttatgggttacaagatagattccgtattactgctatacttggatca
catttacttactctaggaattggagcagccttactctttgcaaaagcagtctatttaggt
ggtctttatgatacatgggcttgtggtggtggagacatgagattgattgaaagggtagag
cttggtttaaatccttatctactagctcagtacttacttcgtgctccatttgggtcatca
ggatggataattagcatcaataacatggaagatctcattggaggtcattattgggtctca
ttttatctaattctaggaagctgttggcatattcaaacaagagctcttacaattacaaca
agagcttttacatggtcagcagaagcttacctctcttatactttatcagctgtaacttta
tgtggttctatcacagcattattttcttggttcaataatactgcatatccaagtgaattc

tatggacctactggggcagaagcctctcaagcacaaagctttacattcctcatacgagat
tggaaattgggtataaagataacttcatccgaaggtcctacagctcttggtaagtatctc
atgagatctccaacaggtgaaataatatttggtggtgaaagtatgagattctggtctatg
caagcacattggcttgaagctctcagaacatcatttggactggacttatctaagattcaa
tctgatattcaaacttggcaagaaagaagagctgcagagtatatgacccatgcaccacta
gggagcttaaattctgttggtggtgttgctactgaaattaactctgtaaactatgtatct
cctagatcttggttaacatcatctcactggttcttagcattctttattttagttggtcat
tggtggcatggagcaagagcaagagcttcagcattatctagtgaaacaggcttatctcga
gtatatgaacctgttctttatatgcgacctattgattag
>REF_DNA_psbC_JX094326
ATGCCTCTCATTTATTCTTTAAAACGACGAACTCTAGTTGGATCCAAGTATGCTTGGTGG
TCAGGTAATGCAAGATTCATTGAGCTAAGTGGTAAATTCCTAGGTGCTCATCTTGTTCAT
ACAGCTCTTATTCTAGTTTGGGCAGGAAGAATGTCTTTGTTTGAATTATCTCACTTCATT
CCAGAAAAACCTCTATATGAGCAAGGCTTTATTCTTCTACCTCATTTGTTTGAACTGGGA
GGCAATCTATATTCACACTTCATAATAAGCATCCTACATCTCATCTCTGCAGGTATCTTA
GCTCTTGGAGGTATTTATCATGCTATAGTTGGTGGTGAAAGACTAGAAGAAACAAGCTAT
AGCTCCCTATTTGCTTATGGGTTACAAGATAGATTCCGTATTACAGCTATACTTGGTTCA
CATTTACTTACTCTAGGAATTGGAGCAGGCTTACTCTTTGCAAAAGCAGTCTATTTAGGT
GGTCTTTATGATACTTGGGCTTGTGGTGGTGGAGACATGAGATTGATTGAAAGAGTAGAG
CTTGGTTTAAATCCTTATCTACTAGCTCAATACTTACTTCGTGCTCCATTTGGTTCATCA
GGATGGATAATTAGCATCAATAACATGGAAGATCTAATTGGAGGCCATTATTGGGTCTCA
TTTTATCTAATTCTAGGTAGCTGTTGGCATATTCAAACAAGAGCTCTTACAATCACAACA
AGAGCTTTTACATGGTCAGCAGAAGCTTACCTCTCTTACACTCTATCAGCTGTAACTTTA
TGTGGTTCTATCACAAGTTTGTTTTCTTGGTACAATAATACTGCATATCCAAGTGAATTC
TATGGACCTACTGGAGCAGAAGCCTCTCAAGCACAAAGCTTTACATTCCTTATACGAGAT
TGGAAATTGGGTATAAAGATAAGTTCATCCGAAGGTCCTACAGCTCTTGGTAAGTATCTC
ATGAGATCTCCAACAGGTGAAATAATATTTGGTGGTGAAAGTATGAGATTCTGGTCTATG
CAAGCACATTGGCTTGAAGCTCTCAGAACATCCTTTGGATTAGACTTATCTAAGATTCAA
TCTGATATTCAAACTTGGCAAGAAAGAAGAGCTGCAGAGTACATGACCCATGCACCACTA
GGGAGCTTAAATTCTGTTGGTGGTGTTGCTACTGAAATTAACTCTGTAAACTATGTATCT
CCTAGATCTTGGTTAACCTCCTCTCATTGGTTCTTGGCCTTCTTTATTTTAGTGGGTCAT
TGGTGGCATGGAGCAAGATCAAGAGCTGCAGCATTGTCTAGTGAAACAGGTTTATCTCGA
GTATATGAACCTGTGCTTTATATGCGACCTATTGATTAG
>REF_RNA_psbC_symbB1.comp52_c0_seq1
atgcctctcatttcttctttaaaacgacgaactctagttggatccaagtatgcttggtgg
tcaggtaatgcaagattcattgagctaagtggtaaattcctaggtgctcatcttgctcat
gcagctcttattctagtttgggcaggaggaatgtctttgtttgaattatctcacttcgtt
ccagaaaaacctctatatgagcaaggctttattcttctacctcatttgtttgaactggga
ggcaatctatattcacacttcgtaataggtgtcctacatctcatctctgcaggtgtctta
gctcttggaggtatttatcatgctatagttggtggtgaaagactagaagaaacaagctat
agctccctatttgcttatgggttacaagatagattccgtattacagctatacttggttca
catttacttgctctaggaattggagcaggcttactctttgcaaaagcagtctatttaggt
ggtctttatgatacttgggcttgtggtggtggagacatgagattgattgaaagagtagag
cttggtttaaatccttatctactagctcaatacttacttcgtgctccatttggttcatca
ggatggataattagcgtcaataacatggaagatctagttggaggccattattgggtctca
ttttatctaattctaggtggctgttggcatattcaaacaagaccttttgcaatcgtaaca
agagcttttacatggtcagcagaagcttacctctcttacactctatcagctgtagcttta
tgtggttttatcgcagctttgttttcttggtacaataatactgcatatccaagtgaattc
tatggacctactggagcagaagcctctcaagcacaaagctttacattccttatacgagat
tggaaattgggtataaagataagttcatccgaaggtcctacagctcttggtaagtatctc
atgagatctccaacaggtgaaataatatttggtggtgaaactatgagattctggtctatg
caagcacattggcttgaagctctcagaacatcctttggattagacttatctaagattcaa
tctgatattcaaacttggcaagaaagaagagctgcagagtacatgacccatgcaccacta
gggagcttaaattctgttggtggtgttgctactgaaattaactctgtaaactatgtatct
cctagatcttggttaacctcctctcattggttcttggccttctttattttagtgggtcat
tggtggcatggagcaagatcaagagctgcagcactgtctagtgaaacaggtttatctcga
gtatatgaacctgtgctttatatgcgacctattgattag

>Hd1_psbD
atgaaactcatctattctacaagattcatggcaagtaaagcttatcttcttggatcactt
actctactagatgactggttaaaacgagatcgatttgtctttattggctggtcaggtcta
ttattatttcctacagcttacttagcagcaggtggatggtttacagccaccgcttttgta
acatctttctttacacatggtctggtcacatcttatctagaagcttgtaatttttaaca
gctgcagtttcaactccagccaactctatggcccattcattactactactctggggacca
gaagcacatcttggatttactgcctgggtttgtataggaggcttatggacatttattgct
tttcatgggctcattggtctcatagcattttccctacgtcagtttgaaatagcacgatta
gttttaattagaccttataatgcacttgcattctcaggaccaatagcagtttatacatct
gtcttcctaatttatccacttggtcaatctagctggttctttgcaccaagctttggtatt
gctgctatcttccgattcctattattccttcaaggttttcataattggacattaaatcct
tttcacatgatgggtgtagctggaatattgggaggtgccctactatcagctatccatggt
gctactgtcattaacactatttatcaagatgctcgtgcatatacaacatttcgtgctttc
tcaccaagtcaaccagaagaaacttattcaatgcttacagctaatcgcttctggtctcaa
atatttggtgtagccttctcaaacaagagatggcttcatttcttcatgctatttgtacct
ctagcaggtatgtggacatcttcaattggtatcattggattagcatttaatctaagagct
tatgattttatttctcaagaattgaaagcagcagaagatcctgaatttgaaacattttat
acaaagaacattctactcaatgaaggtatacgactttggatggctgtccaagatcaagct
catgaaaattatcagttcccagaagaggtattgccaagaggtaattctttgtga
>Hd2_psbD
atgaaactcatctattctacaagattcatggcaagtaaagcttatcttcttggatcactt
actctactagatgactggttaaaacgagatcgatttgtctttattggctggtcaggtcta
ttattatttcctacagcttacttagcagcaggtggatggtttacagccaccgcttttgta
acatctttctttacacatggtctggtcacatcttatctagaagcttgtaatttttaaca
gctgcagtttcaactccagccaactctatggcccattcattactactactctggggacca
gaagcacatcttggatttactgcctgggtttgtataggaggcttatggacatttattgct
tttcatgggctcattggtctcatagcattttccctacgtcagtttgaaatagcacgatta
gttttaattagaccttataatgcacttgcattctcaggaccaatagcagtttatacatct
gtcttcctaatttatccacttggtcaatctagctggttctttgcaccaagctttggtatt
gctgctatcttccgattcctattattccttcaaggttttcataattggacattaaatcct
tttcacatgatgggtgtagctggaatattgggaggtgccctactatcagctatccatggt
gctactgtcattaacactatttatcaagatgctcgtgcatatacaacatttcgtgctttc
tcaccaagtcaaccagaagaaacttattcaatgcttacagctaatcgtttttggtctcag
atatttggtgtagccttctcaaacaagagatggcttcatttcttcatgctatttgtacct
ctagcaggtatgtggacatcttcaattggtatcattggattagcatttaatctaagagct
tatgattttatttctcaagaattgaaagcagcagaagatcctgaatttgaaacattttat
acaaagaacattctactcaatgaaggtatacgactttggatggctgtccaagatcaagct
catgaaaattatcagttcccagaagaggtattgccaagaggtaattctttgtga
>Hd3_psbD
atgaaactcatctattctacaagattcatgtcaagtaaagcttatcttcttggatcactt
actctactagatgactggttaaaacgagatcgatttgtctttattggctggtcaggtcta
ttattatttcctacagcttacttagcagcaggtggatggtttacagccaccgcttttgta
acatctttctttacacatggtctggtcacatcttatctagaagcttgtaatttttaaca
gctgcagtttcaactccagccaactctatggcccattcattactactactctggggacca
gaagcacatcttggatttactgcctgggtttgtataggaggcttatggacatttattgct
tttcatgggctcattggtctcatagcattttccctacgtcagtttgaaatagcacgatta
gttttaattagaccttataatgcacttgcattctcaggaccaatagcagtttatacatct
gtcttcctaatttatccacttggtcaatctagctggttctttgcaccaagctttggtatt
gctgctatcttccgattcctattattccttcaaggttttcataattggacattaaatcct
tttcacatgatgggtgtagctggaatattgggaggtgccctactatcagctatccatggt
gctactgtcattaacactatttatcaagatgctcgtgcatatacaacatttcgtgctttc
tcaccaagtcaaccagaagaaacttattcaatgcttacagctaatcgcttctggtctcaa
atatttggtgtagccttctcaaacaagagatggcttcatttcttcatgctatttgtacct
ctagcaggtatgtggacatcttcaattggtatcattggattagcatttaatctaagagct
tatgattttatttctcaagaattgaaagcagcagaagatcctgaatttgaaacattttat
acaaagaacattctactcaatgaaggtatacgactttggatggctgtccaagatcaagct
catgaaaattatcagttcccagaagaggtattgccaagaggtaattctttgtga

>Hd4_psbD
atgaaactcatctattctacaagattcatggcaagtaaagcttatcttcttggatcactt
actctactagatgactggttaaaacgagatcgatttgtctttattggctggtcaggtcta
ttattatttcctacagcttacttagcagcaggtggatggtttacagccaccgcttttgta
acatctttctttacacatggtctggtcacatcttatctagaagcttgtaattttttaaca
gctgcagtttcaactccagccaactctatggcccattcattactactactctggggacca
gaagcacatcttggatttactgcctgggtttgtataggaggcttatggacatttattgct
tttcatgggctcattggtctcatagcattttccctacgtcagtttgaaatagcacgatta
gttttaattagaccttataatgcacttgcattctcaggaccaatagcagtttatacatct
gtcttcctaatttatccacttggtcaatctagctggttctttgcaccaagctttggtatt
gctgctatcttccgattcctattattccttcaaggttttcataattggacattaaatcct
tttcacatgatgggtgtagctggaatattgggaggtgccctactatcagctatccatggt
gctactgtcattaacactatttatcaagatgctcgtgcatatacaacatttcgtgctttc
tcaccaagtcaaccagaagaaacttattcaatgcttacagctaatcgtttttggtctcag
atatttggtgtagccttctcaaacaagagatggcttcatttcttcatgctatttgtacct
ctagcagatatgtggacatcttcaattggtatcattggattagcatttaatctaagagct
tatgattttatttctcaagaattgaaagcagcagaagatcctgaatttgaaacattttat
acaaagaacattctactcaatgaaggtatacgactttggatggctgtccaagatcaagct
catgaaaattatcagttcccagaagaggtattgccaagaggtaattctttgtga
>Hd5_psbD
atgaaactcatctattctacaagattcatggcaagtaaagcttatcttcttggatcactt
actctactagatgactggttaaaacgagatcgatttgtctttattggctggtcaggtcta
ttattatttcctacagcttacttagcagcaggtggatggtttacagccaccgcttttgta
acatctttctttacacatggtctggtcacatcttatctagaagcttgtaattttttaaca
gctgcagtttcaactccagccaactctatggcccattcattactactactctggggacca
gaagcacatcttggatttactgcctgggtttgtataggaggcttatggacatttattgct
tttcatgggctcattggtctcatagcattttccctacgtcagtttgaaatagcacgatta
gttttaattagaccttataatgcacttgcattctcaggaccaatagcagtttatacatct
gtcttcctaatttatccacttggtcaatctagctggttctttgcaccaagctttggtatt
gctgctatcttccgattcctattattccttcaaggttttcataattggacattaaatcct
tttcacatgatgggtgtagctggaatattgggaggtgccctactatcagctatccatggt
gctactgtcattaacactatttatcaagatgctcgtgcatatacaacatttcgtgctttc
tcaccaagtcaaccagaagaaacttattcaatgcttacagctaatcgtttttggtctcag
atatttggtgtagccttctcaaacaagagatggcttcatttcttcatgctatttgtacct
ctagcaggtatgtggacatcttcaattggtatcattggattagcatttaatctaagagct
tatgattttatttctcaagaattgaaagcagcagaagatcctgaatttgaaacattttat
acaaagaacattctactcaatgaaggtatacgactttggatggctgtccaagatcaagct
catgaaaattatcagttcccagaagaggtattgccaagaggtaattctttgtga
>Hd6_psbD
atgaaactcatctattctacaagattcatggcaagtaaagcttatcttcttggatcactt
actctactagatgactggttaaaacgagatcgatttgtctttattggctggtcaggtcta
ttattatttcctacagcttacttagcagcaggtggatggtttacagccaccgcttttgta
acatctttctttacacatggtctggccacatcttatctagaagcttgtaactttttaaca
gctgcagtttcaactccagccaactctatggcccattcattactactactctggggacca
gaagcacatcttggatttactgcctgggtttgtataggaggcttatggacatttattgct
tttcatgggctcattggtctcatagcattttccctacgtcagtttgaaatagcacgatta
gttttaattagaccttataatgcacttgcattctcaggaccaatagcagtttatacatct
gtcttcctaatttatccacttggtcaatctagctggttctttgcaccaagctttggtatt
gctgctatcttccgattcctattattccttcaaggttttcataattggacattaaatcct
tttcacatgatgggtgtagctggaatattgggaggtgccctactatcagctatccatggt
gctactgtcattaacactatttatcaagatgctcgtgcatatacaacatttcgtgctttc
tcaccaagtcaaccagaagaaacttattcaatgcttacagctaatcgtttttggtctcag
atatttggtgtagccttctcaaacaagagatggcttcatttcttcatgctatttgtacct
ctagcaggtatgtggacatcttcaattggtatcattggattagcatttaatctaagagct
tatgattttatttctcaagaattgaaagcagcagaagatcctgaatttgaaacattttat
acaaagaacattctactcaatgaaggtatacgactttggatggctgtccaagatcaagct
catgaaaattatcagttcccagaagaggtattgccaagaggtaattctttgtga

>Hd7_psbD
atgaaactcatctattctacaagattcatggcaagtaaagcttatcttcttggatcactt
actctactagatgactggttaaaacgagatcgatttgtctttattggctggtcaggtcta
ttattatttcctacagcttacttagcagtaggtggatggtttacagctaccacttttgta
acatctttctttacacatggtctggtcacatcttatctagaagcttgtaatttttaaca
gctgcagtttcaactccagccaactctatggcccattcattactactactctggggacca
gaagcacatcttggatttactgcctgggtttgtataggaggcttatggacatttattgct
tttcatgggctcattggtctcataggattttccctacgtcagtttgaaatagcacgatta
gttttaattagaccttataatgcacttgcattctcaggaccaataggagtttatacatct
gtcttcctaatttatccacttggtcaatctagctggttctttgcaccaagctttgggatt
gctgctatcttccgattcctattattccttcaaggttttcataattggacattaaatcct
tttcacatgatgggtgtagctggaatattgggaggtgccctactatcagctatccatggt
gctactgtcattaacactatttatcaagatgctcgtgcatatacaacatttcgtgctttc
tcaccaagtcaaccagaagaaacttattcaatgcttacagctaatcgtttttggtctcag
atatttggtgtagccttctcaaacaagagatggcttcatttcttcatgctatttgtacct
ctagcaggtatgtggacatcttcaattggtatcattggattagcatttaatctaagagct
tatgattttatttctcaagaattgaaagcagcagaagatcctgaatttgaaacattttat
acaaagaacattctactcaatgaaggtatacgactttggatggctgtccaagatcaagct
catgaaaattatcagttcccagaagaggtattgccaagaggtaattctttgtga
>Hd8_psbD
atgaaactcatctattctacaagattcatggcaagtaaagcttatcttcttggatcactt
actctactagatgactggttaaaacgagatcgatttgtctttattggctggtcaggtcta
ttattatttcctacagcttacttagcagcaggtggatggtttacagccaccgcttttgta
acatctttctttacacatggtctggtcacatcttatctagaagcttgtaatttttaaca
gctgcagtttcaactccagccaactctatggcccattcattactactactctggggacca
gaagcacatcttggatttactgcctgggtttgtataggaggcttatggacatttattgct
tttcatgggctcattggtctcatagcattttccctacgtcagtttgaaatagcacgatta
gttttaattagaccttataatgcacttgcattctcaggaccaatagcagtttatacatct
gtcttcctaatttatccacttggtcaatctagctggttctttgcaccaagctttggtatt
gctgctatcttccgattcctattattccttcaaggttttcataattggacattaaatcct
tttcacatgatgggtgtagctggaatattgggaggtgccctactatcagctatccatggt
gctactgtcattaacactatttatcaagatgctcgtgcatatacaacatttcgtgctttc
tcaccaagtcaaccagaagaaacttattcaatgcttacagctaatcgcttttggtctcaa
atatttggtgtagccttctcaaacaagagatggcttcatttcttcatgctatttgtacct
ctagcaggtatgtggacatcttcaattggtatcattggattagcatttaatctaagagct
tatgattttatttctcaagaattgaaagcagcagaagatcctgaatttgaaacattttat
acaaagaacattctactcaatgaaggtatacgactttggatggctgtccaagatcaagct
catgaaaattatcagttcccagaagaggtattgccaagaggtaattctttgtga
>Hd9_psbD
atgaaactcatctattctacaagattcatggcaagtaaagcttatcttcttggatcactt
actctactagatgactggttaaaacgagatcgatttgtctttattggctggtcaggtcta
ttattatttcctacagcttacttagcagtaggtggatggtttacagctaccacttttgta
acatctttctttacacatggtttggccacatcttatctagaagcttgtaactttttaaca
gctgcagtttcaactccagccaactctatggcccattcattactactactctggggacca
gaagcacatcttggatttactgcctgggtttgtataggaggcttatggacatttattgct
tttcatgggctcattggtctcataggattttccctacgtcagtttgaaatagcacgatta
gttttaattagaccttataatgcacttgcattctcaggaccaatagcagtttatacatct
gtcttcctaatttatccacttggtcaatctagctggttctttgcaccaagctttggtatt
gctgctatcttccgattcctattattccttcaaggttttcataattggacattaaatcct
tttcacatgatgggtgtagctggaatattgggaggtgcattactatcagctatccatggt
gctactgtcattaacactatttatcaagatgctcgtgcatatacaacatttcgtgctttc
tcacctagtcaaccagaagaaacttattcaatgcttacagctaatcgcttctggtctcaa
atatttggtgtagccttctcaaacaagagatggcttcatttcttcatgctatttgtacct
ctagcaggtatgtggacatcttcaattggtatcattggattagcatttaatctaagagct
tatgattttatttctcaagaattgaaagcagcagaagatcctgaatttgaaacattttat
acaaagaacattctactcaatgaaggtatacgactttggatggctgtccaagatcaagct
catgaaaattatcagttcccagaagaggtattgccaagaggtaattctttgtga

>IS1_psbD
atgaaactcatctattctacaagattcatgtcaagtaaatcttatcttcttggatcactt
actctactagatgactggttaaaacgagatcgatttgtctttattggctggtcaggtcta
ttattatttcctacagcttacttagcagtaggtggatggttcacagccacaacttttgta
acatctttctttacacatggtttggccacatcttatctagaagcttgtaacttttttaaca
gctgcagtttcaactccagccaactctatggcccattcattactactactctggggacca
gaagcacatcttggatttactgcctgggtttgtataggaggcttatggacatttattgct
tttcatgggctcattggtctcataggattttccctacgtcagtttgaaatagcacgatta
gttttaattaggccttataatgcacttgcattctcagggccaatagcagtttatacatct
gtcttcctaatttatccacttggtcaatctagctggttctttgcaccaagctttgggatt
gctgctatcttccgattcctattattccttcaaggttttcataattggacattaaatcct
tttcacatgatgggtgtagctggaatattaggaggtgcattactatcagctatccatggt
gctactgtcattaacactatttatcaagatgctcgtgcatatacaacatttcgtgctttc
tcacctagtcaaccagaagaaacttattcaatgcttacagctaatcgtttttggtctcag
gtatttggtgtagctttctcaaacaagagatggcttcatttcttcatgttattcgtacct
gtagcaggtatgtggacatcttcaattggtatcattggattagcatttaatctaagagct
tatgattttatttctcaagaattgaaagcagcagaagatcctgaatttgaaacattttat
acaaagaacattctactcaatgaaggtatacgactttggatggctgtccaagatcaagct
catgaaaattatcagttcccagaagaggtattgccaagaggtaattctttgtga
>IS2_psbD
atgaaactcatctattctacaagattcatggcaagtaaagcttatcttcttggatcactt
actctactagatgactggttaaaacgagatcgatttgtctttattggctggtcaggtcta
ttattatttcctacagcttacttagcagcaggtggatggtttacagccaccgcttttgta
acatctttctttacacatggtctggtcacatcttatctagaagcttgtaatttttttaaca
gctgcagtttcaactccagccaactctatggcccattcattactactactctggggacca
gaagcacatcttggatttactgcctgggtttgtataggaggcttatggacatttattgct
tttcatgggctcattggtctcatagcattttccctacgtcagtttgaaatagcacgatta
gttttaattagaccttataatgcacttgcattctcaggaccaatagcagtttatacatct
gtcttcctaatttatccacttggtcaatctagctggttctttgcaccaagctttggtatt
gctgctatcttccgattcctattattccttcaaggttttcataattggacattaaatcct
tttcacatgatgggtgtagctggaatattgggaggtgccctactatcagctatccatggt
gctactgtcattaacactatttatcaagatgctcgtgcatatacaacatttcgtgctttc
tcaccaagtcaaccagaagaaacttattcaatgcttacagctaatcgtttttggtctcag
atatttggtgtagccttctcaaacaagagatggcttcatttcttcatgctatttgtacct
ctagcaggtatgtggacatcttcaattggtatcattggattagcatttaatctaagagct
tatgattttatttctcaagaattgaaagcagcagaagatcctgaatttgaaacattttat
acaaagaacattctactcaatgaaggtatacgactttggatggctgtccaagatcaagct
catgaaaattatcagttcccagaagaggtattgccaagaggtaattctttgtga
>IS3_psbD
atgaaactcatctattctacaagattcatgtcaagtaaatcttatcttcttggatcactt
actctactagatgactggttaaaacgagatcgctttgtctttattgggtggtcaggtctc
ttattatttcctacagcttacttagcagcaggaggatggttcacagccacaacttttgta
acatctttctttacacatggcttagcgacatcttatcttgaagcttgtaatttttttaaca
gctgcagcttcaactccagccaactctatggcccattcattactcctactctggggtcca
gaagcacatcttggatttactgcctggctttgtataggaggtttatggacatttattgct
tttcatgggcttattgggcttatagcattttcactacgtcagtttgaaatagcaagatta
gttttaattaggccttataatgcacttgcattctcagggccaatagcagtttatacatct
gtcttcctaatttatccccttggacaatctagttggttctttgctcctagctttggtatt
gctgctatattccgattcctattattccttcaaggttttcataattggacattaaatcct
tttcacatgatgggtgtagctggaatattaggaggtgctttactatcagctatccacggt
gctactgttattaatactatttatcaagatgctcgtgcatatacaacattccgtgctttc
tcaccaaatcaaccagaagaaacttattcaatgcttacagctaatcgtttttggtctcag
gtatttggtgtagctttctcaaacaagagatggcttcatttcttcatgttattcgtacct
gtagcaggtatgtggacatcttcaattggtatccttggtttagcatttaatctaagagca
tatgattttatttcccaagaattaaaagcagcagaagatcctgaatttgaaacattttat
acaaagaacattctactcaatgaaggaataagactttggatggcagtacaagatcaacct
catgaaaattatcagtttccagaagaggtattacctagaggtaattctttgtga

>IS4_psbD
atgaaactcatctattctacaagattcatggcaagtaaagcttatcttcttggatcactt
actctactagatgactggttaaaacgagatcgctttgtctttattgggtggtcaggtcta
ttattatttcctacagcttacttagcagcaggtggatggtttacagccacagcttttgta
acatctttctttacacatggcttagcgacatcttatcttgaagcttgtaatttttaaca
gctgcagcttcaactccagctaactctatggctcattcattactcctactctggggtcca
gaagcacatcttggatttactgcctggctttgtataggaggtttatggacatttattgct
tttcatggtcttattgggcttatagcattttcactacgtcagtttgaaatagcaagatta
gttttaattaggccttataatgcacttgcattctcagggccaatagcagtttatacatct
gtcttcctaatttatccccttggacaatctagttggttctttgctcctagctttggtatt
gctgctatattccgattcctattattccttcaaggttttcataattggacattaaatcct
tttcacatgatgggtgtagctggaatattaggaggtgccctactatcagctatccacggg
gctactgtcattaatactatttatcaagatgctggtgcatactccacattccgtgctttc
tcaccaaatcaaccagaagaaacttattcaatgcttacagctaatcgtttttggtctcag
gtatttggtgtagctttctcaaacaagagatggcttcatttcttcatgttattcgtacct
gtagcaggtatgtggacatcttcaattggtatccttggtttagcatttaatctaagagca
tatgattttatttcccaagaattaaaagcagcagaagatcctgaatttgaaacattttat
acaaagaacattctactcaatgaaggaataagactttggatggcagtacaagatcaacct
catgaaaattatcagtttccagaagaggtattacctagaggtaattctttgtga
>IS5_psbD
atgaaactcatctattctacaagattcatgtcaagtaaatcttatcttcttggatcactt
actctactagatgactggttaaaacgagatcgatttgtctttattggctggtcaggtcta
ttattatttcctacagcttacttagcagcaggtggatggtttacagccaccgcttttgta
acatctttctttacacatggtctggtcacatcttatctagaagcttgtaatttttaaca
gctgcagtttcaactccagccaactctatggcccattcattactactactctggggacca
gaagcacatcttggatttactgcctgggtttgtataggaggcttatggacatttattgct
tttcatgggctcattggtctcatagcattttccctacgtcagtttgaaatagcacgatta
gttttaattagaccttataatgcacttgcattctcaggaccaatagcagtttatacatct
gtcttcctaatttatccacttggtcaatctagctggttctttgcaccaagctttggtatt
gctgctatcttccgattcctattattccttcaaggttttcataattggacattaaatcct
tttcacatgatgggtgtagctggaatattgggaggtgccctactatcagctatccatggt
gctactgtcattaacactatttatcaagatgctcgtgcatatacaacatttcgtgctttc
tcaccaagtcaaccagaagaaacttattcaatgcttacagctaatcgtttttggtctcag
atatttggtgtagccttctcaaacaagagatggcttcatttcttcatgctatttgtacct
ctagcaggtatgtggacatcttcaattggtatcattggattagcatttaatctaagagct
tatgattttatttctcaagaattgaaagcagcagaagatcctgaatttgaaacattttat
acaaagaacattctactcaatgaaggtatacgactttggatggctgtccaagatcaagct
catgaaaattatcagttcccagaagaggtattgccaagaggtaattctttgtga
>IS6_psbD
atgaaactcatctattctacaagattcatggcaagtaaagcttatcttcttggatcactt
actctactagatgactggttaaaacgagatcgatttgtctttattggctggtcaggtcta
ttattatttcctacagcttacttagcagcaggtggatggtttacagccaccgcttttgta
acatctttctttacacatggtctggtcacatcttatctagaagcttgtaatttttaaca
gctgcagtttcaactccagccaactctatggcccattcattactactactctggggacca
gaagcacatcttggatttactgcctgggtttgtataggaggcttatggacatttattgct
tttcatgggctcattggtctcatagcattttccctacgtcagtttgaaatagcacgatta
gttttaattagaccttataatgcacttgcattctcaggaccaatagcagtttatacatct
gtcttcctaatttatccacttggtcaatctagctggttctttgcaccaagctttggtatt
gctgctatcttccgattcctattattccttcaaggttttcataattggacattaaatcct
tttcacatgatgggtgtagctggaatattgggaggtgccctactatcagctatccatggt
gctactgtcattaacactatttatcaagatgctcgtgcatatacaacatttcgtgctttc
tcaccaagtcaaccagaagaaacttattcaatgcttacagctaatcgtttctggtctcag
atatttggtgtagccttctcaaacaagagatggcttcatttcttcatgctatttgtacct
ctagcaggtatgtggacatcttcaattggtatcattggattagcatttaatctaagagct
tatgattttatttctcaagaattgaaagcagcagaagatcctgaatttgaaacattttat
acaaagaacattctactcaatgaaggtatacgactttggatggctgtccaagatcaagct
catgaaaattatcagttcccagaagaggtattgccaagaggtaattctttgtga

>IS7_psbD
atgaaactcatctattctacaagattcatggcaagtaaagcttatcttcttggatcactt
actctactagatgactggttaaaacgagatcgatttgtctttattggctggtcaggtcta
ttattatttcctacagcttacttagcagtaggtggatggtttacagccaccacttttgta
acatctttctttacacatggtctggtcacatcttatctagaagcttgtaatttttaaca
gctgcagtttcaactccagccaactctatggcccattcattactactactctggggacca
gaagcacatcttggatttactgcctggctttgtataggaggtttatggacatttattgct
tttcatgggctcattggtctcataggattttccctacgtcagtttgaaatagcaagatta
gttttaattaggccttataatgcacttgcattctcagggccaatagcagtttatacatct
gtcttcctaatttatccacttggtcaatctagctggttctttgcaccaagctttgggatt
gctgctatattccgattcctattattccttcaaggttttcataattggacattaaatcct
tttcacatgatgggtgtagctggaatattaggaggtgctttactatcagctatccatggt
gctactgtcattaacactatttatcaagatgctcgtgcatatacaacatttcgtgctttc
tcaccaagtcaaccagaagaaacttattcaatgcttacagctaatcgcttctggtctcaa
atatttggtgtagccttctcaaacaagagatggcttcatttcttcatgctatttgtacct
ctagcaggtatgtggacatcttcaattggtatcattggtttagcatttaatctaagagca
tatgattttatttcccaagaattaaaagcagcagaagatcctgaatttgaaacattttat
acaaagaacattctactcaatgaaggaataagactttggatggcagtacaagatcaacct
catgaaaattatcagttcccagaagaggtattgccaagaggtaattctttgtga
>IS8_psbD
atgaaactcatctattctacaagattcatggcaagtaaagcttatcttcttggatcactt
actctactagatgactggttaaaacgagatcgatttgtctttattggctggtcaggtcta
ttattatttcctacagcttacttagcagcaggtggatggtttacagccaccgcttttgta
acatctttctttacacatggtctggtcacatcttatctagaagcttgtaatttttaaca
gctgcagtttcaactccagccaactctatggcccattcattactactactctggggacca
gaagcacatcttggatttactgcctgggtttgtataggaggcttatggacatttattgct
tttcatgggctcattggtctcatagcattttccctacgtcagtttgaaatagcacgatta
gttttaattagaccttataatgcacttgcattctcaggaccaatagcagtttatacatct
gtcttcctaatttatccacttggtcaatctagctggttctttgcaccaagctttggtatt
gctgctatcttccgattcctattattccttcaaggttttcataattggacattaaatcct
tttcacatgatgggtgtagctggaatattgggaggtgccctactatcagctatccatggt
gctactgtcattaacactatttatcaagatgctcgtgcatatacaacatttcgtgctttc
tcaccaagtcaaccagaagaaacttattcaatgcttacagctaatcgtttttggtctcag
atatttggtgtagccttctcaaacaagagatggcttcatttcttcatgctatttgtacct
ctagcaggtatgtggacatcttcaattggtatcattggattagcatttaatctaagagct
tatgattttatttctcaagaattgaaagcagcagaagatcctgaatttgaaacattttat
acaaagaacattctactcaatgaaggtatacgactttggatggctgtccaagatcaagct
catgaaaattatcagttcccagaagaggtattgccaagaggtaattctttgtga
>IS9_psbD
atgaaactcatctattctacaagattcatggcaagtaaagcttatcttcttggatcactt
actctactagatgactggttaaaacgagatcgctttgtctttattgggtggtcaggtctc
ttattgtttcctacagcttatttagcagcaggaggatggttcacagccacaacttttgta
acatctttctttacacatggcctagtgacatcttatctagaagcttgtaactttttaaca
gctgcagtttcaactccagccaactctatggcccattcattactactactctggggacca
gaagcacatcttggatttactgcctgggtttgtataggaggcttatggacatttattgct
tttcatgggctcattggtctcatagcattttccctacgtcagtttgaaatagcaagatta
gttttaattagaccttataatgcacttgcattctcaggaccaataggagtttatacatct
gtcttcctaatttatccacttggtcaatctagctggttctttgcacctagctttggtatt
gctgctatattccgattcctattattccttcaaggttttcataattggacattaaatcct
tttcacatgatgggtgtagctggaatattaggaggtgctttactatcagctatccatggg
gctactgttattaacactatttatcaagatgctggtgcatactccacattccgtgctttc
tcaccaaatcaaccagaagaaacttattcaatgcttacagctaatcgtttttggtctcag
atatttggtgtagccttctcaaacaagagatggcttcatttcttcatgctatttgtacct
ctagcaggtatgtggacatcttcaattggtatcattggattagcatttaatctaagagct
tatgattttatttctcaagaattgaaagcagcagaagatcctgaatttgaaacattttat
acaaagaacattctactcaatgaaggtatacgactttggatggctgtccaagatcaagct
catgaaaattatcagttcccagaagaggtattgccaagaggtaattctttgtga

>Ik1_psbD
atgaaactcatctattctacaagattcatggcaagtaaagcttatcttcttggatcactt
actctactagatgactggttaaaacgagatcgatttgtctttattggctggtcaggtcta
ttattatttcctacagcttacttagcagtaggtggatggtttacagccaccgcttttgta
acatctttctttacacatggtctggtcacatcttatctagaagcttgtaatttttaaca
gctgcagtttcaactccagccaactctatggcccattcattactactactctggggacca
gaagcacatcttggatttactgcctgggtttgtataggaggcttatggacatttattgct
tttcatgggctcattggtctcataggattttccctacgtcagtttgaaatagcacgatta
gttttaattagaccttataatgcacttgcattctcaggaccaatagcagtttatacatct
gtcttcctaatttatccacttggtcaatctagctggttctttgcaccaagctttggtatt
gctgctatcttccgattcctattattccttcaaggttttcataattggacattaaatcct
tttcacatgatgggtgtagctggaatattgggaggtgccctactatcagctatccatggt
gctactgtcattaacactatttatcaagatgctcgtgcatatacaacatttcgtgctttc
tcacctagtcaaccagaagaaacttattcaatgcttacagctaatcgtttctggtctcag
atatttggtgtagccttctcaaacaagagatggcttcatttcttcatgctatttgtacct
ctagcaggtatgtggacatcttcaattggtatcattggattagcatttaatctaagagct
tatgattttatttctcaagaattgaaagcagcagaagatcctgaatttgaaacattttat
acaaagaacattctactcaatgaaggtatacgactttggatggctgtccaagatcaagct
catgaaaattatcagttcccagaagaggtattgccaagaggtaattctttgtga
>Ik2_psbD
atgaaactcatctattctacaagattcatggcaagtaaagcttatcttcttggatcactt
actctactagatgactggttaaaacgagatcgatttgtctttattggctggtcaggtcta
ttattatttcctacagcttacttagcagcaggtggatggtttacagccaccgcttttgta
acatctttctttacacatggtctggtcacatcttatctagaagcttgtaatttttaaca
gctgcagtttcaactccagccaactctatggcccattcattactactactctggggacca
gaagcacatcttggatttactgcctgggtttgtataggaggcttatggacatttattgct
tttcatgggctcattggtctcatagcattttccctacgtcagtttgaaatagcacgatta
gttttaattagaccttataatgcacttgcattctcaggaccaatagcagtttatacatct
gtcttcctaatttatccacttggtcaatctagctggttctttgcaccaagctttggtatt
gctgctatcttccgattcctattattccttcaaggttttcataattggacattaaatcct
tttcacatgatgggtgtagctggaatattgggaggtgccctactatcagctatccatggt
gctactgtcattaacactatttatcaagatgctcgtgcatatacaacatttcgtgctttc
tcacctagtcaaccagaagaaacttattcaatgcttacagctaatcgtttttggtctcag
atatttggtgtagccttctcaaacaagagatggcttcatttcttcatgctatttgtacct
ctagcaggtatgtggacatcttcaattggtatcattggattagcatttaatctaagagct
tatgattttatttctcaagaattgaaagcagcagaagatcctgaatttgaaacattttat
acaaagaacattctactcaatgaaggtatacgactttggatggctgtccaagatcaagct
catgaaaattatcagttcccagaagaggtattgccaagaggtaattctttgtga
>Ik3_psbD
atgaaactcatctattctacaagattcatgtcaagtaaagcttatcttcttggatcactt
actctactagatgactggttaaaacgagatcgatttgtctttattggctggtcaggtcta
ttattatttcctacagcttacttagcagcaggtggatggtttacagccaccacttttgta
acatctttctttacacatggtttggccacatcttatctagaagcttgtaatttttaaca
gctgcagtttcaactccagccaactctatggcccattcattactactactctggggacca
gaagcacatcttggatttactgcctgggtttgtataggaggcttatggacatttattgct
tttcatgggctcattggtctcatagcattttccctacgtcagtttgaaatagcacgatta
gttttaattagaccttataatgcacttgcattctcaggaccaatagcagtttatacatct
gtcttcctaatttatccacttggtcaatctagctggttctttgcaccaagctttggtatt
gctgctatcttccgattcctattattccttcaaggttttcataattggacattaaatcct
tttcacatgatgggtgtagctggaatattgggaggtgccctactatcagctatccatggt
gctactgtcattaacactatttatcaagatgctcgtgcatatacaacatttcgtgctttc
tcaccaagtcaaccagaagaaacttattcaatgcttacagctaatcgcttctggtctcaa
atatttggtgtagccttctcaaacaagagatggcttcatttcttcatgctatttgtacct
ctagcaggtatgtggacatcttcaattggtatcattggattagcatttaatctaagagct
tatgattttatttctcaagaattgaaagcagcagaagatcctgaatttgaaacattttat
acaaagaacattctactcaatgaaggtatacgactttggatggctgtccaagatcaagct
catgaaaattatcagttcccagaagaggtattgccaagaggtaattctttgtga

>Ik4_psbD
atgaaactcatctattctacaagattcatggcaagtaaagcttatcttcttggatcactt
actctactagatgactggttaaaacgagatcgatttgtctttattggctggtcaggtcta
ttattatttcctacagcttacttagcagcaggtggatggtttacagccaccgcttttgta
acatctttctttacacatggtctggtcacatcttatctagaagcttgtaattttttaaca
gctgcagtttcaactccagccaactctatggcccattcattactactactctggggacca
gaagcacatcttggatttactgcctgggtttgtataggaggcttatggacatttattgct
tttcatgggctcattggtctcataggattttccctacgtcagtttgaaatagcacgatta
gttttaattagaccttataatgcacttgcattctcaggaccaatagcagtttatacatct
gtcttcctaatttatccacttggtcaatctagctggttctttgcaccaagctttggtatt
gctgctatcttccgattcctattattccttcaaggttttcataattggacattaaatcct
tttcacatgatgggtgtagctggaatattgggaggtgcactactatcagctatccatggt
gctactgtcattaacactatttatcaagatgctcgtgcatatacaacatttcgtgctttc
tcaccaagtcaaccagaagaaacttattcaatgcttacagctaatcgtttttggtctcag
atatttggtgtagccttctcaaacaagagatggcttcatttcttcatgctatttgtacct
ctagcaggtatgtggacatcttcaattggtatcattggattagcatttaatctaagagct
tatgattttatttctcaagaattgaaagcagcagaagatcctgaatttgaaacattttat
acaaagaacattctactcaatgaaggtatacgactttggatggctgtccaagatcaagct
catgaaaattatcagttcccagaagaggtattgccaagaggtaattctttgtga
>Ik5_psbD
atgaaactcatctattctacaagattcatgtcaagtaaatcttatcttcttggatcactt
actctactagatgactggttaaaacgagatcgatttgtctttattggctggtcaggtcta
ttattatttcctacagcttacttagcagcaggtggatggtttacagccaccgcttttgta
acatctttctttacacatggtctggtcacatcttatctagaagcttgtaattttttaaca
gctgcagtttcaactccagccaactctatggcccattcattactactactctggggacca
gaagcacatcttgcatttactgcctgggtttgtataggaggcttatggacatttattgct
tttcatgggctcattggtctcatagcattttccctacgtcagtttgaaatagcacgatta
gttttaattagaccttataatgcacttgcattctcaggaccaatagcagtttatacatct
gtcttcctaatttatccacttggtcaatctagctggttctttgcaccaagctttggtatt
gctgctatcttccgattcctattattccttcaaggttttcataattggacattaaatcct
tttcacatgatgggtgtagctggaatattgggaggtgccctactatcagctatccatggt
gctactgtcattaacactatttatcaagatgctcgtgcatatacaacatttcgtgctttc
tcaccaagtcaaccagaagaaacttattcaatgcttacagctaatcgcttttggtctcag
atatttggtgtagccttctcaaacaagagatggcttcatttcttcatgctatttgtacct
ctagcaggtatgtggacatcttcaattggtatcattggattagcatttaatctaagagct
tatgattttatttctcaagaattgaaagcagcagaagatcctgaatttgaaacattttat
acaaagaacattctactcaatgaaggtatacgactttggatggctgtccaagatcaagct
catgaaaattatcagttcccagaagaggtattgccaagaggtaattctttgtga
>Ik6_psbD
atgaaactcatctattctacaagattcatgtcaagtaaagcttatcttcttggatcactt
actctactagatgactggttaaaacgagatcgatttgtctttattggctggtcaggtcta
ttattatttcctacagcttacttagcagtaggtggatggtttacagctaccacttttgta
acatctttctttacacatggtctggtcacatcttatctagaagcttgtaattttttaaca
gctgcagtttcaactccagccaactctatggcccattcattactactactctggggacca
gaagcacatcttggatttactgcctgggtttgtataggaggcttatggacatttattgct
tttcatgggctcattggtctcataggattttccctacgtcagtttgaaatagcacgatta
gttttaattagaccttataatgcacttgcattctcaggaccaatagcagtttatacatct
gtcttcctaatttatccacttggtcaatctagctggttctttgcaccaagctttgggatt
gctgctatcttccgattcctattattccttcaaggttttcataattggacattaaatcct
tttcacatgatgggtgtagctggaatattgggaggtgccctactatcagctatccatggt
gctactgtcattaacactatttatcaagatgctcgtgcatatacaacatttcgtgctttc
tcaccaagtcaaccagaagaaacttattcaatgcttacagctaatcgtttttggtctcag
atatttggtgtagccttctcaaacaagagatggcttcatttcttcatgctatttgtacct
ctagcaggtatgtggacatcttcaattggtatcattggattagcatttaatctaagagct
tatgattttatttctcaagaattgaaagcagcagaagatcctgaatttgaaacattttat
acaaagaacattctactcaatgaaggtatacgactttggatggctgtccaagatcaagct
catgaaaattatcagttcccagaagaggtattgccaagaggtaattctttgtga

>Ik7_psbD
atgaaactcatctattctacaagattcatggcaagtaaagcttatcttcttggatcactt
actctactagatgactggttaaaacgagatcgatttgtctttattggctggtcaggtcta
ttattatttcctacagcttacttagcagtaggtggatggtttacagctaccacttttgta
acatctttctttacacatggtttggccacatcttatctagaagcttgtaactttttaaca
gctgcagtttcaactccagccaactctatggcccattcattactactactctggggacca
gaagcacatcttggatttactgcctgggtttgtataggaggcttatggacatttattgct
tttcatgggctcattggtctcatagcattttccctacgtcagtttgaaatagcacgatta
gttttaattagaccttataatgcacttgcattctcaggaccaatagcagtttatacatct
gtcttcctaatttatccacttggtcaatctagctggttctttgcaccaagctttggtatt
gctgctatcttccgattcctattattccttcaaggttttcataattggacattaaatcct
tttcacatgatgggtgtagctggaatattgggaggtgccctactatcagctatccatggt
gctactgtcattaacactatttatcaagatgctcgtgcatatacaacatttcgtgctttc
tcacctagtcaaccagaagaaacttattcaatgcttacagctaatcgcttctggtctcaa
atatttggtgtagccttctcaaacaagagatggcttcatttcttcatgctatttgtacct
ctagcaggtatgtggacatcttcaattggtatcattggattagcatttaatctaagagct
tatgattttatttctcaagaattgaaagcagcagaagatcctgaatttgaaacattttat
acaaagaacattctactcaatgaaggtatacgactttggatggctgtccaagatcaagct
catgaaaattatcagttcccagaagaggtattgccaagaggtaattctttgtga
>Ik8_psbD
atgaaactcatctattctacaagattcatgtcaagtaaatcttatcttcttggatcactt
actctactagatgactggttaaaacgagatcgatttgtctttattggctggtcaggtcta
ttattatttcctacagcttacttagcagtaggtggatggtttacagccaccgcttttgta
acatctttctttacacatggtctggtcacatcttatctagaagcttgtaactttttaaca
gctgcagtttcaactccagccaactctatggcccattcattactactactctggggacca
gaagcacatcttggatttactgcctgggtttgtataggaggcttatggacatttattgct
tttcatgggctcattggtctcatagcattttccctacgtcagtttgaaatagcacgatta
gttttaattagaccttataatgcacttgcattctcaggaccaatagcagtttatacatct
gtcttcctaatttatccacttggtcaatctagctggttctttgcaccaagctttggtatt
gctgctatcttccgattcctattattccttcaaggttttcataattggacattaaatcct
tttcacatgatgggtgtagctggaatattgggaggtgccctactatcagctatccatggt
gctactgtcattaacactatttatcaagatgctcgtgcatatacaacatttcgtgctttc
tcaccaagtcaaccagaagaaacttattcaatgcttacagctaatcgtttttggtctcag
atatttggtgtagccttctcaaacaagagatggcttcatttcttcatgctatttgtacct
ctagcaggtatgtggacatcttcaattggtatcattggattagcatttaatctaagagct
tatgattttatttctcaagaattgaaagcagcagaagatcctgaatttgaaacattttat
acaaagaacattctactcaatgaaggtatacgactttggatggctgtccaagatcaagct
catgaaaattatcagttcccagaagaggtattgccaagaggtaattctttgtga
>Ik9_psbD
atgaaactcatctattctacaagattcatggcaagtaaagcttatcttcttggatcactt
actctactagatgactggttaaaacgagatcgatttgtctttattggctggtcaggtcta
ttattatttcctacagcttacttagcagcaggtggatggtttacagccaccgcttttgta
acatctttctttacacatggtctggtcacatcttatctagaagcttgtaatttttttaaca
gctgcagtttcaactccagccaactctatggcccattcattactactactctggggacca
gaagcacatcttggatttactgcctgggtttgtataggaggcttatggacatttattgct
tttcatgggctcattggtctcatagcattttccctacgtcagtttgaaatagcacgatta
gttttaattagaccttataatgcacttgcattctcaggaccaatagcagtttatacatct
gtcttcctaatttatccacttggtcaatctagctggttctttgcaccaagctttggtatt
gctgctatcttccgattcctattattccttcaaggttttcataattggacattaaatcct
tttcacatgatgggtgtagctggaatattgggaggtgccctactatcagctatccatggt
gctactgtcattaacactatttatcaagatgctcgtgcatatacaacatttcgtgctttc
tcaccaagtcaaccagaagaaacttattcaatgcttacagctaatcgtttttggtctcag
atatttggtgtagccttctcaaacaagagatggcttcatttcttcatgctatttgtacct
ctagcaggtatgtggacatcttcaattggtatcattggattagcatttaatctaagagct
tatgattttatttctcaagaattgaaagcagcagaagatcctgaatttgaaacattttat
acaaagaacattctactcaatgaaggtatacgactttggatggctgtccaagatcaagct
catgaaaattatcagttcccagaagaggtattgccaagaggtaattctttgtga

>Irm10_psbD
atgaaactcatctattctacaagattcatggcaagtaaagcttatcttcttggatcactt
actctactagatgactggttaaaacgagatcgatttgtctttattggctggtcaggtcta
ttattatttcctacagcttacttagcagcaggtggatggtttacagccaccgcttttgta
acatctttctttacacatggtctggtcacatcttatctagaagcttgtaatttttaaca
gctgcagtttcaactccagccaactctatggcccattcattactactactctggggacca
gaagcacatcttggatttactgcctgggtttgtataggaggcttatggacatttattgct
tttcatgggctcattggtctcatagcattttccctacgtcagtttgaaatagcacgatta
gttttaattagaccttataatgcacttgcattctcaggaccaatagcagtttatacatct
gtcttcctaatttatccacttggtcaatctagctggttctttgcaccaagctttggtatt
gctgctatcttccgattcctattattccttcaaggttttcataattggacattaaatcct
tttcacatgatgggtgtagctggaatattgggaggtgcattactatcagctatccatggt
gctactgtcattaacactatttatcaagatgctcgtgcatatacaacatttcgtgctttc
tcaccaagtcaaccagaagaaacttattcaatgcttacagctaatcgcttttggtctcag
atatttggtgtagccttctcaaacaagagatggcttcatttcttcatgctatttgtacct
ctagcaggtatgtggacatcttcaattggtatcattggattagcatttaatctaagagct
tatgattttatttctcaagaattgaaagcagcagaagatcctgaatttgaaacattttat
acaaagaacattctactcaatgaaggtatacgactttggatggctgtccaagatcaagct
catgaaaattatcagttcccagaagaggtattgccaagaggtaattctttgtga
>Irm17_psbD
atgaaactcatctattctacaagattcatggcaagtaaagcttatcttcttggatcactt
actctactagatgactggttaaaacgagatcgatttgtctttattggctggtcaggtcta
ttattatttcctacagcttacttagcagcaggtggatggtttacagccaccgcttttgta
acatctttctttacacatggtctggtcacatcttatctagaagcttgtaatttttaaca
gctgcagtttcaactccagccaactctatggcccattcattactactactctggggacca
gaagcacatcttggatttactgcctgggtttgtataggaggcttatggacatttattgct
tttcatgggctcattggtctcatagcattttccctacgtcagtttgaaatagcacgatta
gttttaattagaccttataatgcacttgcattctcaggaccaatagcagtttatacatct
gtcttcctaatttatccacttggtcaatctagctggttctttgcaccaagctttggtatt
gctgctatcttccgattcctattattccttcaaggttttcataattggacattaaatcct
tttcacatgatgggtgtagctggaatattgggaggtgccctactatcagctatccatggt
gctactgtcattaacactatttatcaagatgctcgtgcatatacaacatttcgtgctttc
tcaccaagtcaaccagaagaaacttattcaatgcttacagctaatcgtttttggtctcag
atatttggtgtagccttctcaaacaagagatggcttcatttcttcatgctatttgtacct
ctagcaggtatgtggacatcttcaattggtatcattggattagcatttaatctaagagct
tatgattttatttctcaagaattgaaagcagcagaagatcctgaatttgaaacattttat
acaaagaacattctactcaatgaaggtatacgactttggatggctgtccaagatcaagct
catgaaaattatcagttcccagaagaggtattgccaagaggtaattctttgtga
>Irm2_psbD
atgaaactcatctattctacaagattcatggcaagtaaagcttatcttcttggatcactt
actctactagatgactggttaaaacgagatcgatttgtctttattggctggtcaggtcta
ttattatttcctacagcttacttagcagcaggtggatggtttacagccaccgcttttgta
acatctttctttacacatggtctggtcacatcttatctagaagcttgtaatttttaaca
gctgcagtttcaactccagccaactctatggcccattcattactactactctggggacca
gaagcacatcttggatttactgcctgggtttgtataggaggcttatggacatttattgct
tttcatgggctcattggtctcatagcattttccctacgtcagtttgaaatagcacgatta
gttttaattagaccttataatgcacttgcattctcaggaccaatagcagtttatacatct
gtcttcctaatttatccacttggtcaatctagctggttctttgcaccaagctttggtatt
gctgctatcttccgattcctattattccttcaaggttttcataattggacattaaatcct
tttcacatgatgggtgtagctggaatattgggaggtgccctactatcagctatccatggt
gctactgtcattaacactatttatcaagatgctcgtgcatatacaacatttcgtgctttc
tcaccaagtcaaccagaagaaacttattcaatgcttacagctaatcgtttttggtctcag
atatttggtgtagccttctcaaacaagagatggcttcatttcttcatgctatttgtacct
ctagcaggtatgtggacatcttcaattggtatcattggattagcatttaatctaagagct
tatgattttatttctcaagaattgaaagcagcagaagatcctgaatttgaaacattttat
acaaagaacattctactcaatgaaggtatacgactttggatggctgtccaagatcaagct
catgaaaattatcagttcccagaagaggtattgccaagaggtaattctttgtga

>Irm21_psbD
atgaaactcatctattctacaagattcatggcaagtaaagcttatcttcttggatcactt
actctactagatgactggttaaaacgagatcgatttgtctttattggctggtcaggtcta
ttattatttcctacagcttacttagcagcaggtggatggtttacagccaccgcttttgta
acatctttctttacacatggtctggtcacatcttatctagaagcttgtaatttttaaca
gctgcagtttcaactccagccaactctatggcccattcattactactactctggggacca
gaagcacatcttggatttactgcctgggtttgtataggaggcttatggacatttattgct
tttcatgggctcattggtctcatagcattttccctacgtcagtttgaaatagcacgatta
gttttaattagaccttataatgcacttgcattctcaggaccaatagcagtttatacatct
gtcttcctaatttatccacttggtcaatctagctggttctttgcaccaagctttggtatt
gctgctatcttccgattcctattattccttcaaggttttcataattggacattaaatcct
tttcacatgatgggtgtagctggaatattgggaggtgccctactatcagctatccatggt
gctactgtcattaacactatttatcaagatgctcgtgcatatacaacatttcgtgctttc
tcacctagtcaaccagaagaaacttattcaatgcttacagctaatcgtttttggtctcag
atatttggtgtagccttctcaaacaagagatggcttcatttcttcatgctatttgtacct
ctagcaggtatgtggacatcttcaattggtatcattggattagcatttaatctaagagct
tatgattttatttctcaagaattgaaagcagcagaagatcctgaatttgaaacattttat
acaaagaacattctactcaatgaaggtatacgactttggatggctgtccaagatcaagct
catgaaaattatcagttcccagaagaggtattgccaagaggtaattctttgtga
>Irm22_psbD
atgaaactcatctattctacaagattcatgtcaagtaaatcttatcttcttggatcactt
actctactagatgactggttaaaacgagatcgatttgtctttattggctggtcaggtcta
ttattatttcctacagcttacttagcagcaggtggatggtttacagccaccgcttttgta
acatctttctttacacatggtctggtcacatcttatctagaagcttgtaatttttaaca
gctgcagtttcaactccagccaactctatggcccattcattactactactctggggacca
gaagcacatcttggatttactgcctgggtttgtataggaggcttatggacatttattgct
tttcatgggctcattggtctcataggattttccctacgtcagtttgaaatagcacgatta
gttttaattagaccttataatgcacttgcattctcaggaccaataggagtttatacatct
gtcttcctaatttatccacttggtcaatctagctggttctttgcaccaagctttgggatt
gctgctatcttccgattcctattattccttcaaggttttcataattggacattaaatcct
tttcacatgatgggtgtagctggaatattgggaggtgcattactatcagctatccatggt
gctactgtcattaacactatttatcaagatgctcgtgcatatacaacatttcgtgctttc
tcacctagtcaaccagaagaaacttattcaatgcttacagctaatcgcttctggtctcaa
atatttggtgtagccttctcaaacaagagatggcttcatttcttcatgctatttgtacct
ctagcaggtatgtggacatcttcaattggtatcattggattagcatttaatctaagagct
tatgattttatttctcaagaattgaaagcagcagaagatcctgaatttgaaacattttat
acaaagaacattctactcaatgaaggtatacgactttggatggctgtccaagatcaagct
catgaaaattatcagttcccagaagaggtattgccaagaggtaattctttgtga
>Irm23_psbD
atgaaactcatctattctacaagattcatggcaagtaaagcttatcttcttggatcactt
actctactagatgactggttaaaacgagatcgatttgtctttattggctggtcaggtcta
ttattatttcctacagcttacttagcagcaggtggatggtttacagccaccgcttttgta
acatctttctttacacatggtttggccacatcttatctagaagcttgtaatttttaaca
gctgcagtttcaactccagccaactctatggcccattcattactactactctggggacca
gaagcacatcttggatttactgcctgggtttgtataggaggcttatggacatttattgct
tttcatgggctcattggtctcatagcattttccctacgtcagtttgaaatagcacgatta
gttttaattagaccttataatgcacttgcattctcaggaccaataggagtttatacatct
gtcttcctaatttatccacttggtcaatctagctggttctttgcaccaagctttgggatt
gctgctatcttccgattcctattattccttcaaggttttcataattggacattaaatcct
tttcacatgatgggtgtagctggaatattgggaggtgccctactatcagctatccatggt
gctactgtcattaacactatttatcaagatgctcgtgcatatacaacatttcgtgctttc
tcacctagtcaaccagaagaaacttattcaatgcttacagctaatcgtttttggtctcag
atatttggtgtagccttctcaaacaagagatggcttcatttcttcatgctatttgtacct
ctagcaggtatgtggacatcttcaattggtatcattggattagcatttaatctaagagct
tatgattttatttctcaagaattgaaagcagcagaagatcctgaatttgaaacattttat
acaaagaacattctactcaatgaaggtatacgactttggatggctgtccaagatcaagct
catgaaaattatcagttcccagaagaggtattgccaagaggtaattctttgtga

>Irm24_psbD
atgaaactcatctattctacaagattcatggcaagtaaagcttatcttcttggatcactt
actctactagatgactggttaaaacgagatcgatttgtctttattggctggtcaggtcta
ttattatttcctacagcttacttagcagtaggtggatggtttacagctaccacttttgta
acatctttctttacacatggtttggccacatcttatctagaagcttgtaactttttaaca
gctgcagtttcaactccagccaactctatggcccattcattactactactctggggacca
gaagcacatcttggatttactgcctgggtttgtataggaggcttatggacatttattgct
tttcatgggctcattggtctcataggattttccctacgtcagtttgaaatagcacgatta
gttttaattagaccttataatgcacttgcattctcaggaccaatagcagtttatacatct
gtcttcctaatttatccacttggtcaatctagctggttctttgcaccaagctttgggatt
gctgctatcttccgattcctattattccttcaaggttttcataattggacattaaatcct
tttcacatgatgggtgtagctggaatattgggaggtgcattactatcagctatccatggt
gctactgtcattaacactatttatcaagatgctcgtgcatatacaacatttcgtgctttc
tcaccaagtcaaccagaagaaacttattcaatgcttacagctaatcgtttttggtctcaa
atatttggtgtagccttctcaaacaagagatggcttcatttcttcatgctatttgtacct
ctagcaggtatgtggacatcttcaattggtatcattggattagcatttaatctaagagct
tatgattttatttctcaagaattgaaagcagcagaagatcctgaatttgaaacattttat
acaaagaacattctactcaatgaaggtatacgactttggatggctgtccaagatcaagct
catgaaaattatcagttcccagaagaggtattgccaagaggtaattctttgtga
>Irm25_psbD
atgaaactcatctattctacaagattcatggcaagtaaagcttatcttcttggatcactt
actctactagatgactggttaaaacgagatcgatttgtctttattggctggtcaggtcta
ttattatttcctacagcttacttagcagtaggtggatggtttacagctaccgcttttgta
acatctttctttacacatggtctggtcacatcttatctagaagcttgtaatttttttaaca
gctgcagtttcaactccagccaactctatggcccattcattactactactctggggacca
gaagcacatcttggatttactgcctgggtttgtataggaggcttatggacatttattgct
tttcatgggctcattggtctcataggattttccctacgtcagtttgaaatagcacgatta
gttttaattagaccttataatgcacttgcattctcaggaccaataggagtttatacatct
gtcttcctaatttatccacttggtcaatctagctggttctttgcaccaagctttgggatt
gctgctatcttccgattcctattattccttcaaggttttcataattggacattaaatcct
tttcacatgatgggtgtagctggaatattgggaggtgcattactatcagctatccatggt
gctactgtcattaacactatttatcaagatgctcgtgcatatacaacatttcgtgctttc
tcaccaagtcaaccagaagaaacttattcaatgcttacagctaatcgtttctggtctcag
atatttggtgtagccttctcaaacaagagatggcttcatttcttcatgctatttgtacct
ctagcaggtatgtggacatcttcaattggtatcattggattagcatttaatctaagagct
tatgattttatttctcaagaattgaaagcagcagaagatcctgaatttgaaacattttat
acaaagaacattctactcaatgaaggtatacgactttggatggctgtccaagatcaagct
catgaaaattatcagttcccagaagaggtattgccaagaggtaattctttgtga
>Irm26_psbD
atgaaactcatctattctacaagattcatgtcaagtaaagcttatcttcttggatcactt
actctactagatgactggttaaaacgagatcgatttgtctttattggctggtcaggtcta
ttattatttcctacagcttacttagcagtaggtggatggtttacagccaccacttttgta
acatctttctttacacatggtctggtcacatcttatctagaagcttgtaatttttttaaca
gctgcagtttcaactccagccaactctatggcccattcattactactactctggggacca
gaagcacatcttggatttactgcctgggtttgtataggaggcttatggacatttattgct
tttcatgggctcattggtctcataggattttccctacgtcagtttgaaatagcacgatta
gttttaattagaccttataatgcacttgcattctcaggaccaatagcagtttatacatct
gtcttcctaatttatccacttggtcaatctagctggttctttgcaccaagctttggtatt
gctgctatcttccgattcctattattccttcaaggttttcataattggacattaaatcct
tttcacatgatgggtgtagctggaatattgggaggtgccctactatcagctatccatggt
gctactgtcattaacactatttatcaagatgctcgtgcatatacaacatttcgtgctttc
tcacctagtcaaccagaagaaacttattcaatgcttacagctaatcgtttttggtctcag
atatttggtgtagccttctcaaacaagagatggcttcatttcttcatgctatttgtacct
ctagcaggtatgtggacatcttcaattggtatcattggattagcatttaatctaagagct
tatgattttatttctcaagaattgaaagcagcagaagatcctgaatttgaaacattttat
acaaagaacattctactcaatgaaggtatacgactttggatggctgtccaagatcaagct
catgaaaattatcagttcccagaagaggtattgccaagaggtaattctttgtga

>Irm27_psbD
atgaaactcatctattctacaagattcatgtcaagtaaatcttatcttcttggatcactt
actctactagatgactggttaaaacgagatcgatttgtctttattggctggtcaggtcta
ttattatttcctacagcttacttagcagtaggtggatggtttacagctaccacttttgta
acatctttctttacacatggtttggccacatcttatctagaagcttgtaactttttaaca
gctgcagtttcaactccagccaactctatggcccattcattactcctactctggggacca
gaagcacatcttggatttactgcctggctttgtataggaggtttatggacatttattgct
tttcatgggcttattgggcttataggattttccctacgtcagtttgaaatagcacgatta
gttttaattagaccttataatgcacttgcattctcaggaccaataggagtttatacatct
gtcttcctaatttatccacttggtcaatctagctggttctttgcaccaagctttgggatt
gctgctatcttccgattcctattattccttcaaggttttcataattggacattaaatcct
tttcacatgatgggtgtagctggaatattaggaggtgctttactatcagctatccacggt
gctactgtcattaacactatttatcaagatgctcgtgcatatacaacatttcgtgctttc
tcaccaaatcaaccagaagaaacttattcaatgcttacagctaatcgtttttggtctcag
gtatttggtgtagctttctcaaacaagagatggcttcatttcttcatgttattcgtacct
gtagcaggtatgtggacatcttcaattggtatcattggattagcatttaatctaagagct
tatgattttatttctcaagaattgaaagcagcagaagatcctgaatttgaaacattttat
acaaagaacattctactcaatgaaggtatacgactttggatggctgtccaagatcaagct
catgaaaattatcagttcccagaagaggtattgccaagaggtaattctttgtga
>Irm3_psbD
atgaaactcatctattctacaagattcatgtcaagtaaatcttatcttcttggttcactt
actctattagatgactggttaaaacgagatcgctttgtctttattgggtggtcaggtctc
ttattgtttcctacagcttatttagcagcaggaggatggttcacagccacaacttttgta
acatctttctttacacatggcttagcgacatcttatcttgaagcttgtaatttttttaaca
gctgcagcttcaactccagctaactctatggctcattcattactcctactctggggtcca
gaagcacatcttggatttactgcctggctttgtataggaggtttatggacatttattgct
tttcatggtcttattgggcttataggattttccctacgtcagtttgaaatagcaagatta
gttttaattaggccttataatgcacttgcattctcagggccaatagcagtttatacatct
gtcttcctaatttatccccttggacaatctagttggttctttgctcctagctttggtatt
gctgctatattccgattcctattattccttcaaggttttcataattggacattaaatcct
tttcacatgatgggtgtagctggaatattaggaggtgctttactatcagctatccacggg
gctactgttattaatactatttatcaagatgctggtgcatactccacattccgtgctttc
tcaccaaatcaaccagaagaaacttattcaatgcttacagctaatcgtttttggtctcag
gtatttggtgtagctttctcaaacaagagatggcttcatttcttcatgttattcgtacct
gtagcaggtatgtggacatcttcaattggtatcattggattagcatttaatctaagagct
tatgattttatttcccaagaattaaaagcagcagaagatcctgaatttgaaacattttat
acaaagaacattctactcaatgaaggaataagactttggatggcagtacaagatcaacct
catgaaaattatcagtttccagaagaggtattacctagaggtaattctttgtga
>Irm4_psbD
atgaaactcatctattctacaagattcatgtcaagtaaatcttatcttcttggatcactt
actctactagatgactggttaaaacgagatcgatttgtctttattggctggtcaggtcta
ttattatttcctacagcttacttagcagtaggtggatggtttacagctaccacttttgta
acatctttctttacacatggtttggccacatcttatctagaagcttgtaactttttaaca
gctgcagtttcaactccagccaactctatggcccattcattactactactctggggtcca
gaagcacatcttggatttactgcctggctttgtataggaggtttatggacatttattgct
tttcatggtcttattgggcttatagcattttccctacgtcagtttgaaatagcaagatta
gttttaattaggccttataatgcacttgcattctcagggccaatagcagtttatacatct
gtcttcctaatttatccccttggtcaatctagctggttctttgcaccaagctttgggatt
gctgctatcttccgattcctattattccttcaaggttttcataattggacattaaatcct
tttcacatgatgggtgtagctggaatattgggaggtgccctactatcagctatccatggt
gctactgtcattaacactatttatcaagatgctcgtgcatatacaacatttcgtgctttc
tcaccaagtcaaccagaagaaacttattcaatgcttacagctaatcgcttctggtctcag
atatttggtgtagctttctcaaacaagagatggcttcatttcttcatgttattcgtacct
gtagcaggtatgtggacatcttcaattggtatccttggtttagcatttaatctaagagca
tatgattttatttcccaagaattaaaagcagcagaagatcctgaatttgaaacattttat
acaaagaacattctactcaatgaaggtatacgactttggatggctgtccaagatcaagct
catgaaaattatcagttcccagaagaggtattgccaagaggtaattctttgtga

>Irm5_psbD
atgaaactcatctattctacaagattcatggcaagtaaagcttatcttcttggatcactt
actctactagatgactggttaaaacgagatcgatttgtctttattggctggtcaggtcta
ttattatttcctacagcttacttagcagcaggtggatggtttacagccaccgcttttgta
acatctttctttacacatggtctggtcacatcttatctagaagcttgtaatttttaaca
gctgcagtttcaactccagccaactctatggcccattcattactactactctggggacca
gaagcacatcttggatttactgcctgggtttgtataggaggcttatggacatttattgct
tttcatgggctcattggtctcatagcattttccctacgtcagtttgaaatagcacgatta
gttttaattagaccttataatgcacttgcattctcaggaccaatagcagtttatacatct
gtcttcctaatttatccacttggtcaatctagctggttctttgcaccaagctttggtatt
gctgctatcttccgattcctattattccttcaaggttttcataattggacattaaatcct
tttcacatgatgggtgtagctggaatattgggaggtgccctactatcagctatccatggt
gctactgtcattaacactatttatcaagatgctcgtgcatatacaacatttcgtgctttc
tcaccaagtcaaccagaagaaacttattcaatgcttacagctaatcgtttttggtctcag
atatttggtgtagccttctcaaacaagagatggcttcatttcttcatgctatttgtacct
ctagcaggtatgtggacatcttcaattggtatcattggattagcatttaatctaagagct
tatgattttatttctcaagaattgaaagcagcagaagatcctgaatttgaaacattttat
acaaagaacattctactcaatgaaggtatacgactttggatggctgtccaagatcaagct
catgaaaattatcagttcccagaagaggtattgccaagaggtaattctttgtga
>Irm7_psbD
atgaaactcatctattctacaagattcatggcaagtaaagcttatcttcttggatcactt
actctactagatgactggttaaaacgagatcgatttgtctttattggctggtcaggtcta
ttattatttcctacagcttacttagcagtaggtggatggtttacagccaccgcttttgta
acatctttctttacacatggtctggtcacatcttatctagaagcttgtaatttttaaca
gctgcagtttcaactccagccaactctatggcccattcattactactactctggggacca
gaagcacatcttggatttactgcctgggtttgtataggaggcttatggacatttattgct
tttcatgggctcattggtctcatagcattttccctacgtcagtttgaaatagcacgatta
gttttaattagaccttataatgcacttgcattctcaggaccaataggagtttatacatct
gtcttcctaatttatccacttggtcaatctagctggttctttgcaccaagctttgggatt
gctgctatcttccgattcctattattccttcaaggttttcataattggacattaaatcct
tttcacatgatgggtgtagctggaatattgggaggtgccctactatcagctatccatggt
gctactgtcattaacactatttatcaagatgctcgtgcatatacaacatttcgtgctttc
tcacctagtcaaccagaagaaacttattcaatgcttacagctaatcgtttttggtctcag
atatttggtgtagccttctcaaacaagagatggcttcatttcttcatgctatttgtacct
ctagcaggtatgtggacatcttcaattggtatcattggattagcatttaatctaagagct
tatgattttatttctcaagaattgaaagcagcagaagatcctgaatttgaaacattttat
acaaagaacattctactcaatgaaggtatacgactttggatggctgtccaagatcaagct
catgaaaattatcagttcccagaagaggtattgccaagaggtaattctttgtga
>Irm9_psbD
atgaaactcatctattctacaagattcatgtcaagtaaatcttatcttcttggatcactt
actctactagatgactggttaaaacgagatcgatttgtctttattggctggtcaggtcta
ttattatttcctacagcttacttagcagtaggtggatggtttacagctaccacttttgta
acatctttctttacacatggtttggccacatcttatctagaagcttgtaactttttaaca
gctgcagtttcaactccagccaactctatggcccattcattactactactctggggtcca
gaagcacatcttggatttactgcctggctttgtataggaggtttatggacatttattgct
tttcatggtcttattgggcttatagcattttcactacgtcagtttgaaatagcacgatta
gttttaattagaccttataatgcacttgcattctcaggaccaataggagtttatacatct
gtcttcctaatttatccacttggtcaatctagctggttctttgcaccaagctttgggatt
gctgctatcttccgattcctattattccttcaaggttttcataattggacattaaatcct
tttcacatgatgggtgtagctggaatattgggaggtgcattactatcagctatccatggt
gctactgtcattaacactatttatcaagatgctcgtgcatatacaacatttcgtgctttc
tcacctagtcaaccagaagaaacttattcaatgcttacagctaatcgcttctggtctcaa
atatttggtgtagccttctcaaacaagagatggcttcatttcttcatgctatttgtacct
ctagcaggtatgtggacatcttcaattggtatcattggattagcatttaatctaagagca
tatgattttatttcccaagaattaaaagcagcagaagatcctgaatttgaaacattttat
acaaagaacattctactcaatgaaggaataagactttggatggctgtccaagatcaagct
catgaaaattatcagttcccagaagaggtattgccaagaggtaattctttgtga

>Isy12_psbD
atgaaactcatctattctacaagattcatggcaagtaaagcttatcttcttggatcactt
actctattagatgactggttaaaacgagatcgatttgtctttattggctggtcaggtcta
ttattatttcctacagcttacttagcagcaggtggatggtttacagccaccacttttgta
acatctttctttacacatggtctggtcacatcttatctagaagcttgtaatttttaaca
gctgcagtttcaactccagccaactctatggcccattcattactactactctggggacca
gaagcacatcttggatttactgcctgggtttgtataggaggcttatggacatttattgct
tttcatgggctcattggtctcataggattttccctacgtcagtttgaaatagcacgatta
gttttaattagaccttataatgcacttgcattctcaggaccaatagcagtttatacatct
gtcttcctaatttatccacttggtcaatctagctggttctttgcaccaagctttgggatt
gctgctatcttccgattcctattattccttcaaggttttcataattggacattaaatcct
tttcacatgatgggtgtagctggaatattgggaggtgcattactatcagctatccatggt
gctactgtcattaacactatttatcaagatgctcgtgcatatacaacatttcgtgctttc
tcacctagtcaaccagaagaaacttattcaatgcttacagctaatcgtttttggtctcag
atatttggtgtagccttctcaaacaagagatggcttcatttcttcatgctatttgtacct
ctagcaggtatgtggacatcttcaattggtatcattggattagcatttaatctaagagct
tatgattttatttctcaagaattgaaagcagcagaagatcctgaatttgaaacattttat
acaaagaacattctactcaatgaaggtatacgactttggatggctgtccaagatcaagct
catgaaaattatcagttcccagaagaggtattgccaagaggtaattctttgtga
>Isy15_psbD
atgaaactcatctattctacaagattcatggcaagtaaagcttatcttcttggatcactt
actctactagatgactggttaaaacgagatcgctttgtctttattgggtggtcaggtctc
ttattgtttcctacagcttatttagcagcaggaggatggttcacagccacaacttttgta
acatctttctttacacatggcttagtgacatcttatcttgaagcttgtaatttttaaca
gctgcagcttcaactccagctaactctatggcccattcattactactactctggggacca
gaagcacatcttggatttactgcctggctttgtataggaggtttatggacatttattgct
tttcatggtcttattgggcttatagcattttcactacgtcagtttgaaatagcaagatta
gttttaattaggccttataatgcacttgcattctcagggccaatagcagtttatacatct
gtcttcctaatttatccccttggacaatctagttggttctttgcacctagctttggtatt
gctgctatattccgattcctattattccttcaaggttttcataattggacattaaatcct
tttcacatgatgggtgtagctggaatattaggaggtgctttactatcagctatccatggt
gctactgttattaatactatttatcaagatgctggtgcatactccacattccgtgctttc
tcaccaaatcaaccagaagaaacttattcaatgcttacagctaatcgtttttggtctcag
gtatttggtgtagctttctcaaacaagagatggcttcatttcttcatgttattcgtacct
gtagcaggtatgtggacatcttcaattggtatccttggtttagcatttaatctaagagca
tatgattttatttcccaagaattaaaagcagcagaagatcctgaatttgaaacattttat
acaaagaacattctactcaatgaaggaataagactttggatggcagtacaagatcaacct
catgaaaattatcagttcccagaagaggtattgccaagaggtaattctttgtga
>Isy16_psbD
atgaaactcatctattctacaagattcatggcaagtaaagcttatcttcttggatcactt
actctactagatgactggttaaaacgagatcgatttgtctttattggctggtcaggtcta
ttattatttcctacagcttacttagcagcaggtggatggtttacagccaccgcttttgta
acatctttctttacacatggtctggtcacatcttatctagaagcttgtaatttttaaca
gctgcagtttcaactccagccaactctatggcccattcattactactactctggggacca
gaagcacatcttggatttactgcctgggtttgtataggaggcttatggacatttattgct
tttcatgggctcattggtctcatagcattttccctacgtcagtttgaaatagcacgatta
gttttaattagaccttataatgcacttgcattctcaggaccaatagcagtttatacatct
gtcttcctaatttatccacttggtcaatctagctggttctttgcaccaagctttggtatt
gctgctatcttccgattcctattattccttcaaggttttcataattggacattaaatcct
tttcacatgatgggtgtagctggaatattgggaggtgccctactatcagctatccatggt
gctactgtcattaacactatttatcaagatgctcgtgcatatacaacatttcgtgctttc
tcaccaagtcaaccagaagaaacttattcaatgcttacagctaatcgtttttggtctcag
atatttggtgtagccttctcaaacaagagatggcttcatttcttcatgctatttgtacct
ctagcaggtatgtggacatcttcaattggtatcattggattagcatttaatctaagagct
tatgattttatttctcaagaattgaaagcagcagaagatcctgaatttgaaacattttat
acaaagaacattctactcaatgaaggtatacgactttggatggctgtccaagatcaagct
catgaaaattatcagttcccagaagaggtattgccaagaggtaattctttgtga

>Isy17_psbD
atgaaactcatctattctacaagattcatggcaagtaaagcttatcttcttggatcactt
actctactagatgactggttaaaacgagatcgatttgtctttattggctggtcaggtcta
ttattatttcctacagcttacttagcagcaggtggatggtttacagccaccgcttttgta
acatctttctttacacatggtctggtcacatcttatctagaagcttgtaattttttaaca
gctgcagtttcaactccagccaactctatggcccattcattactactactctggggacca
gaagcacatcttggatttactgcctgggtttgtataggaggcttatggacatttattgct
tttcatgggctcattggtctcatagcattttccctacgtcagtttgaaatagcacgatta
gttttaattagaccttataatgcacttgcattctcaggaccaataggagtttatacatct
gtcttcctaatttatccacttggtcaatctagctggttctttgcaccaagctttggtatt
gctgctatcttccgattcctattattccttcaaggttttcataattggacattaaatcct
tttcacatgatgggtgtagctggaatattgggaggtgccctactatcagctatccatggt
gctactgtcattaacactatttatcaagatgctcgtgcatatacaacatttcgtgctttc
tcaccaagtcaaccagaagaaacttattcaatgcttacagctaatcgtttttggtctcag
atatttggtgtagccttctcaaacaagagatggcttcatttcttcatgctatttgtacct
ctagcaggtatgtggacatcttcaattggtatcattggattagcatttaatctaagagct
tatgattttatttctcaagaattgaaagcagcagaagatcctgaatttgaaacattttat
acaaagaacattctactcaatgaaggtatacgactttggatggctgtccaagatcaagct
catgaaaattatcagttcccagaagaggtattgccaagaggtaattctttgtga
>Isy18_psbD
atgaaactcatctattctacaagattcatgtcaagtaaagcttatcttcttggatcactt
actctactagatgactggttaaaacgagatcgatttgtctttattggctggtcaggtcta
ttattatttcctacagcttacttagcagcaggtggatggtttacagccaccgcttttgta
acatctttctttacacatggtctggtcacatcttatctagaagcttgtaattttttaaca
gctgcagtttcaactccagccaactctatggcccattcattactactactctggggacca
gaagcacatcttggatttactgcctgggtttgtataggaggcttatggacatttattgct
tttcatgggctcattggtctcatagcattttccctacgtcagtttgaaatagcacgatta
gttttaattagaccttataatgcacttgcattctcaggaccaataggagtttatacatct
gtcttcctaatttatccacttggtcaatctagctggttctttgcaccaagctttggtatt
gctgctatcttccgattcctattattccttcaaggttttcataattggacattaaatcct
tttcacatgatgggtgtagctggaatattgggaggtgccctactatcagctatccatggt
gctactgtcattaacactatttatcaagatgctcgtgcatatacaacatttcgtgctttc
tcaccaagtcaaccagaagaaacttattcaatgcttacagctaatcgtttttggtctcag
atatttggtgtagccttctcaaacaagagatggcttcatttcttcatgctatttgtacct
ctagcaggtatgtggacatcttcaattggtatcattggattagcatttaatctaagagct
tatgattttatttctcaagaattgaaagcagcagaagatcctgaatttgaaacattttat
acaaagaacattctactcaatgaaggtatacgactttggatggctgtccaagatcaagct
catgaaaattatcagttcccagaagaggtattgccaagaggtaattctttgtga
>Isy21_psbD
atgaaactcatctattctacaagattcatggcaagtaaagcttatcttcttggatcactt
actctactagatgactggttaaaacgagatcgatttgtctttattggctggtcaggtcta
ttattatttcctacagcttacttagcagcaggtggatggtttacagccaccacttttgta
acatctttctttacacatggtctggtcacatcttatctagaagcttgtaattttttaaca
gctgcagtttcaactccagccaactctatggcccattcattactactactctggggacca
gaagcacatcttggatttactgcctgggtttgtataggaggcttatggacatttattgct
tttcatgggctcattggtctcatagcattttccctacgtcagtttgaaatagcacgatta
gttttaattagaccttataatgcacttgcattctcaggaccaatagcagtttatacatct
gtcttcctaatttatccacttggtcaatctagctggttctttgcaccaagctttgggatt
gctgctatcttccgattcctattattccttcaaggttttcataattggacattaaatcct
tttcacatgatgggtgtagctggaatattgggaggtgccctactatcagctatccatggt
gctactgtcattaacactatttatcaagatgctcgtgcatatacaacatttcgtgctttc
tcaccaagtcaaccagaagaaacttattcaatgcttacagctaatcgtttttggtctcag
atatttggtgtagccttctcaaacaagagatggcttcatttcttcatgctatttgtacct
ctagcaggtatgtggacatcttcaattggtatcattggattagcatttaatctaagagct
tatgattttatttctcaagaattgaaagcagcagaagatcctgaatttgaaacattttat
acaaagaacattctactcaatgaaggtatacgactttggatggctgtccaagatcaagct
catgaaaattatcagttcccagaagaggtattgccaagaggtaattctttgtga

>Isy22_psbD
atgaaactcatctattctacaagattcatggcaagtaaagcttatcttcttggatcactt
actctactagatgactggttaaaacgagatcgatttgtctttattggctggtcaggtcta
ttattatttcctacagcttacttagcagtaggtggatggtttacagccaccgcttttgta
acatctttctttacacatggtctggtcacatcttatctagaagcttgtaatttttaaca
gctgcagtttcaactccagccaactctatggcccattcattactactactctggggacca
gaagcacatcttggatttactgcctgggtttgtataggaggcttatggacatttattgct
tttcatgggctcattggtctcatagcattttccctacgtcagtttgaaatagcacgatta
gttttaattagaccttataatgcacttgcattctcaggaccaatagcagtttatacatct
gtcttcctaatttatccacttggtcaatctagctggttctttgcaccaagctttggtatt
gctgctatcttccgattcctattattccttcaaggttttcataattggacattaaatcct
tttcacatgatgggtgtagctggaatattgggaggtgccctactatcagctatccatggt
gctactgtcattaacactatttatcaagatgctcgtgcatatacaacatttcgtgctttc
tcaccaagtcaaccagaagaaacttattcaatgcttacagctaatcgtttttggtctcag
atatttggtgtagccttctcaaacaagagatggcttcatttcttcatgctatttgtacct
ctagcaggtatgtggacatcttcaattggtatcattggattagcatttaatctaagagct
tatgattttatttctcaagaattgaaagcagcagaagatcctgaatttgaaacattttat
acaaagaacattctactcaatgaaggtatacgactttggatggctgtccaagatcaagct
catgaaaattatcagttcccagaagaggtattgccaagaggtaattctttgtga
>Isy23_psbD
atgaaactcatctattctacaagattcatggcaagtaaagcttatcttcttggatcactt
actctactagatgactggttaaaacgagatcgatttgtctttattggctggtcaggtcta
ttattatttcctacagcttacttagcagcaggtggatggtttacagccaccacttttgta
acatctttctttacacatggtctggtcacatcttatctagaagcttgtaatttttaaca
gctgcagtttcaactccagccaactctatggcccattcattactactactctggggacca
gaagcacatcttggatttactgcctgggtttgtataggaggcttatggacatttattgct
tttcatgggctcattggtctcatagcattttccctacgtcagtttgaaatagcacgatta
gttttaattagaccttataatgcacttgcattctcaggaccaatagcagtttatacatct
gtcttcctaatttatccacttggtcaatctagctggttctttgcaccaagctttgggatt
gctgctatcttccgattcctattattccttcaaggttttcataattggacattaaatcct
tttcacatgatgggtgtagctggaatattgggaggtgccctactatcagctatccatggt
gctactgtcattaacactatttatcaagatgctcgtgcatatacaacatttcgtgctttc
tcaccaagtcaaccagaagaaacttattcaatgcttacagctaatcgtttttggtctcag
atatttggtgtagccttctcaaacaagagatggcttcatttcttcatgctatttgtacct
ctagcaggtatgtggacatcttcaattggtatcattggattagcatttaatctaagagct
tatgattttatttctcaagaattgaaagcagcagaagatcctgaatttgaaacattttat
acaaagaacattctactcaatgaaggtatacgactttggatggctgtccaagatcaagct
catgaaaattatcagttcccagaagaggtattgccaagaggtaattctttgtga
>Isy24_psbD
atgaaactcatctattctacaagattcatggcaagtaaagcttatcttcttggatcactt
actctactagatgactggttaaaacgagatcgatttgtctttattggctggtcaggtcta
ttattatttcctacagcttacttagcagcaggtggatggtttacagccaccgcttttgta
acatctttctttacacatggtctggtcacatcttatctagaagcttgtaatttttaaca
gctgcagtttcaactccagccaactctatggcccattcattactactactctggggacca
gaagcacatcttggatttactgcctgggtttgtataggaggcttatggacatttattgct
tttcatgggctcattggtctcatagcattttccctacgtcagtttgaaatagcacgatta
gttttaattagaccttataatgcacttgcattctcaggaccaatagcagtttatacatct
gtcttcctaatttatccacttggtcaatctagctggttctttgcaccaagctttggtatt
gctgctatcttccgattcctattattccttcaaggttttcataattggacattaaatcct
tttcacatgatgggtgtagctggaatattgggaggtgccctactatcagctatccatggt
gctactgtcattaacactatttatcaagatgctcgtgcatatacaacatttcgtgctttc
tcaccaagtcaaccagaagaaacttattcaatgcttacagctaatcgcttctggtctcaa
atatttggtgtagccttctcaaacaagagatggcttcatttcttcatgctatttgtacct
ctagcaggtatgtggacatcttcaattggtatcattggattagcatttaatctaagagct
tatgattttatttctcaagaattgaaagcagcagaagatcctgaatttgaaacattttat
acaaagaacattctactcaatgaaggtatacgactttggatggctgtccaagatcaagct
catgaaaattatcagttcccagaagaggtattgccaagaggtaattctttgtga

>Isy25_psbD
atgaaactcatctattctacaagattcatggcaagtaaagcttatcttcttggatcactt
actctactagatgactggttaaaacgagatcgatttgtctttattggctggtcaggtcta
ttattatttcctacagcttacttagcagcaggtggatggtttacagccaccacttttgta
acatctttctttacacatggtctggtcacatcttatctagaagcttgtaattttttaaca
gctgcagtttcaactccagccaactctatggcccattcattactactactctggggacca
gaagcacatcttggatttactgcctgggtttgtataggaggcttatggacatttattgct
tttcatgggctcattggtctcatagcattttccctacgtcagtttgaaatagcacgatta
gttttaattagaccttataatgcacttgcattctcaggaccaatagcagtttatacatct
gtcttcctaatttatccacttggtcaatctagctggttctttgcaccaagctttgggatt
gctgctatcttccgattcctattattccttcaaggttttcataattggacattaaatcct
tttcacatgatgggtgtagctggaatattgggaggtgccctactatcagctatccatggt
gctactgtcattaacactatttatcaagatgctcgtgcatatacaacatttcgtgctttc
tcaccaagtcaaccagaagaaacttattcaatgcttacagctaatcgtttttggtctcag
atatttggtgtagccttctcaaacaagagatggcttcatttcttcatgctatttgtacct
ctagcaggtatgtggacatcttcaattggtatcattggattagcatttaatctaagagct
tatgattttatttctcaagaattgaaagcagcagaagatcctgaatttgaaacattttat
acaaagaacattctactcaatgaaggtatacgactttggatggctgtccaagatcaagct
catgaaaattatcagttcccagaagaggtattgccaagaggtaattctttgtga
>Isy26_psbD
atgaaactcatctattctacaagattcatggcaagtaaagcttatcttcttggatcactt
actctactagatgactggttaaaacgagatcgatttgtctttattggctggtcaggtcta
ttattatttcctacagcttacttagcagcaggtggatggtttacagccaccacttttgta
acatctttctttacacatggtctggtcacatcttatctagaagcttgtaattttttaaca
gctgcagtttcaactccagccaactctatggcccattcattactactactctggggacca
gaagcacatcttggatttactgcctgggtttgtataggaggcttatggacatttattgct
tttcatgggctcattggtctcatagcattttccctacgtcagtttgaaatagcacgatta
gttttaattagaccttataatgcacttgcattctcaggaccaatagcagtttatacatct
gtcttcctaatttatccacttggtcaatctagctggttctttgcaccaagctttgggatt
gctgctatcttccgattcctattattccttcaaggttttcataattggacattaaatcct
tttcacatgatgggtgtagctggaatattgggaggtgccctactatcagctatccatggt
gctactgtcattaacactatttatcaagatgctcgtgcatatacaacatttcgtgctttc
tcaccaagtcaaccagaagaaacttattcaatgcttacagctaatcgtttttggtctcag
atatttggtgtagccttctcaaacaagagatggcttcatttcttcatgctatttgtacct
ctagcaggtatgtggacatcttcaattggtatcattggattagcatttaatctaagagct
tatgattttatttctcaagaattgaaagcagcagaagatcctgaatttgaaacattttat
acaaagaacattctactcaatgaaggtatacgactttggatggctgtccaagatcaagct
catgaaaattatcagttcccagaagaggtattgccaagaggtaattctttgtga
>Isy27_psbD
atgaaactcatctattctacaagattcatggcaagtaaagcttatcttcttggatcactt
actctactagatgactggttaaaacgagatcgatttgtctttattggctggtcaggtcta
ttattatttcctacagcttacttagcagcaggtggatggtttacagccaccacttttgta
acatctttctttacacatggtctggtcacatcttatctagaagcttgtaattttttaaca
gctgcagtttcaactccagccaactctatggcccattcattactactactctggggacca
gaagcacatcttggatttactgcctgggtttgtataggaggcttatggacatttattgct
tttcatgggctcattggtctcatagcattttccctacgtcagtttgaaatagcacgatta
gttttaattagaccttataatgcacttgcattctcaggaccaatagcagtttatacatct
gtcttcctaatttatccacttggtcaatctagctggttctttgcaccaagctttgggatt
gctgctatcttccgattcctattattccttcaaggttttcataattggacattaaatcct
tttcacatgatgggtgtagctggaatattgggaggtgccctactatcagctatccatggt
gctactgtcattaacactatttatcaagatgctcgtgcatatacaacatttcgtgctttc
tcaccaagtcaaccagaagaaacttattcaatgcttacagctaatcgtttttggtctcag
atatttggtgtagccttctcaaacaagagatggcttcatttcttcatgctatttgtacct
ctagcaggtatgtggacatcttcaattggtatcattggattagcatttaatctaagagct
tatgattttatttctcaagaattgaaagcagcagaagatcctgaatttgaaacattttat
acaaagaacattctactcaatgaaggtatacgactttggatggctgtccaagatcaagct
catgaaaattatcagttcccagaagaggtattgccaagaggtaattctttgtga

>Isy4_psbD
atgaaactcatctattctacaagattcatggcaagtaaagcttatcttcttggatcactt
actctactagatgactggttaaaacgagatcgatttgtctttattggctggtcaggtcta
ttattatttcctacagcttacttagcagcaggtggatggtttacagccaccgcttttgta
acatctttctttacacatggtctggtcacatcttatctagaagcttgtaattttttaaca
gctgcagtttcaactccagccaactctatggcccattcattactactactctggggacca
gaagcacatcttggatttactgcctgggtttgtataggaggcttatggacatttattgct
tttcatgggctcattggtctcatagcattttccctacgtcagtttgaaatagcacgatta
gttttaattagaccttataatgcacttgcattctcaggaccaatagcagtttatacatct
gtcttcctaatttatccacttggtcaatctagctggttctttgcaccaagctttggtatt
gctgctatcttccgattcctattattccttcaaggttttcataattggacattaaatcct
tttcacatgatgggtgtagctggaatattgggaggtgccctactatcagctatccatggt
gctactgtcattaacactatttatcaagatgctcgtgcatatacaacatttcgtgctttc
tcaccaagtcaaccagaagaaacttattcaatgcttacagctaatcgtttttggtctcag
atatttggtgtagccttctcaaacaagagatggcttcatttcttcatgctatttgtacct
ctagcaggtatgtggacatcttcaattggtatcattggattagcatttaatctaagagct
tatgattttatttctcaagaattgaaagcagcagaagatcctgaatttgaaacattttat
acaaagaacattctactcaatgaaggtatacgactttggatggctgtccaagatcaagct
catgaaaattatcagttcccagaagaggtattgccaagaggtaattctttgtga
>Isy7_psbD
atgaaactcatctattctacaagattcatggcaagtaaagcttatcttcttggatcactt
actctactagatgactggttaaaacgagatcgatttgtctttattggctggtcaggtctc
ttattgtttcctacagcttatttagcagcaggtggatggtttacagccaccgcttttgta
acatctttctttacacatggtctggtcacatcttatctagaagcttgtaattttttaaca
gctgcagtttcaactccagccaactctatggcccattcattactactactctggggacca
gaagcacatcttggatttactgcctgggtttgtataggaggcttatggacatttattgct
tttcatgggctcattggtctcatagcattttccctacgtcagtttgaaatagcacgatta
gttttaattagaccttataatgcacttgcattctcaggaccaatagcagtttatacatct
gtcttcctaatttatccacttggtcaatctagctggttctttgcaccaagctttggtatt
gctgctatcttccgattcctattattccttcaaggttttcataattggacattaaatcct
tttcacatgatgggtgtagctggaatattgggaggtgccctactatcagctatccatggt
gctactgtcattaacactatttatcaagatgctcgtgcatatacaacatttcgtgctttc
tcaccaaatcaaccagaagaaacttattcaatgcttacagctaatcgtttttggtctcag
gtatttggtgtagctttctcaaacaagagatggcttcatttcttcatgctatttgtacct
ctagcaggtatgtggacatcttcaattggtatcattggattagcatttaatctaagagct
tatgattttatttctcaagaattgaaagcagcagaagatcctgaatttgaaacattttat
acaaagaacattctactcaatgaaggtatacgactttggatggctgtccaagatcaagct
catgaaaattatcagttcccagaagaggtattgccaagaggtaattctttgtga
>Isy8_psbD
atgaaactcatctattctacaagattcatggcaagtaaagcttatcttcttggatcactt
actctactagatgactggttaaaacgagatcgatttgtctttattggctggtcaggtcta
ttattatttcctacagcttacttagcagcaggtggatggtttacagccaccacttttgta
acatctttctttacacatggtctggtcacatcttatctagaagcttgtaattttttaaca
gctgcagtttcaactccagccaactctatggcccattcattactactactctggggacca
gaagcacatcttggatttactgcctgggtttgtataggaggcttatggacatttattgct
tttcatgggctcattggtctcatagcattttccctacgtcagtttgaaatagcacgatta
gttttaattagaccttataatgcacttgcattctcaggaccaatagcagtttatacatct
gtcttcctaatttatccacttggtcaatctagctggttctttgcaccaagctttgggatt
gctgctatcttccgattcctattattccttcaaggttttcataattggacattaaatcct
tttcacatgatgggtgtagctggaatattgggaggtgccctactatcagctatccatggt
gctactgtcattaacactatttatcaagatgctcgtgcatatacaacatttcgtgctttc
tcaccaagtcaaccagaagaaacttattcaatgcttacagctaatcgtttttggtctcag
atatttggtgtagccttctcaaacaagagatggcttcatttcttcatgctatttgtacct
ctagcaggtatgtggacatcttcaattggtatcattggattagcatttaatctaagagct
tatgattttatttctcaagaattgaaagcagcagaagatcctgaatttgaaacattttat
acaaagaacattctactcaatgaaggtatacgactttggatggctgtccaagatcaagct
catgaaaattatcagttcccagaagaggtattgccaagaggtaattctttgtga

>KrA1_psbD
atgaaactcatctattctacaagattcatggcaagtaaagcttatcttcttggatcactt
actctactagatgactggttaaaacgagatcgatttgtctttattggctggtcaggtcta
ttattatttcctacagcttacttagcagcaggtggatggtttacagccaccgcttttgta
acatctttctttacacatggtctggtcacatcttatctagaagcttgtaattttttaaca
gctgcagtttcaactccagccaactctatggcccattcattactactactctggggacca
gaagcacatcttggatttactgcctgggtttgtataggaggcttatggacatttattgct
tttcatgggctcattggtctcatagcattttccctacgtcagtttgaaatagcacgatta
gttttaattagaccttataatgcacttgcattctcaggaccaatagcagtttatacatct
gtcttcctaatttatccacttggtcaatctagctggttctttgcaccaagctttggtatt
gctgctatcttccgattcctattattccttcaaggttttcataattggacattaaatcct
tttcacatgatgggtgtagctggaatattgggaggtgccctactatcagctatccatggt
gctactgtcattaacactatttatcaagatgctcgtgcatatacaacatttcgtgctttc
tcaccaagtcaaccagaagaaacttattcaatgcttacagctaatcgtttttggtctcag
atatttggtgtagccttctcaaacaagagatggcttcatttcttcatgctatttgtacct
ctagcaggtatgtggacatcttcaattggtatcattggattagcatttaatctaagagct
tatgattttatttctcaagaattgaaagcagcagaagatcctgaatttgaaacattttat
acaaagaacattctactcaatgaaggtatacgactttggatggctgtccaagatcaagct
catgaaaattatcagttcccagaagaggtattgccaagaggtaattctttgtga
>KrA10_psbD
atgaaactcatctattctacaagattcatggcaagtaaagcttatcttcttggatcactt
actctactagatgactggttaaaacgagatcgatttgtctttattggctggtcaggtcta
ttattatttcctacagcttacttagcagcaggtggatggtttacagccaccgcttttgta
acatctttctttacacatggtctggtcacatcttatctagaagcttgtaattttttaaca
gctgcagtttcaactccagccaactctatggcccattcattactactactctggggacca
gaagcacatcttggatttactgcctgggtttgtataggaggcttatggacatttattgct
tttcatgggctcattggtctcatagcattttccctacgtcactttgaaatagcacgatta
gttttaattagaccttataatgcacttgcattctcaggaccaataggagtttatacatct
gtcttcctaatttatccacttggtcaatctagctggttctttgcaccaagctttggtatt
gctgctatcttccgattcctattattccttcaaggttttcataattggacattaaatcct
tttcacatgatgggtgtagctggaatattgggaggtgccctactatcagctatccatggt
gctactgtcattaacactatttatcaagatgctcgtgcatatacaacatttcgtgctttc
tcaccaagtcaaccagaagaaacttattcaatgcttacagctaatcgtttttggtctcag
atatttggtgtagccttctcaaacaagagatggcttcatttcttcatgctatttgtacct
ctagcaggtatgtggacatcttcaattggtatcattggattagcatttaatctaagagct
tatgattttatttctcaagaattgaaagcagcagaagatcctgaatttgaaacattttat
acaaagaacattctactcaatgaaggtatacgactttggatggctgtccaagatcaagct
catgaaaattatcagttcccagaagaggtattgccaagaggtaattctttgtga
>KrA11_psbD
atgaaactcatctattctacaagattcatggcaagtaaagcttatcttcttggatcactt
actctactagatgactggttaaaacgagatcgatttgtctttattggctggtcaggtcta
ttattatttcctacagcttacttagcagcaggtggatggtttacagccaccgcttttgta
acatctttctttacacatggtctggtcacatcttatctagaagcttgtaattttttaaca
gctgcagtttcaactccagccaactctatggcccattcattactactactctggggacca
gaagcacatcttggatttactgcctgggtttgtataggaggcttatggacatttattgct
tttcatgggctcattggtctcatagcattttccctacgtcagtttgaaatagcacgatta
gttttaattagaccttataatgcacttgcattctcaggaccaatagcagtttatacatct
gtcttcctaatttatccacttggtcaatctagctggttctttgcaccaagctttggtatt
gctgctatcttccgattcctattattccttcaaggttttcataattggacattaaatcct
tttcacatgatgggtgtagctggaatattgggaggtgccctactatcagctatccatggt
gctactgtcattaacactatttatcaagatgctcgtgcatatacaacatttcgtgctttc
tcaccaagtcaaccagaagaaacttattcaatgcttacagctaatcgtttttggtctcag
atatttggtgtagccttctcaaacaagagatggcttcatttcttcatgctatttgtacct
ctagcaggtatgtggacatcttcaattggtatcattggattagcatttaatctaagagct
tatgattttatttctcaagaattgaaagcagcagaagatcctgaatttgaaacattttat
acaaagaacattctactcaatgaaggtatacgactttggatggctgtccaagatcaagct
catgaaaattatcagttcccagaagaggtattgccaagaggtaattctttgtga

>KrA12_psbD
atgaaactcatctattctacaagattcatggcaagtaaagcttatcttcttggatcactt
actctactagatgactggttaaaacgagatcgatttgtctttattggctggtcaggtcta
ttattatttcctacagcttacttagcagtaggtggatggtttacagctaccacttttgta
acatctttctttacacatggtttggccacatcttatctagaagcttgtaatttttaaca
gctgcagtttcaactccagccaactctatggcccattcattactactactctggggacca
gaagcacatcttggatttactgcctgggtttgtataggaggcttatggacatttattgct
tttcatgggctcattggtctcatagcattttccctacgtcagtttgaaatagcacgatta
gttttaattagaccttataatgcacttgcattctcaggaccaatagcagtttatacatct
gtcttcctaatttatccacttggtcaatctagctggttctttgcaccaagctttggtatt
gctgctatcttccgattcctattattccttcaaggttttcataattggacattaaatcct
tttcacatgatgggtgtagctggaatattgggaggtgccctactatcagctatccatggt
gctactgtcattaacactatttatcaagatgctcgtgcatatacaacatttcgtgctttc
tcacctagtcaaccagaagaaacttattcaatgcttacagctaatcgcttctggtctcaa
atatttggtgtagccttctcaaacaagagatggcttcatttcttcatgctatttgtacct
ctagcaggtatgtggacatcttcaattggtatcattggattagcatttaatctaagagct
tatgattttatttctcaagaattgaaagcagcagaagatcctgaatttgaaacattttat
acaaagaacattctactcaatgaaggtatacgactttggatggctgtccaagatcaagct
catgaaaattatcagttcccagaagaggtattgccaagaggtaattctttgtga
>KrA13_psbD
atgaaactcatctattctacaagattcatggcaagtaaagcttatcttcttggatcactt
actctactagatgactggttaaaacgagatcgatttgtctttattggctggtcaggtcta
ttattatttcctacagcttacttagcagcaggtggatggtttacagctaccacttttgta
acatctttctttacacatggtttggccacatcttatctagaagcttgtaactttttaaca
gctgcagtttcaactccagccaactctatggcccattcattactactactctggggacca
gaagcacatcttggatttactgcctgggtttgtataggaggcttatggacatttattgct
tttcatgggctcattggtctcatagcattttccctacgtcagtttgaaatagcacgatta
gttttaattagaccttataatgcacttgcattctcaggaccaatagcagtttatacatct
gtcttcctaatttatccacttggtcaatctagctggttctttgcaccaagctttggtatt
gctgctatcttccgattcctattattccttcaaggttttcataattggacattaaatcct
tttcacatgatgggtgtagctggaatattgggaggtgcattactatcagctatccatggt
gctactgtcattaacactatttatcaagatgctcgtgcatatacaacatttcgtgctttc
tcaccaagtcaaccagaagaaacttattcaatgcttacagctaatcgtttttggtctcag
atatttggtgtagccttctcaaacaagagatggcttcatttcttcatgctatttgtacct
ctagcaggtatgtggacatcttcaattggtatcattggattagcatttaatctaagagct
tatgattttatttctcaagaattgaaagcagcagaagatcctgaatttgaaacattttat
acaaagaacattctactcaatgaaggtatacgactttggatggctgtccaagatcaagct
catgaaaattatcagttcccagaagaggtattgccaagaggtaattctttgtga
>KrA14_psbD
atgaaactcatctattctacaagattcatggcaagtaaagcttatcttcttggatcactt
actctactagatgactggttaaaacgagatcgatttgtctttattggctggtcaggtcta
ttattatttcctacagcttacttagcagcaggtggatggtttacagccaccgcttttgta
acatctttctttacacatggtctggtcacatcttatctagaagcttgtaatttttaaca
gctgcagtttcaactccagccaactctatggcccattcattactactactctggggacca
gaagcacatcttggatttactgcctgggtttgtataggaggcttatggacatttattgct
tttcatgggctcattggtctcatagcattttccctacgtcagtttgaaatagcacgatta
gttttaattagaccttataatgcacttgcattctcaggaccaatagcagtttatacatct
gtcttcctaatttatccacttggtcaatctagctggttctttgcaccaagctttggtatt
gctgctatcttccgattcctattattccttcaaggttttcataattggacattaaatcct
tttcacatgatgggtgtagctggaatattgggaggtgccctactatcagctatccatggt
gctactgtcattaacactatttatcaagatgctcgtgcatatacaacatttcgtgctttc
tcaccaagtcaaccagaagaaacttattcaatgcttacagctaatcgtttttggtctcag
atatttggtgtagccttctcaaacaagagatggcttcatttcttcatgctatttgtacct
ctagcaggtatgtggacatcttcaattggtatcattggattagcatttaatctaagagct
tatgattttatttctcaagaattgaaagcagcagaagatcctgaatttgaaacattttat
acaaagaacattctactcaatgaaggtatacgactttggatggctgtccaagatcaagct
catgaaaattatcagttcccagaagaggtattgccaagaggtaattctttgtga

>KrA15_psbD
atgaaactcatctattctacaagattcatgtcaagtaaagcttatcttcttggatcactt
actctactagatgactggttaaaacgagatcgatttgtctttattggctggtcaggtcta
ttattatttcctacagcttacttagcagcaggtggatggtttacagccaccgcttttgta
acatctttctttacacatggtctggtcacatcttatctagaagcttgtaatttttaaca
gctgcagtttcaactccagccaactctatggcccattcattactactactctggggacca
gaagcacatcttggatttactgcctgggtttgtataggaggcttatggacatttattgct
tttcatgggctcattggtctcatagcattttccctacgtcagtttgaaatagcacgatta
gttttaattagaccttataatgcacttgcattctcaggaccaatagcagtttatacatct
gtcttcctaatttatccacttggtcaatctagctggttctttgcaccaagctttggtatt
gctgctatcttccgattcctattattccttcaaggttttcataattggacattaaatcct
tttcacatgatgggtgtagctggaatattgggaggtgccctactatcagctatccatggt
gctactgtcattaacactatttatcaagatgctcgtgcatatacaacatttcgtgctttc
tcaccaagtcaaccagaagaaacttattcaatgcttacagctaatcgtttttggtctcaa
atatttggtgtagccttctcaaacaagagatggcttcatttcttcatgctatttgtacct
ctagcaggtatgtggacatcttcaattggtatcattggattagcatttaatctaagagct
tatgattttatttctcaagaattgaaagcagcagaagatcctgaatttgaaacattttat
acaaagaacattctactcaatgaaggtatacgactttggatggctgtccaagatcaagct
catgaaaattatcagttcccagaagaggtattgccaagaggtaattctttgtga
>KrA2_psbD
atgaaactcatctattctacaagattcatggcaagtaaagcttatcttcttggatcactt
actctactagatgactggttaaaacgagatcgatttgtctttattggctggtcaggtcta
ttattatttcctacagcttacttagcagcaggtggatggtttacagccaccgcttttgta
acatctttctttacacatggtctggtcacatcttatctagaagcttgtaatttttaaca
gctgcagtttcaactccagccaactctatggcccattcattactactactctggggacca
gaagcacatcttggatttactgcctgggtttgtataggaggcttatggacatttattgct
tttcatgggctcattggtctcatagcattttccctacgtcagtttgaaatagcacgatta
gttttaattagaccttataatgcacttgcattctcaggaccaatagcagtttatacatct
gtcttcctaatttatccacttggtcaatctagctggttctttgcaccaagctttggtatt
gctgctatcttccgattcctattattccttcaaggttttcataattggacattaaatcct
tttcacatgatgggtgtagctggaatattgggaggtgccctactatcagctatccatggt
gctactgtcattaacactatttatcaagatgctcgtgcatatacaacatttcgtgctttc
tcaccaagtcaaccagaagaaacttattcaatgcttacagctaatcgtttttggtctcag
atatttggtgtagccttctcaaacaagagatggcttcatttcttcatgctatttgtacct
ctagcaggtatgtggacatcttcaattggtatcattggattagcatttaatctaagagct
tatgattttatttctcaagaattgaaagcagcagaagatcctgaatttgaaacattttat
acaaagaacattctactcaatgaaggtatacgactttggatggctgtccaagatcaagct
catgaaaattatcagttcccagaagaggtattgccaagaggtaattctttgtga
>KrA3_psbD
atgaaactcatctattctacaagattcatgtcaagtaaatcttatcttcttggatcactt
actctactagatgactggttaaaacgagatcgatttgtctttattggctggtcaggtcta
ttattatttcctacagcttacttagcagcaggtggatggtttacagccaccgcttttgta
acatctttctttacacatggtctggtcacatcttatctagaagcttgtaatttttaaca
gctgcagtttcaactccagccaactctatggcccattcattactactactctggggacca
gaagcacatcttggatttactgcctgggtttgtataggaggcttatggacatttattgct
tttcatgggctcattggtctcatagcattttccctacgtcagtttgaaatagcacgatta
gttttaattagaccttataatgcacttgcattctcaggaccaatagcagtttatacatct
gtcttcctaatttatccacttggtcaatctagctggttctttgcaccaagctttggtatt
gctgctatcttccgattcctattattccttcaaggttttcataattggacattaaatcct
tttcacatgatgggtgtagctggaatattgggaggtgccctactatcagctatccatggt
gctactgtcattaacactatttatcaagatgctcgtgcatatacaacatttcgtgctttc
tcaccaagtcaaccagaagaaacttattcaatgcttacagctaatcgtttttggtctcag
atatttggtgtagccttctcaaacaagagatggcttcatttcttcatgctatttgtacct
ctagcaggtatgtggacatcttcaattggtatcattggattagcatttaatctaagagct
tatgattttatttctcaagaattgaaagcagcagaagatcctgaatttgaaacattttat
acaaagaacattctactcaatgaaggtatacgactttggatggctgtccaagatcaagct
catgaaaattatcagttcccagaagaggtattgccaagaggtaattctttgtga

>KrA4_psbD
atgaaactcatctattctacaagattcatggcaagtaaagcttatcttcttggatcactt
actctactagatgactggttaaaacgagatcgatttgtctttattggctggtcaggtcta
ttattatttcctacagcttacttagcagtaggtggatggtttacagctaccacttttgta
acatctttctttacacatggtctggtcacatcttatctagaagcttgtaattttttaaca
gctgcagtttcaactccagccaactctatggcccattcattactactactctggggacca
gaagcacatcttggatttactgcctgggtttgtataggaggcttatggacatttattgct
tttcatgggctcattggtctcatagcattttccctacgtcagtttgaaatagcacgatta
gttttaattagaccttataatgcacttgcattctcaggaccaataggagtttatacatct
gtcttcctaatttatccacttggtcaatctagctggttctttgcaccaagctttggtatt
gctgctatcttccgattcctattattccttcaaggttttcataattggacattaaatcct
tttcacatgatgggtgtagctggaatattgggaggtgccctactatcagctatccatggt
gctactgtcattaacactatttatcaagatgctcgtgcatatacaacatttcgtgctttc
tcaccaagtcaaccagaagaaacttattcaatgcttacagctaatcgtttttggtctcag
atatttggtgtagccttctcaaacaagagatggcttcatttcttcatgctatttgtacct
ctagcaggtatgtggacatcttcaattggtatcattggattagcatttaatctaagagct
tatgattttatttctcaagaattgaaagcagcagaagatcctgaatttgaaacattttat
acaaagaacattctactcaatgaaggtatacgactttggatggctgtccaagatcaagct
catgaaaattatcagttcccagaagaggtattgccaagaggtaattctttgtga
>KrA5_psbD
atgaaactcatctattctacaagattcatggcaagtaaagcttatcttcttggatcactt
actctactagatgactggttaaaacgagatcgatttgtctttattggctggtcaggtcta
ttattatttcctacagcttacttagcagcaggtggatggtttacagccaccgcttttgta
acatctttctttacacatggtctggtcacatcttatctagaagcttgtaattttttaaca
gctgcagtttcaactccagccaactctatggcccattcattactactactctggggacca
gaagcacatcttggatttactgcctgggtttgtataggaggcttatggacatttattgct
tttcatgggctcattggtctcatagcattttccctacgtcagtttgaaatagcacgatta
gttttaattagaccttataatgcacttgcattctcaggaccaatagcagtttatacatct
gtcttcctaatttatccacttggtcaatctagctggttctttgcaccaagctttggtatt
gctgctatcttccgattcctattattccttcaaggttttcataattggacattaaatcct
tttcacatgatgggtgtagctggaatattgggaggtgccctactatcagctatccatggt
gctactgtcattaacactatttatcaagatgctcgtgcatatacaacatttcgtgctttc
tcaccaagtcaaccagaagaaacttattcaatgcttacagctaatcgtttttggtctcag
atatttggtgtagccttctcaaacaagagatggcttcatttcttcatgctatttgtacct
ctagcaggtatgtggacatcttcaattggtatcattggattagcatttaatctaagagct
tatgattttatttctcaagaattgaaagcagcagaagatcctgaatttgaaacattttat
acaaagaacattctactcaatgaaggtatacgactttggatggctgtccaagatcaagct
catgaaaattatcagttcccagaagaggtattgccaagaggtaattctttgtga
>KrA6_psbD
atgaaactcatctattctacaagattcatggcaagtaaagcttatcttcttggatcactt
actctactagatgactggttaaaacgagatcgatttgtctttattggctggtcaggtcta
ttattatttcctacagcttacttagcagcaggtggatggtttacagccaccgcttttgta
acatctttctttacacatggtctggtcacatcttatctagaagcttgtaattttttaaca
gctgcagtttcaactccagccaactctatggcccattcattactactactctggggacca
gaagcacatcttggatttactgcctgggtttgtataggaggcttatggacatttattgct
tttcatgggctcattggtctcatagcattttccctacgtcagtttgaaatagcacgatta
gttttaattagaccttataatgcacttgcattctcaggaccaatagcagtttatacatct
gtcttcctaatttatccacttggtcaatctagctggttctttgcaccaagctttggtatt
gctgctatcttccgattcctattattccttcaaggttttcataattggacattaaatcct
tttcacatgatgggtgtagctggaatattgggaggtgccctactatcagctatccatggt
gctactgtcattaacactatttatcaagatgctcgtgcatatacaacatttcgtgctttc
tcaccaagtcaaccagaagaaacttattcaatgcttacagctaatcgtttttggtctcag
atatttggtgtagccttctcaaacaagagatggcttcatttcttcatgctatttgtacct
ctagcaggtatgtggacatcttcaattggtatcattggattagcatttaatctaagagct
tatgattttatttctcaagaattgaaagcagcagaagatcctgaatttgaaacattttat
acaaagaacattctactcaatgaaggtatacgactttggatggctgtccaagatcaagct
catgaaaattatcagttcccagaagaggtattgccaagaggtaattctttgtga

>KrA7_psbD
atgaaactcatctattctacaagattcatgtcaagtaaatcttatcttcttggatcactt
actctactagatgactggttaaaacgagatcgatttgtctttattggctggtcaggtcta
ttattatttcctacagcttacttagcagcaggtggatggtttacagccaccgcttttgta
acatctttctttacacatggtctggtcacatcttatctagaagcttgtaatttttaaca
gctgcagtttcaactccagccaactctatggcccattcattactactactctggggacca
gaagcacatcttggatttactgcctgggtttgtataggaggcttatggacatttattgct
tttcatgggctcattggtctcatagcattttccctacgtcagtttgaaatagcacgatta
gttttaattagaccttataatgcacttgcattctcaggaccaatagcagtttatacatct
gtcttcctaatttatccacttggtcaatctagctggttctttgcaccaagctttggtatt
gctgctatcttccgattcctattattccttcaaggttttcataattggacattaaatcct
tttcacatgatgggtgtagctggaatattgggaggtgccctactatcagctatccatggt
gctactgtcattaacactatttatcaagatgctcgtgcatatacaacatttcgtgctttc
tcaccaagtcaaccagaagaaacttattcaatgcttacagctaatcgtttttggtctcag
atatttggtgtagccttctcaaacaagagatggcttcatttcttcatgctatttgtacct
ctagcaggtatgtggacatcttcaattggtatcattggattagcatttaatctaagagct
tatgattttatttctcaagaattgaaagcagcagaagatcctgaatttgaaacattttat
acaaagaacattctactcaatgaaggtatacgactttggatggctgtccaagatcaagct
catgaaaattatcagttcccagaagaggtattgccaagaggtaattctttgtga
>KrA8_psbD
atgaaactcatctattctacaagattcatggcaagtaaagcttatcttcttggatcactt
actctactagatgactggttaaaacgagatcgatttgtctttattggctggtcaggtcta
ttattatttcctacagcttacttagcagcaggtggatggtttacagccaccgcttttgta
acatctttctttacacatggtctggtcacatcttatctagaagcttgtaatttttaaca
gctgcagtttcaactccagccaactctatggcccattcattactactactctggggacca
gaagcacatcttggatttactgcctgggtttgtataggaggcttatggacatttattgct
tttcatgggctcattggtctcatagcattttccctacgtcagtttgaaatagcacgatta
gttttaattagaccttataatgcacttgcattctcaggaccaataggagtttatacatct
gtcttcctaatttatccacttggtcaatctagctggttctttgcaccaagctttggtatt
gctgctatcttccgattcctattattccttcaaggttttcataattggacattaaatcct
tttcacatgatgggtgtagctggaatattgggaggtgccctactatcagctatccatggt
gctactgtcattaacactatttatcaagatgctcgtgcatatacaacatttcgtgctttc
tcacctagtcaaccagaagaaacttattcaatgcttacagctaatcgcttctggtctcag
atatttggtgtagccttctcaaacaagagatggcttcatttcttcatgctatttgtacct
ctagcaggtatgtggacatcttcaattggtatcattggattagcatttaatctaagagct
tatgattttatttctcaagaattgaaagcagcagaagatcctgaatttgaaacattttat
acaaagaacattctactcaatgaaggtatacgactttggatggctgtccaagatcaagct
catgaaaattatcagttcccagaagaggtattgccaagaggtaattctttgtga
>KrA9_psbD
atgaaactcatctattctacaagattcatggcaagtaaagcttatcttcttggatcactt
actctactagatgactggttaaaacgagatcgatttgtctttattggctggtcaggtcta
ttattatttcctacagcttacttagcagcaggtggatggtttacagccaccgcttttgta
acatctttctttacacatggtctggccacatcttatctagaagcttgtaactttttaaca
gctgcagtttcaactccagccaactctatggcccattcattactactactctggggacca
gaagcacatcttggatttactgcctgggtttgtataggaggcttatggacatttattgct
tttcatgggctcattggtctcatagcattttccctacgtcagtttgaaatagcacgatta
gttttaattagaccttataatgcacttgcattctcaggaccaatagcagtttatacatct
gtcttcctaatttatccacttggtcaatctagctggttctttgcaccaagctttggtatt
gctgctatcttccgattcctattattccttcaaggttttcataattggacattaaatcct
tttcacatgatgggtgtagctggaatattgggaggtgccctactatcagctatccatggt
gctactgtcattaacactatttatcaagatgctcgtgcatatacaacatttcgtgctttc
tcaccaagtcaaccagaagaaacttattcaatgcttacagctaatcgtttttggtctcag
atatttggtgtagccttctcaaacaagagatggcttcatttcttcatgctatttgtacct
ctagcaggtatgtggacatcttcaattggtatcattggattagcatttaatctaagagct
tatgattttatttctcaagaattgaaagcagcagaagatcctgaatttgaaacattttat
acaaagaacattctactcaatgaaggtatacgactttggatggctgtccaagatcaagct
catgaaaattatcagttcccagaagaggtattgccaagaggtaattctttgtga

>KrC1_psbD
atgaaactcatctattctacaagattcatggcaagtaaagcttatcttcttggatcactt
actctactagatgactggttaaaacgagatcgatttgtctttattggctggtcaggtcta
ttattatttcctacagcttacttagcagcaggtggatggtttacagccaccgcttttgta
acatctttctttacacatggtctggtcacatcttatctagaagcttgtaattttttaaca
gctgcagtttcaactccagccaactctatggcccattcattactactactctggggacca
gaagcacatcttggatttactgcctgggtttgtataggaggcttatggacatttattgct
tttcatgggctcattggtctcatagcattttccctacgtcagtttgaaatagcacgatta
gttttaattagaccttataatgcacttgcattctcaggaccaatagcagtttatacatct
gtcttcctaatttatccacttggtcaatctagctggttctttgcaccaagctttggtatt
gctgctatcttccgattcctattattccttcaaggttttcataattggacattaaatcct
tttcacatgatgggtgtagctggaatattgggaggtgccctactatcagctatccatggt
gctactgtcattaacactatttatcaagatgctcgtgcatatacaacatttcgtgctttc
tcaccaagtcaaccagaagaaacttattcaatgcttacagctaatcgtttttggtctcag
atatttggtgtagccttctcaaacaagagatggcttcatttcttcatgctatttgtacct
ctagcaggtatgtggacatcttcaattggtatcattggattagcatttaatctaagagct
tatgattttatttctcaagaattgaaagcagcagaagatcctgaatttgaaacattttat
acaaagaacattctactcaatgaaggtatacgactttggatggctgtccaagatcaagct
catgaaaattatcagttcccagaagaggtattgccaagaggtaattctttgtga
>KrC10_psbD
atgaaactcatctattctacaagattcatggcaagtaaagcttatcttcttggatcactt
actctactagatgactggttaaaacgagatcgatttgtctttattggctggtcaggtcta
ttattatttcctacagcttacttagcagcaggtggatggtttacagctaccacttttgta
acatctttctttacacatggtctggtcacatcttatctagaagcttgtaattttttaaca
gctgcagtttcaactccagccaactctatggcccattcattactactactctggggacca
gaagcacatcttggatttactgcctgggtttgtataggaggcttatggacatttattgct
tttcatgggctcattggtctcatagcattttccctacgtcagtttgaaatagcacgatta
gttttaattagaccttataatgcacttgcattctcaggaccaatagcagtttatacatct
gtcttcctaatttatccacttggtcaatctagctggttctttgcaccaagctttggtatt
gctgctatcttccgattcctattattccttcaaggttttcataattggacattaaatcct
tttcacatgatgggtgtagctggaatattgggaggtgccctactatcagctatccatggt
gctactgtcattaacactatttatcaagatgctcgtgcatatacaacatttcgtgctttc
tcaccaagtcaaccagaagaaacttattcaatgcttacagctaatcgtttttggtctcag
atatttggtgtagccttctcaaacaagagatggcttcatttcttcatgctatttgtacct
ctagcaggtatgtggacatcttcaattggtatcattggattagcatttaatctaagagct
tatgattttatttctcaagaattgaaagcagcagaagatcctgaatttgaaacattttat
acaaagaacattctactcaatgaaggtatacgactttggatggctgtccaagatcaagct
catgaaaattatcagttcccagaagaggtattgccaagaggtaattctttgtga
>KrC11_psbD
atgaaactcatctattctacaagattcatggcaagtaaagcttatcttcttggatcactt
actctactagatgactggttaaaacgagatcgatttgtctttattggctggtcaggtcta
ttattatttcctacagcttacttagcagcaggtggatggtttacagccaccgcttttgta
acatctttctttacacatggtctggtcacatcttatctagaagcttgtaattttttaaca
gctgcagtttcaactccagccaactctatggcccattcattactactactctggggacca
gaagcacatcttggatttactgcctgggtttgtataggaggcttatggacatttattgct
tttcatgggctcattggtctcatagcattttccctacgtcagtttgaaatagcacgatta
gttttaattagaccttataatgcacttgcattctcaggaccaatagcagtttatacatct
gtcttcctaatttatccacttggtcaatctagctggttctttgcaccaagctttggtatt
gctgctatcttccgattcctattattccttcaaggttttcataattggacattaaatcct
tttcacatgatgggtgtagctggaatattgggaggtgccctactatcagctatccatggt
gctactgtcattaacactatttatcaagatgctcgtgcatatacaacatttcgtgctttc
tcaccaagtcaaccagaagaaacttattcaatgcttacagctaatcgtttttggtctcag
atatttggtgtagccttctcaaacaagagatggcttcatttcttcatgctatttgtacct
ctagcaggtatgtggacatcttcaattggtatcattggattagcatttaatctaagagct
tatgattttatttctcaagaattgaaagcagcagaagatcctgaatttgaaacattttat
acaaagaacattctactcaatgaaggtatacgactttggatggctgtccaagatcaagct
catgaaaattatcagttcccagaagaggtattgccaagaggtaattctttgtga

>KrC12_psbD
atgaaactcatctattctacaagattcatggcaagtaaagcttatcttcttggatcactt
actctactagatgactggttaaaacgagatcgatttgtctttattggctggtcaggtcta
ttattatttcctacagcttacttagcagcaggtggatggtttacagccaccgcttttgta
acatctttctttacacatggtctggtcacatcttatctagaagcttgtaatttttttaaca
gctgcagtttcaactccagccaactctatggcccattcattactactactctggggacca
gaagcacatcttggatttactgcctgggtttgtataggaggcttatggacatttattgct
tttcatgggctcattggtctcatagcattttccctacgtcagtttgaaatagcacgatta
gttttaattagaccttataatgcacttgcattctcaggaccaatagcagtttatacatct
gtcttcctaatttatccacttggtcaatctagctggttctttgcaccaagctttggtatt
gctgctatcttccgattcctattattccttcaaggttttcataattggacattaaatcct
tttcacatgatgggtgtagctggaatattgggaggtgccctactatcagctatccatggt
gctactgtcattaacactatttatcaagatgctcgtgcatatacaacatttcgtgctttc
tcaccaagtcaaccagaagaaacttattcaatgcttacagctaatcgtttttggtctcag
atatttggtgtagccttctcaaacaagagatggcttcatttcttcatgctatttgtacct
ctagcaggtatgtggacatcttcaattggtatcattggattagcatttaatctaagagct
tatgattttatttctcaagaattgaaagcagcagaagatcctgaatttgaaacattttat
acaaagaacattctactcaatgaaggtatacgactttggatggctgtccaagatcaagct
catgaaaattatcagttcccagaagaggtattgccaagaggtaattctttgtga
>KrC13_psbD
atgaaactcatctattctacaagattcatggcaagtaaagcttatcttcttggatcactt
actctactagatgactggttaaaacgagatcgatttgtctttattggctggtcaggtcta
ttattatttcctacagcttacttagcagcaggtggatggtttacagccaccgcttttgta
acatctttctttacacatggtctggtcacatcttatctagaagcttgtaatttttttaaca
gctgcagtttcaactccagccaactctatggcccattcattactactactctggggacca
gaagcacatcttggatttactgcctgggtttgtataggaggcttatggacatttattgct
tttcatgggctcattggtctcatagcattttccctacgtcagtttgaaatagcacgatta
gttttaattagaccttataatgcacttgcattctcaggaccaatagcagtttatacatct
gtcttcctaatttatccacttggtcaatctagctggttctttgcaccaagctttggtatt
gctgctatcttccgattcctattattccttcaaggttttcataattggacattaaatcct
tttcacatgatgggtgtagctggaatattgggaggtgccctactatcagctatccatggt
gctactgtcattaacactatttatcaagatgctcgtgcatatacaacatttcgtgctttc
tcaccaagtcaaccagaagaaacttattcaatgcttacagctaatcgtttttggtctcag
atatttggtgtagccttctcaaacaagagatggcttcatttcttcatgctatttgtacct
ctagcaggtatgtggacatcttcaattggtatcattggattagcatttaatctaagagct
tatgattttatttctcaagaattgaaagcagcagaagatcctgaatttgaaacattttat
acaaagaacattctactcaatgaaggtatacgactttggatggctgtccaagatcaagct
catgaaaattatcagttcccagaagaggtattgccaagaggtaattctttgtga
>KrC14_psbD
atgaaactcatctattctacaagattcatggcaagtaaagcttatcttcttggatcactt
actctactagatgactggttaaaacgagatcgatttgtctttattggctggtcaggtcta
ttattatttcctacagcttacttagcagcaggtggatggtttacagccaccgcttttgta
acatctttctttacacatggtctggtcacatcttatctagaagcttgtaactttttaaca
gctgcagtttcaactccagccaactctatggcccattcattactactactctggggacca
gaagcacatcttggatttactgcctgggtttgtataggaggcttatggacatttattgct
tttcatgggctcattggtctcatagcattttccctacgtcagtttgaaatagcacgatta
gttttaattagaccttataatgcacttgcattctcaggaccaatagcagtttatacatct
gtcttcctaatttatccacttggtcaatctagctggttctttgcaccaagctttggtatt
gctgctatcttccgattcctattattccttcaaggttttcataattggacattaaatcct
tttcacatgatgggtgtagctggaatattgggaggtgccctactatcagctatccatggt
gctactgtcattaacactatttatcaagatgctcgtgcatatacaacatttcgtgctttc
tcaccaagtcaaccagaagaaacttattcaatgcttacagctaatcgtttttggtctcag
atatttggtgtagccttctcaaacaagagatggcttcatttcttcatgctatttgtacct
ctagcaggtatgtggacatcttcaattggtatcattggattagcatttaatctaagagct
tatgattttatttctcaagaattgaaagcagcagaagatcctgaatttgaaacattttat
acaaagaacattctactcaatgaaggtatacgactttggatggctgtccaagatcaagct
catgaaaattatcagttcccagaagaggtattgccaagaggtaattctttgtga

>KrC15_psbD
atgaaactcatctattctacaagattcatggcaagtaaagcttatcttcttggatcactt
actctactagatgactggttaaaacgagatcgatttgtctttattggctggtcaggtcta
ttattatttcctacagcttacttagcagcaggtggatggtttacagccaccgcttttgta
acatctttctttacacatggtctggtcacatcttatctagaagcttgtaattttttaaca
gctgcagtttcaactccagccaactctatggcccattcattactactactctggggacca
gaagcacatcttggatttactgcctgggtttgtataggaggcttatggacatttattgct
tttcatgggctcattggtctcatagcattttccctacgtcagtttgaaatagcacgatta
gttttaattagaccttataatgcacttgcattctcaggaccaatagcagtttatacatct
gtcttcctaatttatccacttggtcaatctagctggttctttgcaccaagctttggtatt
gctgctatcttccgattcctattattccttcaaggttttcataattggacattaaatcct
tttcacatgatgggtgtagctggaatattgggaggtgccctactatcagctatccatggt
gctactgtcattaacactatttatcaagatgctcgtgcatatacaacatttcgtgctttc
tcaccaagtcaaccagaagaaacttattcaatgcttacagctaatcgtttttggtctcag
atatttggtgtagccttctcaaacaagagatggcttcatttcttcatgctatttgtacct
ctagcaggtatgtggacatcttcaattggtatcattggattagcatttaatctaagagct
tatgattttatttctcaagaattgaaagcagcagaagatcctgaatttgaaacattttat
acaaagaacattctactcaatgaaggtatacgactttggatggctgtccaagatcaagct
catgaaaattatcagttcccagaagaggtattgccaagaggtaattctttgtga
>KrC2_psbD
atgaaactcatctattctacaagattcatggcaagtaaagcttatcttcttggatcactt
actctactagatgactggttaaaacgagatcgatttgtctttattggctggtcaggtcta
ttattatttcctacagcttacttagcagcaggtggatggtttacagccaccgcttttgta
acatctttctttacacatggtctggtcacatcttatctagaagcttgtaattttttaaca
gctgcagtttcaactccagccaactctatggcccattcattactactactctggggacca
gaagcacatcttggatttactgcctgggtttgtataggaggcttatggacatttattgct
tttcatgggctcattggtctcatagcattttccctacgtcagtttgaaatagcacgatta
gttttaattagaccttataatgcacttgcattctcaggaccaatagcagtttatacatct
gtcttcctaatttatccacttggtcaatctagctggttctttgcaccaagctttggtatt
gctgctatcttccgattcctattattccttcaaggttttcataattggacattaaatcct
tttcacatgatgggtgtagctggaatattgggaggtgccctactatcagctatccatggt
gctactgtcattaacactatttatcaagatgctcgtgcatatacaacatttcgtgctttc
tcaccaagtcaaccagaagaaacttattcaatgcttacagctaatcgtttttggtctcag
atatttggtgtagccttctcaaacaagagatggcttcatttcttcatgctatttgtacct
ctagcaggtatgtggacatcttcaattggtatcattggattagcatttaatctaagagct
tatgattttatttctcaagaattgaaagcagcagaagatcctgaatttgaaacattttat
acaaagaacattctactcaatgaaggtatacgactttggatggctgtccaagatcaagct
catgaaaattatcagttcccagaagaggtattgccaagaggtaattctttgtga
>KrC3_psbD
atgaaactcatctattctacaagattcatggcaagtaaagcttatcttcttggatcactt
actctactagatgactggttaaaacgagatcgatttgtctttattggctggtcaggtcta
ttattatttcctacagcttacttagcagcaggtggatggtttacagccaccgcttttgta
acatctttctttacacatggtctggtcacatcttatctagaagcttgtaattttttaaca
gctgcagtttcaactccagccaactctatggcccattcattactactactctggggacca
gaagcacatcttggatttactgcctgggtttgtataggaggcttatggacatttattgct
tttcatgggctcattggtctcatagcattttccctacgtcagtttgaaatagcacgatta
gttttaattagaccttataatgcacttgcattctcaggaccaatagcagtttatacatct
gtcttcctaatttatccacttggtcaatctagctggttctttgcaccaagctttggtatt
gctgctatcttccgattcctattattccttcaaggttttcataattggacattaaatcct
tttcacatgatgggtgtagctggaatattgggaggtgccctactatcagctatccatggt
gctactgtcattaacactatttatcaagatgctcgtgcatatacaacatttcgtgctttc
tcaccaagtcaaccagaagaaacttattcaatgcttacagctaatcgtttttggtctcag
atatttggtgtagccttctcaaacaagagatggcttcatttcttcatgctatttgtacct
ctagcaggtatgtggacatcttcaattggtatcattggattagcatttaatctaagagct
tatgattttatttctcaagaattgaaagcagcagaagatcctgaatttgaaacattttat
acaaagaacattctactcaatgaaggtatacgactttggatggctgtccaagatcaagct
catgaaaattatcagttcccagaagaggtattgccaagaggtaattctttgtga

>KrC4_psbD
atgaaactcatctattctacaagattcatggcaagtaaagcttatcttcttggatcactt
actctactagatgactggttaaaacgagatcgatttgtctttattggctggtcaggtcta
ttattatttcctacagcttacttagcagcaggtggatggtttacagccaccgcttttgta
acatctttctttacacatggtctggtcacatcttatctagaagcttgtaatttttaaca
gctgcagtttcaactccagccaactctatggcccattcattactactactctggggacca
gaagcacatcttggatttactgcctgggtttgtataggaggcttatggaaatttattgct
tttcatgggctcattggtctcatagcattttccctacgtcagtttgaaatagcacgatta
gttttaattagaccttataatgcacttgcattctcaggaccaatagcagtttatacatct
gtcttcctaatttatccacttggtcaatctagctggttctttgcaccaagctttggtatt
gctgctatcttccgattcctattattccttcaaggttttcataattggacattaaatcct
tttcacatgatgggtgtagctggaatattgggaggtgccctactatcagctatccatggt
gctactgtcattaacactatttatcaagatgctcgtgcatatacaacatttcgtgctttc
tcaccaagtcaaccagaagaaacttattcaatgcttacagctaatcgtttttggtctcag
atatttggtgtagccttctcaaacaagagatggcttcatttcttcatgctatttgtacct
ctagcaggtatgtggacatcttcaattggtatcattggattagcatttaatctaagagct
tatgattttatttctcaagaattgaaagcagcagaagatcctgaatttgaaacattttat
acaaagaacattctactcaatgaaggtatacgactttggatggctgtccaagatcaagct
catgaaaattatcagttcccagaagaggtattgccaagaggtaattctttgtga
>KrC5_psbD
atgaaactcatctattctacaagattcatggcaagtaaagcttatcttcttggatcactt
actctactagatgactggttaaaacgagatcgatttgtctttattggctggtcaggtcta
ttattatttcctacagcttacttagcagcaggtggatggtttacagccaccgcttttgta
acatctttctttacacatggtctggtcacatcttatctagaagcttgtaatttttaaca
gctgcagtttcaactccagccaactctatggcccattcattactactactctggggacca
gaagcacatcttggatttactgcctgggtttgtataggaggcttatggacatttattgct
tttcatgggctcattggtctcatagcattttccctacgtcagtttgaaatagcacgatta
gttttaattagaccttataatgcacttgcattctcaggaccaatagcagtttatacatct
gtcttcctaatttatccacttggtcaatctagctggttctttgcaccaagctttggtatt
gctgctatcttccgattcctattattccttcaaggttttcataattggacattaaatcca
tttcatatgatgggtgtagctggaatattgggaggtgccctactatcagctatccatggt
gctactgtcattaacactatttatcaagatgctcgtgcatatacaacatttcgtgctttc
tcaccaagtcaaccagaagaaacttattcaatgcttacagctaatcgtttttggtctcag
atatttggtgtagccttctcaaacaagagatggcttcatttcttcatgctatttgtacct
ctagcaggtatgtggacatcttcaattggtatcattggattagcatttaatctaagagct
tatgattttatttctcaagaattgaaagcagcagaagatcctgaatttgaaacattttat
acaaagaacattctactcaatgaaggtatacgactttggatggctgtccaagatcaagct
catgaaaattatcagttcccagaagaggtattgccaagaggtaattctttgtga
>KrC6_psbD
atgaaactcatctattctacaagattcatggcaagtaaagcttatcttcttggatcactt
actctactagatgactggttaaaacgagatcgatttgtctttattggctggtcaggtcta
ttattatttcctacagcttacttagcagcaggtggatggtttacagccaccgcttttgta
acatctttctttacacatggtctggtcacatcttatctagaagcttgtaatttttaaca
gctgcagtttcaactccagccaactctatggcccattcattactactactctggggacca
gaagcacatcttggatttactgcctgggtttgtataggaggcttatggacatttattgct
tttcatgggctcattggtctcatagcattttccctacgtcagtttgaaatagcacgatta
gttttaattagaccttataatgcacttgcattctcaggaccaatagcagtttatacatct
gtcttcctaatttatccacttggtcaatctagctggttctttgcaccaagctttggtatt
gctgctatcttccgattcctattattccttcaaggttttcataattggacattaaatcct
tttcacatgatgggtgtagctggaatattgggaggtgccctactatcagctatccatggt
gctactgtcattaacactatttatcaagatgctcgtgcatatacaacatttcgtgctttc
tcaccaagtcaaccagaagaaacttattcaatgcttacagctaatcgtttttggtctcag
atatttggtgtagccttctcaaacaagagatggcttcatttcttcatgctatttgtacct
ctagcaggtatgtggacatcttcaattggtatcattggattagcatttaatctaagagct
tatgattttatttctcaagaattgaaagcagcagaagatcctgaatttgaaacattttat
acaaagaacattctactcaatgaaggtatacgactttggatggctgtccaagatcaagct
catgaaaattatcagttcccagaagaggtattgccaagaggtaattctttgtga

>KrC7_psbD
atgaaactcatctattctacaagattcatggcaagtaaagcttatcttcttggatcactt
actctactagatgactggttaaaacgagatcgatttgtctttattggctggtcaggtcta
ttattatttcctacagcttacttagcagcaggtggatggtttacagccaccgcttttgta
acatctttctttacacatggtctggtcacatcttatctagaagcttgtaattttttaaca
gctgcagtttcaactccagccaactctatggcccattcattactactactctggggacca
gaagcacatcttggatttactgcctgggtttgtataggaggcttatggacatttattgct
tttcatgggctcattggtctcatagcattttccctacgtcagtttgaaatagcacgatta
gttttaattagaccttataatgcacttgcattctcaggaccaatagcagtttatacatct
gtcttcctaatttatccacttggtcaatctagctggttctttgcaccaagctttggtatt
gctgctatcttccgattcctattattccttcaaggttttcataattggacattaaatcct
tttcacatgatgggtgtagctggaatattgggaggtgccctactatcagctatccatggt
gctactgtcattaacactatttatcaagatgctcgtgcatatacaacatttcgtgctttc
tcaccaagtcaaccagaagaaacttattcaatgcttacagctaatcgtttttggtctcag
atatttggtgtagccttctcaaacaagagatggcttcatttcttcatgctatttgtacct
ctagcaggtatgtggacatcttcaattggtatcattggattagcatttaatctaagagct
tatgattttatttctcaagaattgaaagcagcagaagatcctgaatttgaaacattttat
acaaagaacattctactcaatgaaggtatacgactttggatggctgtccaagatcaagct
catgaaaattatcagttcccagaagaggtattgccaagaggtaattctttgtga
>KrC8_psbD
atgaaactcatctattctacaagattcatggcaagtaaagcttatcttcttggatcactt
actctactagatgactggttaaaacgagatcgatttgtctttattggctggtcaggtcta
ttattatttcctacagcttacttagcagcaggtggatggtttacagccaccgcttttgta
acatctttctttacacatggtctggtcacatcttatctagaagcttgtaattttttaaca
gctgcagtttcaactccagccaactctatggcccattcattactactactctggggacca
gaagcacatcttggatttactgcctgggtttgtataggaggcttatggacatttattgct
tttcatgggctcattggtctcatagcattttccctacgtcagtttgaaatagcacgatta
gttttaattagaccttataatgcacttgcattctcaggaccaatagcagtttatacatct
gtcttcctaatttatccacttggtcaatctagctggttctttgcaccaagctttggtatt
gctgctatcttccgattcctattattccttcaaggttttcataattggacattaaatcct
tttcacatgatgggtgtagctggaatattgggaggtgccctactatcagctatccatggt
gctactgtcattaacactatttatcaagatgctcgtgcatatacaacatttcgtgctttc
tcaccaagtcaaccagaagaaacttattcaatgcttacagctaatcgtttttggtctcag
atatttggtgtagccttctcaaacaagagatggcttcatttcttcatgctatttgtacct
ctagcaggtatgtggacatcttcaattggtatcattggattagcatttaatctaagagct
tatgattttatttctcaagaattgaaagcagcagaagatcctgaatttgaaacattttat
acaaagaacattctactcaatgaaggtatacgactttggatggctgtccaagatcaagct
catgaaaattatcagttcccagaagaggtattgccaagaggtaattctttgtga
>KrC9_psbD
atgaaactcatctattctacaagattcatggcaagtaaagcttatcttcttggatcactt
actctactagatgactggttaaaacgagatcgatttgtctttattggctggtcaggtcta
ttattatttcctacagcttacttagcagcaggtggatggtttacagctaccgcttttgta
acatctttctttacacatggtttggccacatcttatctagaagcttgtaattttttaaaa
gctgcagtttcaactccagccaactctatggcccattcattactactactctggggacca
gaagcacatcttggatttactgcctgggtttgtataggaggcttatggacatttattgct
tttcatgggctcattggtctcatagcattttccctacgtcagtttgaaatagcacgatta
gttttaattagaccttataatgcacttgcattctcaggaccaatagcagtttatacatct
gtcttcctaatttatccacttggtcaatctagctggttctttgcaccaagctttggtatt
gctgctatcttccgattcctattattccttcaaggttttcataattggacattaaatcct
tttcacatgatgggtgtagctggaatattgggaggtgccctactatcagctatccatggt
gctactgtcattaacactatttatcaagatgctcgtgcatatacaacatttcgtgctttc
tcaccaagtcaaccagaagaaacttattcaatgcttacagctaatcgtttttggtctcag
atatttggtgtagccttctcaaacaagagatggcttcatttcttcatgctatttgtacct
ctagcaggtatgtggacatcttcaattggtatcattggattagcatttaatctaagagct
tatgattttatttctcaagaattgaaagcagcagaagatcctgaatttgaaacattttat
acaaagaacattctactcaatgaaggtatacgactttggatggctgtccaagatcaagct
catgaaaattatcagttcccagaagaggtattgccaagaggtaattctttgtga

>KrD1_psbD
atgaaactcatctattctacaagattcatggcaagtaaagcttatcttcttggatcactt
actctactagatgactggttaaaacgagatcgatttgtctttattggctggtcaggtcta
ttattatttcctacagcttacttagcagcaggtggatggtttacagccaccgcttttgta
acatctttctttacacatggtctggtcacatcttatctagaagcttgtaatttttaaca
gctgcagtttcaactccagccaactctatggcccattcattactactactctggggacca
gaagcacatcttggatttactgcctgggtttgtataggaggcttatggacatttattgct
tttcatgggctcattggtctcatagcattttccctacgtcagtttgaaatagcacgatta
gttttaattagaccttataatgcacttgcattctcaggaccaatagcagtttatacatct
gtcttcctaatttatccacttggtcaatctagctggttctttgcaccaagctttggtatt
gctgctatcttccgattcctattattccttcaaggttttcataattggacattaaatcct
tttcacatgatgggtgtagctggaatattgggaggtgccctactatcagctatccatggt
gctactgtcattaacactatttatcaagatgctcgtgcatatacaacatttcgtgctttc
tcaccaagtcaaccagaagaaacttattcaatgcttacagctaatcgtttttggtctcag
atatttggtgtagccttctcaaacaagagatggcttcatttcttcatgctatttgtacct
ctagcaggtatgtggacatcttcaattggtatcattggattagcatttaatctaagagct
tatgattttatttctcaagaattgaaagcagcagaagatcctgaatttgaaacattttat
acaaagaacattctactcaatgaaggtatacgactttggatggctgtccaagatcaagct
catgaaaattatcagttcccagaagaggtattgccaagaggtaattctttgtga
>KrD11_psbD
atgaaactcatctattctacaagattcatggcaagtaaagcttatcttcttggatcactt
actctactagatgactggttaaaacgagatcgatttgtctttattggctggtcaggtcta
ttattatttcctacagcttacttagcagcaggtggatggtttacagccaccgcttttgta
acatctttctttacacatggtctggtcacatcttatctagaagcttgtaatttttaaca
gctgcagtttcaactccagccaactctatggcccattcattactactactctggggacca
gaagcacatcttggatttactgcctgggtttgtataggaggcttatggacatttattgct
tttcatgggctcattggtctcatagcattttccctacgtcagtttgaaatagcacgatta
gttttaattagaccttataatgcacttgcattctcaggaccaatagcagtttatacatct
gtcttcctaatttatccacttggtcaatctagctggttctttgcaccaagctttggtatt
gctgctatcttccgattcctattattccttcaaggttttcataattggacattaaatcct
tttcacatgatgggtgtagctggaatattgggaggtgccctactatcagctatccatggt
gctactgtcattaacactatttatcaagatgctcgtgcatatacaacatttcgtgctttc
tcaccaagtcaaccagaagaaacttattcaatgcttacagctaatcgtttttggtctcag
atatttggtgtagccttctcaaacaagagatggcttcatttcttcatgctatttgtacct
ctagcaggtatgtggacatcttcaattggtatcattggattagcatttaatctaagagct
tatgattttatttctcaagaattgaaagcagcagaagatcctgaatttgaaacattttat
acaaagaacattctactcaatgaaggtatacgactttgcatggctgtccaagatcaagct
catgaaaattatcagttcccagaagaggtattgccaagaggtaattctttgtga
>KrD13_psbD
atgaaactcatctattctacaagattcatggcaagtaaagcttatcttcttggatcactt
actctactagatgactggttaaaacgagatcgatttgtctttattggctggtcaggtcta
ttattatttcctacagcttacttagcagcaggtggatggtttacagccaccgcttttgta
acatctttctttacacatggtctggtcacatcttatctagaagcttgtaatttttaaca
gctgcagtttcaactccagccaactctatggcccattcattactactactctggggacca
gaagcacatcttggatttactgcctgggtttgtataggaggcttatggacatttattgct
tttcatgggctcattggtctcatagcattttccctacgtcagtttgaaatagcacgatta
gttttaattagaccttataatgcacttgcattctcaggaccaatagcagtttatacatct
gtcttcctaatttatccacttggtcaatctagctggttctttgcaccaagctttggtatt
gctgctatcttccgattcctattattccttcaaggttttcataattggacattaaatcct
tttcacatgatgggtgtagctggaatattgggaggtgccctactatcagctatccatggt
gctactgtcattaacactatttatcaagatgctcgtgcatatacaacatttcgtgctttc
tcaccaagtcaaccagaagaaacttattcaatgcttacagctaatcgtttttggtctcag
atatttggtgtagccttctcaaacaagagatggcttcatttcttcatgctatttgtacct
ctagcaggtatgtggacatcttcaattggtatcattggattagcatttaatctaagagct
tatgattttatttctcaagaattgaaagcagcagaagatcctgaatttgaaacattttat
acaaagaacattctactcaatgaaggtatacgactttggatggctgtccaagatcaagct
catgaaaattatcagttcccagaagaggtattgccaagaggtaattctttgtga

>KrD3_psbD
atgaaactcatctattctacaagattcatggcaagtaaagcttatcttcttggatcactt
actctactagatgactggttaaaacgagatcgatttgtctttattggctggtcaggtcta
ttattatttcctacagcttacttagcagtaggtggatggtttacagccaccgcttttgta
acatctttctttacacatggtctggtcacatcttatctagaagcttgtaattttttaaca
gctgcagtttcaactccagccaactctatggcccattcattactactactctggggacca
gaagcacatcttggatttactgcctgggtttgtataggaggcttatggacatttattgct
tttcatgggctcattggtctcatagcattttccctacgtcagtttgaaatagcacgatta
gttttaattagaccttataatgcacttgcattctcaggaccaatagcagtttatacatct
gtcttcctaatttatccacttggtcaatctagctggttctttgcaccaagctttggtatt
gctgctatcttccgattcctattattccttcaaggttttcataattggacattaaatcct
tttcacatgatgggtgtagctggaatattgggaggtgccctactatcagctatccatggt
gctactgtcattaacactatttatcaagatgctcgtgcatatacaacatttcgtgctttc
tcaccaagtcaaccagaagaaacttattcaatgcttacagctaatcgcttctggtctcaa
atatttggtgtagccttctcaaacaagagatggcttcatttcttcatgctatttgtacct
ctagcaggtatgtggacatcttcaattggtatcattggattagcatttaatctaagagct
tatgattttatttctcaagaattgaaagcagcagaagatcctgaatttgaaacattttat
acaaagaacattctactcaatgaaggtatacgactttggatggctgtccaagatcaagct
catgaaaattatcagttcccagaagaggtattgccaagaggtaattctttgtga
>KrD8_psbD
atgaaactcatctattctacaagattcatggcaagtaaatcttatcttcttggatcactt
actctactagatgactggttaaaacgagatcgatttgtctttattggctggtcaggtcta
ttattatttcctacagcttacttagcagcaggtggatggtttacagccaccgcttttgta
acatctttctttacacatggtctggtcacatcttatctagaagcttgtaattttttaaca
gctgcagtttcaactccagccaactctatggcccattcattactactactctggggacca
gaagcacatcttggatttactgcctgggtttgtataggaggcttatggacatttattgct
tttcatgggctcattggtctcatagcattttccctacgtcagtttgaaatagcacgatta
gttttaattagaccttataatgcacttgcattctcaggaccaatagcagtttatacatct
gtcttcctaatttatccacttggtcaatctagctggttctttgcaccaagctttggtatt
gctgctatcttccgattcctattattccttcaaggttttcataattggacattaaatcct
tttcacatgatgggtgtagctggaatattgggaggtgccctactatcagctatccatggt
gctactgtcattaacactatttatcaagatgctcgtgcatatacaacatttcgtgctttc
tcaccaagtcaaccagaagaaacttattcaatgcttacagctaatcgtttttggtctcag
atatttggtgtagccttctcaaacaagagatggcttcatttcttcatgctatttgtacct
ctagcaggtatgtggacatcttcaattggtatcattggattagcatttaatctaagagct
tatgattttatttctcaagaattgaaagcagcagaagatcctgaatttgaaacattttat
acaaagaacattctactcaatgaaggtatacgactttggatggctgtccaagatcaagct
catgaaaattatcagttcccagaagaggtattgccaagaggtaattctttgtga
>KrD9_psbD
atgaaactcatctattctacaagattcatggcaagtaaagcttatcttcttggatcactt
actctactagatgactggttaaaacgagatcgatttgtctttattggctggtcaggtcta
ttattatttcctacagcttacttagcagcaggtggatggtttacagccaccgcttttgta
acatctttctttacacatggtctggtcacatcttatctagaagcttgtaactttttaaca
gctgcagtttcaactccagccaactctatggcccattcattactactactctggggacca
gaagcacatcttggatttactgcctgggtttgtataggaggcttatggacatttattgct
tttcatgggctcattggtctcatagcattttccctacgtcagtttgaaatagcacgatta
gttttaattagaccttataatgcacttgcattctcaggaccaataggagtttatacatct
gtcttcctaatttatccacttggtcaatctagctggttctttgcaccaagctttgggatt
gctgctatcttccgattcctattattccttcaaggttttcataattggacattaaatcct
tttcacatgatgggtgtagctggaatattgggaggtgccctactatcagctatccatggt
gctactgtcattaacactatttatcaagatgctcgtgcatatacaacatttcgtgctttc
tcaccaagtcaaccagaagaaacttattcaatgcttacagctaatcgtttttggtctcag
atatttggtgtagccttctcaaacaagagatggcttcatttcttcatgctatttgtacct
ctagcaggtatgtggacatcttcaattggtatcattggattagcatttaatctaagagct
tatgattttatttctcaagaattgaaagcagcagaagatcctgaatttgaaacattttat
acaaagaacattctactcaatgaaggtatacgactttggatggctgtccaagatcaagct
catgaaaattatcagttcccagaagaggtattgccaagaggtaattctttgtga

>KrE1_psbD
atgaaactcatctattctacaagattcatggcaagtaaagcttatcttcttggatcactt
actctactagatgactggttaaaacgagatcgatttgtctttattggctggtcaggtcta
ttattatttcctacagcttacttagcagcaggtggatggtttacagccaccgcttttgta
acatctttctttacacatggtctggtcacatcttatctagaagcttgtaattttttaaca
gctgcagtttcaactccagccaactctatggcccattcattactactactctggggacca
gaagcacatcttggatttactgcctgggtttgtataggaggcttatggacatttattgct
tttcatgggctcattggtctcatagcattttccctacgtcagtttgaaatagcacgatta
gttttaattagaccttataatgcacttgcattctcaggaccaatagcagtttatacatct
gtcttcctaatttatccacttggtcaatctagctggttctttgcaccaagctttggtatt
gctgctatcttccgattcctattattccttcaaggttttcataattggacattaaatcct
tttcacatgatgggtgtagctggaatattgggaggtgccctactatcagctatccatggt
gctactgtcattaacactatttatcaagatgctcgtgcatatacaacatttcgtgctttc
tcaccaagtcaaccagaagaaacttattcaatgcttacagctaatcgtttttggtctcag
atatttggtgtagccttctcaaacaagagatggcttcatttcttcatgctatttgtacct
ctagcaggtatgtggacatcttcaattggtatcattggattagcatttaatctaagagct
tatgattttatttctcaagaattgaaagcagcagaagatcctgaatttgaaacattttat
acaaagaacattctactcaatgaaggtatacgactttggatggctgtccaagatcaagct
catgaaaattatcagttcccagaagaggtattgccaagaggtaattctttgtga
>KrE10_psbD
atgaaactcatctattctacaagattcatggcaagtaaagcttatcttcttggatcactt
actctactagatgactggttaaaacgagatcgatttgtctttattggctggtcaggtcta
ttattatttcctacagcttacttagcagcaggtggatggtttacagccaccgcttttgta
acatctttctttacacatggtctggtcacatcttatctagaagcttgtaattttttaaca
gctgcagtttcaactccagccaactctatggcccattcattactactactctggggacca
gaagcacatcttggatttactgcctgggtttgtataggaggcttatggacatttattgct
tttcatgggctcattggtctcataggattttccctacgtcagtttgaaatagcacgatta
gttttaattagaccttataatgcacttgcattctcaggaccaataggagtttatacatct
gtcttcctaatttatccacttggtcaatctagctggttctttgcaccaagctttggtatt
gctgctatcttccgattcctattattccttcaaggttttcataattggacattaaatcct
tttcacatgatgggtgtagctggaatattgggaggtgccctactatcagctatccatggt
gctactgtcattaacactatttatcaagatgctcgtgcatatacaacatttcgtgctttc
tcaccaagtcaaccagaagaaacttattcaatgcttacagctaatcgtttttggtctcag
atatttggtgtagccttctcaaacaagagatggcttcatttcttcatgctatttgtacct
ctagcaggtatgtggacatcttcaattggtatcattggattagcatttaatctaagagct
tatgattttatttctcaagaattgaaagcagcagaagatcctgaatttgaaacattttat
acaaagaacattctactcaatgaaggtatacgactttggatggctgtccaagatcaagct
catgaaaattatcagttcccagaagaggtattgccaagaggtaattctttgtga
>KrE11_psbD
atgaaactcatctattctacaagattcatggcaagtaaagcttatcttcttggatcactt
actctactagatgactggttaaaacgagatcgatttgtctttattggctggtcaggtcta
ttattatttcctacagcttacttagcagcaggtggatggtttacagccaccgcttttgta
acatctttctttacacatggtctggtcacatcttatctagaagcttgtaactttttaaca
gctgcagtttcaactccagccaactctatggcccattcattactactactctggggacca
gaagcacatcttggatttactgcctgggtttgtataggaggcttatggacatttattgct
tttcatgggctcattggtctcatagcattttccctacgtcagtttgaaatagcacgatta
gttttaattagaccttataatgcacttgcattctcaggaccaataggagtttatacatct
gtcttcctaatttatccacttggtcaatctagctggttctttgcaccaagctttggtatt
gctgctatcttccgattcctattattccttcaaggttttcataattggacattaaatcct
tttcacatgatgggtgtagctggaatattgggaggtgccctactatcagctatccatggt
gctactgtcattaacactatttatcaagatgctcgtgcatatacaacatttcgtgctttc
tcaccaagtcaaccagaagaaacttattcaatgcttacagctaatcgtttttggtctcag
atatttggtgtagccttctcaaacaagagatggcttcatttcttcatgctatttgtacct
ctagcaggtatgtggacatcttcaattggtatcattggattagcatttaatctaagagct
tatgattttatttctcaagaattgaaagcagcagaagatcctgaatttgaaacattttat
acaaagaacattctactcaatgaaggtatacgactttggatggctgtccaagatcaagct
catgaaaattatcagttcccagaagaggtattgccaagaggtaattctttgtga

>KrE12_psbD
atgaaactcatctattctacaagattcatggcaagtaaagcttatcttcttggatcactt
actctactagatgactggttaaaacgagatcgatttgtctttattggctggtcaggtcta
ttattatttcctacagcttacttagcagcaggtggatggtttacagccaccgcttttgta
acatctttctttacacatggtctggtcacatcttatctagaagcttgtaatttttaaca
gctgcagtttcaactccagccaactctatggcccattcattactactactctggggacca
gaagcacatcttggatttactgcctgggtttgtataggaggcttatggacatttattgct
tttcatgggctcattggtctcatagcattttccctacgtcagtttgaaatagcacgatta
gttttaattagaccttataatgcacttgcattctcaggaccaatagcagtttatacatct
gtcttcctaatttatccacttggtcaatctagctggttctttgcaccaagctttggtatt
gctgctatcttccgattcctattattccttcaaggttttcataattggacattaaatcct
tttcacatgatgggtgtagctggaatattgggaggtgccctactatcagctatccatggt
gctactgtcattaacactatttatcaagatgctcgtgcatatacaacatttcgtgctttc
tcaccaagtcaaccagaagaaacttattcaatgcttacagctaatcgtttttggtctcag
atatttggtgtagccttctcaaacaagagatggcttcatttcttcatgctatttgtacct
ctagcaggtatgtggacatcttcaattggtatcattggattagcatttaatctaagagct
tatgattttatttctcaagaattgaaagcagcagaagatcctgaatttgaaacattttat
acaaagaacattctactcaatgaaggtatacgactttggatggctgtccaagatcaagct
catgaaaattatcagttcccagaagaggtattgccaagaggtaattctttgtga
>KrE13_psbD
atgaaactcatctattctacaagattcatggcaagtaaagcttatcttcttggatcactt
actctactagatgactggttaaaacgagatcgatttgtctttattggctggtcaggtcta
ttattatttcctacagcttacttagcagcaggtggatggtttacagccaccgcttttgta
acatctttctttacacatggtctggtcacatcttatctagaagcttgtaatttttaaca
gctgcagtttcaactccagccaactctatggcccattcattactactactctggggacca
gaagcacatcttggatttactgcctgggtttgtataggaggcttatggacatttattgct
tttcatgggctcattggtctcatagcattttccctacgtcagtttgaaatagcacgatta
gttttaattagaccttataatgcacttgcattctcaggaccaatagcagtttatacatct
gtcttcctaatttatccacttggtcaatctagctggttctttgcaccaagctttggtatt
gctgctatcttccgattcctattattccttcaaggttttcataattggacattaaatcct
tttcacatgatgggtgtagctggaatattgggaggtgccctactatcagctatccatggt
gctactgtcattaacactatttatcaagatgctcgtgcatatacaacatttcgtgctttc
tcaccaagtcaaccagaagaaacttattcaatgcttacagctaatcgtttttggtctcag
atatttggtgtagccttctcaaacaagagatggcttcatttcttcatgctatttgtacct
ctagcaggtatgtggacatcttcaattggtatcattggattagcatttaatctaagagct
tatgattttatttctcaagaattgaaagcagcagaagatcctgaatttgaaacattttat
acaaagaacattctactcaatgaaggtatacgactttggatggctgtccaagatcaagct
catgaaaattatcagttcccagaagaggtattgccaagaggtaattctttgtga
>KrE14_psbD
atgaaactcatctattctacaagattcatggcaagtaaagcttatcttcttggatcactt
actctactagatgactggttaaaacgagatcgatttgtctttattggctggtcaggtcta
ttattatttcctacagcttacttagcagcaggtggatggtttacagccaccgcttttgta
acatctttctttacacatggtctggtcacatcttatctagaagcttgtaatttttaaca
gctgcagtttcaactccagccaactctatggcccattcattactactactctggggacca
gaagcacatcttggatttactgcctgggtttgtataggaggcttatggacatttattgct
tttcatgggctcattggtctcatagcattttccctacgtcagtttgaaatagcacgatta
gttttaattagaccttataatgcacttgcattctcaggaccaatagcagtttatacatct
gtcttcctaatttatccacttggtcaatctagctggttctttgcaccaagctttggtatt
gctgctatcttccgattcctattattccttcaaggttttcataattggacattaaatcct
tttcacatgatgggtgtagctggaatattgggaggtgccctactatcagctatccatggt
gctactgtcattaacactatttatcaagatgctcgtgcatatacaacatttcgtgctttc
tcaccaagtcaaccagaagaaacttattcaatgcttacagctaatcgtttttggtctcag
atatttggtgtagccttctcaaacaagagatggcttcatttcttcatgctatttgtacct
ctagcaggtatgtggacatcttcaattggtatcattggattagcatttaatctaagagct
tatgattttatttctcaagaattgaaagcagcagaagatcctgaatttgaaacattttat
acaaagaacattctactcaatgaaggtatacgactttggatggctgtccaagatcaagct
catgaaaattatcagttcccagaagaggtattgccaagaggtaattctttgtga

>KrE15_psbD
atgaaactcatctattctacaagattcatggcaagtaaagcttatcttcttggatcactt
actctactagatgactggttaaaacgagatcgatttgtctttattggctggtcaggtcta
ttattatttcctacagcttacttagcagcaggtggatggtttacagccaccgcttttgta
acatctttctttacacatggtctggtcacatcttatctagaagcttgtaatttttaaca
gctgcagtttcaactccagccaactctatggcccattcattactactactctggggacca
gaagcacatcttggatttactgcctgggtttgtataggaggcttatggacatttattgct
tttcatgggctcattggtctcatagcattttccctacgtcagtttgaaatagcacgatta
gttttaattagaccttataatgcacttgcattctcaggaccaatagcagtttatacatct
gtcttcctaatttatccacttggtcaatctagctggttctttgcaccaagctttggtatt
gctgctatcttccgattcctattattccttcaaggttttcataattggacattaaatcct
tttcacatgatgggtgtagctggaatattgggaggtgccctactatcagctatccatggt
gctactgtcattaacactatttatcaagatgctcgtgcatatacaacatttcgtgctttc
tcaccaagtcaaccagaagaaacttattcaatgcttacagctaatcgcttctggtctcaa
atatttggtgtagccttctcaaacaagagatggcttcatttcttcatgctatttgtacct
ctagcaggtatgtggacatcttcaattggtatcattggattagcatttaatctaagagct
tatgattttatttctcaagaattgaaagcagcagaagatcctgaatttgaaacattttat
acaaagaacattctactcaatgaaggtatacgactttggatggctgtccaagatcaagct
catgaaaattatcagttcccagaagaggtattgccaagaggtaattctttgtga
>KrE2_psbD
atgaaactcatctattctacaagattcatggcaagtaaagcttatcttcttggatcactt
actctactagatgactggttaaaacgagatcgatttgtctttattggctggtcaggtcta
ttattatttcctacagcttacttagcagcaggtggatggtttacagccaccgcttttgta
acatctttctttacacatggtctggtcacatcttatctagaagcttgtaatttttaaca
gctgcagtttcaactccagccaactctatggcccattcattactactactctggggacca
gaagcacatcttggatttactgcctgggtttgtataggaggcttatggacatttattgct
tttcatgggctcattggtctcatagcattttccctacgtcagtttgaaatagcacgatta
gttttaattagaccttataatgcacttgcattctcaggaccaatagcagtttatacatct
gtcttcctaatttatccacttggtcaatctagctggttctttgcaccaagctttggtatt
gctgctatcttccgattcctattattccttcaaggttttcataattggacattaaatcct
tttcacatgatgggtgtagctggaatattgggaggtgccctactatcagctatccatggt
gctactgtcattaacactatttatcaagatgctcgtgcatatacaacatttcgtgctttc
tcaccaagtcaaccagaagaaacttattcaatgcttacagctaatcgtttttggtctcag
atatttggtgtagccttctcaaacaagagatggcttcatttcttcatgctatttgtacct
ctagcaggtatgtggacatcttcaattggtatcattggattagcatttaatctaagagct
tatgattttatttctcaagaattgaaagcagcagaagatcctgaatttgaaacattttat
acaaagaacattctactcaatgaaggtatacgactttggatggctgtccaagatcaagct
catgaaaattatcagttcccagaagaggtattgccaagaggtaattctttgtga
>KrE3_psbD
atgaaactcatctattctacaagattcatggcaagtaaagcttatcttcttggatcactt
actctactagatgactggttaaaacgagatcgatttgtctttattggctggtcaggtcta
ttattatttcctacagcttacttagcagcaggtggatggtttacagccaccgcttttgta
acatctttctttacacatggtttggccacatcttatctagaagcttgtaactttttaaca
gctgcagtttcaactccagccaactctatggcccattcattactactactctggggacca
gaagcacatcttggatttactgcctgggtttgtataggaggcttatggacatttattgct
tttcatgggctcattggtctcatagcattttccctacgtcagtttgaaatagcacgatta
gttttaattagaccttataatgcacttgcattctcaggaccaatagcagtttatacatct
gtcttcctaatttatccacttggtcaatctagctggttctttgcaccaagctttggtatt
gctgctatcttccgattcctattattccttcaaggttttcataattggacattaaatcct
tttcacatgatgggtgtagctggaatattgggaggtgccctactatcagctatccatggt
gctactgtcattaacactatttatcaagatgctcgtgcatatacaacatttcgtgctttc
tcaccaagtcaaccagaagaaacttattcaatgcttacagctaatcgtttttggtctcaa
atatttggtgtagccttctcaaacaagagatggcttcatttcttcatgctatttgtacct
ctagcaggtatgtggacatcttcaattggtatcattggattagcatttaatctaagagct
tatgattttatttctcaagaattgaaagcagcagaagatcctgaatttgaaacattttat
acaaagaacattctactcaatgaaggtatacgactttggatggctgtccaagatcaagct
catgaaaattatcagttcccagaagaggtattgccaagaggtaattctttgtga

>KrE4_psbD
atgaaactcatctattctacaagattcatggcaagtaaagcttatcttcttggatcactt
actctactagatgactggttaaaacgagatcgatttgtctttattggctggtcaggtcta
ttattatttcctacagcttacttagcagcaggtggatggtttacagccaccgcttttgta
acatctttctttacacatggtctggtcacatcttatctagaagcttgtaattttttaaca
gctgcagtttcaactccagccaactctatggcccattcattactactactctggggacca
gaagcacatcttggatttactgcctgggtttgtataggaggcttatggacatttattgct
tttcatgggctcattggtctcatagcattttccctacgtcagtttgaaatagcacgatta
gttttaattagaccttataatgcacttgcattctcaggaccaatagcagtttatacatct
gtcttcctaatttatccacttggtcaatctagctggttctttgcaccaagctttggtatt
gctgctatcttccgattcctattattccttcaaggttttcataattggacattaaatcct
tttcacatgatgggtgtagctggaatattgggaggtgccctactatcagctatccatggt
gctactgtcattaacactatttatcaagatgctcgtgcatatacaacatttcgtgctttc
tcaccaagtcaaccagaagaaacttattcaatgcttacagctaatcgtttttggtctcag
atatttggtgtagccttctcaaacaagagatggcttcatttcttcatgctatttgtacct
ctagcaggtatgtggacatcttcaattggtatcattggattagcatttaatctaagagct
tatgattttatttctcaagaattgaaagcagcagaagatcctgaatttgaaacattttat
acaaagaacattctactcaatgaaggtatacgactttggatggctgtccaagatcaagct
catgaaaattatcagttcccagaagaggtattgccaagaggtaattctttgtga
>KrE5_psbD
atgaaactcatctattctacaagattcatggcaagtaaagcttatcttcttggatcactt
actctactagatgactggttaaaacgagatcgatttgtctttattggctggtcaggtcta
ttattatttcctacagcttacttagcagcaggtggatggtttacagccaccgcttttgta
acatctttctttacacatggtctggtcacatcttatctagaagcttgtaattttttaaca
gctgcagtttcaactccagccaactctatggcccattcattactactactctggggacca
gaagcacatcttggatttactgcctgggtttgtataggaggcttatggacatttattgct
tttcatgggctcattggtctcatagcattttccctacgtcagtttgaaatagcacgatta
gttttaattagaccttataatgcacttgcattctcaggaccaatagcagtttatacatct
gtcttcctaatttatccacttggtcaatctagctggttctttgcaccaagctttggtatt
gctgctatcttccgattcctattattccttcaaggttttcataattggacattaaatcct
tttcacatgatgggtgtagctggaatattgggaggtgccctactatcagctatccatggt
gctactgtcattaacactatttatcaagatgctcgtgcatatacaacatttcgtgctttc
tcaccaagtcaaccagaagaaacttattcaatgcttacagctaatcgtttttggtctcag
atatttggtgtagccttctcaaacaagagatggcttcatttcttcatgctatttgtacct
ctagcaggtatgtggacatcttcaattggtatcattggattagcatttaatctaagagct
tatgattttatttctcaagaattgaaagcagcagaagatcctgaatttgaaacattttat
acaaagaacattctactcaatgaaggtatacgactttggatggctgtccaagatcaagct
catgaaaattatcagttcccagaagaggtattgccaagaggtaattctttgtga
>KrE6_psbD
atgaaactcatctattctacaagattcatggcaagtaaagcttatcttcttggatcactt
actctactagatgactggttaaaacgagatcgatttgtctttattggctggtcaggtcta
ttattatttcctacagcttacttagcagcaggtggatggtttacagccaccgcttttgta
acatctttctttacacatggtctggtcacatcttatctagaagcttgtaattttttaaca
gctgcagtttcaactccagccaactctatggcccattcattactactactctggggacca
gaagcacatcttggatttactgcctgggtttgtataggaggcttatggacatttattgct
tttcatgggctcattggtctcatagcattttccctacgtcagtttgaaatagcacgatta
gttttaattagaccttataatgcacttgcattctcaggaccaatagcagtttatacatct
gtcttcctaatttatccacttggtcaatctagctggttctttgcaccaagctttggtatt
gctgctatcttccgattcctattattccttcaaggttttcataattggacattaaatcct
tttcacatgatgggtgtagctggaatattgggaggtgccctactatcagctatccatggt
gctactgtcattaacactatttatcaagatgctcgtgcatatacaacatttcgtgctttc
tcaccaagtcaaccagaagaaacttattcaatgcttacagctaatcgtttttggtctcag
atatttggtgtagccttctcaaacaagagatggcttcatttcttcatgctatttgtacct
ctagcaggtatgtggacatcttcaattggtatcattggattagcatttaatctaagagct
tatgattttatttctcaagaattgaaagcagcagaagatcctgaatttgaaacattttat
acaaagaacattctactcaatgaaggtatacgactttggatggctgtccaagatcaagct
catgaaaattatcagttcccagaagaggtattgccaagaggtaattctttgtga

>KrE7_psbD
atgaaactcatctattctacaagattcatgtcaagtaaagcttatcttcttggatcactt
actctactagatgactggttaaaacgagatcgatttgtctttattggctggtcaggtcta
ttattatttcctacagcttacttagcagcaggtggatggtttacagccaccgcttttgta
acatctttctttacacatggtctggtcacatcttatctagaagcttgtaatttttaaca
gctgcagtttcaactccagccaactctatggcccattcattactactactctggggacca
gaagcacatcttggatttactgcctgggtttgtataggaggcttatggacatttattgct
tttcatgggctcattggtctcatagcattttccctacgtcagtttgaaatagcacgatta
gttttaattagaccttataatgcacttgcattctcaggaccaatagcagtttatacatct
gtcttcctaatttatccacttggtcaatctagctggttctttgcaccaagctttgggatt
gctgctatcttccgattcctattattccttcaaggttttcataattggacattaaatcct
tttcacatgatgggtgtagctggaatattgggaggtgccctactatcagctatccatggt
gctactgtcattaacactatttatcaagatgctcgtgcatatacaacatttcgtgctttc
tcaccaagtcaaccagaagaaacttattcaatgcttacagctaatcgcttctggtctcaa
atatttggtgtagccttctcaaacaagagatggcttcatttcttcatgctatttgtacct
ctagcaggtatgtggacatcttcaattgctatcattggattagcatttaatctaagagct
tatgattttatttctcaagaattgaaagcagcagaagatcctgaatttgaaacattttat
acaaagaacattctactcaatgaaggtatacgactttggatggctgtccaagatcaagct
catgaaaattatcagttcccagaagaggtattgccaagaggtaattctttgtga
>KrE8_psbD
atgaaactcatctattctacaagattcatggcaagtaaagcttatcttcttggatcactt
actctactagatgactggttaaaacgagatcgatttgtctttattggctggtcaggtcta
ttattatttcctacagcttacttagcagcaggtggatggtttacagccaccgcttttgta
acatctttctttacacatggtctggtcacatcttatctagaagcttgtaatttttaaca
gctgcagtttcaactccagccaactctatggcccattcattactactactctggggacca
gaagcacatcttggatttactgcctgggtttgtataggaggcttatggacatttattgct
tttcatgggctcattggtctcatagcattttccctacgtcagtttgaaatagcacgatta
gttttaattagaccttataatgcacttgcattctcaggaccaatagcagtttatacatct
gtcttcctaatttatccacttggtcaatctagctggttctttgcaccaagctttggtatt
gctgctatcttccgattcctattattccttcaaggttttcataattggacattaaatcct
tttcacatgatgggtgtagctggaatattgggaggtgccctactatcagctatccatggt
gctactgtcattaacactatttatcaagatgctcgtgcatatacaacatttcgtgctttc
tcaccaagtcaaccagaagaaacttattcaatgcttacagctaatcgtttttggtctcag
atatttggtgtagccttctcaaacaagagatggcttcatttcttcatgctatttgtacct
ctagcaggtatgtggacatcttcaattggtatcattggattagcatttaatctaagagct
tatgattttatttctcaagaattgaaagcagcagaagatcctgaatttgaaacattttat
acaaagaacattctactcaatgaaggtatacgactttggatggctgtccaagatcaagct
catgaaaattatcagttcccagaagaggtattgccaagaggtaattctttgtga
>KrE9_psbD
atgaaactcatctattctacaagattcatggcaagtaaagcttatcttcttggatcactt
actctactagatgactggttaaaacgagatcgatttgtctttattggctggtcaggtcta
ttattatttcctacagcttacttagcagcaggtggatggtttacagccaccgcttttgta
acatctttctttacacatggtttggccacatcttatctagaagcttgtaactttttaaca
gctgcagtttcaactccagccaactctatggcccattcattactactactctggggacca
gaagcacatcttggatttactgcctgggtttgtataggaggcttatggacatttattgct
tttcatgggctcattggtctcatagcattttccctacgtcagtttgaaatagcacgatta
gttttaattagaccttataatgcacttgcattctcaggaccaatagcagtttatacatct
gtcttcctaatttatccacttggtcaatctagctggttctttgcaccaagctttggtatt
gctgctatcttccgattcctattattccttcaaggttttcataattggacattaaatcct
tttcacatgatgggtgtagctggaatattgggaggtgccctactatcagctatccatggt
gctactgtcattaacactatttatcaagatgctcgtgcatatacaacatttcgtgctttc
tcaccaagtcaaccagaagaaacttattcaatgcttacagctaatcgtttttggtctcag
atatttggtgtagccttctcaaacaagagatggcttcatttcttcatgctatttgtacct
ctagcaggtatgtggacatcttcaattggtatcattggattagcatttaatctaagagct
tatgattttatttctcaagaattgaaagcagcagaagatcctgaatttgaaacattttat
acaaagaacattctactcaatgaaggtatacgactttggatggctgtccaagatcaagct
catgaaaattatcagttcccagaagaggtattgccaagaggtaattctttgtga

>Mz10_psbD
atgaaactcatctattctacaagattcatggcaagtaaagcttatcttcttggatcactt
actctactagatgactggttaaaacgagatcgatttgtctttattggctggtcaggtcta
ttattatttcctacagcttacttagcagtaggtggatggtttacagctaccacttttgta
acatctttctttacacatggtttggccacatcttatctagaagcttgtaactttttaaca
gctgcagtttcaactccagccaactctatggcccattcattactactactctggggacca
gaagcacatcttggatttactgcctgggtttgtataggaggcttatggacatttattgct
tttcatgggctcattggtctcataggattttccctacgtcagtttgaaatagcacgatta
gttttaattagaccttataatgcacttgcattctcaggaccaatagcagtttatacatct
gtcttcctaatttatccacttggtcaatctagctggttctttgcaccaagctttgggatt
gctgctatcttccgattcctattattccttcaaggttttcataattggacattaaatcct
tttcacatgatgggtgtagctggaatattgggaggtgccctactatcagctatccatggt
gctactgtcattaacactatttatcaagatgctcgtgcatatacaacatttcgtgctttc
tcaccaagtcaaccagaagaaacttattcaatgcttacagctaatcgtttttggtctcaa
atatttggtgtagccttctcaaacaagagatggcttcatttcttcatgctatttgtacct
ctagcaggtatgtggacatcttcaattggtatcattggattagcatttaatctaagagct
tatgattttatttctcaagaattgaaagcagcagaagatcctgaatttgaaacattttat
acaaagaacattctactcaatgaaggtatacgactttggatggctgtccaagatcaagct
catgaaaattatcagttcccagaagaggtattgccaagaggtaattctttgtga
>Mz5_psbD
atgaaactcatctattctacaagattcatggcaagtaaagcttatcttcttggatcactt
actctactagatgactggttaaaacgagatcgatttgtctttattggctggtcaggtcta
ttattatttcctacagcttacttagcagcaggtggatggtttacagccaccgcttttgta
acatctttctttacacatggtctggccacatcttatctagaagcttgtaatttttttaaca
gctgcagtttcaactccagccaactctatggcccattcattactactactctggggacca
gaagcacatcttggatttactgcctgggtttgtataggaggcttatggacatttattgct
tttcatgggctcattggtctcatagcattttccctacgtcagtttgaaatagcacgatta
gttttaattagaccttataatgcacttgcattctcaggaccaatagcagtttatacatct
gtcttcctaatttatccacttggtcaatctagctggttctttgcaccaagctttggtatt
gctgctatcttccgattcctattattccttcaaggttttcataattggacattaaatcct
tttcacatgatgggtgtagctggaatattgggaggtgccctactatcagctatccatggt
gctactgtcattaacactatttatcaagatgctcgtgcatatacaacatttcgtgctttc
tcaccaagtcaaccagaagaaacttattcaatgcttacagctaatcgtttttggtctcag
atatttggtgtagccttctcaaacaagagatggcttcatttcttcatgctatttgtacct
ctagcaggtatgtggacatcttcaattggtatcattggattagcatttaatctaagagct
tatgattttatttctcaagaattgaaagcagcagaagatcctgaatttgaaacattttat
acaaagaacattctactcaatgaaggtatacgactttggatggctgtccaagatcaagct
catgaaaattatcagttcccagaagaggtattgccaagaggtaattctttgtga
>Mz6_psbD
atgaaactcatctattctacaagattcatggcaagtaaagcttatcttcttggatcactt
actctactagatgactggttaaaacgagatcgatttgtctttattggctggtcaggtcta
ttattatttcctacagcttacttagcagcaggtggatggtttacagccaccgcttttgta
acatctttctttacacatggtctggtcacatcttatctagaagcttgtaatttttttaaca
gctgcagtttcaactccagccaactctatggcccattcattactactactctggggacca
gaagcacatcttggatttactgcctgggtttgtataggaggcttatggacatttattgct
tttcatgggctcattggtctcatagcattttccctacgtcagtttgaaatagcacgatta
gttttaattagaccttataatgcacttgcattctcaggaccaatagcagtttatacatct
gtcttcctaatttatccacttggtcaatctagctggttctttgcaccaagctttggtatt
gctgctatcttccgattcctattattccttcaaggttttcataattggacattaaatcct
tttcacatgatgggtgtagctggaatattgggaggtgccctactatcagctatccatggt
gctactgtcattaacactatttatcaagatgctcgtgcatatacaacatttcgtgctttc
tcaccaagtcaaccagaagaaacttattcaatgcttacagctaatcgtttttggtctcag
atatttggtgtagccttctcaaacaagagatggcttcatttcttcatgctatttgtacct
ctagcaggtatgtggacatcttcaattggtatcattggattagcatttaatctaagagct
tatgattttatttctcaagaattgaaagcagcagaagatcctgaatttgaaacattttat
acaaagaacattctactcaatgaaggtatacgactttggatggctgtccaagatcaagct
catgaaaattatcagttcccagaagaggtattgccaagaggtaattctttgtga

>Mz7_psbD
atgaaactcatctattctacaagattcatggcaagtaaagcttatcttcttggatcactt
actctactagatgactggttaaaacgagatcgatttgtctttattggctggtcaggtcta
ttattatttcctacagcttacttagcagcaggtggatggtttacagccaccgcttttgta
acatctttctttacacatggtctggtcacatcttatctagaagcttgtaatttttaaca
gctgcagtttcaactccagccaactctatggcccattcattactactactctggggacca
gaagcacatcttggatttactgcctgggtttgtataggaggcttatggacatttattgct
tttcatgggctcattggtctcatagcattttccctacgtcagtttgaaatagcacgatta
gttttaattagaccttataatgcacttgcattctcaggaccaatagcagtttatacatct
gtcttcctaatttatccacttggtcaatctagctggttctttgcaccaagctttggtatt
gctgctatcttccgattcctattattccttcaaggttttcataattggacattaaatcct
tttcacatgatgggtgtagctggaatattgggaggtgcattactatcagctatccatggt
gctactgtcattaacactatttatcaagatgctcgtgcatatacaacatttcgtgctttc
tcaccaagtcaaccagaagaaacttattcaatgcttacagctaatcgtttttggtctcag
atatttggtgtagccttctcaaacaagagatggcttcatttcttcatgctatttgtacct
ctagcaggtatgtggacatcttcaattggtatcattggattagcatttaatctaagagct
tatgattttatttctcaagaattgaaagcagcagaagatcctgaatttgaaacattttat
acaaagaacattctactcaatgaaggtatacgactttggatggctgtccaagatcaagct
catgaaaattatcagttcccagaagaggtattgccaagaggtaattctttgtga
>Mz8_psbD
atgaaactcatctattctacaagattcatggcaagtaaagcttatcttcttggatcactt
actctactagatgactggttaaaacgagatcgatttgtctttattggctggtcaggtcta
ttattatttcctacagcttacttagcagcaggtggatggtttacagctaccacttttgta
acatctttctttacacatggtttggccacatcttatctagaagcttgtaatttttaaca
gctgcagtttcaactccagccaactctatggcccattcattactactactctggggacca
gaagcacatcttggatttactgcctgggtttgtataggaggcttatggacatttattgct
tttcatgggctcattggtctcatagcattttccctacgtcagtttgaaatagcacgatta
gttttaattagaccttataatgcacttgcattctcaggaccaatagcagtttatacatct
gtcttcctaatttatccacttggtcaatctagctggttctttgcaccaagctttgggatt
gctgctatcttccgattcctattattccttcaaggttttcataattggacattaaatcct
tttcacatgatgggtgtagctggaatattgggaggtgcattactatcagctatccatggt
gctactgtcattaacactatttatcaagatgctcgtgcatatacaacatttcgtgctttc
tcaccaagtcaaccagaagaaacttattcaatgcttacagctaatcgtttttggtctcag
atatttggtgtagccttctcaaacaagagatggcttcatttcttcatgctatttgtacct
ctagcaggtatgtggacatcttcaattggtatcattggattagcatttaatctaagagct
tatgattttatttctcaagaattgaaagcagcagaagatcctgaatttgaaacattttat
acaaagaacattctactcaatgaaggtatacgactttggatggctgtccaagatcaagct
catgaaaattatcagttcccagaagaggtattgccaagaggtaattctttgtga
>Mz9_psbD
atgaaactcatctattctacaagattcatggcaagtaaagcttatcttcttggatcactt
actctactagatgactggttaaaacgagatcgatttgtctttattggctggtcaggtcta
ttattatttcctacagcttacttagcagtaggtggatggtttacagctaccacttttgta
acatctttctttacacatggtctggtcacatcttatctagaagcttgtaatttttaaca
gctgcagtttcaactccagccaactctatggcccattcattactactactctggggacca
gaagcacatcttggatttactgcctgggtttgtataggaggcttatggacatttattgct
tttcatgggctcattggtctcatagcattttccctacgtcagtttgaaatagcacgatta
gttttaattagaccttataatgcacttgcattctcaggaccaataggagtttatacatct
gtcttcctaatttatccacttggtcaatctagctggttctttgcaccaagctttgggatt
gctgctatcttccgattcctattattccttcaaggttttcataattggacattaaatcct
tttcacatgatgggtgtagctggaatattgggaggtgcattactatcagctatccatggt
gctactgtcattaacactatttatcaagatgctcgtgcatatacaacatttcgtgctttc
tcaccaagtcaaccagaagaaacttattcaatgcttacagctaatcgtttttggtctcag
atatttggtgtagccttctcaaacaagagatggcttcatttcttcatgctatttgtacct
ctagcaggtatgtggacatcttcaattggtatcattggattagcatttaatctaagagct
tatgattttatttctcaagaattgaaagcagcagaagatcctgaatttgaaacattttat
acaaagaacattctactcaatgaaggtatacgactttggatggctgtccaagatcaagct
catgaaaattatcagttcccagaagaggtattgccaagaggtaattctttgtga

>MzC1_psbD
atgaaactcatctattctacaagattcatggcaagtaaagcttatcttcttggatcactt
actctactagatgactggttaaaacgagatcgatttgtctttattggctggtcaggtcta
ttattatttcctacagcttacttagcagcaggtggatggtttacagccaccgcttttgta
acatctttctttacacatggtctggtcacatcttatctagaagcttgtaatttttaaca
gctgcagtttcaactccagccaactctatggcccattcattactactactctggggacca
gaagcacatcttggatttactgcctgggtttgtataggaggcttatggacatttattgct
tttcatgggctcattggtctcatagcattttccctacgtcagtttgaaatagcacgatta
gttttaattagaccttataatgcacttgcattctcaggaccaatagcagtttatacatct
gtcttcctaatttatccacttggtcaatctagctggttctttgcaccaagctttggtatt
gctgctatcttccgattcctattattccttcaaggttttcataattggacattaaatcct
tttcacatgatgggtgtagctggaatattgggaggtgccctactatcagctatccatggt
gctactgtcattaacactatttatcaagatgctcgtgcatatacaacatttcgtgctttc
tcaccaagtcaaccagaagaaacttattcaatgcttacagctaatcgtttttggtctcag
atatttggtgtagccttctcaaacaagagatggcttcatttcttcatgctatttgtacct
ctagcaggtatgtggacatcttcaattggtatcattggattagcatttaatctaagagct
tatgattttatttctcaagaattgaaagcagcagaagatcctgaatttgaaacattttat
acaaagaacattctactcaatgaaggtatacgactttggatggctgtccaagatcaagct
catgaaaattatcagttcccagaagaggtattgccaagaggtaattctttgtga
>MzC1GaII_psbD
atgaaactcatctattctacaagattcatggcaagtaaagcttatcttcttggatcactt
actctactagatgactggttaaaacgagatcgatttgtctttattggctggtcaggtcta
ttattatttcctacagcttacttagcagcaggtggatggtttacagccaccgcttttgta
acatctttctttacacatggtctggtcacatcttatctagaagcttgtaatttttaaca
gctgcagtttcaactccagccaactctatggcccattcattactactactctggggacca
gaagcacatcttggatttactgcctgggtttgtataggaggcttatggacatttattgct
tttcatgggctcattggtctcatagcattttccctacgtcagtttgaaatagcacgatta
gttttaattagaccttataatgcacttgcattctcaggaccaatagcagtttatacatct
gtcttcctaatttatccacttggtcaatctagctggttctttgcaccaagctttggtatt
gctgctatcttccgattcctattattccttcaaggttttcataattggacattaaatcct
tttcacatgatgggtgtagctggaatattgggaggtgccctactatcagctatccatggt
gctactgtcattaacactatttatcaagatgctcgtgcatatacaacatttcgtgctttc
tcaccaagtcaaccagaagaaacttattcaatgcttacagctaatcgtttttggtctcag
atatttggtgtagccttctcaaacaagagatggcttcatttcttcatgctatttgtacct
ctagcaggtatgtggacatcttcaattggtatcattggattagcatttaatctaagagct
tatgattttatttctcaagaattgaaagcagcagaagatcctgaatttgaaacattttat
acaaagaacattctactcaatgaaggtatacgactttggatggctgtccaagatcaagct
catgaaaattatcagttcccagaagaggtattgccaagaggtaattctttgtga
>MzC2_psbD
atgaaactcatctattctacaagattcatggcaagtaaagcttatcttcttggatcactt
actctactagatgactggttaaaacgagatcgatttgtctttattggctggtcaggtcta
ttattatttcctacagcttacttagcagcaggtggatggtttacagccaccgcttttgta
acatctttctttacacatggtctggtcacatcttatctagaagcttgtaatttttaaca
gctgcagtttcaactccagccaactctatggcccattcattactactactctggggacca
gaagcacatcttggatttactgcctgggtttgtataggaggcttatggacatttattgct
tttcatgggctcattggtctcatagcattttccctacgtcagtttgaaatagcacgatta
gttttaattagaccttataatgcacttgcattctcaggaccaatagcagtttatacatct
gtcttcctaatttatccacttggtcaatctagctggttctttgcaccaagctttggtatt
gctgctatcttccgattcctattattccttcaaggttttcataattggacattaaatcct
tttcacatgatgggtgtagctggaatattgggaggtgccctactatcagctatccatggt
gctactgtcattaacactatttatcaagatgctcgtgcatatacaacatttcgtgctttc
tcaccaagtcaaccagaagaaacttattcaatgcttacagctaatcgtttttggtctcag
atatttggtgtagccttctcaaacaagagatggcttcatttcttcatgctatttgtacct
ctagcaggtatgtggacatcttcaattggtatcattggattagcatttaatctaagagct
tatgattttatttctcaagaattgaaagcagcagaagatcctgaatttgaaacattttat
acaaagaacattctactcaatgaaggtatacgactttggatggctgtccaagatcaagct
catgaaaattatcagttcccagaagaggtattgccaagaggtaattctttgtga

>MzC2GaII_psbD
atgaaactcatctattctacaagattcatggcaagtaaagcttatcttcttggatcactt
actctactagatgactggttaaaacgagatcgatttgtctttattggctggtcaggtcta
ttattatttcctacagcttacttagcagcaggtggatggtttacagccaccgcttttgta
acatctttctttacacatggtctggtcacatcttatctagaagcttgtaattttttaaca
gctgcagtttcaactccagccaactctatggcccattcattactactactctggggacca
gaagcacatcttggatttactgcctgggtttgtataggaggcttatggacatttattgct
tttcatgggctcattggtctcatagcattttccctacgtcagtttgaaatagcacgatta
gttttaattagaccttataatgcacttgcattctcaggaccaatagcagtttatacatct
gtcttcctaatttatccacttggtcaatctagctggttctttgcaccaagctttggtatt
gctgctatcttccgattcctattattccttcaaggttttcataattggacattaaatcct
tttcacatgatgggtgtagctggaatattgggaggtgccctactatcagctatccatggt
gctactgtcattaacactatttatcaagatgctcgtgcatatacaacatttcgtgctttc
tcaccaagtcaaccagaagaaacttattcaatgcttacagctaatcgtttttggtctcag
atatttggtgtagccttctcaaacaagagatggcttcatttcttcatgctatttgtacct
ctagcaggtatgtggacatcttcaattggtatcattggattagcatttaatctaagagct
tatgattttatttctcaagaattgaaagcagcagaagatcctgaatttgaaacattttat
acaaagaacattctactcaatgaaggtatacgactttggatggctgtccaagatcaagct
catgaaaattatcagttcccagaagaggtattgccaagaggtaattctttgtga
>MzC3_psbD
atgaaactcatctattctacaagattcatggcaagtaaagcttatcttcttggatcactt
actctactagatgactggttaaaacgagatcgatttgtctttattggctggtcaggtcta
ttattatttcctacagcttacttagcagcaggtggatggtttacagccaccgcttttgta
acatctttctttacacatggtctggtcacatcttatctagaagcttgtaattttttaaca
gctgcagtttcaactccagccaactctatggcccattcattactactactctggggacca
gaagcacatcttggatttactgcctgggtttgtataggaggcttatggacatttattgct
tttcatgggctcattggtctcatagcattttccctacgtcagtttgaaatagcacgatta
gttttaattagaccttataatgcacttgcattctcaggaccaatagcagtttatacatct
gtcttcctaatttatccacttggtcaatctagctggttctttgcaccaagctttggtatt
gctgctatcttccgattcctattattccttcaaggttttcataattggacattaaatcct
tttcacatgatgggtgtagctggaatattgggaggtgccctactatcagctatccatggt
gctactgtcattaacactatttatcaagatgctcgtgcatatacaacatttcgtgctttc
tcaccaagtcaaccagaagaaacttattcaatgcttacagctaatcgtttttggtctcag
atatttggtgtagccttctcaaacaagagatggcttcatttcttcatgctatttgtacct
ctagcaggtatgtggacatcttcaattggtatcattggattagcatttaatctaagagct
tatgattttatttctcaagaattgaaagcagcagaagatcctgaatttgaaacattttat
acaaagaacattctactcaatgaaggtatacgactttggatggctgtccaagatcaagct
catgaaaattatcagttcccagaagaggtattgccaagaggtaattctttgtga
>MzC4_psbD
atgaaactcatctattctacaagattcatggcaagtaaagcttatcttcttggatcactt
actctactagatgactggttaaaacgagatcgatttgtctttattggctggtcaggtcta
ttattatttcctacagcttacttagcagcaggtggatggtttacagccaccgcttttgta
acatctttctttacacatggtctggtcacatcttatctagaagcttgtaattttttaaca
gctgcagtttcaactccagccaactctatggcccattcattactactactctggggacca
gaagcacatcttggatttactgcctgggtttgtataggaggcttatggacatttattgct
tttcatgggctcattggtctcatagcattttccctacgtcagtttgaaatagcacgatta
gttttaattagaccttataatgcacttgcattctcaggaccaatagcagtttatacatct
gtcttcctaatttatccacttggtcaatctagctggttctttgcaccaagctttggtatt
gctgctatcttccgattcctattattccttcaaggttttcataattggacattaaatcct
tttcacatgatgggtgtagctggaatattgggaggtgccctactatcagctatccatggt
gctactgtcattaacactatttatcaagatgctcgtgcatatacaacatttcgtgctttc
tcaccaagtcaaccagaagaaacttattcaatgcttacagctaatcgtttttggtctcag
atatttggtgtagccttctcaaacaagagatggcttcatttcttcatgctatttgtacct
ctagcaggtatgtggacatcttcaattggtatcattggattagcatttaatctaagagct
tatgattttatttctcaagaattgaaagcagcagaagatcctgaatttgaaacattttat
acaaagaacattctactcaatgaaggtatacgactttggatggctgtccaagatcaagct
catgaaaattatcagttcccagaagaggtattgccaagaggtaattctttgtga

>Od10_psbD
atgaaactcatctattctacaagattcatggcaagtaaagcttatcttcttggatcactt
actctactagatgactggttaaaacgagatcgatttgtctttattggctggtcaggtcta
ttattatttcctacagcttacttagcagcaggtggatggtttacagccaccgcttttgta
acatctttctttacacatggtctggtcacatcttatctagaagcttgtaatttttaaca
gctgcagtttcaactccagccaactctatggcccattcattactactactctggggacca
gaagcacatcttggatttactgcctgggtttgtataggaggcttatggacatttattgct
tttcatgggctcattggtctcatagcattttccctacgtcagtttgaaatagcacgatta
gttttaattagaccttataatgcacttgcattctcaggaccaatagcagtttatacatct
gtcttcctaatttatccacttggtcaatctagctggttctttgcaccaagctttggtatt
gctgctatcttccgattcctattattccttcaaggttttcataattggacattaaatcct
tttcacatgatgggtgtagctggaatattgggaggtgccctactatcagctatccatggt
gctactgtcattaacactatttatcaagatgctcgtgcatatacaacatttcgtgctttc
tcaccaagtcaaccagaagaaacttattcaatgcttacagctaatcgtttttggtctcag
atatttggtgtagccttctcaaacaagagatggcttcatttcttcatgctatttgtacct
ctagcaggtatgtggacatcttcaattggtatcattggattagcatttaatctaagagct
tatgattttatttctcaagaattgaaagcagcagaagatcctgaatttgaaacattttat
acaaagaacattctactcaatgaaggtatacgactttggatggctgtccaagatcaagct
catgaaaattatcagttcccagaagaggtattgccaagaggtaattctttgtga
>Od11_psbD
atgaaactcatctattctacaagattcatggcaagtaaagcttatcttcttggatcactt
actctactagatgactggttaaaacgagatcgatttgtctttattggctggtcaggtcta
ttattatttcctacagcttacttagcagcaggtggatggtttacagccaccgcttttgta
acatctttctttacacatggtctggtcacatcttatctagaagcttgtaatttttaaca
gctgcagtttcaactccagccaactctatggcccattcattactactactctggggacca
gaagcacatcttggatttactgcctgggtttgtataggaggcttatggacatttattgct
tttcatgggctcattggtctcatagcattttccctacgtcagtttgaaatagcacgatta
gttttaattagaccttataatgcacttgcattctcaggaccaatagcagtttatacatct
gtcttcctaatttatccacttggtcaatctagctggttctttgcaccaagctttggtatt
gctgctatcttccgattcctattattccttcaaggttttcataattggacattaaatcct
tttcacatgatgggtgtagctggaatattgggaggtgccctactatcagctatccatggt
gctactgtcattaacactatttatcaagatgctcgtgcatatacaacatttcgtgctttc
tcaccaagtcaaccagaagaaacttattcaatgcttacagctaatcgtttttggtctcag
atatttggtgtagccttctcaaacaagagatggcttcatttcttcatgctatttgtacct
ctagcaggtatgtggacatcttcaattggtatcattggattagcatttaatctaagagct
tatgattttatttctcaagaattgaaagcagcagaagatcctgaatttgaaacattttat
acaaagaacattctactcaatgaaggtatacgactttggatggctgtccaagatcaagct
catgaaaattatcagttcccagaagaggtattgccaagaggtaattctttgtga
>Od12_psbD
atgaaactcatctattctacaagattcatggcaagtaaagcttatcttcttggatcactt
actctactagatgactggttaaaacgagatcgatttgtctttattggctggtcaggtcta
ttattatttcctacagcttacttagcagcaggtggatggtttacagccaccgcttttgta
acatctttctttacacatggtctggtcacatcttatctagaagcttgtaatttttaaca
gctgcagtttcaactccagccaactctatggcccattcattactactactctggggacca
gaagcacatcttggatttactgcctgggtttgtataggaggcttatggacatttattgct
tttcatgggctcattggtctcatagcattttccctacgtcagtttgaaatagcacgatta
gttttaattagaccttataatgcacttgcattctcaggaccaatagcagtttatacatct
gtcttcctaatttatccacttggtcaatctagctggttctttgcaccaagctttggtatt
gctgctatcttccgattcctattattccttcaaggttttcataattggacattaaatcct
tttcacatgatgggtgtagctggaatattgggaggtgccctactatcagctatccatggt
gctactgtcattaacactatttatcaagatgctcgtgcatatacaacatttcgtgctttc
tcaccaagtcaaccagaagaaacttattcaatgcttacagctaatcgtttttggtctcag
atatttggtgtagccttctcaaacaagagatggcttcatttcttcatgctatttgtacct
ctagcaggtatgtggacatcttcaattggtatcattggattagcatttaatctaagagct
tatgattttatttctcaagaattgaaagcagcagaagatcctgaatttgaaacattttat
acaaagaacattctactcaatgaaggtatacgactttggatggctgtccaagatcaagct
catgaaaattatcagttcccagaagaggtattgccaagaggtaattctttgtga

>Od13_psbD
atgaaactcatctattctacaagattcatggcaagtaaagcttatcttcttggatcactt
actctactagatgactggttaaaacgagatcgatttgtctttattggctggtcaggtcta
ttattatttcctacagcttacttagcagcaggtggatggtttacagccaccgcttttgta
acatctttctttacacatggtctggtcacatcttatctagaagcttgtaattttttaaca
gctgcagtttcaactccagccaactctatggcccattcattactactactctggggacca
gaagcacatcttggatttactgcctgggtttgtataggaggcttatggacatttattgct
tttcatgggctcattggtctcatagcattttccctacgtcagtttgaaatagcacgatta
gttttaattagaccttataatgcacttgcattctcaggaccaatagcagtttatacatct
gtcttcctaatttatccacttggtcaatctagctggttctttgcaccaagctttggtatt
gctgctatcttccgattcctattattccttcaaggttttcataattggacattaaatcct
tttcacatgatgggtgtagctggaatattgggaggtgccctactatcagctatccatggt
gctactgtcattaacactatttatcaagatgctcgtgcatatacaacatttcgtgctttc
tcaccaagtcaaccagaagaaacttattcaatgcttacagctaatcgtttttggtctcag
atatttggtgtagccttctcaaacaagagatggcttcatttcttcatgctatttgtacct
ctagcaggtatgtggacatcttcaattggtatcattggattagcatttaatctaagagct
tatgattttatttctcaagaattgaaagcagcagaagatcctgaatttgaaacattttat
acaaagaacattctactcaatgaaggtatacgactttggatggctgtccaagatcaagct
catgaaaattatcagttcccagaagaggtattgccaagaggtaattctttgtga
>Od14_psbD
atgaaactcatctattctacaagattcatggcaagtaaagcttatcttcttggatcactt
actctactagatgactggttaaaacgagatcgatttgtctttattggctggtcaggtcta
ttattatttcctacagcttacttagcagcaggtggatggtttacagccaccgcttttgta
acatctttctttacacatggtctggtcacatcttatctagaagcttgtaattttttaaca
gctgcagtttcaactccagccaactctatggcccattcattactactactctggggacca
gaagcacatcttggatttactgcctgggtttgtataggaggcttatggacatttattgct
tttcatgggctcattggtctcatagcattttccctacgtcagtttgaaatagcacgatta
gttttaattagaccttataatgcacttgcattctcaggaccaatagcagtttatacatct
gtcttcctaatttatccacttggtcaatctagctggttctttgcaccaagctttggtatt
gctgctatcttccgattcctattattccttcaaggttttcataattggacattaaatcct
tttcacatgatgggtgtagctggaatattgggaggtgccctactatcagctatccatggt
gctactgtcattaacactatttatcaagatgctcgtgcatatacaacatttcgtgctttc
tcaccaagtcaaccagaagaaacttattcaatgcttacagctaatcgtttttggtctcag
atatttggtgtagccttctcaaacaagagatggcttcatttcttcatgctatttgtacct
ctagcaggtatgtggacatcttcaattggtatcattggattagcatttaatctaagagct
tatgattttatttctcaagaattgaaagcagcagaagatcctgaatttgaaacattttat
acaaagaacattctactcaatgaaggtatacgactttggatggctgtccaagatcaagct
catgaaaattatcagttcccagaagaggtattgccaagaggtaattctttgtga
>Od15_psbD
atgaaactcatctattctacaagattcatggcaagtaaagcttatcttcttggatcactt
actctactagatgactggttaaaacgagatcgatttgtctttattggctggtcaggtcta
ttattatttcctacagcttacttagcagcaggtggatggtttacagccaccgcttttgta
acatctttctttacacatggtctggtcacatcttatctagaagcttgtaattttttaaca
gctgcagtttcaactccagccaactctatggcccattcattactactactctggggacca
gaagcacatcttggatttactgcctgggtttgtataggaggcttatggacatttattgct
tttcatgggctcattggtctcatagcattttccctacgtcagtttgaaatagcacgatta
gttttaattagaccttataatgcacttgcattctcaggaccaatagcagtttatacatct
gtcttcctaatttatccacttggtcaatctagctggttctttgcaccaagctttggtatt
gctgctatcttccgattcctattattccttcaaggttttcataattggacattaaatcct
tttcacatgatgggtgtagctggaatattgggaggtgccctactatcagctatccatggt
gctactgtcattaacactatttatcaagatgctcgtgcatatacaacatttcgtgctttc
tcaccaagtcaaccagaagaaacttattcaatgcttacagctaatcgtttttggtctcag
atatttggtgtagccttctcaaacaagagatggcttcatttcttcatgctatttgtacct
ctagcaggtatgtggacatcttcaattggtatcattggattagcatttaatctaagagct
tatgattttatttctcaagaattgaaagcagcagaagatcctgaatttgaaacattttat
acaaagaacattctactcaatgaaggtatacgactttggatggctgtccaagatcaagct
catgaaaattatcagttcccagaagaggtattgccaagaggtaattctttgtga

>Od18_psbD
atgaaactcatctattctacaagattcatggcaagtaaagcttatcttcttggatcactt
actctactagatgactggttaaaacgagatcgatttgtctttattggctggtcaggtcta
ttattatttcctacagcttacttagcagcaggtggatggtttacagccaccgcttttgta
acatctttctttacacatggtctggtcacatcttatctagaagcttgtaatttttaaca
gctgcagtttcaactccagccaactctatggcccattcattactactactctggggacca
gaagcacatcttggatttactgcctgggtttgtataggaggcttatggacatttattgct
tttcatgggctcattggtctcatagcattttccctacgtcagtttgaaatagcacgatta
gttttaattagaccttataatgcacttgcattctcaggaccaatagcagtttatacatct
gtcttcctaatttatccacttggtcaatctagctggttctttgcaccaagctttggtatt
gctgctatcttccgattcctattattccttcaaggttttcataattggacattaaatcct
tttcacatgatgggtgtagctggaatattgggaggtgccctactatcagctatccatggt
gctactgtcattaacactatttatcaagatgctcgtgcatatacaacatttcgtgctttc
tcaccaagtcaaccagaagaaacttattcaatgcttacagctaatcgtttttggtctcag
atatttggtgtagccttctcaaacaagagatggcttcatttcttcatgctatttgtacct
ctagcaggtatgtggacatcttcaattggtatcattggattagcatttaatctaagagct
tatgattttatttctcaagaattgaaagcagcagaagatcctgaatttgaaacattttat
acaaagaacattctactcaatgaaggtatacgactttggatggctgtccaagatcaagct
catgaaaattatcagttcccagaagaggtattgccaagaggtaattctttgtga
>Od19_psbD
atgaaactcatctattctacaagattcatggcaagtaaagcttatcttcttggatcactt
actctactagatgactggttaaaacgagatcgatttgtctttattggctggtcaggtcta
ttattatttcctacagcttacttagcagcaggtggatggtttacagccaccgcttttgta
acatctttctttacacatggtctggtcacatcttatctagaagcttgtaatttttaaca
gctgcagtttcaactccagccaactctatggcccattcattactactactctggggacca
gaagcacatcttggatttactgcctgggtttgtataggaggcttatggacatttattgct
tttcatgggctcattggtctcatagcattttccctacgtcagtttgaaatagcacgatta
gttttaattagaccttataatgcacttgcattctcaggaccaatagcagtttatacatct
gtcttcctaatttatccacttggtcaatctagctggttctttgcaccaagctttggtatt
gctgctatcttccgattcctattattccttcaaggttttcataattggacattaaatcct
tttcacatgatgggtgtagctggaatattgggaggtgccctactatcagctatccatggt
gctactgtcattaacactatttatcaagatgctcgtgcatatacaacatttcgtgctttc
tcaccaagtcaaccagaagaaacttattcaatgcttacagctaatcgtttttggtctcag
atatttggtgtagccttctcaaacaagagatggcttcatttcttcatgctatttgtacct
ctagcaggtatgtggacatcttcaattggtatcattggattagcatttaatctaagagct
tatgattttatttctcaagaattgaaagcagcagaagatcctgaatttgaaacattttat
acaaagaacattctactcaatgaaggtatacgactttggatggctgtccaagatcaagct
catgaaaattatcagttcccagaagaggtattgccaagaggtaattctttgtga
>Od2_psbD
atgaaactcatctattctacaagattcatggcaagtaaagcttatcttcttggatcactt
actctactagatgactggttaaaacgagatcgatttgtctttattggctggtcaggtcta
ttattatttcctacagcttacttagcagcaggtggatggtttacagccaccgcttttgta
acatctttctttacacatggtctggtcacatcttatctagaagcttgtaatttttaaca
gctgcagtttcaactccagccaactctatggcccattcattactactactctggggacca
gaagcacatcttggatttactgcctgggtttgtataggaggcttatggacatttattgct
tttcatgggctcattggtctcatagcattttccctacgtcagtttgaaatagcacgatta
gttttaattagaccttataatgcacttgcattctcaggaccaatagcagtttatacatct
gtcttcctaatttatccacttggtcaatctagctggttctttgcaccaagctttggtatt
gctgctatcttccgattcctattattccttcaaggttttcataattggacattaaatcct
tttcacatgatgggtgtagctggaatattgggaggtgccctactatcagctatccatggt
gctactgtcattaacactatttatcaagatgctcgtgcatatacaacatttcgtgctttc
tcaccaagtcaaccagaagaaacttattcaatgcttacagctaatcgtttttggtctcag
atatttggtgtagccttctcaaacaagagatggcttcatttcttcatgctatttgtacct
ctagcaggtatgtggacatcttcaattggtatcattggattagcatttaatctaagagct
tatgattttatttctcaagaattgaaagcagcagaagatcctgaatttgaaacattttat
acaaagaacattctactcaatgaaggtatacgactttggatggctgtccaagatcaagct
catgaaaattatcagttcccagaagaggtattgccaagaggtaattctttgtga

>Od22_psbD
atgaaactcatctattctacaagattcatggcaagtaaagcttatcttcttggatcactt
actctactagatgactggttaaaacgagatcgatttgtctttattggctggtcaggtcta
ttattatttcctacagcttacttagcagcaggtggatggtttacagccaccgcttttgta
acatctttctttacacatggtctggtcacatcttatctagaagcttgtaatttttaaca
gctgcagtttcaactccagccaactctatggcccattcattactactactctggggacca
gaagcacatcttggatttactgcctgggtttgtataggaggcttatggacatttattgct
tttcatgggctcattggtctcataggattttccctacgtcagtttgaaatagcacgatta
gttttaattagaccttataatgcacttgcattctcaggaccaatagcagtttatacatct
gtcttcctaatttatccacttggtcaatctagctggttctttgcaccaagctttggtatt
gctgctatcttccgattcctattattccttcaaggttttcataattggacattaaatcct
tttcacatgatgggtgtagctggaatattgggaggtgccctactatcagctatccatggt
gctactgtcattaacactatttatcaagatgctcgtgcatatacaacatttcgtgctttc
tcaccaagtcaaccagaagaaacttattcaatgcttacagctaatcgtttttggtctcag
atatttggtgtagccttctcaaacaagagatggcttcatttcttcatgctatttgtacct
ctagcaggtatgtggacatcttcaattggtatcattggattagcatttaatctaagagct
tatgattttatttctcaagaattgaaagcagcagaagatcctgaatttgaaacattttat
acaaagaacattctactcaatgaaggtatacgactttggatggctgtccaagatcaagct
catgaaaattatcagttcccagaagaggtattgccaagaggtaattctttgtga
>Od23_psbD
atgaaactcatctattctacaagattcatggcaagtaaagcttatcttcttggatcactt
actctactagatgactggttaaaacgagatcgatttgtctttattggctggtcaggtcta
ttattatttcctacagcttacttagcagcaggtggatggtttacagccaccgcttttgta
acatctttctttacacatggtctggtcacatcttatctagaagcttgtaatttttaaca
gctgcagtttcaactccagccaactctatggcccattcattactactactctggggacca
gaagcacatcttggatttactgcctgggtttgtataggaggcttatggacatttattgct
tttcatgggctcattggtctcatagcattttccctacgtcagtttgaaatagcacgatta
gttttaattagaccttataatgcacttgcattctcaggaccaatagcagtttatacatct
gtcttcctaatttatccacttggtcaatctagctggttctttgcaccaagctttggtatt
gctgctatcttccgattcctattattccttcaaggttttcataattggacattaaatcct
tttcacatgatgggtgtagctggaatattgggaggtgccctactatcagctatccatggt
gctactgtcattaacactatttatcaagatgctcgtgcatatacaacatttcgtgctttc
tcaccaagtcaaccagaagaaacttattcaatgcttacagctaatcgtttttggtctcag
atatttggtgtagccttctcaaacaagagatggcttcatttcttcatgctatttgtacct
ctagcaggtatgtggacatcttcaattggtatcattggattagcatttaatctaagagct
tatgattttatttctcaagaattgaaagcagcagaagatcctgaatttgaaacattttat
acaaagaacattctactcaatgaaggtatacgactttggatggctgtccaagatcaagct
catgaaaattatcagttcccagaagaggtattgccaagaggtaattctttgtga
>Od24_psbD
atgaaactcatctattctacaagattcatggcaagtaaagcttatcttcttggatcactt
actctactagatgactggttaaaacgagatcgatttgtctttattggctggtcaggtcta
ttattatttcctacagcttacttagcagcaggtggatggtttacagccaccgcttttgta
acatctttctttacacatggtctggtcacatcttatctagaagcttgtaatttttaaca
gctgcagtttcaactccagccaactctatggcccattcattactactactctggggacca
gaagcacatcttggatttactgcctgggtttgtataggaggcttatggacatttattgct
tttcatgggctcattggtctcatagcattttccctacgtcagtttgaaatagcacgatta
gttttaattagaccttataatgcacttgcattctcaggaccaatagcagtttatacatct
gtcttcctaatttatccacttggtcaatctagctggttctttgcaccaagctttggtatt
gctgctatcttccgattcctattattccttcaaggttttcataattggacattaaatcct
tttcacatgatgggtgtagctggaatattgggaggtgcattactatcagctatccatggt
gctactgtcattaacactatttatcaagatgctcgtgcatatacaacatttcgtgctttc
tcaccaagtcaaccagaagaaacttattcaatgcttacagctaatcgcttctggtctcaa
atatttggtgtagccttctcaaacaagagatggcttcatttcttcatgctatttgtacct
ctagcaggtatgtggacatcttcaattggtatcattggattagcatttaatctaagagct
tatgattttatttctcaagaattgaaagcagcagaagatcctgaatttgaaacattttat
acaaagaacattctactcaatgaaggtatacgactttggatggctgtccaagatcaagct
catgaaaattatcagttcccagaagaggtattgccaagaggtaattctttgtga

>Od25_psbD
atgaaactcatctattctacaagattcatggcaagtaaagcttatcttcttggatcactt
actctactagatgactggttaaaacgagatcgatttgtctttattggctggtcaggtcta
ttattatttcctacagcttacttagcagcaggtggatggtttacagccaccgcttttgta
acatctttctttacacatggtctggtcacatcttatctagaagcttgtaatttttaaca
gctgcagtttcaactccagccaactctatggcccattcattactactactctggggacca
gaagcacatcttggatttactgcctgggtttgtataggaggcttatggacatttattgct
tttcatgggctcattggtctcatagcattttccctacgtcagtttgaaatagcacgatta
gttttaattagaccttataatgcacttgcattctcaggaccaatagcagtttatacatct
gtcttcctaatttatccacttggtcaatctagctggttctttgcaccaagctttggtatt
gctgctatcttccgattcctattattccttcaaggttttcataattggacattaaatcct
tttcacatgatgggtgtagctggaatattgggaggtgccctactatcagctatccatggt
gctactgtcattaacactatttatcaagatgctcgtgcatatacaacatttcgtgctttc
tcaccaagtcaaccagaagaaacttattcaatgcttacagctaatcgtttttggtctcag
atatttggtgtagccttctcaaacaagagatggcttcatttcttcatgctatttgtacct
ctagcaggtatgtggacatcttcaattggtatcattggattagcatttaatctaagagct
tatgattttatttctcaagaattgaaagcagcagaagatcctgaatttgaaacattttat
acaaagaacattctactcaatgaaggtatacgactttggatggctgtccaagatcaagct
catgaaaattatcagttcccagaagaggtattgccaagaggtaattctttgtga
>Od26_psbD
atgaaactcatctattctacaagattcatggcaagtaaagcttatcttcttggatcactt
actctactagatgactggttaaaacgagatcgatttgtctttattggctggtcaggtcta
ttattatttcctacagcttacttagcagcaggtggatggtttacagccaccgcttttgta
acatctttctttacacatggtctggtcacatcttatctagaagcttgtaatttttaaca
gctgcagtttcaactccagccaactctatggcccattcattactactactctggggacca
gaagcacatcttggatttactgcctgggtttgtataggaggcttatggacatttattgct
tttcatgggctcattggtctcatagcattttccctacgtcagtttgaaatagcacgatta
gttttaattagaccttataatgcacttgcattctcaggaccaatagcagtttatacatct
gtcttcctaatttatccacttggtcaatctagctggttctttgcaccaagctttggtatt
gctgctatcttccgattcctattattccttcaaggttttcataattggacattaaatcct
tttcacatgatgggtgtagctggaatattgggaggtgccctactatcagctatccatggt
gctactgtcattaacactatttatcaagatgctcgtgcatatacaacatttcgtgctttc
tcaccaagtcaaccagaagaaacttattcaatgcttacagctaatcgtttttggtctcag
atatttggtgtagccttctcaaacaagagatggcttcatttcttcatgctatttgtacct
ctagcaggtatgtggacatcttcaattggtatcattggattagcatttaatctaagagct
tatgattttatttctcaagaattgaaagcagcagaagatcctgaatttgaaacattttat
acaaagaacattctactcaatgaaggtatacgactttggatggctgtccaagatcaagct
catgaaaattatcagttcccagaagaggtattgccaagaggtaattctttgtga
>Od27_psbD
atgaaactcatctattctacaagattcatggcaagtaaagcttatcttcttggatcactt
actctactagatgactggttaaaacgagatcgatttgtctttattggctggtcaggtcta
ttattatttcctacagcttacttagcagcaggtggatggtttacagccaccgcttttgta
acatctttctttacacatggtctggtcacatcttatctagaagcttgtaatttttaaca
gctgcagtttcaactccagccaactctatggcccattcattactactactctggggacca
gaagcacatcttggatttactgcctgggtttgtataggaggcttatggacatttattgct
tttcatgggctcattggtctcatagcattttccctacgtcagtttgaaatagcacgatta
gttttaattagaccttataatgcacttgcattctcaggaccaatagcagtttatacatct
gtcttcctaatttatccacttggtcaatctagctggttctttgcaccaagctttggtatt
gctgctatcttccgattcctattattccttcaaggttttcataattggacattaaatcct
tttcacatgatgggtgtagctggaatattgggaggtgccctactatcagctatccatggt
gctactgtcattaacactatttatcaagatgctcgtgcatatacaacatttcgtgctttc
tcaccaagtcaaccagaagaaacttattcaatgcttacagctaatcgtttttggtctcag
atatttggtgtagccttctcaaacaagagatggcttcatttcttcatgctatttgtacct
ctagcaggtatgtggacatcttcaattggtatcattggattagcatttaatctaagagct
tatgattttatttctcaagaattgaaagcagcagaagatcctgaatttgaaacattttat
acaaagaacattctactcaatgaaggtatacgactttggatggctgtccaagatcaagct
catgaaaattatcagttcccagaagaggtattgccaagaggtaattctttgtga

>Od4_psbD
atgaaactcatctattctacaagattcatggcaagtaaagcttatcttcttggatcactt
actctactagatgactggttaaaacgagatcgatttgtctttattggctggtcaggtcta
ttattatttcctacagcttacttagcagcaggtggatggtttacagccaccgcttttgta
acatctttctttacacatggtctggtcacatcttatctagaagcttgtaattttttaaca
gctgcagtttcaactccagccaactctatggcccattcattactactactctggggacca
gaagcacatcttggatttactgcctgggtttgtataggaggcttatggacatttattgct
tttcatgggctcattggtctcatagcattttccctacgtcagtttgaaatagcacgatta
gttttaattagaccttataatgcacttgcattctcaggaccaatagcagtttatacatct
gtcttcctaatttatccacttggtcaatctagctggttctttgcaccaagctttggtatt
gctgctatcttccgattcctattattccttcaaggttttcataattggacattaaatcct
tttcacatgatgggtgtagctggaatattgggaggtgccctactatcagctatccatggt
gctactgtcattaacactatttatcaagatgctcgtgcatatacaacatttcgtgctttc
tcaccaagtcaaccagaagaaacttattcaatgcttacagctaatcgtttttggtctcag
atatttggtgtagccttctcaaacaagagatggcttcatttcttcatgctatttgtacct
ctagcaggtatgtggacatcttcaattggtatcattggattagcatttaatctaagagct
tatgattttatttctcaagaattgaaagcagcagaagatcctgaatttgaaacattttat
acaaagaacattctactcaatgaaggtatacgactttggatggctgtccaagatcaagct
catgaaaattatcagttcccagaagaggtattgccaagaggtaattctttgtga
>Od6_psbD
atgaaactcatctattctacaagattcatggcaagtaaagcttatcttcttggatcactt
actctactagatgactggttaaaacgagatcgatttgtctttattggctggtcaggtcta
ttattatttcctacagcttacttagcagcaggtggatggtttacagccaccgcttttgta
acatctttctttacacatggtctggtcacatcttatctagaagcttgtaattttttaaca
gctgcagtttcaactccagccaactctatggcccattcattactactactctggggacca
gaagcacatcttggatttactgcctgggtttgtataggaggcttatggacatttattgct
tttcatgggctcattggtctcatagcattttccctacgtcagtttgaaatagcacgatta
gttttaattagaccttataatgcacttgcattctcaggaccaatagcagtttatacatct
gtcttcctaatttatccacttggtcaatctagctggttctttgcaccaagctttggtatt
gctgctatcttccgattcctattattccttcaaggttttcataattggacattaaatcct
tttcacatgatgggtgtagctggaatattgggaggtgccctactatcagctatccatggt
gctactgtcattaacactatttatcaagatgctcgtgcatatacaacatttcgtgctttc
tcaccaagtcaaccagaagaaacttattcaatgcttacagctaatcgtttttggtctcag
atatttggtgtagccttctcaaacaagagatggcttcatttcttcatgctatttgtacct
ctagcaggtatgtggacatcttcaattggtatcattggattagcatttaatctaagagct
tatgattttatttctcaagaattgaaagcagcagaagatcctgaatttgaaacattttat
acaaagaacattctactcaatgaaggtatacgactttggatggctgtccaagatcaagct
catgaaaattatcagttcccagaagaggtattgccaagaggtaattctttgtga
>Od8_psbD
atgaaactcatctattctacaagattcatggcaagtaaagcttatcttcttggatcactt
actctactagatgactggttaaaacgagatcgatttgtctttattggctggtcaggtcta
ttattatttcctacagcttacttagcagcaggtggatggtttacagccaccgcttttgta
acatctttctttacacatggtctggtcacatcttatctagaagcttgtaattttttaaca
gctgcagtttcaactccagccaactctatggcccattcattactactactctggggacca
gaagcacatcttggatttactgcctgggtttgtataggaggcttatggacatttattgct
tttcatgggctcattggtctcatagcattttccctacgtcagtttgaaatagcacgatta
gttttaattagaccttataatgcacttgcattctcaggaccaatagcagtttatacatct
gtcttcctaatttatccacttggtcaatctagctggttctttgcaccaagctttggtatt
gctgctatcttccgattcctattattccttcaaggttttcataattggacattaaatcct
tttcacatgatgggtgtagctggaatattgggaggtgccctactatcagctatccatggt
gctactgtcattaacactatttatcaagatgctcgtgcatatacaacatttcgtgctttc
tcaccaagtcaaccagaagaaacttattcaatgcttacagctaatcgtttttggtctcag
atatttggtgtagccttctcaaacaagagatggcttcatttcttcatgctatttgtacct
ctagcaggtatgtggacatcttcaattggtatcattggattagcatttaatctaagagct
tatgattttatttctcaagaattgaaagcagcagaagatcctgaatttgaaacattttat
acaaagaacattctactcaatgaaggtatacgactttggatggctgtccaagatcaagct
catgaaaattatcagttcccagaagaggtattgccaagaggtaattctttgtga

>Od9_psbD
atgaaactcatctattctacaagattcatggcaagtaaagcttatcttcttggatcactt
actctactagatgactggttaaaacgagatcgatttgtctttattggctggtcaggtcta
ttattatttcctacagcttacttagcagcaggtggatggtttacagccaccgcttttgta
acatctttctttacacatggtctggtcacatcttatctagaagcttgtaatttttaaca
gctgcagtttcaactccagccaactctatggcccattcattactactactctggggacca
gaagcacatcttggatttactgcctgggtttgtataggaggcttatggacatttattgct
tttcatgggctcattggtctcatagcattttccctacgtcagtttgaaatagcacgatta
gttttaattagaccttataatgcacttgcattctcaggaccaatagcagtttatacatct
gtcttcctaatttatccacttggtcaatctagctggttctttgcaccaagctttggtatt
gctgctatcttccgattcctattattccttcaaggttttcataattggacattaaatcct
tttcacatgatgggtgtagctggaatattgggaggtgccctactatcagctatccatggt
gctactgtcattaacactatttatcaagatgctcgtgcatatacaacatttcgtgctttc
tcaccaagtcaaccagaagaaacttattcaatgcttacagctaatcgtttttggtctcag
atatttggtgtagccttctcaaacaagagatggcttcatttcttcatgctatttgtacct
ctagcaggtatgtggacatcttcaattggtatcattggattagcatttaatctaagagct
tatgattttatttctcaagaattgaaagcagcagaagatcctgaatttgaaacattttat
acaaagaacattctactcaatgaaggtatacgactttggatggctgtccaagatcaagct
catgaaaattatcagttcccagaagaggtattgccaagaggtaattctttgtga
>Ss5_psbD
atgaaactcatctattctacaagattcatggcaagtaaagcttatcttcttggatcactt
actctactagatgactggttaaaacgagatcgatttgtctttattggctggtcaggtcta
ttattatttcctacagcttacttagcagcaggtggatggtttacagccaccgcttttgta
acatctttctttacacatggtctggtcacatcttatctagaagcttgtaatttttaaca
gctgcagtttcaactccagccaactctatggcccattcattactactactctggggacca
gaagcacatcttggatttactgcctgggtttgtataggaggcttatggacatttattgct
tttcatgggctcattggtctcatagcattttccctacgtcagtttgaaatagcacgatta
gttttaattagaccttataatgcacttgcattctcaggaccaatagcagtttatacatct
gtcttcctaatttatccacttggtcaatctagctggttctttgcaccaagctttggtatt
gctgctatcttccgattcctattattccttcaaggttttcataattggacattaaatcct
tttcacatgatgggtgtagctggaatattgggaggtgccctactatcagctatccatggt
gctactgtcattaacactatttatcaagatgctcgtgcatatacaacatttcgtgctttc
tcaccaagtcaaccagaagaaacttattcaatgcttacagctaatcgtttttggtctcag
atatttggtgtagccttctcaaacaagagatggcttcatttcttcatgctatttgtacct
ctagcaggtatgtggacatcttcaattggtatcattggattagcatttaatctaagagct
tatgattttatttctcaagaattgaaagcagcagaagatcctgaatttgaaacattttat
acaaagaacattctactcaatgaaggtatacgactttggatggctgtccaagatcaagct
catgaaaattatcagttcccagaagaggtattgccaagaggtaattctttgtga
>Ss6_psbD
atgaaactcatctattctacaagattcatggcaagtaaagcttatcttcttggatcactt
actctactagatgactggttaaaacgagatcgatttgtctttattggctggtcaggtcta
ttattatttcctacagcttacttagcagcaggtggatggtttacagccaccgcttttgta
acatctttctttacacatggtctggtcacatcttatctagaagcttgtaatttttaaca
gctgcagtttcaactccagccaactctatggcccattcattactactactctggggacca
gaagcacatcttggatttactgcctgggtttgtataggaggcttatggacatttattgct
tttcatgggctcattggtctcatagcattttccctacgtcagtttgaaatagcacgatta
gttttaattagaccttataatgcacttgcattctcaggaccaatagcagtttatacatct
gtcttcctaatttatccacttggtcaatctagctggttctttgcaccaagctttggtatt
gctgctatcttccgattcctattattccttcaaggttttcataattggacattaaatcct
tttcacatgatgggtgtagctggaatattgggaggtgccctactatcagctatccatggt
gctactgtcattaacactatttatcaagatgctcgtgcatatacaacatttcgtgctttc
tcaccaagtcaaccagaagaaacttattcaatgcttacagctaatcgcttttggtctcag
atatttggtgtagccttctcaaacaagagatggcttcatttcttcatgctatttgtacct
ctagcaggtatgtggacatcttcaattggtatcattggattagcatttaatctaagagct
tatgattttatttctcaagaattgaaagcagcagaagatcctgaatttgaaacattttat
acaaagaacattctactcaatgaaggtatacgactttggatggctgtccaagatcaagct
catgaaaattatcagttcccagaagaggtattgccaagaggtaattctttgtga

>Ss7_psbD
atgaaactcatctattctacaagattcatggcaagtaaagcttatcttcttggatcactt
actctactagatgactggttaaaacgagatcgatttgtctttattggctggtcaggtcta
ttattatttcctacagcttacttagcagcaggtggatggtttacagccaccgcttttgta
acatctttctttacacatggtctggtcacatcttatctagaagcttgtaattttttaaca
gctgcagtttcaactccagccaactctatggcccattcattactactactctggggacca
gaagcacatcttggatttactgcctgggtttgtataggaggcttatggacatttattgct
tttcatgggctcattggtctcatagcattttccctacgtcagtttgaaatagcacgatta
gttttaattagaccttataatgcacttgcattctcaggaccaatagcagtttatacatct
gtcttcctaatttatccacttggtcaatctagctggttctttgcaccaagctttggtatt
gctgctatcttccgattcctattattccttcaaggttttcataattggacattaaatcct
tttcacatgatgggtgtagctggaatattgggaggtgccctactatcagctatccatggt
gctactgtcattaacactatttatcaagatgctcgtgcatatacaacatttcgtgctttc
tcaccaagtcaaccagaagaaacttattcaatgcttacagctaatcgtttttggtctcag
atatttggtgtagccttctcaaacaagagatggcttcatttcttcatgctatttgtacct
ctagcaggtatgtggacatcttcaattggtatcattggattagcatttaatctaagagct
tatgattttatttctcaagaattgaaagcagcagaagatcctgaatttgaaacattttat
acaaagaacattctactcaatgaaggtatacgactttggatggctgtccaagatcaagct
catgaaaattatcagttcccagaagaggtattgccaagaggtaattctttgtga
>Ss8_psbD
atgaaactcatctattctacaagattcatggcaagtaaagcttatcttcttggatcactt
actctactagatgactggttaaaacgagatcgatttgtctttattggctggtcaggtcta
ttattatttcctacagcttacttagcagcaggtggatggtttacagccaccgcttttgta
acatctttctttacacatggtctggtcacatcttatctagaagcttgtaactttttaaca
gctgcagtttcaactccagccaactctatggcccattcattactactactctggggacca
gaagcacatcttggatttactgcctgggtttgtataggaggcttatggacatttattgct
tttcatgggctcattggtctcatagcattttccctacgtcagtttgaaatagcacgatta
gttttaattagaccttataatgcacttgcattctcaggaccaatagcagtttatacatct
gtcttcctaatttatccacttggtcaatctagctggttctttgcaccaagctttggtatt
gctgctatcttccgattcctattattccttcaaggttttcataattggacattaaatcct
tttcacatgatgggtgtagctggaatattgggaggtgccctactatcagctatccatggt
gctactgtcattaacactatttatcaagatgctcgtgcatatacaacatttcgtgctttc
tcaccaagtcaaccagaagaaacttattcaatgcttacagctaatcgtttttggtctcag
atatttggtgtagccttctcaaacaagagatggcttcatttcttcatgctatttgtacct
ctagcaggtatgtggacatcttcaattggtatcattggattagcatttaatctaagagct
tatgattttatttctcaagaattgaaagcagcagaagatcctgaatttgaaacattttat
acaaagaacattctactcaatgaaggtatacgactttggatggctgtccaagatcaagct
catgaaaattatcagttcccagaagaggtattgccaagaggtaattctttgtga
>ohdo1_psbD
atgaaactcatctattctacaagattcatggcaagtaaagcttatcttcttggatcactt
actctactagatgactggttaaaacgagatcgatttgtctttattggctggtcaggtcta
ttattatttcctacagcttacttagcagcaggtggatggtttacagccaccacttttgta
acatctttctttacacatggtctggtcacatcttatctagaagcttgtaattttttaaca
gctgcagtttcaactccagccaactctatggcccattcattactactactctggggacca
gaagcacatcttggatttactgcctgggtttgtataggaggcttatggacatttattgct
tttcatgggctcattggtctcatagcattttccctacgtcagtttgaaatagcacgatta
gttttaattagaccttataatgcacttgcattctcaggaccaatagcagtttatacatct
gtcttcctaatttatccacttggtcaatctagctggttctttgcaccaagctttgggatt
gctgctatcttccgattcctattattccttcaaggttttcataattggacattaaatcct
tttcacatgatgggtgtagctggaatattgggaggtgccctactatcagctatccatggt
gctactgtcattaacactatttatcaagatgctcgtgcatatacaacatttcgtgctttc
tcaccaagtcaaccagaagaaacttattcaatgcttacagctaatcgtttttggtctcag
atatttggtgtagccttctcaaacaagagatggcttcatttcttcatgctatttgtacct
ctagcaggtatgtggacatcttcaattggtatcattggattagcatttaatctaagagct
tatgattttatttctcaagaattgaaagcagcagaagatcctgaatttgaaacattttat
acaaagaacattctactcaatgaaggtatacgactttggatggctgtccaagatcaagct
catgaaaattatcagttcccagaagaggtattgccaagaggtaattctttgtga

>ohdo3_psbD
atgaaactcatctattctacaagattcatggcaagtaaagcttatcttcttggatcactt
actctactagatgactggttaaaacgagatcgatttgtctttattggctggtcaggtcta
ttattatttcctacagcttacttagcagcaggtggatggtttacagccaccgcttttgta
acatctttctttacacatggtctggtcacatcttatctagaagcttgtaactttttaaca
gctgcagtttcaactccagccaactctatggcccattcattactactactctggggacca
gaagcacatcttggatttactgcctgggtttgtataggaggcttatggacatttattgct
tttcatgggctcattggtctcatagcattttccctacgtcagtttgaaatagcacgatta
gttttaattagaccttataatgcacttgcattctcaggaccaatagcagtttatacatct
gtcttcctaatttatccacttggtcaatctagctggttctttgcaccaagctttggtatt
gctgctatcttccgattcctattattccttcaaggttttcataattggacattaaatcct
tttcacatgatgggtgtagctggaatattgggaggtgccctactatcagctatccatggt
gctactgtcattaacactatttatcaagatgctcgtgcatatacaacatttcgtgctttc
tcaccaagtcaaccagaagaaacttattcaatgcttacagctaatcgcttctggtctcaa
atatttggtgtagccttctcaaacaagagatggcttcatttcttcatgctatttgtacct
ctagcaggtatgtggacatcttcaattggtatcattggattagcatttaatctaagagct
tatgattttatttctcaagaattgaaagcagcagaagatcctgaatttgaaacattttat
acaaagaacattctactcaatgaaggtatacgactttggatggctgtccaagatcaagct
catgaaaattatcagttcccagaagaggtattgccaagaggtaattctttgtga
>ohdo5_psbD
atgaaactcatctattctacaagattcatggcaagtaaagcttatcttcttggatcactt
actctactagatgactggttaaaacgagatcgatttgtctttattggctggtcaggtcta
ttattatttcctacagcttacttagcagtaggtggatggtttacagccaccgcttttgta
acatctttctttacacatggtctggtcacatcttatctagaagcttgtaatttttttaaca
gctgcagtttcaactccagccaactctatggcccattcattactactactctggggacca
gaagcacatcttggatttactgcctgggtttgtataggaggcttatggacatttattgct
tttcatgggctcattggtctcatagcattttccctacgtcagtttgaaatagcacgatta
gttttaattagaccttataatgcacttgcattctcaggaccaatagcagtttatacatct
gtcttcctaatttatccacttggtcaatctagctggttctttgcaccaagctttggtatt
gctgctatcttccgattcctattattccttcaaggttttcataattggacattaaatcct
tttcacatgatgggtgtagctggaatattgggaggtgccctactatcagctatccatggt
gctactgtcattaacactatttatcaagatgctcgtgcatatacaacatttcgtgctttc
tcaccaagtcaaccagaagaaacttattcaatgcttacagctaatcgtttttggtctcag
atatttggtgtagccttctcaaacaagagatggcttcatttcttcatgctatttgtacct
ctagcaggtatgtggacatcttcaattggtatcattggattagcatttaatctaagagct
tatgattttatttctcaagaattgaaagcagcagaagatcctgaatttgaaacattttat
acaaagaacattctactcaatgaaggtatacgactttggatggctgtccaagatcaagct
catgaaaattatcagttcccagaagaggtattgccaagaggtaattctttgtga
>ohdo7_psbD
atgaaactcatctattctacaagattcatggcaagtaaagcttatcttcttggatcactt
actctactagatgactggttaaaacgagatcgatttgtctttattggctggtcaggtcta
ttattatttcctacagcttacttagcagcaggtggatggtttacagccaccgcttttgta
acatctttctttacacatggtctggtcacatcttatctagaagcttgtaatttttttaaca
gctgcagtttcaactccagccaactctatggcccattcattactactactctggggacca
gaagcacatcttggatttactgcctgggtttgtataggaggcttatggacatttattgct
tttcatgggctcattggtctcatagcattttccctacgtcagtttgaaatagcacgatta
gttttaattagaccttataatgcacttgcattctcaggaccaatagcagtttatacatct
gtcttcctaatttatccacttggtcaatctagctggttctttgcaccaagctttggtatt
gctgctatcttccgattcctattattccttcaaggttttcataattggacattaaatcct
tttcacatgatgggtgtagctggaatattgggaggtgccctactatcagctatccatggt
gctactgtcattaacactatttatcaagatgctcgtgcatatacaacatttcgtgctttc
tcaccaagtcaaccagaagaaacttattcaatgcttacagctaatcgtttttggtctcag
atatttggtgtagccttctcaaacaagagatggcttcatttcttcatgctatttgtacct
ctagcaggtatgtggacatcttcaattggtatcattggattagcatttaatctaagagct
tatgattttatttctcaagaattgaaagcagcagaagatcctgaatttgaaacattttat
acaaagaacattctactcaatgaaggtatacgactttggatggctgtccaagatcaagct
catgaaaattatcagttcccagaagaggtattgccaagaggtaattctttgtga

>sesoko1_psbD
atgaaactcatctattctacaagattcatggcaagtaaagcttatcttcttggatcactt
actctactagatgactggttaaaacgagatcgatttgtctttattggctggtcaggtcta
ttattatttcctacagcttacttagcagcaggtggatggtttacagccaccgcttttgta
acatctttctttacacatggtctggtcacatcttatctagaagcttgtaatttttaaca
gctgcagtttcaactccagccaactctatggcccattcattactactactctggggacca
gaagcacatcttggatttactgcctgggtttgtataggaggcttatggacatttattgct
tttcatgggctcattggtctcatagcattttccctacgtcagtttgaaatagcacgatta
gttttaattagaccttataatgcacttgcattctcaggaccaatagcagtttatacatct
gtcttcctaatttatccacttggtcaatctagctggttctttgcaccaagctttggtatt
gctgctatcttccgattcctattattccttcaaggttttcataattggacattaaatcct
tttcacatgatgggtgtagctggaatattgggaggtgccctactatcagctatccatggt
gctactgtcattaacactatttatcaagatgctcgtgcatatacaacatttcgtgctttc
tcaccaagtcaaccagaagaaacttattcaatgcttacagctaatcgtttttggtctcag
atatttggtgtagccttctcaaacaagagatggcttcatttcttcatgctatttgtacct
ctagcaggtatgtggacatcttcaattggtatcattggattagcatttaatctaagagct
tatgattttatttctcaagaattgaaagcagcagaagatcctgaatttgaaacattttat
acaaagaacattctactcaatgaaggtatacgactttggatggctgtccaagatcaagct
catgaaaattatcagttcccagaagaggtattgccaagaggtaattctttgtga
>sesoko3_psbD
atgaaactcatctattctacaagattcatggcaagtaaagcttatcttcttggatcactt
actctactagatgactggttaaaacgagatcgatttgtctttattggctggtcaggtcta
ttattatttcctacagcttacttagcagcaggtggatggtttacagccaccgcttttgta
acatctttctttacacatggtctggtcacatcttatctagaagcttgtaatttttaaca
gctgcagtttcaactccagccaactctatggcccattcattactactactctggggacca
gaagcacatcttggatttactgcctgggtttgtataggaggcttatggacatttattgct
tttcatgggctcattggtctcatagcattttccctacgtcagtttgaaatagcacgatta
gttttaattagaccttataatgcacttgcattctcaggaccaatagcagtttatacatct
gtcttcctaatttatccacttggtcaatctagctggttctttgcaccaagctttggtatt
gctgctatcttccgattcctattattccttcaaggttttcataattggacattaaatcct
tttcacatgatgggtgtagctggaatattgggaggtgccctactatcagctatccatggt
gctactgtcattaacactatttatcaagatgctcgtgcatatacaacatttcgtgctttc
tcaccaagtcaaccagaagaaacttattcaatgcttacagctaatcgtttttggtctcag
atatttggtgtagccttctcaaacaagagatggcttcatttcttcatgctatttgtacct
ctagcaggtatgtggacatcttcaattggtatcattggattagcatttaatctaagagct
tatgattttatttctcaagaattgaaagcagcagaagatcctgaatttgaaacattttat
acaaagaacattctactcaatgaaggtatacgactttggatggctgtccaagatcaagct
catgaaaattatcagttcccagaagaggtattgccaagaggtaattctttgtga
>sesoko4_psbD
atgaaactcatctattctacaagattcatggcaagtaaagcttatcttcttggatcactt
actctactagatgactggttaaaacgagatcgatttgtctttattggctggtcaggtcta
ttattatttcctacagcttacttagcagcaggtggatggtttacagccaccgcttttgta
acatctttctttacacatggtctggtcacatcttatctagaagcttgtaatttttaaca
gctgcagtttcaactccagccaactctatggcccattcattactactactctggggacca
gaagcacatcttggatttactgcctgggtttgtataggaggcttatggacatttattgct
tttcatgggctcattggtctcatagcattttccctacgtcagtttgaaatagcacgatta
gttttaattagaccttataatgcacttgcattctcaggaccaatagcagtttatacatct
gtcttcctaatttatccacttggtcaatctagctggttctttgcaccaagctttggtatt
gctgctatcttccgattcctattattccttcaaggttttcataattggacattaaatcct
tttcacatgatgggtgtagctggaatattgggaggtgccctactatcagctatccatggt
gctactgtcattaacactatttatcaagatgctcgtgcatatacaacatttcgtgctttc
tcaccaagtcaaccagaagaaacttattcaatgcttacagctaatcgtttttggtctcag
atatttggtgtagccttctcaaacaagagatggcttcatttcttcatgctatttgtacct
ctagcaggtatgtggacatcttcaattggtatcattggattagcatttaatctaagagct
tatgattttatttctcaagaattgaaagcagcagaagatcctgaatttgaaacattttat
acaaagaacattctactcaatgaaggtatacgactttggatggctgtccaagatcaagct
catgaaaattatcagttcccagaagaggtattgccaagaggtaattctttgtga

>REF_DNA_psbD_JX094327
ATGAAACTCATCTATTCTACAAGATTCATGTCAAGTAAATCTTATCTTCTTGGATCACTT
ACTCTACTAGATGACTGGTTAAAACGAGATCGATTTGTCTTTATTGGCTGGTCAGGTCTA
TTATTATTTCCTACAGCTTACTTAGCAGTAGGTGGATGGTTTACAGCTACCACTTTTGTA
ACATCTTTCTTTACACATGGTTTGGCCACATCTTATCTAGAAGCTTGTAACTTTTTAACA
GCTGCAGTTTCAACTCCAGCCAACTCTATGGCCCATTCATTACTACTACTCTGGGGACCA
GAAGCACATCTTGGATTTACTGCCTGGGTTTGTATAGGAGGCTTATGGACATTTATTGCT
TTTCATGGGCTCATTGGTCTCATAGGATTTTCCCTACGTCAGTTTGAAATAGCACGATTA
GTTTTAATTAGACCTTATAATGCACTTGCATTCTCAGGACCAATAGGAGTTTATACATCT
GTCTTCCTAATTTATCCACTTGGTCAATCTAGCTGGTTCTTTGCACCAAGCTTTGGGATT
GCTGCTATCTTCCGATTCCTATTATTCCTTCAAGGTTTTCATAATTGGACATTAAATCCT
TTTCACATGATGGGTGTAGCTGGAATATTGGGAGGTGCATTACTATCAGCTATCCATGGT
GCTACTGTCATTAACACTATTTATCAAGATGCTCGTGCATATACAACATTTCGTGCTTTC
TCACCTAGTCAACCAGAAGAAACTTATTCAATGCTTACAGCTAATCGCTTCTGGTCTCAA
ATATTTGGTGTAGCCTTCTCAAACAAGAGATGGCTTCATTTCTTCATGCTATTTGTACCT
CTAGCAGGTATGTGGACATCTTCAATTGGTATCATTGGATTAGCATTTAATCTAAGAGCT
TATGATTTTATTTCTCAAGAATTGAAAGCAGCAGAAGATCCTGAATTTGAAACATTTTAT
ACAAAGAACATTCTACTCAATGAAGGTATACGACTTTGGATGGCTGTCCAAGATCAAGCT
CATGAAAATTATCAGTTCCCAGAAGAGGTATTGCCAAGAGGTAATTCTTTGTGA
>REF_RNA_psbD_symbB1.comp12_c0_seq1
atgaaactcatctattctacaagattcatgtcaagtaaagcttatcttcctggatcactt
actctactagatgactggttaaaacgagatcgatttgtctttattggctggtcaggtcta
ttattatttcctacagcttacttagcagtaggtggatggtttacagctaccacttttgta
acatctttctttacacatggtttggccacatcttatctagaagcttgtaactttttaaca
gctgcagcttcaactccagccaactctatggcccattcattactactactctggggacca
gaagcacatcttggatttactgcctgggtttgtataggaggcttatggacatttattgct
tttcatgggctcattggtctcataggattttccctacgtcagtttgaaatagcacgacta
gttttaattagaccttataatgcacttgcattctcaggaccaataggagtttatacatct
gtcttcctaatttatccacttggtcaatctagctggttctttgcaccaagctttggggtt
gctgctatcttccgattcctattattccttcaaggttttcataattggacattaaatcct
tttcacatgatgggtgtagctggaatattgggaggtgcattactatcagctatccatggt
gctactgtcgttaacactatttatcaagatgctcgtgcatatacaacatttcgtgctttc
tcacctagtcaaccagaagaaacttattcaatgcttacagctaatcgcttctggtctcaa
gtatttggtgtagccttctcaaacaagagatggcttcatttcttcatgctatttgtacct
ctagcaggtatgtggacatcttcaattggtatcattggattagcatttaatctaagagct
tatgattttatttctcaagaattgaaagcagcagaagatcctgaatttgaaacattttat
acaaagaacattctactcaatgaaggtatacgactttggatggctgtccaagatcaacct
catgaaaattatcagttcccagaagaggtattgccaagaggtaattctttgtga

>Hd1_psbE
atgagtacagctgaacgtgcattgattgatattgcacaagatcgtcgttattggataatt
cattcaattacaatcccttctttatttgttggtggagttatatttatgctgtctggcttt
gtgtacaagttatttggggctcttaatttcaacaaatacttcgataacgataatagttca
atttctttaataaaggacaggtttagtatatctagctcaatggacgatatttag
>Hd2_psbE
atgagtacagctgaacgtgcattgattgatattgcacaagatcgtcgttattggataatt
cattcaattacaataccttctttatttgttggtggagttatatttatgctgtctggcttt
gtttacaagttatttggggctctaaattttaacaaatacttcgataaggataatagttca
atctctttaataaaggacaggtttagtatatctagctcaatggacgatatttag
>Hd3_psbE
atgagtacagctgaacgtgcattgattgatattgcacaagatcgtcgttattggataatt
cattcaattacaataccttctttatttgttggtggagttatatttatgctgtctggcttt
gtgtacaagttatttggggctctaaatttcaacaaatacttcgataaggataatagttca
atctctttaataaaggacaggtttagtatatctagctcaatggacgatatttag
>Hd4_psbE
atgagtacagctgaacgtgcattgattgatattgcacaagatcggcgttattggataatt
cattcaattacaataccttctttatttgttggtggagttatatttatgctgtctggcttt
gtgtacaagttatttggggctctaaatttcaacaaatacttcgataaggataatagttca
atctctttaataaaggacaggtttagtatatctagctcaatggacgatatttag
>Hd5_psbE
atgagtacagctgaacgtgcattgattgatattgcacaagatcgtcgttattggataatt
cattcaattacaataccttctttatttgttggtggagttatatttatgctgtctggcttt
gtgtacaagttatttggggctctaaatttcaacaaatacttcgataaggataatagttca
atctctttaataaaggacaggtttagtatatctagctcaatggacgatatttag
>Hd6_psbE
atgagtacagctgaacgtgcattgattgatattgcacaagatcgtcgttattggataatt
cattcaattacaataccttctttatttgttggtggagttatatttatgctgtctggcttt
gtgtacaagttatttggggctctaaatttcaacaaatacttcgataaggataatagttca
atctctttaataaaggacaggtttagtatatctagctcaatggacgatatttag
>Hd7_psbE
atgagtacagctgaacgtgcattgattgatattgcacaagatcgtcgttattggataatt
cattcaattacaatcccttctttatttgttggtggagttatatttatgctgtctggcttt
gtgtacaagttatttggggctcttaatttcaacaaatacttcgataacgataatagttca
atttctttaataaaggacaggtttagtatatctagctcaatggacgatatttag
>Hd8_psbE
atgagtacagctgaacgtgcattgattgatattgcacaagatcgtcgttattggataatt
cattcaattacaatcccttctttatttgttggtggagttatatttatgctgtctggcttt
gtgtacaagttatttggggctcttaatttcaacaaatacttcgataacgataatagttca
atttctttaataaaggacaggtttagtatatctagctcaatggacgatatttag
>Hd9_psbE
atgagtacagctgaacgtgcattgattgatattgcacaagatcgtcgttattggataatt
cattcaattacaatcccttctttatttgttggtggagttatatttatgctgtctggcttt
gtgtacaagttatttggggctcttaatttcaacaaatacttcgataacgataatagttca
atttctttaataaaggacaggtttagtatatctagctcaatggacgatatttag
>IS1_psbE
atgagtacagctgaacgtgcattgattgatattgcacaagaccgtcgttattggataatt
cattcaattacaataccttctttatttgttggtggagttatatttatgctgtctggcttt
gtgtacaagttatttggtgctctaaatttcaacaactacttcgataaggataatagttca
atttctttaataaaggacaggtttagtatatctagctcaatggacgatatttag
>IS2_psbE
atgagtacagctgaacgtgcattgattgatattgcacaagatcgtcgttattggataatt
cattcaattacaataccttctttatttgttggtggagttatatttatgctgtctggcttt
gtgtacaagttatttggggctctaaatttcaacaaatacttcgataaggataatagttca
atctctttaataaaggacaggtttagtatatctagctcaatggacgatatttag
>IS3_psbE
atgagtacagctgaacgtccattgattgatattccacaagatcgtcgttattggataatt

cattcaattacaataccttctttatttgttggtggagttatatttatgctatctggcttt
gtgtataagttatttggtgctctaaatttcaacaaatacttcgataacgataatagttca
atttctttaataaaggacaggtttagtatatctagctcaatggacgatatttag
>IS4_psbE
atgagtacagctgaacgtccattgattgatattccacaagaccgtcgttattggataatt
cattcaattacaataccttctttatttgttggtggagttatatttatgctatctggcttt
gtgtataagttatttggtgctctaaatttcaacaaatacttcgataaggataatagttca
atctctttaataaaggacaggtttagtatatctagctcaatggacgatatttag
>IS5_psbE
atgagtacagctgaacgtgcattgattgatattgcacaagatcgtcgttattggataatt
cattcaattacaatcccttctttatttgttggtggagttatatttatgctgtctggcttt
gtgtacaagttatttggggctcttaatttcaacaaatacttcgataacgataatagttca
atttctttaataaaggacaggtttagtatatctagctcaatggacgatatttag
>IS6_psbE
atgagtacagctgaacgtgcattgattgatattgcacaagatcgtcgttattggataatt
cattcaattacaatcccttctttatttgttggtggagttatatttatgctgtctggcttt
gtgtacaagttatttggggctctaaatttcaacaaatacttcgataaggataatagttca
atctctttaataaaggacaggtttagtatatctagctcaatggacgatatttag
>IS7_psbE
atgagtacagctgaacgtccattgattgatattccacaagaccgtcgttattggataatt
cattcaattacaataccttctttatttgttggtggagttatatttatgctatctggcttt
gtgtataagttatttggggctctaaatttcaacaaatacttcgataaggataatagttca
atatctttaataaaggacaggtttagtatatctagctcaatggacgatatttag
>IS8_psbE
atgagtacagctgaacgtgcattgattgatatttcacaagatcgtcgttattggataatt
cattcaattacaataccttctttatttgttggtggagttatatttatgctgtctggcttt
gtgtacaagttatttggggctcttaatttcaacaaatacttcgataaggataatagttca
atctctttaataaaggacaggtttagtatatctagctcaatggacgatatttag
>IS9_psbE
atgagtacagctgaacgtgcattgattgatattccacaagaccgtcgttattggataatt
cattcaattacaataccttctttatttgttggtggagttatatttatgctatctggcttt
gtgtacaagttatttggggctcttaatttcaacaaatacttcgataacgataatagttca
atttctttaataaaggacaggtttagtatatctagctcaatggacgatatttag
>Ik1_psbE
atgagtacagctgaacgtgcattgattgatattgcacaagatcgtcgttattggataatt
cattcaattacaatcccttctttatttgttggtggagttatatttatgctgtctggcttt
gtgtacaagttatttggggctctaaatttcaacaaatacttcgataaggataatagttca
atctctttaataaaggacaggtttagtatatctagctcaatggacgatatttag
>Ik2_psbE
atgagtacagctgaacgtgcattgattgatattgcacaagatcgtcgttattggataatt
cattcaattacaataccttctttatttgttggtggagttatatttatgctgtctggcttt
gtgtacaagttatttggggctcttaatttcaacaaatacttcgataaggataatagttca
atctctttaataaaggacaggtttagtatatctagctcaatggacgatatttag
>Ik3_psbE
atgagtacagctgaacgtgcattgattgatattgcacaagatcgtcgttattggataatt
cattcaattacaataccttctttatttgttggtggagttatatttatgctgtctggcttt
gtgtacaagttatttggggctcttaatttcaacaaatacttcgataacgataatagttca
atttctttaataaaggacaggtttagtatatctagctcaatggacgatatttag
>Ik4_psbE
atgagtacagctgaacgtgcattgattgatattgcacaagatcgtcgttattggataatt
cattcaattacaatcccttctttatttgttggtggagttatatttatgctgtctggcttt
gtgtacaagttatttggggctctaaatttcaacaaatacttcgataaggataatagttca
atctctttaataaaggacaggtttagtatatctagctcaatggacgatatttag
>Ik5_psbE
atgagtacagctgaacgtgcattgattgatattgcacaagatcgtcgttattggataatt
cattcaattacaatcccttctttatttgttggtggagttatatttatgctgtctggcttt
gtgtacaagttatttggggctctaaatttcaacaaatacttcgataaggataatagttca

atctctttaataaaggacaggtttagtatatctagctcaatggacgatatttag
>Ik6_psbE
atgagtacagctgaacgtgcattgattgatattgcacaagatcgtcgttattggataatt
cattcaattacaatcccttctttatttgttggtggagttatatttatgctgtctggcttt
gtgtacaagttatttggggctcttaatttcaacaaatacttcgataacgataatagttca
atttctttaataaaggacaggtttagtatatctagctcaatggacgatatttag
>Ik7_psbE
atgagtacagctgaacgtgcattgattgatattgcacaagatcgtcgttattggataatt
cattcaattacaatcccttctttatttgttggtggagttatatttatgctgtctggcttt
gtgtacaagttatttggggctctaaatttcaacaaatacttcgataaggataatagttca
atctctttaataaaggacaggtttagtatatctagctcaatggacgatatttag
>Ik8_psbE
atgagtacagctgaacgtgcattgattgatattgcacaagatcgtcgttattggataatt
cattcaattacaatcccttctttatttgttggtggagttatatttatgctgtctggcttt
gtgtacaagttatttggggctctaaatttcaacaaatacttcgataacgataatagttca
atttctttaataaaggacaggtttagtatatctagctcaatggacgatatttag
>Ik9_psbE
atgagtacagctgaacgtgcattgattgatattgcacaagatcgtcgttattggataatt
cattcaattacaatcccttctttatttgttggtggagttatatttatgctgtctggcttt
gtgtacaagttatttggggctcttaatttcaacaaatacttcgataaggataatagttca
atttctttaataaaggacaggtttagtatatctagctcaatggacgatatttag
>Irm10_psbE
atgagtacagctgaacgtgcattgattgatattgcacaagatcgtcgttattggataatt
cattcaattacaataccttctttatttgttggtggagttatatttatgctgtctggcttt
gtgtacaagttatttggggctctaaatttcaacaaatacttcgataaggataatagttca
atctctttaataaaggacaggtttagtatatctagctcaatggacgatatttag
>Irm17_psbE
atgagtacagctgaacgtgcattgattgatatttcacaagatcgtcgttattggataatt
cattcaattacaataccttctttatttgttggtggagttatatttatgctgtctggcttt
gtgtacaagttatttggggctctaaatttcaacaaatacttcgataaggataatagttca
atctctttaataaaggacaggtttagtatatctagctcaatggacgatatttag
>Irm2_psbE
atgagtacagctgaacgtgcattgattgatattgcacaagatcgtcgttattggataatt
cattcaattacaatcccttctttatttgttggtggagttatatttatgctgtctggcttt
gtgtacaagttatttggggctctaaatttcaacaaatacttcgataaggataatagttca
atctctttaataaaggacaggtttagtatatctagctcaatggacgatatttag
>Irm21_psbE
atgagtacagctgaacgtgcattgattgatattgcacaagatcgtcgttattggataatt
cattcaattacaatcccttctttatttgttggtggagttatatttatgctgtctggcttt
gtgtacaagttatttggggctctaaatttcaacaaatacttcgataaggataatagttca
atctctttaataaaggacaggtttagtatatctagctcaatggacgatatttag
>Irm22_psbE
atgagtacagctgaacgtgcattgattgatattgcacaagatcgtcgttattggataatt
cattcaattacaatcccttctttatttgttggtggagttatatttatgctgtctggcttt
gtgtacaagttatttggggctcttaatttcaacaaatacttcgataacgataatagttca
atttctttaataaaggacaggtttagtatatctagctcaatggacgatatttag
>Irm23_psbE
atgagtacagctgaacgtgcattgattgatatttcacaagatcgtcgttattggataatt
cattcaattacaataccttctttatttgttggtggagttatatttatgctgtctggcttt
gtgtacaagttatttggggctcttaatttcaacaaatacttcgataacgataatagttca
atttctttaataaaggacaggtttagtatatctagctcaatggacgatatttag
>Irm24_psbE
atgagtacagctgaacgtgcattgattgatattgcacaagatcgtcgttattggataatt
cattcaattacaatcccttctttatttgttggtggagttatatttatgctgtctggcttt
gtgtacaagttatttggggctcttaatttcaacaaatacttcgataacgataatagttca
atttctttaataaaggacaggtttagtatatctagctcaatggacgatatttag
>Irm25_psbE

atgagtacagctgaacgtgcattgattgatattgcacaagatcgtcgttattggataatt
cattcaattacaatcccttctttatttgttggtggagttatatttatgctgtctggcttt
gtgtacaagttatttggggctcttaatttcaacaaatacttcgataacgataatagttca
atttctttaataaaggacaggtttagtatatctagctcaatggacgatatttag
>Irm26_psbE
atgagtacagctgaacgtgcattgattgatattgcacaagatcgtcgttattggataatt
cattcaattacaataccttctttatttgttggtggagttatatttatgctgtctggcttt
gtgtacaagttatttggggctctaaatttcaacaaatacttcgataacgataatagttca
atttctttaataaaggacaggtttagtatatctagctcaatggacgatatttag
>Irm27_psbE
atgagtacagctgaacgtccattgattgatattccacaagaccgtcgttattggataatt
cattcaattacaatcccttctttatttgttggtggagttatatttatgctgtctggcttt
gtgtacaagttatttggggctcttaatttcaacaaatacttcgataacgataatagttca
atttctttaataaaggacaggtttagtatatctagctcaatggacgatatttag
>Irm3_psbE
atgagtacagctgaacgtccattgattgatattccacaagaccgtcgttattggataatt
cattcaattacaataccttctttatttgttggtggagttatatttatgctatctggcttt
gtgtataagttatttggtgctctaaatttcaacaactacttcgataaggataatagttca
atctctttaataaaggacaggtttagtatatctagctcaatggacgatatttag
>Irm4_psbE
atgagtacagctgaacgtccattgattgatattccacaagaccgtcgttattggataatt
cattcaattacaataccttctttatttgttggtggagttatatttatgctatctggcttt
gtgtataagttatttggtgctctaaatttcaacaactacttcgataaggataatagttca
atctctttaataaaggacaggtttagtatatctagctcaatggacgatatttag
>Irm5_psbE
atgagtacagctgaacgtgcattgattgatattgcacaagatcgtcgttattggataatt
cattcaattacaataccttctttatttgttggtggagttatatttatgctgtctggcttt
gtgtacaagttatttggggctctaaatttcaacaaatacttcgataaggataatagttca
atctctttaataaaggacaggtttagtatatctagctcaatggacgatatttag
>Irm7_psbE
atgagtacagctgaacgtgcattgattgatattgcacaagatcgtcgttattggataatt
cattcaattacaatcccttctttatttgttggtggagttatatttatgctgtctggcttt
gtgtacaagttatttggggctctaaatttcaacaaatacttcgataaggataatagttca
atctctttaataaaggacaggtttagtatatctagctcaatggacgatatttag
>Irm9_psbE
atgagtacagctgaacgtgcattgattgatattgcacaagatcgtcgttattggataatt
cattcaattacaataccttctttatttgttggtggagttatatttatgctatctggcttt
gtgtacaagttatttggggctcttaatttcaacaaatacttcgataacgataatagttca
atttctttaataaaggacaggtttagtatatctagctcaatggacgatatttag
>Isy12_psbE
atgagtacagctgaacgtgcattgattgatattgcacaagatcgtcgttattggataatt
cattcaattacaatcccttctttatttgttggtggagttatatttatgctgtctggcttt
gtgtacaagttatttggggctcttaatttcaacaaatacttcgataacgataatagttca
atttctttaataaaggacaggtttagtatatctagctcaatggacgatatttag
>Isy15_psbE
atgagtacagctgaacgtccattgattgatattccacaagaccgtcgttattggataatt
cattcaattacaataccttctttatttgttggtggagttatatttatgctatctggcttt
gtgtataagttatttggtgctctaaatttcaacaactacttcgataaggataatagttca
atttctttaataaaggacaggtttagtatatctagctcaatggacgatatttag
>Isy16_psbE
atgagtacagctgaacgtgcattgattgatatttcacaagatcgtcgttattggataatt
cattcaattacaataccttctttatttgttggtggagttatatttatgctgtctggcttt
gtgtacaagttatttggggctcttaatttcaacaaatacttcgataaggataatagttca
atctctttaataaaggacaggtttagtatatctagctcaatggacgatatttag
>Isy17_psbE
atgagtacagctgaacgtgcattgattgatatttcacaagatcgtcgttattggataatt
cattcaattacaatcccttctttatttgttggtggagttatatttatgctgtctggcttt

gtgtacaagttatttggggctctaaatttcaacaaatacttcgataacgataatagttca
atttctttaataaaggacaggtttagtatatctagctcaatggacgatatttag
>Isy18_psbE
atgagtacagctgaacgtgcattgattgatatttcacaagatcgtcgttattggataatt
cattcaattacaataccttctttatttgttggtggagttatatttatgctgtctggcttt
gtgtacaagttatttggggctctaaatttcaacaaatacttcgataaggataatagttca
atttctttaataaaggacaggtttagtatatctagctcaatggacgatatttag
>Isy21_psbE
atgagtacagctgaacgtgcattgattgatattgcacaagatcgtcgttattggataatt
cattcaattacaataccttctttatttgttggtggagttatatttatgctgtctggcttt
gtgtacaagttatttggggctctaaatttcaacaaatacttcgataaggataatagttca
atatctttaataaaggacaggtttagtatatctagctcaatggacgatatttag
>Isy22_psbE
atgagtacagctgaacgtgcattgattgatatttcacaagatcgtcgttattggataatt
cattcaattacaataccttctttatttgttggtggagttatatttatgctgtctggcttt
gtgtacaagttatttggggctctaaatttcaacaaatacttcgataaggataatagttca
atatctttaataaaggacaggtttagtatatctagctcaatggacgatatttag
>Isy23_psbE
atgagtacagctgaacgtgcattgattgatattgcacaagatcgtcgttattggataatt
cattcaattacaataccttctttatttgttggtggagttatatttatgctgtctggcttt
gtgtacaagttatttggggctctaaatttcaacaaatacttcgataaggataatagttca
atatctttaataaaggacaggtttagtatatctagctcaatggacgatatttag
>Isy24_psbE
atgagtacagctgaacgtgcattgattgatattgcacaagatcgtcgttattggataatt
cattcaattacaataccttctttatttgttggtggagttatatttatgctgtctggcttt
gtgtacaagttatttggggctctaaatttcaacaaatacttcgataaggataatagttca
atctctttaataaaggacaggtttagtatatctagctcaatggacgatatttag
>Isy25_psbE
atgagtacagctgaacgtgcattgattgatattgcacaagatcgtcgttattggataatt
cattcaattacaatcccttctttatttgttggtggagttatatttatgctgtctggcttt
gtgtacaagttatttggggctctaaatttcaacaaatacttcgataaggataatagttca
atatctttaataaaggacaggtttagtatatctagctcaatggacgatatttag
>Isy26_psbE
atgagtacagctgaacgtgcattgattgatattgcacaagatcgtcgttattggataatt
cattcaattacaataccttctttatttgttggtggagttatatttatgctgtctggcttt
gtgtacaagttatttggggctctaaatttcaacaaatacttcgataacgataatagttca
atttctttaataaaggacaggtttagtatatctagctcaatggacgatatttag
>Isy27_psbE
atgagtacagctgaacgtgcattgattgatattgcacaagatcgtcgttattggataatt
cattcaattacaataccttctttatttgttggtggagttatatttatgctgtctggcttt
gtgtacaagttatttggggctctaaatttcaacaaatacttcgataaggataatagttca
atatctttaataaaggacaggtttagtatatctagctcaatggacgatatttag
>Isy4_psbE
atgagtacagctgaacgtgcattgattgatatttcacaagatcgtcgttattggataatt
cattcaattacaataccttctttatttgttggtggagttatatttatgctgtctggcttt
gtgtacaagttatttggggctctaaatttcaacaaatacttcgataaggataatagttca
atctctttaataaaggacaggtttagtatatctagctcaatggacgatatttag
>Isy7_psbE
atgagtacagctgaacgtccattgattgatatttcacaagaccgtcgttattggataatt
cattcaattacaataccttctttatttgttggtggagttatatttatgctatctggcttt
gtgtataagttatttggtgctctaaatttcaacaactacttcgataaggataatagttca
atctctttaataaaggacaggtttagtatatctagctcaatggacgatatttag
>Isy8_psbE
atgagtacagctgaacgtgcattgattgatattgcacaagatcgtcgttattggataatt
cattcaattacaataccttctttatttgttggtggagttatatttatgctgtctggcttt
gtgtacaagttatttggggctctaaatttcaacaaatacttcgataaggataatagttca
atatctttaataaaggacaggtttagtatatctagctcaatggacgatatttag

>KrA1_psbE
atgagtacagctgaacgtgcattgattgatattgcacaagatcgtcgttattggataatt
cattcaattacaataccttctttatttgttggtggagttatatttatgctgtctggcttt
gtgtacaagttatttggggctctaaatttcaacaaatacttcgataaggataatagttca
atctctttaataaaggacaggtttagtatatctagctcaatggacgatatttag
>KrA10_psbE
atgagtacagctgaacgtgcattgattgatattgcacaagatcgtcgttattggataatt
cattcaattacaataccttctttatttgttggtggagttatatttatgctgtctggcttt
gtgtacaagttatttggggctctaaatttcaacaaatacttcgataacgataatagttca
atctctttaataaaggacaggtttagtatatctagctcaatggacgatatttag
>KrA11_psbE
atgagtacagctgaacgtgcattgattgatattgcacaagatcgtcgttattggataatt
cattcaattacaataccttctttatttgttggtggagttatatttatgctgtctggcttt
gtgtacaagttatttggggctctaaatttcaacaaatacttcgataaggataatagttca
atctctttaataaaggacaggtttagtatatctagctcaatggacgatatttag
>KrA12_psbE
atgagtacagctgaacgtgcattgattgatattgcacaagatcgtcgttattggataatt
cattcaattacaatcccttctttatttgttggtggagttatatttatgctgtctggcttt
gtgtacaagttatttggggctcttaatttcaacaaatacttcgataacgataatagttca
atctctttaataaaggacaggtttagtatatctagctcaatggacgatatttag
>KrA13_psbE
atgagtacagctgaacgtgcattgattgatattgcacaagatcgtcgttattggataatt
cattcaattacaatcccttctttatttgttggtggagttatatttatgctgtctggcttt
gtgtacaagttatttggggctcttaatttcaacaaatacttcgataacgataatagttca
atctctttaataaaggacaggtttagtatatctagctcaatggacgatatttag
>KrA14_psbE
atgagtacagctgaacgtgcattgattgatattgcacaagatcgtcgttattggataatt
cattcaattacaatcccttctttatttgttggtggagttatatttatgctgtctggcttt
gtgtacaagttatttggggctcttaatttcaacaaatacttcgataacgataatagttca
atttctttaataaaggacaggtttagtatatctagctcaatggacgatatttag
>KrA15_psbE
atgagtacagctgaacgtgcattgattgatattgcacaagatcgtcgttattggataatt
cattcaattacaatcccttctttatttgttggtggagttatatttatgctgtctggcttt
gtgtacaagttatttggggctcttaatttcaacaaatacttcgataacgataatagttca
atttctttaataaaggacaggtttagtatatctagctcaatggacgatatttag
>KrA2_psbE
atgagtacagctgaacgtgcattgattgatattgcacaagatcgtcgttattggataatt
cattcaattacaataccttctttatttgttggtggagttatatttatgctgtctggcttt
gtgtacaagttatttggggctctaaatttcaacaaatacttcgataaggataatagttca
atctctttaataaaggacaggtttagtatatctagctcaatggacgatatttag
>KrA3_psbE
atgagtacagctgaacgtgcattgattgatattgcacaagatcgtcgttattggataatt
cattcaattacaataccttctttatttgttggtggagttatatttatgctgtctggcttt
gtgtacaagttatttggggctctaaatttcaacaaatacttcgataacgataatagttca
atctctttaataaaggacaggtttagtatatctagctcaatggacgatatttag
>KrA4_psbE
atgagtacagctgaacgtgcattgattgatattgcacaagatcgtcgttattggataatt
cattcaattacaatcccttctttatttgttggtggagttatatttatgctgtctggcttt
gtgtacaagttatttggggctcttaatttcaacaaatacttcgataacgataatagttca
atttctttaataaaggacaggtttagtatatctagctcaatggacgatatttag
>KrA5_psbE
atgagtacagctgaacgtgcattgattgatattgcacaagatcgtcgttattggataatt
cattcaattacaataccttctttatttgttggtggagttatatttatgctgtctggcttt
gtgtacaagttatttggggctcttaatttcaacaaatacttcgataacgataatagttca
atttctttaataaaggacaggtttagtatatctagctcaatggacgatatttag
>KrA6_psbE
atgagtacagctgaacgtgcattgattgatattgcacaagatcgtcgttattggataatt

cattcaattacaatcccttctttatttgttggtggagttatatttatgctgtctggcttt
gtgtacaagttatttggggctcttaatttcaacaaatacttcgataaggataatagttca
atctctttaataaaggacaggtttagtatatctagctcaatggacgatatttag
>KrA7_psbE
atgagtacagctgaacgtgcattgattgatattgcacaagatcgtcgttattggataatt
cattcaattacaataccttctttatttgttggtggagttatatttatgctgtctggcttt
gtgtacaagttatttggggctctaaatttcaacaaatacttcgataaggataatagttca
atctctttaataaaggacaggtttagtatatctagctcaatggacgatatttag
>KrA8_psbE
atgagtacagctgaacgtgcattgattgatattgcacaagatcgtcgttattggataatt
cattcaattacaataccttctttatttgttggtggagttatatttatgctgtctggcttt
gtgtacaagttatttggggctcttaatttcaacaaatacttcgataacgataatagttca
atttctttaataaaggacaggtttagtatatctagctcaatggacgatatttag
>KrA9_psbE
atgagtacagctgaacgtgcattgattgatattgcacaagatcgtcgttattggataatt
cattcaattacaatcccttctttatttgttggtggagttatatttatgctgtctggcttt
gtgtacaagttatttggggctctaaatttcaacaaatacttcgataaggataatagttca
atctctttaataaaggacaggtttagtatatctagctcaatggacgatatttag
>KrC1_psbE
atgagtacagctgaacgtgcattgattgatattgcacaagatcgtcgttattggataatt
cattcaattacaataccttctttatttgttggtggagttatatttatgctgtctggcttt
gtgtacaagttatttggggctctaaatttcaacaaatacttcgataaggataatagttca
atctctttaataaaggacaggtttagtatatctagctcaatggacgatatttag
>KrC10_psbE
atgagtacagctgaacgtgcattgattgatattgcacaagatcgtcgttattggataatt
cattcaattacaatcccttctttatttgttggtggagttatatttatgctgtctggcttt
gtgtacaagttatttggggctctaaatttcaacaaatacttcgataaggataatagttca
atctctttaataaaggacaggtttagtatatctagctcaatggacgatatttag
>KrC11_psbE
atgagtacagctgaacgtgcattgattgatattgcacaagatcgtcgctattggataatt
cattcaattacaataccttctttatttgttggtggagttatatttatgctgtctggcttt
gtgtacaagttatttggggctctaaatttcaacaaatacttcgataaggataatagttca
atctctttaataaaggacaggtttagtatatctagctcaatggacgatatttag
>KrC12_psbE
atgagtacagctgaacgtgcattgattgatattgcacaagatcgtcgttattggataatt
cattcaattacaatcccttctttatttgttggtggagttatatttatgctgtctggcttt
gtgtacaagttatttggggctctaaatttcaacaaatacttcgataaggataatagttca
atctctttaataaaggacaggtttagtatatctagctcaatggacgatatttag
>KrC13_psbE
atgagtacagctgaacgtgcattgattgatattgcacaagatcgtcgctattggataatt
cattcaattacaataccttctttatttgttggtggagttatatttatgctgtctggcttt
gtgtacaagttatttggggctctaaatttcaacaaatacttcgataaggataatagttca
atctctttaataaaggacaggtttagtatatctagctcaatggacgatatttag
>KrC14_psbE
atgagtacagctgaacgtgcattgattgatattgcacaagatcgtcgttattggataatt
cattcaattacaatcccttctttatttgttggtggagttatatttatgctgtctggcttt
gtgtacaagttatttggggctcttaatttcaacaaatacttcgataacgataatagttca
atttctttaataaaggacaggtttagtatatctagctcaatggacgatatttag
>KrC15_psbE
atgagtacagctgaacgtgcattgattgatattgcacaagatcgtcgttattggataatt
cattcaattacaataccttctttatttgttggtggagttatatttatgctgtctggcttt
gtgtacaagttatttggggctctaaatttcaacaaatacttcgataaggataatagttca
atctctttaataaaggacaggtttagtatatctagctcaatggacgatatttag
>KrC2_psbE
atgagtacagctgaacgtgcattgattgatattgcacaagatcgtcgttattggataatt
cattcaattacaataccttctttatttgttggtggagttatatttatgctgtctggcttt
gtgtacaagttatttggggctctaaatttcaacaaatacttcgataaggataatagttca

atctctttaataaaggacaggtttagtatatctagctcaatggacgatatttag
>KrC3_psbE
atgagtacagctgaacgtgcattgattgatattgcacaagatcgtcgctattggataatt
cattcaattacaataccttctttatttgttggtggagttatatttatgctgtctggcttt
gtgtacaagttatttggggctctaaatttcaacaaatacttcgataaggataatagttca
atctctttaataaaggacaggtttagtatatctagctcaatggacgatatttag
>KrC4_psbE
atgagtacagctgaacgtgcattgattgatattgcacaagatcgtcgttattggataatt
cattcaattacaatcccttctttatttgttggtggagttatatttatgctgtctggcttt
gtgtacaagttatttggggctctaaatttcaacaaatacttcgataaggataatagttca
atctctttaataaaggacaggtttagtatatctagctcaatggacgatatttag
>KrC5_psbE
atgagtacagctgaacgtgcattgattgatattgcacaagatcgtcgttattggataatt
cattcaattacaataccttctttatttgttggtggagttatatttatgctgtctggcttt
gtgtacaagttatttggggctctaaatttcaacaaatacttcgataaggataatagttca
atctctttaataaaggacaggtttagtatatctagctcaatggacgatatttag
>KrC6_psbE
atgagtacagctgaacgtgcattgattgatattgcacaagatcgtcgttattggataatt
cattcaattacaataccttctttatttgttggtggagttatatttatgctgtctggcttt
gtgtacaagttatttggggctctaaatttcaacaaatacttcgataaggataatagttca
atctctttaataaaggacaggtttagtatatctagctcaatggacgatatttag
>KrC7_psbE
atgagtacagctgaacgtgcattgattgatattgcacaagatcgtcgttattggataatt
cattcaattacaataccttctttatttgttggtggagttatatttatgctgtctggcttt
gtgtacaagttatttggggctctaaatttcaacaaatacttcgataaggataatagttca
atctctttaataaaggacaggtttagtatatctagctcaatggacgatatttag
>KrC8_psbE
atgagtacagctgaacgtgcattgattgatattgcacaagatcgtcgttattggataatt
cattcaattacaataccttctttatttgttggtggagttatatttatgctgtctggcttt
gtgtacaagttatttggggctctaaatttcaacaaatacttcgataaggataatagttca
atctctttaataaaggacaggtttagtatatctagctcaatggacgatatttag
>KrC9_psbE
atgagtacagctgaacgtgcattgattgatattgcacaagatcgtcgttattggataatt
cattcaattacaatcccttctttatttgttggtggagttatatttatgctgtctggcttt
gtgtacaagttatttggggctctaaatttcaacaaatacttcgataaggataatagttca
atctctttaataaaggacaggtttagtatatctagctcaatggacgatatttag
>KrD1_psbE
atgagtacagctgaacgtgcattgattgatattgcacaagatcgtcgttattggataatt
cattcaattacaatcccttctttatttgttggtggagttatatttatgctgtctggcttt
gtgtacaagttatttggggctcttaatttcaacaaatacttcgataacgataatagttca
atttctttaataaaggacaggtttagtatatctagctcaatggacgatatttag
>KrD11_psbE
atgagtacagctgaacgtgcattgattgatattgcacaagatcgtcgttattggataatt
cattcaattacaataccttctttatttgttgttggagttatatttatgctgtctggcttt
gtgtacaagttatttggggctcttaatttcaacaaatacttcgataacgataatagttca
atttctttaataaaggacaggtttagtatatctagctcaatggacgatatttag
>KrD13_psbE
atgagtacagctgaacgtgcattgattgatattgcacaagatcgtcgttattggataatt
cattcaattacaataccttctttatttgttggtggagttatatttatgctgtctggcttt
gtgtacaagttatttggggctctaaatttcaacaaatacttcgataaggataatagttca
atctctttaataaaggacaggtttagtatatctagctcaatggacgatatttag
>KrD3_psbE
atgagtacagctgaacgtgcattgattgatattgcacaagatcgtcgttattggataatt
cattcaattacaatcccttctttatttgttggtggagttatatttatgctgtctggcttt
gtgtacaagttatttggggctctaaatttcaacaaatacttcgataaggataatagttca
atctctttaataaaggacaggtttagtatatctagctcaatggacgatatttag
>KrD8_psbE

atgagtacagctgaacgtgcattgattgatattgcacaagatcgtcgttattggataatt
cattcaattacaataccttctttatttgttggtggagttatatttatgctgtctggcttt
gtgtacaagttatttggggctctaaatttcaacaaatacttcgataaggataatagttca
atctctttaataaaggacaggtttagtatatctagctcaatggacgatatttag
>KrD9_psbE
atgagtacagctgaacgtgcattgattgatattgcacaagatcgtcgttattggataatt
cattcaattacaatcccttctttatttgttggtggagttatatttatgctgtctggcttt
gtgtacaagttatttggggctcttaatttcaacaaatacttcgataacgataatagttca
atttctttaataaaggacaggtttagtatatctagctcaatggacgatatttag
>KrE1_psbE
atgagtacagctgaacgtgcattgattgatattgcacaagatcgtcgctattggataatt
cattcaattacaataccttctttatttgttggtggagttatatttatgctgtctggcttt
gtgtacaagttatttggggctctaaatttcaacaaatacttcgataaggataatagttca
atctctttaataaaggacaggtttagtatatctagctcaatggacgatatttag
>KrE10_psbE
atgagtacagctgaacgtgcattgattgatattgcacaagatcgtcgttattggataatt
cattcaattacaatcccttctttatttgttggtggagttatatttatgctgtctggcttt
gtgtacaagttatttggggctcttaatttcaacaaatacttcgataacgataatagttca
atttctttaataaaggacaggtttagtatatctagctcaatggacgatatttag
>KrE11_psbE
atgagtacagctgaacgtgcattgattgatattgcacaagatcgtcgttattggataatt
cattcaattacaataccttctttatttgttggtggagttatatttatgctgtctggcttt
gtgtacaagttatttggggctctaaatttcaacaaatacttcgataaggataatagttca
atctctttaataaaggacaggtttagtatatctagctcaatggacgatatttag
>KrE12_psbE
atgagtacagctgaacgtgcattgattgatattgcacaagatcgtcgttattggataatt
cattcaattacaataccttctttatttgttggtggagttatatttatgctgtctggcttt
gtgtacaagttatttggggctctaaatttcaacaaatacttcgataaggataatagttca
atctctttaataaaggacaggtttagtatatctagctcaatggacgatatttag
>KrE13_psbE
atgagtacagctgaacgtgcattgattgatattgcacaagatcgtcgttattggataatt
cattcaattacaataccttctttatttgttggtggagttatatttatgctgtctggcttt
gtgtacaagttatttggggctctaaatttcaacaaatacttcgataaggataatagttca
atctctttaataaaggacaggtttagtatatctagctcaatggacgatatttag
>KrE14_psbE
atgagtacagctgaacgtgcattgattgatattgcacaagatcgtcgttattggataatt
cattcaattacaataccttctttatttgttggtggagttatatttatgctgtctggcttt
gtgtacaagttatttggggctcttaatttcaacaaatacttcgataacgataatagttca
atttctttaataaaggacaggtttagtatatctagctcaatggacgatatttag
>KrE15_psbE
atgagtacagctgaacgtgcattgattgatattgcacaagatcgtcgttattggataatt
cattcaattacaataccttctttatttgttggtggagttatatttatgctgtctggcttt
gtgtacaagttatttggggctctaaatttcaacaaatacttcgataaggataatagttca
atctctttaataaaggacaggtttagtatatctagctcaatggacgatatttag
>KrE2_psbE
atgagtacagctgaacgtgcattgattgatattgcacaagatcgtcgttattggataatt
cattcaattacaataccttctttatttgttggtggagttatatttatgctgtctggcttt
gtgtacaagttatttggggctcttaatttcaacaaatacttcgataacgataatagttca
atctctttaataaaggacaggtttagtatatctagctcaatggacgatatttag
>KrE3_psbE
atgagtacagctgaacgtgcattgattgatattgcacaagatcgtcgttattggataatt
cattcaattacaataccttctttatttgttggtggagttatatttatgctgtctggcttt
gtgtacaagttatttggggctctaaatttcaacaaatacttcgataatgataatagttca
atctatttaataaaggacaggtttagtatatctagctcaatggacgatatttag
>KrE4_psbE
atgagtacagctgaacgtgcattgattgatattgcacaagatcgtcgttattggataatt
cattcaattacaatcccttctttatttgttggtggagttatatttatgctgtctggcttt

gtgtacaagttatttggggctcttaatttcaacaaatacttcgataacgataatagttca
atttctttaataaaggacaggtttagtatatctagctcaatggacgatatttag
>KrE5_psbE
atgagtacagctgaacgtgcattgattgatattgcacaagatcgtcgttattggataatt
cattcaattacaataccttctttatttgttggtggagttatatttatgctgtctggcttt
gtgtacaagttatttggggctctaaatttcaacaaatacttcgataaggataatagttca
atctctttaataaaggacaggtttagtatatctagctcaatggacgatatttag
>KrE6_psbE
atgagtacagctgaacgtgcattgattgatattgcacaagatcgtcgttattggataatt
cattcaattacaatcccttctttatttgttggtggagttatatttatgctgtctggcttt
gtgtacaagttatttggggctctaaatttcaacaaatacttcgataaggataatagttca
atctctttaataaaggacaggtttagtatatctagctcaatggacgatatttag
>KrE7_psbE
atgagtacagctgaacgtgcattgattgatattgcacaagatcgtcgttattggataatt
cattcaattacaatcccttctttatttgttggtggagttatatttatgctgtctggcttt
gtgtacaagttatttggggctcttaatttcaacaaatacttcgataacgataatagttca
atttctttaataaaggacaggtttagtatatctagctcaatggacgatatttag
>KrE8_psbE
atgagtacagctgaacgtgcattgattgatattgcacaagatcgtcgttattggataatt
cattcaattacaataccttctttatttgttggtggagttatatttatgctgtctggcttt
gtgtacaagttatttggggctctaaatttcaacaaatacttcgataaggataatagttca
atctctttaataaaggacaggtttagtatatctagctcaatggacgatatttag
>KrE9_psbE
atgagtacagctgaacgtgcattgattgatattgcacaagatcgtcgttattggataatt
cattcaattacaataccttctttatttgttggtggagttatatttatgctgtctggcttt
gtgtacaagttatttggggctctaaatttcaacaaatacttcgataaggataatagttca
atctctttaataaaggacaggtttagtatatctagctcaatggacgatatttag
>Mz10_psbE
atgagtacagctgaacgtgcattgattgatattgcacaagatcgtcgttattggataatt
cattcaattacaatcccttctttatttgttggtggagttatatttatgctgtctggcttt
gtgtacaagttatttggggctcttaatttcaacaaatacttcgataacgataatagttca
atctctttaataaaggacaggtttagtatatctagctcaatggacgatatttag
>Mz5_psbE
atgagtacagctgaacgtgcattgattgatattgcacaagatcggcgttattggataatt
cattcaattacaataccttctttatttgttggtggagttatatttatgctgtctggcttt
gtgtacaagttatttggggctctaaatttcaacaaatacttcgataaggataatagttca
atctctttaataaaggacaggtttagtatatctagctcaatggacgatatttag
>Mz6_psbE
atgagtacagctgaacgtgcattgattgatattgcacaagatcggcgttattggataatt
cattcaattacaataccttctttatttgttggtggagttatatttatgctgtctggcttt
gtgtacaagttatttggggctctaaatttcaacaaatacttcgataacgataatagttca
atctctttaataaaggacaggtttagtatatctagctcaatggacgatatttag
>Mz7_psbE
atgagtacagctgaacgtgcattgattgatattgcacaagatcggcgttattggataatt
cattcaattacaataccttctttatttgttggtggagttatatttatgctgtctggcttt
gtttacaagttatttggggctcttaatttcaacaaatacttcgataaggataatagttca
atctctttaataaaggacaggtttagtatatctagctcaatggacgatatttag
>Mz8_psbE
atgagtacagctgaacgtgcattgattgatattgcacaagatcggcgttattggataatt
cattcaattacaatcccttctttatttgttggtggagttatatttatgctgtctggcttt
gtgtacaagttatttggggctcttaatttcaacaaatacttcgataacgataatagttca
atttctttaataaaggacaggtttagtatatctagctcaatggacgatatttag
>Mz9_psbE
atgagtacagctgaacgtgcattgattgatattgcacaagatcgtcgttattggataatt
cattcaattacaatcccttctttatttgttggtggagttatatttatgctgtctggcttt
gtgtacaagttatttggggctcttaatttcaacaaatacttcgataacgataatagttca
atttctttaataaaggacaggtttagtatatctagctcaatggacgatatttag

>MzC1_psbE
atgagtacagctgaacgtgcattgattgatattgcacaagatcggcgttattggataatt
cattcaattacaataccttctttatttgttggtggagttatatttatgctgtctggcttt
gtgtacaagttatttggggctctaaatttcaacaaatacttcgataaggataatagttca
atctctttaataaaggacaggtttagtatatctagctcaatggacgatatttag
>MzC1GaII_psbE
atgagtacagctgaacgtgcattgattgatattgcacaagatcggcgttattggataatt
cattcaattacaataccttctttatttgttggtggagttatatttatgctgtctggcttt
gtgtacaagttatttggggctctaaatttcaacaaatacttcgataaggataatagttca
atctctttaataaaggacaggtttagtatatctagctcaatggacgatatttag
>MzC2_psbE
atgagtacagctgaacgtgcattgattgatattgcacaagatcggcgttattggataatt
cattcaattacaataccttctttatttgttggtggagttatatttatgctgtctggcttt
gtgtacaagttatttggggctctaaatttcaacaaatacttcgataaggataatagttca
atctctttaataaaggacaggtttagtatatctagctcaatggacgatatttag
>MzC2GaII_psbE
atgagtacagctgaacgtgcattgattgatattgcacaagatcggcgttattggataatt
cattcaattacaataccttctttatttgttggtggagttatatttatgctgtctggcttt
gtgtacaagttatttggggctctaaatttcaacaaatacttcgataaggataatagttca
atctctttaataaaggacaggtttagtatatctagctcaatggacgatatttag
>MzC3_psbE
atgagtacagctgaacgtgcattgattgatattgcacaagatcggcgttattggataatt
cattcaattacaataccttctttatttgttggtggagttatatttatgctgtctggcttt
gtgtacaagttatttggggctctaaatttcaacaaatacttcgataaggataatagttca
atctctttaataaaggacaggtttagtatatctagctcaatggacgatatttag
>MzC4_psbE
atgagtacagctgaacgtgcattgattgatattgcacaagatcggcgttattggataatt
cattcaattacaataccttctttatttgttggtggagttatatttatgctgtctggcttt
gtgtacaagttatttggggctctaaatttcaacaaatacttcgataaggataatagttca
atctctttaataaaggacaggtttagtatatctagctcaatggacgatatttag
>Od10_psbE
atgagtacagctgaacgtgcattgattgatattgcacaagatcgtcgttattggataatt
cattcaattacaataccttctttatttgttggtggagttatatttatgctgtctggcttt
gtgtacaagttatttggggctctaaatttcaacaaatacttcgataaggataatagttca
atctctttaataaaggacaggtttagtatatctagctcaatggacgatatttag
>Od11_psbE
atgagtacagctgaacgtgcattgattgatattgcacaagatcgtcgttattggataatt
cattcaattacaataccttctttatttgttggtggagttatatttatgctgtctggcttt
gtgtacaagttatttggggctctaaatttcaacaaatacttcgataaggataatagttca
atctctttaataaaggacaggtttagtatatctagctcaatggacgatatttag
>Od12_psbE
atgagtacagctgaacgtgcattgattgatattgcacaagatcgtcgttattggataatt
cattcaattacaataccttctttatttgttggtggagttatatttatgctgtctggcttt
gtgtacaagttatttggggctctaaatttcaacaaatacttcgataaggataatagttca
atttctttaataaaggacaggtttagtatatctagctcaatggacgatatttag
>Od13_psbE
atgagtacagctgaacgtgcattgattgatattgcacaagatcgtcgttattggataatt
cattcaattacaataccttctttatttgttggtggagttatatttatgctgtctggcttt
gtgtacaagttatttggggctctaaatttcaacaaatacttcgataaggataatagttca
atctctttaataaaggacaggtttagtatatctagctcaatggacgatatttag
>Od14_psbE
atgagtacagctgaacgtgcattgattgatattgcacaagatcgtcgttattggataatt
cattcaattacaataccttctttatttgttggtggagttatatttatgctgtctggcttt
gtgtacaagttatttggggctctaaatttcaacaaatacttcgataaggataatagttca
atctctttaataaaggacaggtttagtatatctagctcaatggacgatatttag
>Od15_psbE
atgagtacagctgaacgtgcattgattgatattgcacaagatcgtcgttattggataatt

cattcaattacaataccttctttatttgttggtggagttatatttatgctgtctggcttt
gtgtacaagttatttggggctctaaatttcaacaaatacttcgataaggataatagttca
atctctttaataaaggacaggtttagtatatctagctcaatggacgatatttag
>Od18_psbE
atgagtacagctgaacgtgcattgattgatattgcacaagatcgtcgttattggataatt
cattcaattacaataccttctttatttgttggtggagttatatttatgctgtctggcttt
gtgtacaagttatttggggctctaaatttcaacaaatacttcgataaggataatagttca
atctctttaataaaggacaggtttagtatatctagctcaatggacgatatttag
>Od19_psbE
atgagtacagctgaacgtgcattgattgatattgcacaagatcgtcgttattggataatt
cattcaattacaataccttctttatttgttggtggagttatatttatgctgtctggcttt
gtgtacaagttatttggggctctaaatttcaacaaatacttcgataaggataatagttca
atctctttaataaaggacaggtttagtatatctagctcaatggacgatatttag
>Od2_psbE
atgagtacagctgaacgtgcattgattgatattgcacaagatcgtcgttattggataatt
cattcaattacaataccttctttatttgttggtggagttatatttatgctgtctggcttt
gtgtacaagttatttggggctctaaatttcaacaaatacttcgataaggataatagttca
atctctttaataaaggacaggtttagtatatctagctcaatggacgatatttag
>Od22_psbE
atgagtacagctgaacgtgcattgattgatattgcacaagatcgtcgttattggataatt
cattcaattacaataccttctttatttgttggtggagttatatttatgctgtctggcttt
gtgtacaagttatttggggctctaaatttcaacaaatacttcgataaggataatagttca
atctctttaataaaggacaggtttagtatatctagctcaatggacgatatttag
>Od23_psbE
atgagtacagctgaacgtgcattgattgatattgcacaagatcgtcgttattggataatt
cattcaattacaataccttctttatttgttggtggagttatatttatgctgtctggcttt
gtgtacaagttatttggggctctaaatttcaacaaatacttcgataaggataatagttca
atctctttaataaaggacaggtttagtatatctagctcaatggacgatatttag
>Od24_psbE
atgagtacagctgaacgtgcattgattgatattgcacaagatcgtcgttattggataatt
cattcaattacaataccttctttatttgttggtggagttatatttatgctgtctggcttt
gtgtacaagttatttggggctcttaatttcaacaaatacttcgataacgataatagttca
atttctttaataaaggacaggtttagtatatctagctcaatggacgatatttag
>Od25_psbE
atgagtacagctgaacgtgcattgattgatattgcacaagatcgtcgttattggataatt
cattcaattacaataccttctttatttgttggtggagttatatttatgctgtctggcttt
gtgtacaagttatttggggctctaaatttcaacaaatacttcgataaggataatagttca
atctctttaataaaggacaggtttagtatatctagctcaatggacgatatttag
>Od26_psbE
atgagtacagctgaacgtgcattgattgatattgcacaagatcgtcgttattggataatt
cattcaattacaataccttctttatttgttggtggagttatatttatgctgtctggcttt
gtgtacaagttatttggggctctaaatttcaacaaatacttcgataaggataatagttca
atctctttaataaaggacaggtttagtatatctagctcaatggacgatatttag
>Od27_psbE
atgagtacagctgaacgtgcattgattgatattgcacaagatcgtcgttattggataatt
cattcaattacaataccttctttatttgttggtggagttatatttatgctgtctggcttt
gtgtacaagttatttggggctctaaatttcaacaaatacttcgataaggataatagttca
atctctttaataaaggacaggtttagtatatctagctcaatggacgatatttag
>Od4_psbE
atgagtacagctgaacgtgcattgattgatattgcacaagatcgtcgttattggataatt
cattcaattacaataccttctttatttgttggtggagttatatttatgctgtctggcttt
gtgtacaagttatttggggctctaaatttcaacaaatacttcgataaggataatagttca
atctctttaataaaggacaggtttagtatatctagctcaatggacgatatttag
>Od6_psbE
atgagtacagctgaacgtgcattgattgatattgcacaagatcgtcgttattggataatt
cattcaattacaataccttctttatttgttggtggagttatatttatgctgtctggcttt
gtgtacaagttatttggggctctaaatttcaacaaatacttcgataaggataatagttca

atctctttaataaaggacaggtttagtatatctagctcaatggacgatatttag
>Od8_psbE
atgagtacagctgaacgtgcattgattgatattgcacaagatcgtcgttattggataatt
cattcaattacaataccttctttatttgttggtggagttatatttatgctgtctggcttt
gtgtacaagttatttggggctctaaatttcaacaaatacttcgataaggataatagttca
atctctttaataaaggacaggtttagtatatctagctcaatggacgatatttag
>Od9_psbE
atgagtacagctgaacgtgcattgattgatattgcacaagatcgtcgttattggataatt
cattcaattacaataccttctttatttgttggtggagttatatttatgctgtctggcttt
gtgtacaagttatttggggctctaaatttcaacaaatacttcgataaggataatagttca
atctctttaataaaggacaggtttagtatatctagctcaatggacgatatttag
>Ss5_psbE
atgagtacagctgaacgtgcattgattgatattgcacaagatcgtcgttattggataatt
cattcaattacaataccttctttatttgttggtggagttatatttatgctgtctggcttt
gtgtacaagttatttggggctcttaatttcaacaaatacttcgataaggataatagttca
atctctttaataaaggacaggtttagtatatctagctcaatggacgatatttag
>Ss6_psbE
atgagtacagctgaacgtgcattgattgatattgcacaagatcgtcgttattggataatt
cattcaattacaataccttctttatttgttggtggagttatatttatgctgtctggcttt
gtgtacaagttatttggggctcttaatttcaacaaatacttcgataaggataatagttca
atctctttaataaaggacaggtttagtatatctagctcaatggacgatatttag
>Ss7_psbE
atgagtacagctgaacgtgcattgattgatattgcacaagatcgtcgttattggataatt
cattcaattacaataccttctttatttgttggtggagttatatttatgctgtctggcttt
gtgtacaagttatttggggctctaaatttcaacaaatacttcgataaggataatagttca
atctctttaataaaggacaggtttagtatatctagctcaatggacgatatttag
>Ss8_psbE
atgagtacagctgaacgtgcattgattgatattgcacaagatcgtcgttattggataatt
cattcaattacaataccttctttatttgttggtggagttatatttatgctgtctggcttt
gtgtacaagttatttggggctctaaatttcaacaaatacttcgataaggataatagttca
atctctttaataaaggacaggtttagtatatctagctcaatggacgatatttag
>ohdo1_psbE
atgagtacagctgaacgtgcattgattgatatttcacaagatcgtcgttattggataatt
cattcaattacaataccttctttatttgttggtggagttatatttatgctgtctggcttt
gtgtacaagttatttggggctctaaatttcaacaaatacttcgataaggataatagttca
atatctttaataaaggacaggtttagtatatctagctcaatggacgatatttag
>ohdo3_psbE
atgagtacagctgaacgtgcattgattgatattgcacaagatcgtcgttattggataatt
cattcaattacaataccttctttatttgttggtggagttatatttatgctgtctggcttt
gtgtacaagttatttggggctcttaatttcaacaaatacttcgataacgataatagttca
atttctttaataaaggacaggtttagtatatctagctcaatggacgatatttag
>ohdo5_psbE
atgagtacagctgaacgtgcattgattgatattgcacaagatcgtcgttattggataatt
cattcaattacaataccttctttatttgttggtggagttatatttatgctgtctggcttt
gtgtacaagttatttggggctctaaatttcaacaaatacttcgataaggataatagttca
atctctttaataaaggacaggtttagtatatctagctcaatggacgatatttag
>ohdo7_psbE
atgagtacagctgaacgtgcattgattgatattgcacaagatcgtcgttattggataatt
cattcaattacaataccttctttatttgttggtggagttatatttatgctgtctggcttt
gtgtacaagttatttggggctctaaatttcaacaaatacttcgataaggataatagttca
atctctttaataaaggacaggtttagtatatctagctcaatggacgatatttag
>sesoko1_psbE
atgagtacagctgaacgtgcattgattgatattgcacaagatcgtcgttattggataatt
cattcaattacaataccttctttatttgttggtggagttatatttatgctgtctggcttt
gtgtacaagttatttggggctctaaatttcaacaaatacttcgataaggataatagttca
atctctttaataaaggacaggtttagtatatctagctcaatggacgatatttag
>sesoko3_psbE

atgagtacagctgaacgtgcattgattgatattgcacaagatcgtcgttattggataatt
cattcaattacaataccttctttatttgttggtggagttatatttatgctgtctggcttt
gtgtacaagttatttggggctctaaatttcaacaaatacttcgataaggataatagttca
atctctttaataaaggacaggtttagtatatctagctcaatggacgatatttag
>sesoko4_psbE
atgagtacagctgaacgtgcattgattgatattgcacaagatcgtcgttattggataatt
cattcaattacaataccttctttatttgttggtggagttatatttatgctgtctggcttt
gtgtacaagttatttggggctctaaatttcaacaaatacttcgataaggataatagttca
atctctttaataaaggacaggtttagtatatctagctcaatggacgatatttag
>REF_DNA_psbE_JX094328
ATGAGTACAGCTGAACGTGCATTGATTGATATTGCACAAGATCGTCGTTATTGGATAATT
CATTCAATTACAATCCCTTCTTTATTTGTTGGTGGAGTTATATTTATGCTGTCTGGCTTT
GTGTACAAGTTATTTGGGGCTCTTAATTTCAACAAATACTTCGATAACGATAATAGTTCA
ATTTCTTTAATAAAGGACAGGTTTAGTATATCTAGCTCAATGGACGATATTTAG
>REF_RNA_psbE_symbB1.comp2_c0_seq1
ATGAGTACAGCTGAACGTCCATTGATTGATATTGTGCAAGATCGTCGTTATTGGATAATT
CATTCAATTACAATCCCTTCTTTATTTATTGGTGGAGTTATATTTGTGCTGTCTGGCTTT
GTGTACAAGTTATTTGGGGCTCTTAATTTCAACAACTACTTCGATAACGATAATAGTTCA
ATTTCTTTAGTAAACGACAGGTTTAGTGTATCTAGCTCAATGGACGATATTTAG

>Hd1_psbI
ttgctcaggtcttgtgtctattttgtgcttgctctatcaataagcttatttatcgctggc
tttttatcaggagatttgtctaggtcctcttctaatgctgcaatttaa
>Hd2_psbI
ttgctcaggtcttgtgtctattttgtgcttgctctttcatgcagcttatttatcgctggc
tttttatcaggagatttgtctaggtcctcttctaatgctgcaatttaa
>Hd3_psbI
ttgctcaggtcttgtgtctattttgtgcttgctctttcatgcagcttatttattgctggc
tttttatcaggagatttgtctaggtcctcttctaatgctgcaatttaa
>Hd4_psbI
ttgctcaggtcttgtgtctattttgtgcttgctctttcatgcagcttatttattgctggc
tttttatcaggagatttgtctaggtcctcttccaatgctgcattttaa
>Hd5_psbI
ttgctcaggtcttgtgtctattttgggattgctctatcaatcagcttatttatcgctggc
tttttatcaggagatttgtctaggtcctcttctaatgctgcattttaa
>Hd6_psbI
ttgctcaggtcttgtgtctattttgggattgctctatcaataagcttatttatcgctggc
tttttatcaggagatttgtctaggtcctcttctaatgctgcaatttaa
>Hd7_psbI
ttgctcaggtcttgtgtctattttgtgattgctctatcaataagcttatttatcgctggc
tttttatcaggagatttgtctaggtcctcttctaatgctgcattttaa
>Hd8_psbI
ttgctcaggtcttgtgtctattttgtgcttgctctttcatgcagcttatttattgctggc
tttttatcaggagatttgtctaggtcctcttccaatgctgcattttaa
>Hd9_psbI
ttgctcaggtcttgtgtctattttgtgcttgctctttcatgcagcttatttattgctggc
tttttatcaggagatttgtctaggtcctcttctaatgctgcaatttaa
>IS1_psbI
ttgctcaggtcttgtgtctattttgtgcttgctctttcatgcagcttatttattgctggc
tttttatcaggagatttgtctaggtcctcttccaatgctgcattttaa
>IS2_psbI
ttgctcaggtcttgtgtctattttgtgcttgctctatcaataagcttatttatcgctggc
tttttatcaggagatttgtctaggtcctcttctaatgctgcaatttaa
>IS3_psbI
ttgctcaggtcttgtgtctattttgtgcttgctctttcatgcagtttatttattgctggc
tttttatcaggagatttgtctagatcctcttcaaacgctgcattttaa
>IS4_psbI
ttgctcaggtcttgtgtctattttgtgattgctctatcatgcagtttatttattgctggc
tttttatcaggagatttgtctagatcctcttccaatgctgcattttaa
>IS5_psbI
ttgctcaggtcttgtgtctattttgtgcttgctctttcatgcagcttatttatcgctggc
tttttatcaggagatttgtctaggtcctcttctaatgctgcaatttaa
>IS6_psbI
ttgctcaggtcttgtgtctattttgtgcttgctctttcaagaagcttatttatcgctggc
tttttatcaggagatttgtctaggtcctcttctaatgctgcaatttaa
>IS7_psbI
ttgctcaggtcttgtgtctattttgtgcttgctctttcatgcagcttatttattgctggc
tttttatcaggagatttgtctagatcctcttccaatgctgcattttaa
>IS8_psbI
ttgctcaggtcttgtgtctattttgtgcttgctctttcatgcagcttatttattgctggc
tttttatcaggagatttgtctaggtcctcttctaatgctgcaatttaa
>IS9_psbI
ttgctcaggtcttgtgtctattttgtgcttgctctttcatgcagtttatttattgctggc
tttttatcaggagatttgtctagatcctcttccaatgctgcattttaa
>Ik1_psbI
ttgctcaggtcttgtgtctattttgtgcttgctctttcatgcagcttatttatcgctggc
tttttatcaggagatttgtctaggtcctcttccaatgctgcattttaa

>Ik2_psbI
ttgctcaggtcttgtgtctattttgggattgctctatcaagcagcttatttatcgctggc
tttttatcaggagatttgtctaggtcctcttctaatgctgcattttaa
>Ik3_psbI
ttgctcaggtcttgtgtctattttgtgcttgctctttcatgcagcttatttattgctggc
tttttatcaggagatttgtctaggtcctcttccaatgctgcattttaa
>Ik4_psbI
ttgctcaggtcttgtgtctattttgtgcttgctctttcatgcagcttatttatcgctggc
tttttatcaggagatttgtctaggtcctcttctaatgctgcattttaa
>Ik5_psbI
ttgctcaggtcttgtgtctattttgtgattgctctatcaataagcttatttatcgctggc
tttttatcaggagatttgtctaggtcctcttctaatgctgcattttaa
>Ik6_psbI
ttgctcaggtcttgtgtctattttgggcttgctctatcaataagcttatttatcgctggc
tttttatcaggagatttgtctaggtcctcttctaatgctgcattttaa
>Ik7_psbI
ttgctcaggtcttgtgtctattttgtgcttgctctttcatgcagcttatttattgctggc
tttttatcaggagatttgtctaggtcctcttccaatgctgcattttaa
>Ik8_psbI
ttgctcaggtcttgtgtctattttgtgcttgctctttcatgcagcttatttattgctggc
tttttatcaggagatttgtctaggtcctcttccaatgctgcattttaa
>Ik9_psbI
ttgctcaggtcttgtgtctattttgtgcttgctctttcatgcagcttatttattgctggc
tttttatcaggagatttgtctaggtcctcttccaatgctgcattttaa
>Irm10_psbI
ttgctcaggtcttgtgtctattttgtgcttgctctttcatgcagcttatttattgctggc
tttttatcaggagatttgtctaggtcctcttccaatgctgcattttaa
>Irm17_psbI
ttgctcaggtcttgtgtctattttgtgcttgctctttcatgcagcttatttattgctggc
tttttatcaggagatttgtctaggtcctcttccaatgctgcattttaa
>Irm2_psbI
ttgctcaggtcttgtgtctattttgtgcttgctctttcatgcagcttatttatcgctggc
tttttatcaggagatttgtctaggtcctcttctaatgctgcaatttaa
>Irm21_psbI
ttgctcaggtcttgtgtctattttgtgcttgctctttcatgcagcttatttattgctggc
tttttatcaggagatttgtctaggtcctcttccaatgctgcattttaa
>Irm22_psbI
ttgctcaggtcttgtgtctattttgtgcttgctctttcatgcagcttatttattgctggc
tttttatcaggagatttgtctaggtcctcttccaatgctgcattttaa
>Irm23_psbI
ttgctcaggtcttgtgtctattttgtgcttgctctttcatgcagcttatttattgctggc
tttttatcaggagatttgtctaggtcctcttccaatgctgcattttaa
>Irm24_psbI
ttgctcaggtcttgtgtctattttgtgcttgctctttcatgcagcttatttattgctggc
tttttatcaggagatttgtctaggtcctcttccaatgctgcattttaa
>Irm25_psbI
ttgctcaggtcttgtgtctattttgtgcttgctctttcatgcagcttatttattgctggc
tttttatcaggagatttgtctaggtcctcttccaatgctgcattttaa
>Irm26_psbI
ttgctcaggtcttgtgtctattttgtgcttgctctttcatgcagcttatttattgctggc
tttttatcaggagatttgtctaggtcctcttccaatgctgcattttaa
>Irm27_psbI
ttgctcaggtcttgtgtctattttgtgcttgctctttcatgcagcttatttattgctggc
tttttatcaggagatttgtctagatcctcttccaatgctgcattttaa
>Irm3_psbI
ttgctcaggtcttgtgtatattttgtgcttgctctgtcctgcagtttatttattgctggc
tttttatcaggagatttgtctagatcctcttccaatgctgcattttaa

>Irm4_psbI
ttgctcaggtcttgtgtctattttgtgcttgctctttcatgcagtttatttattgctggc
tttttatcaggagatttgtctagatcctcttcaaacgctgcattttaa
>Irm5_psbI
ttgctcaggtcttgtgtctattttgtgcttgctctttcatgcagcttatttattgctggc
tttttatcaggagatttgtctaggtcctcttccaatgctgcattttaa
>Irm7_psbI
ttgctcaggtcttgtgtctattttgtgcttgctctttcatgcagcttatttattgctggc
tttttatcaggagatttgtctagatcctcttccaatgctgcattttaa
>Irm9_psbI
ttgctcaggtcttgtgtctattttgtgcttgctctttcatgcagtttatttattgctggc
tttttatcaggagatttgtctagatcctcttccaatgctgcattttaa
>Isy12_psbI
ttgctcaggtcttgtgtctattttgggattgctctatcaataagcttatttatcgctggc
tttttatcaggagatttgtctaggtcctcttctaatgctgcaatttaa
>Isy15_psbI
ttgctcaggtcttgtgtctattttgtgcttgctctgtcctgcagtttatttattgctggc
tttttatcaggagatttgtctagatcctcttccaatgctgcattttaa
>Isy16_psbI
ttgctcaggtcttgtgtctattttgggattgctctatcaataagcttatttatcgctggc
tttttatcaggagatttgtctaggtcctcttctaatgctgcattttaa
>Isy17_psbI
ttgctcaggtcttgtgtctattttgggattgctctatcaataagcttatttatcgctggc
tttttatcaggagatttgtctaggtcctcttctaatgctgcaatttaa
>Isy18_psbI
ttgctcaggtcttgtgtctattttgtgcttgctctttcatgcagcttatttattgctggc
tttttatcaggagatttgtctaggtcctcttccaatgctgcattttaa
>Isy21_psbI
ttgctcaggtcttgtgtctattttgtgcttgctctttcatgcagcttatttatcgctggc
tttttatcaggagatttgtctaggtcctcttccaatgctgcattttaa
>Isy22_psbI
ttgctcaggtcttgtgtctattttgggattgctctatcaataagcttatttatcgctggc
tttttatcaggagatttgtctaggtcctcttccaatgctgcattttaa
>Isy23_psbI
ttgctcaggtcttgtgtctattttgggattgctctatcaataagcttatttatcgctggc
tttttatcaggagatttgtctaggtcctcttccaatgctgcattttaa
>Isy24_psbI
ttgctcaggtcttgtgtctattttgtgattgctctatcaataagcttatttatcgctggc
tttttatcaggagatttgtctaggtcctcttctaatgctgcattttaa
>Isy25_psbI
ttgctcaggtcttgtgtctattttgtgcttgctctttcatgcagcttatttattgctggc
tttttatcaggagatttgtctaggtcctcttccaatgctgcattttaa
>Isy26_psbI
ttgctcaggtcttgtgtctattttgtgcttgctctttcatgcagcttatttattgctggc
tttttatcaggagatttgtctaggtcctcttccaatgctgcattttaa
>Isy27_psbI
ttgctcaggtcttgtgtctattttgtgattgctctatcatgaagcttatttattgctggc
tttttatcaggagatttgtctaggtcctcttccaatgctgcattttaa
>Isy4_psbI
ttgctcaggtcttgtgtctattttgggattgctctatcaataagcttatttatcgctggc
tttttatcaggagatttgtctaggtcctcttctaatgctgcattttaa
>Isy7_psbI
ttgctcaggtcttgtgtctattttgtgcttgctctttcatgcagcttatttattgctggc
tttttatcaggagatttgtctaggtcctcttctaatgctgcaatttaa
>Isy8_psbI
ttgctcaggtcttgtgtctattttgtgcttgctctttcatgcagcttatttatcgctggc
tttttatcaggagatttgtctaggtcctcttccaatgctgcattttaa

>KrA1_psbI
ttgctcaggtcttgtgtctattttgggattgctctatcaataagcttatttatcgctggc
tttttatcaggagatttgtctaggtcctcttctaatgctgcaatttaa
>KrA10_psbI
ttgcttaggtcttgtgtctattttgggattgctctatcaataagcttatttatcgctggc
tttttatcaggagatttgtctaggtcctcttctaatgctgcaatttaa
>KrA11_psbI
ttgctcaggtcttgtgtctattttgtgcttgctctttcatgcagcttatttattgctggc
tttttatcaggagatttgtctaggtcctcttctaatgctgcaatttaa
>KrA12_psbI
ttgctcaggtcttgtgtctattttgtgcttgctctttcatgcagcttatttatcgctggc
tttttatcaggagatttgtctaggtcctcttctaatgctgcaatttaa
>KrA13_psbI
ttgctcaggtcttgtgtctattttgtgcttgctctttcatgcagcttatttattgctggc
tttttatcaggagatttgtctaggtcctcttccaatgctgcattttaa
>KrA14_psbI
ttgctcaggtcttgtgtctattttgtgcttgctctttcatgcagcttatttattgctggc
tttttatcaggagatttgtctaggtcctcttccaatgctgcattttaa
>KrA15_psbI
ttgctcaggtcttgtgtctattttgtgcttgctctttcatgcagcttatttattgctggc
tttttatcaggagatttgtctaggtcctcttccaatgctgcattttaa
>KrA2_psbI
ttgctcaggtcttgtgtctattttgggattgctctatcaataagcttatttatcgctggc
tttttatcaggagatttgtctaggtcctcttctaatgctgcaatttaa
>KrA3_psbI
ttgctcaggtcttgtgtctattttgggattgctctatcaataagcttatttatcgctggc
tttttatcaggagatttgtctaggtcctcttctaatgctgcattttaa
>KrA4_psbI
ttgctcaggtcttgtgtctattttgtgcttgctctttcatgcagcttatttatcgctggc
tttttatcaggagatttgtctaggtcctcttctaatgctgcaatttaa
>KrA5_psbI
ttgctcaggtcttgtgtctattttgtgcttgctctttcatgcagcttatttattgctggc
tttttatcaggagatttgtctaggtcctcttccaatgctgcattttaa
>KrA6_psbI
ttgctcaggtcttgtgtctattttgtgcttgctctttcatgcagcttatttattgctggc
tttttatcaggagatttgtctaggtcctcttccaatgctgcattttaa
>KrA7_psbI
ttgctcaggtcttgtgtctattttgtgcttgctctttcatgcagcttatttattgctggc
tttttatcaggagatttgtctaggtcctcttccaatgctgcattttaa
>KrA8_psbI
ttgctcaggtcttgtgtctattttgtgcttgctctttcatgcagcttatttattgctggc
tttttatcaggagatttgtctaggtcctcttccaatgctgcattttaa
>KrA9_psbI
ttgctcaggtcttgtgtctattttgtgattgctctatcattaagcttatttattgctggc
tttttatcaggagatttgtctaggtcctcttctaatgctgcattttaa
>KrC1_psbI
ttgctcaggtcttgtgtctattttgggattgctctatcaataagcttatttatcgctggc
tttttatcaggagatttgtctaggtcctcttccaatgctgcattttaa
>KrC10_psbI
ttgctcaggtcttgtgtctattttgtgcttgctctttcatgcagcttatttattgctggc
tttttatcaggagatttgtctaggtcctcttccaatgctgcattttaa
>KrC11_psbI
ttgcttaggtcttgtgtctattttgggattgctctatcaataagcttatttatcgctggc
tttttatcaggagatttgtctaggtcctcttctaatgctgcattttaa
>KrC12_psbI
ttgctcaggtcttgtgtctattttgtgcttgctctttcatgcagcttatttattgctggc
tttttatcaggagatttgtctaggtcctcttccaatgctgcattttaa

>KrC13_psbI
ttgctcaggtcttgtgtctattttgtgcttgctctttcatgcagcttatttatcgctggc
tttttatcaggagatttgtctaggtcctcttctaatgctgcaatttaa
>KrC14_psbI
ttgctcaggtcttgtgtctattttgtgcttgctctttcatgcagcttatttattgctggc
tttttatcaggagatttgtctaggtcctcttccaatgctgcattttaa
>KrC15_psbI
ttgctcaggtcttgtgtctattttgggattgctctatcaataagcttatttatcgctggc
tttttatcaggagatttgtctaggtcctcttctaatgctgcaatttaa
>KrC2_psbI
ttgctcaggtcttgtgtctattttgtgcttgctctttcatgcagcttatttattgctggc
tttttatcaggagatttgtctaggtcctcttccaatgctgcattttaa
>KrC3_psbI
ttgctcaggtcttgtgtctattttgggattgctctatcaataagcttatttatcgctggc
tttttatcaggagatttgtctaggtcctcttctaatgctgcaatttaa
>KrC4_psbI
ttgctcaggtcttgtgtctattttgggattgctctatcaataagcttatttatcgctggc
tttttatcaggagatttgtctaggtcctcttctaatgctgcaatttaa
>KrC5_psbI
ttgctcaggtcttgtgtctattttgggattgctctatcaataagcttatttatcgctggc
tttttatcaggagatttgtctaggtcctcttctaatgctgcaatttaa
>KrC6_psbI
ttgctcaggtcttgtgtctattttgtgcttgctctttcatgcagcttatttattgctggc
tttttatcaggagatttgtctaggtcctcttccaatgctgcattttaa
>KrC7_psbI
ttgctcaggtcttgtgtctattttgtgcttgctctttcatgcagcttatttatcgctggc
tttttatcaggagatttgtctaggtcctcttctaatgctgcaatttaa
>KrC8_psbI
ttgctcaggtcttgtgtctattttgggattgctctatcaataagcttatttatcgctggc
tttttatcaggagatttgtctaggtcctcttccaatgctgcattttaa
>KrC9_psbI
ttgctcaggtcttgtgtctattttgggattgctctatcaataagcttatttatcgctggc
tttttatcaggagatttgtctaggtcctcttctaatgctgcaatttaa
>KrD1_psbI
ttgctcaggtcttgtgtctattttgtgcttgctctttcatgcagcttatttatcgctggc
tttttatcaggagatttgtctaggtcctcttctaatgctgcaatttaa
>KrD11_psbI
ttgctcaggtcttgtgtctattttgtgcttgctctttcatgcagcttatttatcgctggc
tttttatcaggagatttgtctaggtcctcttctaatgctgcaatttaa
>KrD13_psbI
ttgctcaggtcttgtgtctattttgtgcttgctctttcatgcagcttatttatcgctggc
tttttatcaggagatttgtctaggtcctcttctaatgctgcattttaa
>KrD3_psbI
ttgctcaggtcttgtgtctattttgtgcttgctctttcatgcagcttatttattgctggc
tttttatcaggagatttgtctaggtcctcttccaatgctgcattttaa
>KrD8_psbI
ttgctcaggtcttgtgtctattttgggattgctctatcaataagcttatttatcgctggc
tttttatcaggagatttgtctaggtcctcttctaatgctgcattttaa
>KrD9_psbI
ttgctcaggtcttgtgtctattttgtgcttgctctttcatgcagcttatttatcgctggc
tttttatcaggagatttgtctaggtcctcttccaatgctgcattttaa
>KrE1_psbI
ttgctcaggtcttgtgtctattttgtgcttgctctttcatgcagcttatttatcgctggc
tttttatcaggagatttgtctaggtcctcttctaatgctgcaatttaa
>KrE10_psbI
ttgctcaggtcttgtgtctattttgtgcttgctctttcatgcagcttatttattgctggc
tttttatcaggagatttgtctaggtcctcttccaatgctgcattttaa

>KrE11_psbI
ttgctcaggtcttgtgtctattttgtgcttgctctttcatgcagcttatttattgctggc
tttttatcaggagatttgtctaggtcctcttccaatgctgcattttaa
>KrE12_psbI
atgcttaggtcttgtgtctattttgggattgctctatcaataagcttatttatcgctggc
tttttatcaggagatttgtctaggtcctcttctaatgctgcaatttaa
>KrE13_psbI
atgcttaggtcttgtgtctattttgggattgctctatcaataagcttatttatcgctggc
tttttatcaggagatttgtctaggtcctcttctaatgctgcaatttaa
>KrE14_psbI
ttgcttaggtcttgtgtctattttgggattgctctatcaataagcttatttatcgctggc
tttttatcaggagatttgtctaggtcctcttctaatgctgcaatttaa
>KrE15_psbI
ttgctcaggtcttgtgtctattttgggattgctctatcaataagcttatttatcgctggc
tttttatcaggagatttgtctaggtcctcttctaatgctgcaatttaa
>KrE2_psbI
ttgctcaggtcttgtgtctattttgggattgctctatcaataagcttatttatcgctggc
tttttatcaggagatttgtctaggtcctcttctaatgctgcattttaa
>KrE3_psbI
ttgctcaggtcttgtgtctattttgggattgctctatcaataagcttatttatcgctggc
tttttatcaggagatttgtctaggtcctcttctaatgctgcaatttaa
>KrE4_psbI
ttgcttaggtcttgtgtctattttgggattgctctatcaataagcttatttatcgctggc
tttttatcaggagatttgtctaggtcctcttctaatgctgcaatttaa
>KrE5_psbI
ttgcttaggtcttgtgtctattttgggattgctctatcaataagcttatttatcgctggc
tttttatcaggagatttgtctaggtcctcttctaatgctgcaatttaa
>KrE6_psbI
ttgctcaggtcttgtgtctattttgggattgctctatcaataagcttatttatcgctggc
tttttatcaggagatttgtctaggtcctcttctaatgctgcaatttaa
>KrE7_psbI
ttgctcaggtcttgtgtctattttgtgcttgctctttcatgcagcttatttattgctggc
tttttatcaggagatttgtctaggtcctcttccaatgctgcattttaa
>KrE8_psbI
ttgctcaggtcttgtgtctattttgtgcttgctctttcatgcagcttatttattgctggc
tttttatcaggagatttgtctaggtcctcttccaatgctgcattttaa
>KrE9_psbI
ttgctcaggtcttgtgtctattttgggattgctctatcaataagcttatttatcgctggc
tttttatcaggagatttgtctaggtcctcttctaatgctgcaatttaa
>Mz10_psbI
ttgctcaggtcttgtgtctattttgtgcttgctctttcatgcagcttatttattgctggc
tttttatcaggagatttgtctaggtcctcttccaatgctgcattttaa
>Mz5_psbI
ttgctcaggtcttgtgtctattttgtgcttgctctatcaataagcttatttatcgctggc
tttttatcaggagatttgtctaggtcctcttccaatgctgcattttaa
>Mz6_psbI
ttgctcaggtcttgtgtctattttgtgcttgctctttcatgcagcttatttattgctggc
tttttatcaggagatttgtctaggtcctcttccaatgctgcattttaa
>Mz7_psbI
ttgctcaggtcttgtgtctattttgtgcttgctctttcatgcagcttatttattgctggc
tttttatcaggagatttgtctaggtcctcttccaatgctgcattttaa
>Mz8_psbI
ttgctcaggtcttgtgtctattttgtgcttgctctttcatgcagcttatttattgctggc
tttttatcaggagatttgtctaggtcctcttccaatgctgcaatttaa
>Mz9_psbI
ttgctcaggtcttgtgtctattttgtgcttgctctttcatgcagcttatttattgctggc
tttttatcaggagatttgtctaggtcctcttccaatgctgcattttaa

>MzC1_psbI
ttgctcaggtcttgtgtctattttgggattgctctatcaataagcttatttatcgctggc
tttttatcaggagatttgtctaggtcctcttctaatgctgcattttaa
>MzC1GaII_psbI
ttgctcaggtcttgtgtctattttgggattgctctatcaataagcttatttatcgctggc
tttttatcaggagatttgtctaggtcctcttctaatgctgcaatttaa
>MzC2_psbI
ttgctcaggtcttgtgtctattttgtgcttgctctttcaagaagcttatttattgctggc
tttttatcaggagatttgtctaggtcctcttctaatgctgcaatttaa
>MzC2GaII_psbI
ttgctcaggtcttgtgtctattttgggattgctctatcaagaagcttatttatcgctggc
tttttatcaggagatttgtctaggtcctcttctaatgctgcaatttaa
>MzC3_psbI
ttgctcaggtcttgtgtctattttgtgcttgctctttcatgcagcttatttattgctggc
tttttatcaggagatttgtctaggtcctcttccaatgctgcattttaa
>MzC4_psbI
ttgctcaggtcttgtgtctattttgggattgctctatcaataagcttatttatcgctggc
tttttatcaggagatttgtctaggtcctcttctaatgctgcaatttaa
>Od10_psbI
ttgcttaggtcttgtgtctattttgtgattgctctatcaatcagcttatttatcgctggc
tttttatcaggagatttgtctaggtcctcttctaatgctgcaatttaa
>Od11_psbI
ttgctcaggtcttgtgtctattttgggattgctctatcaataagcttatttatcgctggc
tttttatcaggagatttgtctaggtcctcttctaatgctgcaatttaa
>Od12_psbI
ttgctcaggtcttgtgtctattttgtgcttgctctttcatgcagcttatttattgctggc
tttttatcaggagatttgtctaggtcctcttccaatgctgcattttaa
>Od13_psbI
ttgctcaggtcttgtgtctattttgggattgctctatcaataagcttatttatcgctggc
tttttatcaggagatttgtctaggtcctcttctaatgctgcaatttaa
>Od14_psbI
ttgctcaggtcttgtgtctattttgggattgctctatcaataagcttatttatcgctggc
tttttatcaggagatttgtctaggtcctcttctaatgctgcaatttaa
>Od15_psbI
ttgctcaggtcttgtgtctattttgggattgctctatcaataagcttatttatcgctggc
tttttatcaggagatttgtctaggtcctcttctaatgctgcaatttaa
>Od18_psbI
ttgctcaggtcttgtgtctattttgggattgctctatcaataagcttatttatcgctggc
tttttatcaggagatttgtctaggtcctcttctaatgctgcaatttaa
>Od19_psbI
ttgcttaggtcttgtgtctattttgggattgctctatcaataagcttatttatcgctggc
tttttatcaggagatttgtctaggtcctcttctaatgctgcaatttaa
>Od2_psbI
ttgctcaggtcttgtgtctattttgggattgctctatcaataagcttatttatcgctggc
tttttatcaggagatttgtctaggtcctcttctaatgctgcattttaa
>Od22_psbI
ttgctcaggtcttgtgtctattttgggattgctctatcaataagcttatttatcgctggc
tttttatcaggagatttgtctaggtcctcttctaatgctgcaatttaa
>Od23_psbI
ttgcttaggtcttgtgtctattttgggattgctctatcaataagcttatttatcgctggc
tttttatcaggagatttgtctaggtcctcttctaatgctgcaatttaa
>Od24_psbI
ttgctcaggtcttgtgtctattttgtgcttgctctttcatgcagcttatttattgctggc
tttttatcaggagatttgtctaggtcctcttccaatgctgcattttaa
>Od25_psbI
ttgctcaggtcttgtgtctattttgggattgctctatcaataagcttatttatcgctggc
tttttatcaggagatttgtctaggtcctcttctaatgctgcaatttaa

>Od26_psbI
ttgctcaggtcttgtgtctattttgtgcttgctctttcatgcagcttatttattgctggc
tttttatcaggagatttgtctaggtcctcttccaatgctgcattttaa
>Od27_psbI
ttgctcaggtcttgtgtctattttgtgcttgctctttcatgcagcttatttatcgctggc
tttttatcaggagatttgtctaggtcctcttctaatgctgcaatttaa
>Od4_psbI
ttgctcaggtcttgtgtctattttgtgcttgctctttcatgcagcttatttatcgctggc
tttttatcaggagatttgtctaggtcctcttctaatgctgcaatttaa
>Od6_psbI
ttgctcaggtcttgtgtctattttgggattgctctatcaataagcttatttatcgctggc
tttttatcaggagatttgtctaggtcctcttccaatgctgcaatttaa
>Od8_psbI
ttgctcaggtcttgtgtctattttgggattgctctatcaataagcttatttatcgctggc
tttttatcaggagatttgtctaggtcctcttctaatgctgcaatttaa
>Od9_psbI
ttgcttaggtcttgtgtctattttgggattgctctatcaataagcttatttatcgctggc
tttttatcaggagatttgtctaggtcctcttctaatgctgcattttaa
>Ss5_psbI
ttgctcaggtcttgtgtctattttgtgcttgctctttcatgcagcttatttatcgctggc
tttttatcaggagatttgtctaggtcctcttctaatgctgcaatttaa
>Ss6_psbI
ttgctcaggtcttgtgtctattttgtgcttgctctttcatgcagcttatttattgctggc
tttttatcaggagatttgtctaggtcctcttccaatgctgcattttaa
>Ss7_psbI
ttgctcaggtcttgtgtctattttgggattgctctatcaataagcttatttatcgctggc
tttttatcaggagatttgtctaggtcctcttctaatgctgcattttaa
>Ss8_psbI
ttgctcaggtcttgtgtctattttgtgcttgctctttcatgcagcttatttatcgctggc
tttttatcaggagatttgtctaggtcctcttctaatgctgcaatttaa
>ohdo1_psbI
ttgctcaggtcttgtgtctattttgtgcttgctctttcatgcagcttatttattgctggc
tttttatcaggagatttgtctaggtcctcttccaatgctgcattttaa
>ohdo3_psbI
ttgctcaggtcttgtgtctattttgtgcttgctctttcatgcagcttatttatcgctggc
tttttatcaggagatttgtctaggtcctcttccaatgctgcattttaa
>ohdo5_psbI
ttgctcaggtcttgtgtctattttgtgcttgctctttcatgcagcttatttatcgctggc
tttttatcaggagatttgtctaggtcctcttctaatgctgcaatttaa
>ohdo7_psbI
ttgcttaggtcttgtgtctattttgggattgctctatcaataagcttatttatcgctggc
tttttatcaggagatttgtctaggtcctcttctaatgctgcattttaa
>sesoko1_psbI
ttgctcaggtcttgtgtctattttgtgcttgctctttcatgcagcttatttatcgctggc
tttttatcaggagatttgtctaggtcctcttctaatgctgcaatttaa
>sesoko3_psbI
ttgctcaggtcttgtgtctattttgggattgctctatcaataagcttatttatcgctggc
tttttatcaggagatttgtctaggtcctcttctaatgctgcaatttaa
>sesoko4_psbI
ttgcttaggtcttgtgtctattttgggattgctctatcaataagcttatttatcgctggc
tttttatcaggagatttgtctaggtcctcttctaatgctgcattttaa
>REF_DNA_psbI_JX094330
TTGCTCAGGTCTTGTGTCTATTTTGTGCTTGCTCTTTCATGCAGCTTATTTATTGCTGGC
TTTTTATCAGGAGATTTGTCTAGGTCCTCTTCCAATGCTGCATTTTAA
>REF_RNA_psbI_symbB1.comp1832_c0_seq1
TTGCTCAGGTCTTGTGTCTATTTTGTGCTTGCTCTTTTATGCAGCTTATTTATTGCTGGC
TTTTTATCAGGAGATTTGTCTAGGTCCTCTTCCAATACTGCATCTTAA

>Hd1_petB
atgtggctttatgattggtctgaagaaggtttagaaattcaatgtattggagatgatatt
ctaggtaaattagtaccacctcatgttaatatattttactgctttggaggtgtggtatcc
cttttgctcttatttcaaataatcagtggtcttggattgacaatgtattacagtccaagt
gttgtctctgcattttcatcggtacttaatatagttggccaagtacatttaggttggtta
aatcgttctattcataggtggtctggatctagtatggtatctgcactaatattgcatgct
tttcgtgtttatttaacaggaggatttaaaaaagctcgtgagctaatttggatgacaggt
ataattttaggagtttgcaccgtgttgtttggagtaacaggatattgattagcttgggat
caagtagcttattgggcatgtaaaatagtgacaggagtacctgaaggactagacaagttg
ttatttggtgttggcttgcttttggttctaataattagaggtggctttagtgttagttct
ggtaggctcacaagattctatagcattcacacatttcttctcccaattgtaacattgagt
ttagttatcattcactttattcaaatcagaaaacaaggtatatctggacctctttaa
>Hd2_petB
atgtggctttatgattggtctgaagaaggtttagaaattcaatgtattggagatgatatt
ctaggtaaattagtaccacctcatgttaatatattttactgctttggaggtgtggtatcc
cttttgctcttatttcaaataatcagtggtcttggattgacaatgtattacagtccaagt
gttgtctctgcattttcatcggtacttaatatagttggccaagtacatttaggttggtta
aatcgttctattcacagatggtctggatctagtatggtatctgcactaatattgcatgct
tttcgtgtttatttaacaggaggatttaaaaaagctcgtgagctaatttggatgacaggt
ataattttaggagtttgcaccgtgttgtttggagtaacaggatattgattagcttgggat
caagtagcttattgggcatgtaaaatagtgacaggagtacctgaaggactagacaagttg
ttatttggtgttggcttgcttttggttctaataattagaggtggctttagtgttagttct
ggtaggctcacaagattctatagcattcacacatttcttctcccaattgtaacattgagt
ttagttatcattcactttattcaaatcagaaaacaaggtatatctggacctctttaa
>Hd3_petB
atgtggctttatgattggtctgaagaaggtttagaaattcaatgtattggagatgatatt
ctaggtaaattagtaccacctcatgttaatatattttactgctttggaggtgtggtatcc
cttttgctcttatttcaaataatcagtggtcttggattgacaatgtattacactccaagt
gttgtctctgcattttcatcggtacttaatatagttggccaagtacatttaggttggtta
aatcgttctattcacagatggtctggatctagtatggtatctgcactaatattgcatgct
tttcgtgtttatttaacaggaggatttaaaaaagctcgtgagctaatttggatgacaggt
ataattttaggagtttgcaccgtgttgtttggagtaacaggatattgattagcttgggat
caagtaggttattgggcatgtaaaatagtgacaggagtacctgaaggactagacaagttg
ttattcggtgttggctttcttttggttctaataattagaggtggctttagtgttagttct
ggtaggctcacaagattctatagcattcacacatttcttctcccaattgtaacattgagt
ttagttatcattcactttattcaaatcagaaaacaaggtatatctggacctctttaa
>Hd4_petB
atgtggctttatgattggtctgaagaaggtttagaaattcaatgtattggagatgatatt
ctaggtaaattagtaccacctcatgttaatatattttactgctttggaggtgtggtatcc
cttttgctcttatttcaaataatcagtggtcttggattgacaatgtattacagtccaagt
gttgtctctgcattttcatcggtacttaatatagttggccaagtacatttaggttggtta
aatcgttctattcacagatggtctggatctagtatggtatctgcactaatattgcatgct
tttcgtgtttatttaacaggaggatttaaaaaagctcgtgagctaatttggatgacaggt
ataattttaggagtttgcaccgtgttgtttggagtaacaggatattgattagcttgggat
caagtagcttattgggcatgtaaaatagtgacaggagtacctgaaggactagacaagttg
ttatttggtgttggcttgcttttggttctaataattagaggtggctttagtgttagttct
ggtaggctcacaagattctatagcattcacacattccttctcccaattgtaacattaagt
ttagttatcattcactttattcaaatcagaaaacaaggtatatctggacctctttaa
>Hd5_petB
atgtggctttatgattggtctgaagaaggtttagaaattcaatgtattggagatgatatt
ctaggtaaattagtaccacctcatgttaatatattttactgctttggaggtgtggtatcc
cttttgctcttatttcaaataatcagtggtcttggattgacaatgtattacagtccaagt
gttgtctctgcattttcatcggtacttaatatagttggccaagtacatttaggttggtta
aatcgttctattcacagatggtctggatctagtatggtatctgcactaatattgcatgct
tttcgtgtttatttaacaggaggatttaaaaaagctcgtgagctaatttggatgacaggt
ataattttaggagtttgcaccgtgttgtttggagtaacaggatattgattagcttgggat
caagtagcttattgggcatgtaaaatagtgacaggagtacctgaaggactagacaagttg

ttatttggtgttggcttgcttttggttctaataattagaggtggctttagtgttagttct
ggtaggctcacaagattctatagcattcacacatttcttctcccaattgtaacattgagt
ttagttatcattcactttattcaaatcagaaaacaaggtatatctggacctctttaa
>Hd6_petB
atgtggctttatgattggtctgaagaaggtttagaaattcaatgtattggagatgatatt
ctaggtaaattagtaccacctcatgttaatatattttactgctttggaggtgtggtatcc
cttttgctcttatttcaaataatcagtggtcttggattgacaatgtattacactccaagt
gttgtctctgcattttcatcggtacttaatatagttggccaagtatatttaggttggtta
aatcgttctattcatagatggtctggatctagtatggtatctgcactaatattgcatgct
tttcgtgtttatttaacaggaggatttaaaaaagctcgtgagctaatttggatgacaggt
ataattttaggagtttgcaccgtgttgtttggagtaacaggatattgattagcttgggat
caagtaggttattgggcatgtaaaatagtgacaggagtacctgaaggactagacaagttg
ttatttggtgttggcttgcttttggttctaataattagaggtggctttagtgttagttct
ggtaggctcacaagattctatagcattcacacattccttctcccaattgtaacattgagt
ttagttatcattcactttattcaaatcagaaaacaaggtatatctggacctctttaa
>Hd7_petB
atgtggctttatgattggtctgaagaaggtttagaaattcaatgtattggagatgatatt
ctaggtaaattagtaccacctcatgttaatatattttactgctttggaggtgtggtatcc
cttttgctcttatttcaaataatcagtggtcttggattgacaatgtattacactccaagt
gttgtctctgcattttcatcggtacttaatatagttggccaagtacatttaggttggtta
aatcgttctattcataggtggtctggatctagtatggtatctgcactaatattgcatgct
tttcgtgtttatttaacaggaggatttaaaaaagctcgtgagctaatttggatgacaggt
ataattttaggagtttgcaccgtgttgtttggagtaacaggatattgattagcttgggat
caagtaggttattgggcatgtaaaatagtgacaggagtacctgaaggactagacaagttg
ttatttggtgttggctttcttttagttctaataattagaggtggttttagtgttagttct
ggtaggctcacaagattctatagcattcacacattccttctcccaattgtaacattaagt
ttagttatcattcactttattcaaatcagaaaacaaggtatatctggacctctttaa
>Hd8_petB
atgtggctttatgattggtctgaagaaggtttagaaattcaatgtattggagatgatatt
ctaggtaaattagtaccacctcatgttaatatattttactgctttggaggtgtggtatcc
cttttgctcttatttcaaataatcagtggtcttggattgacaatgtattacagtccaagt
gttgtctctgcattttcatcggtacttaatatagttggccaagtacatttaggttggtta
aatcgttctattcacagatggtctggatctagtatggtatctgcactaatattgcatgct
tttcgtgtttatttaacaggaggatttaaaaaagctcgtgagctaatttggatgacaggt
ataattttaggagtttgcaccgtgttgtttggagtaacaggatattgattagcttgggat
caagtagcttattgggcatgtaaaatagtgacaggagtacctgaaggactagacaagttg
ttatttggtgttggctttcttttggttctaataattagaggtggctttagtgttagttct
ggtaggctcacaagattctatagcattcacacatttcttctccctattgtaacattaagt
ttagttatcattcactttattcaaatcagaaaacaaggtatatctggacctctttaa
>Hd9_petB
atgtggctttatgattggtctgaagaaggtttagaaattcaatgtattggagatgatatt
ctaggtaaattagtaccacctcatgttaatatattttactgctttggaggtgtggtatcc
cttttgctcttatttcaaataatcagtggtcttggattgacaatgtattacactccaagt
gttgtctctgcattttcatcggtacttaatatagttggccaagtatatttaggttggtta
aatcgttctattcataggtggtctggatctagtatggtatctgcactaatattgcatgct
tttcgtgtttatttaacaggaggatttaaaaaagctcgtgagctaatttggatgacaggt
ataattctaggagtttgcaccgtgttgtttggagtaacaggatattgattagcttgggat
caagtaggttattgggcatgtaaaatagtgacaggagtacctgaaggactagacaagttg
ttattcggtgttggctttcttttagttctaataattagaggtggctttagtgttagttct
ggtaggctcacaagattctatagcattcacacattccttctcccaattgtaacattaagt
ttagttatcattcactttattcaaatcagaaaacaaggtatatctggacctctttaa
>IS1_petB
atgtggctttatgattggtctgaagaaggtttagaaattcaatgtattggagatgatatt
ctaggaaaattagtaccaccccatgttaatatattttactgctttggaggtgtggtatcc
cttttgctcttatttcaaataatcagtggtcttggattgacaatgtattacactccaagt
gttgtctctgcattttcatcggtacttaatatagttggccaagtatatttaggttggtta
aatcgttctattcataggtggtctggatctagtatggtatctgcactaatattgcatgct

ttccgtgtttatttaacaggaggatttaaaaaagctcgtgagctaatctggatgacaggt
ataattctaggagtttgcaccgtgttgtttggagtaacaggatattgattagcttgggat
caattaggttattgggcatgtaaaatagtgacaggagtacctgaaggactagacaagttg
ttattcggtgttggctttcttttagttctaataattagaggtggctttagtgttagttct
ggtaggctcacaagattctatagcattcacacattccttctcccaattgtaacattgact
ttagttatcattcactttattcaaatcagaaaacaaggtatatctggacctctttaa
>IS2_petB
atgtggctttatgattggtctgaagaaggtttagaaattcaatgtattggagatgatatt
ctaggtaaattagtaccacctcatgttaatatattttactgctttggaggtgtggtatcc
cttttgctcttatttcaaataatcagtggtcttggattgacaatgtattacagtccaagt
gttgtctctgcattttcatcggtacttaatatagttggccaagtacatttaggttggtta
aatcgttctattcacagatggtctggatctagtatggtatctgcactaatattgcatgct
tttcgtgtttatttaacaggaggatttaaaaaagctcgtgagctaatttggatgacaggt
ataattttaggagtttgcaccgtgttgtttggagtaacaggatattgattagcttgggat
caattagcttattgggcatgtaaaatagtgacaggagtacctgaaggactagataagttt
ttatttggtgttggcttgcttttggttctaataattagaggtggctttagtgttagttct
ggtaggctcacaagattctatagcattcacacatttcttctcccaattgtaacattgagt
ttagttatcattcattttattcaaatcagaaaacaaggtatatctggacctctataa
>IS3_petB
atgtggctttatgattggtctgaagaaggtttagaaattcaatgtattggagatgatatt
ctaggaaaattagtaccaccccatgttaatatattttactgctttggaggtgtggtatcc
cttttgctcttatttcaaataatcagtggtcttggattgacaatgtattacactccaagt
gttgtctctgcattttcatcggtacttaatatagttggacaagtatatttaggttggtta
aatcgttctattcacaggtggtctggggctagtatggtatctgcactaatattgcatgct
tttcgtgtttatttaacaggaggatttaaaaaagctcgtgagctaatttggatgacaggt
ataattctaggagtttgcaccgtgttgtttggagtaacaggatattgattagcttgggat
caagtaggttattgggcatgtaaaatagtgacaggagtacctgaaggactagacaagttg
ttattcggtgttggctttcttttagttctaataattagaggtggctttagtgttagttct
ggtaggctcacaagattctatagcattcacacattccttctcccaattgtaacattaagt
ttagttatcattcactttattcaaatcagaaaacaaggtatatccggacctctttaa
>IS4_petB
atgtggctttatgattggtctgaagaaggtttagaaattcaatgtattggagatgatatt
ctaggaaaattagtaccaccccatgttaatatattttactgctttggaggtgtggtatcc
cttttgctcttatttcaaataatcagtggtcttggattgacaatgtattacagtccaagt
gttgtttctgcattttcatcggtacttaatatagttggacaagtatatttaggttggtta
aatcgttctattcacaggtggtctggggctagtatggtatctgcactaatattgcatgct
ttccgtgtttatttaacaggaggatttaaaaaagctcgtgagctaatctggatgacaggt
ataattttaggagtttgtaccgtgttgtttggagtaacaggatattgattagcttgggat
caattaggttattgggcatgtaaaatagtgacaggagtacctgaaggactagataagttt
ttatttggtgttggctcgctcttggttttaataattagaggtggctttagtgttagttct
ggtaggctcacaagattctatagcattcacacattccttctcccaattgtaacattgact
ttagttatcattcactttattcaaatcagaaaacaaggtatatccggacctctttaa
>IS5_petB
atgtggctttatgattggtctgaagaaggtttagaaattcaatgtattggagatgatatt
ctaggtaaattagtaccacctcatgttaatatattttactgctttggaggtgtggtatcc
cttttgctcttatttcaaataatcagtggtcttggattgacaatgtattacagtccaagt
gttgtctctgcattttcatcggtacttaatatagttggccaagtatatttaggttggtta
aatcgttctattcacaggtggtctggatctagtatggtatctgcactaatattgcatgct
tttcgtgtttatttaacaggaggatttaaaaaagctcgtgagctaatttggatgacaggt
ataattctaggagtttgcaccgtgttgtttggagtaacaggatattgattagcttgggat
caagtaggttattgggcatgtaaaatagtgacaggagtacctgaaggactagacaagttg
ttatttggtgttggctttcttttggttctaataattagaggtggctttagtgttagttct
ggtaggctcacaagattctatagcattcacacatttcttctcccaattgtaacattgagt
ttagttatcattcattttattcaaatcagaaaacaaggtatatctggacctctttaa
>IS6_petB
atgtggctttatgattggtctgaagaaggtttagaaattcaatgtattggagatgatatt
ctaggtaaattagtaccacctcatgttaatatattttactgctttggaggtgtggtatcc

cttttgctcttatttcaaataatcagtggtcttggattgacaatgtattacagtccaagt
gttgtctctgtattttcatcggtacttaatatagttagccaagtatatttaggttggtta
aatcgttctattcataggtggtctggatctagtatggtatctgcactaatattgcatgct
tttcgtgtttatttaacaggaggatttaaaaaagctcgtgagctaatttggatgacaggt
ataattctaggagtttgcaccgtgttgtttggagtaacaggatattgattagcttgggat
caagtaggttattgggcatgtaaaatagtgacaggagtacctgaaggactagacaagttg
ttatttggtgttggcttgcttttggttctaataattagaggtggctttagtgttagttct
ggtaggctcacaagattctatagcattcacacattccttctcccaattgtaacattgagt
ttagttatcattcactttattcaaatcagaaaacaaggtatatctggacctctttaa
>IS7_petB
atgtggctttatgattggtctgaagaaggtttagaaattcaatgtattggagatgatatt
ctaggaaaattagtaccaccccatgttaatatattttactgctttggaggtgtggtatcc
cttttgctcttatttcaaataatcagtggtcttggattgacaatgtattacagtccaagt
gttgtctctgcattttcatcggtacttaatatagttggccaagtacatttaggttggtta
aatcgttctattcacaggtggtctggagctagtatggtatctgcactaatattgcatgct
ttccgtgtttatttaacaggaggatttaaaaaagctcgtgagctaatctggatgacaggt
ataattttaggagtttgcaccgtgttgtttggagtaacaggatattgattagcttgggat
caattaggttattgggcatgtaaaatagtgacaggagtacctgaaggactagataagttt
ttatttggtgttggctttcttttagttctaataattagaggtggctttagtgttagttct
ggtaggctcacaagattctatagcattcacacattccttctcccaattgtaacattaagt
ttagttatcattcactttattcaaatcagaaaacaaggtatatctggacctctttaa
>IS8_petB
atgtggctttatgattggtctgaagaaggtttagaaattcaatgtattggagatgatatt
ctaggtaaattagtaccacctcatgttaatatattttactgctttggaggtgtggtatcc
cttttgctcttatttcaaataatcagtggtcttggattgacaatgtattacagtccaagt
gttgtctctgcattttcatcggtacttaatatagttggccaagtacatttaggttggtta
aatcgttctattcacagatggtctggatctagtatggtatctgcactaatattgcatgct
tttcgtgtttatttaacaggaggatttaaaaaagctcgtgagctaatttggatgacaggt
ataattttaggagtttgcaccgtgttgtttggagtaacaggatattgattagcttgggat
caagtagcttattgggcatgtaaaatagtgacaggagtacctgaaggactagacaagttg
ttatttggtgttggcttgcttttggttctaataattagaggtggctttagtgttagttct
ggtaggctcacaagattctatagcattcacacatttcttctcccaattgtaacattgagt
ttagttatcattcactttattcaaatcagaaaacaaggtatatctggacctctttaa
>IS9_petB
atgtggctttatgattggtctgaagaaggtttagaaattcaatgtattggagatgatatt
ctaggaaaattagtaccaccccatgttaatatattttactgctttggaggtgtggtatcc
cttttgctcttatttcaaataatcagtggtcttggattgacaatgtattacagtccaagt
gttgtttctgcattttcatcggtacttaatatagttggccaagtatatttaggttggtta
aatcgttctattcataggtggtctggggctagtatggtatctgcactaatattgcatgct
tttcgtgtttatttaacaggaggatttaaaaaagctcgtgagctaatttggatgacaggt
ataattctaggagtttgcaccgtgttgtttggagtaacaggatattgattagcttgggat
caagtaggttattgggcatgtaaaatagtgacaggagtacctgaaggactagataagttt
ttatttggtgttggcttgcttttggttctaataattagaggtggctttagtgttagttct
ggtaggctcacaagattctatagcattcacacattccttctcccaattgtaacattaagt
ttagttatcattcactttattcaaatcagaaaacaaggtatatctggacctctttaa
>Ik1_petB
atgtggctttatgattggtctgaagaaggtttagaaattcaatgtattggagatgatatt
ctaggtaaattagtaccacctcatgttaatatattttactgctttggaggtgtggtatcc
cttttgctcttatttcaaataatcagtggtcttggattgacaatgtattacagtccaagt
gttgtctctgcattttcatcggtacttaatatagttggccaagtacatttaggttggtta
aatcgttctattcataggtggtctggatctagtatggtatctgcactaatattgcatgct
tttcgtgtttatttaacaggaggatttaaaaaagctcgtgagctaatttggatgacaggt
ataattctaggagtttgcaccgtgttgtttggagtaacaggatattgattagcttgggat
caagtaggttattgggcatgtaaaatagtgacaggagtacctgaaggactagacaagttg
ttattcggtgttggctttcttttagttctaataattagaggtggctttagtgttagttct
ggtaggctcacaagattctatagcattcacacattccttctcccaattgtaacattaagt
ttagttatcattcactttattcaaatcagaaaacaaggtatatctggacctctttaa

>Ik2_petB
atgtggctttatgattggtctgaagaaggtttagaaattcaatgtattggagatgatatt
ctaggtaaattagtaccacctcatgttaatatattttactgctttggaggtgtggtatcc
cttttgctcttatttcaaataatcagtggtcttggattgacaatgtattacactccaagt
gttgtctctgcattttcatcggtacttaatatagttggccaagtatatttaggttggtta
aatcgttctattcatagatggtctggatctagtatggtatctgcactaatattgcatgct
tttcgtgtttatttaacaggaggatttaaaaaagctcgtgagctaatttggatgacaggt
ataattctaggagtttgcaccgtgttgtttggagtaacaggatattgattagcttgggat
caagtaggttattgggcatgtaaaatagtgacaggagtacctgaaggactagacaagttg
ttatttggtgttggcttgcttttggttctaataattagaggtggctttagtgttagttct
ggtaggctcacaagattctatagcattcacacatttcttctcccaattgtaacattgagt
ttagttatcattcactttattcaaatcagaaaacaaggtatatctggacctctttaa
>Ik3_petB
atgtggctttatgattggtctgaagaaggtttagaaattcaatgtattggagatgatatt
ctaggtaaattagtaccacctcatgttaatatattttactgctttggaggtgtggtatcc
cttttgctcttatttcaaataatcagtggtcttggattgacaatgtattacagtccaagt
gttgtctctgcattttcatcggtacttaatatagttggccaagtacatttaggttggtta
aatcgttctattcacagatggtctggatctagtatggtatctgcactaatattgcatgct
tttcgtgtttatttaacaggaggatttaaaaaagctcgtgagctaatttggatgacaggt
ataattctaggagtttgcaccgtgttgtttggagtaacaggatattgattagcttgggat
caagtagcttattgggcatgtaaaatagtgacaggagtacctgaaggactagacaagttg
ttatttggtgttggcttgcttttggttctaataattagaggtggctttagtgttagttct
ggtaggctcacaagattctatagcattcacacattccttctcccaattgtaacattaagt
ttagttatcattcactttattcaaatcagaaaacaaggtatatctggacctctttaa
>Ik4_petB
atgtggctttatgattggtctgaagaaggtttagaaattcaatgtattggagatgatatt
ctaggtaaattagtaccacctcatgttaatatattttactgctttggaggtgtggtatcc
cttttgctcttatttcaaataatcagtggtcttggattgacaatgtattacactccaagt
gttgtctctgcattttcatcggtacttaatatagttggccaagtatatttaggttggtta
aatcgttctattcataggtggtctggatctagtatggtatctgcactaatattgcatgct
tttcgtgtttatttaacaggaggatttaaaaaagctcgtgagctaatttggatgacaggt
ataattttaggagtttgcaccgtgttgtttggagtaacaggatattgattagcttgggat
caagtagcttattgggcatgtaaaatagtgacaggagtacctgaaggactagacaagttg
ttattcggtgttggctttcttttagttctaataattagaggtggctttagtgttagttct
ggtaggctcacaagattctatagcattcacacattccttctcccaattgtaacattaagt
ttagttatcattcactttattcaaatcagaaaacaaggtatatctggacctctttaa
>Ik5_petB
atgtggctttatgattggtctgaagaaggtttagaaattcaatgtattggagatgatatt
ctaggtaaattagtaccacctcatgttaatatattttactgctttggaggtgtggtatcc
cttttgctcttatttcaaataatcagtggtcttggattgacaatgtattacagtccaagt
gttgtctctgcattttcatcggtacttaatatagttggccaagtatatttaggttggtta
aatcgttctattcataggtggtctggatctagtatggtatctgcactaatattgcatgct
tttcgtgtttatttaacaggaggatttaaaaaagctcgtgagctaatttggatgacaggt
ataattttaggagtttgcaccgtgttgtttggagtaacaggatattgattagcttgggat
caagtagcttattgggcatgtaaaatagtgacaggagtacctgaaggactagacaagttg
ttatttggtgttggcttgcttttggttctaataattagaggtggctttagtgttagttct
ggtaggctcacaagattctatagcattcacacatttcttctcccaattgtaacattgagt
ttagttatcattcactttattcaaatcagaaaacaaggtatatctggacctctttaa
>Ik6_petB
atgtggctttatgattggtctgaagaaggtttagaaattcaatgtattggagatgatatt
ctaggtaaattagtaccacctcatgttaatatattttactgctttggaggtgtggtatcc
cttttgctcttatttcaaataatcagtggtcttggattgacaatgtattacagtccaagt
gttgtctctgcattttcatcggtacttaatatagttggccaagtacatttaggttggtta
aatcgttctattcataggtggtctggatctagtatggtatctgcactaatattgcatgct
tttcgtgtttatttaacaggaggatttaaaaaagctcgtgagctaatttggatgacaggt
ataattttaggagtttgcaccgtgttgtttggagtaacaggatattgattagcttgggat
caagtaggttattgggcatgtaaaatagtgacaggagtacctgaaggactagacaagttg

ttattcggtgttggctttcttttagttctaataattagaggtggctttagtgttagttct
ggtaggctcacaagattctatagcattcacacattccttctcccaattgtaacattgagt
ttagttatcattcactttattcaaatcagaaaacaaggtatatctggacctctttaa
>Ik7_petB
atgtggctttatgattggtctgaagaaggtttagaaattcaatgtattggagatgatatt
ctaggtaaattagtaccacctcatgttaatatattttactgctttggaggtgtggtatcc
cttttgctcttatttcaaataatcagtggtcttggattgacaatgtattacagtccaagt
gttgtctctgcattttcatcggtacttaatatagttggccaagtatatttaggttggtta
aatcgttctattcataggtggtctggatctagtatggtatctgcactaatattgcatgct
tttcgtgtttatttaacaggaggatttaaaaaagctcgtgagctaatttggatgacaggt
ataattctaggagtttgcaccgtgttgtttggagtaacaggatattgattagcttgggat
caagtagcttattgggcatgtaaaatagtgacaggagtacctgaaggactagacaagttg
ttatttggtgttggcttgcttttggttctaataattagaggtggctttagtgttagttct
ggtaggctcacaagattctatagcattcacacattccttctcccaattgtaacattgagt
ttagttatcattcactttattcaaatcagaaaacaaggtatatctggacctctttaa
>Ik8_petB
atgtggctttatgattggtctgaagaaggtttagaaattcaatgtattggagatgatatt
ctaggtaaattagtaccacctcatgttaatatattttactgctttggaggtgtggtatcc
cttttgctcttatttcaaataatcagtggtcttggattgacaatgtattacagtccaagt
gttgtctctgcattttcatcggtacttaatatagttggccaagtacatttaggttggtta
aatcgttctattcacagatggtctggatctagtatggtatctgcactaatattgcatgct
tttcgtgtttatttaacaggaggatttaaaaaagctcgtgagctaatttggatgacaggt
ataattttaggagtttgcaccgtgttgtttggagtaacaggatattgattagcttgggat
caagtaggttattgggcatgtaaaatagtgacaggagtacctgaaggactagacaagttg
ttattcggtgttggctttcttttagttctaataattagaggtggctttagtgttagttct
ggtaggctcacaagattctatagcattcacacatttcttctcccaattgtaacattgagt
ttagttatcattcactttattcaaatcagaaaacaaggtatatctggacctctttaa
>Ik9_petB
atgtggctttatgattggtctgaagaaggtttagaaattcaatgtattggagatgatatt
ctaggtaaattagtaccacctcatgttaatatattttactgctttggaggtgtggtatcc
cttttgctcttatttcaaataatcagtggtcttggattgacaatgtattacactccaagt
gttgtctctgcattttcatcggtacttaatatagttggccaagtacatttaggttggtta
aatcgttctattcacagatggtctggatctagtatggtatctgcactaatattgcatgct
tttcgtgtttatttaacaggaggatttaaaaaagctcgtgagctaatttggatgacaggt
ataattttaggagtttgcaccgtgttgtttggagtaacaggatattgattagcttgggat
caagtagcttattgggcatgtaaaatagtgacaggagtacctgaaggactagacaagttg
ttatttggtgttggctttcttttagttctaataattagaggtggctttagtgttagttct
ggtaggctcacaagattctatagcattcacacatttcttctcccaattgtaacattaagt
ttagttatcattcactttattcaaatcagaaaacaaggtatatctggacctctttaa
>Irm10_petB
atgtggctttatgattggtctgaagaaggtttagaaattcaatgtattggagatgatatt
ctaggtaaattagtaccacctcatgttaatatattttactgctttggaggtgtggtatcc
cttttgctcttatttcaaataatcagtggtcttggattgacaatgtattacactccaagt
gttgtctctgcattttcatcggtacttaatatagttggccaagtacatttaggttggtta
aatcgttctattcacagatggtctggatctagtatggtatctgcactaatattgcatgct
tttcgtgtttatttaacaggaggatttaaaaaagctcgtgagctaatttggatgacaggt
ataattctaggagtttgcaccgtgttgtttggagtaacaggatattgattagcttgggat
caagtaggttattgggcatgtaaaatagtgacaggagtacctgaaggactagacaagttg
ttatttggtgttggcttgcttttggttctaataattagaggtggctttagtgttagttct
ggtaggctcacaagattctatagcattcacacatttcttctcccaattgtaacattgagt
ttagttatcattcactttattcaaatcagaaaacaaggtatatctggacctctttaa
>Irm17_petB
atgtggctttatgattggtctgaagaaggtttagaaattcaatgtattggagatgatatt
ctaggtaaattagtaccacctcatgttaatatattttactgctttggaggtgtggtatcc
cttttgctcttatttcaaataatcagtggtcttggattgacaatgtattacagtccaagt
gttgtctctgcattttcatcggtacttaatatagttggccaagtacatttaggttggtta
aatcgttctattcacagatggtctggatctagtatggtatctgcactaatattgcatgct

tttcgtgtttatttaacaggaggatttaaaaaagctcgtgagctaatttggatgacaggt
ataattttaggagtttgcaccgtgttgtttggagtaacaggatattgattagcttgggat
caagtagcttattgggcatgtaaaatagtgacaggagtacctgaaggactagacaagttg
ttatttggtgttggcttgcttttggttctaataattagaggtggctttagtgttagttct
ggtaggctcacaagattctatagcattcacacatttcttctcccaattgtaacattgagt
ttagttatcattcactttattcaaatcagaaaacaaggtatatctggacctctttaa
>Irm2_petB
atgtggctttatgattggtctgaagaaggtttagaaattcaatgtattggagatgatatt
ctaggtaaattagtaccacctcatgttaatatattttactgctttggaggtgtggtatcc
cttttgctcttatttcaaataatcagtggtcttggattgacaatgtattacagtccaagt
gttgtctctgcattttcatcggtacttaatatagttggccaagtacatttaggttggtta
aatcgttctattcacagatggtctggatctagtatggtatctgcactaatattgcatgct
tttcgtgtttatttaacaggaggatttaaaaaagctcgtgagctaatttggatgacaggt
ataattttaggagtttgcaccgtgttgtttggagtaacaggatattgattagcttgggat
caagtagcttattgggcatgtaaaatagtgacaggagtacctgaaggactagacaagttg
ttatttggtgttggcttgcttttggttctaataattagaggtggctttagtgttagttct
ggtaggctcacaagattctatagcattcacacatttcttctcccaattgtaacattgagt
ttagttatcattcactttattcaaatcagaaaacaaggtatatctggacctctttaa
>Irm21_petB
atgtggctttatgattggtctgaagaaggtttagaaattcaatgtattggagatgatatt
ctaggtaaattagtaccacctcatgttaatatattttactgctttggaggtgtggtatcc
cttttgctcttatttcaaataatcagtggtcttggattgacaatgtattacagtccaagt
gttgtctctgcattttcatcggtacttaatatagttggccaagtatatttaggttggtta
aatcgttctattcataggtggtctggatctagtatggtatctgcactaatattgcatgct
tttcgtgtttatttaacaggaggatttaaaaaagctcgtgagctaatttggatgacaggt
ataattttaggagtttgcaccgtgttgtttggagtaacaggatattgattagcttgggat
caagtagcttattgggcatgtaaaatagtgacaggagtacctgaaggactagacaagttg
ttatttggtgttggcttgcttttggttctaataattagaggtggctttagtgttagttct
ggtaggctcacaagattctatagcattcacacattccttctcccaattgtaacattaagt
ttagttatcattcactttattcaaatcagaaaacaaggtatatctggacctctttaa
>Irm22_petB
atgtggctttatgattggtctgaagaaggtttagaaattcaatgtattggagatgatatt
ctaggtaaattagtaccacctcatgttaatatattttactgctttggaggtgtggtatcc
cttttgctcttatttcaaataatcagtggtcttggattgacaatgtattacactccaagt
gttgtctctgcattttcatcggtacttaatatagttggccaagtatatttaggttggtta
aatcgttctattcataggtggtctggatctagtatggtatctgcactaatattgcatgct
tttcgtgtttatttaacaggaggatttaaaaaagctcgtgagctaatttggatgacaggt
ataattctaggagtttgcaccgtgttgtttggagtaacaggatattgattagcttgggat
caagtaggttattgggcatgtaaaatagtgacaggagtacctgaaggactagacaagttg
ttattcggtgttggctttcttttagttctaataattagaggtggctttagtgttagttct
ggtaggctcacaagattctatagcattcacacattccttctcccaattgtaacattaagt
ttagttatcattcactttattcaaatcagaaaacaaggtatatctggacctctttaa
>Irm23_petB
atgtggctttatgattggtctgaagaaggtttagaaattcaatgtattggagatgatatt
ctaggtaaattagtaccacctcatgttaatatattttactgctttggaggtgtggtatcc
cttttgctcttatttcaaataatcagtggtcttggattgacaatgtattacactccaagt
gttgtctctgcattttcatcggtacttaatatagttggccaagtatatttaggttggtta
aatcgttctattcataggtggtctggatctagtatggtatctgcactaatattgcatgct
tttcgtgtttatttaacaggaggatttaaaaaagctcgtgagctaatttggatgacaggt
ataattttaggagtttgcaccgtgttgtttggagtaacaggatattgattagcttgggat
caagtagcttattgggcatgtaaaatagtgacaggagtacctgaaggactagacaagttg
ttattcggtgttggctttcttttagttctaataattagaggtggctttagtgttagttct
ggtaggctcacaagattctatagcattcacacattccttctcccaattgtaacattaagt
ttagttatcattcactttattcaaatcagaaaacaaggtatatctggacctctttaa
>Irm24_petB
atgtggctttatgattggtctgaagaaggtttagaaattcaatgtattggagatgatatt
ctaggtaaattagtaccacctcatgttaatatattttactgctttggaggtgtggtatcc

cttttgctcttatttcaaataatcagtggtcttggattgacaatgtattacactccaagt
gttgtctctgcattttcatcggtacttaatatagttggccaagtatatttaggttggtta
aatcgttctattcataggtggtctggatctagtatggtatctgcactaatattgcatgct
tttcgtgtttatttaacaggaggatttaaaaaagctcgtgagctaatttggatgacaggt
ataattctaggagtttgcaccgtgttgtttggagtaacaggatattgattagcttgggat
caagtaggttattgggcatgtaaaatagtgacaggagtacctgaaggactagacaagttg
ttattcggtgttggctttcttttagttctaataattagaggtggctttagtgttagttct
ggtaggctcacaagattctatagcattcacacattccttctcccaattgtaacattaagt
ttagttatcattcactttattcaaatcagaaaacaaggtatatctggacctctttaa
>Irm25_petB
atgtggctttatgattggtctgaagaaggtttagaaattcaatgtattggagatgatatt
ctaggtaaattagtaccacctcatgttaatatattttactgctttggaggtgtggtatcc
cttttgctcttatttcaaataatcagtggtcttggattgacaatgtattacactccaagt
gttgtctctgcattttcatcggtacttaatatagttggccaagtatatttaggttggtta
aatcgttctattcataggtggtctggatctagtatggtatctgcactaatattgcatgct
tttcgtgtttatttaacaggaggatttaaaaaagctcgtgagctaatttggatgacaggt
ataattctaggagtttgcaccgtgttgtttggagtaacaggatattgattagcttgggat
caagtaggttattgggcatgtaaaatagtgacaggagtacctgaaggactagacaagttg
ttattcggtgttggctttcttttagttctaataattagaggtggctttagtgttagttct
ggtaggctcacaagattctatagcattcacacattccttctcccaattgtaacattaagt
ttagttatcattcactttattcaaatcagaaaacaaggtatatctggacctctttaa
>Irm26_petB
atgtggctttatgattggtctgaagaaggtttagaaattcaatgtattggagatgatatt
ctaggtaaattagtaccacctcatgttaatatattttactgctttggaggtgtggtatcc
cttttgctcttatttcaaataatcagtggtcttggattgacaatgtattacactccaagt
gttgtctctgcattttcatcggtacttaatatagttggccaagtatatttaggttggtta
aatcgttctattcataggtggtctggatctagtatggtatctgcactaatattgcatgct
tttcgtgtttatttaacaggaggatttaaaaaagctcgtgagctaatttggatgacaggt
ataattctaggagtttgcaccgtgttgtttggagtaacaggatattgattagcttgggat
caagtaggttattgggcatgtaaaatagtgacaggagtacctgaaggactagacaagttg
ttattcggtgttggctttcttttagttctaataattagaggtggctttagtgttagttct
ggtaggctcacaagattctatagcattcacacattccttctcccaattgtaacattaagt
ttagttatcattcactttattcaaatcagaaaacaaggtatatctggacctctttaa
>Irm27_petB
atgtggctttatgattggtctgaagaaggtttagaaattcaatgtattggagatgatatt
ctaggtaaattagtaccacctcatgttaatatattttactgctttggaggtgtggtatcc
cttttgctcttatttcaaataatcagtggtcttggattgacaatgtattacactccaagt
gttgtctctgcattttcatcggtacttaatatagttggccaagtatatttaggttggtta
aatcgttctattcataggtggtctggatctagtatggtatctgcactaatattgcatgct
tttcgtgtttatttaacaggaggatttaaaaaagctcgtgagctaatttggatgacaggt
ataattctaggagtttgcaccgtgttgtttggagtaacaggatattgattagcttgggat
caagtaggttattgggcatgtaaaatagtgacaggagtacctgaaggactagacaagttg
ttattcggtgttggctttcttttagttctaataattagaggtggctttagtgttagttct
ggtaggctcacaagattctatagcattcacacattccttctcccaattgtaacattaagt
ttagttatcattcactttattcaaatcagaaaacaaggtatatctggacctctttaa
>Irm3_petB
atgtggctttatgattggtctgaagaaggtttagaaattcaatgtattggagatgatatt
ctaggaaaattagtaccaccccatgttaatatattttactgctttggaggtgtggtatcc
cttttgctcttatttcaaataatcagtggtcttggattgacaatgtattacagtccaagt
gttgtttctgcattttcatcggtacttaatatagttggacaagtatatttaggttggtta
aatcgttctattcataggtggtctggatctagtatggtatctgcactaatattgcatgct
ttccgtgtttatttaacaggaggatttaaaaaagctcgtgagctaatctggatgacaggt
ataattttaggagtttgtaccgtgttgtttggagtaacaggatattgattagcttgggat
caattaggttattgggcatgtaaaatagtgacaggagtacctgaaggactagataagttt
ttatttggtgttggctttcttttagttttaataattagaggtggctttagtgttagttct
ggtaggctcacaagattctatagcattcacacattccttctcccaattgtaacattgact
ttagttatcattcactttattcaaatcagaaaacaaggtatatccggacctctttaa

>Irm4_petB
atgtggctttatgattggtctgaagaaggtttagaaattcaatgtattggagatgatatt
ctaggaaaattagtaccaccccatgttaatatattttactgctttggaggtgtggtatcc
cttttgctcttatttcaaataatcagtggtcttggattgacaatgtattacagtccaagt
gttgtttctgcattttcatcggtacttaatatagttggccaagtacatttaggttggtta
aatcgttctattcacaggtggtctggggctagtatggtatctgcactaatattgcatgct
ttccgtgtttatttaacaggaggatttaaaaaagctcgtgagctaatctggatgacaggt
ataattttaggagtttgtaccgtgttgtttggagtaacaggatattgattagcttgggat
caattaggttattgggcatgtaaaatagtgacaggagtacctgaaggactagataagttt
ttatttggtgttggctcgctcttggttttaataattagaggtggctttagtgttagttct
ggtaggctcacaagattctatagcattcacacattccttctcccaattgtaacattgact
ttagttatcattcactttattcaaatcagaaaacaaggtatatccggacctctttaa
>Irm5_petB
atgtggctttatgattggtctgaagaaggtttagaaattcaatgtattggagatgatatt
ctaggtaaattagtaccacctcatgttaatatattttactgctttggaggtgtggtatcc
cttttgctcttatttcaaataatcagtggtcttggattgacaatgtattacactccaagt
gttgtctctgcattttcatcggtacttaatatagttggccaagtacatttaggttggtta
aatcgttctattcacagatggtctggatctagtatggtatctgcactaatattgcatgct
tttcgtgtttatttaacaggaggatttaaaaaagctcgtgagctaatttggatgacaggt
ataattttaggagtttgcaccgtgttgtttggagtaacaggatattgattagcttgggat
caagtagcttattgggcatgtaaaatagtgacaggagtacctgaaggactagacaagttg
ttatttggtgttggcttgcttttggttctaataattagaggtggctttagtgttagttct
ggtaggctcacaagattctatagcattcacacatttcttctcccaattgtaacattgagt
ttagttatcattcactttattcaaatcagaaaacaaggtatatctggacctctttaa
>Irm7_petB
atgtggctttatgattggtctgaagaaggtttagaaattcaatgtattggagatgatatt
ctaggtaaattagtaccacctcatgttaatatattttactgctttggaggtgtggtatcc
cttttgctcttatttcaaataatcagtggtcttggattgacaatgtattacactccaagt
gttgtctctgcattttcatcggtacttaatatagttggccaagtacatttaggttggtta
aatcgttctattcacagatggtctgggtctagtatggtatctgcactaatattgcatgct
ttccgtgtttatttaacaggaggatttaaaaaagctcgtgagctaatttggatgacaggt
ataattttaggagtttgcaccgtgttgtttggagtaacaggatattgattagcttgggat
caagtaggttattgggcatgtaaaatagtgacaggagtacctgaaggactagacaagttg
ttattcggtgttggctttcttttagttctaataattagaggtggctttagtgttagttct
ggtaggctcacaagattctatagcattcacacattccttctcccaattgtaacattaagt
ttagttatcattcactttattcaaatcagaaaacaaggtatatctggacctctttaa
>Irm9_petB
atgtggctttatgattggtctgaagaaggtttagaaattcaatgtattggagatgatatt
ctaggtaaattagtaccacctcatgttaatatattttactgctttggaggtgtggtatcc
cttttgctcttatttcaaataatcagtggtcttggattgacaatgtattacagtccaagt
gttgtttctgcattttcatcggtacttaatatagttggacaagtatatttaggttggtta
aatcgttctattcataggtggtctggatctagtatggtatctgcactaatattgcatgct
tttcgtgtttatttaacaggaggatttaaaaaagctcgtgagctaatctggatgacaggt
ataattttaggagtttgtaccgtgttgtttggagtaacaggatattgattagcttgggat
caagtaggttattgggcatgtaaaatagtgacaggagtacctgaaggactagacaagttg
ttattcggtgttggctttcttttagttctaataattagaggtggctttagtgttagttct
ggtaggctcacaagattctatagcattcacacattccttctcccaattgtaacattgact
ttagttatcattcactttattcaaatcagaaaacaaggtatatccggacctctttaa
>Isy12_petB
atgtggctttatgattggtctgaagaaggtttagaaattcaatgtattggagatgatatt
ctaggtaaattagtaccacctcatgttaatatattttactgctttggaggtgtggtatcc
cttttgctcttatttcaaataatcagtggtcttggattgacaatgtattacagtccaagt
gttgtctctgcattttcatcggtacttaatatagttggccaagtatatttaggttggtta
aatcgttctattcataggtggtctggatctagtatggtatctgcactaatattgcatgct
tttcgtgtttatttaacaggaggatttaaaaaagctcgtgagctaatttggatgacaggt
ataattctaggagtttgcaccgtgttgtttggagtaacaggatattgattagcttgggat
caagtaggttattgggcatgtaaaatagtgacaggagtacctgaaggactagacaagttg

ttatttggtgttggcttgcttttggttctaataattagaggtggctttagtgttagttct
ggtaggctcacaagattctatagcattcacacattccttctcccaattgtaacattgact
ttagttatcattcactttattcaaatcagaaaacaaggtatatctggacctctttaa
>Isy15_petB
atgtggctttatgattggtctgaagaaggtttagaaattcaatgtattggagatgatatt
ctaggaaaattagtaccaccccatgttaatatattttactgctttggaggtgtggtatcc
cttttgctcttatttcaaataatcagtggtcttggattgacaatgtattacagtccaagt
gttgtttctgcattttcatcggtacttaatatagttggacaagtatatttaggttggtta
aatcgttctattcacaggtggtctggggctagtatggtatctgcactaatattgcatgct
ttccgtgtttatttaacaggaggatttaaaaaagctcgtgagctaatctggatgacaggt
ataattttaggagtttgtaccgtgttgtttggagtaacaggatattgattagcttgggat
caattaggttattgggcatgtaaaatagtgacaggagtacctgaaggactagataagttt
ttatttggtgttggctcgctcttggttttaataattagaggtggctttagtgttagttct
ggtaggctcacaagattctatagcattcacacattccttctcccaattgtaacattgact
ttagttatcattcactttattcaaatcagaaaacaaggtatatccggacctctttaa
>Isy16_petB
atgtggctttatgattggtctgaagaaggtttagaaattcaatgtattggagatgatatt
ctaggtaaattagtaccacctcatgttaatatattttactgctttggaggtgtggtatcc
cttttgctcttatttcaaataatcagtggtcttggattgacaatgtattacagtccaagt
gttgtctctgcattttcatcggtacttaatatagttggccaagtacatttaggttggtta
aatcgttctattcacagatggtctggatctagtatggtatctgcactaatattgcatgct
tttcgtgtttatttaacaggaggatttaaaaaagctcgtgagctaatttggatgacaggt
ataattttaggagtttgcaccgtgttgtttggagtaacaggatattgattagcttgggat
caagtagcttattgggcatgtaaaatagtgacaggagtacctgaaggactagacaagttg
ttatttggtgttggcttgcttttggttctaataattagaggtggctttagtgttagttct
ggtaggctcacaagattctatagcattcacacatttcttctcccaattgtaacattgagt
ttagttatcattcactttattcaaatcagaaaacaaggtatatctggacctctttaa
>Isy17_petB
atgtggctttatgattggtctgaagaaggtttagaaattcaatgtattggagatgatatt
ctaggtaaattagtaccacctcatgttaatatattttactgctttggaggtgtggtatcc
cttttgctcttatttcaaataatcagtggtcttggattgacaatgtattacagtccaagt
gttgtctctgcattttcatcggtacttaatatagttggccaagtatatttaggttggtta
aatcgttctattcacagatggtctggatctagtatggtatctgcactaatattgcatgct
tttcgtgtttatttaacaggaggatttaaaaaagctcgtgagctaatttggatgacaggt
ataattttaggagtttgcaccgtgttgtttggagtaacaggatattgattagcttgggat
caagtagcttattgggcatgtaaaatagtgacaggagtacctgaaggactagacaagttg
ttatttggtgttggcttgcttttggttctaataattagaggtggctttagtgttagttct
ggtaggctcacaagattctatagcattcacacatttcttctcccaattgtaacattgagt
ttagttatcattcactttattcaaatcagaaaacaaggtatatctggacctctttaa
>Isy18_petB
atgtggctttatgattggtctgaagaaggtttagaaattcaatgtattggagatgatatt
ctaggtaaattagtaccacctcatgttaatatattttactgctttggaggtgtggtatcc
cttttgctcttatttcaaataatcagtggtcttggattgacaatgtattacagtccaagt
gttgtctctgcattttcatcggtacttaatatagttggccaagtacatttaggttggtta
aatcgttctattcacagatggtctggatctagtatggtatctgcactaatattgcatgct
tttcgtgtttatttaacaggaggatttaaaaaagctcgtgagctaatttggatgacaggt
ataattctaggagtttgcaccgtgttgtttggagtaacaggatattgattagcttgggat
caagtagcttattgggcatgtaaaatagtgacaggagtacctgaaggactagacaagttg
ttatttggtgttggcttgcttttggttctaataattagaggtggctttagtgttagttct
ggtaggctcacaagattctatagcattcacacatttcttctcccaattgtaacattgagt
ttagttatcattcactttattcaaatcagaaaacaaggtatatctggacctctttaa
>Isy21_petB
atgtggctttatgattggtctgaagaaggtttagaaattcaatgtattggagatgatatt
ctaggtaaattagtaccacctcatgttaatatattttactgctttggaggtgtggtatcc
cttttgctcttatttcaaataatcagtggtcttggattgacaatgtattacagtccaagt
gttgtctctgcattttcatcggtacttaatatagttggccaagtatatttaggttggtta
aatcgttctattcacaggtggtctggatctagtatggtatctgcactaatattgcatgct

tttcgtgtttatttaacaggaggatttaaaaaagctcgtgagctaatttggatgacaggt
ataattttaggagtttgcaccgtgttgtttggagtaacaggatattgattagcttgggat
caagtagcttattgggcatgtaaaatagtgacaggagtacctgaaggactagacaagttg
ttatttggtgttggcttgcttttggttctaataattagaggtggctttagtgttagttct
ggtaggctcacaagattctatagcattcacacattccttctcccaattgtaacattaagt
ttagttatcattcactttattcaaatcagaaaacaaggtatatctggacctctttaa
>Isy22_petB
atgtggctttatgattggtctgaagaaggtttagaaattcaatgtattggagatgatatt
ctaggtaaattagtaccacctcatgttaatatattttactgctttggaggtgtggtatcc
cttttgctcttatttcaaataatcagtggtcttggattgacaatgtattacagtccaagt
gttgtctctgcattttcatcggtacttaatatagttggccaagtatatttaggttggtta
aatcgttctattcataggtggtctggatctagtatggtatctgcactaatattgcatgct
tttcgtgtttatttaacaggaggatttaaaaaagctcgtgagctaatttggatgacaggt
ataattttaggagtttgcaccgtgttgtttggagtaacaggatattgattagcttgggat
caagtagcttattgggcatgtaaaatagtgacaggagtacctgaaggactagacaagttg
ttatttggtgttggcttgcttttggttctaataattagaggtggctttagtgttagttct
ggtaggctcacaagattctatagcattcacacattccttctcccaattgtaacattaagt
ttagttatcattcactttattcaaatcagaaaacaaggtatatctggacctctttaa
>Isy23_petB
atgtggctttatgattggtctgaagaaggtttagaaattcaatgtattggagatgatatt
ctaggtaaattagtaccacctcatgttaatatattttactgctttggaggtgtggtatcc
cttttgctcttatttcaaataatcagtggtcttggattgacaatgtattacactccaagt
gttgtctctgcattttcatcggtacttaatatagttggccaagtacatttaggttggtta
aatcgttctattcacaggtggtctggatctagtatggtatctgcactaatattgcatgct
tttcgtgtttatttaacaggaggatttaaaaaagctcgtgagctaatttggatgacaggt
ataattttaggagtttgcaccgtgttgtttggagtaacaggatattgattagcttgggat
caagtagcttattgggcatgtaaaatagtgacaggagtacctgaaggactagacaagttg
ttatttggtgttggcttgcttttggttctaataattagaggtggctttagtgttagttct
ggtaggctcacaagattctatagcattcacacattccttctcccaattgtaacattgagt
ttagttatcattcactttattcaaatcagaaaacaaggtatatctggacctctttaa
>Isy24_petB
atgtggctttatgattggtctgaagaaggtttagaaattcaatgtattggagatgatatt
ctaggtaaattagtaccacctcatgttaatatattttactgctttggaggtgtggtatcc
cttttgctcttatttcaaataatcagtggtcttggattgacaatgtattacagtccaagt
gttgtctctgcattttcatcggtacttaatatagttggccaagtacatttaggttggtta
aatcgttctattcacagatggtctggatctagtatggtatctgcactaatattgcatgct
tttcgtgtttatttaacaggaggatttaaaaaagctcgtgagctaatttggatgacaggt
ataattttaggagtttgcaccgtgttgtttggagtaacaggatattgattagcttgggat
caagtagcttattgggcatgtaaaatagtgacaggagtacctgaaggactagacaagttg
ttatttggtgttggcttgcttttggttctaataattagaggtggctttagtgttagttct
ggtaggctcacaagattctatagcattcacacatttcttctcccaattgtaacattgagt
ttagttatcattcactttattcaaatcagaaaacaaggtatatctggacctctttaa
>Isy25_petB
atgtggctttatgattggtctgaagaaggtttagaaattcaatgtattggagatgatatt
ctaggtaaattagtaccacctcatgttaatatattttactgctttggaggtgtggtatcc
cttttgctcttatttcaaataatcagtggtcttggattgacaatgtattacagtccaagt
gttgtctctgcattttcatcggtacttaatatagttggccaagtatatttaggttggtta
aatcgttctattcataggtggtctggatctagtatggtatctgcactaatattgcatgct
tttcgtgtttatttaacaggaggatttaaaaaagctcgtgagctaatttggatgacaggt
ataattttaggagtttgcaccgtgttgtttggagtaacaggatattgattagcttgggat
caagtagcttattgggcatgtaaaatagtgacaggagtacctgaaggactagacaagttg
ttatttggtgttggcttgcttttggttctaataattagaggtggctttagtgttagttct
ggtaggctcacaagattctatagcattcacacattccttctcccaattgtaacattgagt
ttagttatcattcactttattcaaatcagaaaacaaggtatatctggacctctttaa
>Isy26_petB
atgtggctttatgattggtctgaagaaggtttagaaattcaatgtattggagatgatatt
ctaggtaaattagtaccacctcatgttaatatattttactgctttggaggtgtggtatcc

cttttgctcttatttcaaataatcagtggtcttggattgacaatgtattacagtccaagt
gttgtctctgcattttcatcggtacttaatatagttggccaagtacatttaggttggtta
aatcgttctattcacaggtggtctggatctagtatggtatctgcactaatattgcatgct
tttcgtgtttatttaacaggaggatttaaaaaagctcgtgagctaatttggatgacaggt
ataattctaggagtttgcaccgtgttgtttggagtaacaggatattgattagcttgggat
caagtagcttattgggcatgtaaaatagtgacaggagtacctgaaggactagacaagttg
ttatttggtgttggcttgcttttggttctaataattagaggtggctttagtgttagttct
ggtaggctcacaagattctatagcattcacacattccttctcccaattgtaacattgagt
ttagttatcattcactttattcaaatcagaaaacaaggtatatctggacctctttaa
>Isy27_petB
atgtggctttatgattggtctgaagaaggtttagaaattcaatgtattggagatgatatt
ctaggtaaattagtaccacctcatgttaatatattttactgctttggaggtgtggtatcc
cttttgctcttatttcaaataatcagtggtcttggattgacaatgtattacagtccaagt
gttgtctctgcattttcatcggtacttaatatagttggccaagtacatttaggttggtta
aatcgttctattcacaggtggtctggatctagtatggtatctgcactaatattgcatgct
tttcgtgtttatttaacaggaggatttaaaaaagctcgtgagctaatttggatgacaggt
ataattttaggagtttgcaccgtgttgtttggagtaacaggatattgattagcttgggat
caagtagcttattgggcatgtaaaatagtgacaggagtacctgaaggactagacaagttg
ttatttggtgttggcttgcttttggttctaataattagaggtggctttagtgttagttct
ggtaggctcacaagattctatagcattcacacattccttctcccaattgtaacattgagt
ttagttatcattcactttattcaaatcagaaaacaaggtatatctggacctctataa
>Isy4_petB
atgtggctttatgattggtctgaagaaggtttagaaattcaatgtattggagatgatatt
ctaggtaaattagtaccacctcatgttaatatattttactgctttggaggtgtggtatcc
cttttgctcttatttcaaataatcagtggtcttggattgacaatgtattacagtccaagt
gttgtctctgcattttcatcggtacttaatatagttggccaagtacatttaggttggtta
aatcgttctattcacagatggtctggatctagtatggtatctgcactaatattgcatgct
tttcgtgtttatttaacaggaggatttaaaaaagctcgtgagctaatttggatgacaggt
ataattttaggagtttgcaccgtgttgtttggagtaacaggatattgattagcttgggat
caagtagcttattgggcatgtaaaatagtgacaggagtacctgaaggactagacaagttg
ttatttggtgttggcttgcttttggttctaataattagaggtggctttagtgttagttct
ggtaggctcacaagattctatagcattcacacatttcttctcccaattgtaacattgagt
ttagttatcattcactttattcaaatcagaaaacaaggtatatctggacctctttaa
>Isy7_petB
atgtggctttatgattggtctgaagaaggtttagaaattcaatgtattggagatgatatt
ctaggtaaattagtaccacctcatgttaatatattttactgctttggaggtgtggtatcc
cttttgctcttatttcaaataatcagtggtcttggattgacaatgtattacagtccaagt
gttgtctctgcattttcatcggtacttaatatagttggccaagtacatttaggttggtta
aatcgttctattcacagatggtctggatctagtatggtatctgcactaatattgcatgct
tttcgtgtttatttaacaggaggatttaaaaaagctcgtgagctaatttggatgacaggt
ataattttaggagtttgcaccgtgttgtttggagtaacaggatattgattagcttgggat
caattagcttattgggcatgtaaaatagtgacaggagtacctgaaggactagataagttg
ttatttggtgttggcttgcttttggttctaataattagaggtggctttagtgttagttct
ggtaggctcacaagattctatagcattcacacatttcttctcccaattgtaacattgagt
ttagttatcattcactttattcaaatcagaaaacaaggtatatctggacctctttaa
>Isy8_petB
atgtggctttatgattggtctgaagaaggtttagaaattcaatgtattggagatgatatt
ctaggtaaattagtaccacctcatgttaatatattttactgctttggaggtgtggtatcc
cttttgctcttatttcaaataatcagtggtcttggattgacaatgtattacagtccaagt
gttgtctctgcattttcatcggtacttaatatagttggccaagtacatttaggttggtta
aatcgttctattcacaggtggtctggatctagtatggtatctgcactaatattgcatgct
tttcgtgtttatttaacaggaggatttaaaaaagctcgtgagctaatttggatgacaggt
ataattttaggagtttgcaccgtgttgtttggagtaacaggatattgattagcttgggat
caagtagcttattgggcatgtaaaatagtgacaggagtacctgaaggactagacaagttg
ttatttggtgttggcttgcttttggttctaataattagaggtggctttagtgttagttct
ggtaggctcacaagattctatagcattcacacattccttctcccaattgtaacattgagt
ttagttatcattcactttattcaaatcagaaaacaaggtatatctggacctctttaa

>KrA1_petB
atgtggctttatgattggtctgaagaaggtttagaaattcaatgtattggagatgatatt
ctaggtaaattagtaccacctcatgttaatatattttactgctttggaggtgtggtatcc
cttttgctcttatttcaaataatcagtggtcttggattgacaatgtattacagtccaagt
gttgtctctgcattttcatcggtacttaatatagttggccaagtacatttaggttggtta
aatcgttctattcacagatggtctggatctagtatggtatctgcactaatattgcatgct
tttcgtgtttatttaacaggaggatttaaaaaagctcgtgagctaatttggatgacaggt
ataattttaggagtttgcaccgtgttgtttggagtaacaggatattgattagcttgggat
caagtagcttattgggcatgtaaaatagtgacaggagtacctgaaggactagacaagttg
ttatttggtgttggcttgcttttggttctaataattagaggtggctttagtgttagttct
ggtaggctcacaagattctatagcattcacacatttcttctcccaattgtaacattgagt
ttagttatcattcactttattcaaatcagaaaacaaggtatatctggacctctttaa
>KrA10_petB
atgtggctttatgattggtctgaagaaggtttagaaattcaatgtattggagatgatatt
ctaggtaaattagtaccacctcatgttaatatattttactgctttggaggtgtggtatcc
cttttgctcttatttcaaataatcagtggtcttggattgacaatgtattacagtccaagt
gttgtctctgcattttcatcggtacttaatatagttggccaagtatatttaggttggtta
aatcgttctattcataggtggtctggatctagtatggtatctgcactaatattgcatgct
tttcgtgtttatttaacaggaggatttaaaaaagctcgtgagctaatttggatgacaggt
ataattttaggagtttgcaccgtgttgtttggagtaacaggatattgattagcttgggat
caagtagcttattgggcatgtaaaatagtgacaggagtacctgaaggactagacaagttg
ttatttggtgttggcttgcttttggttctaataattagaggtggctttagtgttagttct
ggtaggctcacaagattctatagcattcacacatttcttctcccaattgtaacattgagt
ttagttatcattcactttattcaaatcagaaaacaaggtatatctggacctctttaa
>KrA11_petB
atgtggctttatgattggtctgaagaaggtttagaaattcaatgtattggagatgatatt
ctaggtaaattagtaccacctcatgttaatatattttactgctttggaggtgtggtatcc
cttttgctcttatttcaaataatcagtggtcttggattgacaatgtattacagtccaagt
gttgtctctgcattttcatcggtacttaatatagttggccaagtatatttaggttggtta
aatcgttctattcataggtggtctggatctagtatggtatctgcactaatattgcatgct
tttcgtgtttatttaacaggaggatttaaaaaagctcgtgagctaatttggatgacaggt
ataattttaggagtttgcaccgtgttgtttggagtaacaggatattgattagcttgggat
caagtaggttattgggcatgtaaaatagtgacaggagtacctgaaggactagacaagttg
ttatttggtgttggcttgcttttggttctaataattagaggtggctttagtgttagttct
ggtaggctcacaagattctatagcattcacacatttcttctcccaattgtaacattgagt
ttagttatcattcactttattcaaatcagaaaacaaggtatatctggacctctttaa
>KrA12_petB
atgtggctttatgattggtctgaagaaggtttagaaattcaatgtattggagatgatatt
ctaggtaaattagtaccacctcatgttaatatattttactgctttggaggtgtggtatcc
cttttgctcttatttcaaataatcagtggtcttggattgacaatgtattacagtccaagt
gttgtctctgcattttcatcggtacttaatatagttggccaagtacatttaggttggtta
aatcgttctattcacagatggtctggatctagtatggtatctgcactaatattgcatgct
tttcgtgtttatttaacaggaggatttaaaaaagctcgtgagctaatttggatgacaggt
ataattctaggagtttgcaccgtgttgtttggagtaacaggatattgattagcttgggat
caagtaggttattgggcatgtaaaatagtgacaggagtacctgaaggactagacaagttg
ttattcggtgttggctttcttttagttctaataattagaggtggctttagtgttagttct
ggtaggctcacaagattctatagcattcacacattccttctcccaattgtaacattaagt
ttagttatcattcactttattcaaatcagaaaacaaggtatatctggacctctttaa
>KrA13_petB
atgtggctttatgattggtctgaagaaggtttagaaattcaatgtattggagatgatatt
ctaggtaaattagtaccacctcatgttaatatattttactgctttggaggtgtggtatcc
cttttgctcttatttcaaataatcagtggtcttggattgacaatgtattacagtccaagt
gttgtctctgcattttcatcggtacttaatatagttggccaagtatatttaggttggtta
aatcgttctattcatagatggtctggatctagtatggtatctgcactaatattgcatgct
tttcgtgtttatttaacaggaggatttaaaaaagctcgtgagctaatttggatgacaggt
ataagtttaggagtttgcaccgtgttgtttggagtaacaggatattgattagcttgggat
caagtagcttattgggcatgtaaaatagtgacaggagtacctgaaggactagacaagttg

ttattcggtgttggctttcttttagttctaataattagaggtggctttagtgttagttct
ggtaggctcacaagattctatagcattcacacattccttctcccaattgtaacattaagt
ttagttatcattcactttattcaaatcagaaaacaaggtatatctggacctctttaa
>KrA14_petB
atgtggctttatgattggtctgaagaaggtttagaaattcaatgtattggagatgatatt
ctaggtaaattagtaccacctcatgttaatatattttactgctttggaggtgtggtatcc
cttttgctcttatttcaaataatcagtggtcttggattgacaatgtattacagtccaagt
gttgtctctgcattttcatcggtacttaatatagttggccaagtacatttaggttggtta
aatcgttctattcacagatggtctggatctagtatggtatctgcactaatattgcatgct
tttcgtgtttatttaacaggaggatttaaaaaagctcgtgagctaatttggatgacaggt
ataattctaggagtttgcaccgtgttgtttggagtaacaggatattgattagcttgggat
caagtagcttattgggcatgtaaaatagtgacaggagtacctgaaggactagacaagttg
ttatttggtgttggcttgcttttggttctaataattagaggtggctttagtgttagttct
ggtaggctcacaagattctatagcattcacacatttcttctcccaattgtaacattaagt
ttagttatcattcactttattcaaatcagaaaacaaggtatatctggacctctttaa
>KrA15_petB
atgtggctttatgattggtctgaagaaggtttagaaattcaatgtattggagatgatatt
ctaggtaaattagtaccacctcatgttaatatattttactgctttggaggtgtggtatcc
cttttgctcttatttcaaataatcagtggtcttggattgacaatgtattacagtccaagt
gttgtctctgcattttcatcggtacttaatatagttggccaagtatatttaggttggtta
aatcgttctattcataggtggtctggatctagtatggtatctgcactaatattgcatgct
tttcgtgtttatttaacaggaggatttaaaaaagctcgtgagctaatttggatgacaggt
ataattttaggagtttgcaccgtgttgtttggagtaacaggatattgattagcttgggat
caagtagcttattgggcatgtaaaatagtgacaggagtacctgaaggactagacaagttg
ttatttggtgttggcttgcttttggttctaataattagaggtggctttagtgttagttct
ggtaggctcacaagattctatagcattcacacatttcttctcccaattgtaacattgagt
ttagttatcattcactttattcaaatcagaaaacaaggtatatctggacctctttaa
>KrA2_petB
atgtggctttatgattggtctgaagaaggtttagaaattcaatgtattggagatgatatt
ctaggtaaattagtaccacctcatgttaatatattttactgctttggaggtgtggtatcc
cttttgctcttatttcaaataatcagtggtcttggattgacaatgtattacagtccaagt
gttgtctctgcattttcatcggtacttaatatagttggccaagtacatttaggttggtta
aatcgttctattcacagatggtctggatctagtatggtatctgcactaatattgcatgct
tttcgtgtttatttaacaggaggatttaaaaaagctcgtgagctaatttggatgacaggt
ataattttaggagtttgcaccgtgttgtttggagtaacaggatattgattagcttgggat
caagtagcttattgggcatgtaaaatagtgacaggagtacctgaaggactagacaagttg
ttatttggtgttggcttgcttttggttctaataattagaggtggctttagtgttagttct
ggtaggctcacaagattctatagcattcacacatttcttctcccaattgtaacattgagt
ttagttatcattcactttattcaaatcagaaaacaaggtatatctggacctctttaa
>KrA3_petB
atgtggctttatgattggtctgaagaaggtttagaaattcaatgtattggagatgatatt
ctaggtaaattagtaccacctcatgttaatatattttactgctttggaggtgtggtatcc
cttttgctcttatttcaaataatcagtggtcttggattgacaatgtattacagtccaagt
gttgtctctgcattttcatcggtacttaatatagttggccaagtacatttaggttggtta
aatcgttctattcataggtggtctggatctagtatggtatctgcactaatattgcatgct
tttcgtgtttatttaacaggaggatttaaaaaagctcgtgagctaatttggatgacaggt
ataattttaggagtttgcaccgtgttgtttggagtaacaggatattgattagcttgggat
caagtagcttattgggcatgtaaaatagtgacaggagtacctgaaggactagacaagttg
ttatttggtgttggcttgcttttggttctaataattagaggtggctttagtgttagttct
ggtaggctcacaagattctatagcattcacacattccttctcccaattgtaacattgagt
ttagttatcattcactttattcaaatcagaaaacaaggtatatctggacctctttaa
>KrA4_petB
atgtggctttatgattggtctgaagaaggtttagaaattcaatgtattggagatgatatt
ctaggtaaattagtaccacctcatgttaatatattttactgctttggaggtgtggtatcc
cttttgctcttatttcaaataatcagtggtcttggattgacaatgtattacactccaagt
gttgtctctgcattttcatcggtacttaatatagttggccaagtatatttaggttggtta
aatcgttctattcataggtggtctggatctagtatggtatctgcactaatattgcatgct

tttcgtgtttatttaacaggaggatttaaaaaagctcgtgagctaatttggatgacaggt
ataattctaggagtttgcaccgtgttgtttggagtaacaggatattgattagcttgggat
caagtagcttattgggcatgtaaaatagtgacaggagtacctgaaggactagacaagttg
ttatttggtgttggcttgcttttggttctaataattagaggtggctttagtgttagttct
ggtaggctcacaagattctatagcattcacacatttcttctcccaattgtaacattgagt
ttagttatcattcactttattcaaatcagaaaacaaggtatatctggacctctttaa
>KrA5_petB
atgtggctttatgattggtctgaagaaggtttagaaattcaatgtattggagatgatatt
ctaggtaaattagtaccacctcatgttaatatattttactgctttggaggtgtggtatcc
cttttgctcttatttcaaataatcagtggtcttggattgacaatgtattacagtccaagt
gttgtctctgcattttcatcggtacttaatatagttggccaagtacatttaggttggtta
aatcgttctattcacagatggtctggatctagtatggtatctgcactaatattgcatgct
tttcgtgtttatttaacaggaggatttaaaaaagctcgtgagctaatttggatgacaggt
ataattttaggagtttgcaccgtgttgtttggagtaacaggatattgattagcttgggat
caagtagcttattgggcatgtaaaatagtgacaggagtacctgaaggactagacaagttg
ttatttggtgttggcttgcttttggttctaataattagaggtggctttagtgttagttct
ggtaggctcacaagattctatagcattcacacattccttctcccaattgtaacattaagt
ttagttatcattcactttattcaaatcagaaaacaaggtatatctggacctctttaa
>KrA6_petB
atgtggctttatgattggtctgaagaaggtttagaaattcaatgtattggagatgatatt
ctaggtaaattagtaccacctcatgttaatatattttactgctttggaggtgtggtatcc
cttttgctcttatttcaaataatcagtggtcttggattgacaatgtattacagtccaagt
gttgtctctgcattttcatcggtacttaatatagttggccaagtacatttaggttggtta
aatcgttctattcacagatggtctggatctagtatggtatctgcactaatattgcatgct
tttcgtgtttatttaacaggaggatttaaaaaagctcgtgagctaatttggatgacaggt
ataattttaggagtttgcaccgtgttgtttggagtaacaggatattgattagcttgggat
caagtagcttattgggcatgtaaaatagtgacaggagtacctgaaggactagacaagttg
ttatttggtgttggcttgcttttggttctaataattagaggtggctttagtgttagttct
ggtaggctcacaagattctatagcattcacacatttcttctcccaattgtaacattgagt
ttagttatcattcactttattcaaatcagaaaacaaggtatatctggacctctttaa
>KrA7_petB
atgtggctttatgattggtctgaagaaggtttagaaattcaatgtattggagatgatatt
ctaggtaaattagtaccacctcatgttaatatattttactgctttggaggtgtggtatcc
cttttgctcttatttcaaataatcagtggtcttggattgacaatgtattacagtccaagt
gttgtctctgcattttcatcggtacttaatatagttggccaagtatatttaggttggtta
aatcgttctattcataggtggtctggatctagtatggtatctgcactaatattgcatgct
tttcgtgtttatttaacaggaggatttaaaaaagctcgtgagctaatttggatgacaggt
ataattctaggagtttgcaccgtgttgtttggagtaacaggatattgattagcttgggat
caagtagcttattgggcatgtaaaatagtgacaggagtacctgaaggactagacaagttg
ttattcggtgttggctttcttttagttctaataattagaggtggctttagtgttagttct
ggtaggctcacaagattctatagcattcacacatttcttctcccaattgtaacattgagt
ttagttatcattcactttattcaaatcagaaaacaaggtatatctggacctctttaa
>KrA8_petB
atgtggctttatgattggtctgaagaaggtttagaaattcaatgtattggagatgatatt
ctaggtaaattagtaccacctcatgttaatatattttactgctttggaggtgtggtatcc
cttttgctcttatttcaaataatcagtggtcttggattgacaatgtattacagtccaagt
gttgtctctgcattttcatcggtacttaatatagttggccaagtacatttaggttggtta
aatcgttctattcacagatggtctggatctagtatggtatctgcactaatattgcatgct
tttcgtgtttatttaacaggaggatttaaaaaagctcgtgagctaatttggatgacaggt
ataattttaggagtttgcaccgtgttgtttggagtaacaggatattgattagcttgggat
caagtagcttattgggcatgtaaaatagtgacaggagtacctgaaggactagacaagttg
ttatttggtgttggcttgcttttggttctaataattagaggtggctttagtgttagttct
ggtaggctcacaagattctatagcattcacacatttcttctcccaattgtaacattgagt
ttagttatcattcactttattcaaatcagaaaacaaggtatatctggacctctttaa
>KrA9_petB
atgtggctttatgattggtctgaagaaggtttagaaattcaatgtattggagatgatatt
ctaggtaaattagtaccacctcatgttaatatattttactgctttggaggtgtggtatcc

cttttgctcttatttcaaataatcagtggtcttggattgacaatgtattacagtccaagt
gttgtctctgcattttcatcggtacttaatatagttggccaagtacatttaggttggtta
aatcgttctattcacagatggtctggatctagtatggtatctgcactaatattgcatgct
tttcgtgtttatttaacaggaggatttaaaaaagctcgtgagctaatttggatgacaggt
ataattttaggagtttgcaccgtgttgtttggagtaacaggatattgattagcttgggat
caagtagcttattgggcatgtaaaatagtgacaggagtacctgaaggactagacaagttg
ttatttggtgttggcttgcttttggttctaataattagaggtggctttagtgttagttct
ggtaggctcacaagattctatagcattcacacatttcttctcccaattgtaacattgagt
ttagttatcattcactttattcaaatcagaaaacaaggtatatctggacctctttaa
>KrC1_petB
atgtggctttatgattggtctgaagaaggtttagaaattcaatgtattggagatgatatt
ctaggtaaattagtaccacctcatgttaatatattttactgctttggaggtgtggtatcc
cttttgctcttatttcaaataatcagtggtcttggattgacaatgtattacagtccaagt
gttgtctctgcattttcatcggtacttaatatagttggccaagtacatttaggttggtta
aatcgttctattcacagatggtctggatctagtatggtatctgcactaatattgcatgct
tttcgtgtttatttaacaggaggatttaaaaaagctcgtgagctaatttggatgacaggt
ataattttaggagtttgcaccgtgttgtttggagtaacaggatattgattagcttgggat
caagtagcttattgggcatgtaaaatagtgacaggagtacctgaaggactagacaagttg
ttatttggtgttggcttgcttttggttctaataattagaggtggctttagtgttagttct
ggtaggctcacaagattctatagcattcacacatttcttctcccaattgtaacattgagt
ttagttatcattcactttattcaaatcagaaaacaaggtatatctggacctctttaa
>KrC10_petB
atgtggctttatgattggtctgaagaaggtttagaaattcaatgtattggagatgatatt
ctaggtaaattagtaccacctcatgttaatatattttactgctttggaggtgtggtatcc
cttttgctcttatttcaaataatcagtggtcttggattgacaatgtattacagtccaagt
gttgtctctgcattttcatcggtacttaatatagttggccaagtacatttaggttggtta
aatcgttctattcacagatggtctggatctagtatggtatctgcactaatattgcatgct
tttcgtgtttatttaacaggaggatttaaaaaagctcgtgagctaatttggatgacaggt
ataattttaggagtttgcaccgtgttgtttggagtaacaggatattgattagcttgggat
caagtagcttattgggcatgtaaaatagtgacaggagtacctgaaggactagacaagttg
ttatttggtgttggcttgcttttggttctaataattagaggtggttttagtgttagttct
ggtaggctcacaagattctatagcattcacacatttcttctcccaattgtaacattgagt
ttagttatcattcactttattcaaatcagaaaacaaggtatatctggacctctttaa
>KrC11_petB
atgtggctttatgattggtctgaagaaggtttagaaattcaatgtattggagatgatatt
ctaggtaaattagtaccacctcatgttaatatattttactgctttggaggtgtggtatcc
cttttgctcttatttcaaataatcagtggtcttggattgacaatgtattacagtccaagt
gttgtctctgcattttcatcggtacttaatatagttggccaagtacatttaggttggtta
aatcgttctattcacagatggtctggatctagtatggtatctgcactaatattgcatgct
tttcgtgtttatttaacaggaggatttaaaaaagctcgtgagctaatttggatgacaggt
ataattttaggagtttgcaccgtgttgtttggagtaacaggatattgattagcttgggat
caagtagcttattgggcatgtaaaatagtgacaggagtacctgaaggactagacaagttg
ttatttggtgttggcttgcttttggttctaataattagaggtggttttagtgttagttct
ggtaggctcacaagattctatagcattcacacatttcttctcccaattgtaacattgagt
ttagttatcattcactttattcaaatcagaaaacaaggtatatctggacctctttaa
>KrC12_petB
atgtggctttatgattggtctgaagaaggtttagaaattcaatgtattggagatgatatt
ctaggtaaattagtaccacctcatgttaatatattttactgctttggaggtgtggtatcc
cttttgctcttatttcaaataatcagtggtcttggattgacaatgtattacagtccaagt
gttgtctctgcattttcatcggtacttaatatagttggccaagtatatttaggttggtta
aatcgttctattcacagatggtctggatctagtatggtatctgcactaatattgcatgct
tttcgtgtttatttaacaggaggatttaaaaaagctcgtgagctaatttggatgacaggt
ataattttaggagtttgcaccgtgttgtttggagtaacaggatattgattagcttgggat
caagtagcttattgggcatgtaaaatagtgacaggagtacctgaaggactagacaagttg
ttatttggtgttggcttgcttttggttctaataattagaggtggctttagtgttagttct
ggtaggctcacaagattctatagcattcacacatttcttctcccaattgtaacattgagt
ttagttatcattcactttattcaaatcagaaaacaaggtatatctggacctctttaa

>KrC13_petB
atgtggctttatgattggtctgaagaaggtttagaaattcaatgtattggagatgatatt
ctaggtaaattagtaccacctcatgttaatatattttactgctttggaggtgtggtatcc
cttttgctcttatttcaaataattagtggtcttggattgacaatgtattacagtccaagt
gttgtctctgcattttcatcggtacttaatatagttggccaagtacatttaggttggtta
aatcgttctattcataggtggtctggatctagtatggtatctgcactaatattgcatgct
tttcgtgtttatttaacaggaggatttaaaaaagctcgtgagctaatttggatgacaggt
ataattttaggagtttgcaccgtgttgtttggagtaacaggatattgattagcttgggat
caagtaggttattgggcatgtaaaatagtgacaggagtacctgaaggactagacaagttg
ttatttggtgttggcttgcttttggttctaataattagaggtggttttagtgttagttct
ggtaggctcacaagattctatagcattcacacatttcttctcccaattgtaacattgagt
ttagttatcattcactttattcaaatcagaaaacaaggtatatctggacctctttaa
>KrC14_petB
atgtggctttatgattggtctgaagaaggtttagaaattcaatgtattggagatgatatt
ctaggtaaattagtaccacctcatgttaatatattttactgctttggaggtgtggtatcc
cttttgctcttatttcaaataatcagtggtcttggattgacaatgtattacactccaagt
gttgtctctgcattttcatcggtacttaatatagttggccaagtatatttaggttggtta
aatcgttctattcataggtggtctggatctagtatggtatctgcactaatattgcatgct
tttcgtgtttatttaacaggaggatttaaaaaagctcgtgagctaatttggatgacaggt
ataattttaggagtttgcaccgtgttgtttggagtaacaggatattgattagcttgggat
caagtagcttattgggcatgtaaaatagtgacaggagtacctgaaggactagacaagttg
ttattcggtgttggctttcttttagttctaataattagaggtggctttagtgttagttct
ggtaggctcacaagattctatagcattcacacatttcttctcccaattgtaacattgagt
ttagttatcattcactttattcaaatcagaaaacaaggtatatctggacctctttaa
>KrC15_petB
atgtggctttatgattggtctgaagaaggtttagaaattcaatgtattggagatgatatt
ctaggtaaattagtaccacctcatgttaatatattttactgctttggaggtgtggtatcc
cttttgctcttatttcaaataatcagtggtcttggattgacaatgtattacagtccaagt
gttgtctctgcattttcatcggtacttaatatagttggccaagtacatttaggttggtta
aatcgttctattcacagatggtctggatctagtatggtatctgcactaatattgcatgct
tttcgtgtttatttaacaggaggatttaaaaaagctcgtgagctaatttggatgacaggt
ataattttaggagtttgcaccgtgttgtttggagtaacaggatattgattagcttgggat
caagtagcttattgggcatgtaaaatagtgacaggagtacctgaaggactagacaagttg
ttatttggtgttggcttgcttttggttctaataattagaggtggctttagtgttagttct
ggtaggctcacaagattctatagcattcacacatttcttctcccaattgtaacattgagt
ttagttatcattcactttattcaaatcagaaaacaaggtatatctggacctctttaa
>KrC2_petB
atgtggctttatgattggtctgaagaaggtttagaaattcaatgtattggagatgatatt
ctaggtaaattagtaccacctcatgttaatatattttactgctttggaggtgtggtatcc
cttttgctcttatttcaaataatcagtggtcttggattgacaatgtattacagtccaagt
gttgtctctgcattttcatcggtacttaatatagttggccaagtacatttaggttggtta
aatcgttctattcacagatggtctggatctagtatggtatctgcattaatattgcatgct
tttcgtgtttatttaacaggaggatttaaaaaagctcgtgagctaatttggatgacaggt
ataattctaggagtttgcaccgtgttgtttggagtaacaggatattgattagcttgggat
caagtagcttattgggcatgtaaaatagtgacaggagtacctgaaggactagacaagttg
ttattcggtgttggcttgcttttggttctaataattagaggtggctttagtgttagttct
ggtaggctcacaagattctatagcattcacacatttcttctcccaattgtaacattgagt
ttagttatcattcactttattcaaatcagaaaacaaggtatatctggacctctttaa
>KrC3_petB
atgtggctttatgattggtctgaagaaggtttagaaattcaatgtattggagatgatatt
ctaggtaaattagtaccacctcatgttaatatattttactgctttggaggtgtggtatcc
cttttgctcttatttcaaataatcagtggtcttggattgacaatgtattacagtccaagt
gttgtctctgcattttcatcggtacttaatatagttggccaagtacatttaggttggtta
aatcgttctattcacagatggtctggatctagtatggtatctgcactaatattgcatgct
tttcgtgtttatttaacaggaggatttaaaaaagctcgtgagctaatttggatgacaggt
ataattttaggagtttgcaccgtgttgtttggagtaacaggatattgattagcttgggat
caagtagcttattgggcatgtaaaatagtgacaggagtacctgaaggactagacaagttg

ttatttggtgttggcttgattttggttctaataattagaggtggttttagtgttagttct
ggtaggctcacaagattctatagcattcacacatttcttctcccaattgtaacattgagt
ttagttatcattcactttattcaaatcagaaaacaaggtatatctggacctctttaa
>KrC4_petB
atgtggctttatgattggtctgaagaaggtttagaaattcaatgtattggagatgatatt
ctaggtaaattagtaccacctcatgttaatatattttactgctttggaggtgtggtatcc
cttttgctcttatttcaaataatcagtggtcttggattgacaatgtattacagtccaagt
gttgtctctgcattttcatcggtacttaatatagttggccaagtacatttaggttggtta
aatcgttctattcacagatggtctggatctagtatggtatctgcactaatattgcatgct
tttcgtgtttatttaacaggaggatttaaaaaagctcgtgagctaatttggatgacaggt
ataattttaggagtttgcaccgtgttgtttggagtaacaggatattgattagcttgggat
caagtagcttattgggcatgtaaaatagtgacaggagtacctgaaggactagacaagttg
ttattcggtgttggctttcttttagttctaataattagaggtggctttagtgttagttct
ggtaggctcacaagattctatagcattcacacatttcttctcccaattgtaacattaagt
ttagttatcattcactttattcaaatcagaaaacaaggtatatctggacctctttaa
>KrC5_petB
atgtggctttatgattggtctgaagaaggtttagaaattcaatgtattggagatgatatt
ctaggtaaattagtaccacctcatgttaatatattttactgctttggaggtgtggtatcc
cttttgctcttatttcaaataatcagtggtcttggattgacaatgtattacagtccaagt
gttgtctctgcattttcatcggtacttaatatagttggccaagtacatttaggttggtta
aatcgttctattcacagatggtctggatctagtatggtatctgcattaatattgcatgct
tttcgtgtttatttaacaggaggatttaaaaaagctcgtgagctaatttggatgacaggt
ataattttaggagtttgcaccgtgttgtttggagtaacaggatattgattagcttgggat
caagtaggttattgggcatgtaaaatagtgacaggagtacctgaaggactagacaagttg
ttatttggtgttggcttgcttttggttctaataattagaggtggctttagtgttagttct
ggtaggctcacaagattctatagcattcacacattccttctcccaattgtaacattgagt
ttagttatcattcactttattcaaatcagaaaacaaggtatatctggacctctttaa
>KrC6_petB
atgtggctttatgattggtctgaagaaggtttagaaattcaatgtattggagatgatatt
ctaggtaaattagtaccacctcatgttaatatattttactgctttggaggtgtggtatcc
cttttgctcttatttcaaataatcagtggtcttggattgacaatgtattacagtccaagt
gttgtctctgcattttcatcggtacttaatatagttggccaagtacatttaggttggtta
aatcgttctattcacagatggtctggatctagtatggtatctgcactaatattgcatgct
tttcgtgtttatttaacaggaggatttaaaaaagctcgtgagctaatttggatgacaggt
ataattttaggagtttgcaccgtgttgtttggagtaacaggatattgattagcttgggat
caagtagcttattgggcatgtaaaatagtgacaggagtacctgaaggactagacaagttg
ttatttggtgttggcttgcttttggttctaataattagaggtggctttagtgttagttct
ggtaggctcacaagattctatagcattcacacatttcttctcccaattgtaacattgagt
ttagttatcattcactttattcaaatcagaaaacaaggtatatctggacctctttaa
>KrC7_petB
atgtggctttatgattggtctgaagaaggtttagaaattcaatgtattggagatgatatt
ctaggtaaattagtaccacctcatgttaatatattttactgctttggaggtgtggtatcc
cttttgctcttatttcaaataatcagtggtcttggattgacaatgtattacagtccaagt
gttgtctctgcattttcatcggtacttaatatagttggccaagtacatttaggttggtta
aatcgttctattcacagatggtctggatctagtatggtatctgcactaatattgcatgct
tttcgtgtttatttaacaggaggatttaaaaaagctcgtgagctaatttggatgacaggt
ataattctaggagtttgcaccgtgttgtttggagtaacaggatattgattagcttgggat
caagtagcttattgggcatgtaaaatagtgacaggagtacctgaaggactagacaagttg
ttatttggtgttggcttgcttttggttctaataattagaggtggctttagtgttagttct
ggtaggctcacaagattctatagcattcacacattccttctcccaattgtaacattaagt
ttagttatcattcactttattcaaatcagaaaacaaggtatatctggacctctttaa
>KrC8_petB
atgtggctttatgattggtctgaagaaggtttagaaattcaatgtattggagatgatatt
ctaggtaaattagtaccacctcatgttaatatattttactgctttggaggtgtggtatcc
cttttgctcttatttcaaataatcagtggtcttggattgacaatgtattacagtccaagt
gttgtctctgcattttcatcggtacttaatatagttggccaagtacatttaggttggtta
aatcgttctattcacagatggtctggatctagtatggtatctgcactaatattgcatgct

tttcgtgtttatttaacaggaggatttaaaaaagctcgtgagctaatttggatgacaggt
ataattttaggagtttgcaccgtgttgtttggagtaacaggatattgattagcttgggat
caagtagcttattgggcatgtaaaatagtgacaggagtacctgaaggactagacaagttg
ttatttggtgttggcttgcttttggttctaataattagaggtggctttagtgttagttct
ggtaggctcacaagattctatagcattcacacatttcttctcccaattgtaacattgagt
ttagttatcattcactttattcaaatcagaaaacaaggtatatctggacctctttaa
>KrC9_petB
atgtggctttatgattggtctgaagaaggtttagaaattcaatgtattggagatgatatt
ctaggtaaattagtaccacctcatgttaatatattttactgctttggaggtgtggtatcc
cttttgctcttatttcaaataatcagtggtcttggattgacaatgtattacactccaagt
gttgtctctgcattttcatcggtacttaatatagttggccaagtacatttaggttggtta
aatcgttctattcacagatggtctggatctagtatggtatctgcactaatattgcatgct
tttcgtgtttatttaacaggaggatttaaaaaagctcgtgagctaatttggatgacaggt
ataattttaggagtttgcaccgtgttgtttggagtaacaggatattgattagcttgggat
caagtagcttattgggcatgtaaaatagtgacaggagtacctgaaggactagacaagttg
ttatttggtgttggctttcttttagttctaataattagaggtggctttagtgttagttct
ggtaggctcacaagattctatagcattcacacatttcttctcccaattgtaacattgagt
ttagttatcattcactttattcaaatcagaaaacaaggtatatctggacctctttaa
>KrD1_petB
atgtggctttatgattggtctgaagaaggtttagaaattcaatgtattggagatgatatt
ctaggtaaattagtaccacctcatgttaatatattttactgctttggaggtgtggtatcc
cttttgctcttatttcaaataatcagtggtcttggattgacaatgtattacagtccaagt
gttgtctctgcattttcatcggtacttaatatagttggccaagtatatttaggttggtta
aatcgttctattcataggtggtctggatctagtatggtatctgcactaatattgcatgct
tttcgtgtttatttaacaggaggatttaaaaaagctcgtgagctaatttggatgacaggt
ataattttaggagtttgcaccgtgttgtttggagtaacaggatattgattagcttgggat
caagtagcttattgggcatgtaaaatagtgacaggagtacctgaaggactagacaagttg
ttatttggtgttggcttgcttttggttctaataattagaggtggctttagtgttagttct
ggtaggctcacaagattctatagcattcacacatttcttctcccaattgtaacattgagt
ttagttatcattcactttattcaaatcagaaaacaaggtatatctggacctctttaa
>KrD11_petB
atgtggctttatgattggtctgaagaaggtttagaaattcaatgtattggagatgatatt
ctaggtaaattagtaccacctcatgttaatatattttactgctttggaggtgtggtatcc
cttttgctcttatttcaaataatcagtggtcttggattgacaatgtattacactccaagt
gttgtctctgcattttcatcggtacttaatatagttggccaagtatatttaggttggtta
aatcgttctattcataggtggtctggatctagtatggtatctgcactaatattgcatgct
tttcgtgtttatttaacaggaggatttaaaaaagctcgtgagctaatttggatgacaggt
ataattctaggagtttgcaccgtgttgtttggagtaacaggatattgattagcttgggat
caagtaggttattgggcatgtaaaatagtgacaggagtacctgaaggactagacaagttg
ttatttggtgttggcttgcttttggttctaataattagaggtggctttagtgttagttct
ggtaggctcacaagattctatagcattcacacattccttctcccaattgtaacattaagt
ttagttatcattcactttattcaaatcagaaaacaaggtatatctggacctctttaa
>KrD13_petB
atgtggctttatgattggtctgaagaaggtttagaaattcaatgtattggagatgatatt
ctaggtaaattagtaccacctcatgttaatatattttactgctttggaggtgtggtatcc
cttttgctcttatttcaaataatcagtggtcttggattgacaatgtattacagtccaagt
gttgtctctgcattttcatcggtacttaatatagttggccaagtacatttaggttggtta
aatcgttctattcacagatggtctggatctagtatggtatctgcactaatattgcatgct
tttcgtgtttatttaacaggaggatttaaaaaagctcgtgagctaatttggatgacaggt
ataattttaggagtttgcaccgtgttgtttggagtaacaggatattgattagcttgggat
caagtagcttattgggcatgtaaaatagtgacaggagtacctgaaggactagacaagttg
ttatttggtgttggcttgcttttggttctaataattagaggtggctttagtgttagttct
ggtaggctcacaagattctatagcattcacacattccttctcccaattgtaacattgagt
ttagttatcattcactttattcaaatcagaaaacaaggtatatctggacctctttaa
>KrD3_petB
atgtggctttatgattggtctgaagaaggtttagaaattcaatgtattggagatgatatt
ctaggtaaattagtaccacctcatgttaatatattttactgctttggaggtgtggtatcc

cttttgctcttatttcaaataatcagtggtcttggattgacaatgtattacactccaagt
gttgtctctgcattttcatcggtacttaatatagttggccaagtacatttaggttggtta
aatcgttctattcatagatggtctggatctagtatggtatctgcactaatattgcatgct
tttcgtgtttatttaacaggaggatttaaaaaagctcgtgagctaatttggatgacaggt
ataattctaggagtttgcaccgtgttgtttggagtaacaggatattgattagcttgggat
caagtaggttattgggcatgtaaaatagtgacaggagtacctgaaggactagacaagttg
ttattcggtgttggctttcttttagttctaataattagaggtggctttagtgttagttct
ggtaggctcacaagattctatagcattcacacattccttctcccaattgtaacattaagt
ttagttatcattcactttattcaaatcagaaaacaaggtatatctggacctctttaa
>KrD8_petB
atgtggctttatgattggtctgaagaaggtttagaaattcaatgtattggagatgatatt
ctaggtaaattagtaccacctcatgttaatatattttactgctttggaggtgtggtatcc
cttttgctcttatttcaaataatcagtggtcttggattgacaatgtattacagtccaagt
gttgtctctgcattttcatcggtacttaatatagttggccaagtatatttaggttggtta
aatcgttctattcataggtggtctggatctagtatggtatctgcactaatattgcatgct
tttcgtgtttatttaacaggaggatttaaaaaagctcgtgagctaatttggatgacaggt
ataattctaggagtttgcaccgtgttgtttggagtaacaggatattgattagcttgggat
caagtaggttattgggcatgtaaaatagtgacaggagtacctgaaggactagacaagttg
ttatttggtgttggcttgcttttggttctaataattagaggtggctttagtgttagttct
ggtaggctcacaagattctatagcattcacacattccttctcccaattgtaacattaagt
ttagttatcattcactttattcaaatcagaaaacaaggtatatctggacctctttaa
>KrD9_petB
atgtggctttatgattggtctgaagaaggtttagaaattcaatgtattggagatgatatt
ctaggtaaattagtaccacctcatgttaatatattttactgctttggaggtgtggtatcc
cttttgctcttatttcaaataatcagtggtcttggattgacaatgtattacactccaagt
gttgtctctgcattttcatcggtacttaatatagttggccaagtacatttaggttggtta
aatcgttctattcacagatggtctggatctagtatggtatctgcactaatattgcatgct
tttcgtgtttatttaacaggaggatttaaaaaagctcgtgagctaatttggatgacaggt
ataattttaggagtttgcaccgtgttgtttggagtaacaggatattgattagcttgggat
caagtagcttattgggcatgtaaaatagtgacaggagtacctgaaggactagacaagttg
ttattcggtgttggctttcttttggttctaataattagaggtggctttagtgttagttct
ggtaggctcacaagattctatagcattcacacattccttctcccaattgtaacattgagt
ttagttatcattcactttattcaaatcagaaaacaaggtatatctggacctctttaa
>KrE1_petB
atgtggctttatgattggtctgaagaaggtttagaaattcaatgtattggagatgatatt
ctaggtaaattagtaccacctcatgttaatatattttactgctttggaggtgtggtatcc
cttttgctcttatttcaaataatcagtggtcttggattgacaatgtattacagtccaagt
gttgtctctgcattttcatcggtacttaatatagttggccaagtacatttaggttggtta
aatcgttctattcacagatggtctggatctagtatggtatctgcactaatattgcatgct
tttcgtgtttatttaacaggaggatttaaaaaagctcgtgagctaatttggatgacaggt
ataattttaggagtttgcaccgtgttgtttggagtaacaggatattgattagcttgggat
caagtagcttattgggcatgtaaaatagtgacaggagtacctgaaggactagacaagttg
ttatttggtgttggcttgcttttggttctaataattagaggtggttttagtgttagttct
ggtaggctcacaagattctatagcattcacacatttcttctcccaattgtaacattgagt
ttagttatcattcactttattcaaatcagaaaacaaggtatatctggacctctttaa
>KrE10_petB
atgtggctttatgattggtctgaagaaggtttagaaattcaatgtattggagatgatatt
ctaggtaaattagtaccacctcatgttaatatattttactgctttggaggtgtggtatcc
cttttgctcttatttcaaataatcagtggtcttggattgacaatgtattacactccaagt
gttgtctctgcattttcatcggtacttaatatagttggccaagtacatttaggttggtta
aatcgttctattcacagatggtctggatctagtatggtatctgcactaatattgcatgct
tttcgtgtttatttaacaggaggatttaaaaaagctcgtgagctaatttggatgacaggt
ataattttaggagtttgcaccgtgttgtttggagtaacaggatattgattagcttgggat
caagtaggttattgggcatgtaaaatagtgacaggagtacctgaaggactagacaagttg
ttatttggtgttggcttgcttttggttctaataattagaggtggctttagtgttagttct
ggtaggctcacaagattctatagcattcacacattccttctcccaattgtaacattaagt
ttagttatcattcactttattcaaatcagaaaacaaggtatatctggacctctttaa

>KrE11_petB
atgtggctttatgattggtctgaagaaggtttagaaattcaatgtattggagatgatatt
ctaggtaaattagtaccacctcatgttaatatattttactgctttggaggtgtggtatcc
cttttgctcttatttcaaataatcagtggtcttggattgacaatgtattacagtccaagt
gttgtctctgcattttcatcggtacttaatatagttggccaagtacatttaggttggtta
aatcgttctattcacagatggtctggatctagtatggtatctgcactaatattgcatgct
tttcgtgtttatttaacaggaggatttaaaaaagctcgtgagctaatttggatgacaggt
ataattctaggagtttgcaccgtgttgtttggagtaacaggatattgattagcttgggat
caagtagcttattgggcatgtaaaatagtgacaggagtacctgaaggactagacaagttg
ttatttggtgttggcttgcttttggttctaataattagaggtggctttagtgttagttct
ggtaggctcacaagattctatagcattcacacattccttctcccaattgtaacattgagt
ttagttatcattcactttattcaaatcagaaaacaaggtatatctggacctctttaa
>KrE12_petB
atgtggctttatgattggtctgaagaaggtttagaaattcaatgtattggagatgatatt
ctaggtaaattagtaccacctcatgttaatatattttactgctttggaggtgtggtatcc
cttttgctcttatttcaaataatcagtggtcttggattgacaatgtattacagtccaagt
gttgtctctgcattttcatcggtacttaatatagttggccaagtacatttaggttggtta
aatcgttctattcacagatggtctggatctagtatggtatctgcactaatattgcatgct
tttcgtgtttatttaacaggaggatttaaaaaagctcgtgagctaatttggatgacaggt
ataattttaggagtttgcaccgtgttgtttggagtaacaggatattgattagcttgggat
caagtagcttattgggcatgtaaaatagtgacaggagtacctgaaggactagacaagttg
ttatttggtgttggcttgcttttagttctaataattagaggtggctttagtgttagttct
ggtaggctcacaagattctatagcattcacacatttcttctcccaattgtaacattcagt
ttagttatcattcactttattcaaatcagaaaacaaggtatatctggacctctttaa
>KrE13_petB
atgtggctttatgattggtctgaagaaggtttagaaattcaatgtattggagatgatatt
ctaggtaaattagtaccacctcatgttaatatattttactgctttggaggtgtggtatcc
cttttgctcttatttcaaataatcagtggtcttggattgacaatgtattacagtccaagt
gttgtctctgcattttcatcggtacttaatatagttggccaagtatatttaggttggtta
aatcgttctattcacagatggtctggatctagtatggtatctgcactaatattgcatgct
tttcgtgtttatttaacaggaggatttaaaaaagctcgtgagctaatttggatgacaggt
ataattttaggagtttgcaccgtgttgtttggagtaacaggatattgattagcttgggat
caagtagcttattgggcatgtaaaatagtgacaggagtacctgaaggactagacaagttg
ttatttggtgttggcttgcttttggttctaataattagaggtggctttagtgttagttct
ggtaggctcacaagattctatagcattcacacattccttctcccaattgtaacattgagt
ttagttatcattcactttattcaaatcagaaaacaaggtatatctggacctctttaa
>KrE14_petB
atgtggctttatgattggtctgaagaaggtttagaaattcaatgtattggagatgatatt
ctaggtaaattagtaccacctcatgttaatatattttactgctttggaggtgtggtatcc
cttttgctcttatttcaaataatcagtggtcttggattgacaatgtattacagtccaagt
gttgtctctgcattttcatcggtacttaatatagttggccaagtacatttaggttggtta
aatcgttctattcacagatggtctggatctagtatggtatctgcactaatattgcatgct
tttcgtgtttatttaacaggaggatttaaaaaagctcgtgagctaatttggatgacaggt
ataattttaggagtttgcaccgtgttgtttggagtaacaggatattgattagcttgggat
caagtagcttattgggcatgtaaaatagtgacaggagtacctgaaggactagacaagttg
ttatttggtgttggcttgcttttggttctaataattagaggtggctttagtgttagttct
ggtaggctcacaagattctatagcattcacacatttcttctcccaattgtaacattgagt
ttagttatcattcactttattcaaatcagaaaacaaggtatatctggacctctttaa
>KrE15_petB
atgtggctttatgattggtctgaagaaggtttagaaattcaatgtattggagatgatatt
ctaggtaaattagtaccacctcatgttaatatattttactgctttggaggtgtggtatcc
cttttgctcttatttcaaataatcagtggtcttggattgacaatgtattacagtccaagt
gttgtctctgcattttcatcggtacttaatatagttggccaagtacatttaggttggtta
aatcgttctattcacagatggtctggatctagtatggtatctgcactaatattgcatgct
tttcgtgtttatttaacaggaggatttaaaaaagctcgtgagctaatttggatgacaggt
ataattttaggagtttgcaccgtgttgtttggagtaacaggatattgattagcttgggat
caagtagcttattgggcatgtaaaatagtgacaggagtacctgaaggactagacaagttg

ttatttggtgttggcttgcttttggttctaataattagaggtggctttagtgttagttct
ggtaggctcacaagattctatagcattcacacattccttctcccaattgtaacattaagt
ttagttatcattcactttattcaaatcagaaaacaaggtatatctggacctctttaa
>KrE2_petB
atgtggctttatgattggtctgaagaaggtttagaaattcaatgtattggagatgatatt
ctaggtaaattagtaccacctcatgttaatatattttactgctttggaggtgtggtatcc
cttttgctcttatttcaaataatcagtggtcttggattgacaatgtattacagtccaagt
gttgtctctgcattttcatcggtacttaatatagttggccaagtatatttaggttggtta
aatcgttctattcataggtggtctggatctagtatggtatctgcactaatattgcatgct
tttcgtgtttatttaacaggaggatttaaaaaagctcgtgagctaatttggatgacaggt
ataattttaggagtttgcaccgtgttgtttggagtaacaggatattgattagcttgggat
caagtagcttattgggcatgtaaaatagtgacaggagtacctgaaggactagacaagttg
ttatttggtgttggcttgcttttggttctaataattagaggtggctttagtgttagttct
ggtaggctcacaagattctatagcattcacacatttcttctcccaattgtaacattgagt
ttagttatcattcactttattcaaatcagaaaacaaggtatatctggacctctttaa
>KrE3_petB
atgtggctttatgattggtctgaagaaggtttagaaattcaatgtattggagatgatatt
ctaggtaaattagtaccacctcatgttaatatattttactgctttggaggtgtggtatcc
cttttgctcttatttcaaataatcagtggtcttggattgacaatgtattacactccaagt
gttgtctctgcattttcatcggtacttaatatagttggccaagtacatttaggttggtta
aatcgttctattcacagatggtctggatctagtatggtatctgcactaatattgcatgct
tttcgtgtttatttaacaggaggatttaaaaaagctcgtgagctaatttggatgacaggt
ataattttaggagtttgcaccgtgttgtttggagtaacaggatattgattagcttgggat
caagtagcttattgggcatgtaaaatagtgacaggagtacctgaaggactagacaagttg
ttatttggtgttggcttgcttttggttctaataattagaggtggctttagtgttagttct
ggtaggctcacaagattctatagcattcacacatttcttctcccaattgtaacattgagt
ttagttatcattcactttattcaaatcagaaaacaaggtatatctggacctctttaa
>KrE4_petB
atgtggctttatgattggtctgaagaaggtttagaaattcaatgtattggagatgatatt
ctaggtaaattagtaccacctcatgttaatatattttactgctttggaggtgtggtatcc
cttttgctcttatttcaaataatcagtggtcttggattgacaatgtattacagtccaagt
gttgtctctgcattttcatcggtacttaatatagttggccaagtacatttaggttggtta
aatcgttctattcataggtggtctggatctagtatggtatctgcactaatattgcatgct
tttcgtgtttatttaacaggaggatttaaaaaagctcgtgagctaatttggatgacaggt
ataattctaggagtttgcaccgtgttgtttggagtaacaggatattgattagcttgggat
caagtaggttattgggcatgtaaaatagtgacaggagtacctgaaggactagacaagttg
ttattcggtgttggctttcttttagttctaataattagaggtggctttagtgttagttct
ggtaggctcacaagattctatagcattcacacatttcttctcccaattgtaacattgagt
ttagttatcattcactttattcaaatcagaaaacaaggtatatctggacctctttaa
>KrE5_petB
atgtggctttatgattggtctgaagaaggtttagaaattcaatgtattggagatgatatt
ctaggtaaattagtaccacctcatgttaatatattttactgctttggaggtgtggtatcc
cttttgctcttatttcaaataatcagtggtcttggattgacaatgtattacagtccaagt
gttgtctctgcattttcatcggtacttaatatagttggccaagtacatttaggttggtta
aatcgttctattcacagatggtctggatctagtatggtatctgcactaatattgcatgct
tttcgtgtttatttaacaggaggatttaaaaaagctcgtgagctaatttggatgacaggt
ataattttaggagtttgcaccgtgttgtttggagtaacaggatattgattagcttgggat
caagtagcttattgggcatgtaaaatagtgacaggagtacctgaaggactagacaagttg
ttatttggtgttggcttgcttttggttctaataattagaggtggctttagtgttagttct
ggtaggctcacaagattctatagcattcacacatttcttctcccaattgtaacattgagt
ttagttatcattcactttattcaaatcagaaaacaaggtatatctggacctctttaa
>KrE6_petB
atgtggctttatgattggtctgaagaaggtttagaaattcaatgtattggagatgatatt
ctaggtaaattagtaccacctcatgttaatatattttactgctttggaggtgtggtatcc
cttttgctcttatttcaaataatcagtggtcttggattgacaatgtattacagtccaagt
gttgtctctgcattttcatcggtacttaatatagttggccaagtacatttaggttggtta
aatcgttctattcatagatggtctggatctagtatggtatctgcactaatattgcatgct

tttcgtgtttatttaacaggaggatttaaaaaagctcgtgagctaatttggatgacaggt
ataattttaggagtttgcaccgtgttgtttggagtaacaggatattgattagcttgggat
caagtaggttattgggcatgtaaaatagtgacaggagtacctgaaggactagacaagttg
ttatttggtgttggcttgcttttggttctaataattagaggtggctttagtgttagttct
ggtagtctcacaagattctatagcattcacacattccttctcccaattgtaacattgagt
ttagttatcattcactttattcaaatcagaaaacaaggtatatctggacctctttaa
>KrE7_petB
atgtggctttatgattggtctgaagaaggtttagaaattcaatgtattggagatgatatt
ctaggtaaattagtaccacctcatgttaatatattttactgctttggaggtgtggtatcc
cttttgctcttatttcaaataatcagtggtcttggattgacaatgtattacactccaagt
gttgtctctgcattttcatcggtacttaatatagttggccaagtatatttaggttggtta
aatcgttctattcataggtggtctggatctagtatggtatctgcactaatattgcatgct
tttcgtgtttatttaacaggaggatttaaaaaagctcgtgagctaatttggatgacaggt
ataattctaggagtttgcaccgtgttgtttggagtaacaggatattgattagcttgggat
caagtagcttattgggcatgtaaaatagtgacaggagtacctgaaggactagacaagttg
ttatttggtgttggcttgcttttagttctaataattagaggtggctttagtgttagttct
ggtaggctcacaagattctatagcattcacacatttcttctcccaattgtaacattgagt
ttagttatcattcactttattcaaatcagaaaacaaggtatatctggacctctttaa
>KrE8_petB
atgtggctttatgattggtctgaagaaggtttagaaattcaatgtattggagatgatatt
ctaggtaaattagtaccacctcatgttaatatattttactgctttggaggtgtggtatcc
cttttgctcttatttcaaataatcagtggtcttggattgacaatgtattacagtccaagt
gttgtctctgcattttcatcggtacttaatatagttggccaagtacatttaggttggtta
aatcgttctattcacagatggtctggatctagtatggtatctgcactaatattgcatgct
tttcgtgtttatttaacaggaggatttaaaaaagctcgtgagctaatttggatgacaggt
ataattttaggagtttgcaccgtgttgtttggagtaacaggatattgattagcttgggat
caagtagcttattgggcatgtaaaatagtgacaggagtacctgaaggactagacaagttg
ttatttggtgttggcttgcttttggttctaataattagaggtggctttagtgttagttct
ggtaggctaacaagattctatagcattcacacatttcttctcccaattgtaacattgagt
ttagttatcattcactttattcaaatcagaaaacaaggtatatctggacctctttaa
>KrE9_petB
atgtggctttatgattggtctgaagaaggtttagaaattcaatgtattggagatgatatt
ctaggtaaattagtaccacctcatgttaatatattttactgctttggaggtgtggtatcc
cttttgctcttatttcaaataatcagtggtcttggattgacaatgtattacagtccaagt
gttgtctctgcattttcatcggtacttaatatagttggccaagtacatttaggttggtta
aatcgttctattcacagatggtctggatctagtatggtatctgcactaatattgcatgct
tttcgtgtttatttaacaggaggatttaaaaaagctcgtgagctaatttggatgacaggt
ataattttaggagtttgcaccgtgttgtttggagtaacaggatattgattagcttgggat
caagtagcttattgggcatgtaaaatagtgacaggagtacctgaaggactagacaagttg
ttatttggtgttggcttgcttttggttctaataattagaggtggctttagtgttagttct
ggtaggctcacaagattctatagcattcacacatttcttctcccaattgtaacattgagt
ttagttatcattcactttattcaaatcagaaaacaaggtatatctggacctctttaa
>Mz10_petB
atgtggctttatgattggtctgaagaaggtttagaaattcaatgtattggagatgatatt
ctaggtaaattagtaccacctcatgttaatatattttactgctttggaggtgtggtatcc
cttttgctcttatttcaaataatcagtggtcttggattgacaatgtattacagtccaagt
gttgtctctgcattttcatcggtacttaatatagttggccaagtatatttaggttggtta
aatcgttctattcataggtggtctggatctagtatggtatctgcactaatattgcatgct
tttcgtgtttatttaacaggaggatttaaaaaagctcgtgagctaatttggatgacaggt
ataattctaggagtttgcaccgtgttgtttggagtaacaggatattgattagcttgggat
caagtagcttattgggcatgtaaaatagtgacaggagtacctgaaggactagacaagttg
ttatttggtgttggcttgcttttggttctaataattagaggtggctttagtgttagttct
ggtaggctcacaagattctatagcattcacacatttcttctccctattgtaacattgagt
ttagttatcattcactttattcaaatcagaaaacaaggtatatctggacctctttaa
>Mz5_petB
atgtggctttatgattggtctgaagaaggtttagaaattcaatgtattggagatgatatt
ctaggtaaattagtaccacctcatgttaatatattttactgctttggaggtgtggtatcc

cttttgctcttatttcaaataatcagtggtcttggattgacaatgtattacagtccaagt
gttgtctctgcattttcatcggtacttaatatagttggccaagtacatttaggttggtta
aatcgttctattcacagatggtctggatctagtatggtatctgcactaatattacatgct
tttcgtgtttatttaacaggaggatttaaaaaagctcgtgagctaatttggatgacaggt
ataattttaggagtttgcaccgtgttgtttggagtaacaggatattgattagcttgggat
caagtagcttattgggcatgtaaaatagtgacaggagtacctgaaggactagacaagttg
ttatttggtgttggcttgcttttggttctaataattagaggtggctttagtgttagttct
ggtaggctcacaagattctatagcattcacacatttcttctcccaattgtaacattgagt
ttagttatcattcactttattcaaatcagaaaacaaggtatatctggacctctttaa
>Mz6_petB
atgtggctttatgattggtctgaagaaggtttagaaattcaatgtattggagatgatatt
ctaggtaaattagtaccacctcatgttaatatattttactgctttggaggtgtggtatcc
cttttgctcttatttcaaataatcagtggtcttggattgacaatgtattacagtccaagt
gttgtctctgcattttcatcggtacttaatatagttggccaagtatatttaggttggtta
aatcgttctattcataggtggtctggatctagtatggtatctgcactaatattgcatgct
tttcgtgtttatttaacaggaggatttaaaaaagctcgtgagctaatttggatgacaggt
ataattttaggagtttgcaccgtgttgtttggagtaacaggatattgattagcttgggat
caagtagcttattgggcatgtaaaatagtgacaggagtacctgaaggactagacaagttg
ttatttggtgttggcttgcttttggttctaataattagaggtggctttagtgttagttct
ggtaggctcacaagattctatagcattcacacatttcttctcccaattgtaacattaagt
ttagttatcattcactttattcaaatcagaaaacaaggtatatctggacctctttaa
>Mz7_petB
atgtggctttatgattggtctgaagaaggtttagaaattcaatgtattggagatgatatt
ctaggtaaattagtaccacctcatgttaatatattttactgctttggaggtgtggtatcc
cttttgctcttatttcaaataatcagtggtcttggattgacaatgtattacactccaagt
gttgtctctgcattttcatcggtacttaatatagttggccaagtatatttaggttggtta
aatcgttctattcacagatggtctggatctagtatggtatctgcactaatattgcatgct
tttcgtgtttatttaacaggaggatttaaaaaagctcgtgagctaatttggatgacaggt
ataattctaggagtttgcaccgtgttgtttggagtaacaggatattgattagcttgggat
caagtaggttattgggcatgtaaaatagtgacaggagtacctgaaggactagacaagttg
ttatttggtgttggcttgcttttggttctaataattagaggtggctttagtgttagttct
ggtaggctcacaagattctatagcattcacacatttcttctcccaattgtaacattgagt
ttagttatcattcactttattcaaatcagaaaacaaggtatatctggacctctttaa
>Mz8_petB
atgtggctttatgattggtctgaagaaggtttagaaattcaatgtattggagatgatatt
ctaggtaaattagtaccacctcatgttaatatattttactgctttggaggtgtggtatcc
cttttgctcttatttcaaataatcagtggtcttggattgacaatgtattacactccaagt
gttgtttctgcattttcatcggtacttaatatagttggacaagtatatttaggttggtta
aatcgttctattcataggtggtctggatctagtatggtatctgcactaatattgcatgct
tttcgtgtttatttaacaggaggatttaaaaaagctcgtgagctaatttggatgacaggt
ataattctaggagtttgcaccgtgttgtttggagtaacaggatattgattagcttgggat
caagtaggttattgggcatgtaaaatagtgacaggagtacctgaaggactagacaagttg
ttattcggtgttggctttcttttagttctaataattagaggtggctttagtgttagttct
ggtaggctcacaagattctatagcattcacacatttcttctcccaattgtaacattgagt
ttagttatcattcactttattcaaatcagaaaacaaggtatatctggacctctttaa
>Mz9_petB
atgtggctttatgattggtctgaagaaggtttagaaattcaatgtattggagatgatatt
ctaggtaaattagtaccacctcatgttaatatattttactgctttggaggtgtggtatcc
cttttgctcttatttcaaataatcagtggtcttggattgacaatgtattacactccaagt
gttgtctctgcattttcatcggtacttaatatagttggccaagtatatttaggttggtta
aatcgttctattcataggtggtctggatctagtatggtatctgcactaatattgcatgct
tttcgtgtttatttaacaggaggatttaaaaaagctcgtgagctaatttggatgacaggt
ataattttaggagtttgcaccgtgttgtttggagtaacaggatattgattagcttgggat
caagtagcttattgggcatgtaaaatagtgacaggagtacctgaaggactagacaagttg
ttattcggtgttggctttcttttagttctaataattagaggtggctttagtgttagttct
ggtaggctcacaagattctatagcattcacacattccttctcccaattgtaacattgagt
ttagttatcattcactttattcaaatcagaaaacaaggtatatctggacctctttaa

>MzC1_petB
atgtggctttatgattggtctgaagaaggtttagaaattcaatgtattggagatgatatt
ctaggtaaattagtaccacctcatgttaatatattttactgctttggaggtgtggtatcc
cttttgctcttatttcaaataatcagtggtcttggattgacaatgtattacagtccaagt
gttgtctctgcattttcatcggtacttaatatagttggccaagtacatttaggttggtta
aatcgttctattcacagatggtctggatctagtatggtatctgcactaatattgcatgct
tttcgtgtttatttaacaggaggatttaaaaaagctcgtgagctaatttggatgacaggt
ataattttaggagtttgcaccgtgttgtttggagtaacaggatattgattagcttgggat
caagtagcttattgggcatgtaaaatagtgacaggagtacctgaaggactagacaagttg
ttatttggtgttggcttgcttttggttctaataattagaggtggctttagtgttagttct
ggtaggctcacaagattctatagcattcacacatttcttctcccaattgtaacattaagt
ttagttatcattcactttattcaaatcagaaaacaaggtatatctggacctctttaa
>MzC1GaII_petB
atgtggctttatgattggtctgaagaaggtttagaaattcaatgtattggagatgatatt
ctaggtaaattagtaccacctcatgttaatatattttactgctttggaggtgtggtatcc
cttttgctcttatttcaaataatcagtggtcttggattgacaatgtattacagtccaagt
gttgtctctgcattttcatcggtacttaatatagttggccaagtacatttaggttggtta
aatcgttctattcacagatggtctggatctagtatggtatctgcactaatattgcatgct
tttcgtgtttatttaacaggaggatttaaaaaagctcgtgagctaatttggatgacaggt
ataattttaggagtttgcaccgtgttgtttggagtaacaggatattgattagcttgggat
caagtagcttattgggcatgtaaaatagtgacaggagtacctgaaggactagacaagttg
ttatttggtgttggcttgcttttggttctaataattagaggtggctttagtgttagttct
ggtaggctcacaagattctatagcattcacacatttcttctcccaattgtaacattgagt
ttagttatcattcactttattcaaatcagaaaacaaggtatatctggacctctttaa
>MzC2_petB
atgtggctttatgattggtctgaagaaggtttagaaattcaatgtattggagatgatatt
ctaggtaaattagtaccacctcatgttaatatattttactgctttggaggtgtggtatcc
cttttgctcttatttcaaataatcagtggtcttggattgacaatgtattacagtccaagt
gttgtctctgcattttcatcggtacttaatatagttggccaagtacatttaggttggtta
aatcgttctattcacagatggtctggatctagtatggtatctgcactaatattgcatgct
tttcgtgtttatttaacaggaggatttaaaaaagctcgtgagctaatttggatgacaggt
ataattttaggagtttgcaccgtgttgtttggagtaacaggatattgattagcttgggat
caagtagcttattgggcatgtaaaatagtgacaggagtacctgaaggactagacaagttg
ttatttggtgttggcttgcttttggttctaataattagaggtggctttagtgttagttct
ggtaggctcacaagattctatagcattcacacatttcttctcccaattgtaacattgagt
ttagttatcattcactttattcaaatcagaaaacaaggtatatctggacctctttaa
>MzC2GaII_petB
atgtggctttatgattggtctgaagaaggtttagaaattcaatgtattggagatgatatt
ctaggtaaattagtaccacctcatgttaatatattttactgctttggaggtgtggtatcc
cttttgctcttatttcaaataatcagtggtcttggattgacaatgtattacagtccaagt
gttgtctctgcattttcatcggtacttaatatagttggccaagtacatttaggttggtta
aatcgttctattcacagatggtctggatctagtatggtatctgcactaatattgcatgct
tttcgtgtttatttaacaggaggatttaaaaaagctcgtgagctaatttggatgacaggt
ataattttaggagtttgcaccgtgttgtttggagtaacaggatattgattagcttgggat
caagtagcttattgggcatgtaaaatagtgacaggagtacctgaaggactagacaagttg
ttatttggtgttggcttgcttttggttctaataattagaggtggctttagtgttagttct
ggtaggctcacaagattctatagcattcacacatttcttctcccaattgtaacattgagt
ttagttatcattcactttattcaaatcagaaaacaaggtatatctggacctctttaa
>MzC3_petB
atgtggctttatgattggtctgaagaaggtttagaaattcaatgtattggagatgatatt
ctaggtaaattagtaccacctcatgttaatatattttactgctttggaggtgtggtatcc
cttttgctcttatttcaaataatcagtggtcttggattgacaatgtattacactccaagt
gttgtctctgcattttcatcggtacttaatatagttggccaagtacatttaggttggtta
aatcgttctattcacagatggtctggatctagtatggtatctgcactaatattgcatgct
tttcgtgtttatttaacaggaggatttaaaaaagctcgtgagctaatttggatgacaggt
ataattttaggagtttgcaccgtgttgtttggagtaacaggatattgattagcttgggat
caagtagcttattgggcatgtaaaatagtgacaggagtacctgaaggactagacaagttg

ttatttggtgttggcttgcttttggttctaataattagaggtggctttagtgttagttct
ggtaggctcacaagattctatagcattcacacatttcttctcccaattgtaacattgagt
ttagttatcattcactttattcaaatcagaaaacaaggtatatctggacctctttaa
>MzC4_petB
atgtggctttatgattggtctgaagaaggtttagaaattcaatgtattggagatgatatt
ctaggtaaattagtaccacctcatgttaatatattttactgctttggaggtgtggtatcc
cttttgctcttatttcaaataatcagtggtcttggattgacaatgtattacagtccaagt
gttgtctctgcattttcatcggtacttaatatagttggccaagtacatttaggttggtta
aatcgttctattcacagatggtctggatctagtatggtatctgcactaatattgcatgct
tttcgtgtttatttaacaggaggatttaaaaaagctcgtgagctaatttggatgacaggt
ataattttaggagtttgcaccgtgttgtttggagtaacaggatattgattagcttgggat
caagtaggttattgggcatgtaaaatagtgacaggagtacctgaaggactagacaagttg
ttattcggtgttggctttcttttggttctaataattagaggtggctttagtgttagttct
ggtaggctcacaagattctatagcattcacacatttcttctcccaattgtaacattgagt
ttagttatcattcactttattcaaatcagaaaacaaggtatatctggacctctttaa
>Od10_petB
atgtggctttatgattggtctgaagaaggtttagaaattcaatgtattggagatgatatt
ctaggtaaattagtaccacctcatgttaatatattttactgctttggaggtgtggtatcc
cttttgctcttatttcaaataatcagtggtcttggattgacaatgtattacagtccaagt
gttgtctctgcattttcatcggtacttaatatagttggccaagtacatttaggttggtta
aatcgttctattcacagatggtctggatctagtatggtatctgcactaatattgcatgct
tttcgtgtttatttaacaggaggatttaaaaaagctcgtgagctaatttggatgacaggt
ataattttaggagtttgcaccgtgttgtttggagtaacaggatattgattagcttgggat
caagtagcttattgggcatgtaaaatagtgacaggagtacctgaaggactagacaagttg
ttatttggtgttggcttgcttttggttctaataattagaggtggctttagtgttagttct
ggtaggctcacaagattctatagcattcacacatttcttctcccaattgtaacattgagt
ttagttatcattcactttattcaaatcagaaaacaaggtatatctggacctctttaa
>Od11_petB
atgtggctttatgattggtctgaagaaggtttagaaattcaatgtattggagatgatatt
ctaggtaaattagtaccacctcatgttaatatattttactgctttggaggtgtggtatcc
cttttgctcttatttcaaataatcagtggtcttggattgacaatgtattacagtccaagt
gttgtctctgcattttcatcggtacttaatatagttggccaagtacatttaggttggtta
aatcgttctattcacagatggtctggatctagtatggtatctgcactaatattgcatgct
tttcgtgtttatttaacaggaggatttaaaaaagctcgtgagctaatttggatgacaggt
ataattttaggagtttgcaccgtgttgtttggagtaacaggatattgattagcttgggat
caagtagcttattgggcatgtaaaatagtgacaggagtacctgaaggactagacaagttg
ttatttggtgttggcttgcttttggttctaataattagaggtggctttagtgttagttct
ggtaggctcacaagattctatagcattcacacatttcttctcccaattgtaacattgagt
ttagttatcattcactttattcaaatcagaaaacaaggtatatctggacctctttaa
>Od12_petB
atgtggctttatgattggtctgaagaaggtttagaaattcaatgtattggagatgatatt
ctaggtaaattagtaccacctcatgttaatatattttactgctttggaggtgtggtatcc
cttttgctcttatttcaaataatcagtggtcttggattgacaatgtattacagtccaagt
gttgtctctgcattttcatcggtacttaatatagttggccaagtacatttaggttggtta
aatcgttctattcacagatggtctggatctagtatggtatctgcactaatattgcatgct
tttcgtgtttatttaacaggaggatttaaaaaagctcgtgagctaatttggatgacaggt
ataattttaggagtttgcaccgtgttgtttggagtaacaggatattgattagcttgggat
caagtagcttattgggcatgtaaaatagtgacaggagtacctgaaggactagacaagttg
ttatttggtgttggcttgcttttggttctaataattagaggtggctttagtgttagttct
ggtaggctcacaagattctatagcattcacacatttcttctcccaattgtaacattgagt
ttagttatcattcactttattcaaatcagaaaacaaggtatatctggacctctttaa
>Od13_petB
atgtggctttatgattggtctgaagaaggtttagaaattcaatgtattggagatgatatt
ctaggtaaattagtaccacctcatgttaatatattttactgctttggaggtgtggtatcc
cttttgctcttatttcaaataatcagtggtcttggattgacaatgtattacagtccaagt
gttgtctctgcattttcatcggtacttaatatagttggccaagtacatttaggttggtta
aatcgttctattcacagatggtctggatctagtatggtatctgcactaatattgcatgct

tttcgtgtttatttaacaggaggatttaaaaaagctcgtgagctaatttggatgacaggt
ataattttaggagtttgcaccgtgttgtttggagtaacaggatattgattagcttgggat
caagtagcttattgggcatgtaaaatagtgacaggagtacctgaaggactagacaagttg
ttatttggtgttggcttgcttttggttctaataattagaggtggctttagtgttagttct
ggtaggctcacaagattctatagcattcacacatttcttctcccaattgtaacattgagt
ttagttatcattcactttattcaaatcagaaaacaaggtatatctggacctctttaa
>Od14_petB
atgtggctttatgattggtctgaagaaggtttagaaattcaatgtattggagatgatatt
ctaggtaaattagtaccacctcatgttaatatattttactgctttggaggtgtggtatcc
cttttgctcttatttcaaataatcagtggtcttggattgacaatgtattacagtccaagt
gttgtctctgcattttcatcggtacttaatatagttggccaagtacatttaggttggtta
aatcgttctattcataggtggtctggatctagtatggtatctgcactaatattgcatgct
tttcgtgtttatttaacaggaggatttaaaaaagctcgtgagctaatttggatgacaggt
ataattttaggagtttgcaccgtgttgtttggagtaacaggatattgattagcttgggat
caagtagcttattgggcatgtaaaatagtgacaggagtacctgaaggactagacaagttg
ttatttggtgttggcttgcttttggttctaataattagaggtggctttagtgttagttct
ggtaggctcacaagattctatagcattcacacatttcttctcccaattgtaacattgagt
ttagttatcattcactttattcaaatcagaaaacaaggtatatctggacctctttaa
>Od15_petB
atgtggctttatgattggtctgaagaaggtttagaaattcaatgtattggagatgatatt
ctaggtaaattagtaccacctcatgttaatatattttactgctttggaggtgtggtatcc
cttttgctcttatttcaaataatcagtggtcttggattgacaatgtattacagtccaagt
gttgtctctgcattttcatcggtacttaatatagttggccaagtacatttaggttggtta
aatcgttctattcacagatggtctggatctagtatggtatctgcactaatattgcatgct
tttcgtgtttatttaacaggaggatttaaaaaagctcgtgagctaatttggatgacaggt
ataattttaggagtttgcaccgtgttgtttggagtaacaggatattgattagcttgggat
caagtagcttattgggcatgtaaaatagtgacaggagtacctgaaggactagacaagttg
ttatttggtgttggcttgcttttggttctaataattagaggtggctttagtgttagttct
ggtaggctcacaagattctatagcattcacacatttcttctcccaattgtaacattgagt
ttagttatcattcactttattcaaatcagaaaacaaggtatatctggacctctttaa
>Od18_petB
atgtggctttatgattggtctgaagaaggtttagaaattcaatgtattggagatgatatt
ctaggtaaattagtaccacctcatgttaatatattttactgctttggaggtgtggtatcc
cttttgctcttatttcaaataatcagtggtcttggattgacaatgtattacagtccaagt
gttgtctctgcattttcatcggtacttaatatagttggccaagtacatttaggttggtta
aatcgttctattcacagatggtctggatctagtatggtatctgcactaatattgcatgct
tttcgtgtttatttaacaggaggatttaaaaaagctcgtgagctaatttggatgacaggt
ataattttaggagtttgcaccgtgttgtttggagtaacaggatattgattagcttgggat
caagtagcttattgggcatgtaaaatagtgacaggagtacctgaaggactagacaagttg
ttatttggtgttggcttgcttttggttctaataattagaggtggctttagtgttagttct
ggtaggctcacaagattctatagcattcacacatttcttctcccaattgtaacattgagt
ttagttatcattcactttattcaaatcagaaaacaaggtatatctggacctctttaa
>Od19_petB
atgtggctttatgattggtctgaagaaggtttagaaattcaatgtattggagatgatatt
ctaggtaaattagtaccacctcatgttaatatattttactgctttggaggtgtggtatcc
cttttgctcttatttcaaataatcagtggtcttggattgacaatgtattacagtccaagt
gttgtctctgcattttcatcggtacttaatatagttggccaagtacatttaggttggtta
aatcgttctattcacagatggtctggatctagtatggtatctgcactaatattgcatgct
tttcgtgtttatttaacaggaggatttaaaaaagctcgtgagctaatttggatgacaggt
ataattttaggagtttgcaccgtgttgtttggagtaacaggatattgattagcttgggat
caagtagcttattgggcatgtaaaatagtgacaggagtacctgaaggactagacaagttg
ttatttggtgttggcttgcttttggttctaataattagaggtggctttagtgttagttct
ggtaggctcacaagattctatagcattcacacatttcttctcccaattgtaacattgagt
ttagttatcattcactttattcaaatcagaaaacaaggtatatctggacctctataa
>Od2_petB
atgtggctttatgattggtctgaagaaggtttagaaattcaatgtattggagatgatatt
ctaggtaaattagtaccacctcatgttaatatattttactgctttggaggtgtggtatcc

cttttgctcttatttcaaataatcagtggtcttggattgacaatgtattacagtccaagt
gttgtctctgcattttcatcggtacttaatatagttggccaagtacatttaggttggtta
aatcgttctattcacagatggtctggatctagtatggtatctgcactaatattgcatgct
tttcgtgtttatttaacaggaggatttaaaaaagctcgtgagctaatttggatgacaggt
ataattttaggagtttgcaccgtgttgtttggagtaacaggatattgattagcttgggat
caagtagcttattgggcatgtaaaatagtgacaggagtacctgaaggactagacaagttg
ttatttggtgttggcttgcttttggttctaataattagaggtggctttagtgttagttct
ggtaggctcacaagattctatagcattcacacatttcttctcccaattgtaacattgagt
ttagttatcattcactttattcaaatcagaaaacaaggtatatctggacctctttaa
>Od22_petB
atgtggctttatgattggtctgaagaaggtttagaaattcaatgtattggagatgatatt
ctaggtaaattagtaccacctcatgttaatatattttactgctttggaggtgtggtatcc
cttttgctcttatttcaaataatcagtggtcttggattgacaatgtattacagtccaagt
gttgtctctgcattttcatcggtacttaatatagttggccaagtacatttaggttggtta
aatcgttctattcataggtggtctggatctagtatggtatctgcactaatattgcatgct
tttcgtgtttatttaacaggaggatttaaaaaagctcgtgagctaatttggatgacaggt
ataattttaggagtttgcaccgtgttgtttggagtaacaggatattgattagcttgggat
caagtagcttattgggcatgtaaaatagtgacaggagtacctgaaggactagacaagttg
ttatttggtgttggcttgcttttggttctaataattagaggtggctttagtgttagttct
ggtaggctcacaagattctatagcattcacacatttcttctcccaattgtaacattgagt
ttagttatcattcactttattcaaatcagaaaacaaggtatatctggacctctttaa
>Od23_petB
atgtggctttatgattggtctgaagaaggtttagaaattcaatgtattggagatgatatt
ctaggtaaattagtaccacctcatgttaatatattttactgctttggaggtgtggtatcc
cttttgctcttatttcaaataatcagtggtcttggattgacaatgtattacagtccaagt
gttgtctctgcattttcatcggtacttaatatagttggccaagtatatttaggttggtta
aatcgttctattcataggtggtctggatctagtatggtatctgcactaatattgcatgct
tttcgtgtttatttaacaggaggatttaaaaaagctcgtgagctaatttggatgacaggt
ataattttaggagtttgcaccgtgttgtttggagtaacaggatattgattagcttgggat
caagtagcttattgggcatgtaaaatagtgacaggagtacctgaaggactagacaagttg
ttatttggtgttggcttgcttttggttctaataattagaggtggctttagtgttagttct
ggtaggctcacaagattctatagcattcacacatttcttctcccaattgtaacattgagt
ttagttatcattcactttattcaaatcagaaaacaaggtatatctggacctctttaa
>Od24_petB
atgtggctttatgattggtctgaagaaggtttagaaattcaatgtattggagatgatatt
ctaggtaaattagtaccacctcatgttaatatattttactgctttggaggtgtggtatcc
cttttgctcttatttcaaataatcagtggtcttggattgacaatgtattacagtccaagt
gttgtctctgcattttcatcggtacttaatatagttggccaagtacatttaggttggtta
aatcgttctattcacagatggtctggatctagtatggtatctgcactaatattgcatgct
tttcgtgtttatttaacaggaggatttaaaaaagctcgtgagctaatttggatgacaggt
ataattctaggagtttgcaccgtgttgtttggagtaacaggatattgattagcttgggat
caagtagcttattgggcatgtaaaatagtgacaggagtacctgaaggactagacaagttg
ttattcggtgttggctttcttttagttctaataattagaggtggctttagtgttagttct
ggtaggctcacaagattctatagcattcacacatttcttctcccaattgtaacattgagt
ttagttatcattcactttattcaaatcagaaaacaaggtatatctggacctctttaa
>Od25_petB
atgtggctttatgattggtctgaagaaggtttagaaattcaatgtattggagatgatatt
ctaggtaaattagtaccacctcatgttaatatattttactgctttggaggtgtggtatcc
cttttgctcttatttcaaataatcagtggtcttggattgacaatgtattacagtccaagt
gttgtctctgcattttcatcggtacttaatatagttggccaagtacatttaggttggtta
aatcgttctattcacagatggtctggatctagtatggtatctgcactaatattgcatgct
tttcgtgtttatttaacaggaggatttaaaaaagctcgtgagctaatttggatgacaggt
ataattttaggagtttgcaccgtgttgtttggagtaacaggatattgattagcttgggat
caagtagcttattgggcatgtaaaatagtgacaggagtacctgaaggactagacaagttg
ttatttggtgttggcttgcttttggttctaataattagaggtggctttagtgttagttct
ggtaggctcacaagattctatagcattcacacatttcttctcccaattgtaacattgagt
ttagttatcattcactttattcaaatcagaaaacaaggtatatctggacctctttaa

>Od26_petB
atgtggctttatgattggtctgaagaaggtttagaaattcaatgtattggagatgatatt
ctaggtaaattagtaccacctcatgttaatatattttactgctttggaggtgtggtatcc
cttttgctcttatttcaaataatcagtggtcttggattgacaatgtattacagtccaagt
gttgtctctgcattttcatcggtacttaatatagttggccaagtacatttaggttggtta
aatcgttctattcacagatggtctggatctagtatggtatctgcactaatattgcatgct
tttcgtgtttatttaacaggaggatttaaaaaagctcgtgagctaatttggatgacaggt
ataattctaggagtttgcaccgtgttgtttggagtaacaggatattgattagcttgggat
caagtagcttattgggcatgtaaaatagtgacaggagtacctgaaggactagacaagttg
ttatttggtgttggcttgcttttggttctaataattagaggtggctttagtgttagttct
ggtaggctcacaagattctatagcattcacacatttcttctcccaattgtaacattgagt
ttagttatcattcactttattcaaatcagaaaacaaggtatatctggacctctttaa
>Od27_petB
atgtggctttatgattggtctgaagaaggtttagaaattcaatgtattggagatgatatt
ctaggtaaattagtaccacctcatgttaatatattttactgctttggaggtgtggtatcc
cttttgctcttatttcaaataatcagtggtcttggattgacaatgtattacagtccaagt
gttgtctctgcattttcatcggtacttaatatagttggccaagtacatttaggttggtta
aatcgttctattcacagatggtctggatctagtatggtatctgcactaatattgcatgct
tttcgtgtttatttaacaggaggatttaaaaaagctcgtgagctaatttggatgacaggt
ataattttaggagtttgcaccgtgttgtttggagtaacaggatattgattagcttgggat
caagtagcttattgggcatgtaaaatagtgacaggagtacctgaaggactagacaagttg
ttatttggtgttggcttgcttttggttctaataattagaggtggctttagtgttagttct
ggtaggctcacaagattctatagcattcacacatttcttctcccaattgtaacattgagt
ttagttatcattcactttattcaaatcagaaaacaaggtatatctggacctctttaa
>Od4_petB
atgtggctttatgattggtctgaagaaggtttagaaattcaatgtattggagatgatatt
ctaggtaaattagtaccacctcatgttaatatattttactgctttggaggtgtggtatcc
cttttgctcttatttcaaataatcagtggtcttggattgacaatgtattacagtccaagt
gttgtctctgcattttcatcggtacttaatatagttggccaagtacatttaggttggtta
aatcgttctattcacagatggtctggatctagtatggtatctgcactaatattgcatgct
tttcgtgtttatttaacaggaggatttaaaaaagctcgtgagctaatttggatgacaggt
ataattttaggagtttgcaccgtgttgtttggagtaacaggatattgattagcttgggat
caagtagcttattgggcatgtaaaatagtgacaggagtacctgaaggactagacaagttg
ttatttggtgttggcttgcttttggttctaataattagaggtggctttagtgttagttct
ggtaggctcacaagattctatagcattcacacatttcttctcccaattgtaacattgagt
ttagttatcattcactttattcaaatcagaaaacaaggtatatctggacctctttaa
>Od6_petB
atgtggctttatgattggtctgaagaaggtttagaaattcaatgtattggagatgatatt
ctaggtaaattagtaccacctcatgttaatatattttactgctttggaggtgtggtatcc
cttttgctcttatttcaaataatcagtggtcttggattgacaatgtattacagtccaagt
gttgtctctgcattttcatcggtacttaatatagttggccaagtacatttaggttggtta
aatcgttctattcacagatggtctggatctagtatggtatctgcactaatattgcatgct
tttcgtgtttatttaacaggaggatttaaaaaagctcgtgagctaatttggatgacaggt
ataattctaggagtttgcaccgtgttgtttggagtaacaggatattgattagcttgggat
caagtagcttattgggcatgtaaaatagtgacaggagtacctgaaggactagacaagttg
ttatttggtgttggcttgcttttggttctaataattagaggtggctttagtgttagttct
ggtaggctcacaagattctatagcattcacacatttcttctcccaattgtaacattgagt
ttagttatcattcactttattcaaatcagaaaacaaggtatatctggacctctttaa
>Od8_petB
atgtggctttatgattggtctgaagaaggtttagaaattcaatgtattggagatgatatt
ctaggtaaattagtaccacctcatgttaatatattttactgctttggaggtgtggtatcc
cttttgctcttatttcaaataatcagtggtcttggattgacaatgtattacagtccaagt
gttgtctctgcattttcatcggtacttaatatagttggccaagtacatttaggttggtta
aatcgttctattcacagatggtctggatctagtatggtatctgcactaatattgcatgct
tttcgtgtttatttaacaggaggatttaaaaaagctcgtgagctaatttggatgacaggt
ataattttaggagtttgcaccgtgttgtttggagtaacaggatattgattagcttgggat
caagtagcttattgggcatgtaaaatagtgacaggagtacctgaaggactagacaagttg

ttatttggtgttggcttgcttttggttctaataattagaggtggctttagtgttagttct
ggtaggctcacaagattctatagcattcacacatttcttctcccaattgtaacattgagt
ttagttatcattcactttattcaaatcagaaaacaaggtatatctggacctctttaa
>Od9_petB
atgtggctttatgattggtctgaagaaggtttagaaattcaatgtattggagatgatatt
ctaggtaaattagtaccacctcatgttaatatattttactgctttggaggtgtggtatcc
cttttgctcttatttcaaataatcagtggtcttggattgacaatgtattacagtccaagt
gttgtctctgcattttcatcggtacttaatatagttggccaagtacatttaggttggtta
aatcgttctattcacagatggtctggatctagtatggtatctgcactaatattgcatgct
tttcgtgtttatttaacaggaggatttaaaaaagctcgtgagctaatttggatgacaggt
ataattttaggagtttgcaccgtgttgtttggagtaacaggatattgattagcttgggat
caagtagcttattgggcatgtaaaatagtgacaggagtacctgaaggactagacaagttg
ttatttggtgttggcttgcttttggttctaataattagaggtggctttagtgttagttct
ggtaggctcacaagattctatagcattcacacatttcttctcccaattgtaacattgagt
ttagttatcattcactttattcaaatcagaaaacaaggtatatctggacctctttaa
>Ss5_petB
atgtggctttatgattggtctgaagaaggtttagaaattcaatgtattggagatgatatt
ctaggtaaattagtaccacctcatgttaatatattttactgctttggaggtgtggtatcc
cttttgctcttatttcaaataatcagtggtcttggattgacaatgtattacagtccaagt
gttgtctctgcattttcatcggtacttaatatagttggccaagtacatttaggttggtta
aatcgttctattcacagatggtctggatctagtatggtatctgcactaatattgcatgct
tttcgtgtttatttaacaggaggatttaaaaaagctcgtgagctaatttggatgacaggt
ataattttaggagtttgcaccgtgttgtttggagtaacaggatattgattagcttgggat
caagtagcttattgggcatgtaaaatagtgacaggagtacctgaaggactagacaagttg
ttatttggtgttggcttgcttttggttctaataattagaggtggctttagtgttagttct
ggtaggctcacaagattctatagcattcacacatttcttctcccaattgtaacattgagt
ttagttatcattcactttattcaaatcagaaaacaaggtatatctggacctctttaa
>Ss6_petB
atgtggctttatgattggtctgaagaaggtttagaaattcaatgtattggagatgatatt
ctaggtaaattagtaccacctcatgttaatatattttactgctttggaggtgtggtatcc
cttttgctcttatttcaaataatcagtggtcttggattgacaatgtattacagtccaagt
gttgtctctgcattttcatcggtacttaatatagttggccaagtacatttaggttggtta
aatcgttctattcacaggtggtctggatctagtatggtatctgcactaatattgcatgct
tttcgtgtttatttaacaggaggatttaaaaaagctcgtgagctaatttggatgacaggt
ataattttaggagtttgcaccgtgttgtttggagtaacaggatattgattagcttgggat
caagtagcttattgggcatgtaaaatagtgacaggagtacctgaaggactagacaagttg
ttatttggtgttggcttgcttttggttctaataattagaggtggctttagtgttagttct
ggtaggctcacaagattctatagcattcacacatttcttctcccaattgtaacattgagt
ttagttatcattcactttattcaaatcagaaaacaaggtatatctggacctctttaa
>Ss7_petB
atgtggctttatgattggtctgaagaaggtttagaaattcaatgtattggagatgatatt
ctaggtaaattagtaccacctcatgttaatatattttactgctttggaggtgtggtatcc
cttttgctcttatttcaaataatcagtggtcttggattgacaatgtattacagtccaagt
gttgtctctgcattttcatcggtacttaatatagttggccaagtacatttaggttggtta
aatcgttctattcacagatggtctggatctagtatggtatctgcactaatattgcatgct
tttcgtgtttatttaacaggaggatttaaaaaagctcgtgagctaatttggatgacaggt
ataattttaggagtttgcaccgtgttgtttggagtaacaggatattgattagcttgggat
caagtagcttattgggcatgtaaaatagtgacaggagtacctgaaggactagacaagttg
ttatttggtgttggcttgcttttggttctaataattagaggtggctttagtgttagttct
ggtaggctcacaagattctatagcattcacacatttcttctcccaattgtaacattgagt
ttagttatcattcactttattcaaatcagaaaacaaggtatatctggacctctttaa
>Ss8_petB
atgtggctttatgattggtctgaagaaggtttagaaattcaatgtattggagatgatatt
ctaggtaaattagtaccacctcatgttaatatattttactgctttggaggtgtggtatcc
cttttgctcttatttcaaataatcagtggtcttggattgacaatgtattacagtccaagt
gttgtctctgcattttcatcggtacttaatatagttggccaagtacatttaggttggtta
aatcgttctattcacagatggtctggatctagtatggtatctgcactaatattgcatgct

tttcgtgtttatttaacaggaggatttaaaaaagctcgtgagctaatttggatgacaggt
ataattttaggagtttgcaccgtgttgtttggagtaacaggatattgattagcttgggat
caagtagcttattgggcatgtaaaatagtgacaggagtacctgaaggactagacaagttg
ttatttggtgttggcttgcttttggttctaataattagaggtggctttagtgttagttct
ggtaggctcacaagattctatagcattcacacatttcttctcccaattgtaacattgagt
ttagttatcattcactttattcaaatcagaaaacaaggtatatctggacctctttaa
>ohdo1_petB
atgtggctttatgattggtctgaagaaggtttagaaattcaatgtattggagatgatatt
ctaggtaaattagtaccacctcatgttaatatattttactgctttggaggtgtggtatcc
cttttgctcttatttcaaataatcagtggtcttggattgacaatgtattacagtccaagt
gttgtctctgcattttcatcggtacttaatatagttggccaagtacatttaggttggtta
aatcgttctattcacaggtggtctggatctagtatggtatctgcactaatattgcatgct
tttcgtgtttatttaacaggaggatttaaaaaagctcgtgagctaatttggatgacaggt
ataattttaggagtttgcaccgtgttgtttggagtaacaggatattgattagcttgggat
caagtagcttattgggcatgtaaaatagtgacaggagtacctgaaggactagacaagttg
ttatttggtgttggcttgcttttggttctaataattagaggtggctttagtgttagttct
ggtaggctcacaagattctatagcattcacacattccttctcccaattgtaacattgagt
ttagttatcattcactttattcaaatcagaaaacaaggtatatctggacctctttaa
>ohdo3_petB
atgtggctttatgattggtctgaagaaggtttagaaattcaatgtattggagatgatatt
ctaggtaaattagtaccacctcatgttaatatattttactgctttggaggtgtggtatcc
cttttgctcttatttcaaataatcagtggtcttggattgacaatgtattacactccaagt
gttgtctctgcattttcatcggtacttaatatagttggccaagtacatttaggttggtta
aatcgttctattcacagatggtctggatctagtatggtatctgcactaatattgcatgct
tttcgtgtttatttaacaggaggatttaaaaaagctcgtgagctaatttggatgacaggt
ataattttaggagtttgcaccgtgttgtttggagtaacaggatattgattagcttgggat
caagtagcttattgggaatgtaaaatagtgacaggagtacctgaaggactagacaagttg
ttatttggtgttggcttgcttttggttctaataattagaggtggctttagtgttagttct
ggtaggctcacaagattctatagcattcacacatttcttctcccaattgtaacattgagt
ttagttatcattcactttattcaaatcagaaaacaaggtatatctggacctctttaa
>ohdo5_petB
atgtggctttatgattggtctgaagaaggtttagaaattcaatgtattggagatgatatt
ctaggtaaattagtaccacctcatgttaatatattttactgctttggaggtgtggtatcc
cttttgctcttatttcaaataatcagtggtcttggattgacaatgtattacagtccaagt
gttgtctctgcattttcatcggtacttaatatagttggccaagtatatttaggttggtta
aatcgttctattcacagatggtctggatctagtatggtatctgcactaatattgcatgct
tttcgtgtttatttaacaggaggatttaaaaaagctcgtgagctaatttggatgacaggt
ataattttaggagtttgcaccgtgttgtttggagtaacaggatattgattagcttgggat
caagtagcttattgggcatgtaaaatagtgacaggagtacctgaaggactagacaagttg
ttatttggtgttggcttgcttttggttctaataattagaggtggctttagtgttagttct
ggtaggctcacaagattctatagcattcacacattccttctcccaattgtaacattgagt
ttagttatcattcactttattcaaatcagaaaacaaggtatatctggacctctttaa
>ohdo7_petB
atgtggctttatgattggtctgaagaaggtttagaaattcaatgtattggagatgatatt
ctaggtaaattagtaccacctcatgttaatatattttactgctttggaggtgtggtatcc
cttttgctcttatttcaaataatcagtggtcttggattgacaatgtattacagtccaagt
gttgtctctgcattttcatcggtacttaatatagttggccaagtacatttaggttggtta
aatcgttctattcacagatggtctggatctagtatggtatctgcactaatattgcatgct
tttcgtgtttatttaacaggaggatttaaaaaagctcgtgagctaatttggatgacaggt
ataattttaggagtttgcaccgtgttgtttggagtaacaggatattgattagcttgggat
caagtagcttattgggcatgtaaaatagtgacaggagtacctgaaggactagacaagttg
ttatttggtgttggcttgcttttggttctaataattagaggtggctttagtgttagttct
ggtaggctcacaagattctatagcattcacacatttcttctcccaattgtaacattgagt
ttagttatcattcactttattcaaatcagaaaacaaggtatatctggacctctttaa
>sesoko1_petB
atgtggctttatgattggtctgaagaaggtttagaaattcaatgtattggagatgatatt
ctaggtaaattagtaccacctcatgttaatatattttactgctttggaggtgtggtatcc

cttttgctcttatttcaaataatcagtggtcttggattgacaatgtattacagtccaagt
gttgtctctgcattttcatcggtacttaatatagttggccaagtacatttaggttggtta
aatcgttctattcacagatggtctggatctagtatggtatctgcactaatattgcatgct
tttcgtgtttatttaacaggaggatttaaaaaagctcgtgagctaatttggatgacaggt
ataattttaggagtttgcaccgtgttgtttggagtaacaggatattgattagcttgggat
caagtagcttattgggcatgtaaaatagtgacaggagtacctgaaggactagacaagttg
ttatttggtgttggcttgcttttggttctaataattagaggtggctttagtgttagttct
ggtaggctcacaagattctatagcattcacacatttcttctcccaattgtaacattgagt
ttagttatcattcactttattcaaatcagaaaacaaggtatatctggacctctttaa
>sesoko3_petB
atgtggctttatgattggtctgaagaaggtttagaaattcaatgtattggagatgatatt
ctaggtaaattagtaccacctcatgttaatatattttactgctttggaggtgtggtatcc
cttttgctcttatttcaaataatcagtggtcttggattgacaatgtattacagtccaagt
gttgtctctgcattttcatcggtacttaatatagttggccaagtacatttaggttggtta
aatcgttctattcacagatggtctggatctagtatggtatctgcactaatattgcatgct
tttcgtgtttatttaacaggaggatttaaaaaagctcgtgagctaatttggatgacaggt
ataattttaggagtttgcaccgtgttgtttggagtaacaggatattgattagcttgggat
caagtagcttattgggcatgtaaaatagtgacaggagtacctgaaggactagacaagttg
ttatttggtgttggcttgcttttggttctaataattagaggtggctttagtgttagttct
ggtaggctcacaagattctatagcattcacacatttcttctcccaattgtaacattgagt
ttagttatcattcactttattcaaatcagaaaacaaggtatatctggacctctttaa
>sesoko4_petB
atgtggctttatgattggtctgaagaaggtttagaaattcaatgtattggagatgatatt
ctaggtaaattagtaccacctcatgttaatatattttactgctttggaggtgtggtatcc
cttttgctcttatttcaaataatcagtggtcttggattgacaatgtattacagtccaagt
gttgtctctgcattttcatcggtacttaatatagttggccaagtacatttaggttggtta
aatcgttctattcacagatggtctggatctagtatggtatctgcactaatattgcatgct
tttcgtgtttatttaacaggaggatttaaaaaagctcgtgagctaatttggatgacaggt
ataattttaggagtttgcaccgtgttgtttggagtaacaggatattgattagcttgggat
caagtagcttattgggcatgtaaaatagtgacaggagtacctgaaggactagacaagttg
ttatttggtgttggcttgcttttggttctaataattagaggtggctttagtgttagttct
ggtaggctcacaagattctatagcattcacacatttcttctcccaattgtaacattgagt
ttagttatcattcactttattcaaatcagaaaacaaggtatatctggacctctttaa
>REF_DNA_petB_JX094310
ATGTGGCTTTATGATTGGTCTGAAGAAGGTTTAGAAATTCAATGTATTGGAGATGATATT
CTAGGTAAATTAGTACCACCTCATGTTAATATATTTTACTGCTTTGGAGGTGTGGTATCC
CTTTTGCTCTTATTTCAAATAATCAGTGGTCTTGGATTGACAATGTATTACACTCCAAGT
GTTGTCTCTGCATTTTCATCGGTACTTAATATAGTTGGCCAAGTATATTTAGGTTGGTTA
AATCGTTCTATTCATAGGTGGTCTGGATCTAGTATGGTATCTGCACTAATATTGCATGCT
TTTCGTGTTTATTTAACAGGAGGATTTAAAAAAGCTCGTGAGCTAATTTGGATGACAGGT
ATAATTCTAGGAGTTTGCACCGTGTTGTTTGGAGTAACAGGATATTGATTAGCTTGGGAT
CAAGTAGGTTATTGGGCATGTAAAATAGTGACAGGAGTACCTGAAGGACTAGACAAGTTG
TTATTCGGTGTTGGCTTTCTTTTAGTTCTAATAATTAGAGGTGGCTTTAGTGTTAGTTCT
GGTAGGCTCACAAGATTCTATAGCATTCACACATTCCTTCTCCCAATTGTAACATTAAGT
TTAGTTATCATTCACTTTATTCAAATCAGAAAACAAGGTATATCTGGACCTCTTTAA
>REF_RNA_petB_symbB1.comp54_c0_seq1
ATGTGGCTTTATGATTGGTTTGAAGAACGTTTAGAAATTCAATGTATTGCAGATGATATT
CTAGGTAAATTAGTACCACCTCATGTTAATATATTTTACTGCTTTGGAGGTGTGGTATCC
CTTTTGTTCTTATTTCAAGTAATCAGTGGTCTTGGATTGACAATGTATTACACTCCAAGT
GTTGTCTCTGCATTTTCATCGGTACTTAATATAGTTGGCCAAGTACATTTAGGTTGGTTA
AATCGTTCTATTCATAGGTGGTCTGGATCTAGTATGGTATCTGCACTAATATTGCATGCT
TTTCGTGTTTATTTAACAGGAGGATTTAAAAAACCTCGTGAGCTAATTTGGATGACAGGT
GTAATTCTAGGAGTTTGCACCGTGTCGTTTGGAGTAACAGGATATTCATTACCTTGGGAT
CAAGTAGGTTATTGGGCATGTAAAATAGTGACAGCAGTACCTGAAGCACTAGACGAGTTC
TTACCCGGTGTTGGCTCTCTTTTAGTTCTAACAATTAGAGGTGGCTTTAGTGTTGGTTCT
GGTACGCTCACAAGATTCTATAGCATTCACACATTCCTTCTCCCAGTTATAACATTAAGT
TTAGTTATCATTCACTTTATTCAAATCAGAAAACAAGGTATATCTGGACCTCTTTAA

>Hd1_petD
atatgtgtagtcaaggtggcaaatcttaaaagttgtatgttgttggctaagttatcttgt
gggatgggacataacagttatggtgaagctgcatgaccaaatgatattgtgtacatattt
gcagttctgatatatggcttaggagtgctgatatttggcttatctattgcttgtccttta
gaaataatgtcttgttcaaatgctttctctactccattagaaattgtacctgagtggtat
ctcctactgtgctttaacttattgcgtattgtcacttccaaaggaataggtgttaaggaa
atggtgatcttagtcccaattattatttgcttgtgcattatagagaatgttagtgtatat
tctaatccttttagaagagctataatggtatgtgtaactttagtgtatagtatttttgca
atctggttaagtgttggctcactagttcgaatagataaggctttacctcttttgtaa
>Hd2_petD
atatgtgtagtcaaggtggcaaatcttaaaagttgtatgttgttggctaagttatcttgt
gggatgggacataacagttatggtgaagctgcatgaccaaatgatattgtgtacatattt
gcagttctgatatatggcttaggagtgctgatatttggcttatctattgcttgtccttta
gaaataatgtcttgttcaaatgctttctctactccattagaaattgtacctgagtggtat
ctcctactgtgctttaacttattgcgtattgtcacttccaaaggaataggtgttaaggaa
atggtgatcttagtcccaattattatttgcttgtgcattatagagaatgttagtgtatat
tctaatccttttagaagagctataatggtatgtgtaactttagtgtatagtatttttgca
atctggttaagtgttggctcactagttcgaatagataaggctttacctcttttgtaa
>Hd3_petD
atatgtgtagtcaaggtggcaaatcttaaaagttgtatgttgttggctaagttatcttgt
gggatgggacataacagttatggtgaagctgcatgaccaaatgatattgtgtacatattt
gcagttctgatatatggcttaggagtgctgatacttggcttatctattgcttgtccttta
gaaataatgtcttgttcaaatgctttctctactccattagaaattgtacctgagtggtat
ctcctactgtgctttaacttattgcgtattgtcacttccaaaggaataggtgttaaggaa
atggtgatcttagtcccaattattgtttgcttgtgcattatagagaatgttagtgtatat
tctaatccttttagaagagctataatggtatgtgtaactttagtgtatagtatttttgca
atctggttaagtgttggctcactagttcgaatagataaggctttacctcttttgtaa
>Hd4_petD
atatgtgtagtcaaggtggcaaatcttaaaagttgtatgttgttggctaagttatcttgt
gggatgggacataacagttatggtgaagctgcatgaccaaatgatattgtgtacatattt
gcagttctgatatatggcttaggagtgctgatatttggcttatctattgcttgtccttta
gaaataatgtcttgttcaaatgctttctctactccattagaaattgtacctgagtggtat
ctcctactgtgctttaacttattgcgtattgtcacttccaaaggaataggtgttaaggaa
atggtgatcttagtcccaattattatttgcttgtgcattatagagaatgttagtgtatat
tctaatccttttagaagagctataatggtatgtgtaactttagtgtatagtatttttgca
atctggttaagtgttggctcactagttcgaatagataaggctttacctcttttgtaa
>Hd5_petD
atatgtgtagtcaaggtggcaaatcttaaaagttgtatgttgttggctaagttatcttgt
gggatgggacataacagttatggtgaagctgcatgaccaaatgatattgtgtacatattt
gcagttctgatatatggcttaggagtgctgatatttggcttatctattgcttgtccttta
gaaataatgtctttttcaaatgctttctctactccattagaaattgtacctgagtggtat
ctcctactgtgctttaacttattgcgtattgtcacttccaaaggaataggtgttaaggaa
atggtgatcttagtcccaattattatttgcttgtgcattatagagaatgttagtgtatat
tctaatccttttagaagagctataatggtatgtgtaactttagtgtatagtatttttgca
atctggttaagtgttggctcactagttcgaatagataaggctttacctcttttgtaa
>Hd6_petD
atatgtgtagtcaaggtggcaaatcttaaaagttgtatgttgttggctaagttatcttgt
gggatgggacataacagttatggtgaagctgcatgaccaaatgatattgtgtacatattt
gcagttctgatatatggcttaggagtgctgatatttggcttatctattgcttgtccttta
gaaataatgtcttgttcaaatgctttctctactccattagaaattgtacctgagtggtat
ctcctactgtgctttaacttattgcgtattgtcacttccaaaggaataggtgttaaggaa
atggtgatcttagtcccaattattatttgcttgtgcattatagagaatgttagtgtatat
tctaatccttttagaagagctataatggtatgtgtaactttagtgtatagtatttttgca
atctggttaagtgttggctcactagttcgaatagataaggctttacctcttttgtaa
>Hd7_petD
atatgtgtagtcaaggtggcaaatcttaaaagttgtatgttgttggctaagttatcttgt
gggatgggacataacagttatggtgaagctgcatgaccaaatgatattgtgtacatattt

gcagttctgatatatggcttaggagtgctgatacttggcttatctattgcttgtccttta
gaaataatgtcttgttcaaatgctttctctactccattagaaattgtacctgagtggtat
ctcctactgtgctttaacttattgcgtattgtcacttccaaaggaataggtgttaaggaa
atggtgatcttagtcccaattattatttgcttgtgcattatagagaatgttagtgtatat
tctaatccttttagaagagctataatggtatgtgtaactttagtgtatagtatttttgca
atctggttaagtgttggctcactagttcgaatagataaggctttacctcttttgtaa
>Hd8_petD
atatgtgtagtcaaggtggcaaatcttaaaagttgtatgttgttggctaagttatcttgt
gggatgggacataacagttatggtgaagctacatgaccaaatgatattgtgtacatattt
gcagttctgatatatggcttaggagtgctgatatttggcttatctattgcttgtccttta
gaaataatgtcttgttcaaatgctttctctactccattagaaattgtacctgagtggtat
ctcctactgtgctttaacttattgcgtattgtcacttccaaaggaataggtgttaaggaa
atggtgatcttagtcccaattattatttgcttgtgcattatagagaatgttagtgtatat
tctaatccttttagaagagctataatggtatgtgtaactttagtgtatagtatttttgca
atctggttaagtgttggctcactagttcgaatagataaggctttacctcttttgtaa
>Hd9_petD
atatgtgtagtcaaggtggcaaatcttaaaagttgtatgttgttggctaagttatcttgt
gggatgggacataacagttatggtgaagctgcatgaccaaatgatattgtgtacatattt
gcagttctgatatatggcttaggagtgctgatatttggcttatctattgcttgtccttta
gaaataatgtcttgttcaaatgctttctctactccattagaaattgtacctgagtggtat
ctcctactgtgctttaacttattgcgtattgtcacttccaaaggaataggtgttaaggaa
atggtgatcttagtcccaattattatttgcttgtgcattatagagaatgttagtgtatat
tctaatccttttagaagagctataatggtatgtgtaactttagtgtatagtatttttgca
atctggttaagtgttggctcactagttcgaatagataaggctttacctcttttgtaa
>IS1_petD
atatgtgtagtcaaggtggcaaatcttaaaagttgtatgttgttggctaagttatcttgt
gggatgggacataacagttatggtgaagctgcatgaccaaatgatattgtgtacatattt
gcagttctgatatatggcttaggagtgctgatacttggcttatctattgcttgtccttta
gaaataatgtcttgttcaaatgctttctctactccattagaaattgtacctgagtggtat
ctcctactgtgctttaacttattgcgtattgtcacttccaaaggaataggtgttaaggaa
atggtgatcttagtcccaattattgtttgcttgtgcattatagagaatgttagtgtatat
tctaatccttttagaagagctataatggtatgtgtaactttagtgtatagtatttttgca
atctggttaagtgttggctcactagttcgaatagataaggctttacctcttttgtaa
>IS2_petD
atatgtgtagtcaaggtggcaaatcttaaaagttgtatgttgttggctaagttatcttgt
ggtatgggacataacagttatggtgaagctgcatgaccaaatgatattgtgtacatattt
gcagttctgatatatggcttaggagtgctgatatttggcttatctattgcttgtccttta
gaaataatgtcttgttcaaatgctttctctactccattagaaattgtacctgagtgttat
ctcctactgtgctttaacttattgcgtattgtcacttccaaaggaataggtgttaaggaa
atggtgatcttagtcccaattattatttgcttgtgcattatagagaatgttagtgtatat
tctaatccttttagaagagctataatggtatgtgtaactttagtgtatagtatttttgca
atctggttaagtgttggctcactagttcgaatagataaggctttacctcttttgtaa
>IS3_petD
atatgtgtagtcaaggtggcaaatcttaaaagtcgtatgttgttggctaagttatcttgt
ggtatggggcataacagttatggtgaagctgcatgacccaatgatattgtgtacatattt
gcagttctgatatatggcttaggagtgctgatatttggcttatctattgcttgcccttta
gaaataatgtcttgttcaaatgctttctctaccccattagaaattgtacctgagtattat
ctcctactgtgctttaacttattgcgtattatcacttccaaaggaataggtgttatggaa
atggtgatcttagtcctaattattatttgcttgtgcattatagagaatgttagtgtatat
tctaatccttttagaagagctataatgctatgtgtaactttagtgtatagtatttttgca
atctggttaagtgttggttcactagttcgaatagataaggctttacctcttttgtaa
>IS4_petD
atatgtgtagtcaaggtggcaaatcttaaaagtcgtatgttgttggctaagttatcttgt
ggtatggggcataacagttatggtgaagctgcatgacccaatgatattgtgtacatattt
gcagttctgatatatggcttaggagtgctgatatttggcttatctattgcttgcccttta
gaaataatgtcttgttcaaatgctttctctaccccattagaaattgtacctgagtggtat
ctcctactgtgctttaacttattgcgtattatcacttccaaaggaataggtgttatggaa

atggtgatcttagtcctaattattatttcttgtgcattatagagaatgttagtgtatat
tctaatccttttagaagagctataatgctatgtgtaactttagtgtatagtatttttgca
atctggttaagtgttggttcactagttcgaatagataaggctttacctcttttgtaa
>IS5_petD
atatgtgtagtcaaggtggcaaatcttaaaagttgtatgttgttggctaagttatcttgt
ggtatgggacataacagttatggtgaagctgcatgaccaaatgatattgtgtacatattt
gcagttctgatatatggcttaggagtgctgatacttggcttatctattgcttgtccttta
gaaataatgtcttgttcaaatgctttctctactccattagaaattgtacctgagtggtat
ctcctactgtgctttaacttattgcgtattgtcacttccaaaggaataggtgttaaggaa
atggtgatcttagtcccaattattgtttgcttgtgcattatagagaatgttagtgtatat
tctaatccttttagaagagctataatggtatgtgtaactttagtgtatagtatttttgca
atctggttaagtgttggttcactagttcgaatagataaggctttacctcttttgtaa
>IS6_petD
atatgtgtagtcaaggtggcaaatcttaaaagttgtatgttgttggctaagttatcttgt
gggatgggacataacagttatggtgaagctgcatgaccaaatgatattgtgtacatattt
gcagttctgatatatggcttaggagtgctgatatttggcttatctattgcttgtccttta
gaaataatgtcttgttcaaatgctttctctactccattagaaattgtacctgagtggtat
ctcctactgtgctttaacttattgcgtattgtcacttccaaaggaataggtgttaaggaa
atggtgatcttagtcccaattattatttgcttgtgcattatagagaatgttagtgtatat
tctaatccttttagaagagctataatggtatgtgtaactttagtgtatagtatttttgca
atctggttaagtgttggctcactagttcgaatagataaggctttacctcttttgtaa
>IS7_petD
atatgtgtagtcaaggtggcaaatcttaaaagtcgtatgttgttggctaagttatcttgt
ggtatggggcataacagttatggtgaagctgcatgacccaatgatattgtgtacatattt
gcagttctgatatatggcttaggagtgctgatacttggcttatctattgcttgtccttta
gaaataatgtcttgttcaaatgctttctctactccattagaaattgtacctgagtggtat
ctcctactgtgctttaacttattgcgtattgtcacttccaaaggaataggtgttaaggaa
atggtgatcttagtcccaattattatttgcttgtgcattatagagaatgttagtgtatat
tctaatccttttagaagagctataatggtatgtgtaactttagtgtatagtatttttgca
atctggttaagtgttggttcactagttcgaatagataaggctttacctcttttgtaa
>IS8_petD
atatgtgtagtcaaggtggcaaatcttaaaagttgtatgttgttggctaagttatcttgt
gggatgggacataacagttatggtgaagctgcatgaccaaatgatattgtgtacatattt
gcagttctgatatatggcttaggagtgctgatatttggcttatctattgcttgtccttta
gaaataatgtcttgttcaaatgctttctctactccattagaaattgtacctgagtggtat
ctcctactgtgctttaacttattgcgtattgtcacttccaaaggaataggtgttaaggaa
atggtgatcttagtcccaattattatttgcttgtgcattatagagaatgttagtgtatat
tctaatccttttagaagagctataatggtatgtgtaactttagtgtatagtatttttgca
atctggttaagtgttggctcactagttcgaatagataaggctttacctcttttgtaa
>IS9_petD
atatgtgtagtcaaggtggcaaatcttaaaagttgtatgttgttggctaagttatcttgt
ggtatggggcataacagttatggtgaagctgcatgacccaatgatattgtgtacatattt
gcagttctgatatatggcttaggagtgctgatatttggcttatctattgcttgtccttta
gaaataatgtcttgttcaaatgctttctctactccattagaaattgtacctgagtagtat
ctcctactgtgctttaacttattgcgtattatcacttccaaaggaataggtgttaaggaa
atggtgatcttagtcccaattattgtttgcttgtgcattatagagaatgttagtgtatat
tctaatccttttagaagagctataatgctatgtgtaactttagtgtatagtatttttgca
atctggttaagtgttggttcactagttcgaatagataaggctttacctcttttgtaa
>Ik1_petD
atatgtgtagtcaaggtggcaaatcttaaaagttgtatgttgttggctaagttatcttgt
gggatgggacataacagttatggtgaagctgcatgaccaaatgatattgtgtacatattt
gcagttctgatatatggcttaggagtgctgatatttggcttatctattgcttgtccttta
gaaataatgtcttgttcaaatgctttctctactccattagaaattgtacctgagtggtat
ctcctactgtgctttaacttattgcgtattgtcacttccaaaggaataggtgttaaggaa
atggtgatcttagtcccaattattatttgcttgtgcattatagagaatgttagtgtatat
tctaatccttttagaagagctataatggtatgtgtaactttagtgtatagtatttttgca
atctggttaagtgttggctcactagttcgaatagataaggctttacctcttttgtaa

>Ik2_petD
atatgtgtagtcaaggtggcaaatcttaaaagttgtatgttgttggctaagttatcttgt
gggatgggacataacagttatggtgaagctgcatgaccaaatgatattgtgtacatattt
gcagttctgatatatggcttaggagtgctgatatttggcttatctattgcttgtccttta
gaaataatgtcttgttcaaatgctttctctactccattagaaattgtacctgagtggtat
ctcctactgtgctttaacttattgcgtattgtcacttccaaaggaataggtgttaaggaa
atggtgatcttagtcccaattattgtttgcttgtgcattatagagaatgttagtgtatat
tctaatccttttagaagagctataatggtatgtgtaactttagtgtatagtatttttgca
atctggttaagtgttggctcactagttcgaatagataaggctttacctcttttgtaa
>Ik3_petD
atatgtgtagtcaaggtggcaaatcttaaaagttgtatgttgttggctaagttatcttgt
gggatgggacataacagttatggtgaagctgcatgaccaaatgatattgtgtacatattt
gcagttctgatatatggcttaggagtgctgatatttggcttatctattgcttgtccttta
gaaataatgtcttgttcaaatgctttctctactccattagaaattgtacctgagtggtat
ctcctactgtgctttaacttattgcgtattgtcacttccaaaggaataggtgttaaggaa
atggtgatcttagtcccaattattgtttgcttgtgcattatagagaatgttagtgtatat
tctaatccttttagaagagctataatggtatgtgtaactttagtgtatagtatttttgca
atctggttaagtgttggctcactagttcgaatagataaggctttacctcttttgtaa
>Ik4_petD
atatgtgtagtcaaggtggcaaatcttaaaagttgtatgttgttggctaagttatcttgt
gggatgggacataacagttatggtgaagctgcatgaccaaatgatattgtgtacatattt
gcagttctgatatatggcttaggagtgctgatatttggcttatctattgcttgtccttta
gaaataatgtcttgttcaaatgctttctctactccattagaaattgtacctgagtggtat
ctcctactgtgctttaacttattgcgtattgtcacttccaaaggaataggtgttaaggaa
atggtgatcttagtcccaattattatttgcttgtgcattatagagaatgttaatgtatat
tctaatccttttagaagagctataatggtatgtgtaactttagtgtatagtatttttgca
atctggttaagtgttggctcactagttcgaatagataaggctttacctcttttgtaa
>Ik5_petD
atatgtgtagtcaaggtggcaaatcttaaaagttgtatgttgttggctaagttatcttgt
gggatgggacataacagttatggtgaagctgcatgaccaaatgatattgtgtacatattt
gcagttctgatatatggcttaggagtgctgatatttggcttatctattgcttgtccttta
gaaataatgtcttgttcaaatgctttctctactccattagaaattgtacctgagtggtat
ctcctactgtgctttaacttattgcgtattgtcacttccaaaggaataggtgttaaggaa
atggtgatcttagtcccaattattatttgcttgtgcattatagagaatgttagtgtatat
tctaatccttttagaagagctataatggtatgtgtaactttagtgtatagtatttttgca
atctggttaagtgttggctcactagttcgaatagataaggctttacctcttttgtaa
>Ik6_petD
atatgtgtagtcaaggtggcaaatcttaaaagttgtatgttgttggctaagttatcttgt
gggatgggacataacagttatggtgaagctgcatgaccaaatgatattgtgtacatattt
gcagttctgatatatggcttaggagtgctgatatttggcttatctattgcttgtccttta
gaaataatgtcttgttcaaatgctttctctactccattagaaattgtacctgagtggtat
ctcctactgtgctttaacttattgcgtattgtcacttccaaaggaataggtgttaaggaa
atggtgatcttagtcccaattattgtttgcttgtgcattatagagaatgttagtgtatat
tctaatccttttagaagagctataatggtatgtgtaactttagtgtatagtatttttgca
atctggttaagtgttggctcactagttcgaatagataaggctttacctcttttgtaa
>Ik7_petD
atatgtgtagtcaaggtggcaaatcttaaaagttgtatgttgttggctaagttatcttgt
gggatgggacataacagttatggtgaagctgcatgaccaaatgatattgtgtacatattt
gcagttctgatatatggcttaggagtgctgatatttggcttatctattgcttgtccttta
gaaataatgtcttgttcaaatgctttctctactccattagaaattgtacctgagtggtat
ctcctactgtgctttaacttattgcgtattgtcacttccaaaggaataggtgttaaggaa
atggtgatcttagtcccaattattatttgcttgtgcattatagagaatgttagtgtatat
tctaatccttttagaagagctataatggtatgtgtaactttagtgtatagtatttttgca
atctggttaagtgttggctcactagttcgaatagataaggctttacctcttttgtaa
>Ik8_petD
atatgtgtagtcaaggtggcaaatcttaaaagttgtatgttgttggctaagttatcttgt
gggatgggacataacagttatggtgaagctgcatgaccaaatgatattgtgtacatattt

gcagttctgatatatggcttaggagtgctgatatttggcttatctattgcttgtccttta
gaaataatgtcttgttcaaatgctttctctactccattagaaattgtacctgagtggtat
ctcctactgtgctttaacttattgcgtattgtcacttccaaaggaataggtgttaaggaa
atggtgatcttagtcccaattattgtttgcttgtgcattatagagaatgttagtgtatat
tctaatccttttagaagagctataatggtatgtgtaactttagtgtatagtatttttgca
atctggttaagtgttggctcactagttcgaatagataaggctttacctcttttgtaa
>Ik9_petD
atatgtgtagtcaaggtggcaaatcttaaaagttgtatgttgttggctaagttatcttgt
gggatgggacataacagttatggtgaagctgcatgaccaaatgatattgtgtacatattt
gcagttctgatatatggcttaggagtgctgatatttggcttatctattgcttgtccttta
gaaataatgtcttgttcaaatgctttctctactccattagaaattgtacctgagtggtat
ctcctactgtgctttaacttattgcgtattgtcacttccaaaggaataggtgttaaggaa
atggtgatcttagtcccaattattatttgcttgtgcattatagagaatgttagtgtatat
tctaatccttttagaagagctataatggtatgtgtaactttagtgtatagtatttttgca
atctggttaagtgttggctcactagttcgaatagataaggctttacctcttttgtaa
>Irm10_petD
atatgtgtagtcaaggtggcaaatcttaaaagttgtatgttgttggctaagttatcttgt
gggatgggacataacagttatggtgaagctgcatgaccaaatgatattgtgtacatattt
gcagttctgatatatggcttaggagtgctgatacttggcttatctattgcttgtccttta
gaaataatgtcttgttcaaatgctttctctactccattagaaattgtacctgagtggtat
ctcctactgtgctttaacttattgcgtattgtcacttccaaaggaataggtgttaaggaa
atggtgatcttagtcccaattattatttgcttgtgcattatagagaatgttagtgtatat
tctaatccttttagaagagctataatggtatgtgtaactttagtgtatagtatttttgca
atctggttaagtgttggctcactagttcgaatagataaggctttacctcttttgtaa
>Irm17_petD
atatgtgtagtcaaggtggcaaatcttaaaagttgtatgttgttggctaagttatcttgt
gggatgggacataacagttatggtgaagctgcatgaccaaatgatattgtgtacatattt
gcagttctgatatatggcttaggagtgctgatatttggcttatctattgcttgtccttta
gaaataatgtcttgttcaaatgctttctctactccattagaaattgtacctgagtggtat
ctcctactgtgctttaacttattgcgtattgtcacttccaaaggaataggtgttaaggaa
atggtgatcttagtcccaattattatttgcttgtgcattatagagaatgttagtgtatat
tctaatccttttagaagagctataatggtatgtgtaactttagtgtatagtatttttgca
atctggttaagtgttggctcactagttcgaatagataaggctttacctcttttgtaa
>Irm2_petD
atatgtgtagtcaaggtggcaaatcttaaaagttgtatgttgttggctaagttatcttgt
gggatgggacataacagttatggtgaagctgcatgaccaaatgatattgtgtacatattt
gcagttctgatatatggcttaggagtgctgatatttggcttatctattgcttgtccttta
gaaataatgtcttgttcaaatgctttctctactccattagaaattgtacctgagtggtat
ctcctactgtgctttaacttattgcgtattgtcacttccaaaggaataggtgttaaggaa
atggtgatcttagtcccaattattatttgcttgtgcattatagagaatgttagtgtatat
tctaatccttttagaagagctataatggtatgtgtaactttagtgtatagtatttttgca
atctggttaagtgttggctcactagttcgaatagataaggctttacctcttttgtaa
>Irm21_petD
atatgtgtagtcaaggtggcaaatcttaaaagttgtatgttgttggctaagttatcttgt
gggatgggacataacagttatggtgaagctgcatgaccaaatgatattgtgtacatattt
gcagttctgatatatggcttaggagtgctgatacttggcttatctattgcttgtccttta
gaaataatgtcttgttcaaatgctttctctactccattagaaattgtacctgagtggtat
ctcctactgtgctttaacttattgcgtattgtcacttccaaaggaataggtgttaaggaa
atggtgatcttagtcccaattattatttgcttgtgcattatagagaatgttagtgtatat
tctaatccttttagaagagctataatggtatgtgtaactttagtgtatagtatttttgca
atctggttaagtgttggctcactagttcgaatagataaggctttacctcttttgtaa
>Irm22_petD
atatgtgtagtcaaggtggcaaatcttaaaagttgtatgttgttggctaagttatcttgt
gggatgggacataacagttatggtgaagctgcatgaccaaatgatattgtgtacatattt
gcagttctgatatatggcttaggagtgctgatacttggcttatctattgcttgtccttta
gaaataatgtcttgttcaaatgctttctctactccattagaaattgtacctgagtggtat
ctcctactgtgctttaacttattgcgtattgtcacttccaaaggaataggtgttaaggaa

atggtgatcttagtcccaattattgtttgcttgtgcattatagagaatgttagtgtatat
tctaatccttttagaagagctataatggtatgtgtaactttagtgtatagtatttttgca
atctggttaagtgttggctcactagttcgaatagataaggctttacctcttttgtaa
>Irm23_petD
atatgtgtagtcaaggtggcaaatcttaaaagttgtatgttgttggctaagttatcttgt
gggatgggacataacagttatggtgaagctgcatgaccaaatgatattgtgtacatattt
gcagttctgatatatggcttaggagtgctgatacttggcttatctattgcttgtccttta
gaaataatgtcttgttcaaatgctttctctactccattagaaattgtacctgagtggtat
ctcctactgtgctttaacttattgcgtattgtcacttccaaaggaataggtgttaaggaa
atggtgatcttagtcccaattattgtttgcttgtgcattatagagaatgttagtgtatat
tctaatccttttagaagagctataatggtatgtgtaactttagtgtatagtatttttgca
atctggttaagtgttggctcactagttcgaatagataaggctttacctcttttgtaa
>Irm24_petD
atatgtgtagtcaaggtggcaaatcttaaaagttgtatgttgttggctaagttatcttgt
gggatgggacataacagttatggtgaagctgcatgaccaaatgatattgtgtacatattt
gcagttctgatatatggcttaggagtgctgatacttggcttatctattgcttgtccttta
gaaataatgtcttgttcaaatgctttctctactccattagaaattgtacctgagtggtat
ctcctactgtgctttaacttattgcgtattgtcacttccaaaggaataggtgttaaggaa
atggtgatcttagtcccaattattatttgcttgtgcattatagagaatgttagtgtatat
tctaatccttttagaagagctataatggtatgtgtaactttagtgtatagtatttttgca
atctggttaagtgttggctcactagttcgaatagataaggctttacctcttttgtaa
>Irm25_petD
atatgtgtagtcaaggtggcaaatcttaaaagttgtatgttgttggctaagttatcttgt
gggatgggacataacagttatggtgaagctgcatgaccaaatgatattgtgtacatattt
gcagttctgatatatggcttaggagtgctgatatttggcttatctattgcttgtccttta
gaaataatgtcttgttcaaatgctttctctactccattagaaattgtacctgagtggtat
ctcctactgtgctttaacttattgcgtattgtcacttccaaaggaataggtgttaaggaa
atggtgatcttagtcctaattattatttgcttgtgcattatagagaatgttagtgtatat
tctaatccttttagaagagctataatggtatgtgtaactttagtgtatagtatttttgca
atctggttaagtgttggctcactagttcgaatagataaggctttacctcttttgtaa
>Irm26_petD
atatgtgtagtcaaggtggcaaatcttaaaagttgtatgttgttggctaagttatcttgt
gggatgggacataacagttatggtgaagctgcatgaccaaatgatattgtgtacatattt
gcagttctgatatatggcttaggagtgctgatatttggcttatctattgcttgtccttta
gaaataatgtcttgttcaaatgctttctctactccattagaaattgtacctgagtggtat
ctcctactgtgctttaacttattgcgtattgtcacttccaaaggaataggtgttaaggaa
atggtgatcttagtcccaattattgtttgcttgtgcattatagagaatgttagtgtatat
tctaatccttttagaagagctataatggtatgtgtaactttagtgtatagtatttttgca
atctggttaagtgttggctcactagttcgaatagataaggctttacctcttttgtaa
>Irm27_petD
atatgtgtagtcaaggtggcaaatcttaaaagttgtatgttgttggctaagttatcttgt
gggatgggacataacagttatggtgaagctgcatgaccaaatgatattgtgtacatattt
gcagttctgatatatggcttaggagtgctgatatttggcttatctattgcttgcccttta
gaaataatgtcttgttcaaatgctttctctaccccattagaaattgtacctgagtattat
ctcctactgtgctttaacttattgcgtattgtcacttccaaaggaataggtgttatggaa
atggtgatcttagtcctaattattattttcttgtgcattatagagaatgttagtgtatat
tctaatccttttagaagagctataatggtatgtgtaactttagtgtatagtatttttgca
atctggttaagtgttggctcactagttcgaatagataaggctttacctcttttgtaa
>Irm3_petD
atatgtgtagtcaaggtggcaaatcttaaaagtcgtatgttgttggctaagttatcttgt
ggtatggggcataacagttatggtgaagctgcatgacccaatgatattgtgtacatattt
gcagttctgatatatggcttaggagtgctgatatttggcttatctattgcttgcccttta
gaaataatgtcttgttcaaatgctttctctaccccattagaaattgtacctgagtattat
ctcctactgtgctttaacttattgcgtattatcacttccaaaggaataggtgttatggaa
atggtgatcttagtcctaattattatttgcttgtgcattatagagaatgttagtgtatat
tctaatccttttagaagagctataatgctatgtgtaactttagtgtatagtatttttgca
atctggttaagtgttggttcactagttcgaatagataaggctttacctcttttgtaa

>Irm4_petD
atatgtgtagtcaaggtggcaaatcttaaaagttgtatgttgttggctaagttatcttgt
gggatggggcataacagttatggtgaagctgcatgacccaatgatattgtgtacatattt
gcagttctgatatatggcttaggagtgctgatatttggcttatctattgcttgtccttta
gaaataatgtcttgttcaaatgctttctctactccattagaaattgtacctgagtggtat
ctcctactgtgctttaacttattgcgtattatcacttccaaaggaataggtgttatggaa
atggtgatcttagtcctaattattattttcttgtgcattatagagaatgttagtgtatat
tctaatccttttagaagagctataatgctatgtgtaactttagtgtatagtatttttgca
atctggttaagtgttggttcactagttcgaatagataaggctttacctcttttgtaa
>Irm5_petD
atatgtgtagtcaaggtggcaaatcttaaaagttgtatgttgttggctaagttatcttgt
gggatgggacataacagttatggtgaagctgcatgaccaaatgatattgtgtacatattt
gcagttctgatatatggcttaggagtgctgatatttggcttatctattgcttgtccttta
gaaataatgtcttgttcaaatgctttctctactccattagaaattgtacctgagtggtat
ctcctactgtgctttaacttattgcgtattgtcacttccaaaggaataggtgttaaggaa
atggtgatcttagtcccaattattatttgcttgtgcattatagagaatgttagtgtatat
tctaatccttttagaagagctataatggtatgtgtaactttagtgtatagtatttttgca
atctggttaagtgttggctcactagttcgaatagataaggctttacctcttttgtaa
>Irm7_petD
atatgtgtagtcaaggtggcaaatcttaaaagttgtatgttgttggctaagttatcttgt
gggatgggacataacagttatggtgaagctgcatgaccaaatgatattgtgtacatattt
gcagttctgatatatggcttaggagtgctgatacttggcttatctattgcttgtccttta
gaaataatgtcttgttcaaatgctttctctactccattagaaattgtacctgagtggtat
ctcctactgtgctttaacttattgcgtattgtcacttccaaaggaataggtgttaaggaa
atggtgatcttagtcccaattattatttgcttgtgcattatagagaatgttagtgtatat
tctaatccttttagaagagctataatggtatgtgtaactttagtgtatagtatttttgca
atctggttaagtgttggctcactagttcgaatagataaggctttacctcttttgtaa
>Irm9_petD
atatgtgtagtcaaggtggcaaatcttaaaagtcgtatgttgttggctaagttatcttgt
ggtatggggcataacagttatggtgaagctgcatgaccaaatgatattgtgtacatattt
gcagttctgatatatggcttaggagtgctgatacttggcttatctattgcttgtccttta
gaaataatgtcttgttcaaatgctttctctactccattagaaattgtacctgagtattat
ctcctactgtgctttaacttattgcgtattatcacttccaaaggaataggtgttatggaa
atggtgatcttagtcccaattattgtttgcttgtgcattatagagaatgttagtgtatat
tctaatccttttagaagagctataatgctatgtgtaactttagtgtatagtatttttgca
atctggttaagtgttggttcactagttcgaatagataaggctttacctcttttgtaa
>Isy12_petD
atatgtgtagtcaaggtggcaaatcttaaaagttgtatgttgttggctaagttatcttgt
gggatgggacataacagttatggtgaagctgcatgaccaaatgatattgtgtacatattt
gcagttctgatatatggcttaggagtgctgatacttggcttatctattgcttgtccttta
gaaataatgtcttgttcaaatgctttctctactccattagaaattgtacctgagtggtat
ctcctactgtgctttaacttattgcgtattgtcacttccaaaggaataggtgttaaggaa
atggtgatcttagtcccaattattatttgcttgtgcattatagagaatgttagtgtatat
tctaatccttttagaagagctataatggtatgtgtaactttagtgtatagtatttttgca
atctggttaagtgttggctcactagttcgaatagataaggctttacctcttttgtaa
>Isy15_petD
atatgtgtagtcaaggtggcaaatcttaaaagtcgtatgttgttggctaagttatcttgt
ggtatggggcataacagttatggtgaagctgcatgacccaatgatattgtgtacatattt
gcagttctgatatatggcttaggagtgctgatatttggcttatctattgcttgccccttta
gaaataatgtcttgttcaaatgctttctctaccccattagaaattgtacctgagtattat
ctcctactgtgctttaacttattgcgtattatcacttccaaaggaataggtgttatggaa
atggtgatcttagtcctaattattatttgcttgtgcattatagagaatgttagtgtatat
tctaatccttttagaagagctataatgctatgtgtaactttagtgtatagtatttttgca
atctggttaagtgttggttcactagttcgaatagataaggctttacctcttttgtaa
>Isy16_petD
atatgtgtagtcaaggtggcaaatcttaaaagttgtatgttgttggctaagttatcttgt
gggatgggacataacagttatggtgaagctgcatgaccaaatgatattgtgtacatattt

gcagttctgatatatggcttaggagtgctgatatttggcttatctattgcttgtccttta
gaaataatgtcttgttcaaatgctttctctactccattagaaattgtacctgagtggtat
ctcctactgtgctttaacttattgcgtattgtcacttccaaaggaataggtgttaaggaa
atggtgatcttagtcccaattattatttgcttgtgcattatagagaatgttagtgtatat
tctaatccttttagaagagctataatggtatgtgtaactttagtgtatagtatttttgca
atctggttaagtgttggctcactagttcgaatagataaggctttacctcttttgtaa
>Isy17_petD
atatgtgtagtcaaggtggcaaatcttaaaagttgtatgttgttggctaagttatcttgt
gggatgggacataacagttatggtgaagctgcatgaccaaatgatattgtgtacatattt
gcagttctgatatatggcttaggagtgctgatatttggcttatctattgcttgtccttta
gaaataatgtcttgttcaaatgctttctctactccattagaaattgtacctgagtggtat
ctcctactgtgctttaacttattgcgtattgtcacttccaaaggaataggtgttaaggaa
atggtgatcttagtcccaattattgtttgcttgtgcattatagagaatgttagtgtatat
tctaatccttttagaagagctataatggtatgtgtaactttagtgtatagtatttttgca
atctggttaagtgttggctcactagttcgaatagataaggctttacctcttttgtaa
>Isy18_petD
atatgtgtagtcaaggtggcaaatcttaaaagttgtatgttgttggctaagttatcttgt
gggatgggacataacagttatggtgaagctgcatgaccaaatgatattgtgtacatattt
gcagttctgatatatggcttaggagtgctgatatttggcttatctattgcttgtccttta
gaaataatgtcttgttcaaatgctttctctactccattagaaattgtacctgagtggtat
ctcctactgtgctttaacttattgcgtattgtcacttccaaaggaataggtgttaaggaa
atggtgatcttagtcccaattattatttgcttgtgcattatagagaatgttagtgtatat
tctaatccttttagaagagctataatggtatgtgtaactttagtgtatagtatttttgca
atctggttaagtgttggctcactagttcgaatagataaggctttacctcttttgtaa
>Isy21_petD
atatgtgtagtcaaggtggcaaatcttaaaagttgtatgttgttggctaagttatcttgt
gggatgggacataacagttatggtgaagctgcatgaccaaatgatattgtgtacatattt
gcagttctgatatatggcttaggagtgctgatatttggcttatctattgcttgtccttta
gaaataatgtcttgttcaaatgctttctctactccattagaaattgtacctgagtggtat
ctcctactgtgctttaacttattgcgtattgtcacttccaaaggaataggtgttaaggaa
atggtgatcttagtcccaattattatttgcttgtgcattatagagaatgttagtgtatat
tctaatccttttagaagagctataatggtatgtgtaactttagtgtatagtatttttgca
atctggttaagtgttggctcactagttcgaatagataaggctttacctcttttgtaa
>Isy22_petD
atatgtgtagtcaaggtggcaaatcttaaaagttgtatgttgttggctaagttatcttgt
gggatgggacataacagttatggtgaagctgcatgaccaaatgatattgtgtacatattt
gcagttctgatatatggcttaggagtgctgatacttggcttatctattgcttgtccttta
gaaataatgtcttgttcaaatgctttctctactccattagaaattgtacctgagtggtat
ctcctactgtgctttaacttattgcgtattgtcacttccaaaggaataggtgttaaggaa
atggtgatcttagtcccaattattatttgcttgtgcattatagagaatgttagtgtatat
tctaatccttttagaagagctataatggtatgtgtaactttagtgtatagtatttttgca
atctggttaagtgttggctcactagttcgaatagataaggctttacctcttttgtaa
>Isy23_petD
atatgtgtagtcaaggtggcaaatcttaaaagttgtatgttgttggctaagttatcttgt
gggatgggacataacagttatggtgaagctgcatgaccaaatgatattgtgtacatattt
gcagttctgatatatggcttaggagtgctgatatttggcttatctattgcttgtccttta
gaaataatgtcttgttcaaatgctttctctactccattagaaattgtacctgagtggtat
ctcctactgtgctttaacttattgcgtattgtcacttccaaaggaataggtgttaaggaa
atggtgatcttagtcccaattattatttgcttgtgcattatagagaatgttagtgtatat
tctaatccttttagaagagctataatggtatgtgtaactttagtgtatagtatttttgca
atctggttaagtgttggctcactagttcgaatagataaggctttacctcttttgtaa
>Isy24_petD
atatgtgtagtcaaggtggcaaatcttaaaagttgtatgttgttggctaagttatcttgt
gggatgggacataacagttatggtgaagctgcatgaccaaatgatattgtgtacatattt
gcagttctgatatatggcttaggagtgctgatatttggcttatctattgcttgtccttta
gaaataatgtcttgttcaaatgctttctctactccattagaaattgtacctgagtggtat
ctcctactgtgctttaacttattgcgtattgtcacttccaaaggaataggtgttaaggaa

atggtgatcttagtcccaattattgtttgcttgtgcattatagagaatgttagtgtatat
tctaatccttttagaagagctataatggtatgtgtaactttagtgtatagtatttttgca
atctggttaagtgttggctcactagttcgaatagataaggctttacctcttttgtaa
>Isy25_petD
atatgtgtagtcaaggtggcaaatcttaaaagttgtatgttgttggctaagttatcttgt
gggatgggacataacagttatggtgaagctgcatgaccaaatgatattgtgtacatattt
gcagttctgatatatggcttaggagtgctgatatttggcttatctattgcttgtccttta
gaaataatgtcttgttcaaatgctttctctactccattagaaattgtacctgagtggtat
ctcctactgtgctttaacttattgcgtattgtcacttccaaaggaataggtgttaaggaa
atggtgatcttagtcccaattattatttgcttgtgcattatagagaatgttagtgtatat
tctaatccttttagaagagctataatggtatgtgtaactttagtgtatagtatttttgca
atctggttaagtgttggctcactagttcgaatagataaggctttacctcttttgtaa
>Isy26_petD
atatgtgtagtcaaggtggcaagtcttaaaagttgtatgttgttggctaagttatcttgt
gggatgggacataacagttatggtgaagctgcatgaccaaatgatattgtgtacatattt
gcagttctgatatatggcttaggagtgctgatatttggcttatctattgcttgtccttta
gaaataatgtcttgttcaaatgctttctctactccattagaaattgtacctgagtggtat
ctcctactgtgctttaacttattgcgtattgtcacttccaaaggaataggtgttaaggaa
atggtgatcttagtcccaattattatttgcttgtgcattatagagaatgttagtgtatat
tctaatccttttagaagagctataatggtatgtgtaactttagtgtatagtatttttgca
atctggttaagtgttggctcactagttcgaatagataaggctttacctcttttgtaa
>Isy27_petD
atatgtgtagtcaaggtggcaaatcttaaaagttgtatgttgttggctaagttatcttgt
gggatgggacataacagttatggtgaagctgcatgaccaaatgatattgtgtacatattt
gcagttctgatatatggcttaggagtgctgatatttggcttatctattgcttgtccttta
gaaataatgtcttgttcaaatgctttctctactccattagaaattgtacctgagtggtat
ctcctactgtgctttaacttattgcgtattgtcacttccaaaggaataggtgttaaggaa
atggtgatcttagtcccaattattatttgcttgtgcattatagagaatgttagtgtatat
tctaatccttttagaagagctataatggtatgtgtaactttagtgtatagtatttttgca
atctggttaagtgttggctcactagttcgaatagataaggctttacctcttttgtaa
>Isy4_petD
atatgtgtagtcaaggtggcaaatcttaaaagttgtatgttgttggctaagttatcttgt
gggatgggacataacagttatggtgaagctgcatgaccaaatgatattgtgtacatattt
gcagttctgatatatggcttaggagtgctgatatttggcttatctattgcttgtccttta
gaaataatgtcttgttcaaatgctttctctactccattagaaattgtacctgagtggtat
ctcctactgtgctttaacttattgcgtattgtcacttccaaaggaataggtgttaaggaa
atggtgatcttagtcccaattattatttgcttgtgcattatagagaatgttagtgtatat
tctaatccttttagaagagctataatggtatgtgtaactttagtgtatagtatttttgca
atctggttaagtgttggctcactagttcgaatagataaggctttacctcttttgtaa
>Isy7_petD
atatgtgtagtcaaggtggcaaatcttaaaagttgtatgttgttggctaagttatcttgt
gggatgggacataacagttatggtgaagctgcatgaccaaatgatattgtgtacatattt
gcagttctgatatatggcttaggagtgctgatatttggcttatctattgcttgtccttta
gaaataatgtcttgttcaaatgctttctctaccccattagaaattgtacctgagtggtat
ctcctactgtgctttaacttattgcgtattgtcacttccaaaggaataggtgttaaggaa
atggtgatcttagtcccaattattatttgcttgtgcattatagagaatgttagtgtatat
tctaatccttttagaagagctataatggtatgtgtaactttagtgtatagtatttttgca
atctggttaagtgttggctcactagttcgaatagataaggctttacctcttttgtaa
>Isy8_petD
atatgtgtagtcaaggtggcaagtcttaaaagttgtatgttgttggctaagttatcttgt
gggatgggacataacagttatggtgaagctgcatgaccaaatgatattgtgtacatattt
gcagttctgatatatggcttaggagtgctgatatttggcttatctattgcttgtccttta
gaaataatgtcttgttcaaatgctttctctactccattagaaattgtacctgagtggtat
ctcctactgtgctttaacttattgcgtattgtcacttccaaaggaataggtgttaaggaa
atggtgatcttagtcccaattattatttgcttgtgcattatagagaatgttagtgtatat
tctaatccttttagaagagctataatggtatgtgtaactttagtgtatagtatttttgca
atctggttaagtgttggctcactagttcgaatagataaggctttacctcttttgtaa

>KrA1_petD
atatgtgtagtcaaggtggcaaatcttaaaagttgtatgttgttggctaagttatcttgt
gggatgggacataacagttatggtgaagctgcatgaccaaatgatattgtgtacatattt
gcagttctgatatatggcttaggagtgctgatatttggcttatctattgcttgtccttta
gaaataatgtcttgttcaaatgctttctctactccattagaaattgtacctgagtggtat
ctcctactgtgctttaacttattgcgtattgtcacttccaaaggaataggtgttaaggaa
atggtgatcttagtcccaattattatttgcttgtgcattatagagaatgttagtgtatat
tctaatccttttagaagagctataatggtatgtgtaactttagtgtatagtatttttgca
atctggttaagtgttggctcactagttcgaatagataaggctttacctcttttgtaa
>KrA10_petD
atatgtgtagtcaaggtggcaaatcttaaaagttgtatgttgttggctaagttatcttgt
gggatgggacataacagttatggtgaagctgcatgaccaaatgatattgtgtacatattt
gcagttctgatatatggcttaggagtgctgatatttggcttatctattgcttgtccttta
gaaataatgtcttgttcaaatgctttctctactccattagaaattgtacctgagtggtat
ctcctactgtgctttaacttattgcgtattgtcacttccaaaggaataggtgttaaggaa
atggtgatcttagtcccaattattgtttgcttgtgcattatagagaatgttagtgtatat
tctaatccttttagaagagctataatggtatgtgtaactttagtgtatagtatttttgca
atctggttaagtgttggctcactagttcgaatagataaggctttacctcttttgtaa
>KrA11_petD
atatgtgtagtcaaggtggcaaatcttaaaagttgtatgttgttggctaagttatcttgt
gggatgggacataacagttatggtgaagctgcatgaccaaatgatattgtgtacatattt
gcagttctgatatatggcttaggagtgctgatatttggcttatctattgcttgtccttta
gaaataatgtcttgttcaaatgctttctctactccattagaaattgtacctgagtggtat
ctcctactgtgctttaacttattgcgtattgtcacttccaaaggaataggtgttaaggaa
atggtgatcttagtcccaattattatttgcttgtgcattatagagaatgttagtgtatat
tctaatccttttagaagagctataatggtatgtgtaactttagtgtatagtatttttgca
atctggttaagtgttggctcactagttcgaatagataaggctttacctcttttgtaa
>KrA12_petD
atatgtgtagtcaaggtggcaaatcttaaaagttgtatgttgttggctaagttatcttgt
gggatgggacataacagttatggtgaagctgcatgaccaaatgatattgtgtacatattt
gcagttctgatatatggcttaggagtgctgatatttggcttatctattgcttgtccttta
gaaataatgtcttgttcaaatgctttctctactccattagaaattgtacctgagtggtat
ctcctactgtgctttaacttattgcgtattgtcacttccaaaggaataggtgttaaggaa
atggtgatcttagtcccaattattgtttgcttgtgcattatagagaatgttagtgtatat
tctaatccttttagaagagctataatggtatgtgtaactttagtgtatagtatttttgca
atctggttaagtgttggctcactagttcgaatagataaggctttacctcttttgtaa
>KrA13_petD
atatgtgtagtcaaggtggcaaatcttaaaagttgtatgttgttggctaagttatcttgt
gggatgggacataacagttatggtgaagctgcatgaccaaatgatattgtgtacatattt
gcagttctgatatatggcttaggagtgctgatatttggcttatctattgcttgtccttta
gaaataatgtcttgttcaaatgctttctctactccattagaaattgtacctgagtggtat
ctcctactgtgctttaacttattgcgtattgtcacttccaaaggaataggtgttaaggaa
atggtgatcttagtcccaattattatttgcttgtgcattatagagaatgttagtgtatat
tctaatccttttagaagagctataatggtatgtgtaactttagtgtatagtatttttgca
atctggttaagtgttggctcactagttcgaatagataaggctttacctcttttgtaa
>KrA14_petD
atatgtgtagtcaaggtggcaaatcttaaaagttgtatgttgttggctaagttatcttgt
gggatgggacataacagttatggtgaagctgcatgaccaaatgatattgtgtacatattt
gcagttctgatatatggcttaggagtgctgatatttggcttatctattgcttgtccttta
gaaataatgtcttgttcaaatgctttctctactccattagaaattgtacctgagtggtat
ctcctactgtgctttaacttattgcgtattgtcacttccaaaggaataggtgttaaggaa
atggtgatcttagtcccaattattatttgcttgtgcattatagagaatgttagtgtatat
tctaatccttttagaagagctataatggtatgtgtaactttagtgtatagtatttttgca
atctggttaagtgttggctcactagttcgaatagataaggctttacctcttttgtaa
>KrA15_petD
atatgtgtagtcaaggtggcaaatcttaaaagttgtatgttgttggctaagttatcttgt
gggatgggacataacagttatggtgaagctgcatgaccaaatgatattgtgtacatattt

gcagttctgatatatggcttaggagtgctgatacttggcttatctattgcttgtccttta
gaaataatgtcttgttcaaatgctttctctactccattagaaattgtacctgagtggtat
ctcctactgtgctttaacttattgcgtattgtcacttccaaaggaataggtgttaaggaa
atggtgatcttagtcccaattattatttgcttgtgcattatagagaatgttagtgtatat
tctaatccttttagaagagctataatggtatgtgtaactttagtgtatagtatttttgca
atctggttaagtgttggctcactagttcgaatagataaggctttacctcttttgtaa
>KrA2_petD
atatgtgtagtcaaggtggcaaatcttaaaagttgtatgttgttggctaagttatcttgt
gggatgggacataacagttatggtgaagctgcatgaccaaatgatattgtgtacatattt
gcagttctgatatatggcttaggagtgctgatatttggcttatctattgcttgtccttta
gaaataatgtcttgttcaaatgctttctctactccattagaaattgtacctgagtggtat
ctcctactgtgctttaacttattgcgtattgtcacttccaaaggaataggtgttaaggaa
atggtgatcttagtcccaattattatttgcttgtgcattatagagaatgttagtgtatat
tctaatccttttagaagagctataatggtatgtgtaactttagtgtatagtatttttgca
atctggttaagtgttggctcactagttcgaatagataaggctttacctcttttgtaa
>KrA3_petD
atatgtgtagtcaaggtggcaaatcttaaaagttgtatgttgttggctaagttatcttgt
gggatgggacataacagttatggtgaagctgcatgaccaaatgatattgtgtacatattt
gcagttctgatatatggcttaggagtgctgatatttggcttatctattgcttgtccttta
gaaataatgtcttgttcaaatgctttctctactccattagaaattgtacctgagtggtat
ctcctactgtgctttaacttattgcgtattgtcacttccaaaggaataggtgttaaggaa
atggtgatcttagtcccaattattatttgcttgtgcattatagagaatgttagtgtatat
tctaatccttttagaagagctataatggtatgtgtaactttagtgtatagtatttttgca
atctggttaagtgttggctcactagttcgaatagataaggctttacctcttttgtaa
>KrA4_petD
atatgtgtagtcaaggtggcaaatcttaaaagttgtatgttgttggctaagttatcttgt
gggatgggacataacagttatggtgaagctgcatgaccaaatgatattgtgtacatattt
gcagttctgatatatggcttaggagtgctgatatttggcttatctattgcttgtccttta
gaaataatgtcttgttcaaatgctttctctactccattagaaattgtacctgagtggtat
ctcctactgtgctttaacttattgcgtattgtcacttccaaaggaataggtgttaaggaa
atggtgatcttagtcccaattattatttgcttgtgcattatagagaatgttagtgtatat
tctaatccttttagaagagctataatggtatgtgtaactttagtgtatagtatttttgca
atctggttaagtgttggctcactagttcgaatagataaggctttacctcttttgtaa
>KrA5_petD
atatgtgtagtcaaggtggcaaatcttaaaagttgtatgttgttggctaagttatcttgt
gggatgggacataacagttatggtgaagctgcatgaccaaatgatattgtgtacatattt
gcagttctgatatatggcttaggagtgctgatatttggcttatctattgcttgtccttta
gaaataatgtcttgttcaaatgctttctctactccattagaaattgtacctgagtggtat
ctcctactgtgctttaacttattgcgtattgtcacttccaaaggaataggtgttaaggaa
atggtgatcttagtcccaattattatttgcttgtgcattatagagaatgttagtgtatat
tctaatccttttagaagagctataatggtatgtgtaactttagtgtatagtatttttgca
atctggttaagtgttggctcactagttcgaatagataaggctttacctcttttgtaa
>KrA6_petD
atatgtgtagtcaaggtggcaaatcttaaaagttgtatgttgttggctaagttatcttgt
gggatgggacataacagttatggtgaagctgcatgaccaaatgatattgtgtacatattt
gcagttctgatatatggcttaggagtgctgatatttggcttatctattgcttgtccttta
gaaataatgtcttgttcaaatgctttctctactccattagaaattgtacctgagtggtat
ctcctactgtgctttaacttattgcgtattgtcacttccaaaggaataggtgttaaggaa
atggtgatcttagtcccaattattatttgcttgtgcattatagagaatgttagtgtatat
tctaatccttttagaagagctataatggtatgtgtaactttagtgtatagtatttttgca
atctggttaagtgttggctcactagttcgaatagataaggctttacctcttttgtaa
>KrA7_petD
atatgtgtagtcaaggtggcaaatcttaaaagttgtatgttgttggctaagttatcttgt
gggatgggacataacagttatggtgaagctgcatgaccaaatgatattgtgtacatattt
gcagttctgatatatggcttaggagtgctgatatttggcttatctattgcttgtccttta
gaaataatgtcttgttcaaatgctttctctactccattagaaattgtacctgagtggtat
ctcctactgtgctttaacttattgcgtattgtcacttccaaaggaataggtgttaaggaa

atggtgatcttagtcccaattattatttgcttgtgcattatagagaatgttagtgtatat
tctaatccttttagaagagctataatggtatgtgtaactttagtgtatagtatttttgca
atctggttaagtgttggctcactagttcgaatagataaggctttacctcttttgtaa
>KrA8_petD
atatgtgtagtcaaggtggcaaatcttaaaagttgtatgttgttggctaagttatcttgt
gggatgggacataacagttatggtgaagctgcatgaccaaatgatattgtgtacatattt
gcagttctgatatatggcttaggagtgctgatatttggcttatctattgcttgtccttta
gaaataatgtcttgttcaaatgctttctctactccattagaaattgtacctgagtggtat
ctcctactgtgctttaacttattgcgtattgtcacttccaaaggaataggtgttaaggaa
atggtgatcttagtcccaattattgtttgcttgtgcattatagagaatgttagtgtatat
tctaatccttttagaagagctataatggtatgtgtaactttagtgtatagtatttttgca
atctggttaagtgttggctcactagttcgaatagataaggctttacctcttttgtaa
>KrA9_petD
atatgtgtagtcaaggtggcaaatcttaaaagttgtatgttgttggctaagttatcttgt
gggatgggacataacagttatggtgaagctgcatgaccaaatgatattgtgtacatattt
gcagttctgatatatggcttaggagtgctgatatttggcttatctattgcttgtccttta
gaaataatgtcttgttcaaatgctttctctactccattagaaattgtacctgagtggtat
ctcctactgtgctttaacttattgcgtattgtcacttccaaaggaataggtgttaaggaa
atggtgatcttagtcccaattattgtttgcttgtgcattatagagaatgttagtgtatat
tctaatccttttagaagagctataatggtatgtgtaactttagtgtatagtatttttgca
atctggttaagtgttggctcactagttcgaatagataaggctttacctcttttgtaa
>KrC1_petD
atatgtgtagtcaaggtggcaaatcttaaaagttgtatgttgttggctaagttatcttgt
gggatgggacataacagttatggtgaagctgcatgaccaaatgatattgtgtacatattt
gcagttctgatatatggcttaggagtgctgatatttggcttatctattgcttgtccttta
gaaataatgtcttgttcaaatgctttctctactccattagaaattgtacctgagtggtat
ctcctactgtgctttaacttattgcgtattgtcacttccaaaggaataggtgttaaggaa
atggtgatcttagtcccaattattatttgcttgtgcattatagagaatgttagtgtatat
tctaatccttttagaagagctataatggtatgtgtaactttagtgtatagtatttttgca
atctggttaagtgttggctcactagttcgaatagataaggctttacctcttttgtaa
>KrC10_petD
atatgtgtagtcaaggtggcaaatcttaaaagttgtatgttgttggctaagttatcttgt
gggatgggacataacagttatggtgaagctgcatgaccaaatgatattgtgtacatattt
gcagttctgatatatggattaggagtgctgatatttggcttatctattgcttgtccttta
gaaataatgtcttgttcaaatgctttctctactccattagaaattgtacctgagtggtat
ctcctactgtgctttaacttattgcgtattgtcacttccaaaggaataggtgttaaggaa
atggtgatcttagtcccaattattatttgcttgtgcattatagagaatgttagtgtatat
tctaatccttttagaagagctataatggtatgtgtaactttagtgtatagtatttttgca
atctggttaagtgttggctcactagttcgaatagataaggctttacctcttttgtaa
>KrC11_petD
atatgtgtagtcaaggtggcaaatcttaaaagttgtatgttgttggctaagttatcttgt
gggatgggacataacagttatggtgaagctgcatgaccaaatgatattgtgtacatattt
gcagttctgatatatggattaggagtgctgatatttggcttatctattgcttgtccttta
gaaataatgtcttgttcaaatgctttctctactccattagaaattgtacctgagtggtat
ctcctactgtgctttaacttattgcgtattgtcacttccaaaggaataggtgttaaggaa
atggtgatcttagtcccaattattatttgcttgtgcattatagagaatgttagtgtatat
tctaatccttttagaagagctataatggtatgtgtaactttagtgtatagtatttttgca
atctggttaagtgttggctcactagttcgaatagataaggctttacctcttttgtaa
>KrC12_petD
atatgtgtagtcaaggtggcaaatcttaaaagttgtatgttgttggctaagttatcttgt
gggatgggacataacagttatggtgaagctgcatgaccaaatgatattgtgtacatattt
gcagttctgatatatggcttaggagtgctgatatttggcttatctattgcttgtccttta
gaaataatgtcttgttcaaatgctttctctactccattagaaattgtacctgagtggtat
ctcctactgtgctttaacttattgcgtattgtcacttccaaaggaataggtgttaaggaa
atggtgatcttagtcccaattattgtttgcttgtgcattatagagaatgttagtgtatat
tctaatccttttagaagagctataatggtatgtgtaactttagtgtatagtatttttgca
atctggttaagtgttggctcactagttcgaatagataaggctttacctcttttgtaa

>KrC13_petD
atatgtgtagtcaaggtggcaaatcttaaaagttgtatgttgttggctaagttatcttgt
gggatgggacataacagttatggtgaagctgcatgaccaaatgatattgtgtacatattt
gcagttctgatatatggattaggagtgctgatatttggcttatctattgcttgtccttta
gaaataatgtcttgttcaaatgctttctctactccattagaaattgtacctgagtggtat
ctcctactgtgctttaacttattgcgtattgtcacttccaaaggaataggtgttaaggaa
atggtgatcttagtcccaattattatttgcttgtgcattatagagaatgttagtgtatat
tctaatccttttagaagagctataatggtatgtgtaactttagtgtatagtatttttgca
atctggttaagtgttggctcactagttcgaatagataaggctttacctcttttgtaa
>KrC14_petD
atatgtgtagtcaaggtggcaaatcttaaaagttgtatgttgttggctaagttatcttgt
gggatgggacataacagttatggtgaagctgcatgaccaaatgatattgtgtacatattt
gcagttctgatatatggcttaggagtgctgatatttggcttatctattgcttgtccttta
gaaataatgtcttgttcaaatgctttctctactccattagaaattgtacctgagtggtat
ctcctactgtgctttaacttattgcgtattgtcacttccaaaggaataggtgttaaggaa
atggtgatcttagtcccaattattatttgcttgtgcattatagagaatgttagtgtatat
tctaatccttttagaagagctataatggtatgtgtaactttagtgtatagtatttttgca
atctggttaagtgttggctcactagttcgaatagataaggctttacctcttttgtaa
>KrC15_petD
atatgtgtagtcaaggtggcaaatcttaaaagttgtatgttgttggctaagttatcttgt
gggatgggacataacagttatggtgaagctgcatgaccaaatgatattgtgtacatattt
gcagttctgatatatggcttaggagtgctgatatttggcttatctattgcttgtccttta
gaaataatgtcttgttcaaatgctttctctactccattagaaattgtacctgagtggtat
ctcctactgtgctttaacttattgcgtattgtcacttccaaaggaataggtgttaaggaa
atggtgatcttagtcccaattattatttgcttgtgcattatagagaatgttagtgtatat
tctaatccttttagaagagctataatggtatgtgtaactttagtgtatagtatttttgca
atctggttaagtgttggctcactagttcgaatagataaggctttacctcttttgtaa
>KrC2_petD
atatgtgtagtcaaggtggcaaatcttaaaagttgtatgttgttggctaagttatcttgt
gggatgggacataacagttatggtgaagctgcatgaccaaatgatattgtgtacatattt
gcagttctgatatatggcttaggagtgctgatacttggcttatctattgcttgtccttta
gaaataatgtcttgttcaaatgctttctctactccattagaaattgtacctgagtggtat
ctcctactgtgctttaacttattgcgtattgtcacttccaaaggaataggtgttaaggaa
atggtgatcttagtcccaattattatttgcttgtgcattatagagaatgttagtgtatat
tctaatccttttagaagagctataatggtatgtgtaactttagtgtatagtatttttgca
atctggttaagtgttggctcactagttcgaatagataaggctttacctcttttgtaa
>KrC3_petD
atatgtgtagtcaaggtggcaaatcttaaaagttgtatgttgttggctaagttatcttgt
gggatgggacataacagttatggtgaagctgcatgaccaaatgatattgtgtacatattt
gcagttctgatatatggattaggagtgctgatatttggcttatctattgcttgtccttta
gaaataatgtcttgttcaaatgctttctctactccattagaaattgtacctgagtggtat
ctcctactgtgctttaacttattgcgtattgtcacttccaaaggaataggtgttaaggaa
atggtgatcttagtcccaattattatttgcttgtgcattatagagaatgttagtgtatat
tctaatccttttagaagagctataatggtatgtgtaactttagtgtatagtatttttgca
atctggttaagtgttggctcactagttcgaatagataaggctttacctcttttgtaa
>KrC4_petD
atatgtgtagtcaaggtggcaaatcttaaaagttgtatgttgttggctaagttatcttgt
gggatgggacataacagttatggtgaagctgcatgaccaaatgatattgtgtacatattt
gcagttctgatatatggcttaggagtgctgatatttggcttatctattgcttgtccttta
gaaataatgtcttgttcaaatgctttctctactccattagaaattgtacctgagtggtat
ctcctactgtgctttaacttattgcgtattgtcacttccaaaggaataggtgttaaggaa
atggtgatcttagtcccaattattgtttgcttgtgcattatagagaatgttagtgtatat
tctaatccttttagaagagctataatggtatgtgtaactttagtgtatagtatttttgca
atctggttaagtgttggctcactagttcgaatagataaggctttacctcttttgtaa
>KrC5_petD
atatgtgtagtcaaggtggcaaatcttaaaagttgtatgttgttggctaagttatcttgt
gggatgggacataacagttatggtgaagctgcatgaccaaatgatattgtgtacatattt

gcagttctgatatatggcttaggagtgctgatatttggcttatctattgcttgtccttta
gaaataatgtcttgttcaaatgctttctctactccattagaaattgtacctgagtggtat
ctcctactgtgctttaacttattgcgtattgtcacttccaaaggaataggtgttaaggaa
atggtgatcttagtcccaattattatttgcttgtgcattatagagaatgttagtgtatat
tctaatccttttagaagagctataatggtatgtgtaactttagtgtatagtatttttgca
atctggttaagtgttggctcactagttcgaatagataaggctttacctcttttgtaa
>KrC6_petD
atatgtgtagtcaaggtggcaaatcttaaaagttgtatgttgttggctaagttatcttgt
gggatgggacataacagttatggtgaagctgcatgaccaaatgatattgtgtacatattt
gcagttctgatatatggcttaggagtgctgatatttggcttatctattgcttgtccttta
gaaataatgtcttgttcaaatgctttctctactccattagaaattgtacctgagtggtat
ctcctactgtgctttaacttattgcgtattgtcacttccaaaggaataggtgttaaggaa
atggtgatcttagtcccaattattatttgcttgtgcattatagagaatgttagtgtatat
tctaatccttttagaagagctataatggtatgtgtaactttagtgtatagtatttttgca
atctggttaagtgttggctcactagttcgaatagataaggctttacctcttttgtaa
>KrC7_petD
atatgtgtagtcaaggtggcaaatcttaaaagttgtatgttgttggctaagttatcttgt
gggatgggacataacagttatggtgaagctgcatgaccaaatgatattgtgtacatattt
gcagttctgatatatggcttaggagtgctgatatttggcttatctattgcttgtccttta
gaaataatgtcttgttcaaatgctttctctactccattagaaattgtacctgagtggtat
ctcctactgtgctttaacttattgcgtattgtcacttccaaaggaataggtgttaaggaa
atggtgatcttagtcccaattattatttgcttgtgcattatagagaatgttagtgtatat
tctaatccttttagaagagctataatggtatgtgtaactttagtgtatagtatttttgca
atctggttaagtgttggctcactagttcgaatagataaggctttacctcttttgtaa
>KrC8_petD
atatgtgtagtcaaggtggcaaatcttaaaagttgtatgttgttggctaagttatcttgt
gggatgggacataacagttatgatgaagctgcatgaccaaatgatattgtgtacatattt
gcagttctgatatatggcttaggagtgctgatatttggcttatctattgcttgtccttta
gaaataatgtcttgttcaaatgctttctctactccattagaaattgtacctgagtggtat
ctcctactgtgctttaacttattgcgtattgtcacttccaaaggaataggtgttaaggaa
atggtgatcttagtcccaattattatttgcttgtgcattatagagaatgttagtgtatat
tctaatccttttagaagagctataatggtatgtgtaactttagtgtatagtatttttgca
atctggttaagtgttggctcactagttcgaatagataaggctttacctcttttgtaa
>KrC9_petD
atatgtgtagtcaaggtggcaaatcttaaaagttgtatgttgttggctaagttatcttgt
gggatgggacataacagttatggtgaagctgcatgaccaaatgatattgtgtacatattt
gcagttctgatatatggattaggagtgctgatatttggcttatctattgcttgtccttta
gaaataatgtcttgttcaaatgctttctctactccattagaaattgtacctgagtggtat
ctcctactgtgctttaacttattgcgtattgtcacttccaaaggaataggtgttaaggaa
atggtgatcttagtcccaattattgtttgcttgtgcattatagagaatgttagtgtatat
tctaatccttttagaagagctataatggtatgtgtaactttagtgtatagtatttttgca
atctggttaagtgttggctcactagttcgaatagataaggctttacctcttttgtaa
>KrD1_petD
atatgtgtagtcaaggtggcaaatcttaaaagttgtatgttgttggctaagttatcttgt
gggatgggacataacagttatggtgaagctgcatgaccaaatgatattgtgtacatattt
gcagttctgatatatggcttaggagtgctgatatttggcttatctattgcttgtccttta
gaaataatgtcttgttcaaatgctttctctactccattagaaattgtacctgagtggtat
ctcctactgtgctttaacttattgcgtattgtcacttccaaaggaataggtgttaaggaa
atggtgatcttagtcccaattattatttgcttgtgcattatagagaatgttagtgtatat
tctaatccttttagaagagctataatggtatgtgtaactttagtgtatagtatttttgca
atctggttaagtgttggctcactagttcgaatagataaggctttacctcttttgtaa
>KrD11_petD
atatgtgtagtcaaggtggcaaatcttaaaagttgtatgttgttggctaagttatcttgt
gggatgggacataacagttatggtgaagctgcatgaccaaatgatattgtgtacatattt
gcagttctgatatatggcttaggagtgctgatatttggcttatctattgcttgtccttta
gaaataatgtcttgttcaaatgctttctctactccattagaaattgtacctgagtggtat
ctcctactgtgctttaacttattgcgtattgtcacttccaaaggaataggtgttaaggaa

atggtgatcttagtcccaattattatttgcttgtgcattatagagaatgttagtgtatat
tctaatccttttagaagagctataatggtatgtgtaactttagtgtatagtatttttgca
atctggttaagtgttggctcactagttcgaatagataaggctttacctcttttgtaa
>KrD13_petD
atatgtgtagtcaaggtggcaaatcttaaaagttgtatgttgttggctaagttatcttgt
gggatgggacataacagttatggtgaagctgcatgaccaaatgatattgtgtacatattt
gcagttctgatatatggcttaggagtgctgatatttggcttatctattgcttgtccttta
gaaataatgtcttgttcaaatgctttctctactccattagaaattgtacctgagtggtat
ctcctactgtgctttaacttattgcgtattgtcacttccaaaggaataggtgttaaggaa
atggtgatcttagtcccaattattatttgcttgtgcattatagagaatgttagtgtatat
tctaatccttttagaagagctataatggtatgtgtaactttagtgtatagtatttttgca
atctggttaagtgttggctcactagttcgaatagataaggctttacctcttttgtaa
>KrD3_petD
atatgtgtagtcaaggtggcaaatcttaaaagttgtatgttgttggctaagttatcttgt
gggatgggacataacagttatggtgaagctgcatgaccaaatgatattgtgtacatattt
gcagttctgatatatggcttaggagtgctgatacttggcttatctattgcttgtccttta
gaaataatgtcttgttcaaatgctttctctactccattagaaattgtacctgagtggtat
ctcctactgtgctttaacttattgcgtattgtcacttccaaaggaataggtgttaaggaa
atggtgatcttagtcccaattattatttgcttgtgcattatagagaatgttagtgtatat
tctaatccttttagaagagctataatggtatgtgtaactttagtgtatagtatttttgca
atctggttaagtgttggctcactagttcgaatagataaggctttacctcttttgtaa
>KrD8_petD
atatgtgtagtcaaggtggcaaatcttaaaagttgtatgttgttggctaagttatcttgt
gggatgggacataacagttatggtgaagctgcatgaccaaatgatattgtgtacatattt
gcagttctgatatatggcttaggagtgctgatacttggcttatctattgcttgtccttta
gaaataatgtcttgttcaaatgctttctctactccattagaaattgtacctgagtggtat
ctcctactgtgctttaacttattgcgtattgtcacttccaaaggaataggtgttaaggaa
atggtgatcttagtcccaattattgtttgcttgtgcattatagagaatgttagtgtatat
tctaatccttttagaagagctataatggtatgtgtaactttagtgtatagtatttttgca
atctggttaagtgttggctcactagttcgaatagataaggctttacctcttttgtaa
>KrD9_petD
atatgtgtagtcaaggtggcaaatcttaaaagttgtatgttgttggctaagttatcttgt
gggatgggacataacagttatggtgaagctgcatgaccaaatgatattgtgtacatattt
gcagttctgatatatggcttaggagtgctgatatttggcttatctattgcttgtccttta
gaaataatgtcttgttcaaatgctttctctactccattagaaattgtacctgagtggtat
ctcctactgtgctttaacttattgcgtattgtcacttccaaaggaataggtgttaaggaa
atggtgatcttagtcccaattattgtttgcttgtgcattatagagaatgttagtgtatat
tctaatccttttagaagagctataatggtatgtgtaactttagtgtatagtatttttgca
atctggttaagtgttggctcactagttcgaatagataaggctttacctcttttgtaa
>KrE1_petD
atatgtgtagtcaaggtggcaaatcttaaaagttgtatgttgttggctaagttatcttgt
gggatgggacataacagttatggtgaagctgcatgaccaaatgatattgtgtacatattt
gcagttctgatatatggattaggagtgctgatatttggcttatctattgcttgtccttta
gaaataatgtcttgttcaaatgctttctctactccattagaaattgtacctgagtggtat
ctcctactgtgctttaacttattgcgtattgtcacttccaaaggaataggtgttaaggaa
atggtgatcttagtcccaattattatttgcttgtgcattatagagaatgttagtgtatat
tctaatccttttagaagagctataatggtatgtgtaactttagtgtatagtatttttgca
atctggttaagtgttggctcactagttcgaatagataaggctttacctcttttgtaa
>KrE10_petD
atatgtgtagtcaaggtggcaaatcttaaaagttgtatgttgttggctaagttatcttgt
gggatgggacataacagttatggtgaagctgcatgaccaaatgatattgtgtacatattt
gcagttctgatatatggcttaggagtgctgatatttggcttatctattgcttgtccttta
gaaataatgtcttgttcaaatgctttctctactccattagaaattgtacctgagtggtat
ctcctactgtgctttaacttattgcgtattgtcacttccaaaggaataggtgttaaggaa
atggtgatcttagtcccaattattatttgcttgtgcattatagagaatgttagtgtatat
tctaatccttttagaagagctataatggtatgtgtaactttagtgtatagtatttttgca
atctggttaagtgttggctcactagttcgaatagataaggctttacctcttttgtaa

>KrE11_petD
atatgtgtagtcaaggtggcaaatcttaaaagttgtatgttgttggctaagttatcttgt
gggatgggacataacagttatggtgaagctgcatgaccaaatgatattgtgtacatattt
gcagttctgatatatggcttaggagtgctgatatttggcttatctattgcttgtccttta
gaaataatgtcttgttcaaatgctttctctactccattagaaattgtacctgagtggtat
ctcctactgtgctttaacttattgcgtattgtcacttccaaaggaataggtgttaaggaa
atggtgatcttagtcccaattattatttgcttgtgcattatagagaatgttagtgtatat
tctaatccttttagaagagctataatggtatgtgtaactttagtgtatagtatttttgca
atctggttaagtgttggctcactagttcgaatagataaggctttacctcttttgtaa
>KrE12_petD
atatgtgtagtcaaggtggcaaatcttaaaagttgtatgttgttggctaagttatcttgt
gggatgggacataacagttatggtgaagctgcatgaccaaatgatattgtgtacatattt
gcagttctgatatatggcttaggagtgctgatatttggcttatctattgcttgtccttta
gaaataatgtcttgttcaaatgctttctctactccattagaaattgtacctgagtggtat
ctcctactgtgctttaacttattgcgtattgtcacttccaaaggaataggtgttaaggaa
atggtgatcttagtcccaattattatttgcttgtgcattatagagaatgttagtgtatat
tctaatccttttagaagagctataatggtatgtgtaactttagtgtatagtatttttgca
atctggttaagtgttggctcactagttcgaatagataaggctttacctcttttgtaa
>KrE13_petD
atatgtgtagtcaaggtggcaaatcttaaaagttgtatgttgttggctaagttatcttgt
gggatgggacataacagttatggtgaagctgcatgaccaaatgatattgtgtacatattt
gcagttctgatatatggcttaggagtgctgatatttggcttatctattgcttgtccttta
gaaataatgtcttgttcaaatgctttctctactccattagaaattgtacctgagtggtat
ctcctactgtgctttaacttattgcgtattgtcacttccaaaggaataggtgttaaggaa
atggtgatcttagtcccaattattatttgcttgtgcattatagagaatgttagtgtatat
tctaatccttttagaagagctataatggtatgtgtaactttagtgtatagtatttttgca
atctggttaagtgttggctcactagttcgaatagataaggctttacctcttttgtaa
>KrE14_petD
atatgtgtagtcaaggtggcaaatcttaaaagttgtatgttgttggctaagttatcttgt
gggatgggacataacagttatggtgaagctgcatgaccaaatgatattgtgtacatattt
gcagttctgatatatggcttaggagtgctgatatttggcttatctattgcttgtccttta
gaaataatgtcttgttcaaatgctttctctactccattagaaattgtacctgagtggtat
ctcctactgtgctttaacttattgcgtattgtcacttccaaaggaataggtgttaaggaa
atggtgatcttagtcccaattattatttgcttgtgcattatagagaatgttagtgtatat
tctaatccttttagaagagctataatggtatgtgtaactttagtgtatagtatttttgca
atctggttaagtgttggctcactagttcgaatagataaggctttacctcttttgtaa
>KrE15_petD
atatgtgtagtcaaggtggcaaatcttaaaagttgtatgttgttggctaagttatcttgt
gggatgggacataacagttatggtgaagctgcatgaccaaatgatattgtgtacatattt
gcagttctgatatatggcttaggagtgctgatatttggcttatctattgcttgtccttta
gaaataatgtcttgttcaaatgctttctctactccattagaaattgtacctgagtggtat
ctcctactgtgctttaacttattgcgtattgtcacttccaaaggaataggtgttaaggaa
atggtgatcttagtcccaattattatttgcttgtgcattatagagaatgttagtgtatat
tctaatccttttagaagagctataatggtatgtgtaactttagtgtatagtatttttgca
atctggttaagtgttggctcactagttcgaatagataaggctttacctcttttgtaa
>KrE2_petD
atatgtgtagtcaaggtggcaaatcttaaaagttgtatgttgttggctaagttatcttgt
gggatgggacataacagttatggtgaagctgcatgaccaaatgatattgtgtacatattt
gcagttctgatatatggcttaggagtgctgatatttggcttatctattgcttgtccttta
gaaataatgtcttgttcaaatgctttctctactccattagaaattgtacctgagtggtat
ctcctactgtgctttaacttattgcgtattgtcacttccaaaggaataggtgttaaggaa
atggtgatcttagtcccaattattatttgcttgtgcattatagagaatgttagtgtatat
tctaatccttttagaagagctataatggtatgtgtaactttagtgtatagtatttttgca
atctggttaagtgttggctcactagttcgaatagataaggctttacctcttttgtaa
>KrE3_petD
atatgtgtagtcaaggtggcaaatcttaaaagttgtatgttgttggctaagttatcttgt
gggatgggacataacagttatggtgaagctgcatgaccaaatgatattgtgtacatattt

gcagttctgatatatggcttaggagtgctgatatttggcttatctattgcttgtccttta
gaaataatgtcttgttcaaatgctttctctactccattagaaattgtacctgagtggtat
ctcctactgtgctttaacttattgcgtattgtcacttccaaaggaataggtgttaaggaa
atggtgatcttagtcccaattattatttgcttgtgcattatagagaatgttagtgtatat
tctaatccttttagaagagctataatggtatgtgtaactttagtgtatagtatttttgca
atctggttaagtgttggctcactagttcgaatagataaggctttacctcttttgtaa
>KrE4_petD
atatgtgtagtcaaggtggcaaatcttaaaagttgtatgttgttggctaagttatcttgt
gggatgggacataacagttatggtgaagctgcatgaccaaatgatattgtgtacatattt
gcagttctgatatatggcttaggagtgctgatatttggcttatctattgcttgtccttta
gaaataatgtcttgttcaaatgctttctctactccattagaaattgtacctgagtggtat
ctcctactgtgctttaacttattgcgtattgtcacttccaaaggaataggtgttaaggaa
atggtgatcttagtcccaattattatttgcttgtgcattatagagaatgttagtgtatat
tctaatccttttagaagagctataatggtatgtgtaactttagtgtatagtatttttgca
atctggttaagtgttggctcactagttcgaatagataaggctttacctcttttgtaa
>KrE5_petD
atatgtgtagtcaaggtggcaaatcttaaaagttgtatgttgttggctaagttatcttgt
gggatgggacataacagttatggtgaagctgcatgaccaaatgatattgtgtacatattt
gcagttctgatatatggcttaggagtgctgatatttggcttatctattgcttgtccttta
gaaataatgtcttgttcaaatgctttctctactccattagaaattgtacctgagtggtat
ctcctactgtgctttaacttattgcgtattgtcacttccaaaggaataggtgttaaggaa
atggtgatcttagtcccaattattatttgcttgtgcattatagagaatgttagtgtatat
tctaatccttttagaagagctataatggtatgtgtaactttagtgtatagtatttttgca
atctggttaagtgttggctcactagttcgaatagataaggctttacctcttttgtaa
>KrE6_petD
atatgtgtagtcaaggtggcaaatcttaaaagttgtatgttgttggctaagttatcttgt
gggatgggacataacagttatggtgaagctgcatgaccaaatgatattgtgtacatattt
gcagttctgatatatggcttaggagtgctgatatttggcttatctattgcttgtccttta
gaaataatgtcttgttcaaatgctttctctactccattagaaattgtacctgagtggtat
ctcctactgtgctttaacttattgcgtattgtcacttccaaaggaataggtgttaaggaa
atggtgatcttagtcccaattattatttgcttgtgcattatagagaatgttagtgtatat
tctaatccttttagaagagctataatggtatgtgtaactttagtgtatagtatttttgca
atctggttaagtgttggctcactagttcgaatagataaggctttacctcttttgtaa
>KrE7_petD
atatgtgtagtcaaggtggcaaatcttaaaagttgtatgttgttggctaagttatcttgt
gggatgggacataacagttatggtgaagctgcatgaccaaatgatattgtgtacatattt
gcagttctgatatatggcttaggagtgctgatatttggcttatctattgcttgtccttta
gaaataatgtcttgttcaaatgctttctctactccattagaaattgtacctgagtggtat
ctcctactgtgctttaacttattgcgtattgtcacttccaaaggaataggtgttaaggaa
atggtgatcttagtcccaattattatttgcttgtgcattatagagaatgttagtgtatat
tctaatccttttagaagagctataatggtatgtgtaactttagtgtatagtatttttgca
atctggttaagtgttggctcactagttcgaatagataaggctttacctcttttgtaa
>KrE8_petD
atatgtgtagtcaaggtggcaaatcttaaaagttgtatgttgttggctaagttatcttgt
gggatgggacataacagttatggtgaagctgcatgaccaaatgatattgtgtacatattt
gcagttctgatatatggcttaggagtgctgatatttggcttatctattgcttgtccttta
gaaataatgtcttgttcaaatgctttctctactccattagaaattgtacctgagtggtat
ctcctactgtgctttaacttattgcgtattgtcacttccaaaggaataggtgttaaggaa
atggtgatcttagtcccaattattatttgcttgtgcattatagagaatgttagtgtatat
tctaatccttttagaagagctataatggtatgtgtaactttagtgtatagtatttttgca
atctggttaagtgttggctcactagttcgaatagataaggctttacctcttttgtaa
>KrE9_petD
atatgtgtagtcaaggtggcaaatcttaaaagttgtatgttgttggctaagttatcttgt
gggatgggacataacagttatggtgaagctgcatgaccaaatgatattgtgtacatattt
gcagttctgatatatggcttaggagtgctgatatttggcttatctattgcttgtccttta
gaaataatgtcttgttcaaatgctttctctactccattagaaattgtacctgagtggtat
ctcctactgtgctttaacttattgcgtattgtcacttccaaaggaataggtgttaaggaa

atggtgatcttagtcccaattattatttgcttgtgcattatagagaatgttagtgtatat
tctaatccttttagaagagctataatggtatgtgtaactttagtgtatagtatttttgca
atctggttaagtgttggctcactagttcgaatagataaggctttacctcttttgtaa
>Mz10_petD
atatgtgtagtcaaggtggcaaatcttaaaagttgtatgttgttggctaagttatcttgt
gggatgggacataacagttatggtgaagctgcatgaccaaatgatattgtgtacatattt
gcagttctgatatatggcttaggagtgctgatatttggcttatctattgcttgtccttta
gaaataatgtcttgttcaaatgctttctctactccattagaaattgtacctgagtggtat
ctcctactgtgctttaacttattgcgtattgtcacttccaaaggaataggtgttaaggaa
atggtgatcttagtcccaattattatttgcttgtgcattatagagaatgttagtgtatat
tctaatccttttagaagagctataatggtatgtgtaactttagtgtatagtatttttgca
atctggttaagtgttggctcactagttcgaatagataaggctttacctcttttgtaa
>Mz5_petD
atatgtgtagtcaaggtggcaaatcttaaaagttgtatgttgttggctaagttatcttgt
gggatgggacataacagttatggtgaagctgcatgaccaaatgatattgtgtacatattt
gcagttctgatatatggcttaggagtgctgatatttggcttatctattgcttgtccttta
gaaataatgtcttgttcaaatgctttctctactccattagaaattgtacctgagtggtat
ctcctactgtgctttaacttattgcgtattgtcacttccaaaggaataggtgttaaggaa
atggtgatcttagtcccaattattatttgcttgtgcattatagagaatgttagtgtatat
tctaatccttttagaagagctataatggtatgtgtaactttagtgtatagtatttttgca
atctggttaagtgttggctcactagttcgaatagataaggctttacctcttttgtaa
>Mz6_petD
atatgtgtagtcaaggtggcaaatcttaaaagttgtatgttgttggctaagttatcttgt
gggatgggacataacagttatggtgaagctgcatgaccaaatgatattgtgtacatattt
gcagttctgatatatggcttaggagtgctgatatttggcttatctattgcttgtccttta
gaaataatgtcttgttcaaatgctttctctactccattagaaattgtacctgagtggtat
ctcctactgtgctttaacttattgcgtattgtcacttccaaaggaataggtgttaaggaa
atggtgatcttagtcccaattattatttgcttgtgcattatagagaatgttagtgtatat
tctaatccttttagaagagctataatggtatgtgtaactttagtgtatagtatttttgca
atctggttaagtgttggctcactagttcgaatagataaggctttacctcttttgtaa
>Mz7_petD
atatgtgtagtcaaggtggcaaatcttaaaagttgtatgttgttggctaagttatcttgt
gggatgggacataacagttatggtgaagctgcatgaccaaatgatattgtgtacatattt
gcagttctgatatatggcttaggagtgctgatatttggcttatctattgcttgtccttta
gaaataatgtcttgttcaaatgctttctctactccattagaaattgtacctgagtggtat
ctcctactgtgctttaacttattgcgtattgtcacttccaaaggaataggtgttaaggaa
atggtgatcttagtcccaattattatttgcttgtgcattatagagaatgttagtgtatat
tctaatccttttagaagagctataatggtatgtgtaactttagtgtatagtatttttgca
atctggttaagtgttggctcactagttcgaatagataaggctttacctcttttgtaa
>Mz8_petD
atatgtgtagtcaaggtggcaaatcttaaaagttgtatgttgttggctaagttatcttgt
gggatgggacataacagttatggtgaagctgcatgaccaaatgatattgtgtacatattt
gcagttctgatatatggcttaggagtgctgatacttggcttatctattgcttgtccttta
gaaataatgtcttgttcaaatgctttctctactccattagaaattgtacctgagtggtat
ctcctactgtgctttaacttattgcgtattgtcacttccaaaggaataggtgttaaggaa
atggtgatcttagtcccaattattatttgcttgtgcattatagagaatgttagtgtatat
tctaatccttttagaagagctataatggtatgtgtaactttagtgtatagtatttttgca
atctggttaagtgttggctcactagttcgaatagataaggctttacctcttttgtaa
>Mz9_petD
atatgtgtagtcaaggtggcaaatcttaaaagttgtatgttgttggctaagttatcttgt
gggatgggacataacagttatggtgaagctgcatgaccaaatgatattgtgtacatattt
gcagttctgatatatggcttaggagtgttgatatttggcttatctattgcttgtccttta
gaaataatgtcttgttcaaatgctttctctactccattagaaattgtacctgagtggtat
ctcctactgtgctttaacttattgcgtattgtcacttccaaaggaataggtgttaaggaa
atggtgatcttagtcccaattattgtttgcttgtgcattatagagaatgttagtgtatat
tctaatccttttagaagagctataatggtatgtgtaactttagtgtatagtatttttgca
atctggttaagtgttggctcactagttcgaatagataaggctttacctcttttgtaa

>MzC1_petD
atatgtgtagtcaaggtggcaaatcttaaaagttgtatgttgttggctaagttatcttgt
gggatgggacataacagttatggtgaagctgcatgaccaaatgatattgtgtacatattt
gcagttctgatatatggcttaggagtgctgatatttggcttatctattgcttgtccttta
gaaataatgtcttgttcaaatgctttctctactccattagaaattgtacctgagtggtat
ctcctactgtgctttaacttattgcgtattgtcacttccaaaggaataggtgttaaggaa
atggtgatcttagtcccaattattatttgcttgtgcattatagagaatgttagtgtatat
tctaatccttttagaagagctataatggtatgtgtaactttagtgtatagtatttttgca
atctggttaagtgttggctcactagttcgaatagataaggctttacctcttttgtaa
>MzC1GaII_petD
atatgtgtagtcaaggtggcaaatcttaaaagttgtatgttgttggctaagttatcttgt
gggatgggacataacagttatggtgaagctgcatgaccaaatgatattgtgtacatattt
gcagttctgatatatggcttaggagtgctgatatttggcttatctattgcttgtccttta
gaaataatgtcttgttcaaatgctttctctactccattagaaattgtacctgagtggtat
ctcctactgtgctttaacttattgcgtattgtcacttccaaaggaataggtgttaaggaa
atggtgatcttagtcccaattattatttgcttgtgcattatagagaatgttagtgtatat
tctaatccttttagaagagctataatggtatgtgtaactttagtgtatagtatttttgca
atctggttaagtgttggctcactagttcgaatagataaggctttacctcttttgtaa
>MzC2_petD
atatgtgtagtcaaggtggcaaatcttaaaagttgtatgttgttggctaagttatcttgt
gggatgggacataacagttatggtgaagctgcatgaccaaatgatattgtgtacatattt
gcagttctgatatatggcttaggagtgctgatatttggcttatctattgcttgtccttta
gaaataatgtcttgttcaaatgctttctctactccattagaaattgtacctgagtggtat
ctcctactgtgctttaacttattgcgtattgtcacttccaaaggaataggtgttaaggaa
atggtgatcttagtcccaattattatttgcttgtgcattatagagaatgttagtgtatat
tctaatccttttagaagagctataatggtatgtgtaactttagtgtatagtatttttgca
atctggttaagtgttggctcactagttcgaatagataaggctttacctcttttgtaa
>MzC2GaII_petD
atatgtgtagtcaaggtggcaaatcttaaaagttgtatgttgttggctaagttatcttgt
gggatgggacataacagttatggtgaagctgcatgaccaaatgatattgtgtacatattt
gcagttctgatatatggcttaggagtgctgatatttggcttatctattgcttgtccttta
gaaataatgtcttgttcaaatgctttctctactccattagaaattgtacctgagtggtat
ctcctactgtgctttaacttattgcgtattgtcacttccaaaggaataggtgttaaggaa
atggtgatcttagtcccaattattatttgcttgtgcattatagagaatgttagtgtatat
tctaatccttttagaagagctataatggtatgtgtaactttagtgtatagtatttttgca
atctggttaagtgttggctcactagttcgaatagataaggctttacctcttttgtaa
>MzC3_petD
atatgtgtagtcaaggtggcaaatcttaaaagttgtatgttgttggctaagttatcttgt
gggatgggacataacagttatggtgaagctgcatgaccaaatgatattgtgtacatattt
gcagttctgatatatggcttaggagtgctgatatttggcttatctattgcttgtccttta
gaaataatgtcttgttcaaatgctttctctactccattagaaattgtacctgagtggtat
ctcctactgtgctttaacttattgcgtattgtcacttccaaaggaataggtgttaaggaa
atggtgatcttagtcccaattattatttgcttgtgcattatagagaatgttagtgtatat
tctaatccttttagaagagctataatggtatgtgtaactttagtgtatagtatttttgca
atctggttaagtgttggctcactagttcgaatagataaggctttacctcttttgtaa
>MzC4_petD
atatgtgtagtcaaggtggcaaatcttaaaagttgtatgttgttggctaagttatcttgt
gggatgggacataacagttatggtgaagctgcatgaccaaatgatattgtgtacatattt
gcagttctgatatatggcttaggagtgctgatatttggcttatctattgcttgtccttta
gaaataatgtcttgttcaaatgctttctctactccattagaaattgtacctgagtggtat
ctcctactgtgctttaacttattgcgtattgtcacttccaaaggaataggtgttaaggaa
atggtgatcttagtcccaattattatttgcttgtgcattatagagaatgttagtgtatat
tctaatccttttagaagagctataatggtatgtgtaactttagtgtatagtatttttgca
atctggttaagtgttggctcactagttcgaatagataaggctttacctcttttgtaa
>Od10_petD
atatgtgtagtcaaggtggcaaatcttaaaagttgtatgttgttggctaagttatcttgt
gggatgggacataacagttatggtgaagctgcatgaccaaatgatattgtgtacatattt

gcagttctgatatatggcttaggagtgctgatatttggcttatctattgcttgtccttta
gaaataatgtcttgttcaaatgctttctctactccattagaaattgtacctgagtggtat
ctcctactgtgctttaacttattgcgtattgtcacttccaaaggaataggtgttaaggaa
atggtgatcttagtcccaattattatttgcttgtgcattatagagaatgttagtgtatat
tctaatccttttagaagagctataatggtatgtgtaactttagtgtatagtatttttgca
atctggttaagtgttggctcactagttcgaatagataaggctttacctcttttgtaa
>Od11_petD
atatgtgtagtcaaggtggcaaatcttaaaagttgtatgttgttggctaagttatcttgt
gggatgggacataacagttatggtgaagctgcatgaccaaatgatattgtgtacatattt
gcagttctgatatatggcttaggagtgctgatatttggcttatctattgcttgtccttta
gaaataatgtcttgttcaaatgctttctctactccattagaaattgtacctgagtggtat
ctcctactgtgctttaacttattgcgtattgtcacttccaaaggaataggtgttaaggaa
atggtgatcttagtcccaattattatttgcttgtgcattatagagaatgttagtgtatat
tctaatccttttagaagagctataatggtatgtgtaactttagtgtatagtatttttgca
atctggttaagtgttggctcactagttcgaatagataaggctttacctcttttgtaa
>Od12_petD
atatgtgtagtcaaggtggcaaatcttaaaagttgtatgttgttggctaagttatcttgt
gggatgggacataacagttatggtgaagctgcatgaccaaatgatattgtgtacatattt
gcagttctgatatatggcttaggagtgctgatatttggcttatctattgcttgtccttta
gaaataatgtcttgttcaaatgctttctctactccattagaaattgtacctgagtggtat
ctcctactgtgctttaacttattgcgtattgtcacttccaaaggaataggtgttaaggaa
atggtgatcttagtcccaattattatttgcttgtgcattatagagaatgttagtgtatat
tctaatccttttagaagagctataatggtatgtgtaactttagtgtatagtatttttgca
atctggttaagtgttggctcactagttcgaatagataaggctttacctcttttgtaa
>Od13_petD
atatgtgtagtcaaggtggcaaatcttaaaagttgtatgttgttggctaagttatcttgt
gggatgggacataacagttatggtgaagctgcatgaccaaatgatattgtgtacatattt
gcagttctgatatatggcttaggagtgctgatatttggcttatctattgcttgtccttta
gaaataatgtcttgttcaaatgctttctctactccattagaaattgtacctgagtggtat
ctcctactgtgctttaacttattgcgtattgtcacttccaaaggaataggtgttaaggaa
atggtgatcttagtcccaattattatttgcttgtgcattatagagaatgttagtgtatat
tctaatccttttagaagagctataatggtatgtgtaactttagtgtatagtatttttgca
atctggttaagtgttggctcactagttcgaatagataaggctttacctcttttgtaa
>Od14_petD
atatgtgtagtcaaggtggcaaatcttaaaagttgtatgttgttggctaagttatcttgt
gggatgggacataacagttatggtgaagctgcatgaccaaatgatattgtgtacatattt
gcagttctgatatatggcttaggagtgctgatatttggcttatctattgcttgtccttta
gaaataatgtcttgttcaaatgctttctctactccattagaaattgtacctgagtggtat
ctcctactgtgctttaacttattgcgtattgtcacttccaaaggaataggtgttaaggaa
atggtgatcttagtcccaattattatttgcttgtgcattatagagaatgttagtgtatat
tctaatccttttagaagagctataatggtatgtgtaactttagtgtatagtatttttgca
atctggttaagtgttggctcactagttcgaatagataaggctttacctcttttgtaa
>Od15_petD
atatgtgtagtcaaggtggcaaatcttaaaagttgtatgttgttggctaagttatcttgt
gggatgggacataacagttatggtgaagctgcatgaccaaatgatattgtgtacatattt
gcagttctgatatatggcttaggagtgctgatatttggcttatctattgcttgtccttta
gaaataatgtcttgttcaaatgctttctctactccattagaaattgtacctgagtggtat
ctcctactgtgctttaacttattgcgtattgtcacttccaaaggaataggtgttaaggaa
atggtgatcttagtcccaattattatttgcttgtgcattatagagaatgttagtgtatat
tctaatccttttagaagagctataatggtatgtgtaactttagtgtatagtatttttgca
atctggttaagtgttggctcactagttcgaatagataaggctttacctcttttgtaa
>Od18_petD
atatgtgtagtcaaggtggcaaatcttaaaagttgtatgttgttggctaagttatcttgt
gggatgggacataacagttatggtgaagctgcatgaccaaatgatattgtgtacatattt
gcagttctgatatatggcttaggagtgctgatatttggcttatctattgcttgtccttta
gaaataatgtcttgttcaaatgctttctctactccattagaaattgtacctgagtggtat
ctcctactgtgctttaacttattgcgtattgtcacttccaaaggaataggtgttaaggaa

atggtgatcttagtcccaattattatttgcttgtgcattatagagaatgttagtgtatat
tctaatccttttagaagagctataatggtatgtgtaactttagtgtatagtatttttgca
atctggttaagtgttggctcactagttcgaatagataaggctttacctcttttgtaa
>Od19_petD
atatgtgtagtcaaggtggcaaatcttaaaagttgtatgttgttggctaagttatcttgt
gggatgggacataacagttatggtgaagctgcatgaccaaatgatattgtgtacatattt
gcagttctgatatatggcttaggagtgctgatatttggcttatctattgcttgtccttta
gaaataatgtcttgttcaaatgctttctctactccattagaaattgtacctgagtggtat
ctcctactgtgctttaacttattgcgtattgtcacttccaaaggaataggtgttaaggaa
atggtgatcttagtcccaattattatttgcttgtgcattatagagaatgttagtgtatat
tctaatccttttagaagagctataatggtatgtgtaactttagtgtatagtatttttgca
atctggttaagtgttggctcactagttcgaatagataaggctttacctcttttgtaa
>Od2_petD
atatgtgtagtcaaggtggcaaatcttaaaagttgtatgttgttggctaagttatcttgt
gggatgggacataacagttatggtgaagctgcatgaccaaatgatattgtgtacatattt
gcagttctgatatatggcttaggagtgctgatatttggcttatctattgcttgtccttta
gaaataatgtcttgttcaaatgctttctctactccattagaaattgtacctgagtggtat
ctcctactgtgctttaacttattgcgtattgtcacttccaaaggaataggtgttaaggaa
atggtgatcttagtcccaattattatttgcttgtgcattatagagaatgttagtgtatat
tctaatccttttagaagagctataatggtatgtgtaactttagtgtatagtatttttgca
atctggttaagtgttggctcactagttcgaatagataaggctttacctcttttgtaa
>Od22_petD
atatgtgtagtcaaggtggcaaatcttaaaagttgtatgttgttggctaagttatcttgt
gggatgggacataacagttatggtgaagctgcatgaccaaatgatattgtgtacatattt
gcagttctgatatatggcttaggagtgctgatatttggcttatctattgcttgtccttta
gtaataatgtcttgttcaaatgctttctctactccattagaaattgtacctgagtggtat
ctcctactgtgctttaacttattgcgtattgtcacttccaaaggaataggtgttaaggaa
atggtgatcttagtcccaattattatttgcttgtgcattatagagaatgttagtgtatat
tctaatccttttagaagagctataatggtatgtgtaactttagtgtatagtatttttgca
atctggttaagtgttggctcactagttcgaatagataaggctttacctcttttgtaa
>Od23_petD
atatgtgtagtcaaggtggcaaatcttaaaagttgtatgttgttggctaagttatcttgt
gggatgggacataacagttatggtgaagctgcatgaccaaatgatattgtgtacatattt
gcagttctgatatatggcttaggagtgctgatatttggcttatctattgcttgtccttta
gaaataatgtcttgttcaaatgctttctctactccattagaaattgtacctgagtggtat
ctcctactgtgctttaacttattgcgtattgtcacttccaaaggaataggtgttaaggaa
atggtgatcttagtcccaattattatttgcttgtgcattatagagaatgttagtgtatat
tctaatccttttagaagagctataatggtatgtgtaactttagtgtatagtatttttgca
atctggttaagtgttggctcactagttcgaatagataaggctttacctcttttgtaa
>Od24_petD
atatgtgtagtcaaggtggcaaatcttaaaagttgtatgttgttggctaagttatcttgt
gggatgggacataacagttatggtgaagctgcatgaccaaatgatattgtgtacatattt
gcagttctgatatatggcttaggagtgctgatatttggcttatctattgcttgtccttta
gaaataatgtcttgttcaaatgctttctctactccattagaaattgtacctgagtggtat
ctcctactgtgctttaacttattgcgtattgtcacttccaaaggaataggtgttaaggaa
atggtgatcttagtcccaattattgtttgcttgtgcattatagagaatgttagtgtatat
tctaatccttttagaagagctataatggtatgtgtaactttagtgtatagtatttttgca
atctggttaagtgttggctcactagttcgaatagataaggctttacctcttttgtaa
>Od25_petD
atatgtgtagtcaaggtggcaaatcttaaaagttgtatgttgttggctaagttatcttgt
gggatgggacataacagttatggtgaagctgcatgaccaaatgatattgtgtacatattt
gcagttctgatatatggcttaggagtgctgatatttggcttatctattgcttgtccttta
gaaataatgtcttgttcaaatgctttctctactccattagaaattgtacctgagtggtat
ctcctactgtgctttaacttattgcgtattgtcacttccaaaggaataggtgttaaggaa
atggtgatcttagtcccaattattatttgcttgtgcattatagagaatgttagtgtatat
tctaatccttttagaagagctataatggtatgtgtaactttagtgtatagtatttttgca
atctggttaagtgttggctcactagttcgaatagataaggctttacctcttttgtaa

>Od26_petD
atatgtgtagtcaaggtggcaaatcttaaaagttgtatgttgttggctaagttatcttgt
gggatgggacataacagttatggtgaagctgcatgaccaaatgatattgtgtacatattt
gcagttctgatatatggcttaggagtgctgatatttggcttatctattgcttgtccttta
gaaataatgtcttgttcaaatgctttctctactccattagaaattgtacctgagtggtat
ctcctactgtgctttaacttattgcgtattgtcacttccaaaggaataggtgttaaggaa
atggtgatcttagtcccaattattatttgcttgtgcattatagagaatgttagtgtatat
tctaatccttttagaagagctataatggtatgtgtaactttagtgtatagtatttttgca
atctggttaagtgttggctcactagttcgaatagataaggctttacctcttttgtaa
>Od27_petD
atatgtgtagtcaaggtggcaaatcttaaaagttgtatgttgttggctaagttatcttgt
gggatgggacataacagttatggtgaagctgcatgaccaaatgatattgtgtacatattt
gcagttctgatatatggcttaggagtgctgatatttggcttatctattgcttgtccttta
gaaataatgtcttgttcaaatgctttctctactccattagaaattgtacctgagtggtat
ctcctactgtgctttaacttattgcgtattgtcacttccaaaggaataggtgttaaggaa
atggtgatcttagtcccaattattatttgcttgtgcattatagagaatgttagtgtatat
tctaatccttttagaagagctataatggtatgtgtaactttagtgtatagtatttttgca
atctggttaagtgttggctcactagttcgaatagataaggctttacctcttttgtaa
>Od4_petD
atatgtgtagtcaaggtggcaaatcttaaaagttgtatgttgttggctaagttatcttgt
gggatgggacataacagttatggtgaagctgcatgaccaaatgatattgtgtacatattt
gcagttctgatatatggcttaggagtgctgatatttggcttatctattgcttgtccttta
gaaataatgtcttgttcaaatgctttctctactccattagaaattgtacctgagtggtat
ctcctactgtgctttaacttattgcgtattgtcacttccaaaggaataggtgttaaggaa
atggtgatcttagtcccaattattatttgcttgtgcattatagagaatgttagtgtatat
tctaatccttttagaagagctataatggtatgtgtaactttagtgtatagtatttttgca
atctggttaagtgttggctcactagttcgaatagataaggctttacctcttttgtaa
>Od6_petD
atatgtgtagtcaaggtggcaaatcttaaaagttgtatgttgttggctaagttatcttgt
gggatgggacataacagttatggtgaagctgcatgaccaaatgatattgtgtacatattt
gcagttctgatatatggcttaggagtgctgatatttggcttatctattgcttgtccttta
gaaataatgtcttgttcaaatgctttctctactccattagaaattgtacctgagtggtat
ctcctactgtgctttaacttattgcgtattgtcacttccaaaggaataggtgttaaggaa
atggtgatcttagtcccaattattatttgcttgtgcattatagagaatgttagtgtatat
tctaatccttttagaagagctataatggtatgtgtaactttagtgtatagtatttttgca
atctggttaagtgttggctcactagttcgaatagataaggctttacctcttttgtaa
>Od8_petD
atatgtgtagtcaaggtggcaaatcttaaaagttgtatgttgttggctaagttatcttgt
gggatgggacataacagttatggtgaagctgcatgaccaaatgatattgtgtacatattt
gcagttctgatatatggcttaggagtgctgatatttggcttatctattgcttgtccttta
gaaataatgtcttgttcaaatgctttctctactccattagaaattgtacctgagtggtat
ctcctactgtgctttaacttattgcgtattgtcacttccaaaggaataggtgttaaggaa
atggtgatcttagtcccaattattatttgcttgtgcattatagagaatgttagtgtatat
tctaatccttttagaagagctataatggtatgtgtaactttagtgtatagtatttttgca
atctggttaagtgttggctcactagttcgaatagataaggctttacctcttttgtaa
>Od9_petD
atatgtgtagtcaaggtggcaaatcttaaaagttgtatgttgttggctaagttatcttgt
gggatgggacataacagttatggtgaagctgcatgaccaaatgatattgtgtacatattt
gcagttctgatatatggcttaggagtgctgatatttggcttatctattgcttgtccttta
gaaataatgtcttgttcaaatgctttctctactccattagaaattgtacctgagtggtat
ctcctactgtgctttaacttattgcgtattgtcacttccaaaggaataggtgttaaggaa
atggtgatcttagtcccaattattatttgcttgtgcattatagagaatgttagtgtatat
tctaatccttttagaagagctataatggtatgtgtaactttagtgtatagtatttttgca
atctggttaagtgttggctcactagttcgaatagataaggctttacctcttttgtaa
>Ss5_petD
atatgtgtagtcaaggtggcaaatcttaaaagttgtatgttgttggctaagttatcttgt
gggatgggacataacagttatggtgaagctgcatgaccaaatgatattgtgtacatattt

gcagttctgatatatggcttaggagtgctgatatttggcttatctattgcttgtccttta
gaaataatgtcttgttcaaatgctttctctactccattagaaattgtacctgagtggtat
ctcctactgtgctttaacttattgcgtattgtcacttccaaaggaataggtgttaaggaa
atggtgatcttagtcccaattattatttgcttgtgcattatagagaatgttagtgtatat
tctaatccttttagaagagctataatggtatgtgtaactttagtgtatagtatttttgca
atctggttaagtgttggctcactagttcgaatagataaggctttacctcttttgtaa
>Ss6_petD
atatgtgtagtcaaggtggcaaatcttaaaagttgtatgttgttggctaagttatcttgt
gggatgggacataacagttatggtgaagctgcatgaccaaatgatattgtgtacatattt
gcagttctgatatatggcttaggagtgctgatatttggcttatctattgcttgtccttta
gaaataatgtcttgttcaaatgctttctctactccattagaaattgtacctgagtggtat
ctcctactgtgctttaacttattgcgtattgtcacttccaaaggaataggtgttaaggaa
atggtgatcttagtcccaattattatttgcttgtgcattatagagaatgttagtgtatat
tctaatccttttagaagagctataatggtatgtgtaactttagtgtatagtatttttgca
atctggttaagtgttggctcactagttcgaatagataaggctttacctcttttgtaa
>Ss7_petD
atatgtgtagtcaaggtggcaaatcttaaaagttgtatgttgttggctaagttatcttgt
gggatgggacataacagttatggtgaagctgcatgaccaaatgatattgtgtacatattt
gcagttctgatatatggcttaggagtgctgatatttggcttatctattgcttgtccttta
gaaataatgtcttgttcaaatgctttctctactccattagaaattgtacctgagtggtat
ctcctactgtgctttaacttattgcgtattgtcacttccaaaggaataggtgttaaggaa
atggtgatcttagtcccaattattatttgcttgtgcattatagagaatgttagtgtatat
tctaatccttttagaagagctataatggtatgtgtaactttagtgtatagtatttttgca
atctggttaagtgttggctcactagttcgaatagataaggctttacctcttttgtaa
>Ss8_petD
atatgtgtagtcaaggtggcaaatcttaaaagttgtatgttgttggctaagttatcttgt
gggatgggacataacagttatggtgaagctgcatgaccaaatgatattgtgtacatattt
gcagttctgatatatggcttaggagtgctgatatttggcttatctattgcttgtccttta
gaaataatgtcttgttcaaatgctttctctactccattagaaattgtacctgagtggtat
ctcctactgtgctttaacttattgcgtattgtcacttccaaaggaataggtgttaaggaa
atggtgatcttagtcccaattattatttgcttgtgcattatagagaatgttagtgtatat
tctaatccttttagaagagctataatggtatgtgtaactttagtgtatagtatttttgca
atctggttaagtgttggctcactagttcgaatagataaggctttacctcttttgtaa
>ohdo1_petD
atatgtgtagtcaaggtggcaactcttaaaagttgtatgttgttggctaagttatcttgt
gggatggtacataacagttatggtgaagctgcatgaccaaatgatattgtgtacatattt
gcagttctgatatatggcttaggagtgctgatatttggcttatctattgcttgtccttta
gaaataatgtcttgttcaaatgctttctctactccattagaaattgtacctgagtggtat
ctcctactgtgctttaacttattgcgtattgtcacttccaaaggaataggtgttaaggaa
atggtgatcttagtcccaattattatttgcttgtgcattatagagaatgttagtgtatat
tctaatccttttagaagagctataatggtatgtgtaactttagtgtatagtatttttgca
atctggttaagtgttggctcactagttcgaatagataaggctttacctcttttgtaa
>ohdo3_petD
atatgtgtagtcaaggtggcaaatcttaaaagttgtatgttgttggctaagttatcttgt
gggatgggacataacagttatggtgaagctgcatgaccaaatgatattgtgtacatattt
gcagttctgatatatggcttaggagtgctgatatttggcttatctattgcttgtccttta
gaaataatgtcttgttcaaatgctttctctactccattagaaattgtacctgagtggtat
ctcctactgtgctttaacttattgcgtattgtcacttccaaaggaataggtgttaaggaa
atggtgatcttagtcccaattattatttgcttgtgcattatagagaatgttagtgtatat
tctaatccttttagaagagctataatggtatgtgtaactttagtgtatagtatttttgca
atctggttaagtgttggctcactagttcgaatagataaggctttacctcttttgtaa
>ohdo5_petD
atatgtgtagtcaaggtggcaaatcttaaaagttgtatgttgttggctaagttatcttgt
gggatgggacataacagttatggtgaagctgcatgaccaaatgatattgtgtacatattt
gcagttctgatatatggcttaggagtgctgatatttggcttatctattgcttgtccttta
gaaataatgtcttgttcaaatgctttctctactccattagaaattgtacctgagtggtat
ctcctactgtgctttaacttattgcgtattgtcacttccaaaggaataggtgttaaggaa

atggtgatcttagtcccaattattatttgcttgtgcattatagagaatgttagtgtatat
tctaatccttttagaagagctataatggtatgtgtaactttagtgtatagtatttttgca
atctggttaagtgttggctcactagttcgaatagataaggctttacctcttttgtaa
>ohdo7_petD
atatgtgtagtcaaggtggcaaatcttaaaagttgtatgttgttggctaagttatcttgt
gggatgggacataacagttatggtgaagctgcatgaccaaatgatattgtgtacatattt
gcagttctgatatatggcttaggagtgctgatatttggcttatctattgcttgtccttta
gaaataatgtcttgttcaaatgctttctctactccattagaaattgtacctgagtggtat
ctcctactgtgctttaacttattgcgtattgtcacttccaaaggaataggtgttaaggaa
atggtgatcttagtcccaattattatttgcttgtgcattatagagaatgttagtgtatat
tctaatccttttagaagagctataatggtatgtgtaactttagtgtatagtatttttgca
atctggttaagtgttggctcactagttcgaatagataaggctttacctcttttgtaa
>sesoko1_petD
atatgtgtagtcaaggtggcaaatcttaaaagttgtatgttgttggctaagttatcttgt
gggatgggacataacagttatggtgaagctgcatgaccaaatgatattgtgtacatattt
gcagttctgatatatggcttaggagtgctgatatttggcttatctattgcttgtccttta
gaaataatgtcttgttcaaatgctttctctactccattagaaattgtacctgagtggtat
ctcctactgtgctttaacttattgcgtattgtcacttccaaaggaataggtgttaaggaa
atggtgatcttagtcccaattattatttgcttgtgcattatagagaatgttagtgtatat
tctaatccttttagaagagctataatggtatgtgtaactttagtgtatagtatttttgca
atctggttaagtgttggctcactagttcgaatagataaggctttacctcttttgtaa
>sesoko3_petD
atatgtgtagtcaaggtggcaaatcttaaaagttgtatgttgttggctaagttatcttgt
gggatgggacataacagttatggtgaagctgcatgaccaaatgatattgtgtacatattt
gcagttctgatatatggcttaggagtgctgatatttggcttatctattgcttgtccttta
gaaataatgtcttgttcaaatgctttctctactccattagaaattgtacctgagtggtat
ctcctactgtgctttaacttattgcgtattgtcacttccaaaggaataggtgttaaggaa
atggtgatcttagtcccaattattatttgcttgtgcattatagagaatgttagtgtatat
tctaatccttttagaagagctataatggtatgtgtaactttagtgtatagtatttttgca
atctggttaagtgttggctcactagttcgaatagataaggctttacctcttttgtaa
>sesoko4_petD
atatgtgtagtcaaggtggcaaatcttaaaagttgtatgttgttggctaagttatcttgt
gggatgggacataacagttatggtgaagctgcatgaccaaatgatattgtgtacatattt
gcagttctgatatatggcttaggagtgctgatatttggcttatctattgcttgtccttta
gaaataatgtcttgttcaaatgctttctctactccattagaaattgtacctgagtggtat
ctcctactgtgctttaacttattgcgtattgtcacttccaaaggaataggtgttaaggaa
atggtgatcttagtcccaattattatttgcttgtgcattatagagaatgttagtgtatat
tctaatccttttagaagagctataatggtatgtgtaactttagtgtatagtatttttgca
atctggttaagtgttggctcactagttcgaatagataaggctttacctcttttgtaa
>REF_DNA_petD_JX094314
ATATGTGTAGTCAAGGTGGCAAATCTTAAAAGTTGTATGTTGTTGGCTAAGTTATCTTGT
GGGATGGGACATAACAGTTATGGTGAAGCTGCATGACCAAATGATATTGTGTACATATTT
GCAGTTCTGATATATGGCTTAGGAGTGCTGATACTTGGCTTATCTATTGCTTGTCCTTTA
GAAATAATGTCTTGTTCAAATGCTTTCTCTACTCCATTAGAAATTGTACCTGAGTGGTAT
CTCCTACTGTGCTTTAACTTATTGCGTATTGTCACTTCCAAAGGAATAGGTGTTAAGGAA
ATGGTGATCTTAGTCCCAATTATTGTTTGCTTGTGCATTATAGAGAATGTTAGTGTATAT
TCTAATCCTTTTAGAAGAGCTATAATGGTATGTGTAACTTTAGTGTATAGTATTTTTGCA
ATCTGGTTAAGTGTTGGCTCACTAGTTCGAATAGATAAGGCTTTACCTCTTTTGTAA
>REF_RNA_petD_symbB1.comp26_c0_seq1
atatctgtagtcaaggtggcaaatcttaaaagtcgtatgttgttggctaagttatcttct
gggatgggacataacagttatggtgaacctgcatggccaaatgatattgcgtacatattt
ccagttctgatatatggcttaggagtgctgatacttggcttatctgttgcttgtccttta
gaaatagtgtctccttcaaatgctttctctactccattagaaattctacctgagtggtat
ttcctaccgtgctttaacttattgcgtgttgtcacttccaaaggaatgggtgttgcagga
atggtgatcttagtcctaattgttatttccttgtgcattatagagaatgctagtgtatat
tctaatccttttagaagacctataatgatgtgtgtagctttaacgtatagtatttttgca
atctggttaggtgttggctcactagttcgaatagatgaggctttacctcttttgtaa

>Hd1_psaA
atgcacatcttcagatatatcaacacaacactgtggtccaaagcaggacattttaataaa
gctttatcaaaaggagctaagacaactacttggatatggaatctacatgattatgctcat
gattttgacattcaacaaagatccacaggtttaatagcaagaaaagtcttctcatccaat
ctagctcatctatctttggtattcttttggattagtggaatgcaccttcatggggcatat
ttgtctaattatgatatttggttaaaagatcccaaatccatcaccccatcatcacatcta
gcctactctttaattggtcaagatattcttaactcatatacctcagaatacttctcagga
atcactatcacatctggctttttccaactctaccgttctgagggtatcattacacagtcc
cagctgaaatatgcatgtgctacttccctaatagctacacttatctgcctttctggatct
tatctccacatgcagcttatgtccaaattcactagcttttacaagaagttccagtcctta
tcgcaagatcatctcataattatatttggctccagctccacatccctctccgctcatcag
atacacaaaatgcttccagctaaccctttactagattcaggtatctccaacccaagtatc
ctacaagtaatctccaacagtctcagtgaaaacctagcactgttttcaacaaatctaagc
tctacaggtaaattgttaaatccctcaacaagaagtgtattcctttctcaagttgcagca
catcataagacaacaggtgtagtctttatcacactagggcttatcagattcctcaccatg
tataactctcaatttagtatacttacatcttatatagattaccacattgtgctatctatt
aatttagcattaattgcatctttatcaataatagtagctgatcatctcacaagaacccca
atctatcctcacaactcaacctcataccctacaatcctttgcctctccatacatcatgca
tggttatccggtttcctcatcattggttcaggagctcatgcatcaatattcaacttgcga
gcatcaccaacatctgagataagacatcgagaccccatatactcacacctcatctgggta
tgtatagcaataggagtacactcattcagtctctactgtcataacgacactttagaagca
ttaggacgtccagaagatatctttcatgacaactctatccagttaaaagcaatatttgcc
aagcaatcctttctaagagcagagctccagccagatatcgagatgttagataaaaagatt
atacgtataacccaagaattgggaacagcagattttatagttcaccacattcatgcattt
acaatccatgttactctcttaattctttcaaagggtgtactttatgctagaaactctaga
tttgtatcagataaattggaacttggttttacttatccctgtgatggtccaggtagaggt
ggtacatgtcaaatatcaccttgggatcacttattctcagctgtattttggatgtataac
tgtttaaatgtagtaacattccactacttttggaagatgcaatcagatgtttggggcttt
gtatcgatccaaaagcacatatctcactacagccaaggtgattttagtgtcaactctatt
accatcaatggttggttgagaaacttattatggtccgaggcatcccaagtaatccaatcc
tatgctctttcatccatttgtccatatgggttcattttcctcatagctcatttcatctgg
gcatttagtttaatgttcctctttagtggtagagcatactggcaagaattaattgagtcc
attctattgtcacatcataaattaaagattatacctcatatccaaccaagggcattaagc
atttctcaaggaagagcagttggtttcattcattacactctaggaggtatcggctcaacc
tgggcatttattatttcaagactgctagtattaacttcttga
>Hd2_psaA
atgcacatcttcagatatatcaacacaacactgtggtccaaagcaggacattttaataaa
gctttatcaaaaggagctaagacaactacttggatatggaatctacatgattatgctcat
gattttgacattcaacaaagatccacaggtttaatagcaagaaaagtcttctcatccaat
ctagctcatctatctttggtattcttttggattagtggaatgcaccttcatggggcatat
ttgtctaattatgatatttggttaaaagatcccaaatccatcaccccatcatcacatcta
gcctactctttaattggtcaagatattcttaactcatatacctcagaatacttctcagga
atcactatcacatctggctttttccaactctaccgttctgagggtatcattacacagtcc
cagctgaaatatgcatgtgctacttccctaatagctacacttatctgcctttctggatct
tatctccacatgcagcttatgtccaaattcactagcttttacaagaagttccagtcctta
tcgcaagatcatctcataattatatttggctccagctccacatccctctccgctcatcag
atacacaaaatgcttccagctaaccctttactagattcaggtatctccaacccaagtatc
ctacaagtaatctccaacagtctcagtgaaaacctagcactgttttcaacaaatctaagc
tctacaggtaaattgttaaatccctcaacaagaagtgtattcctttctcaagttgcagca
catcataagacaacaggtgtagtctttatcacactagggcttatcagattcctcaccatg
tataactctcaatttagtatacttacatcttatatagattaccacattgtgctatctatt
aatttagcattaattgcatctttatcaataatagtagctgatcatctcacaagaacccca
atctatcctcacaactcaacctcataccctacaatcctttgcctctccatacatcatgca
tggttatccggtttcctcatcattggttcaggagctcatgcatcaatattcaacttgcga
gcatcaccaacatctgagataagacatcgagaccccatatactcacacctcatctgggta
tgtatagcaataggattacactcattcagtctctactgtcataacgacactttagaagca
ttaggacgtccagaagatatctttcatgacaactctatccagttaaaagcaatatttgcc

aagcaatcctttctaagagcagagctccagccagatatcgagatgttagataaaaagatt
atacgtataacccaagaattgggaacagcagattttatagttcaccacattcatgcattt
acaatccatgttactctcttaattctttcaaagggtgtactttatgctagaaactctaga
tttgtatcagataaattggaacttggttttacttatccctgtgatggtccaggtagaggt
ggtacatgtcaaatatcaccttgggatcacttattctcagctgtattttggatgtataac
tgtttaaatgtagtaacattccactacttttggaagatgcaatcagatgtttggggcttt
gtatcgatccaaaagcacatatctcactacagccaaggtgattttagtgtcaactctatt
accatcaatggttggttgagaaacttattatggtccgaggcatcccaagtaatccaatcc
tatgctctttcatccatttgtccatatgggttcattttcctcatagctcatttcatctgg
gcatttagtttaatgttcctctttagtggtagagcatactggcaagaattaattgagtcc
attctatggtcacatcataaattaaagattatacctcatatccaaccaagggcattaagc
atttctcaaggaagagcagttggtttcattcattacactctaggaggtatcggctcaacc
tgggcatttattatttcaagactgctagtattaacttcttga
>Hd3_psaA
atgcacatcttcagatatatcaacacaacactgtggtccaaagcaggacattttaataaa
gctttatcaaaaggagctaagacaactactcggatatggaatctacatgattatgctcat
gattttgacattcaacaaagatccacaggtttaatagcaagaaaagtcttctcatccaat
ctagctcatctatctttggtattcttttggattagtggaatgcaccttcatggggcatat
ttgtctaattatgatatttggttaaaagatcccaaatccatcaccccatcatcacatcta
gcctactctttaattggtcaagatattcttaactcatatacctcagaatacttctcagga
atcactatcacatctggctttttccaactctaccgttctgagggtatcattacacagtcc
cagctgaaatatgcatgtgctacttccctaatagctacacttatctgcctttctggatct
tatctccacatgcagcttatgtccaaattcactagcttttacaagaagttccagtcctta
tcgcaagatcatctcataattatatttggctccagctccacatccctctccgctcatcag
atacacaaaatgcttccagctaaccctttactagattcaggtatctccaagccaagtatc
ctacaagtaatctccaacagtctcagctatacactagcactgttttcaacaaatctaagc
tctacaggtaaattgttaaatccctcaacaagaagtgtattcctttctcaagttgcagca
catcataagacaacaggtgtagtctttatcacactagggcttatcagattcctcaccatg
tataactctcaatttagtatacttacatcttatatagattaccacattgtgctatctatt
aatttagcattaattgcatctttatcaataatagtagctgatcatctcacaagaacccca
atctatcctcacaagtcaacctcataccctacaatcctttgcctctccatacatcatgca
tggttatccggtttcctcatcattggttcaggagctcatgcatcaatattcaacttgctt
ggatcaccaacatctgagataagacatcgagaccccatatactcacacctcatctgggta
tgtatagcaataggattacactcattcagtctctactgtcataacgacactttagaagca
ttaggacgtccagaagatatctttcatgacaactctatccagttaaaagcaatatttgcc
aagcaatcctttctaagagcagagctccagccagatatcgagatgttagataaaaagatt
atacgtataacccaagaattgggaacagcagattttatagttcaccacattcatgcattt
acaatccatgttactctcttaattctttcaaagggtgtactttatgctagaaactctaga
tttgtatcagataaattggaacttggttttacttatccctgtgatggtccaggtagaggt
ggtacatgtcaaatatcaccttgggatcacttattctcagctgtattttggatgtataac
tgtttaaatgtagtaacattccactacttttggaagatgcaatcagatgtttggggcttt
gtatcgatccaaaagcacatatctcactacagccaaggtgattttagtgtcaactctatt
accatcaatggttggttgagaaacttattatggtccgaggcatcccaagtaatccaatcc
tatgctctttcatccatttgtccatatgggttcattttcctcataggtcatttcatctgg
gcatttagtttaatgttcctctttagtggtagagcatactggcaagaattaattgagtcc
attctatggtcacatcataaattaaagattatacctcatatccaaccaagggcattaagc
atttctcaaggaagagcagttggtttcattcattacactctaggaggtatcggctcaacc
tgggcatttattatttcaagactgctagtattaacttcttga
>Hd4_psaA
atgcacatcttcagatatatcaacacaacactgtggtccaaagcaggacattttaataaa
gctttatcaaaaggagctaagacaactacttggatatggaatctacatgattatgctcat
gattttgacattcaacaaagatccacaggtttaatagcaagaaaagtcttctcatccaat
ctagctcatctatctttggtattcttttggattagtggaatgcaccttcatggggcatat
ttgtctaattatgatatttggttaaaagatcccaaatccatcaccccatcatcacatcta
gcctactctttaattggtcaagatattcttaactcatatacctcagaatacttctcagga
atcactatcacatctggctttttccaactctaccgttctgagggtatcattacacagtcc
cagctgaaatatgcatgtgctacttccctaatagctacacttatctgcctttctggatct

tatctccacatgcagcttatgtccaaattcactagcttttacaagaagttccagtcctta
tcgcaagatcatctcataattatatttggctccagctccacatccctctccgctcatcag
atacacaaaatgcttccagctaacccttttactagattcaggtatctccaacccaagtatc
ctacaagtaatctccaacagtctcagtgaaaacctagcactgttttcaacaaatctaagc
tctacaggtaaattgttaaatccctcaacaagaagtgtattcctttctcaagttgcagca
catcataagacaacaggtgtagtctttatcacactagggcttatcagattcctcaccatg
tataactctcaatttagtatacttacatcttatatagattaccacattgtgctatctatt
aatttagcattaattgcatctttatcaataatagtagctgatcatctcacaagaacccca
atctatcctcacaactcaacctcataccctacaatcctttgcctctccatacatcatgca
tggttatccggtttcctcatcattggttcaggagctcatgcatcaatattcaacttgcga
gcatcaccaacatctgagataagacatcgagaccccatatactcacacctcatctgggta
tgtatagcaataggattacactcattcagtctctactgtcataacgacactttagaagca
ttaggacgtccagaagatatctttcatgacaactctatccagttaaaagcaatatttgcc
aagcaatcctttctaagagcagagctccagccagatatcgagatgttagataaaaagatt
atacgtataacccaagaattgggaacagcagattttatagttcaccacattcatgcattt
acaatccatgttactctcttaattctttcaaagggtgtactttatgctagaaactctaga
tttgtatcagataaattggaacttggttttacttatccctgtgatggtccaggtagaggt
ggtacatgtcaaatatcaccttgggatcacttattctcagctgtattttggatgtataac
tgtttaaatgtagtaacattccactacttttggaagatgcaatcagatgtttggggcttt
gtatcgatccaaaagcacatatctcactacagccaaggtgattttagtgtcaactctatt
accatcaatggttggttgagaaacttattatggtccgaggcatcccaagtaatccaatcc
tatgctctttcatccatttgtccatatgggttcattttcctcatagctcatttcatctgg
gcatttagtttaatgttcctctttagtggtagagcatactggcaagaattaattgagtcc
attctatggtcacatcataaattaaagattatacctcatatccaaccaagggcattaagc
atttctcaaggaagagcagttggtttcattcattacactctaggaggtatcggctcaacc
tgggcatttattatttcaagactgctagtattaacttcttga
>Hd5_psaA
atgcacatcttcagatatatcaacacaacactgtggtccaaagcaggacattttaataaa
gctttatcaaaaggagctaagacaactacttggatatggaatctacatgattatgctcat
gattttgacattcaacaaagatccacaggtttaatagcaagaaaagtcttctcatccaat
ctagctcatctatctttggtattcttttggattagtggaatgcaccttcatggggcatat
ttgtctaattatgatatttggttaaaagatcccaaatccatcaccccatcatcacatcta
gcctactctttaattggtcaagatattcttaactcatatacctcagaatacttctcagga
atcactatcacatctggctttttccaactctaccgttctgagggtatcattacacagtcc
cagctgaaatatgcatgtgctacttccctaatagctacacttatctgcctttctggatct
tatctccacatgcagcttatgtccaaattcactagcttttacaagaagttccagtcctta
tcgcaagatcatctcataattatatttggctccagctccacatccctctccgctcatcag
atacacaaaatgcttccagctaacccttttactagattcaggtatctccaacccaagtatc
ctacaagtaatctccaacagtctcagtgaaaacctagcactgttttcaacaaatctaagc
tctacaggtaaattgttaaatccctcaacaagaagtgtattcctttctcaagttgcagca
catcataagacaacaggtgtagtctttatcacactagggcttatcagattcctcaccatg
tataactctcaatttagtatacttacatcttatatagattaccacattgtgctatctatt
aatttagcattaattgcatctttatcaataatagtagctgatcatctcacaagaacccca
atctatcctcacaactcaacctcataccctacaatcctttgcctctccatacatcatgca
tggttatccggtttcctcatcattggttcaggagctcatgcatcaatattcaacttgctt
ggatcaccaacatctgagataagacatcgagaccccatatactcacacctcatctgggta
tgtatagcaataggattacactcattcagtctctactgtcataacgacactttagaagca
ttaggacgtccagaagatatctttcatgacaactctatccagttaaaagcaatatttgcc
aagcaatcctttctaagagcagagctccagccagatatcgagatgttagataaaaagatt
atacgtataacccaagaattgggaacagcagattttatagttcaccacattcatgcattt
acaatccatgttactctcttaattctttcaaagggtgtactttatgctagaaactctaga
tttgtatcagataaattggaacttggttttacttatccctgtgatggtccaggtagaggt
ggtacatgtcaaatatcaccttgggatcacttattctcagctgtattttggatgtataac
tgtttaaatgtagtaacattccactacttttggaagatgcaatcagatgtttggggcttt
gtatcgatccaaaagcacatatctcactacagccaaggtgattttagtgtcaactctatt
accatcaatggttggttgagaaacttattatggtccgaggcatcccaagtaatccaatcc
tatgctctttcatccatttgtccatatgggttcattttcctcatagctcatttcatctgg

gcatttagtttaatgttcctctttagtggtagagcatactggcaagaattaattgagtcc
attctatggtcacatcataaattaaagattatacctcatatccaaccaagggcattaagc
atttctcaaggaagagcagttggtttcattcattacactctaggaggtatcggctcaacc
tgggcatttattatttcaagactgctagtattaacttcttga
>Hd6_psaA
atgcacatcttcagatatatcaacacaacactgtggtccaaagcaggacattttaataaa
gctttatcaaaaggagctaagacaactacttggatatggaatctacatgattatgctcat
gattttgacattcaacaaagatccacaggtttaatagcaagaaaagtcttctcatccaat
ctagctcatctatctttggtattcttttggattagtggaatgcaccttcatggagcatat
ttgtctaattatgatatttggttaaaagatcccaaatccatcacccccatcatcacatcta
gcctactctttaattggtcaagatattcttaactcatatacctcagaatacttctcagga
atcactatcacatctggctttttccaactctaccgttctgagggtatcattacacagtcc
cagctcaaatatgcatgtgctacttccctaatagctacacttatctgcctttctggatct
tatctccacatgcagcttatgtccaaattcactagcttttacaagaagttccagtcctta
tcgcaagatcatctcataattatatttggctccagctccacatccctctccgctcatcag
atacacaaaatgcttccagctaaccctttactagattcaggtatctccaacccaagtatc
ctacaagtaatctccaacagtctcagctatacactagcactgttttcaacaaatctaagc
tctacaggtaaattgttaaatccctcaacaagaagtgtattcctttctcaagttgcagca
catcataagacaacaggtgtagtctttatcacactagggcttatcagattcctcaccatg
tataactctcaatttagtatacttacatcttatatagattaccacattgtgctatctatt
aatttagcattaattgcatctttatcaataatagtagctgatcatctcacaagaacccca
atctatcctcacaactcaacctcataccctacaatcctttgcctctccatacatcatgca
tggttatccggtttcctcatcattggttcaggagctcatgcatcaatattcaacttgcga
gcatcaccaacatctgagataagatatcgagaccccatatactcacacctcatctgggta
tgtatagcaataggattacactcattcagtctctactgtcataacgacactttagaagca
ttaggacgtccagaagatatctttcatgacaactctatccagttaaaagcaatatttgcc
aagcaatcctttctaagagcagagctccagccagatatcgagatgttagataaaaagatt
atacgtataacccaagaattgggaacagcagattttatagttcaccacattcatgcattt
acaatccatgttactctcttaattctttcaaagggtgtactttatgctagaaactctaga
tttgtatcagataaattggaacttggttttacttatccctgtgatgggccaggtagaggt
ggtacatgtcaaatatcaccttgggatcacttattctcagctgtattttggatgtataac
tgtttaaatgtagtaacattccactacttttggaagatgcaatcagatgtttggggcttt
gtatcgatccaaaagcacatatctcactacagccaaggtgattttagtgtcaactctatt
accatcaatggttggttgagaaacttattatggtccgaggcatcccaagtaatccaatcc
tatgctctttcatccatttgtccatatgggttcattttcctcatagctcatttcatctgg
gcatttagtttaatgttcctctttagtggtagagcatactggcaagaattaattgagtcc
attctatggtcacatcataaattaaagattatacctcatatccaaccaagggcattaagc
atttctcaaggaagagcagttggtttcattcattacactctaggaggtatcggctcaacc
tgggcatttattatttcaagactgctagtattaacttcttga
>Hd7_psaA
atgcacatcttcagatatatcaacacaacactgtggtccaaagcaggacattttaataaa
gctttatcaaaaggagctaagacaactacttggatatggaatctacatgattatgctcat
gattttgacattcaacaaagatccacaggtttaatagcaagaaaagtcttctcatccaat
ctagctcatctatctttggtattcttttggattagtggaatgcaccttcatggagcatat
ttgtctaattatgatatttggttaaaagatcccaaatccatcacgccatcatcacatcta
gcctactctttaattggtcaagatattcttaactcatatacctcagaatacttctcagga
atcactatcacatctggctttttccaactctaccgttctgagggtatcattacacagtcc
cagctcaaatatgcatgtgctacttccctaatagctacacttatctgcctttctggatct
tatctccacatgcagcttatgtccaaattcactagcttttacaagaagttccagtcctta
tcgcaagatcatctcataattatatttggctccagctccacatccctctccgctcatcag
atacacaaaatgcttccagctaaccctttactagattcaggtatctccaacccaagtatc
ctacaagtaatctccaacagtctcagcgaaacactagcactgttttcaacaaatctaagc
tctacaggtaaattgttaaatccctcaacaagaagtgtattcctttctcaagttgcagca
catcataagacaacaggtgtagtctttatcacactagggcttatcagattcctcaccatg
tataactctcaatttagtatacttacatcttatatagattaccacattgtgctatctatt
aatttagcattaattgcatctttatcaataatagtagctgatcatctcacaagaacccca
atctatcctcacaactcaacctcataccctacaatcctttgcctctccatacatcatgca

tggttatccggtttcctcatcattggttcaggagctcatgcatcaatattcaacttgcgt
ggatcaccaacatctgagataagacatcgagaccccatatactcacacctcatctgggta
tgtatagcaataggattacactcattcagtctctactgtcataacgacactttagaagca
ttaggacgtccagaagatatctttcatgacaactctatccagttaaaagcaatatttgcc
aagcaatcctttctaagagcagagctccagccagatatcgagatgttagataaaaagatt
atacgtataacccaagaattgggaacagcagattttatagttcaccacattcatgcattt
acaatccatgttactctcttaattctttcaaagggtgtactttatgctagaaactctaga
tttgtatcagataaattggaacttggttttacttatccctgtgatggtccaggtagaggt
ggtacatgtcaaatatcaccttgggatcacttattctcagctgtattttggatgtataac
tgtttaaatgtagtaacattccactacttttggaagatgcaatcagatgtttggggcttt
gtatcgatccaaaagcacatatctcactacagccaaggtgattttagtgtcaactctatt
accatcaatggttggttgagaaacttattatggtccgaggcatcccaagtaatccaatcc
tatgctctttcatccatttgtccatatgggttcattttcctcatagctcatttcatctgg
gcatttagtttaatgttcctctttagtggtagagcatactggcaagaattaattgagtcc
attctatggtcacatcataaattaaagattatacctcatatccaaccaagggcattaagc
atttctcaaggaagagcagttggtttcattcattacactctaggaggtatcggctcaacc
tgggcatttattatttcaagactgctagtattaacttcttga
>Hd8_psaA
atgcacatcttcagatatatcaacacaacactgtggtccaaagcaggacattttaataaa
gctttatcaaaaggagctaagacaactacttggatatggaatctacatgattatgctcat
gattttgacattcaacaaagatccacaggtttaatagcaagaaaagtcttctcatccaat
ctagctcatctatctttggtattcttttggattagtggaatgcaccttcatggggcatat
ttgtctaattatgatatttggttaaaagatcccaaatccatcaccccatcatcacatcta
gcctactctttaattggtcaagatattcttaactcatatacctcagaatacttctcagga
atcactatcacatctggctttttccaactctaccgttctgagggtatcattacacagtcc
cagctgaaatatgcatgtgctacttccctaatagctacacttatctgcctttctggatct
tatctccacatgcagcttatgtccaaattcactagcttttacaagaagttccagtcctta
tcgcaagatcatctcataattatatttggctccagctccacatccctctccgctcatcag
atacacaaaatgcttccagctaaccctttactagattcaggtatctccaacccaagtatc
ctacaagtaatctccaacagtctcagtgaaaacctagcactgttttcaacaaatctaagc
tctacaggtaaattgttaaatccctcaacaagaagtgtattcctttctcaagttgcagca
catcataagacaacaggtgtagtctttatcacactagggcttatcagattcctcaccatg
tataactctcaatttagtatacttacatcttatatagattaccacattgtgctatctatt
aatttagcattaattgcatctttatcaataatagtagctgatcatctcacaagaacccca
atctatcctcacaactcaacctcataccctacaatcctttgcctctccatacatcatgca
tggttatccggtttcctcatcattggttcaggagctcatgcatcaatattcaacttgcga
gcatcaccaacatctgagataagacatcgagaccccatatactcacacctcatctgggta
tgtatagcaataggattacactcattcagtctctactgtcataacgacactttagaagca
ttaggacgtccagaagatatctttcatgacaactctatccagttaaaagcaatatttgcc
aagcaatcctttctaagagcagagctccagccagatatcgagatgttagataaaaagatt
atacgtataacccaagaattgggaacagcagattttatagttcaccacattcatgcattt
acaatccatgttactctcttaattctttcaaagggtgtactttatgctagaaactctaga
tttgtatcagataaattggaacttggttttacttatccctgtgatgggccaggtagaggt
ggtacatgtcaaatatcaccttgggatcacttattctcagctgtattttggatgtataac
tgtttaaatgtagtaacattccactacttttggaagatgcaatcagatgtttggggcttt
gtatcgatccaaaagcacatatctcactacagccaaggtgattttagtgtcaactctatt
accatcaatggttagttgagaaacttattatggtccgaggcatcccaagtaatccaatcc
tatgctctttcatccatttgtccatatgggttcattttcctcatagctcatttcatctgg
gcatttagtttaatgttcctctttagtggtagagcatactggcaagaattaattgagtcc
attctatggtcacatcataaattaaagattatacctcatatccaaccaagggcattaagc
atttctcaaggaagagcagttggtttcattcattacactctaggaggtatcggctcaacc
tgggcatttattatttcaagactgctagtattaacttcttga
>Hd9_psaA
atgcacatcttcagatatatcaacacaacactgtggtccaaagcaggacattttaataaa
gctttatcaaaaggagctaagacaactacttggatatggaatctacatgattatgctcat
gattttgacattcaacaaagatccacaggtttaatagcaagaaaagtcttctcatccaat
ctagctcatctatctttggtattcttttggattagtggaatgcaccttcatggagcatat

ttgtctaattatgatatttggttaaaagatcccaaatccatcacgccatcatcacatcta
gcctactctttaattggtcaagatattcttaactcatatacctcagaatacttctcagga
atcactatcacatctggctttttccaactctaccgttctgagggtatcattacacagtcc
cagctgaaatatgcatgtgctacttccctaatagctacacttatctgcctttctggatct
tatctccacatgcagcttatgtccaaattcactagcttttacaagaagttccagtcctta
tcgcaagatcatctcataattatatttggctccaggtccacatccctctccgctcatcag
atacacaaaatgcttccagctaaccctttactagattcaggtatctccaagccaagtatc
ctacaagtaatctccaacagtctcagtgaaaacctagcactgttttcaacaaatctaagc
tctacaggtaaattgttaaatccctcaacaagaagtgtattcctttctcaagttgcagca
catcataagacaacaggtgtagtctttatcacactagggcttatcagattcctcaccatg
tataactctcaatttagtatacttacatcttatatagattaccacattgtgctatctatt
aatttagcattaattgcatctttatcaataatagtagctgatcatctcacaagaacccca
atctatcctcacaactcaacctcataccctacaatcctttgcctctccatacatcatgca
tggttatccggtttcctcatcattggttcaggagctcatgcatcaatattcaacttgctt
ggatcaccaacatctgagataagacatcgagaccccatatactcacacctcatctgggta
tgtatagcaataggattacactcattcagtctctactgtcataacgacactttagaagca
ttaggacgtccagaagatatctttcatgacaactctatccagttaaaagcaatatttgcc
aagcaatcctttctaagagcagagctccagccagatatcgagatgttagataaaaagatt
atacgtataacccaagaattgggaacagcagattttatagttcaccacattcatgcattt
acaatccatgttactctcttaattctttcaaagggtgtactttatgctagaaactctaga
tttgtatcagataaattggaacttggttttacttatccctgtgatgggccaggtagaggt
ggtacatgtcaaatatcaccttgggatcacttattctcagctgtattttggatgtataac
tgtttaaatgtagtaacattccactacttttggaagatgcaatcagatgtttggggcttt
gtatcgatccaaaagcacatatctcactacagccaaggtgattttagtgtcaactctatt
accatcaatggttggttgagaaacttattatggtccgaggcatcccaagtaatccaatcc
tatgctctttcatccatttgtccatatgggttcattttcctcatagctcatttcatctgg
gcatttagtttaatgttcctctttagtggtagagcatactggcaagaattaattgagtcc
attctatggtcacatcataaattaaagattatacctcatatccaaccaagggcattaagc
atttctcaaggaagagcagttggtttcattcattacactctaggaggtatcggctcaacc
tgggcatttattatttcaagactgctagtattaacttcttga
>IS1_psaA
atgcacatcttcagatatatcaatactacactgtggtccaaagcaggacactttaataaa
gctttatcaaaaggagctaagacaactacttggatatggaatctacatgattatgctcat
gattttgacattcaacaaagatccacaggtttaatagcaagaaaagtcttctcatccaat
ctagctcatctatctttggtattcttttggattagtggaatgcaccttcatggagcatat
ttgtctaattatgatatttggttaaaagatcccaaatccatcacgccatcatcacatcta
gcctactctttagttggccaagatattcttaactcatatacctcagaatacttctcagga
atcactatcacatctggctttttccaactctaccgttctgagggtatcattacacagtct
cagctgaaatatgcatgtgctacttccctaatagctacacttatctgcctttctggatct
tatctccacatgcaagtgatgtccaaatttagtagcttttataagaagttccagtcctta
tcgcaagatcatctcataattatatttggctccaggtccacatccctctccgctcatcag
atacacaaaatgcttccagctaactatcaactagattcaggcatctccaatccaagtatc
ctacaagtaatctccaacagtctcagctatacactagcactgttttcaacaaatctaagc
tctacaggtaaattgttaaatccctcaacaagaagtgtattcctttctcaagttgcagca
catcataagacaataggtatagtacttatcacactagggcttatcagattcctcaccatt
tataattctcaatttagtatacttacatcttatatagattaccacattgtgctatctatt
aatttagcattaattgcatctttatcaataatagtagctgatcatctcacaagaacccca
atctatcctcacaagtcaacctcataccctacaatcctttgcctctccatacatcatgca
tggttatccggtttcctcatcattggttcaggagctcatgcatcaatattcaacttgctt
ggatcaccaacatctgagataagacatcgagaccccatatactcacacctcatctgggta
tgtatagctttaggattacactcatttagtctctactgccataacgacactttagaagct
ttaggacgtccagaagatatctttcatgacaactctatccagttaaaagcaatatttgcc
aagcaatcctttctaagagcagagctccagccagatatcgagatcttagataaaaagatt
atacgtataactcaagaattgggaacagctgattttatagttcaccacattcatgcattt
acaatccatgttactctcttaattctttcaaagggtgtactttatgctagaaactctaga
tttgtatcagataaattggaacttggttttacttatccttgtgatgggccaggtagaggt
ggtacatgtcaaatatcaccttgggatcacttattctcagctgtattttggatgtataac

tgtttaaatgtagtaacattccactacttttggaagatgcaatcagatgtttggggcttt
gtatccatccaaaggcaaatttcccactacagccaaggtgattttagtgtaaattctatt
accatcaatggctggttgagaaacttattatggtccgaggcatcccaagtaatccaatcc
tatgctctttcatccatttgtccatatgggttcattttcctcataggtcatttcatctgg
gcatttagtttaatgttcctctttagtggtagagcatactggcaagaattaattgagtcc
attctatggtcacatcataaattaaagattatacctcatatccaaccaagggcattaagc
atttctcaaggaagagcagttggtttcattcattacactctaggaggtatcggctcaacc
tgggcatttattatttcaagactgctagtattaacttcttga
>IS2_psaA
atgcacatcttcagatatatcaatacaacactgtggtccaaagcaggacattttaataaa
gctttatcaaaaggagctaagacaactacttggatatggaatctacatgattatgctcat
gattttgacattcaacaaagatccacaggtttaatagcaagaaaagtcttctcatccaat
ctagctcatctatctttggtattcttttggattagtggaatgcaccttcatggtgcatac
ttgtctaattatgatatttggttaaaagatcccaaatccatcaccccatcatcacatcta
gcctactctttaattggtcaagatattcttaactcatatacctcagaatacttctcagga
atcactatcacatctggctttttccaactctaccgttctgagggtataattacacagtct
cagctgaaatatgcatgtgctacttccctaatagctacacttatctgcctttctggatct
tatctccacatgcagcttatgtccaaattcactagcttttacaagaagttccagtcctta
tcgcaagatcatctcataattatatttggctccagctccacatccctctccgctcatcag
atacacaaaatgcttccagctaaccctttactagattcaggtatctccaacccaagtatc
ctacaagtaatctccaacagtctcagtgaaaacctagcactgttttcaacaaatctaagc
tctacaggtaaattgttaaatccctcaacaagaagtgtattcctttctcaagttgcagca
catcataagacaacaggtgtagtctttatcacactagggcttatcagattcctcaccatg
tataactctcaatttagtatacttacatcttatatagattaccacattgtgctatctatt
aatttagcattaattgcatctttatcaataatagtagctgatcatctcacaagaacccca
atctatcctcacaactcaacctcataccctacaatcctttgcctctccatacatcatgca
tggttatccggtttcctcatcattggttcaggagctcatgcatcaatattcaacttgcga
gcatcaccaacatctgagataagacatcgagaccccatatactcacacctcatctgggta
tgtatagcaataggattacactcattcagtctctactgtcataacgacactttagaagca
ttaggacgtccagaagatatctttcatgacaactctatccagttaaaagcaatatttgcc
aagcaatcctttctaagagcagagctccagccagatatcgagatgttagataaaaagatt
atacgtataacccaagaattgggaacagcagattttatagttcaccacattcatgcattt
acaatccatgttactctcttaattctttcaaagggtgtactttatgctagaaactctaga
tttgtatcagataaattggaacttggttttacttatccctgtgatggtccaggtagaggt
ggtacatgtcaaatatcaccttgggatcacttattctcagctgtattttggatgtataac
tgtttaaatatagtaacattccactacttttggaagatgcaatcagatgtttggggcttt
gtatcgatccaaaagcacatatctcactacagccaaggtgattttagtgtcaactctatt
accatcaatggttggttgagaaacttattatggtccgaggcatcccaagtaatccaatcc
tatgctctttcatccatttgtccatatgggttcattttcctcatagctcatttcatctgg
gcatttagtttaatgttcctctttagtggtagagcatactggcaagaattaattgagtcc
attctatggtcacatcataaattaaagattatacctcatatccaaccaagggcattaagc
atttctcaaggaagagcagttggtttcattcattacactctaggaggtatcggctcaacc
tgggcatttattatttcaagactgctagtattaacttcttga
>IS3_psaA
atgcacatcttcagatatatcaatactacactgtggtccaaagcaggacactttaataaa
gctttatcaaaaggagctaagacaactacttggatatggaatctacatgattatgcacat
gattttgatattcaacaaagctccgcaggtttaatagcaagaaaagtattctcatccaac
ctagcccatttatctttggtattcttttggattagtggaatgcaccttcatggtgcatac
ttgtctaattatgatatttggttaaaagatcctaaatccatcacgccatcatcacagcta
gcttactctttagttggccaagatattcttaactcatatacctcagaatacttctcagga
atcactattacttcaggactatttcaactctaccgttctgagggtataattacacagtct
cagctgaaatatgcatgtgctacttccctaatagctacacttatctgtcttactggatct
tatctccacatgcaagtgatgtccaaatttagtagcttttataagaagttccagtcctta
tcgcaagatcatctcataattatctttggctccagatctatatctctctccgctcatcag
atacacaaaatgcttccagctaactatcaactagattcaggcatctccaatccaagtatc
ctacaagtaatctccaacagtctcagctatacactagcactgttttcaacaaatctaagc
tctacaggcaaattgttaaatccctcaacaagaagtatattcctttctcaagttgcagca

catcataagacaataggtatagtacttatcacactagggcttatcagattcctcaccatt
tataattctcaatttagtatacttacatcttatatagattaccaccttgtcctatctact
aacttagcattaattggatctttatcaataataacagctgatcatctcacaagaacccca
atctatcctcacaagtcaacctcataccctacaatcctttgcctctccatacatcatgca
tggttatccggtttcctcatcattggttcaggagctcatgcatcaatattcaacttgctt
ggatcaccaacatctgagataagacatcgagaccccatatactcacacctcatctggata
tgtatagctttaggattacactcatttagtctctactgccataacgacactttagaagct
ttaggacgtccagaagatatctttcacgataactctatccagttaaaagctatatttgct
aagcaatcctttctaagagcagagctccagccagatatcgagatcttagataaaaagatt
atacgtataactcaagaattgggaacagctgattttatagttcaccacattcatgcattt
acaatccatgttactctcttaattctttcaaagggtgtactttatgctagaaagtctaga
tttgtatcagataaattggaacttggttttacttatccttgtgatgggccaggtagaggt
ggtacatgtcaaatatcaccttgggatcacttattctcagctgtattttggatgtataat
tgtttaaatatagtagctttccacttcttttggaagatgcaatcagatatttggggcttt
gtatccatccaaaggcaaatttcccactacagccaaggtgattttagtgtaaattctatt
accatcaacggctggttgagaaacttattatggtctcaggcatctcaagtaatacaatcc
tatgctcttccatctatttgtccatatgggttcattttcctcacagctcatttcatatgg
gcattctctttaatgttcctatttagtggtagagcatactggcaagaattaattgagtct
attctatggtcccaccataaattaaagattatacctcatatccaaccaagggccttaagc
atttctcaaggaagagcagttggcttcactcattatactctaggtggtatcggctcaacc
tgggcatttattatttcaagactgctagtattaacttcttga
>IS4_psaA
atgcacatcttcagatatatcaatactacactgtggtccaaagcaggacactttaataaa
gctttatcaaaaggagctaagacaactacttggatatggaatctacatgattatgcacat
gattttgatattcaacaaagctccgcaggtttaatagcaagaaaagtattctcatccaac
ctagcccatttatctttggtattcttttggattagtggaatgcaccttcatggtgcatac
ttgtctaattatgatatttggttaaaagatcctaaatctataagcccttcatcacagcta
gcttactctttagttggccaagatattcttaactcatatacctcagaatacttctcagga
atcactattacttcaggactatttcaactctaccgttctgagggtataattacacagtct
cagctgaaatatgcatgtgctacttccctaatagctacacttatctgtcttactggatct
tatctccacatgcaagtgatgtccaaatttagtagcttttataagaagttccagtcctta
tcgcaagatcatctcataattatctttggctccagatctatatctctctccgctcatcag
atacacaaaatgcttccagctaactatcaactagattcaggcatctccaacccaagtatc
ctacaagtaatctccaacagtctcagtgaaaacctagcactgttttcaacaaatctaagc
tctacaggcaaattgttaaatccctcaacaagaagtatattcctttctcaagttgcagca
catcataagacaataggtatagtacttatcacactagggcttatcagattcctcaccatt
tataattctcaatttagtatacttacatcttatatagattaccaccttgtcctatctact
aacttagcattaattgcatctttatcaataatagtagctgatcatctcacaagaacccca
atctatcctcacaactcaacctcataccctacaatcctttgcctctccatacatcatgca
tggttatccggtttcctcatcattggttcaggagctcatgcatcaatattcaacttgcga
gcatcaccaacatctgagataagacatcgagaccccatatactcacacctcatctggata
tgtatagctttaggattacactcatttagtctctactgccataacgacactttagaagct
ttaggacgtccagaagatatctttcacgataactctatccagttaaaagctatatttgct
aagcaatcctttctaagagcagagctccagccagatatcgagatcttagataaaaagatt
atacgtataactcaagaattgggaacagctgattttatagttcaccacattcatgcattt
acaatccatgttactctcttaattctttcaaagggtgtactttatgctagaaagtctaga
tttgtatcagataaattggaacttggttttacttatccttgtgatgggccaggtagaggt
ggtacatgtcaaatatcaccttgggatcacttattctcagctgtattttggatgtataat
tgtttaaatatagtagctttccacttcttttggaagatgcaatcagatatttggggcttt
gtatccatccaaaggcaaatttcccactacagccaaggtgattttagtgtaaattctatt
accatcaacggctggttgagaaacttattatggtctcaggcatctcaagtaatacaatcc
tatgctcttccatctatttgtccatatgggttcattttcctcacagctcatttcatatgg
gcattctctttaatgttcctatttagtggtagagcatactggcaagaattaattgagtct
attctatggtcccaccataaattaaagattatacctcatatccaacctagggccttaagc
atttctcaaggaagagcagttggcttcactcattatactctaggtggtatcggctcaacc
tgggcatttattatttcaagactgctagtattaacttcttga
>IS5_psaA

atgcacatcttcagatatatcaacacaacactgtggtccaaagcaggacattttaataaa
gctttatcaaaaggagctaagacaactacttggatatggaatctacatgattatgcacat
gattttgacattcaacaaagatccacaggtttaatagcaagaaaagtcttctcatccaat
ctagctcatctatctttggtattcttttggattagtggaatgcaccttcatggagcatat
ttgtctaattatgatatttggttaaaagatcccaaatccatcaccccatcatcacatcta
gcctactctttaattggtcaagatattcttaactcatatacctcagaatacttctcagga
atcactatcacatctggctttttccaactctaccgttctgagggtatcattacacagtcc
cagctgaaatatgcatgtgctacttccctaatagctacacttatctgcctttctggatct
tatctccacatgcagcttatgtccaaattcactagcttttacaagaagttccagtcctta
tcgcaagatcatctcataattatatttggctccagctccacatccctctccgctcatcag
atacacaaaatgcttccagctaaccctttactagattcaggtatctccaacccaagtatc
ctacaagtaatctccaacagtctcagtgaaaacctagcactgttttcaacaaatctaagc
tctacaggtaaattgttaaatccctcaacaagaagtgtattcctttctcaagttgcagca
catcataagacaacaggtgtagtctttatcacactagggcttatcagattcctcaccatg
tataactctcaatttagtatacttacatcttatatagattaccacattgtgctatctatt
aatttagcattaattgcatctttatcaataatagtagctgatcatctcacaagaacccca
atctatcctcacaagtcaacctcataccctacaatcctttgcctctccatacatcatgca
tggttatccggtttcctcatcattggttcaggagctcatgcatcaatattcaacttgcga
gcatcaccaacatctgagataagacatcgagaccccatatactcacacctcatctgggta
tgtatagcaataggattacactcattcagtctctactgtcataacgacactttagaagca
ttaggacgtccagaagatatctttcatgacaactctatccagttaaaagcaatatttgcc
aagcaatcctttctaagagcagagctccagccagatatcgagatgttagataaaaagatt
atacgtataactcaagaattgggaacagctgattttatagttcaccacattcatgcattt
acaatccatgttactctcttaattctttcaaagggtgtactttatgctagaaactctaga
tttgtatcagataaattggaacttggttttacttatccctgtgatgggccaggtagaggt
ggtacatgtcaaatatcaccttgggatcacttattctcagctgtattttggatgtataac
tgtttaaatgtagtaacattccactacttttggaagatgcaatcagatgtttggggcttt
gtatcgatccaaaagcacatatctcactacagccaaggtgattttagtgtcaactctatt
accatcaatggttggttgagaaacttattatggtccgaggcatcccaagtaatccaatcc
tatgctctttcatccatttgtccatatgggttcattttcctcatagctcatttcatctgg
gcatttagtttaatgttcctctttagtggtagagcatactggcaagaattaattgagtcc
attctatggtcacatcataaattaaagattatacctcatatccaaccaagggcattaagc
atttctcaaggaagagcagttggtttcattcattacactctaggaggtatcggctcaacc
tgggcatttattatttcaagactgctagtattaacttcttga
>IS6_psaA
atgcacatcttcagatatatcaacacaacactgtggtccaaagcaggacattttaataaa
gctttatcaaaaggagctaagacaactacttggatatggaatctacatgattatgctcat
gattttgacattcaacaaagatccacaggtttaatagcaagaaaagtcttctcatccaat
ctagctcatctatctttggtattcttttggattagtggaatgcaccttcatggggcatat
ttgtctaattatgatatttggttaaaagatcccaaatccatcaccccatcatcacatcta
gcctactctttaattggtcaagatattcttaactcatatacctcagaatacttctcagga
atcactatcacatctggctttttccaactctaccgttctgagggtatcattacacagtcc
cagctgaaatatgcatgtgctacttccctaatagctacacttatctgcctttctggatct
tatctccacatgcagcttatgtccaaattcactagcttttacaagaagttccagtcctta
tcgcaagatcatctcataattatatttggctccagctccacatccctctccgctcatcag
atacacaaaatgcttccagctaaccctttactagattcaggtatctccaacccaagtatc
ctacaagtaatctccaacagtctcagtgaaaacctagcactgttttcaacaaatctaagc
tctacaggtaaattgttaaatccctcaacaagaagtgtattcctttctcaagttgcagca
catcataagacaacaggtgtagtctttatcacactagggcttatcagattcctcaccatg
tataactctcaatttagtatacttacatcttatatagattaccacattgtgctatctatt
aatttagcattaattgcatctttatcaataatagtagctgatcatctcacaagaacccca
atctatcctcacaagtcaacctcataccctacaatcctttgcctctccatacatcatgca
tggttatccggtttcctcatcattggttcaggagctcatgcatcaatattcaacttgcga
gcatcaccaacatctgagataagacatcgagaccccatatactcacacctcatctgggta
tgtatagcaataggattacactcattcagtctctactgtcataacgacactttagaagca
ttaggacgtccagaagatatctttcatgacaactctatccagttaaaagcaatatttgcc
aagcaatcctttctaagagcagagctccagccagatatcgagatgttagataaaaagatt

atacgtataacccaagaattgggaacagcagattttatagttcaccacattcatgcattt
acaatccatgttactctcttaattctttcaaagggtgtactttatgctagaaactctaga
tttgtatcagataaattggaacttggttttacttatccctgtgatggtccaggtagaggt
ggtacatgtcaaatatcaccttgggatcacttattctcagctgtattttggatgtataac
tgtttaaatgtagtaacattccactacttttggaagatgcaatcagatgtttggggcttt
gtatcgatccaaaagcacatatctcactacagccaaggtgattttagtgtcaactctatt
accatcaatggttggttgagaaacttattatggtccgaggcatcccaagtaatccaatcc
tatgctctttcatccatttgtccatatgggttcattttcctcatagctcatttcatctgg
gcatttagtttaatgttcctctttagtggtagagcatactggcaagaattaattgagtcc
attctatggtcacatcataaattaaagattatacctcatatccaaccaagggcattaagc
atttctcaaggaagagcagttggtttcattcattacactctaggaggtatcggctcaacc
tgggcatttattatttcaagactgctagtattaacttcttga
>IS7_psaA
atgcacatcttcagatatatcaatactacactgtggtccaaagcaggacactttaataaa
gctttatcaaaaggagctaagacaactacttggatatggaatctacatgattatgcacat
gattttgatattcaacaaagatccacaggtttaatagcaagaaaagtcttctcatccaat
ctagctcatctatctttggtattcttttggattagtggaatgcaccttcatggggcatat
ttgtctaattatgatatttggttaaaagatcctaaatctataagtccttcatcacagcta
gcttactctttagttggccaagatattcttaactcatatacctcagaatacttctcagga
atcactattacttcaggactttttcaactctaccgttctgagggtataattacacagtct
cagctgaaatatgcatgtgctacttccctaatagctacacttatctgtcttactggatct
tatctccacatgcagcttatgtccaaattcactagcttttacaagaagttccagtcctta
tcgcaagatcatctcataattatctttggctccagctctatatctctctccgctcatcag
atacacaaaatgcttccagctaaccatcaactagattcaggcatctccaatccaagtatc
ctacaagtaatctccaacagtctcagtgaaaacctagcactgttttcaacaaatctaagc
tctacaggtaaattgttaaatccctcaacaagaagtgtattcctttctcaagttgcagca
catcataagacaataggtatagtacttatcacactagggcttatcagattcctcaccatt
tataattctcaatttagtatacttacatcttatatagattaccaccttgtcctatctact
aacttagcattaattggatctttatcaataataacagctgatcatctcacaagaacccca
atctatcctcacaactcaacctcataccctacaatcctttgcctctccatacatcatgca
tggttatccggtttcctcatcattggttcaggagctcatgcatcaatattcaacttgcga
gcatcaccaacatctgagataagacatcgagaccccatatactcacacctcatctgggta
tgtatagctataggattacactcattcagtctctactgtcataacgacactttagaagca
ttaggacgtccagaagatatctttcacgataactctatccagttaaaagctatatttgct
aagcaatcctttctaagagcagagctccagccagatatcgagatgttagataaaaagatt
atacgtataacccaagaattgggaacagcagattttatagttcaccacattcatgcattt
acaatccatgttactcttttaattctttcaaagggtgtactttatgctagaaagtctaga
tttgtatcagataaattggaacttggttttacttatccctgtgatgggccaggtagaggt
ggtacatgtcaaatatcaccttgggatcacttattctcagctgtattttggatgtataat
tgtttaaatatagtagctttccacttcttttggaagatgcaatcagatatttggggcttt
gtatccatccaaaagcacatatctcactacagccaaggtgattttagtgtaaattctatt
accatcaatggttggttgagaaacttattatggtctcaggcatctcaagtaatccaatcc
tatgctctttcatccatttgtccatatgggttcattttcctcacagctcatttcatatgg
gcattctctttaatgttcctctttagtggtagagcatactggcaagaattaattgagtcc
attctatggtcacatcataaattaaagattatacctcatatccaacctagggccttaagc
atttctcaaggaagagcagttggcttcactcattacactctaggaggtatcggctcaacc
tgggcatttattatttcaagactgctagtattaacttcttga
>IS8_psaA
atgcacatcttcagatatatcaacacaacactgtggtccaaagcaggacattttaataaa
gctttatcaaaaggagctaagacaactacttggatatggaatctacatgattatgctcat
gattttgacattcaacaaagatccacaggtttaatagcaagaaaagtcttctcatccaat
ctagctcatctatctttggtattcttttggattagtggaatgcaccttcatggggcatat
ttgtctaattatgatatttggttaaaagatcccaaatccatcaccccatcatcacatcta
gcctactctttaattggtcaagatattcttaactcatatacctcagaatacttctcagga
atcactatcacatctggctttttccaactctaccgttctgagggtatcattacacagtcc
cagctcaaatatgcatgtgctacttccctaatagctacacttatctgcctttctggatct
tatctccacatgcagcttatgtccaaattcactagcttttacaagaagttccagtcctta

tcgcaagatcatctcataattatatttggctccagctccacatccctctccgctcatcag
atacacaaaatgcttccagctaactatcaactagattcaggcatctccaacccaagtatc
ctacaagtaatctccaacagtctcagtgaaaacctagcactgttttcaacaaatctaagc
tctacaggtaaattgttaaatccctcaacaagaagtgtattcctttctcaagttgcagca
catcataagacaataggtgtagtctttatcacactagggcttatcagattcctcaccatg
tataactctcaatttagtatacttacatcttatatagattaccacattgtgctatctatt
aatttagcattaattgcatctttatcaataatagtagctgatcatctcacaagaacccca
atctatcctcacaactcaacctcataccctacaatcctttgcctctccatacatcatgca
tggttatccggtttcctcatcattggttcaggagctcatgcatcaatattcaacttgcga
gcatcaccaacatctgagataagacatcgagaccccatatactcacacctcatctgggta
tgtatagcaataggattacactcattcagtctctactgtcataacgacactttagaagca
ttaggacgtccagaagatatctttcatgacaactctatccagttaaaagcaatatttgcc
aagcaatcctttctaagagcagagctccagccagatatcgagatgttagataaaaagatt
atacgtataacccaagaattgggaacagcagattttatagttcaccacattcatgcattt
acaatccatgttactctcttaattctttcaaagggtgtactttatgctagaaactctaga
tttgtatcagataaattggaacttggttttacttatccctgtgatggtccaggtagaggt
ggtacatgtcaaatatcaccttgggatcacttattctcagctgtattttggatgtataac
tgtttaaatgtagtaacattccactacttttggaagatgcaatcagatgtttggggcttt
gtatcgatccaaaagcacatatctcactacagccaaggtgattttagtgtcaactctatt
accatcaatggttggttgagaaacttattatggtccgaggcatcccaagtaatccaatcc
tatgctctttcatccatttgtccatatgggttcattttcctcataggtcatttcatctgg
gcatttagtttaatgttcctctttagtggtagagcatactggcaagaattaattgagtcc
attctatggtcacatcataaattaaagattatacctcatatccaaccaagggcattaagc
atttctcaaggaagagcagttggtttcattcattacactctaggaggtatcggctcaacc
tgggcatttattatttcaagactgctagtattaacttcttga
>IS9_psaA
atgcacatcttcagatatatcaatactacactgtggtccaaagcaggacactttaataaa
gctttatcaaaaggagctaagacaactacttggatatggaatctacatgattatgcacat
gattttgacattcaacaaagatccgcaggtttaatagcaagaaaagtattctcatccaac
ctagctcatctatctttggtattcttttggattagtggaatgcaccttcatggggcatat
ttgtctaattatgatatttggttaaaagatcctaaatctataagtccttcatcacagcta
gcttactctttagttggccaagatattcttaactcatatacctcagaatacttctcagga
atcactattacttcaggactatttcaactctaccgttctgagggtataattacacagtct
cagctgaaatatgcatgtgctacttccctaatagctacacttatctgtcttactggatct
tatctccacatgcaagtgatgtccaaatttagtagcttttataagaagttccagtcctta
tcgcaagatcatctcataattatctttggctccagatctatatctctctccgctcatcag
atacacaaaatgcttccagctaactatcaactagattcaggcatctccaatccaagtatc
ctacaagtaatctccaacagtctcagctatacactagcactgttttcaacaaatctaagc
tctacaggcaaattgttaaatccctcaacaagaagtatattcctttctcaagttgcagca
catcataagacaataggtatagtacttatcacactagggcttatcagattcctcaccatt
tataattctcaatttagtatacttacatcttatatagattaccacattgtcctatctatt
aatttagcattaattgcatctttatcaataatagtagctgatcatctcacaagaacccca
atctatcctcacaactcaacctcataccctacaatcctttgcctctccatacatcatgca
tggttatccggtttcctcatcattggttcaggagctcatgcatcaatattcaacttgcga
gcatcaccaacatctgagataagacatcgagaccccatatactcacacctcatctggata
tgtatagctttaggattacactcatttagtctctactgccataacgacactttagaagct
ttaggacgtccagaagatatctttcacgataactctatccagttaaaagctatatttgct
aagcaatcctttctaagagcagagctccagccagatatcgagatcttagataaaaagatt
atacgtataactcaagaattgggaacagctgattttatagttcaccacattcatgcattt
acaatccatgttactctcttaattctttcaaagggtgtactttatgctagaaagtctaga
tttgtatcagataaattggaacttggttttacttatccttgtgatggtccaggtagaggt
ggtacatgtcaaatatcaccttgggatcacttattctcagctgtattttggatgtataac
tgtttaaatgtagtaacattccacttcttttggaagatgcaatcagatgtttggggcttt
gtatcgatccaaaagcacatatctcactacagccaaggtgattttagtgtaaattctatt
accatcaacggctggttgagaaacttattatggtctcaggcatctcaagtaatacaatcc
tatgctctttcatccatttgtccatatgggttcattttcctcataggtcatttcatctgg
gcattctctttaatgttcctatttagtggtagagcatactggcaagaattaattgagtct

attctatggtcacaccataaattaaagattatacctcatatccaacctagggccttaagc
atttctcaaggaagagcagttggcttcactcattacactctaggtggtatcggctcaacc
tgggcatttattatttcaagactgctagtattaacttcttga
>Ik1_psaA
atgcacatcttcagatatatcaacacaacactgtgggccaaagcaggacattttaataaa
gctttatcaaaaggagctaagacaactacttggatatggaatctacatgattatgctcat
gattttgacattcaacaaagatccacaggtttaatagcaagaaaagtcttctcatccaat
ctagctcatctatctttggtattcttttggattagtggaatgcaccttcatggggcatat
ttgtctaattatgatatttggttaaaagatcccaaatccatcaccccatcatcacatcta
gcctactctttaattggtcaagatattcttaactcatatacctcagaatacttctcagga
atcactatcacatctggctttttccaactctaccgttctgagggtatcattacacagtcc
cagctcaaatatgcatgtgctacttccctaatagctacacttatctgcctttctggatct
tatctccacatgcagcttatgtccaaattcactagcttttacaagaagttccagtcctta
tcgcaagatcatctcataattatatttggctccaggtccacatccctctccgctcatcag
atacacaaaatgcttccagctaacccttactagattcaggtatctccaacccaagtatc
ctacaagtaatctccaacagtctcagctatacactagcactgttttcaacaaatctaagc
tctacaggtaaattgttaaatccctcaacaagaagtgtattcctttctcaagttgcagca
catcataagacaacaggtgtagtctttatcacactagggcttatcagattcctcaccatg
tataactctcaatttagtatacttacatcttatatagattaccacattgtgctatctatt
aatttagcattaattgcatctttatcaataatagtagctgatcatctcacaagaacccca
atctatcctcacaagtcaacctcataccctacaatcctttgcctctccatacatcatgca
tggttatccggtttcctcatcattggttcaggagctcatgcatcaatattcaacttgcta
gcatcaccaacatctgagataagacatcgagaccccatatactcacacctcatctgggta
tgtatagcaataggattacactcattcagtctctactgtcataacgacactttagaagca
ttaggacgtccagaagatatctttcatgacaactctatccagttaaaagcaatatttgcc
aagcaatcctttctaagagcagagctccagccagatatcgagatgttagataaaaagatt
atacgtataacccaagaattgggaacagcagattttatagttcaccacattcatgcattt
acaatccatgttactctcttaattctttcaaagggtgtactttatgctagaaactctaga
tttgtatcagataaattggaacttggttttacttatccctgtgatggtccaggtagaggt
ggtacatgtcaaatatcaccttgggatcacttattctcagctgtattttggatgtataac
tgtttaaatgtagtaacattccactacttttggaagatgcaatcagatgtttggggcttt
gtatcgatccaaaagcacatatctcactacagccaaggtgattttagtgtcaactctatt
accatcaatggttggttgagaaacttattatggtccgaggcatcccaagtaatccaatcc
tatgctctttcatccatttgtccatatgggttcattttcctcatagctcatttcatctgg
gcatttagtttaatgttcctctttagtggtagagcatactggcaagaattaattgagtcc
attctatggtcacatcataaattaaagattatacctcatatccaaccaagggcattaagc
atttctcaaggaagagcagttggtttcattcattacactctaggaggtatcggctcaacc
tgggcatttattatttcaagactgctagtattaacttcttga
>Ik2_psaA
atgcacatcttcagatatatcaacacaacactgtggtccaaagcaggacattttaataaa
gctttatcaaaaggagctaagacaactacttggatatggaatctacatgattatgctcat
gattttgacattcaacaaagatccacaggtttaatagcaagaaaagtcttctcatccaat
ctagctcatctatctttggtattcttttggattagtggaatgcaccttcatggggcatat
ttgtctaattatgatatttggttaaaagatcccaaatccatcaccccatcatcacatcta
gcctactctttaattggtcaagatattcttaactcatatacctcagaatacttctcagga
atcactatcacatctggctttttccaactctaccgttctgagggtatcattacacagtcc
cagctcaaatatgcatgtgctacttccctaatagctacacttatctgcctttctggatct
tatctccacatgcagcttatgtccaaattcactagcttttacaagaagttccagtcctta
tcgcaagatcatctcataattatatttggctccaggtccacatccctctccgctcatcag
atacacaaaatgcttccagctaacccttactagattcaggtatctccaacccaagtatc
ctacaagtaatctccaacagtctcagtgaaaacctagcactgttttcaacaaatctaagc
tctacaggtaaattgttaaatccctcaacaagaagtgtattcctttctcaagttgcagca
catcataagacaacaggtgtagtctttatcacactagggcttatcagattcctcaccatg
tataactctcaatttagtatacttacatcttatatagattaccacattgtgctatctatt
aatttagcattaattgcatctttatcaataatagtagctgatcatctcacaagaacccca
atctatcctcacaactcaacctcataccctacaatcctttgcctctccatacatcatgca
tggttatccggtttcctcatcattggttcaggagctcatgcatcaatattcaacttgcga

gcatcaccaacatctgagataagacatcgagaccccatatactcacacctcatctgggta
tgtatagcaataggattacactcattcagtctctactgtcataacgacactttagaagca
ttaggacgtccagaagatatctttcatgacaactctatccagttaaaagcaatatttgcc
aagcaatcctttctaagagcagagctccagccagatatcgagatgttagataaaaagatt
atacgtataacccaagaattgggaacagcagattttatagttcaccacattcatgcattt
acaatccatgttactctcttaattctttcaaagggtgtactttatgctagaaactctaga
tttgtatcagataaattggaacttggttttacttatccctgtgatggtccaggtagaggt
ggtacatgtcaaatatcaccttgggatcacttattctcagctgtattttggatgtataac
tgtttaaatgtagtaacattccactacttttggaagatgcaatcagatgtttggggcttt
gtatcgatccaaaagcacatatctcactacagccaaggtgattttagtgtcaactctatt
accatcaatggttggttgagaaacttattatggtccgaggcatcccaagtaatccaatcc
tatgctctttcatccatttgtccatatgggttcattttcctcatagctcatttcatctgg
gcatttagtttaatgttcctctttagtggtagagcatactggcaagaattaattgagtcc
attctatggtcacatcataaattaaagattatacctcatatccaaccaagggcattaagc
atttctcaaggaagagcagttggtttcattcattacactctaggaggtatcggctcaacc
tgggcatttattatttcaagactgctagtattaacttcttga
>Ik3_psaA
atgcacatcttcagatatatcaacacaacactgtgggccaaagcaggacattttaataaa
gctttatcaaaaggagctaagacaactacttggatatggaatctacatgattatgctcat
gattttgacattcaacaaagatccacaggtttaatagcaagaaaagtcttctcatccaat
ctagctcatctatctttggtattcttttggattagtggaatgcaccttcatggggcatat
ttgtctaattatgatatttggttaaaagatcccaaatccatcacgccatcatcacatcta
gcctactctttaattggtcaagatattcttaactcatatacctcagaatacttctcagga
atcactatcacatctggctttttccaactctaccgttctgagggtatcattacacagtcc
cagctgaaatatgcatgtgctacttccctaatagctacacttatctgcctttctggatct
tatctccacatgcagcttatgtccaaattcactagcttttacaagaagttccagtcctta
tcgcaagatcatctcataattatatttggctccaggtccacatccctctccgctcatcag
atacacaaaatgcttccagctaaccctttactagattcaggtatctccaacccaagtatc
ctacaagtaatctccaacagtctcagcgataccctagcactgttttcaacaaatctaagc
tctacaggtaaattgttaaatccctcaacaagaagtgtattcctttctcaagttgcagca
catcataagacaacaggtgtagtctttatcacactagggcttatcagattcctcaccatg
tataagtctcaatttagtatacttacatcttatatagattaccacattgtgctatctatt
aatttagcattaattgcatctttatcaataatagtagctgatcatctcacaagaacccca
atctatcctcacaagtcaacctcataccctacaatcctttgcctctccatacatcatgca
tggttatccggtttcctcatcattggttcaggagctcatgcatcaatattcaacttgctt
ggatcaccaacatctgagataagacatcgagaccccatatactcacacctcatctgggta
tgtatagcaataggattacactcattcagtctctactgtcataacgacactttagaagca
ttaggacgtccagaagatatctttcatgacaactctatccagttaaaagcaatatttgcc
aagcaatcctttctaagagcagagctccagccagatatcgagatgttagataaaaagatt
atacgtataacccaagaattgggaacagcagattttatagttcaccacattcatgcattt
acaatccatgttactctcttaattctttcaaagggtgtactttatgctagaaactctaga
tttgtatcagataaattggaacttggttttacttatccctgtgatggtccaggtagaggt
ggtacatgtcaaatatcaccttgggatcacttattctcagctgtattttggatgtataac
tgtttaaatgtagtaacattccactacttttggaagatgcaatcagatgtttggggcttt
gtatcgatccaaaagcacatatctcactacagccaaggtgattttagtgtcaactctatt
accatcaatggttggttgagaaacttattatggtccgaggcatcccaagtaatccaatcc
tatgctctttcatccatttgtccatatgggttcattttcctcatagctcatttcatctgg
gcatttagtttaatgttcctctttagtggtagagcatactggcaagaattaattgagtcc
attctatggtcacatcataaattaaagattatacctcatatccaaccaagggcattaagc
atttctcaaggaagagcagttggtttcattcattacactctaggaggtatcggctcaacc
tgggcatttattatttcaagactgctagtattaacttcttga
>Ik4_psaA
atgcacatcttcagatatatcaacacaacactgtggtccaaagcaggacattttaataaa
gctttatcaaaaggagctaagacaactacttggatatggaatctacatgattatgctcat
gattttgacattcaacaaagatccacaggtttaatagcaagaaaagtcttctcatccaat
ctagctcatctatctttggtattcttttggattagtggaatgcaccttcatggggcatat
ttgtctaattatgatatttggttaaaagatcccaaatccatcaccccatcatcacatcta

gcctactctttaattggtcaagatattcttaactcatatacctcagaatacttctcagga
atcactatcacatctggctttttccaactctaccgttctgagggtatcattacacagtcc
cagctgaaatatgcatgtgctacttccctaatagctacacttatctgcctttctggatct
tatctccacatgcagcttatgtccaaattcactagcttttacaagaagttccagtcctta
tcgcaagatcatctcataattatatttggctccagctccacatccctctccgctcatcag
atacacaaaatgcttccagctaaccctttactagattcaggtatctccaacccaagtatc
ctacaagtaatctccaacagtctcagtgaaaacctagcactgttttcaacaaatctaagc
tctacaggtaaattgttaaatccctcaacaagaagtgtattcctttctcaagttgcagca
catcataagacaacaggtgtagtctttatcacactagggcttatcagattcctcaccatg
tataactctcaatttagtatacttacatcttatatagattaccacattgtgctatctatt
aatttagcattaattgcatctttatcaataatagtagctgatcatctcacaagaacccca
atctatcctcacaagtcaacctcataccctacaatcctttgcctctccatacatcatgca
tggttatccggtttcctcatcattggttcaggagctcatgcatcaatattcaacttgctt
ggatcaccaacatctgagataagacatcgagaccccatatactcacacctcatctgggta
tgtatagcaataggattacactcattcagtctctactgtcataacgacactttagaagca
ttaggacgtccagaagatatctttcatgacaactctatccagttaaaagcaatatttgcc
aagcaatcctttctaagagcagagctccagccagatatcgagatgttagataaaaagatt
atacgtataacccaagaattgggaacagcagattttatagttcaccacattcatgcattt
acaatccatgttactctcttaattctttcaaagggtgtactttatgctagaaactctaga
tttgtatcagataaattggaacttggttttacttatccctgtgatggtccaggtagaggt
ggtacatgtcaaatatcaccttgggatcacttattctcagctgtattttggatgtataac
tgtttaaatgtagtaacattccactacttttggaagatgcaatcagatgtttggggcttt
gtatcgatccaaaagcacatatctcactacagccaaggtgattttagtgtcaactctatt
accatcaatggttggttgagaaacttattatggtccgaggcatcccaagtaatccaatcc
tatgctctttcatccatttgtccatatgggttcattttcctcataggtcatttcatctgg
gcatttagtttaatgttcctctttagtggtagagcatactggcaagaattaattgagtcc
attctatggtcacatcataaattaaagattatacctcatatccaaccaagggcattaagc
atttctcaaggaagagcagttggtttcattcattacactctaggaggtatcggctcaacc
tgggcatttattatttcaagactgctagtattaacttcttga
>Ik5_psaA
atgcacatcttcagatatatcaacacaacactgtgggccaaagcaggacattttaataaa
gctttatcaaaaggagctaagacaactacttggatatggaatctacatgattatgctcat
gattttgacattcaacaaagatccacaggtttaatagcaagaaaagtcttctcatccaat
ctagctcatctatctttggtattcttttggattagtggaatgcaccttcatggggcatat
ttgtctaattatgatatttggttaaaagatcccaaatccatcaccccatcatcacatcta
gcctactctttaattggtcaagatattcttaactcatatacctcagaatacttctcagga
atcactatcacatctggctttttccaactctaccgttctgagggtatcattacacagtcc
cagctgaaatatgcatgtgctacttccctaatagctacacttatctgcctttctggatct
tatctccacatgcagcttatgtccaaattcactagcttttacaagaagttccagtcctta
tcgcaagatcatctcataattatatttggctccaggtccacatccctctccgctcatcag
atacacaaaatgcttccagctaaccctttactagattcaggtatctccaagccaagtatc
ctacaagtaatctccaacagtctcagctatacactagcactgttttcaacaaatctaagc
tctacaggtaaattgttaaatccctcaacaagaagtgtattcctttctcaagttgcagca
catcataagacaacaggtgtagtctttatcacactagggcttatcagattcctcaccatg
tataactctcaatttagtatacttacatcttatatagattaccacattgtgctatctatt
aatttagcattaattgcatctttatcaataatagtagctgatcatctcacaagaacccca
atctatcctcacaactcaacctcataccctacaatcctttgcctctccatacatcatgca
tggttatccggtttcctcatcattggttcaggagctcatgcatcaatattcaacttgcga
gcatcaccaacatctgagataagacatcgagaccccatatactcacacctcatctgggta
tgtatagcaataggattacactcattcagtctctactgtcataacgacactttagaagca
ttaggacgtccagaagatatctttcatgacaactctatccagttaaaagcaatatttgcc
aagcaatcctttctaagagcagagctccagccagatatcgagatgttagataaaaagatt
atacgtataacccaagaattgggaacagcagattttatagttcaccacattcatgcattt
acaatccatgttactctcttaattctttcaaagggtgtactttatgctagaaactctaga
tttgtatcagataaattggaacttggttttacttatccctgtgatgggccaggtagaggt
ggtacatgtcaaatatcaccttgggatcacttattctcagctgtattttggatgtataac
tgtttaaatgtagtaacattccactacttttggaagatgcaatcagatgtttggggcttt

gtatcgatccaaaagcacatatctcactacagccaaggtgattttagtgtcaactctatt
accatcaatggttggttgagaaacttattatggtccgaggcatcccaagtaatccaatcc
tatgctctttcatccatttgtccatatgggttcattttcctcatagctcatttcatctgg
gcatttagtttaatgttcctctttagtggtagagcatactggcaagaattaattgagtcc
attctatggtcacatcataaattaaagattatacctcatatccaaccaagggcactaagc
atttctcaaggaagagcagttggtttcattcattacactctaggaggtatcggctcaacc
tgggcatttattatttcaagactgctagtattaacttcttga
>Ik6_psaA
atgcacatcttcagatatatcaacacaacactgtggtccaaagcaggacattttaataaa
gctttatcaaaaggagctaagacaactacttggatatggaatctacatgattatgctcat
gattttgacattcaacaaagatccacaggtttaatagcaagaaaagtcttctcatccaat
ctagctcatctatctttggtattcttttggattagtggaatgcaccttcatggggcatat
ttgtctaattatgatatttggttaaaagatcccaaatccatcaccccatcatcacatcta
gcctactctttaattggtcaagatattcttaactcatatacctcagaatacttctcagga
atcactatcacatctggctttttccaactctaccgttctgagggtatcattacacagtcc
cagctgaaatatgcatgtgctacttccctaatagctacacttatctgcctttctggatct
tatctccacatgcagcttatgtccaaattcactagcttttacaagaagttccagtcctta
tcgcaagatcatctcataattatatttggctccagctccacatccctctccgctcatcag
atacacaaaatgcttccagctaaccctttactagattcaggtatctccaagccaagtatc
ctacaagtaatctccaacagtctcagctatacactagcactgttttcaacaaatctaagc
tctacaggtaaattgttaaatccctcaacaagaagtgtattcctttctcaagttgcagca
catcataagacaacaggtgtagtctttatcacactagggcttatcagattcctcaccatg
tataagtctcaatttagtatacttacatcttatatagattaccacattgtgctatctatt
aatttagcattaattgcatctttatcaataatagtagctgatcatctcacaagaacccca
atctatcctcacaagtcaacctcatacctacaatcctttgcctctccatacatcatgca
tggttatccggtttcctcatcattggttcaggagctcatgcatcaatattcaacttgcga
gcatcaccaacatctgagataagacatcgagaccccatatactcacacctcatctgggta
tgtatagcaataggattacactcattcagtctctactgtcataacgacactttagaagca
ttaggacgtccagaagatatctttcatgacaactctatccagttaaaagcaatatttgcc
aagcaatcctttctaagagcagagctccagccagatatcgagatgttagataaaaagatt
atacgtataacccaagaattgggaacagcagattttatagttcaccacattcatgcattt
acaatccatgttactctcttaattctttcaaagggtgtactttatgctagaaactctaga
tttgtatcagataaattggaacttggttttacttatccctgtgatgggccaggtagaggt
ggtacatgtcaaatatcaccttgggatcacttattctcagctgtattttggatgtataac
tgtttaaatgtagtaacattccactacttttggaagatgcaatcagatgtttggggcttt
gtatcgatccaaaagcacatatctcactacagccaaggtgattttagtgtcaactctatt
accatcaatggttggttgagaaacttattatggtccgaggcatcccaagtaatccaatcc
tatgctctttcatccatttgtccatatgggttcattttcctcatagctcatttcatctgg
gcatttagtttaatgttcctctttagtggtagagcatactggcaagaattaattgagtcc
attctatggtcacatcataaattaaagattatacctcatatccaaccaagggcattaagc
atttctcaaggaagagcagttggtttcattcattacactctaggaggtatcggctcaacc
tgggcatttattatttcaagactgctagtattaacttcttga
>Ik7_psaA
atgcacatcttcagatatatcaacacaacactgtgggccaaagcaggacattttaataaa
gctttatcaaaaggagctaagacaactacttggatatggaatctacatgattatgctcat
gattttgacattcaacaaagatccacaggtttaatagcaagaaaagtcttctcatccaat
ctagctcatctatctttggtattcttttggattagtggaatgcaccttcatggggcatat
ttgtctaattatgatatttggttaaaagatcccaaatccatcaccccatcatcacatcta
gcctactctttaattggtcaagatattcttaactcatatacctcagaatacttctcagga
atcactatcacatctggctttttccaactctaccgttctgagggtatcattacacagtcc
cagctgaaatatgcatgtgctacttccctaatagctacacttatctgcctttctggatct
tatctccacatgcagcttatgtccaaattcactagcttttacaagaagttccagtcctta
tcgcaagatcatctcataattatatttggctccagctccacatccctctccgctcatcag
atacacaaaatgcttccagctaaccctttactagattcaggtatctccaagccaagtatc
ctacaagtaatctccaacagtctcagctatacactagcactgttttcaacaaatctaagc
tctacaggtaaattgttaaatccctcaacaagaagtgtattcctttctcaagttgcagca
catcataagacaacaggtgtagtctttatcacactagggcttatcagattcctcaccatg

tataactctcaatttagtatacttacatcttatatagattaccacattgtgctatctatt
aatttagcattaattgcatctttatcaataatagtagctgatcatctcacaagaacccca
atctatcctcacaagtcaacctcataccctacaatcctttgcctctccatacatcatgca
tggttatccggtttcctcatcattggttcaggagctcatgcatcaatattcaacttgcga
gcatcaccaacatctgagataagacatcgagaccccatatactcacacctcatctgggta
tgtatagcaataggattacactcattcagtctctactgtcataacgacactttagaagca
ttaggacgtccagaagatatctttcatgacaactctatccagttaaaagcaatatttgcc
aagcaatcctttctaagagcagagctccagccagatatcgagatgttagataaaaagatt
atacgtataacccaagaattgggaacagcagattttatagttcaccacattcatgcattt
acaatccatgttactctcttaattctttcaaagggtgtactttatgctagaaactctaga
tttgtatcagataaattggaacttggttttacttatccctgtgatggtccaggtagaggt
ggtacatgtcaaatatcaccttgggatcacttattctcagctgtattttggatgtataac
tgtttaaatgtagtaacattccactacttttggaagatgcaatcagatgtttggggcttt
gtatcgatccaaaagcacatatctcactacagccaaggtgattttagtgtcaactctatt
accatcaatggttggttgagaaacttattatggtccgaggcatcccaagtaatccaatcc
tatgctctttcatccatttgtccatatgggttcattttcctcataggtcatttcatctgg
gcatttagtttaatgttcctctttagtggtagagcatactggcaagaattaattgagtcc
attctatggtcacatcataaattaaagattatacctcatatccaaccaagggcattaagc
atttctcaaggaagagcagttggtttcattcattacactctaggaggtatcggctcaacc
tgggcatttattatttcaagactgctagtattaacttcttga
>Ik8_psaA
atgcacatcttcagatatatcaacacaacactgtggtccaaagcaggacattttaataaa
gctttatcaaaaggagctaagacaactacttggatatggaatctacatgattatgctcat
gattttgacattcaacaaagatccacaggtttaatagcaagaaaagtcttctcatccaat
ctagctcatctatctttggtattcttttggattagtggaatgcaccttcatggagcatat
ttgtctaattatgatatttggttaaaagatcccaaatccatcacgccatcatcacatcta
gcctactctttaattggtcaagatattcttaactcatatacctcagaatacttctcagga
atcactatcacatctggctttttccaactctaccgttctgagggtatcattacacagtcc
cagctcaaatatgcatgtgctacttccctaatagctacacttatctgcctttctggatct
tatctccacatgcagcttatgtccaaattcactagcttttacaagaagttccagtcctta
tcgcaagatcatctcataattatatttggctccaggtccacatccctctccgctcatcag
atacacaaaatgcttccagctaaccctttactagattcaggtatctccaacccaagtatc
ctacaagtaatctccaacagtctcagctatacactagcactgttttcaacaaatctaagc
tctacaggtaaattgttaaatccctcaacaagaagtgtattcctttctcaagttgcagca
catcataagacaacaggtgtagtctttatcacactagggcttatcagattcctcaccatg
tataagtctcaatttagtatacttacatcttatatagattaccacattgtgctatctatt
aatttagcattaattgcatctttatcaataatagtagctgatcatctcacaagaacccca
atctatcctcacaactcaacctcataccctacaatcctttgcctctccatacatcatgca
tggttatccggtttcctcatcattggttcaggagctcatgcatcaatattcaacttgcga
gcatcaccaacatctgagataagacatcgagaccccatatactcacacctcatctgggta
tgtatagcaataggattacactcattcagtctctactgtcataacgacactttagaagca
ttaggacgtccagaagatatctttcatgacaactctatccagttaaaagcaatatttgcc
aagcaatcctttctaagagcagagctccagccagatatcgagatgttagataaaaagatt
atacgtataacccaagaattgggaacagcagattttatagttcaccacattcatgcattt
acaatccatgttactctcttaattctttcaaagggtgtactttatgctagaaactctaga
tttgtatcagataaattggaacttggttttacttatccctgtgatgggccaggtagaggt
ggtacatgtcaaatatcaccttgggatcacttattctcagctgtattttggatgtataac
tgtttaaatgtagtaacattccactacttttggaagatgcaatcagatgtttggggcttt
gtatcgatccaaaagcacatatctcactacagccaaggtgattttagtgtcaactctatt
accatcaatggttggttgagaaacttattatggtccgaggcatcccaagtaatccaatcc
tatgctctttcatccatttgtccatatgggttcattttcctcatagctcatttcatctgg
gcatttagtttaatgttcctctttagtggtagagcatactggcaagaattaattgagtcc
attctatggtcacatcataaattaaagattatacctcatatccaaccaagggcattaagc
atttctcaaggaagagcagttggtttcattcattacactctaggaggtatcggctcaacc
tgggcatttattatttcaagactgctagtattaacttcttga
>Ik9_psaA
atgcacatcttcagatatatcaacacaacactgtggtccaaagcaggacattttaataaa

gctttatcaaaaggagctaagacaactacttggatatggaatctacatgattatgctcat
gattttgacattcaacaaagatccacaggtttaatagcaagaaaagtcttctcatccaat
ctagctcatctatctttggtattcttttggattagtggaatgcaccttcatggggcatat
ttgtctaattatgatatttggttaaaagatcccaaatccatcaccccatcatcacatcta
gcctactctttaattggtcaagatattcttaactcatatacctcagaatacttctcagga
atcactatcacatctggctttttccaactctaccgttctgagggtatcattacacagtcc
cagctgaaatatgcatgtgctacttccctaatagctacacttatctgcctttctggatct
tatctccacatgcagcttatgtccaaattcactagcttttacaagaagttccagtcctta
tcgcaagatcatctcataattatatttggctccagctccacatccctctccgctcatcag
atacacaaaatgcttccagctaaccctttactagattcaggtatctccaacccaagtatc
ctacaagtaatctccaacagtctcagtgaaaacctagcactgttttcaacaaatctaagc
tctacaggtaaattgttaaatccctcaacaagaagtgtattcctttctcaagttgcagca
catcataagacaacaggtgtagtctttatcacactagggcttatcagattcctcaccatg
tataagtctcaatttagtatacttacatcttatatagattaccacattgtgctatctatt
aatttagcattaattgcatctttatcaataatagtagctgatcatctcacaagaacccca
atctatcctcacaactcaacctcataccctacaatcctttgcctctccatacatcatgca
tggttatccggtttcctcatcattggttcaggagctcatgcatcaatattcaacttgctt
ggatcaccaacatctgagataagacatcgagaccccatatactcacacctcatctgggta
tgtatagcaataggattacactcattcagtctctactgtcataacgacactttagaagca
ttaggacgtccagaagatatctttcatgacaactctatccagttaaaagcaatatttgcc
aagcaatcctttctaagagcagagctccagccagatatcgagatgttagataaaaagatt
atacgtataacccaagaattgggaacagcagattttatagttcaccacattcatgcattt
acaatccatgttactctcttaattctttcaaagggtgtactttatgctagaaactctaga
tttgtatcagataaattggaacttggttttacttatccctgtgatggtccaggtagaggt
ggtacatgtcaaatatcaccttgggatcacttattctcagctgtattttggatgtataac
tgtttaaatgtagtaacattccactacttttggaagatgcaatcagatgtttggggcttt
gtatcgatccaaaagcacatatctcactacagccaaggtgattttagtgtcaactctatt
accatcaatggttggttgagaaacttattatggtccgaggcatcccaagtaatccaatcc
tatgctctttcatccatttgtccatatgggttcattttcctcatagctcatttcatctgg
gcatttagtttaatgttcctctttagtggtagagcatactggcaagaattaattgagtcc
attctatggtcacatcataaattaaagattatacctcatatccaaccaagggcattaagc
atttctcaaggaagagcagttggtttcattcattacactctaggaggtatcggctcaacc
tgggcatttattatttcaagactgctagtattaacttcttga
>Irm10_psaA
atgcacatcttcagatatatcaacacaacactgtggtccaaagcaggacattttaataaa
gctttatcaaaaggagctaagacaactacttggatatggaatctacatgattatgctcat
gattttgacattcaacaaagatccacaggtttaatagcaagaaaagtcttctcatccaat
ctagctcatctatctttggtattcttttggattagtggaatgcaccttcatggagcatat
ttgtctaattatgatatttggttaaaagatcccaaatccatcaccccatcatcacatcta
gcctactctttaattggtcaagatattcttaactcatatacctcagaatacttctcagga
atcactatcacatctggctttttccaactctaccgttctgagggtatcattacacagtcc
cagctgaaatatgcatgtgctacttccctaatagctacacttatctgcctttctggatct
tatctccacatgcagcttatgtccaaattcactagcttttacaagaagttccagtcctta
tcgcaagatcatctcataattatatttggctccaggtccacatccctctccgctcatcag
atacacaaaatgcttccagctaaccctttactagattcaggtatctccaacccaagtatc
ctacaagtaatctccaacagtctcagtgaaaacctagcactgttttcaacaaatctaagc
tctacaggtaaattgttaaatccctcaacaagaagtgtattcctttctcaagttgcagca
catcataagacaacaggtgtagtctttatcacactagggcttatcagattcctcaccatg
tataactctcaatttagtatacttacatcttatatagattaccacattgtgctatctatt
aatttagcattaattgcatctttatcaataatagtagctgatcatctcacaagaacccca
atctatcctcacaactcaacctcataccctacaatcctttgcctctccatacatcatgca
tggttatccggtttcctcatcattggttcaggagctcatgcatcaatattcaacttgctt
ggatcaccaacatctgagataagacatcgagaccccatatactcacacctcatctgggta
tgtatagcaataggattacactcattcagtctctactgtcataacgacactttagaagca
ttaggacgtccagaagatatctttcatgacaactctatccagttaaaagcaatatttgcc
aagcaatcctttctaagagcagagctccagccagatatcgagatgttagataaaaagatt
atacgtataacccaagaattgggaacagcagattttatagttcaccacattcatgcattt

acaatccatgttactctcttaattctttcaaagggtgtactttatgctagaaactctaga
tttgtatcagataaattggaacttggttttacttatccctgtgatggtccaggtagaggt
ggtacatgtcaaatatcaccttgggatcacttattctcagctgtattttggatgtataac
tgtttaaatgtagtaacattccactacttttggaagatgcaatcagatgtttggggcttt
gtatcgatccaaaagcacatatctcactacagccaaggtgattttagtgtcaactctatt
accatcaatggttggttgagaaacttattatggtccgaggcatcccaagtaatccaatcc
tatgctctttcatccatttgtccatatgggttcattttcctcatagctcatttcatctgg
gcatttagtttaatgttcctctttagtggtagagcatactggcaagaattaattgagtcc
attctatggtcacatcataaattaaagattatacctcatatccaaccaagggcattaagc
atttctcaaggaagagcagttggtttcattcattacactctaggaggtatcggctcaacc
tgggcatttattatttcaagactgctagtattaacttcttga
>Irm17_psaA
atgcacatcttcagatatatcaacacaacactgtggtccaaagcaggacattttaataaa
gctttatcaaaaggagctaagacaactacttggatatggaatctacatgattatgctcat
gattttgacattcaacaaagatccacaggtttaatagcaagaaaagtcttctcatccaat
ctagctcatctatctttggtattcttttggattagtggaatgcaccttcatggggcatat
ttgtctaattatgatatttggttaaaagatcccaaatccatcaccccatcatcacatcta
gcctactctttaattggtcaagatattcttaactcatatacctcagaatacttctcagga
atcactatcacatctggctttttccaactctaccgttctgagggtatcattacacagtcc
cagctgaaatatgcatgtgctacttccctaatagctacacttatctgcctttctggatct
tatctccacatgcagcttatgtccaaattcactagcttttacaagaagttccagtcctta
tcgcaagatcatctcataattatatttggctccagctccacatccctctccgctcatcag
atacacaaaatgcttccagctaaccctttactagattcaggtatctccaacccaagtatc
ctacaagtaatctccaacagtctcagctatacactagcactgttttcaacaaatctaagc
tctacaggtaaattgttaaatccctcaacaagaagtgtattcctttctcaagttgcagca
catcataagacaacaggtgtagtctttatcacactagggcttatcagattcctcaccatg
tataactctcaatttagtatacttacatcttatatagattaccacattgtgctatctatt
aatttagcattaattgcatctttatcaataatagtagctgatcatctcacaagaacccca
atctatcctcacaactcaacctcataccctacaatcctttgcctctccatacatcatgca
tggttatccggtttcctcatcattggttcaggagctcatgcatcaatattcaacttgcga
gcatcaccaacatctgagataagacatcgagaccccatatactcacacctcatctgggta
tgtatagcaataggattacactcattcagtctctactgtcataacgacactttagaagca
ttaggacgtccagaagatatctttcatgacaactctatccagttaaaagcaatatttgcc
aagcaatcctttctaagagcagagctccagccagatatcgagatgttagataaaaagatt
atacgtataacccaagaattgggaacagcagattttatagttcaccacattcatgcattt
acaatccatgttactctcttaattctttcaaagggtgtactttatgctagaaactctaga
tttgtatcagataaattggaacttggttttacttatccctgtgatggtccaggtagaggt
ggtacatgtcaaatatcaccttgggatcacttattctcagctgtattttggatgtataac
tgtttaaatgtagtaacattccactacttttggaagatgcaatcagatgtttggggcttt
gtatcgatccaaaagcacatatctcactacagccaaggtgattttagtgtcaactctatt
accatcaatggttggttgagaaacttattatggtccgaggcatcccaagtaatccaatcc
tatgctctttcatccatttgtccatatgggttcattttcctcatagctcatttcatctgg
gcatttagtttaatgttcctctttagtggtagagcatactggcaagaattaattgagtcc
attctatggtcacatcataaattaaagattatacctcatatccaaccaagggcattaagc
atttctcaaggaagagcagttggtttcattcattacactctaggaggtatcggctcaacc
tgggcatttattatttcaagactgctagtattaacttcttga
>Irm2_psaA
atgcacatcttcagatatatcaacacaacactgtggtccaaagcaggacattttaataaa
gctttatcaaaaggagctaagacaactacttggatatggaatctacatgattatgctcat
gattttgacattcaacaaagatccacaggtttaatagcaagaaaagtcttctcatccaat
ctagctcatctatctttggtattcttttggattagtggaatgcaccttcatggggcatat
ttgtctaattatgatatttggttaaaagatcccaaatccatcaccccatcatcacatcta
gcctactctttaattggtcaagatattcttaactcatatacctcagaatacttctcagga
atcactatcacatctggctttttccaactctaccgttctgagggtatcattacacagtcc
cagctgaaatatgcatgtgctacttccctaatagctacacttatctgcctttctggatct
tatctccacatgcagcttatgtccaaattcactagcttttacaagaagttccagtcctta
tcgcaagatcatctcataattatatttggctccagctccacatccctctccgctcatcag

atacacaaaatgcttccagctaaccctttactagattcaggtatctccaacccaagtatc
ctacaagtaatctccaacagtctcagtgaaaacctagcactgttttcaacaaatctaagc
tctacaggtaaattgttaaatccctcaacaagaagtgtattcctttctcaagttgcagca
catcataagacaacaggtgtagtctttatcacactagggcttatcagattcctcaccatg
tataactctcaatttagtatacttacatcttatatagattaccacattgtgctatctatt
aatttagcattaattgcatctttatcaataatagtagctgatcatctcacaagaacccca
atctatcctcacaactcaacctcataccctacaatcctttgcctctccatacatcatgca
tggttatccggtttcctcatcattggttcaggagctcatgcatcaatattcaacttgcga
gcatcaccaacatctgagataagacatcgagaccccatatactcacacctcatctgggta
tgtatagcaataggattacactcattcagtctctactgtcataacgacactttagaagca
ttaggacgtccagaagatatctttcatgacaactctatccagttaaaagcaatatttgcc
aagcaatcctttctaagagcagagctccagccagatatcgagatgttagataaaaagatt
atacgtataacccaagaattgggaacagcagattttatagttcaccacattcatgcattt
acaatccatgttactctcttaattctttcaaagggtgtactttatgctagaaactctaga
tttgtatcagataaattggaacttggttttacttatccctgtgatggtccaggtagaggt
ggtacatgtcaaatatcaccttgggatcacttattctcagctgtattttggatgtataac
tgtttaaatgtagtaacattccactacttttggaagatgcaatcagatgtttggggcttt
gtatcgatccaaaagcacatatctcactacagccaaggtgattttagtgtcaactctatt
accatcaatggttggttgagaaacttattatggtccgaggcatcccaagtaatccaatcc
tatgctctttcatccatttgtccatatgggttcattttcctcatagctcatttcatctgg
gcatttagtttaatgttcctctttagtggtagagcatactggcaagaattaattgagtcc
attctatggtcacatcataaattaaagattatacctcatatccaaccaagggcattaagc
atttctcaaggaagagcagttggtttcattcattacactctaggaggtatcggctcaacc
tgggcatttattatttcaagactgctagtattaacttcttga
>Irm21_psaA
atgcacatcttcagatatatcaacacaacactgtggtccaaagcaggacattttaataaa
gctttatcaaaaggagctaagacaactacttggatatggaatctacatgattatgctcat
gattttgacattcaacaaagatccacaggtttaatagcaagaaaagtcttctcatccaat
ctagctcatctatctttggtattcttttggattagtggaatgcaccttcatggggcatat
ttgtctaattatgatatttggttaaaagatcccaaatccatcaccccatcatcacatcta
gcctactctttaattggtcaagatattcttaactcatatacctcagaatacttctcagga
atcactatcacatctggctttttccaactctaccgttctgagggtatcattacacagtcc
cagctcaaatatgcatgtgctacttccctaatagctacacttatctgcctttctggatct
tatctccacatgcagcttatgtccaaattcactagcttttacaagaagttccagtcctta
tcgcaagatcatctcataattatatttggctccagctccacatccctctccgctcatcag
atacacaaaatgcttccagctaaccctttactagattcaggtatctccaacccaagtatc
ctacaagtaatctccaacagtctcagtgaaaacctagcactgttttcaacaaatctaagc
tctacaggtaaattgttaaatccctcaacaagaagtgtattcctttctcaagttgcagca
catcataagacaacaggtgtagtctttatcacactagggcttatcagattcctcaccatg
tataactctcaatttagtatacttaaatcttatatagattaccacattgtgctatctatt
aatttagcattaattgcatctttatcaataatagtagctgatcatctcacaagaacccca
atctatcctcacaactcaacctcataccctacaatcctttgcctctccatacatcatgca
tggttatccggtttcctcatcattggttcaggagctcatgcatcaatattcaacttgctt
ggatcaccaacatctgagataagacatcgagaccccatatactcacacctcatctgggta
tgtatagcaataggattacactcattcagtctctactgtcataacgacactttagaagca
ttaggacgtccagaagatatctttcatgacaactctatccagttaaaagcaatatttgcc
aagcaatcctttctaagagcagagctccagccagatatcgagatgttagataaaaagatt
atacgtataacccaagaattgggaacagcagattttatagttcaccacattcatgcattt
acaatccatgttactctcttaattctttcaaagggtgtactttatgctagaaactctaga
tttgtatcagataaattggaacttggttttacttatccctgtgatggtccaggtagaggt
ggtacatgtcaaatatcaccttgggatcacttattctcagctgtattttggatgtataac
tgtttaaatgtagtaacattccactacttttggaagatgcaatcagatgtttggggcttt
gtatcgatccaaaagcacatatctcactacagccaaggtgattttagtgtcaactctatt
accatcaatggttggttgagaaacttattatggtccgaggcatcccaagtaatccaatcc
tatgctctttcatccatttgtccatatgggttcattttcctcataggtcatttcatctgg
gcatttagtttaatgttcctctttagtggtagagcatactggcaagaattaattgagtcc
attctatggtcacatcataaattaaagattatacctcatatccaaccaagggcattaagc

atttctcaaggaagagcagttggtttcattcattacactctaggaggtatcggctcaacc
tgggcatttattatttcaagactgctagtattaacttcttga
>Irm22_psaA
atgcacatcttcagatatatcaacacaacactgtgggccaaagcaggacattttaataaa
gctttatcaaaaggagctaagacaactacttggatatggaatctacatgattatgctcat
gattttgacattcaacaaagatccacaggtttaatagcaagaaaagtcttctcatccaat
ctagctcatctatctttggtattcttttggattagtggaatgcaccttcatggagcatat
ttgtctaattatgatatttggttaaaagatcccaaatccatcacgccatcatcacatcta
gcctactctttaattggtcaagatattcttaactcatatacctcagaatacttctcagga
atcactatcacatctggctttttccaactctaccgttctgagggtatcattacacagtcc
cagctgaaatatgcatgtgctacttccctaatagctacacttatctgcctttctggatct
tatctccacatgcagcttatgtccaaattcactagcttttacaagaagttccagtcctta
tcgcaagatcatctcataattatatttggctccaggtccacatccctctccgctcatcag
atacacaaaatgcttccagctaaccctttactagattcaggtatctccaagccaagtatc
ctacaagtaatctccaacagtctcagctatacactagcactgttttcaacaaatctaagc
tctacaggtaaattgttaaatccctcaacaagaagtgtattcctttctcaagttgcagca
catcataagacaacaggtgtagtctttatcacactagggcttatcagattcctcaccatg
tataagtctcaatttagtatacttacatcttatatagattaccacattgtgctatctatt
aatttagcattaattgcatctttatcaataatagtagctgatcatctcacaagaacccca
atctatcctcacaagtcaacctcataccctacaatcctttgcctctccatacatcatgca
tggttatccggtttcctcatcattggttcaggagctcatgcatcaatattcaacttgctt
ggatcaccaacatctgagataagacatcgagaccccatatactcacacctcatctgggta
tgtatagcaataggattacactcattcagtctctactgtcataacgacactttagaagca
ttaggacgtccagaagatatctttcatgacaactctatccagttaaaagcaatatttgcc
aagcaatcctttctaagagcagagctccagccagatatcgagatgttagataaaaagatt
atacgtataacccaagaattgggaacagcagattttatagttcaccacattcatgcattt
acaatccatgttactctcttaattctttcaaagggtgtactttatgctagaaactctaga
tttgtatcagataaattggaacttggttttacttatccctgtgatgggccaggtagaggt
ggtacatgtcaaatatcaccttgggatcacttattctcagctgtattttggatgtataac
tgtttaaatgtagtaacattccactacttttggaagatgcaatcagatgtttggggcttt
gtatcgatccaaaagcacatatctcactacagccaaggtgattttagtgtcaactctatt
accatcaatggttggttgagaaacttattatggtccgaggcatcccaagtaatccaatcc
tatgctctttcatccatttgtccatatgggttcattttcctcatagctcatttcatctgg
gcatttagtttaatgttcctctttagtggtagagcatactggcaagaattaattgagtcc
attctatggtcacatcataaattaaagattatacctcatatccaaccaagggcattaagc
atttctcaaggaagagcagttggtttcattcattacactctaggaggtatcggctcaacc
tgggcatttattatttcaagactgctagtattaacttcttga
>Irm23_psaA
atgaacatcttcagatatatcaacacaacactgtggtccaaagcaggacattttaataaa
gctttatcaaaaggagctaagacaactacttggatatggaatctacatgattatgctcat
gattttgacattcaacaaagatccacaggtttaatagcaagaaaagtcttctcatccaat
ctagctcatctatctttggtattcttttggattagtggaatgcaccttcatggagcatat
ttgtctaattatgatatttggttaaaagatcccaaatccatcacgccatcatcacatcta
gcctactctttaattggtcaagatattcttaactcatatacctcagaatacttctcagga
atcactatcacatctggctttttccaactctaccgttctgagggtatcattacacagtcc
cagctgaaatatgcatgtgctacttccctaatagctacacttatctgcctttctggatct
tatctccacatgcagcttatgtccaaattcactagcttttacaagaagttccagtcctta
tcgcaagatcatctcataattatatttggctccaggtccacatccctctccgctcatcag
atacacaaaatgcttccagctaaccctttactagattcaggtatctccaacccaagtatc
ctacaagtaatctccaacagtctcagtgaaaacctagcactgttttcaacaaatctaagc
tctacaggtaaattgttaaatccctcaacaagaagtgtattcctttctcaagttgcagca
catcataagacaacaggtgtagtctttatcacactagggcttatcagattcctcaccatg
tataagtctcaatttagtatacttacatcttatatagattaccacattgtgctatctatt
aatttagcattaattgcatctttatcaataatagtagctgatcatctcacaagaacccca
atctatcctcacaagtcaacctcataccctacaatcctttgcctctccatacatcatgca
tggttatccggtttcctcatcattggttcaggagctcatgcatcaatattcaacttgcga
gcatcaccaacatctgagataagacatcgagaccccatatactcacacctcatctgggta

tgtatagcaataggattacactcattcagtctctactgtcataacgacactttagaagca
ttaggacgtccagaagatatctttcatgacaactctatccagttaaaagcaatatttgcc
aagcaatcctttctaagagcagagctccagccagatatcgagatgttagataaaaagatt
atacgtataacccaagaattgggaacagcagattttatagttcaccacattcatgcattt
acaatccatgttactctcttaattctttcaaagggtgtactttatgctagaaactctaga
tttgtatcagataaattggaacttggttttacttatccctgtgatgggccaggtagaggt
ggtacatgtcaaatatcaccttgggatcacttattctcagctgtattttggatgtataac
tgtttaaatgtagtaacattccactacttttggaagatgcaatcagatgtttggggcttt
gtatcgatccaaaagcacatatctcactacagccaaggtgattttagtgtcaactctatt
accatcaatggttggttgagaaacttattatggtccgaggcatcccaagtaatccaatcc
tatgctctttcatccatttgtccatatgggttcattttcctcatagctcatttcatctgg
gcatttagtttaatgttcctctttagtggtagagcatactggcaagaattaattgagtcc
attctatggtcacatcataaattaaagattatacctcatatccaaccaagggcattaagc
atttctcaaggaagagcagttggtttcattcattacactctaggaggtatcggctcaacc
tgggcatttattatttcaagactgctagtattaacttcttga
>Irm24_psaA
atgcacatcttcagatatatcaacacaacactgtggtccaaagcaggacattttaataaa
gctttatcaaaaggagctaagacaactacttggatatggaatctacatgattatgctcat
gattttgacattcaacaaagatccacaggtttaatagcaagaaaagtcttctcatccaat
ctagctcatctatctttggtattcttttggattagtggaatgcaccttcatggggcatat
ttgtctaattatgatatttggttaaaagatcccaaatccatcaccccatcatcacatcta
gcctactctttaattggtcaagatattcttaactcatatacctcagaatacttctcagga
atcactatcacatctggctttttccaactctaccgttctgagggtatcattacacagtcc
cagctcaaatatgcatgtgctacttccctaatagctacacttatctgcctttctggatct
tatctccacatgcagcttatgtccaaattcactagcttttacaagaagttccagtcctta
tcgcaagatcatctcataattatatttggctccaggtccacatccctctccgctcatcag
atacacaaaatgcttccagctaaccctttactagattcaggtatctccaacccaagtatc
ctacaagtaatctccaacagtctcagtgaaaacctagcactgttttcaacaaatctaagc
tctacaggtaaattgttaaatccctcaacaagaagtgtattcctttctcaagttgcagca
catcataagacaacaggtgtagtctttatcacactagggcttatcagattcctcaccatg
tataagtctcaatttagtatacttacatcttatatagattaccacattgtgctatctatt
aatttagcattaattgcatctttatcaataatagtagctgatcatctcacaagaacccca
atctatcctcacaagtcaacctcataccctacaatcctttgcctctccatacatcatgca
tggttatccggtttcctcatcattggttcaggagctcatgcatcaatattcaacttgctt
ggatcaccaacatctgagataagacatcgagaccccatatactcacacctcatctgggta
tgtatagcaataggattacactcattcagtctctactgtcataacgacactttagaagca
ttaggacgtccagaagatatctttcatgacaactctatccagttaaaagcaatatttgcc
aagcaatcctttctaagagcagagctccagccagatatcgagatgttagataaaaagatt
atacgtataacccaagaattgggaacagcagattttatagttcaccacattcatgcattt
acaatccatgttactctcttaattctttcaaagggtgtactttatgctagaaactctaga
tttgtatcagataaattggaacttggttttacttatccctgtgatgggccaggtagaggt
ggtacatgtcaaatatcaccttgggatcacttattctcagctgtattttggatgtataac
tgtttaaatgtagtaacattccactacttttggaagatgcaatcagatgtttggggcttt
gtatcgatccaaaagcacatatctcactacagccaaggtgattttagtgtcaactctatt
accatcaatggttggttgagaaacttattatggtccgaggcatcccaagtaatccaatcc
tatgctctttcatccatttgtccatatgggttcattttcctcataggtcatttcatctgg
gcatttagtttaatgttcctctttagtggtagagcatactggcaagaattaattgagtcc
attctatggtcacatcataaattaaagattatacctcatatccaaccaagggcattaagc
atttctcaaggaagagcagttggtttcattcattacactctaggaggtatcggctcaacc
tgggcatttattatttcaagactgctagtattaacttcttga
>Irm25_psaA
atgcacatcttcagatatatcaacacaacactgtgggccaaagcaggacattttaataaa
gctttatcaaaaggagctaagacaactacttggatatggaatctacatgattatgctcat
gattttgacattcaacaaagatccacaggtttaatagcaagaaaagtcttctcatccaat
ctagctcatctatctttggtattcttttggattagtggaatgcaccttcatggagcatat
ttgtctaattatgatatttggttaaaagatcccaaatccatcacgccatcatcacatcta
gcctactctttaattggtcaagatattcttaactcatatacctcagaatacttctcagga

atcactatcacatctggctttttccaactctaccgttctgagggtatcattacacagtcc
cagctcaaatatgcatgtgctacttccctaatagctacacttatctgcctttctggatct
tatctccacatgcagcttatgtccaaattcactagcttttacaagaagttccagtcctta
tcgcaagatcatctcataattatatttggctccagctccacatccctctccgctcatcag
atacacaaaatgcttccagctaaccctttactagattcaggtatctccaagccaagtatc
ctacaagtaatctccaacagtctcagtgaaaacctagcactgttttcaacaaatctaagc
tctacaggtaaattgttaaatccctcaacaagaagtgtattcctttctcaagttgcagca
catcataagacaacaggtgtagtctttatcacactagggcttatcagattcctcaccatg
tataactctcaatttagtatacttacatcttatatagattaccacattgtgctatctatt
aatttagcattaattgcatctttatcaataatagtagctgatcatctcacaagaacccca
atctatcctcacaactcaacctcataccctacaatcctttgcctctccatacatcatgca
tggttatccggtttcctcatcattggttcaggagctcatgcatcaatattcaacttgcga
gcatcaccaacatctgagataagacatcgagaccccatatactcacacctcatctgggta
tgtatagcaataggattacactcattcagtctctactgtcataacgacactttagaagca
ttaggacgtccagaagatatctttcatgacaactctatccagttaaaagcaatatttgcc
aagcaatcctttctaagagcagagctccagccagatatcgagatgttagataaaaagatt
atacgtataacccaagaattgggaacagcagattttatagttcaccacattcatgcattt
acaatccatgttactctcttaattctttcaaagggtgtactttatgctagaaactctaga
tttgtatcagataaattggaacttggttttacttatccctgtgatgggccaggtagaggt
ggtacatgtcaaatatcaccttgggatcacttattctcagctgtattttggatgtataac
tgtttaaatgtagtaacattccactacttttggaagatgcaatcagatgtttggggcttt
gtatcgatccaaaagcacatatctcactacagccaaggtgattttagtgtcaactctatt
accatcaatggttggttgagaaacttattatggtccgaggcatcccaagtaatccaatcc
tatgctctttcatccatttgtccatatgggttcattttcctcatagctcatttcatctgg
gcatttagtttaatgttcctctttagtggtagagcatactggcaagaattaattgagtcc
attctatggtcacatcataaattaaagattatacctcatatccaaccaagggcattaagc
atttctcaaggaagagcagttggtttcattcattacactctaggaggtatcggctcaacc
tgggcatttattatttcaagactgctagtattaacttcttga
>Irm26_psaA
atgcacatcttcagatatatcaacacaacactgtggtccaaagcaggacattttaataaa
gctttatcaaaaggagctaagacaactacttggatatggaatctacatgattatgctcat
gattttgacattcaacaaagatccacaggtttaatagcaagaaaagtcttctcatccaat
ctagctcatctatctttggtattcttttggattagtggaatgcaccttcatggggcatat
ttgtctaattatgatatttggttaaaagatcccaaatccatcaccccatcatcacatcta
gcctactctttaattggtcaagatattcttaactcatatacctcagaatacttctcagga
atcactatcacatctggctttttccaactctaccgttctgagggtatcattacacagtcc
cagctgaaatatgcatgtgctacttccctaatagctacacttatctgcctttctggatct
tatctccacatgcagcttatgtccaaattcactagcttttacaagaagttccagtcctta
tcgcaagatcatctcataattatatttggctccagctccacatccctctccgctcatcag
atacacaaaatgcttccagctaaccctttactagattcaggtatctccaacccaagtatc
ctacaagtaatctccaacagtctcagctatacactagcactgttttcaacaaatctaagc
tctacaggtaaattgttaaatccctcaacaagaagtgtattcctttctcaagttgcagca
catcataagacaacaggtgtagtctttatcacactagggcttatcagattcctcaccatg
tataactctcaatttagtatacttacatcttatatagattaccacattgtgctatctatt
aatttagcattaattgcatctttatcaataatagtagctgatcatctcacaagaacccca
atctatcctcacaagtcaacctcataccctacaatcctttgcctctccatacatcatgca
tggttatccggtttcctcatcattggttcaggagctcatgcatcaatattcaacttgcga
gcatcaccaacatctgagataagacatcgagaccccatatactcacacctcatctgggta
tgtatagcaataggattacactcattcagtctctactgtcataacgacactttagaagca
ttaggacgtccagaagatatctttcatgacaactctatccagttaaaagcaatatttgcc
aagcaatcctttctaagagcagagctccagccagatatcgagatgttagataaaaagatt
atacgtataacccaagaattgggaacagcagattttatagttcaccacattcatgcattt
acaatccatgttactctcttaattctttcaaagggtgtactttatgctagaaactctaga
tttgtatcagataaattggaacttggttttacttatccctgtgatggtccaggtagaggt
ggtacatgtcaaatatcaccttgggatcacttattctcagctgtattttggatgtataac
tgtttaaatgtagtaacattccactacttttggaagatgcaatcagatgtttggggcttt
gtatcgatccaaaagcacatatctcactacagccaaggtgattttagtgtcaactctatt

accatcaatggttggttgagaaacttattatggtccgaggcatcccaagtaatccaatcc
tatgctctttcatccatttgtccatatgggttcattttcctcatagctcatttcatctgg
gcatttagtttaatgttcctctttagtggtagagcatactggcaagaattaattgagtcc
attctatggtcacatcataaattaaagattatacctcatatccaaccaagggcattaagc
atttctcaaggaagagcagttggtttcattcattacactctaggaggtatcggctcaacc
tgggcatttattatttcaagactgctagtattaacttcttga
>Irm27_psaA
atgcacatcttcagatatatcaacacaacactgtggtccaaagcaggacactttaataaa
gctttatcaaaaggagctaagacaactacttggatatggaatctacatgattatgcacat
gattttgatattcaacaaagctccgcaggtttaatagcaagaaaagtattctcatccaac
ctagcccatttatctttggtattcttttggattagtggaatgcaccttcatggagcatat
ttgtctaattatgatatttggttaaaagatcccaaatccatcacgccatcatcacatcta
gcctactctttaattggtcaagatattcttaactcatatacctcagaatacttctcagga
atcactatcacatctggctttttccaactctaccgttctgagggtataattacacagtct
cagctgaaatatgcatgtgctacttccctaatagctacacttatctgcctttctggatct
tatctccacatgcagcttatgtccaaattcactagcttttacaagaagttccagtcctta
tcgcaagatcatctcataattatatttggctccagatctatatctctctccgctcatcag
atacacaaaatgcttccagctaactatcaactagattcaggcatctccaatccaagtatc
ctacaagtaatctccaacagtctcagctatacactagcactgttttcaacaaatctaagc
tctacaggtaaattgttaaatccctcaacaagaagtgtattcctttctcaagttgcagca
catcataagacaacaggtgtagtctttatcacactaggcttatcagattcctcaccatg
tataagtctcaatttagtatacttacatcttatatagattaccaccttgtcctatctact
aacttagcattaattggatctttatcaataataacagctgatcatctcacaagaacccca
atctatcctcacaagtcaacctcataccctacaatcctttgcctctccatacatcatgca
tggttatccggtttcctcatcattggttcaggagctcatgcatcaatattcaacttgctt
ggatcaccaacatctgagataagacatcgagaccccatatactcacacctcatctgggta
tgtatagcaataggattacactcattcagtctctactgtcataacgacactttagaagca
ttaggacgtccagaagatatctttcatgacaactctatccagttaaaagcaatatttgcc
aagcaatcctttctaagagcagagctccagccagatatcgagatgttagataaaaagatt
atacgtataacccaagaattgggaacagcagattttatagttcaccacattcatgcattt
acaatccatgttactctcttaattctttcaaagggtgtactttatgctagaaagtctaga
tttgtatcagataaattggaacttggttttacttatccttgtgatgggccaggtagaggt
ggtacatgtcaaatatcaccttgggatcacttattctcagctgtattttggatgtataat
tgtttaaatatagtagctttccacttcttttggaagatgcaatcagatatttggggcttt
gtatcgatccaaaagcacatatctcactacagccaaggtgattttagtgtcaactctatt
accatcaatggttggttgagaaacttattatggtccgaggcatcccaagtaatccaatcc
tatgctctttcatccatttgtccatatgggttcattttcctcataggtcatttcatctgg
gcatttagtttaatgttcctctttagtggtagagcatactggcaagaattaattgagtcc
attctatggtcacatcataaattaaagattatacctcatatccaaccaagggcattaagc
atttctcaaggaagagcagttggtttcattcattacactctaggaggtatcggctcaacc
tgggcatttattatttcaagactgctagtattaacttcttga
>Irm3_psaA
atgcacatcttcagatatatcaatactacactgtggtccaaagcaggacactttaataaa
gctttatcaaaaggagctaagacaactacttggatatggaatctacatgattatgcacat
gattttgatattcaacaaagctccgcaggtttaatagcaagaaaagtattctcatccaac
ctagcccatttatctttggtattcttttggattagtggaatgcaccttcatggtgcatac
ttgtctaattatgatatttggttaaaagatcctaaatctataagtccttcatcacagcta
gcttactctttagttggccaagatattcttaactcatatacctcagaatacttctcagga
atcactattacttcaggactatttcaactctaccgttctgagggtataattacacagtct
cagctgaaatatgcatgtgctacttccctaatagctacacttatctgtcttactggatct
tatctccacatgcaagtgatgtccaaatttagtagcttttataagaagttccagtcctta
tcgcaagatcatctcataattatctttggctccaggtccacatccctctccgctcatcag
atacacaaaatgcttccagctaaccctttactagattcaggtatctccaagccaagtatc
ctacaagtaatctccaacagtctcagctatacactagcactgttttcaacaaatctaagc
tctacaggcaaattgttaaatccctcaacaagaagtatattcctttctcaagttgcagca
catcataagacaataggtatagtacttatcacactaggcttatcagattcctcaccatt
tataattctcaatttagtatacttacatcttatatagattaccaccttgtcctatctact

aacttagcattaattggatctttatcaataatagtagctgatcatctcacaagaacccca
atctatcctcacaagtcaacctcataccctacaatcctttgcctctccatacatcatgca
tggttatccggtttcctcatcattggttcaggagctcatgcatcaatattcaacttgctt
ggatcaccaacatctgagataagacatcgagaccccatatactcacacctcatctggata
tgtatagctttaggattacactcatttagtctctactgccataacgacactttagaagct
ttaggacgtccagaagatatctttcacgataactctatccagttaaaagctatatttgct
aagcaatcctttctaagagcagagctccagccagatatcgagatcttagataaaaagatt
atacgtataactcaagaattgggaacagctgattttatagttcaccacattcatgcattt
acaatccatgttactctcttaattctttcaaagggtgtactttatgctagaaagtctaga
tttgtatcagataaattggaacttggttttacttatccttgtgatgggccaggtagaggt
ggtacatgtcaaatatcaccttgggatcacttattctcagctgtattttggatgtataat
tgtttaaatatagtagctttccacttcttttggaagatgcaatcagatatttggggcttt
gtatccatccaaaggcaaatttcccactacagccaaggtgattttagtgtaaattctatt
accatcaacggctggttgagaaacttattatggtctcaggcatctcaagtaatacaatcc
tatgctcttccatctatttgtacatacgggttcattttcctcataggtcatttcatctgg
gcatttagtttaatgttcctatttagtggtagagcatactggcaagaattaattgagtct
attctatggtcccaccataaattaaagattatacctcatatccaacctagggccttaagc
atttctcaaggaagagcagttggcttcactcattatactctaggtggtatcggctcaacc
tgggcatttattatttcaagactgctagtattaacttcttga
>Irm4_psaA
atgcacatcttcagatatatcaacacaacactgtggtccaaagcaggacattttaataaa
gctttatcaaaaggagctaagacaactacttggatatggaatctacatgattatgcacat
gattttgatattcaacaaagctccgcaggtttaatagcaagaaaagtattctcatccaac
ctagcccatttatctttggtattcttttggattagtggaatgcaccttcatggtgcatac
ttgtctaattatgatatttggttaaaagatcctaaatctataagtccttcatcacagcta
gcttactctttaattggtcaagatattcttaactcatatacctcagaatacttctcagga
atcactatcacatctggctttttccaactctaccgttctgagggtatcattacacagtcc
cagctcaaatatgcatgtgctacttccctaatagctacacttatctgcctttctggatct
tatctccacatgcagcttatgtccaaattcactagcttttacaagaagttccagtcctta
tcgcaagatcatctcataattatatttggctccagctctatatctctctccgctcatcag
atacacaaaatgcttccagctaaccctttactagattcaggtatctccaacccaagtatc
ctacaagtaatctccaacagtctcagtgaaaacctagcactgttttcaacaaatctaagc
tctacaggcaaattgttaaatccctcaacaagaagtatattcctttctcaagttgcagca
catcataagacaacaggtgtagtctttatcacactagggcttatcagattcctcaccatg
tataactctcaatttagtatacttacatcttatatagattaccacattgtgctatctatt
aatttagcattaattgcatctttatcaataatagtagctgatcatctcacaagaacccca
atctatcctcacaactcaacctcataccctacaatcctttgcctctccatacatcatgca
tggttatccggtttcctcatcattggttcaggagctcatgcatcaatattcaacttgctt
ggatcaccaacatctgagataagacatcgagaccccatatactcacacctcatctggata
tgtatagcaataggattacactcattcagtctctactgtcataacgacactttagaagca
ttaggacgtccagaagatatctttcatgacaactctatccagttaaaagcaatatttgcc
aagcaatcctttctaagagcagagctccagccagatatcgagatcttagataaaaagatt
atacgtataactcaagaattgggaacagctgattttatagttcaccacattcatgcattt
acaatccatgttactctcttaattctttcaaagggtgtactttatgctagaaagtctaga
tttgtatcagataaattggaacttggttttacttatccttgtgatgggccaggtagaggt
ggtacatgtcaaatatcaccttgggatcacttattctcagctgtattttggatgtataat
tgtttaaatatagtagctttccacttcttttggaagatgcaatcagatatttggggcttt
gtatccatccaaaagcacatatctcactacagccaaggtgattttagtgtcaactctatt
accatcaacggctggttgagaaacttattatggtctcaggcatctcaagtaatacaatcc
tatgctcttccatctatttgtccatatgggttcattttcctcatagctcatttcatctgg
gcatttagtttaatgttcctatttagtggtagagcatactggcaagaattaattgagtct
attctatggtcacaccataaattaaagattatacctcatatccaacctagggccttaagc
atttctcaaggaagagcagttggcttcactcattatactctaggtggtatcggctcaacc
tgggcatttattatttcaagactgctagtattaacttcttga
>Irm5_psaA
atgcacatcttcagatatatcaacacaacactgtggtccaaagcaggacattttaataaa
gctttatcaaaaggagctaagacaactacttggatatggaatctacatgattatgctcat

gattttgacattcaacaaagatccacaggtttaatagcaagaaaagtcttctcatccaat
ctagctcatctatctttggtattcttttggattagtggaatgcaccttcatggggcatat
ttgtctaattatgatatttggttaaaagatcccaaatccatcaccccatcatcacatcta
gcctactctttaattggtcaagatattcttaactcatatacctcagaatacttctcagga
atcactatcacatctggctttttccaactctaccgttctgagggtatcattacacagtcc
cagctgaaatatgcatgtgctacttccctaatagctacacttatctgcctttctggatct
tatctccacatgcagcttatgtccaaattcactagcttttacaagaagttccagtcctta
tcgcaagatcatctcataattatatttggctccagctccacatccctctccgctcatcag
atacacaaaatgcttccagctaaccctttactagattcaggtatctccaacccaagtatc
ctacaagtaatctccaacagtctcagtgaaaacctagcactgttttcaacaaatctaagc
tctacaggtaaattgttaaatccctcaacaagaagtgtattcctttctcaagttgcagca
catcataagacaacaggtgtagtctttatcacactagggcttatcagattcctcaccatg
tataactctcaatttagtatacttacatcttatatagattaccacattgtgctatctatt
aatttagcattaattgcatctttatcaataatagtagctgatcatctcacaagaacccca
atctatcctcacaactcaacctcataccctacaatcctttgcctctccatacatcatgca
tggttatccggtttcctcatcattggttcaggagctcatgcatcaatattcaacttgcga
gcatcaccaacatctgagataagacatcgagaccccatatactcacacctcatctgggta
tgtatagcaataggattacactcattcagtctctactgtcataacgacactttagaagca
ttaggacgtccagaagatatctttcatgacaactctatccagttaaaagcaatatttgcc
aagcaatcctttctaagagcagagctccagccagatatcgagatgttagataaaaagatt
atacgtataacccaagaattgggaacagcagattttatagttcaccacattcatgcattt
acaatccatgttactctcttaattctttcaaagggtgtactttatgctagaaactctaga
tttgtatcagataaattggaacttggttttacttatccctgtgatggtccaggtagaggt
ggtacatgtcaaatatcaccttgggatcacttattctcagctgtattttggatgtataac
tgtttaaatgtagtaacattccactacttttggaagatgcaatcagatgtttggggcttt
gtatcgatccaaaagcacatatctcactacagccaaggtgattttagtgtcaactctatt
accatcaatggttggttgagaaacttattatggtccgaggcatcccaagtaatccaatcc
tatgctctttcatccatttgtccatatgggttcattttcctcatagctcatttcatctgg
gcatttagtttaatgttcctctttagtggtagagcatactggcaagaattaattgagtcc
attctatggtcacatcataaattaaagattatacctcatatccaaccaagggcattaagc
atttctcaaggaagagcagttggtttcattcattacactctaggaggtatcggctcaacc
tgggcatttattatttcaagactgctagtattaacttcttga
>Irm7_psaA
atgcacatcttcagatatatcaacacaacactgtggtccaaagcaggacattttaataaa
gctttatcaaaaggagctaagacaactacttggatatggaatctacatgattatgctcat
gattttgacattcaacaaagatccacaggtttaatagcaagaaaagtcttctcatccaat
ctagctcatctatctttggtattcttttggattagtggaatgcaccttcatggagcatat
ttgtctaattatgatatttggttaaaagatcccaaatccatcacgccatcatcacatcta
gcctactctttaattggtcaagatattcttaactcatatacctcagaatacttctcagga
atcactatcacatctggctttttccaactctaccgttctgagggtatcattacacagtcc
cagctgaaatatgcatgtgctacttccctaatagctacacttatctgcctttctggatct
tatctccacatgcagcttatgtccaaattcactagcttttacaagaagttccagtcctta
tcgcaagatcatctcataattatatttggctccagctccacatccctctccgctcatcag
atacacaaaatgcttccagctaaccctttactagattcaggtatctccaacccaagtatc
ctacaagtaatctccaacagtctcagtgaaaacctagcactgttttcaacaaatctaagc
tctacaggtaaattgttaaatccctcaacaagaagtgtattcctttctcaagttgcagca
catcataagacaacaggtgtagtctttatcacactagggcttatcagattcctcaccatg
tataactctcaatttagtatacttacatcttatatagattaccacattgtgctatctatt
aatttagcattaattgcatctttatcaataatagtagctgatcatctcacaagaacccca
atctatcctcacaagtcaacctcataccctacaatcctttgcctctccatacatcatgca
tggttatccggtttcctcatcattggttcaggagctcatgcatcaatattcaacttgctt
ggatcaccaacatctgagataagacatcgagaccccatatactcacacctcatctgggta
tgtatagcaataggattacactcattcagtctctactgtcataacgacactttagaagca
ttaggacgtccagaagatatctttcatgacaactctatccagttaaaagcaatatttgcc
aagcaatcctttctaagagcagagctccagccagatatcgagatgttagataaaaagatt
atacgtataacccaagaattgggaacagcagattttatagttcaccacattcatgcattt
acaatccatgttactctcttaattctttcaaagggtgtactttatgctagaaactctaga

tttgtatcagataaattggaacttggttttacttatccctgtgatggtccaggtagaggt
ggtacatgtcaaatatcaccttgggatcacttattctcagctgtattttggatgtataac
tgtttaaatgtagtaacattccactacttttggaagatgcaatcagatgtttggggcttt
gtatcgatccaaaagcacatatctcactacagccaaggtgattttagtgtcaactctatt
accatcaatggttggttgagaaacttattatggtccgaggcatcccaagtaatccaatcc
tatgctctttcatccatttgtccatatgggttcattttcctcatagctcatttcatctgg
gcatttagtttaatgttcctctttagtggtagagcatactggcaagaattaattgagtcc
attctatggtcacatcataaattaaagattatacctcatatccaaccaagggcattaagc
atttctcaaggaagagcagttggtttcattcattacactctaggaggtatcggctcaacc
tgggcatttattatttcaagactgctagtattaacttcttga
>Irm9_psaA
atgcacatcttcagatatatcaacacaacactgtgggccaaagcaggacattttaataaa
gctttatcaaaaggagctaagacaactacttggatatggaatctacatgattatgctcat
gattttgacattcaacaaagatccacaggtttaatagcaagaaaagtcttctcatccaat
ctagctcatctatctttggtattcttttggattagtggaatgcaccttcatggagcatat
ttgtctaattatgatatttggttaaaagatcccaaatccatcacgccatcatcacatcta
gcctactctttaattggtcaagatattcttaactcatatacctcagaatacttctcagga
atcactatcacatctggctttttccaactctaccgttctgagggtataattacacagtct
cagctgaaatatgcatgtgctacttccctaatagctacacttatctgtcttactggatct
tatctccacatgcaagtgatgtccaaatttactagcttttacaagaagttccagtcctta
tcgcaagatcatctcataattatatttggctccagatctatatctctctccgctcatcag
atacacaaaatgcttccagctaactatcaactagattcaggcatctccaatccaagtatc
ctacaagtaatctccaacagtctcagctatacactagcactgttttcaacaaatctaagc
tctacaggcaaattgttaaatccctcaacaagaagtatattcctttctcaagttgcagca
catcataagacaataggtatagtacttatcacactagggcttatcagattcctcaccatt
tataattctcaatttagtatacttacatcttatatagattaccacattgtgctatctatt
aatttagcattaattgcatctttatcaataatagtagctgatcatctcacaagaacccca
atctatcctcacaagtcaacctcataccctacaatcctttgcctctccatacatcatgca
tggttatccggtttcctcatcattggttcaggagctcatgcatcaatattcaacttgctt
ggatcaccaacatctgagataagacatcgagaccccatatactcacacctcatctgggta
tgtatagcaataggattacactcatttagtctctactgccataacgacactttagaagct
ttaggacgtccagaagatatctttcacgataactctatccagttaaaagctatatttgcc
aagcaatcctttctaagagcagagctccagccagatatcgagatgttagataaaaagatt
atacgtataacccaagaattgggaacagcagattttatagttcaccacattcatgcattt
acaatccatgttactctcttaattctttcaaagggtgtactttatgctagaaactctaga
tttgtatcagataaattggaacttggttttacttatccttgtgatgggccaggtagaggt
ggtacatgtcaaatatcaccttgggatcacttattctcagctgtattttggatgtataat
tgtttaaatatagtagctttccacttcttttggaagatgcaatcagatatttggggcttt
gtatccatccaaaggcaaatttcccactacagccaaggtgattttagtgtaaattctatt
accatcaacggctggttgagaaacttattatggtctcaggcatctcaagtaatacaatcc
tatgctcttccatctatttgtacatacggctttattttcctcacagctcatttcatatgg
gcattctctttaatgttcctatttagtggtagagcatactggcaagaattaattgagtct
attctatggtcacatcataaattaaagattatacctcatatccaaccaagggcattaagc
atttctcaaggaagagcagttggtttcattcattacactctaggaggtatcggctcaacc
tgggcatttattatttcaagactgctagtattaacttcttga
>Isy12_psaA
atgcacatcttcagatatatcaacacaacactgtggtccaaagcaggacattttaataaa
gctttatcaaaaggagctaagacaactacttggatatggaatctacatgattatgctcat
gattttgacattcaacaaagatccacaggtttaatagcaagaaaagtcttctcatccaat
ctagctcatctatctttggtattcttttggattagtggaatgcaccttcatggggcatat
ttgtctaattatgatatttggttaaaagatcccaaatccatcaccccatcatcacatcta
gcctactctttaattggtcaagatattcttaactcatatacctcagaatacttctcagga
atcactatcacatctggctttttccaactctaccgttctgagggtatcattacacagtcc
cagctgaaatatgcatgtgctacttccctaatagctacacttatctgcctttctggatct
tatctccacatgcagcttatgtccaaattcactagcttttacaagaagttccagtcctta
tcgcaagatcatctcataattatatttggctccaggtccacatccctctccgctcatcag
atacacaaaatgcttccagctaaccctttactagattcaggtatctccaacccaagtatc

ctacaagtaatctccaacagtctcagtgaaaacctagcactgttttcaacaaatctaagc
tctacaggtaaattgttaaatccctcaacaagaagtgtattcctttctcaagttgcagca
catcataagacaacaggtgtagtctttatcacactagggcttatcagattcctcaccatg
tataactctcaatttagtatacttacatcttatatagattaccacattgtgctatctatt
aatttagcattaattgcatctttatcaataatagtagctgatcatctcacaagaacccca
atctatcctcacaactcaacctcataccctacaatcctttgcctctccatacatcatgca
tggttatccggtttcctcatcattggttcaggagctcatgcatcaatattcaacttgcga
gcatcaccaacatctgagataagacatcgagaccccatatactcacacctcatctgggta
tgtatagcaataggattacactcattcagtctctactgtcataacgacactttagaagca
ttaggacgtccagaagatatctttcatgacaactctatccagttaaaagcaatatttgcc
aagcaatcctttctaagagcagagctccagccagatatcgagatgttagataaaaagatt
atacgtataacccaagaattgggaacagcagattttatagttcaccacattcatgcattt
acaatccatgttactctcttaattctttcaaagggtgtactttatgctagaaactctaga
tttgtatcagataaattggaacttggttttacttatccctgtgatgggccaggtagaggt
ggtacatgtcaaatatcaccttgggatcacttattctcagctgtattttggatgtataac
tgtttaaatgtagtaacattccactacttttggaagatgcaatcagatgtttggggcttt
gtatcgatccaaaagcacatatctcactacagccaaggtgattttagtgtcaactctatt
accatcaatggttggttgagaaacttattatggtccgaggcatcccaagtaatccaatcc
tatgctctttcatccatttgtccatatgggttcattttcctcatagctcatttcatctgg
gcatttagtttaatgttcctctttagtggtagagcatactggcaagaattaattgagtcc
attctatggtcacatcataaattaaagattatacctcatatccaaccaagggcattaagc
atttctcaaggaagagcagttggtttcattcattacactctaggaggtatcggctcaacc
tgggcatttattatttcaagactgctagtattaacttcttga
>Isy15_psaA
atgcacatctttagatatatcaatactacactgtggtccaaagcaggacactttaataaa
gctttatcaaaaggagctaagacaactacttggatatggaatctacatgattatgcacat
gattttgatattcaacaaagctccacaggtttaatagcaagaaaagtattctcatccaac
ctagcccatttatctttggtattcttttggattagtggaatgcaccttcatggtgcatac
ttgtctaattatgatatttggttaaaagatcctaaatctataagtccttcatcacagcta
gcttactctttagttggccaagatattcttaactcatatacctcagaatacttctcagga
atcactattacttcaggactatttcaactctaccgttctgagggtataattacacagtct
cagctgaaatatgcatgtgctacttccctaatagctacacttatctgtcttactggatct
tatctccacatgcaagtgatgtccaaatttagtagcttttataagaagttccagtcctta
tcgcaagatcatctcataattatctttggctccagatctatatctctctccgctcatcag
atacacaaaatgcttccagctaactatcaactagattcaggcatctccaatccaagtatc
ctacaagtaatctccaacagtctcagctatacactagcactgttttcaacaaatctaagc
tctacaggtaaattgttaaatccctcaacaagaagtatattcctttctcaagttgcagca
catcataagacaataggtatagtacttatcacactagggcttatcagattcctcaccatt
tataattctcaatttagtatacttacatcttatatagattaccaccttgtcctatctact
aacttagcattaattggatctttatcaataatagtagctgatcatctcacaagaacccca
atctatcctcacaactcaacctcataccctacaatcctttgcctctccatacatcatgca
tggttatccggtttcctcatcattggttcaggagctcatgcatcaatattcaacttgcga
gcatcaccaacatctgagataagacatcgagaccccatatactcacacctcatctggata
tgtatagctttaggattacactcatttagtctctactgccataacgacactttagaagct
ttaggacgtccagaagatatctttcacgataactctatccagttaaaagctatatttgct
aagcaatcctttctaagagcagagctccagccagatatcgagatcttagataaaaagatt
atacgtataactcaagaattgggaacagctgattttatagttcaccacattcatgcattt
acaatccatgttactctcttaattctttcaaagggtgtactttatgctagaaagtctaga
tttgtatcagataaattggaacttggttttacttatccttgtgatgggccaggtagaggt
ggtacatgtcaaatatcaccttgggatcacttattctcagctgtattttggatgtataat
tgtttaaatatagtagctttccacttcttttggaagatgcaatcagatatttggggcttt
gtatccatccaaaggcaaatttcccactacagccaaggtgattttagtgtcaattctatt
accatcaacggctggttgagaaacttattatggtctcaggcatctcaagtaatccaatcc
tatgctctttcatccatttgtccatatgggttcattttcctcatagctcatttcatctgg
gcattctgtttaatgttcctatttagtggtagagcatactggcaagaattaattgagtct
attctatggtcccaccataaattaaagattatacctcatatccaacctagggccttaagc
atttctcaaggaagagcagttggcttcactcattatactctaggtggtatcggctcaacc

tgggcatttattatttcaagactgctagtattaacttcttga
>Isy16_psaA
atgcacatcttcagatatatcaacacaacactgtggtccaaagcaggacattttaataaa
gctttatcaaaaggagctaagacaactacttggatatggaatctacatgattatgctcat
gattttgacattcaacaaagatccacaggtttaatagcaagaaaagtcttctcatccaat
ctagctcatctatctttggtattcttttggattagtggaatgcaccttcatggggcatat
ttgtctaattatgatatttggttaaaagatcccaaatccatcaccccatcatcacatcta
gcctactctttaattggtcaagatattcttaactcatatacctcagaatacttctcagga
atcactatcacatctggctttttccaactctaccgttctgagggtatcattacacagtcc
cagctgaaatatgcatgtgctacttccctaatagctacacttatctgcctttctggatct
tatctccacatgcagcttatgtccaaattcactagcttttacaagaagttccagtcctta
tcgcaagatcatctcataattatatttggctccagctccacatccctctccgctcatcag
atacacaaaatgcttccagctaaccctttactagattcaggtatctccaacccaagtatc
ctacaagtaatctccaacagtctcagtgaaaacctagcactgttttcaacaaatctaagc
tctacaggtaaattgttaaatccctcaacaagaagtgtattcctttctcaagttgcagca
catcataagacaacaggtgtagtctttatcacactagggcttatcagattcctcaccatg
tataactctcaatttagtatacttacatcttatatagattaccacattgtgctatctatt
aatttagcattaattgcatctttatcaataatagtagctgatcatctcacaagaacccca
atctatcctcacaactcaacctcataccctacaatcctttgcctctccatacatcatgca
tggttatccggtttcctcatcattggttcaggagctcatgcatcaatattcaacttgcga
gcatcaccaacatctgagataagacatcgagaccccatatactcacacctcatctgggta
tgtatagcaataggattacactcattcagtctctactgtcataacgacactttagaagca
ttaggacgtccagaagatatctttcatgacaactctatccagttaaaagcaatatttgcc
aagcaatcctttctaagagcagagctccagccagatatcgagatgttagataaaaagatt
atacgtataacccaagaattgggaacagcagattttatagttcaccacattcatgcattt
acaatccatgttactctcttaattctttcaaagggtgtactttatgctagaaactctaga
tttgtatcagataaattggaacttggttttacttatccctgtgatggtccaggtagaggt
ggtacatgtcaaatatcaccttgggatcacttattctcagctgtattttggatgtataac
tgtttaaatgtagtaacattccactacttttggaagatgcaatcagatgtttggggcttt
gtatcgatccaaaagcacatatctcactacagccaaggtgattttagtgtcaactctatt
accatcaatggttggttgagaaacttattatggtccgaggcatcccaagtaatccaatcc
tatgctctttcatccatttgtccatatgggttcattttcctcatagctcatttcatctgg
gcatttagtttaatgttcctctttagtggtagagcatactggcaagaattaattgagtcc
attctatggtcacatcataaattaaagattatacctcatatccaaccaagggcattaagc
atttctcaaggaagagcagttggtttcattcattacactctaggaggtatcggctcaacc
tgggcatttattatttcaagactgctagtattaacttcttga
>Isy17_psaA
atgcacatcttcagatatatcaacacaacactgtggtccaaagcaggacattttaataaa
gctttatcaaaaggagctaagacaactacttggatatggaatctacatgattatgctcat
gattttgacattcaacaaagatccacaggtttaatagcaagaaaagtcttctcatccaat
ctagctcatctatctttggtattcttttggattagtggaatgcaccttcatggggcatat
ttgtctaattatgatatttggttaaaagatcccaaatccatcaccccatcatcacatcta
gcctactctttaattggtcaagatattcttaactcatatacctcagaatacttctcagga
atcactatcacatctggctttttccaactctaccgttctgagggtatcattacacagtcc
cagctgaaatatgcatgtgctacttccctaatagctacacttatctgcctttctggatct
tatctccacatgcagcttatgtccaaattcactagcttttacaagaagttccagtcctta
tcgcaagatcatctcataattatatttggctccagctccacatccctctccgctcatcag
atacacaaaatgcttccagctaaccctttactagattcaggtatctccaagccaagtatc
ctacaagtaatctccaacagtctcagtgaaaacctagcactgttttcaacaaatctaagc
tctacaggtaaattgttaaatccctcaacaagaagtgtattcctttctcaagttgcagca
catcataagacaacaggtgtagtctttatcacactagggcttatcagattcctcaccatg
tataactctcaatttagtatacttacatcttatatagattaccacattgtgctatctatt
aatttagcattaattgcatctttatcaataatagtagctgatcatctcacaagaacccca
atctatcctcacaactcaacctcataccctacaatcctttgcctctccatacatcatgca
tggttatccggtttcctcatcattggttcaggagctcatgcatcaatattcaacttgcga
gcatcaccaacatctgagataagacatcgagaccccatatactcacacctcatctgggta
tgtatagcaataggattacactcattcagtctctactgtcataacgacactttagaagca

ttaggacgtccagaagatatctttcatgacaactctatccagttaaaagcaatatttgcc
aagcaatcctttctaagagcagagctccagccagatatcgagatgttagataaaaagatt
atacgtataacccaagaattgggaacagcagattttatagttcaccacattcatgcattt
acaatccatgttactctcttaattctttcaaagggtgtactttatgctagaaactctaga
tttgtatcagataaattggaacttggttttacttatccctgtgatggtccaggtagaggt
ggtacatgtcaaatatcaccttgggatcacttattctcagctgtattttggatgtataac
tgtttaaatgtagtaacattccactacttttggaagatgcaatcagatgtttggggcttt
gtatcgatccaaaagcacatatctcactacagccaaggtgattttagtgtcaactctatt
accatcaatggttggttgagaaacttattatggtccgaggcatcccaagtaatccaatcc
tatgctctttcatccatttgtccatatgggttcattttcctcatagctcatttcatctgg
gcatttagtttaatgttcctctttagtggtagagcatactggcaagaattaattgagtcc
attctatggtcacatcataaattaaagattatacctcatatccaaccaagggcattaagc
atttctcaaggaagagcagttggtttcattcattacactctaggaggtatcggctcaacc
tgggcatttattatttcaagactgctagtattaacttcttga
>Isy18_psaA
atgcacatcttcagatatatcaacacaacactgtgggccaaagcaggacattttaataaa
gctttatcaaaaggagctaagacaactacttggatatggaatctacatgattatgctcat
gattttgacattcaacaaagatccacaggtttaatagcaagaaaagtcttctcatccaat
ctagctcatctatctttggtattcttttggattagtggaatgcaccttcatggggcatat
ttgtctaattatgatatttggttaaaagatcccaaatccatcaccccatcatcacatcta
gcctactctttaattggtcaagatattcttaactcatatacctcagaatacttctcagga
atcactatcacatctggctttttccaactctaccgttctgagggtatcattacacagtcc
cagctgaaatatgcatgtgctacttccctaatagctacacttatctgcctttctggatct
tatctccacatgcagcttatgtccaaattcactagcttttacaagaagttccagtcctta
tcgcaagatcatctcataattatatttggctccagctccacatccctctccgctcatcag
atacacaaaatgcttccagctaaccctttactagattcaggtatctccaacccaagtatc
ctacaagtaatctccaacagtctcagtgaaaacctagcactgttttcaacaaatctaagc
tctacaggtaaattgttaaatccctcaacaagaagtgtattcctttctcaagttgcagca
catcataagacaacaggtgtagtctttatcacactagggcttatcagattcctcaccatg
tataactctcaatttagtatacttacatcttatatagattaccacattgtgctatctatt
aatttagcattaattgcatctttatcaataatagtagctgatcatctcacaagaacccca
atctatcctcacaactcaacctcatacctacaatcctttgcctctccatacatcatgca
tggttatccggtttcctcatcattggttcaggagctcatgcatcaatattcaacttgcga
gcatcaccaacatctgagataagacatcgagaccccatatactcacacctcatctgggta
tgtatagcaataggattacactcattcagtctctactgtcataacgacactttagaagca
ttaggacgtccagaagatatctttcatgacaactctatccagttaaaagcaatatttgcc
aagcaatcctttctaagagcagagctccagccagatatcgagatgttagataaaaagatt
atacgtataacccaagaattgggaacagcagattttatagttcaccacattcatgcattt
acaatccatgttactctcttaattctttcaaagggtgtactttatgctagaaactctaga
tttgtatcagataaattggaacttggttttacttatccctgtgatggtccaggtagaggt
ggtacatgtcaaatatcaccttgggatcacttattctcagctgtattttggatgtataac
tgtttaaatgtagtaacattccactacttttggaagatgcaatcagatgtttggggcttt
gtatcgatccaaaagcacatatctcactacagccaaggtgattttagtgtcaactctatt
accatcaatggttggttgagaaacttattatggtccgaggcatcccaagtaatccaatcc
tatgctctttcatccatttgtccatatgggttcattttcctcatagctcatttcatctgg
gcatttagtttaatgttcctctttagtggtagagcatactggcaagaattaattgagtcc
attctatggtcacatcataaattaaagattatacctcatatccaaccaagggcattaagc
atttctcaaggaagagcagttggtttcattcattacactctaggaggtatcggctcaacc
tgggcatttattatttcaagactgctagtattaacttcttga
>Isy21_psaA
atgaacatcttcagatatatcaacacaacactgtggtccaaagcaggacattttaataaa
gctttatcaaaaggagctaagacaactacttggatatggaatctacatgattatgctcat
gattttgacattcaacaaagatccacaggtttaatagcaagaaaagtcttctcatccaat
ctagctcatctatctttggtattcttttggattagtggaatgcaccttcatggggcatat
ttgtctaattatgatatttggttaaaagatcccaaatccatcaccccatcatcacatcta
gcctactctttaattggtcaagatattcttaactcatatacctcagaatacttctcagga
atcactatcacatctggctttttccaactctaccgttctgagggtatcattacacagtcc

cagctgaaatatgcatgtgctacttccctaatagctacacttatctgcctttctggatct
tatctccacatgcagcttatgtccaaattcactagcttttacaagaagttccagtcctta
tcgcaagatcatctcataattatatttggctccagctccacatccctctccgctcatcag
atacacaaaatgcttccagctaaccctttactagattcaggtatctccaacccaagtatc
ctacaagtaatctccaacagtctcagtgaaaacctagcactgttttcaacaaatctaagc
tctacaggtaaattgttaaatccctcaacaagaagtgtattcctttctcaagttgcagca
catcataagacaacaggtgtagtctttatcacactagggcttatcagattcctcaccatg
tataactctcaatttagtatacttacatcttatatagattaccacattgtgctatctatt
aatttagcattaattgcatctttatcaataatagtagctgatcatctcacaagaacccca
atctatcctcacaactcaacctcataccctacaatcctttgcctctccatacatcatgca
tggttatccggtttcctcatcattggttcaggagctcatgcatcaatattcaacttgcga
gcatcaccaacatctgagataagacatcgagaccccatatactcacacctcatctgggta
tgtatagcaataggattacactcattcagtctctactgtcataacgacactttagaagca
ttaggacgtccagaagatatctttcatgacaactctatccagttaaaagcaatatttgcc
aagcaatcctttctaagagcagagctccagccagatatcgagatgttagataaaaagatt
atacgtataacccaagaattgggaacagcagattttatagttcaccacattcatgcattt
acaatccatgttactctcttaattctttcaaagggtgtactttatgctagaaactctaga
tttgtatcagataaattggaacttggttttacttatccctgtgatgggccaggtagaggt
ggtacatgtcaaatatcaccttgggatcacttattctcagctgtattttggatgtataac
tgtttaaatgtagtaacattccactacttttggaagatgcaatcagatgtttggggcttt
gtatcgatccaaaagcacatatctcactacagccaaggtgattttagtgtcaactctatt
accatcaatggttggttgagaaacttattatggtccgaggcatcccaagtaatccaatcc
tatgctctttcatccatttgtccatatgggttcattttcctcatagctcatttcatctgg
gcatttagtttaatgttcctctttagtggtagagcatactggcaagaattaattgagtcc
attctatggtcacatcataaattaaagattatacctcatatccaaccaagggcattaagc
atttctcaaggaagagcagttggtttcattcattacactctaggaggtatcggctcaacc
tgggcatttattatttcaagactgctagtattaacttcttga
>Isy22_psaA
atgcacatcttcagatatatcaacacaacactgtggtccaaagcaggacattttaataaa
gctttatcaaaaggagctaagacaactacttggatatggaatctacatgattatgctcat
gattttgacattcaacaaagatccacaggtttaatagcaagaaaagtcttctcatccaat
ctagctcatctatctttggtattcttttggattagtggaatgcaccttcatggggcatat
ttgtctaattatgatatttggttaaaagatcccaaatccatcaccccatcatcacatcta
gcctactctttaattggtcaagatattcttaactcatatacctcagaatacttctcagga
atcactatcacatctggctttttccaactctaccgttctgagggtatcattacacagtcc
cagctgaaatatgcatgtgctacttccctaatagctacacttatctgcctttctggatct
tatctccacatgcagcttatgtccaaattcactagcttttacaagaagttccagtcctta
tcgcaagatcatctcataattatatttggctccagctccacatccctctccgctcatcag
atacacaaaatgcttccagctaaccctttactagattcaggtatctccaacccaagtatc
ctacaagtaatctccaacagtctcagtgaaaacctagcactgttttcaacaaatctaagc
tctacaggtaaattgttaaatccctcaacaagaagtgtattcctttctcaagttgcagca
catcataagacaacaggtgtagtctttatcacactagggcttatcagattcctcaccatg
tataactctcaatttagtatacttacatcttatatagattaccacattgtgctatctatt
aatttagcattaattgcatctttatcaataatagtagctgatcatctcacaagaacccca
atctatcctcacaactcaacctcataccctacaatcctttgcctctccatacatcatgca
tggttatccggtttcctcatcattggttcaggagctcatgcatcaatattcaacttgcga
gcatcaccaacatctgagataagacatcgagaccccatatactcacacctcatctgggta
tgtatagcaataggattacactcattcagtctctactgtcataacgacactttagaagca
ttaggacgtccagaagatatctttcatgacaactctatccagttaaaagcaatatttgcc
aagcaatcctttctaagagcagagctccagccagatatcgagatgttagataaaaagatt
atacgtataacccaagaattgggaacagcagattttatagttcaccacattcatgcattt
acaatccatgttactctcttaattctttcaaagggtgtactttatgctagaaactctaga
tttgtatcagataaattggaacttggttttacttatccctgtgatggtccaggtagaggt
ggtacatgtcaaatatcaccttgggatcacttattctcagctgtattttggatgtataac
tgtttaaatgtagtaacattccactacttttggaagatgcaatcagatgtttggggcttt
gtatcgatccaaaagcacatatctcactacagccaaggtgattttagtgtcaactctatt
accatcaatggttggttgagaaacttattatggtccgaggcatcccaagtaatccaatcc

tatgctctttcatccatttgtccatatgggttcattttcctcatagctcatttcatctgg
gcatttagtttaatgttcctctttagtggtagagcatactggcaagaattaattgagtcc
attctatggtcacatcataaattaaagattatacctcatatccaaccaagggcattaagc
atttctcaaggaagagcagttggtttcattcattacactctaggaggtatcggctcaacc
tgggcatttattatttcaagactgctagtattaacttcttga
>Isy23_psaA
atgcacatcttcagatatatcaacacaacactgtggtccaaagcaggacattttaataaa
gctttatcaaaaggagctaagacaactacttggatatggaatctacatgattatgctcat
gattttgacattcaacaaagatccacaggtttaatagcaagaaaagtcttctcatccaat
ctagctcatctatctttggtattcttttggattagtggaatgcaccttcatggggcatat
ttgtctaattatgatatttggttaaaagatcccaaatccatcaccccatcatcacatcta
gcctactctttaattggtcaagatattcttaactcatatacctcagaatacttctcagga
atcactatcacatctggctttttccaactctaccgttctgagggtatcattacacagtcc
cagctgaaatatgcatgtgctacttccctaatagctacacttatctgcctttctggatct
tatctccacatgcagcttatgtccaaattcactagcttttacaagaagttccagtcctta
tcgcaagatcatctcataattatatttggctccagctccacatccctctccgctcatcag
atacacaaaatgcttccagctaaccctttactagattcaggtatctccaacccaagtatc
ctacaagtaatctccaacagtctcagtgaaaacctagcactgttttcaacaaatctaagc
tctacaggtaaattgttaaatccctcaacaagaagtgtattcctttctcaagttgcagca
catcataagacaacaggtgtagtctttatcacactagggcttatcagattcctcaccatg
tataactctcaatttagtatacttacatcttatatagattaccacattgtgctatctatt
aatttagcattaattgcatctttatcaataatagtagctgatcatctcacaagaacccca
atctatcctcacaactcaacctcataccctacaatcctttgcctctccatacatcatgca
tggttatccggtttcctcatcattggttcaggagctcatgcatcaatattcaacttgcga
gcatcaccaacatctgagataagacatcgagaccccatatactcacacctcatctgggta
tgtatagcaataggattacactcattcagtctctactgtcataacgacactttagaagca
ttaggacgtccagaagatatctttcatgacaactctatccagttaaaagcaatatttgcc
aagcaatcctttctaagagcagagctccagccagatatcgagatgttagataaaaagatt
atacgtataacccaagaattgggaacagcagattttatagttcaccacattcatgcattt
acaatccatgttactctcttaattctttcaaagggtgtactttatgctagaaactctaga
tttgtatcagataaattggaacttggttttacttatccctgtgatgggccaggtagaggt
ggtacatgtcaaatatcaccttgggatcacttattctcagctgtattttggatgtataac
tgtttaaatgtagtaacattccactacttttggaagatgcaatcagatgtttggggcttt
gtatcgatccaaaagcacatatctcactacagccaaggtgattttagtgtcaactctatt
accatcaatggttggttgagaaacttattatggtccgaggcatcccaagtaatccaatcc
tatgctctttcatccatttgtccatatgggttcattttcctcatagctcatttcatctgg
gcatttagtttaatgttcctctttagtggtagagcatactggcaagaattaattgagtcc
attctatggtcacatcataaattaaagattatacctcatatccaaccaagggcattaagc
atttctcaaggaagagcagttggtttcattcattacactctaggaggtatcggctcaacc
tgggcatttattatttcaagactgctagtattaacttcttga
>Isy24_psaA
atgcacatcttcagatatatcaacacaacactgtggtccaaagcaggacattttaataaa
gctttatcaaaaggagctaagacaactacttggatatggaatctacatgattatgctcat
gattttgacattcaacaaagatccacaggtttaatagcaagaaaagtcttctcatccaat
ctagctcatctatctttggtattcttttggattagtggaatgcaccttcatggggcatat
ttgtctaattatgatatttggttaaaagatcccaaatccatcaccccatcatcacatcta
gcctactctttaattggtcaagatattcttaactcatatacctcagaatacttctcagga
atcactatcacatctggctttttccaactctaccgttctgagggtatcattacacagtcc
cagctgaaatatgcatgtgctacttccctaatagctacacttatctgcctttctggatct
tatctccacatgcagcttatgtccaaattcactagcttttacaagaagttccagtcctta
tcgcaagatcatctcataattatatttggctccagctccacatccctctccgctcatcag
atacacaaaatgcttccagctaaccctttactagattcaggtatctccaacccaagtatc
ctacaagtaatctccaacagtctcagtgaaaacctagcactgttttcaacaaatctaagc
tctacaggtaaattgttaaatccctcaacaagaagtgtattcctttctcaagttgcagca
catcataagacaacaggtgtagtctttatcacactagggcttatcagattcctcaccatg
tataactctcaatttagtatacttacatcttatatagattaccacattgtgctatctatt
aatttagcattaattgcatctttatcaataatagtagctgatcatctcacaagaacccca

atctatcctcacaactcaacctcataccctacaatcctttgcctctccatacatcatgca
tggttatccggtttcctcatcattggttcaggagctcatgcatcaatattcaacttgcga
gcatcaccaacatctgagataagacatcgagaccccatatactcacacctcatctgggta
tgtatagcaataggattacactcattcagtctctactgtcataacgacactttagaagca
ttaggacgtccagaagatatctttcatgacaactctatccagttaaaagcaatatttgcc
aagcaatcctttctaagagcagagctccagccagatatcgagatgttagataaaaagatt
atacgtataacccaagaattgggaacagcagattttatagttcaccacattcatgcattt
acaatccatgttactctcttaattctttcaaagggtgtactttatgctagaaactctaga
tttgtatcagataaattggaacttggttttacttatccctgtgatggtccaggtagaggt
ggtacatgtcaaatatcaccttgggatcacttattctcagctgtattttggatgtataac
tgtttaaatgtagtaacattccactacttttggaagatgcaatcagatgtttggggcttt
gtatcgatccaaaagcacatatctcactacagccaaggtgattttagtgtcaactctatt
accatcaatggttggttgagaaacttattatggtccgaggcatcccaagtaatccaatcc
tatgctctttcatccatttgtccatatgggttcattttcctcatagctcatttcatctgg
gcatttagtttaatgttcctctttagtggtagagcatactggcaagaattaattgagtcc
attctatggtcacatcataaattaaagattatacctcatatccaaccaagggcattaagc
atttctcaaggaagagcagttggtttcattcattacactctaggaggtatcggctcaacc
tgggcatttattatttcaagactgctagtattaacttcttga
>Isy25_psaA
atgcacatcttcagatatatcaacacaacactgtggtccaaagcaggacattttaataaa
gctttatcaaaaggagctaagacaactacttggatatggaatctacatgattatgctcat
gattttgacattcaacaaagatccacaggtttaatagcaagaaaagtcttctcatccaat
ctagctcatctatctttggtattcttttggattagtggaatgcaccttcatggggcatat
ttgtctaattatgatatttggttaaaagatcccaaatccatcaccccatcatcacatcta
gcctactctttaattggtcaagatattcttaactcatatacctcagaatacttctcagga
atcactatcacatctggctttttccaactctaccgttctgagggtatcattacacagtcc
cagctgaaatatgcatgtgctacttccctaatagctacacttatctgcctttctggatct
tatctccacatgcagcttatgtccaaattcactagcttttacaagaagttccagtcctta
tcgcaagatcatctcataattatatttggctccagctccacatccctctccgctcatcag
atacacaaaatgcttccagctaacccttttactagattcaggtatctccaacccaagtatc
ctacaagtaatctccaacagtctcagtgaaaacctagcactgttttcaacaaatctaagc
tctacaggtaaattgttaaatccctcaacaagaagtgtattcctttctcaagttgcagca
catcataagacaacaggtgtagtctttatcacactagggcttatcagattcctcaccatg
tataactctcaatttagtatacttacatcttatatagattaccacattgtgctatctatt
aatttagcattaattgcatctttatcaataatagtagctgatcatctcacaagaacccca
atctatcctcacaactcaacctcataccctacaatcctttgcctctccatacatcatgca
tggttatccggtttcctcatcattggttcaggagctcatgcatcaatattcaacttgcga
gcatcaccaacatctgagataagacatcgagaccccatatactcacacctcatctgggta
tgtatagcaataggattacactcattcagtctctactgtcataacgacactttagaagca
ttaggacgtccagaagatatctttcatgacaactctatccagttaaaagcaatatttgcc
aagcaatcctttctaagagcagagctccagccagatatcgagatgttagataaaaagatt
atacgtataacccaagaattgggaacagcagattttatagttcaccacattcatgcattt
acaatccatgttactctcttaattctttcaaagggtgtactttatgctagaaactctaga
tttgtatcagataaattggaacttggttttacttatccctgtgatgggccaggtagaggt
ggtacatgtcaaatatcaccttgggatcacttattctcagctgtattttggatgtataac
tgtttaaatgtagtaacattccactacttttggaagatgcaatcagatgtttggggcttt
gtatcgatccaaaagcacatatctcactacagccaaggtgattttagtgtcaactctatt
accatcaatggttggttgagaaacttattatggtccgaggcatcccaagtaatccaatcc
tatgctctttcatccatttgtccatatgggttcattttcctcatagctcatttcatctgg
gcatttagtttaatgttcctctttagtggtagagcatactggcaagaattaattgagtcc
attctatggtcacatcataaattaaagattatacctcatatccaaccaagggcattaagc
atttctcaaggaagagcagttggtttcattcattacactctaggaggtatcggctcaacc
tgggcatttattatttcaagactgctagtattaacttcttga
>Isy26_psaA
atgcacatcttcagatatatcaacacaacactgtggtccaaagcaggacattttaataaa
gctttatcaaaaggagctaagacaactacttggatatggaatctacatgattatgctcat
gattttgacattcaacaaagatccacaggtttaatagcaagaaaagtcttctcatccaat

ctagctcatctatctttggtattcttttggattagtggaatgcaccttcatggggcatat
ttgtctaattatgatatttggttaaaagatcccaaatccatcaccccatcatcacatcta
gcctactctttaattggtcaagatattcttaactcatatacctcagaatacttctcagga
atcactatcacatctggctttttccaactctaccgttctgagggtatcattacacagtcc
cagctgaaatatgcatgtgctacttccctaatagctacacttatctgccttctggatct
tatctccacatgcagcttatgtccaaattcactagcttttacaagaagttccagtcctta
tcgcaagatcatctcataattatatttggctccagctccacatccctctccgctcatcag
atacacaaaatgcttccagctaaccctttactagattcaggtatctccaacccaagtatc
ctacaagtaatctccaacagtctcagtgaaaacctagcactgttttctacaaatctaagc
tctacaggtaaattgttaaatccctcaacaagaagtgtattcctttctcaagttgcagca
catcataagacaacaggtgtagtctttatcacactagggcttatcagattcctcaccatg
tataactctcaatttagtatacttacatcttatatagattaccacattgtgctatctatt
aatttagcattaattgcatctttatcaataatagtagctgatcatctcacaagaacccca
atctatcctcacaactcaacctcataccctacaatcctttgcctctccatacatcatgca
tggttatccggtttcctcatcattggttcaggagctcatgcatcaatattcaacttgcga
gcatcaccaacatctgagataagacatcgagaccccatatactcacacctcatctgggta
tgtatagcaataggattacactcattcagtctctactgtcataacgacactttagaagca
ttaggacgtccagaagatatctttcatgacaactctatccagttaaaagcaatatttgcc
aagcaatcctttctaagagcagagctccagccagatatcgagatgttagataaaaagatt
atacgtataacccaagaattgggaacagcagattttatagttcaccacattcatgcattt
acaatccatgttactctcttaattctttcaaagggtgtactttatgctagaaactctaga
tttgtatcagataaattggaacttggttttacttatccctgtgatgggccaggtagaggt
ggtacatgtcaaatatcaccttgggatcacttattctcagctgtattttggatgtataac
tgtttaaatgtagtaacattccactacttttggaagatgcaatcagatgtttggggcttt
gtatcgatccaaaagcacatatctcactacagccaaggtgattttagtgtcaactctatt
accatcaatggttggttgagaaacttattatggtccgaggcatcccaagtaatccaatcc
tatgctctttcatccatttgtccatatgggttcattttcctcatagctcatttcatctgg
gcatttagtttaatgttcctctttagtggtagagcatactggcaagaattaattgagtcc
attctatggtcacatcataaattaaagattatacctcatatccaaccaagggcattaagc
atttctcaaggaagagcagttggtttcattcattacactctaggaggtatcggctcaacc
tgggcatttattatttcaagactgctagtattaacttcttga
>Isy27_psaA
atgcacatctttagatatatcaacacaacactgtggtccaaagcaggacattttaataaa
gctttatcaaaaggagctaagacaactacttggatatggaatctacatgattatgctcat
gattttgacattcaacaaagatccacaggtttaatagcaagaaaagtcttctcatccaat
ctagctcatctatctttggtattcttttggattagtggaatgcaccttcatggggcatat
ttgtctaattatgatatttggttaaaagatcccaaatccatcaccccatcatcacatcta
gcctactctttaattggtcaagatattcttaactcatatacctcagaatacttctcagga
atcactatcacatctggctttttccaactctaccgttctgagggtatcattacacagtcc
cagctgaaatatgcatgtgctacttccctaatagctacacttatctgccttctggatct
tatctccacatgcagcttatgtccaaattcactagcttttacaagaagttccagtcctta
tcgcaagatcatctcataattatatttggctccagctccacatccctctccgctcatcag
atacacaaaatgcttccagctaaccctttactagattcaggtatctccaacccaagtatc
ctacaagtaatctccaacagtctcagtgaaaacctagcactgttttcaacaaatctaagc
tctacaggtaaattgttaaatccctcaacaagaagtgtattcctttctcaagttgcagca
catcataagacaacaggtgtagtctttatcacactagggcttatcagattcctcaccatg
tataactctcaatttagtatacttacatcttatatagattaccacattgtgctatctatt
aatttagcattaattgcatctttatcaataatagtagctgatcatctcacaagaacccca
atctatcctcacaactcaacctcataccctacaatcctttgcctctccatacatcatgca
tggttatccggtttcctcatcattggttcaggagctcatgcatcaatattcaacttgcga
gcatcaccaacatctgagataagacatcgagaccccatatactcacacctcatctgggta
tgtatagcaataggattacactcattcagtctctactgtcataacgacactttagaagca
ttaggacgtccagaagatatctttcatgacaactctatccagttaaaagcaatatttgcc
aagcaatcctttctaagagcagagctccagccagatatcgagatgttagataaaaagatt
atacgtataacccaagaattgggaacagcagattttatagttcaccacattcatgcattt
acaatccatgttactctcttaattctttcaaagggtgtactttatgctagaaactctaga
tttgtatcagataaattggaacttggttttacttatccctgtgatgggccaggtagaggt

ggtacatgtcaaatatcaccttgggatcacttattctcagctgtattttggatgtataac
tgtttaaatgtagtaacattccactacttttggaagatgcaatcagatgtttggggcttt
gtatcgatccaaaagcacatatctcactacagccaaggtgattttagtgtcaactctatt
accatcaatggttggttgagaaacttattatggtccgaggcatcccaagtaatccaatcc
tatgctctttcatccatttgtccatatgggttcattttcctcatagctcatttcatctgg
gcatttagtttaatgttcctctttagtggtagagcatactggcaagaattaattgagtcc
attctatggtcacatcataaattaaagattatacctcatatccaaccaagggcattaagc
atttctcaaggaagagcagttggtttcattcattacactctaggaggtatcggctcaacc
tgggcatttattatttcaagactgctagtattaacttcttga
>Isy4_psaA
atgcacatcttcagatatatcaacacaacactgtggtccaaagcaggacattttaataaa
gctttatcaaaaggagctaagacaactacttggatatggaatctacatgattatgctcat
gattttgacattcaacaaagatccacaggtttaatagcaagaaaagtcttctcatccaat
ctagctcatctatctttggtattcttttggattagtggaatgcaccttcatggggcatat
ttgtctaattatgatatttggttaaaagatcccaaatccatcaccccatcatcacatcta
gcctactctttaattggtcaagatattcttaactcatatacctcagaatacttctcagga
atcactatcacatctggctttttccaactctaccgttctgagggtatcattacacagtcc
cagctgaaatatgcatgtgctacttccctaatagctacacttatctgcctttctggatct
tatctccacatgcagcttatgtccaaattcactagcttttacaagaagttccagtcctta
tcgcaagatcatctcataattatatttggctccagctccacatccctctccgctcatcag
atacacaaaatgcttccagctaaccctttactagattcaggtatctccaacccaagtatc
ctacaagtaatctccaacagtctcagtgaaaacctagcactgttttcaacaaatctaagc
tctacaggtaaattgttaaatccctcaacaagaagtgtattcctttctcaagttgcagca
catcataagacaacaggtgtagtctttatcacactagggcttatcagattcctcaccatg
tataactctcaatttagtatacttacatcttatatagattaccacattgtgctatctatt
aatttagcattaattgcatctttatcaataatagtagctgatcatctcacaagaacccca
atctatcctcacaactcaacctcataccctacaatcctttgcctctccatacatcatgca
tggttatccggtttcctcatcattggttcaggagctcatgcatcaatattcaacttgcga
gcatcaccaacatctgagataagacatcgagaccccatatactcacacctcatctgggta
tgtatagcaataggattacactcattcagtctctactgtcataacgacactttagaagca
ttaggacgtccagaagatatctttcatgacaactctatccagttaaaagcaatatttgcc
aagcaatcctttctaagagcagagctccagccagatatcgagatgttagataaaaagatt
atacgtataacccaagaattgggaacagcagattttatagttcaccacattcatgcattt
acaatccatgttactctcttaattctttcaaagggtgtactttatgctagaaactctaga
tttgtatcagataaattggaacttggttttacttatccctgtgatggtccaggtagaggt
ggtacatgtcaaatatcaccttgggatcacttattctcagctgtattttggatgtataac
tgtttaaatgtagtaacattccactacttttggaagatgcaatcagatgtttggggcttt
gtatcgatccaaaagcacatatctcactacagccaaggtgattttagtgtcaactctatt
accatcaatggttggttgagaaacttattatggtccgaggcatcccaagtaatccaatcc
tatgctctttcatccatttgtccatatgggttcattttcctcatagctcatttcatctgg
gcatttagtttaatgttcctctttagtggtagagcatactggcaagaattaattgagtcc
attctatggtcacatcataaattaaagattatacctcatatccaaccaagggcattaagc
atttctcaaggaagagcagttggtttcattcattacactctaggaggtatcggctcaacc
tgggcatttattatttcaagactgctagtattaacttcttga
>Isy7_psaA
atgcacatcttcagatatatcaatactacactgtggtccaaagcaggacactttaataaa
gctttatcaaaaggagctaagacaactacttggatatggaatctacatgattatgcacat
gattttgatattcaacaaagctccgcaggtttaatagcaagaaaagtcttctcatccaat
ctagctcatctatctttggtattcttttggattagtggaatgcaccttcatggggcatac
ttgtctaattatgatatttggttaaaagatcctaaatctataagcccttcatcacagcta
gcttactctttagttggccaagatattcttaactcatatacctcagaatacttctcagga
atcactattacttcaggactttttcaactctaccgttctgagggtataattacacagtct
cagctgaaatatgcatgtgctacttccctaatagctacacttatctgcctttctggatct
tatctccacatgcagcttatgtccaaattcactagcttttacaagaagttccagtcctta
tcgcaagatcatctcataattatatttggctccagctccacatccctctccgctcatcag
atacacaaaatgcttccagctaaccctttactagattcaggtatctccaacccaagtatc
ctacaagtaatctccaacagtctcagtgaaaacctagcactgttttcaacaaatctaagc

tctacaggtaaattgttaaatccctcaacaagaagtgtattcctttctcaagttgcagca
catcataagacaacaggtgtagtctttatcacactagggcttatcagattcctcaccatg
tataactctcaatttagtatacttacatcttatatagattaccaccttgtcctatctact
aacttagcattaattggatctttatcaataataacagctgatcatctcacaagaacccca
atctatcctcacaactcaacctcataccctacaatcctttgcctctccatacatcatgca
tggttatccggtttcctcatcattggttcaggagctcatgcatcaatattcaacttgcga
gcatcaccaacatctgagataagacatcgagaccccatatactcacacctcatctggata
tgtatagctttaggattacactcatttagtctctactgccataacgacactttagaagct
ttaggacgtccagaagatatctttcacgataactctatccagttaaaagctatatttgct
aagcaatcctttctaagagcagagctccagccagatatcgagatgttagataaaaagatt
atacgtataacccaagaattgggaacagcagattttatagttcaccacattcatgcattt
acaatccatgttactctcttaattctttcaaagggtgtactttatgctagaaactctaga
tttgtatcagataaattggaacttggttttacttatccttgtgatggtccaggtagaggt
ggtacatgtcaaatatcaccttgggatcacttattctcagctgtattttggatgtataat
tgtttaaatgtagtaacattccactacttttggaagatgcaatcagatgtttggggcttt
gtatcgatccaaaagcacatatctcactacagccaaggtgattttagtgtcaactctatt
accatcaatggttggttgagaaacttattatggtccgaggcatcccaagtaatccaatcc
tatgctctttcatccatttgtccatatgggttcattttcctcatagctcatttcatctgg
gcatttagtttaatgttcctctttagtggtagagcatactggcaagaattaattgagtcc
attctatggtcacatcataaattaaagattatacctcatatccaaccaagggcattaagc
atttctcaaggaagagcagttggtttcattcattacactctaggaggtatcggctcaacc
tgggcatttattatttcaagactgctagtattaacttcttga
>Isy8_psaA
atgcacatctttagatatatcaacacaacactgtggtccaaagcaggacattttaataaa
gctttatcaaaaggagctaagacaactacttggatatggaatctacatgattatgctcat
gattttgacattcaacaaagatccacaggtttaatagcaagaaaagtcttctcatccaat
ctagctcatctatctttggtattcttttggattagtggaatgcaccttcatggggcatat
ttgtctaattatgatatttggttaaaagatcccaaatccatcaccccatcatcacatcta
gcctactctttaattggtcaagatattcttaactcatatacctcagaatacttctcagga
atcactatcacatctggctttttccaactctaccgttctgagggtatcattacacagtcc
cagctgaaatatgcatgtgctacttccctaatagctacacttatctgcctttctggatct
tatctccacatgcagcttatgtccaaattcactagcttttacaagaagttccagtcctta
tcgcaagatcatctcataattatatttggctccagctccacatccctctccgctcatcag
atacacaaaatgcttccagctaacccttactagattcaggtatctccaacccaagtatc
ctacaagtaatctccaacagtctcagtgaaaacctagcactgttttcaacaaatctaagc
tctacaggtaaattgttaaatccctcaacaagaagtgtattcctttctcaagttgcagca
catcataagacaacaggtgtagtctttatcacactagggcttatcagattcctcaccatg
tataactctcaatttagtatacttacatcttatatagattaccacattgtgctatctatt
aatttagcattaattgcatctttatcaataatagtagctgatcatctcacaagaacccca
atctatcctcacaactcaacctcataccctacaatcctttgcctctccatacatcatgca
tggttatccggtttcctcatcattggttcaggagctcatgcatcaatattcaacttgcga
gcatcaccaacatctgagataagacatcgagaccccatatactcacacctcatctgggta
tgtatagcaataggattacactcattcagtctctactgtcataacgacactttagaagca
ttaggacgtccagaagatatctttcatgacaactctatccagttaaaagcaatatttgcc
aagcaatcctttctaagagcagagctccagccagatatcgagatgttagataaaaagatt
atacgtataacccaagaattgggaacagcagattttatagttcaccacattcatgcattt
acaatccatgttactctcttaattctttcaaagggtgtactttatgctagaaactctaga
tttgtatcagataaattggaacttggttttacttatccctgtgatgggccaggtagaggt
ggtacatgtcaaatatcaccttgggatcacttattctcagctgtattttggatgtataac
tgtttaaatgtagtaacattccactacttttggaagatgcaatcagatgtttggggcttt
gtatcgatccaaaagcacatatctcactacagccaaggtgattttagtgtcaactctatt
accatcaatggttggttgagaaacttattatggtccgaggcatcccaagtaatccaatcc
tatgctctttcatccatttgtccatatgggttcattttcctcatagctcatttcatctgg
gcatttagtttaatgttcctctttagtggtagagcatactggcaagaattaattgagtcc
attctatggtcacatcataaattaaagattatacctcatatccaaccaagggcattaagc
atttctcaaggaagagcagttggtttcattcattacactctaggaggtatcggctcaacc
tgggcatttattatttcaagactgctagtattaacttcttga

>KrA1_psaA
atgcacatcttcagatatatcaacacaacactgtggtccaaagcaggacattttaataaa
gctttatcaaaaggagctaagacaactacttggatatggaatctacatgattatgctcat
gattttgacattcaacaaagatccacaggtttaatagcaagaaaagtcttctcatccaat
ctagctcatctatctttggtattcttttggattagtggaatgcaccttcatggggcatat
ttgtctaattatgatatttggttaaaagatcccaaatccatcaccccatcatcacatcta
gcctactctttaattggtcaagatattcttaactcatatacctcagaatacttctcagga
atcactatcacatctggctttttccaactctaccgttctgagggtatcattacacagtcc
cagctgaaatatgcatgtgctacttccctaatagctacacttatctgcctttctggatct
tatctccacatgcagcttatgtccaaattcactagcttttacaagaagttccagtcctta
tcgcaagatcatctcataattatatttggctccagctccacatccctctccgctcatcag
atacacaaaatgcttccagctaaccctttactagattcaggtatctccaacccaagtatc
ctacaagtaatctccaacagtctcagtgaaaacctagcactgttttcaacaaatctaagc
tctacaggtaaattgttaaatccctcaacaagaagtgtattcctttctcaagttgcagca
catcataagacaacaggtgtagtctttatcacactagggcttatcagattcctcaccatg
tataactctcaatttagtatacttacatcttatatagattaccacattgtgctatctatt
aatttagcattaattgcatctttatcaataatagtagctgatcatctcacaagaacccca
atctatcctcacaactcaacctcataccctacaatcctttgcctctccatacatcatgca
tggttatccggtttcctcatcattggttcaggagctcatgcatcaatattcaacttgcga
gcatcaccaacatctgagataagacatcgagaccccatatactcacacctcatctgggta
tgtatagcaataggattacactcattcagtctctactgtcataacgacactttagaagca
ttaggacgtccagaagatatctttcatgacaactctatccagttaaaagcaatatttgcc
aagcaatcctttctaagagcagagctccagccagatatcgagatgttagataaaaagatt
atacgtataacccaagaattgggaacagcagattttatagttcaccacattcatgcattt
acaatccatgttactctcttaattctttcaaagggtgtactttatgctagaaactctaga
tttgtatcagataaattggaacttggttttacttatccctgtgatggtccaggtagaggt
ggtacatgtcaaatatcaccttgggatcacttattctcagctgtattttggatgtataac
tgtttaaatgtagtaacattccactacttttggaagatgcaatcagatgtttggggcttt
gtatcgatccaaaagcacatatctcactacagccaaggtgattttagtgtcaactctatt
accatcaatggttggttgagaaacttattatggtccgaggcatcccaagtaatccaatcc
tatgctctttcatccatttgtccatatgggttcattttcctcatagctcatttcatctgg
gcatttagtttaatgttcctctttagtggtagagcatactggcaagaattaattgagtcc
attctatggtcacatcataaattaaagattatacctcatatccaaccaagggcattaagc
atttctcaaggaagagcagttggtttcattcattacactctaggaggtatcggctcaacc
tgggcatttattatttcaagactgctagtattaacttcttga
>KrA10_psaA
atgcacatcttcagatatatcaacacaacactgtggtccaaagcaggacattttaataaa
gctttatcaaaaggagctaagacaactacttggatatggaatctacatgattatgctcat
gattttgacattcaacaaagatccacaggtttaatagcaagaaaagtcttctcatccaat
ctagctcatctatctttggtattcttttggattagtggaatgcaccttcatggggcatat
ttgtctaattatgatatttggttaaaagatcccaaatccatcaccccatcatcacatcta
gcctactctttaattggtcaagatattcttaactcatatacctcagaatacttctcagga
atcactatcacatctggctttttccaactctaccgttctgagggtatcattacacagtcc
cagctgaaatatgcatgtgctacttccctaatagctacacttatctgcctttctggatct
tatctccacatgcagcttatgtccaaattcactagcttttacaagaagttccagtcctta
tcgcaagatcatctcataattatatttggctccagctccacatccctctccgctcatcag
atacacaaaatgcttccagctaaccctttactagattcaggtatctccaacccaagtatc
ctacaagtaatctccaacagtctcagtgaaaacctagcactgttttcaacaaatctaagc
tctacaggtaaattgttaaatccctcaacaagaagtgtattcctttctcaagttgcagca
catcataagacaacaggtgtagtctttatcacactagggcttatcagattcctcaccatg
tataactctcaatttagtatacttacatcttatatagattaccacattgtgctatctatt
aatttagcattaattgcatctttatcaataatagtagctgatcatctcacaagaacccca
atctatcctcacaactcaacctcataccctacaatcctttgcctctccatacatcatgca
tggttatccggtttcctcatcattggttcaggagctcatgcatcaatattcaacttgcga
gcatcaccaacatctgagataagacatcgagaccccatatactcacacctcatctgggta
tgtatagcaataggattacactcattcagtctctactgtcataacgacactttagaagca
ttaggacgtccagaagatatctttcatgacaactctatccagttaaaagcaatatttgcc

aagcaatcctttctaagagcagagctccagccagatatcgagatgttagataaaaagatt
atacgtataacccaagaattgggaaaagcagattttatagttcaccacattcatgcattt
acaatccatgttactctcttaattctttcaaagggtgtactttatgctagaaactctaga
tttgtatcagataaattggaacttggttttacttatccctgtgatggtccaggtagaggt
ggtacatgtcaaatatcaccttgggatcacttattctcagctgtattttggatgtataac
tgtttaaatgtagtaacattccactacttttggaagatgcaatcagatgtttggggcttt
gtatcgatccaaaagcacatatctcactacagccaaggtgattttagtgtcaactctatt
accatcaatggttggttgagaaacttattatggtccgaggcatcccaagtaatccaatcc
tatgctctttcatccatttgtccatatgggttcattttcctcatagctcatttcatctgg
gcatttagtttaatgttcctctttagtggtagagcatactggcaagaattaattgagtcc
attctatggtcacatcataaattaaagattatacctcatatccaaccaagggcattaagc
atttctcaaggaagagcagttggtttcattcattacactctaggaggtatcggctcaacc
tgggcatttattatttcaagactgctagtattaacttcttga
>KrA11_psaA
atgcacatcttcagatatatcaacacaacactgtggtccaaagcaggacattttaataaa
gctttatcaaaaggagctaagacaactacttggatatggaatctacatgattatgctcat
gattttgacattcaacaaagatccacaggtttaatagcaagaaaagtcttctcatccaat
ctagctcatctatctttggtattcttttggattagtggaatgcaccttcatggggcatat
ttgtctaattatgatatttggttaaaagatcccaaatccatcaccccatcatcacatcta
gcctactctttaattggtcaagatattcttaactcatatacctcagaatacttctcagga
atcactatcacatctggctttttccaactctaccgttctgagggtatcattacacagtcc
cagctcaaatatgcatgtgctacttccctaatagctacacttatctgcctttctggatct
tatctccacatgcagcttatgtccaaattcactagcttttacaagaagttccagtcctta
tcgcaagatcatctcataattatatttggctccagctccacatccctctccgctcatcag
atacacaaaatgcttccagctaaccctttactagattcaggtatctccaacccaagtatc
ctacaagtaatctccaacagtctcagtgaaaacctagcactgttttcaacaaatctaagc
tctacaggtaaattgttaaatccctcaacaagaagtgtattcctttctcaagttgcagca
catcataagacaacaggtgtagtctttatcacactaggcttatcagattcctcaccatg
tataactctcaatttagtatacttacatcttatatagattaccacattgtgctatctatt
aatttagcattaattgcatctttatcaataatagtagctgatcatctcacaagaacccca
atctatcctcacaactcaacctcataccctacaatcctttgcctctccatacatcatgca
tggttatccggtttcctcatcattggttcaggagctcatgcatcaatattcaacttgcga
gcatcaccaacatctgagataagacatcgagaccccatatactcacacctcatctgggta
tgtatagcaataggattacactcattcagtctctactgtcataacgacactttagaagca
ttaggacgtccagaagatatctttcatgacaactctatccagttaaaagcaatatttgcc
aagcaatcctttctaagagcagagctccagccagatatcgagatgttagataaaaagatt
atacgtataacccaagaattgggaacagcagattttatagttcaccacattcatgcattt
acaatccatgttactctcttaattctttcaaagggtgtactttatgctagaaactctaga
tttgtatcagataaattggaacttggttttacttatccctgtgatggtccaggtagaggt
ggtacatgtcaaatatcaccttgggatcacttattctcagctgtattttggatgtataac
tgtttaaatgtagtaacattccactacttttggaagatgcaatcagatgtttggggcttt
gtatcgatccaaaagcacatatctcactacagccaaggtgattttagtgtcaactctatt
accatcaatggttggttgagaaacttattatggtccgaggcatcccaagtaatccaatcc
tatgctctttcatccatttgtccatatgggttcattttcctcatagctcatttcatctgg
gcatttagtttaatgttcctctttagtggtagagcatactggcaagaattaattgagtcc
attctatggtcacatcataaattaaagattatacctcatatccaaccaagggcattaagc
atttctcaaggaagagcagttggtttcattcattacactctaggaggtatcggctcaacc
tgggcatttattatttcaagactgctagtattaacttcttga
>KrA12_psaA
atgcacatcttcagatatatcaacacaacactgtggtccaaagcaggacattttaataaa
gctttatcaaaaggagctaagacaactacttggatatggaatctacatgattatgctcat
gattttgacattcaacaaagatccacaggtttaatagcaagaaaagtcttctcatccaat
ctagctcatctatctttggtattcttttggattagtggaatgcaccttcatggagcatat
ttgtctaattatgatatttggttaaaagatcccaaatccatcacgccatcatcacatcta
gcctactctttaattggtcaagatattcttaactcatatacctcagaatacttctcagga
atcactatcacatctggctttttccaactctaccgttctgagggtatcattacacagtcc
cagctgaaatatgcatgtgctacttccctaatagctacacttatctgcctttctggatct

tatctccacatgcagcttatgtccaaattcactagcttttacaagaagttccagtcctta
tcgcaagatcatctcataattatatttggctccaggtccacatccctctccgctcatcag
atacacaaaatgcttccagctaacccttttactagattcaggtatctccaacccaagtatc
ctacaagtaatctccaacagtctcagtgaaaacctagcactgttttcaacaaatctaagc
tctacaggtaaattgttaaatccctcaacaagaagtgtattcctttctcaagttgcagca
catcataagacaacaggtgtagtctttatcacactagggcttatcagattcctcaccatg
tataagtctcaatttagtatacttacatcttatatagattaccacattgtgctatctatt
aatttagcattaattgcatctttatcaataatagtagctgatcatctcacaagaacccca
atctatcctcacaactcaacctcataccctacaatcctttgcctctccatacatcatgca
tggttatccggtttcctcatcattggttcaggagctcatgcatcaatattcaacttgctt
ggatcaccaacatctgagataagacatcgagaccccatatactcacacctcatctgggta
tgtatagcaataggattacactcattcagtctctactgtcataacgacactttagaagca
ttaggacgtccagaagatatctttcatgacaactctatccagttaaaagcaatatttgcc
aagcaatcctttctaagagcagagctccagccagatatcgagatgttagataaaaagatt
atacgtataacccaagaattgggaacagcagattttatagttcaccacattcatgcattt
acaatccatgttactctcttaattctttcaaagggtgtactttatgctagaaactctaga
tttgtatcagataaattggaacttggttttacttatccctgtgatggtccaggtagaggt
ggtacatgtcaaatatcaccttgggatcacttattctcagctgtattttggatgtataac
tgtttaaatgtagtaacattccactacttttggaagatgcaatcagatgtttggggcttt
gtatcgatccaaaagcacatatctcactacagccaaggtgattttagtgtcaactctatt
accatcaatggttggttgagaaacttattatggtccgaggcatcccaagtaatccaatcc
tatgctctttcatccatttgtccatatgggttcattttcctcatagctcatttcatctgg
gcatttagtttaatgttcctctttagtggtagagcatactggcaagaattaattgagtcc
attctatggtcacatcataaattaaagattatacctcatatccaaccaagggcattaagc
atttctcaaggaagagcagttggtttcattcattacactctaggaggtatcggctcaacc
tgggcatttattatttcaagactgctagtattaacttcttga
>KrA13_psaA
atgcacatcttcagatatatcaacacaacactgtggtccaaagcaggacattttaataaa
gctttatcaaaaggagctaagacaactacttggatatggaatctacatgattatgctcat
gattttgacattcaacaaagatccacaggtttaatagcaagaaaagtcttctcatccaat
ctagctcatctatctttggtattcttttggattagtggaatgcaccttcatggggcatat
ttgtctaattatgatatttggttaaaagatcccaaatccatcaccccatcatcacatcta
gcctactctttaattggtcaagatattcttaactcatatacctcagaatacttctcagga
atcactatcacatctggctttttccaactctaccgttctgagggtatcattacacagtcc
cagctgaaatatgcatgtgctacttccctaatagctacacttatctgcctttctggatct
tatctccacatgcagcttatgtccaaattcactagcttttacaagaagttccagtcctta
tcgcaagatcatctcataattatatttggctccagctccacatccctctccgctcatcag
atacacaaaatgcttccagctaacccttttactagattcaggtatctccaacccaagtatc
ctacaagtaatctccaacagtctcagctatacactagcactgttttcaacaaatctaagc
tctacaggtaaattgttaaatccctcaacaagaagtgtattcctttctcaagttgcagca
catcataagacaacaggtgtagtctttatcacactagggcttatcagattcctcaccatg
tataactctcaatttagtatacttacatcttatatagattaccacattgtgctatctatt
aatttagcattaattgcatctttatcaataatagtagctgatcatctcacaagaacccca
atctatcctcacaactcaacctcataccctacaatcctttgcctctccatacatcatgca
tggttatccggtttcctcatcattggttcaggagctcatgcatcaatattcaacttgcga
gcatcaccaacatctgagataagacatcgagaccccatatactcacacctcatctgggta
tgtatagcaataggattacactcattcagtctctactgtcataacgacactttagaagca
ttaggacgtccagaagatatctttcatgacaactctatccagttaaaagcaatatttgcc
aagcaatcctttctaagagcagagctccagccagatatcgagatgttagataaaaagatt
atacgtataacccaagaattgggaacagcagattttatagttcaccacattcatgcattt
acaatccatgttactctcttaattctttcaaagggtgtactttatgctagaaactctaga
tttgtatcagataaattggaacttggttttacttatccctgtgatggtccaggtagaggt
ggtacatgtcaaatatcaccttgggatcacttattctcagctgtattttggatgtataac
tgtttaaatgtagtaacattccactacttttggaagatgcaatcagatgtttggggcttt
gtatcgatccaaaagcacatatctcactacagccaaggtgattttagtgtcaactctatt
accatcaatggttggttgagaaacttattatggtccgaggcatcccaagtaatccaatcc
tatgctctttcatccatttgtccatatgggttcattttcctcatagctcatttcatctgg

gcatttagtttaatgttcctctttagtggtagagcatactggcaagaattaattgagtcc
attctatggtcacatcataaattaaagattatacctcatatccaaccaagggcattaagc
atttctcaaggaagagcagttggtttcattcattacactctaggaggtatcggctcaacc
tgggcatttattatttcaagactgctagtattaacttcttga
>KrA14_psaA
atgcacatcttcagatatatcaacacaacactgtggtccaaagcaggacattttaataaa
gctttatcaaaaggagctaagacaactacttggatatggaatctacatgattatgctcat
gattttgacattcaacaaagatccacaggtttaatagcaagaaaagtcttctcatccaat
ctagctcatctatctttggtattcttttggattagtggaatgcaccttcatggggcatat
ttgtctaattatgatatttggttaaaagatcccaaatccatcaccccatcatcacatcta
gcctactctttaattggtcaagatattcttaactcatatacctcagaatacttctcagga
atcactatcacatctggctttttccaactctaccgttctgagggtatcattacacagtcc
cagctcaaatatgcatgtgctacttccctaatagctacacttatctgcctttctggatct
tatctccacatgcagcttatgtccaaattcactagcttttacaagaagttccagtcctta
tcgcaagatcatctcataattatatttggctccagctccacatccctctccgctcatcag
atacacaaaatgcttccagctaaccctttactagattcaggtatctccaacccaagtatc
ctacaagtaatctccaacagtctcagctatacactagcactgttttcaacaaatctaagc
tctacaggtaaattgttaaatccctcaacaagaagtgtattcctttctcaagttgcagca
catcataagacaacaggtgtagtctttatcacactagggcttatcagattcctcaccatg
tataagtctcaatttagtatacttacatcttatatagattaccacattgtgctatctatt
aatttagcattaattgcatctttatcaataatagtagctgatcatctcacaagaacccca
atctatcctcacaactcaacctcataccctacaatcctttgcctctccatacatcatgca
tggttatccggtttcctcatcattggttcaggagctcatgcatcaatattcaacttgcga
gcatcaccaacatctgagataagacatcgagaccccatatactcacacctcatctgggta
tgtatagcaataggattacactcattcagtctctactgtcataacgacactttagaagca
ttaggacgtccagaagatatctttcatgacaactctatccagttaaaagcaatatttgcc
aagcaatcctttctaagagcagagctccagccagatatcgagatgttagataaaaagatt
atacgtataacccaagaattgggaacagcagattttatagttcaccacattcatgcattt
acaatccatgttactctcttaattctttcaaagggtgtactttatgctagaaactctaga
tttgtatcagataaattggaacttggttttacttatccctgtgatggtccaggtagaggt
ggtacatgtcaaatatcaccttgggatcacttattctcagctgtattttggatgtataac
tgtttaaatgtagtaacattccactacttttggaagatgcaatcagatgtttggggcttt
gtatcgatccaaaagcacatatctcactacagccaaggtgattttagtgtcaactctatt
accatcaatggttggttgagaaacttattatggtccgaggcatcccaagtaatccaatcc
tatgctctttcatccatttgtccatatgggttcattttcctcatagctcatttcatctgg
gcatttagtttaatgttcctctttagtggtagagcatactggcaagaattaattgagtcc
attctatggtcacatcataaattaaagattatacctcatatccaaccaagggcattaagc
atttctcaaggaagagcagttggtttcattcattacactctaggaggtatcggctcaacc
tgggcatttattatttcaagactgctagtattaacttcttga
>KrA15_psaA
atgcacatcttcagatatatcaacacaacactgtgggccaaagcaggacattttaataaa
gctttatcaaaaggagctaagacaactacttggatatggaatctacatgattatgctcat
gattttgacattcaacaaagatccacaggtttaatagcaagaaaagtcttctcatccaat
ctagctcatctatctttggtattcttttggattagtggaatgcaccttcatggggcatat
ttgtctaattatgatatttggttaaaagatcccaaatccatcaccccatcatcacatcta
gcctactctttaattggtcaagatattcttaactcatatacctcagaatacttctcagga
atcactatcacatctggctttttccaactctaccgttctgagggtatcattacacagtcc
cagctgaaatatgcatgtgctacttccctaatagctacacttatctgcctttctggatct
tatctccacatgcagcttatgtccaaattcactagcttttacaagaagttccagtcctta
tcgcaagatcatctcataattatatttggctccagctccacatccctctccgctcatcag
atacacaaaatgcttccagctaaccctttactagattcaggtatctccaacccaagtatc
ctacaagtaatctccaacagtctcagtgaaaacctagcactgttttcaacaaatctaagc
tctacaggtaaattgttaaatccctcaacaagaagtgtattcctttctcaagttgcagca
catcataagacaacaggtgtagtctttatcacactagggcttatcagattcctcaccatg
tataactctcaatttagtatacttacatcttatatagattaccacattgtgctatctatt
aatttagcattaattgcatctttatcaataatagtagctgatcatctcacaagaacccca
atctatcctcacaactcaacctcataccctacaatcctttgcctctccatacatcatgca

tggttatccggtttcctcatcattggttcaggagctcatgcatcaatattcaacttgcga
gcatcaccaacatctgagataagacatcgagaccccatatactcacacctcatctgggta
tgtatagcaataggattacactcattcagtctctactgtcataacgacactttagaagca
ttaggacgtccagaagatatctttcatgacaactctatccagttaaaagcaatatttgcc
aagcaatcctttctaagagcagagctccagccagatatcgagatgttagataaaaagatt
atacgtataacccaagaattgggaacagcagatttatagttcaccacattcatgcattt
acaatccatgttactctcttaattctttcaaagggtgtactttatgctagaaactctaga
tttgtatcagataaattggaacttggttttacttatccctgtgatggtccaggtagaggt
ggtacatgtcaaatatcaccttgggatcacttattctcagctgtattttggatgtataac
tgtttaaatgtagtaacattccactacttttggaagatgcaatcagatgtttggggcttt
gtatcgatccaaaagcacatatctcactacagccaaggtgattttagtgtcaactctatt
accatcaatggttggttgagaaacttattatggtccgaggcatcccaagtaatccaatcc
tatgctctttcatccatttgtccatatgggttcattttcctcatagctcatttcatctgg
gcatttagtttaatgttcctctttagtggtagagcatactggcaagaattaattgagtcc
attctatggtcacatcataaattaaagattatacctcatatccaaccaagggcattaagc
atttctcaaggaagagcagttggtttcattcattacactctaggaggtatcggctcaacc
tgggcatttattatttcaagactgctagtattaacttcttga
>KrA2_psaA
atgcacatcttcagatatatcaacacaacactgtggtccaaagcaggacattttaataaa
gctttatcaaaaggagctaagacaactacttggatatggaatctacatgattatgctcat
gattttgacattcaacaaagatccacaggtttaatagcaagaaaagtcttctcatccaat
ctagctcatctatctttggtattcttttggattagtggaatgcaccttcatggggcatat
ttgtctaattatgatatttggttaaaagatcccaaatccatcaccccatcatcacatcta
gcctactctttaattggtcaagatattcttaactcatatacctcagaatacttctcagga
atcactatcacatctggctttttccaactctaccgttctgagggtatcattacacagtcc
cagctgaaatatgcatgtgctacttccctaatagctacacttatctgcctttctggatct
tatctccacatgcagcttatgtccaaattcactagcttttacaagaagttccagtcctta
tcgcaagatcatctcataattatatttggctccagctccacatccctctccgctcatcag
atacacaaaatgcttccagctaaccctttactagattcaggtatctccaacccaagtatc
ctacaagtaatctccaacagtctcagtgaaaacctagcactgttttcaacaaatctaagc
tctacaggtaaattgttaaatccctcaacaagaagtgtattcctttctcaagttgcagca
catcataagacaacaggtgtagtctttatcacactagggcttatcagattcctcaccatg
tataactctcaatttagtatacttacatcttatatagattaccacattgtgctatctatt
aatttagcattaattgcatctttatcaataatagtagctgatcatctcacaagaacccca
atctatcctcacaactcaacctcataccctacaatcctttgcctctccatacatcatgca
tggttatccggtttcctcatcattggttcaggagctcatgcatcaatattcaacttgcga
gcatcaccaacatctgagataagacatcgagaccccatatactcacacctcatctgggta
tgtatagcaataggattacactcattcagtctctactgtcataacgacactttagaagca
ttaggacgtccagaagatatctttcatgacaactctatccagttaaaagcaatatttgcc
aagcaatcctttctaagagcagagctccagccagatatcgagatgttagataaaaagatt
atacgtataacccaagaattgggaacagcagatttatagttcaccacattcatgcattt
acaatccatgttactctcttaattctttcaaagggtgtactttatgctagaaactctaga
tttgtatcagataaattggaacttggttttacttatccctgtgatggtccaggtagaggt
ggtacatgtcaaatatcaccttgggatcacttattctcagctgtattttggatgtataac
tgtttaaatgtagtaacattccactacttttggaagatgcaatcagatgtttggggcttt
gtatcgatccaaaagcacatatctcactacagccaaggtgattttagtgtcaactctatt
accatcaatggttggttgagaaacttattatggtccgaggcatcccaagtaatccaatcc
tatgctctttcatccatttgtccatatgggttcattttcctcatagctcatttcatctgg
gcatttagtttaatgttcctctttagtggtagagcatactggcaagaattaattgagtcc
attctatggtcacatcataaattaaagattatacctcatatccaaccaagggcattaagc
atttctcaaggaagagcagttggtttcattcattacactctaggaggtatcggctcaacc
tgggcatttattatttcaagactgctagtattaacttcttga
>KrA3_psaA
atgcacatcttcagatatatcaacacaacactgtgggccaaagcaggacattttaataaa
gctttatcaaaaggagctaagacaactacttggatatggaatctacatgattatgctcat
gattttgacattcaacaaagatccacaggtttaatagcaagaaaagtcttctcatccaat
ctagctcatctatctttggtattcttttggattagtggaatgcaccttcatggggcatat

ttgtctaattatgatatttggttaaaagatcccaaatccatcaccccatcatcacatcta
gcctactctttaattggtcaagatattcttaactcatatacctcagaatacttctcagga
atcactatcacatctggctttttccaactctaccgttctgagggtatcattacacagtcc
cagctgaaatatgcatgtgctacttccctaatagctacacttatctgcctttctggatct
tatctccacatgcagcttatgtccaaattcactagcttttacaagaagttccagtcctta
tcgcaagatcatctcataattatatttggctccagctccacatccctctccgctcatcag
atacacaaaatgcttccagctaaccctttactagattcaggtatctccaacccaagtatc
ctacaagtaatctccaacagtctcagtgaaaacctagcactgttttcaacaaatctaagc
tctacaggtaaattgttaaatccctcaacaagaagtgtattcctttctcaagttgcagca
catcataagacaacaggtgtagtctttatcacactagggcttatcagattcctcaccatg
tataactctcaatttagtatacttacatcttatatagattaccacattgtgctatctatt
aatttagcattaattgcatctttatcaataatagtagctgatcatctcacaagaacccca
atctatcctcacaactcaacctcataccctacaatcctttgcctctccatacatcatgca
tggttatccggtttcctcatcattggttcaggagctcatgcatcaatattcaacttgcga
gcatcaccaacatctgagataagacatcgagaccccatatactcacacctcatctgggta
tgtatagcaataggattacactcattcagtctctactgtcataacgacactttagaagca
ttaggacgtccagaagatatctttcatgacaactctatccagttaaaagcaatatttgcc
aagcaatcctttctaagagcagagctccagccagatatcgagatgttagataaaaagatt
atacgtataacccaagaattgggaacggcagattttatagttcaccacattcatgcattt
acaatccatgttactctcttaattctttcaaagggtgtactttatgctagaaactctaga
tttgtatcagataaattggaacttggttttacttatccctgtgatggtccaggtagaggt
ggtacatgtcaaatatcaccttgggatcacttattctcagctgtattttggatgtataac
tgtttaaatgtagtaacattccactacttttggaagatgcaatcagatgtttggggcttt
gtatcgatccaaaagcacatatctcactacagccaaggtgattttagtgtcaactctatt
accatcaatggttggttgagaaacttattatggtccgaggcatcccaagtaatccaatcc
tatgctctttcatccatttgtccatatgggttcattttcctcatagctcatttcatctgg
gcatttagtttaatgttcctctttagtggtagagcatactggcaagaattaattgagtcc
attctatggtcacatcataaattaaagattatacctcatatccaaccaagggcattaagc
atttctcaaggaagagcagttggtttcattcattacactctaggaggtatcggctcaacc
tgggcatttattatttcaagactgctagtattaacttcttga
>KrA4_psaA
atgcacatcttcagatatatcaacacaacactgtggtccaaagcaggacattttaataaa
gctttatcaaaaggagctaagacaactacttggatatggaatctacatgattatgctcat
gattttgacattcaacaaagatccacaggtttaatagcaagaaaagtcttctcatccaat
ctagctcatctatctttggtattcttttggattagtggaatgcaccttcatggggcatat
ttgtctaattatgatatttggttaaaagatcccaaatccatcaccccatcatcacatcta
gcctactctttaattggtcaagatattcttaactcatatacctcagaatacttctcagga
atcactatcacatctggctttttccaactctaccgttctgagggtatcattacacagtcc
cagctgaaatatgcatgtgctacttccctaatagctacacttatctgcctttctggatct
tatctccacatgcagcttatgtccaaattcactagcttttacaagaagttccagtcctta
tcgcaagatcatctcataattatatttggctccagctccacatccctctccgctcatcag
atacacaaaatgcttccagctaaccctttactagattcaggtatctccaacccaagtatc
ctacaagtaatctccaacagtctcagctatacactagcactgttttcaacaaatctaagc
tctacaggtaaattgttaaatccctcaacaagaagtgtattcctttctcaagttgcagca
catcataagacaacaggtgtagtctttatcacactagggcttatcagattcctcaccatg
tataactctcaatttagtatacttacatcttatatagattaccacattgtgctatctatt
aatttagcattaattgcatctttatcaataatagtagctgatcatctcacaagaacccca
atctatcctcacaactcaacctcataccctacaatcctttgcctctccatacatcatgca
tggttatccggtttcctcatcattggttcaggagctcatgcatcaatattcaacttgcga
gcatcaccaacatctgagataagacatcgagaccccatatactcacacctcatctgggta
tgtatagcaataggattacactcattcagtctctactgtcataacgacactttagaagca
ttaggacgtccagaagatatctttcatgacaactctatccagttaaaagcaatatttgcc
aagcaatcctttctaagagcagagctccagccagatatcgagatgttagataaaaagatt
atacgtataacccaagaattgggaacagcagattttatagttcaccacattcatgcattt
acaatccatgttactctcttaattctttcaaagggtgtactttatgctagaaactctaga
tttgtatcagataaattggaacttggttttacttatccctgtgatggtccaggtagaggt
ggtacatgtcaaatatcaccttgggatcacttattctcagctgtattttggatgtataac

tgtttaaatgtagtaacattccactacttttggaagatgcaatcagatgtttggggcttt
gtatcgatccaaaagcacatatctcactacagccaaggtgattttagtgtcaactctatt
accatcaatggttggttgagaaacttattatggtccgaggcatcccaagtaatccaatcc
tatgctctttcatccatttgtccatatgggttcattttcctcatagctcatttcatctgg
gcatttagtttaatgttcctctttagtggtagagcatactggcaagaattaattgagtcc
attctatggtcacatcataaattaaagattatacctcatatccaaccaagggcattaagc
atttctcaaggaagagcagttggtttcattcattacactctaggaggtatcggctcaacc
tgggcatttattatttcaagactgctagtattaacttcttga
>KrA5_psaA
atgcacatcttcagatatatcaacacaacactgtggtccaaagcaggacattttaataaa
gctttatcaaaaggagctaagacaactacttggatatggaatctacatgattatgctcat
gattttgacattcaacaaagatccacaggtttaatagcaagaaaagtcttctcatccaat
ctagctcatctatctttggtattcttttggattagtggaatgcaccttcatggggcatat
ttgtctaattatgatatttggttaaaagatcccaaatccatcaccccatcatcacatcta
gcctactctttaattggtcaagatattcttaactcatatacctcagaatacttctcagga
atcactatcacatctggctttttccaactctaccgttctgagggtatcattacacagtcc
cagctgaaatatgcatgtgctacttccctaatagctacacttatctgcctttctggatct
tatctccacatgcagcttatgtccaaattcactagcttttacaagaagttccagtcctta
tcgcaagatcatctcataattatatttggctccagctccacatccctctccgctcatcag
atacacaaaatgcttccagctaaccctttactagattcaggtatctccaacccaagtatc
ctacaagtaatctccaacagtctcagctatacactagcactgttttcaacaaatctaagc
tctacaggtaaattgttaaatccctcaacaagaagtgtattcctttctcaagttgcagca
catcataagacaacaggtgtagtctttatcacactagggcttatcagattcctcaccatg
tataactctcaatttagtatacttacatcttatatagattaccacattgtgctatctatt
aatttagcattaattgcatctttatcaataatagtagctgatcatctcacaagaacccca
atctatcctcacaactcaacctcataccctacaatcctttgcctctccatacatcatgca
tggttatccggtttcctcatcattggttcaggagctcatgcatcaatattcaacttgcga
gcatcaccaacatctgagataagacatcgagaccccatatactcacacctcatctgggta
tgtatagcaataggattacactcattcagtctctactgtcataacgacactttagaagca
ttaggacgtccagaagatatctttcatgacaactctatccagttaaaagcaatatttgcc
aagcaatcctttctaagagcagagctccagccagatatcgagatgttagataaaaagatt
atacgtataacccaagaattgggaacagcagattttatagttcaccacattcatgcattt
acaatccatgttactctcttaattctttcaaagggtgtactttatgctagaaactctaga
tttgtatcagataaattggaacttggttttacttatccctgtgatggtccaggtagaggt
ggtacatgtcaaatatcaccttgggatcacttattctcagctgtattttggatgtataac
tgtttaaatgtagtaacattccactacttttggaagatgcaatcagatgtttggggcttt
gtatcgatccaaaagcacatatctcactacagccaaggtgattttagtgtcaactctatt
accatcaatggttggttgagaaacttattatggtccgaggcatcccaagtaatccaatcc
tatgctctttcatccatttgtccatatgggttcattttcctcatagctcatttcatctgg
gcatttagtttaatgttcctctttagtggtagagcatactggcaagaattaattgagtcc
attctatggtcacatcataaattaaagattatacctcatatccaaccaagggcattaagc
atttctcaaggaagagcagttggtttcattcattacactctaggaggtatcggctcaacc
tgggcatttattatttcaagactgctagtattaacttcttga
>KrA6_psaA
atgcacatcttcagatatatcaacacaacactgtggtccaaagcaggacattttaataaa
gctttatcaaaaggagctaagacaactacttggatatggaatctacatgattatgctcat
gattttgacattcaacaaagatccacaggtttaatagcaagaaaagtcttctcatccaat
ctagctcatctatctttggtattcttttggattagtggaatgcaccttcatggggcatat
ttgtctaattatgatatttggttaaaagatcccaaatccatcacgccatcatcacatcta
gcctactctttaattggtcaagatattcttaactcatatacctcagaatacttctcagga
atcactatcacatctggctttttccaactctaccgttctgagggtatcattacacagtcc
cagctcaaatatgcatgtgctacttccctaatagctacacttatctgcctttctggatct
tatctccacatgcagcttatgtccaaattcactagcttttacaagaagttccagtcctta
tcgcaagatcatctcataattatatttggctccagctccacatccctctccgctcatcag
atacacaaaatgcttccagctaaccctttactagattcaggtatctccaacccaagtatc
ctacaagtaatctccaacagtctcagtgaaaacctagcactgttttcaacaaatctaagc
tctacaggtaaattgttaaatccctcaacaagaagtgtattcctttctcaagttgcagca

catcataagacaacaggtgtagtctttatcacactagggcttatcagattcctcaccatg
tataactctcaatttagtatacttacatcttatatagattaccacattgtgctatctatt
aatttagcattaattgcatctttatcaataatagtagctgatcatctcacaagaacccca
atctatcctcacaactcaacctcataccctacaatcctttgcctctccatacatcatgca
tggttatccggtttcctcatcattggttcaggagctcatgcatcaatattcaacttgcga
gcatcaccaacatctgagataagacatcgagaccccatatactcacacctcatctgggta
tgtatagcaataggattacactcattcagtctctactgtcataacgacactttagaagca
ttaggacgtccagaagatatctttcatgacaactctatccagttaaaagcaatatttgcc
aagcaatcctttctaagagcagagctccagccagatatcgagatgttagataaaaagatt
atacgtataacccaagaattgggaacagcatattttatagttcaccacattcatgcattt
acaatccatgttactctcttaattctttcaaagggtgtactttatgctagaaactctaga
tttgtatcagataaattggaacttggttttacttatccctgtgatggtccaggtagaggt
ggtacatgtcaaatatcaccttgggatcacttattctcagctgtattttggatgtataac
tgtttaaatgtagtaacattccactacttttggaagatgcaatcagatgtttggggcttt
gtatcgatccaaaagcacatatctcactacagccaaggtgattttagtgtcaactctatt
accatcaatggttggttgagaaacttattatggtccgaggcatcccaagtaatccaatcc
tatgctctttcatccatttgtccatatgggttcattttcctcatagctcatttcatctgg
gcatttagtttaatgttcctctttagtggtagagcatactggcaagaattaattgagtcc
attctatggtcacatcataaattaaagattatacctcatatccaaccaagggcattaagc
atttctcaaggaagagcagttggtttcattcattacactctaggaggtatcggctcaacc
tgggcatttattatttcaagactgctagtattaacttcttga
>KrA7_psaA
atgcacatcttcagatatatcaacacaacactgtggtccaaagcaggacattttaataaa
gctttatcaaaaggagctaagacaactacttggatatggaatctacatgattatgctcat
gattttgacattcaacaaagatccacaggtttaatagcaagaaaagtcttctcatccaat
ctagctcatctatctttggtattcttttggattagtggaatgcaccttcatggggcatat
ttgtctaattatgatatttggttaaaagatcccaaatccatcaccccatcatcacatcta
gcctactctttaattggtcaagatattcttaactcatatacctcagaatacttctcagga
atcactatcacatctggctttttccaactctaccgttctgagggtatcattacacagtcc
cagctgaaatatgcatgtgctacttccctaatagctacacttatctgcctttctggatct
tatctccacatgcagcttatgtccaaattcactagcttttacaagaagttccagtcctta
tcgcaagatcatctcataattatatttggctccagctccacatccctctccgctcatcag
atacacaaaatgcttccagctaacccttttactagattcaggtatctccaacccaagtatc
ctacaagtaatctccaacagtctcagcgaaaacctagcactgttttcaacaaatctaagc
tctacaggtaaattgttaaatccctcaacaagaagtgtattcctttctcaagttgcagca
catcataagacaacaggtgtagtctttatcacactagggcttatcagattcctcaccatg
tataactctcaatttagtatacttacatcttatatagattaccacattgtgctatctatt
aatttagcattaattgcatctttatcaataatagtagctgatcatctcacaagaacccca
atctatcctcacaactcaacctcataccctacaatcctttgcctctccatacatcatgca
tggttatccggtttcctcatcattggttcaggagctcatgcatcaatattcaacttgcga
gcatcaccaacatctgagataagacatcgagaccccatatactcacacctcatctgggta
tgtatagcaataggattacactcattcagtctctactgtcataacgacactttagaagca
ttaggacgtccagaagatatctttcatgacaactctatccagttaaaagcaatatttgcc
aagcaatcctttctaagagcagagctccagccagatatcgagatgttagataaaaagatt
atacgtataacccaagaattgggaacagcagattttatagttcaccacattcatgcattt
acaatccatgttactctcttaattctttcaaagggtgtactttatgctagaaactctaga
tttgtatcagataaattggaacttggttttacttatccctgtgatgggccaggtagaggt
ggtacatgtcaaatatcaccttgggatcacttattctcagctgtattttggatgtataac
tgtttaaatgtagtaacattccactacttttggaagatgcaatcagatgtttggggcttt
gtatcgatccaaaagcacatatctcactacagccaaggtgattttagtgtcaactctatt
accatcaatggttggttgagaaacttattatggtccgaggcatcccaagtaatccaatcc
tatgctctttcatccatttgtccatatgggttcattttcctcatagctcatttcatctgg
gcatttagtttaatgttcctctttagtggtagagcatactggcaagaattaattgagtcc
attctatggtcacatcataaattaaagattatacctcatatccaaccaagggcattaagc
atttctcaaggaagagcagttggtttcattcattacactctaggaggtatcggctcaacc
tgggcatttattatttcaagactgctagtattaacttcttga
>KrA8_psaA

atgcacatcttcagatatatcaacacaacactgtggtccaaagcaggacattttaataaa
gctttatcaaaaggagctaagacaactacttggatatggaatctacatgattatgctcat
gattttgacattcaacaaagatccacaggtttaatagcaagaaaagtcttctcatccaat
ctagctcatctatctttggtattcttttggattagtggaatgcaccttcatggggcatat
ttgtctaattatgatatttggttaaaagatcccaaatccatcaccccatcatcacatcta
gcctactctttaattggtcaagatattcttaactcatatacctcagaatacttctcagga
atcactatcacatctggctttttccaactctaccgttctgagggtatcattacacagtcc
cagctgaaatatgcatgtgctacttccctaatagctacacttatctgcctttctggatct
tatctccacatgcagcttatgtccaaattcactagcttttacaagaagttccagtcctta
tcgcaagatcatctcataattatatttggctccagctccacatccctctccgctcatcag
atacacaaaatgcttccagctaaccctttactagattcaggtatctccaacccaagtatc
ctacaagtaatctccaacagtctcagtgaaaacctagcactgttttcaacaaatctaagc
tctacaggtaaattgttaaatccctcaacaagaagtgtattcctttctcaagttgcagca
catcataagacaacaggtgtagtctttatcacactagggcttatcagattcctcaccatg
tataactctcaatttagtatacttacatcttatatagattaccacattgtgctatctatt
aatttagcattaattgcatctttatcaataatagtagctgatcatctcacaagaacccca
atctatcctcacaactcaacctcataccctacaatcctttgcctctccatacatcatgca
tggttatccggtttcctcatcattggttcaggagctcatgcatcaatattcaacttgcga
gcatcaccaacatctgagataagacatcgagaccccatatactcacacctcatctgggta
tgtatagcaataggattacactcattcagtctctactgtcataacgacactttagaagca
ttaggacgtccagaagatatctttcatgacaactctatccagttaaaagcaatatttgcc
aagcaatcctttctaagagcagagctccagccagatatcgagatgttagataaaaagatt
atacgtataacccaagaattgggaacagcagattttatagttcaccacattcatgcattt
acaatccatgttactctcttaattctttcaaagggtgtactttatgctagaaactctaga
tttgtatcagataaattggaacttggttttacttatccctgtgatggtccaggtagaggt
ggtacatgtcaaatatcaccttgggatcacttattctcagctgtattttggatgtataac
tgtttaaatgtagtaacattccactacttttggaagatgcaatcagatgtttggggcttt
gtatcgatccaaaagcacatatctcactacagccaaggtgattttagtgtcaactctatt
accatcaatggttggttgagaaacttattatggtccgaggcatcccaagtaatccaatcc
tatgctctttcatccatttgtccatatgggttcattttcctcatagctcatttcatctgg
gcatttagtttaatgttcctctttagtggtagagcatactggcaagaattaattgagtcc
attctatggtcacatcataaattaaagattatacctcatatccaaccaagggcattaagc
atttctcaaggaagagcagttggtttcattcattacactctaggaggtatcggctcaacc
tgggcatttattatttcaagactgctagtattaacttcttga
>KrA9_psaA
atgcacatcttcagatatatcaacacaacactgtggtccaaagcaggacattttaataaa
gctttatcaaaaggagctaagacaactacttggatatggaatctacatgattatgctcat
gattttgacattcaacaaagatccacaggtttaatagcaagaaaagtcttctcatccaat
ctagctcatctatctttggtattcttttggattagtggaatgcaccttcatggggcatat
ttgtctaattatgatatttggttaaaagatcccaaatccatcaccccatcatcacatcta
gcctactctttaattggtcaagatattcttaactcatatacctcagaatacttctcagga
atcactatcacatctggctttttccaactctaccgttctgagggtatcattacacagtcc
cagctgaaatatgcatgtgctacttccctaatagctacacttatctgcctttctggatct
tatctccacatgcagcttatgtccaaattcactagcttttacaagaagttccagtcctta
tcgcaagatcatctcataattatatttggctccagctccacatccctctccgctcatcag
atacacaaaatgcttccagctaaccctttactagattcaggtatctccaacccaagtatc
ctacaagtaatctccaacagtctcagtgaaaacctagcactgttttcaacaaatctaagc
tctacaggtaaattgttaaatccctcaacaagaagtgtattcctttctcaagttgcagca
catcataagacaacaggtgtagtctttatcacactagggcttatcagattcctcaccatg
tataactctcaatttagtatacttacatcttatatagattaccacattgtgctatctatt
aatttagcattaattgcatctttatcaataatagtagctgatcatctcacaagaacccca
atctatcctcacaactcaacctcataccctacaatcctttgcctctccatacatcatgca
tggttatccggtttcctcatcattggttcaggagctcatgcatcaatattcaacttgcga
gcatcaccaacatctgagataagacatcgagaccccatatactcacacctcatctgggta
tgtatagcaataggattacactcattcagtctctactgtcataacgacactttagaagca
ttaggacgtccagaagatatctttcatgacaactctatccagttaaaagcaatatttgcc
aagcaatcctttctaagagcagagctccagccagatatcgagatgttagataaaaagatt

atacgtataacccaagaattgggaacagcagattttatagttcaccacattcatgcattt
acaatccatgttactctcttaattctttcaaagggtgtactttatgctagaaactctaga
tttgtatcagataaattggaacttggttttacttatccctgtgatggtccaggtagaggt
ggtacatgtcaaatatcaccttgggatcacttattctcagctgtattttggatgtataac
tgtttaaatgtagtaacattccactacttttggaagatgcaatcagatgtttggggcttt
gtatcgatccaaaagcacatatctcactacagccaaggtgattttagtgtcaactctatt
accatcaatggttggttgagaaacttattatggtccgaggcatcccaagtaatccaatcc
tatgctctttcatccatttgtccatatgggttcattttcctcatagctcatttcatctgg
gcatttagtttaatgttcctctttagtggtagagcatactggcaagaattaattgagtcc
attctatggtcacatcataaattaaagattatacctcatatccaaccaagggcattaagc
atttctcaaggaagagcagttggtttcattcattacactctaggaggtatcggctcaacc
tgggcatttattatttcaagactgctagtattaacttcttga
>KrC1_psaA
atgcacatcttcagatatatcaacacaacactgtggtccaaagcaggacattttaataaa
gctttatcaaaaggagctaagacaactacttggatatggaatctacatgattatgctcat
gattttgacattcaacaaagatccacaggtttaatagcaagaaaagtcttctcatccaat
ctagctcatctatctttggtattcttttggattagtggaatgcaccttcatggggcatat
ttgtctaattatgatatttggttaaaagatcccaaatccatcaccccatcatcacatcta
gcctactctttaattggtcaagatattcttaactcatatacctcagaatacttctcagga
atcactatcacatctggctttttccaactctaccgttctgagggtatcattacacagtcc
cagctgaaatatgcatgtgctacttccctaatagctacacttatctgcctttctggatct
tatctccacatgcagcttatgtccaaattcactagcttttacaagaagttccagtcctta
tcgcaagatcatctcataattatatttggctccagctccacatccctctccgctcatcag
atacacaaaatgcttccagctaaccctttactagattcaggtatctccaacccaagtatc
ctacaagtaatctccaacagtctcagtgaaaacctagcactgttttcaacaaatctaagc
tctacaggtaaattgttaaatccctcaacaagaagtgtattcctttctcaagttgcagca
catcataagacaacaggtgtagtctttatcacactagggcttatcagattcctcaccatg
tataactctcaatttagtatacttacatcttatatagattaccacattgtgctatctatt
aatttagcattaattgcatctttatcaataatagtagctgatcatctcacaagaacccca
atctatcctcacaactcaacctcataccctacaatcctttgcctctccatacatcatgca
tggttatccggtttcctcatcattggttcaggagctcatgcatcaatattcaacttgcga
gcatcaccaacatctgagataagacatcgagaccccatatactcacacctcatctgggta
tgtatagcaataggattacactcattcagtctctactgtcataacgacactttagaagca
ttaggacgtccagaagatatctttcatgacaactctatccagttaaaagcaatatttgcc
aagcaatcctttctaagagcagagctccagccagatatcgagatgttagataaaaagatt
atacgtataacccaagaattgggaacagcagattttatagttcaccacattcatgcattt
acaatccatgttactctcttaattctttcaaagggtgtactttatgctagaaactctaga
tttgtatcagataaattggaacttggttttacttatccctgtgatggtccaggtagaggt
ggtacatgtcaaatatcaccttgggatcacttattctcagctgtattttggatgtataac
tgtttaaatgtagtaacattccactacttttggaagatgcaatcagatgtttggggcttt
gtatcgatccaaaagcacatatctcactacagccaaggtgattttagtgtcaactctatt
accatcaatggttggttgagaaacttattatggtccgaggcatcccaagtaatccaatcc
tatgctctttcatccatttgtccatatgggttcattttcctcatagctcatttcatctgg
gcatttagtttaatgttcctctttagtggtagagcatactggcaagaattaattgagtcc
attctatggtcacatcataaattaaagattatacctcatatccaaccaagggcattaagc
atttctcaaggaagagcagttggtttcattcattacactctaggaggtatcggctcaacc
tgggcatttattatttcaagactgctagtattaacttcttga
>KrC10_psaA
atgcacatcttcagatatatcaacacaacactgtggtccaaagcaggacattttaataaa
gctttatcaaaaggagctaagacaactacttggatatggaatctacatgattatgctcat
gattttgacattcaacaaagatccacaggtttaatagcaagaaaagtcttctcatccaat
ctagctcatctatctttggtattcttttggattagtggaatgcaccttcatggggcatat
ttgtctaattatgatatttggttaaaagatcccaaatccatcaccccatcatcacatcta
gcctactctttaattggtcaagatattcttaactcatatacctcagaatacttctcagga
atcactatcacatctggctttttccaactctaccgttctgagggtatcattacacagtcc
cagctgaaatatgcatgtgctacttccctaatagctacacttatctgcctttctggatct
tatctccacatgcagcttatgtccaaattcactagcttttacaagaagttccagtcctta

tcgcaagatcatctcataattatatttggctccagctccacatccctctccgctcatcag
atacacaaaatgcttccagctaaccctttactagattcaggtatctccaacccaagtatc
ctacaagtaatctccaacagtctcagtgaaaacctagcactgttttcaacaaatctaagc
tctacaggtaaattgttaaatccctcaacaagaagtgtattcctttctcaagttgcagca
catcataagacaacaggtgtagtctttatcacactagggcttatcagattcctcaccatg
tataactctcaatttagtatacttacatcttatatagattaccacattgtgctatctatt
aatttagcattaattgcatctttatcaataatagtagctgatcatctcacaagaacccca
atctatcctcacaactcaacctcataccctacaatcctttgcctctccatacatcatgca
tggttatccggtttcctcatcattggttcaggagctcatgcatcaatattcaacttgcga
gcatcaccaacatctgagataagacatcgagaccccatatactcacacctcatctgggta
tgtatagcaataggattacactcattcagtctctactgtcataacgacactttagaagca
ttaggacgtccagaagatatctttcatgacaactctatccagttaaaagcaatatttgcc
aagcaatcctttctaagagcagagctccagccagatatcgagatgttagataaaaagatt
atacgtataacccaagaattgggaacagcagattttatagttcaccacattcatgcattt
acaatccatgttactctcttaattctttcaaagggtgtactttatgctagaaactctaga
tttgtatcagataaattggaacttggttttacttatccctgtgatggtccaggtagaggt
ggtacatgtcaaatatcaccttgggatcacttattctcagctgtattttggatgtataac
tgtttaaatgtagtaacattccactacttttggaagatgcaatcagatgtttggggcttt
gtatcgatccaaaagcacatatctcactacagccaaggtgattttagtgtcaactctatt
accatcaatggttggttgagaaacttattatggtccgaggcatcccaagtaatccaatcc
tatgctctttcatccatttgtccatatgggttcattttcctcatagctcatttcatctgg
gcatttagtttaatgttcctctttagtggtagagcatactggcaagaattaattgagtcc
attctatggtcacatcataaattaaagattatacctcatatccaaccaagggcattaagc
atttctcaaggaagagcagttggtttcattcattacactctaggaggtatcggctcaacc
tgggcatttattatttcaagactgctagtattaacttcttga
>KrC11_psaA
atgcacatcttcagatatatcaacacaacactgtggtccaaagcaggacattttaataaa
gctttatcaaaaggagctaagacaactacttggatatggaatctacatgattatgctcat
gattttgacattcaacaaagatccacaggtttaatagcaagaaaagtcttctcatccaat
ctagctcatctatctttggtattcttttggattagtggaatgcaccttcatggggcatat
ttgtctaattatgatatttggttaaaagatcccaaatccatcaccccatcatcacatcta
gcctactctttaattggtcaagatattcttaactcatatacctcagaatacttctcagga
atcactatcacatctggctttttccaactctaccgttctgagggtatcattacacagtcc
cagctgaaatatgcatgtgctacttccctaatagctacacttatctgcctttctggatct
tatctccacatgcagcttatgtccaaattcactagcttttacaagaagttccagtcctta
tcgcaagatcatctcataattatatttggctccagctccacatccctctccgctcatcag
atacacaaaatgcttccagctaaccctttactagattcaggtatctccaacccaagtatc
ctacaagtaatctccaacagtctcagtgaaaacctagcactgttttcaacaaatctaagc
tctacaggtaaattgttaaatccctcaacaagaagtgtattcctttctcaagttgcagca
catcataagacaacaggtgtagtctttatcacactagggcttatcagattcctcaccatg
tataactctcaatttagtatacttacatcttatatagattaccacattgtgctatctatt
aatttagcattaattgcatctttatcaataatagtagctgatcatctcacaagaacccca
atctatcctcacaactcaacctcataccctacaatcctttgcctctccatacatcatgca
tggttatccggtttcctcatcattggttcaggagctcatgcatcaatattcaacttgcga
gcatcaccaacatctgagataagacatcgagaccccatatactcacacctcatctgggta
tgtatagcaataggattacactcattcagtctctactgtcataacgacactttagaagca
ttaggacgtccagaagatatctttcatgacaactctatccagttaaaagcaatatttgcc
aagcaatcctttctaagagcagagctccagccagatatcgagatgttagataaaaagatt
atacgtataacccaagaattgggaacagcagattttatagttcaccacattcatgcattt
acaatccatgttactctcttaattctttcaaagggtgtactttatgctagaaactctaga
tttgtatcagataaattggaacttggttttacttatccctgtgatggtccaggtagaggt
ggtacatgtcaaatatcaccttgggatcacttattctcagctgtattttggatgtataac
tgtttaaatgtagtaacattccactacttttggaagatgcaatcagatgtttggggcttt
gtatcgatccaaaagcacatatctcactacagccaaggtgattttagtgtcaactctatt
accatcaatggttggttgagaaacttattatggtccgaggcatcccaagtaatccaatcc
tatgctctttcatccatttgtccatatgggttcattttcctcatagctcatttcatctgg
gcatttagtttaatgttcctctttagtggtagagcatactggcaagaattaattgagtcc

attctatggtcacatcataaattaaagattatacctcatatccaaccaagggcattaagc
atttctcaaggaagagcagttggtttcattcattacactctaggaggtatcggctcaacc
tgggcatttattatttcaagactgctagtattaacttcttga
>KrC12_psaA
atgcacatcttcagatatatcaacacaacactgtggtccaaagcaggacattttaataaa
gctttatcaaaaggagctaagacaactacttggatatggaatctacatgattatgctcat
gattttgacattcaacaaagatccacaggtttaatagcaagaaaagtcttctcatccaat
ctagctcatctatctttggtattcttttggattagtgcaatgcaccttcatggggcatat
ttgtctaattatgatatttggttaaaagatcccaaatccatcaccccatcatcacatcta
gcctactctttaattggtcaagatattcttaactcatatacctcagaatacttctcagga
atcactatcacatctggctttttccaactctaccgttctgagggtatcattacacagtcc
cagctgaaatatgcatgtgctacttccctaatagctacacttatctgcctttctggatct
tatctccacatgcagcttatgtccaaattcactagcttttacaagaagttccagtcctta
tcgcaagatcatctcataattatatttggctccagctccacatccctctccgctcatcag
atacacaaaatgcttccagctaaccctttactagattcaggtatctccaacccaagtatc
ctacaagtaatctccaacagtctcagtgaaaacctagcactgttttcaacaaatctaagc
tctacaggtaaattgttaaatccctcaacaagaagtgtattcctttctcaagttgcagca
catcataagacaacaggtgtagtctttatcacactagggcttatcagattcctcaccatg
tataactctcaatttagtatacttacatcttatatagattaccacattgtgctatctatt
aatttagcattaattgcatctttatcaataatagtagctgatcatctcacaagaacccca
atctatcctcacaactcaacctcataccctacaatcctttgcctctccatacatcatgca
tggttatccggtttcctcatcattggttcaggagctcatgcatcaatattcaacttgcga
gcatcaccaacatctgagataagacatcgagaccccatatactcacacctcatctgggta
tgtatagcaataggattacactcattcagtctctactgtcataacgacactttagaagca
ttaggacgtccagaagatatctttcatgacaactctatccagttaaaagcaatatttgcc
aagcaatcctttctaagagcagagctccagccagatatcgagatgttagataaaaagatt
atacgtataacccaagaattgggaacagcagattttatagttcaccacattcatgcattt
acaatccatgttactctcttaattctttcaaagggtgtactttatgctagaaactctaga
tttgtatcagataaattggaacttggttttacttatccctgtgatgggccaggtagaggt
ggtacatgtcaaatatcaccttgggatcacttattctcagctgtattttggatgtataac
tgtttaaatgtagtaacattccactacttttggaagatgcaatcagatgtttggggcttt
gtatcgatccaaaagcacatatctcactacagccaaggtgattttagtgtcaactctatt
accatcaatggttggttgagaaacttattatggtccgaggcatcccaagtaatccaatcc
tatgctctttcatccatttgtccatatgggttcattttcctcatagctcatttcatctgg
gcatttagtttaatgttcctctttagtggtagagcatactggcaagaattaattgagtcc
attctatggtcacatcataaattaaagattatacctcatatccaaccaagggcattaagc
atttctcaaggaagagcagttggtttcattcattacactctaggaggtatcggctcaacc
tgggcatttattatttcaagactgctagtattaacttcttga
>KrC13_psaA
atgcacatcttcagatatatcaacacaacactgtggtccaaagcaggacattttaataaa
gctttatcaaaaggagctaagacaactacttggatatggaatctacatgattatgctcat
gattttgacattcaacaaagatccacaggtttaatagcaagaaaagtcttctcatccaat
ctagctcatctatctttggtattcttttggattagtggaatgcaccttcatggggcatat
ttgtctaattatgatatttggttaaaagatcccaaatccatcaccccatcatcacatcta
gcctactctttaattggtcaagatattcttaactcatatacctcagaatacttctcagga
atcactatcacatctggctttttccaactctaccgttctgagggtatcattacacagtcc
cagctgaaatatgcatgtgctacttccctaatagctacacttatctgcctttctggatct
tatctccacatgcagcttatgtccaaattcactagcttttacaagaagttccagtcctta
tcgcaagatcatctcataattatatttggctccagctccacatccctctccgctcatcag
atacacaaaatgcttccagctaaccctttactagattcaggtatctccaacccaagtatc
ctacaagtaatctccaacagtctcagtgaaaacctagcactgttttcaacaaatctaagc
tctacaggtaaattgttaaatccctcaacaagaagtgtattcctttctcaagttgcagca
catcataagacaacaggtgtagtctttatcacactagggcttatcagattcctcaccatg
tataactctcaatttagtatacttacatcttatatagattaccacattgtgctatctatt
aatttagcattaattgcatctttatcaataatagtagctgatcatctcacaagaacccca
atctatcctcacaactcaacctcataccctacaatcctttgcctctccatacatcatgca
tggttatccggtttcctcatcattggttcaggagctcatgcatcaatattcaacttgcga

gcatcaccaacatctgagataagacatcgagaccccatatactcacacctcatctgggta
tgtatagcaataggattacactcattcagtctctactgtcataacgacactttagaagca
ttaggacgtccagaagatatctttcatgacaactctatccagttaaaagcaatatttgcc
aagcaatcctttctaagagcagagctccagccagatatcgagatgttagataaaaagatt
atacgtataacccaagaattgggaacagcagattttatagttcaccacattcatgcattt
acaatccatgttactctcttaattctttcaaagggtgtactttatgctagaaactctaga
tttgtatcagataaattggaacttggttttacttatccctgtgatggtccaggtagaggt
ggtacatgtcaaatatcaccttgggatcacttattctcagctgtattttggatgtataac
tgtttaaatgtagtaacattccactacttttggaagatgcaatcagatgtttggggcttt
gtatcgatccaaaagcacatatctcactacagccaaggtgattttagtgtcaactctatt
accatcaatggttggttgagaaacttattatggtccgaggcatcccaagtaatccaatcc
tatgctctttcatccatttgtccatatgggttcattttcctcatagctcatttcatctgg
gcatttagtttaatgttcctctttagtggtagagcatactggcaagaattaattgagtcc
attctatggtcacatcataaattaaagattatacctcatatccaaccaagggcattaagc
atttctcaaggaagagcagttggtttcattcattacactctaggaggtatcggctcaacc
tgggcatttattatttcaagactgctagtattaacttcttga
>KrC14_psaA
atgcacatcttcagatatatcaacacaacactgtggtccaaagcaggacattttaataaa
gctttatcaaaaggagctaagacaactacttggatatggaatctacatgattatgctcat
gattttgacattcaacaaagatccacaggtttaatagcaagaaaagtcttctcatccaat
ctagctcatctatctttggtattcttttggattagtggaatgcaccttcatggggcatat
ttgtctaattatgatatttggttaaaagatcccaaatccatcaccccatcatcacatcta
gcctactctttaattggtcaagatattcttaactcatatacctcagaatacttctcagga
atcactatcacatctggctttttccaactctaccgttctgagggtatcattacacagtcc
cagctgaaatatgcatgtgctacttccctaatagctacacttatctgcctttctggatct
tatctccacatgcagcttatgtccaaattcactagcttttacaagaagttccagtcctta
tcgcaagatcatctcataattatatttggctccagctccacatccctctccgctcatcag
atacacaaaatgcttccagctaaccctttactagattcaggtatctccaacccaagtatc
ctacaagtaatctccaacagtctcagtgaaaacctagcactgttttcaacaaatctaagc
tctacaggtaaattgttaaatccctcaacaagaagtgtattcctttctcaagttgcagca
catcataagacaacaggtgtagtctttatcacactagggcttatcagattcctcaccatg
tataactctcaatttagtatacttacatcttatatagattaccacattgtgctatctatt
aatttagcattaattgcatctttatcaataatagtagctgatcatctcacaagaacccca
atctatcctcacaactcaacctcataccctacaatcctttgcctctccatacatcatgca
tggttatccggtttcctcatcattggttcaggagctcatgcatcaatattcaacttgcga
gcatcaccaacatctgagataagacatcgagaccccatatactcacacctcatctgggta
tgtatagcaataggattacactcattcagtctctactgtcataacgacactttagaagca
ttaggacgtccagaagatatctttcatgacaactctatccagttaaaagcaatatttgcc
aagcaatcctttctaagagcagagctccagccagatatcgagatgttagataaaaagatt
atacgtataacccaagaattgggaacagcagattttatagttcaccacattcatgcattt
acaatccatgttactctcttaattctttcaaagggtgtactttatgctagaaactctaga
tttgtatcagataaattggaacttggttttacttatccctgtgatggtccaggtagaggt
ggtacatgtcaaatatcaccttgggatcacttattctcagctgtattttggatgtataac
tgtttaaatgtagtaacattccactacttttggaagatgcaatcagatgtttggggcttt
gtatcgatccaaaagcacatatctcactacagccaaggtgattttagtgtcaactctatt
accatcaatggttggttgagaaacttattatggtccgaggcatcccaagtaatccaatcc
tatgctctttcatccatttgtccatatgggttcattttcctcatagctcatttcatctgg
gcatttagtttaatgttcctctttagtggtagagcatactggcaagaattaattgagtcc
attctatggtcacatcataaattaaagattatacctcatatccaaccaagggcattaagc
atttctcaaggaagagcagttggtttcattcattacactctaggaggtatcggctcaacc
tgggcatttattatttcaagactgctagtattaacttcttga
>KrC15_psaA
atgcacatcttcagatatatcaacacaacactgtggtccaaagcaggacattttaataaa
gctttatcaaaaggagctaagacaactacttggatatggaatctacatgattatgctcat
gattttgacattcaacaaagatccacaggtttaatagcaagaaaagtcttctcatccaat
ctagctcatctatctttggtattcttttggattagtggaatgcaccttcatggggcatat
ttgtctaattatgatatttggttaaaagatcccaaatccatcaccccatcatcacatcta

gcctactctttaattggtcaagatattcttaactcatatacctcagaatacttctcagga
atcactatcacatctggctttttccaactctaccgttctgagggtatcattacacagtcc
cagctgaaatatgcatgtgctacttccctaatagctacacttatctgcctttctggatct
tatctccacatgcagcttatgtccaaattcactagcttttacaagaagttccagtcctta
tcgcaagatcatctcataattatatttggctccagctccacatccctctccgctcatcag
atacacaaaatgcttccagctaaccctttactagattcaggtatctccaacccaagtatc
ctacaagtaatctccaacagtctcagtgaaaacctagcactgttttcaacaaatctaagc
tctacaggtaaattgttaaatccctcaacaagaagtgtattcctttctcaagttgcagca
catcataagacaacaggtgtagtctttatcacactagggcttatcagattcctcaccatg
tataactctcaatttagtatacttacatcttatatagattaccacattgtgctatctatt
aatttagcattaattgcatctttatcaataatagtagctgatcatctcacaagaacccca
atctatcctcacaactcaacctcataccctacaatcctttgcctctccatacatcatgca
tggttatccggtttcctcatcattggttcaggagctcatgcatcaatattcaacttgcga
gcatcaccaacatctgagataagacatcgagaccccatatactcacacctcatctgggta
tgtatagcaataggattacactcattcagtctctactgtcataacgacactttagaagca
ttaggacgtccagaagatatctttcatgacaactctatccagttaaaagcaatatttgcc
aagcaatcctttctaagagcagagctccagccagatatcgagatgttagataaaaagatt
atacgtataacccaagaattgggaacagcagattttatagttcaccacattcatgcattt
acaatccatgttactctcttaattctttcaaagggtgtactttatgctagaaactctaga
tttgtatcagataaattggaacttggttttacttatccctgtgatggtccaggtagaggt
ggtacatgtcaaatatcaccttgggatcacttattctcagctgtattttggatgtataac
tgtttaaatgtagtaacattccactacttttggaagatgcaatcagatgtttggggcttt
gtatcgatccaaaagcacatatctcactacagccaaggtgattttagtgtcaactctatt
accatcaatggttggttgagaaacttattatggtccgaggcatcccaagtaatccaatcc
tatgctctttcatccatttgtccatatgggttcattttcctcatagctcatttcatctgg
gcatttagtttaatgttcctctttagtggtagagcatactggcaagaattaattgagtcc
attctatggtcacatcataaattaaagattatacctcatatccaaccaagggcattaagc
atttctcaaggaagagcagttggtttcattcattacactctaggaggtatcggctcaacc
tgggcatttattatttcaagactgctagtattaacttcttga
>KrC2_psaA
atgcacatcttcagatatatcaacacaacactgtggtccaaagcaggacattttaataaa
gctttatcaaaaggagctaagacaactacttggatatggaatctacatgattatgctcat
gattttgacattcaacaaagatccacaggtttaatagcaagaaaagtcttctcatccaat
ctagctcatctatctttggtattcttttggattagtggaatgcaccttcatggggcatat
ttgtctaattatgatatttggttaaaagatcccaaatccatcaccccatcatcacatcta
gcctactctttaattggtcaagatattcttaactcatatacctcagaatacttctcagga
atcactatcacatctggctttttccaactctaccgttctgagggtatcattacacagtcc
cagctgaaatatgcatgtgctacttccctaatagctacacttatctgcctttctggatct
tatctccacatgcagcttatgtccaaattcactagcttttacaagaagttccagtcctta
tcgcaagatcatctcataattatatttggctccagctccacatccctctccgctcatcag
atacacaaaatgcttccagctaaccctttactagattcaggtatctccaacccaagtatc
ctacaagtaatctccaacagtctcagtgaaaacctagcactgttttcaacaaatctaagc
tctacaggtaaattgttaaatccctcaacaagaagtgtattcctttctcaagttgcagca
catcataagacaacaggtgtagtctttatcacactagggcttatcagattcctcaccatg
tataactctcaatttagtatacttacatcttatatagattaccacattgtgctatctatt
aatttagcattaattgcatctttatcaataatagtagctgatcatctcacaagaacccca
atctatcctcacaactcaacctcataccctacaatcctttgcctctccatacatcatgca
tggttatccggtttcctcatcattggttcaggagctcatgcatcaatattcaacttgcga
gcatcaccaacatctgagataagacatcgagaccccatatactcacacctcatctgggta
tgtatagcaataggattacactcattcagtctctactgtcataacgacactttagaagca
ttaggacgtccagaagatatctttcatgacaactctatccagttaaaagcaatatttgcc
aagcaatcctttctaagagcagagctccagccagatatcgagatgttagataaaaagatt
atacgtataacccaagaattgggaacagcagattttatagttcaccacattcatgcattt
acaatccatgttactctcttaattctttcaaagggtgtactttatgctagaaactctaga
tttgtatcagataaattggaacttggttttacttatccctgtgatggtccaggtagaggt
ggtacatgtcaaatatcaccttgggatcacttattctcagctgtattttggatgtataac
tgtttaaatgtagtaacattccactacttttggaagatgcaatcagatgtttggggcttt

gtatcgatccaaaagcacatatctcactacagccaaggtgattttagtgtcaactctatt
accatcaatggttggttgagaaacttattatggtccgaggcatcccaagtaatccaatcc
tatgctctttcatccatttgtccatatgggttcattttcctcatagctcatttcatctgg
gcatttagtttaatgttcctctttagtggtagagcatactggcaagaattaattgagtcc
attctatggtcacatcataaattaaagattatacctcatatccaaccaagggcattaagc
atttctcaaggaagagcagttggtttcattcattacactctaggaggtatcggctcaacc
tgggcatttattatttcaagactgctagtattaacttcttga
>KrC3_psaA
atgcacatcttcagatatatcaacacaacactgtggtccaaagcaggacattttaataaa
gctttatcaaaaggagctaagacaactacttggatatggaatctacatgattatgctcat
gattttgacattcaacaaagatccacaggtttaatagcaagaaaagtcttctcatccaat
ctagctcatctatctttggtattcttttggattagtggaatgcaccttcatggggcatat
ttgtctaattatgatatttggttaaaagatcccaaatccatcaccccatcatcacatcta
gcctactctttaattggtcaagatattcttaactcatatacctcagaatacttctcagga
atcactatcacatctggctttttccaactctaccgttctgagggtatcattacacagtcc
cagctgaaatatgcatgtgctacttccctaatagctacacttatctgcctttctggatct
tatctccacatgcagcttatgtccaaattcactagcttttacaagaagttccagtcctta
tcgcaagatcatctcataattatatttggctccagctccacatccctctccgctcatcag
atacacaaaatgcttccagctaacccttactagattcaggtatctccaacccaagtatc
ctacaagtaatctccaacagtctcagtgaaaacctagcactgttttcaacaaatctaagc
tctacaggtaaattgttaaatccctcaacaagaagtgtattcctttctcaagttgcagca
catcataagacaacaggtgtagtctttatcacactagggcttatcagattcctcaccatg
tataactctcaatttagtatacttacatcttatatagattaccacattgtgctatctatt
aatttagcattaattgcatctttatcaataatagtagctgatcatctcacaagaacccca
atctatcctcacaactcaacctcataccctacaatcctttgcctctccatacatcatgca
tggttatccggtttcctcatcattggttcaggagctcatgcatcaatattcaacttgcga
gcatcaccaacatctgagataagacatcgagaccccatatactcacacctcatctgggta
tgtatagcaataggattacactcattcagtctctactgtcataacgacactttagaagca
ttaggacgtccagaagatatctttcatgacaactctatccagttaaaagcaatatttgcc
aagcaatcctttctaagagcagagctccagccagatatcgagatgttagataaaaagatt
atacgtataacccaagaattgggaacagcagattttatagttcaccacattcatgcattt
acaatccatgttactctcttaattctttcaaagggtgtactttatgctagaaactctaga
tttgtatcagataaattggaacttggttttacttatccctgtgatggtccaggtagaggt
ggtacatgtcaaatatcaccttgggatcacttattctcagctgtattttggatgtataac
tgtttaaatgtagtaacattccactacttttggaagatgcaatcagatgtttggggcttt
gtatcgatccaaaagcacatatctcactacagccaaggtgattttagtgtcaactctatt
accatcaatggttggttgagaaacttattatggtccgaggcatcccaagtaatccaatcc
tatgctctttcatccatttgtccatatgggttcattttcctcatagctcatttcatctgg
gcatttagtttaatgttcctctttagtggtagagcatactggcaagaattaattgagtcc
attctatggtcacatcataaattaaagattatacctcatatccaaccaagggcattaagc
atttctcaaggaagagcagttggtttcattcattacactctaggaggtatcggctcaacc
tgggcatttattatttcaagactgctagtattaacttcttga
>KrC4_psaA
atgcacatcttcagatatatcaacacaacactgtggtccaaagcaggacattttaataaa
gctttatcaaaaggagctaagacaactacttggatatggaatctacatgattatgctcat
gattttgacattcaacaaagatccacaggtttaatagcaagaaaagtcttctcatccaat
ctagctcatctatctttggtattcttttggattagtggaatgcaccttcatggggcatat
ttgtctaattatgatatttggttaaaagatcccaaatccatcaccccatcatcacatcta
gcctactctttaattggtcaagatattcttaactcatatacctcagaatacttctcagta
atcactatcacatctggctttttccaactctaccgttctgagggtatcattacacagtcc
cagctcaaatatgcatgtgctacttccctaatagctacacttatctgcctttctggatct
tatctccacatgcagcttatgtccaaattcactagcttttacaagaagttccagtcctta
tcgcaagatcatctcataattatatttggctccagctccacatccctctccgctcatcag
atacacaaaatgcttccagctaacccttactagattcaggtatctccaacccaagtatc
ctacaagtaatctccaacagtctcagctatacactagcactgttttcaacaaatctaagc
tctacaggtaaattgttaaatccctcaacaagaagtgtattcctttctcaagttgcagca
catcataagacaacaggtgtagtctttatcacactagggcttatcagattcctcaccatg

tataactctcaatttagtatacttacatcttatatagattaccacattgtgctatctatt
aatttagcattaattgcatctttatcaataatagtagctgatcatctcacaagaacccca
atctatcctcacaactcaacctcataccctacaatcctttgcctctccatacatcatgca
tggttatccggtttcctcatcattggttcaggagctcatgcatcaatattcaacttgcga
gcatcaccaacatctgagataagacatcgagaccccatatactcacacctcatctgggta
tgtatagcaataggattacactcattcagtctctactgtcataacgacactttagaagca
ttaggacgtccagaagatatctttcatgacaactctatccagttaaaagcaatatttgcc
aagcaatcctttctaagagcagagctccagccagatatcgagatgttagataaaaagatt
atacgtataacccaagaattgggaacagcagattttatagttcaccacattcatgcattt
acaatccatgttactctcttaattctttcaaagggtgtactttatgctagaaactctaga
tttgtatcagataaattggaacttggttttacttatccctgtgatgggccaggtagaggt
ggtacatgtcaaatatcaccttgggatcacttattctcagctgtattttggatgtataac
tgtttaaatgtagtaacattccactacttttggaagatgcaatcagatgtttggggcttt
gtatcgatccaaaagcacatatctcactacagccaaggtgattttagtgtcaactctatt
accatcaatggttggttgagaaacttattatggtccgaggcatcccaagtaatccaatcc
tatgctctttcatccatttgtccatatgggttcattttcctcatagctcatttcatctgg
gcatttagtttaatgttcctctttagtggtagagcatactggcaagaattaattgagtcc
attctatggtcacatcataaattaaagattatacctcatatccaaccaagggcattaagc
atttctcaaggaagagcagttggtttcattcattacactctaggaggtatcggctcaacc
tgggcatttattatttcaagactgctagtattaacttcttga
>KrC5_psaA
atgcacatcttcagatatatcaacacaacactgtggtccaaagcaggacattttaataaa
gctttatcaaaaggagctaagacaactacttggatatggaatctacatgattatgctcat
gattttgacattcaacaaagatccacaggtttaatagcaagaaaagtcttctcatccaat
ctagctcatctatctttggtattcttttggattagtggaatgcaccttcatggggcatat
ttgtctaattatgatatttggttaaaagatcccaaatccatcaccccatcatcacatcta
gcctactctttaattggtcaagatattcttaactcatatacctcagaatacttctcagga
atcactatcacatctggctttttccaactctaccgttctgagggtatcattacacagtcc
cagctgaaatatgcatgtgctacttccctaatagctacacttatctgcctttctggatct
tatctccacatgcagcttatgtccaaattcactagcttttacaagaagttccagtcctta
tcgcaagatcatctcataattatatttggctccagctccacatccctctccgctcatcag
atacacaaaatgcttccagctaacccttttactagattcaggtatctccaacccaagtatc
ctacaagtaatctccaacagtctcagtgaaaacctagcactgttttcaacaaatctaagc
tctacaggtaaattgttaaatccctcaacaagaagtgtattcctttctcaagttgcagca
catcataagacaacaggtgtagtctttatcacactagggcttatcagattcctcaccatg
tataactctcaatttagtatacttacatcttatatagattaccacattgtgctatctatt
aatttagcattaattgcatctttatcaataatagtagctgatcatctcacaagaacccca
atctatcctcacaactcaacctcataccctacaatcctttgcctctccatacatcatgca
tggttatccggtttcctcatcattggttcaggagctcatgcatcaatattcaacttgcga
gcatcaccaacatctgagataagacatcgagaccccatatactcacacctcatctgggta
tgtatagcaataggattacactcattcagtctctactgtcataacgacactttagaagca
ttaggacgtccagaagatatctttcatgacaactctatccagttaaaagcaatatttgcc
aagcaatcctttctaagagcagagctccagccagatatcgagatgttagataaaaagatt
atacgtataacccaagaattgggaacagcagattttatagttcaccacattcatgcattt
acaatccatgttactctcttaattctttcaaagggtgtactttatgctagaaactctaga
tttgtatcagataaattggaacttggttttacttatccctgtgatggtccaggtagaggt
ggtacatgtcaaatatcaccttgggatcacttattctcagctgtattttggatgtataac
tgtttaaatgtagtaacattccactacttttggaagatgcaatcagatgtttggggcttt
gtatcgatccaaaagcacatatctcactacagccaaggtgattttagtgtcaactctatt
accatcaatggttggttgagaaacttattatggtccgaggcatcccaagtaatccaatcc
tatgctctttcatccatttgtccatatgggttcattttcctcatagctcatttcatctgg
gcatttagtttaatgttcctctttagtggtagagcatactggcaagaattaattgagtcc
attctatggtcacatcataaattaaagattatacctcatatccaaccaagggcattaagc
atttctcaaggaagagcagttggtttcattcattacactctaggaggtatcggctcaacc
tgggcatttattatttcaagactgctagtattaacttcttga
>KrC6_psaA
atgcacatcttcagatatatcaacacaacactgtggtccaaagcaggacattttaataaa

gctttatcaaaaggagctaagacaactacttggatatggaatctacatgattatgctcat
gattttgacattcaacaaagatccacaggtttaatagcaagaaaagtcttctcatccaat
ctagctcatctatctttggtattcttttggattagtggaatgcaccttcatggggcatat
ttgtctaattatgatatttggttaaaagatcccaaatccatcaccccatcatcacatcta
gcctactctttaattggtcaagatattcttaactcatatacctcagaatacttctcagga
atcactatcacatctggctttttccaactctaccgttctgagggtatcattacacagtcc
cagctgaaatatgcatgtgctacttccctaatagctacacttatctgcctttctggatct
tatctccacatgcagcttatgtccaaattcactagcttttacaagaagttccagtcctta
tcgcaagatcatctcataattatatttggctccagctccacatccctctccgctcatcag
atacacaaaatgcttccagctaaccctttactagattcaggtatctccaacccaagtatc
ctacaagtaatctccaacagtctcagtgaaaacctagcactgttttcaacaaatctaagc
tctacaggtaaattgttaaatccctcaacaagaagtgtattcctttctcaagttgcagca
catcataagacaacaggtgtagtctttatcacactagggcttatcagattcctcaccatg
tataactctcaatttagtatacttacatcttatatagattaccatattgtgctatctatt
aatttagcattaattgcatctttatcaataatagtagctgatcatctcacaagaacccca
atctatcctcacaactcaacctcataccctacaatcctttgcctctccatacatcatgca
tggttatccggtttcctcatcattggttcaggagctcatgcatcaatattcaacttgcga
gcatcaccaacatctgagataagacatcgagaccccatatactcacacctcatctgggta
tgtatagcaataggattacactcattcagtctctactgtcataacgacactttagaagca
ttaggacgtccagaagatatctttcatgacaactctatccagttaaaagcaatatttgcc
aagcaatcctttctaagagcagagctccagccagatatcgagatgttagataaaaagatt
atacgtataacccaagaattgggaacagcagattttatagttcaccacattcatgcattt
acaatccatgttactctcttaattctttcaaagggtgtactttatgctagaaactctaga
tttgtatcagataaattggaacttggttttacttatccctgtgatggtccaggtagaggt
ggtacatgtcaaatatcaccttgggatcacttattctcagctgtattttggatgtataac
tgtttaaatgtagtaacattccactacttttggaagatgcaatcagatgtttggggcttt
gtatcgatccaaaagcacatatctcactacagccaaggtgattttagtgtcaactctatt
accatcaatggttggttgagaaacttattatggtccgaggcatcccaagtaatccaatcc
tatgctctttcatccatttgtccatatgggttcattttcctcatagctcatttcatctgg
gcatttagtttaatgttcctctttagtggtagagcatactggcaagaattaattgagtcc
attctatggtcacatcataaattaaagattatacctcatatccaaccaagggcattaagc
atttctcaaggaagagcagttggtttcattcattacactctaggaggtatcggctcaacc
tgggcatttattatttcaagactgctagtattaacttcttga
>KrC7_psaA
atgcacatcttcagatatatcaacacaacactgtggtccaaagcaggacattttaataaa
gctttatcaaaaggagctaagacaactacttggatatggaatctacatgattatgctcat
gattttgacattcaacaaagatccacaggtttaatagcaagaaaagtcttctcatccaat
ctagctcatctatctttggtattcttttggattagtggaatgcaccttcatggggcatat
ttgtctaattatgatatttggttaaaagatcccaaatccatcaccccatcatcacatcta
gcctactctttaattggtcaagatattcttaactcatatacctcagaatacttctcagga
atcactatcacatctggctttttccaactctaccgttctgagggtatcattacacagtcc
cagctgaaatatgcatgtgctacttccctaatagctacacttatctgcctttctggatct
tatctccacatgcagcttatgtccaaattcactagcttttacaagaagttccagtcctta
tcgcaagatcatctcataattatatttggctccagctccacatccctctccgctcatcag
atacacaaaatgcttccagctaaccctttactagattcaggtatctccaacccaagtatc
ctacaagtaatctccaacagtctcagtgaaaacctagcactgttttcaacaaatctaagc
tctacaggtaaattgttaaatccctcaacaagaagtgtattcctttctcaagttgcagca
catcataagacaacaggtgtagtctttatcacactagggcttatcagattcctcaccatg
tataactctcaatttagtatacttacatcttatatagattaccacattgtgctatctatt
aatttagcattaattgcatctttatcaataatagtagctgatcatctcacaagaacccca
atctatcctcacaactcaacctcataccctacaatcctttgcctctccatacatcatgca
tggttatccggtttcctcatcattggttcaggagctcatgcatcaatattcaacttgcga
ggatcaccaacatctgagataagacatcgagaccccatatactcacacctcatctgggta
tgtatagcgataggattacactcattcagtctctactgtcataacgacactttagaagca
ttaggacgtccagaagatatctttcatgacaactctatccagttaaaagcaatatttgcc
aagcaatcctttctaagagcagagctccagccagatatcgagatgttagataaaaagatt
atacgtataacccaagaattgggaacagcagattttatagttcaccacattcatgcattt

acaatccatgttactctcttaattctttcaaagggtgtactttatgctagaaactctaga
tttgtatcagataaattggaacttggttttacttatccctgtgatggtccaggtagaggt
ggtacatgtcaaatatcaccttgggatcacttattctcagctgtattttggatgtataac
tgtttaaatgtagtaacattccactacttttggaagatgcaatcagatgtttggggcttt
gtatcgatccaaaagcacatatctcactacagccaaggtgattttagtgtcaactctatt
accatcaatggttggttgagaaacttattatggtccgaggcatcccaagtaatccaatcc
tatgctctttcatccatttgtccatatgggttcattttcctcatagctcatttcatctgg
gcatttagtttaatgttcctctttagtggtagagcatactggcaagaattaattgagtcc
attctatggtcacatcataaattaaagattatacctcatatccaaccaagggcattaagc
atttctcaaggaagagcagttggtttcattcattacactctaggaggtatcggctcaacc
tgggcatttattatttcaagactgctagtattaacttcttga
>KrC8_psaA
atgcacatcttcagatatatcaacacaacactgtggtccaaagcaggacattttaataaa
gctttatcaaaaggagctaagacaactacttggatatggaatctacatgattatgctcat
gattttgacattcaacaaagatccacaggtttaatagcaagaaaagtcttctcatccaat
ctagctcatctatctttggtattcttttggattagtggaatgcaccttcatggagcatat
ttgtctaattatgatatttggttaaaagatcccaaatccatcaccccatcatcacatcta
gcctactctttaattggtcaagatattcttaactcatatacctcagaatacttctcagga
atcactatcacatctggctttttccaactctaccgttctgagggtatcattacacagtcc
cagctcaaatatgcatgtgctacttccctaatagctacacttatctgcctttctggatct
tatctccacatgcagcttatgtccaaattcactagcttttacaagaagttccagtcctta
tcgcaagatcatctcataattatatttggctccagctccacatccctctccgctcatcag
atacacaaaatgcttccagctaaccctttactagattcaggtatctccaacccaagtatc
ctacaagtaatctccaacagtctcagtgaaaacctagcactgttttcaacaaatctaagc
tctacaggtaaattgttaaatccctcaacaagaagtgtattcctttctcaagttgcagca
catcataagacaacaggtgtagtctttatcacactagggcttatcagattcctcaccatg
tataactctcaatttagtatacttacatcttatatagattaccacattgtgctatctatt
aatttagcattaattgcatctttatcaataatagtagctgatcatctcacaagaacccca
atctatcctcacaactcaacctcataccctacaatcctttgcctctccatacatcatgca
tggttatccggtttcctcatcattggttcaggagctcatgcatcaatattcaacttgcga
gcatcaccaacatctgagataagacatcgagaccccatatactcacacctcatctgggta
tgtatagcaataggattacactcattcagtctctactgtcataacgacactttagaagca
ttaggacgtccagaagatatctttcatgacaactctatccagttaaaagcaatatttgcc
aagcaatcctttctaagagcagagctccagccagatatcgagatgttagataaaaagatt
atacgtataacccaagaattgggaacagcagattttatagttcaccacattcatgcattt
acaatccatgttactctcttaattctttcaaagggtgtactttatgctagaaactctaga
tttgtatcagataaattggaacttggttttacttatccctgtgatggtccaggtagaggt
ggtacatgtcaaatatcaccttgggatcacttattctcagctgtattttggatgtataac
tgtttaaatgtagtaacattccactacttttggaagatgcaatcagatgtttggggcttt
gtatcgatccaaaagcacatatctcactacagccaaggtgattttagtgtcaactctatt
accatcaatggttggttgagaaacttattatggtccgaggcatcccaagtaatccaatcc
tatgctctttcatccatttgtccatatgggttcattttcctcatagctcatttcatctgg
gcatttagtttaatgttcctctttagtggtagagcatactggcaagaattaattgagtcc
attctatggtcacatcataaattaaagattatacctcatatccaaccaagggcattaagc
atttctcaaggaagagcagttggtttcattcattacactctaggaggtatcggctcaacc
tgggcatttattatttcaagactgctagtattaacttcttga
>KrC9_psaA
atgcacatcttcagatatatcaacacaacactgtggtccaaagcaggacattttaataaa
gctttatcaaaaggagctaagacaactacttggatatggaatctacatgattatgctcat
gattttgacattcaacaaagatccacaggtttaatagcaagaaaagtcttctcatccaat
ctagctcatctatctttggtattcttttggattagtggaatgcaccttcatggggcatat
ttgtctaattatgatatttggttaaaagatcccaaatccatcaccccatcatcacatcta
gcctactctttaattggtcaagatattcttaactcatatacctcagaatacttctcagga
atcactatcacatctggctttttccaactctaccgttctgagggtatcattacacagtcc
cagctgaaatatgcatgtgctacttccctaatagctacacttatctgcctttctggatct
tatctccacatgcagcttatgtccaaattcactagcttttacaagaagttccagtcctta
tcgcaagatcatctcataattatatttggctccagctccacatccctctccgctcatcag

atacacaaaatgcttccagctaaccctttactagattcaggtatctccaacccaagtatc
ctacaagtaatctccaacagtctcagttaaaacctagcactgttttcaacaaatctaagc
tctacaggtaaattgttaaatccctcaacaagaagtgtattcctttctcaagttgcagca
catcataagacaacaggtgtagtctttatcacactagggcttatcagattcctcaccatg
tataactctcaatttagtatacttacatcttatatagattaccacattgtgctatctatt
aatttagcattaattgcatctttatcaataatagtagctgatcatctcacaagaacccca
atctatcctcacaactcaacctcataccctacaatcctttgcctctccatacatcatgca
tggttatccggtttcctcatcattggttcaggagctcatgcatcaatattcaacttgcga
gcatcaccaacatctgagataagacatcgagaccccatatactcacacctcatctgggta
tgtatagcaataggattacactcattcagtctctactgtcataacgacactttagaagca
ttaggacgtccagaagatatctttcatgacaactctatccagttaaaagcaatatttgcc
aagcaatcctttctaagagcagagctccagccagatatcgagatgttagataaaaagatt
atacgtataacccaagaattgggaacagcagattttatagttcaccacattcatgcattt
acaatccatgttactctcttaattctttcaaagggtgtactttatgctagaaactctaga
tttgtatcagataaattggaacttggttttacttatccctgtgatggtccaggtagaggt
ggtacatgtcaaatatcaccttgggatcacttattctcagctgtattttggatgtataac
tgtttaaatgtagtaacattccactacttttggaagatgcaatcagatgtttggggcttt
gtatcgatccaaaagcacatatctcactacagccaaggtgattttagtgtcaactctatt
accatcaatggttggttgagaaacttattatggtccgaggcatcccaagtaatccaatcc
tatgctctttcatccatttgtccatatgggttcattttcctcatagctcatttcatctgg
gcatttagtttaatgttcctctttagtggtagagcatactggcaagaattaattgagtcc
attctatggtcacatcataaattaaagattatacctcatatccaaccaagggcattaagc
atttctcaaggaagagcagttggtttcattcattacactctaggaggtatcggctcaacc
tgggcatttattatttcaagactgctagtattaacttcttga
>KrD1_psaA
atgaacatcttcagatatatcaacacaacactgtggtccaaagcaggacattttaataaa
gctttatcaaaaggagctaagacaactacttggatatggaatctacatgattatgctcat
gattttgacattcaacaaagatccacaggtttaatagcaagaaaagtcttctcatccaat
ctagctcatctatctttggtattcttttggattagtggaatgcaccttcatggggcatat
ttgtctaattatgatatttggttaaaagatcccaaatccatcaccccatcatcacatcta
gcctactctttaattggtcaagatattcttaactcatatacctcagaatacttctcagga
atcactatcacatctggctttttccaactctaccgttctgagggtatcattacacagtcc
cagctgaaatatgcatgtgctacttccctaatagctacacttatctgcctttctggatct
tatctccacatgcagcttatgtccaaattcactagcttttacaagaagttccagtcctta
tcgcaagatcatctcataattatatttggctccagctccacatccctctccgctcatcag
atacacaaaatgcttccagctaaccctttactagattcaggtatctccaacccaagtatc
ctacaagtaatctccaacagtctcagtgaaaacctagcactgttttcaacaaatctaagc
tctacaggtaaattgttaaatccctcaacaagaagtgtattcctttctcaagttgcagca
catcataagacaacaggtgtagtctttatcacactagggcttatcagattcctcaccatg
tataactctcaatttagtatacttacatcttatatagattaccacattgtgctatctatt
aatttagcattaattgcatctttatcaataatagtagctgatcatctcacaagaacccca
atctatcctcacaactcaacctcataccctacaatcctttgcctctccatacatcatgca
tggttatccggtttcctcatcattggttcaggagctcatgcatcaatattcaacttgcga
gcatcaccaacatctgagataagacatcgagaccccatatactcacacctcatctgggta
tgtatagcaataggattacactcattcagtctctactgtcataacgacactttagaagca
ttaggacgtccagaagatatctttcatgacaactctatccagttaaaagcaatatttgcc
aagcaatcctttctaagagcagagctccagccagatatcgagatgttagataaaaagatt
atacgtataacccaagaattgggaacagcagattttatagttcaccacattcatgcattt
acaatccatgttactctcttaattctttcaaagggtgtactttatgctagaaactctaga
tttgtatcagataaattggaacttggttttacttatccctgtgatggtccaggtagaggt
ggtacatgtcaaatatcaccttgggatcacttattctcagctgtattttggatgtataac
tgtttaaatgtagtaacattccactacttttggaagatgcaatcagatgtttggggcttt
gtatcgatccaaaagcacatatctcactacagccaaggtgattttagtgtcaactctatt
accatcaatggttggttgagaaacttattatggtccgaggcatcccaagtaatccaatcc
tatgctctttcatccatttgtccatatgggttcattttcctcatagctcatttcatctgg
gcatttagtttaatgttcctctttagtggtagagcatactggcaagaattaattgagtcc
attctatggtcacatcataaattaaagattatacctcatatccaaccaagggcattaagc

atttctcaaggaagagcagttggtttcattcattacactctaggaggtatcggctcaacc
tgggcatttattatttcaagactgctagtattaacttcttga
>KrD11_psaA
atgcacatcttcagatatatcaacacaacactgtggtccaaagcaggacattttaataaa
gctttatcaaaaggagctaagacaactacttggatatggaatctacatgattatgctcat
gattttgacattcaacaaagatccacaggtttaatagcaagaaaagtcttctcatccaat
ctagctcatctatctttggtattcttttggattagtggaatgcaccttcatggggcatat
ttgtctaattatgatatttggttaaaagatcccaaatccatcaccccatcatcacatcta
gcctactctttaattggtcaagatattcttaactcatatacctcagaatacttctcagga
atcactatcacatctggctttttccaactctaccgttctgagggtatcattacacagtcc
cagctgaaatatgcatgtgctacttccctaatagctacacttatctgcctttctggatct
tatctccacatgcagcttatgtccaaattcactagcttttacaagaagttccagtcctta
tcgcaagatcatctcataattatatttggctccaggtccacatccctctccgctcatcag
atacacaaaatgcttccagctaaccctttactagattcaggtatctccaagccaagtatc
ctacaagtaatctccaacagtctcagctatacactagcactgttttcaacaaatctaagc
tctacaggtaaattgttaaatccctcaacaagaagtgtattcctttctcaagttgcagca
catcataagacaacaggtgtagtctttatcacactagggcttatcagattcctcaccatg
tataactctcaatttagtatacttacatcttatatagattaccacattgtgctatctatt
aatttagcattaattgcatctttatcaataatagtagctgatcatctcacaagaacccca
atctatcctcacaactcaacctcataccctacaatcctttgcctctccatacatcatgca
tggttatccggtttcctcatcattggttcaggagctcatgcatcaatattcaacttgctt
ggatcaccaacatctgagataagacatcgagaccccatatactcacacctcatctgggta
tgtatagcaataggattacactcattcagtctctactgtcataacgacactttagaagca
ttaggacgtccagaagatatctttcatgacaactctatccagttaaaagcaatatttgcc
aagcaatcctttctaagagcagagctccagccagatatcgagatgttagataaaaagatt
atacgtataacccaagaattgggaacagcagattttatagttcaccacattcatgcattt
acaatccatgttactctcttaattctttcaaagggtgtactttatgctagaaactctaga
tttgtatcagataaattggaacttggttttacttatccctgtgatggtccaggtagaggt
ggtacatgtcaaatatcaccttgggatcacttattctcagctgtattttggatgtataac
tgtttaaatgtagtaacattccactacttttggaagatgcaatcagatgtttggggcttt
gtatcgatccaaaagcacatatctcactacagccaaggtgattttagtgtcaactctatt
accatcaatggttggttgagaaacttattatggtccgaggcatcccaagtaatccaatcc
tatgctctttcatccatttgtccatatgggttcattttcctcatagctcatttcatctgg
gcatttagtttaatgttcctctttagtggtagagcatactggcaagaattaattgagtcc
attctatggtcacatcataaattaaagattatacctcatatccaaccaagggcattaagc
atttctcaaggaagagcagttggtttcattcattacactctaggaggtatcggctcaacc
tgggcatttattatttcaagactgctagtattaacttcttga
>KrD13_psaA
atgcacatcttcagatatatcaacacaacactgtggtccaaagcaggacattttaataaa
gctttatcaaaaggagctaagacaactacttggatatggaatctacatgattatgctcat
gattttgacattcaacaaagatccacaggtttaatagcaagaaaagtcttctcatccaat
ctagctcatctatctttggtattcttttggattagtggaatgcaccttcatggggcatat
ttgtctaattatgatatttggttaaaagatcccaaatccatcacgccatcatcacatcta
gcctactctttaattggtcaagatattcttaactcatatacctcagaatacttctcagga
atcactatcacatctggctttttccaactctaccgttctgagggtatcattacacagtcc
cagctgaaatatgcatgtgctacttccctaatagctacacttatctgcctttctggatct
tatctccacatgcagcttatgtccaaattcactagcttttacaagaagttccagtcctta
tcgcaagatcatctcataattatatttggctccagctccacatccctctccgctcatcag
atacacaaaatgcttccagctaaccctttactagattcaggtatctccaacccaagtatc
ctacaagtaatctccaacagtctcagtgataaactagcactgttttcaacaaatctaagc
tctacaggtaaattgttaaatccctcaacaagaagtgtattcctttctcaagttgcagca
catcataagacaacaggtgtagtctttatcacactagggcttatcagattcctcaccatg
tataactctcaatttagtatacttacatcttatatagattaccacattgtgctatctatt
aatttagcattaattgcatctttatcaataatagtagctgatcatctcacaagaacccca
atctatcctcacaactcaacctcataccctacaatcctttgcctctccatacatcatgca
tggttatccggtttcctcatcattggttcaggagctcatgcatcaatattcaacttgcga
gcatcaccaacatctgagataagacatcgagaccccatatactcacacctcatctgggta

tgtatagcaataggattacactcattcagtctctactgtcataacgacactttagaagca
ttaggacgtccagaagatatctttcatgacaactctatccagttaaaagcaatatttgcc
aagcaatcctttctaagagcagagctccagccagatatcgagatgttagataaaaagatt
atacgtataacccaagaattgggaacagcagattttatagttcaccacattcatgcattt
acaatccatgttactctcttaattctttcaaagggtgtactttatgctagaaactctaga
tttgtatcagataaattggaacttggttttacttatccctgtgatggtccaggtagaggt
ggtacatgtcaaatatcaccttgggatcacttattctcagctgtattttggatgtataac
tgtttaaatgtagtaacattccactacttttggaagatgcaatcagatgtttggggcttt
gtatcgatccaaaagcacatatctcactacagccaaggtgattttagtgtcaactctatt
accatcaatggttggttgagaaacttattatggtccgaggcatcccaagtaatccaatcc
tatgctctttcatccatttgtccatatgggttcattttcctcatagctcatttcatctgg
gcatttagtttaatgttcctctttagtggtagagcatactggcaagaattaattgagtcc
attctatggtcacatcataaattaaagattatacctcatatccaaccaagggcattaagc
atttctcaaggaagagcagttggtttcattcattacactctaggaggtatcggctcaacc
tgggcatttattatttcaagactgctagtattaacttcttga
>KrD3_psaA
atgcacatcttcagatatatcaacacaacactgtggtccaaagcaggacattttaataaa
gctttatcaaaaggagctaagacaactacttggatatggaatctacatgattatgctcat
gattttgacattcaacaaagatccacaggtttaatagcaagaaaagtcttctcatccaat
ctagctcatctatctttggtattcttttggattagtggaatgcaccttcatggagcatat
ttgtctaattatgatatttggttaaaagatcccaaatccatcaccccatcatcacatcta
gcctactctttaattggtcaagatattcttaactcatatacctcagaatacttctcagga
atcactatcacatctggctttttccaactctaccgttctgagggtatcattacacagtcc
cagctgaaatatgcatgtgctacttccctaatagctacacttatctgcctttctggatct
tatctccacatgcagcttatgtccaaattcactagcttttacaagaagttccagtcctta
tcgcaagatcatctcataattatatttggctccagctccacatccctctccgctcatcag
atacacaaaatgcttccagctaaccctttactagattcaggtatctccaacccaagtatc
ctacaagtaatctccaacagtctcagtgaaaacctagcactgttttcaacaaatctaagc
tctacaggtaaattgttaaatccctcaacaagaagtgtattcctttctcaagttgcagca
catcataagacaacaggtgtagtctttatcacactagggcttatcagattcctcaccatg
tataactctcaatttagtatacttacatcttatatagattaccacattgtgctatctatt
aatttagcattaattgcatctttatcaataatagtagctgatcatctcacaagaacccca
atctatcctcacaactcaacctcataccctacaatcctttgcctctccatacatcatgca
tggttatccggtttcctcatcattggttcaggagctcatgcatcaatattcaacttgcta
ggatcaccaacatctgagataagacatcgagaccccatatactcacacctcatctgggta
tgtatagcaataggattacactcattcagtctctactgtcataacgacactttagaagca
ttaggacgtccagaagatatctttcatgacaactctatccagttaaaagcaatatttgcc
aagcaatcctttctaagagcagagctccagccagatatcgagatgttagataaaaagatt
atacgtataacccaagaattgggaacagcagattttatagttcaccacattcatgcattt
acaatccatgttactctcttaattctttcaaagggtgtactttatgctagaaactctaga
tttgtatcagataaattggaacttggttttacttatccctgtgatggtccaggtagaggt
ggtacatgtcaaatatcaccttgggatcacttattctcagctgtattttggatgtataac
tgtttaaatgtagtaacattccactacttttggaagatgcaatcagatgtttggggcttt
gtatcgatccaaaagcacatatctcactacagccaaggtgattttagtgtcaactctatt
accatcaatggttggttgagaaacttattatggtccgaggcatcccaagtaatccaatcc
tatgctctttcatccatttgtccatatgggttcattttcctcatagctcatttcatctgg
gcatttagtttaatgttcctctttagtggtagagcatactggcaagaattaattgagtcc
attctatggtcacatcataaattaaagattatacctcatatccaaccaagggcattaagc
atttctcaaggaagagcagttggtttcattcattacactctaggaggtatcggctcaacc
tgggcatttattatttcaagactgctagtattaacttcttga
>KrD8_psaA
atgcacatcttcagatatatcaacacaacactgtgggccaaagcaggacattttaataaa
gctttatcaaaaggagctaagacaactacttggatatggaatctacatgattatgctcat
gattttgacattcaacaaagatccacaggtttaatagcaagaaaagtcttctcatccaat
ctagctcatctatctttggtattcttttggattagtggaatgcaccttcatggagcatat
ttgtctaattatgatatttggttaaaagatcccaaatccatcacgccatcatcacatcta
gcctactctttaattggtcaagatattcttaactcatatacctcagaatacttctcagga

atcactatcacatctggctttttccaactctaccgttctgagggtatcattacacagtcc
cagctgaaatatgcatgtgctacttccctaatagctacacttatctgccttctggatct
tatctccacatgcagcttatgtccaaattcactagcttttacaagaagttccagtcctta
tcgcaagatcatctcataattatatttggctccagctccacatccctctccgctcatcag
atacacaaaatgcttccagctaaccctttactagattcaggtatctccaagccaagtatc
ctacaagtaatctccaacagtctcagctatacactagcactgttttcaacaaatctaagc
tctacaggtaaattgttaaatccctcaacaagaagtgtattcctttctcaagttgcagca
catcataagacaacaggtgtagtctttatcacactagggcttatcagattcctcaccatg
tataactctcaatttagtatacttacatcttatatagattaccacattgtgctatctatt
aatttagcattaattgcatctttatcaataatagtagctgatcatctcacaagaacccca
atctatcctcacaagtcaacctcataccctacaatcctttgcctctccatacatcatgca
tggttatccggtttcctcatcattggttcaggagctcatgcatcaatattcaacttgcga
gcatcaccaacatctgagataagacatcgagaccccatatactcacacctcatctgggta
tgtatagcaataggattacactcattcagtctctactgtcataacgacactttagaagca
ttaggacgtccagaagatatctttcatgacaactctatccagttaaaagcaatatttgcc
aagcaatcctttctaagagcagagctccagccagatatcgagatgttagataaaaagatt
atacgtataacccaagaattgggaacagcagattttatagttcaccacattcatgcattt
acaatccatgttactctcttaattctttcaaagggtgtactttatgctagaaactctaga
tttgtatcagataaattggaacttggttttacttatccctgtgatggtccaggtagaggt
ggtacatgtcaaatatcaccttgggatcacttattctcagctgtattttggatgtataac
tgtttaaatgtagtaacattccactacttttggaagatgcaatcagatgtttggggcttt
gtatcgatccaaaagcacatatctcactacagccaaggtgattttagtgtcaactctatt
accatcaatggttggttgagaaacttattatggtccgaggcatcccaagtaatccaatcc
tatgctctttcatccatttgtccatatgggttcattttcctcatagctcatttcatctgg
gcatttagtttaatgttcctctttagtggtagagcatactggcaagaattaattgagtcc
attctatggtcacatcataaattaaagattatacctcatatccaaccaagggcattaagc
atttctcaaggaagagcagttggtttcattcattacactctaggaggtatcggctcaacc
tgggcatttattatttcaagactgctagtattaacttcttga
>KrD9_psaA
atgcacatcttcagatatatcaacacaacactgtgggccaaagcaggacattttaataaa
gctttatcaaaaggagctaagacaactacttggatatggaatctacatgattatgctcat
gattttgacattcaacaaagatccacaggtttaatagcaagaaaagtcttctcatccaat
ctagctcatctatctttggtattcttttggattagtggaatgcaccttcatggagcatat
ttgtctaattatgatatttggttaaaagatcccaaatccatcacgccatcatcacatcta
gcctactctttaattggtcaagatattcttaactcatatacctcagaatacttctcagga
atcactatcacatctggctttttccaactctaccgttctgagggtatcattacacagtcc
cagctcaaatatgcatgtgctacttccctaatagctacacttatctgccttctggatct
tatctccacatgcagcttatgtccaaattcactagcttttacaagaagttccagtcctta
tcgcaagatcatctcataattatatttggctccagctccacatccctctccgctcatcag
atacacaaaatgcttccagctaaccctttactagattcaggtatctccaacccaagtatc
ctacaagtaatctccaacagtctcagtgataccctagcactgttttcaacaaatctaagc
tctacaggtaaattgttaaatccctcaacaagaagtgtattcctttctcaagttgcagca
catcataagacaacaggtgtagtctttatcacactagggcttatcagattcctcaccatg
tataactctcaatttagtatacttacatcttatatagattaccacattgtgctatctatt
aatttagcattaattgcatctttatcaataatagtagctgatcatctcacaagaacccca
atctatcctcacaagtcaacctcataccctacaatcctttgcctctccatacatcatgca
tggttatccggtttcctcatcattggttcaggagctcatgcatcaatattcaacttgctt
ggatcaccaacatctgagataagacatcgagaccccatatactcacacctcatctgggta
tgtatagcaataggattacactcattcagtctctactgtcataacgacactttagaagca
ttaggacgtccagaagatatctttcatgacaactctatccagttaaaagcaatatttgcc
aagcaatcctttctaagagcagagctccagccagatatcgagatgttagataaaaagatt
atacgtataacccaagaattgggaacagcagattttatagttcaccacattcatgcattt
acaatccatgttactctcttaattctttcaaagggtgtactttatgctagaaactctaga
tttgtatcagataaattggaacttggttttacttatccctgtgatggtccaggtagaggt
ggtacatgtcaaatatcaccttgggatcacttattctcagctgtattttggatgtataac
tgtttaaatgtagtaacattccactacttttggaagatgcaatcagatgtttggggcttt
gtatcgatccaaaagcacatatctcactacagccaaggtgattttagtgtcaactctatt

accatcaatggttggttgagaaacttattatggtccgaggcatcccaagtaatccaatcc
tatgctctttcatccatttgtccatatgggttcattttcctcatagctcatttcatctgg
gcatttagtttaatgttcctctttagtggtagagcatactggcaagaattaattgagtcc
attctatggtcacatcataaattaaagattatacctcatatccaaccaagggcattaagc
atttctcaaggaagagcagttggtttcattcattacactctaggaggtatcggctcaacc
tgggcatttattatttcaagactgctagtattaacttcttga
>KrE1_psaA
atgaacatcttcagatatatcaacacaacactgtggtccaaagcaggacattttaataaa
gctttatcaaaaggagctaagacaactacttggatatggaatctacatgattatgctcat
gattttgacattcaacaaagatccacaggtttaatagcaagaaaagtcttctcatccaat
ctagctcatctatctttggtattcttttggattagtggaatgcaccttcatggggcatat
ttgtctaattatgatatttggttaaaagatcccaaatccatcaccccatcatcacatcta
gcctactctttaattggtcaagatattcttaactcatatacctcagaatacttctcagga
atcactatcacatctggctttttccaactctaccgttctgagggtatcattacacagtcc
cagctgaaatatgcatgtgctacttccctaatagctacacttatctgcctttctggatct
tatctccacatgcagcttatgtccaaattcactagcttttacaagaagttccagtcctta
tcgcaagatcatctcataattatatttggctccagctccacatccctctccgctcatcag
atacacaaaatgcttccagctaaccctttactagattcaggtatctccaacccaagtatc
ctacaagtaatctccaacagtctcagtgaaaacctagcactgttttcaacaaatctaagc
tctacaggtaaattgttaaatccctcaacaagaagtgtattcctttctcaagttgcagca
catcataagacaacaggtgtagtctttatcacactagggcttatcagattcctcaccatg
tataactctcaatttagtatacttacatcttatatagattaccacattgtgctatctatt
aatttagcattaattgcatctttatcaataatagtagctgatcatctcacaagaacccca
atctatcctcacaactcaacctcataccctacaatcctttgcctctccatacatcatgca
tggttatccggtttcctcatcattggttcaggagctcatgcatcaatattcaacttgcga
gcatcaccaacatctgagataagacatcgagaccccatatactcacacctcatctgggta
tgtatagcaataggattacactcattcagtctctactgtcataacgacactttagaagca
ttaggacgtccagaagatatctttcatgacaactctatccagttaaaagcaatatttgcc
aagcaatcctttctaagagcagagctccagccagatatcgagatgttagataaaaagatt
atacgtataacccaagaattgggaacagcagattttatagttcaccacattcatgcattt
acaatccatgttactctcttaattctttcaaagggtgtactttatgctagaaactctaga
tttgtatcagataaattggaacttggttttacttatccctgtgatggtccaggtagaggt
ggtacatgtcaaatatcaccttgggatcacttattctcagctgtattttggatgtataac
tgtttaaatgtagtaacattccactacttttggaagatgcaatcagatgtttggggcttt
gtatcgatccaaaagcacatatctcactacagccaaggtgattttagtgtcaactctatt
accatcaatggttggttgagaaacttattatggtccgaggcatcccaagtaatccaatcc
tatgctctttcatccatttgtccatatgggttcattttcctcatagctcatttcatctgg
gcatttagtttaatgttcctctttagtggtagagcatactggcaagaattaattgagtcc
attctatggtcacatcataaattaaagattatacctcatatccaaccaagggcattaagc
atttctcaaggaagagcagttggtttcattcattacactctaggaggtatcggctcaacc
tgggcatttattatttcaagactgctagtattaacttcttga
>KrE10_psaA
atgcacatcttcagatatatcaacacaacactgtgggccaaagcaggacattttaataaa
gctttatcaaaaggagctaagacaactacttggatatggaatctacatgattatgctcat
gattttgacattcaacaaagatccacaggtttaatagcaagaaaagtcttctcatccaat
ctagctcatctatctttggtattcttttggattagtggaatgcaccttcatggggcatat
ttgtctaattatgatatttggttaaaagatcccaaatccatcaccccatcatcacatcta
gcctactctttaattggtcaagatattcttaactcatatacctcagaatacttctcagga
atcactatcacatctggctttttccaactctaccgttctgagggtatcattacacagtcc
cagctgaaatatgcatgtgctacttccctaatagctacacttatctgcctttctggatct
tatctccacatgcagcttatgtccaaattcactagcttttacaagaagttccagtcctta
tcgcaagatcatctcataattatatttggctccagctccacatccctctccgctcatcag
atacacaaaatgcttccagctaaccctttactagattcaggtatctccaacccaagtatc
ctacaagtaatctccaacagtctcagtgaaaacctagcactgttttcaacaaatctaagc
tctacaggtaaattgttaaatccctcaacaagaagtgtattcctttctcaagttgcagca
catcataagacaacaggtgtagtctttatcacactagggcttatcagattcctcaccatg
tataactctcaatttagtatacttacatcttatatagattaccacattgtgctatctatt

aatttagcattaattgcatctttatcaataatagtagctgatcatctcacaagaacccca
atctatcctcacaactcaacctcataccctacaatcctttgcctctccatacatcatgca
tggttatccggtttcctcatcattggttcaggagctcatgcatcaatattcaacttgcga
gcatcaccaacatctgagataagacatcgagaccccatatactcacacctcatctgggta
tgtatagcaataggattacactcattcagtctctactgtcataacgacactttagaagca
ttaggacgtccagaagatatctttcatgacaactctatccagttaaaagcaatatttgcc
aagcaatcctttctaagagcagagctccagcaagatatcgagatgttagataaaaagatt
atacgtataacccaagaattgggaacagcagattttatagttcaccacattcatgcattt
acaatccatgttactctcttaattctttcaaagggtgtactttatgctagaaactctaga
tttgtatcagataaattggaacttggttttacttatccctgtgatggtccaggtagaggt
ggtacatgtcaaatatcaccttgggatcacttattctcagctgtattttggatgtataac
tgtttaaatgtagtaacattccactacttttggaagatgcaatcagatgtttggggcttt
gtatcgatccaaaagcacatatctcactacagccaaggtgattttagtgtcaactctatt
accatcaatggttggttgagaaacttattatggtccgaggcatcccaagtaatccaatcc
tatgctctttcatccatttgtccatatgggttcattttcctcatagctcatttcatctgg
gcatttagtttaatgttcctctttagtggtagagcatactggcaagaattaattgagtcc
attctatggtcacatcataaattaaagattatacctcatatccaaccaagggcattaagc
atttctcaaggaagagcagttggtttcattcattacactctaggaggtatcggctcaacc
tgggcatttattatttcaagactgctagtattaacttcttga
>KrE11_psaA
atgcacatcttcagatatatcaacacaacactgtgggccaaagcaggacattttaataaa
gctttatcaaaaggagctaagacaactacttggatatggaatctacatgattatgctcat
gattttgacattcaacaaagatccacaggtttaatagcaagaaaagtcttctcatccaat
ctagctcatctatctttggtattcttttggattagtggaatgcaccttcatggggcatat
ttgtctaattatgatatttggttaaaagatcccaaatccatcacgccatcatcacatcta
gcctactctttaattggtcaagatattcttaactcatatacctcagaatacttctcagga
atcactatcacatctggctttttccaactctaccgttctgagggtatcattacacagtcc
cagctgaaatatgcatgtgctacttccctaatagctacacttatctgcctttctggatct
tatctccacatgcagcttatgtccaaattcactagcttttacaagaagttccagtcctta
tcgcaagatcatctcataattatatttggctccagctccacatccctctccgctcatcag
atacacaaaatgcttccagctaaccctttactagattcaggtatctccaacccaagtatc
ctacaagtaatctccaacagtctcagtgaaaacctagcactgttttcaacaaatctaagc
tctacaggtaaattgttaaatccctcaacaagaagtgtattcctttctcaagttgcagca
catcataagacaacaggtgtagtctttatcacactagggcttatcagattcctcaccatg
tataactctcaatttagtatacttacatcttatatagattaccacattgtgctatctatt
aatttagcattaattgcatctttatcaataatagtagctgatcatctcacaagaacccca
atctatcctcacaactcaacctcataccctacaatcctttgcctctccatacatcatgca
tggttatccggtttcctcatcattggttcaggagctcatgcatcaatattcaacttgcga
gcatcaccaacatctgagataagacatcgagaccccatatactcacacctcatctgggta
tgtatagcaataggattacactcattcagtctctactgtcataacgacactttagaagca
ttaggacgtccagaagatatctttcatgacaactctatccagttaaaagcaatatttgcc
aagcaatcctttctaagagcagagctccagccagatatcgagatgttagataaaaagatt
atacgtataacccaagaattgggaacagcagattttatagttcaccacattcatgcattt
acaatccatgttactctcttaattctttcaaagggtgtactttatgctagaaactctaga
tttgtatcagataaattggaacttggttttacttatccctgtgatggtccaggtagaggt
ggtacatgtcaaatatcaccttgggatcacttattctcagctgtattttggatgtataac
tgtttaaatgtagtaacattccactacttttggaagatgcaatcagatgtttggggcttt
gtatcgatccaaaagcacatatctcactacagccaaggtgattttagtgtcaactctatt
accatcaatggttggttgagaaacttattatggtccgaggcatcccaagtaatccaatcc
tatgctctttcatccatttgtccatatgggttcattttcctcatagctcatttcatctgg
gcatttagtttaatgttcctctttagtggtagagcatactggcaagaattaattgagtcc
attctatggtcacatcataaattaaagattatacctcatatccaaccaagggcattaagc
atttctcaaggaagagcagttggtttcattcattacactctaggaggtatcggctcaacc
tgggcatttattatttcaagactgctagtattaacttcttga
>KrE12_psaA
atgcacatcttcagatatatcaacacaacactgtggtccaaagcaggacattttaataaa
gctttatcaaaaggagctaagacaactacttggatatggaatctacatgattatgctcat

gattttgacattcaacaaagatccacaggtttaatagcaagaaaagtcttctcatccaat
ctagctcatctatctttggtattcttttggattagtggaatgcaccttcatggggcatat
ttgtctaattatgatatttggttaaaagatcccaaatccatcaccccatcatcacatcta
gcctactctttaattggtcaagatattcttaactcatatacctcagaatacttctcagga
atcactatcacatctggctttttccaactctaccgttctgagggtatcattacacagtcc
cagctgaaatatgcatgtgctacttccctaatagctacacttatctgcctttctggatct
tatctccacatgcagcttatgtccaaattcactagcttttacaagaagttccagtcctta
tcgcaagatcatctcataattatatttggctccagctccacatccctctccgctcatcag
atacacaaaatgcttccagctaaccctttactagattcaggtatctccaacccaagtatc
ctacaagtaatctccaacagtctcagtgaaaacctagcactgttttcaacaaatctaagc
tctacaggtaaattgttaaatccctcaacaagaagtgtattcctttctcaagttgcagca
catcataagacaacaggtgtagtctttatcacactagggcttatcagattcctcaccatg
tataactctcaatttagtatacttacatcttatatagattaccacattgtgctatctatt
aatttagcattaattgcatctttatcaataatagtagctgatcatctcacaagaacccca
atctatcctcacaactcaacctcataccctacaatcctttgcctctccatacatcatgca
tggttatccggtttcctcatcattggttcaggagctcatgcatcaatattcaacttgcga
gcatcaccaacatctgagataagacatcgagaccccatatactcacacctcatctgggta
tgtatagcaataggattacactcattcagtctctactgtcataacgacactttagaagca
ttaggacgtccagaagatatctttcatgacaactctatccagttaaaagcaatatttgcc
aagcaatcctttctaagagcagagctccagccagatatcgagatgttagataaaaagatt
atacgtataacccaagaattgggaacagcagattttatagttcaccacattcatgcattt
acaatccatgttactctcttaattctttcaaagggtgtactttatgctagaaactctaga
tttgtatcagataaattggaacttggttttacttatccctgtgatggtccaggtagaggt
ggtacatgtcaaatatcaccttgggatcacttattctcagctgtattttggatgtataac
tgtttaaatgtagtaacattccactacttttggaagatgcaatcagatgtttggggcttt
gtatcgatccaaaagcacatatctcactacagccaaggtgattttagtgtcaactctatt
accatcaatggttggttgagaaacttattatggtccgaggcatcccaagtaatccaatcc
tatgctctttcatccatttgtccatatgggttcattttcctcatagctcatttcatctgg
gcatttagtttaatgttcctctttagtggtagagcatactggcaagaattaattgagtcc
attctatggtcacatcataaattaaagattatacctcatatccaaccaagggcattaagc
atttctcaaggaagagcagttggtttcattcattacactctaggaggtatcggctcaacc
tgggcatttattatttcaagactgctagtattaacttcttga
>KrE13_psaA
atgcacatcttcagatatatcaacacaacactgtggtccaaagcaggacattttaataaa
gctttatcaaaaggagctaagacaactacttggatatggaatctacatgattatgctcat
gattttgacattcaacaaagatccacaggtttaatagcaagaaaagtcttctcatccaat
ctagctcatctatctttggtattcttttggattagtggaatgcaccttcatggggcatat
ttgtctaattatgatatttggttaaaagatcccaaatccatcaccccatcatcacatcta
gcctactctttaattggtcaagatattcttaactcatatacctcagaatacttctcagga
atcactatcacatctggctttttccaactctaccgttctgagggtatcattacacagtcc
cagctgaaatatgcatgtgctacttccctaatagctacacttatctgcctttctggatct
tatctccacatgcagcttatgtccaaattcactagcttttacaagaagttccagtcctta
tcgcaagatcatctcataattatatttggctccagctccacatccctctccgctcatcag
atacacaaaatgcttccagctaaccctttactagattcaggtatctccaacccaagtatc
ctacaagtaatctccaacagtctcagtgaaaacctagcactgttttcaacaaatctaagc
tctacaggtaaattgttaaatccctcaacaagaagtgtattcctttctcaagttgcagca
catcataagacaacaggtgtagtctttatcacactagggcttatcagattcctcaccatg
tataactctcaatttagtatacttacatcttatatagattaccacattgtgctatctatt
aatttagcattaattgcatctttatcaataatagtagctgatcatctcacaagaacccca
atctatcctcacaactcaacctcataccctacaatcctttgcctctccatacatcatgca
tggttatccggtttcctcatcattggttcaggagctcatgcatcaatattcaacttgcga
gcatcaccaacatctgagataagacatcgagaccccatatactcacacctcatctgggta
tgtatagcaataggattacactcattcagtctctactgtcataacgacactttagaagca
ttaggacgtccagaagatatctttcatgacaactctatccagttaaaagcaatatttgcc
aagcaatcctttctaagagcagagctccagccagatatcgagatgttagataaaaagatt
atacgtataacccaagaattgggaacagcagattttatagttcaccacattcatgcattt
acaatccatgttactctcttaattctttcaaagggtgtactttatgctagaaactctaga

tttgtatcagataaattggaacttggttttacttatccctgtgatggtccaggtagaggt
ggtacatgtcaaatatcaccttgggatcacttattctcagctgtattttggatgtataac
tgtttaaatgtagtaacattccactacttttggaagatgcaatcagatgtttggggcttt
gtatcgatccaaaagcacatatctcactacagccaaggtgattttagtgtcaactctatt
accatcaatggttggttgagaaacttattatggtccgaggcatcccaagtaatccaatcc
tatgctctttcatccatttgtccatatgggttcattttcctcatagctcatttcatctgg
gcatttagtttaatgttcctctttagtggtagagcatactggcaagaattaattgagtcc
attctatggtcacatcataaattaaagattatacctcatatccaaccaagggcattaagc
atttctcaaggaagagcagttggtttcattcattacactctaggaggtatcggctcaacc
tgggcatttattatttcaagactgctagtattaacttcttga
>KrE14_psaA
atgcacatcttcagatatatcaacacaacactgtggtccaaagcaggacattttaataaa
gctttatcaaaaggagctaagacaactacttggatatggaatctacatgattatgctcat
gattttgacattcaacaaagatccacaggtttaatagcaagaaaagtcttctcatccaat
ctagctcatctatctttggtattcttttggattagtggaatgcaccttcatggggcatat
ttgtctaattatgatatttggttaaaagatcccaaatccatcaccccatcatcacatcta
gcctactctttaattggtcaagatattcttaactcatatacctcagaatacttctcagga
atcactatcacatctggctttttccaactctaccgttctgagggtatcattacacagtcc
cagctgaaatatgcatgtgctacttccctaatagctacacttatctgcctttctggatct
tatctccacatgcagcttatgtccaaattcactagcttttacaagaagttccagtcctta
tcgcaagatcatctcataattatatttggctccagctccacatccctctccgctcatcag
atacacaaaatgcttccagctaaccctttactagattcaggtatctccaacccaagtatc
ctacaagtaatctccaacagtctcagtgaaaacctagcactgttttcaacaaatctaagc
tctacaggtaaattgttaaatccctcaacaagaagtgtattcctttctcaagttgcagca
catcataagacaacaggtgtagtctttatcacactagggcttatcagattcctcaccatg
tataactctcaatttagtatacttacatcttatatagattaccacattgtgctatctatt
aatttagcattaattgcatctttatcaataatagtagctgatcatctcacaagaacccca
atctatcctcacaactcaacctcataccctacaatcctttgcctctccatacatcatgca
tggttatccggtttcctcatcattggttcaggagctcatgcatcaatattcaacttgcga
gcatcaccaacatctgagataagacatcgagaccccatatactcacacctcatctgggta
tgtatagcaataggattacactcattcagtctctactgtcataacgacactttagaagca
ttaggacgtccagaagatatctttcatgacaactctatccagttaaaagcaatatttgcc
aagcaatcctttctaagagcagagctccagccagatatcgagatgttagataaaaagatt
atacgtataacccaagaattgggaacagcagattttatagttcaccacattcatgcattt
acaatccatgttactctcttaattctttcaaagggtgtactttatgctagaaactctaga
tttgtatcagataaattggaacttggttttacttatccctgtgatggtccaggtagaggt
ggtacatgtcaaatatcaccttgggatcacttattctcagctgtattttggatgtataac
tgtttaaatgtagtaacattccactacttttggaagatgcaatcagatgtttggggcttt
gtatcgatccaaaagcacatatctcactacagccaaggtgattttagtgtcaactctatt
accatcaatggttggttgagaaacttattatggtccgaggcatcccaagtaatccaatcc
tatgctctttcatccatttgtccatatgggttcattttcctcatagctcatttcatctgg
gcatttagtttaatgttcctctttagtggtagagcatactggcaagaattaattgagtcc
attctatggtcacatcataaattaaagattatacctcatatccaaccaagggcattaagc
atttctcaaggaagagcagttggtttcattcattacactctaggaggtatcggctcaacc
tgggcatttattatttcaagactgctagtattaacttcttga
>KrE15_psaA
atgcacatcttcagatatatcaacacaacactgtggtccaaagcaggacattttaataaa
gctttatcaaaaggagctaagacaactacttggatatggaatctacatgattatgctcat
gattttgacattcaacaaagatccacaggtttaatagcaagaaaagtcttctcatccaat
ctagctcatctatctttggtattcttttggattagtggaatgcaccttcatggggcatat
ttgtctaattatgatatttggttaaaagatcccaaatccatcaccccatcatcacatcta
gcctactctttaattggtcaagatattcttaactcatatacctcagaatacttctcagga
atcactatcacatctggctttttccaactctaccgttctgagggtatcattacacagtcc
cagctgaaatatgcatgtgctacttccctaatagctacacttatctgcctttctggatct
tatctccacatgcagcttatgtccaaattcactagcttttacaagaagttccagtcctta
tcgcaagatcatctcataattatatttggctccagctccacatccctctccgctcatcag
atacacaaaatgcttccagctaaccctttactagattcaggtatctccaacccaagtatc

ctacaagtaatctccaacagtctcagtgaaaacctagcactgttttcaacaaatctaagc
tctacaggtaaattgttaaatccctcaacaagaagtgtattcctttctcaagttgcagca
catcataagacaacaggtgtagtctttatcacactagggcttatcagattcctcaccatg
tataagtctcaatttagtatacttacatcttatatagattaccacattgtgctatctatt
aatttagcattaattgcatctttatcaataatagtagctgatcatctcacaagaacccca
atctatcctcacaactcaacctcataccctacaatcctttgcctctccatacatcatgca
tggttatccggtttcctcatcattggttcaggagctcatgcatcaatattcaacttgcga
gcatcaccaacatctgagataagacatcgagaccccatatactcacacctcatctgggta
tgtatagcaataggattacactcattcagtctctactgtcataacgacactttagaagca
ttaggacgtccagaagatatctttcatgacaactctatccagttaaaagcaatatttgcc
aagcaatcctttctaagagcagagctccagccagatatcgagatgttagataaaaagatt
atacgtataacccaagaattgggaacagcagattttatagttcaccacattcatgcattt
acaatccatgttactctcttaattctttcaaagggtgtactttatgctagaaactctaga
tttgtatcagataaattggaacttggttttacttatccctgtgatggtccaggtagaggt
ggtacatgtcaaatatcaccttgggatcacttattctcagctgtattttggatgtataac
tgtttaaatgtagtaacattccactacttttggaagatgcaatcagatgtttggggcttt
gtatcgatccaaaagcacatatctcactacagccaaggtgattttagtgtcaactctatt
accatcaatggttggttgagaaacttattatggtccgaggcatcccaagtaatccaatcc
tatgctctttcatccatttgtccatatgggttcattttcctcatagctcatttcatctgg
gcatttagtttaatgttcctctttagtggtagagcatactggcaagaattaattgagtcc
attctatggtcacatcataaattaaagattatacctcatatccaaccaagggcattaagc
atttctcaaggaagagcagttggtttcattcattacactctaggaggtatcggctcaacc
tgggcatttattatttcaagactgctagtattaacttcttga
>KrE2_psaA
atgcacatcttcagatatatcaacacaacaatgtggtccaaagcaggacattttaataaa
gctttatcaaaaggagctaagacaactacttggatatggaatctacatgattatgctcat
gattttgacattcaacaaagatccacaggtttaatagcaagaaaagtcttctcatccaat
ctagctcatctatctttggtattcttttggattagtggaatgcaccttcatggggcatat
ttgtctaattatgatatttggttaaaagatcccaaatccatcaccccatcatcacatcta
gcctactctttaattggtcaagatattcttaactcatatacctcagaatacttctcagga
atcactatcacatctggctttttccaactctaccgttctgagggtatcattacacagtcc
cagctgaaatatgcatgtgctacttccctaatagctacacttatctgcctttctggatct
tatctccacatgcagcttatgtccaaattcactagcttttacaagaagttccagtcctta
tcgcaagatcatctcataattatatttggctccagctccacatccctctccgctcatcag
atacacaaaatgcttccagctaacccttttactagattcaggtatctccaacccaagtatc
ctacaagtaatctccaacagtctcagtgaaaacctagcactgttttcaacaaatctaagc
tctacaggtaaattgttaaatccctcaacaagaagtgtattcctttctcaagttgcagca
catcataagacaacaggtgtagtctttatcacactagggcttatcagattcctcaccatg
tataactctcaatttagtatacttacatcttatatagattaccacattgtgctatctatt
aatttagcattaattgcatctttatcaataatagtagctgatcatctcacaagaacccca
atctatcctcacaactcaacctcataccctacaatcctttgcctctccatacatcatgca
tggttatccggtttcctcatcattggttcaggagctcatgcatcaatattcaacttgcga
gcatcaccaacatctgagataagacatcgagaccccatatactcacacctcatctgggta
tgtatagcaataggattacactcattcagtctctactgtcataacgacactttagaagca
ttaggacgtccagaagatatctttcatgacaactctatccagttaaaagcaatatttgcc
aagcaatcctttctaagagcagagctccagccagatatcgagatgttagataaaaagatt
atacgtataacccaagaattgggaacagcagattttatagttcaccacattcatgcattt
acaatccatgttactctcttaattctttcaaagggtgtactttatgctagaaactctaga
tttgtatcagataaattggaacttggttttacttatccctgtgatggtccaggtagaggt
ggtacatgtcaaatatcaccttgggatcacttattctcagctgtattttggatgtataac
tgtttaaatgtagtaacattccactacttttggaagatgcaatcagatgtttggggcttt
gtatcgatccaaaagcacatatctcactacagccaaggtgattttagtgtcaactctatt
accatcaatggttggttgagaaacttattatggtccgaggcatcccaagtaatccaatcc
tatgctctttcatccatttgtccatatgggttcattttcctcatagctcatttcatctgg
gcatttagtttaatgttcctctttagtggtagagcatactggcaagaattaattgagtcc
attctatggtcacatcataaattaaagattatacctcatatccaaccaagggcattaagc
atttctcaaggaagagcagttggtttcattcattacactctaggaggtatcggctcaacc

tgggcatttattatttcaagactgctagtattaacttcttga
>KrE3_psaA
atgcacatcttcagatatatcaacacaacactgtggtccaaagcaggacattttaataaa
gctttatcaaaaggagctaagacaactacttggatatggaatctacatgattatgctcat
gattttgacattcaacaaagatccacaggtttaatagcaagaaaagtcttctcatccaat
ctagctcatctatctttggtattcttttggattagtggaatgcaccttcatggagcatat
ttgtctaattatgatatttggttaaaagatcccaaatccatcacgccatcatcacatcta
gcctactctttaattggtcaagatattcttaactcatatacctcagaatacttctcagga
atcactatcacatctggctttttccaactctaccgttctgagggtatcattacacagtcc
cagctgaaatatgcatgtgctacttccctaatagctacacttatctgcctttctggatct
tatctccacatgcagcttatgtccaaattcactagcttttacaagaagttccagtcctta
tcgcaagatcatctcataattatatttggctccagctccacatccctctccgctcatcag
atacacaaaatgcttccagctaaccctttactagattcaggtatctccaacccaagtatc
ctacaagtaatctccaacagtctcagtgaaaacctagcactgttttcaacaaatctaagc
tctacaggtaaattgttaaatccctcaacaagaagtgtattcctttctcaagttgcagca
catcataagacaacaggtgtagtctttatcacactagggcttatcagattcctcaccatg
tataactctcaatttagtatacttacatcttatatagattaccacattgtgctatctatt
aatttagcattaattgcatctttatcaataatagtagctgatcatctcacaagaacccca
atctatcctcacaactcaacctcataccctacaatcctttgcctctccatacatcatgca
tggttatccggtttcctcatcattggttcaggagctcatgcatcaatattcaacttgcga
gcatcaccaacatctgagataagacatcgagaccccatatactcacacctcatctgggta
tgtatagcaataggattacactcattcagtctctactgtcataacgacactttagaagca
ttaggacgtccagaagatatctttcatgacaactctatccagttaaaagcaatatttgcc
aagcaatcctttctaagagcagagctccagccagatatcgagatgttagataaaaagatt
atacgtataacccaagaattgggaacagcagattttatagttcaccacattcatgcattt
acaatccatgttactctcttaattctttcaaagggtgtactttatgctagaaactctaga
tttgtatcagataaattggaacttggttttacttatccctgtgatggtccaggtagaggt
ggtacatgtcaaatatcaccttgggatcacttattctcagctgtattttggatgtataac
tgtttaaatgtagtaacattccactacttttggaagatgcaatcagatgtttggggcttt
gtatcgatccaaaagcacatatctcactacagccaaggtgattttagtgtcaactctatt
accatcaatggttggttgagaaacttattatggtccgaggcatcccaagtaatccaatcc
tatgctctttcatccatttgtccatatgggttcattttcctcatagctcatttcatctgg
gcatttagtttaatgttcctctttagtggtagagcatactggcaagaattaattgagtcc
attctatggtcacatcataaattaaagattatacctcatatccaaccaagggcattaagc
atttctcaaggaagagcagttggtttcattcattacactctaggaggtatcggctcaacc
tgggcatttattatttcaagactgctagtattaacttcttga
>KrE4_psaA
atgcacatcttcagatatatcaacacaacactgtgggccaaagcaggacattttaataaa
gctttatcaaaaggagctaagacaactacttggatatggaatctacatgattatgctcat
gattttgacattcaacaaagatccacaggtttaatagcaagaaaagtcttctcatccaat
ctagctcatctatctttggtattcttttggattagtggaatgcaccttcatggggcatat
ttgtctaattatgatatttggttaaaagatcccaaatccatcacccccatcatcacatcta
gcctactctttaattggtcaagatattcttaactcatatacctcagaatacttctcagga
atcactatcacatctggctttttccaactctaccgttctgagggtatcattacacagtcc
cagctgaaatatgcatgtgctacttccctaatagctacacttatctgcctttctggatct
tatctccacatgcagcttatgtccaaattcactagcttttacaagaagttccagtcctta
tcgcaagatcatctcataattatatttggctccagctccacatccctctccgctcatcag
atacacaaaatgcttccagctaaccctttactagattcaggtatctccaacccaagtatc
ctacaagtaatctccaacagtctcagtgaaaacctagcactgttttcaacaaatctaagc
tctacaggtaaattgttaaatccctcaacaagaagtgtattcctttctcaagttgcagca
catcataagacaacaggtgtagtctttatcacactagggcttatcagattcctcaccatg
tataactctcaatttagtatacttacatcttatatagattaccacattgtgctatctatt
aatttagcattaattgcatctttatcaataatagtagctgatcatctcacaagaacccca
atctatcctcacaactcaacctcataccctacaatcctttgcctctccatacatcatgca
tggttatccggtttcctcatcattggttcaggagctcatgcatcaatattcaacttgcga
gcatcaccaacatctgagataagacatcgagaccccatatactcacacctcatctgggta
tgtatagcaataggattacactcattcagtctctactgtcataacgacactttagaagca

ttaggacgtccagaagatatctttcatgacaactctatccagttaaaagcaatatttgcc
aagcaatcctttctaagagcagagctccagccagatatcgagatgttagataaaaagatt
atacgtataacccaagaattgggaacagcagattttatagttcaccacattcatgcattt
acaatccatgttactctcttaattctttcaaagggtgtactttatgctagaaactctaga
tttgtatcagataaattggaacttggttttacttatccctgtgatggtccaggtagaggt
ggtacatgtcaaatatcaccttgggatcacttattctcagctgtattttggatgtataac
tgtttaaatgtagtaacattccactacttttggaagatgcaatcagatgtttggggcttt
gtatcgatccaaaagcacatatctcactacagccaaggtgattttagtgtcaactctatt
accatcaatggttggttgagaaacttattatggtccgaggcatcccaagtaatccaatcc
tatgctctttcatccatttgtccatatgggttcattttcctcatagctcatttcatctgg
gcatttagtttaatgttcctctttagtggtagagcatactggcaagaattaattgagtcc
attctatggtcacatcataaattaaagattatacctcatatccaaccaagggcattaagc
atttctcaaggaagagcagttggtttcattcattacactctaggaggtatcggctcaacc
tgggcatttattatttcaagactgctagtattaacttcttga
>KrE5_psaA
atgcacatcttcagatatatcaacacaacactgtggtccaaagcaggacattttaataaa
gctttatcaaaaggagctaagacaactacttggatatggaatctacatgattatgctcat
gattttgacattcaacaaagatccacaggtttaatagcaagaaaagtcttctcatccaat
ctagctcatctatctttggtattcttttggattagtggaatgcaccttcatggggcatat
ttgtctaattatgatatttggttaaaagatcccaaatccatcaccccatcatcacatcta
gcctactctttaattggtcaagatattcttaactcatatacctcagaatacttctcagga
atcactatcacatctggctttttccaactctaccgttctgagggtatcattacacagtcc
cagctgaaatatgcatgtgctacttccctaatagctacacttatctgcctttctggatct
tatctccacatgcagcttatgtccaaattcactagcttttacaagaagttccagtcctta
tcgcaagatcatctcataattatatttggctccagctccacatccctctccgctcatcag
atacacaaaatgcttccagctaaccctttactagattcaggtatctccaacccaagtatc
ctacaagtaatctccaacagtctcagtgaaaacctagcactgttttcaacaaatctaagc
tctacaggtaaattgttaaatccctcaacaagaagtgtattcctttctcaagttgcagca
catcataagacaacaggtgtagtctttatcacactagggcttatcagattcctcaccatg
tataactctcaatttagtatacttacatcttatatagattaccacattgtgctatctatt
aatttagcattaattgcatctttatcaataatagtagctgatcatctcacaagaacccca
atctatcctcacaactcaacctcataccctacaatcctttgcctctccatacatcatgca
tggttatccggtttcctcatcattggttcaggagctcatgcatcaatattcaacttgcga
gcatcaccaacatctgagataagacatcgagaccccatatactcacacctcatctgggta
tgtatagcaataggattacactcattcagtctctactgtcataacgacactttagaagca
ttaggacgtccagaagatatctttcatgacaactctatccagttaaaagcaatatttgcc
aagcaatcctttctaagagcagagctccagccagatatcgagatgttagataaaaagatt
atacgtataacccaagaattgggaacagcagattttatagttcaccacattcatgcattt
acaatccatgttactctcttaattctttcaaagggtgtactttatgctagaaactctaga
tttgtatcagataaattggaacttggttttacttatccctgtgatggtccaggtagaggt
ggtacatgtcaaatatcaccttgggatcacttattctcagctgtattttggatgtataac
tgtttaaatgtagtaacattccactacttttggaagatgcaatcagatgtttggggcttt
gtatcgatccaaaagcacatatctcactacagccaaggtgattttagtgtcaactctatt
accatcaatggttggttgagaaacttattatggtccgaggcatcccaagtaatccaatcc
tatgctctttcatccatttgtccatatgggttcattttcctcatagctcatttcatctgg
gcatttagtttaatgttcctctttagtggtagagcatactggcaagaattaattgagtcc
attctatggtcacatcataaattaaagattatacctcatatccaaccaagggcattaagc
atttctcaaggaagagcagttggtttcattcattacactctaggaggtatcggctcaacc
tgggcatttattatttcaagactgctagtattaacttcttga
>KrE6_psaA
atgcacatcttcagatatatcaacacaacactgtggtccaaagcaggacattttaataaa
gctttatcaaaaggagctaagacaactacttggatatggaatctacatgattatgctcat
gattttgacattcaacaaagatccacaggtttaatagcaagaaaagtcttctcatccaat
ctagctcatctatctttggtattcttttggattagtggaatgcaccttcatggggcatat
ttgtctaattatgatatttggttaaaagatcccaaatccatcaccccatcatcacatcta
gcctactctttaattggtcaagatattcttaactcatatacctcagaatacttctcagga
atcactatcacatctggctttttccaactctaccgttctgagggtatcattacacagtcc

cagctgaaatatgcatgtgctacttccctaatagctacacttatctgcctttctggatct
tatctccacatgcagcttatgtccaaattcactagcttttacaagaagttccagtcctta
tcgcaagatcatctcataattatatttggctccagctccacatccctctccgctcatcag
atacacaaaatgcttccagctaaccctttactagattcaggtatctccaacccaagtatc
ctacaagtaatctccaacagtctcagtgaaaacctagcactgttttcaacaaatctaagc
tctacaggtaaattgttaaatccctcaacaagaagtgtattcctttctcaagttgcagca
catcataagacaacaggtgtagtctttatcacactagggcttatcagattcctcaccatg
tataactctcaatttagtatacttacatcttatatagattaccacattgtgctatctatt
aatttagcattaattgcatctttatcaataatagtagctgatcatctcacaagaacccca
atctatcctcacaactcaacctcataccctacaatcctttgcctctccatacatcatgca
tggttatccggtttcctcatcattggttcaggagctcatgcatcaatattcaacttgcga
gcatcaccaacatctgagataagacatcgagaccccatatactcacacctcatctgggta
tgtatagcaataggattacactcattcagtctctactgtcataacgacactttagaagca
ttaggacgtccagaagatatctttcatgacaactctatccagttaaaagcaatatttgcc
aagcaatcctttctaagagcagagctccagccagatatcgagatgttagataaaaagatt
atacgtataacccaagaattgggaacagcagattttatagttcaccacattcatgcattt
acaatccatgttactctcttaattctttcaaagggtgtactttatgctagaaactctaga
tttgtatcagataaattggaacttggttttacttatccctgtgatggtccaggtagaggt
ggtacatgtcaaatatcaccttgggatcacttattctcagctgtattttggatgtataac
tgtttaaatgtagtaacattccactacttttggaagatgcaatcagatgtttggggcttt
gtatcgatccaaaagcacatatctcactacagccaaggtgattttagtgtcaactctatt
accatcaatggttggttgagaaacttattatggtccgaggcatcccaagtaatccaatcc
tatgctctttcatccatttgtccatatgggttcattttcctcatagctcatttcatctgg
gcatttagtttaatgttcctctttagtggtagagcatactggcaagaattaattgagtcc
attctatggtcacatcataaattaaagattatacctcatatccaaccaagggcattaagc
atttctcaaggaagagcagttggtttcattcattacactctaggaggtatcggctcaacc
tgggcatttattatttcaagactgctagtattaacttcttga
>KrE7_psaA
atgcacatcttcagatatatcaacacaacactgtggtccaaagcaggacattttaataaa
gctttatcaaaaggagctaagacaactacttggatatggaatctacatgattatgctcat
gattttgacattcaacaaagatccacaggtttaatagcaagaaaagtcttctcatccaat
ctagctcatctatctttggtattcttttggattagtggaatgcaccttcatggggcatat
ttgtctaattatgatatttggttaaaagatcccaaatccatcaccccatcatcacatcta
gcctactctttaattggtcaagatattcttaactcatatacctcagaatacttctcagga
atcactatcacatctggctttttccaactctaccgttctgagggtatcattacacagtcc
cagctgaaatatgcatgtgctacttccctaatagctacacttatctgcctttctggatct
tatctccacatgcagcttatgtccaaattcactagcttttacaagaagttccagtcctta
tcgcaagatcatctcataattatatttggctccagctccacatccctctccgctcatcag
atacacaaaatgcttccagctaaccctttactagattcaggtatctccaacccaagtatc
ctacaagtaatctccaacagtctcagtgaaaacctagcactgttttcaacaaatctaagc
tctacaggtaaattgttaaatccctcaacaagaagtgtattcctttctcaagttgcagca
catcataagacaacaggtgtagtctttatcacactagggcttatcagattcctcaccatg
tataactctcaatttagtatacttacatcttatatagattaccacattgtgctatctatt
aatttagcattaattgcatctttatcaataatagtagctgatcatctcacaagaacccca
atctatcctcacaactcaacctcataccctacaatcctttgcctctccatacatcatgca
tggttatccggtttcctcatcattggttcaggagctcatgcatcaatattcaacttgcga
gcatcaccaacatctgagataagacatcgagaccccatatactcacacctcatctgggta
tgtatagcaataggattacactcattcagtctctactgtcataacgacactttagaagca
ttaggacgtccagaagatatctttcatgacaactctatccagttaaaagcaatatttgcc
aagcaatcctttctaagagcagagctccagccagatatcgagatgttagataaaaagatt
atacgtataacccaagaattgggaacagcagattttatagttcaccacattcatgcattt
acaatccatgttactctcttaattctttcaaagggtgtactttatgctagaaactctaga
tttgtatcagataaattggaacttggttttacttatccctgtgatgggccaggtagaggt
ggtacatgtcaaatatcaccttgggatcacttattctcagctgtattttggatgtataac
tgtttaaatgtagtaacattccactacttttggaagatgcaatcagatgtttggggcttt
gtatcgatccaaaagcacatatctcactacagccaaggtgattttagtgtcaactctatt
accatcaatggttggttgagaaacttattatggtccgaggcatcccaagtaatccaatcc

tatgctctttcatccatttgtccatatgggttcattttcctcataggtcatttcatctgg
gcatttagtttaatgttcctctttagtggtagagcatactggcaagaattaattgagtcc
attctatggtcacatcataaattaaagattatacctcatatccaaccaagggcattaagc
atttctcaaggaagagcagttggtttcattcattacactctaggaggtatcggctcaacc
tgggcatttattatttcaagactgctagtattaacttcttga
>KrE8_psaA
atgcacatcttcagatatatcaacacaacactgtggtccaaagcaggacattttaataaa
gctttatcaaaaggagctaagacaactacttggatatggaatctacatgattatgctcat
gattttgacattcaacaaagatccacaggtttaatagcaagaaaagtcttctcatccaat
ctagctcatctatctttggtattcttttggattagtggaatgcaccttcatggggcatat
ttgtctaattatgatatttggttaaaagatcccaaatccatcaccccatcatcacatcta
gcctactctttaattggtcaagatattcttaactcatatacctcagaatacttctcagga
atcactatcacatctggctttttccaactctaccgttctgagggtatcattacacagtcc
cagctgaaatatgcatgtgctacttccctaatagctacacttatctgcctttctggatct
tatctccacatgcagcttatgtccaaattcactagcttttacaagaagttccagtcctta
tcgcaagatcatctcataattatatttggctccagctccacatccctctccgctcatcag
atacacaaaatgcttccagctaaccctttactagattcaggtatctccaacccaagtatc
ctacaagtaatctccaacagtctcagtgaaaacctagcactgttttcaacaaatctaagc
tctacaggtaaattgttaaatccctcaacaagaagtgtattcctttctcaagttgcagca
catcataagacaacaggtgtagtctttatcacactagggcttatcagattcctcaccatg
tataactctcaatttagtatacttacatcttatatagattaccacattgtgctatctatt
aatttagcattaattgcatctttatcaataatagtagctgatcatctcacaagaacccca
atctatcctcacaactcaacctcataccctacaatcctttgcctctccatacatcatgca
tggttatccggtttcctcatcattggttcaggagctcatgcatcaatattcaacttgcga
gcatcaccaacatctgagataagacatcgagaccccatatactcacacctcatctgggta
tgtatagcaataggattacactcattcagtctctactgtcataacgacactttagaagca
ttaggacgtccagaagatatctttcatgacaactctatccagttaaaagcaatatttgcc
aagcaatcctttctaagagcagagctccagccagatatcgagatgttagataaaaagatt
atacgtataacccaagaattgggaacagcagattttatagttcaccacattcatgcattt
acaatccatgttactctcttaattctttcaaagggtgtactttatgctagaaactctaga
tttgtatcagataaattggaacttggttttacttatccctgtgatggtccaggtagaggt
ggtacatgtcaaatatcaccttgggatcacttattctcagctgtattttggatgtataac
tgtttaaatgtagtaacattccactacttttggaagatgcaatcagatgtttggggcttt
gtatcgatccaaaagcacatatctcactacagccaaggtgattttagtgtcaactctatt
accatcaatggttggttgagaaacttattatggtccgaggcatcccaagtaatccaatcc
tatgctctttcatccatttgtccatatgggttcattttcctcatagctcatttcatctgg
gcatttagtttaatgttcctctttagtggtagagcatactggcaagaattaattgagtcc
attctatggtcacatcataaattaaagattatacctcatatccaaccaagggcattaagc
atttctcaaggaagagcagttggtttcattcattacactctaggaggtatcggctcaacc
tgggcatttattatttcaagactgctagtattaacttcttga
>KrE9_psaA
atgcacatcttcagatatatcaacacaacactgtggtccaaagcaggacattttaataaa
gctttatcaaaaggagctaagacaactacttggatatggaatctacatgattatgctcat
gattttgacattcaacaaagatccacaggtttaatagcaagaaaagtcttctcatccaat
ctagctcatctatctttggtattcttttggattagtggaatgcaccttcatggggcatat
ttgtctaattatgatatttggttaaaagatcccaaatccatcaccccatcatcacatcta
gcctactctttaattggtcaagatattcttaactcatatacctcagaatacttctcagga
atcactatcacatctggctttttccaactctaccgttctgagggtatcattacacagtcc
cagctgaaatatgcatgtgctacttccctaatagctacacttatctgcctttctggatct
tatctccacatgcagcttatgtccaaattcactagcttttacaagaagttccagtcctta
tcgcaagatcatctcataattatatttggctccagctccacatccctctccgctcatcag
atacacaaaatgcttccagctaaccctttactagattcaggtatctccaacccaagtatc
ctacaagtaatctccaacagtctcagtgaaaacctagcactgttttcaacaaatctaagc
tctacaggtaaattgttaaatccctcaacaagaagtgtattcctttctcaagttgcagca
catcataagacaacaggtgtagtctttatcacactagggcttatcagattcctcaccatg
tataactctcaatttagtatacttacatcttatatagattaccacattgtgctatctatt
aatttagcattaattgcatctttatcaataatagtagctgatcatctcacaagaacccca

atctatcctcacaactcaacctcataccctacaatcctttgcctctccatacatcatgca
tggttatccggtttcctcatcattggttcaggagctcatgcatcaatattcaacttgcga
gcatcaccaacatctgagataagacatcgagaccccatatactcacacctcatctgggta
tgtatagcaataggattacactcattcagtctctactgtcataacgacactttagaagca
ttaggacgtccagaagatatctttcatgacaactctatccagttaaaagcaatatttgcc
aagcaatcctttctaagagcagagctccagccagatatcgagatgttagataaaaagatt
atacgtataacccaagaattgggaacagcagattttatagttcaccacattcatgcattt
acaatccatgttactctcttaattctttcaaagggtgtactttatgctagaaactctaga
tttgtatcagataaattggaacttggttttacttatccctgtgatggtccaggtagaggt
ggtacatgtcaaatatcaccttgggatcacttattctcagctgtattttggatgtataac
tgtttaaatgtagtaacattccactacttttggaagatgcaatcagatgtttggggcttt
gtatcgatccaaaagcacatatctcactacagccaaggtgattttagtgtcaactctatt
accatcaatggttggttgagaaacttattatggtccgaggcatcccaagtaatccaatcc
tatgctctttcatccatttgtccatatgggttcattttcctcatagctcatttcatctgg
gcatttagtttaatgttcctctttagtggtagagcatactggcaagaattaattgagtcc
attctatggtcacatcataaattaaagattatacctcatatccaaccaagggcattaagc
atttctcaaggaagagcagttggtttcattcattacactctaggaggtatcggctcaacc
tgggcatttattatttcaagactgctagtattaacttcttga
>Mz10_psaA
atgcacatcttcagatatatcaacacaacactgtggtccaaagcaggacattttaataaa
gctttatcaaaaggagctaagacaactacttggatatggaatctacatgattatgctcat
gattttgacattcaacaaagatccacaggtttaatagcaagaaaagtcttctcatccaat
ctagctcatctatctttggtattcttttggattagtggaatgcaccttcatggggcatat
ttgtctaattatgatatttggttaaaagatcccaaatccatcaccccatcatcacatcta
gcctactctttaattggtcaagatattcttaactcatatacctcagaatacttctcagga
atcactatcacatctggctttttccaactctaccgttctgagggtatcattacacagtct
cagctgaaatatgcatgtgctacttccctaatagctacacttatctgcctttctggatct
tatctccacatgcagcttatgtccaaattcactagcttttacaagaagttccagtcctta
tcgcaagatcatctcataattatatttggctccagctccacatccctctccgctcatcag
atacacaaaatgcttccagctaaccctttactagattcaggtatctccaacccaagtatc
ctacaagtaatctccaacagtctcagtgaaaacctagcactgttttcaacaaatctaagc
tctacaggtaaattgttaaatccctcaacaagaagtgtattcctttctcaagttgcagca
catcataagacaacaggtgtagtctttatcacactagggcttatcagattcctcaccatg
tataactctcaatttagtatacttacatcttatatagattaccacattgtgctatctatt
aatttagcattaattgcatctttatcaataatagtagctgatcatctcacaagaacccca
atctatcctcacaactcaacctcataccctacaatcctttgcctctccatacatcatgca
tggttatccggtttcctcatcattggttcaggagctcatgcatcaatattcaacttgcgt
ggatcaccaacatctgagataagacatcgagaccccatatactcacacctcatctgggta
tgtatagcaataggattacactcattcagtctctactgtcataacgacactttagaagca
ttaggacgtccagaagatatctttcatgacaactctatccagttaaaagcaatatttgcc
aagcaatcctttctaagagcagagctccagccagatatcgagatgttagataaaaagatt
atacgtataacccaagaattgggaacagcagattttatagttcaccacattcatgcattt
acaatccatgttactctcttaattctttcaaagggtgtactttatgctagaaactctaga
tttgtatcagataaattggaacttggttttacttatccctgtgatggtccaggtagaggt
ggtacatgtcaaatatcaccttgggatcacttattctcagctgtattttggatgtataac
tgtttaaatgtagtaacattccactacttttggaagatgcaatcagatgtttggggcttt
gtatcgatccaaaagcacatatctcactacagccaaggtgattttagtgtcaactctatt
accatcaatggttggttgagaaacttattatggtccgaggcatcccaagtaatccaatcc
tatgctctttcatccatttgtccatatgggttcattttcctcataggtcatttcatctgg
gcatttagtttaatgttcctctttagtggtagagcatactggcaagaattaattgagtcc
attctatggtcacatcataaattaaagattatacctcatatccaaccaagggcattaagc
atttctcaaggaagagcagttggtttcattcattacactctaggaggtatcggctcaacc
tgggcatttattatttcaagactgctagtattaacttcttga
>Mz5_psaA
atgcacatcttcagatatatcaacacaacactgtggtccaaagcaggacattttaataaa
gctttatcaaaaggagctaagacaactacttggatatggaatctacatgattatgctcat
gattttgacattcaacaaagatccacaggtttaatagcaagaaaagtcttctcatccaat

ctagctcatctatctttggtattcttttggattagtggaatgcaccttcatggggcatat
ttgtctaattatgatatttggttaaaagatcccaaatccatcaccccatcatcacatcta
gcctactctttaattggtcaagatattcttaactcatatacctcagaatacttctcagga
atcactatcacatctggctttttccaactctaccgttctgagggtatcattacacagtcc
cagctgaaatatgcatgtgctacttccctaatagctacacttatctgcctttctggatct
tatctccacatgcagcttatgtccaaattcactagcttttacaagaagttccagtcctta
tcgcaagatcatctcataattatatttggctccagctccacatccctctccgctcatcag
atacacaaaatgcttccagctaaccctttactagattcaggtatctccaacccaagtatc
ctacaagtaatctccaacagtctcagtgaaaacctagcactgttttcaacaaatctaagc
tctacaggtaaattgttaaatccctcaacaagaagtgtattcctttctcaagttgcagca
catcataagacaacaggtgtagtctttatcacactagggcttatcagattcctcaccatg
tataactctcaatttagtatacttacatcttatatagattaccacattgtgctatctatt
aatttagcattaattgcatctttatcaataatagtagctgatcatctcacaagaacccca
atctatcctcacaactcaacctcatacccta caatcctttgcctctccatacatcatgca
tggttatccggtttcctcatcattggttcaggagctcatgcatcaatattcaacttgcga
ggatcaccaacatctgagataagacatcgagaccccatatactcacacctcatctgggta
tgtatagcaataggattacactcattcagtctctactgtcataacgacactttagaagca
ttaggacgtccagaagatatctttcatgacaactctatccagttaaaagcaatatttgcc
aagcaatcctttctaagagcagagctccagccagatatcgagatgttagataaaaagatt
atacgtataacccaagaattgggaacagcagattttatagttcaccacattcatgcattt
acaatccatgttactctcttaattctttcaaagggtgtactttatgctagaaactctaga
tttgtatcagataaattggaacttggttttacttatccctgtgatggtccaggtagaggt
ggtacatgtcaaatatcaccttgggatcacttattctcagctgtattttggatgtataac
tgtttaaatgtagtaacattccactacttttggaagatgcaatcagatgtttggggcttt
gtatcgatccaaaagcacatatctcactacagccaaggtgattttagtgtcaactctatt
accatcaatggttggttgagaaacttattatggtccgaggcatcccaagtaatccaatcc
tatgctctttcatccatttgtccatatgggttcattttcctcataggtcatttcatctgg
gcatttagtttaatgttcctctttagtggtagagcatactggcaagaattaattgagtcc
attctatggtcacatcataaattaaagattatacctcatatccaaccaagggcattaagc
atttctcaaggaagagcagttggtttcattcattacactctaggaggtatcggctcaacc
tgggcatttattatttcaagactgctagtattaacttcttga
>Mz6_psaA
atgcacatcttcagatatatcaacacaacactgtggtccaaagcaggacattttaataaa
gctttatcaaaaggagctaagacaactacttggatatggaatctacatgattatgctcat
gattttgacattcaacaaagatccacaggtttaatagcaagaaaagtcttctcatccaat
ctagctcatctatctttggtattcttttggattagtggaatgcaccttcatggggcatat
ttgtctaattatgatatttggttaaaagatcccaaatccatcaccccatcatcacatcta
gcctactctttaattggtcaagatattcttaactcatatacctcagaatacttctcagga
atcactatcacatctggctttttccaactctaccgttctgagggtatcattacacagtcc
cagctgaaatatgcatgtgctacttccctaatagctacacttatctgcctttctggatct
tatctccacatgcagcttatgtccaaattcactagcttttacaagaagttccagtcctta
tcgcaagatcatctcataattatatttggctccagctccacatccctctccgctcatcag
atacacaaaatgcttccagctaaccctttactagattcaggtatctccaacccaagtatc
ctacaagtaatctccaacagtctcagtgaaaacctagcactgttttcaacaaatctaagc
tctacaggtaaattgttaaatccctcaacaagaagtgtattcctttctcaagttgcagca
catcataagacaacaggtgtagtctttatcacactagggcttatcagattcctcaccatg
tataactctcaatttagtatacttacatcttatatagattaccacattgtgctatctatt
aatttagcattaattgcatctttatcaataatagtagctgatcatctcacaagaacccca
atctatcctcacaactcaacctcatacccta caatcctttgcctctccatacatcatgca
tggttatccggtttcctcatcattggttcaggagctcatgcatcaatattcaacttgcga
gcatcaccaacatctgagataagacatcgagaccccatatactcacacctcatctgggta
tgtatagcaataggattacactcattcagtctctactgtcataacgacactttagaagca
ttaggacgtccagaagatatctttcatgacaactctatccagttaaaagcaatatttgcc
aagcaatcctttctaagagcagagctccagccagatatcgagatgttagataaaaagatt
atacgtataacccaagaagtgggaacagcagattttatagttcaccacattcatgcattt
acaatccatgttactctcttaattctttcaaagggtgtactttatgctagaaactctaga
tttgtatcagataaattggaacttggttttacttatccctgtgatggtccaggtagaggt

ggtacatgtcaaatatcaccttgggatcacttattctcagctgtattttggatgtataac
tgtttaaatgtagtaacattccactacttttggaagatgcaatcagatgtttggggcttt
gtatcgatccaaaagcacatatctcactacagccaaggtgattttagtgtcaactctatt
accatcaatggttggttgagaaacttattatggtccgaggcatcccaagtaatccaatcc
tatgctctttcatccatttgtccatatgggttcattttcctcatagctcatttcatctgg
gcatttagtttaatgttcctctttagtggtagagcatactggcaagaattaattgagtcc
attctatggtcacatcataaattaaagattatacctcatatccaaccaagggcattaagc
atttctcaaggaagagcagttggtttcattcattacactctaggaggtatcggctcaacc
tgggcatttattatttcaagactgctagtattaacttcttga
>Mz7_psaA
atgcacatcttcagatatatcaacacaacactgtggtccaaagcaggacattttaataaa
gctttatcaaaaggagctaagacaactacttggatatggaatctacatgattatgctcat
gattttgacattcaacaaagatccacaggtttaatagcaagaaaagtcttctcatccaat
ctagctcatctatctttggtattcttttggattagtggaatgcaccttcatggggcatat
ttgtctaattatgatatttggttaaaagatcccaaatccatcaccccatcatcacatcta
gcctactctttaattggtcaagatattcttaactcatatacctcagaatacttctcagga
atcactatcacatctggctttttccaactctaccgttctgagggtatcattacacagtcc
cagctgaaatatgcatgtgctacttccctaatagctacacttatctgcctttctggatct
tatctccacatgcagcttatgtccaaattcactagcttttacaagaagttccagtcctta
tcgcaagatcatctcataattatatttggctccagctccacatccctctccgctcatcag
atacacaaaatgcttccagctaaccctttactagattcaggtatctccaacccaagtatc
ctacaagtaatctccaacagtctcagtgaaaacctagcactgttttcaacaaatctaagc
tctacaggtaaattgttaaatccctcaacaagaagtgtattcctttctcaagttgcagca
catcataagacaacaggtgtagtctttatcacactagggcttatcagattcctcaccatg
tataactctcaatttagtatacttacatcttatatagattaccacattgtgctatctatt
aatttagcattaattgcatctttatcaataatagtagctgatcatctcacaagaacccca
atctatcctcacaactcaacctcataccctacaatcctttgcctctccatacatcatgca
tggttatccggtttcctcatcattggttcaggagctcatgcatcaatattcaacttgcga
gcatcaccaacatctgagataagacatcgagaccccatatactcacacctcatctgggta
tgtatagcaataggattacactcattcagtctctactgtcataacgacactttagaagca
ttaggacgtccagaagatatctttcatgacaactctatccagttaaaagcaatatttgcc
aagcaatcctttctaagagcagagctccagccagatatcgagatgttagataaaaagatt
atacgtataacccaagaattgggaacagcagattttatagttcaccacattcatgcattt
acaatccatgttactctcttaattctttcaaagggtgtactttatgctagaaactctaga
tttgtatcagataaattggaacttggttttacttatccctgtgatggtccaggtagaggt
ggtacatgtcaaatatcaccttgggatcacttattctcagctgtattttggatgtataac
tgtttaaatgtagtaacattccactacttttggaagatgcaatcagatgtttggggcttt
gtatcgatccaaaagcacatatctcactacagccaaggtgattttagtgtcaactctatt
accatcaatggttggttgagaaacttattatggtccgaggcatcccaagtaatccaatcc
tatgctctttcatccatttgtccatatgggttcattttcctcatagctcatttcatctgg
gcatttagtttaatgttcctctttagtggtagagcatactggcaagaattaattgagtcc
attctatggtcacatcataaattaaagattatacctcatatccaaccaagggcattaagc
atttctcaaggaagagcagttggtttcattcattacactctaggaggtatcggctcaacc
tgggcatttattatttcaagactgctagtattaacttcttga
>Mz8_psaA
atgcacatcttcagatatatcaacacaacactgtggtccaaagcaggacattttaataaa
gctttatcaaaaggagctaagacaactacttggatatggaatctacatgattatgctcat
gattttgacattcaacaaagatccacaggtttaatagcaagaaaagtcttctcatccaat
ctagctcatctatctttggtattcttttggattagtggaatgcaccttcatggagcatat
ttgtctaattatgatatttggttaaaagatcccaaatccatcacgccatcatcacatcta
gcctactctttaattggtcaagatattcttaactcatatacctcagaatacttctcagga
atcactatcacatctggctttttccaactctaccgttctgagggtatcattacacagtcc
cagctcaaatatgcatgtgctacttccctaatagctacacttatctgcctttctggatct
tatctccacatgcagcttatgtccaaattcactagcttttacaagaagttccagtcctta
tcgcaagatcatctcataattatatttggctccagctccacatccctctccgctcatcag
atacacaaaatgcttccagctaaccctttactagattcaggtatctccaagccaagtatc
ctacaagtaatctccaacagtctcagtgaaaacctagcactgttttcaacaaatctaagc

tctacaggtaaattgttaaatccctcaacaagaagtgtattcctttctcaagttgcagca
catcataagacaacaggtgtagtctttatcacactagggcttatcagattcctcaccatg
tataagtctcaatttagtatacttacatcttatatagattaccacattgtgctatctatt
aatttagcattaattgcatctttatcaataatagtagctgatcatctcacaagaacccca
atctatcctcacaagtcaacctcataccctacaatcctttgcctctccatacatcatgca
tggttatccggtttcctcatcattggttcaggagctcatgcatcaatattcaacttgcga
gcatcaccaacatctgagataagacatcgagaccccatatactcacacctcatctgggta
tgtatagcaataggattacactcattcagtctctactgtcataacgacactttagaagca
ttaggacgtccagaagatatctttcatgacaactctatccagttaaaagcaatatttgcc
aagcaatcctttctaagagcagagctccagccagatatcgagatgttagataaaaagatt
atacgtataacccaagaattgggaacagcagattttatagttcaccacattcatgcattt
acaatccatgttactctcttaattctttcaaagggtgtactttatgctagaaactctaga
tttgtatcagataaattggaacttggttttacttatccctgtgatggtccaggtagaggt
ggtacatgtcaaatatcaccttgggatcacttattctcagctgtattttggatgtataac
tgtttaaatgtagtaacattccactacttttggaagatgcaatcagatgtttggggcttt
gtatcgatccaaaagcacatatctcactacagccaaggtgattttagtgtcaactctatt
accatcaatggttggttgagaaacttattatggtccgaggcatcccaagtaatccaatcc
tatgctctttcatccatttgtccatatgggttcattttcctcataggtcatttcatctgg
gcatttagtttaatgttcctctttagtggtagagcatactggcaagaattaattgagtcc
attctatggtcacatcataaattaaagattatacctcatatccaaccaagggcattaagc
atttctcaaggaagagcagttggtttcattcattacactctaggaggtatcggctcaacc
tgggcatttattatttcaagactgctagtattaacttcttga
>Mz9_psaA
atgcacatcttcagatatatcaacacaacactgtgggccaaagcaggacattttaataaa
gctttatcaaaaggagctaagacaactacttggatatggaatctacatgattatgctcat
gattttgacattcaacaaagatccacaggtttaatagcaagaaaagtcttctcatccaat
ctagctcatctatctttggtattcttttggattagtggaatgcaccttcatggagcatat
ttgtctaattatgatatttggttaaaagatcccaaatccatcaccccatcatcacatcta
gcctactctttaattggtcaagatattcttaactcatatacctcagaatacttctcagga
atcactatcacatctggctttttccaactctaccgttctgagggtatcattacacagtcc
cagctcaaatatgcatgtgctacttccctaatagctacacttatctgcctttctggatct
tatctccacatgcagcttatgtccaaattcactagcttttacaagaagttccagtcctta
tcgcaagatcatctcataattatatttggctccagctccacatccctctccgctcatcag
atacacaaaatgcttccagctaaccctttactagattcaggtatctccaacccaagtatc
ctacaagtaatctccaacagtctcagtgaaaacctagcactgttttcaacaaatctaagc
tctacaggtaaattgttaaatccctcaacaagaagtgtattcctttctcaagttgcagca
catcataagacaacaggtgtagtctttatcacactagggcttatcagattcctcaccatg
tataagtctcaatttagtatacttacatcttatatagattaccacattgtgctatctatt
aatttagcattaattgcatctttatcaataatagtagctgatcatctcacaagaacccca
atctatcctcacaactcaacctcataccctacaatcctttgcctctccatacatcatgca
tggttatccggtttcctcatcattggttcaggagctcatgcatcaatattcaacttgctt
ggatcaccaacatctgagataagacatcgagaccccatatactcacacctcatctgggta
tgtatagcaataggattacactcattcagtctctactgtcataacgacactttagaagca
ttaggacgtccagaagatatctttcatgacaactctatccagttaaaagcaatatttgcc
aagcaatcctttctaagagcagagctccagccagatatcgagatgttagataaaaagatt
atacgtataacccaagaattgggaacagcagattttatagttcaccacattcatgcattt
acaatccatgttactctcttaattctttcaaagggtgtactttatgctagaaactctaga
tttgtatcagataaattggaacttggttttacttatccctgtgatggtccaggtagaggt
ggtacatgtcaaatatcaccttgggatcacttattctcagctgtattttggatgtataac
tgtttaaatgtagtaacattccactacttttggaagatgcaatcagatgtttggggcttt
gtatcgatccaaaagcacatatctcactacagccaaggtgattttagtgtcaactctatt
accatcaatggttggttgagaaacttattatggtccgaggcatcccaagtaatccaatcc
tatgctctttcatccatttgtccatatgggttcattttcctcataggtcatttcatctgg
gcatttagtttaatgttcctctttagtggtagagcatactggcaagaattaattgagtcc
attctatggtcacatcataaattaaagattatacctcatatccaaccaagggcattaagc
atttctcaaggaagagcagttggtttcattcattacactctaggaggtatcggctcaacc
tgggcatttattatttcaagactgctagtattaacttcttga

>MzC1_psaA
atgcacatcttcagatatatcaacacaacactgtggtccaaagcaggacattttaataaa
gctttatcaaaaggagctaagacaactacttggatatggaatctacatgattatgctcat
gattttgacattcaacaaagatccacaggtttaatagcaagaaaagtcttctcatccaat
ctagctcatctatctttggtattcttttggattagtggaatgcaccttcatggggcatat
ttgtctaattatgatatttggttaaaagatcccaaatccatcaccccatcatcacatcta
gcctactctttaattggtcaagatattcttaactcatatacctcagaatacttctcagga
atcactatcacatctggctttttccaactctaccgttctgagggtatcattacacagtcc
cagctgaaatatgcatgtgctacttccctaatagctacacttatctgcctttctggatct
tatctccacatgcagcttatgtccaaattcactagcttttacaagaagttccagtcctta
tcgcaagatcatctcataattatatttggctccagctccacatccctctccgctcatcag
atacacaaaatgcttccagctaaccctttactagattcaggtatctccaacccaagtatc
ctacaagtaatctccaacagtctcagtgaaaacctagcactgttttcaacaaatctaagc
tctacaggtaaattgttaaatccctcaacaagaagtgtattcctttctcaagttgcagca
catcataagacaacaggtgtagtctttatcacactagggcttatcagattcctcaccatg
tataactctcaatttagtatacttacatcttatatagattaccacattgtgctatctatt
aatttagcattaattgcatctttatcaataatagtagctgatcatctcacaagaacccca
atctatcctcacaactcaacctcataccctacaatcctttgcctctccatacatcatgca
tggttatccggtttcctcatcattggttcaggagctcatgcatcaatattcaacttgcga
gcatcaccaacatctgagataagacatcgagaccccatatactcacacctcatctgggta
tgtatagcaataggattacactcattcagtctctactgtcataacgacactttagaagca
ttaggacgtccagaagatatctttcatgacaactctatccagttaaaagcaatatttgcc
aagcaatcctttctaagagcagagctccagccagatatcgagatgttagataaaaagatt
atacgtataacccaagaattgggaacagcagattttatagttcaccacattcatgcattt
acaatccatgttactctcttaattctttcaaagggtgtactttatgctagaaactctaga
tttgtatcagataaattggaacttggttttacttatccctgtgatggtccaggtagaggt
ggtacatgtcaaatatcaccttgggatcacttattctcagctgtattttggatgtataac
tgtttaaatgtagtaacattccactacttttggaagatgcaatcagatgtttggggcttt
gtatcgatccaaaagcacatatctcactacagccaaggtgattttagtgtcaactctatt
accatcaatggttggttgagaaacttattatggtccgaggcatcccaagtaatccaatcc
tatgctctttcatccatttgtccatatgggttcattttcctcatagctcatttcatctgg
gcatttagtttaatgttcctctttagtggtagagcatactggcaagaattaattgagtcc
attctatggtcacatcataaattaaagattatacctcatatccaaccaagggcattaagc
atttctcaaggaagagcagttggtttcattcattacactctaggaggtatcggctcaacc
tgggcatttattatttcaagactgctagtattaacttcttga
>MzC1GaII_psaA
atgcacatcttcagatatatcaacacaacactgtggtccaaagcaggacattttaataaa
gctttatcaaaaggagctaagacaactacttggatatggaatctacatgattatgctcat
gattttgacattcaacaaagatccacaggtttaatagcaagaaaagtcttctcatccaat
ctagctcatctatctttggtattcttttggattagtggaatgcaccttcatggggcatat
ttgtctaattatgatatttggttaaaagatcccaaatccatcaccccatcatcacatcta
gcctactctttaattggtcaagatattcttaactcatatacctcagaatacttctcagga
atcactatcacatctggctttttccaactctaccgttctgagggtatcattacacagtcc
cagctgaaatatgcatgtgctacttccctaatagctacacttatctgcctttctggatct
tatctccacatgcagcttatgtccaaattcactagcttttacaagaagttccagtcctta
tcgcaagatcatctcataattatatttggctccagctccacatccctctccgctcatcag
atacacaaaatgcttccagctaaccctttactagattcaggtatctccaacccaagtatc
ctacaagtaatctccaacagtctcagtgaaaacctagcactgttttcaacaaatctaagc
tctacaggtaaattgttaaatccctcaacaagaagtgtattcctttctcaagttgcagca
catcataagacaacaggtgtagtctttatcacactagggcttatcagattcctcaccatg
tataactctcaatttagtatacttacatcttatatagattaccacattgtgctatctatt
aatttagcattaattgcatctttatcaataatagtagctgatcatctcacaagaacccca
atctatcctcacaactcaacctcataccctacaatcctttgcctctccatacatcatgca
tggttatccggtttcctcatcattggttcaggagctcatgcatcaatattcaacttgcga
gcatcaccaacatctgagataagacatcgagaccccatatactcacacctcatctgggta
tgtatagcaataggattacactcattcagtctctactgtcataacgacactttagaagca
ttaggacgtccagaagatatctttcatgacaactctatccagttaaaagcaatatttgcc

aagcaatcctttctaagagcagagctccagccagatatcgagatgttagataaaaagatt
atacgtataacccaagaattgggaacagcagattttatagttcaccacattcatgcattt
acaatccatgttactctcttaattctttcaaagggtgtactttatgctagaaactctaga
tttgtatcagataaattggaacttggttttacttatccctgtgatggtccaggtagaggt
ggtacatgtcaaatatcaccttgggatcacttattctcagctgtattttggatgtataac
tgtttaaatgtagtaacattccactacttttggaagatgcaatcagatgtttggggcttt
gtatcgatccaaaagcacatatctcactacagccaaggtgattttagtgtcaactctatt
accatcaatggttggttgagaaacttattatggtccgaggcatcccaagtaatccaatcc
tatgctctttcatccatttgtccatatgggttcattttcctcatagctcatttcatctgg
gcatttagtttaatgttcctctttagtggtagagcatactggcaagaattaattgagtcc
attctatggtcacatcataaattaaagattatacctcatatccaaccaagggcattaagc
atttctcaaggaagagcagttggtttcattcattacactctaggaggtatcggctcaacc
tgggcatttattatttcaagactgctagtattaacttcttga
>MzC2_psaA
atgcacatcttcagatatatcaacacaacactgtggtccaaagcaggacattttaataaa
gctttatcaaaaggagctaagacaactacttggatatggaatctacatgattatgctcat
gattttgacattcaacaaagatccacaggtttaatagcaagaaaagtcttctcatccaat
ctagctcatctatctttggtattcttttggattagtggaatgcaccttcatggggcatat
ttgtctaattatgatatttggttaaaagatcccaaatccatcaccccatcatcacatcta
gcctactctttaattggtcaagatattcttaactcatatacctcagaatacttctcagga
atcactatcacatctggctttttccaactctaccgttctgagggtatcattacacagtcc
cagctgaaatatgcatgtgctacttccctaatagctacacttatctgcctttctggatct
tatctccacatgcagcttatgtccaaattcactagcttttacaagaagttccagtcctta
tcgcaagatcatctcataattatatttggctccagctccacatccctctccgctcatcag
atacacaaaatgcttccagctaaccctttactagattcaggtatctccaagccaagtatc
ctacaagtaatctccaacagtctcagtgaaaacctagcactgttttcaacaaatctaagc
tctacaggtaaattgttaaatccctcaacaagaagtgtattcctttctcaagttgcagca
catcataagacaacaggtgtagtctttatcacactagggcttatcagattcctcaccatg
tataactctcaatttagtatacttacatcttatatagattaccacattgtgctatctatt
aatttagcattaattgcatctttatcaataatagtagctgatcatctcacaagaacccca
atctatcctcacaactcaacctcataccctacaatcctttgcctctccatacatcatgca
tggttatccggtttcctcatcattggttcaggagctcatgcatcaatattcaacttgcga
gcatcaccaacatctgagataagacatcgagaccccatatactcacacctcatctgggta
tgtatagcaataggattacactcattcagtctctactgtcataacgacactttagaagca
ttaggacgtccagaagatatctttcatgacaactctatccagttaaaagcaatatttgcc
aagcaatcctttctaagagcagagctccagccagatatcgagatgttagataaaaagatt
atacgtataacccaagaattgggaacagcagattttatagttcaccacattcatgcattt
acaatccatgttactctcttaattctttcaaagggtgtactttatgctagaaactctaga
tttgtatcagataaattggaacttggttttacttatccctgtgatggtccaggtagaggt
ggtacatgtcaaatatcaccttgggatcacttattctcagctgtattttggatgtataac
tgtttaaatgtagtaacattccactacttttggaagatgcaatcagatgtttggggcttt
gtatcgatccaaaagcacatatctcactacagccaaggtgattttagtgtcaactctatt
accatcaatggttggttgagaaacttattatggtccgaggcatcccaagtaatccaatcc
tatgctctttcatccatttgtccatatgggttcattttcctcatagctcatttcatctgg
gcatttagtttaatgttcctctttagtggtagagcatactggcaagaattaattgagtcc
attctatggtcacatcataaattaaagattatacctcatatccaaccaagggcattaagc
atttctcaaggaagagcagttggtttcattcattacactctaggaggtatcggctcaacc
tgggcatttattatttcaagactgctagtattaacttcttga
>MzC2GaII_psaA
atgcacatcttcagatatatcaacacaacactgtggtccaaagcaggacattttaataaa
gctttatcaaaaggagctaagacaactacttggatatggaatctacatgattatgctcat
gattttgacattcaacaaagatccacaggtttaatagcaagaaaagtcttctcatccaat
ctagctcatctatctttggtattcttttggattagtggaatgcaccttcatggggcatat
ttgtctaattatgatatttggttaaaagatcccaaatccatcaccccatcatcacatcta
gcctactctttaattggtcaagatattcttaactcatatacctcagaatacttctcagga
atcactatcacatctggctttttccaactctaccgttctgagggtatcattacacagtcc
cagctgaaatatgcatgtgctacttccctaatagctacacttatctgcctttctggatct

tatctccacatgcagcttatgtccaaattcactagcttttacaagaagttccagtcctta
tcgcaagatcatctcataattatatttggctccagctccacatccctctccgctcatcag
atacacaaaatgcttccagctaacccttttactagattcaggtatctccaacccaagtatc
ctacaagtaatctccaacagtctcagtgaaaacctagcactgttttcaacaaatctaagc
tctacaggtaaattgttaaatccctcaacaagaagtgtattcctttctcaagttgcagca
catcataagacaacaggtgtagtctttatcacactagggcttatcagattcctcaccatg
tataactctcaatttagtatacttacatcttatatagattaccacattgtgctatctatt
aatttagcattaattgcatctttatcaataatagtagctgatcatctcacaagaacccca
atctatcctcacaactcaacctcataccctacaatcctttgcctctccatacatcatgca
tggttatccggtttcctcatcattggttcaggagctcatgcatcaatattcaacttgcga
gcatcaccaacatctgagataagacatcgagaccccatatactcacacctcatctgggta
tgtatagcaataggattacactcattcagtctctactgtcataacgacactttagaagca
ttaggacgtccagaagatatctttcatgacaactctatccagttaaaagcaatatttgcc
aagcaatcctttctaagagcagagctccagccagatatcgagatgttagataaaaagatt
atacgtataacccaagaattgggaacagcagattttatagttcaccacattcatgcattt
acaatccatgttactctcttaattctttcaaagggtgtactttatgctagaaactctaga
tttgtatcagataaattggaacttggttttacttatccctgtgatggtccaggtagaggt
ggtacatgtcaaatatcaccttgggatcacttattctcagctgtattttggatgtataac
tgtttaaatgtagtaacattccactacttttggaagatgcaatcagatgtttggggcttt
gtatcgatccaaaagcacatatctcactacagccaaggtgattttagtgtcaactctatt
accatcaatggttggttgagaaacttattatggtccgaggcatcccaagtaatccaatcc
tatgctctttcatccatttgtccatatgggttcattttcctcatagctcatttcatctgg
gcatttagtttaatgttcctctttagtggtagagcatactggcaagaattaattgagtcc
attctatggtcacatcataaattaaagattatacctcatatccaaccaagggcattaagc
atttctcaaggaagagcagttggtttcattcattacactctaggaggtatcggctcaacc
tgggcatttattatttcaagactgctagtattaacttcttga
>MzC3_psaA
atgcacatcttcagatatatcaacacaacactgtggtccaaagcaggacattttaataaa
gctttatcaaaaggagctaagacaactacttggatatggaatctacatgattatgctcat
gattttgacattcaacaaagatccacaggtttaatagcaagaaaagtcttctcatccaat
ctagctcatctatctttggtattcttttggattagtggaatgcaccttcatggggcatat
ttgtctaattatgatatttggttaaaagatcccaaatccatcaccccatcatcacatcta
gcctactctttaattggtcaagatattcttaactcatatacctcagaatacttctcagga
atcactatcacatctggctttttccaactctaccgttctgagggtatcattacacagtcc
cagctgaaatatgcatgtgctacttccctaatagctacacttatctgcctttctggatct
tatctccacatgcagcttatgtccaaattcactagcttttacaagaagttccagtcctta
tcgcaagatcatctcataattatatttggctccagctccacatccctctccgctcatcag
atacacaaaatgcttccagctaacccttttactagattcaggtatctccaacccaagtatc
ctacaagtaatctccaacagtctcagtgaaaacctagcactgttttcaacaaatctaagc
tctacaggtaaattgttaaatccctcaacaagaagtgtattcctttctcaagttgcagca
catcataagacaacaggtgtagtctttatcacactagggcttatcagattcctcaccatg
tataactctcaatttagtatacttacatcttatatagattaccacattgtgctatctatt
aatttagcattaattgcatctttatcaataatagtagctgatcatctcacaagaacccca
atctatcctcacaactcaacctcataccctacaatcctttgcctctccatacatcatgca
tggttatccggtttcctcatcattggttcaggagctcatgcatcaatattcaacttgcga
gcatcaccaacatctgagataagacatcgagaccccatatactcacacctcatctgggta
tgtatagcaataggattacactcattcagtctctactgtcataacgacactttagaagca
ttaggacgtccagaagatatctttcatgacaactctatccagttaaaagcaatatttgcc
aagcaatcctttctaagagcagagctccagccagatatcgagatgttagataaaaagatt
atacgtataacccaagaattgggaacagcagattttatagttcaccacattcatgcattt
acaatccatgttactctcttaattctttcaaagggtgtactttatgctagaaactctaga
tttgtatcagataaattggaacttggttttacttatccctgtgatggtccaggtagaggt
ggtacatgtcaaatatcaccttgggatcacttattctcagctgtattttggatgtataac
tgtttaaatgtagtaacattccactacttttggaagatgcaatcagatgtttggggcttt
gtatcgatccaaaagcacatatctcactacagccaaggtgattttagtgtcaactctatt
accatcaatggttggttgagaaacttattatggtccgaggcatcccaagtaatccaatcc
tatgctctttcatccatttgtccatatgggttcattttcctcatagctcatttcatctgg

gcatttagtttaatgttcctctttagtggtagagcatactggcaagaattaattgagtcc
attctatggtcacatcataaattaaagattatacctcatatccaaccaagggcattaagc
atttctcaaggaagagcagttggtttcattcattacactctaggaggtatcggctcaacc
tgggcatttattatttcaagactgctagtattaacttcttga
>MzC4_psaA
atgaacatcttcagatatatcaacacaacactgtggtccaaagcaggacattttaataaa
gctttatcaaaaggagctaagacaactacttggatatggaatctacatgattatgctcat
gattttgacattcaacaaagatccacaggtttaatagcaagaaaagtcttctcatccaat
ctagctcatctatctttggtattcttttggattagtggaatgcaccttcatggggcatat
ttgtctaattatgatatttggttaaaagatcccaaatccatcaccccatcatcacatcta
gcctactctttaattggtcaagatattcttaactcatatacctcagaatacttctcagga
atcactatcacatctggctttttccaactctaccgttctgagggtatcattacacagtcc
cagctgaaatatgcatgtgctacttccctaatagctacacttatctgcctttctggatct
tatctccacatgcagcttatgtccaaattcactagcttttacaagaagttccagtcctta
tcgcaagatcatctcataattatatttggctccagctccacatccctctccgctcatcag
atacacaaaatgcttccagctaaccctttactagattcaggtatctccaacccaagtatc
ctacaagtaatctccaacagtctcagtgaaaacctagcactgttttcaacaaatctaagc
tctacaggtaaattgttaaatccctcaacaagaagtgtattcctttctcaagttgcagca
catcataagacaacaggtgtagtctttatcacactagggcttatcagattcctcaccatg
tataactctcaatttagtatacttacatcttatatagattaccacattgtgctatctatt
aatttagcattaattgcatctttatcaataatagtagctgatcatctcacaagaacccca
atctatcctcacaactcaacctcataccctacaatcctttgcctctccatacatcatgca
tggttatccggtttcctcatcattggttcaggagctcatgcatcaatattcaacttgcga
gcatcaccaacatctgagataagacatcgagaccccatatactcacacctcatctgggta
tgtatagcaataggattacactcattcagtctctactgtcataacgacactttagaagca
ttaggacgtccagaagatatctttcatgacaactctatccagttaaaagcaatatttgcc
aagcaatcctttctaagagcagagctccagccagatatcgagatgttagataaaaagatt
atacgtataacccaagaattgggaacagcagattttatagttcaccacattcatgcattt
acaatccatgttactctcttaattctttcaaagggtgtactttatgctagaaactctaga
tttgtatcagataaattggaacttggttttacttatccctgtgatggtccaggtagaggt
ggtacatgtcaaatatcaccttgggatcacttattctcagctgtattttggatgtataac
tgtttaaatgtagtaacattccactacttttggaagatgcaatcagatgtttggggcttt
gtatcgatccaaaagcacatatctcactacagccaaggtgattttagtgtcaactctatt
accatcaatggttggttgagaaacttattatggtccgaggcatcccaagtaatccaatcc
tatgctctttcatccatttgtccatatgggttcattttcctcatagctcatttcatctgg
gcatttagtttaatgttcctctttagtggtagagcatactggcaagaattaattgagtcc
attctatggtcacatcataaattaaagattatacctcatatccaaccaagggcattaagc
atttctcaaggaagagcagttggtttcattcattacactctaggaggtatcggctcaacc
tgggcatttattatttcaagactgctagtattaacttcttga
>Od10_psaA
atgcacatcttcagatatatcaacacaacactgtggtccaaagcaggacattttaataaa
gctttatcaaaaggagctaagacaactacttggatatggaatctacatgattatgctcat
gattttgacattcaacaaagatccacaggtttaatagcaagaaaagtcttctcatccaat
ctagctcatctatctttggtattcttttggattagtggaatgcaccttcatggggcatat
ttgtctaattatgatatttggttaaaagatcccaaatccatcaccccatcatcacatcta
gcctactctttaattggtcaagatattcttaactcatatacctcagaatacttctcagga
atcactatcacatctggctttttccaactctaccgttctgagggtatcattacacagtcc
cagctgaaatatgcatgtgctacttccctaatagctacacttatctgcctttctggatct
tatctccacatgcagcttatgtccaaattcactagcttttacaagaagttccagtcctta
tcgcaagatcatctcataattatatttggctccagctccacatccctctccgctcatcag
atacacaaaatgcttccagctaaccctttactagattcaggtatctccaacccaagtatc
ctacaagtaatctccaacagtctcagtgaaaacctagcactgttttcaacaaatctaagc
tctacaggtaaattgttaaatccctcaacaagaagtgtattcctttctcaagttgcagca
catcataagacaacaggtgtagtctttatcacactagggcttatcagattcctcaccatg
tataactctcaatttagtatacttacatcttatatagattaccacattgtgctatctatt
aatttagcattaattgcatctttatcaataatagtagctgatcatctcacaagaacccca
atctatcctcacaactcaacctcataccctacaatcctttgcctctccatacatcatgca

tggttatccggtttcctcatcattggttcaggagctcatgcatcaatattcaacttgcga
gcatcaccaacatctgagataagacatcgagaccccatatactcacacctcatctgggta
tgtatagcaataggattacactcattcagtctctactgtcataacgacactttagaagca
ttaggacgtccagaagatatctttcatgacaactctatccagttaaaagcaatatttgcc
aagcaatcctttctaagagcagagctccagccagatatcgagatgttagataaaaagatt
atacgtataacccaagaattgggaacagcagattttatagttcaccacattcatgcattt
acaatccatgttactctcttaattctttcaaagggtgtactttatgctagaaactctaga
tttgtatcagataaattggaacttggttttacttatccctgtgatggtccaggtagaggt
ggtacatgtcaaatatcaccttgggatcacttattctcagctgtattttggatgtataac
tgtttaaatgtagtaacattccactacttttggaagatgcaatcagatgtttggggcttt
gtatcgatccaaaagcacatatctcactacagccaaggtgattttagtgtcaactctatt
accatcaatggttggttgagaaacttattatggtccgaggcatcccaagtaatccaatcc
tatgctctttcatccatttgtccatatgggttcattttcctcatagctcatttcatctgg
gcatttagtttaatgttcctctttagtggtagagcatactggcaagaattaattgagtcc
attctatggtcacatcataaattaaagattatacctcatatccaaccaagggcattaagc
atttctcaaggaagagcagttggtttcattcattacactctaggaggtatcggctcaacc
tgggcatttattatttcaagactgctagtattaacttcttga
>Od11_psaA
atgcacatcttcagatatatcaacacaacactgtggtccaaagcaggacattttaataaa
gctttatcaaaaggagctaagacaactacttggatatggaatctacatgattatgctcat
gattttgacattcaacaaagatccacaggtttaatagcaagaaaagtcttctcatccaat
ctagctcatctatctttggtattcttttggattagtggaatgcaccttcatggggcatat
ttgtctaattatgatatttggttaaaagatcccaaatccatcaccccatcatcacatcta
gcctactctttaattggtcaagatattcttaactcatatacctcagaatacttctcagga
atcactatcacatctggctttttccaactctaccgttctgagggtatcattacacagtcc
cagctgaaatatgcatgtgctacttccctaatagctacacttatctgcctttctggatct
tatctccacatgcagcttatgtccaaattcactagcttttacaagaagttccagtcctta
tcgcaagatcatctcataattatatttggctccagctccacatccctctccgctcatcag
atacacaaaatgcttccagctaaccctttactagattcaggtatctccaacccaagtatc
ctacaagtaatctccaacagtctcagtgaaaacctagcactgttttcaacaaatctaagc
tctacaggtaaattgttaaatccctcaacaagaagtgtattcctttctcaagttgcagca
catcataagacaacaggtgtagtctttatcacactagggcttatcagattcctcaccatg
tataactctcaatttagtatacttacatcttatatagattaccacattgtgctatctatt
aatttagcattaattgcatctttatcaataatagtagctgatcatctcacaagaacccca
atctatcctcacaactcaacctcataccctacaatcctttgcctctccatacatcatgca
tggttatccggtttcctcatcattggttcaggagctcatgcatcaatattcaacttgcga
gcatcaccaacatctgagataagacatcgagaccccatatactcacacctcatctgggta
tgtatagcaataggattacactcattcagtctctactgtcataacgacactttagaagca
ttaggacgtccagaagatatctttcatgacaactctatccagttaaaagcaatatttgcc
aagcaatcctttctaagagcagagctccagccagatatcgagatgttagataaaaagatt
atacgtataacccaagaattgggaacagcagattttatagttcaccacattcatgcattt
acaatccatgttactctcttaattctttcaaagggtgtactttatgctagaaactctaga
tttgtatcagataaattggaacttggttttacttatccctgtgatggtccaggtagaggt
ggtacatgtcaaatatcaccttgggatcacttattctcagctgtattttggatgtataac
tgtttaaatgtagtaacattccactacttttggaagatgcaatcagatgtttggggcttt
gtatcgatccaaaagcacatatctcactacagccaaggtgattttagtgtcaactctatt
accatcaatggttggttgagaaacttattatggtccgaggcatcccaagtaatccaatcc
tatgctctttcatccatttgtccatatgggttcattttcctcatagctcatttcatctgg
gcatttagtttaatgttcctctttagtggtagagcatactggcaagaattaattgagtcc
attctatggtcacatcataaattaaagattatacctcatatccaaccaagggcattaagc
atttctcaaggaagagcagttggtttcattcattacactctaggaggtatcggctcaacc
tgggcatttattatttcaagactgctagtattaacttcttga
>Od12_psaA
atgcacatcttcagatatatcaacacaacactgtggtccaaagcaggacattttaataaa
gctttatcaaaaggagctaagacaactacttggatatggaatctacatgattatgctcat
gattttgacattcaacaaagatccacaggtttaatagcaagaaaagtcttctcatccaat
ctagctcatctatctttggtattcttttggattagtggaatgcaccttcatggggcatat

ttgtctaattatgatatttggttaaaagatcccaaatccatcaccccatcatcacatcta
gcctactctttaattggtcaagatattcttaactcatatacctcagaatacttctcagga
atcactatcacatctggctttttccaactctaccgttctgagggtatcattacacagtcc
cagctgaaatatgcatgtgctacttccctaatagctacacttatctgcctttctggatct
tatctccacatgcagcttatgtccaaattcactagcttttacaagaagttccagtcctta
tcgcaagatcatctcataattatatttggctccagctccacatccctctccgctcatcag
atacacaaaatgcttccagctaaccctttactagattcaggtatctccaacccaagtatc
ctacaagtaatctccaacagtctcagtgaaaacctagcactgttttcaacaaatctaagc
tctacaggtaaattgttaaatccctcaacaagaagtgtattcctttctcaagttgcagca
catcataagacaacaggtgtagtctttatcacactagggcttatcagattcctcaccatg
tataactctcaatttagtatacttacatcttatatagattaccacattgtgctatctatt
aatttagcattaattgcatctttatcaataatagtagctgatcatctcacaagaacccca
atctatcctcacaactcaacctcataccctacaatcctttgcctctccatacatcatgca
tggttatccggtttcctcatcattggttcaggagctcatgcatcaatattcaacttgcga
gcatcaccaacatctgagataagacatcgagaccccatatactcacacctcatctgggta
tgtatagcaataggattacactcattcagtctctactgtcataacgacactttagaagca
ttaggacgtccagaagatatctttcatgacaactctatccagttaaaagcaatatttgcc
aagcaatcctttctaagagcagagctccagccagatatcgagatgttagataaaaagatt
atacgtataacccaagaattgggaacagcagattttatagttcaccacattcatgcattt
acaatccatgttactctcttaattctttcaaagggtgtactttatgctagaaactctaga
tttgtatcagataaattggaacttggttttacttatccctgtgatggtccaggtagaggt
ggtacatgtcaaatatcaccttgggatcacttattctcagctgtattttggatgtataac
tgtttaaatgtagtaacattccactacttttggaagatgcaatcagatgtttggggcttt
gtatcgatccaaaagcacatatctcactacagccaaggtgattttagtgtcaactctatt
accatcaatggttggttgagaaacttattatggtccgaggcatcccaagtaatccaatcc
tatgctctttcatccatttgtccatatgggttcattttcctcatagctcatttcatctgg
gcatttagtttaatgttcctctttagtggtagagcatactggcaagaattaattgagtcc
attctatggtcacatcataaattaaagattatacctcatatccaaccaagggcattaagc
atttctcaaggaagagcagttggtttcattcattacactctaggaggtatcggctcaacc
tgggcatttattatttcaagactgctagtattaacttcttga
>Od13_psaA
atgcacatcttcagatatatcaacacaacactgtggtccaaagcaggacattttaataaa
gctttatcaaaaggagctaagacaactacttggatatggaatctacatgattatgctcat
gattttgacattcaacaaagatccacaggtttaatagcaagaaaagtcttctcatccaat
ctagctcatctatctttggtattcttttggattagtggaatgcaccttcatggggcatat
ttgtctaattatgatatttggttaaaagatcccaaatccatcaccccatcatcacatcta
gcctactctttaattggtcaagatattcttaactcatatacctcagaatacttctcagga
atcactatcacatctggctttttccaactctaccgttctgagggtatcattacacagtcc
cagctgaaatatgcatgtgctacttccctaatagctacacttatctgcctttctggatct
tatctccacatgcagcttatgtccaaattcactagcttttacaagaagttccagtcctta
tcgcaagatcatctcataattatatttggctccagctccacatccctctccgctcatcag
atacacaaaatgcttccagctaaccctttactagattcaggtatctccaacccaagtatc
ctacaagtaatctccaacagtctcagtgaaaacctagcactgttttcaacaaatctaagc
tctacaggtaaattgttaaatccctcaacaagaagtgtattcctttctcaagttgcagca
catcataagacaacaggtgtagtctttatcacactagggcttatcagattcctcaccatg
tataactctcaatttagtatacttacatcttatatagattaccacattgtgctatctatt
aatttagcattaattgcatctttatcaataatagtagctgatcatctcacaagaacccca
atctatcctcacaactcaacctcataccctacaatcctttgcctctccatacatcatgca
tggttatccggtttcctcatcattggttcaggagctcatgcatcaatattcaacttgcga
gcatcaccaacatctgagataagacatcgagaccccatatactcacacctcatctgggta
tgtatagcaataggattacactcattcagtctctactgtcataacgacactttagaagca
ttaggacgtccagaagatatctttcatgacaactctatccagttaaaagcaatatttgcc
aagcaatcctttctaagagcagagctccagccagatatcgagatgttagataaaaagatt
atacgtataacccaagaattgggaacagcagattttatagttcaccacattcatgcattt
acaatccatgttactctcttaattctttcaaagggtgtactttatgctagaaactctaga
tttgtatcagataaattggaacttggttttacttatccctgtgatggtccaggtagaggt
ggtacatgtcaaatatcaccttgggatcacttattctcagctgtattttggatgtataac

tgtttaaatgtagtaacattccactacttttggaagatgcaatcagatgtttggggcttt
gtatcgatccaaaagcacatatctcactacagccaaggtgattttagtgtcaactctatt
accatcaatggttggttgagaaacttattatggtccgaggcatcccaagtaatccaatcc
tatgctctttcatccatttgtccatatgggttcattttcctcatagctcatttcatctgg
gcatttagtttaatgttcctctttagtggtagagcatactggcaagaattaattgagtcc
attctatggtcacatcataaattaaagattatacctcatatccaaccaagggcattaagc
atttctcaaggaagagcagttggtttcattcattacactctaggaggtatcggctcaacc
tgggcatttattatttcaagactgctagtattaacttcttga
>Od14_psaA
atgcacatcttcagatatatcaacacaacactgtggtccaaagcaggacattttaataaa
gctttatcaaaaggagctaagacaactacttggatatggaatctacatgattatgctcat
gattttgacattcaacaaagatccacaggtttaatagcaagaaaagtcttctcatccaat
ctagctcatctatctttggtattcttttggattagtggaatgcaccttcatggggcatat
ttgtctaattatgatatttggttaaaagatcccaaatccatcaccccatcatcacatcta
gcctactctttaattggtcaagatattcttaactcatatacctcagaatacttctcagga
atcactatcacatctggctttttccaactctaccgttctgagggtatcattacacagtcc
cagctgaaatatgcatgtgctacttccctaatagctacacttatctgcctttctggatct
tatctccacatgcagcttatgtccaaattcactagcttttacaagaagttccagtcctta
tcgcaagatcatctcataattatatttggctccagctccacatccctctccgctcatcag
atacacaaaatgcttccagctaaccctttactagattcaggtatctccaacccaagtatc
ctacaagtaatctccaacagtctcagtgaaaacctagcactgttttcaacaaatctaagc
tctacaggtaaattgttaaatccctcaacaagaagtgtattcctttctcaagttgcagca
catcataagacaacaggtgtagtctttatcacactagggcttatcagattcctcaccatg
tataactctcaatttagtatacttacatcttatatagattaccacattgtgctatctatt
aatttagcattaattgcatctttatcaataatagtagctgatcatctcacaagaacccca
atctatcctcacaactcaacctcataccctacaatcctttgcctctccatacatcatgca
tggttatccggtttcctcatcattggttcaggagctcatgcatcaatattcaacttgcga
gcatcaccaacatctgagataagacatcgagaccccatatactcacacctcatctgggta
tgtatagcaataggattacactcattcagtctctactgtcataacgacactttagaagca
ttaggacgtccagaagatatctttcatgacaactctatccagttaaaagcaatatttgcc
aagcaatcctttctaagagcagagctccagccagatatcgagatgttagataaaaagatt
atacgtataacccaagaattgggaacagcagattttatagttcaccacattcatgcattt
acaatccatgttactctcttaattctttcaaagggtgtactttatgctagaaactctaga
tttgtatcagataaattggaacttggttttacttatccctgtgatggtccaggtagaggt
ggtacatgtcaaatatcaccttgggatcacttattctcagctgtattttggatgtataac
tgtttaaatgtagtaacattccactacttttggaagatgcaatcagatgtttggggcttt
gtatcgatccaaaagcacatatctcactacagccaaggtgattttagtgtcaactctatt
accatcaatggttggttgagaaacttattatggtccgaggcatcccaagtaatccaatcc
tatgctctttcatccatttgtccatatgggttcattttcctcatagctcatttcatctgg
gcatttagtttaatgttcctctttagtggtagagcatactggcaagaattaattgagtcc
attctatggtcacatcataaattaaagattatacctcatatccaaccaagggcattaagc
atttctcaaggaagagcagttggtttcattcattacactctaggaggtatcggctcaacc
tgggcatttattatttcaagactgctagtattaacttcttga
>Od15_psaA
atgcacatcttcagatatatcaacacaacactgtggtccaaagcaggacattttaataaa
gctttatcaaaaggagctaagacaactacttggatatggaatctacatgattatgctcat
gattttgacattcaacaaagatccacaggtttaatagcaagaaaagtcttctcatccaat
ctagctcatctatctttggtattcttttggattagtggaatgcaccttcatggggcatat
ttgtctaattatgatatttggttaaaagatcccaaatccatcaccccatcatcacatcta
gcctactctttaattggtcaagatattcttaactcatatacctcagaatacttctcagga
atcactatcacatctggctttttccaactctaccgttctgagggtatcattacacagtcc
cagctgaaatatgcatgtgctacttccctaatagctacacttatctgcctttctggatct
tatctccacatgcagcttatgtccaaattcactagcttttacaagaagttccagtcctta
tcgcaagatcatctcataattatatttggctccagctccacatccctctccgctcatcag
atacacaaaatgcttccagctaaccctttactagattcaggtatctccaacccaagtatc
ctacaagtaatctccaacagtctcagtgaaaacctagcactgttttcaacaaatctaagc
tctacaggtaaattgttaaatccctcaacaagaagtgtattcctttctcaagttgcagca

catcataagacaacaggtgtagtctttatcacactagggcttatcagattcctcaccatg
tataactctcaatttagtatacttacatcttatatagattaccacattgtgctatctatt
aatttagcattaattgcatctttatcaataatagtagctgatcatctcacaagaacccca
atctatcctcacaactcaacctcataccctacaatcctttgcctctccatacatcatgca
tggttatccggtttcctcatcattggttcaggagctcatgcatcaatattcaacttgcga
gcatcaccaacatctgagataagacatcgagaccccatatactcacacctcatctgggta
tgtatagcaataggattacactcattcagtctctactgtcataacgacactttagaagca
ttaggacgtccagaagatatctttcatgacaactctatccagttaaaagcaatatttgcc
aagcaatcctttctaagagcagagctccagccagatatcgagatgttagataaaaagatt
atacgtataacccaagaattgggaacagcagattttatagttcaccacattcatgcattt
acaatccatgttactctcttaattctttcaaagggtgtactttatgctagaaactctaga
tttgtatcagataaattggaacttggttttacttatccctgtgatggtccaggtagaggt
ggtacatgtcaaatatcaccttgggatcacttattctcagctgtattttggatgtataac
tgtttaaatgtagtaacattccactacttttggaagatgcaatcagatgtttggggcttt
gtatcgatccaaaagcacatatctcactacagccaaggtgattttagtgtcaactctatt
accatcaatggttggttgagaaacttattatggtccgaggcatcccaagtaatccaatcc
tatgctctttcatccatttgtccatatgggttcattttcctcatagctcatttcatctgg
gcatttagtttaatgttcctctttagtggtagagcatactggcaagaattaattgagtcc
attctatggtcacatcataaattaaagattatacctcatatccaaccaagggcattaagc
atttctcaaggaagagcagttggtttcattcattacactctaggaggtatcggctcaacc
tgggcatttattatttcaagactgctagtattaacttcttga
>Od18_psaA
atgcacatcttcagatatatcaacacaacactgtggtccaaagcaggacattttaataaa
gctttatcaaaaggagctaagacaactacttggatatggaatctacatgattatgctcat
gattttgacattcaacaaagatccacaggtttaatagcaagaaaagtcttctcatccaat
ctagctcatctatctttggtattcttttggattagtggaatgcaccttcatggggcatat
ttgtctaattatgatatttggttaaaagatcccaaatccatcaccccatcatcacatcta
gcctactctttaattggtcaagatattcttaactcatatacctcagaatacttctcagga
atcactatcacatctggctttttccaactctaccgttctgagggtatcattacacagtcc
cagctgaaatatgcatgtgctacttccctaatagctacacttatctgcctttctggatct
tatctccacatgcagcttatgtccaaattcactagcttttacaagaagttccagtcctta
tcgcaagatcatctcataattatatttggctccagctccacatccctctccgctcatcag
atacacaaaatgcttccagctaaccctttactagattcaggtatctccaacccaagtatc
ctacaagtaatctccaacagtctcagtgaaaacctagcactgttttcaacaaatctaagc
tctacaggtaaattgttaaatccctcaacaagaagtgtattcctttctcaagttgcagca
catcataagacaacaggtgtagtctttatcacactagggcttatcagattcctcaccatg
tataactctcaatttagtatacttacatcttatatagattaccacattgtgctatctatt
aatttagcattaattgcatctttatcaataatagtagctgatcatctcacaagaacccca
atctatcctcacaactcaacctcataccctacaatcctttgcctctccatacatcatgca
tggttatccggtttcctcatcattggttcaggagctcatgcatcaatattcaacttgcga
gcatcaccaacatctgagataagacatcgagaccccatatactcacacctcatctgggta
tgtatagcaataggattacactcattcagtctctactgtcataacgacactttagaagca
ttaggacgtccagaagatatctttcatgacaactctatccagttaaaagcaatatttgcc
aagcaatcctttctaagagcagagctccagccagatatcgagatgttagataaaaagatt
atacgtataacccaagaattgggaacagcagattttatagttcaccacattcatgcattt
acaatccatgttactctcttaattctttcaaagggtgtactttatgctagaaactctaga
tttgtatcagataaattggaacttggttttacttatccctgtgatggtccaggtagaggt
ggtacatgtcaaatatcaccttgggatcacttattctcagctgtattttggatgtataac
tgtttaaatgtagtaacattccactacttttggaagatgcaatcagatgtttggggcttt
gtatcgatccaaaagcacatatctcactacagccaaggtgattttagtgtcaactctatt
accatcaatggttggttgagaaacttattatggtccgaggcatcccaagtaatccaatcc
tatgctctttcatccatttgtccatatgggttcattttcctcatagctcatttcatctgg
gcatttagtttaatgttcctctttagtggtagagcatactggcaagaattaattgagtcc
attctatggtcacatcataaattaaagattatacctcatatccaaccaagggcattaagc
atttctcaaggaagagcagttggtttcattcattacactctaggaggtatcggctcaacc
tgggcatttattatttcaagactgctagtattaacttcttga
>Od19_psaA

atgcacatcttcagatatatcaacacaacactgtggtccaaagcaggacattttaataaa
gctttatcaaaaggagctaagacaactacttggatatggaatctacatgattatgctcat
gattttgacattcaacaaagatccacaggtttaatagcaagaaaagtcttctcatccaat
ctagctcatctatctttggtattcttttggattagtggaatgcaccttcatggggcatat
ttgtctaattatgatatttggttaaaagatcccaaatccatcaccccatcatcacatcta
gcctactctttaattggtcaagatattcttaactcatatacctcagaatacttctcagga
atcactatcacatctggctttttccaactctaccgttctgagggtatcattacacagtcc
cagctgaaatatgcatgtgctacttccctaatagctacacttatctgcctttctggatct
tatctccacatgcagcttatgtccaaattcactagcttttacaagaagttccagtcctta
tcgcaagatcatctcataattatatttggctccagctccacatccctctccgctcatcag
atacacaaaatgcttccagctaacccttactagattcaggtatctccaacccaagtatc
ctacaagtaatctccaacagtctcagtgaaaacctagcactgttttcaacaaatctaagc
tctacaggtaaattgttaaatccctcaacaagaagtgtattcctttctcaagttgcagca
catcataagacaacaggtgtagtctttatcacactagggcttatcagattcctcaccatg
tataactctcaatttagtatacttacatcttatatagattaccacattgtgctatctatt
aatttagcattaattgcatctttatcaataatagtagctgatcatctcacaagaacccca
atctatcctcacaactcaacctcataccctacaatcctttgcctctccatacatcatgca
tggttatccggtttcctcatcattggttcaggagctcatgcatcaatattcaacttgcga
gcatcaccaacatctgagataagacatcgagaccccatatactcacacctcatctgggta
tgtatagcaataggattacactcattcagtctctactgtcataacgacactttagaagca
ttaggacgtccagaagatatctttcatgacaactctatccagttaaaagcaatatttgcc
aagcaatcctttctaagagcagagctccagccagatatcgagatgttagataaaaagatt
atacgtataacccaagaattgggaacagcagattttatagttcaccacattcatgcattt
acaatccatgttactctcttaattctttcaaagggtgtactttatgctagaaactctaga
tttgtatcagataaattggaacttggttttacttatccctgtgatggtccaggtagaggt
ggtacatgtcaaatatcaccttgggatcacttattctcagctgtattttggatgtataac
tgtttaaatgtagtaacattccactacttttggaagatgcaatcagatgtttggggcttt
gtatcgatccaaaagcacatatctcactacagccaaggtgattttagtgtcaactctatt
accatcaatggttggttgagaaacttattatggtccgaggcatcccaagtaatccaatcc
tatgctctttcatccatttgtccatatgggttcattttcctcatagctcatttcatctgg
gcatttagtttaatgttcctctttagtggtagagcatactggcaagaattaattgagtcc
attctatggtcacatcataaattaaagattatacctcatatccaaccaagggcattaagc
atttctcaaggaagagcagttggtttcattcattacactctaggaggtatcggctcaacc
tgggcatttattatttcaagactgctagtattaacttcttga
>Od2_psaA
atgcacatcttcagatatatcaacacaacactgtggtccaaagcaggacattttaataaa
gctttatcaaaaggagctaagacaactacttggatatggaatctacatgattatgctcat
gattttgacattcaacaaagatccacaggtttaatagcaagaaaagtcttctcatccaat
ctagctcatctatctttggtattcttttggattagtggaatgcaccttcatggggcatat
ttgtctaattatgatatttggttaaaagatcccaaatccatcaccccatcatcacatcta
gcctactctttaattggtcaagatattcttaactcatatacctcagaatacttctcagga
atcactatcacatctggctttttccaactctaccgttctgagggtatcattacacagtcc
cagctgaaatatgcatgtgctacttccctaatagctacacttatctgcctttctggatct
tatctccacatgcagcttatgtccaaattcactagcttttacaagaagttccagtcctta
tcgcaagatcatctcataattatatttggctccagctccacatccctctccgctcatcag
atacacaaaatgcttccagctaacccttactagattcaggtatctccaacccaagtatc
ctacaagtaatctccaacagtctcagtgaaaacctagcactgttttcaacaaatctaagc
tctacaggtaaattgttaaatccctcaacaagaagtgtattcctttctcaagttgcagca
catcataagacaacaggtgtagtctttatcacactagggcttatcagattcctcaccatg
tataactctcaatttagtatacttacatcttatatagattaccacattgtgctatctatt
aatttagcattaattgcatctttatcaataatagtagctgatcatctcacaagaacccca
atctatcctcacaactcaacctcataccctacaatcctttgcctctccatacatcatgca
tggttatccggtttcctcatcattggttcaggagctcatgcatcaatattcaacttgcga
gcatcaccaacatctgagataagacatcgagaccccatatactcacacctcatctgggta
tgtatagcaataggattacactcattcagtctctactgtcataacgacactttagaagca
ttaggacgtccagaagatatctttcatgacaactctatccagttaaaagcaatatttgcc
aagcaatcctttctaagagcagagctccagccagatatcgagatgttagataaaaagatt

atacgtataacccaagaattgggaacagcagattttatagttcaccacattcatgcattt
acaatccatgttactctcttaattctttcaaagggtgtactttatgctagaaactctaga
tttgtatcagataaattggaacttggttttacttatccctgtgatggtccaggtagaggt
ggtacatgtcaaatatcaccttgggatcacttattctcagctgtattttggatgtataac
tgtttaaatgtagtaacattccactacttttggaagatgcaatcagatgtttggggcttt
gtatcgatccaaaagcacatatctcactacagccaaggtgattttagtgtcaactctatt
accatcaatggttggttgagaaacttattatggtccgaggcatcccaagtaatccaatcc
tatgctctttcatccatttgtccatatgggttcattttcctcatagctcatttcatctgg
gcatttagtttaatgttcctctttagtggtagagcatactggcaagaattaattgagtcc
attctatggtcacatcataaattaaagattatacctcatatccaaccaagggcattaagc
atttctcaaggaagagcagttggtttcattcattacactctaggaggtatcggctcaacc
tgggcatttattatttcaagactgctagtattaagttcttga
>Od22_psaA
atgcacatcttcagatatatcaacacaacactgtggtccaaagcaggacattttaataaa
gctttatcaaaaggagctaagacaactacttggatatggaatctacatgattatgctcat
gattttgacattcaacaaagatccacaggtttaatagcaagaaaagtcttctcatccaat
ctagctcatctatctttggtattcttttggattagtggaatgcaccttcatggggcatat
ttgtctaattatgatatttggttaaaagatcccaaatccatcaccccatcatcacatcta
gcctactctttaattggtcaagatattcttaactcatatacctcagaatacttctcagga
atcactatcacatctggctttttccaactctaccgttctgagggtatcattacacagtcc
cagctgaaatatgcatgtgctacttccctaatagctacacttatctgcctttctggatct
tatctccacatgcagcttatgtccaaattcactagcttttacaagaagttccagtcctta
tcgcaagatcatctcataattatatttggctccagctccacatccctctccgctcatcag
atacacaaaatgcttccagctaaccctttactagattcaggtatctccaacccaagtatc
ctacaagtaatctccaacagtctcagtgaaaacctagcactgttttcaacaaatctaagc
tctacaggtaaattgttaaatccctcaacaagaagtgtattcctttctcaagttgcagca
catcataagacaacaggtgtagtctttatcacactagggcttatcagattcctcaccatg
tataactctcaatttagtatacttacatcttatatagattaccacattgtgctatctatt
aatttagcattaattgcatctttatcaataatagtagctgatcatctcacaagaacccca
atctatcctcacaactcaacctcataccctacaatcctttgcctctccatacatcatgca
tggttatccggtttcctcatcattggttcaggagctcatgcatcaatattcaacttgcga
gcatcaccaacatctgagataagacatcgagaccccatatactcacacctcatctgggta
tgtatagcaataggattacactcattcagtctctactgtcataacgacactttagaagca
ttaggacgtccagaagatatctttcatgacaactctatccagttaaaagcaatatttgcc
aagcaatcctttctaagagcagagctccagccagatatcgagatgttagataaaaagatt
atacgtataacccaagaattgggaacagcagattttatagttcaccacattcatgcattt
acaatccatgttactctcttaattctttcaaagggtgtactttatgctagaaactctaga
tttgtatcagataaattggaacttggttttacttatccctgtgatggtccaggtagaggt
ggtacatgtcaaatatcaccttgggatcacttattctcagctgtattttggatgtataac
tgtttaaatgtagtaacattccactacttttggaagatgcaatcagatgtttggggcttt
gtatcgatccaaaagcacatatctcactacagccaaggtgattttagtgtcaactctatt
accatcaatggttggttgagaaacttattatggtccgaggcatcccaagtaatccaatcc
tatgctctttcatccatttgtccatatgggttcattttcctcatagctcatttcatctgg
gcatttagtttaatgttcctctttagtggtagagcatactggcaagaattaattgagtcc
attctatggtcacatcataaattaaagattatacctcatatccaaccaagggcattaagc
atttctcaaggaagagcagttggtttcattcattacactctaggaggtatcggctcaacc
tgggcatttattatttcaagactgctagtattaacttcttga
>Od23_psaA
atgcacatcttcagatatatcaacacaacactgtggtccaaagcaggacattttaataaa
gctttatcaaaaggagctaagacaactacttggatatggaatctacatgattatgctcat
gattttgacattcaacaaagatccacaggtttaatagcaagaaaagtcttctcatccaat
ctagctcatctatctttggtattcttttggattagtggaatgcaccttcatggggcatat
ttgtctaattatgatatttggttaaaagatcccaaatccatcaccccatcatcacatcta
gcctactctttaattggtcaagatattcttaactcatatacctcagaatacttctcagga
atcactatcacatctggctttttccaactctaccgttctgagggtatcattacacagtcc
cagctgaaatatgcatgtgctacttccctaatagctacacttatctgcctttctggatct
tatctccacatgcagcttatgtccaaattcactagcttttacaagaagttccagtcctta

tcgcaagatcatctcataattatatttggctccagctccacatccctctccgctcatcag
atacacaaaatgcttccagctaaccctttactagattcaggtatctccaacccaagtatc
ctacaagtaatctccaacagtctcagtgaaaacctagcactgttttcaacaaatctaagc
tctacaggtaaattgttaaatccctcaacaagaagtgtattcctttctcaagttgcagca
catcataagacaacaggtgtagtctttatcacactagggcttatcagattcctcaccatg
tataactctcaatttagtatacttacatcttatatagattaccacattgtgctatctatt
aatttagcattaattgcatctttatcaataatagtagctgatcatctcacaagaacccca
atctatcctcacaactcaacctcataccctacaatcctttgcctctccatacatcatgca
tggttatccggtttcctcatcattggttcaggagctcatgcatcaatattcaacttgcga
gcatcaccaacatctgagataagacatcgagaccccatatactcacacctcatctgggta
tgtatagcaataggattacactcattcagtctctactgtcataacgacactttagaagca
ttaggacgtccagaagatatctttcatgacaactctatccagttaaaagcaatatttgcc
aagcaatcctttctaagagcagagctccagccagatatcgagatgttagataaaaagatt
atacgtataacccaagaattgggaacagcagattttatagttcaccacattcatgcattt
acaatccatgttactctcttaattctttcaaagggtgtactttatgctagaaactctaga
tttgtatcagataaattggaacttggttttacttatccctgtgatggtccaggtagaggt
ggtacatgtcaaatatcaccttgggatcacttattctcagctgtattttggatgtataac
tgtttaaatgtagtaacattccactacttttggaagatgcaatcagatgtttggggcttt
gtatcgatccaaaagcacatatctcactacagccaaggtgattttagtgtcaactctatt
accatcaatggttggttgagaaacttattatggtccgaggcatcccaagtaatccaatcc
tatgctctttcatccatttgtccatatgggttcattttcctcatagctcatttcatctgg
gcatttagtttaatgttcctctttagtggtagagcatactggcaagaattaattgagtcc
attctatggtcacatcataaattaaagattatacctcatatccaaccaagggcattaagc
atttctcaaggaagagcagttggtttcattcattacactctaggaggtatcggctcaacc
tgggcatttattatttcaagactgctagtattaacttcttga
>Od24_psaA
atgcacatcttcagatatatcaacacaacactgtggtccaaagcaggacattttaataaa
gctttatcaaaaggagctaagacaactacttggatatggaatctacatgattatgctcat
gattttgacattcaacaaagatccacaggtttaatagcaagaaaagtcttctcatccaat
ctagctcatctatctttggtattcttttggattagtggaatgcaccttcatggagcatat
ttgtctaattatgatatttggttaaaagatcccaaatccatcaccccatcatcacatcta
gcctactctttaattggtcaagatattcttaactcatatacctcagaatacttctcagga
atcactatcacatctggctttttccaactctaccgttctgagggtatcattacacagtcc
cagctgaaatatgcatgtgctacttccctaatagctacacttatctgcctttctggatct
tatctccacatgcagcttatgtccaaattcactagcttttacaagaagttccagtcctta
tcgcaagatcatctcataattatatttggctccagctccacatccctctccgctcatcag
atacacaaaatgcttccagctaaccctttactagattcaggtatctccaagccaagtatc
ctacaagtaatctccaacagtctcagtgaaaacctagcactgttttcaacaaatctaagc
tctacaggtaaattgttaaatccctcaacaagaagtgtattcctttctcaagttgcagca
catcataagacaacaggtgtagtctttatcacactagggcttatcagattcctcaccatg
tataactctcaatttagtatacttacatcttatatagattaccacattgtgctatctatt
aatttagcattaattgcatctttatcaataatagtagctgatcatctcacaagaacccca
atctatcctcacaagtcaacctcataccctacaatcctttgcctctccatacatcatgca
tggttatccggtttcctcatcattggttcaggagctcatgcatcaatattcaacttgcga
gcatcaccaacatctgagataagacatcgagaccccatatactcacacctcatctgggta
tgtatagcaataggattacactcattcagtctctactgtcataacgacactttagaagca
ttaggacgtccagaagatatctttcatgacaactctatccagttaaaagcaatatttgcc
aagcaatcctttctaagagcagagctccagccagatatcgagatgttagataaaaagatt
atacgtataacccaagaattgggaacagcagattttatagttcaccacattcatgcattt
acaatccatgttactctcttaattctttcaaagggtgtactttatgctagaaactctaga
tttgtatcagataaattggaacttggttttacttatccctgtgatggtccaggtagaggt
ggtacatgtcaaatatcaccttgggatcacttattctcagctgtattttggatgtataac
tgtttaaatgtagtaacattccactacttttggaagatgcaatcagatgtttggggcttt
gtatcgatccaaaagcacatatctcactacagccaaggtgattttagtgtcaactctatt
accatcaatggttggttgagaaacttattatggtccgaggcatcccaagtaatccaatcc
tatgctctttcatccatttgtccatatgggttcattttcctcatagctcatttcatctgg
gcatttagtttaatgttcctctttagtggtagagcatactggcaagaattaattgagtcc

attctatggtcacatcataaattaaagattatacctcatatccaaccaagggcattaagc
atttctcaaggaagagcagttggtttcattcattacactctaggaggtatcggctcaacc
tgggcatttattatttcaagactgctagtattaacttcttga
>Od25_psaA
atgcacatcttcagatatatcaacacaacactgtggtccaaagcaggacattttaataaa
gctttatcaaaaggagctaagacaactacttggatatggaatctacatgattatgctcat
gattttgacattcaacaaagatccacaggtttaatagcaagaaaagtcttctcatccaat
ctagctcatctatctttggtattcttttggattagtggaatgcaccttcatggggcatat
ttgtctaattatgatatttggttaaaagatcccaaatccatcaccccatcatcacatcta
gcctactctttaattggtcaagatattcttaactcatatacctcagaatacttctcagga
atcactatcacatctggctttttccaactctaccgttctgagggtatcattacacagtcc
cagctgaaatatgcatgtgctacttccctaatagctacacttatctgcctttctggatct
tatctccacatgcagcttatgtccaaattcactagcttttacaagaagttccagtcctta
tcgcaagatcatctcataattatatttggctccagctccacatccctctccgctcatcag
atacacaaaatgcttccagctaaccctttactagattcaggtatctccaacccaagtatc
ctacaagtaatctccaacagtctcagtgaaaacctagcactgttttcaacaaatctaagc
tctacaggtaaattgttaaatccctcaacaagaagtgtattcctttctcaagttgcagca
catcataagacaacaggtgtagtctttatcacactagggcttatcagattcctcaccatg
tataactctcaatttagtatacttacatcttatatagattaccacattgtgctatctatt
aatttagcattaattgcatctttatcaataatagtagctgatcatctcacaagaacccca
atctatcctcacaactcaacctcataccctacaatcctttgcctctccatacatcatgca
tggttatccggtttcctcatcattggttcaggagctcatgcatcaatattcaacttgcga
gcatcaccaacatctgagataagacatcgagaccccatatactcacacctcatctgggta
tgtatagcaataggattacactcattcagtctctactgtcataacgacactttagaagca
ttaggacgtccagaagatatctttcatgacaactctatccagttaaaagcaatatttgcc
aagcaatcctttctaagagcagagctccagccagatatcgagatgttagataaaaagatt
atacgtataacccaagaattgggaacagcagattttatagttcaccacattcatgcattt
acaatccatgttactctcttaattctttcaaagggtgtactttatgctagaaactctaga
tttgtatcagataaattggaacttggttttacttatccctgtgatggtccaggtagaggt
ggtacatgtcaaatatcaccttgggatcacttattctcagctgtattttggatgtataac
tgtttaaatgtagtaacattccactacttttggaagatgcaatcagatgtttggggcttt
gtatcgatccaaaagcacatatctcactacagccaaggtgattttagtgtcaactctatt
accatcaatggttggttgagaaacttattatggtccgaggcatcccaagtaatccaatcc
tatgctctttcatccatttgtccatatgggttcattttcctcatagctcatttcatctgg
gcatttagtttaatgttcctctttagtggtagagcatactggcaagaattaattgagtcc
attctatggtcacatcataaattaaagattatacctcatatccaaccaagggcattaagc
atttctcaaggaagagcagttggtttcattcattacactctaggaggtatcggctcaacc
tgggcatttattatttcaagactgctagtattaacttcttga
>Od26_psaA
atgcacatcttcagatatatcaacacaacactgtggtccaaagcaggacattttaataaa
gctttatcaaaaggagctaagacaactacttggatatggaatctacatgattatgctcat
gattttgacattcaacaaagatccacaggtttaatagcaagaaaagtcttctcatccaat
ctagctcatctatctttggtattcttttggattagtggaatgcaccttcatggggcatat
ttgtctaattatgatatttggttaaaagatcccaaatccatcaccccatcatcacatcta
gcctactctttaattggtcaagatattcttaactcatatacctcagaatacttctcagga
atcactatcacatctggctttttccaactctaccgttctgagggtatcattacacagtcc
cagctgaaatatgcatgtgctacttccctaatagctacacttatctgcctttctggatct
tatctccacatgcagcttatgtccaaattcactagcttttacaagaagttccagtcctta
tcgcaagatcatctcataattatatttggctccagctccacatccctctccgctcatcag
atacacaaaatgcttccagctaaccctttactagattcaggtatctccaacccaagtatc
ctacaagtaatctccaacagtctcagtgaaaacctagcactgttttcaacaaatctaagc
tctacaggtaaattgttaaatccctcaacaagaagtgtattcctttctcaagttgcagca
catcataagacaacaggtgtagtctttatcacactagggcttatcagattcctcaccatg
tataactctcaatttagtatacttacatcttatatagattaccacattgtgctatctatt
aatttagcattaattgcatctttatcaataatagtagctgatcatctcacaagaacccca
atctatcctcacaactcaacctcataccctacaatcctttgcctctccatacatcatgca
tggttatccggtttcctcatcattggttcaggagctcatgcatcaatattcaacttgcga

gcatcaccaacatctgagataagacatcgagaccccatatactcacacctcatctgggta
tgtatagcaataggattacactcattcagtctctactgtcataacgacactttagaagca
ttaggacgtccagaagatatctttcatgacaactctatccagttaaaagcaatatttgcc
aagcaatcctttctaagagcagagctccagccagatatcgagatgttagataaaaagatt
atacgtataacccaagaattgggaacagcagattttatagttcaccacattcatgcattt
acaatccatgttactctcttaattctttcaaagggtgtactttatgctagaaactctaga
tttgtatcagataaattggaacttggttttacttatccctgtgatggtccaggtagaggt
ggtacatgtcaaatatcaccttgggatcacttattctcagctgtattttggatgtataac
tgtttaaatgtagtaacattccactacttttggaagatgcaatcagatgtttggggcttt
gtatcgatccaaaagcacatatctcactacagccaaggtgattttagtgtcaactctatt
accatcaatggttggttgagaaacttattatggtccgaggcatcccaagtaatccaatcc
tatgctctttcatccatttgtccatatgggttcattttcctcatagctcatttcatctgg
gcatttagtttaatgttcctctttagtggtagagcatactggcaagaattaattgagtcc
attctatggtcacatcataaattaaagattatacctcatatccaaccaagggcattaagc
atttctcaaggaagagcagttggtttcattcattacactctaggaggtatcggctcaacc
tgggcatttattatttcaagactgctagtattaacttcttga
>Od27_psaA
atgcacatcttcagatatatcaacacaacactgtggtccaaagcaggacattttaataaa
gctttatcaaaaggagctaagacaactacttggatatggaatctacatgattatgctcat
gattttgacattcaacaaagatccacaggtttaatagcaagaaaagtcttctcatccaat
ctagctcatctatctttggtattcttttggattagtggaatgcaccttcatggggcatat
ttgtctaattatgatatttggttaaaagatcccaaatccatcaccccatcatcacatcta
gcctactctttaattggtcaagatattcttaactcatatacctcagaatacttctcagga
atcactatcacatctggctttttccaactctaccgttctgagggtatcattacacagtcc
cagctgaaatatgcatgtgctacttccctaatagctacacttatctgcctttctggatct
tatctccacatgcagcttatgtccaaattcactagcttttacaagaagttccagtcctta
tcgcaagatcatctcataattatatttggctccagctccacatccctctccgctcatcag
atacacaaaatgcttccagctaaccctttactagattcaggtatctccaacccaagtatc
ctacaagtaatctccaacagtctcagtgaaaacctagcactgttttcaacaaatctaagc
tctacaggtaaattgttaaatccctcaacaagaagtgtattcctttctcaagttgcagca
catcataagacaacaggtgtagtctttatcacactagggcttatcagattcctcaccatg
tataactctcaatttagtatacttacatcttatatagattaccacattgtgctatctatt
aatttagcattaattgcatctttatcaataatagtagctgatcatctcacaagaacccca
atctatcctcacaactcaacctcataccctacaatcctttgcctctccatacatcatgca
tggttatccggtttcctcatcattggttcaggagctcatgcatcaatattcaacttgcga
gcatcaccaacatctgagataagacatcgagaccccatatactcacacctcatctgggta
tgtatagcaataggattacactcattcagtctctactgtcataacgacactttagaagca
ttaggacgtccagaagatatctttcatgacaactctatccagttaaaagcaatatttgcc
aagcaatcctttctaagagcagagctccagccagatatcgagatgttagataaaaagatt
atacgtataacccaagaattgggaacagcagattttatagttcaccacattcatgcattt
acaatccatgttactctcttaattctttcaaagggtgtactttatgctagaaactctaga
tttgtatcagataaattggaacttggttttacttatccctgtgatggtccaggtagaggt
ggtacatgtcaaatatcaccttgggatcacttattctcagctgtattttggatgtataac
tgtttaaatgtagtaacattccactacttttggaagatgcaatcagatgtttggggcttt
gtatcgatccaaaagcacatatctcactacagccaaggtgattttagtgtcaactctatt
accatcaatggttggttgagaaacttattatggtccgaggcatcccaagtaatccaatcc
tatgctctttcatccatttgtccatatgggttcattttcctcatagctcatttcatctgg
gcatttagtttaatgttcctctttagtggtagagcatactggcaagaattaattgagtcc
attctatggtcacatcataaattaaagattatacctcatatccaaccaagggcattaagc
atttctcaaggaagagcagttggtttcattcattacactctaggaggtatcggctcaacc
tgggcatttattatttcaagactgctagtattaacttcttga
>Od4_psaA
atgcacatcttcagatatatcaacacaacactgtggtccaaagcaggacattttaataaa
gctttatcaaaaggagctaagacaactacttggatatggaatctacatgattatgctcat
gattttgacattcaacaaagatccacaggtttaatagcaagaaaagtcttctcatccaat
ctagctcatctatctttggtattcttttggattagtggaatgcaccttcatggggcatat
ttgtctaattatgatatttggttaaaagatcccaaatccatcaccccatcatcacatcta

gcctactctttaattggtcaagatattcttaactcatatacctcagaatacttctcagga
atcactatcacatctggctttttccaactctaccgttctgagggtatcattacacagtcc
cagctgaaatatgcatgtgctacttccctaatagctacacttatctgcctttctggatct
tatctccacatgcagcttatgtccaaattcactagcttttacaagaagttccagtcctta
tcgcaagatcatctcataattatatttggctccagctccacatccctctccgctcatcag
atacacaaaatgcttccagctaacccttactagattcaggtatctccaacccaagtatc
ctacaagtaatctccaacagtctcagtgaaaacctagcactgttttcaacaaatctaagc
tctacaggtaaattgttaaatccctcaacaagaagtgtattcctttctcaagttgcagca
catcataagacaacaggtgtagtctttatcacactagggcttatcagattcctcaccatg
tataactctcaatttagtatacttacatcttatatagattaccacattgtgctatctatt
aatttagcattaattgcatctttatcaataatagtagctgatcatctcacaagaacccca
atctatcctcacaactcaacctcataccctacaatcctttgcctctccatacatcatgca
tggttatccggtttcctcatcattggttcaggagctcatgcatcaatattcaacttgcga
gcatcaccaacatctgagataagacatcgagaccccatatactcacacctcatctgggta
tgtatagcaataggattacactcattcagtctctactgtcataacgacactttagaagca
ttaggacgtccagaagatatctttcatgacaactctatccagttaaaagcaatatttgcc
aagcaatcctttctaagagcagagctccagccagatatcgagatgttagataaaaagatt
atacgtataacccaagaattgggaacagcagattttatagttcaccacattcatgcattt
acaatccatgttactctcttaattctttcaaagggtgtactttatgctagaaactctaga
tttgtatcagataaattggaacttggttttacttatccctgtgatggtccaggtagaggt
ggtacatgtcaaatatcaccttgggatcacttattctcagctgtattttggatgtataac
tgtttaaatgtagtaacattccactacttttggaagatgcaatcagatgtttggggcttt
gtatcgatccaaaagcacatatctcactacagccaaggtgattttagtgtcaactctatt
accatcaatggttggttgagaaacttattatggtccgaggcatcccaagtaatccaatcc
tatgctctttcatccatttgtccatatgggttcattttcctcatagctcatttcatctgg
gcatttagtttaatgttcctctttagtggtagagcatactggcaagaattaattgagtcc
attctatggtcacatcataaattaaagattatacctcatatccaaccaagggcattaagc
atttctcaaggaagagcagttggtttcattcattacactctaggaggtatcggctcaacc
tgggcatttattatttcaagactgctagtattaacttcttga
>Od6_psaA
atgcacatcttcagatatatcaacacaacactgtggtccaaagcaggacattttaataaa
gctttatcaaaaggagctaagacaactacttggatatggaatctacatgattatgctcat
gattttgacattcaacaaagatccacaggtttaatagcaagaaaagtcttctcatccaat
ctagctcatctatctttggtattcttttggattagtggaatgcaccttcatggggcatat
ttgtctaattatgatatttggttaaaagatcccaaatccatcaccccatcatcacatcta
gcctactctttaattggtcaagatattcttaactcatatacctcagaatacttctcagga
atcactatcacatctggctttttccaactctaccgttctgagggtatcattacacagtcc
cagctgaaatatgcatgtgctacttccctaatagctacacttatctgcctttctggatct
tatctccacatgcagcttatgtccaaattcactagcttttacaagaagttccagtcctta
tcgcaagatcatctcataattatatttggctccagctccacatccctctccgctcatcag
atacacaaaatgcttccagctaacccttactagattcaggtatctccaacccaagtatc
ctacaagtaatctccaacagtctcagtgaaaacctagcactgttttcaacaaatctaagc
tctacaggtaaattgttaaatccctcaacaagaagtgtattcctttctcaagttgcagca
catcataagacaacaggtgtagtctttatcacactagggcttatcagattcctcaccatg
tataactctcaatttagtatacttacatcttatatagattaccacattgtgctatctatt
aatttagcattaattgcatctttatcaataatagtagctgatcatctcacaagaacccca
atctatcctcacaactcaacctcataccctacaatcctttgcctctccatacatcatgca
tggttatccggtttcctcatcattggttcaggagctcatgcatcaatattcaacttgcga
gcatcaccaacatctgagataagacatcgagaccccatatactcacacctcatctgggta
tgtatagcaataggattacactcattcagtctctactgtcataacgacactttagaagca
ttaggacgtccagaagatatctttcatgacaactctatccagttaaaagcaatatttgcc
aagcaatcctttctaagagcagagctccagccagatatcgagatgttagataaaaagatt
atacgtataacccaagaattgggaacagcagattttatagttcaccacattcatgcattt
acaatccatgttactctcttaattctttcaaagggtgtactttatgctagaaactctaga
tttgtatcagataaattggaacttggttttacttatccctgtgatggtccaggtagaggt
ggtacatgtcaaatatcaccttgggatcacttattctcagctgtattttggatgtataac
tgtttaaatgtagtaacattccactacttttggaagatgcaatcagatgtttggggcttt

gtatcgatccaaaagcacatatctcactacagccaaggtgattttagtgtcaactctatt
accatcaatggttggttgagaaacttattatggtccgaggcatcccaagtaatccaatcc
tatgctctttcatccatttgtccatatgggttcattttcctcatagctcatttcatctgg
gcatttagtttaatgttcctctttagtggtagagcatactggcaagaattaattgagtcc
attctatggtcacatcataaattaaagattatacctcatatccaaccaagggcattaagc
atttctcaaggaagagcagttggtttcattcattacactctaggaggtatcggctcaacc
tgggcatttattatttcaagactgctagtattaacttcttga
>Od8_psaA
atgcacatcttcagatatatcaacacaacactgtggtccaaagcaggacattttaataaa
gctttatcaaaaggagctaagacaactacttggatatggaatctacatgattatgctcat
gattttgacattcaacaaagatccacaggtttaatagcaagaaaagtcttctcatccaat
ctagctcatctatctttggtattcttttggattagtggaatgcaccttcatggggcatat
ttgtctaattatgatatttggttaaaagatcccaaatccatcaccccatcatcacatcta
gcctactctttaattggtcaagatattcttaactcatatacctcagaatacttctcagga
atcactatcacatctggctttttccaactctaccgttctgagggtatcattacacagtcc
cagctgaaatatgcatgtgctacttccctaatagctacacttatctgcctttctggatct
tatctccacatgcagcttatgtccaaattcactagcttttacaagaagttccagtcctta
tcgcaagatcatctcataattatatttggctccagctccacatccctctccgctcatcag
atacacaaaatgcttccagctaaccctttactagattcaggtatctccaacccaagtatc
ctacaagtaatctccaacagtctcagtgaaaacctagcactgttttcaacaaatctaagc
tctacaggtaaattgttaaatccctcaacaagaagtgtattcctttctcaagttgcagca
catcataagacaacaggtgtagtctttatcacactagggcttatcagattcctcaccatg
tataactctcaatttagtatacttacatcttatatagattaccacattgtgctatctatt
aatttagcattaattgcatctttatcaataatagtagctgatcatctcacaagaacccca
atctatcctcacaactcaacctcataccctacaatcctttgcctctccatacatcatgca
tggttatccggtttcctcatcattggttcaggagctcatgcatcaatattcaacttgcga
gcatcaccaacatctgagataagacatcgagaccccatatactcacacctcatctgggta
tgtatagcaataggattacactcattcagtctctactgtcataacgacactttagaagca
ttaggacgtccagaagatatctttcatgacaactctatccagttaaaagcaatatttgcc
aagcaatcctttctaagagcagagctccagccagatatcgagatgttagataaaaagatt
atacgtataacccaagaattgggaacagcagattttatagttcaccacattcatgcattt
acaatccatgttactctcttaattctttcaaagggtgtactttatgctagaaactctaga
tttgtatcagataaattggaacttggttttacttatccctgtgatggtccaggtagaggt
ggtacatgtcaaatatcaccttgggatcacttattctcagctgtattttggatgtataac
tgtttaaatgtagtaacattccactacttttggaagatgcaatcagatgtttggggcttt
gtatcgatccaaaagcacatatctcactacagccaaggtgattttagtgtcaactctatt
accatcaatggttggttgagaaacttattatggtccgaggcatcccaagtaatccaatcc
tatgctctttcatccatttgtccatatgggttcattttcctcatagctcatttcatctgg
gcatttagtttaatgttcctctttagtggtagagcatactggcaagaattaattgagtcc
attctatggtcacatcataaattaaagattatacctcatatccaaccaagggcattaagc
atttctcaaggaagagcagttggtttcattcattacactctaggaggtatcggctcaacc
tgggcatttattatttcaagactgctagtattaacttcttga
>Od9_psaA
atgcacatcttcagatatatcaacacaacactgtggtccaaagcaggacattttaataaa
gctttatcaaaaggagctaagacaactacttggatatggaatctacatgattatgctcat
gattttgacattcaacaaagatccacaggtttaatagcaagaaaagtcttctcatccaat
ctagctcatctatctttggtattcttttggattagtggaatgcaccttcatggggcatat
ttgtctaattatgatatttggttaaaagatcccaaatccatcaccccatcatcacatcta
gcctactctttaattggtcaagatattcttaactcatatacctcagaatacttctcagga
atcactatcacatctggctttttccaactctaccgttctgagggtatcattacacagtcc
cagctgaaatatgcatgtgctacttccctaatagctacacttatctgcctttctggatct
tatctccacatgcagcttatgtccaaattcactagcttttacaagaagttccagtcctta
tcgcaagatcatctcataattatatttggctccagctccacatccctctccgctcatcag
atacacaaaatgcttccagctaaccctttactagattcaggtatctccaacccaagtatc
ctacaagtaatctccaacagtctcagtgaaaacctagcactgttttcaacaaatctaagc
tctacaggtaaattgttaaatccctcaacaagaagtgtattcctttctcaagttgcagca
catcataagacaacaggtgtagtctttatcacactagggcttatcagattcctcaccatg

tataactctcaatttagtatacttacatcttatatagattaccacattgtgctatctatt
aatttagcattaattgcatctttatcaataatagtagctgatcatctcacaagaacccca
atctatcctcacaactcaacctcataccctacaatcctttgcctctccatacatcatgca
tggttatccggtttcctcatcattggttcaggagctcatgcatcaatattcaacttgcga
gcatcaccaacatctgagataagacatcgagaccccatatactcacacctcatctgggta
tgtatagcaataggattacactcattcagtctctactgtcataacgacactttagaagca
ttaggacgtccagaagatatctttcatgacaactctatccagttaaaagcaatatttgcc
aagcaatcctttctaagagcagagctccagccagatatcgagatgttagataaaaagatt
atacgtataacccaagaattgggaacagcagattttatagttcaccacattcatgcattt
acaatccatgttactctcttaattctttcaaagggtgtactttatgctagaaactctaga
tttgtatcagataaattggaacttggttttacttatccctgtgatggtccaggtagaggt
ggtacatgtcaaatatcaccttgggatcacttattctcagctgtattttggatgtataac
tgtttaaatgtagtaacattccactacttttggaagatgcaatcagatgtttggggcttt
gtatcgatccaaaagcacatatctcactacagccaaggtgattttagtgtcaactctatt
accatcaatggttggttgagaaacttattatggtccgaggcatcccaagtaatccaatcc
tatgctctttcatccatttgtccatatgggttcattttcctcatagctcatttcatctgg
gcatttagtttaatgttcctctttagtggtagagcatactggcaagaattaattgagtcc
attctatggtcacatcataaattaaagattatacctcatatccaaccaagggcattaagc
atttctcaaggaagagcagttggtttcattcattacactctaggaggtatcggctcaacc
tgggcatttattatttcaagactgctagtattaacttcttga
>Ss5_psaA
atgaacatcttcagatatatcaacacaacactgtggtccaaagcaggacattttaataaa
gctttatcaaaaggagctaagacaactacttggatatggaatctacatgattatgctcat
gattttgacattcaacaaagatccacaggtttaatagcaagaaaagtcttctcatccaat
ctagctcatctatctttggtattcttttggattagtggaatgcaccttcatggggcatat
ttgtctaattatgatatttggttaaaagatcccaaatccatcaccccatcatcacatcta
gcctactctttaattggtcaagatattcttaactcatatacctcagaatacttctcagga
atcactatcacatctggctttttccaactctaccgttctgagggtatcattacacagtcc
cagctgaaatatgcatgtgctacttccctaatagctacacttatctgcctttctggatct
tatctccacatgcagcttatgtccaaattcactagcttttacaagaagttccagtcctta
tcgcaagatcatctcataattatatttggctccagctccacatccctctccgctcatcag
atacacaaaatgcttccagctaaccctttactagattcaggtatctccaacccaagtatc
ctacaagtaatctccaacagtctcagtgaaaacctagcactgttttcaacaaatctaagc
tctacaggtaaattgttaaatccctcaacaagaagtgtattcctttctcaagttgcagca
catcataagacaacaggtgtagtctttatcacactagggcttatcagattcctcaccatg
tataactctcaatttagtatacttacatcttatatagattaccacattgtgctatctatt
aatttagcattaattgcatctttatcaataatagtagctgatcatctcacaagaacccca
atctatcctcacaactcaacctcataccctacaatcctttgcctctccatacatcatgca
tggttatccggtttcctcatcattggttcaggagctcatgcatcaatattcaacttgcga
gcatcaccaacatctgagataagacatcgagaccccatatactcacacctcatctgggta
tgtatagcaataggattacactcattcagtctctactgtcataacgacactttagaagca
ttaggacgtccagaagatatctttcatgacaactctatccagttaaaagcaatatttgcc
aagcaatcctttctaagagcagagctccagccagatatcgagatgttagataaaaagatt
atacgtataacccaagaattgggaacagcagattttatagttcaccacattcatgcattt
acaatccatgttactctcttaattctttcaaagggtgtactttatgctagaaactctaga
tttgtatcagataaattggaacttggttttacttatccctgtgatggtccaggtagaggt
ggtacatgtcaaatatcaccttgggatcacttattctcagctgtattttggatgtataac
tgtttaaatgtagtaacattccactacttttggaagatgcaatcagatgtttggggcttt
gtatcgatccaaaagcacatatctcactacagccaaggtgattttagtgtcaactctatt
accatcaatggttggttgagaaacttattatggtccgaggcatcccaagtaatccaatcc
tatgctctttcatccatttgtccatatgggttcattttcctcatagctcatttcatctgg
gcatttagtttaatgttcctctttagtggtagagcatactggcaagaattaattgagtcc
attctatggtcacatcataaattaaagattatacctcatatccaaccaagggcattaagc
atttctcaaggaagagcagttggtttcattcattacactctaggaggtatcggctcaacc
tgggcatttattatttcaagactgctagtattaacttcttga
>Ss6_psaA
atgcacatcttcagatatatcaacacaacactgtggtccaaagcaggacattttaataaa

gctttatcaaaaggagctaagacaactacttggatatggaatctacatgattatgctcat
gattttgacattcaacaaagatccacaggtttaatagcaagaaaagtcttctcatccaat
ctagctcatctatctttggtattcttttggattagtggaatgcaccttcatggggcatat
ttgtctaattatgatatttggttaaaagatcccaaatccatcaccccatcatcacatcta
gcctactctttaattggtcaagatattcttaactcatatacctcagaatacttctcagga
atcactatcacatctggctttttccaactctaccgttctgagggtatcattacacagtcc
cagctgaaatatgcatgtgctacttccctaatagctacacttatctgcctttctggatct
tatctccacatgcagcttatgtccaaattcactagcttttacaagaagttccagtcctta
tcgcaagatcatctcataattatatttggctccagctccacatccctctccgctcatcag
atacacaaaatgcttccagctaaccctttactagattcaggtatctccaacccaagtatc
ctacaagtaatctccaacagtctcagtgaaaacctagcactgttttcaacaaatctaagc
tctacaggtaaattgttaaatccctcaacaagaagtgtattcctttctcaagttgcagca
catcataagacaacaggtgtagtctttatcacactagggcttatcagattcctcaccatg
tataactctcaatttagtatacttacatcttatatagattaccacattgtgctatctatt
aatttagcattaattgcatctttatcaataatagtagctgatcatctcacaagaacccca
atctatcctcacaactcaacctcataccctacaatcctttgcctctccatacatcatgca
tggttatccggtttcctcatcattggttcaggagctcatgcatcaatattcaacttgcga
gcatcaccaacatctgagataagacatcgagaccccatatactcacacctcatctgggta
tgtatagcaataggattacactcattcagtctctactgtcataacgacactttagaagca
ttaggacgtccagaagatatctttcatgacaactctatccagttaaaagcaatatttgcc
aagcaatcctttctaagagcagagctccagccagatatcgagatgttagataaaaagatt
atacgtataacccaagaattgggaacagcagattttatagttcaccacattcatgcattt
acaatccatgttactctcttaattctttcaaagggtgtactttatgctagaaactctaga
tttgtatcagataaattggaacttggttttacttatccctgtgatggtccaggtagaggt
ggtacatgtcaaatatcaccttgggatcacttattctcagctgtattttggatgtataac
tgtttaaatgtagtaacattccactacttttggaagatgcaatcagatgtttggggcttt
gtatcgatccaaaagcacatatctcactacagccaaggtgattttagtgtcaactctatt
accatcaatggttggttgagaaacttattatggtccgaggcatcccaagtaatccaatcc
tatgctctttcatccatttgtccatatgggttcattttcctcatagctcatttcatctgg
gcatttagtttaatgttcctctttagtggtagagcatactggcaagaattaattgagtcc
attctatggtcacatcataaattaaagattatacctcatatccaaccaagggcattaagc
atttctcaaggaagagcagttggtttcattcattacactctaggaggtatcggctcaacc
tgggcatttattatttcaagactgctagtattaacttcttga
>Ss7_psaA
atgcacatcttcagatatatcaacacaacactgtggtccaaagcaggacattttaataaa
gctttatcaaaaggagctaagacaactacttggatatggaatctacatgattatgctcat
gattttgacattcaacaaagatccacaggtttaatagcaagaaaagtcttctcatccaat
ctagctcatctatctttggtattcttttggattagtggaatgcaccttcatggggcatat
ttgtctaattatgatatttggttaaaagatcccaaatccatcaccccatcatcacatcta
gcctactctttaattggtcaagatattcttaactcatatacctcagaatacttctcagga
atcactatcacatctggctttttccaactctaccgttctgagggtatcattacacagtcc
cagctgaaatatgcatgtgctacttccctaatagctacacttatctgcctttctggatct
tatctccacatgcagcttatgtccaaattcactagcttttacaagaagttccagtcctta
tcgcaagatcatctcataattatatttggctccagctccacatccctctccgctcatcag
atacacaaaatgcttccagctaaccctttactagattcaggtatctccaacccaagtatc
ctacaagtaatctccaacagtctcagtgaaaacctagcactgttttcaacaaatctaagc
tctacaggtaaattgttaaatccctcaacaagaagtgtattcctttctcaagttgcagca
catcataagacaacaggtgtagtctttatcacactagggcttatcagattcctcaccatg
tataactctcaatttagtatacttacatcttatatagattaccacattgtgctatctatt
aatttagcattaattgcatctttatcaataatagtagctgatcatctcacaagaacccca
atctatcctcacaactcaacctcataccctacaatcctttgcctctccatacatcatgca
tggttatccggtttcctcatcattggttcaggagctcatgcatcaatattcaacttgcga
gcatcaccaacatctgagataagacatcgagaccccatatactcacacctcatctgggta
tgtatagcaataggattacactcattcagtctctactgtcataacgacactttagaagca
ttaggacgtccagaagatatctttcatgacaactctatccagttaaaagcaatatttgcc
aagcaatcctttctaagagcagagctccagccagatatcgagatgttagataaaaagatt
atacgtataacccaagaattgggaacagcagattttatagttcaccacattcatgcattt

acaatccatgttactctcttaattctttcaaagggtgtactttatgctagaaactctaga
tttgtatcagataaattggaacttggttttacttatccctgtgatggtccaggtagaggt
ggtacatgtcaaatatcaccttgggatcacttattctcagctgtattttggatgtataac
tgtttaaatgtagtaacattccactacttttggaagatgcaatcagatgtttggggcttt
gtatcgatccaaaagcacatatctcactacagccaaggtgattttagtgtcaactctatt
accatcaatggttggttgagaaacttattatggtccgaggcatcccaagtaatccaatcc
tatgctctttcatccatttgtccatatgggttcattttcctcatagctcatttcatctgg
gcatttagtttaatgttcctctttagtggtagagcatactggcaagaattaattgagtcc
attctatggtcacatcataaattaaagattatacctcatatccaaccaagggcattaagc
atttctcaaggaagagcagttggtttcattcattacactctaggaggtatcggctcaacc
tgggcatttattatttcaagactgctagtattaacttcttga
>Ss8_psaA
atgcacatcttcagatatatcaacacaacactgtggtccaaagcaggacattttaataaa
gctttatcaaaaggagctaagacaactacttggatatggaatctacatgattatgctcat
gattttgacattcaacaaagatccacaggtttaatagcaagaaaagtcttctcatccaat
ctagctcatctatctttggtattcttttggattagtggaatgcaccttcatggggcatat
ttgtctaattatgatatttggttaaaagatcccaaatccatcaccccatcatcacatcta
gcctactctttaattggtcaagatattcttaactcatatacctcagaatacttctcagga
atcactatcacatctggctttttccaactctaccgttctgagggtatcattacacagtcc
cagctgaaatatgcatgtgctacttccctaatagctacacttatctgcctttctggatct
tatctccacatgcagcttatgtccaaattcactagcttttacaagaagttccagtcctta
tcgcaagatcatctcataattatatttggctccagctccacatccctctccgctcatcag
atacacaaaatgcttccagctaaccctttactagattcaggtatctccaacccaagtatc
ctacaagtaatctccaacagtctcagtgaaaacctagcactgttttcaacaaatctaagc
tctacaggtaaattgttaaatccctcaacaagaagtgtattcctttctcaagttgcagca
catcataagacaacaggtgtagtctttatcacactagggcttatcagattcctcaccatg
tataactctcaatttagtatacttacatcttatatagattaccacattgtgctatctatt
aatttagcattaattgcatctttatcaataatagtagctgatcatctcacaagaacccca
atctatcctcacaactcaacctcataccctacaatcctttgcctctccatacatcatgca
tggttatccggtttcctcatcattggttcaggagctcatgcatcaatattcaacttgcga
gcatcaccaacatctgagataagacatcgagaccccatatactcacacctcatctgggta
tgtatagcaataggattacactcattcagtctctactgtcataacgacactttagaagca
ttaggacgtccagaagatatctttcatgacaactctatccagttaaaagcaatatttgcc
aagcaatcctttctaagagcagagctccagccagatatcgagatgttagataaaaagatt
atacgtataacccaagaattgggaacagcagattttatagttcaccacattcatgcattt
acaatccatgttactctcttaattctttcaaagggtgtactttatgctagaaactctaga
tttgtatcagataaattggaacttggttttacttatccctgtgatgggccaggtagaggt
ggtacatgtcaaatatcaccttgggatcacttattctcagctgtattttggatgtataac
tgtttaaatgtagtaacattccactacttttggaagatgcaatcagatgtttggggcttt
gtatcgatccaaaagcacatatctcactacagccaaggtgattttagtgtcaactctatt
accatcaatggttggttgagaaacttattatggtccgaggcatcccaagtaatccaatcc
tatgctctttcatccatttgtccatatgggttcattttcctcatagctcatttcatctgg
gcatttagtttaatgttcctctttagtggtagagcatactggcaagaattaattgagtcc
attctatggtcacatcataaattaaagattatacctcatatccaaccaagggcattaagc
atttctcaaggaagagcagttggtttcattcattacactctaggaggtatcggctcaacc
tgggcatttattatttcaagactgctagtattaacttcttga
>ohdo1_psaA
atgcacatcttcagatatatcaacacaacactgtggtccaaagcaggacattttaataaa
gctttatcaaaaggagctaagacaactacttggatatggaatctacatgattatgctcat
gattttgacattcaacaaagatccacaggtttaatagcaagaaaagtcttctcatccaat
ctagctcatctatctttggtattcttttggattagtggaatgcaccttcatggggcatat
ttgtctaattatgatatttggttaaaagatcccaaatccatcaccccatcatcacatcta
gcctactctttaattggtcaagatattcttaactcatatacctcagaatacttctcagga
atcactatcacatctggctttttccaactctaccgttctgagggtatcattacacagtcc
cagctgaaatatgcatgtgctacttccctaatagctacacttatctgcctttctggatct
tatctccacatgcagcttatgtccaaattcactagcttttacaagaagttccagtcctta
tcgcaagatcatctcataattatatttggctccagctccacatccctctccgctcatcag

atacacaaaatgcttccagctaaccctttactagattcaggtatctccaacccaagtatc
ctacaagtaatctccaacagtctcagtgaaaacctagcactgttttcaacaaatctaagc
tctacaggtaaattgttaaatccctcaacaagaagtgtattcctttctcaagttgcagca
catcataagacaacaggtgtagtctttatcacactagggcttatcagattcctcaccatg
tataactctcaatttagtatacttacatcttatatagattaccacattgtgctatctatt
aatttagcattaattgcatctttatcaataatagtagctgatcatctcacaagaacccca
atctatcctcacaactcaacctcataccctacaatcctttgcctctccatacatcatgca
tggttatccggtttcctcatcattggttcaggagctcatgcatcaatattcaacttgcga
gcatcaccaacatctgagataagacatcgagaccccatatactcacacctcatctgggta
tgtatagcaataggattacactcattcagtctctactgtcataacgacactttagaagca
ttaggacgtccagaagatatctttcatgacaactctatccagttaaaagcaatatttgcc
aagcaatcctttctaagagcagagctccagccagatatcgagatgttagataaaaagatt
atacgtataacccaagaattgggaacagcagattttatagttcaccacattcatgcattt
acaatccatgttactctcttaattctttcaaagggtgtactttatgctagaaactctaga
tttgtatcagataaattggaacttggttttacttatccctgtgatgggccaggtagaggt
ggtacatgtcaaatatcaccttgggatcacttattctcagctgtattttggatgtataac
tgtttaaatgtagtaacattccactacttttggaagatgcaatcagatgtttggggcttt
gtatcgatccaaaagcacatatctcactacagccaaggtgattttagtgtcaactctatt
accatcaatggttggttgagaaacttattatggtccgaggcatcccaagtaatccaatcc
tatgctctttcatccatttgtccatatgggttcattttcctcatagctcatttcatctgg
gcatttagtttaatgttcctctttagtggtagagcatactggcaagaattaattgagtcc
attctatggtcacatcataaattaaagattatacctcatatccaaccaagggcattaagc
atttctcaaggaatagcagttggtttcattcattacactctaggaggtatcggctcaacc
tgggcatttattatttcaagactgctagtattaacttcttga
>ohdo3_psaA
atgcacatcttcagatatatcaacacaacactgtggtccaaagcaggacattttaataaa
gctttatcaaaaggagctaagacaactacttggatatggaatctacatgattatgctcat
gattttgacattcaacaaagatccacaggtttaatagcaagaaaagtcttctcatccaat
ctagctcatctatctttggtattcttttggattagtggaatgcaccttcatggggcatat
ttgtctaattatgatatttggttaaaagatcccaaatccatcaccccatcatcacatcta
gcctactctttaattggtcaagatattcttaactcatatacctcagaatacttctcagga
atcactatcacatctggctttttccaactctaccgttctgagggtatcattacacagtcc
cagctgaaatatgcatgtgctacttccctaatagctacacttatctgcctttctggatct
tatctccacatgcagcttatgtccaaattcactagcttttacaagaagttccagtcctta
tcgcaagatcatctcataattatatttggctccagctccacatccctctccgctcatcag
atacacaaaatgcttccagctaaccctttactagattcaggtatctccaacccaagtatc
ctacaagtaatctccaacagtctcagtgaaaacctagcactgttttcaacaaatctaagc
tctacaggtaaattgttaaatccctcaacaagaagtgtattcctttctcaagttgcagca
catcataagacaacaggtgtagtctttatcacactagggcttatcagattcctcaccatg
tataactctcaatttagtatacttacatcttatatagattaccacattgtgctatctatt
aatttagcattaattgcatctttatcaataatagtagctgatcatctcacaagaacccca
atctatcctcacaactcaacctcataccctacaatcctttgcctctccatacatcatgca
tggttatccggtttcctcatcattggttcaggagctcatgcatcaatattcaacttgcga
gcatcaccaacatctgagataagacatcgagaccccatatactcacacctcatctgggta
tgtatagcaataggattacactcattcagtctctactgtcataacgacactttagaagca
ttaggacgtccagaagatatctttcatgacaactctatccagttaaaagcaatatttgcc
aagcaatcctttctaagagcagagctccagccagatatcgagatgttagataaaaagatt
atacgtataacccaagaattgggaacagcagattttatagttcaccacattcatgcattt
acaatccatgttactctcttaattctttcaaagggtgtactttatgctagaaactctaga
tttgtatcagataaattggaacttggttttacttatccctgtgatggtccaggtagaggt
ggtacatgtcaaatatcaccttgggatcacttattctcagctgtattttggatgtataac
tgtttaaatgtagtaacattccactacttttggaagatgcaatcagatgtttggggcttt
gtatcgatccaaaagcacatatctcactacagccaaggtgattttagtgtcaactctatt
accatcaatggttggttgagaaacttattatggtccgaggcatcccaagtaatccaatcc
tatgctctttcatccatttgtccatatgggttcattttcctcatagctcatttcatctgg
gcatttagtttaatgttcctctttagtggtagagcatactggcaagaattaattgagtcc
attctatggtcacatcataaattaaagattatacctcatatccaaccaagggcattaagc

atttctcaaggaagagcagttggtttcattcattacactctaggaggtatcggctcaacc
tgggcatttattatttcaagactgctagtattaacttcttga
>ohdo5_psaA
atgcacatcttcagatatatcaacacaacactgtggtccaaagcaggacattttaataaa
gctttatcaaaaggagctaagacaactacttggatatggaatctacatgattatgctcat
gattttgacattcaacaaagatccacaggtttaatagcaagaaaagtcttctcatccaat
ctagctcatctatctttggtattcttttggattagtggaatgcaccttcatggggcatat
ttgtctaattatgatatttggttaaaagatcccaaatccatcaccccatcatcacatcta
gcctactctttaattggtcaagatattcttaactcatatacctcagaatacttctcagga
atcactatcacatctggctttttccaactctaccgttctgagggtatcattacacagtcc
cagctgaaatatgcatgtgctacttccctaatagctacacttatctgcctttctggatct
tatctccacatgcagcttatgtccaaattcactagcttttacaagaagttccagtcctta
tcgcaagatcatctcataattatatttggctccagctccacatccctctccgctcatcag
atacacaaaatgcttccagctaaccctttactagattcaggtatctccaacccaagtatc
ctacaagtaatctccaacagtctcagtgaaaacctagcactgttttcaacaaatctaagc
tctacaggtaaattgttaaatccctcaacaagaagtgtattcctttctcaagttgcagca
catcataagacaacaggtgtagtctttatcacactagggcttatcagattcctcaccatg
tataactctcaatttagtatacttacatcttatatagattaccacattgtgctatctatt
aatttagcattaattgcatctttatcaataatagtagctgatcatctcacaagaacccca
atctatcctcacaactcaacctcataccctacaatcctttgcctctccatacatcatgca
tggttatccggtttcctcatcattggttcaggagctcatgcatcaatattcaacttgcga
gcatcaccaacatctgagataagacatcgagaccccatatactcacacctcatctgggta
tgtatagcaataggattacactcattcagtctctactgtcataacgacactttagaagca
ttaggacgtccagaagatatctttcatgacaactctatccagttaaaagcaatatttgcc
aagcaatcctttctaagagcagagctccagccagatatcgagatgttagataaaaagatt
atacgtataacccaagaattgggaacagcagattttatagttcaccacattcatgcattt
acaatccatgttactctcttaattctttcaaagggtgtactttatgctagaaactctaga
tttgtatcagataaattggaacttggttttacttatccctgtgatggtccaggtagaggt
ggtacatgtcaaatatcaccttgggatcacttattctcagctgtattttggatgtataac
tgtttaaatgtagtaacattccactacttttggaagatgcaatcagatgtttggggcttt
gtatcgatccaaaagcacatatctcactacagccaaggtgattttagtgtcaactctatt
accatcaatggttggttgagaaacttattatggtccgaggcatcccaagtaatccaatcc
tatgctctttcatccatttgtccatatgggttcattttcctcatagctcatttcatctgg
gcatttagtttaatgttcctctttagtggtagagcatactggcaagaattaattgagtcc
attctatggtcacatcataaattaaagattatacctcatatccaaccaagggcattaagc
atttctcaaggaagagcagttggtttcattcattacactctaggaggtatcggctcaacc
tgggcatttattatttcaagactgctagtattaacttcttga
>ohdo7_psaA
atgaacatcttcagatatatcaacacaacactgtggtccaaagcaggacattttaataaa
gctttatcaaaaggagctaagacaactacttggatatggaatctacatgattatgctcat
gattttgacattcaacaaagatccacaggtttaatagcaagaaaagtcttctcatccaat
ctagctcatctatctttggtattcttttggattagtggaatgcaccttcatggggcatat
ttgtctaattatgatatttggttaaaagatcccaaatccatcaccccatcatcacatcta
gcctactctttaattggtcaagatattcttaactcatatacctcagaatacttctcagga
atcactatcacatctggctttttccaactctaccgttctgagggtatcattacacagtcc
cagctgaaatatgcatgtgctacttccctaatagctacacttatctgcctttctggatct
tatctccacatgcagcttatgtccaaattcactagcttttacaagaagttccagtcctta
tcgcaagatcatctcataattatatttggctccagctccacatccctctccgctcatcag
atacacaaaatgcttccagctaaccctttactagattcaggtatctccaacccaagtatc
ctacaagtaatctccaacagtctcagtgaaaacctagcactgttttcaacaaatctaagc
tctacaggtaaattgttaaatccctcaacaagaagtgtattcctttctcaagttgcagca
catcataagacaacaggtgtagtctttatcacactagggcttatcagattcctcaccatg
tataactctcaatttagtatacttacatcttatatagattaccacattgtgctatctatt
aatttagcattaattgcatctttatcaataatagtagctgatcatctcacaagaacccca
atctatcctcacaactcaacctcataccctacaatcctttgcctctccatacatcatgca
tggttatccggtttcctcatcattggttcaggagctcatgcatcaatattcaacttgcga
gcatcaccaacatctgagataagacatcgagaccccatatactcacacctcatctgggta

tgtatagcaataggattacactcattcagtctctactgtcataacgacactttagaagca
ttaggacgtccagaagatatctttcatgacaactctatccagttaaaagcaatatttgcc
aagcaatcctttctaagagcagagctccagccagatatcgagatgttagataaaaagatt
atacgtataacccaagaattgggaacagcagattttatagttcaccacattcatgcattt
acaatccatgttactctcttaattctttcaaagggtgtactttatgctagaaactctaga
tttgtatcagataaattggaacttggttttacttatccctgtgatggtccaggtagaggt
ggtacatgtcaaatatcaccttgggatcacttattctcagctgtattttggatgtataac
tgtttaaatgtagtaacattccactacttttggaagatgcaatcagatgtttggggcttt
gtatcgatccaaaagcacatatctcactacagccaaggtgattttagtgtcaactctatt
accatcaatggttggttgagaaacttattatggtccgaggcatcccaagtaatccaatcc
tatgctctttcatccatttgtccatatgggttcattttcctcatagctcatttcatctgg
gcatttagtttaatgttcctctttagtggtagagcatactggcaagaattaattgagtcc
attctatggtcacatcataaattaaagattatacctcatatccaaccaagggcattaagc
atttctcaaggaagagcagttggtttcattcattacactctaggaggtatcggctcaacc
tgggcatttattatttcaagactgctagtattaacttcttga
>sesoko1_psaA
atgcacatcttcagatatatcaacacaacactgtggtccaaagcaggacattttaataaa
gctttatcaaaaggagctaagacaactacttggatatggaatctacatgattatgctcat
gattttgacattcaacaaagatccacaggtttaatagcaagaaaagtcttctcatccaat
ctagctcatctatctttggtattcttttggattagtggaatgcaccttcatggggcatat
ttgtctaattatgatatttggttaaaagatcccaaatccatcaccccatcatcacatcta
gcctactctttaattggtcaagatattcttaactcatatacctcagaatacttctcagga
atcactatcacatctggctttttccaactctaccgttctgagggtatcattacacagtcc
cagctgaaatatgcatgtgctacttccctaatagctacacttatctgcctttctggatct
tatctccacatgcagcttatgtccaaattcactagcttttacaagaagttccagtcctta
tcgcaagatcatctcataattatatttggctccagctccacatccctctccgctcatcag
atacacaaaatgcttccagctaacccttttactagattcaggtatctccaacccaagtatc
ctacaagtaatctccaacagtctcagtgaaaacctagcactgttttcaacaaatctaagc
tctacaggtaaattgttaaatccctcaacaagaagtgtattcctttctcaagttgcagca
catcataagacaacaggtgtagtctttatcacactagggcttatcagattcctcaccatg
tataactctcaatttagtatacttacatcttatatagattaccacattgtgctatctatt
aatttagcattaattgcatctttatcaataatagtagctgatcatctcacaagaacccca
atctatcctcacaactcaacctcataccctacaatcctttgcctctccatacatcatgca
tggttatccggtttcctcatcattggttcaggagctcatgcatcaatattcaacttgcga
gcatcaccaacatctgagataagacatcgagaccccatatactcacacctcatctgggta
tgtatagcaataggattacactcattcagtctctactgtcataacgacactttagaagca
ttaggacgtccagaagatatctttcatgacaactctatccagttaaaagcaatatttgcc
aagcaatcctttctaagagcagagctccagccagatatcgagatgttagataaaaagatt
atacgtataacccaagaattgggaacagcagattttatagttcaccacattcatgcattt
acaatccatgttactctcttaattctttcaaagggtgtactttatgctagaaactctaga
tttgtatcagataaattggaacttggttttacttatccctgtgatggtccaggtagaggt
ggtacatgtcaaatatcaccttgggatcacttattctcagctgtattttggatgtataac
tgtttaaatgtagtaacattccactacttttggaagatgcaatcagatgtttggggcttt
gtatcgatccaaaagcacatatctcactacagccaaggtgattttagtgtcaactctatt
accatcaatggttggttgagaaacttattatggtccgaggcatcccaagtaatccaatcc
tatgctctttcatccatttgtccatatgggttcattttcctcatagctcatttcatctgg
gcatttagtttaatgttcctctttagtggtagagcatactggcaagaattaattgagtcc
attctatggtcacatcataaattaaagattatacctcatatccaaccaagggcattaagc
atttctcaaggaagagcagttggtttcattcattacactctaggaggtatcggctcaacc
tgggcatttattatttcaagactgctagtattaagttcttga
>sesoko3_psaA
atgaacatcttcagatatatcaacacaacactgtggtccaaagcaggacattttaataaa
gctttatcaaaaggagctaagacaactacttggatatggaatctacatgattatgctcat
gattttgacattcaacaaagatccacaggtttaatagcaagaaaagtcttctcatccaat
ctagctcatctatctttggtattcttttggattagtggaatgcaccttcatggggcatat
ttgtctaattatgatatttggttaaaagatcccaaatccatcaccccatcatcacatcta
gcctactctttaattggtcaagatattcttaactcatatacctcagaatacttctcagga

atcactatcacatctggctttttccaactctaccgttctgagggtatcattacacagtcc
cagctgaaatatgcatgtgctacttccctaatagctacacttatctgcctttctggatct
tatctccacatgcagcttatgtccaaattcactagcttttacaagaagttccagtcctta
tcgcaagatcatctcataattatatttggctccagctccacatccctctccgctcatcag
atacacaaaatgcttccagctaacccttactagattcaggtatctccaacccaagtatc
ctacaagtaatctccaacagtctcagtgaaaacctagcactgttttcaacaaatctaagc
tctacaggtaaattgttaaatccctcaacaagaagtgtattcctttctcaagttgcagca
catcataagacaacaggtgtagtctttatcacactagggcttatcagattcctcaccatg
tataactctcaatttagtatacttacatcttatatagattaccacattgtgctatctatt
aatttagcattaattgcatctttatcaataatagtagctgatcatctcacaagaacccca
atctatcctcacaactcaacctcataccctacaatcctttgcctctccatacatcatgca
tggttatccggtttcctcatcattggttcaggagctcatgcatcaatattcaacttgcga
gcatcaccaacatctgagataagacatcgagaccccatatactcacacctcatctgggta
tgtatagcaataggattacactcattcagtctctactgtcataacgacactttagaagca
ttaggacgtccagaagatatctttcatgacaactctatccagttaaaagcaatatttgcc
aagcaatcctttctaagagcagagctccagccagatatcgagatgttagataaaaagatt
atacgtataacccaagaattgggaacagcagattttatagttcaccacattcatgcattt
acaatccatgttactctcttaattctttcaaagggtgtactttatgctagaaactctaga
tttgtatcagataaattggaacttggttttacttatccctgtgatggtccaggtagaggt
ggtacatgtcaaatatcaccttgggatcacttattctcagctgtattttggatgtataac
tgtttaaatgtagtaacattccactacttttggaagatgcaatcagatgtttggggcttt
gtatcgatccaaaagcacatatctcactacagccaaggtgattttagtgtcaactctatt
accatcaatggttggttgagaaacttattatggtccgaggcatcccaagtaatccaatcc
tatgctctttcatccatttgtccatatgggttcattttcctcatagctcatttcatctgg
gcatttagtttaatgttcctctttagtggtagagcatactggcaagaattaattgagtcc
attctatggtcacatcataaattaaagattatacctcatatccaaccaagggcattaagc
atttctcaaggaagagcagttggtttcattcattacactctaggaggtatcggctcaacc
tgggcatttattatttcaagactgctagtattaacttcttgga
>sesoko4_psaA
atgaacatcttcagatatatcaacacaacactgtggtccaaagcaggacattttaataaa
gctttatcaaaaggagctaagacaactacttggatatggaatctacatgattatgctcat
gattttgacattcaacaaagatccacaggtttaatagcaagaaaagtcttctcatccaat
ctagctcatctatctttggtattcttttggattagtggaatgcaccttcatggggcatat
ttgtctaattatgatatttggttaaaagatcccaaatccatcaccccatcatcacatcta
gcctactctttaattggtcaagatattcttaactcatatacctcagaatacttctcagga
atcactatcacatctggctttttccaactctaccgttctgagggtatcattacacagtcc
cagctgaaatatgcatgtgctacttccctaatagctacacttatctgcctttctggatct
tatctccacatgcagcttatgtccaaattcactagcttttacaagaagttccagtcctta
tcgcaagatcatctcataattatatttggctccagctccacatccctctccgctcatcag
atacacaaaatgcttccagctaacccttactagattcaggtatctccaacccaagtatc
ctacaagtaatctccaacagtctcagtgaaaacctagcactgttttcaacaaatctaagc
tctacaggtaaattgttaaatccctcaacaagaagtgtattcctttctcaagttgcagca
catcataagacaacaggtgtagtctttatcacactagggcttatcagattcctcaccatg
tataactctcaatttagtatacttacatcttatatagattaccacattgtgctatctatt
aatttagcattaattgcatctttatcaataatagtagctgatcatctcacaagaacccca
atctatcctcacaactcaacctcataccctacaatcctttgcctctccatacatcatgca
tggttatccggtttcctcatcattggttcaggagctcatgcatcaatattcaacttgcga
gcatcaccaacatctgagataagacatcgagaccccatatactcacacctcatctgggta
tgtatagcaataggattacactcattcagtctctactgtcataacgacactttagaagca
ttaggacgtccagaagatatctttcatgacaactctatccagttaaaagcaatatttgcc
aagcaatcctttctaagagcagagctccagccagatatcgagatgttagataaaaagatt
atacgtataacccaagaattgggaacagcagattttatagttcaccacattcatgcattt
acaatccatgttactctcttaattctttcaaagggtgtactttatgctagaaactctaga
tttgtatcagataaattggaacttggttttacttatccctgtgatggtccaggtagaggt
ggtacatgtcaaatatcaccttgggatcacttattctcagctgtattttggatgtataac
tgtttaaatgtagtaacattccactacttttggaagatgcaatcagatgtttggggcttt
gtatcgatccaaaagcacatatctcactacagccaaggtgattttagtgtcaactctatt

accatcaatggttggttgagaaacttattatggtccgaggcatcccaagtaatccaatcc
tatgctctttcatccatttgtccatatgggttcattttcctcatagctcatttcatctgg
gcatttagtttaatgttcctctttagtggtagagcatactggcaagaattaattgagtcc
attctatggtcacatcataaattaaagattatacctcatatccaaccaagggcattaagc
atttctcaaggaagagcagttggtttcattcattacactctaggaggtatcggctcaacc
tgggcatttattatttcaagactgctagtattaacttcttga
>REF_DNA_psaA_JX094317
ATGCACATCTTCAGATATATCAACACAACACTGTGGGCCAAAGCAGGACATTTTAATAAA
GCTTTATCAAAAGGAGCTAAGACAACTACTTGGATATGGAATCTACATGATTATGCTCAT
GATTTTGACATTCAACAAAGATCCACAGGTTTAATAGCAAGAAAAGTCTTCTCATCCAAT
CTAGCTCATCTATCTTTGGTATTCTTTTGGATTAGTGGAATGCACCTTCATGGAGCATAT
TTGTCTAATTATGATATTTGGTTAAAAGATCCCAAATCCATCACGCCATCATCACATCTA
GCCTACTCTTTAATTGGTCAAGATATTCTTAACTCATATACCTCAGAATACTTCTCAGGA
ATCACTATCACATCTGGCTTTTTCCAACTCTACCGTTCTGAGGGTATCATTACACAGTCC
CAGCTCAAATATGCATGTGCTACTTCCCTAATAGCTACACTTATCTGCCTTTCTGGATCT
TATCTCCACATGCAGCTTATGTCCAAATTCACTAGCTTTTACAAGAAGTTCCAGTCCTTA
TCGCAAGATCATCTCATAATTATATTTGGCTCCAGGTCCACATCCCTCTCCGCTCATCAG
ATACACAAAATGCTTCCAGCTAACCCTTTACTAGATTCAGGTATCTCCAAGCCAAGTATC
CTACAAGTAATCTCCAACAGTCTCAGCTATACACTAGCACTGTTTTCAACAAATCTAAGC
TCTACAGGTAAATTGTTAAATCCCTCAACAAGAAGTGTATTCCTTTCTCAAGTTGCAGCA
CATCATAAGACAACAGGTGTAGTCTTTATCACACTAGGGCTTATCAGATTCCTCACCATG
TATAAGTCTCAATTTAGTATACTTACATCTTATATAGATTACCACATTGTGCTATCTATT
AATTTAGCATTAATTGCATCTTTATCAATAATAGTAGCTGATCATCTCACAAGAACCCCA
ATCTATCCTCACAAGTCAACCTCATACCCTACAATCCTTTGCCTCTCCATACATCATGCA
TGGTTATCCGGTTTCCTCATCATTGGTTCAGGAGCTCATGCATCAATATTCAACTTGCTT
GGATCACCAACATCTGAGATAAGACATCGAGACCCCATATACTCACACCTCATCTGGGTA
TGTATAGCAATAGGATTACACTCATTCAGTCTCTACTGTCATAACGACACTTTAGAAGCA
TTAGGACGTCCAGAAGATATCTTTCATGACAACTCTATCCAGTTAAAAGCAATATTTGCC
AAGCAATCCTTTCTAAGAGCAGAGCTCCAGCCAGATATCGAGATGTTAGATAAAAAGATT
ATACGTATAACCCAAGAATTGGGAACAGCAGATTTTATAGTTCACCACATTCATGCATTT
ACAATCCATGTTACTCTCTTAATTCTTTCAAAGGGTGTACTTTATGCTAGAAACTCTAGA
TTTGTATCAGATAAATTGGAACTTGGTTTTACTTATCCCTGTGATGGGCCAGGTAGAGGT
GGTACATGTCAAATATCACCTTGGGATCACTTATTCTCAGCTGTATTTTGGATGTATAAC
TGTTTAAATGTAGTAACATTCCACTACTTTTGGAAGATGCAATCAGATGTTTGGGGCTTT
GTATCGATCCAAAAGCACATATCTCACTACAGCCAAGGTGATTTTAGTGTCAACTCTATT
ACCATCAATGGTTGGTTGAGAAACTTATTATGGTCCGAGGCATCCCAAGTAATCCAATCC
TATGCTCTTTCATCCATTTGTCCATATGGGTTCATTTTCCTCATAGGTCATTTCATCTGG
GCATTTAGTTTAATGTTCCTCTTTAGTGGTAGAGCATACTGGCAAGAATTAATTGAGTCC
ATTCTATGGTCACATCATAAATTAAAGATTATACCTCATATCCAACCAAGGGCATTAAGC
ATTTCTCAAGGAAGAGCAGTTGGTTTCATTCATTACACTCTAGGAGGTATCGGCTCAACC
TGGGCATTTATTATTTCAAGACTGCTAGTATTAACTTCTTGA
>REF_RNA_psaA_symbB1.comp56_c0_seq1
atgcacatcttcagatatgtcaacgcaacactgtgggccaaagcaggacattttaataaa
gctttatcaaaaggagctaagacaactacttggatatggaatctacatgcttctgctcat
gattttgacattcaacaaggatccgcaggtttaatagcaagaaaagtcttctcatccaat
ctagctcatctatctttggtattcttttggcttggtggaatgcactttcatggagcatat
ttgtctaattatagtgcttggttaaaagatcccaaatccgtcgcgccatcatcacatcta
gcctactctttagttggtcaagatattcttaactcatatacctcagaatacttctcagga
atcactatcacatctggctttttccaactctaccgttctgagggtatcattacacagtcc
cagctcaaatatgcatgtgctgcttccctaatagctacacttatctgcctttctggatct
tatctccacatgcagcttatgtccaaattcactagcttttacaagaagttccagtcctta
tcgcaaagtcatctcataattatgtttggcttcgggtccatatccttctccgctcatcag
atacacgaagcgcttccagttaactctttactagattcaggtatctccgagccaagtatc
ctacaagtaatctccaacagttccagctgtacactagcactgttttcaacaaatctaagc
tctacaggtaaattgttaaatccctcaacaggaagtatattcctttctcaaattgcagca
catcatgcagcagtaggtgtagtctttatcacactagggcttatcagattcctcaccaca
tatgagtctcaatttagtatacttgcatcttatatagattaccacattgtgctatctact

aatttagcattaattgcatctttatcaatagtagcagctcatcatctcgcagaaatccca
atctatccttacgagtcaatctcataccctacagtcctttgcctcttcgtacatcatgca
tggttatccggtttcctcgtcattggttcaggagctcatgcatcaatattcagcttgctt
ggatcaccaacatctgagataagacatcgagaccccatatgctcacacctcatctgggta
tgtatagcactaggattacactcattcggtctctactgtcataacgacactttagaagca
ttaggacgtccagaagatacctttgtgacaactctatccagttaaaaccaatatttgcc
aagcaattctttctaggagcagagctccagccaggtgttgaggtgttagatggaaagatt
gtacgtatgacccaagaattgggaacagcagattttatggttcaccacattcatgcattt
acaatccatgttactctcttaattcttttaaagggtgtactttatgctagaaactctaga
cttgtatcagataaattggaacttggttttgcttatccctgtgatgggccaggtagaggt
ggtacatgtcaaatatcaccttgggatcacttattcttagctgtattttggatgtataac
tgtttaagtatagtagtattccactacttttggaagatgcaatcagatgtttggggcttt
gtatcggcccaaaagcacatatctcactacagccaaggtgattttagtgtcaactctatt
accatcaatggttggttgagaaacttattatggtccgaggcatcccaagtaatccaatcc
tatgctcttccatccatttgttcatatgggttcattttcctcgcaggtcatttcgtctgg
gcatttagtttaatgttcctctttagtggtagagcatactggcaagaattaattgagtcc
gttctatggtcacatcataaattaaagattgtacctcatatccaaccaagggcattaagc
atttctcaaggaagagcagttggtctcactcattacattctaggaggtatcggctcaacc
tgggcatttattatttcaagactgctagtattaacttcttga

>Hd1_psaB
ttttccattgcgaaattacggttagcctccaggccaaatccacatcaaaatcaccaatgc
acacaccaatgcaccttgactgatagctttatatgcttaaatggtagatgtgctagttca
agatatttacaagttctgggttctattcatgatatagaatctggctttggtatagataat
actctttctttaaatctacagatattcacagcacattggggtcacctaactataatctta
atatgggtatcaggcaatctttatcacatagcatccaatgctaactattctctctgggtt
aaaaatccaattccaagcatacctatagcacataatatttgggatccacattttactaac
tctacttccactccatattcccataccatcatcaccacaatcctcattgcttactcaggt
atttataaccaactatacacttctggatttaacactattaatcagatatacaagaccaca
tttacatcctcttgcctggcagtaatatcaatactattagctaagatacatatcaatact
cattcagaactattacacaagcttgcaactcatgcaagtcaaatcccatcattcttccag
cttctctactttctggatgtcggtatctcatctgttaatattcgattcaattttcacact
ggaattctagttggacttttctcaatagcacacacaggacatcttctagacatcacaata
ccagcctctcgagctccactaattcatacctcactctcttacttaacattctttggtggt
ttaaaatcgaatacaacttcattgtaccttacagatattgcacatcatcacttagcaatt
ggtatcacattcatccatacagggcatctatactcctctttcagggcagcccttggaaca
tatatacgagatatattatatacatcacaccttacacacagcataaaatcactacactta
gccctctcactcattctagccagttgtacagcacttacatcaaccactgctcagcacatc
tattctttaacaccatatttttatttgtcttatgatcacatctattttacggctctatat
gtccatcactcctacatcacatcattcttaaccattggttctcatgcacatactgctatc
accttagtaagagattggatcacaccattagaactagaatccagctctaagcggataaga
atacatacacataaagctgccattattccacacttatcctgggtcagtctttggcttggt
tttcacacactagcagtatactctcataatgacacatgtatagcatttaactctccatct
aagcaaatcttaatagaagcaagcaatgctcaacttattcaacaagcatcaggaaaagct
ttatatgggaccatcaactccatcaacaactataataaatcatttgattcatttatacac
cctatcagtccaggtgattcatatgtacatcatgcaatagcacttggcttacatataact
gtcctcatcctactaaagggaggtcttgaagctcgtgggtctaagctaatgccagacaag
atggagcactcatttggcttttcatgtgatggtccaggtagaggtggcacatgtgatata
tcagcttgggattccttttatctagcaacattctggatgcttaatagcaacacatggata
agcttctacttccactacaagcaccttacacctcgtcaattctcagaaagttcaacttat
ctagaatcatggtttcgagattatctgtggtttaactctacacctttaattcatggttat
tctacactaggaactaatgatctatccgtccaatcctggtccttcctacttactcatctt
gcatgggcaagtggatttatgttccttattagttggagaggttactggcaagaattaatt
gatatcatcctctacatacatttgaaaacaccaattcttatcaatctttggaatggagac
atctatacacctctcgccttatctattgtacaagctagatttattgggcttgttcacttc
tcaaccggtctaattttaacttaccctccttttataataggtgctacaagttag
>Hd2_psaB
ttttccattgcgaaattacggttagcctccaggccaaatccacatcaaaatcaccaatgc
acacaccaatgcaccttgactgatagctttatatctttaaatggtaggtgtgcgacttca
agatatttacaagtggtgggctctattcatgatatagaatcttactttggtatagataat
actctttctttaaatctacagatattcacagcacattggggtcacctaactataatctta
atatgggtatcaggcaatctttatcacatagcatccaatgctaactattctctctgggtt
aaaaatccaattccaagcatacctatagcacacaatatttgggatccacattttactaac
tctacttccactccatattcccataccatcatcaccacaatcctcattgcttactcaggt
atttataaccaactatacacttctggatttaacactattaatcagatatacaagaccaca
tttacatcctcttgcctggcagtaatatcaatactattagctaagatacatatcaatact
cattcagaactattacacaagcttgcaactcatgcaagtcaaatcccatcattcttccag
cttctctactttctggatgtcggtatctcatctgttaatattcgattcaattttcacact
ggaattctagttggacttttctcaatagcacacacaggacatcttctagacatcacaata
ccagcctctcgagctccactaattcatacctcactctcttacttaacattctttggtggt
ttaaaatcgaatacaacttcattgtaccttacagatattgcacatcatcacttagcaatt
ggtatcacattcatccatacagggcatctatactcctctttcagggcagcccttggaaca
tatatacgagatacattatatacatcacaccttacacacagcataaaatcactacactta
gccctctcactcattctagccagttgtacagcacttacatctaccaccactcagcacatc
tattctttaacaccatacttttatttgtcttatgatcacatctattttacagctctatat
gtccatcactcctacatcacatcattcttaaccattggttctcatgcacatacagctatc
accttagtaagagattggatcacaccattagaactagaatccagctctaagcagataaga

atacatacacataaagctgccattattccacacttatcctgggtcagtctttggcttggt
tttcacacactagcagtatactctcataatgacacatgtatagcatttaactctccatct
aagcaaatcttaatagaagcaagcaatgctcaacttattcaacaagcatcaggaaaagct
ttatatgggaccatcaactccatcaacaactataataaatcatttgattcatttatacac
cctatcagtccaggtgattcatatgtacatcatgcaatagcacttggcttacatataact
gtcctcatcctactaaagggaggtcttgaagctcgtgggtctaagctaatgccagacaag
atggagcactcatttggcttttcatgtgatggtccaggtagaggtggcacatgtgatata
tcagcttgggattccttttatctagcaacattctggatgcttaatagcaacacatggata
agcttctacttccactacaagcaccttacacctcgtcaattctcagaaagttcaacttat
ctagaatcatggtttcgagattatctgtggtttaactctacacctttaattcatggttat
tctacactaggaactaatgatctatccgtccaatcctggtccttcctacttactcacctt
gcatgggcaagtggatttatgttccttattagttggagaggttactggcaagaattaatt
gatatcatcctctacatacatttgaaaacaccaattcttatcaatctttggaatggagac
atctatacacctctcgccttatctattgtacaagctagatttattgggcttgttcacttc
tcaaccggtctaattttaacttaccctccttttataataggtgctactagttag
>Hd3_psaB
ttttccattgcgaaattacggttagcctccaggccaaatccacatcaaaatcaccaatgc
acacaccaatgcacctttactgatagctttatatccttaaatggtaggtgtgcgagttca
agatatttacaagttctgggctctattcatgatatagaatgtggctttggtatagataat
actctttctttaaatctacagatattcacagcacattggggtcacctaactataatctta
atatgggtatcaagcaatctttaccacatagcatccaatgctaactattctctctgggtt
aaaaatccaattccaagcatacctatagcacataatatttgggatccacattttactaac
tctacttccactccatattcccataccatcatcaccacaatcctcattgcttactcaggt
atttataaccaactatacacttctggatttaacactattaatcagatatacaagaccaca
tttacatcctcttgcctggcagtaatatcaatactattagctaagatacatatcaatact
cattcagaactattacacaagcttgcaactcatacaagtcaaatcccatcattcttccag
cttctctactttctggatgtcggtatctcatctgttaatattcgattcaattttcacact
ggaattctagttggacttttctcaatagcacacacaggacatcttctagacatcacaata
ccagcctctcgagctccactaattcatacctcactctcttacttaacattctttggtggt
ttaaaatcgaatacaacttcattgtaccttacagatattgcacatcatcacttagcaatt
ggtatcacatccatccatacagggcatctatactcctctttcagggcagcccttggaaca
tatatacgagatatattatatacatcacaccttacacacagcataaaatcactacactta
gccctctcactcattctagccagttgtacagcacttacatctaccaccactcagcacatc
tattctttaacaccatacttttatttgtcttatgatcacatctattttacagctctatat
gtccatcactcctacatcacatcattcttaaccattggttctcatgcacatacagctatc
accttagtaagagattggatcacaccattagaacaagaatccagctctaagcagatacga
atacatacacataaagctgccattattccacacttatcctgggtcagtctttggcttggt
tttcacacactagcagtatactctcataatgacacatgtatagcatttaactctccatct
aagcaaatcttaatagaagcaagcaatgctcaacttattcaacaagcatcaggaaaagct
ttatatgggaccatcaactccatcaacaactataataaatcatttgattcatttatacac
cctatcagtccaggtgatttatatgtacatcatgcaatagcccttggtttacatataact
gtcctcatcctactaaagggaggtcttgaagctcgtggttctaagctaatgccagacaag
atggagcactcatttggcttttcatgtgatggtccaggtagaggtggcacatgtgatata
tcagcttgggattccttttatctagcaacattctggatgcttaatagcaacacatggata
agcttctacttccactacaagcaccttacacctcgtcaattctcagaaagttcaacttat
ctagaatcatggtttcgagattatctgtggtttaactctacacctttaattcatggttat
tctacactaggaactaatgatctatccgtccaatcctggtccttcctacttactcacctt
gcatgggcaagtggatttatgttccttattagttggagaggttactggcaagaattaatt
gatatcatcctttacatacatttgaaaacaccaattcttattaatctttggaatggagat
atctatacacctctagccttatctattgtacaagctagatttattgggcttgttcacttc
tcaaccggtctaattttaacttaccctccttttataataggtgctacaagttag
>Hd4_psaB
ttttccattgcgaaattacggttagcctccaggccaaatccacatcaaaatcaccaatgc
acacaccaatgcaccttgactgatagctttatatctttaaatggtaggtgtgcgacttca
agatatttacaagtggtgggctctattcatgatatagaatcttactttggtatagataat
actctttctttaaatctacagatattcacagcacattggggtcacctaactataatctta
atatgggtatcaggcaatctttatcacatagcatccaatgctaactattctctctgggtt

aaaaatccaattccaagcatacctatagcacataatatttgggatccacattttactaac
tctacttccactccatattcccataccatcatcaccacaatcctcattgcttactcaggt
atttataaccaactatacacttctggatttaacactattaatcagatatacaagaccaca
tttacatcctcttgcctggcagtaatatcaatactattagctaagatacatatcaatact
cattcagaactattacacaagcttgcaactcatgcaagtcaaatcccatcattcttccag
cttctctactttctggatgtcggtatctcatctgttaatattcgattcaattttcacact
ggaattctagttggacttttctcaatagcacacacaggacatcttctagacatcacaata
ccagcctctcgagctccactaattcatacctcactctcttacttaacattctttggtggt
ttaaaatcgaatacaacttcattgtaccttacagatattgcacatcatcacttagcaatt
ggtatcacattcatccatacagggcatctatactcctctttcagggcagcccttggaaca
tatatacgagatacattatatacatcacaccttacacacagcataaaatcactacactta
gccctctcactcattctagccagttgtacagcacttacatctaccaccactcagcacatc
tattctttaacaccatacttttatttgtcttatgatcacatctattttacagctctatat
gtccatcactcctacatcacatcattcttaaccattggttctcatgcacatacagctatc
accttagtaagagattggatcacaccattagaactagaatccagctctaagcggataaga
atacatacacataaagctgccattattccacacttatcctgggtcagtctttggcttggt
tttcacacactagcagtatactctcataatgacacatgtatagcatttaactctccatct
aagcaaatcttaatagaagcaagcaatgctcaacttattcaacaagcatcaggaaaagct
ttatatgggaccatcaactccatcaacaactataataaatcatttgattcatttatacac
cctatcagtccaggtgattcatatgtacatcatgcaatagcccttggtttacatataact
gtcctcatcctactaaagggaggtcttgaagctcgtggttctaagctaatgccagacaag
atggagcactcatttggcttttcatgtgatggtccaggtagaggtggcacatgtgatata
tcagcttgggattccttttatctagcaacattctggatgcttaatagcaacacatggata
agcttctacttccactacaagcaccttacacctcgtcaattctcagaaagttcaacttat
ctagaatcatggtttcgagattatctgtggtttaactctacacctttaattcatggttat
tctacactaggaactaatgatctatccgtccaatcctggtccttcctacttactcacctt
gcatgggcaagtggatttatgttccttattagttggagaggttactggcaagaattaatt
gatatcatcctctacatacatttgaaaacaccaattcttatcaatctttggaatggagac
atctatacacctctcgccttatctattgtacaagctagatttattgggcttgttcacttc
tcaaccggtctaattttaacttaccctccttttataataggtgctacaagttag
>Hd5_psaB
ttttccattgcgaaattacggttagcctccaggccaaatccacatcaaaatcaccaatgc
acacaccaatgcaccttgactgatagctttatatgcttaaatggtaggtgtgcgacttca
agatatttacaagtggtgggctctattcatgatatagaatcttactttggtatagataat
actctttctttaaatctacagatattcacagcacattggggtcacctaactataatctta
atatgggtatcaggcaatctttatcacatagcatccaatgctaactattctctctgggtt
aaaaatccaattccaagcatgcctatagcacataatatttgggatccacattttactaac
tctacttccactccatattcccataccatcatcaccacaatcctcattgcttactcaggt
atttataaccaactatacacttctggatttaacactattaatcagatatacaagaccaca
tttacatcctcttgcctggcagtaatatcaatactattagctaagatacatatcaatact
cattcagaactattacacaagcttgcaactcatgcaagtcaaatcccatcattcttccag
cttctctactttctggatgtcggtatctcatctgttaatattcgattcaattttcacact
ggaattctagttggacttttctcaatagcacacacaggacatcttctagacatcacaata
ccagcctctcgagctccactaattcatacctcactctcttacttaacattctttggtggt
ttaaaatcgaatacaacttcattgtaccttacagatattgcacatcatcacttagcaatt
ggtatcacattcatccatacagggcatctatactcctctttcagggcagcccttggaaca
tatatacgagatacattatatacatcacaccttacacacagcataaaatcactacactta
gccctctcactcattctagccagttgtacagcacttacatctaccaccactcagcacatc
tattctttaacaccatacttttatttgtcttatgatcacatctattttacagctctatat
gtccatcactcctacatcacatcattcttaaccattggttctcatgcacatacagctatc
accttagtaagagattggatcacaccattagaactagaatccagctctaagcggataaga
atacatacacataaagctgccattattccacacttatcctgggtcagtctttggcttggt
tttcatacactagcagtatactctcataatgacacatgtatagcatttaactctccatct
aagcaaatcttaatagaagcaagcaatgctcaacttattcaacaagcatcaggaaaagct
ttatatgggaccatcaactccatcaacaactataataaatcatttgattcatttatacac
cctatcagtccaggtgattcatatgtacatcatgcaatagcccttggcttacatataact
gtcctcatcctactaaagggaggtcttgaagctcgtgggtctaagctaatgccagacaag

atggagcactcatttggcttttcatgtgatgggccaggtagaggtggcacatgtgatata
tcagcttgggattccttttatctagcaacattctggatgcttaatagcaacacatggata
agcttctacttccactacaagcaccttacacctcgtcaattctcagaaagttcaacttat
ctagaatcatggtttcgagattatctgtggtttaactctacacctttaattcatggttat
tctacactaggaactaatgatctatccgtccaatcctggtccttcctacttactcacctt
gcatgggcaagtggatttatgttccttattagttggagaggttactggcaagaattaatt
gatatcatcctctacatacatttgaaaacaccaattcttatcaatctttggaatggagac
atctatacacctctcgccttatctattgtacaagctagatttattgggcttgttcacttc
tcaactgggttaattctaacttaccctccttttataataggtgctacaagttag
>Hd6_psaB
ttttccattgcgaaattacggttagcctccaggccaaatccacatcaaaatcaccaatgc
acacaccaatgcaccttgactgatagctttatatgcttaaatggtagatgtgctagttca
agatatttacaagtggtgggctctattcatgatatagaatcttactttggtatagataat
actctttctttaaatctacaaatattcacagcacattggggccacctaactataatctta
atatgggtatcaagcaatctttatcacatagcatccaatgctaactattctctctgggtt
aaaaatccaattccaagcatacctatagcacataatatttgggatccacattttactaac
tctacttccactccatattcccataccatcatcaccacaatcctcattgcttactcaggt
atttataaccaactatacacttctggatttaacactattaatcagatatacaagaccaca
tttacatcctcttgcctggcagtaatatcaatactattagctaagatacatatcaatact
cattcagaactattacacaagcttgcaactcatgcaagtcaaatcccatcattcttccag
cttctctactttctggatgtggccatctcatctgttaatattcgattcaattttcacact
ggaattctagttggacttttctcaatagggtacacaggacatcttctagacatcacaata
ccagcctctcgagctccactaattcatacctcaccctcttacttaacattctttggtggt
ttaaaatcgaatacaacttcattgtaccttacagatattgcacatcatcacttagcaatt
ggtatcacattcatccatacagggcatctatactcctctttcagggcagcccttggaaca
tatatacgagatacattatatacatcacaccttacacacagcataaaatcactacactta
gccctctcactcattctagccagttgtacagcacttacatctaccaccactcagcacatc
tattctttaacaccatacttttatttgtcttatgatcacatctattttacagctctctat
gtccatcactcctacatcacatcattcttagccattgcttctcatgcacatacagctatc
accttagtaagagattggatcacaccattagaactagaatccagctctaagcggatacga
atccatacccataaagctgccattatttcacacttatcctgggtcagtctttggcttggt
tttcacacactagcagtatactctcataatgacacatgtatagcatttaactctccatct
aagcaaatcttaatagaagcaagcaatggtcaacttattcaacaagcatcaggaaaagct
ttatatgggaccatcaactccatcaacaactataataaatcatttgattcatttatacac
cctatcagtccaggtgattcatatgtacatcatgcaatagcacttggcttacatataact
gtcctcatcctactaaagggaggtcttgaagctcgtgggtctaagctaatgccagacaag
atggagcactcatttggcttttcatgtgatggtccaggtagaggtggcacatgtgatata
tcagcttgggattccttttatctagcaacattctggatgcttaatagcaacacatggata
agcttctacttccactacaagcaccttacacctcgtcaattctcagaaagttcaacttat
ctagaatcatggtttcgagattatctgtggtttaactctacacctttaattcatggttat
tctacactaggagctaatgatctatccgtccaatcttggtccttcctacttactcacctt
gcatgggcaagtggatttatgttccttattagttggagaggttactggcaagaattaatt
gatatcatcctctacatacatttgaaaacaccaattcttatcaatctttggaatggagac
atctatacacctctcgccttatctattgtacaagctagatttattgggcttgttcacttc
tcaaccggtctaattttaacttaccctccttttataataggtgctacaagttag
>Hd7_psaB
ttttccattgcgaaattacggttagcctccaggccaaatccacatcaaaatcaccaatgc
acacaccaatgcaccttgactgatagctttatatgtttaaatggtaggtgtgcgacttca
agatatttacaagtggtgggctctattcatgatatagaatgtggctttggtatagataat
actctatctttaaatctacaaatattcacagcacattggggccacctaactataatctta
atatgggtatcaggcaatctttatcacatagcatccaatgctaactattctctctgggtt
aaaaatccaattccaggcatacctatagcacataatatttgggatccacattttactaac
tctacttccactccatattcccataccattatcaccacaatcctcattgcttactcaggt
atttataaccaactatacacttctggatttaacactattaatcagatatacaagaccaca
tttacatcctcttgcctggcagtaatatcaatactattagctaagatacatatcaatact
cattcagaactattacacaagcttgcaactcatgcaagtcaaatcccatcattcttccag
cttctctactttctggatgtcggtatctcatctgttaatattcgattcaattttcacact

ggaattctagttggacttttctcaatagcgcacacaggacatcttctagacatcacaata
ccagcctctcgagctccactaattcacacctcaccctcttacttaacattctttggtggt
ttaaaatcgaatacaacttcattgtaccttacagatattgcacatcatcacttagcaatt
ggtatcatatccatccatacaggccatctatactcctctttcagggcagcccttggaaca
tatataagagatacattatatacatcacaccttacacacagcataaaatcactacactta
gccctctcactcattctagccagttgtacagcacttacatctaccaccactcagcacatc
tattctttaacaccatacttttatttgtcttatgatcacatctattttacagctctatat
gtccatcactcctacatcacatcattcttaaccattggttctcatgcacatacagctatc
accttagtaagagattgggtcgcaccattagaacaagaatccagctctaagcagatacga
atacatacacataaagctgccattattccacacttatcctgggtcagtctttggcttggt
tttcacacactagcagtatactctcataatgacacatgtatagcatttaactctccatct
aagcaaatcttaatagaagcaagcaatgctcaacttattcaacaagcatcaggaaaagct
ttatatgggaccatcaactccatcaacaactataataaatcatttgattcatttatacac
cctatcagtccaggtgatttatatgtacatcatgcaatagcccttggtttacatataact
gtcctcatcctactaaagggaggtcttgaagctcgtgggtctaagctaatgccagacaag
atggagcactcatttggcttttcatgtgatgggccaggtagaggtggaacatgtgatata
tcagcttgggattccttttatctagcaacattctggatgcttaatagcaacacatggata
agcttctacttccactacaagcaccttacacctcgtcaattctcagaaagttcaacttat
ctagaatcatggtttcgagattatctgtggtttaattctacacctttaattcatggttat
tctacactaggaactaatgatctatccgtccaatcctggtccttcctacttactcacctt
gcttgggcaagtggatttatgttccttattagttggagaggttactggcaagaattaatt
gatatcatcctttacatacatttgaaaacaccaattcttattaatctttggaatggagat
atctatacacctctagccttatctattgtacaagctagatttattgggcttgttcacttc
tcaactgggttaattctaacttaccctccttttataataggtgctacaagttag
>Hd8_psaB
ttttccattgcgaaattacggttagcctccaggccaaatccacatcaaaatcaccaatgc
acacaccaatgcaccttgactgatagctttatatgcttaaatggtagatgtgctagttca
agatatttacaagttctgggttctattcatgatatagaatgtggctttggtatagataat
actctatctttaaatctacaaatattcacagcacattggggccacctaactataatctta
atatgggtatcaggcaatctttatcacatagcatccaatgctaactattctctctgggtt
aaaaatccaattccaagcatacctatagcacataatatttgggatccacattttactaac
tctacttccactccatattcccataccatcatcaccacaatcctcattgcttactcaggt
atttataaccaactatacacttctggatttaacactattaatcagatatacaagaccaca
tttacatcctcttgcctggcagtaatatcaatactattagctaagatacatatcaatact
cattcagaactattacacaagcttgcaactcatgcaagtcaaatcccatcattcttccag
cttctctactttctggatgtcggtatctcatctgttaatattagattcaatttccacact
ggaattctagttggacttttctcaatagcacacacaggacatcttctagacatcacaata
ccagcctctcgagctccactaattcatacctcactctcttacttaacattctttggtggt
ttaaaatcgaatacaacttcattgtaccttacagatattgcacatcatcacttagcaatt
ggtatcacattcatccatacagggcatctatactcctctttcagggcagcccttggaaca
tatatacgagatacattatatacatcacaccttacacacagcataaaatcactacactta
gccctctcactcattctagccagttgtacagcacttacatctaccaccactcagcacatc
tattctttaacaccatatttttatttgtcttatgatcacatctattttacagctctatat
gtccatcactcctacatcacatcattcttaaccattggttctcatgcacatacagctatc
accttagtaagagattggatcgcaccattagaacaagaatccagctctaagcggatacga
atacatacacataaagctgccattattccacacttatcctgggtcagtctttggcttggt
tttcacacactagcagtatactctcataatgacacatgtatagcatttaactctccatct
aagcaaatcttaatagaagcaagcaatggtcaacttattcaacaagcatcaggaaaagct
ttatatgggaccatcaactccatcaacaactataataaatcatttgattcatttatacac
cctatcagtccaggtgattcatatgtacatcatgcaatagcacttggcttacatataact
gtcctcatcctactaaagggaggtcttgaagctcgtgggtctaagctaatgccagacaag
atggagcactcatttggcttttcatgtgatggtccaggtagaggtggcacatgtgatata
tcagcttgggattccttttatctagcaacattctggatgcttaatagcaacacatggata
agcttctacttccactacaagcaccttacacctcgtcaattctcagaaagttcaacttat
ctagaatcatggtttcgagattatctgtggtttaactctacacctttaattcatggttat
tctacactaggaactaatgatctatccgtccaatcctggtccttcctacttactcacctt
gcatgggcaagtggatttatgttccttattagttggagaggttactggcaagaattaatt

gatatcatcctttacatacatttgaaaacaccaattcttatcaatctttggaatggagac
atctatacacctctcgccttatctattgtacaagctagatttattgggcttgttcacttc
tcaaccgggctaattttaacttaccctccttttataataggtgctacaagttag
>Hd9_psaB
ttttccattgcgaaattacggttagcctccaggccaaatccacatcaaaatcaccaatgc
acacaccaatgcaccttgactgatagctttatatctttaaatggtaggtgtgctacttca
agatatttacaagtggtgggctctattcatgatatagaatcttactttggtatagataat
actctttctttaaatctacagatattcacagcacattggggtcacctaactataatctta
atatgggtatcaggcaatctttatcacatagcatccaatgctaactattctctctgggtt
aaaaatccaattccaagcatgcctatagcacataatatttgggatccacattttactaac
tctacttccactccatattcccataccatcatcaccacaatcctcattgcttactcaggt
atttataaccaactatacacttctggatttaacactattaatcagatatacaagaccaca
tttacattctcttgccttgcagtaatttcaatactattagccaagatacacatcaacact
cattcagaactattacataagcttgcaagtcatacaagtcaaatcccatcattcttccag
cttctctactttctggatgtcggtatctcatctgttaatattcgattcaattttcacact
ggaattctagttggacttttctcaatagcacacacaggacatcttctagacatcacaata
ccagcctctcgagctccactaattcatacctcaccctcttacttaacattctttggtggt
ttaaaatcgaatacaagttcattgtaccttacagatattgcacatcatcacttagcaatt
ggtatcacattcatccatacagggcatctatactcctctttcagggcagcccttggaaca
tatatacgagatacattatatacatcacaccttacacacagcataaaatcactacactta
gccctctcacttattctagccagttgtacagcacttacatctaccaccactcagcacatc
tattctttaacaccatacttttatttgtcttatgatcacatctattctacggctctctat
gtccatcactcctacatcacctcattcttagctattgcttctcatgcacatactgctatc
accttagtaagagattgggtcgcaccattagaacaagaatccagctctaagcagataaga
atacatacccataaagctgccattattccacacttatcctgggtcagtctttggcttggt
tttcatacactagcagtatactctcataatgacacatgtatagcatttaactctccatct
aagcaaatcttaatagaagcaagcaatgctcaacttattcaacaagcatcaggaaaagct
ttatatgggaccatcaactccatcaacaactataataaatcatttgattcatttatacac
cctatcagtccaggtgatttatatgtacatcatgcaatagcccttggtttacatataact
gtcctcatcctactaaagggaggtcttgaagctcgtggttctaagctaatgccagacaag
atggagcactcatttggcttttcatgtgatggtccaggtagaggtggcacatgtgatata
tcagcttgggattccttttatctagcaacattctggatgcttaatagcaacacatggata
agcttctacttccactacaagcaccttacacctcgtcaattctcagaaagttcaacttat
ctagaatcatggtttcgagattatctgtggtttaattctacacctttaattcatggttat
tctacactaggagctaatgatctatccgtccaatcttggtccttcctacttactcatctt
gcttgggcaagtggatttatgttccttattagttggagaggttactggcaagaattaatt
gatatcatcctttacatacatttgaaaacaccaattcttatcaatctttggaatggagac
atctatacacctctcgccttatctattgtacaagctagatttattgggcttgttcacttc
tcaaccggtttaattttaacttaccctccttttataataggtgctacaagttag
>IS1_psaB
ttttccattgcgaaattacggttagcctccaggccaaatccacatcaaaatcaccaatgc
acacaccaatgcaccttgactgatagctttatatgcttaaatggtagatgtgctagttca
agatatttacaagttctgggttctattcatgatatagaatgtggctttggtatagataat
actctatctttaaatctacaaatattcacagcacattggggccacctaactataatctta
atatgggtatcaagcaatctttaccacatagcatccaatgctaactattctctctgggtt
aaaaatccaattccaagtatgcctatagcacacaatatttgggatccacatttcactaac
tctacttccactccatattcccataccattatcaccacaatcctcattgcttactcaggt
atttataaccaattatacacttctggatttaacactattaatcagatatataagaccaca
tttacactttcttgtttggcagtaatatcaatactattagccaagatacacatcaacact
cattcagaactattacataagcttgcaagtcatacaagtcaaatcccatcattcttccag
cttctctactttctggatgtggccatctcatctgttaatattagattcaatttccacact
ggaattctagttggacttttctcaatagggtacacaggacatcttctagacatcacaata
ccagcctctcgagctccactaattcacacctcaccctcttacttaacattctttggtggt
ttaaaatcgaatacaagttcattgtaccttacagatattgcacatcatcacttagcaatt
ggtatcatatctatccttacaggccatctatactcctctttcagggcagcccttggaaca
tatataagagatatattatatacatcacaccttacacacagcataaaatcactccactta
gccctctcacttattctagccagttgtacaccacttacatcaaccactgctcagcacatc

tattctttaacaccatatttttatttgtcttatgatcacgtctattctgcagctctctat
gtccatcactcctatatcacatcattcttagctattgcttctcatgcacatactgctatc
accttagtaagagattgggtcgcaccattagaacaagaatccagctctaagcagatacga
atccatacccataaagctgccattatttcacacttatcctggatcagtctttggcttggt
tttcacacactagcagtatactctcataatgacacatgtatagcatttaactctccatct
aagcaaatcctaatagaagcaagcaatggtcaacttattcaacaagcatcaggaaaagct
ttatatgggaccatcaactccatcaacaactataataaatcatttgattcatttatacac
cctatcagtccaggtgatttatatgtacatcatgcaatagcccttggtttacatataact
gtcctcatcctactaaagggaggtcttgaagctcgtggttctaagctaatgccagacaag
atggagcactcatttggcttttcatgtgatgggccaggtagaggtggaacgtgtgatata
tctgcttgggattctttttatctagcaacattctggatgcttaatagtaatgcatggata
agcttctacttccactacaagcaccttacacctcgtcaattctcagaaagttcaacttat
ctagaatcatggtttcgagattatctgtggtttaattctacacctttaattcatggttat
tctacactaggagctaatgatctatccgtccaatcttggtccttcctccttactcatctt
gcatgggcaagtggatttatgttccttattagttggagaggttactggcaggaattaatt
gatatcatcctttacatacatttgaaaacaccaattcttattaatctttggaatggagat
atctatacacctctcgccttatctattgtacaagctagatttattgggcttgttcacttc
tcaactgggttaattctaacttaccctccttttataataggtgctacaagttag
>IS2_psaB
ttttccattgcgaaattacggttagcctccaggccaaatccacatcaaaatcaccaatgc
acacaccaatgcaccttgactgatagctttatatgcttaaatggtagatgtgctagttca
agatatttacaagtggtgggctctattcatgatatagaatcttactttggtatagataat
actctttctttaaatctacagatattcacagcacattggggtcacctaactataatctta
atatgggtatcaggcaatctttatcacatagcatccaatgctaactattctctctgggtt
aaaaatccaattccaagcatacctatagcacataatatttgggatccacattttactaac
tctacttccactccatattcccataccatcatcaccacaatcctcattgcttactcaggt
atttataaccaactatacacttctggatttaacactattaatcagatatacaagaccaca
tttacatcctcttgcctggcagtaatatcaatactattagctaagatacatatcaatact
cattcagaactattacacaagcttgcaactcatgcaagtcaaatcccatcattcttccag
cttctctactttctggatgtcggtatctcatctgttaatattcgattcaattttcacact
ggaattctagttggacttttctcaatagcacacacaggacatcttctagacatcacaata
ccagcctctcgagctccactaattcatacctcactctcttacttaacattctttggtggt
ttaaaatcgaatacaacttcattgtaccttacagatattgcacatcatcacttagcaatt
ggtatcacattcatccatacagggcatctatactcctctttcagggcagcccttggaaca
tatatacgagatacattatatacatcacaccttacacacagcataaaatcactacactta
gccctctcactcattctagccagttgtacagcacttacatctaccaccactcagcacatc
tattctttaacaccatacttttatttgtcttatgatcacatctattttacagctctatat
gtccatcactcctacatcacatcattcttaaccattggttctcatgcacatacagctatc
accttagtaagagattggatcacaccattagaactagaatccagctctaagcggataaga
atacatacacataaagctgccattattccacacttatcctgggtcagtctttggcttggt
tttcatacactagcagtatactctcataatgacacatgtatagcatttaactctccatct
aagcaaatcttaatagaagcaagcaatgctcaacttattcaacaagcatcaggaaaagct
ttatatgggaccatcaactccatcaacaactataataaatcatttgattcatttatatac
cctatcagcccaggtgattcatatgtacatcatgcaatagcacttggcttacatataact
gtcctcatcctactaaagggaggtcttgaagctcgtggttctaagctaatgccagacaag
atggagcactcatttggcttttcatgtgatggtccaggtagaggtggcacatgtgatata
tcagcttgggattccttttatctagcaacattctggatgcttaatagcaacacatggata
agcttctacttccactacaagcaccttacacctcgtcaattctcagaaagttcaacttat
ctagaatcatggtttcgagattatctgtggtttaactctacacctttaattcatggttat
tctacactaggaactaatgatctatccgtccaatcctggtccttcctacttactcacctt
gcatgggcaagtggatttatgttccttattagttggagaggttactggcaagaattaatt
gatatcatcctctacatacatttgaaaacaccaattcttatcaatctttggaatggagac
atctatacacctctcgccttatctattgtacaagctagatttattgggcttgttcacttc
tcaaccggtctaattttaacttaccctccttttataataggtgctactagttag
>IS3_psaB
ttttccattgcgaaattacggttagcctccaggccaaatccacatcaaaatcaccaatgc
acacaccaatgcaccttgactgatagctttatatgcttaaatggtagatgtgctagttca

agatatttacaagttctgggttctattcatgatatagaatgtggctttggtatagataat
actctatctttaaatctacagatctttacagcacattggggtcatctaactataatctta
atatgggtatcaagtaatctttatcacatagcctccaatgctaactattctctctgggtt
caaaatccaattccaagcatacctatagcacacaatatttgggacccccacttcactagc
tccacttccactccatattcccataccattatcaccacaatcctcattgcttactcaggt
atctataaccaactatacacttctggatttaactctattaatcagatatataagaccaca
tttacactttcttgtttggcagtaatatcaatactattagccaagatacacatcaacact
cattcagaactactacacaagcttgcaactaatacaagtcaaatcccatcattcttccag
cttctctactttcttgatgttgccatctcatctgttaatattcgattcaattttcacact
ggaattctagttggacttttctcaatagcacacacaggacatcttctagacatcacaata
ccagcctctcgagctccactaattcacacctcaccctcttacttaacattcctgggtggt
ttaaaatcgaatacaacttcattgtaccttacagatattgcacatcatcacttagcaatt
ggtatcacatccatacacacaggtcatctatactcctctttcagggcagcccttggaaca
tatatacgagatacattatatacatcacaccttacacatagtataaaatcactccactta
gccctctcactcattctagccagttgtgcagcacttacatctatcaccactcaacacatc
tattctttaacaccttacttttatttgtcttatgatcacgtctattctgcagctctctat
gtccatcactcctatatcacatcattattagcaataggttcacatgcacatgcagctatc
accttagtaagagattggatcacaccattagaactagaatccagctctaagcagatacga
atccatacccataaagctgccattattccacacttatcctggattagtctttggcttggt
tttcacacactagcagtatactctcataatgacacatgtatagcatttaactctccatct
aagcaaatcttaatagaagcaagcaatggtcaacttattcaacaagcatcaggaaaagct
ttatatgggaccatcaactccatcaacaactataataaatcatttgattcatttatatac
cctatcagcccaggtgatttatatgtacatcatgcaatagcactgggtttgcatgttact
gtactcatcctactaaagggagggcttgaagctcgtgggtctaagctaatgccagacaag
atggaacactcatttggcttttcatgtgatggtccaggtagaggtggtacgtgtgatata
tctgcttgggattctttttatctagcaacattctggatgcttaatagtaatgcatggata
agcttctacttccactacaagcaccttacacctcgccaattctcagaaagttcaacttat
ctagaatcgtggtttcgagattatctgtggtttaattctacacctttaattcacggttat
tctacactagggactaatgacttatctgtacagtcttggtccttcctccttactcatctt
gcatgggcaagtggatttatgttccttattagttggagaggttactggcaggaattaatt
gatatcatcctctacatacatttgaaaacaccaattcttataaatctttggaatggagac
atctatacacctctcgccttatctattgtacaagctagatttattgggcttgttcacttc
tcaactggtctaattttaacttaccccccttttataataggtgctacaagttag
>IS4_psaB
ttttccattgcgaaattacggttagcctccaggccaaatccacatcaaaatcaccaatgc
acacaccaatgcaccttgactgatagctttatatgcttaaatggtagatgtgctagttca
agatatttacaagttctgggttctattcatgatatagaatgtggctttggtatagataat
actctatctctaaatctacagatctttacagcacattggggtcatctaactataatctta
atatgggtatcaagtaatctttatcacatagcctccaatgctaactattctctctgggtt
caaaatccaattccaagcatacctatagcacataatatttgggatccccactttactagc
tccacttccactccatattcccataccatcatcaccacaatcctcattgcttattcagga
atctataaccaactatacacttctggatttaactctattaatcagatatataagaccaca
tttacactttcttgtttggcagtaatatcaatactattagccaagatacacatcaacact
cattcagaactactacacaagcttgcaactcatacaagtcaaatcccatcattcttccag
cttctctactttcttgatgttggcatctcatctgttaatattcgattcaattttcacact
ggaattctagttggacttttctcaatagcacacacaggacatcttctagacatcacaata
ccagcctctcgagctccactaattcatacctcactctcttacttaacattcctgggtggt
ttaaaatcgaatacaacttcattgtaccttacagatattgcacatcatcacttagcaatt
ggtatcacattcatccatacaggtcatctatactcctctttcagggcagcccttggaaca
tatatacgagatacattatatacatcacaccttacacacagcataaaatcactccactta
gccctctcactcattctagccagttgtgcagcacttacatctatcaccactcaacacatc
tattctttaacaccttacttttatttgtcttatgatcacgtctattctgcggctctctat
gtccatcactcctatatcacatcattattagcaataggttcacatgcacatgcagctatc
accttagtaagagattggatcacaccattagaactagaatccagctctaagcggatacga
atccatacacataaagctgccattattccacacttatcctggattagtctttggcttggt
tttcacacactagcagtatactctcataatgacacatgtatagcatttaactctccatct
aagcaaatcttaatagaagcaagcaatgctcaacttattcaacaagcatcaggaaaagct

ttatatgggaccatcaactccatcaacaactataataaatcatttgattcatttatatac
cctatcagcccaggtgattcatatgtacatcatgcaatagcacttggcttacatattact
gtactcatcctactaaaaggagggcttgaagctcgtgggtctaagctaatgccagacaag
atggaacactcatttggcttttcatgtgatggtccaggtagaggtggtacgtgtgatata
tctgcttgggattctttttatctagcaacattctggatgcttaatagtaacgcatggata
agcttctacttccactacaagcaccttacacctcgccaattctcagaaagttcaacttat
ctagaatcgtggtttcgagattatctgtggtttaattctacacctttaattcacggttat
tctacactagggactaatgacttatctgtacagtcttggtccttcctccttactcacctt
gcatgggcaagtggatttatgttccttattagttggagaggttactggcaggaattaatt
gatatcatcctctacatacatttgaaaacaccaattcttataaatctttggaatggagac
atctatacacctctcgccttatctattgtacaagctagatttattgggcttgttcacttc
tcaactgggttaattttaacttacccccccttttataataggtgctacaagttag
>IS5_psaB
ttttccattgcgaaattacggttagcctccaggccaaatccacatcaaaatcaccaatgc
acacaccaatgcaccttgactgatagctttatatgcttaaatggtagatgtgctagttca
agatatttacaagttctgggttctattcatgatatagaatgtggctttggtatagataat
actctttctttaaatctacagatattcacagcacattggggtcacctaactataatctta
atatgggtatcaagcaatctttaccacatagcatccaatgctaactattctctctgggtt
aaaaatccaattccaagcatacctatagcacataatatttgggatccacattttactaac
tctacttccactccatattcccataccatcatcaccacaatcctcattgcttactcaggt
atttataaccaactatacacttctggatttaacactattaatcagatatacaagaccaca
tttacatcctcttgcctggcagtaatatcaatactattagctaagatacatatcaatact
cattcagaactattacacaagcttgcaactcatgcaagtcaaatcccatcattcttccag
cttctctactttctggatgtcggtatctcatctgttaatattcgattcaattttcacact
ggaattctagttggacttttctcaatagggtacacaggacatcttctagacatcacaata
ccagcctctcgagctccactaattcatacctcactctcttacttaacattctttggtggt
ttaaaatcgaatacaacttcattgtaccttacagatattgcacatcatcacttagcaatt
ggtatcatatctatccttacaggccatctatactcctctttcagggcagcccttggaaca
tatatacgagatacattatatacatcacaccttacacacagcataaaatcactacactta
gccctctcactcattctagccagttgtacagcacttacatctaccaccactcagcacatc
tattctttaacaccatacttttatttgtcttatgatcacatctattttacagctctctat
gtccatcactcctacatcacctcattcttagctattgcttctcatgcacatactgctatc
accttagtaagagattgggtcgcaccattagaacaagaatccagctctaagcagatacga
atccatacccataaagctgccattattccacacttatcctgggtcagtctttggcttggt
tttcacacactagcagtatactctcataatgacacatgtatagcatttaactctccatct
aagcaaatcttaatagaagcaagcaatgctcaacttattcaacaagcatcaggaaaagct
ttatatgggaccatcaactccatcaacaactataataaatcatttgattcatttatacac
cctatcagtccaggtgattcatatgtacatcatgcaatagcacttggcttacatataact
gtcctcatcctactaaagggaggtcttgaagctcgtgggtctaagctaatgccagacaag
atggagcactcatttggcttttcatgtgatgggccaggtagaggtggcacatgtgatata
tcagcttgggattccttttatctagcaacattctggatgcttaatagcaacacatggata
agcttctacttccactacaagcaccttacacctcgtcaattctcagaaagttcaacttat
ctagaatcatggtttcgagattatctgtggtttaattctacacctttaattcatggttat
tctacactaggagctaatgatctatccgtccaatcttggtccttcctacttactcatctt
gcttgggcaagtggatttatgttccttattagttggagaggttactggcaagaattaatt
gatatcatcctctacatacatttgaaaacaccaattcttattaatctttggaatggagat
atctatacacctctagccttatctattgtacaagctagatttattgggcttgttcacttc
tcaactgggttaattctaacttaccctccttttataataggtgctacaagttag
>IS6_psaB
ttttccattgcgaaattacggttagcctccaggccaaatccacatcaaaatcaccaatgc
acacaccaatgcaccttgactgatagctttatatgcttaaatggtagatgtgctagttca
agatatttacaagttgtgggctctattcatgatatagaatcttactttggtatagataat
actctttctttaaatctacagatattcacagcacattggggtcacctaactataatctta
atatgggtatcaggcaatctttaccacatagcatccaatgctaactattctctctgggtt
aaaaatccaattccaagcatacctatagcacacaatatttgggatccacatttcactaac
tctacttccactccatattcccataccatcatcaccacaatcctcattgcttactcaggt
atttataaccaactatacacttctggatttaacactattaatcagatatacaagaccaca

tttacatcctcttgcctggcagtaatatcaatactattagctaagatacacatcaacact
cattcagaactattacacaagcttgcaactcatgcaagtcaaatcccatcattcttccag
cttctctactttctggatgtcggtatctcatctgttaatattcgattcaattttcacact
ggaattctagttggacttttctcaatagggtacacaggacatcttctagacatcacaata
ccagcctctcgagctccactaattcatacctcactctcttacttaacattctttggtggt
ttaaaatcgaatacaacttcattgtaccttacagatattgcacatcatcacttagcaatt
ggtatcacattcatccatacagggcatctatactcctctttcagggcagcccttggaaca
tatatacgagatacattatatacatcacaccttacacacagcataaaatcactacactta
gccctctcactcattctagccagttgtacagcacttacatctaccaccactcagcacatc
tattctttaacaccatacttttatttgtcttatgatcacatctattttacagctctatat
gtccatcactcctacatcacatcattcttaaccattggttctcatgcacatacagctatc
accttagtaagagattgggtcgcaccattagaacaagaatccagctctaagcagataaga
atacatacacataaagctgccattattccacacttatcctgggtcagtctttggcttggt
tttcacacactagcagtatactctcataatgacacatgtatagcatttaactctccatct
aagcaaatcttaatagaagcaagcaatgctcaacttattcaacaagcatcaggaaaagct
ttatatgggaccatcaactccatcaacaactataataaatcatttgattcatttatacac
cctatcagtccaggtgattcatatgtacatcatgcaatagcacttggcttacatataact
gtcctcatcctactaaagggaggtcttgaagctcgtgggtctaagctaatgccagacaag
atggagcactcatttggcttttcatgtgatggtccaggtagaggtggcacatgtgatata
tcagcttgggattccttttatctagcaacattctggatgcttaatagcaacgcatggata
agcttctacttccactacaagcaccttacacctcgtcaattctcagaaagttcaacttat
ctagaatcatggtttcgagattatctgtggtttaactctacacctttaattcatggttat
tctacactaggaactaatgatctatccgtccaatcctggtccttcctacttactcacctt
gcatgggcaagtggatttatgttccttattagttggagaggttactggcaagaattaatt
gatatcatcctctacatacatttgaaaacaccaattcttatcaatctttggaatggagac
atctatacacctctcgccttatctattgtacaagctagatttattgggcttgttcacttc
tcaaccggtctaattttaacttaccctccttttataataggtgctacaagttag
>IS7_psaB
ttttccattgcgaaattacggttagcctccaggccaaatccacatcaaaatcaccaatgc
acacaccaatgcaccttgactgatagctttatatgtttaaatggtaggtgtgcgacttca
agatatttacaagtggtgggctctattcatgatatagaatcttactttggtatagataat
actctttctttaaatctacagatattcacagcacattggggtcacctaactataatctta
atatgggtatcaggcaatctttatcacatagcatccaatgctaactattctctctgggtt
aaaaatccaattccaagtatgcctatagcacacaatatttgggacccccacttcactagc
tccacttccactccatattcccataccatcatcaccacaatcctcattgcttactcaggt
atttataaccaactatacacttctggatttaactctattaatcagatatataagaccaca
tttacattctcttgcctggcagtaatatcaatactattagccaagatacacatcaacact
cattcagaactactacacaagcttgcaagtaatacaagtcaaatcccatcattcttccag
cttctctactttcttgatgttggcatctcatctgttaatattcgattcaattttcacact
ggaattctagttggacttttctcaatagcacacacaggacatcttctagacatcacaata
ccagcctctcgagctccactaattcacacctcactctcttacttaacattctttggtggt
ttaaaatcgaatacaacttcattgtaccttacagatattgcacatcatcacttagcaatt
ggtatcacattcatccatacaggtcatctatactcctctttcagggcagcccttggaaca
tatatacgagatacattatatacatcacaccttacacacagcataaaatcactacactta
gccctctcactcattctagccagttgtacagcacttacatctaccaccactcagcacatc
tattctttaacaccatacttttatttgtcttatgatcacatctattttacagctctatat
gtccatcactcctacatcacatcattcttaaccattggttctcatgcacatacagctatc
accttagtaagagattggatcacaccattagaactagaatccagctctaagcagataaga
atacatacacataaagctgccattattccacacttatcctgggtcagtctttggcttggt
tttcacacactagcagtatactctcataatgacacatgtatagcatttaactctccatct
aagcaaatcttaatagaagcaagcaatgctcaacttattcaacaagcatcaggaaaagct
ttatatgggaccatcaactccatcaacaactataataaatcatttgattcatttatatac
cctatcagcccaggtgatttatatgtacatcatgcaatagccctgggtttgcatgttact
gtactcatcctactaaaaggagggcttgaagctcgtgggtctaagctaatgccagacaag
atggaacactcatttggcttttcatgtgatggtccaggtagaggtggcacatgtgatata
tcagcttgggattccttttatctagcaacattctggatgcttaatagtaatacatggata
agcttctacttccactacaagcaccttacacctcgtcaattctcagaaagttcaacttat

ctagaatcatggtttcgagattatctgtggtttaactctacacctttaattcatggttat
tctacactaggagctaatgatctatccgtccaatcttggtccttcctccttactcatctt
gcatgggcaagtggatttatgttccttattagttggagaggttactggcaagaattaatt
gatatcatcctctacatacattt-aaaacaccaattcttatcaatctttggaatggagac
atctatacacctctcgccttatctattgtacaagctagatttattgggcttgttcacttc
tcaaccggtctaattttaacttacccccttttataataggtgctactagttag
>IS8_psaB
ttttccattgcgaaattacggttagcctccaggccaaatccacatcaaaatcaccaatgc
acacaccaatgcaccttgactgatagctttatatgcttaaatggtaggtgtgcgagttca
agatatttacaagtggtgggctctattcatgatatagaatcttactttggtatagataat
actctttctttaaatctacagatattcacagcacattggggtcacctaactataatctta
atatgggtatcaagcaatctttaccacatagcatccaatgctaactattctctctgggtt
aaaaatccaattccaagcatacctatagcacataatatttgggatccacattttactaac
tctacttccactccatattcccataccatcatcaccacaatcctcattgcttactcaggt
atttataaccaactatacacttctggatttaacactattaatcagatatacaagaccaca
tttacatcctcttgcctggcagtaatatcaatactattagctaagatacatatcaatact
cattcagaactattacacaagcttgcaactcatgcaagtcaaatcccatcattcttccag
cttctctactttctggatgtcggtatctcatctgttaatattcgattcaattttcacact
ggaattctagttggacttttctcaatagcacacacaggacatcttctagacatcacaata
ccagcctctcgagctccactaattcatacctcactctcttacttaacattctttggtggt
ttaaaatcgaatacaacttcattgtaccttacagatattgcacatcatcacttagcaatt
ggtatcacattcatccatacagggcatctatactcctctttcagggcagcccttggaaca
tatatacgagatacattatatacatcacaccttacacacagcataaaatcactacactta
gccctctcactcattctagccagttgtacagcacttacatctaccaccactcagcacatc
tattctttaacaccatacttttatttgtcttatgatcacatctattctacagctctatat
gtccatcactcctacatcacatcattcttaaccattggttctcatgcacatacagctatc
accttagtaagagattgggtcgcaccattagaacaagaatccagctctaagcggataaga
atacatacacataaagctgccattattccacacttatcctgggtcagtctttggcttggt
tttcacacactagcagtatactctcataatgacacatgtatagcatttaactctccatct
aagcaaatcttaatagaagcaagcaatgctcaacttattcaacaagcatcaggaaaagct
ttatatgggaccatcaactccatcaacaactataataaatcatttgattcatttatacac
cctatcagtccaggtgattcatatgtacatcatgcaatagcacttggcttacatataact
gtcctcatcctactaaagggaggtcttgaagctcgtgggtctaagctaatgccagacaag
atggagcactcatttggcttttcatgtgatggtccaggtagaggtggcacatgtgatata
tcagcttgggattccttttatctagcaacattctggatgcttaatagcaacacatggata
agcttctacttccactacaagcaccttacacctcgtcaattctcagaaagttcaacttat
ctagaatcatggtttcgagattatctgtggtttaattctacacctttaattcatggttat
tctacactaggagctaatgatctatccgtccaatcctggtccttcctacttactcacctt
gcatgggcaagtggatttatgttccttattagttggagaggttactggcaagaattaatt
gatatcatcctctacatacatttgaaaacaccaattcttatcaatctttggaatggagac
atctatacacctctcgccttatctattgtacaagctagatttattgggcttgttcacttc
tcaaccggtctaattttaacttaccctccttttataataggtgctacaagttag
>IS9_psaB
ttttccattgcgaaattacggttagcctccaggccaaatccacatcaaaatcaccaatgc
acacaccaatgcaccttgactgatagctttatatgtttaaatggtaggtgtgcgacttca
agatatttacaagtggtgggctctattcatgatatagaatgtggctttggtatagataat
actctatctttaaatctacaaatattcacagcacattggggccatctaactataatctta
atatgggtatcaagtaatctttatcacatagcatccaatgctaactattctctctgggtt
aaaaatccaattccaagtatgcctatagcacacaatatttgggatccacattttactaac
tctacttccactccatattcccataccattatcaccacaatcctcattgcttattcagga
atctataaccaactatacacttctggatttaactctattaatcagatatataagaccaca
tttacactctcttgtctggcagtaatttcaatactattagccaagatacacatcaacact
cattcagaactactacacaagcttgcaactcatgcaagtcaaatcccatcattcttccag
cttctctactttctggatgtcggcatctcatctgttaatattagattcaattttcacact
ggaattctagttggacttttctcaatagcacacacaggacatcttctagacatcacaata
ccagcctctcgagctccactaattcatacctcactctcttacttaacattctttggtggt
ttaaaatcgaatacaacttcattgtaccttacagatattgcacatcatcacttagcaatt

ggtatcatatctatccttacaggccatctatactcctctttcagggcagcccttggaaca
tatatacgagatacattatatacatcacaccttacacacagcataaaatcactacactta
gccctctcactcattctagccagttgtacagcacttacatctaccaccactcagcacatc
tattctttaacaccatacttttatttgtcttatgatcacatctattttacagctctatat
gtccatcactcctatatcacatcattattaaccataggttcacatgcacatgcagctatc
accttagtaagagattggatcacaccattagaactagaatccagctctaagcggataaga
atacatacacataaagctgccattattccacacttatcctggattagtctttggcttggt
tttcatacactagcagtatactctcataatgacacatgtatagcatttaactctccatct
aagcaaatcctaatagaagcaagcaatggtcaacttattcaacaagcatcaggaaaagct
ttatatgggaccatcaactccatcaacaactataataaatcatttgattcatttatacac
cctatcagtccaggtgatttatatgtacatcatgcaatagccctgggtttacatgtaact
gtactcatcctactaaaaggagggcttgaagctcgtgggtctaagctaatgccagacaag
atggaacactcatttggcttttcatgtgatggtccaggtagaggtggtacgtgtgatata
tctgcttgggattctttttatctagcaacattctggatgcttaatagtaatacatggata
agcttctacttccactacaagcaccttacacctcgccaattctcagaaagttcaacttat
ctagaatcatggtttcgagattatctgtggtttaactctacacctttaattcatggttat
tctacactaggaactaatgatctatccgtccaatcttggtccttcctccttactcatctt
gcatgggcaagtggatttatgttccttattagttggagaggttactggcaagaattaatt
gatatcatcctctacatacatttgaaaacaccaattcttatcaatctttggaatggagac
atctatacacctctcgccttatctattgtacaagctagatttattgggcttgttcacttc
tcaactggtttaattctaacttaccctccttttataataggtgctacaagttag
>Ik1_psaB
ttttccattgcgaaattacggttagcctccaggccaaatccacatcaaaatcaccaatgc
acacaccaatgcaccttgactgatagctttatatgcttaaatggtagatgtgctagttca
agatatttacaagtggtgggctctattcatgatatagaatcttactttggtatagataat
actctttctttaaatctacagatattcacagcacattggggtcacctaactataatctta
atatgggtatcaggcaatctttatcacatagcatccaatgctaactattctctctgggtt
aaaaatccaattccaagcatacctatagcacataatatttgggatccacattttactaac
tctacttccactccatattcccataccatcatcaccacaatcctcattgcttactcaggt
atttataaccaactatacacttctggatttaacactattaatcagatatacaagaccaca
tttacatcctcttgcctggcagtaatatcaatactattagctaagatacatatcaatact
cattcagaactattacataagcttgcaagtcatacaagtcaaatcccatcattcttccag
cttctctactttctggatgtcggtatctcatctgttaatattcgattcaattttcacact
ggaattctagttggacttttctcaatagcacacacaggacatcttctagacatcacaata
ccagcctctcgagctccactaattcatacctcactctcttacttaacattctttggtggt
ttaaaatcgaatacaagttcattgtaccttacagatattgcacatcatcacttagcaatt
ggtatcacattcatccatacagggcatctatactcctctttcagggcagcccttggaaca
tatatacgagatacattatatacatcacaccttacacacagcataaaatcactacactta
gccctctcactcattctagccagttgtacagcacttacatctaccaccactcagcacatc
tattctttaacaccatacttttatttgtcttatgatcacatctattctacggctctctat
gtccatcactcctacatcacctcattcttagctattgcttctcatgcacatactgctatc
accttagtaagagattgggtcgcaccattagaacaagaatccagctctaagcagatacga
atccatacccataaagctgccattatttcacacttatcctgggtcagtctttggcttggt
tttcatacactagcagtatactctcataatgacacatgtatagcatttaactctccatct
aagcaaatcctaatagaagcaagcaatggtcaacttattcaacaagcatcaggaaaagct
ttatatgggaccatcaactccatcaacaactataataaatcatttgattcatttatacac
cctatcagtccaggtgattcatatgtacatcatgcaatagcacttggtttacatataact
gtcctcatcctactaaagggaggtcttgaagctcgtgggtctaagctaatgccagacaag
atggagcactcatttggcttttcatgtgatggtccaggtagaggtggcacatgtgatata
tcagcttgggattccttttatctagcaacattctggatgcttaatagcaacacatggata
agcttctacttccactacaagcaccttacacctcgtcaattctcagaaagttcaacttat
ctagaatcatggtttcgagattatctgtggtttaactctacacctttaattcatggttat
tctacactaggagctaatgatctatccgtccaatcctggtccttcctacttactcacctt
gcatgggcaagtggatttatgttccttattagttggagaggttactggcaagaattaatt
gatatcatcctctacatacatttgaaaacaccaattcttatcaatctttggaatggagac
atctatacacctctcgccttatctattgtacaagctagatttattgggcttgttcacttc
tcaactgggttaattctaacttaccctccttttataataggtgctacaagttag

>Ik2_psaB
ttttccattgcgaaattacggttagcctccaggccaaatccacatcaaaatcaccaatgc
acacaccaatgcaccttgactgatagctttatatgcttaaatggtagatgtgctagttca
agatatttacaagttctgggttctattcatgatatagaatgtggctttggtatagataat
actctatctttaaatctacaaatattcacagcacattggggtcacctaactataatctta
atatgggtatcaggcaatctttatcacatagcatccaatgctaactattctctctgggtt
aaaaatccaattccaagtatgcctatagcacacaatatttgggatccacatttcactaac
tctacttccactccatattcccataccattatcaccacaatcctcattgcttactcaggt
atttataaccaactatacacttctggatttaacactattaatcagatatacaagaccaca
tttacattctcttgccttgcagtaatttcaatactattagctaagatacatatcaatact
cattcagaactattacacaagcttgcaactcatgcaagtcaaatcccatcattcttccag
cttctctactttctggatgtggccatctcatctgttaatattcgattcaattttcacact
ggaattctagttggacttttctcaatagcacacacaggacatcttctagacatcacaata
ccagcctctcgagctccactaattcatacctcactctcttacttaacattctttggtggt
ttaaaatcgaatacaacttcattgtaccttacagatattgcacatcatcacttagcaatt
ggtatcatatctatccttacaggccatctatactcctctttcagggcagcccttggaaca
tatatacgagatacattatatacatcacaccttacacacagcataaaatcactacactta
gccctctcacttattctagccagttgtacagcacttacatctaccaccgctcagcacatc
tattctttaacaccatacttttatttgtcttatgatcacatctattctacggctctatat
gtccatcactcctacatcacatcattcttaaccattggttctcatgcacatactgctatc
accttagtaagagattggatcacaccattagaactagaatccagctctaagcggataaga
atacatacacataaagctgccattattccacacttatcctgggtcagtctttggcttggt
tttcacacactagcagtatactctcataatgacacatgtatagcatttaactctccatct
aagcaaatcctaatagaagcaagcaatgctcaacttattcaacaagcatcaggaaaagct
ttatatgggaccatcaactccatcaacaactataataaatcatttgattcatttatacac
cctatcagtccaggtgattcatatgtacatcatgcaatagcacttggcttacatataact
gtcctcatcctactaaagggaggtcttgaagctcgtggttctaagctaatgccagacaag
atggagcactcatttggcttttcatgtgatggtccaggtagaggtggcacatgtgatata
tcagcttgggattccttttatctagcaacattctggatgcttaatagcaacacatggata
agcttctacttccactacaagcaccttacacctcgtcaattctcagaaagttcaacttat
ctagaatcatggtttcgagattatctgtggtttaactctacacctttaattcatggttat
tctacactaggaactaatgatctatccgtccaatcctggtccttcctacttactcacctt
gcatgggcaagtggatttatgttccttattagttggagaggttactggcaagaattaatt
gatatcatcctttacatacatttgaaaacaccaattcttattaatctttggaatggagat
atctatacacctctcgccttatctattgtacaagctagatttattgggcttgttcacttc
tcaaccggtctaattttaacttaccctccttttataataggtgctacaagttag
>Ik3_psaB
ttttccattgcgaaattacggttagcctccaggccaaatccacatcaaaatcaccaatgc
acacaccaatgcaccttgactgatagctttatatgcttaaatggtagatgtgctagttca
agatatttacaagttgtgggctctattcatgatatagaatcttactttggtatagataat
actctttctttaaatctacagatattcacagcacattggggtcacctaactataatctta
atatgggtatcaggcaatctttatcacatagcatccaatgctaactattctctctgggtt
aaaaatccaattccaagcatacctatagcacataatatttgggatccacatttcactaac
tctacttccactccatattcccataccattatcaccacaatcctcattgcttactcaggt
atttataaccaattatacacttctggatttaacactattaatcagatatacaagaccaca
tttacatcctcttgcctggcagtaatatcaatactattagctaagatacatatcaatact
cattcagaactattacacaagcttgcaactcatacaagtcaaatcccatcattcttccag
cttctctactttctggatgtcggtatctcatctgttaatattagattcaatttccacact
ggaattctagttggacttttctcaatagggcacacaggacatcttctagacatcacaata
ccagcctctcgagctccactaattcatacctcaccctcttacttaacattctttggtggt
ttaaaatcgaatacaagttcattgtaccttacagatattgcacatcatcacttagcaatt
ggtatcatatctatccttacaggccatctatactcctctttcagggcagcccttggaaca
tatataagagatacattatatacatcacaccttacacacagcataaaatcactccactta
gccctctcacttattctagccagttgtacagcacttacatctaccaccactcagcacatc
tattctttaacaccatacttttatttgtcttatgatcacatctattttacagctctatat
gtccatcactcctacatcacatcattcttaaccattggttctcatgcacatactgctatc
accttagtaagagattgggtcgcaccattagaacaagaatccagctctaagcggataaga

atacatacacataaagctgccattattccacactcatcctgggtcagtctttggcttggt
tttcacacactagcagtatactctcataatgacacatgtatagcatttaactctccatct
aagcaaatcttaatagaagcaagcaatgctcaacttattcaacaagcatcaggaaaagct
ttatatgggaccatcaactccatcaacaactataataaatcatttgattcatttatacac
cctatcagtccaggtgattcatatgtacatcatgcaatagcacttggcttacatataact
gtcctcatcctactaaagggaggtcttgaagctcgtggttctaagctaatgccagacaag
atggagcactcatttggcttttcatgtgatgggccaggtagaggtggaacatgtgatata
tcagcttgggattccttttatctagcaacattctggatgcttaatagcaacacatggata
agcttctacttccactacaagcaccttacacctcgtcaattctcagaaagttcaacttat
ctagaatcatggtttcgagattatctgtggtttaattctacacctttaattcatggttat
tctacactaggagctaatgatctatccgtccaatcctggtccttcctacttactcacctt
gcatgggcaagtggatttatgttccttattagttggagaggttactggcaagaattaatt
gatatcatcctctacatacatttgaaaacaccaattcttattaatctttggaatggagat
atctatacacctctagccttatctattgtacaagctagatttattgggcttgttcacttc
tcaactgggttaattctaacttaccctccttttataataggtgctacaagttag
>Ik4_psaB
ttttccattgcgaaattacggttagcctccaggccaaatccacatcaaaatcaccaatgc
acacaccaatgcaccttgactgatagctttatatgcttaaatggtaggtgtgcgacttca
agatatttacaagtggtgggctctattcatgatatagaatcttactttggtatagataat
actctttctttaaatctacagatattcacagcacattggggtcacctaactataatctta
atatgggtatcaggcaatctttatcacatagcatccaatgctaactattctctctgggtt
aaaaatccaattccaagcatacctatagcacataatatttgggatccacattttactaac
tctacttccactccatattcccataccatcatcaccacaatcctcattgcttactcaggt
atttataaccaactatacacttctggatttaacactattaatcagatatacaagaccaca
tttacatcctcttgcctggcagtaatatcaatactattagctaagatacatatcaatact
cattcagaactattacacaagcttgcaactcatgcaagtcaaatcccatcattcttccag
cttctctactttctggatgtcggtatctcatctgttaatattcgattcaattttcacact
ggaattctagttggacttttctcaatagcgcacacaggacatcttctagacatcacaata
ccagcctctcgagctccactaattcatacctcactctcttacttaacattctttggtggt
ttaaaatcgaatacaacttcattgtaccttacagatattgcacatcatcacttagcaatt
ggtatcacattcatccatacagggcatctatactcctctttcagggcagcccttggaaca
tatataagagatatattatatacatcacaccttacacacagcataaaatcactacactta
gccctctcactcattctagccagttgtacagcacttacatctaccaccactcagcacatc
tattctttaacaccatacttttatttgtcttatgatcacatctattttacagctctatat
gtccatcactcctacatcacatcattcttaaccattggttctcatgcacatacagctatc
accttagtaagagattggatcacaccattagaactagaatccagctctaagcagatacga
atccatacccataaagctgccattatttcacacttatcctgggtcagtctttggcttggt
tttcacacactagcagtatactctcataatgacacatgtatagcatttaactctccatct
aagcaaatcctaatagaagcaagcaatgctcaacttattcaacaagcatcaggaaaagct
ttatatgggaccatcaactccatcaacaactataataaatcatttgattcatttatacac
cctatcagtccaggtgattcatatgtacatcatgcaatagcacttggcttacatataact
gtcctcatcctactaaagggaggtcttgaagctcgtgggtctaagctaatgccagacaag
atggagcactcatttggcttttcatgtgatggtccaggtagaggtggcacatgtgatata
tcagcttgggattccttttatctagcaacattctggatgcttaatagcaacacatggata
agcttctacttccactacaagcaccttacacctcgtcaattctcagaaagttcaacttat
ctagaatcatggtttcgagattatctgtggtttaactctacacctttaattcatggttat
tctacactaggaactaatgatctatccgtccaatcctggtccttcctacttactcacctt
gcatgggcaagtggatttatgttccttattagttggagaggttactggcaagaattaatt
gatatcatcctctacatacatttgaaaacaccaattcttatcaatctttggaatggagac
atctatacacctctcgccttatctattgtacaagctagatttattgggcttgttcacttc
tcaaccggtctaattttaacttaccctccttttataataggtgctacaagttag
>Ik5_psaB
ttttccattgcgaaattacggttagcctccaggccaaatccacatcaaaatcaccaatgc
acacaccaatgcaccttgactgatagctttatatctttaaatggtaggtgtgcgacttca
agatatttacaagtggtgggctctattcatgatatagaatgtggctttggtatagataat
actctatctttaaatctacaaatattcacagcacattggggccacctaactataatctta
atatgggtatcaggcaatctttatcacatagcatccaatgctaactattctctctgggtt

aaaaatccaattccaagcatacctatagcacataatatttgggatccacattttactaac
tctacttccactccatattcccataccatcatcaccacaatcctcattgcttactcaggt
atttataaccaactatacacttctggatttaacactattaatcagatatacaagaccaca
tttacatcctcttgcctggcagtaatatcaatactattagctaagatacatatcaatact
cattcagaactattacacaagcttgcaactcatgcaagtcaaatcccatcattcttccag
cttctctactttctggatgtcggtatctcatctgttaatattcgattcaattttcacact
ggaattctagttggacttttctcaataggacacacaggacatcttctagacatcacaata
ccagcctctcgagctccactaattcacacctcaccctcttacttaacattctttggtggt
ttaaaatcgaatacaacttcattgtaccttacagatattgcacatcatcacttagcaatt
ggtatcatatctatccttacaggccatctatactcctctttcagggcagcccttggaaca
tatataagagatatattatatacatcacaccttacacacagcataaaatcactccactta
gccctctcactcattctagccagttgtacagcacttacatctaccaccactcagcacatc
tattctttaacaccatacttttatttgtcttatgatcacatctattctacggctctctat
gtccatcactcctacatcacatcattcttaaccattggttctcatgcacatacagctatc
accttagtaagagattggatcacaccattagaactagaatccagctctaagcggataaga
atacatacacataaagctgccattattccacacttatcctgggtcagtctttggcttggt
tttcacacactagcagtatactctcataatgacacatgtatagcatttaactctccatct
aagcaaatcttaatagaagcaagcaatgctcaacttattcaacaagcatcaggaaaagct
ttatatgggaccatcaactccatcaacaactataataaatcatttgattcatttatacac
cctatcagtccaggtgattcatatgtacatcatgcaatagcacttggcttacatataact
gtcctcatcctactaaagggaggtcttgaagctcgtgggtctaagctaatgccagacaag
atggagcactcatttggcttttcatgtgatggtccaggtagaggtggcacatgtgatata
tcagcttgggattccttttatctagcaacattctggatgcttaatagcaacacatggata
agcttctacttccactacaagcaccttacacctcgtcaattctcagaaagttcaacttat
ctagaatcatggtttcgagattatctgtggtttaattctacacctttaattcatggttat
tctacactaggaactaatgatctatccgtccaatcctggtccttcctacttactcacctt
gcatgggcaagtggatttatgttccttattagttggagaggttactggcaagaattaatt
gatatcatccttacatacatttgaaaacaccaattcttattaatctttggaatggagac
atctatacacctctcgccttatctattgtacaagctagatttattgggcttgttcacttc
tcaaccggtctaattttaacttaccctccttttataataggtgctactagttag
>Ik6_psaB
ttttccattgcgaaattacggttagcctccaggccaaatccacatcaaaatcaccaatgc
acacaccaatgcaccttgactgatagctttatatgcttaaatggtagatgtgctagttca
agatatttacaagttctgggttctattcatgatatagaatgtggctttggtatagataat
actctatctttaaatctacaaatattcacagcacattggggtcacctaactataatctta
atatgggtatcaggcaatctttatcacatagcatccaatgctaactattctctctgggtt
aaaaatccaattccaagcatacctatagcacataatatttgggatccacatttcactaac
tctacttccactccatattcccataccattatcaccacaatcctcattgcttactcaggt
atttataaccaattatacacttctggatttaacactattaatcagatatacaagaccaca
tttacatcctcttgcctggcagtaatttcaatactattagccaagatacacatcaacact
cattcagaactattacataagcttgcaagtcatacaagtcaaatcccatcattcttccag
cttctctactttctggatgtggccatctcatctgttaatattcgattcaattttcacact
ggaattctagttggacttttctcaatagggcacacaggacatcttctagacatcacaata
ccagcctctcgagctccactaattcacacctcaccctcttacttaacattctttggtggt
ttaaaatcgaatacaagttcattgtaccttacagatattgcacatcatcacttagcaatt
ggtatcatatctatccttacaggccatctatactcctctttcagggcagcccttggaaca
tatatacgagatacattatatacatcacaccttacacacagcataaaatcactccactta
gccctctcacttattctagccagttgtacaccacttacatcaaccactgctcagcacatc
tattctttaacaccatatttttatttgtcttatgatcacatctattttacagctctatat
gtccatcactcctacatcacctcattcttagccattgcttctcatgcacatacagctatc
accttagtaagagattggatcgcaccattagaacaagaatccagctctaagcagataaga
atccatacacataaagctgccattattccacacttatcctgggtcagtctttggcttggt
tttcacacactagcagtatactctcataatgacacatgtatagcatttaactctccatct
aagcaaatcctaatagaagcaagcaatggtcaacttattcaacaagcatcaggaaaagct
ttatatgggaccatcaactccatcaacaactataataaatcatttgattcatttatacac
cctatcagtccaggtgatttatatgtacatcatgcaatagcacttggcttacatataact
gtcctcatcctactaaagggaggtcttgaagctcgtgggtctaagctaatgccagacaag

atggagcactcatttggcttttcatgtgatgggccaggtagaggtggcacatgtgatata
tcagcttgggattccttttatctagcaacattctggatgcttaatagcaacacatggata
agcttctacttccactacaagcaccttacacctcgtcaattctcagaaagttcaacttat
ctagaatcatggtttcgagattatctgtggtttaactctacacctttaattcatggttat
tctacactaggaactaatgatctatccgtccaatcctggtccttcctacttactcacctt
gcatgggcaagtggatttatgttccttattagttggagaggttactggcaagaattaatt
gatatcatcctctacatacatttgaaaacaccaattcttatcaatctttggaatggagat
atctatacacctctagccttatctattgtacaagctagatttattgggcttgttcacttc
tcaactgggttaattctaacttaccctccttttataataggtgctacaagttag
>Ik7_psaB
ttttccattgcgaaattacggttagcctccaggccaaatccacatcaaaatcaccaatgc
acacaccaatgcaccttgactgatagctttatatgcttaaatggtagatgtgctagttca
agatatttacaagttctgggttctattcatgatatagaatgtggctttggtatagataat
actctatctttaaatctacagatattcacagcacattggggtcacctaactataatctta
atatgggtatcaagcaatctttaccacatagcatccaatgctaactattctctctgggtt
aaaaatccaattccaagcatgcctatagcacacaatatttgggatccacatttcactaac
tctacttccactccatattcccataccattatcaccacaatcctcattgcttactcaggt
atttataaccaattatacacttctggatttaacactattaatcagatatacaagaccaca
tttacatcctcttgcctggcagtaatttcaatactattagccaagatacacatcaacact
cattcagaactattacacaagcttgcaactcatgcaagtcaaatcccatcattcttccag
cttctctactttctggatgtcggtatctcatctgttaatattagattcaatttccacact
ggaattctagttggacttttctcaatagggcacacaggacatcttctagacatcacaata
ccagcctctcgagctccactaattcacacctcactctcttacttaacattctttggtggt
ttaaaatcgaatacaacttcattgtaccttacagatattgcacatcatcacttagcaatt
ggtatcacattcatccatacagggcatctatactcctctttcagggcagcccttggaaca
tatatacgagatatattatatacatcacaccttacacacagcataaaatcactacactta
gccctctcactcattctagccagttgtacagcacttacatcaaccaccgctcagcacatc
tattctttaacaccatacttttatttgtcttatgatcacatctattctacggctctctat
gtccatcactcctacatcacctcattcttagctattgcttctcatgcacatacagctatc
accttagtaagagattggatcacaccattagaactagaatccagctctaagcggataaga
atacatacacataaagctgccattattccacacttatcctgggtcagtctttggcttggt
tttcacacactagcagtatactctcataatgacacatgtatagcatttaactctccatct
aagcaaatcctaatagaagcaagcaatggtcaacttattcaacaagcatcaggaaaagct
ttatatgggaccatcaactccatcaacaactataataaatcatttgattcatttatacac
cctatcagtccaggtgattcatatgtacatcatgcaatagcacttggcttacatataact
gtcctcatcctactaaagggaggtcttgaagctcgtggttctaagctaatgccagacaag
atggagcactcatttggcttttcatgtgatggtccaggtagaggtggcacatgtgatata
tcagcttgggattccttttatctagcaacattctggatgcttaatagcaacacatggata
agcttctacttccactacaagcaccttacacctcgtcaattctcagaaagttcaacttat
ctagaatcatggtttcgagattatctgtggtttaattctacacctttaattcatggttat
tctacactaggagctaatgatctatccgtccaatcttggtccttcctacttactcatctt
gcttgggcaagtggatttatgttccttattagttggagaggttactggcaagaattaatt
gatatcatcctctacatacatttgaaaacaccaattcttatcaatctttggaatggagat
atctatacacctctcgccttatctattgtacaagctagatttattgggcttgttcacttc
tcaaccggtctaattttaacttaccctccttttataataggtgctacaagttag
>Ik8_psaB
ttttccattgcgaaattacggttagcctccaggccaaatccacatcaaaatcaccaatgc
acacaccaatgcaccttgactgatagctttatatgcttaaatggtagatgtgctagttca
agatatttacaagtggtgggctctattcatgatatagaatcttactttggtatagataat
actctatctttaaatctacaaatattcacagcacattggggccacctaactataatctta
atatgggtatcaagcaatctttaccacatagcatccaatgctaactattctctctgggtt
aaaaatccaattccaagtatacctatagcacataatatttgggatccacattttactaac
tctacttccactccatattcccataccattatcaccacaatcctcattgcttactcaggt
atttataaccaactatacacttctggatttaacactattaatcagatatacaagaccaca
tttacatcctcttgccttgcagtaatttcaatactattagccaagatacacatcaacact
cattcagaactattacataagcttgcaagtcatacaagtcaaatcccatcattcttccag
cttctctactttctggatgtggccatctcatctgttaatattagattcaatttccacact

ggaattctagttggacttttctcaatagggtacacaggacatcttctagacatcacaata
ccagcctctcgagctccactaattcacacctcaccctcttacttaacattctttggtggt
ttaaaatcgaatacaagttcattgtaccttacagatattgcacatcatcacttagcaatt
ggtatcacattcatccatacagggcatctatactcctctttcagggcagcccttggaaca
tatataagagatatattatatacatcacaccttacacacagcataaaatcactccactta
gccctctcactcattctagccagttgtacagcacttacatctaccaccgctcagcacatc
tattctttaacaccatatttttatttgtcttatgatcacatctattctacggctctctat
gtccatcactcctacatcacatcattcttaaccattggttctcatgcacatacagctatc
accttagtaagagattgggtcacaccattagaactagaatccagctctaagcggataaga
atacatacacataaagctgccattattccacacttatcctgggtcagtctttggcttggt
tttcatacactagcagtatactctcataatgacacatgtatagcatttaactctccatct
aagcaaatcttaatagaagcaagcaatgctcaacttattcaacaagcatcaggaaaagct
ttatatgggaccatcaactccatcaacaactataataaatcatttgattcatttatacac
cctatcagtccaggtgatttatatgtacatcatgcaatagcacttggcttacatataact
gtcctcatcctactaaagggaggtcttgaagctcgtgggtctaagctaatgccagacaag
atggagcactcatttggcttttcatgtgatgggccaggtagaggtggaacatgtgatata
tcagcttgggattccttttatctagcaacattctggatgcttaatagcaacacatggata
agcttctacttccactacaagcaccttacacctcgtcaattctcagaaagttcaacttat
ctagaatcatggtttcgagattatctgtggtttaactctacacctttaattcatggttat
tctacactaggaactaatgatctatccgtccaatcctggtccttcctacttactcacctt
gcatgggcaagtggatttatgttccttattagttggagaggttactggcaagaattaatt
gatatcatcctttacatacatttgaaaacaccaattcttattaatctttggaatggagat
atctatacacctctagccttatctattgtacaagctagatttattgggcttgttcacttc
tcaaccggtctaattttaacttaccctccttttataataggtgctacaagttag
>Ik9_psaB
ttttccattgcgaaattacggttagcctccaggccaaatccacatcaaaatcaccaatgc
acacaccaatgcaccttgactgatagctttatatgcttaaatggtagatgtgctagttca
agatatttacaagtggtgggctctattcatgatatagaatcttactttggtatagataat
actctttctttaaatctacagatattcacagcacattggggtcacctaactataatctta
atatgggtatcaggcaatctttaccacatagcatccaatgctaactattctctctgggtt
aaaaatccaattctaagcatacctatagcacataatatttgggatccacattttactaac
tctacttccactccatattcccataccatcatcaccacaatcctcattgcttactcaggt
atttataaccaactatacacttctggatttaacactattaatcagatatacaagaccaca
tttacatcctcttgcctggcagtaatatcaatactattagccaagatacacatcaacact
cattcagaactattacataagcttgcaagtcatacaagtcaaatcccatcattcttccag
cttctctactttctggatgtggccatctcatctgttaatattagattcaatttccacact
ggaattctagttggacttttctcaatagggtacacaggacatcttctagacatcacaata
ccagcctctcgagctccactaattcatacctcactctcttacttaacattctttggtggt
ttaaaatcgaatacaacttcattgtaccttacagatattgcacatcatcacttagcaatt
ggtatcacattcatccatacaggccatctatactcctctttcagggcagcccttggaaca
tatataagagatatattatatacatcacaccttacacacagcataaaatcactccactta
gccctctcactcattctagccagttgtacagcacttacatctaccaccactcagcacatc
tattctttaacaccatacttttatttgtcttatgatcacatctattttacagctctatat
gtccatcactcctacatcacatcattcttaaccattggttctcatgcacatacagctatc
accttagtaagagattgggtcgcaccattagaacaagaatccagctctaagcagatacga
atccatacccataaagctgccattatttcacacttatcctgggtcagtctttggcttggt
tttcacacactagcagtatactctcataatgacacatgtatagcatttaactctccatct
aagcaaatcttaatagaagcaagcaatgctcaacttattcaacaagcatcaggaaaagct
ttatatgggaccatcaactccatcaacaactataataaatcatttgattcatttatacac
cctatcagtccaggtgatttatatgtacatcatgcaatagcacttggcttacatataact
gtcctcatcctactaaagggaggtcttgaagctcgtggttctaagctaatgccagacaag
atggagcactcatttggcttttcatgtgatggtccaggtagaggtggcacatgtgatata
tcagcttgggattccttttatctagcaacattctggatgcttaatagcaacgcatggata
agcttctacttccactacaagcaccttacacctcgtcaattctcagaaagttcaacttat
ctagaatcatggtttcgagattatctgtggtttaactctacacctttaattcatggttat
tctacactaggaactaatgatctatccgtccaatcctggtccttcctacttactcacctt
gcatgggcaagtggatttatgttccttattagttggagaggttactggcaagaattaatt

gatatcatcctctacatacatttgaaaacaccaattcttatcaatctttggaatggagac
atctatacacctctcgccttatctattgtacaagctagatttattgggcttgttcacttc
tcaactggtttaattctaacttaccctccttttataataggtgctacaagttag
>Irm10_psaB
ttttccattgcgaaattacggttagcctccaggccaaatccacatcaaaatcaccaatgc
acacaccaatgcaccttgactgatagctttatatctttaaatggtaggtgtgcgacttca
agatatttacaagtggtgggctctattcatgatatagaatcttactttggtatagataat
actctttctttaaatctacagatattcacagcacattggggtcacctaactataatctta
atatgggtatcaggcaatctttatcacatagcatccaatgctaactattctctctgggtt
aaaaatccaattccaagtatgcctatagcacacaatatttgggatccacatttcactaac
tctacttccactccatattcccataccatcatcaccacaatcctcattgcttactcaggt
atttataaccaactatacacttctggatttaacactattaatcagatatacaagaccaca
tttacatcctcttgcctggcagtaatatcaatactattagctaagatacatatcaatact
cattcagaactattacacaagcttgcaactcatacaagtcaaatcccatcattcttccag
cttctctactttctggatgtggccatctcatctgttaatattcgattcaattttcacact
ggaattctagttggacttttctcaatagcacacacaggacatcttctagacatcacaata
ccagcctctcgagctccactaattcatacctcactctcttacttaacattctttggtggt
ttaaaatcgaatacaacttcattgtaccttacagatattgcacatcatcacttagcaatt
ggtatcacattcatccatacagggcatctatactcctctttcagggcagcccttggaaca
tatatacgagatacattatatacatcacaccttacacacagcataaaatcactacactta
gccctctcactcattctagccagttgtacagcacttacatctaccaccactcagcacatc
tattctttaacaccatacttttatttgtcttatgatcacatctattttacagctctatat
gtccatcactcctacatcacatcattcttaaccattggttctcatgcacatacagctatc
accttagtaagagattggatcacaccattagaactagaatccagctctaagcggataaga
atacatacacataaagctgccattattccacacttatcctgggtcagtctttggcttggt
tttcacacactagcagtatactctcataatgacacatgtatagcatttaactctccatct
aagcaaatcttaatagaagcaagcaatgctcaacttattcaacaagcatcaggaaaagct
ttatatgggaccatcaactccatcaacaactataataaatcatttgattcatttatacac
cctatcagtccaggtgattcatatgtacatcatgcaatagcacttggcttacatataact
gtcctcatcctactaaagggaggtcttgaagctcgtggttctaagctaatgccagacaag
atggagcactcatttggcttttcatgtgatgggccaggtagaggtggaacatgtgatata
tcagcttgggattccttttatctagcaacattctggatgcttaatagcaacgcatggata
agcttctacttccactacaagcaccttacacctcgtcaattctcagaaagttcaacttat
ctagaatcatggtttcgagattatctgtggtttaactctacacctttaattcatggttat
tctacactaggaactaatgatctatccgtccaatcctggtccttcctacttactcacctt
gcatgggcaagtggatttatgttccttattagttggagaggttactggcaagaattaatt
gatatcatcctctacatacatttgaaaacaccaattcttatcaatctttggaatggagac
atctatacacctctcgccttatctattgtacaagctagatttattgggcttgttcacttc
tcaaccggtctaattttaacttaccctccttttataataggtgctacaagttag
>Irm17_psaB
ttttccattgcgaaattacggttagcctccaggccaaatccacatcaaaatcaccaatgc
acacaccaatgcaccttgactgatagctttatatctttaaatggtaggtgtgcgacttca
agatatttacaagtggtgggctctattcatgatatagaatcttactttggtatagataat
actctttctttaaatctacagatattcacagcacattggggtcacctaactataatctta
atatgggtatcaggcaatctttatcacatagcatccaatgctaactattctctctgggtt
aaaaatccaattccaagcatacctatagcacataatatttgggatccacattttactaac
tctacttccactccatattcccataccatcatcaccacaatcctcattgcttactcaggt
atttataaccaactatacacttctggatttaacactattaatcagatatacaagaccaca
tttacatcctcttgcctggcagtaatatcaatactattagctaagatacatatcaatact
cattcagaactattacacaagcttgcaactcatgcaagtcaaatcccatcattcttccag
cttctctactttctggatgtcggtatctcatctgttaatattcgattcaattttcacact
ggaattctagttggacttttctcaatagcacacacaggacatcttctagacatcacaata
ccagcctctcgagctccactaattcatacctcactctcttacttaacattctttggtggt
ttaaaatcgaatacaacttcattgtaccttacagatattgcacatcatcacttagcaatt
ggtatcatatctatccttacaggccatctatactcctctttcagggcagcccttggaaca
tatatacgagatacattatatacatcacaccttacacacagcataaaatcactacactta
gccctctcactcattctagccagttgtacagcacttacatctaccaccactcagcacatc

tattctttaacaccatatttttatttgtcttatgatcacatctattttacagctctatat
gtccatcactcctacatcacatcattcttaaccattggttctcatgcacatacagctatc
accttagtaagagattggatcacaccattagaactagaatccagctctaagcggataaga
atacatacacataaagctgccattattccacacttatcctgggtcagtctttggcttggt
tttcacacactagcagtatactctcataatgacacatgtatagcatttaactctccatct
aagcaaatcttaatagaagcaagcaatgatcaacttattcaacaagcatcaggaaaagct
ttatatgggaccatcaactccatcaacaactataataaatcatttgattcatttatacac
cctatcagtccaggtgattcatatgtacatcatgcaatagcacttggcttacatataact
gtcctcatcctactaaagggaggtcttgaagctcgtgggtctaagctaatgccagacaag
atggagcactcatttggcttttcatgtgatggtccaggtagaggtggcacatgtgatata
tcagcttgggattccttttatctagcaacattctggatgcttaatagcaacgcatggata
agcttctacttccactacaagcaccttacacctcgtcaattctcagaaagttcaacttat
ctagaatcatggtttcgagattatctgtggtttaactctacacctttaattcatggttat
tctacactaggaactaatgatctatccgtccaatcctggtccttcctacttactcacctt
gcatgggcaagtggatttatgttccttattagttggagaggttactggcaagaattaatt
gatatcatcctctacatacatttgaaaacaccaattcttatcaatctttggaatggagac
atctatacacctctcgccttatctattgtacaagctagatttattgggcttgttcacttc
tcaaccggtttaattttaacttaccctccttttataataggtgctacaagttag
>Irm2_psaB
ttttccattgcgaaattacggttagcctccaggccaaatccacatcaaaatcaccaatgc
acacaccaatgcaccttgactgatagctttatatctttaaatggtaggtgtgcgacttca
agatatttacaagtggtgggctctattcatgatatagaatcttactttggtatagataat
actctttctttaaatctacagatattcacagcacattggggtcacctaactataatctta
atatgggtatcaggcaatctttatcacatagcatccaatgctaactattctctctgggtt
aaaaatccaattccaagcatacctatagcacataatatttgggatccacattttactaac
tctacttccactccatattcccataccatcatcaccacaatcctcattgcttactcaggt
atttataaccaattatacacttctggatttaacactattaatcagatatacaagaccaca
tttacatcctcttgcctggcagtaatatcaatactattagctaagatacatatcaatact
cattcagaactattacacaagcttgcaactcatgcaagtcaaatcccatcattcttccag
cttctctactttctggatgtcggtatctcatctgttaatattcgattcaattttcacact
ggaattctagttggacttttctcaatagcacacacaggacatcttctagacatcacaata
ccagcctctcgagctccactaattcatacctcactctcttacttaacattctttggtggt
ttaaaatcgaatacaacttcattgtaccttacagatattgcacatcatcacttagcaatt
ggtatcacattcatccatacagggcatctatactcctctttcagggcagcccttggaaca
tatatacgagatacattatatacatc-caccttacacacagcataaaatcactacactta
gccctctcactcattctagccagttgtacagcacttacatctaccaccactcagcacatc
tattctttaacaccatatttttatttgtcttatgatcacatctattttacagctctatat
gtccatcactcctacatcacatcattcttaaccattggttctcatgcacatacagctatc
accttagtaagagattggatcacaccattagaactagaatccagctctaagcggataaga
atacatacacataaagctgccattattccacacttatcctgggtcagtctttggcttggt
tttcacacactagcagtatactctcataatgacacatgtatagcatttaactctccatct
aagcaaatcttaatagaagcaagcaatgctcaacttattcaacaagcatcaggaaaagct
ttatatgggaccatcaactccatcaacaactataataaatcatttgattcatttatacac
cctatcagtccaggtgattcatatgtacatcatgcaatagcacttggcttacatataact
gtcctcatcctactaaagggaggtcttgaagctcgtgggtctaagctaatgccagacaag
atggagcactcatttggcttttcatgtgatggtccaggtagaggtggcacatgtgatata
tcagcttgggattccttttatctagcaacattctggatgcttaatagcaacacatggata
agcttctacttccactacaagcaccttacacctcgtcaattctcagaaagttcaacttat
ctagaatcatggtttcgagattatctgtggtttaactctacacctttaattcatggttat
tctacactaggaactaatgatctatccgtccaatcctggtccttcctacttactcacctt
gcatgggcaagtggatttatgttccttattagttggagaggttactggcaagaattaatt
gatatcatcctctacatacatttgaaaacaccaattcttatcaatctttggaatggagac
atctatacacctctcgccttatctattgtacaagctagatttattgggcttgttcacttc
tcaaccggtctaattttaacttaccctccttttataataggtgctacaagttag
>Irm21_psaB
ttttccattgcgaaattacggttagcctccaggccaaatccacatcaaaatcaccaatgc
acacaccaatgcaccttgactgatagctttatatgcttaaatggtagatgtgctagttca

agatatttacaagttctgggttctattcatgatatagaatgtggctttggtatagataat
actctatctttaaatctacaaatattcacagcacattggggccacctaactataatctta
atatgggtatcaggcaatctttatcacatagcatccaatgctaactattctctctgggtt
aaaaatccaattccaagcatacctatagcacataatatttgggatccacattttactaac
tctacttccactccatattcccataccatcatcaccacaatcctcattgcttactcaggt
atttataaccaattatacacttctggatttaacactattaatcagatatacaagaccaca
tttacattctcttgccttgcagtaatatcaatactattagctaagatacatatcaatact
cattcagaactattacacaagcttgcaactcatgcaagtcaaatcccatcattcttccag
cttctctactttctggatgtcggtatctcatctgttaatattcgattcaattttcacact
ggaattctagttggacttttctcaatagggtacacaggacatcttctagacatcacaata
ccagcctctcgagctccactaattcatacctcactctcttacttaacattctttggtggt
ttaaaatcgaatacaacttcattgtaccttacagatattgcacatcatcacttagcaatt
ggtatcatatctatccttacaggccatctatactcctctttcagggcagcccttggaaca
tatataagagatacattatatacatcacaccttacacacagcataaaatcactacactta
gccctctcactcattctagccagttgtacagcacttacatctaccaccactcagcacatc
tattctttaacaccatatttttatttgtcttatgatcacatctattttacagctctatat
gtccatcactcctacatcacatcattcttaaccattggttctcatgcacatacagctatc
accttagtaagagattggatcacaccattagaactagaatccagctctaagcagatacga
atccatacccataaagctgccattatttcacacttatcctgggtcagtctttggcttggt
tttcatacactagcagtatactctcataatgacacatgtatagcatttaactctccatct
aagcaaatcctaatagaagcaagcaatggtcaacttattcaacaagcatcaggaaaagct
ttatatgggaccatcaactccatcaacaactataataaatcatttgattcatttatacac
cctatcagtccaggtgattcatatgtacatcatgcaatagcacttggcttacatataact
gtcctcatcctactaaagggaggtcttgaagctcgtgggtctaagctaatgccagacaag
atggagcactcatttggcttttcatgtgatggtccaggtagaggtggcacatgtgatata
tcagcttgggattccttttatctagcaacattctggatgcttaatagcaacgcatggata
agcttctacttccactacaagcaccttacacctcgtcaattctcagaaagttcaacttat
ctagaatcatggtttcgagattatctgtggtttaactctacacctttaattcatggttat
tctacactaggaactaatgatctatacgtccaatcctggtccttcctacttactcacctt
gcatgggcaagtggatttatgttccttattagttggagaggttactggcaagaattaatt
gatatcatcctctacatacatttgaaaacaccaattcttatcaatctttggaatggagac
atctatacacctctcgccttatctattgtacaagctagatttattgggcttgttcacttc
tcaaccggtctaattttaacttaccctccttttataataggtgctacaagttag
>Irm22_psaB
ttttccattgcgaaattacggttagcctccaggccaaatccacatcaaaatcaccaatgc
acacaccaatgcaccttgactgatagctttatatgcttaaatggtagatgtgctagttca
agatatttacaagttctgggttctattcatgatatagaatgtggctttggtatagataat
actctatctttaaatctacaaatattcacagcacattggggccacctaactataatctta
atatgggtatcaagcaatctttaccacatagcatccaatgctaactattctctctgggtt
aaaaatccaattccaagtatgcctatagcacacaatatttgggatccacattttactaac
tctacttccactccatattcccataccatcatcaccacaatcctcattgcttactcaggt
atttataaccaactatacacttctggatttaacactattaatcagatatacaagaccaca
tttacatcctcttgcctggcagtaatatcaatactattagccaagatacacatcaacact
cattcagaactattacataagcttgcaagtcatacaagtcaaatcccatcattcttccag
cttctctactttctggatgtggccatctcatctgttaatattagattcaatttccacact
ggaattctagttggacttttctcaatagcacacacaggacatcttctagacatcacaata
ccagcctctcgagctccactaattcatacctcaccctcttacttaacattctttggtggt
ttaaaatcgaatacaagttcattgtaccttacagatattgcacatcatcacttagcaatt
ggtatcatatctatccttacaggccatctatactcctctttcagggcagcccttggaaca
tatataagagatatattatatacatcacaccttacacacagcataaaatcactccactta
gccctctcacttattctagccagttgtacaccacttacatcaaccactgctcagcacatc
tattctttaacaccatatttttatttgtcttatgatcacatctattctacggctctctat
gtccatcactcctacatcacctcattcttagctattgcttctcatgcacatactgctatc
accttagtaagagattgggtcgcaccattagaacaagaatccagctctaagcagatacga
atccatacccataaagctgccattatttcacacttatcctgggtcagtctttggcttggt
tttcatacactagcagtatactctcataatgacacatgtatagcatttaactctccatct
aagcaaatcctaatagaagcaagcaatggtcaacttattcaacaagcatcaggaaaagct

ttatatgggaccatcaactccatcaacaactataataaatcatttgattcatttatacac
cctatcagtccaggtgatttatatgtacatcatgcaatagcccttggtttacatataact
gtcctcatcctactaaagggaggtcttgaagctcgtggttctaagctaatgccagacaag
atggagcactcatttggcttttcatgtgatgggccaggtagaggtggaacatgtgatata
tcagcttgggattccttttatctagcaacattctggatgcttaatagcaacgcatggata
agcttctacttccactacaagcaccttacacctcgtcaattctcagaaagttcaacttat
ctagaatcatggtttcgagattatctgtggtttaattctacacctttaattcatggttat
tctacactaggagctaatgatctatccgtccaatcctggtccttcctacttactcacctt
gcatgggcaagtggatttatgttccttattagttggagaggttactggcaagaattaatt
gatatcatcctctacatacatttgaaaacaccaattcttatcaatctttggaatggagac
atctatacacctctcgccttatctattgtacaagctagatttattgggcttgttcacttc
tcaactgggttaattctaacttaccctccttttataataggtgctacaagttag
>Irm23_psaB
ttttccattgcgaaattacggttagcctccaggccaaatccacatcaaaatcaccaatgc
acacaccaatgcaccttgactgatagctttatatgcttaaatggtagatgtgctagttca
agatatttacaagttctgggttctattcatgatatagaatgtggctttggtatagataat
actctatctttaaatctacagatattcacagcacattggggtcacctaactataatctta
atatgggtatcaggcaatctttatcacatagcatccaatgctaactattctctctgggtt
aaaaatccaattccaagcatacctatagcacataatatttgggatccacattttactaac
tctacttccactccatattcccataccatcatcaccacaatcctcattgcttactcaggt
atttataaccaattatacacttctggatttaacactattaatcagatatacaagaccaca
tttacatcctcttgcctggcagtaatatcaatactattagctaagatacatatcaatact
cattcagaactattacacaagcttgcaactcatgcaagtcaaatcccatcattcttccag
cttctctactttctggatgtggccatctcatctgttaatattagattcaatttccacact
ggaattctagttggacttttctcaatagggtacacaggacatcttctagacatcacaata
ccagcctctcgagctccactaattcatacctcactctcttacttaacattctttggtggt
ttaaaatcgaatacaagttcattgtaccttacagatattgcacatcatcacttagcaatt
ggtatcatatctatccttacaggccatctatactcctctttcagggcagcccttggaaca
tatataagagatatattatatacatcacaccttacacacagcataaaatcactacactta
gccctctcactcattctagccagttgtacagcacttacatctaccaccactcagcacatc
tattctttaacaccatacttttatttgtcttatgatcacatctattttacagctctatat
gtccatcactcctacatcacatcattcttagctattgcttctcatgcacatactgctatc
accttagtaagagattgggtcgcaccattagaacaagaatccagctctaagcggataaga
atacatacacataaagctgccattattccacacttatcctgggtcagtctttggcttggt
tttcacacactagcagtatactctcataatgacacatgtatagcatttaactctccatct
aagcaaatcttaatagaagcaagcaatgctcaacttattcaacaagcatcaggaaaagct
ttatatgggaccatcaactccatcaacaactataataaatcatttgattcatttatacac
cctatcagtccaggtgattcatatgtacatcatgcaatagcacttggcttacatataact
gtcctcatcctactaaagggaggtcttgaagctcgtggttctaagctaatgccagacaag
atggagcactcatttggcttttcatgtgatgggccaggtagaggtggcacatgtgatata
tcagcttgggattccttttatctagcaacattctggatgcttaatagcaacacatggata
agcttctacttccactacaagcaccttacacctcgtcaattctcagaaagttcaacttat
ctagaatcatggtttcgagattatctgtggtttaactctacacctttaattcatggttat
tctacactaggaactaatgatctatccgtccaatcctggtccttcctacttactcatctt
gcttgggcaagtggatttatgttccttattagttggagaggttactggcaagaattaatt
gatatcatcctctacatacatttgaaaacaccaattcttatcaatctttggaatggagac
atctatacacctctcgccttatctattgtacaagctagatttattgggcttgttcacttc
tcaaccggtctaattttaacttaccctccttttataataggtgctacaagttag
>Irm24_psaB
ttttccattgcgaaattacggttagcctccaggccaaatccacatcaaaatcaccaatgc
acacaccaatgcaccttgactgatagctttatatgcttaaatggtagatgtgctagttca
agatatttacaagttctgggttctattcatgatatagaatgtggctttggtatagataat
actctatctttaaatctacagatattcacagcacattggggtcacctaactataatctta
atatgggtatcaggcaatctttatcacatagcatccaatgctaactattctctctgggtt
aaaaatccaattccaagcatacctatagcacataatatttgggatccacattttactaac
tctacttccactccatattcccataccattatcaccacaatcctcattgcttactcaggt
atttataaccaattatacacttctggatttaacactattaatcagatatacaagaccaca

tttacattctcttgccttgcagtaatttcaatactattagccaagatacacatcaacact
cattcagaactattacataagcttgcaagtcatacaagtcaaatcccatcattcttccag
cttctctactttctggatgtggccatctcatctgttaatattagattcaatttccacact
ggaattctagttggacttttctcaatagggtacacaggacatcttctagacatcacaata
ccagcctctcgagctccactaattcacacctcaccctcttacttaacattctttggtggt
ttaaaatcgaatacaagttcattgtaccttacagatattgcacatcatcacttagcaatt
ggtatcacattcatccatacagggcatctatactcctctttcagggcagcccttggaaca
tatatacgagatacattatatacatcacaccttacacacagcataaaatcactccactta
gccctctcacttattctagccagttgtacaccacttacatcaaccactgctcagcacatc
tattctttaacaccatatttttatttgtcttatgatcacatctattctacggctctctat
gtccatcactcctacatcacctcattcttaaccattggttctcatgcacatacagctatc
accttagtaagagattggatcacaccattagaactagaatccagctctaagcagatacga
atccatacccataaagctgccattatttcacacttatcctgggtcagtctttggcttggt
tttcatacactagcagtatactctcataatgacacatgtatagcatttaactctccatct
aagcaaatcctaatagaagcaagcaatggtcaacttattcaacaagcatcaggaaaagct
ttatatgggaccatcaactccatcaacaactataataaatcatttgattcatttatacac
cctatcagtccaggtgatttatatgtacatcatgcaatagcccttggtttacatataact
gtcctcatcctactaaagggaggtcttgaagctcgtggttctaagctaatgccagacaag
atggagcactcatttggcttttcatgtgatgggccaggtagaggtggaacatgtgatata
tcagcttgggattccttttatctagcaacattctggatgcttaatagcaacgcatggata
agcttctacttccactacaagcaccttacacctcgtcaattctcagaaagttcaacttat
ctagaatcatggtttcgagattatctgtggtttaactctacacctttaattcatggttat
tctacactaggaactaatgatctatccgtccaatcttggtccttcctacttactcatctt
gcttgggcaagtggatttatgttccttattagttggagaggttactggcaagaattaatt
gatatcatccctttacatacatttgaaaacaccaattcttattaatctttggaatggagat
atctatacacctctagccttatctattgtacaagctagatttattgggcttgttcacttc
tcaactgggttaattctaacttaccctccttttataataggtgctacaagttag
>Irm25_psaB
ttttccattgcgaaattacggttagcctccaggccaaatccacatcaaaatcaccaatgc
acacaccaatgcaccttgactgatagctttatatgcttaaatggtagatgtgctagttca
agatatttacaagttctgggttctattcatgatatagaatcttactttggtatagataat
actctttctttaaatctacagatattcacagcacattggggtcacctaactataatctta
atatgggtatcaggcaatctttatcacatagcatccaatgctaactattctctctgggtt
aaaaatccaattccaagtatgcctatagcacacaatatttgggatccacattttactaac
tctacttccactccatattcccataccatcatcaccacaatcctcattgcttactcaggt
atttataaccaattatacacttctggatttaacactattaatcagatatacaagaccaca
tttacattctcttgcctggcagtaatatcaatactattagctaagatacatatcaatact
cattcagaactattacacaagcttgcaactcatgcaagtcaaatcccatcattcttccag
cttctctactttctggatgtggccatctcatctgttaatattagattcaatttccacact
ggaattctagttggacttttctcaatagggtacacaggacatcttctagacatcacaata
ccagcctctcgagctccactaattcacacctcaccctcttacttaacattctttggtggt
ttaaaatcgaatacaacttcattgtaccttacagatattgcacatcatcacttagcaatt
ggtatcacattcatccatacagggcatctatactcctctttcagggcagcccttggaaca
tatataagagatatattatatacatcacaccttacacacagcataaaatcactccactta
gccctctcacttattctagccagttgtacaccacttacatcaaccactgctcagcacatc
tattctttaacaccatatttttatttgtcttatgatcacatctattctacggctctctat
gtccatcactcctacatcacctcattcttagctattgcttctcatgcacatactgctatc
accttagtaagagattgggtcgcaccattagaacaagaatccagctctaagcggataaga
atacatacacataaagctgccattattccacacttatcctgggtcagtctttggcttggt
tttcacacactagcagtatactctcataatgacacatgtatagcatttaactctccatct
aagcaaatcctaatagaagcaagcaatggtcaacttattcaacaagcatcaggaaaagct
ttatatgggaccatcaactccatcaacaactataataaatcatttgattcatttatacac
cctatcagtccaggtgattcatatgtacatcatgcaatagcccttggtttacatataact
gtcctcatcctactaaagggaggtcttgaagctcgtggttctaagctaatgccagacaag
atggagcactcatttggcttttcatgtgatggtccaggtagaggtggcacatgtgatata
tcagcttgggattccttttatctagcaacattctggatgcttaatagcaacacatggata
agcttctacttccactacaagcaccttacacctcgtcaattctcagaaagttcaacttat

ctagaatcatggtttcgagattatctgtggtttaactctacacctttaattcatggttat
tctacactaggaactaatgatctatccgtccaatcctggtccttcctacttactcatctt
gcttgggcaagtggatttatgttccttattagttggagaggttactggcaagaattaatt
gatatcatcctttacatacatttgaaaacaccaattcttattaatctttggaatggagat
atctatacacctctagccttatctattgtacaagctagatttattgggcttgttcacttc
tcaactgggttaattctaacttaccctccttttataataggtgctacaagttag
>Irm26_psaB
ttttccattgcgaaattacggttagcctccaggccaaatccacatcaaaatcaccaatgc
acacaccaatgcaccttgactgatagctttatatgcttaaatggtagatgtgctagttca
agatatttacaagtggtgggctctattcatgatatagaatcttactttggtatagataat
actctttctttaaatctacagatattcacagcacattggggtcacctaactataatctta
atatgggtatcaggcaatctttatcacatagcatccaatgctaactattctctctgggtt
aaaaatccaattccaagcatacctatagcacataatatttgggatccacattttactaac
tctacttccactccatattcccataccatcatcaccacaatcctcattgcttactcaggt
atttataaccaactatacacttctggatttaacactattaatcagatatacaagaccaca
tttacatcctcttgcctggcagtaatatcaatactattagctaagatacatatcaatact
cattcagaactattacacaagcttgcaactcatgcaagtcaaatcccatcattcttccag
cttctctactttctggatgtggccatctcatctgttaatattagattcaatttccacact
ggaattctagttggacttttctcaatagggtacacaggacatcttctagacatcacaata
ccagcctctcgagctccactaattcatacctcactctcttacttaacattctttggtggt
ttaaaatcgaatacaagttcattgtaccttacagatattgcacatcatcacttagcaatt
ggtatcatatctatccttacaggccatctatactcctctttcagggcagcccttggaaca
tatataagagatatattatatacatcacaccttacacacagcataaaatcactccactta
gccctctcactcattctagccagttgtacagcacttacatctaccaccactcagcacatc
tattctttaacaccatacttttatttgtcttatgatcacatctattttacggctctctat
gtccatcactcctacatcacatcattcttaaccattggttctcatgcacatactgctatc
accttagtaagagattggatcacaccattagaactagaatccagctctaagcggataaga
atacatacacataaagctgccattattccacacttatcctgggtcagtctttggcttggt
tttcacacactagcagtatactctcataatgacacatgtatagcatttaactctccatct
aagcaaatcttaatagaagcaagcaatgctcaacttattcaacaagcatcaggaaaagct
ttatatgggaccatcaactccatcaacaactataataaatcatttgattcatttatacac
cctatcagtccaggtgatttatatgtacatcatgcaatagcccttggtttacatataact
gtcctcatcctactaaagggaggtcttgaagctcgtggttctaagctaatgccagacaag
atggagcactcatttggcttttcatgtgatgggccaggtagaggtggaacatgtgatata
tcagcttgggattccttttatctagcaacattctggatgcttaatagcaacgcatggata
agcttctacttccactacaagcaccttacacctcgtcaattctcagaaagttcaacttat
ctagaatcatggtttcgagattatctgtggtttaactctacacctttaattcatggttat
tctacactaggaactaatgatctatccgtccaatcctggtccttcctacttactcacctt
gcatgggcaagtggatttatgttccttattagttggagaggttactggcaagaattaatt
gatatcatcctctacatacatttgaaaacaccaattcttatcaatctttggaatggagac
atctatacacctctcgccttatctattgtacaagctagatttattgggcttgttcacttc
tcaaccggtctaattttaacttaccctccttttataataggtgctacaagttag
>Irm27_psaB
ttttccattgcgaaattacggttagcctccaggccaaatccacatcaaaatcaccaatgc
acacaccaatgcaccttgactgatagctttatatgcttaaatggtagatgtgctagttca
agatatttacaagttctgggttctattcatgatatagaatgtggctttggtatagataat
actctatctttaaatctacagatctttacagcacattggggtcatctaactataatctta
atatgggtatcaagtaatctttatcacatagcatccaatgctaactattctctctgggtt
caaaatccaattccaagcatacctatagcacacaatatttgggacccccacttcactagc
tccacttccactccatattcccataccattatcaccacaatcctcattgcttactcaggt
atttataaccaattatacacttctggatttaacactattaatcagatatacaagaccaca
tttacattctcttgccttgcagtaatttcaatactattagccaagatacacatcaacact
cattcagaactactacacaagcttgcaactaatacaagtcaaatcccatcattcttccag
cttctctactttctggatgtggccatctcatctgttaatattagattcaatttccacact
ggaattctagttggacttttctcaatagggtacacaggacatcttctagacatcacaata
ccagcctctcgagctccactaattcacacctcaccctcttacttaacattcctgggtggt
ttaaaatcgaatacaacttcattgtaccttacagatattgcacatcatcacttagcaatt

ggtatcatatctatccttacaggccatctatactcctctttcagggcagcccttggaaca
tatataagagatatattatatacatcacaccttacacacagcataaaatcactccactta
gccctctcacttattctagccagttgtacaccacttacatcaaccactgctcagcacatc
tattctttaacaccatatttttatttgtcttatgatcacatctattctacggctctctat
gtccatcactcctacatcacctcattcttagctattgcttctcatgcacatactgctatc
accttagtaagagattgggtcgcaccattagaacaagaatccagctctaagcagatacga
atccatacccataaagctgccattattccacacttatcctggattagtctttggcttggt
tttcacacactagcagtatactctcataatgacacatgtatagcatttaactctccatct
aagcaaatcctaatagaagcaagtaatgctcaacttattcaacaagcatcaggaaaagct
ttatatgggaccatcaactccatcaacaactataataaatcatttgattcatttatacac
cctatcagtccaggtgatttatatgtacatcatgcaatagcccttggtttacatataact
gtcctcatcctactaaagggaggtcttgaagctcgtggttctaagctaatgccagacaag
atggagcactcatttggcttttcatgtgatgggccaggtagaggtggaacatgtgatata
tctgcttgggattctttttatctagcaacattctggatgcttaatagtaatacatggata
agcttctacttccactacaagcaccttacacctcgccaattctcagaaagttcaacttat
ctagaatcgtggtttcgagattatctgtggtttaattctacacctttaattcacggttat
tctacactagggactaatgacttatctgtacagtcttggtccttcctccttactcatctt
gcttgggcaagtggatttatgttccttattagttggagaggttactggcaagaattaatt
gatatcatcctttacatacatttgaaaacaccaattcttataaatctttggaatggagac
atctatacacctctcgccttatctattgtacaagctagatttattgggcttgttcacttc
tcaactggtctaattttaacttaccccccttttataataggtgctacaagttag
>Irm3_psaB
ttttccattgcgaaattacggttagcctccaggccaaatccacatcaaaatcaccaatgc
acacaccaatgcaccttgactgatagctttatatgcttaaatggtagatgtgctagttca
agatatttacaagttctgggttctattcatgatatagaatgtggctttggtatagataat
actctatctctaaatctacagatctttacagcacattggggtcatctaactataatctta
atatgggtatcaagtaatctttatcacatagcctccaatgctaactattctctctgggtt
caaaatccaattccaagcatacctatagcacacaatatttgggacccccacttcactagc
tccacttccactccatattcccataccattatcaccacaatactaattgcttattcagga
atctataaccaactatacacttctggatttaactctattaatcagatatataagaccaca
tttacactttcttgtttggcagtaatatcaatactattagccaagatacacatcaacact
cattcagaactactacacaagcttgcaactaatacaagtcaaatcccatcattcttccag
cttctctactttcttgatgttggcatctcatctgttaatattcgattcaattttcacact
ggaattctagttggacttttctcaatagcacacacaggacatcttctagacatcacaata
ccagcctctcgagctccactaattcacacctcaccctcttacttaacattcctgggtggt
ttaaaatcgaatacaacttcattgtaccttacagatattgcacatcatcacttagcaatt
ggtatcacatccatacacacaggtcatctatactcctctttcagggcagcccttggaaca
tatatacgagatacattatatacatcacaccttacacatagtataaaatcactccactta
gccctctcactcattctagccagttgtgcagcacttacatctatcaccactcaacacatc
tattctttaacaccttacttttatttgtcttatgatcacgtctattctgcagctctctat
gtccatcactcctatatcacatcattattagcaataggttcacatgcacatgcagctatc
accttagtaagagattggatcacaccattagaactagaatccagctacaatactatacga
atccatacacataaagctgccattattccacacttatcctggattagtctttggcttggt
tttcacacactagcagtatactctcataatgacacatgtatagcatttaactctccatct
aagcaaatcttaatagaagcaagcaatggtcaacttattcaacaagcatcaggaaaagct
ttatatgggaccatcaactccatcaacaactataataaatcatttgattcatttatatac
cctatcagcccaggtgatttatatgtacatcatgcaatagcactgggtttgcatgttact
gtactcatcctactaaaaggagggcttgaagctcgtgggtctaagctaatgccagacaag
atggaacactcatttggcttttcatgtgatggtccaggtagaggtggtacgtgtgatata
tctgcttgggattctttttatctagcaacattctggatgcttaatagtaatacatggata
agcttctacttccactacaagcaccttacacctcgccaattctcagaaagttcaacttat
ctagaatcgtggtttcgagattatctgtggtttaattctacacctttaattcacggttat
tctacactagggactaatgacttatctgtacagtcttggtccttcctccttactcatctt
gcatgggcaagtggatttatgttccttattagttggagaggttactggcaggaattaatt
gatatcatcctctacatacatttgaaaacaccaattcttataaatctttggaatggagac
atctatacacctctcgccttatctattgtacaagctagatttattgggcttgttcacttc
tcaactggtctaattttaacttaccccccttttataataggtgctacaagttag

>Irm4_psaB
ttttccattgcgaaattacggttagcctccaggccaaatccacatcaaaatcaccaatgc
acacaccaatgcaccttgactgatagctttatatgcttaaatggtagatgtgctagttca
agatatttacaagttctgggttctattcatgatatagaatgtggctttggcatagataat
actctatctctaaatctacagatctttacagcacattggggtcatctaactataatctta
atatgggtatcaggtaatctttatcacatagcctccaatgctaactattctctctgggtt
caaaatccaattccaagcatacctatagcacacaatatttgggacccccactttactagc
tctacttccactccatattcccataccatcatcaccacaatcctcattgcttattcagga
atctataaccaactatacacttctggatttaactctattaatcagatatataagaccaca
tttacactttcttgtttggcagtaatatcaatactattagccaagatacacatcaatact
cattcagaactactacacaagcttgcaactcatgcaagtcaaatcccatcattcttccag
cttctctactttcttgatgttggcatctcatctgttaatattcgattcaattttcacact
ggaattctagttggacttttctcaatagcacacacaggacatcttctagacatcacaata
ccagcctctcgagctccactaattcacacctcactctcttacttaacattctttggtggt
ttaaaatcgaatacaacttcattgtaccttacagatattgcacatcatcacttagcaatt
ggtatcacatccatacacacaggtcatctatactcctctttcagggcagcccttggaaca
tatatacgagatacattatatacatcacaccttacacacagcataaaatcactccactta
gccctctcactcattctagccagttgtgcagcacttacatcaaccactgctcagcacatc
tattctttaacaccatatttttatttgtcttatgatcacatctattctacagctctatat
gtccatcactcctatatcacatcattattaacaataggttcacatgcacatgcagctatc
accttagtaagagattggatcacaccattagaactagaatccagctctaagcggataaga
atacatacacataaagctgccattattccacacttatcctggattagtctttggcttggt
tttcacacactagcagtatactctcataatgacacatgtatagcatttaactctccatct
aagcaaatcttaatagaagcaagcaatggtcaacttattcaacaagcatcaggaaaagct
ttatatgggaccatcaactccatcaacaactataataaatcatttgattcatttatatac
cctatcagcccaggtgatttatatgtacatcatgcaatagcactgggtttgcatgttact
gtactcatcctactaaaaggagggcttgaagctcgtgggtctaagctaatgccagacaag
atggagcactcatttggcttttcatgtgatggtccaggtagaggtggcacgtgtgatata
tctgcttgggattctttttatctagcaacattctggatgcttaatagtaatacatggata
agcttctacttccactacaagcaccttacacctcgccaattctcagaaagttcaacttat
ctagaatcgtggtttcgagattatctgtggtttaattctacacctttaattcatggttat
tctacactaggagctaatgatctatccgtccaatcttggtccttcctccttactcatctt
gcatgggcaagtggatttatgttccttattagttggagaggttactggcaggaattaatt
gatatcatcctctacatacatttgaaaacaccaattcttataaatctttggaatggagac
atctatacacctctcgccttatctattgtacaagctagatttattgggcttgttcacttc
tcaactggtctaattttaacttacccccctttttataataggtgctacaagttag
>Irm5_psaB
ttttccattgcgaaattacggttagcctccaggccaaatccacatcaaaatcaccaatgc
acacaccaatgcaccttgactgatagctttatatctttaaatggtaggtgtgcgacttca
agatatttacaagtggtgggctctattcatgatatagaatcttactttggtatagataat
actctttctttaaatctacagatattcacagcacattggggtcacctaactataatctta
atatgggtatcaggcaatctttatcacatagcatccaatgctaactattctctctgggtt
aaaaatccaattccaagcatacctatagcacataatatttgggatccacattttactaac
tctacttccactccatattcccataccatcatcaccacaatcctcattgcttactcaggt
atttataaccaactatacacttctggatttaacactattaatcagatatacaagaccaca
tttacatcctcttgcctggcagtaatatcaatactattagctaagatacatatcaatact
cattcagaactattacacaagcttgcaactcatgcaagtcaaatcccatcattcttccag
cttctctactttctggatgtcggtatctcatctgttaatattcgattcaattttcacact
ggaattctagttggacttttctcaatagcacacacaggacatcttctagacatcacaata
ccagcctctcgagctccactaattcatacctcactctcttacttaacattctttggtggt
ttaaaatcgaatacaacttcattgtaccttacagatattgcacatcatcacttagcaatt
ggtatcacattcatccatacagggcatctatactcctctttcagggcagcccttggaaca
tatatacgagatacattatatacatcacaccttacacacagcataaaatcactacactta
gccctctcactcattctagccagttgtacagcacttacatctaccaccactcagcacatc
tattctttaacaccatacttttatttgtcttatgatcacatctattttacagctctatat
gtccatcactcctacatcacatcattcttaaccattggttctcatgcacatacagctatc
accttagtaagagattggatcacaccattagaactagaatccagctctaagcggataaga

atacatacacataaagctgccattattccacacttatcctgggtcagtctttggcttggt
tttcacacactagcagtatactctcataatgacacatgtatagcatttaactctccatct
aagcaaatcttaatagaagcaagcaatgctcaacttattcaacaagcatcaggaaaagct
ttatatgggaccatcaactccatcaacaactataataaatcatttgattcatttatacac
cctatcagtccaggtgattcatatgtacatcatgcaatagcacttggcttacatataact
gtcctcatcctactaaagggaggtcttgaagctcgtgggtctaagctaatgccagacaag
atggagcactcatttggcttttcatgtgatgggccaggtagaggtggcacatgtgatata
tcagcttgggattccttttatctagcaacattctggatgcttaatagcaacacatggata
agcttctacttccactacaagcaccttacacctcgtcaattctcagaaagttcaacttat
ctagaatcatggtttcgagattatctgtggtttaactctacacctttaattcatggttat
tctacactaggaactaatgatctatccgtccaatcctggtccttcctacttactcacctt
gcatgggcaagtggatttatgttccttattagttggagaggttactggcaagaattaatt
gatatcatcctctacatacatttgaaaacaccaattcttatcaatctttggaatggagac
atctatacacctctcgccttatctattgtacaagctagatttattgggcttgttcacttc
tcaaccggtctaattttaacttaccctccttttataataggtgctactagttag
>Irm7_psaB
ttttccattgcgaaattacggttagcctccaggccaaatccacatcaaaatcaccaatgc
acacaccaatgcaccttgactgatagctttatatgcttaaatggtagatgtgctagttca
agatatttacaagttctgggttctattcatgatatagaatgtggctttggtatagataat
actctatctttaaatctacaaatattcacagcacattggggccacctaactataatctta
atatgggtatcaagcaatctttaccacatagcatccaatgctaactattctctctgggtt
aaaaatccaattccaagtatgcctatagcacacaatatttgggatccacatttcactaac
tctacttccactccatattcccataccatcatcaccacaatcctcattgcttattcagga
atctataaccaactatacacttctggatttaactctattaatcagatatataagaccaca
tttacatcctcttgcctggcagtaatatcaatactattagctaagatacatatcaatact
cattcagaactattacataagcttgcaagtcatacaagtcaaatcccatcattcttccag
cttctctactttctggatgtggccatctcatctgttaatattagattcaatttccacact
ggaattctagttggacttttctcaatagggtacacaggacatcttctagacatcacaata
ccagcctctcgagctccactaattcacacctcaccctcttacttaacattctttggtggt
ttaaaatcgaatacaagttcattgtaccttacagatattgcacatcatcacttagcaatt
ggtatcatatctatccttacaggccatctatactcctctttcagggcagcccttggaaca
tatataagagatatattatatacatcacaccttacacacagcataaaatcactacactta
gccctctcactcattctagccagttgtacagcacttacatcaaccactgctcagcacatc
tattctttaacaccatatttttatttgtcttatgatcacatctattctacggctctctat
gtccatcactcctacatcacctcattcttagctattgcttctcatgcacatactgctatc
accttagtaagagattgggtcgcaccattagaactagaatccagctctaagcagataaga
atacatacacataaagctgccattattccacacttatcctgggtcagtctttggcttggt
tttcacacactagcagtatactctcataatgacacatgtatagcatttaactctccatct
aagcaaatcctaatagaagcaagcaatggtcaacttattcaacaagcatcaggaaaagct
ttatatgggaccatcaactccatcaacaactataataaatcatttgattcatttatacac
cctatcagtccaggtgattcatatgtacatcatgcaatagcacttggcttacatataact
gtcctcatcctactaaagggaggtcttgaagctcgtgggtctaagctaatgccagacaag
atggagcactcatttggcttttcatgtgatggtccaggtagaggtggaacatgtgatata
tcagcttgggattccttttatctagcaacattctggatgcttaatagcaacgcatggata
agcttctacttccactacaagcaccttacacctcgtcaattctcagaaagttcaacttat
ctagaatcatggtttcgagattatctgtggtttaactctacacctttaattcatggttat
tctacactaggagctaatgatctatccgtccaatcttggtccttcctacttactcatctt
gcttgggcaagtggatttatgttccttattagttggagaggttactggcaagaattaatt
gatatcatcctttacatacatttgaaaacaccaattcttattaatctttggaatggagat
atctatacacctctagccttatctattgtacaagctagatttattgggcttgttcacttc
tcaactgggttaattctaacttaccctccttttataataggtgctacaagttag
>Irm9_psaB
ttttccattgcgaaattacggttagcctccaggccaaatccacatcaaaatcaccaatgc
acacaccaatgcaccttgactgatagctttatatgcttaaatggtagatgtgctagttca
agatatttacaagttctgggttctattcatgatatagaatgtggctttggtatagataat
actctatctctaaatctacagatctttacagcacattggggtcatctaactataatctta
atatgggtatcaagtaatctttatcacatagcctccaatgctaactattctctctgggtt

caaaatccaattccaagcatacctatagcacacaatatttgggacccccacttcactagc
tccacttccactccatattcccataccattatcaccacaatcctcattgcttattcagga
atctataaccaactatacacttctggatttaactctattaatcagatatataagaccaca
tttacattctcttgccttgcagtaatatcaatactattagccaagatacacatcaacact
cattcagaactactacacaagcttgcaactaatacaagtcaaatcccatcattcttccag
cttctctactttctggatgtggccatctcatctgttaatattagattcaatttccacact
ggaattctagttggacttttctcaatagcacacacaggacatcttctagacatcacaata
ccagcctctcgagctccactaattcacacctcaccctcttacttaacattcctgggtggt
ttaaaatcgaatacaacttcattgtaccttacagatattgcacatcatcacttagcaatt
ggtatcacatccatacacacaggtcatctatactcctctttcagggcagcccttggaaca
tatataagagatatattatatacatcacaccttacacacagcataaaatcactccactta
gccctctcactcattctagccagttgtgcagcacttacatcaaccactgctcagcacatc
tattctttaacaccatatttttatttgtcttatgatcacatctattctacggctctctat
gtccatcactcctacatcacctcattcttagctattgcttctcatgcacatgcagctatc
accttagtaagagattggatcacaccattagaactagaatccagctctaagcagatacga
atccatacacataaagctgccattattccacacttatcctggattagtctttggcttggt
tttcacacactagcagtatactctcataatgacacatgtatagcatttaactctccatct
aagcaaatcttaatagaagcaagcaatggtcaacttattcaacaagcatcaggaaaagct
ttatatgggaccatcaactccatcaacaactataataaatcatttgattcatttatatac
cctatcagcccaggtgatttatatgtacatcatgcaatagcactgggtttgcatgttact
gtactcatcctactaaaaggagggcttgaagctcgtgggtctaagctaatgccagacaag
atggaacactcatttggcttttcatgtgatggtccaggtagaggtggtacgtgtgatata
tctgcttgggattctttttatctagcaacattctggatgcttaatagcaacgcatggata
agcttctacttccactacaagcaccttacacctcgccaattctcagaaagttcaacttat
ctagaatcgtggtttcgagattatctgtggtttaattctacacctttaattcacggttat
tctacactaggagctaatgatctatccgtccaatcttggtccttcctacttactcatctt
gcttgggcaagtggatttatgttccttattagttggagaggttactggcaggaattaatt
gatatcatcctctacatacatttgaaaacaccaattcttattaatctttggaatggagat
atctatacacctctagccttatctattgtacaagctagatttattgggcttgttcacttc
tcaactggtctaattttaacttaccctccttttataataggtgctacaagttag
>Isy12_psaB
ttttccattgcgaaattacggttagcctccaggccaaatccacatcaaaatcaccaatgc
acacaccaatgcaccttgactgatagctttatatctttaaatggtaggtgtgcgacttca
agatatttacaagtggtgggctctattcatgatatagaatcttactttggtatagataat
actctttctttaaatctacagatattcacagcacattggggtcacctaactataatctta
atatgggtatcaggtaatctttatcacatagcatccaatgctaactattctctctgggtt
aaaaatccaattccaagcatacctatagcacataatatttgggatccacattttactaac
tctacttccactccatattcccataccatcatcaccacaatcctcattgcttactcaggt
atttataaccaactatacacttctggatttaacactattaatcagatatacaagaccaca
tttacatcctcttgcctggcagtaatatcaatactattagctaagatacacatcaatact
cattcagaactattacacaagcttgcaactcatacaagtcaaatcccatcattcttccag
cttctctactttctggatgtggccatctcatctgttaatattagattcaatttccacact
ggaattctagttggacttttctcaatagcacacacaggacatcttctagacatcacaata
ccagcctctcgagctccactaattcatacctcactctcttacttaacattctttggtggt
ttaaaatcgaatacaacttcattgtaccttacagatattgcacatcatcacttagcaatt
ggtatcacattcatccatacaggtcatctatactcctctttcagggcagcccttggaaca
tatatacgagatacattatatacatcacaccttacacacagcataaaatcactacactta
gccctctcactcattctagccagttgtacagcacttacatctaccaccactcagcacatc
tattctttaacaccatacttttatttgtcttatgatcacatctattttacagctctatat
gtccatcactcctacatcacatcattcttagctattgcttctcatgcacatacagctatc
accttagtaagagattggatcacaccattagaactagaatccagctctaagcagataaga
atacatacacataaagctgccattattccacacttatcctgggtcagtctttggcttggt
tttcacacactagcagtatactctcataatgacacatgtatagcatttaactctccatct
aagcaaatcttaatagaagcaagcaatgctcaacttattcaacaagcatcaggaaaagct
ttatatgggaccatcaactccatcaacaactataataaatcatttgattcatttatacac
cctatcagtccaggtgattcatatgtacatcatgcaatagcacttggcttacatataact
gtcctcatcctactaaagggaggtcttgaagctcgtgggtctaagctaatgccagacaag

atggagcactcatttggcttttcatgtgatggtccaggtagaggtggcacatgtgatata
tcagcttgggattccttttatctagcaacattctggatgcttaatagcaacacatggata
agcttctacttccactacaagcaccttacacctcgtcaattctcagaaagttcaacttat
ctagaatcatggtttcgagattatctgtggtttaactctacacctttaattcatggttat
tctacactaggaactaatgatctatccgtccaatcctggtccttcctacttactcacctt
gcatgggcaagtggatttatgttccttattagttggagaggttactggcaagaattaatt
gatatcatcctctacatacattt-aaaacaccaattcttatcaatctttggaatggagac
atctatacacctctcgccttatctattgtacaagctagatttattgggcttgttcacttc
tcaaccggtctaattttaacttaccctccttttataataggtgctacaagttag
>Isy15_psaB
ttttccattgcgaaattacggttagcctccaggccaaatccacatcaaaatcaccaatgc
acacaccaatgcaccttgactgatagctttatatgcttaaatggtagatgtgctagttca
agatatttacaagttctgggttctattcatgatatagaatgtggctttggtatagataat
actctatctctaaatctacagatctttacagcacattggggtcatctaactataatctta
atatgggtatcaagtaatctttatcacatagcctccaatgctaactattctctctgggtt
caaaatccaattccaagcatacctatagcacacaatatttgggatccacatttcactaac
tctacttccactccatattcccataccatcatcaccacaatcctcattgcttattcagga
atctataaccaactatacacttctggatttaactctattaatcagatatataagaccaca
tttacactttcttgtttggcagtaatatcaatactattagccaagatacacatcaacact
cattcagaactattacataagcttgcaagtcatacaagtcaaatcccatcattcttccag
cttctctactttcttgatgttggcatctcatctgttaatattcgattcaattttcacact
ggaattctagttggacttttctcaatagcacacacaggacatcttctagacatcacaata
ccagcctctcgagctccactaattcacacctcaccctcttacttaacattcctgggtggt
ttaaaatcgaatacaacttcattgtaccttacagatattgcacatcatcacttagcaatt
ggtatcacatccatacacacaggtcatctatactcctctttcagggcagcccttggaaca
tatatacgagatacattatatacatcacaccttacacatagtataaaatcactccactta
gccctctcactcattctagccagttgtgcagcacttacatctatcaccgctcaacacatc
tattctttaacaccttacttttatttgtcttatgatcacgtctattctgcagctctctat
gtccatcactcctatatcacatcattattagcaataggttcacatgcacatgcagctatc
accttagtaagagattggatcacaccattagaactagaatccagctctaagcagataaga
atacatacacataaagctgccattattccacacttatcctggattagtctttggcttggt
tttcacacactagcagtatactctcataatgacacatgtatagcatttaactctccatct
aagcaaatcttaatagaagcaagcaatgctcaacttattcaacaagcatcaggaaaagct
ttatatgggaccatcaactccatcaacaactataataaatcatttgattcatttatatac
cctatcagcccaggtgatttatatgtacatcatgcaatagcactgggtttgcatgttact
gtactcatcctactaaaaggagggcttgaagctcgtgggtctaagctaatgccagacaag
atggaacactcatttggcttttcatgtgatggtccaggtagaggtggtacgtgtgatata
tctgcttgggattctttttatctagcaacattctggatgcttaatagtaatacatggata
agcttctacttccactacaagcaccttacacctcgccaattctcagaaagttcaacttat
ctagaatcgtggtttcgagattatctgtggtttaattctacacctttaattcacggttat
tctacactagggactaatgacttatctgtacagtcctggtccttcctccttactcacctt
gcatgggcaagtggatttatgttccttattagttggagaggttactggcaggaattaatt
gatatcatcctctacatacatttgaaaacaccaattcttataaatctttggaatggagac
atctatacacctctcgccttatctattgtacaagctagatttattgggcttgttcacttc
tcaaccggtctaattttaacttacccccccttttataataggtgctacaagttag
>Isy16_psaB
ttttccattgcgaaattacggttagcctccaggccaaatccacatcaaaatcaccaatgc
acacaccaatgcaccttgactgatagctttatatctttaaatggtaggtgtgcgacttca
agatatttacaagtggtgggctctattcatgatatagaatcttactttggtatagataat
actctttctttaaatctacagatattcacagcacattggggtcacctaactataatctta
atatgggtatcaggcaatctttatcacatagcatccaatgctaactattctctctgggtt
aaaaatccaattccaagcatacctatagcacataatatttgggatccacattttactaac
tctacttccactccatattcccataccatcatcaccacaatcctcattgcttactcaggt
atttataaccaactatacacttctggatttaacactattaatcagatatacaagaccaca
tttacatcctcttgcctggcagtaatatcaatactattagctaagatacatatcaatact
cattcagaactattacacaagcttgcaactcatgcaagtcaaatcccatcattcttccag
cttctctactttctggatgtcggtatctcatctgttaatattcgattcaattttcacact

ggaattctagttggacttttctcaatagcacacacaggacatcttctagacatcacaata
ccagcctctcgagctccactaattcatacctcactctcttacttaacattctttggtggt
ttaaaatcgaatacaacttcattgtaccttacagatattgcacatcatcacttagcaatt
ggtatcacattcatccatacagggcatctatactcctctttcagggcagcccttggaaca
tatatacgagatacattatatacatcacaccttacacacagcataaaatcactacactta
gccctctcactcattctagccagttgtacagcacttacatctaccaccactcagcacatc
tattctttaacaccatacttttatttgtcttatgatcacatctattttacagctctatat
gtccatcactcctacatcacatcattcttaaccattggttctcatgcacatacagctatc
accttagtaagagattggatcacaccattagaactagaatccagctctaagcggataaga
atacatacacataaagctgccattattccacacttatcctgggtcagtctttggcttggt
tttcacacactagcagtatactctcataatgacacatgtatagcatttaactctccatct
aagcaaatcttaatagaagcaagcaatgctcaacttattcaacaagcatcaggaaaagct
ttatatgggaccatcaactccatcaacaactataataaatcatttgattcatttatacac
cctatcagtccaggtgattcatatgtacatcatgcaatagcacttggcttacatataact
gtcctcatcctactaaagggaggtcttgaagctcgtgggtctaagctaatgccagacaag
atggagcactcatttggcttttcatgtgatggtccaggtagaggtggcacatgtgatata
tcagcttgggattccttttatctagcaacattctggatgcttaatagcaacacatggata
agcttctacttccactacaagcaccttacacctcgtcaattctcagaaagttcaacttat
ctagaatcatggtttcgagattatctgtggtttaactctacacctttaattcatggttat
tctacactaggaactaatgatctatccgtccaatcctggtccttcctacttactcacctt
gcatgggcaagtggatttatgttccttattagttggagaggttactggcaagaattaatt
gatatcatcctctacatacatttgaaaacaccaattcttatcaatctttggaatggagac
atctatacacctctcgccttatctattgtacaagctagatttattgggcttgttcacttc
tcaaccggtctaattttaacttaccctccttttataataggtgctactagttag
>Isy17_psaB
ttttccattgcgaaattacggttagcctccaggccaaatccacatcaaaatcaccaatgc
acacaccaatgcaccttgactgatagctttatatctttaaatggtaggtgtgcgacttca
agatatttacaagtggtgggctctattcatgatatagaatcttactttggtatagataat
actctttctttaaatctacagatattcacagcacattggggtcacctaactataatctta
atatgggtatcaggcaatctttatcacatagcatccaatgctaactattctctctgggtt
aaaaatccaattccaagtatgcctatagcacataatatttgggatccacattttactaac
tctacttccactccatattcccataccatcatcaccacaatcctcattgcttactcaggt
atttataaccaactatacacttctggatttaacactattaatcagatatacaagaccaca
tttacatcctcttgcctggcagtaatatcaatactattagctaagatacatatcaatact
cattcagaactattacacaagcttgcaactcatgcaagtcaaatcccatcattcttccag
cttctctactttctggatgtcggtatctcatctgttaatattcgattcaattttcacact
ggaattctagttggacttttctcaatagcacacacaggacatcttctagacatcacaata
ccagcctctcgagctccactaattcatacctcaccctcttacttaacattctttggtggt
ttaaaatcgaatacaacttcattgtaccttacagatattgcacatcatcacttagcaatt
ggtatcacattcatccatacagggcatctatactcctctttcagggcagcccttggaaca
tatatacgagatacattatatacatcacaccttacacacagcataaaatcactacactta
gccctctcactcattctagccagttgtacaccacttacatcaaccactgctcagcacatc
tattctttaacaccatacttttatttgtcttatgatcacatctattttacagctctatat
gtccatcactcctacatcacatcattcttaaccattggttctcatgcacatacagctatc
accttagtaagagattggatcacaccattagaactagaatccagctctaagcggataaga
atacatacacataaagctgccattattccacacttatcctgggtcagtctttggcttggt
tttcacacactagcagtatactctcataatgacacatgtatagcatttaactctccatct
aagcaaatcctaatagaagcaagcaatgctcaacttattcaacaagcatcaggaaaagct
ttatatgggaccatcaactccatcaacaactataataaatcatttgattcatttatacac
cctatcagtccaggtgattcatatgtacatcatgcaatagcacttggcttacatataact
gtcctcatcctactaaagggaggtcttgaagctcgtgggtctaagctaatgccagacaag
atggagcactcatttggcttttcatgtgatggtccaggtagaggtggcacatgtgatata
tcagcttgggattccttttatctagcaacattctggatgcttaatagcaacacatggata
agcttctacttccactacaagcaccttacacctcgtcaattctcagaaagttcaacttat
ctagaatcatggtttcgagattatctgtggtttaattctacacctttaattcatggttat
tctacactaggagctaatgatctatccgtccaatcctggtccttcctacttactcacctt
gcatgggcaagtggatttatgttccttattagttggagaggttactggcaagaattaatt

gatatcatcctttacatacatttgaaaacaccaattcttattaatctttggaatggagat
atctatacacctctagccttatctattgtacaagctagatttattgggcttgttcacttc
tcaaccggtctaattttaacttaccctccttttataataggtgctacaagttag
>Isy18_psaB
ttttccattgcgaaattacggttagcctccaggccaaatccacatcaaaatcaccaatgc
acacaccaatgcaccttgactgatagctttatatgtttaaatggtaggtgtgcgacttca
agatatttacaagtggtgggctctattcatgatatagaatcttactttggtatagataat
actctttctttaaatctacagatattcacagcacattggggtcacctaactataatctta
atatgggtatcaggcaatctttatcacatagcatccaatgctaactattctctctgggtt
aaaaatccaattccaagcatacctatagcacataatatttgggatccacattttactaac
tctacttccactccatattcccataccattatcaccacaatcctcattgcttactcaggt
atttataaccaactatacacttctggatttaacactattaatcagatatacaagaccaca
tttacatcctcttgcctggcagtaatatcaatactattagctaagatacatatcaatact
cattcagaactattacataagcttgcaagtcatacaagtcaaatcccatcattcttccag
cttctctactttctggatgtcggtatctcatctgttaatattcgattcaattttcacact
ggaattctagttggacttttctcaatagcacacacaggacatcttctagacatcacaata
ccagcctctcgagctccactaattcatacctcaccctcttacttaacattctttggtggt
ttaaaatcgaatacaacttcattgtaccttacagatattgcacatcatcacttagcaatt
ggtatcacattcatccatacagggcatctatactcctctttcagggcagcccttggaaca
tatataagagatatattatatacatcacaccttacacacagcataaaatcactccactta
gccctctcacttattctagccagttgtacaccacttacatcaaccactgctcagcacatc
tattctttaacaccatacttttatttgtcttatgatcacatctattttacagctctatat
gtccatcactcctacatcacatcattcttaaccattggttctcatgcacatacagctatc
accttagtaagagattggatcacaccattagaactagaatccagctctaagcggataaga
atacatacacataaagctgccattattccacacttatcctgggtcagtctttggcttggt
tttcacacactagcagtatactctcataatgacacatgtatagcatttaactctccatct
aagcaaatcttaatagaagcaagcaatgctcaacttattcaacaagcatcaggaaaagct
ttatatgggaccatcaactccatcaacaactataataaatcatttgattcatttatacac
cctatcagtccaggtgattcatatgtacatcatgcaatagcacttggcttacatataact
gtcctcatcctactaaagggaggtcttgaagctcgtgggtctaagctaatgccagacaag
atggagcactcatttggcttttcatgtgatggtccaggtagaggtggcacatgtgatata
tcagcttgggattccttttatctagcaacattctggatgcttaatagcaacgcatggata
agcttctacttccactacaagcaccttacacctcgtcaattctcagaaagttcaacttat
ctagaatcatggtttcgagattatctgtggtttaactctacacctttaattcatggttat
tctacactaggaactaatgatctatccgtccaatcctggtccttcctacttactcacctt
gcatgggcaagtggatttatgttccttattagttggagaggttactggcaagaattaatt
gatatcatcctttacatacatttgaaaacaccaattcttatcaatctttggaatggagac
atctatacacctctcgccttatctattgtacaagctagatttattgggcttgttcacttc
tcaaccggtctaattttaacttaccctccttttataataggtgctactagttag
>Isy21_psaB
ttttccattgcgaaattacggttagcctccaggccaaatccacatcaaaatcaccaatgc
acacaccaatgcaccttgactgatagctttatatctttaaatggtaggtgtgcgacttca
agatatttacaagtggtgggctctattcatgatatagaatcttactttggtatagataat
actctttctttaaatctacagatattcacagcacattggggtcacctaactataatctta
atatgggtatcaggtaatctttatcacatagcatccaatgctaactattctctctgggtt
aaaaatccaattccaagcatacctatagcacataatatttgggatccacattttactaac
tctacttccactccatattcccataccatcatcaccacaatcctcattgcttactcaggt
atttataaccaactatacacttctggatttaacactattaatcagatatacaagaccaca
tttacatcctcttgcctggcagtaatatcaatactattagctaagatacacatcaatact
cattcagaactattacacaagcttgcaactcatgcaagtcaaatcccatcattcttccag
cttctctactttctggatgtcggtatctcatctgttaatattcgattcaattttcacact
ggaattctagttggacttttctcaatagcacacacaggacatcttctagacatcacaata
ccagcctctcgagctccactaattcatacctcactctcttacttaacattctttggtggt
ttaaaatcgaatacaacttcattgtaccttacagatattgcacatcatcacttagcaatt
ggtatcacattcatccatacaggtcatctatactcctctttcagggcagcccttggaaca
tatatacgagatacattatatacatcacaccttacacacagcataaaatcactacactta
gccctctcactcattctagccagttgtacagcacttacatctaccaccactcagcacatc

tattctttaacaccatacttttatttgtcttatgatcacatctattttacagctctatat
gtccatcactcctacatcacatcattcttaaccattggttctcatgcacatacagctatc
accttagtaagagattggatcacaccattagaactagaatccagctctaagcagataaga
atacatacacataaagctgccattattccacacttatcctgggtcagtctttggcttggt
tttcacacactagcagtatactctcataatgacacatgtatagcatttaactctccatct
aagcaaatcttaatagaagcaagcaatgctcaacttattcaacaagcatcaggaaaagct
ttatatgggaccatcaactccatcaacaactataataaatcatttgattcatttatacac
cctatcagtccaggtgattcatatgtacatcatgcaatagcacttggcttacatataact
gtcctcatcctactaaagggaggtcttgaagctcgtgggtctaagctaatgccagacaag
atggagcactcatttggcttttcatgtgatggtccaggtagaggtggcacatgtgatata
tcagcttgggattccttttatctagcaacattctggatgcttaatagcaacacatggata
agcttctacttccactacaagcaccttacacctcgtcaattctcagaaagttcaacttat
ctagaatcatggtttcgagattatctgtggtttaactctacacctttaattcatggttat
tctacactaggaactaatgatctatccgtccaatcctggtccttcctacttactcacctt
gcatgggcaagtggatttatgttccttattagttggagaggttactggcaagaattaatt
gatatcatcctctacatacattt-aaaacaccaattcttatcaatctttggaatggagac
atctatacacctctcgccttatctattgtacaagctagatttattgggcttgttcacttc
tcaaccggtctaattttaacttaccctccttttataataggtgctactagttag
>Isy22_psaB
ttttccattgcgaaattacggttagcctccaggccaaatccacatcaaaatcaccaatgc
acacaccaatgcaccttgactgatagctttatatccttaaatggtaggtgtgcgacttca
agatatttacaagtggtgggctctattcatgatatagaatcttactttggtatagataat
actctttctttaaatctacagatattcacagcacattggggtcacctaactataatctta
atatgggtatcaggcaatctttatcacatagcatccaatgctaactattctctctgggtt
aaaaatccaattccaagcatacctatagcacataatatttgggatccacatttcactaac
tctacttccactccatattcccataccattatcaccacaatcctcattgcttactcaggt
atttataaccaattatacacttctggatttaacactattaatcagatatacaagaccaca
tttacattctcttgccttgcagtaatatcaatactattagctaagatacatatcaatact
cattcagaactattacacaagcttgcaactcatgcaagtcaaatcccatcattcttccag
cttctctactttctggatgtcggtatctcatctgttaatattcgattcaattttcacact
ggaattctagttggacttttctcaatagcacacacaggacatcttctagacatcacaata
ccagcctctcgagctccactaattcatacctcactctcttacttaacattctttggtggt
ttaaaatcgaatacaacttcattgtaccttacagatattgcacatcatcacttagcaatt
ggtatcacattcatccatacaggtcatctatactcctctttcagggcagcccttggaaca
tatatacgagatacattatatacatcacaccttacacacagcataaaatcactacactta
gccctctcactcattctagccagttgtacagcacttacatctaccaccactcagcacatc
tattctttaacaccatacttttatttgtcttatgatcacatctattctacagctctatat
gtccatcactcctacatcacatcattcttaaccattggttctcatgcacatacagctatc
accttagtaagagattggatcacaccattagaactagaatccagctctaagcggataaga
atacatacacataaagctgccattattccacacttatcctgggtcagtctttggcttggt
tttcacacactagcagtatactctcataatgacacatgtatagcatttaactctccatct
aagcaaatcttaatagaagcaagcaatggtcaacttattcaacaagcatcaggaaaagct
ttatatgggaccatcaactccatcaacaactataataaatcatttgattcatttatacac
cctatcagtccaggtgattcatatgtacatcatgcaatagcacttggcttacatataact
gtcctcatcctactaaagggaggtcttgaagctcgtgggtctaagctaatgccagacaag
atggagcactcatttggcttttcatgtgatggtccaggtagaggtggcacatgtgatata
tcagcttgggattccttttatctagcaacattctggatgcttaatagcaacacatggata
agcttctacttccactacaagcaccttacacctcgtcaattctcagaaagttcaacttat
ctagaatcatggtttcgagattatctgtggtttaactctacacctttaattcatggttat
tctacactaggagctaatgatctatccgtccaatcttggtccttcctacttactcatctt
gcttgggcaagtggatttatgttccttattagttggagaggttactggcaagaattaatt
gatatcatcctctacatacatttgaaaacaccaattcttatcaatctttggaatggagac
atctatacacctctcgccttatctattgtacaagctagatttattgggcttgttcacttc
tcaaccgggctaattttaacttaccctccttttataataggtgctactagttag
>Isy23_psaB
ttttccattgcgaaattacggttagcctccaggccaaatccacatcaaaatcaccaatgc
acacaccaatgcaccttgactgatagctttatatctttaaatggtaggtgtgcgacttca

agatatttacaagtggtgggctctattcatgatatagaatcttactttggtatagataat
actctttctttaaatctacagatattcacagcacattggggtcacctaactataatctta
atatgggtatcaggtaatctttatcacatagcatccaatgctaactattctctctgggtt
aaaaatccaattccaagcatacctatagcacataatatttgggatccacattttactaac
tctacttccactccatattcccataccatcatcaccacaatcctcattgcttactcaggt
atttataaccaactatacacttctggatttaacactattaatcagatatacaagaccaca
tttacatcctcttgcctggcagtaatatcaatactattagctaagatacatatcaatact
cattcagaactattacacaagcttgcaactcatgcaagtcaaatcccatcattcttccag
cttctctactttctggatgtcggtatctcatctgttaatattcgattcaattttcacact
ggaattctagttggacttttctcaatagcacacacaggacatcttctagacatcacaata
ccagcctctcgagctccactaattcatacctcactctcttacttaacattctttggtggt
ttaaaatcgaatacaacttcattgtaccttacagatattgcacatcatcacttagcaatt
ggtatcacattcatccatacaggtcatctatactcctctttcagggcagcccttggaaca
tatatacgagatacattatatacatcacaccttacacacagcataaaatcactacactta
gccctctcactcattctagccagttgtacagcacttacatctaccaccactcagcacatc
tattctttaacaccatacttttatttgtcttatgatcacatctattttacagctctatat
gtccatcactcctacatcacatcattcttaaccattggttctcatgcacatacagctatc
accttagtaagagattggatcacaccattagaactagaatccagctctaagcggataaga
atacatacacataaagctgccattattccacacttatcctgggtcagtctttggcttggt
tttcacacactagcagtatactctcataatgacacatgtatagcatttaactctccatct
aagcaaatcttaatagaagcaagcaatgctcaacttattcaacaagcatcaggaaaagct
ttatatgggaccatcaactccatcaacaactataataaatcatttgattcatttatacac
cctatcagtccaggtgattcatatgtacatcatgcaatagcacttggcttacatataact
gtcctcatcctactaaagggaggtcttgaagctcgtgggtctaagctaatgccagacaag
atggagcactcatttggcttttcatgtgatggtccaggtagaggtggcacatgtgatata
tcagcttgggattccttttatctagcaacattctggatgcttaatagcaacacatggata
agcttctacttccactacaagcaccttacacctcgtcaattctcagaaagttcaacttat
ctagaatcatggtttcgagattatctgtggtttaattctacacctttaattcatggttat
tctacactaggaactaatgatctatccgtccaatcctggtccttcctacttactcacctt
gcatgggcaagtggatttatgttccttattagttggagaggttactggcaagaattaatt
gatatcatcctctacatacatttgaaaacaccaattcttatcaatctttggaatggagac
atctatacacctctcgccttatctattgtacaagctagatttattgggcttgttcacttc
tcaaccggtctaattttaacttaccctcctttataataggtgctacaagttag
>Isy24_psaB
ttttccattgcgaaattacggttagcctccaggccaaatccacatcaaaatcaccaatgc
acacaccaatgcaccttgactgatagctttatatctttaaatggtaggtgtgcgacttca
agatatttacaagtggtgggctctattcatgatatagaatcttactttggtatagataat
actctttctttaaatctacagatattcacagcacattggggtcacctaactataatctta
atatgggtatcaggcaatctttatcacatagcatccaatgctaactattctctctgggtt
aaaaatccaattccaagcatacctatagcacataatatttgggatccacattttactaac
tctacttccactccatattcccataccatcatcaccacaatcctcattgcttactcaggt
atttataaccaactatacacttctggatttaacactattaatcagatatacaagaccaca
tttacatcctcttgccttgcagtaatatcaatactattagctaagatacatatcaatact
cattcagaactattacacaagcttgcaactcatgcaagtcaaatcccatcattcttccag
cttctctactttctggatgtcggtatctcatctgttaatattcgattcaattttcacact
ggaattctagttggacttttctcaatagggtacacaggacatcttctagacatcacaata
ccagcctctcgagctccactaattcatacctcactctcttacttaacattctttggtggt
ttaaaatcgaatacaacttcattgtaccttacagatattgcacatcatcacttagcaatt
ggtatcacattcatccatacagggcatctatactcctctttcagggcagcccttggaaca
tatatacgagatacattatatacatcacaccttacacacagcataaaatcactacactta
gccctctcactcattctagccagttgtacagcacttacatctaccaccactcagcacatc
tattctttaacaccatatttttatttgtcttatgatcacatctattctacggctctctat
gtccatcactcctacatcacctcattcttagctattgcttctcatgcacatacagctatc
accttagtaagagattggatcacaccattagaactagaatccagctctaagcggataaga
atacatacacataaagctgccattattccacacttatcctgggtcagtctttggcttggt
tttcacacactagcagtatactctcataatgacacatgtatagcatttaactctccatct
aagcaaatcttaatagaagcaagcaatgctcaacttattcaacaagcatcaggaaaagct

ttatatgggaccatcaactccatcaacaactataataaatcatttgattcatttatacac
cctatcagtccaggtgattcatatgtacatcatgcaatagcacttggcttacatataact
gtcctcatcctactaaagggaggtcttgaagctcgtgggtctaagctaatgccagacaag
atggagcactcatttggcttttcatgtgatggtccaggtagaggtggcacatgtgatata
tcagcttgggattccttttatctagcaacattctggatgcttaatagcaacacatggata
agcttctacttccactacaagcaccttacacctcgtcaattctcagaaagttcaacttat
ctagaatcatggtttcgagattatctgtggtttaactctacacctttaattcatggttat
tctacactaggaactaatgatctatccgtccaatcctggtccttcctacttactcacctt
gcatgggcaagtggatttatgttccttattagttggagaggttactggcaagaattaatt
gatatcatcctctacatacatttgaaaacaccaattcttatcaatctttggaatggagac
atctatacacctctcgccttatctattgtacaagctagatttattgggcttgttcacttc
tcaaccggtctaattttaacttaccctccttttataataggtgctacaagttag
>Isy25_psaB
ttttccattgcgaaattacggttagcctccaggccaaatccacatcaaaatcaccaatgc
acacaccaatgcaccttgactgatagctttatatctttaaatggtaggtgtgcgacttca
agatatttacaagtggtgggctctattcatgatatagaatcttactttggtatagataat
actctttctttaaatctacagatattcacagcacattggggtcacctaactataatctta
atatgggtatcaggtaatctttatcacatagcatccaatgctaactattctctctgggtt
aaaaatccaattccaagcatacctatagcacataatatttgggatccacattttactaac
tctacttccactccatattcccataccatcatcaccacaatcctcattgcttactcaggt
atttataaccaactatacacttctggatttaacactattaatcagatatacaagaccaca
tttacattctcttgccttgcagtaatttcaatactattagccaagatacacatcaacact
cattcagaactattacacaagcttgcaactcatgcaagtcaaatcccatcattcttccag
cttctctactttctggatgtcggtatctcatctgttaatattcgattcaattttcacact
ggaattctagttggacttttctcaatagcacacacaggacatcttctagacatcacaata
ccagcctctcgagctccactaattcatacctcactctcttacttaacattctttggtggt
ttaaaatcgaatacaacttcattgtaccttacagatattgcacatcatcacttagcaatt
ggtatcacattcatccatacaggtcatctatactcctctttcagggcagcccttggaaca
tatatacgagatacattatatacatcacaccttacacacagcataaaatcactacactta
gccctctcactcattctagccagttgtacagcacttacatctaccaccactcagcacatc
tattctttaacaccatacttttatttgtcttatgatcacatctattttacagctctatat
gtccatcactcctacatcacatcattcttaaccattggttctcatgcacatacagctatc
accttagtaagagattggatcacaccattagaactagaatccagctctaagcagataaga
atacatacacataaagctgccattattccacacttatcctgggtcagtctttggcttggt
tttcacacactagcagtatactctcataatgacacatgtatagcatttaactctccatct
aagcaaatcttaatagaagcaagcaatgctcaacttattcaacaagcatcaggaaaagct
ttatatgggaccatcaactccatcaacaactataataaatcatttgattcatttatacac
cctatcagtccaggtgattcatatgtacatcatgcaatagcacttggcttacatataact
gtcctcatcctactaaagggaggtcttgaagctcgtgggtctaagctaatgccagacaag
atggagcactcatttggcttttcatgtgatggtccaggtagaggtggcacatgtgatata
tcagcttgggattccttttatctagcaacattctggatgcttaatagcaacgcatggata
agcttctacttccactacaagcaccttacacctcgtcaattctcagaaagttcaacttat
ctagaatcatggtttcgagattatctgtggtttaactctacacctttaattcatggttat
tctacactaggaactaatgatctatccgtccaatcctggtccttcctacttactcacctt
gcatgggcaagtggatttatgttccttattagttggagaggttactggcaagaattaatt
gatatcatcctctacatacattt-aaaacaccaattcttatcaatctttggaatggagac
atctatacacctctcgccttatctattgtacaagctagatttattgggcttgttcacttc
tcaaccggtctaattttaacttaccctccttttataataggtgctactagttag
>Isy26_psaB
ttttccattgcgaaattacggttagcctccaggccaaatccacatcaaaatcaccaatgc
acacaccaatgcaccttgactgatagctttatatctttaaatggtaggtgtgcgacttca
agatatttacaagtggtgggctctattcatgatatagaatcttactttggtatagataat
actctttctttaaatctacagatattcacagcacattggggtcacctaactataatctta
atatgggtatcaggtaatctttatcacatagcatccaatgctaactattctctctgggtt
aaaaatccaattccaagcatacctatagcacataatatttgggatccacattttactaac
tctacttccactccatattcccataccatcatcaccacaatcctcattgcttactcaggt
atttataaccaactatacacttctggatttgacactattaatcagatatacaagaccaca

tttacattctcttgccttgcagtaatttcaatactattagctaagatacacatcaatact
cattcagaactattacacaagcttgcaactcatgcaagtcaaatcccatcattcttccag
cttctctactttctggatgtcggtatctcatctgttaatattcgattcaattttcacact
ggaattctagttggacttttctcaatagcacacacaggacatcttctagacatcacaata
ccagcctctcgagctccactaattcatacctcactctcttacttaacattctttggtggt
ttaaaatcgaatacaacttcattgtaccttacagatattgcacatcatcacttagcaatt
ggtatcacattcatccatacaggtcatctatactcctctttcagggcagcccttggaaca
tatatacgagatacattatatacatcacaccttacacacagcataaaatcactacactta
gccctctcactcattctagccagttgtacagcacttacatctaccaccactcagcacatc
tattctttaacaccatacttttatttgtcttatgatcacatctattttacagctctatat
gtccatcactcctacatcacatcattcttaaccattggttctcatgcacatacagctatc
accttagtaagagattggatcacaccattagaactagaatccagctctaagcagataaga
atacatacacataaagctgccattattccacacttatcctgggtcagtctttggcttggt
tttcacacactagcagtatactctcataatgacacatgtatagcatttaactctccatct
aagcaaatcttaatagaagcaagcaatgctcaacttattcaacaagcatcaggaaaagct
ttatatgggaccatcaactccatcaacaactataataaatcatttgattcatttatacac
cctatcagtccaggtgattcatatgtacatcatgcaatagcacttggcttacatataact
gtcctcatcctactaaagggaggtcttgaagctcgtgggtctaagctaatgccagacaag
atggagcactcatttggcttttcatgtgatggtccaggtagaggtggcacatgtgatata
tcagcttgggattccttttatctagcaacattctggatgcttaatagcaacacatggata
agcttctacttccactacaagcaccttacacctcgtcaattctcagaaagttcaacttat
ctagaatcatggtttcgagattatctgtggtttaactctacacctttaattcatggttat
tctacactaggaactaatgatctatccgtccaatcctggtccttcctacttactcacctt
gcatgggcaagtggatttatgttccttattagttggagaggttactggcaagaattaatt
gatatcatcctctacatacattt-aaaacaccaattcttatcaatctttggaatggagac
atctatacacctctcgccttatctattgtacaagctagatttattgggcttgttcacttc
tcaaccggtctaattttaacttaccctccttttataataggtgctactagttag
>Isy27_psaB
ttttccattgcgaaattacggttagcctccaggccaaatccacatcaaaatcaccaatgc
acacaccaatgcaccttgactgatagctttatatctttaaatggtaggtgtgcgacttca
agatatttacaagtggtgggctctattcatgatatagaatcttactttggtatagataat
actctttctttaaatctacagatattcacagcacattggggtcacctaactataatctta
atatgggtatcaggtaatctttatcacatagcatccaatgctaactattctctctgggtt
aaaaatccaattccaagcatacctatagcacataatatttgggatccacattttactaac
tctacttccactccatattcccataccatcatcaccacaatcctcattgcttactcaggt
atttataaccaactatacacttctggatttaacactattaatcagatatacaagaccaca
tttacattctcttgccttgcagtaatatcaatactattagctaagatacatatcaatact
cattcagaactattacacaagcttgcaactcatgcaagtcaaatcccatcattcttccag
cttctctactttctggatgtcggtatctcatctgttaatattcgattcaattttcacact
ggaattctagttggacttttctcaatagggcacacaggacatcttctagacatcacaata
ccagcctctcgagctccactaattcatacctcactctcttacttaacattctttggtggt
ttaaaatcgaatacaacttcattgtaccttacagatattgcacatcatcacttagcaatt
ggtatcacattcatccatacaggtcatctatactcctctttcagggcagcccttggaaca
tatatacgagatacattatatacatc-caccttacacacagcataaaatcactacactta
gccctctcactcattctagccagttgtacagcacttacatctaccaccactcagcacatc
tattctttaacaccatacttttatttgtcttatgatcacatctattttacagctctatat
gtccatcactcctacatcacatcattcttaaccattggttctcatgcacatacagctatc
accttagtaagagattggatcacaccattagaactagaatccagctctaagcagataaga
atacatacacataaagctgccattattccacacttatcctgggtcagtctttggcttggt
tttcacacactagcagtatactctcataatgacacatgtatagcatttaactctccatct
aagcaaatcttaatagaagcaagcaatgctcaacttattcaacaagcatcaggaaaagct
ttatatgggaccatcaactccatcaacaactataataaatcatttgattcatttatacac
cctatcagtccaggtgattcatatgtacatcatgcaatagcacttggcttacatataact
gtcctcatcctactaaagggaggtcttgaagctcgtgggtctaagctaatgccagacaag
atggagcactcatttggcttttcatgtgatggtccaggtagaggtggcacatgtgatata
tcagcttgggattccttttatctagcaacattctggatgcttaatagcaacacatggata
agcttctacttccactacaagcaccttacacctcgtcaattctcagaaagttcaacttat

ctagaatcatggtttcgagattatctgtggtttaactctacacctttaattcatggttat
tctacactaggaactaatgatctatccgtccaatcctggtccttcctacttactcacctt
gcatgggcaagtggatttatgttccttattagttggagaggttactggcaagaattaatt
gatatcatcctctacatacattt-aaaacaccaattcttatcaatctttggaatggagac
atctatacacctctcgccttatctattgtacaagctagatttattgggcttgttcacttc
tcaaccggtctaattttaacttaccctccttttataataggtgctactagttag
>Isy4_psaB
ttttccattgcgaaattacggttagcctccaggccaaatccacatcaaaatcaccaatgc
acacaccaatgcaccttgactgatagctttatatgcttaaatggtaggtgtgcgacttca
agatatttacaagtggtgggctctattcatgatatagaatcttactttggtatagataat
actctttctttaaatctacagatattcacagcacattggggtcacctaactataatctta
atatgggtatcaggcaatctttatcacatagcatccaatgctaactattctctctgggtt
aaaaatccaattccaagcatacctatagcacataatatttgggatccacattttactaac
tctacttccactccatattcccataccatcatcaccacaatcctcattgcttactcaggt
atttataaccaactatacacttctggatttaacactattaatcagatatacaagaccaca
tttacatcctcttgcctggcagtaatatcaatactattagctaagatacatatcaatact
cattcagaactattacacaagcttgcaactcatgcaagtcaaatcccatcattcttccag
cttctctactttctggatgtcggtatctcatctgttaatattcgattcaattttcacact
ggaattctagttggacttttctcaatagcacacacaggacatcttctagacatcacaata
ccagcctctcgagctccactaattcatacctcactctcttacttaacattctttggtggt
ttaaaatcgaatacaacttcattgtaccttacagatattgcacatcatcacttagcaatt
ggtatcacattcatccatacagggcatctatactcctctttcagggcagcccttggaaca
tatatacgagatacattatatacatcacaccttacacacagcataaaatcactacactta
gccctctcactcattctagccagttgtacagcacttacatctaccaccactcagcacatc
tattctttaacaccatacttttatttgtcttatgatcacatctattttacagctctatat
gtccatcactcctacatcacatcattcttaaccattggttctcatgcacatacagctatc
accttagtaagagattggatcacaccattagaactagaatccagctctaagcggataaga
atacatacacataaagctgccattattccacacttatcctgggtcagtctttggcttggt
tttcacacactagcagtatactctcataatgacacatgtatagcatttaactctccatct
aagcaaatcttaatagaagcaagcaatgctcaacttattcaacaagcatcaggaaaagct
ttatatgggaccatcaactccatcaacaactataataaatcatttgattcatttatacac
cctatcagtccaggtgattcatatgtacatcatgcaatagcacttggcttacatataact
gtcctcatcctactaaagggaggtcttgaagctcgtgggtctaagctaatgccagacaag
atggagcactcatttggcttttcatgtgatggtccaggtagaggtggcacatgtgatata
tcagcttgggattccttttatctagcaacattctggatgcttaatagcaacacatggata
agcttctacttccactacaagcaccttacacctcgtcaattctcagaaagttcaacttat
ctagaatcatggtttcgagattatctgtggtttaactctacacctttaattcatggttat
tctacactaggaactaatgatctatccgtccaatcctggtccttcctacttactcacctt
gcatgggcaagtggatttatgttccttattagttggagaggttactggcaagaattaatt
gatatcatcctctacatacatttgaaaacaccaattcttatcaatctttggaatggagac
atctatacacctctcgccttatctattgtacaagctagatttattgggcttgttcacttc
tcaaccggtctaattttaacttaccctccttttataataggtgctactagttag
>Isy7_psaB
ttttccattgcgaaattacggttagcctccaggccaaatccacatcaaaatcaccaatgc
acacaccaatgcaccttgactgatagctttatatctttaaatggtaggtgtgcgacttca
agatatttacaagtggtgggctctattcatgatatagaatcttactttggtatagataat
actctttctttaaatctacagatattcacagcacattggggtcatctaactataatctta
atatgggtatcaggtaatctttatcacatagcatccaatgctaactattctctctgggtt
aaaaatccaattccaagcatacctatagcacataatatttgggatccacattttactaac
tctacttccactccatattcccataccatcatcaccacaatcctcattgcttactcaggt
atttataaccaactatacacttctggatttaacactattaatcagatatacaagaccaca
tttacatcctcttgcctggcagtaatatcaatactattagctaagatacatatcaatact
cattcagaactattacataagcttgcaagtcatgcaagtcaaatcccatcattcttccag
cttctctactttctggatgtcggtatctcatctgttaatattcgattcaattttcacact
ggaattctagttggacttttctcaatagcacacacaggacatcttctagacatcacaata
ccagcctctcgagctccactaattcatacctcactctcttacttaacattctttggtggt
ttaaaatcgaatacaacttcattgtaccttacagatattgcacatcatcacttagcaatt

ggtatcacattcatccatacagggcatctatactcctctttcagggcagcccttggaaca
tatatacgagatacattatatacatcacaccttacacacagtataaaatcactacactta
gccctctcactcattctagccagttgtgcagcacttacatctatcaccactcaacacatc
tattctttaacaccttacttttatttgtcttatgatcacgtctattttgcagctctatat
gtccatcactcctatatcacatcattattaaccataggttcacatgcacatgcagctatc
accttagtaagagattggatcacaccattagaactagaatccagctctaagcggataaga
atacatacacataaagctgccattattccacacttatcctgggtcagtctttggcttggt
tttcacacactagcagtatactctcataatgacacatgtatagcatttaactctccatct
aagcaaatcttaatagaagcaagcaatgctcaacttattcaacaagcatcaggaaaagct
ttatatgggaccatcaactccatcaacaactataataaatcatttgattcatttatatac
cctatcagcccaggtgatttatatgtacatcatgcaatagcactgggcttgcatgttact
gtactcatcctactaaaaggagggcttgaagctcgtgggtctaagctaatgccagacaag
atggagcactcatttggcttttcatgtgatggtccaggtagaggtggcacatgtgatata
tcagcttgggattctttttatctagcaacattctggatgcttaatagtaatacatggata
agcttctacttccactacaagcaccttacacctcgccaattctcagaaagttcaacttat
ctagaatcgtggtttcgagattatctgtggtttaactctacacctttaattcatggttat
tctacactagggactaatgacttatctgtacaatcttggtccttcctccttactcatctt
gcatgggcaagtggatttatgttccttattagttggagaggttactggcaagaattaatt
gatatcatcctctacatacatttgaaaacaccaattcttatcaatctttggaatggagac
atctatacacctctcgccttatctattgtacaagctagatttattgggcttgttcacttc
tcaaccggtctaattttaacttaccctccttttataataggtgctactagttag
>Isy8_psaB
ttttccattgcgaaattacggttagcctccaggccaaatccacatcaaaatcaccaatgc
acacaccaatgcaccttgactgatagctttatatctttaaatggtaggtgtgcgacttca
agatatttacaagtggtgggctctattcatgatatagaatcttactttggtatagataat
actctttctttaaatctacagatattcacagcacattggggtcacctaactataatctta
atatgggtatcaggtaatctttatcacatagcatccaatgctaactattctctctgggtt
aaaaatccaattccaagcatacctatagcacataatatttgggatccacattttactaac
tctacttccactccatattcccataccatcatcaccacaatcctcattgcttactcaggt
atttataaccaactatacacttctggatttaacactattaatcagatatacaagaccaca
tttacatcctcttgcctggcagtaatatcaatactattagctaagatacacatcaatact
cattcagaactattacacaagcttgcaactcatgcaagtcaaatcccatcattcttccag
cttctctactttctggatgtcggtatctcatctgttaatattcgattcaattttcacact
ggaattctagttggacttttctcaatagcacacacaggacatcttctagacatcacaata
ccagcctctcgagctccactaattcatacctcactctcttacttaacattctttggtggt
ttaaaatcgaatacaacttcattgtaccttacagatattgcacatcatcacttagcaatt
ggtatcacattcatccatacaggtcatctatactcctctttcagggcagcccttggaaca
tatatacgagatacattatatacatcacaccttacacacagcataaaatcactacactta
gccctctcactcattctagccagttgtacagcacttacatctaccaccactcagcacatc
tattctttaacaccatacttttatttgtcttatgatcacatctattttacagctctatat
gtccatcactcctacatcacatcattcttaaccattggttctcatgcacatacagctatc
accttagtaagagattggatcacaccattagaactagaatccagctctaagcagataaga
atacatacacataaagctgccattattccacacttatcctgggtcagtctttggcttggt
tttcacacactagcagtatactctcataatgacacatgtatagcatttaactctccatct
aagcaaatcttaatagaagcaagcaatgctcaacttattcaacaagcatcaggaaaagct
ttatatgggaccatcaactccatcaacaactataataaatcatttgattcatttatacac
cctatcagtccaggtgattcatatgtacatcatgcaatagcacttggcttacatataact
gtcctcatcctactaaagggaggtcttgaagctcgtgggtctaagctaatgccagacaag
atggagcactcatttggcttttcatgtgatggtccaggtagaggtggcacatgtgatata
tcagcttgggattccttttatctagcaacattctggatgcttaatagcaacacatggata
agcttctacttccactacaagcaccttacacctcgtcaattctcagaaagttcaacttat
ctagaatcatggtttcgagattatctgtggtttaactctacacctttaattcatggttat
tctacactaggaactaatgatctatccgtccaatcctggtccttcctacttactcacctt
gcatgggcaagtggatttatgttccttattagttggagaggttactggcaagaattaatt
gatatcatcctctacatacattt-aaaacaccaattcttatcaatctttggaatggagac
atctatacacctctcgccttatctattgtacaagctagatttattgggcttgttcacttc
tcaaccggtctaattttaacttaccctccttttataataggtgctactagttag

>KrA1_psaB
ttttccattgcgaaattacggttagcctccaggccaaatccacatcaaaatcaccaatgc
acacaccaatgcaccttgactgatagctttatatctttaaatggtaggtgtgcgacttca
agatatttacaagtggtgggctctattcatgatatagaatcttactttggtatagataat
actctttctttaaatctacagatattcacagcacattggggtcacctaactataatctta
atatgggtatcaggcaatctttatcacatagcatccaatgctaactattctctctgggtt
aaaaatccaattccaagcatacctatagcacataatatttgggatccacattttactaac
tctacttccactccatattcccataccatcatcaccacaatcctcattgcttactcaggt
atttataaccaactatacacttctggatttaacactattaatcagatatacaagaccaca
tttacatcctcttgcctggcagtaatatcaatactattagctaagatacatatcaatact
cattcagaactattacacaagcttgcaactcatgcaagtcaaatcccatcattcttccag
cttctctactttctggatgtcggtatctcatctgttaatattcgattcaattttcacact
ggaattctagttggacttttctcaatagcacacacaggacatcttctagacatcacaata
ccagcctctcgagctccactaattcatacctcactctcttacttaacattctttggtggt
ttaaaatcgaatacaacttcattgtaccttacagatattgcacatcatcacttagcaatt
ggtatcacattcatccatacagggcatctatactcctctttcagggcagcccttggaaca
tatatacgagatacattatatacatcacaccttacacacagcataaaatcactacactta
gccctctcactcattctagccagttgtacagcacttacatctaccaccactcagcacatc
tattctttaacaccatacttttatttgtcttatgatcacatctattttacagctctatat
gtccatcactcctacatcacatcattcttaaccattggttctcatgcacatacagctatc
accttagtaagagattggatcacaccattagaactagaatccagctctaagcggataaga
atacatacacataaagctgccattattccacacttatcctgggtcagtctttggcttggt
tttcacacactagcagtatactctcataatgacacatgtatagcatttaactctccatct
aagcaaatcttaatagaagcaagcaatgctcaacttattcaacaagcatcaggaaaagct
ttatatgggaccatcaactccatcaacaactataataaatcatttgattcatttatacac
cctatcagtccaggtgattcatatgtacatcatgcaatagcacttggcttacatataact
gtcctcatcctactaaagggaggtcttgaagctcgtgggtctaagctaatgccagacaag
atggagcactcatttggcttttcatgtgatggtccaggtagaggtggcacatgtgatata
tcagcttgggattccttttatctagcaacattctggatgcttaatagcaacacatggata
agcttctacttccactacaagcaccttacacctcgtcaattctcagaaagttcaacttat
ctagaatcatggtttcgagattatctgtggtttaactctacacctttaattcatggttat
tctacactaggaactaatgatctatccgtccaatcctggtccttcctacttactcacctt
gcatgggcaagtggatttatgttccttattagttggagaggttactggcaagaattaatt
gatatcatcctctacatacatttgaaaacaccaattcttatcaatctttggaatggagac
atctatacacctctcgccttatctattgtacaagctagatttattgggcttgttcacttc
tcaaccggtctaattttaacttaccctccttttataataggtgctactagttag
>KrA10_psaB
ttttccattgcgaaattacggttagcctccaggccaaatccacatcaaaatcaccaatgc
acacaccaatgcaccttgactgatagctttatatctttaaatggtaggtgtgcgacttca
agatatttacaagtggtgggctctattcatgatatagaatcttactttggtatagataat
actctttctttaaatctacagatattcacagcacattggggtcacctaactataatctta
atatgggtatcaggcaatctttatcacatagcatccaatgctaactattctctctgggtt
aaaaatccaattccaagcatacctatagcacataatatttgggatccacattttactaac
tctacttccactccatattcccataccatcatcaccacaatcctcattgcttactcaggt
atttataaccaactatacacttctggatttaacactattaatcagatatacaagaccaca
tttacatcctcttgcctggcagtaatatcaatactattagctaagatacatatcaatact
cattcagaactattacacaagcttgcaactcatgcaagtcaaatcccatcattcttccag
cttctctactttctggatgtcggtatctcatctgttaatattcgattcaattttcacact
ggaattctagttggacttttctcaatagggtacacaggacatcttctagacatcacaata
ccagcctctcgagctccactaattcatacctcactctcttacttaacattctttggtggt
ttaaaatcgaatacaacttcattgtaccttacagatattgcacatcatcacttagcaatt
ggtatcacattcatccatacagggcatctatactcctctttcagggcagcccttggaaca
tatatacgagatacattatatacatc-caccttacacacagcataaaatcactacactta
gccctctcactcattctagccagttgtacagcacttacatctaccaccactcagcacatc
tattctttaacaccatacttttatttgtcttatgatcacatctattttacagctctatat
gtccatcactcctacatcacatcattcttaaccattggttctcatgcacatacagctatc
accttagtaagagattggatcacaccattagaactagaatccagctctaagcggataaga

atacatacacataaagctgccattattccacacttatcctgggtcagtctttggcttggt
tttcacacactagcagtatactctcataatgacacatgtatagcatttaactctccatct
aagcaaatcttaatagaagcaagcaatgctcaacttattcaacaagcatcaggaaaagct
ttatatgggaccatcaactccatcaacaactataataaatcatttgattcatttatacac
cctatcagtccaggtgattcatatgtacatcatgcaatagcacttggcttacatataact
gtcctcatcctactaaagggaggtcttgaagctcgtgggtctaagctaatgccagacaag
atggagcactcatttggcttttcatgtgatggtccaggtagaggtggcacatgtgatata
tcagcttgggattccttttatctagcaacattctggatgcttaatagcaacacatggata
agcttctacttccactacaagcaccttacacctcgtcaattctcagaaagttcaacttat
ctagaatcatggtttcgagattatctgtggtttaactctacacctttaattcatggttat
tctacactaggaactaatgatctatccgtccaatcctggtccttcctacttactcacctt
gcatgggcaagtggatttatgttccttattagttggagaggttactggcaagaattaatt
gatatcatcctctacatacatttgaaaacaccaattcttatcaatctttggaatggagac
atctatacacctctagccttatctattgtacaagctagatttattgggcttgttcacttc
tcaaccggtctaattttaacttaccctccttttataataggtgctacaagttag
>KrA11_psaB
ttttccattgcgaaattacggttagcctccaggccaaatccacatcaaaatcaccaatgc
acacaccaatgcaccttgactgatagctttatatctttaaatggtaggtgtgctacttca
agatatttacaagtggtgggctctattcatgatatagaatcttactttggtatagataat
actctttctttaaatctacagatattcacagcacattggggtcacctaactataatctta
atatgggtatcaggcaatctttatcacatagcatccaatgctaactattctctctgggtt
aaaaatccaattccaagcatacctatagcacataatatttgggatccacattttactaac
tctacttccactccatattcccataccatcatcaccacaatcctcattgcttactcaggt
atttataaccaactatacacttctggatttaacactattaatcagatatacaagaccaca
tttacatcctcttgcctggcagtaatatcaatactattagctaagatacatatcaatact
cattcagaactattacacaagcttgcaactcatgcaagtcaaatcccatcattcttccag
cttctctactttctggatgtcggtatctgatctgttaatattcgattcaattttcacact
ggaattctagttggacttttctcaatagcacacacaggacatcttctagacatcacaata
ccagcctctcgagctccactaattcatacctcactctcttacttaacattctttggtggt
ttaaaatcgaatacaacttcattgtaccttacagatattgcacatcatcacttagcaatt
ggtatcacattcatccatacagggcatctatactcctctttcagggcagcccttggaaca
tatataagagatatattatatacatcacaccttacacacagcataaaatcactccactta
gccctctcactcattctagccagttgtacagcacttacatctaccaccactcagcacatc
tattctttaacaccatacttttatttgtcttatgatcacatctattttacagctctatat
gtccatcactcctacatcacatcattcttaaccattggttctcatgcacatacagctatc
accttagtaagagattggatcacaccattagaactagaatccagctctaagcggataaga
atacatacacataaagctgccattattccacacttatcctgggtcagtctttggcttggt
tttcacacactagcagtatactctcataatgacacatgtatagcatttaactctccatct
aagcaaatcttaatagaagcaagcaatgctcaacttattcaacaagcatcaggaaaagct
ttatatgggaccatcaactccatcaacaactataataaatcatttgattcatttatacac
cctatcagtccaggtgattcatatgtacatcatgcaatagcacttggcttacatataact
gtcctcatcctactaaagggaggtcttgaagctcgtgggtctaagctaatgccagacaag
atggagcactcatttggcttttcatgtgatggtccaggtagaggtggcacatgtgatata
tcagcttgggattccttttatctagcaacattctggatgcttaatagcaacacatggata
agcttctacttccactacaagcaccttacacctcgtcaattctcagaaagttcaacttat
ctagaatcatggtttcgagattatctgtggtttaactctacacctttaattcatggttat
tctacactaggaactaatgatctatccgtccaatcctggtccttcctacttactcacctt
gcatgggcaagtggatttatgttccttattagttggagaggttactggcaagaattaatt
gatatcatcctttacatacatttgaaaacaccaattcttatcaatctttggaatggagac
atctatacacctctcgccttatctattgtacaagctagatttattgggcttgttcacttc
tcaaccggtctaattttaacttaccctccttttataataggtgctacaagttag
>KrA12_psaB
ttttccattgcgaaattacggttagcctccaggccaaatccacatcaaaatcaccaatgc
acacaccaatgcaccttgactgatagctttatatgcttaaatggtagatgtgctagttca
agatatttacaagttctgggttctattcatgatatagaatgtggctttggtatagataat
actctttctttaaatctacagatattcacagcacattggggccacctaactataatctta
atatgggtatcaggcaatctttaccacatagcatccaatgctaactattctctctgggtt

aaaaatccaattccaagtatgcctatagcacacaatatttgggatccacatttcactaac
tctacttccactccatattcccataccattatcaccacaatcctcattgcttactcaggt
atttataaccaattatacacttctggatttaacactattaatcagatatacaagaccaca
tttacatcctcttgcctggcagtaatatcaatactattagctaagatacatatcaatact
cattcagaactattacacaagcttgcaactcatgcaagtcaaatcccatcattcttccag
cttctctactttctggatgtcggtatctcatctgttaatattcgattcaattttcacact
ggaattctagttggacttttctcaatagcacacacaggacatcttctagacatcacaata
ccagcctctcgagctccactaattcacacctcaccctcttacttaacattctttggtggt
ttaaaatcgaatacaagttcattgtaccttacagatattgcacatcatcacttagcaatt
ggtatcacattcatccatacagggcatctatactcctctttcagggcagcccttggaaca
tatatacgagatacattatatacatcacaccttacacacagcataaaatcactccactta
gccctctcacttattctagccagttgtacaccacttacatcaaccactgctcagcacatc
tattctttaacaccatatttttatttgtcttatgatcacatctattctacggctctctat
gtccatcactcctacatcacctcattcttagctattgcttctcatgcacatactgctatc
accttagtaagagattgggtcgcaccattagaacaagaatccagctctaagcagatacga
atccatacccataaagctgccattatttcacacttatcctgggtcagtctttggcttggt
tttcatacactagcagtatactctcataatgacacatgtatagcatttaactctccatct
aagcaaatcttaatagaagcaagcaatgctcaacttattcaacaagcatcaggaaaagct
ttatatgggaccatcaactccatcaacaactataataaatcatttgattcatttatacac
cctatcagtccaggtgattcatatgtacatcatgcaatagcacttggcttacatataact
gtcctcatcctactaaagggaggtcttgaagctcgtggttctaagctaatgccagacaag
atggagcactcatttggcttttcatgtgatgggccaggtagaggtggaacatgtgatata
tcagcttgggattccttttatctagcaacattctggatgcttaatagcaacgcatggata
agcttctacttccactacaagcaccttacacctcgtcaattctcagaaagttcaacttat
ctagaatcatggtttcgagattatctgtggtttaactctacacctttaattcatggttat
tctacactaggaactaatgatctatccgtccaatcctggtccttcctacttactcatctt
gcttgggcaagtggatttatgttccttattagttggagaggttactggcaagaattaatt
gatatcatcctctacatacatttgaaaacaccaattcttattaatctttggaatggagat
atctatacacctctagccttatctattgtacaagctagatttattgggcttgttcacttc
tcaaccggtctaattttaacttaccctccttttataataggtgctacaagttag
>KrA13_psaB
ttttccattgcgaaattacggttagcctccaggccaaatccacatcaaaatcaccaatgc
acacaccaatgcaccttgactgatagctttatatgcttaaatggtaggtgtgcgacttca
agatatttacaagtggtgggctctattcatgatatagaatcttactttggtatagataat
actctttctttaaatctacaaatattcacagcacattggggtcacctaactataatctta
atatgggtatcaggcaatctttatcacatagcatccaatgctaactattctctctgggtt
aaaaatccaattccaagcatacctatagcacataatatttgggatccacatttcactaac
tctacttccactccatattcccataccattatcaccacaatcctcattgcttactcaggt
atttataaccaattatacacttctggatttaacactattaatcagatatacaagaccaca
tttacatcctcttgcctggcagtaatatcaatactattagctaagatacatatcaatact
cattcagaactattacacaagcttgcaagtcatacaagtcaaatcccatcattcttccag
cttctctactttctggatgtggccatctcatctgttaatattagattcaatttccacact
ggaattctagttggacttttctcaatagcacacacaggacatcttctagacatcacaata
ccagcctctcgagctccactaattcatacctcactctcttacttaacattctttggtggt
ttaaaatcgaatacaacttcattgtaccttacagatattgcacatcatcacttagcaatt
ggtatcacattcatccatacagggcatctatactcctctttcagggcagcccttggaaca
tatatacgagatacattatatacatcacaccttacacacagcataaaatcactacactta
gccctctcactcattctagccagttgtacaccacttacatctaccaccactcagcacatc
tattctttaacaccatacttttatttgtcttatgatcacatctattttacagctctatat
gtccatcactcctacatcacatcattcttaaccattggttctcatgcacatacagctatc
accttagtaagagattggatcacaccattagaactagaatccagctctaagcggatacga
atacatacacataaagctgccattattccacacttatcctgggtcagtctttggcttggt
tttcacacactagcagtatactctcataatgacacatgtatagcatttaactctccatct
aagcaaatcttaatagaagcaagcaatgctcaacttattcaacaagcatcaggaaaagct
ttatatgggaccatcaactccatcaacaactataataaatcatttgattcatttatacac
cctatcagtccaggtgatttatatgtacatcatgcaatagcccttggtttacatataact
gtcctcatcctactaaagggaggtcttgaagctcgtggttctaagctaatgccagacaag

atggagcactcatttggcttttcatgtgatgggccaggtagaggtggaacatgtgatata
tcagcttgggattccttttatctagcaacattctggatgcttaatagcaacacatggata
agcttctacttccactacaagcaccttacacctcgtcaattctcagaaagttcaacttat
ctagaatcatggtttcgagattatctgtggtttaactctacacctttaattcatggttat
tctacactaggaactaatgatctatccgtccaatcctggtccttcctacttactcacctt
gcatgggcaagtggatttatgttccttattagttggagaggttactggcaagaattaatt
gatatcatcctctacatacatttgaaaacaccaattcttatcaatctttggaatggagac
atctatacacctctcgccttatctattgtacaagctagatttattgggcttgttcacttc
tcaactgggttaattttaacttaccctccttttataataggtgctacaagttag
>KrA14_psaB
ttttccattgcgaaattacggttagcctccaggccaaatccacatcaaaatcaccaatgc
acacaccaatgcaccttgactgatagctttatatctttaaatggtaggtgtgcgacttca
agatatttacaagtggtgggctctattcatgatatagaatcttactttggtatagataat
actctttctttaaatctacagatattcacagcacattggggccacctaactataatctta
atatgggtatcaagcaatctttaccacatagcatccaatgctaactattctctctgggtt
aaaaatccaattccaagcatacctatagcacataatatttgggatccacattttactaac
tctacttccactccatattcccataccatcatcaccacaatcctcattgcttactcaggt
atttataaccaactatacacttctggatttaacactattaatcagatatacaagaccaca
tttacatcctcttgcctggcagtaatatcaatactattagctaagatacatatcaatact
cattcagaactattacacaagcttgcaactcatgcaagtcaaatcccatcattcttccag
cttctctactttctggatgtcggtatctcatctgttaatattcgattcaattttcacact
ggaattctagttggacttttctcaatagcacacacaggacatcttctagacatcacaata
ccagcctctcgagctccactaattcatacctcactctcttacttaacattctttggtggt
ttaaaatcgaatacaacttcattgtaccttacagatattgcacatcatcacttagcaatt
ggtatcatatctatccttacaggccatctatactcctctttcagggcagcccttggaaca
tatataagagatatattatatacatcacaccttacacacagcataaaatcactccactta
gccctctcacttattctagccagttgtacaccacttacatcaaccactgctcagcacatc
tattctttaacaccatacttttatttgtcttatgatcacatctattttacagctctatat
gtccatcactcctacatcacatcattcttaaccattggttctcatgcacatacagctatc
accttagtaagagattggatcacaccattagaactagaatccagctctaagcggataaga
atacatacacataaagctgccattattccacacttatcctgggtcagtctttggcttggt
tttcacacactagcagtatactctcataatgacacatgtatagcatttaactctccatct
aagcaaatcttaatagaagcaagcaatgctcaacttattcaacaagcatcaggaaaagct
ttatatgggaccatcaactccatcaacaactataataaatcatttgattcatttatacac
cctatcagtccaggtgattcatatgtacatcatgcaatagcacttggcttacatataact
gtcctcatcctactaaagggaggtcttgaagctcgtgggtctaagctaatgccagacaag
atggagcactcatttggcttttcatgtgatggtccaggtagaggtggcacatgtgatata
tcagcttgggattccttttatctagcaacattctggatgcttaatagcaacacatggata
agcttctacttccactacaagcaccttacacctcgtcaattctcagaaagttcaacttat
ctagaatcatggtttcgagattatctgtggtttaactctacacctttaattcatggttat
tctacactaggaactaatgatctatccgtccaatcctggtccttcctacttactcacctt
gcatgggcaagtggatttatgttccttattagttggagaggttactggcaagaattaatt
gatatcatcctctacatacatttgaaaacaccaattcttattaatctttggaatggagat
atctatacacctctagccttatctattgtacaagctagatttattgggcttgttcacttc
tcaactgggttaattctaacttaccctccttttataataggtgctacaagttag
>KrA15_psaB
ttttccattgcgaaattacggttagcctccaggccaaatccacatcaaaatcaccaatgc
acacaccaatgcaccttgactgatagctttatatctttaaatggtaggtgtgcgacttca
agatatttacaagtggtgggctctattcatgatatagaatcttactttggtatagataat
actctttctttaaatctacagatattcacagcacattggggtcacctaactataatctta
atatgggtatcaggcaatctttatcacatagcatccaatgctaactattctctctgggtt
aaaaatccaattccaagcatacctatagcacataatatttgggatccacattttactaac
tctacttccactccatattcccataccattatcaccacaatcctcattgcttactcaggt
atttataaccaattatacacttctggatttaacactattaatcagatatacaagaccaca
tttacatcctcttgcctggcagtaatatcaatactattagctaagatacatatcaatact
cattcagaactattacacaagcttgcaactcatgcaagtcaaatcccatcattcttccag
cttctctactttctggatgtcggtatctcatctgttaatattcgattcaatttccacact

ggaattctagttggacttttctcaatagcacacacaggacatcttctagacatcacaata
ccagcctctcgagctccactaattcatacctcactctcttacttaacattctttggtggt
ttaaaatcgaatacaacttcattgtaccttacagatattgcacatcatcacttagcaatt
ggtatcacattcatccatacagggcatctatactcctctttcagggcagcccttggaaca
tatatacgagatacattatatacatcacaccttacacacagcataaaatcactacactta
gccctctcactcattctagccagttgtacagcacttacatctaccaccactcagcacatc
tattctttaacaccatacttttatttgtcttatgatcacatctattttacagctctatat
gtccatcactcctacatcacatcattcttaaccattggttctcatgcacatacagctatc
accttagtaagagattggatcacaccattagaactagaatccagctctaagcggataaga
atacatacacataaagctgccattattccacacttatcctgggtcagtctttggcttggt
tttcatacactagcagtatactctcataatgacacatgtatagcatttaactctccatct
aagcaaatcttaatagaagcaagcaatgctcaacttattcaacaagcatcaggaaaagct
ttatatgggaccatcaactccatcaacaactataataaatcatttgattcatttatacac
cctatcagtccaggtgatgcatatgtacatcatgcaatagcacttggcttacatataact
gtcctcatcctactaaagggaggtcttgaagctcgtgggtctaagctaatgccagacaag
atggagcactcatttggcttttcatgtgatggtccaggtagaggtggaacatgtgatata
tcagcttgggattccttttatctagcaacattctggatgcttaatagcaacacatggata
agcttctacttccactacaagcaccttacacctcgtcaattctcagaaagttcaacttat
ctagaatcatggtttcgagattatctgtggtttaactctacacctttaattcatggttat
tctacactaggagctaatgatctatccgtccaatcttggtccttcctacttactcatctt
gcttgggcaagtggatttatgttccttattagttggagaggttactggcaagaattaatt
gatatcatcctttacatacatttgaaaacaccaattcttattaatctttggaatggagac
atctatacacctctcgccttatctattgtacaagctagatttattgggcttgttcacttc
tcaaccggtctaattttaacttaccctccttttataataggtgctacaagttag
>KrA2_psaB
ttttccattgcgaaattacggttagcctccaggccaaatccacatcaaaatcaccaatgc
acacaccaatgcaccttgactgatagctttatatctttaaatggtaggtgtgcgacttca
agatatttacaagtggtgggctctattcatgatatagaatcttactttggtatagataat
actctttctttaaatctacagatattcacagcacattggggtcacctaactataatctta
atatgggtatcaggcaatctttatcacatagcatccaatgctaactattctctctgggtt
aaaaatccaattccaagcatacctatagcacataatatttgggatccacattttactaac
tctacttccactccatattcccataccatcatcaccacaatcctcattgcttactcaggt
atttataaccaactatacacttctggatttaacactattaatcagatatacaagaccaca
tttacatcctcttgcctggcagtaatatcaatactattagctaagatacatatcaatact
cattcagaactattacacaagcttgcaactcatgcaagtcaaatcccatcattcttccag
cttctctactttctggatgtcggtatctcatctgttaatattcgattcaattttcacact
ggaattctagttggacttttctcaatagcacacacaggacatcttctagacatcacaata
ccagcctctcgagctccactaattcatacctcactctcttacttaacattctttggtggt
ttaaaatcgaatacaacttcattgtaccttacagatattgcacatcatcacttagcaatt
ggtatcacattcatccatacagggcatctatactcctctttcagggcagcccttggaaca
tatatacgagatacattatatacatcacaccttacacacagcataaaatcactacactta
gccctctcactcattctagccagttgtacagcacttacatctaccaccactcagcacatc
tattctttaacaccatacttttatttgtcttatgatcacatctattttacagctctatat
gtccatcactcctacatcacatcattcttaaccattggttctcatgcacatacagctatc
accttagtaagagattggatcacaccattagaactagaatccagctctaagcggataaga
atacatacacataaagctgccattattccacacttatcctgggtcagtctttggcttggt
tttcacacactagcagtatactctcataatgacacatgtatagcatttaactctccatct
aagcaaatcttaatagaagcaagcaatgctcaacttattcaacaagcatcaggaaaagct
ttatatgggaccatcaactccatcaacaactataataaatcatttgattcatttatacac
cctatcagtccaggtgattcatatgtacatcatgcaatagcacttggcttacatataact
gtcctcatcctactaaagggaggtcttgaagctcgtgggtctaagctaatgccagacaag
atggagcactcatttggcttttcatgtgatggtccaggtagaggtggcacatgtgatata
tcagcttgggattccttttatctagcaacattctggatgcttaatagcaacacatggata
agcttctacttccactacaagcaccttacacctcgtcaattctcagaaagttcaacttat
ctagaatcatggtttcgagattatctgtggtttaactctacacctttaattcatggttat
tctacactaggaactaatgatctatccgtccaatcctggtccttcctacttactcacctt
gcatgggcaagtggatttatgttccttattagttggagaggttactggcaagaattaatt

gatatcatcctctacatacatttgaaaacaccaattcttatcaatctttggaatggagac
atctatacacctctcgccttatctattgtacaagctagatttattgggcttgttcacttc
tcaaccggtctaattttaacttaccctccttttataataggtgctactagttag
>KrA3_psaB
ttttccattgcgaaattacggttagcctccaggccaaatccacatcaaaatcaccaatgc
acacaccaatgcaccttgactgatagctttatatgcttaaatggtaggtgtgcgacttca
agatatttacaagtggtgggctctattcatgatatagaatcttactttggtatagataat
actctttctttaaatctacagatattcacagcacattggggtcacctaactataatttta
atatgggtatcaggcaatctttatcacatagcatccaatgctaactattctctctgggtt
aaaaatccaattccaagcatacctatagcacataatatttgggatccacattttactaac
tctacttccactccatattcccataccatcatcaccacaatcctcattgcttactcaggt
atttataaccaactatacacttctggatttaacactattaatcagatatacaagaccaca
tttacatcctcttgcctggcagtaatatcaatactattagccaagatacatatcaatact
cattcagaactattacacaagcttgcaactcatgcaagtcaaatcccatcattcttccag
cttctctactttctggatgtcggtatctcatctgttaatattcgattcaattttcacact
ggaattctagttggacttttctcaatagcacacacaggacatcttctagacatcacaata
ccagcctctcgagctccactaattcatacctcactctcttacttaacattctttggtggt
ttaaaatcgaatacaacttcattgtaccttacagatattgcacatcatcacttagcaatt
ggtatcacattcatccatacagggcatctatactcctctttcagggcagcccttggaaca
tatatacgagatacattatatacatcacaccttacacacagcataaaatcactacactta
gccctctcactcattctagccagttgtacaccacttacatcaaccactgctcagcacatc
tattctttaacaccatatttttatttgtcttatgatcacatctattttacagctctatat
gtccatcactcctacatcacatcattcttaaccattggttctcatgcacatacagctatc
accttagtaagagattgggtcgcaccattagaacaagaatccagctctaagcggataaga
atacatacacataaagctgccattattccacacttatcctgggtcagtctttggcttggt
tttcacacactagcagtatactctcataatgacacatgtatagcatttaactctccatct
aagcaaatcttaatagaagcaagcaatgctcaacttattcaacaagcatcaggaaaagct
ttatatgggaccatcaactccatcaacaactataataaatcatttgattcatttatacac
cctatcagtccaggtgattcatatgtacatcatgcaatagcacttggcttacatataact
gtcctcatcctactaaagggaggtcttgaagctcgtgggtctaagctaatgccagacaag
atggagcactcatttggcttttcatgtgatggtccaggtagaggtggcacatgtgatata
tcagcttgggattccttttatctagcaacattctggatgcttaatagcaacacatggata
agcttctacttccactacaagcaccttacacctcgtcaattctcagaaagttcaacttat
ctagaatcatggtttcgagattatctgtggtttaactctacacctttaattcatggttat
tctacactaggagctaatgatctatccgtccaatcctggtccttcctacttactcacctt
gcatgggcaagtggatttatgttccttattagttggagaggttactggcaagaattaatt
gatatcatcctctacatacatttgaaaacaccaattcttatcaatctttggaatggagac
atctatacacctctcgccttatctattgtacaagctagatttattgggcttgttcacttc
tcaaccggtctaattttaacttaccctccttttataataggtgctacaagttag
>KrA4_psaB
ttttccattgcgaaattacggttagcctccaggccaaatccacatcaaaatcaccaatgc
acacaccaatgcaccttgactgatagctttatatgcttaaatggtagatgtgctagttca
agatatttacaagtggtgggctctattcatgatatagaatcttactttggtatagataat
actctttctttaaatctacagatattcacagcacattggggtcacctaactataatctta
atatgggtatcaggcaatctttatcacatagcatccaatgctaactattctctctgggtt
aaaaatccaattccaagcatacctatagcacataatatttgggatccacatttcactaac
tctacttccactccatattcccataccatcatcaccacaatcctcattgcttactcaggt
atttataaccaactatacacttctggatttaacactattaatcagatatacaagaccaca
tttacattctcttgccttgcagtaatttcaatactattagccaagatacacatcaacact
cattcagaactattacataagcttgcaagtcatacaagtcaaatcccatcattcttccag
cttctctactttctggatgtggccatctcatctgttaatattagattcaattttcacact
ggaattctagttggacttttctcaatagcacacacaggacatcttctagacatcacaata
ccagcctctcgagctccactaattcatacctcactctcttacttaacattctttggtggt
ttaaaatcgaatacaacttcattgtaccttacagatattgcacatcatcacttagcaatt
ggtatcacattcatccatacagggcatctatactcctctttcagggcagcccttggaaca
tatatacgagatacattatatacatcacaccttacacacagcataaaatcactacactta
gccctctcactcattctagccagttgtacagcacttacatctaccaccactcagcacatc

tattctttaacaccatacttttatttgtcttatgatcacatctattttacagctctatat
gtccatcactcctacatcacatcattcttaaccattggttctcatgcacatacagctatc
accttagtaagagattggatcacaccattagaactagaatccagctctaagcggataaga
atacatacacataaagctgccattattccacacttatcctgggtcagtctttggcttggt
tttcatacactagcagtatactctcataatgacacatgtatagcatttaactctccatct
aagcaaatcctaatagaagcaagcaatggtcaacttattcaacaagcatcaggaaaagct
ttatatgggaccatcaactccatcaacaactataataaatcatttgattcatttatacac
cctatcagtccaggtgatttatatgtacatcatgcaatagcacttggcttacatataact
gtcctcatcctactaaagggaggtcttgaagctcgtggttctaagctaatgccagacaag
atggagcactcatttggcttttcatgtgatgggccaggtagaggtggaacatgtgatata
tcagcttgggattccttttatctagcaacattctggatgcttaatagcaacacatggata
agcttctacttccactacaagcaccttacacctcgtcaattctcagaaagttcaacttat
ctagaatcatggtttcgagattatctgtggtttaactctacacctttaattcatggttat
tctacactaggaactaatgatctatccgtccaatcttggtccttcctacttactcatctt
gcttgggcaagtggatttatgttccttattagttggagaggttactggcaagaattaatt
gatatcatcctctacatacatttgaaaacaccaattcttatcaatctttggaatggagac
atctatacacctctagccttatctattgtacaagctagatttattgggcttgttcacttc
tcaactgggttaattctaacttaccctccttttataataggtgctacaagttag
>KrA5_psaB
ttttccattgcgaaattacggttagcctccaggccaaatccacatcaaaatcaccaatgc
acacaccaatgcaccttgactgatagctttatatctttaaatggtaggtgtgcgacttca
agatatttacaagtggtgggctctattcatgatatagaatcttactttggtatagataat
actctttctttaaatctacagatattcacagcacattggggtcacctaactataatctta
atatgggtatcaggcaatctttatcacatagcatccaatgctaactattctctctgggtt
aaaaatccaattccaagcatacctatagcacataatatttgggatccacattttactaac
tctacttccactccatattcccataccatcatcaccacaatcctcattgcttactcaggt
atttataaccaactatacacttctggatttaacactattaatcagatatacaagaccaca
tttacatcctcttgcctggcagtaatatcaatactattagctaagatacatatcaatact
cattcagaactattacacaagcttgcaactcatgcaagtcaaatcccatcattcttccag
cttctctactttctggatgtcggtatctcatctgttaatattcgattcaattttcacact
ggaattctagttggacttttctcaatagcacacacaggacatcttctagacatcacaata
ccagcctctcgagctccactaattcatacctcactctcttacttaacattctttggtggt
ttaaaatcgaatacaacttcattgtaccttacagatattgcacatcatcacttagcaatt
ggtatcacattcatccatacagggcatctatactcctctttcagggcagcccttggaaca
tatatacgagatacattatatacatcacaccttacacacagcataaaatcactacactta
gccctctcactcattctagccagttgtacagcacttacatctaccaccactcagcacatc
tattctttaacaccatacttttatttgtcttatgatcacatctattttacagctctatat
gtccatcactcctacatcacatcattcttaaccattggttctcatgcacatactgctatc
accttagtaagagattggatcacaccattagaactagaatccagctctaagcggataaga
atacatacacataaagctgccattattccacacttatcctgggtcagtctttggcttggt
tttcacacactagcagtatactctcataatgacacatgtatagcatttaactctccatct
aagcaaatcttaatagaagcaagcaatgctcaacttattcaacaagcatcaggaaaagct
ttatatgggaccatcaactccatcaacaactataataaatcatttgattcatttatacac
cctatcagtccaggtgattcatatgtacatcatgcaatagcacttggcttacatataact
gtcctcatcctactaaagggaggtcttgaagctcgtgggtctaagctaatgccagacaag
atggagcactcatttggcttttcatgtgatggtccaggtagaggtggcacatgtgatata
tcagcttgggattccttttatctagcaacattctggatgcttaatagcaacacatggata
agcttctacttccactacaagcaccttacacctcgtcaattctcagaaagttcaacttat
ctagaatcatggtttcgagattatctgtggtttaactctacacctttaattcatggttat
tctacactaggaactaatgatctatccgtccaatcctggtccttcctacttactcacctt
gcatgggcaagtggatttatgttccttattagttggagaggttactggcaagaattaatt
gatatcatcctctacatacatttgaaaacaccaattcttatcaatctttggaatggagac
atctatacacctctcgccttatctattgtacaagctagatttattgggcttgttcacttc
tcaaccggtctaattttaacttaccctccttttataataggtgctactagttag
>KrA6_psaB
ttttccattgcgaaattacggttagcctccaggccaaatccacatcaaaatcaccaatgc
acacaccaatgcaccttgactgatagctttatatctttaaatggtaggtgtgcgacttca

agatatttacaagtggtgggctctattcatgatatagaatcttactttggtatagataat
actctttctttaaatctacagatattcacagcacattggggtcacctaactataatctta
atttgggtatcaggcaatctttatcacatagcatccaatgctaactattctctctgggtt
aaaaatccaattccaagcatacctatagcacataatatttgggatccacattttactaac
tctacttccactccatattcccataccatcatcaccacaatcctcattgcttactcaggt
atttataaccaactatacacttctggatttaacactattaatcagatatacaagaccaca
tttacatcctcttgcctggcagtaatatcaatactattagctaagatacatatcaatact
cattcagaactattacacaagcttgcaactcatgcaagtcaaatcccatcattcttccag
cttctctactttctggatgtcggtatctcatctgttaatattcgattcaattttcacact
ggaattctagttggacttttctcaatagcacacacaggacatcttctagacatcacaata
ccagcctctcgagctccactaattcatacctcactctcttacttaacattctttggtggt
ttaaaatcgaatacaacttcattgtaccttacagatattgcacatcatcacttagcaatt
ggtatcacattcatccatacagggcatctatactcctctttcagggcagcccttggaaca
tatatacgagatacattatatacatcacaccttacacacagcataaaatcactacactta
gccctctcactcattctagccagttgtacagcacttacatctaccaccactcagcacatc
tattctttaacaccatacttttatttgtcttatgatcacatctattttacagctctatat
gtccatcactcctacatcacatcattcttaaccattggttctcatgcacatacagctatc
accttagtaagagattggatcacaccattagaactagaatccagctctaagcggataaga
atacatacacataaagctgccattattccacacttatcctgggtcagtctttggcttggt
tttcacacactagcagtatactctcataatgacacatgtatagcatttaactctccatct
aagcaaatcttaatagaagcaagcaatgctcaacttattcaacaagcatcaggaaaagct
ttatatgggaccatcaactccatcaacaactataataaatcatttgattcatttatacac
cctatcagtccaggtgattcatatgtacatcatgcaatagcacttggcttacatataact
gtcctcatcctactaaagggaggtcttgaagctcgtggttctaagctaatgccagacaag
atggagcactcatttggcttttcatgtgatggtccaggtagaggtggcacatgtgatata
tcagcttgggattccttttatctagcaacattctggatgcttaatagcaacacatggata
agcttctacttccactacaagcaccttacacctcgtcaattctcagaaagttcaacttat
ctagaatcatggtttcgagattatctgtggtttaactctacacctttaattcatggttat
tctacactaggaactaatgatctatccgtccaatcctggtccttcctacttactcacctt
gcatgggcaagtggatttatgttccttattagttggagaggttactggcaagaattaatt
gatatcatcctctacatacatttgaaaacaccaattcttatcaatctttggaatggagac
atctatacacctctcgccttatctattgtacaagctagatttattgggcttgttcacttc
tcaaccggtctaattttaacttaccctccttttataataggtgctactagttag
>KrA7_psaB
ttttccattgcgaaattacggttagcctccaggccaaatccacatcaaaatcaccaatgc
acacaccaatgcaccttgactgatagctttatatgcttaaatggtagatgtgctagttca
agatatttacaagttctgggttctattcatgatatagaatgtggctttggtatagataat
actctatctttaaatctacaaatattcacagcacattggggccacctaactataatctta
atatgggtatcaagcaatctttaccacatagcatccaatgctaactattctctctgggtt
aaaaatccaattccaagcatacctatagcacataatatttgggatccacattttactaac
tctacttccactccatattcccataccattatcaccacaatcctcattgcttactcaggt
atttataaccaattatacacttctggatttaacactattaatcagatatacaagaccaca
tttacattctcttgccttgcagtaatttcaatactattagctaagatacatatcaatact
cattcagaactattacacaagcttgcaactcatgcaagtcaaatcccatcattcttccag
cttctctactttctggatgtggccatctcatctgttaatattagattcaatttccacact
ggaattctagttggacttttctcaatagggtacacaggacatcttctagacatcacaata
ccagcctctcgagctccactaattcatacctcactctcttacttaacattctttggtggt
ttaaaatcgaatacaacttcattgtaccttacagatattgcacatcatcacttagcaatt
ggtatcacattcatccatacagggcatctatactcctctttcagggcagcccttggaaca
tatatacgagatacattatatacatcacaccttacacacagcataaaatcactacactta
gccctctcactcattctagccagttgtacaccacttacatcaaccactgctcagcacatc
tattctttaacaccatatttttatttgtcttatgatcacatctattttacagctctatat
gtccatcactcctacatcacatcattcttaaccattggttctcatgcacatacagctatc
accttagtaagagattggatcacaccattagaactagaatccagctctaagcggataaga
atacatacacataaagctgccattattccacacttatcctgggtcagtctttggcttggt
tttcatacactagcagtatactctcataatgacacatgtatagcatttaactctccatct
aagcaaatcttaatagaagcaagcaatgctcaacttattcaacaagcatcaggaaaagct

ttatatgggaccatcaactccatcaacaactataataaatcatttgattcatttatacac
cctatcagtccaggtgattcatatgtacatcatgcaatagcacttggcttacatataact
gtcctcatcctactaaagggaggtcttgaagctcgtggttctaagctaatgccagacaag
atggagcactcatttggcttttcatgtgatgggccaggtagaggtggaacatgtgatata
tcagcttgggattccttttatctagcaacattctggatgcttaatagcaacacatggata
agcttctacttccactacaagcaccttacacctcgtcaattctcagaaagttcaacttat
ctagaatcatggtttcgagattatctgtggtttaactctacacctttaattcatggttat
tctacactaggaactaatgatctatccgtccaatcctggtccttcctacttactcacctt
gcatgggcaagtggatttatgttccttattagttggagaggttactggcaagaattaatt
gatatcatcctctacatacatttgaaaacaccaattcttatcaatctttggaatggagac
atctatacacctctcgccttatctattgtacaagctagatttattgggcttgttcacttc
tcaaccggtctaattttaacttaccctccttttataataggtgctactagttag
>KrA8_psaB
ttttccattgcgaaattacggttagcctccaggccaaatccacatcaaaatcaccaatgc
acacaccaatgcaccttgactgatagctttatatgcttaaatggtagatgtgctagttca
agatatttacaagttctgggctctattcatgatatagaatcttactttggtatagataat
actctttctttaaatctacagatattcacagcacattggggtcacctaactataatctta
atatgggtatcaggcaatctttatcacatagcatccaatgctaactattctctctgggtt
aaaaatccaattccaagcatacctatagcacataatatttgggatccacattttactaac
tctacttccactccatattcccataccatcatcaccacaatcctcattgcttactcaggt
atttataaccaactatacacttctggatttaacactattaatcagatatacaagaccaca
tttacattctcttgccttgcagtaatatcaatactattagctaagatacatatcaatact
cattcagaactattacacaagcttgcaactcatgcaagtcaaatcccatcattcttccag
cttctctactttctggatgtggccatctcatctgttaatattcgattcaattttcacact
ggaattctagttggacttttctcaatagcacacacaggacatcttctagacatcacaata
ccagcctctcgagctccactaattcatacctcactctcttacttaacattctttggtggt
ttaaaatcgaatacaacttcattgtaccttacagatattgcacatcatcacttagcaatt
ggtatcacattcatccatacagggcatctatactcctctttcagggcagcccttggaaca
tatatacgagatacattatatacatcacaccttacacacagcataaaatcactacactta
gccctctcactcattctagccagttgtacagcacttacatctaccaccactcagcacatc
tattctttaacaccatacttttatttgtcttatgatcacatctattttacagctctatat
gtccatcactcctacatcacatcattcttaaccattggttctcatgcacatacagctatc
accttagtaagagattggatcacaccattagaactagaatccagctctaagcggataaga
atacatacacataaagctgccattattccacacttatcctgggtcagtctttggcttggt
tttcatacactagcagtatactctcataatgacacatgtatagcatttaactctccatct
aagcaaatcttaatagaagcaagcaatgctcaacttattcaacaagcatcaggaaaagct
ttatatgggaccatcaactccatcaacaactataataaatcatttgattcatttatacac
cctatcagtccaggtgattcatatgtacatcatgcaatagcacttggcttacatataact
gtcctcatcctactaaagggaggtcttgaagctcgtgggtctaagctaatgccagacaag
atggagcactcatttggcttttcatgtgatggtccaggtagaggtggaacatgtgatata
tcagcttgggattccttttatctagcaacattctggatgcttaatagcaacacatggata
agcttctacttccactacaagcaccttacacctcgtcaattctcagaaagttcaacttat
ctagaatcatggtttcgagattatctgtggtttaactctacacctttaattcatggttat
tctacactaggaactaatgatctatccgtccaatcctggtccttcctacttactcacctt
gcatgggcaagtggatttatgttccttattagttggagaggttactggcaagaattaatt
gatatcatcctctacatacatttgaaaacaccaattcttatcaatctttggaatggagat
atctatacacctctagccttatctattgtacaagctagatttattgggcttgttcacttc
tcaactgggttaattctaacttaccctccttttataataggtgctacaagttag
>KrA9_psaB
ttttccattgcgaaattacggttagcctccaggccaaatccacatcaaaatcaccaatgc
acacaccaatgcaccttgactgatagctttatatgcttaaatggtaggtgtgcgacttca
agatatttacaagtgctgggctctattcatgatatagaatgtggctttggtatagataat
actctttctttaaatctacagatattcacagcacattggggtcacctaactataatctta
atatgggtatcaggcaatctttatcacatagcatccaatgctaactattctctctgggtt
aaaaatccaattccaagtatgcctatagcacataatatttgggatccacattttactaac
tctacttccactccatattcccataccatcatcaccacaatcctcattgcttactcaggt
atttataaccaactatacacttctggatttaacactattaatcagatatacaagaccaca

tttacatcctcttgcctggcagtaatatcaatactattagctaagatacatatcaatact
cattcagaactattacacaagcttgcaactcatgcaagtcaaatcccatcattcttccag
cttctctactttctggatgtcggtatctcatctgttaatattcgattcaattttcacact
ggaattctagttggacttttctcaatagcacacacaggacatcttctagacatcacaata
ccagcctctcgagctccactaattcatacctcactctcttacttaacattctttggtggt
ttaaaatcgaatacaacttcattgtaccttacagatattgcacatcatcacttagcaatt
ggtatcacattcatccatacagggcatctatactcctctttcagggcagcccttggaaca
tatatacgagatacattatatacatcacaccttacacacagcataaaatcactacactta
gccctctcactcattctagccagttgtacagcacttacatctaccaccactcagcacatc
tattctttaacaccatacttttatttgtcttatgatcacatctattttacagctctatat
gtccatcactcctacatcacatcattcttaaccattggttctcatgcacatacagctatc
accttagtaagagattggatcacaccattagaactagaatccagctctaagcggataaga
atacatacacataaagctgccattattccacacttatcctgggtcagtctttggcttggt
tttcacacactagcagtatactctcataatgacacatgtatagcatttaactctccatct
aagcaaatcttaatagaagcaagcaatgctcaacttattcaacaagcatcaggaaaagct
ttatatgggaccatcaactccatcaacaactataataaatcatttgattcatttatacac
cctatcagtccaggtgattcatatgtacatcatgcaatagcacttggcttacatataact
gtcctcatcctactaaagggaggtcttgaagctcgtgggtctaagctaatgccagacaag
atggagcactcatttggcttttcatgtgatggtccaggtagaggtggcacatgtgatata
tcagcttgggattccttttatctagcaacattctggatgcttaatagcaacacatggata
agcttctacttccactacaagcaccttacacctcgtcaattctcagaaagttcaacttat
ctagaatcatggtttcgagattatctgtggtttaattctacacctttaattcatggttat
tctacactaggaactaatgatctatccgtccaatcctggtccttcctacttactcacctt
gcatgggcaagtggatttatgttccttattagttggagaggttactggcaagaattaatt
gatatcatcctctacatacatttgaaaacaccaattcttatcaatctttggaatggagac
atctatacacctctcgccttatctattgtacaagctagatttattgggcttgttcacttc
tcaaccggtctaattttaacttaccctccttttataataggtgctacaagttag
>KrC1_psaB
ttttccattgcgaaattacggttagcctccaggccaaatccacatcaaaatcaccaatgc
acacaccaatgcaccttgactgatagctttatatgcttaaatggtaggtgtgcgacttca
agatatttacaagtggtgggctctattcatgatatagaatcttactttggtatagataat
actctttctttaaatctacagatattcacagcacattggggtcacctaactataatctta
atatgggtatcaggcaatctttatcacatagcatccaatgctaactattctctctgggtt
aaaaatccaattccaagcatacctatagcacataatatttgggatccacattttactaac
tctacttccactccatattcccataccatcatcaccacaatcctcattgcttactcaggt
atttataaccaactatacacttctggatttaacactattaatcagatatacaagaccaca
tttacatcctcttgcctggcagtaatatcaatactattagctaagatacatatcaatact
cattcagaactattacacaagcttgcaactcatgcaagtcaaatcccatcattcttccag
cttctctactttctggatgtggccatctcatctgttaatattagattcaatttccacact
ggaattctagttggacttttctcaatagcacacacaggacatcttctagacatcacaata
ccagcctctcgagctccactaattcatacctcactctcttacttaacattctttggtggt
ttaaaatcgaatacaacttcattgtaccttacagatattgcacatcatcacttagcaatt
ggtatcacattcatccatacagggcatctatactcctctttcagggcagcccttggaaca
tatataagagatatattatatacatcacaccttacacacagcataaaatcactacactta
gccctctcactcattctagccagttgtacagcacttacatctaccaccactcagcacatc
tattctttaacaccatacttttatttgtcttatgatcacatctattttacagctctatat
gtccatcactcctacatcacatcattcttaaccattggttctcatgcacatacagctatc
accttagtaagagattggatcacaccattagaactagaatccagctctaagcggataaga
atacatacacataaagctgccattattccacacttatcctgggtcagtctttggcttggt
tttcacacactagcagtatactctcataatgacacatgtatagcatttaactctccatct
aagcaaatcttaatagaagcaagcaatgctcaacttattcaacaagcatcaggaaaagct
ttatatgggaccatcaactccatcaacaactataataaatcatttgattcatttatacac
cctatcagtccaggtgattcatatgtacatcatgcaatagcacttggcttacatataact
gtcctcatcctactaaagggaggtcttgaagctcgtgggtctaagctaatgccagacaag
atggagcactcatttggcttttcatgtgatggtccaggtagaggtggcacatgtgatata
tcagcttgggattccttttatctagcaacattctggatgcttaatagcaacacatggata
agcttctacttccactacaagcaccttacacctcgtcaattctcagaaagttcaacttat

ctagaatcatggtttcgagattatctgtggtttaactctacacctttaattcatggttat
tctacactaggaactaatgatctatccgtccaatcctggtccttcctacttactcacctt
gcatgggcaagtggatttatgttccttattagttggagaggttactggcaagaattaatt
gatatcatcctctacatacatttgaaaacaccaattcttatcaatctttggaatggagac
atctatacacctctcgccttatctattgtacaagctagatttattgggcttgttcacttc
tcaaccggtctaattttaacttaccctccttttataataggtgctacaagttag
>KrC10_psaB
ttttccattgcgaaattacggttagcctccaggccaaatccacatcaaaatcaccaatgc
acacaccaatgcaccttgactgatagctttatatccttaaatggtaggtgtgcgacttca
agatatttacaagtggtgggctctattcatgatatagaatcttactttggtatagataat
actctttctttaaatctacagatattcacagcacattggggtcacctaactataatctta
atatgggtatcaggcaatctttatcacatagcatccaatgctaactattctctctgggtt
aaaaatccaattccaagcatacctatagcacataatatttgggatccacattttactaac
tctacttccactccatattcccataccatcatcaccacaatcctcattgcttactcaggt
atttataaccaactatacacttctggatttaacactattaatcagatatacaagaccaca
tttacatcctcttgcctggcagtaatatcaatactattagctaagatacatatcaacact
cattcagaactattacacaagcttgcaactcatgcaagtcaaatcccatcattcttccag
cttctctactttctggatgtcggcatctcatctgttaatattcgattcaattttcacact
ggaattctagttggacttttctcaatagcacacacaggacatcttctagacatcacaata
ccagcctctcgagctccactaattcatacctcactctcttacttaacattctttggtggt
ttaaaatcgaatacaacttcattgtaccttacagatattgcacatcatcacttagcaatt
ggtatcacattcatccatacagggcatctatactcctctttcagggcagcccttggaaca
tatatacgagatacattatatacatcacaccttacacacagcataaaatcactacactta
gccctctcactcattctagccagttgtacagcacttacatctaccaccactcagcacatc
tattctttaacaccatacttttatttgtcttatgatcacatctattttacagctctatat
gtccatcactcctacatcacatcattcttaaccattggttctcatgcacatacagctatc
accttagtaagagattggatcacaccattagaacaagaatccagctctaagcagatacga
atccatacccataaagctgccattatttcacacttatcctgggtcagtctttggcttggt
tttcatacactagcagtatactctcataatgacacatgtatagcatttaactctccatct
aagcaaatcttaatagaagcaagcaatgctcaacttattcaacaagcatcaggaaaagct
ttatatgggaccatcaactccatcaacaactataataaatcatttgattcatttatacac
cctatcagtccaggtgattcatatgtacatcatgcaatagcacttggcttacatataact
gtcctcatcctactaaagggaggtcttgaagctcgtgggtctaagctaatgccagacaag
atggagcactcatttggcttttcatgtgatgggccaggtagaggtggaacatgtgatata
tcagcttgggattccttttatctagcaacattctggatgcttaatagcaacacatggata
agcttctacttccactacaagcaccttacacctcgtcaattctcagaaagttcaacttat
ctagaatcatggtttcgagattatctgtggtttaactctacacctttaattcatggttat
tctacactaggaactaatgatctatccgtccaatcctggtccttcctacttactcacctt
gcatgggcaagtggatttatgttccttattagttggagaggttactggcaagaattaatt
gatatcatcctctacatacatttgaaaacaccaattcttatcaatctttggaatggagac
atctatacacctctcgccttatctattgtacaagctagatttattgggcttgttcacttc
tcaaccggtctaattttaacttaccctccttttataataggtgctacaagttag
>KrC11_psaB
ttttccattgcgaaattacggttagcctccaggccaaatccacatcaaaatcaccaatgc
acacaccaatgcaccttgactgatagctttatatctttaaatggtaggtgtgcgacttca
agatatttacaagtggtgggctctattcatgatatagaatcttactttggtatagataat
actctttctttaaatctacagatattcacagcacattggggtcacctaactataatctta
atatgggtatcaggcaatctttatcacatagcatccaatgctaactattctctctgggtt
aaaaatccaattccaagcatacctatagcacataatatttgggatccacattttactaac
tctacttccactccatattcccataccatcatcaccacaatcctcattgcttactcaggt
atttataaccaactatacacttctggatttaacactattaatcagatatacaagaccaca
tttacatcctcttgcctggcagtaatatcaatactattagctaagatacatatcaatact
cattcagaactattacacaagcttgcaactcatgcaagtcaaatcccatcattcttccag
cttctctactttctggatgtcggtatctcatctgttaatattcgattcaattttcacact
ggaattctagttggacttttctcaatagcacacacaggacatcttctagacatcacaata
ccagcctctcgagctccactaattcatacctcactctcttacttaacattctttggtggt
ttaaaatcgaatacaacttcattgtaccttacagatattgcacatcatcacttagcaatt

ggtatcacattcatccatacagggcatctatactcctctttcagggcagcccttggaaca
tatatacgagatacattatatacatc-caccttacacacagcataaaatcactacactta
gccctctcactcattctagccagttgtacagcacttacatctaccaccactcagcacatc
tattctttaacaccatacttttatttgtcttatgatcacatctattttacagctctatat
gtccatcactcctacatcacatcattcttaaccattggttctcatgcacatacagctatc
accttagtaagagattggatcacaccattagaactagaatccagctctaagcggataaga
atacatacacataaagctgccattattccacacttatcctgggtcagtctttggcttggt
tttcacacactagcagtatactctcataatgacacatgtatagcatttaactctccatct
aagcaaatcttaatagaagcaagcaatgctcaacttattcaacaagcatcaggaaaagct
ttatatgggaccatcaactccatcaacaactataataaatcatttgattcatttatacac
cctatcagtccaggtgattcatatgtacatcatgcaatagcacttggcttacatataact
gtcctcatcctactaaagggaggtcttgaagctcgtgggtctaagctaatgccagacaag
atggagcactcatttggcttttcatgtgatggtccaggtagaggtggcacatgtgatata
tcagcttgggattccttttatctagcaacattctggatgcttaatagcaacacatggata
agcttctacttccactacaagcaccttacacctcgtcaattctcagaaagttcaacttat
ctagaatcatggtttcgagattatctgtggtttaactctacacctttaattcatggttat
tctacactaggaactaatgatctatccgtccaatcctggtccttcctacttactcacctt
gcatgggcaagtggatttatgttccttattagttggagaggttactggcaagaattaatt
gatatcatcctctacatacatttgaaaacaccaattcttatcaatctttggaatggagac
atctatacacctctcgccttatctattgtacaagctagatttattgggcttgttcacttc
tcaaccggtctaattttaacttaccctccttttataataggtgctactagttag
>KrC12_psaB
ttttccattgcgaaattacggttagcctccaggccaaatccacatcaaaatcaccaatgc
acacaccaatgcaccttgactgatagctttatatctttaaatggtaggtgtgcgacttca
agatatttacaagtggtgggctctattcatgatatagaatcttactttggtatagataat
actctttctttaaatctacagatattcacagcacattggggtcacctaactataatctta
atatgggtatcaggcaatctttatcacatagcatccaatgctaactattctctctgggtt
aaaaatccaattccaagcatacctatagcacataatatttgggatccacattttactaac
tctacttccactccatattcccataccatcatcaccacaatcctcattgcttactcaggt
atttataaccaactatacacttctggatttaacactattaatcagatatacaagaccaca
tttacatcctcttgcctggcagtaatatcaatactattagctaagatacatatcaatact
cattcagaactattacacaagcttgcaactcatgcaagtcaaatcccatcattcttccag
cttctctactttctggatgtcggtatctcatctgttaatattcgattcaattttcacact
ggaattctagttggacttttctcaatagcacacacaggacatcttctagacatcacaata
ccagcctctcgagctccactaattcatacctcactctcttacttaacattctttggtggt
ttaaaatcgaatacaacttcattgtaccttacagatattgcacatcatcacttagcaatt
ggtatcacattcatccatacagggcatctatactcctctttcagggcagcccttggaaca
tatatacgagatacattatatacatcacaccttacacacagcataaaatcactacactta
gccctctcactcattctagccagttgtacagcacttacatctaccaccactcagcacatc
tattctttaacaccatacttttatttgtcttatgatcacatctattttacagttctatat
gtccatcactcctacatcacatcattcttaaccattggttctcatgcacatacagctatc
accttagtaagagattggatcacaccattagaactagaatccagctctaagcggataaga
atacatacacataaagctgccattattccacacttatcctgggtcagtctttggcttggt
tttcacacactagcagtatactctcataatgacacatgtatagcatttaactctccatct
aagcaaatcttaatagaagcaagcaatgctcaacttattcaacaagcatcaggaaaagct
ttatatgggaccatcaactccatcaacaactataataaatcatttgattcatttatacac
cctatcagtccaggtgattcatatgtacatcatgcaatagcacttggcttacatataact
gtcctcatcctactaaagggaggtcttgaagctcgtgggtctaagctaatgccagacaag
atggagcactcatttggcttttcatgtgatggtccaggtagaggtggcacatgtgatata
tcagcttgggattccttttatctagcaacattctggatgcttaatagcaacacatggata
agcttctacttccactacaagcaccttacacctcgtcaattctcagaaagttcaacttat
[truncated: 692,886 more chars]
